# Supplementary material for: Causal networks for climate model evaluation and constrained projections
Source: Nat Commun. 2020 Mar 16;11:1415. doi: 10.1038/s41467-020-15195-y (PMC7076021; doi:10.1038/s41467-020-15195-y)
Supplement: Supplementary file 1 — Supplementary Information [file 41467_2020_15195_MOESM1_ESM.pdf]

Causal networks for climate model evaluation and constrained projections

Peer Nowack[1,2,3,4*], Jakob Runge[5,1], Veronika Eyring[6,7], Joanna D. Haigh[1,2]

[1]Grantham Institute, Imperial College London, London, SW7 2AZ, UK.

[2]Department of Physics, Faculty of Natural Sciences, Imperial College London, London, SW7 2AZ, UK.

[3]Data Science Institute, Imperial College London, London, SW7 2AZ, UK.

[4]School of Environmental Sciences, University of East Anglia, Norwich, NR4 7TJ, UK.

[5]Deutsches Zentrum für Luft- und Raumfahrt (DLR), Institute of Data Science, Jena, 07745, Germany.

[6]Deutsches Zentrum für Luft- und Raumfahrt (DLR), Institute of Atmospheric Physics, Oberpfaffenhofen, 82234, Germany.

[7]University of Bremen, Institute of Environmental Physics, Bremen, 28359, Germany.

Supplementary Information - Section 1: Figures and Tables

Supplementary Figures

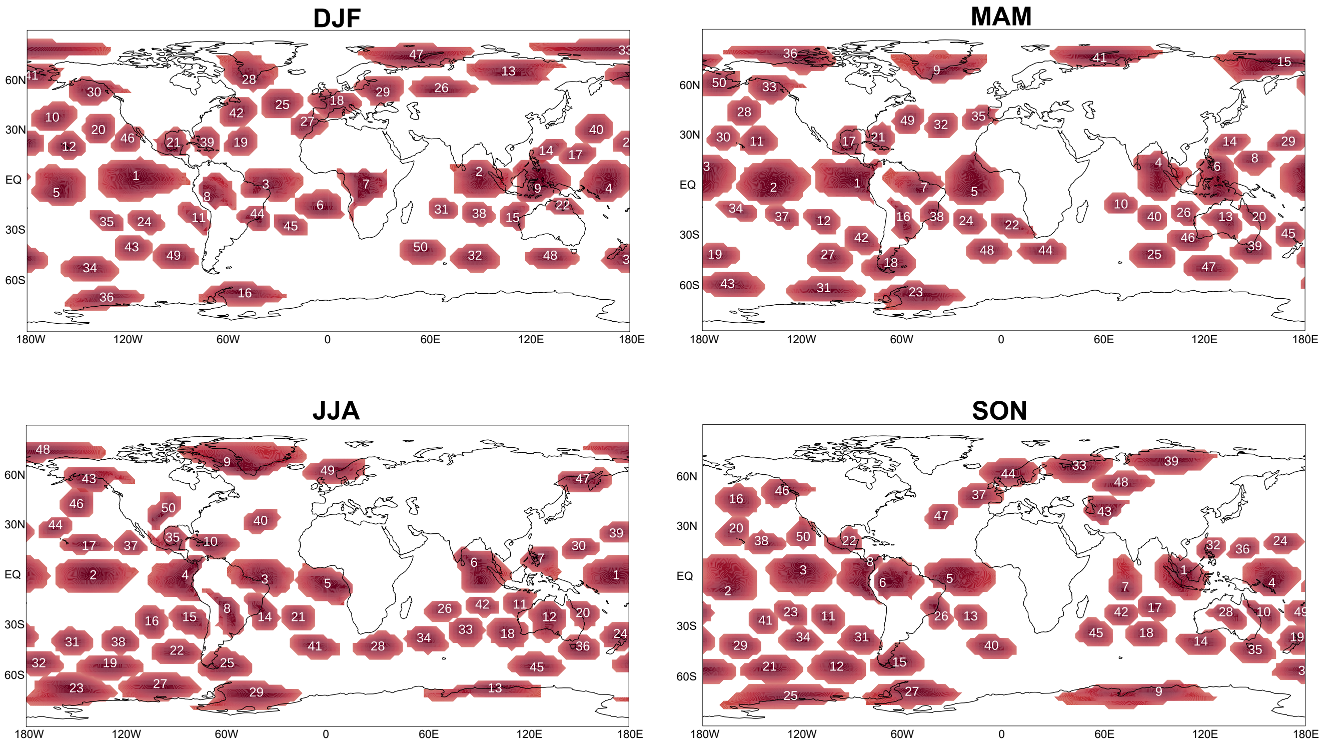

Supplementary Figure 1 | Maps of spatial core loadings of the Varimax principal components. Gridded daily-mean sea level pressure data from the NCAR-NCEP reanalysis is dimension-reduced using PCA-Varimax to a set of regionally confined climate modes of variability (see Methods). The same transformation is done separately for the four the meteorological seasons December-January-February (DJF), March-April-May (MAM), June-July-August (JJA) and September-October-November (SON) (as labelled). Core component regions are indicated in red, see also Figure 1b.

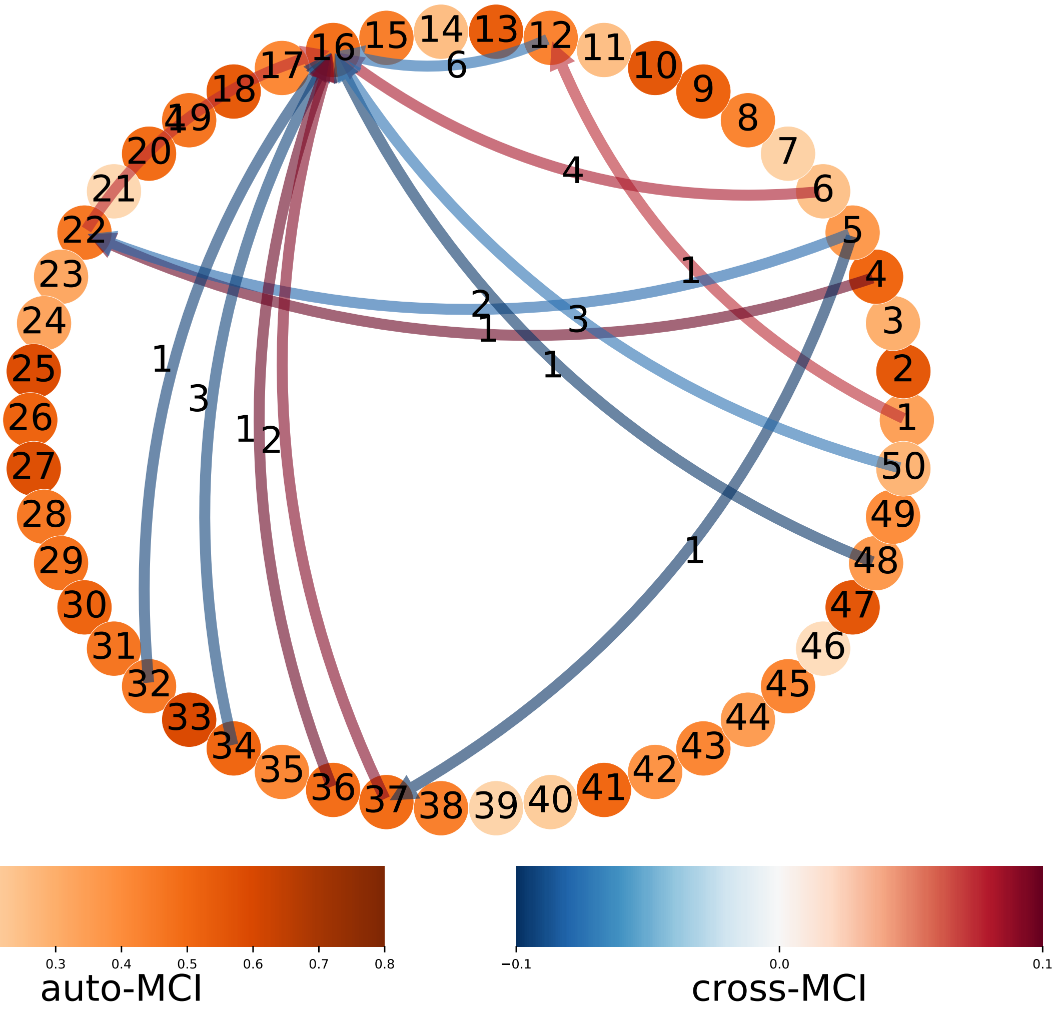

Supplementary Figure 2 | Illustration of indirect links in the causal model evaluation framework. As Figure 1d in the main text for NCEP-NCAR reanalysis data (approximating observations), but showing only a subset of links. Specifically, we show all incoming links (at α=10^{-3}) to node 16, which resembles a part of the Pacific-South American (PSA) pattern around Antarctica (see the equivalent node 20 in Supplementary section 2A below). There is no direct link between nodes representing the ENSO (nodes 1, 4, 5) and node 16, despite known connections between ENSO and PSA (1; 2). However, we do find indirect links via 1→12→16, 4→22→16, 5→22→16, 5→37→16 following routes from the tropical Pacific to the Southern Hemisphere mid-latitudes to Antarctica. This highlights the more complex, indirect connections within the framework used here, which provides sophisticated metrics characterizing the dynamical coupling of Earth's atmosphere. We note that this figure still shows a highly simplified picture as we do not include the multitude of other possible indirect links via two or more intermediate nodes. The cross-MCI colour scale is oversaturated for visualization purposes.

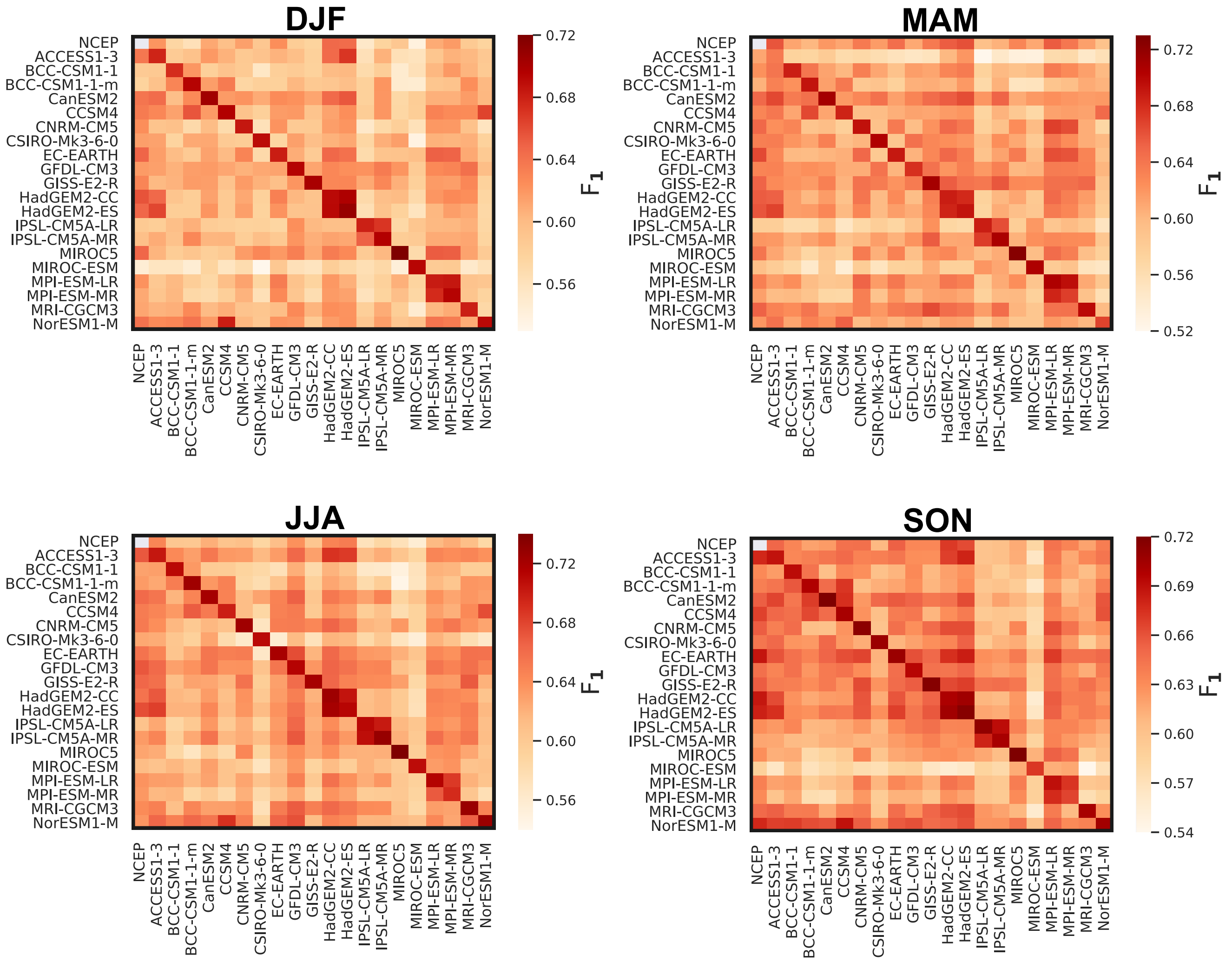

Supplementary Figure 3 | Seasonal $\mathbf{F_1}$-scores for the historical network intercomparison. Matrices of average F_1-scores for pair-wise network comparisons between the NCAR-NCEP reanalysis and 20 CMIP5 models (labelled following CMIP5 nomenclature in capital letters). As in Figure 3a but individually for the four meteorological seasons, i.e. December-January-February (DJF), March-April-May (MAM), June-July-August (JJA) and September-October-November (SON), as labelled. In addition, each first row/column shows the comparisons with networks constructed from the NCAR-NCEP reanalysis data. The α-significance threshold for PCMCI p-values is 10^{-4}, which results in around 400-500 links for each season.

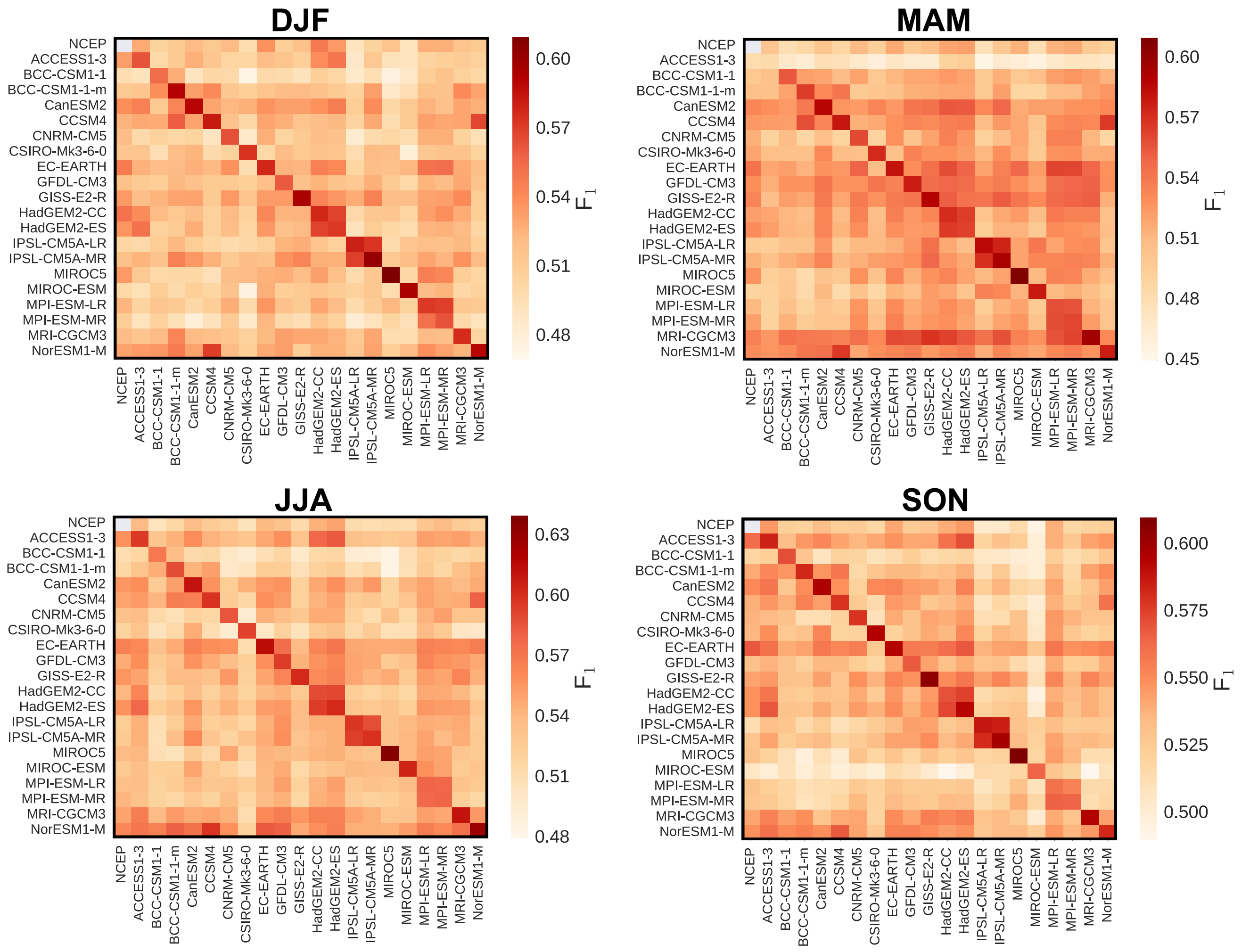

Supplementary Figure 4 | Seasonal $\mathbf{F_1}$-scores for the historical network intercomparison. Matrices of average F_1-scores for pair-wise network comparisons between the NCAR-NCEP reanalysis and 20 CMIP5 models (labelled following CMIP5 nomenclature in capital letters). As in Supplementary Figure 3, but for α=0.01. As a result, the general scores are lower (due to more noisy links taken into account), but the same major sub-groups are easy to recognize in the intercomparison matrices for each season.

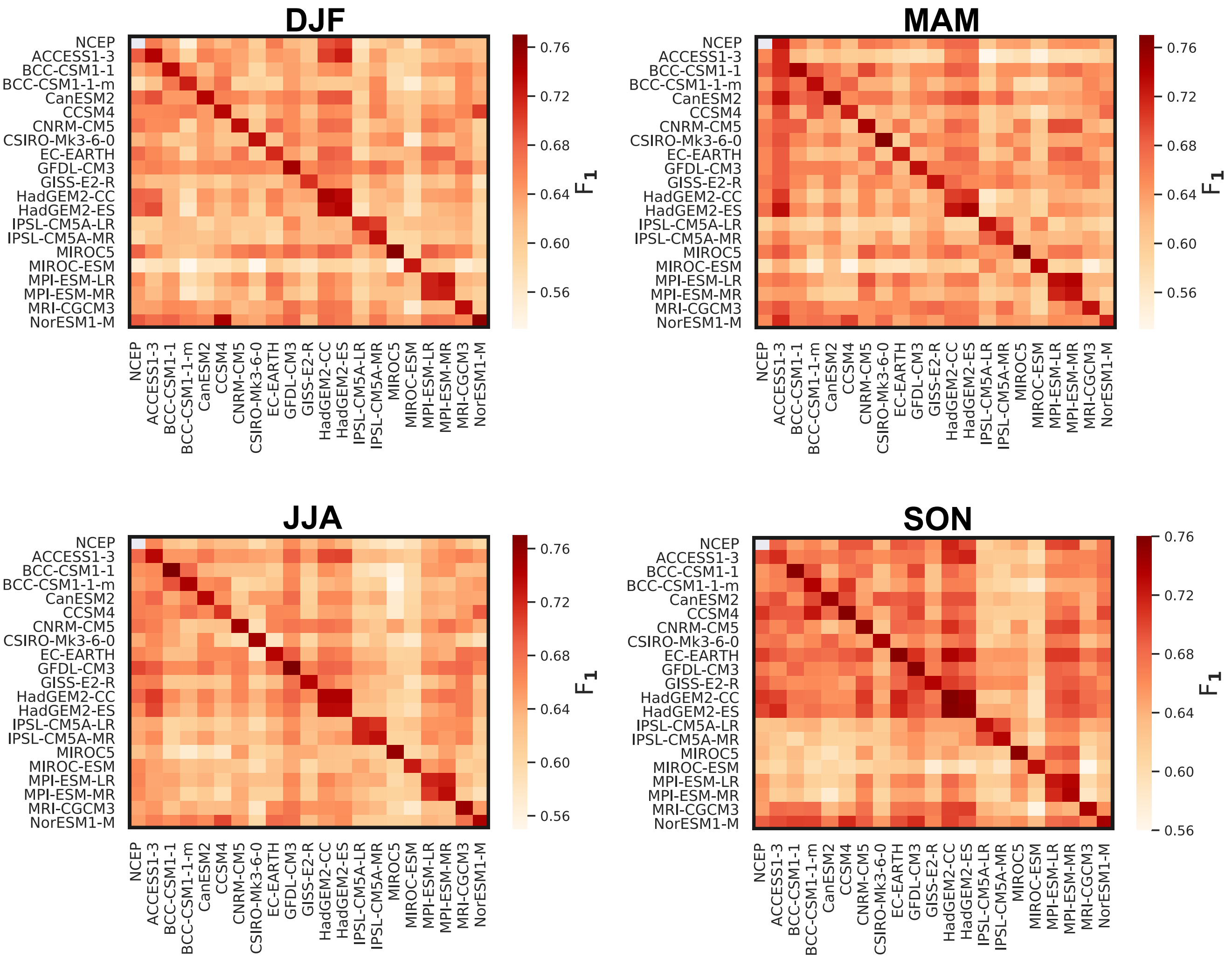

Supplementary Figure 5 | Seasonal F_1-scores for the historical network intercomparison. Matrices of average F_1-scores for pair-wise network comparisons between the NCAR-NCEP reanalysis and 20 CMIP5 models (labelled following CMIP5 nomenclature in capital letters). As in Supplementary Figure 3, but for α=10^{-6}, which results in 200-300 links per season. Consequently, the general scores are higher, but the same major sub-groups are easy to recognize in the intercomparison matrices for each season.

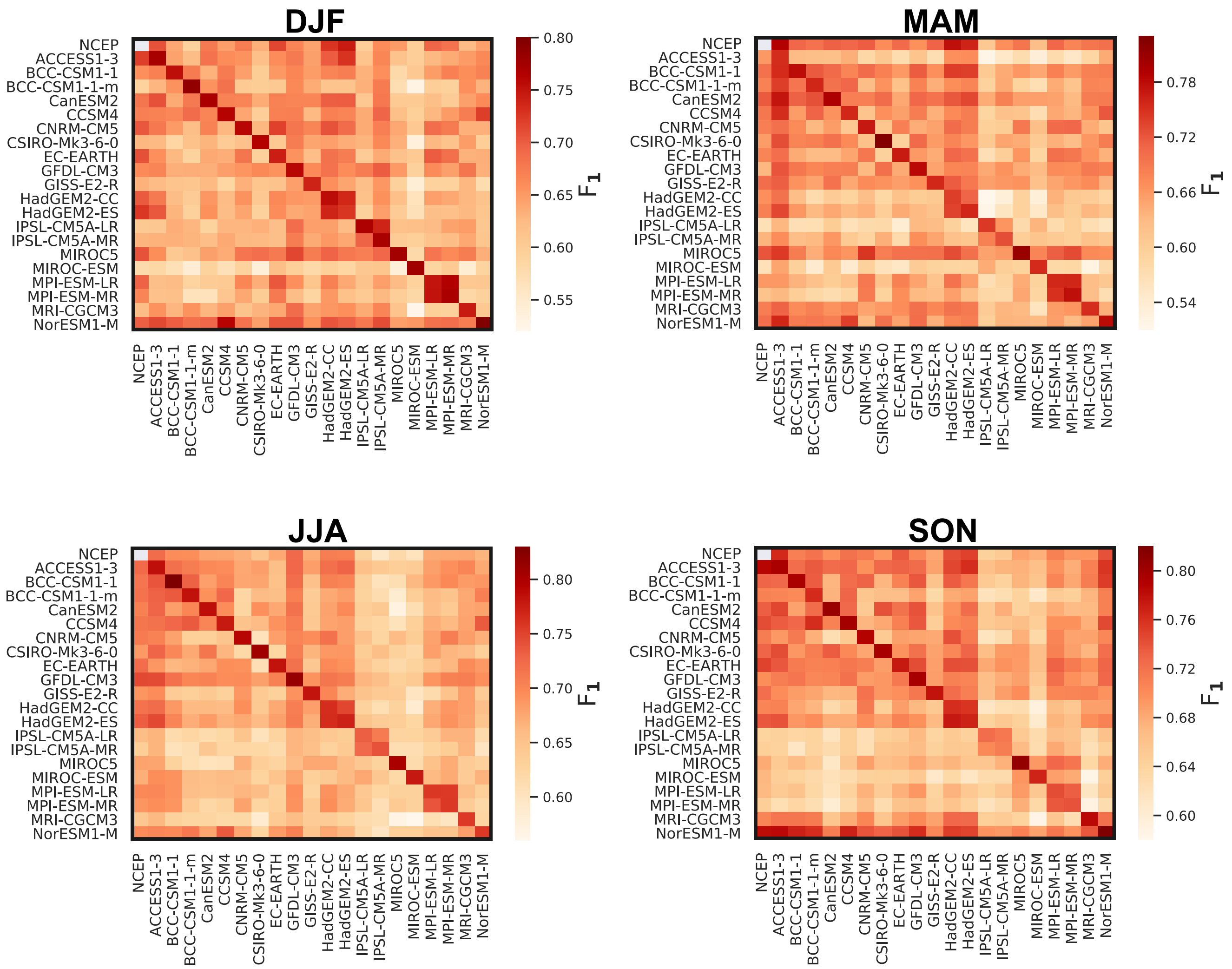

Supplementary Figure 6 | Seasonal F_1-scores for the historical network intercomparison. Matrices of average F_1-scores for pair-wise network comparisons between the NCAR-NCEP reanalysis and 20 CMIP5 models (labelled following CMIP5 nomenclature in capital letters). As in Supplementary Figure 3, but for α=10^{-9}, which results in 150-180 links per season. Consequently, the general scores are higher, but the same major sub-groups are easy to recognize in the intercomparison matrices for each season.

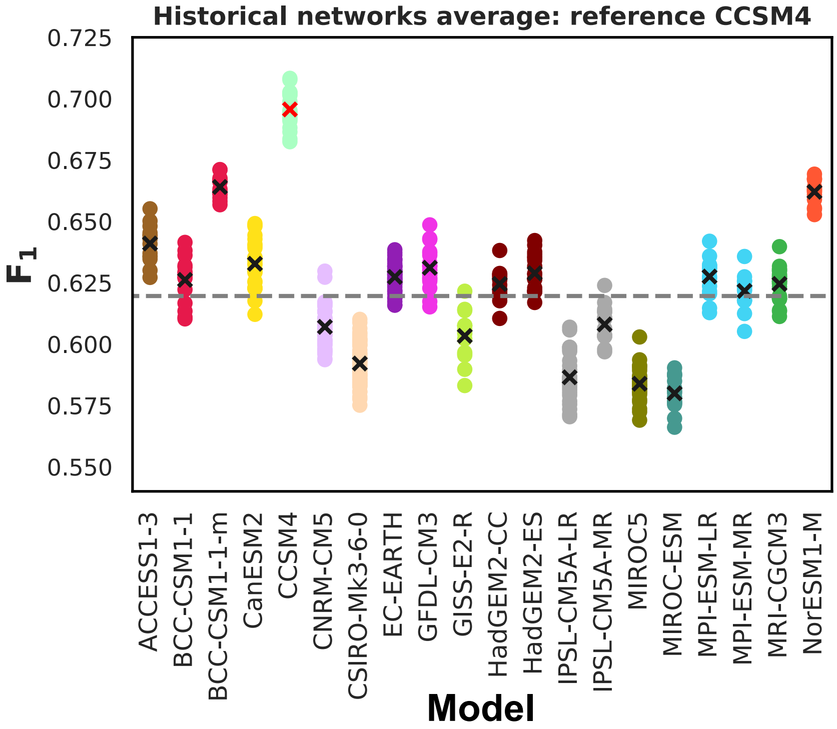

Supplementary Figure 7 | Historical simulations model intercomparison results. Addition to Figure 3 in the main text showing F_1-scores if CCSM4 is taken as the reference case. Note that NorESM1-M is highly similar to CCSM4, a model that uses a substantial number of CCSM model components (3). The two models are therefore considered as another subgroup.

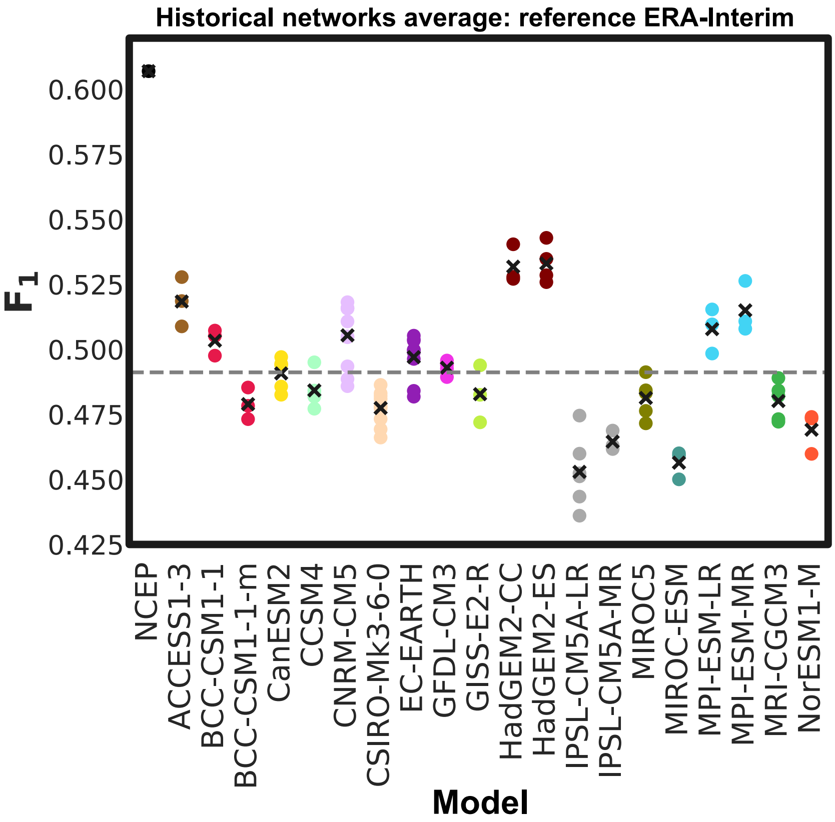

Supplementary Figure 8 | Historical network intercomparison results if the reference network is constructed from ERA-Interim reanalysis data. To test the robustness of the results depending on the reanalysis dataset used, we constructed another network from the shorter ERA-Interim reanalysis over the period 1st January 1979-31st December 2017 (4). Taking the ERA network as the ground truth, the NCAR-NCEP network is by far the most consistent (F_1-score=0.61) as compared to the model range (0.45-0.53) as shown in the figure above. Here we used a significance threshold of α=10^{-4} as done in the main text. Due to the forty years shorter dataset to consolidate the statistical relationships, the overall F_1-scores are lower than if the NCAR-NCEP reanalysis is taken as the reference. However, the model ranking is also almost the same as when the NCAR-NCEP reanalysis is used (Supplementary Table 1), underlining the robustness of the method to detect characteristic causal fingerprints. Note that the F_1-score is not expected to be perfect for the NCAR-NCEP reanalysis due to a variety of factors, including the slightly different periods of the observational records which the respective networks are based on (5), some statistical uncertainty and also existing differences in the reanalyses themselves even if they were spanning the same time period. Nevertheless, the NCAR-NCEP reanalysis is indeed the most consistent dataset among all, providing further evidence for the skill of CME to detect similar datasets as such.

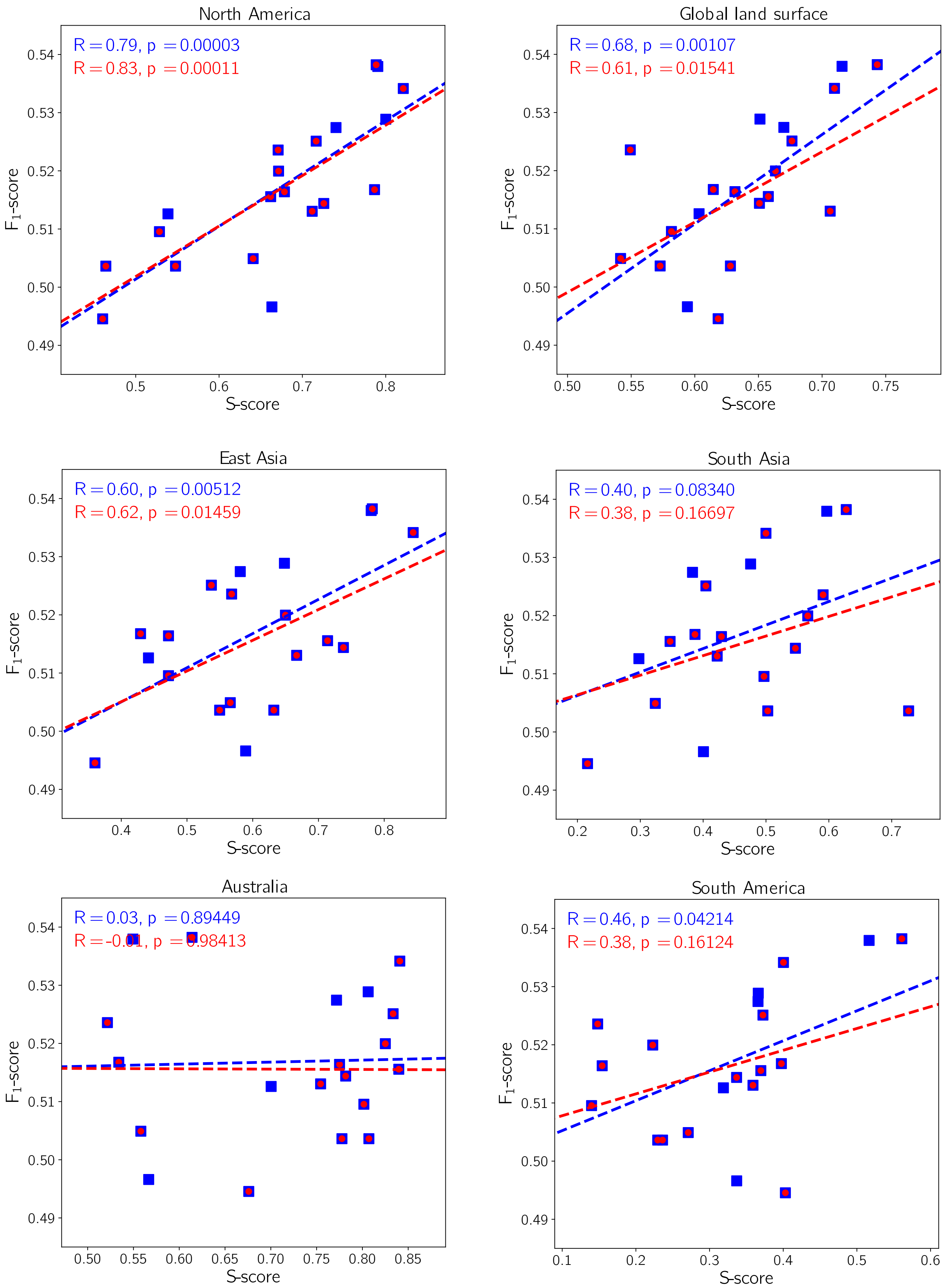

Supplementary Figure 9 | Correlations between the causal fingerprint and precipitation modelling scores for twenty CMIP5 models. As in Figure 4a in the main text, but using a different α-significance threshold of 0.01, i.e. more links are included in the network comparison.

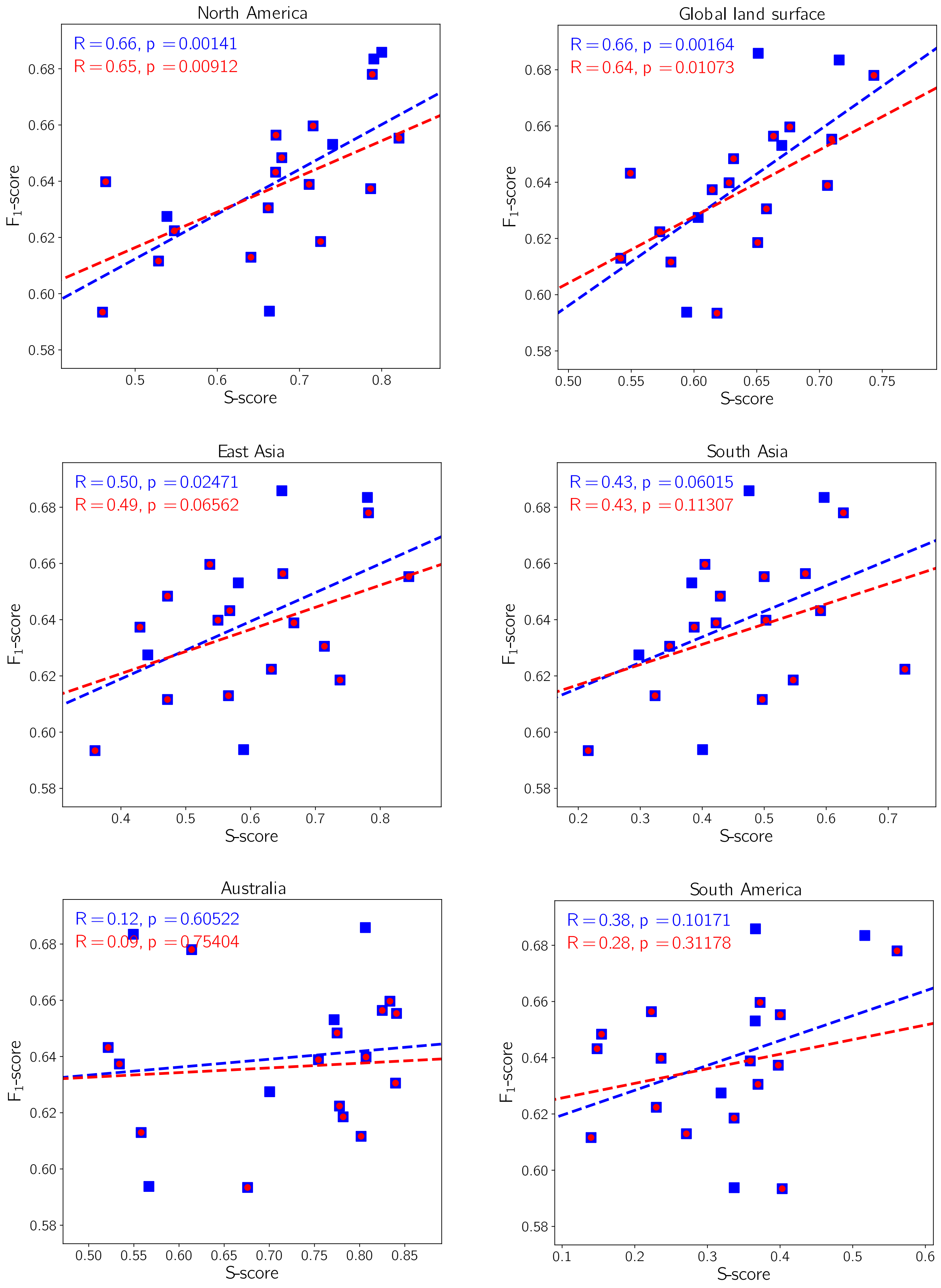

Supplementary Figure 10 | Correlations between the causal fingerprint and precipitation modelling scores for twenty CMIP5 models. As in Figure 4a in the main text, but using a different α-significance threshold of 10^{-6}, i.e. fewer links are included in the network comparison.

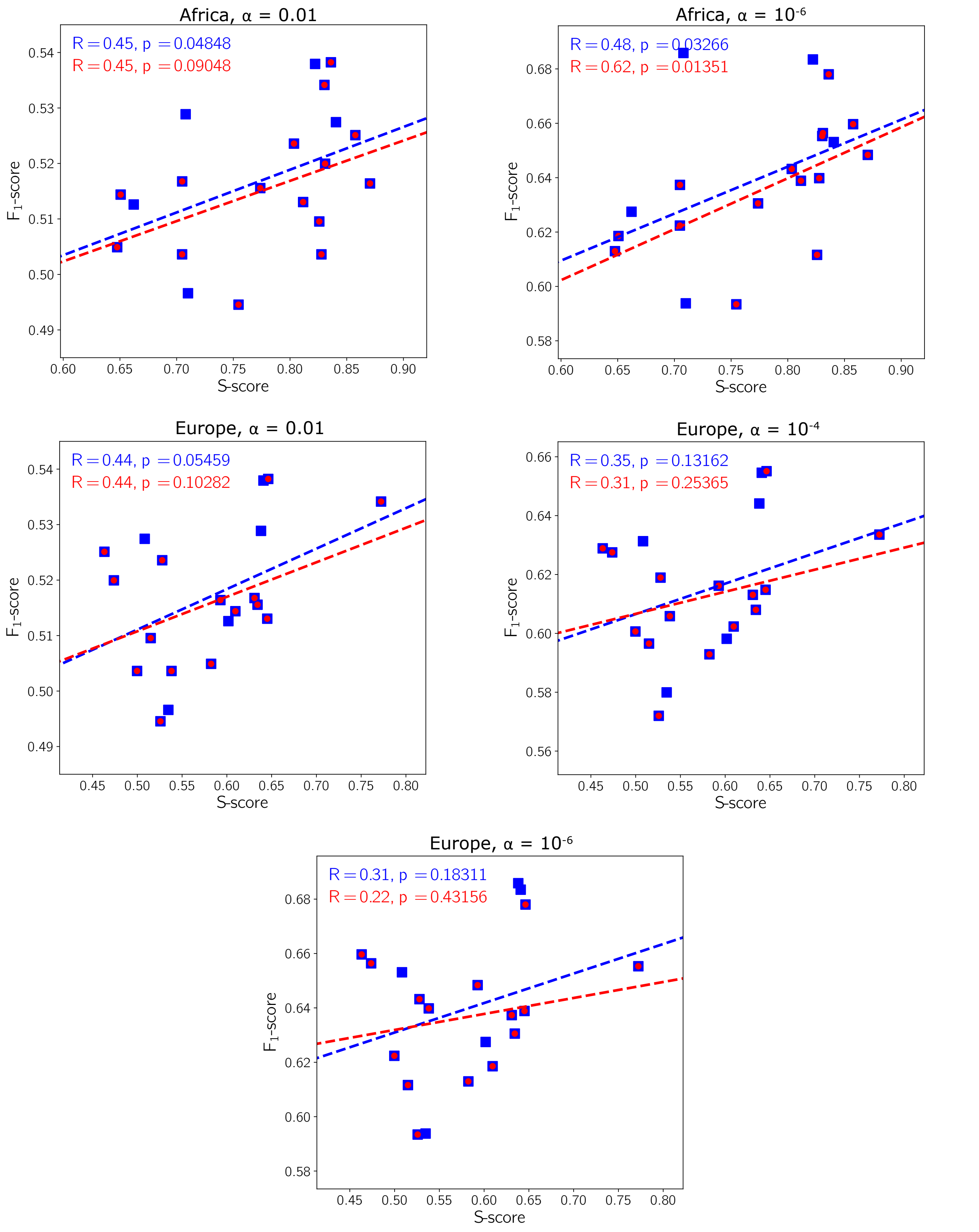

Supplementary Figure 11 | Correlations between the causal fingerprint and precipitation modelling scores for twenty CMIP5 models. As in Figure 4a in the main text, but using a different α-significance threshold of 0.01 and 10^{-6} for Africa (as labelled). In addition, we show results for Europe for all three significance thresholds, where Europe is defined spatially as the sum of the three IPCC AR5 regions North Europe, Central Europe and South Europe/Mediterranean. As evident from the figures, the correlations for Africa have the tendency to become more significant as fewer links are considered, whereas the opposite tendency is observable for Europe.

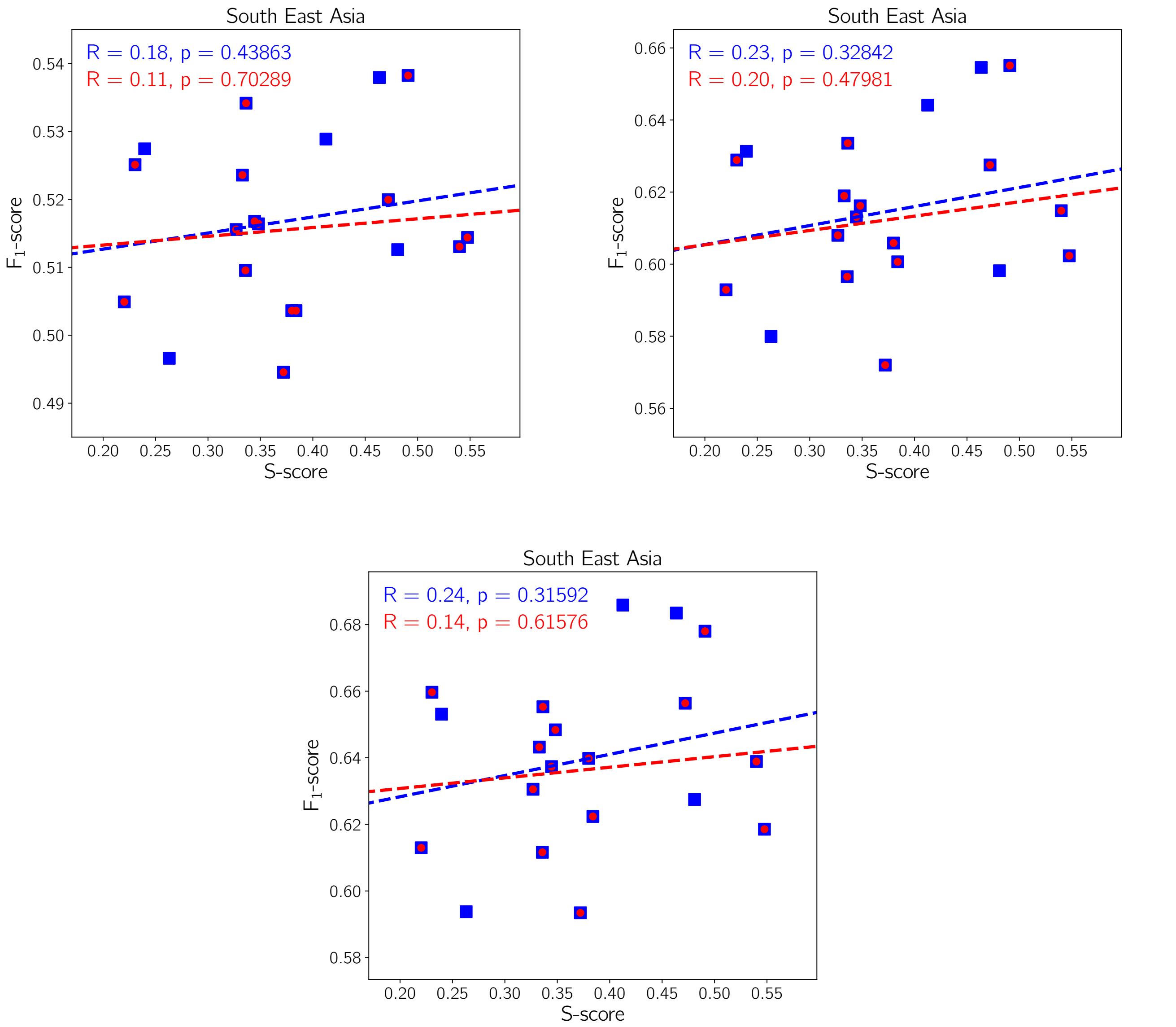

Supplementary Figure 12 | Correlations between the causal fingerprint and precipitation modelling scores for twenty CMIP5 models. As in Figure 4a in the main text, but using a different α-significance threshold of 0.01, 10^{-4} and 10^{-6} for South-East Asia (10S-20N, 90E-155E; following IPCC AR5 definitions), from left to right, top to bottom. There is an insignificant correlation between the two metrics; similar to Australia.

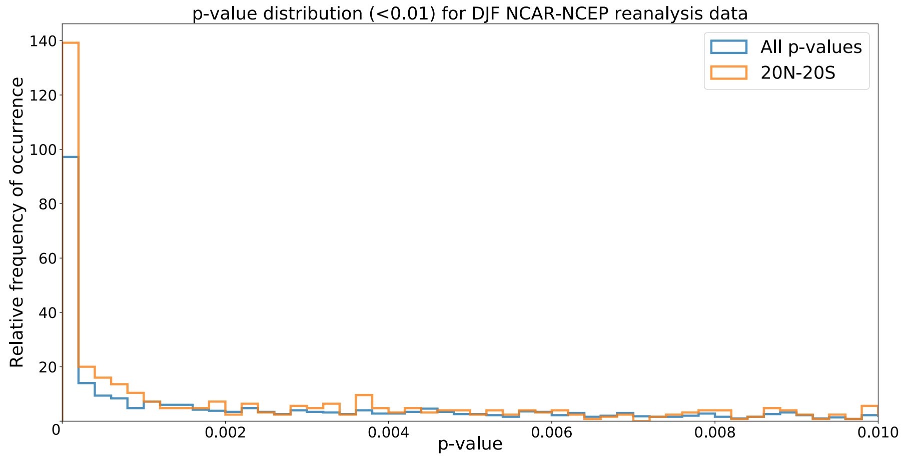

Supplementary Figure 13 | DJF *p*-value distributions of the network links learned from NCAR-NCEP reanalysis data. Normalised histograms of *p*-values (<0.01) for all network links (blue) and links between components whose maxima are located within 20N-20S (orange), see Supplementary Figure 1 for component locations. The link indices are [1,2,3,4,5,6,7,8,9,11,12,14,15,17,19,21,22,24,31,35,38,39,40,44,46] for components selected for 20N-20S. There are relatively more highly significant links in 20N-20S than for the overall network. As a result, choosing very small α-levels will select sub-networks that are, relatively, more focused on tropical interactions, whereas larger networks define F_1-scores which are increasingly more reflective of the extratropics.

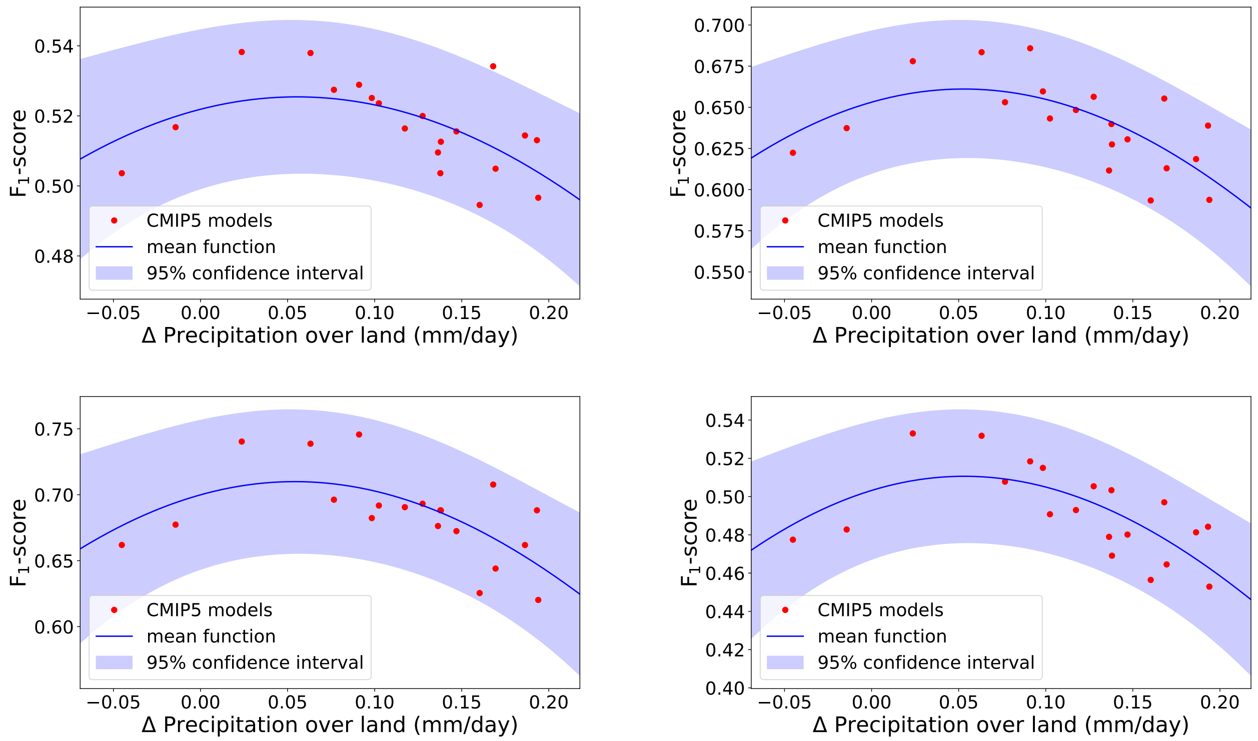

Supplementary Figure 14 | $\mathbf{F_1}$-scores and precipitation rates. As Figure 4b in the main text (explained in Methods), but for different network sizes: $\alpha = 0.01$ (top left), $\alpha = 10^{-6}$ (top right), $\alpha = 10^{-9}$ (bottom left). The log-marginal likelihoods of the fits are 54.41, 41.92, 36.97 (in the same order). We also show results if the network reconstructed from the shorter ERA-Interim reanalysis is taken as the ground truth reference on which the CMIP5 model F_1-scores are based (bottom right). Notably, for this satellite era data, the parabolic structure of the relationship becomes even more statistically robust, see also Supplementary Figure 15. The corresponding log-marginal likelihood is 45.60.

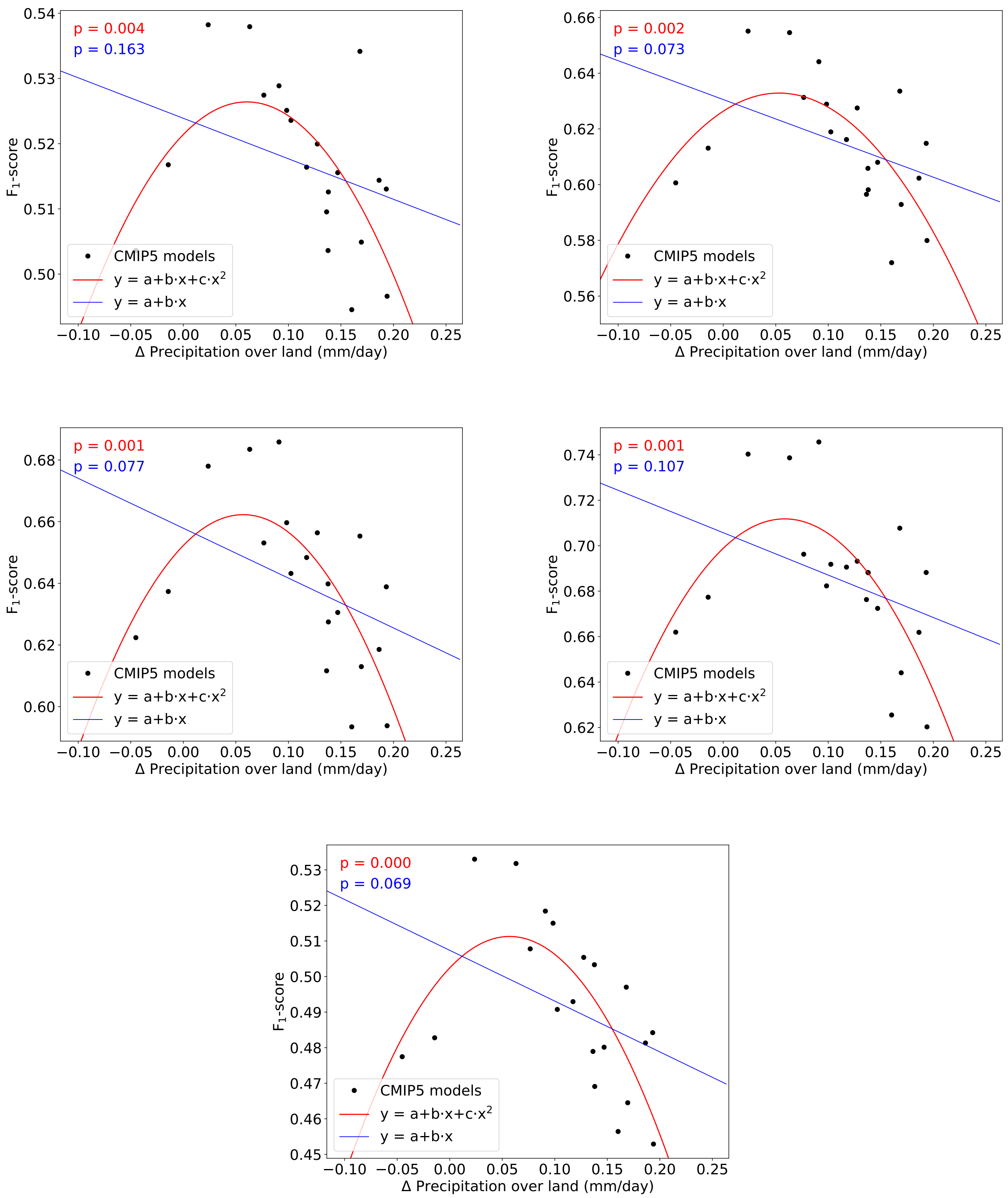

Supplementary Figure 15 | F_1-scores and their relationship to projected precipitation rate changes. As Figure 4b in the main text, but applying linear and parabolic fits to the data. The linear relationship appears physically implausible as it would imply that higher F_1-scores are generally associated with strongly negative trends in precipitation. In addition, it cannot explain the two models with negative trends, which are additionally causally independent. Indeed, we find that a parabolic relationship is statistically significant (p=0.002) whereas a linear relationship is not (p=0.07) at a 5% significance level using the same α-significance threshold in the PCMCI-constructed networks for links as done in the main text (10^{-4}; top right). We also show results for α=0.01 (top left), 10^{-6} (middle left) and 10^{-9} (middle right). The parabolic fit is statistically significant throughout. Note that correlating global S-scores (measuring historic precipitation biases) with future rainfall projection changes does not yield a clear relationship (Figure 4c). The figure in the bottom row shows the same fit if ERA-Interim reanalysis data is used as the reference case for the network construction and $\alpha = 10^{-4}$. The models with negative precipitation trends are GISS-E2-R and CSIRO-Mk3-6-0.

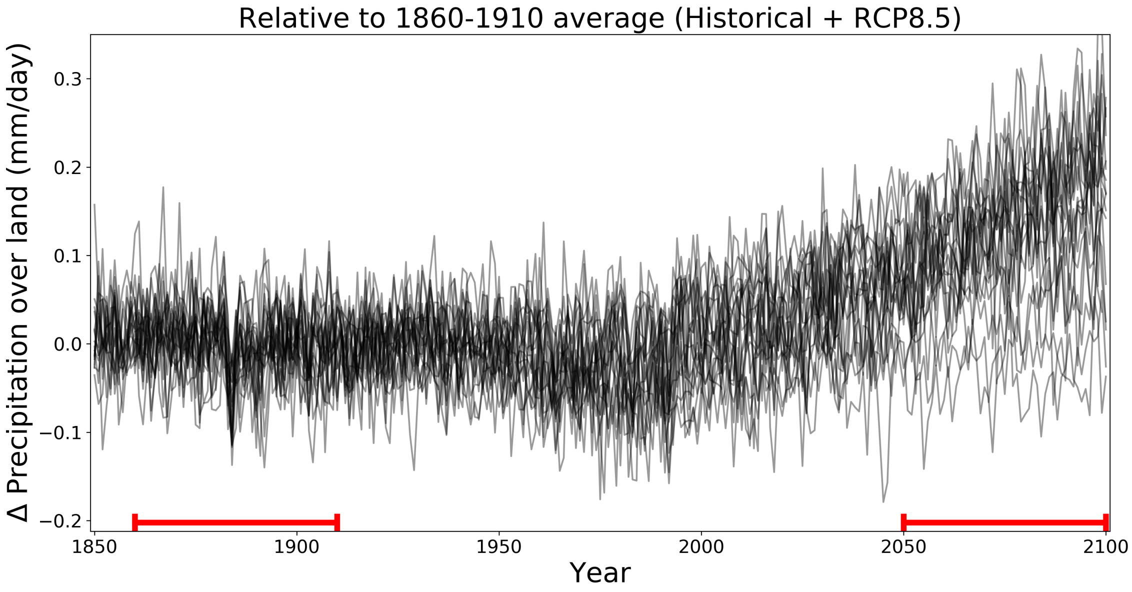

Supplementary Figure 16 | CMIP5 land surface precipitation projections under the historical and RCP8.5 scenario (except Antarctica). For single ensemble members of the models used for Figures 4b,c. Anomalies are shown relative to the individual models' 1860-1910 averages. Red intervals mark the periods used for calculating the model-specific precipitation rate changes.

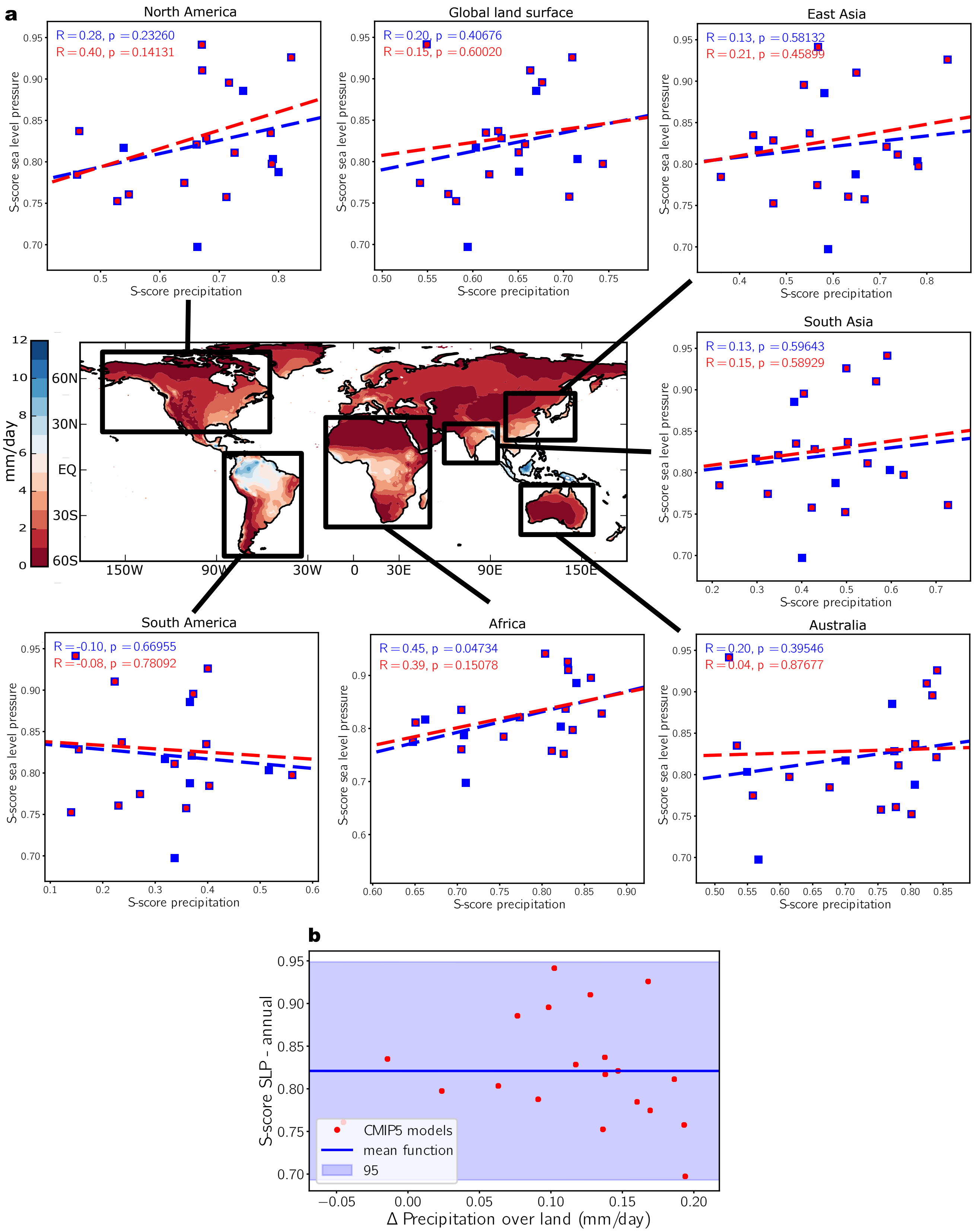

Supplementary Figure 17 | Correlations for annual mean sea level pressure skill metric. As Figure 4 in the main text, but with the F_1-score replaced by a Taylor S-score for global annual mean sea level pressure. The correlations of this simple dynamical metric with precipitation modelling skill are generally weaker and do not provide any emergent relationship for future precipitation changes (**b**).

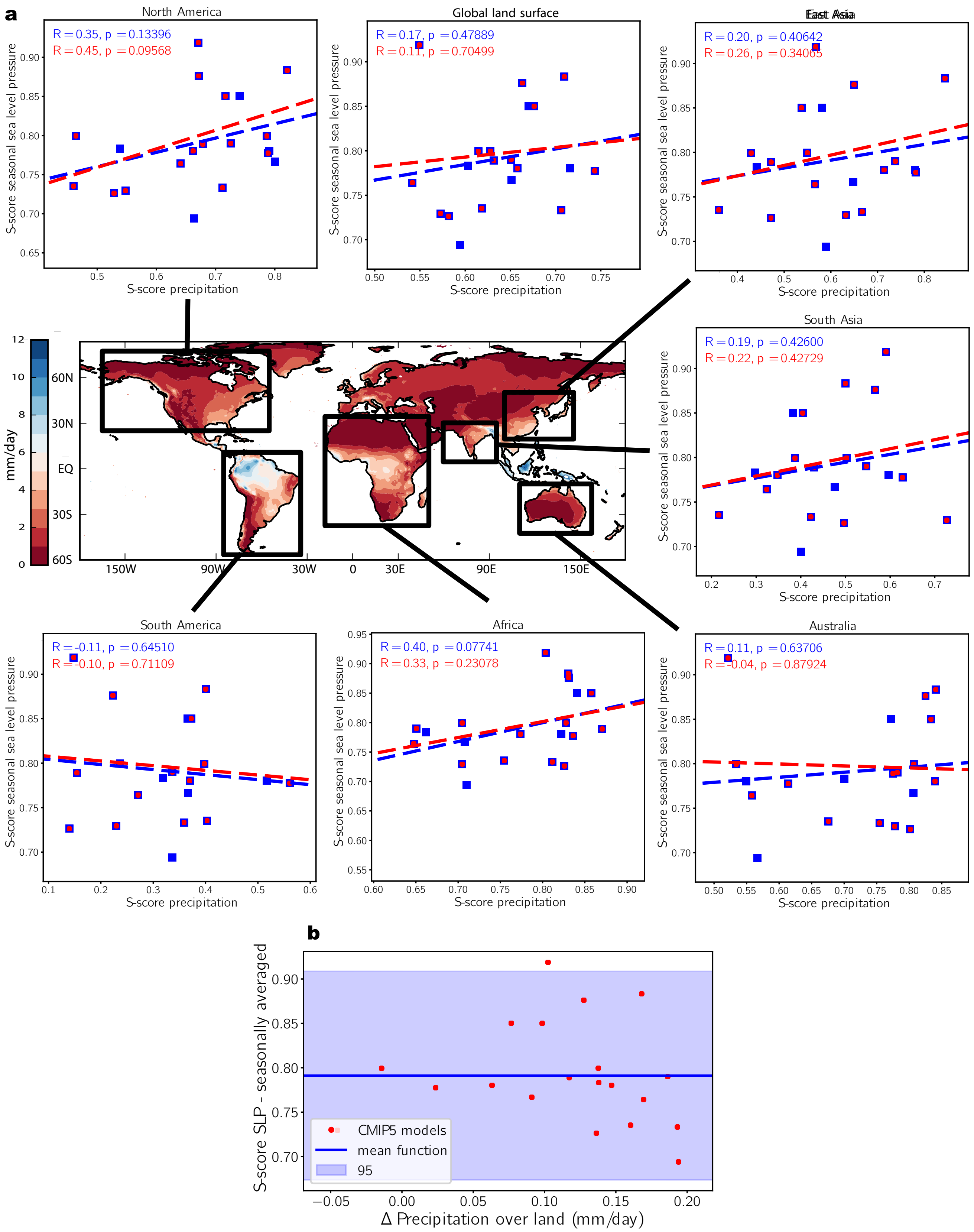

Supplementary Figure 18 | Correlations for seasonal average sea level pressure skill metric. As Figure 4 in the main text, but with the F_1-score replaced by a Taylor S-score for sea level pressure relative to NCAR-NCEP reanalysis data. The scores are calculated for each of the four meteorological seasons (DJF, MAM, JJA, SON) and then averaged to give an annual mean score. The correlations of this simple dynamical metric with precipitation modelling skill are generally weaker and do not provide any emergent relationship for future precipitation changes (**b**).

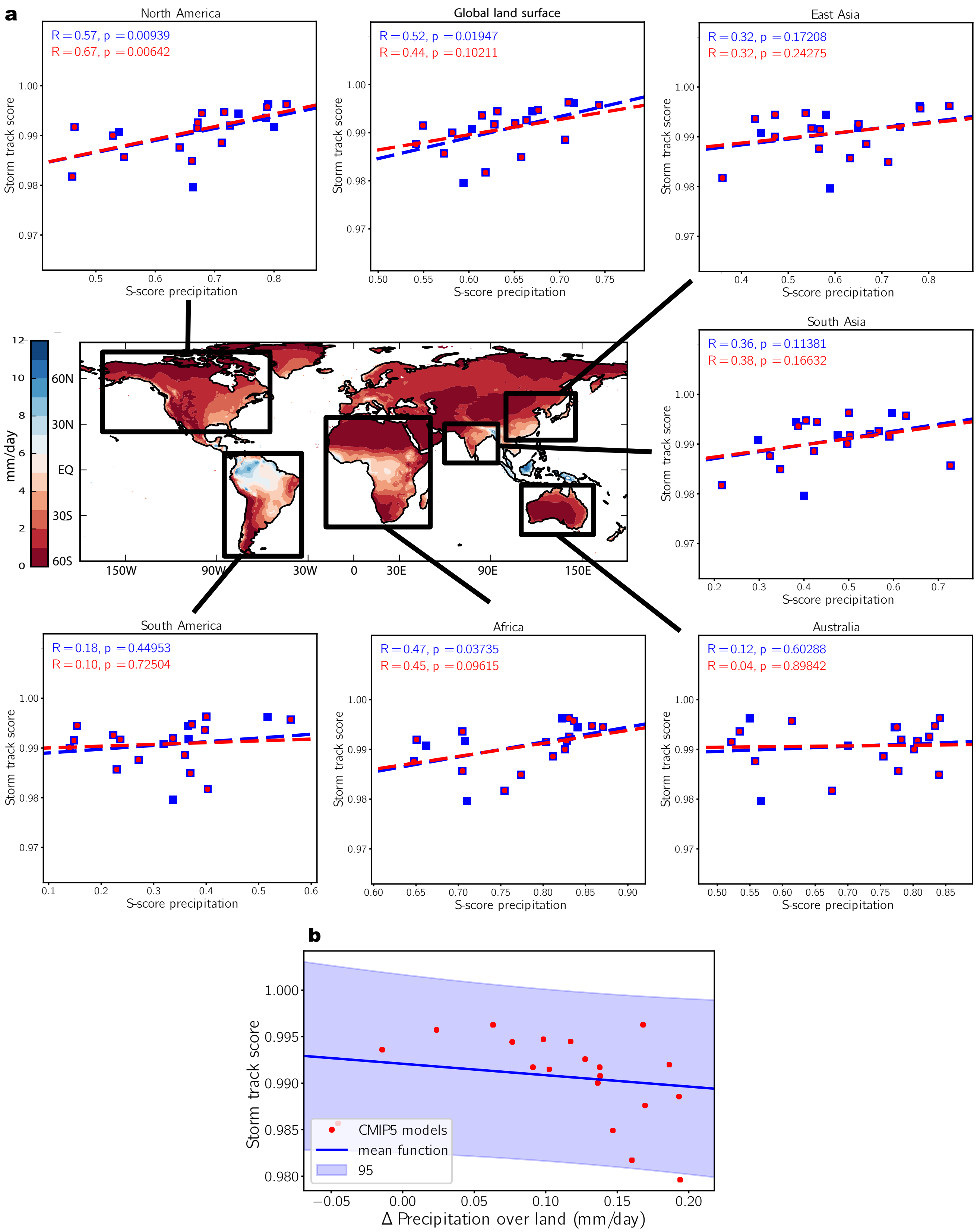

Supplementary Figure 19 | Correlations for the storm track skill metric. As Figure 4 in the main text, but with the F_1-score replaced by a metric measuring the skill of models in terms of their representation of global storm tracks when compared to the NCAR-NCEP reanalysis data. The metric is based on grid-cell-wise standard deviations of 2-6 days bandpass-filtered daily mean sea level pressure data (6). We compare each model with the reanalysis data by calculating the area-weighted pattern correlation of these quantities according to equation 6 in the Methods section. Overall the correlations are greater than for the purely pressure-based *S*-scores shown in Supplementary Figures S17 and S18. Next to the general dominance of short-term links in the causal networks (Figure 1d), this implies that the causal discovery method in particular picks up on synoptic variability coupling related to waveguides/storm tracks. However, we also note that the causal networks generally produce still higher correlations with the precipitation scores and also more significant correlations (compare Figure 4). In addition, the storm track score does not yield a clear emergent relationship for future precipitation change projections (**b**). Note that the pattern correlation values cannot be greater than 1 so that the uncertainty shading in (**b**) necessarily covers some physically implausible range. In addition note that the relationship in **b** is already much closer to the one for Figure 4b in the main text, as evident also from a direct second-order polynomial fit (Supplementary Figure 20).

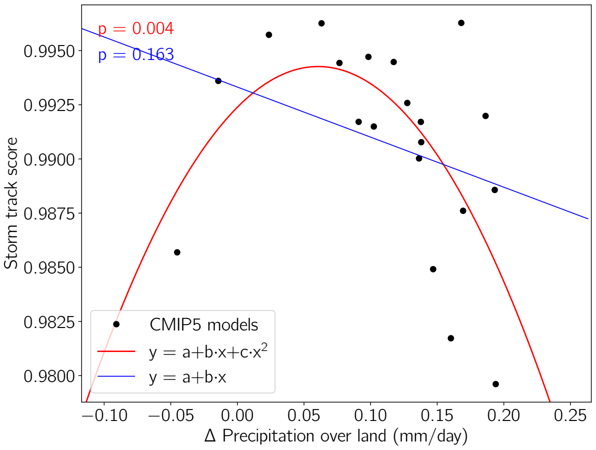

Supplementary Figure 20 | Correlations for the storm track skill metric with land precipitation rate changes. As Supplementary Figure 15, but for the storm track metric. Assuming a parabolic relationship, despite not indicated by the non-parametric Gaussian Process regression (Supplementary Figure 19), provides a statistically significant *p*-value. This *p*-value, however, is lower than for the F_1-scores, compare Supplementary Figure 15. Complementary to the results shown in Supplementary Figure 19, this underlines that storm tracks play an important role with regard to the physical interpretation of the causal fingerprint network links, while representing only a part of the overall physical interactions which altogether lead to the emergent relationship for future precipitation rate changes.

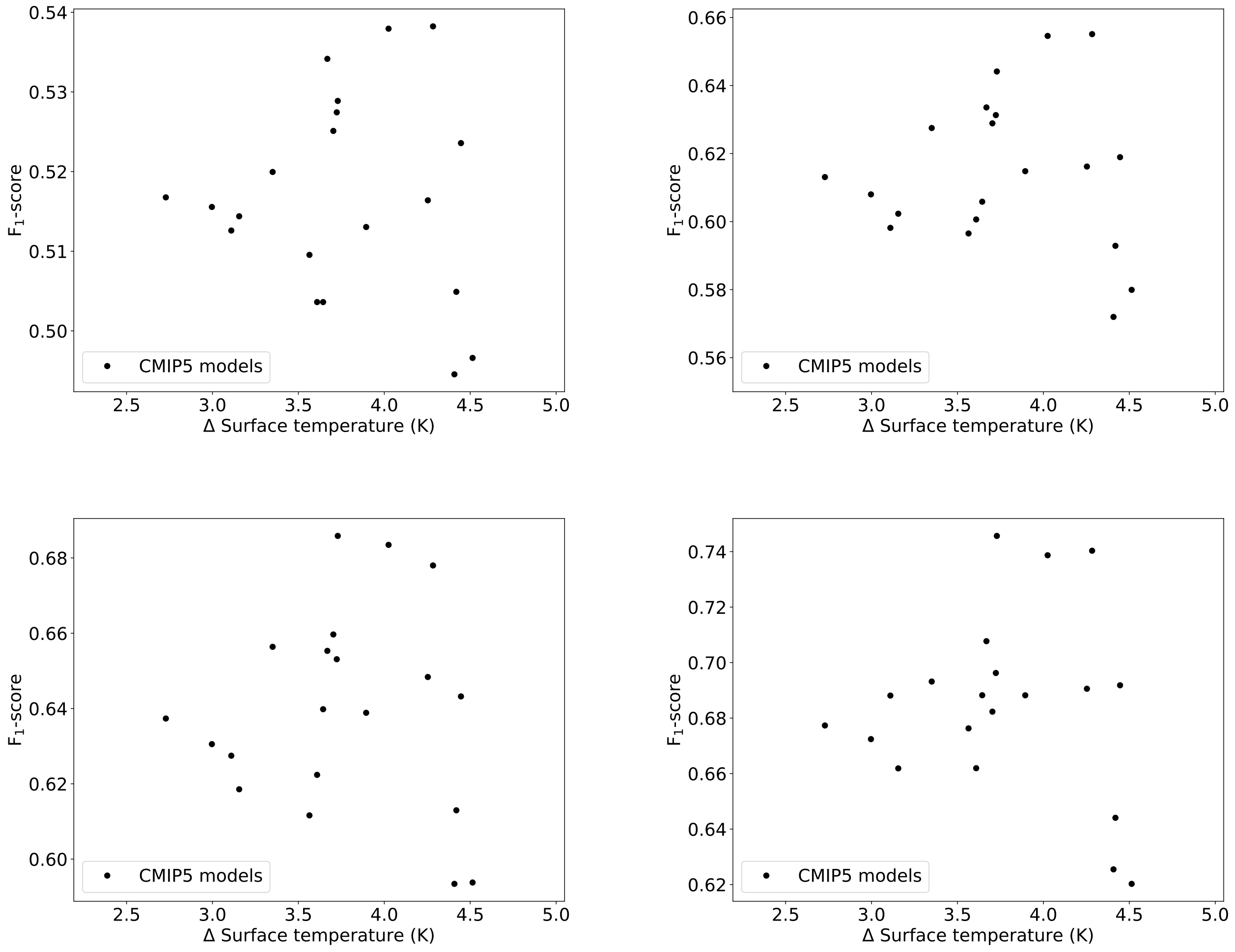

Supplementary Figure 21 | F_1-scores and their relationship to projected surface temperature changes. As for Figure 4b in the main text, but for global mean surface temperature changes under the RCP8.5 scenario. The relationship is noisier and more complex, but processes such as convection known to be involved in driving both dynamical interactions as well as model differences in climate sensitivity (7) could explain why the data is not entirely unstructured.

Supplementary Tables

$\alpha = 10^{-4}$ (NCEP)	$\alpha = 0.01$ (NCEP)	$\alpha = 10^{-6}$ (NCEP)	$\alpha = 10^{-9}$ (NCEP)	$\alpha = 10^{-4}$ (ERA)
20. HadGEM2-ES	HadGEM2-ES	ACCESS1-3	ACCESS1-3	HadGEM2-ES
19. HadGEM2-CC	HadGEM2-CC	HadGEM2-CC	HadGEM2-ES	HadGEM2-CC
18. ACCESS1-3	EC-EARTH	HadGEM2-ES	HadGEM2-CC	ACCESS1-3
17. EC-EARTH	ACCESS1-3	MPI-ESM-MR	EC-EARTH	MPI-ESM-MR
16. MPI-ESM-LR	MPI-ESM-LR	CNRM-CM5	MPI-ESM-LR	MPI-ESM-LR
15. MPI-ESM-MR	MPI-ESM-MR	EC-EARTH	CNRM-CM5	CNRM-CM5
14. CNRM-CM5	CanESM2	MPI-ESM-LR	CanESM2	BCC-CSM1-1
13. CanESM2	CNRM-CM5	GFDL-CM3	GFDL-CM3	EC-EARTH
12. GFDL-CM3	GISS-E2-R	CanESM2	BCC-CSM1-1	GFDL-CM3
11. CCSM4	GFDL-CM3	BCC-CSM1-1	CCSM4	CanESM2
10. GISS-E2-R	MRI-CGCM3	CCSM4	NorESM1-M	CCSM4
9. MRI-CGCM3	MIROC5	GISS-E2-R	MPI-ESM-MR	GISS-E2-R
8. BCC-CSM1-1	CCSM4	MRI-CGCM3	GISS-E2-R	MIROC5
7. MIROC5	NorESM1-M	NorESM1-M	BCC-CSM1-1-m	MRI-CGCM3
6. CSIRO-Mk3-6-0	BCC-CSM1-1-m	CSIRO-Mk3-6-0	MRI-CGCM3	BCC-CSM1-1-m
5. NorESM1-M	IPSL-CM5A-MR	MIROC5	CSIRO-Mk3-6-0	CSIRO-Mk3-6-0
4. BCC-CSM1-1-m	CSIRO-Mk3-6-0	IPSL-CM5A-MR	MIROC5	NorESM1-M
3. IPSL-CM5A-MR	BCC-CSM1-1	BCC-CSM1-1-m	IPSL-CM5A-MR	IPSL-CM5A-MR
2. IPSL-CM5A-LR	IPSL-CM5A-LR	IPSL-CM5A-LR	MIROC-ESM	MIROC-ESM
1. MIROC-ESM	MIROC-ESM	MIROC-ESM	IPSL-CM5A-LR	IPSL-CM5A-LR

Supplementary Table 1 | Key to the model numbers in Figure 3b (outer left column, $\alpha = 10^{-4}$). We also show results using different significance thresholds, which determine how many links are considered in the network based on their *p*-values given by PCMCI. The choice affects the model ranking slightly, but overall the results are relatively robust. The outermost right column shows results for the case when the network reconstructed from ERA-Interim data (rather than NCAR-NCEP) is instead used as the reference network, see also Supplementary Figure 8. Note that the α-level here is not the pc-α-level used in the condition-selection step of the PCMCI algorithm (Methods).

Modelling Center (or Group)	Model Names: Simulations (Variables)
Commonwealth Scientific and Industrial Research Organization (CSIRO) and Bureau of Meteorology (BOM), Australia (8)	ACCESS1.0: preindustrial: r1i1p1 (psl)<br>ACCESS1.3: preindustrial: r1i1p1 (psl) / historical: r1i1p1 (psl,pr), r2i1p1 (psl), r3i1p1 (psl) / RCP8.5: r1i1p1 (psl,pr)
Beijing Climate Center, China Meteorological Administration (9; 10)	BCC-CSM1.1: preindustrial: r1i1p1 (psl,pr) / historical: r1i1p1 (psl), r2i1p1 (psl), r3i1p1 (psl) / RCP8.5: r1i1p1 (psl,pr)<br>BCC-CSM1.1(m): preindustrial: r1i1p1 (psl) / preindustrial: r1i1p1 (psl,pr) / historical: r1i1p1 (psl), r2i1p1 (psl), r3i1p1 (psl) / RCP8.5: r1i1p1 (psl,pr)
Canadian Centre for Climate Modelling and Analysis (11; 12)	CanESM2: preindustrial: r1i1p1 (psl) / historical: r1i1p1 (psl,pr), r2i1p1 (psl), r3i1p1 (psl) / RCP8.5: r1i1p1 (psl,pr)
National Center for Atmospheric Research (13)	CCSM4: historical: r1i1p1 (psl,pr), r2i1p1 (psl), r6i1p1 (psl) / RCP8.5-r1i1p1(psl,pr)
Centre National de Recherches Meteorologiques / Centre Europeen de Recherche et Formation Avancee en Calcul Scientifique (14)	CNRM-CM5: preindustrial: r1i1p1 (psl) / historical: r1i1p1 (psl,pr), r2i1p1 (psl), r3i1p1 (psl), r4i1p1 (psl), r5i1p1 (psl), r6i1p1 (psl), r7i1p1 (psl), r8i1p1 (psl, r9i1p1 (psl), r10i1p1 (psl) / RCP8.5: r1i1p1 (pr)
Commonwealth Scientific and Industrial Research Organization in collaboration with Queensland Climate Change Centre of Excellence (15)	CSIRO-Mk3.6.0: historical: r1i1p1 (psl,pr), r2i1p1 (psl), r3i1p1 (psl), r4i1p1 (psl), r5i1p1 (psl), r6i1p1 (psl), r7i1p1 (psl), r8i1p1 (psl, r9i1p1 (psl), r10i1p1 (psl) / RCP8.5: r1i1p1 (pr), r4i1p1 (psl), r5i1p1 (psl), r6i1p1 (psl), r7i1p1 (psl), r8i1p1 (psl), r9i1p1 (psl), r10i1p1 (psl)
EC-EARTH consortium (16)	EC-EARTH: preindustrial: r1i1p1 (psl) / historical-r1i1p1 (psl,pr), r2i1p1 (psl), r7i1p1 (psl), r8i1p1 (psl), r9i1p1 (psl), r11i1p1 (psl), r12i1p1 (psl), r13i1p1 (psl), r14i1p1 (psl) / RCP8.5: r1i1p1 (psl,pr), r12i1p1 (psl), r13i1p1 (psl), r14i1p1 (psl)
NOAA Geophysical Fluid Dynamics Laboratory (17; 18)	GFDL-CM3: historical: r1i1p1 (psl,pr), r2i1p1 (psl), r3i1p1 (psl), r4i1p1 (psl / RCP8.5: r1i1p1 (psl,pr)<br>GFDL-ESM2G: preindustrial: r1i1p1 (psl)<br>GFDL-ESM2M: preindustrual: r1i1p1 (psl)
NASA Goddard Institute for Space Studies (19)	GISS-E2-R: historical-r6i1p1 (psl,pr), r6i1p2 (psl), r6i1p3 (psl) / RCP8.5: r1i1p1 (pr)
Met Office Hadley Centre (20; 21) (additional HadGEM2-ES realizations contributed by Instituto Nacional de Pesquisas Espaciais)	HadGEM2-CC: preindustrial: r1i1p1 (psl), historical: r1i1p1 (psl,pr), r2i1p1 (psl), r3i1p1 (psl) / RCP8.5: r1i1p1 (psl,pr), r2i1p1 (psl), r3i1p1 (psl)<br>HadGEM2-ES: pre-industrial: r1i1p1 (psl) / historical: r1i1p1 (psl,pr), r2i1p1 (psl), r3i1p1 (psl), r4i1p1 (psl) / RCP8.5: r1i1p1 (psl,pr), r2i1p1 (psl), r3i1p1 (psl), r4i1p1 (psl)
Institute for Numerical Mathematics (22)	preindustrial: INM-CM4: preindustrial: r1i1p1 (psl)
Institut Pierre-Simon Laplace (23)	IPSL-CM5A-LR: preindustrial: r1i1p1 (psl) / historical: r1i1p1 (psl,pr), r2i1p1 (psl), r3i1p1 (psl), r4i1p1 (psl), r5i1p1 (psl), r6i1p1 (psl) / RCP8.5: r1i1p1 (psl,pr), r2i1p1 (psl), r3i1p1 (psl), r4i1p1 (psl)<br>IPSL-CM5A-MR: preindustrial: r1i1p1 (psl) / historical: r1i1p1 (psl,pr), r2i1p1 (psl), r3i1p1 (psl) / RCP8.5: r1i1p1 (psl,pr)
Japan Agency for Marine-Earth Science and Technology, Atmosphere and Ocean Research Institute (The University of Tokyo), and National Institute for Environmental Studies (24)	MIROC-ESM: preindustrial: r1i1p1 (psl) / historical: r1i1p1 (psl,pr), r2i1p1 (psl), r3i1p1 (psl) / RCP8.5: r1i1p1 (psl,pr)<br>MIROC-ESM-CHEM: preindustrial: r1i1p1 (psl)
Atmosphere and Ocean Research Institute (The University of Tokyo), National Institute for Environmental Studies, and Japan Agency for Marine-Earth Science and Technology (25)	MIROC5: preindustrial: r1i1p1 (psl) / historical: r1i1p1 (psl,pr), r2i1p1 (psl), r3i1p1 (psl), r4i1p1 (psl), r5i1p1 (psl) / RCP8.5: r1i1p1 (psl,pr), r2i1p1 (psl), r3i1p1 (psl)
Max-Planck-Institut für Meteorologie (Max Planck Institute for Meteorology) (26)	MPI-ESM-MR: preindustrial: r1i1p1 (psl) / historical: r1i1p1 (psl,pr), r2i1p1 (psl), r3i1p1 (psl) / RCP8.5: r1i1p1 (psl,pr)<br>MPI-ESM-LR: preindustrial: r1i1p1 (psl) / historical: r1i1p1 (psl,pr), r2i1p1 (psl), r3i1p1 (psl) / RCP8.5: r1i1p1 (psl,pr), r2i1p1 (psl), r3i1p1 (psl)<br>MPI-ESM-P: preindustrial: r1i1p1 (psl)
Meteorological Research Institute (27)	MRI-CGCM3: preindustrial: r1i1p1 (psl) / historical: r1i1p1 (psl,pr), r2i1p1 (psl), r3i1p1 (psl), r4i1p2 (psl), r5i1p2 (psl) / RCP8.5: r1i1p1 (psl,pr)
Norwegian Climate Centre (3; 28)	NorESM1-M: preindustrial: r1i1p1 (psl) / historical: r1i1p1 (psl,pr), r2i1p1 (psl), r3i1p1 (psl) / RCP8.5: r1i1p1 (psl,pr)

Supplementary Table 2 | Interim and CMIP5 climate model datasets used in the main paper. As defined on `https://cmip.llnl.gov/cmip5/docs/CMIP5_modeling_groups.pdf` for the CMIP5 models. Variables used for each scenario are given in parentheses.

Supplementary References

[1] Karoly, D. J. Southern Hemisphere Circulation Features Associated with El Niño-Southern Oscillation Events (1989).

[2] Yeh, S. W. *et al.* ENSO Atmospheric Teleconnections and Their Response to Greenhouse Gas Forcing. *Reviews of Geophysics* **56**, 185–206 (2018).

[3] Bentsen, M. *et al.* The Norwegian Earth System Model, NorESM1-M â Part 1: Description and basic evaluation of the physical climate. *Geoscientific Model Development* **6**, 687–720 (2013).

[4] Dee, D. P. *et al.* The ERA-Interim reanalysis: Configuration and performance of the data assimilation system. *Quarterly Journal of the Royal Meteorological Society* **137**, 553–597 (2011).

[5] Tennant, W. Considerations when using pre-1979 NCEP/NCAR reanalyses in the southern hemisphere. *Geophysical Research Letters* **31**, L11112 (2004).

[6] Ulbrich, U. *et al.* Changing Northern Hemisphere storm tracks in an ensemble of IPCC climate change simulations. *Journal of Climate* **21**, 1669–1679 (2008).

[7] Sherwood, S. C., Bony, S. & Dufresne, J.-L. Spread in model climate sensitivity traced to atmospheric convective mixing. *Nature* **505**, 37–42 (2014).

[8] Dix, M. *et al.* The ACCESS coupled model: documentation of core CMIP5 simulations and initial results. *Australian Meteorological and Oceanographic Journal* **63**, 83–99 (2013).

[9] Xin, X.-G. *et al.* How Well does BCC_CSM1.1 Reproduce the 20th Century Climate Change over China? *Atmospheric and Oceanic Science Letters* **6**, 21–26 (2013).

[10] Wu, T. *et al.* An Overview of BCC Climate System Model Development and Application for Climate Change Studies. *Journal of Meteorological Research* **28**, 34–56 (2014).

[11] Arora, V. K. *et al.* Carbon emission limits required to satisfy future representative concentration pathways of greenhouse gases. *Geophysical Research Letters* **38**, L05805 (2011).

[12] Von Salzen, K. *et al.* The Canadian fourth generation atmospheric global climate model (CanAM4). Part I: Representation of physical processes. *Atmosphere - Ocean* **51**, 104–125 (2013).

[13] Gent, P. R. *et al.* The Community Climate System Model Version 4. *Journal of Climate* **24**, 4973–4991 (2011).

[14] Voldoire, A. *et al.* The CNRM-CM5.1 global climate model: Description and basic evaluation. *Climate Dynamics* **40**, 2091–2121 (2013).

[15] Jeffrey, S. *et al.* Australia's CMIP5 submission using the CSIRO-Mk3.6 model. *Australian meteorological and Oceanographic Journal* **63**, 1–13 (2013).

[16] Hazeleger, W. *et al.* EC-Earth V2.2: Description and validation of a new seamless earth system prediction model. *Climate Dynamics* **39**, 2611–2629 (2012).

[17] Donner, L. J. *et al.* The dynamical core, physical parameterizations, and basic simulation characteristics of the atmospheric component AM3 of the GFDL global coupled model CM3. *Journal of Climate* **24**, 3484–3519 (2011).

[18] Dunne, J. P. *et al.* GFDL's ESM2 Global Coupled Climate-Carbon Earth System Models. Part I: Physical Formulation and Baseline Simulation Characteristics. *Journal of Climate* **25**, 6646–6665 (2012).

[19] Schmidt, G. A. *et al.* Configuration and assessment of the GISS ModelE2 contributions to the CMIP5 archive. *Journal of Advances in Modeling Earth Systems* **6**, 141–184 (2014).

[20] Collins, W. J. *et al.* Development and evaluation of an Earth-System model â HadGEM2. *Geoscientific Model Development* **4**, 1051–1075 (2011). URL `http://www.geosci-model-dev.net/4/1051/2011/`.

[21] Jones, C. D. *et al.* The HadGEM2-ES implementation of CMIP5 centennial simulations. *Geoscientific Model Development* **4**, 543–570 (2011).

[22] Volodin, E. M., Dianskii, N. A. & Gusev, A. V. Simulating present-day climate with the INMCM4.0 coupled model of the atmospheric and oceanic general circulations. *Izvestiya, Atmospheric and Oceanic Physics* **46**, 414–431 (2010).

[23] Dufresne, J. L. *et al.* Climate change projections using the IPSL-CM5 Earth System Model: From CMIP3 to CMIP5. *Climate Dynamics* **40**, 2123–2165 (2013).

[24] Watanabe, S. *et al.* MIROC-ESM 2010: model description and basic results of CMIP5-20c3m experiments. *Geoscientific Model Development* **4**, 845–872 (2011).

[25] Watanabe, M. *et al.* Improved Climate Simulation by MIROC5: Mean States, Variability, and Climate Sensitivity. *Journal of Climate* **23**, 6312–6335 (2010).

[26] Giorgetta, M. A. *et al.* Climate and carbon cycle changes from 1850 to 2100 in MPI-ESM simulations for the Coupled Model Intercomparison Project phase 5. *Journal of Advances in Modeling Earth Systems* **5**, 572–597 (2013).

[27] Yukimoto, S. *et al.* A New Global Climate Model of the Meteorological Research Institute: MRI-CGCM3. *Journal of the Meteorological Society of Japan* **90A**, 23–64 (2012).

[28] Iversen, T. *et al.* The Norwegian Earth System Model, NorESM1-M â Part 2: Climate response and scenario projections. *Geoscientific Model Development* **6**, 389–415 (2013).

Supplementary Section 2 - Varimax Components

Supplementary Figs. 22 to 421 below provide an overview of all Varimax principal components for December-January-February (DJF), March-April-May (MAM), June-July-August (JJA) and September-October-November (SON), as obtained through the application of PCA Varimax to anomalised sea level pressure NCAR-NCEP reanalysis data (see Methods section in the main text). The table on the next page shows the indices of the components selected as the network nodes for each season.

DJF		MAM		JJA		SON	
Name	comps	Name	comps	Name	comps	Name	comps
c1	0	c1	0	c1	0	c1	0
c2	1	c2	1	c2	1	c2	1
c3	2	c3	2	c3	2	c3	2
c4	3	c4	3	c4	3	c4	3
c5	4	c5	4	c5	4	c5	4
c6	6	c6	5	c6	6	c6	5
c7	7	c7	7	c7	8	c7	6
c8	8	c8	9	c8	10	c8	8
c9	10	c9	10	c9	11	c9	11
c10	11	c10	14	c10	12	c10	14
c11	13	c11	15	c11	13	c11	15
c12	14	c12	16	c12	14	c12	16
c13	15	c13	17	c13	15	c13	17
c14	17	c14	18	c14	16	c14	18
c15	19	c15	20	c15	17	c15	19
c16	20	c16	21	c16	18	c16	20
c17	21	c17	22	c17	21	c17	21
c18	22	c18	23	c18	22	c18	22
c19	23	c19	25	c19	23	c19	23
c20	24	c20	26	c20	25	c20	24
c21	25	c21	27	c21	26	c21	27
c22	26	c22	28	c22	28	c22	28
c23	28	c23	30	c23	29	c23	29
c24	29	c24	31	c24	30	c24	31
c25	30	c25	32	c25	32	c25	32
c26	31	c26	33	c26	33	c26	33
c27	32	c27	34	c27	35	c27	36
c28	33	c28	35	c28	36	c28	37
c29	34	c29	38	c29	37	c29	38
c30	35	c30	41	c30	38	c30	39
c31	37	c31	42	c31	40	c31	40
c32	39	c32	43	c32	41	c32	41
c33	43	c33	44	c33	42	c33	43
c34	44	c34	45	c34	43	c34	44
c35	45	c35	46	c35	44	c35	45
c36	46	c36	48	c36	45	c36	46
c37	47	c37	49	c37	46	c37	47
c38	48	c38	50	c38	47	c38	48
c39	49	c39	51	c39	50	c39	49
c40	50	c40	52	c40	51	c40	50
c41	51	c41	54	c41	53	c41	57
c42	52	c42	55	c42	55	c42	58
c43	53	c43	56	c43	56	c43	59
c44	54	c44	57	c44	57	c44	60
c45	56	c45	58	c45	59	c45	61
c46	57	c46	59	c46	61	c46	62
c47	58	c47	61	c47	63	c47	63
c48	59	c48	63	c48	65	c48	64
c49	60	c49	64	c49	71	c49	65
c50	67	c50	71	c50	74	c50	66

Supplementary Table 3 | Indices of the Varimax principal components. The list of 50 selected indices out of 100 components in total for each season. The selected 50 components then form the nodes of the seasonal causal networks. Note that the numeration for components shown in Figure 1 in the main text start at 1, whereas the labelling for the nodes in these Supplementary Figures starts with index 0 so that indices are offset by 1.

Supplementary Figures 22 to 121

DJF PCA Varimax components. For each component, we provide the component index and the component's spatial loadings (top subfigure), the component time series (centre) as well as the component's power spectrum (bottom left) and autocorrelation properties (bottom right). For each component we further provide the fraction of explained variance as defined prior to the Varimax rotation.

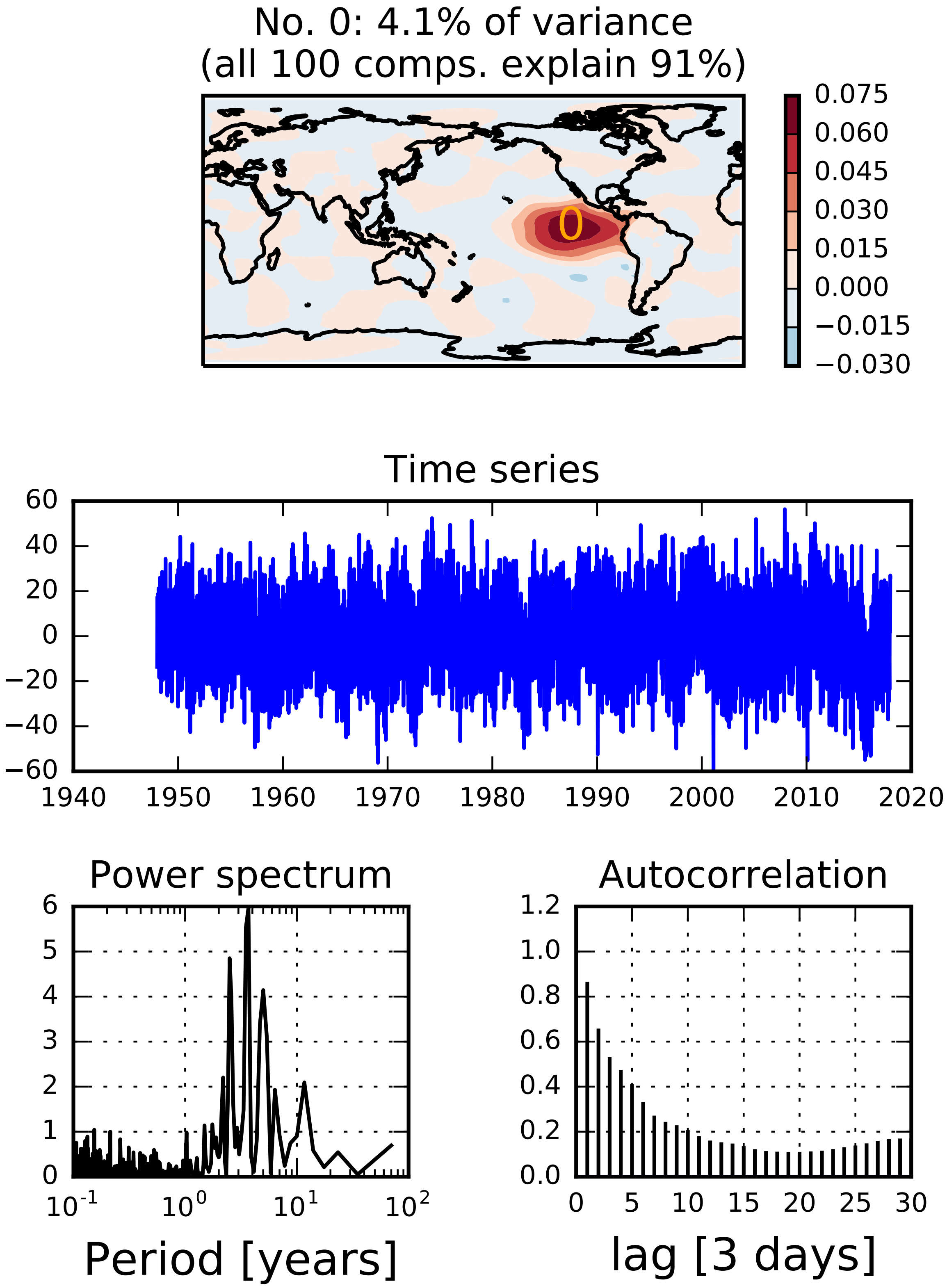
No. 0: 4.1% of variance
(all 100 comps. explain 91%)
0
0.075
0.060
0.045
0.030
0.015
0.000
−0.015
−0.030
Time series
60
40
20
0
−20
−40
−60
1940
1950
1960
1970
1980
1990
2000
2010
2020
Power spectrum
6
5
4
3
2
1
0
10^{-1}
10^{0}
10^{1}
10^{2}
Period [years]
Autocorrelation
1.2
1.0
0.8
0.6
0.4
0.2
0.0
0
5
10
15
20
25
30
lag [3 days]

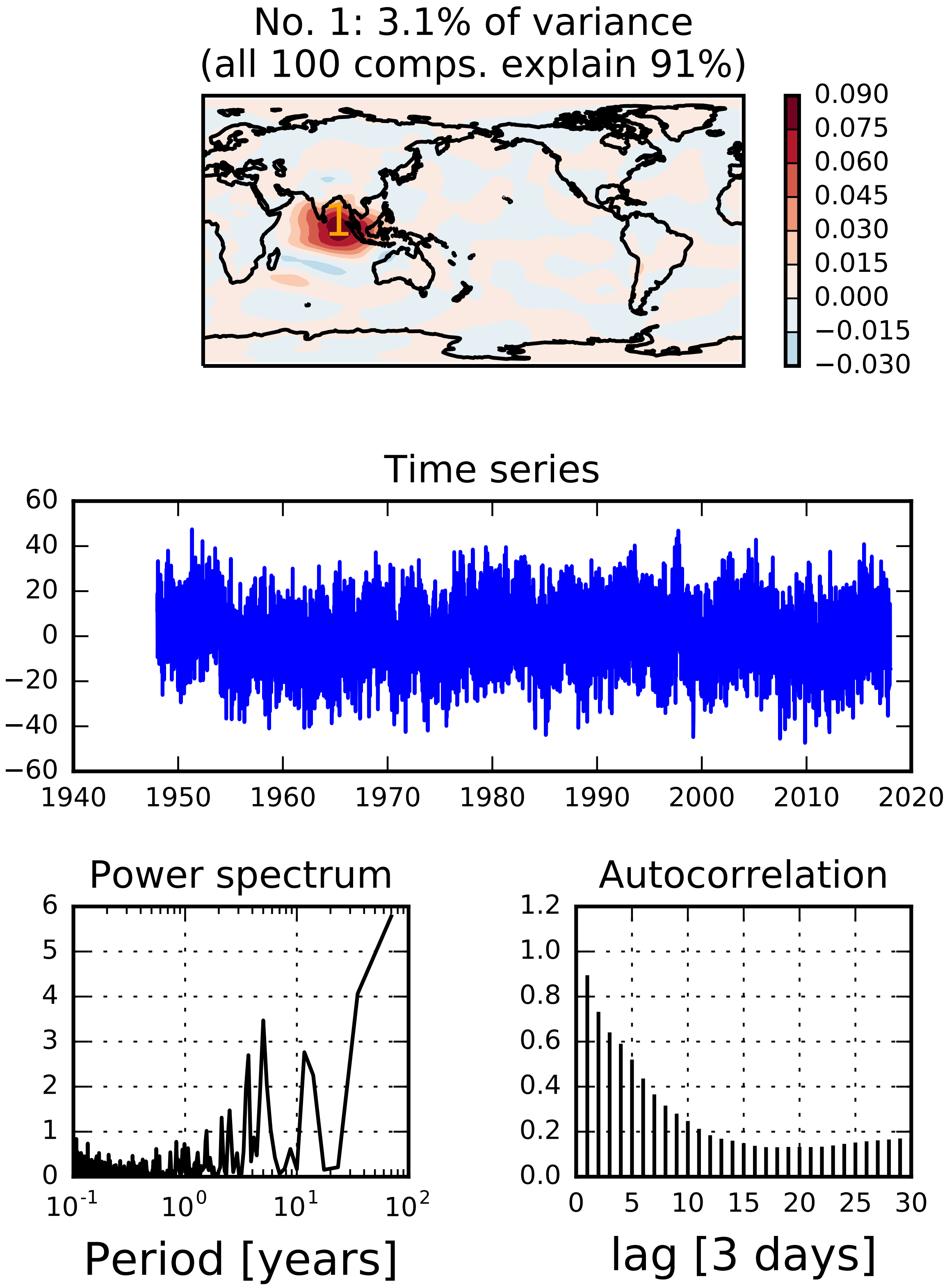
No. 1: 3.1% of variance
(all 100 comps. explain 91%)
1
0.090
0.075
0.060
0.045
0.030
0.015
0.000
−0.015
−0.030
Time series
60
40
20
0
−20
−40
−60
1940
1950
1960
1970
1980
1990
2000
2010
2020
Power spectrum
6
5
4
3
2
1
0
10^-1
10^0
10^1
10^2
Period [years]
Autocorrelation
1.2
1.0
0.8
0.6
0.4
0.2
0.0
0
5
10
15
20
25
30
lag [3 days]

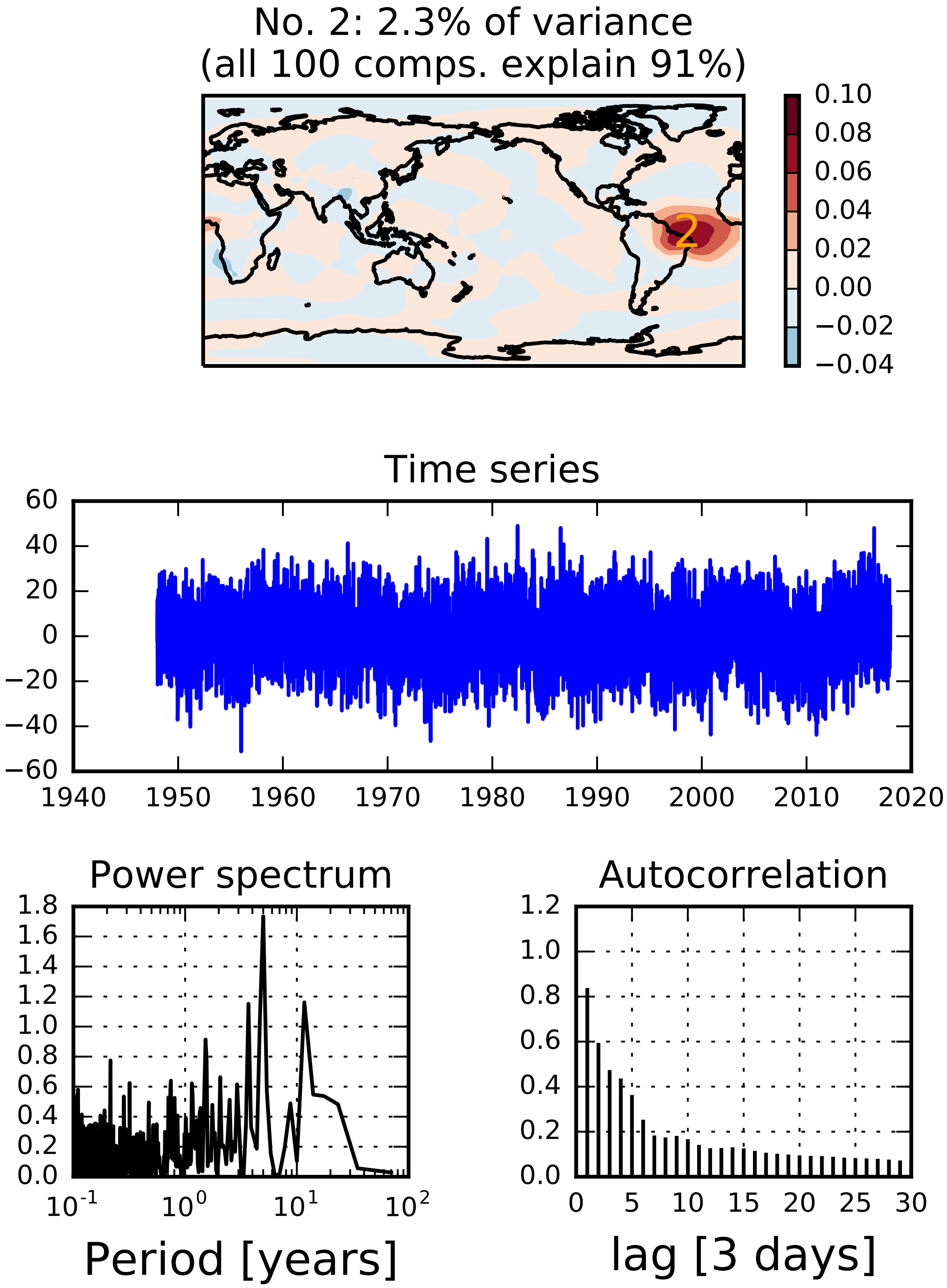
No. 2: 2.3% of variance
(all 100 comps. explain 91%)
2
0.10
0.08
0.06
0.04
0.02
0.00
−0.02
−0.04
Time series
60
40
20
0
−20
−40
−60
1940
1950
1960
1970
1980
1990
2000
2010
2020
Power spectrum
1.8
1.6
1.4
1.2
1.0
0.8
0.6
0.4
0.2
0.0
10^{-1}
10^{0}
10^{1}
10^{2}
Period [years]
Autocorrelation
1.2
1.0
0.8
0.6
0.4
0.2
0.0
0
5
10
15
20
25
30
lag [3 days]

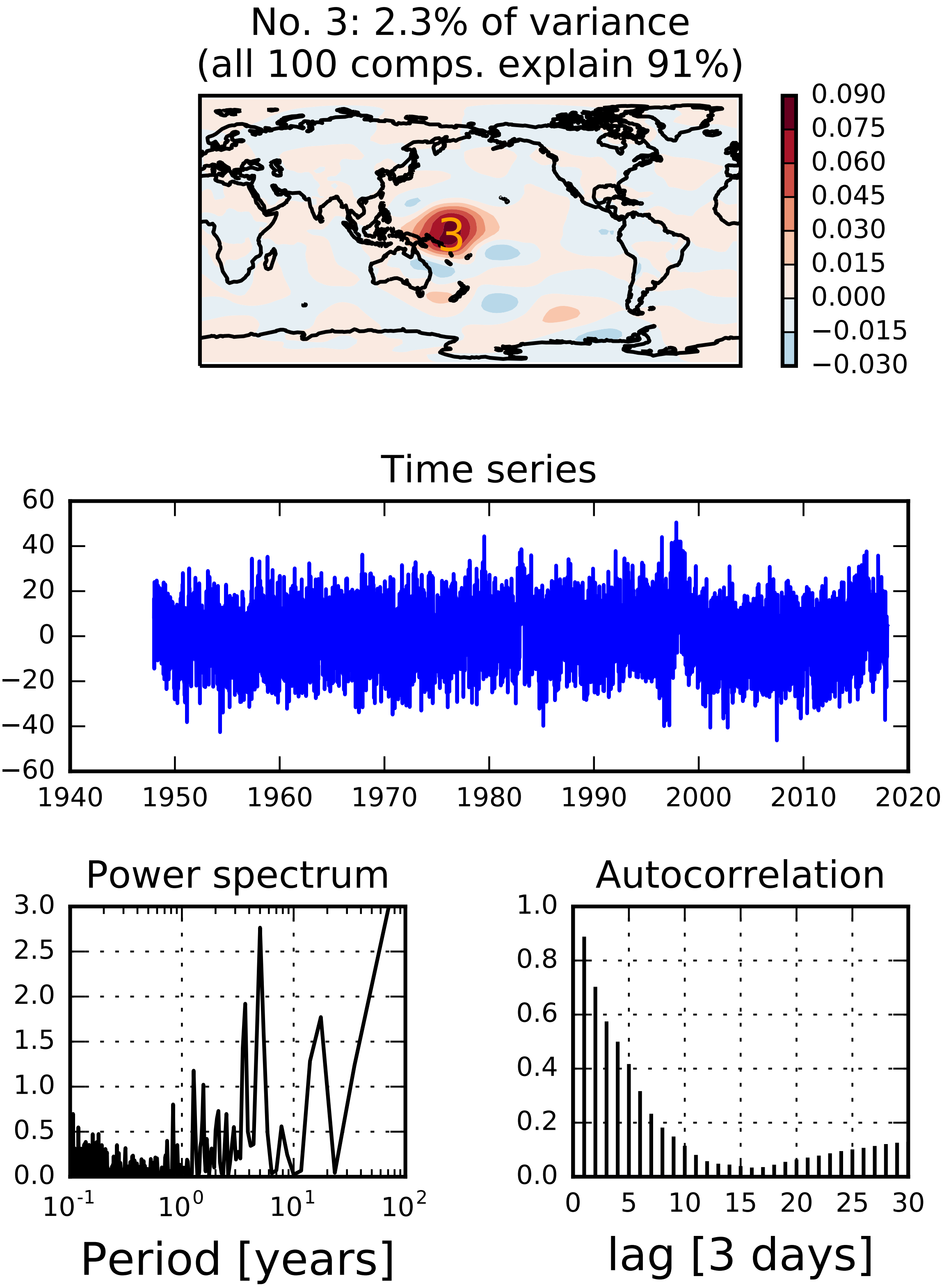
No. 3: 2.3% of variance
(all 100 comps. explain 91%)
3
0.090
0.075
0.060
0.045
0.030
0.015
0.000
−0.015
−0.030
Time series
60
40
20
0
−20
−40
−60
1940
1950
1960
1970
1980
1990
2000
2010
2020
Power spectrum
3.0
2.5
2.0
1.5
1.0
0.5
0.0
10^-1
10^0
10^1
10^2
Period [years]
Autocorrelation
1.0
0.8
0.6
0.4
0.2
0.0
0
5
10
15
20
25
30
lag [3 days]

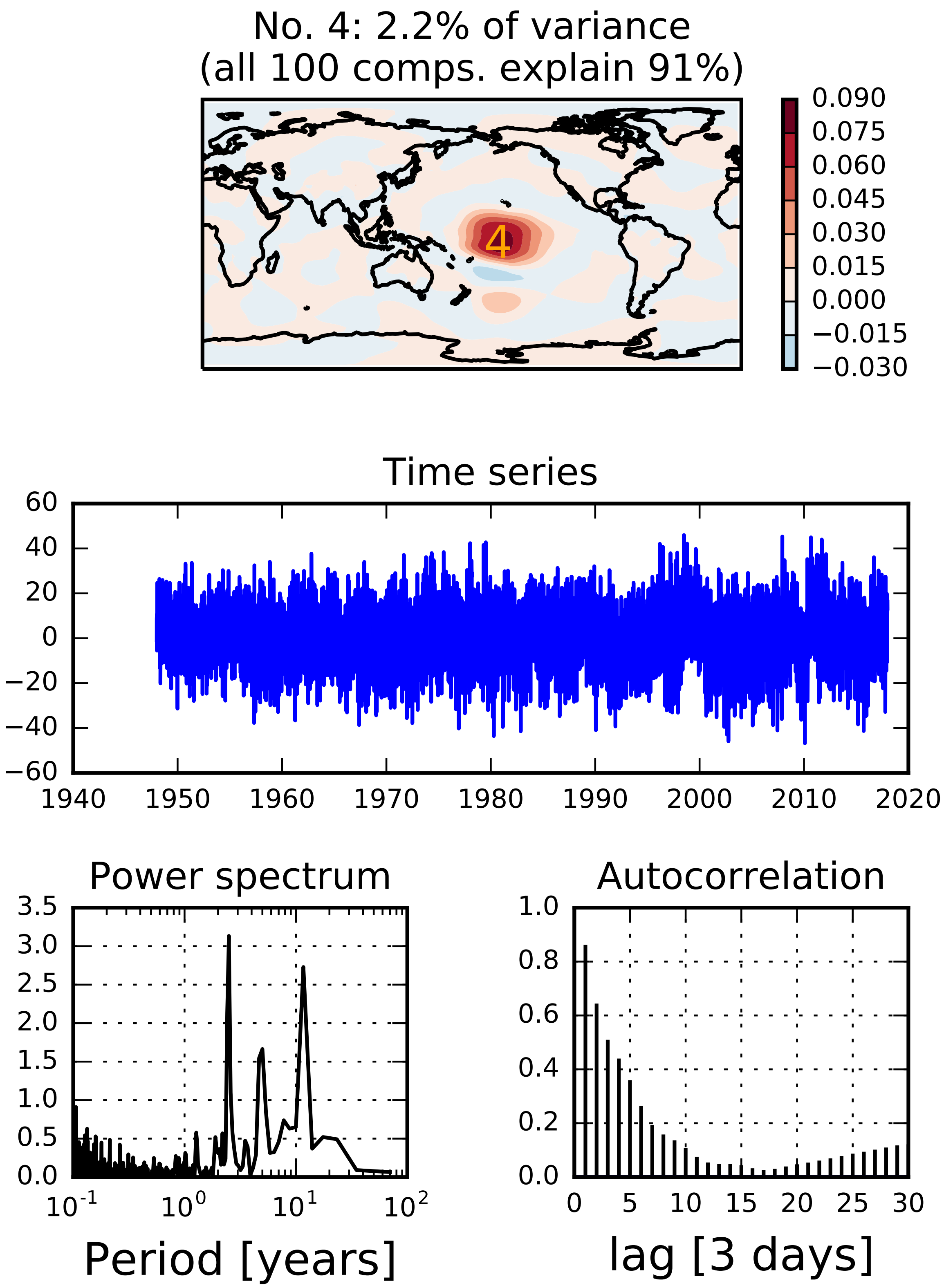
No. 4: 2.2% of variance
(all 100 comps. explain 91%)
4
0.090
0.075
0.060
0.045
0.030
0.015
0.000
−0.015
−0.030
Time series
60
40
20
0
−20
−40
−60
1940
1950
1960
1970
1980
1990
2000
2010
2020
Power spectrum
3.5
3.0
2.5
2.0
1.5
1.0
0.5
0.0
10^{-1}
10^{0}
10^{1}
10^{2}
Period [years]
Autocorrelation
1.0
0.8
0.6
0.4
0.2
0.0
0
5
10
15
20
25
30
lag [3 days]

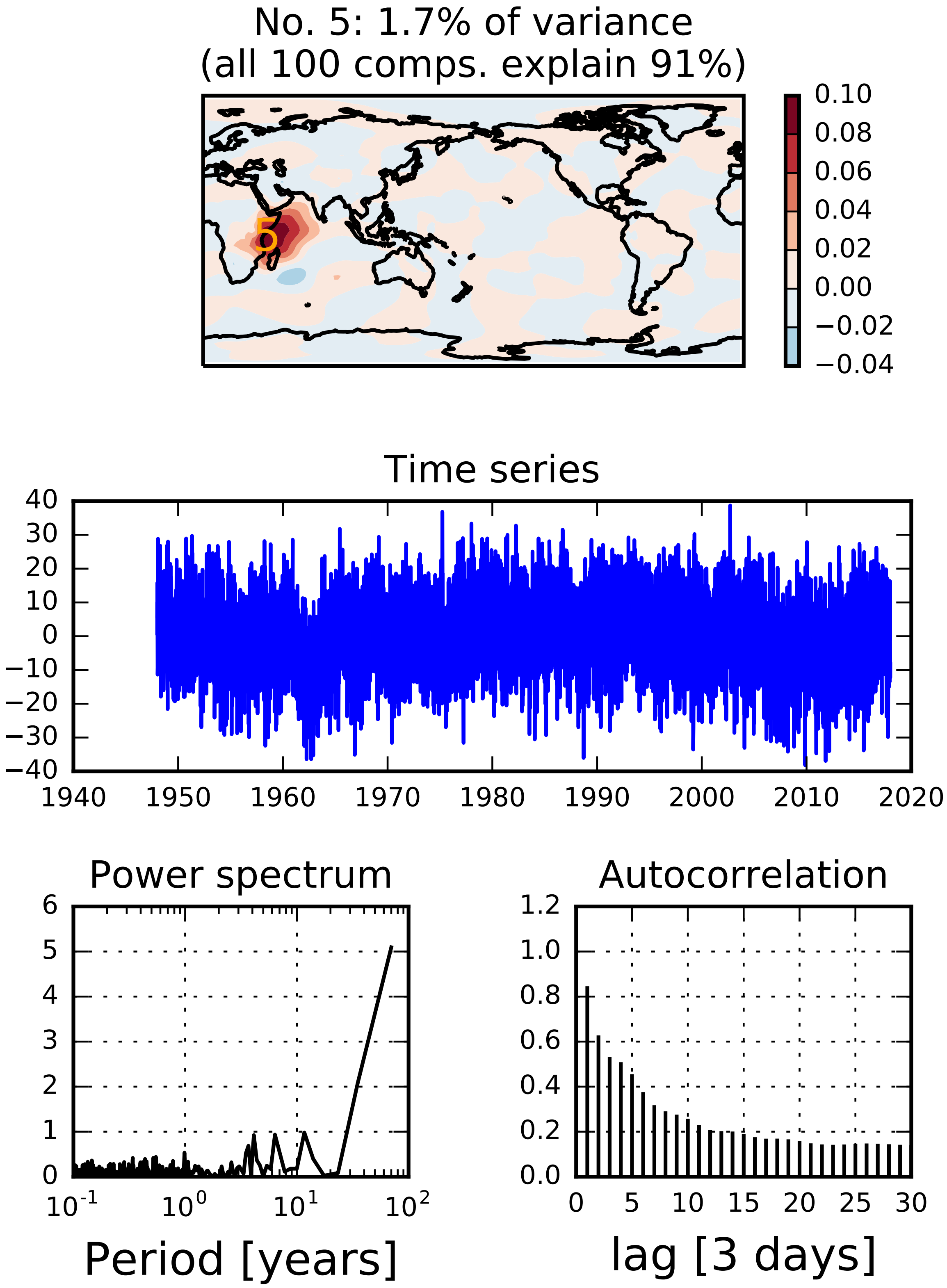
No. 5: 1.7% of variance
(all 100 comps. explain 91%)
5
0.10
0.08
0.06
0.04
0.02
0.00
−0.02
−0.04
Time series
40
30
20
10
0
−10
−20
−30
−40
1940
1950
1960
1970
1980
1990
2000
2010
2020
Power spectrum
6
5
4
3
2
1
0
10^-1
10^0
10^1
10^2
Period [years]
Autocorrelation
1.2
1.0
0.8
0.6
0.4
0.2
0.0
0
5
10
15
20
25
30
lag [3 days]

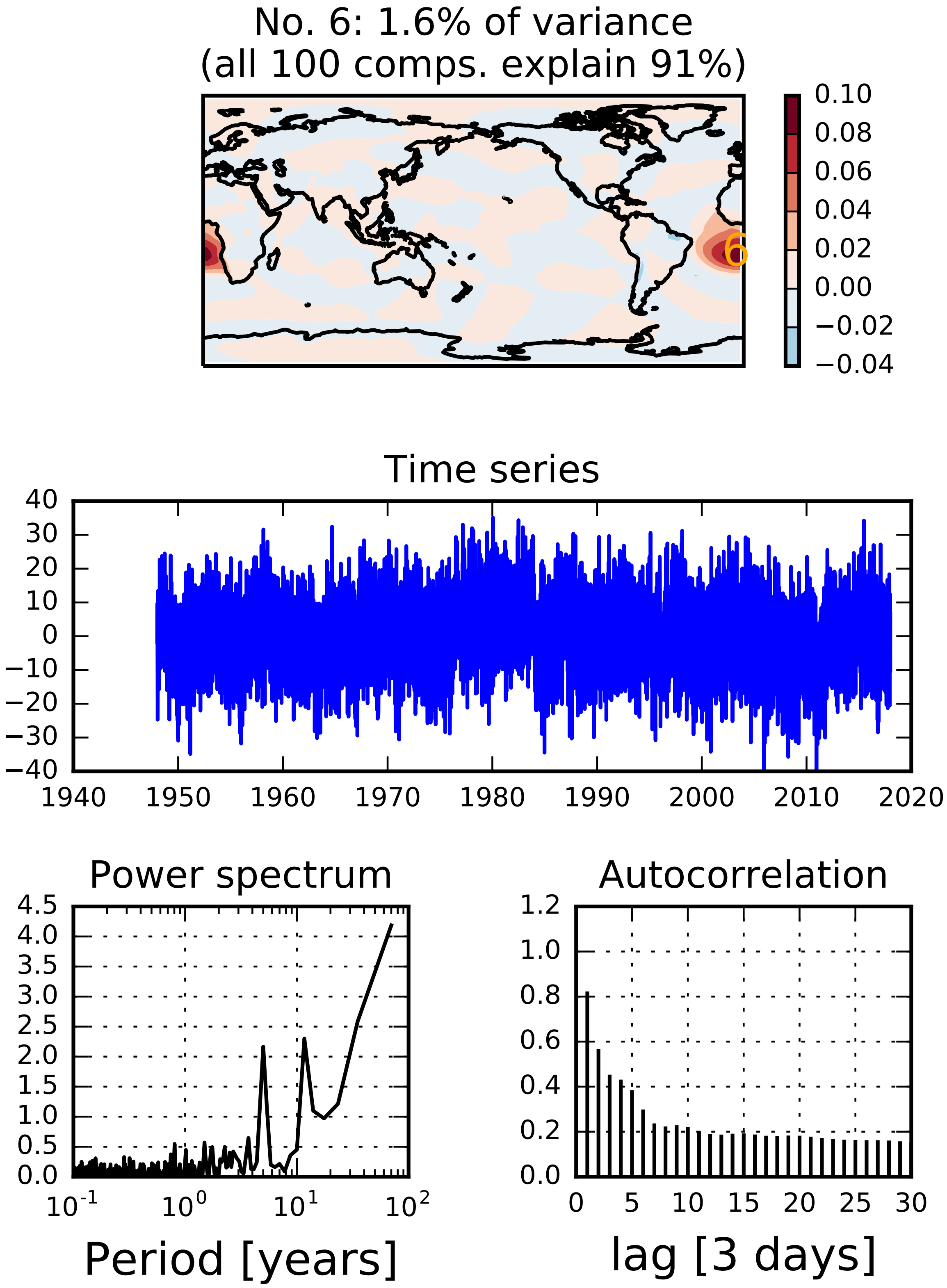
No. 6: 1.6% of variance
(all 100 comps. explain 91%)
6
0.10
0.08
0.06
0.04
0.02
0.00
−0.02
−0.04
Time series
40
30
20
10
0
−10
−20
−30
−40
1940
1950
1960
1970
1980
1990
2000
2010
2020
Power spectrum
4.5
4.0
3.5
3.0
2.5
2.0
1.5
1.0
0.5
0.0
10^{-1}
10^{0}
10^{1}
10^{2}
Period [years]
Autocorrelation
1.2
1.0
0.8
0.6
0.4
0.2
0.0
0
5
10
15
20
25
30
lag [3 days]

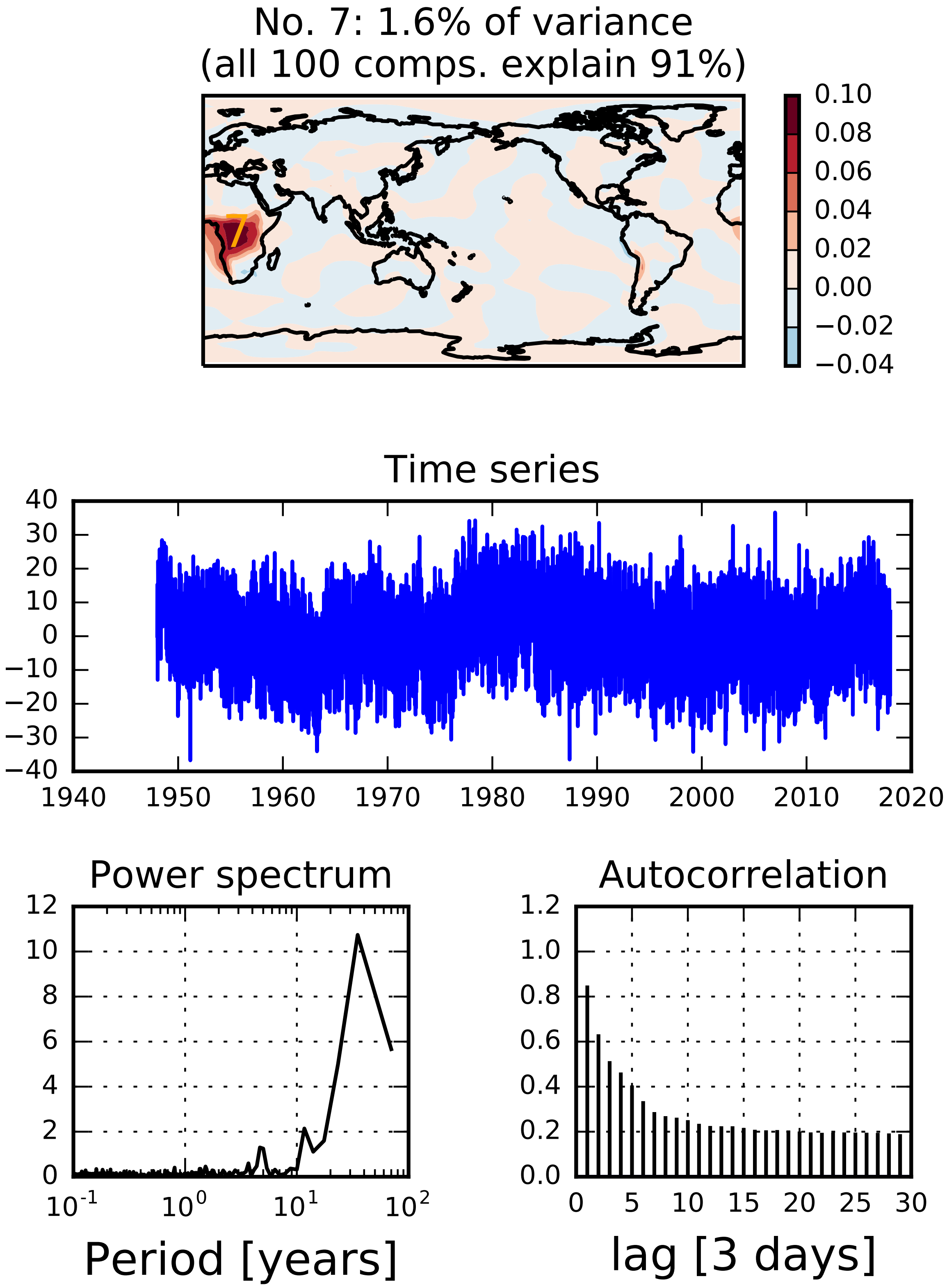
No. 7: 1.6% of variance
(all 100 comps. explain 91%)
0.10
0.08
0.06
0.04
0.02
0.00
−0.02
−0.04
7
Time series
40
30
20
10
0
−10
−20
−30
−40
1940
1950
1960
1970
1980
1990
2000
2010
2020
Power spectrum
12
10
8
6
4
2
0
10^-1
10^0
10^1
10^2
Period [years]
Autocorrelation
1.2
1.0
0.8
0.6
0.4
0.2
0.0
0
5
10
15
20
25
30
lag [3 days]

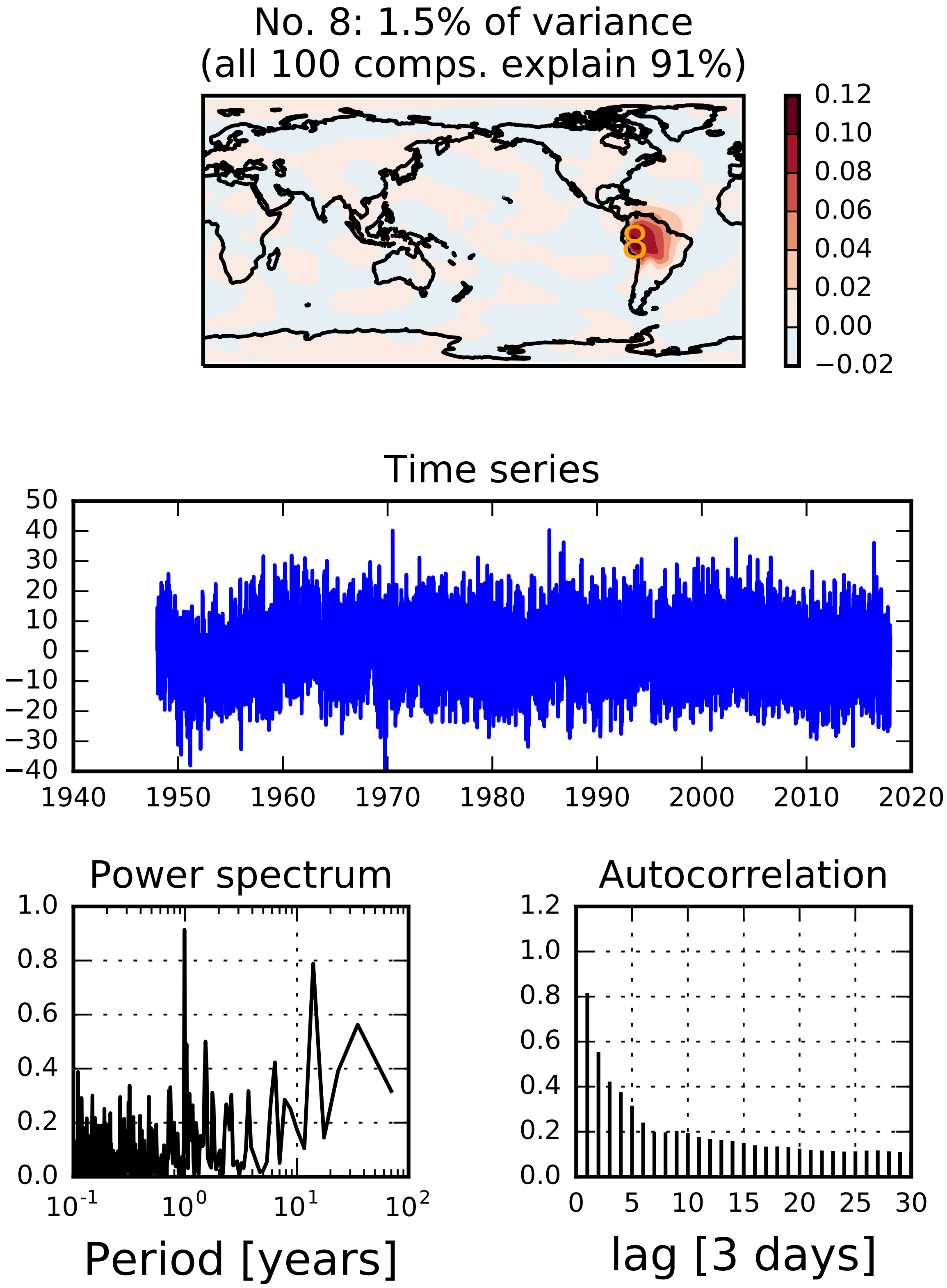
No. 8: 1.5% of variance
(all 100 comps. explain 91%)
8
0.12
0.10
0.08
0.06
0.04
0.02
0.00
−0.02
Time series
50
40
30
20
10
0
−10
−20
−30
−40
1940
1950
1960
1970
1980
1990
2000
2010
2020
Power spectrum
1.0
0.8
0.6
0.4
0.2
0.0
10^-1
10^0
10^1
10^2
Period [years]
Autocorrelation
1.2
1.0
0.8
0.6
0.4
0.2
0.0
0
5
10
15
20
25
30
lag [3 days]

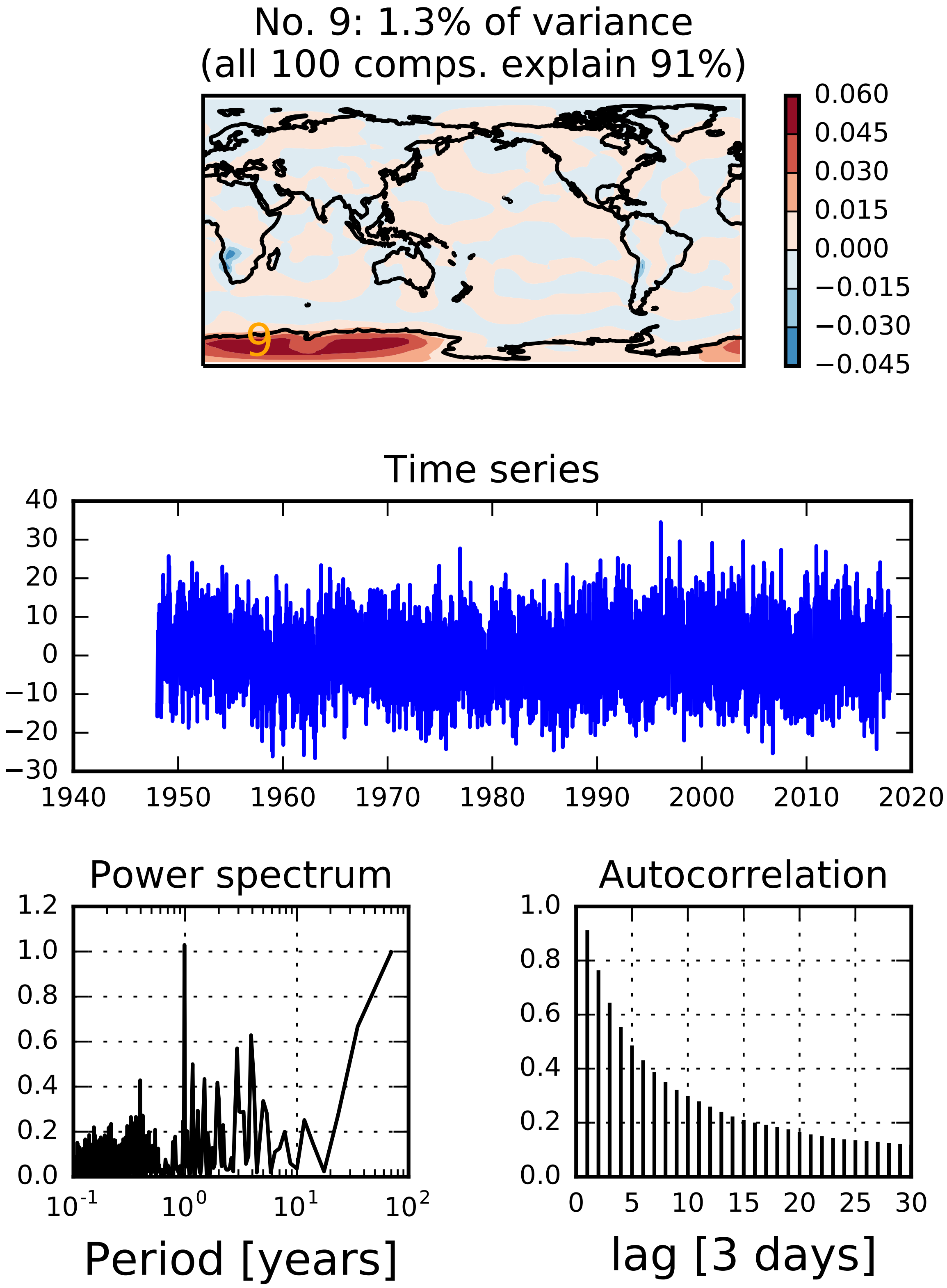
No. 9: 1.3% of variance
(all 100 comps. explain 91%)
0.060
0.045
0.030
0.015
0.000
−0.015
−0.030
−0.045
9
Time series
40
30
20
10
0
−10
−20
−30
1940
1950
1960
1970
1980
1990
2000
2010
2020
Power spectrum
1.2
1.0
0.8
0.6
0.4
0.2
0.0
10^{-1}
10^{0}
10^{1}
10^{2}
Period [years]
Autocorrelation
1.0
0.8
0.6
0.4
0.2
0.0
0
5
10
15
20
25
30
lag [3 days]

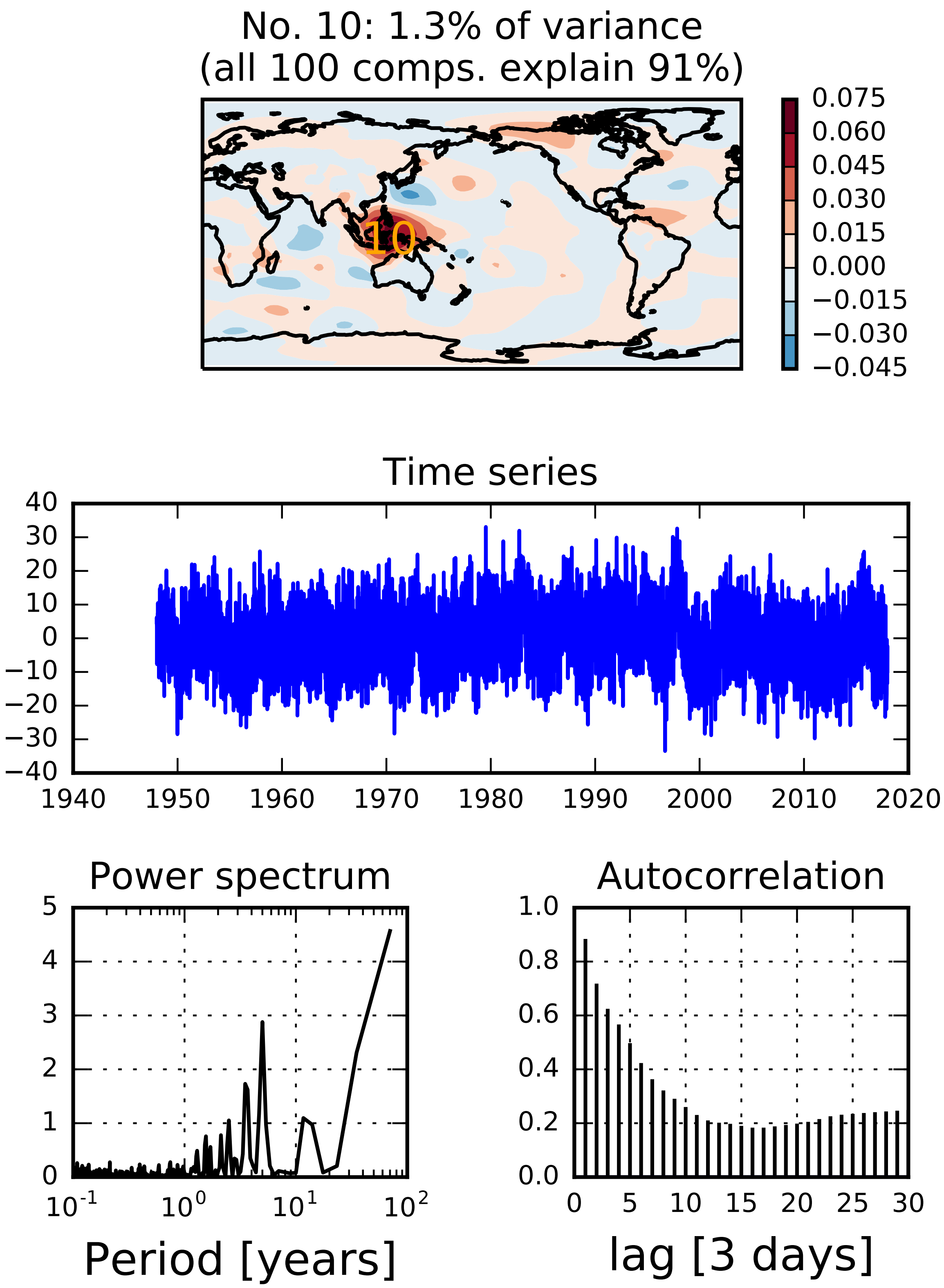
No. 10: 1.3% of variance
(all 100 comps. explain 91%)
10
0.075
0.060
0.045
0.030
0.015
0.000
−0.015
−0.030
−0.045
Time series
40
30
20
10
0
−10
−20
−30
−40
1940 1950 1960 1970 1980 1990 2000 2010 2020
Power spectrum
5 4 3 2 1 0
10^-1 10^0 10^1 10^2
Period [years]
Autocorrelation
1.0 0.8 0.6 0.4 0.2 0.0
0 5 10 15 20 25 30
lag [3 days]

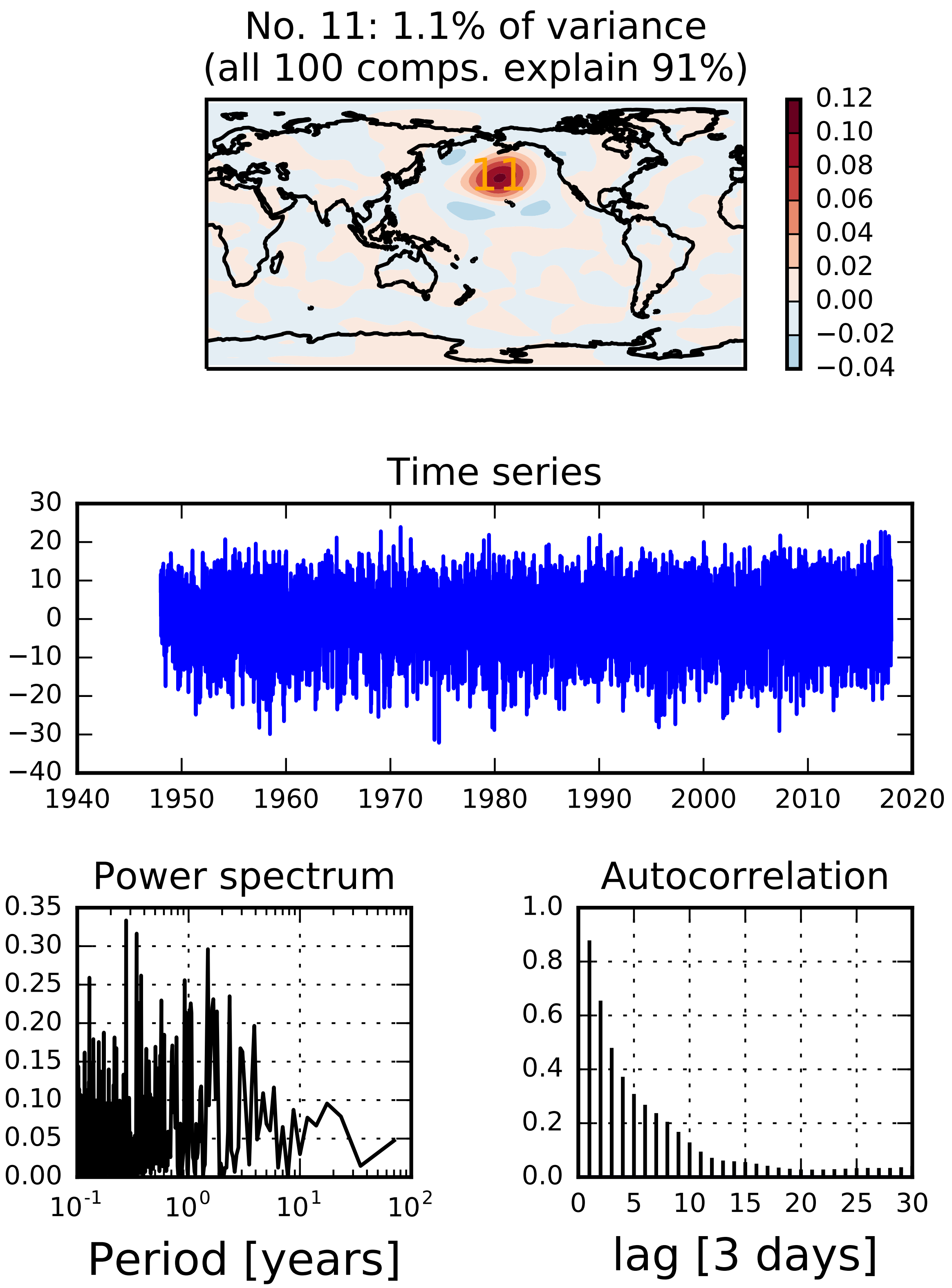
No. 11: 1.1% of variance
(all 100 comps. explain 91%)
11
0.12
0.10
0.08
0.06
0.04
0.02
0.00
−0.02
−0.04
Time series
30
20
10
0
−10
−20
−30
−40
1940
1950
1960
1970
1980
1990
2000
2010
2020
Power spectrum
0.35
0.30
0.25
0.20
0.15
0.10
0.05
0.00
10^-1
10^0
10^1
10^2
Period [years]
Autocorrelation
1.0
0.8
0.6
0.4
0.2
0.0
0
5
10
15
20
25
30
lag [3 days]

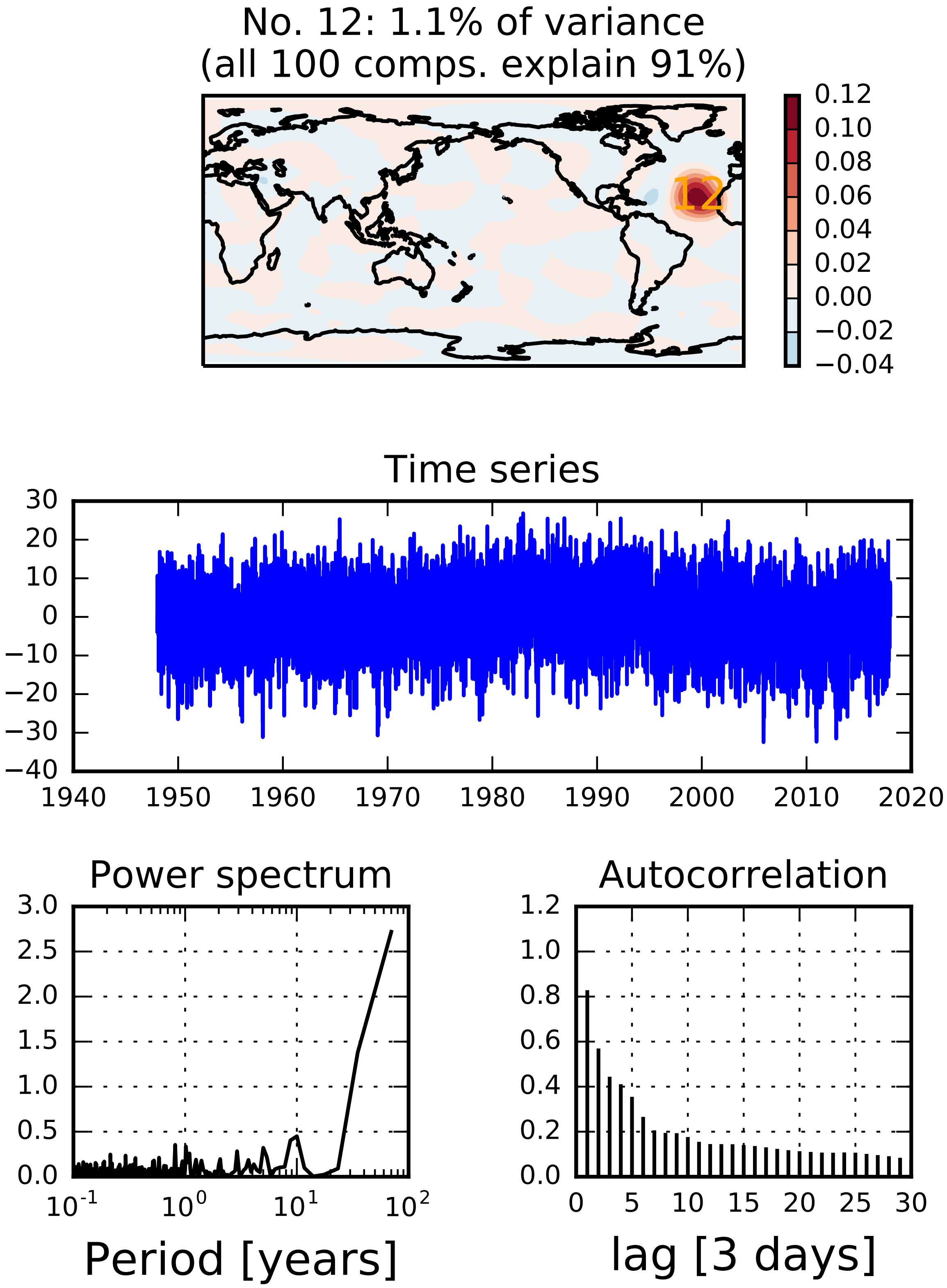
No. 12: 1.1% of variance
(all 100 comps. explain 91%)
12
0.12
0.10
0.08
0.06
0.04
0.02
0.00
−0.02
−0.04
Time series
30
20
10
0
−10
−20
−30
−40
1940
1950
1960
1970
1980
1990
2000
2010
2020
Power spectrum
3.0
2.5
2.0
1.5
1.0
0.5
0.0
10^{-1}
10^{0}
10^{1}
10^{2}
Period [years]
Autocorrelation
1.2
1.0
0.8
0.6
0.4
0.2
0.0
0
5
10
15
20
25
30
lag [3 days]

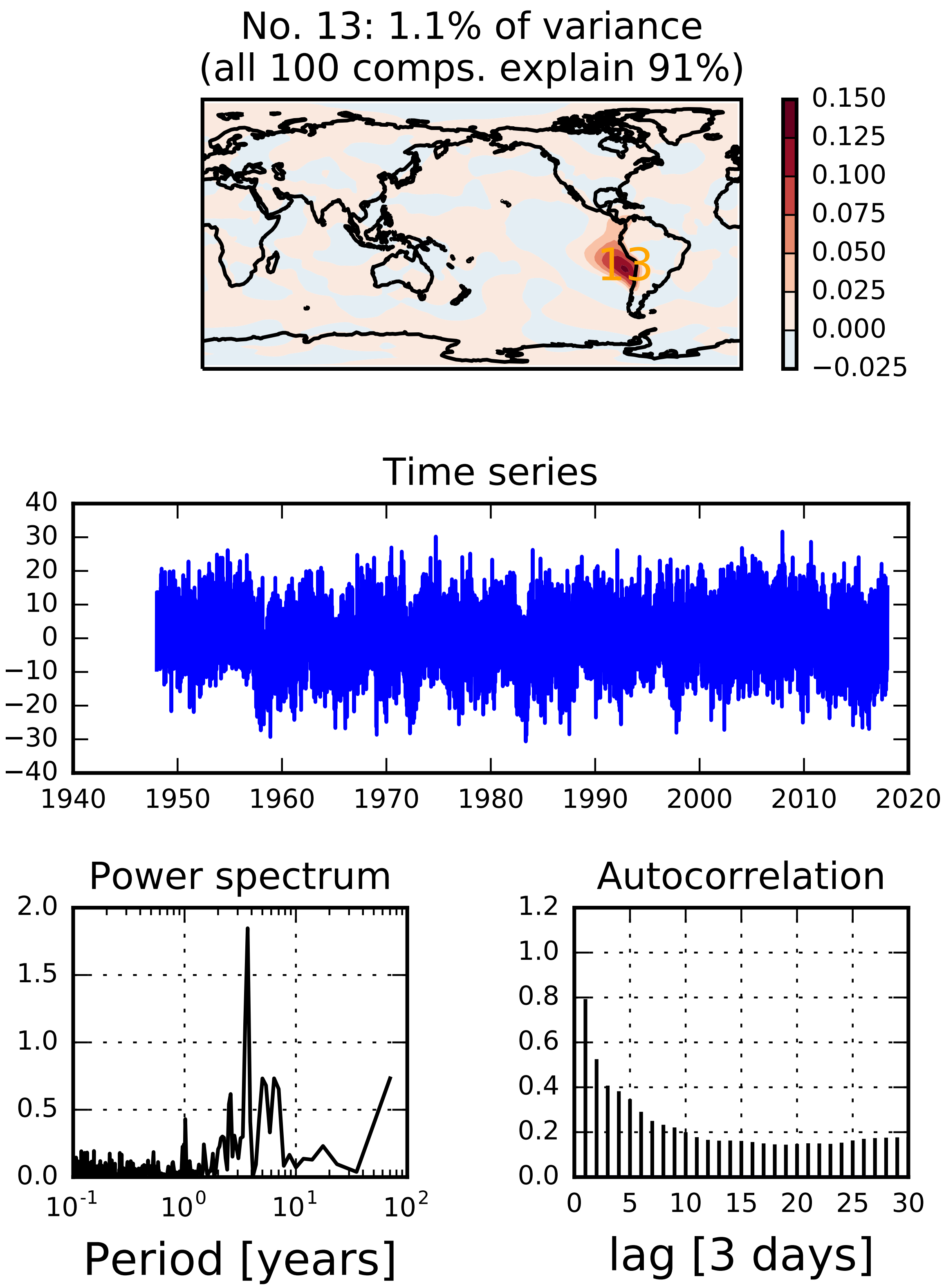
No. 13: 1.1% of variance
(all 100 comps. explain 91%)
13
0.150
0.125
0.100
0.075
0.050
0.025
0.000
−0.025
Time series
40
30
20
10
0
−10
−20
−30
−40
1940
1950
1960
1970
1980
1990
2000
2010
2020
Power spectrum
2.0
1.5
1.0
0.5
0.0
10^{-1}
10^{0}
10^{1}
10^{2}
Period [years]
Autocorrelation
1.2
1.0
0.8
0.6
0.4
0.2
0.0
0
5
10
15
20
25
30
lag [3 days]

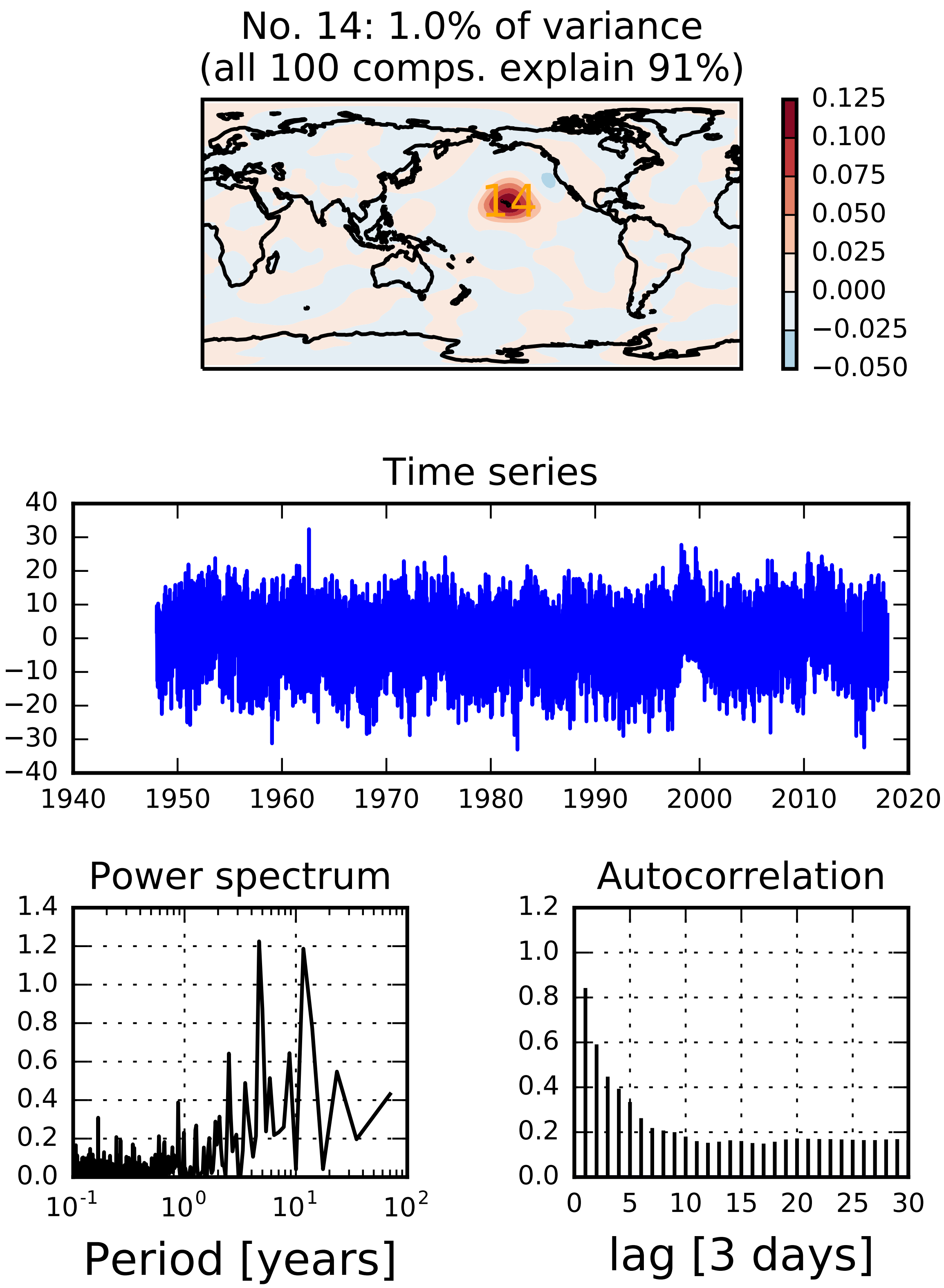
No. 14: 1.0% of variance
(all 100 comps. explain 91%)
14
0.125
0.100
0.075
0.050
0.025
0.000
−0.025
−0.050
Time series
40
30
20
10
0
−10
−20
−30
−40
1940
1950
1960
1970
1980
1990
2000
2010
2020
Power spectrum
1.4
1.2
1.0
0.8
0.6
0.4
0.2
0.0
10^{-1}
10^{0}
10^{1}
10^{2}
Period [years]
Autocorrelation
1.2
1.0
0.8
0.6
0.4
0.2
0.0
0
5
10
15
20
25
30
lag [3 days]

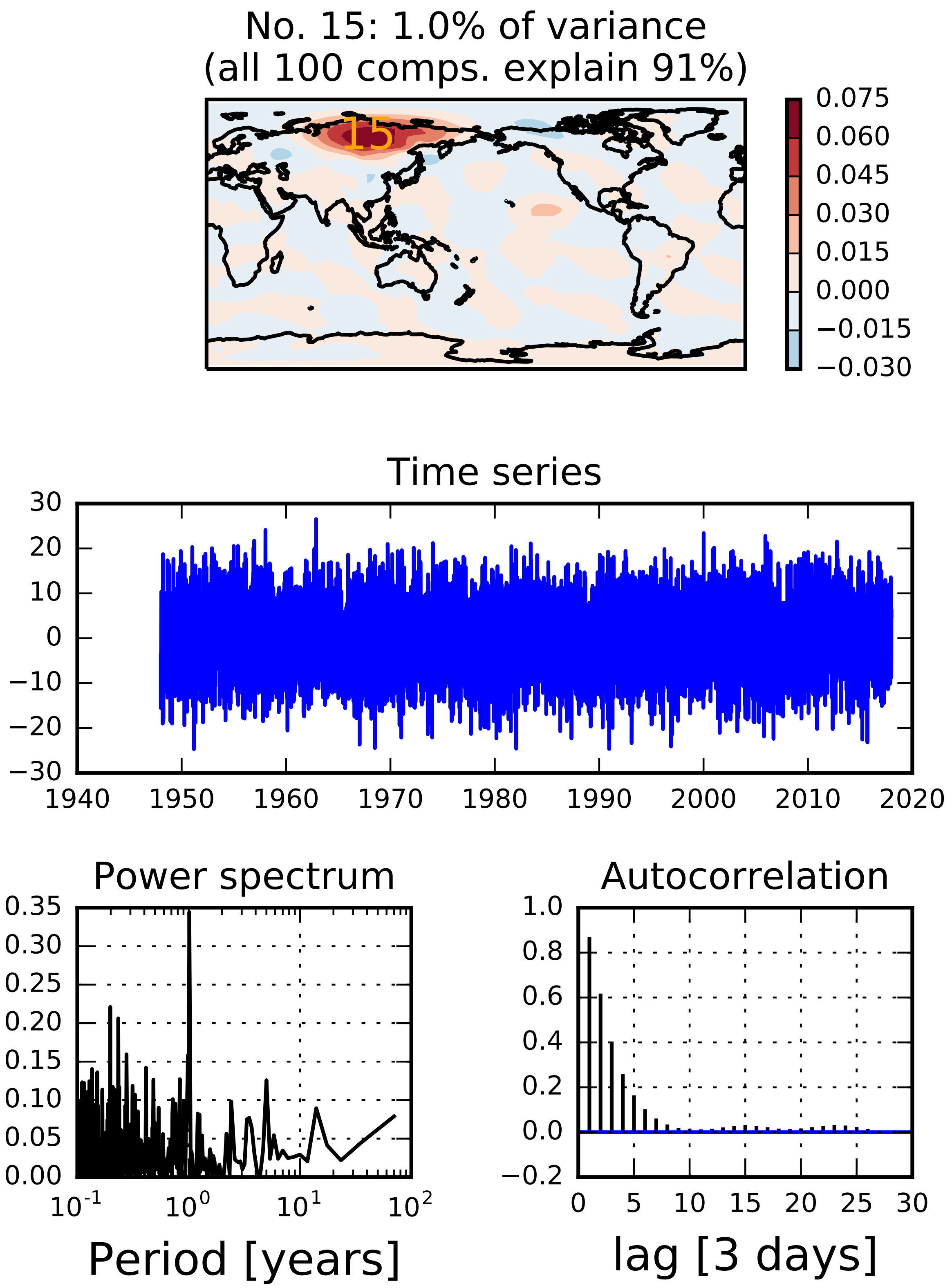
No. 15: 1.0% of variance
(all 100 comps. explain 91%)
15
0.075
0.060
0.045
0.030
0.015
0.000
−0.015
−0.030
Time series
Power spectrum
Period [years]
Autocorrelation
lag [3 days]

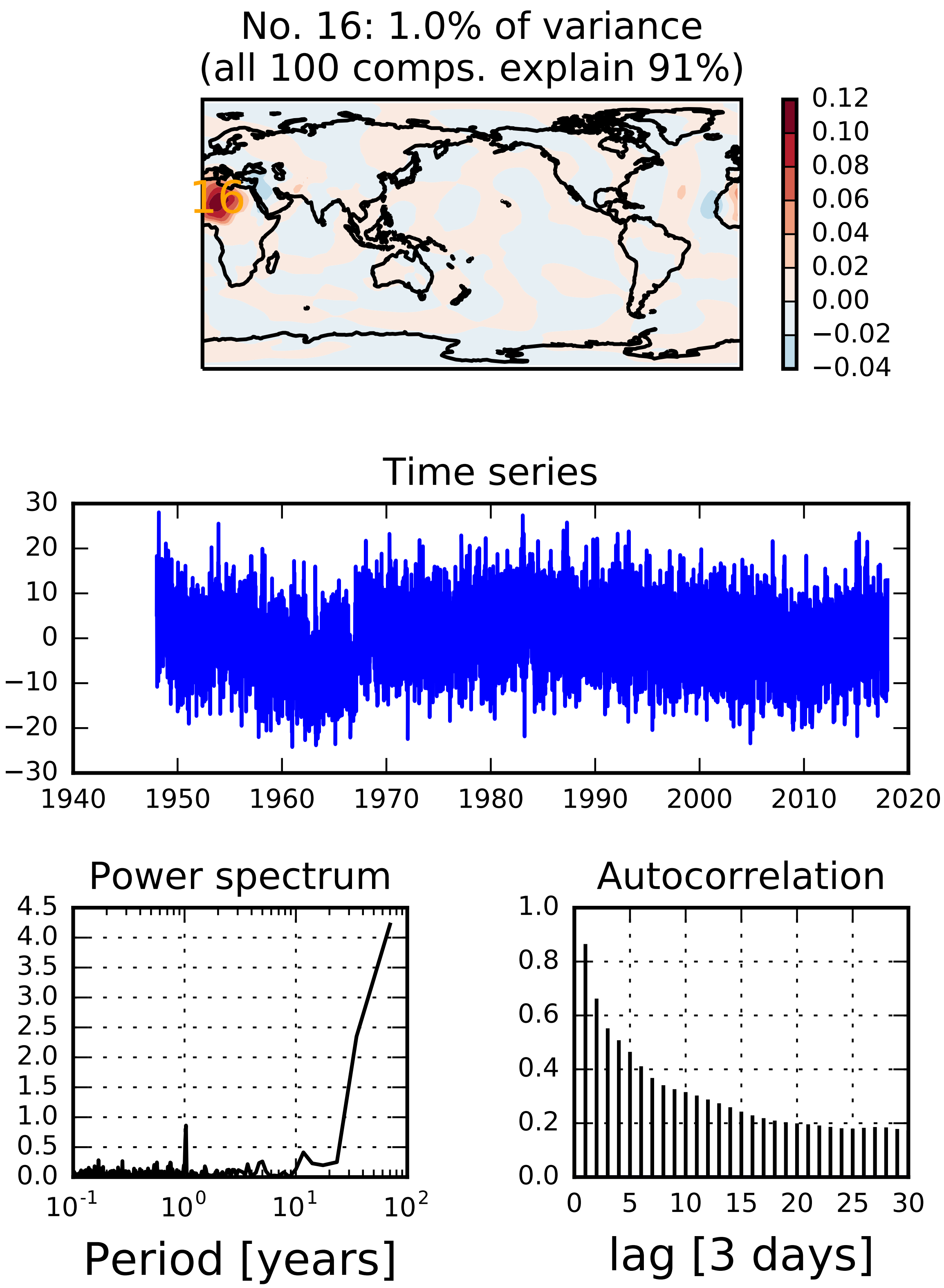
No. 16: 1.0% of variance
(all 100 comps. explain 91%)
16
0.12
0.10
0.08
0.06
0.04
0.02
0.00
−0.02
−0.04
Time series
30
20
10
0
−10
−20
−30
1940
1950
1960
1970
1980
1990
2000
2010
2020
Power spectrum
4.5
4.0
3.5
3.0
2.5
2.0
1.5
1.0
0.5
0.0
10^{-1}
10^{0}
10^{1}
10^{2}
Period [years]
Autocorrelation
1.0
0.8
0.6
0.4
0.2
0.0
0
5
10
15
20
25
30
lag [3 days]

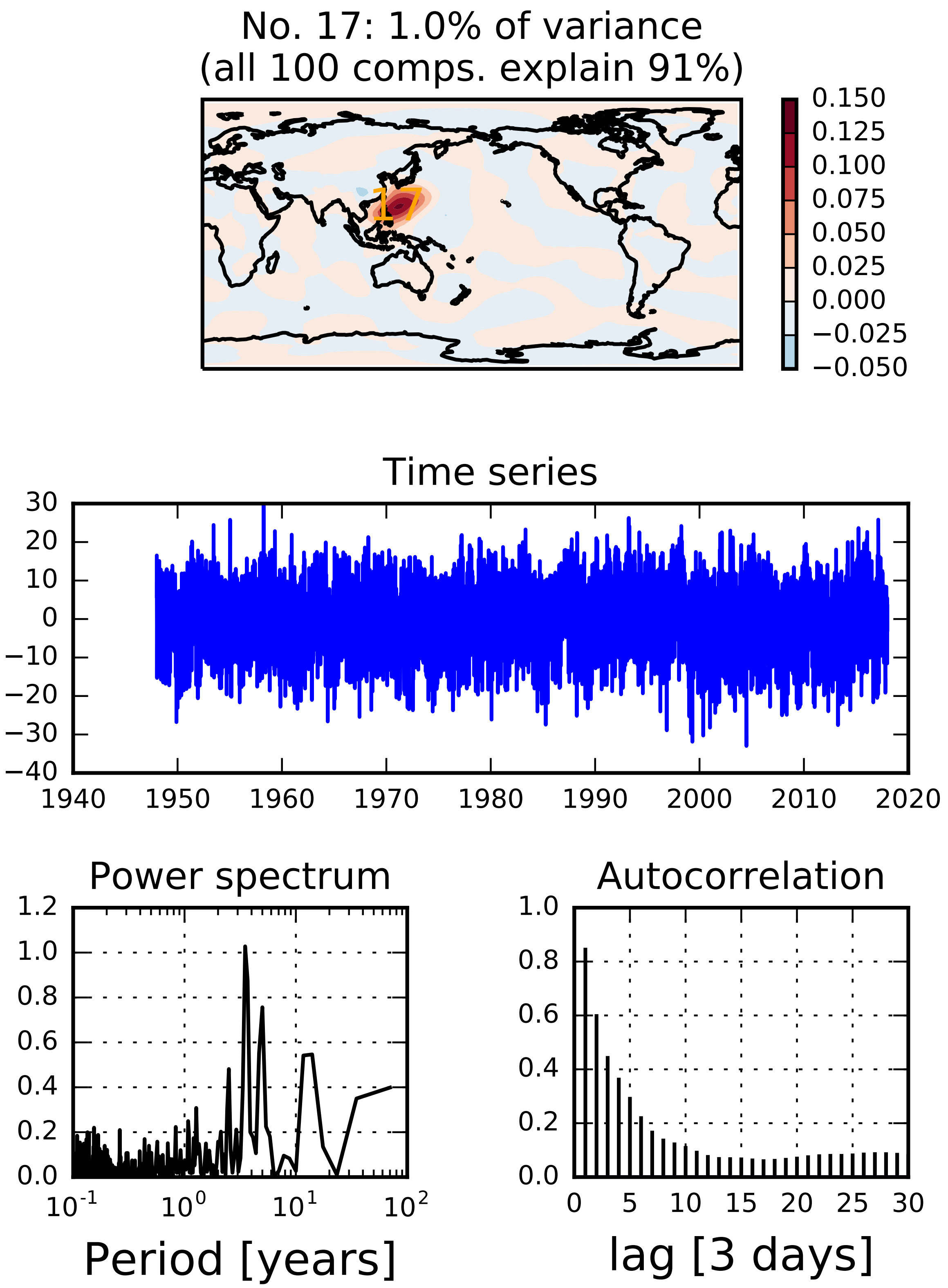
No. 17: 1.0% of variance
(all 100 comps. explain 91%)
0.150
0.125
0.100
0.075
0.050
0.025
0.000
−0.025
−0.050
17
Time series
30
20
10
0
−10
−20
−30
−40
1940
1950
1960
1970
1980
1990
2000
2010
2020
Power spectrum
1.2
1.0
0.8
0.6
0.4
0.2
0.0
10^-1
10^0
10^1
10^2
Period [years]
Autocorrelation
1.0
0.8
0.6
0.4
0.2
0.0
0
5
10
15
20
25
30
lag [3 days]

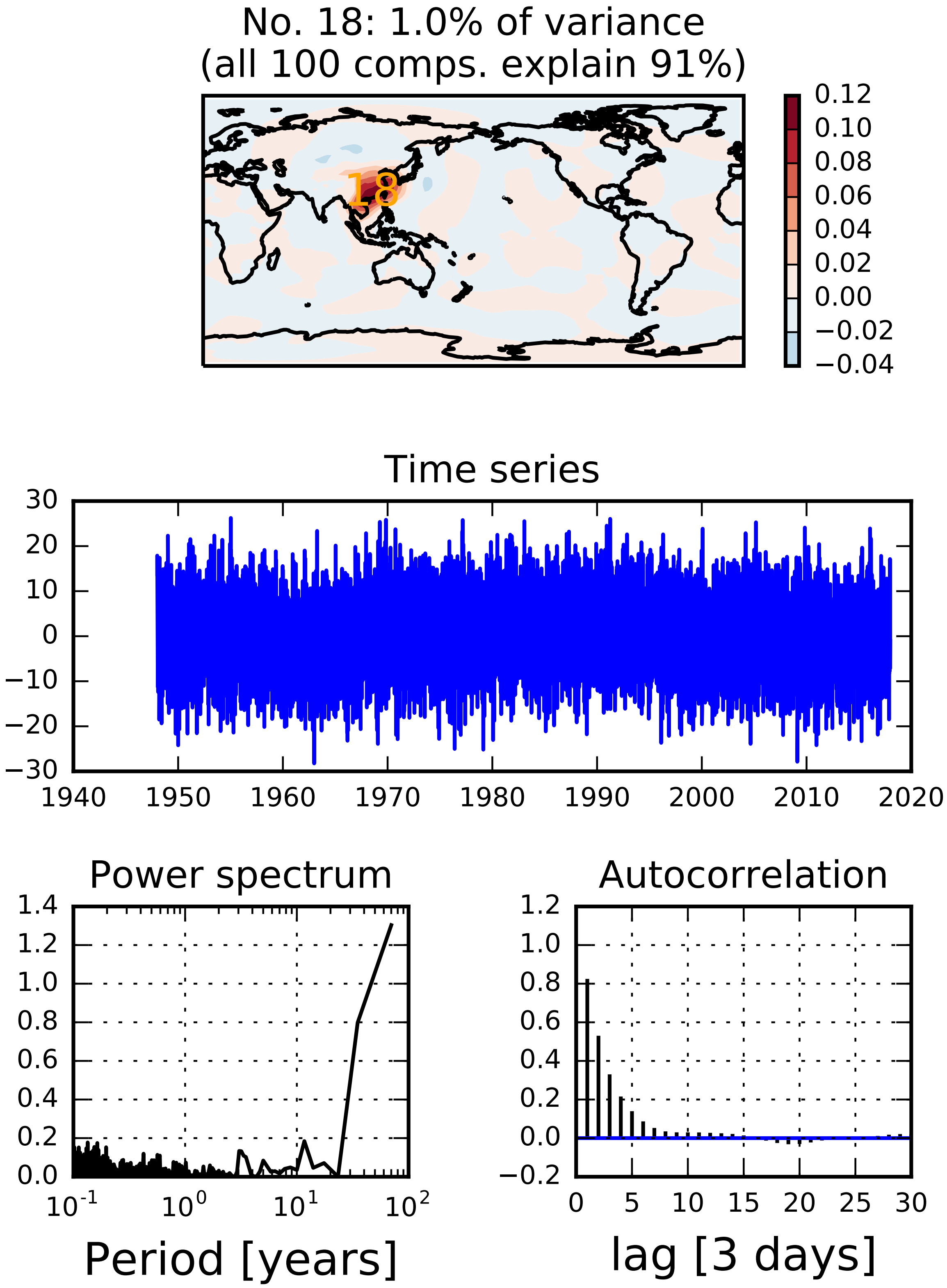
No. 18: 1.0% of variance
(all 100 comps. explain 91%)
18
0.12
0.10
0.08
0.06
0.04
0.02
0.00
−0.02
−0.04
Time series
30
20
10
0
−10
−20
−30
1940
1950
1960
1970
1980
1990
2000
2010
2020
Power spectrum
1.4
1.2
1.0
0.8
0.6
0.4
0.2
0.0
10^{-1}
10^{0}
10^{1}
10^{2}
Period [years]
Autocorrelation
1.2
1.0
0.8
0.6
0.4
0.2
0.0
−0.2
0
5
10
15
20
25
30
lag [3 days]

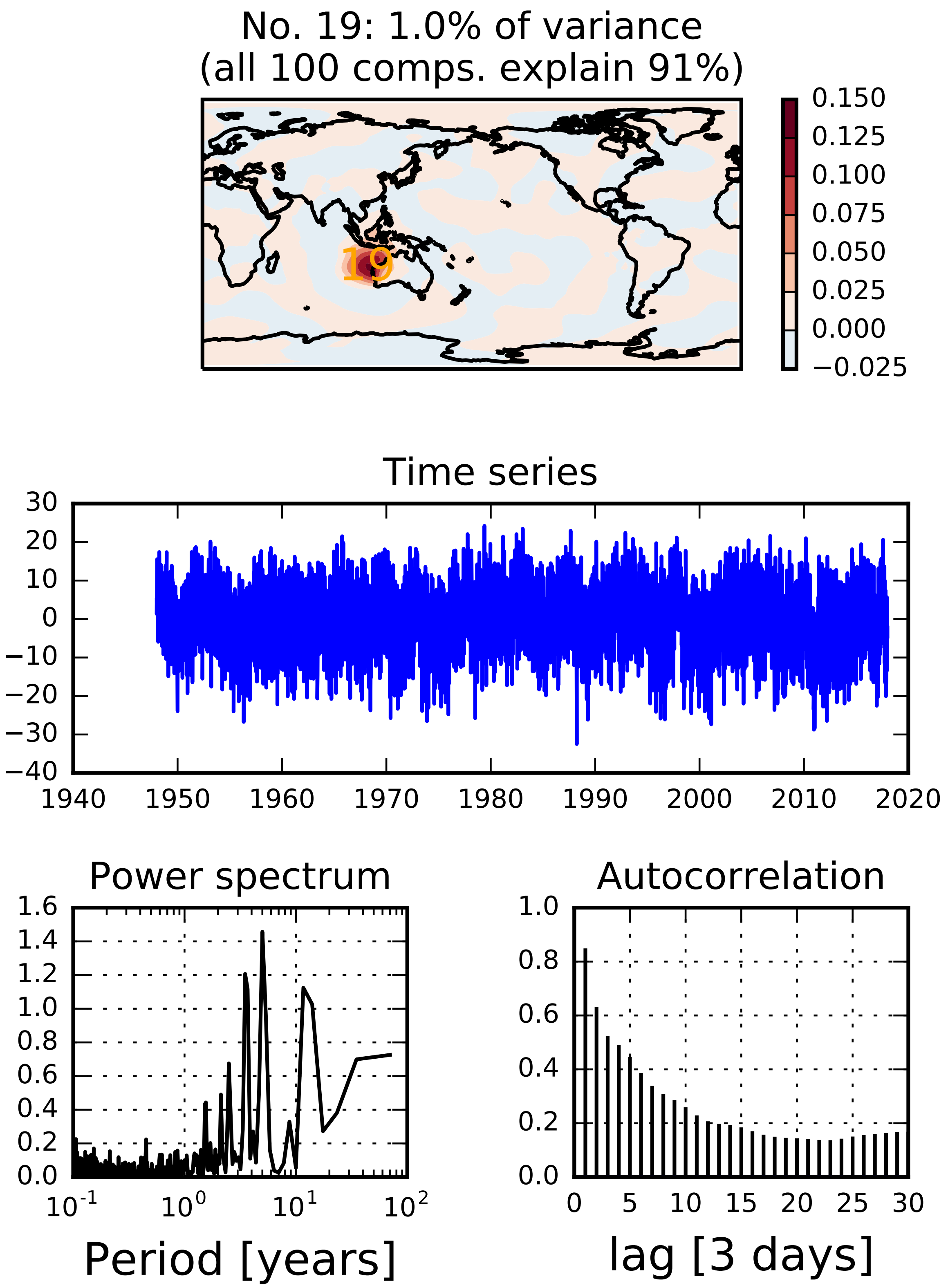
No. 19: 1.0% of variance
(all 100 comps. explain 91%)
19
0.150
0.125
0.100
0.075
0.050
0.025
0.000
−0.025
Time series
30
20
10
0
−10
−20
−30
−40
1940
1950
1960
1970
1980
1990
2000
2010
2020
Power spectrum
1.6
1.4
1.2
1.0
0.8
0.6
0.4
0.2
0.0
10^{-1}
10^{0}
10^{1}
10^{2}
Period [years]
Autocorrelation
1.0
0.8
0.6
0.4
0.2
0.0
0
5
10
15
20
25
30
lag [3 days]

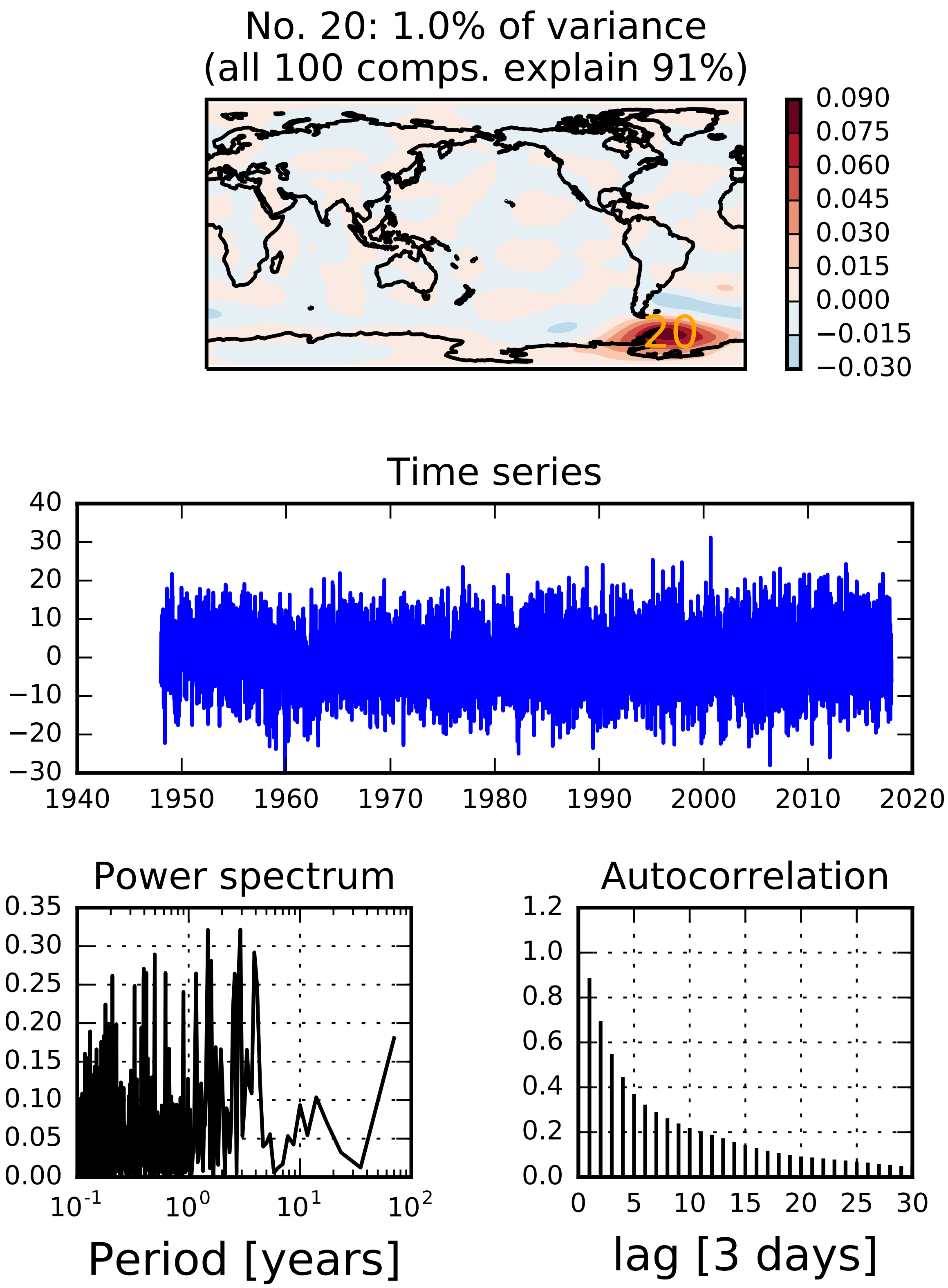
No. 20: 1.0% of variance
(all 100 comps. explain 91%)
20
0.090
0.075
0.060
0.045
0.030
0.015
0.000
−0.015
−0.030
Time series
40
30
20
10
0
−10
−20
−30
1940
1950
1960
1970
1980
1990
2000
2010
2020
Power spectrum
0.35
0.30
0.25
0.20
0.15
0.10
0.05
0.00
10^{-1}
10^{0}
10^{1}
10^{2}
Period [years]
Autocorrelation
1.2
1.0
0.8
0.6
0.4
0.2
0.0
0
5
10
15
20
25
30
lag [3 days]

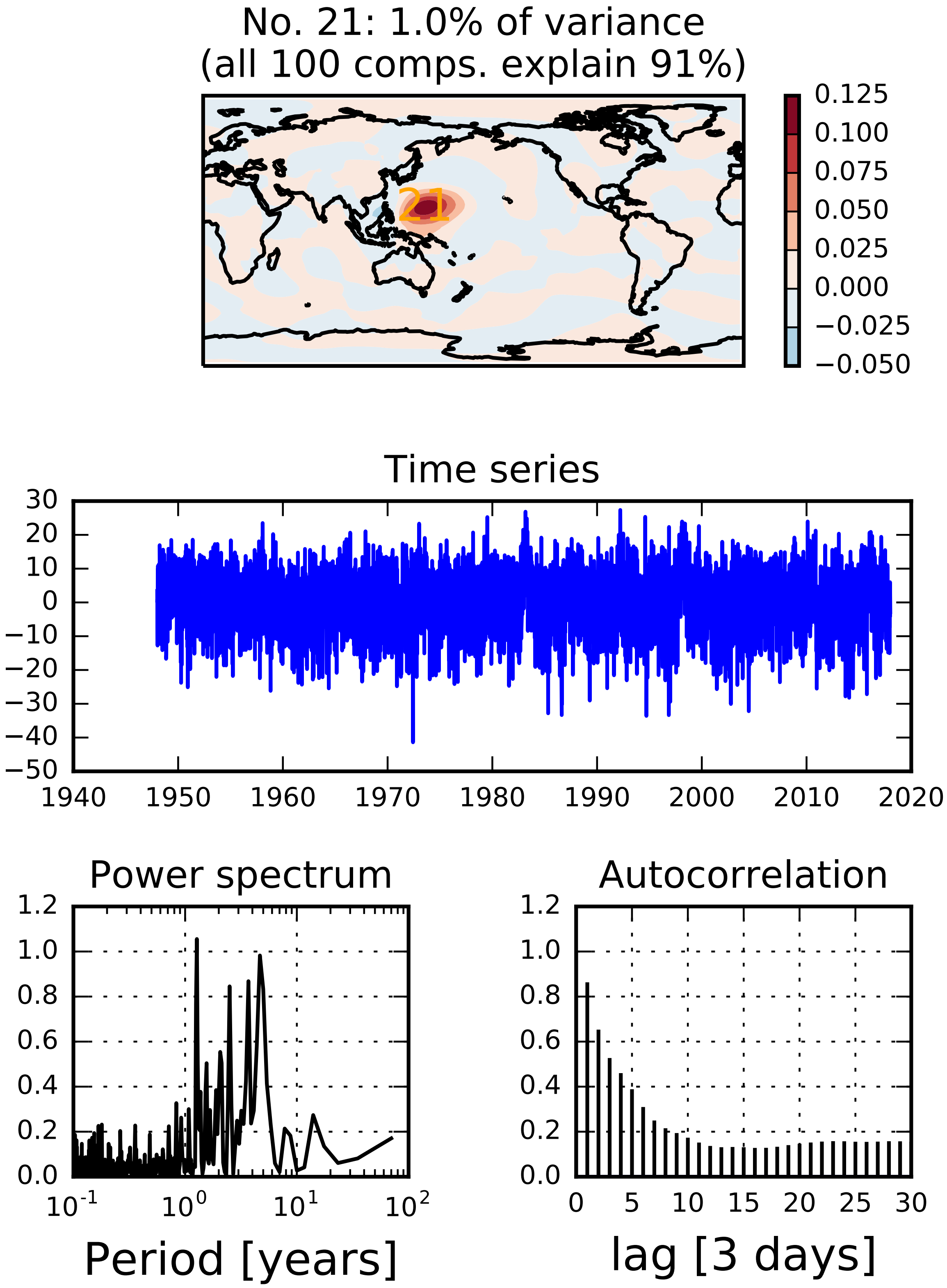
No. 21: 1.0% of variance
(all 100 comps. explain 91%)
21
0.125
0.100
0.075
0.050
0.025
0.000
−0.025
−0.050
Time series
30
20
10
0
−10
−20
−30
−40
−50
1940
1950
1960
1970
1980
1990
2000
2010
2020
Power spectrum
1.2
1.0
0.8
0.6
0.4
0.2
0.0
10^-1
10^0
10^1
10^2
Period [years]
Autocorrelation
1.2
1.0
0.8
0.6
0.4
0.2
0.0
0
5
10
15
20
25
30
lag [3 days]

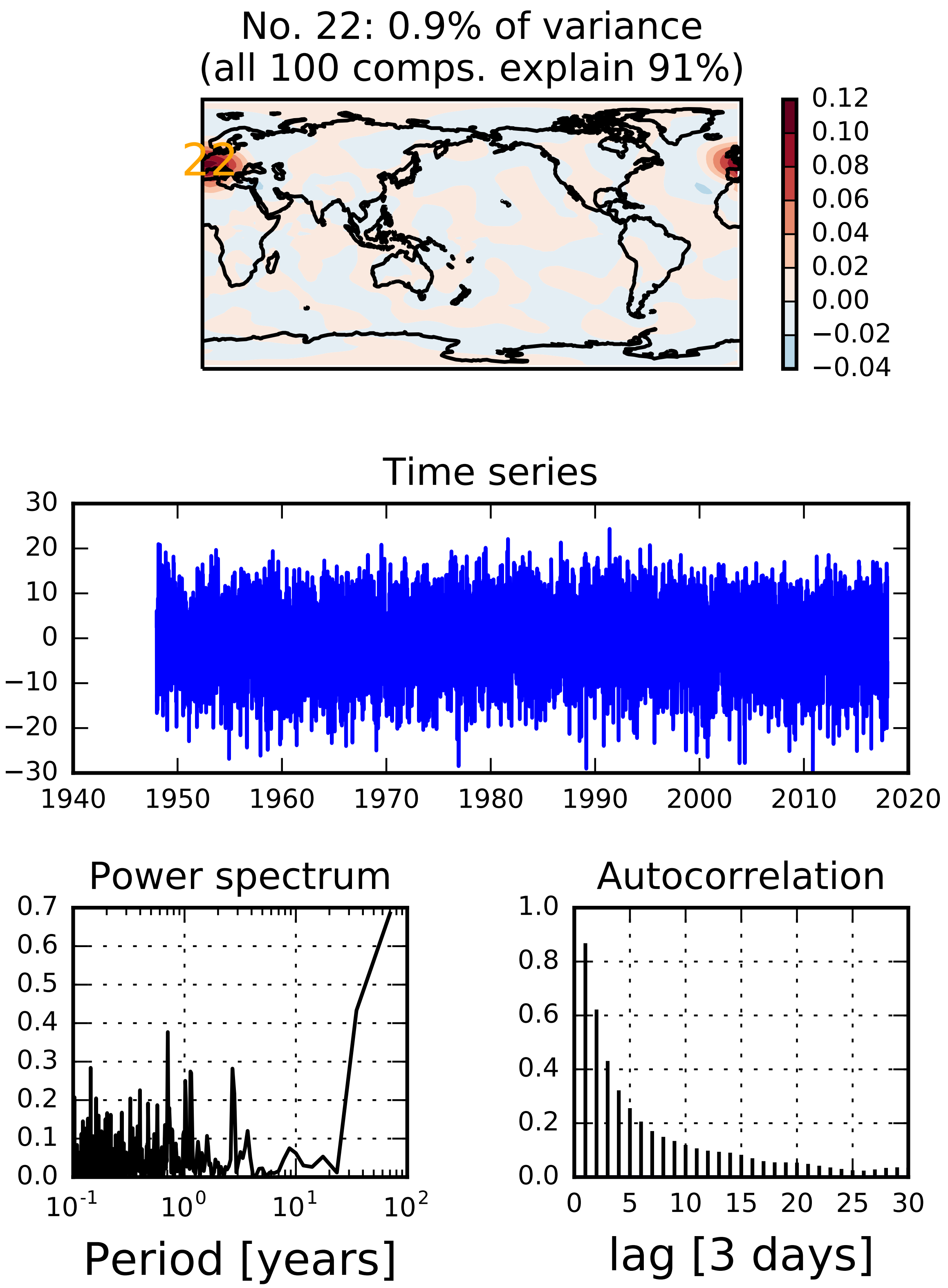
No. 22: 0.9% of variance
(all 100 comps. explain 91%)
22
0.12
0.10
0.08
0.06
0.04
0.02
0.00
−0.02
−0.04
Time series
30
20
10
0
−10
−20
−30
1940
1950
1960
1970
1980
1990
2000
2010
2020
Power spectrum
0.7
0.6
0.5
0.4
0.3
0.2
0.1
0.0
10^{-1}
10^{0}
10^{1}
10^{2}
Period [years]
Autocorrelation
1.0
0.8
0.6
0.4
0.2
0.0
0
5
10
15
20
25
30
lag [3 days]

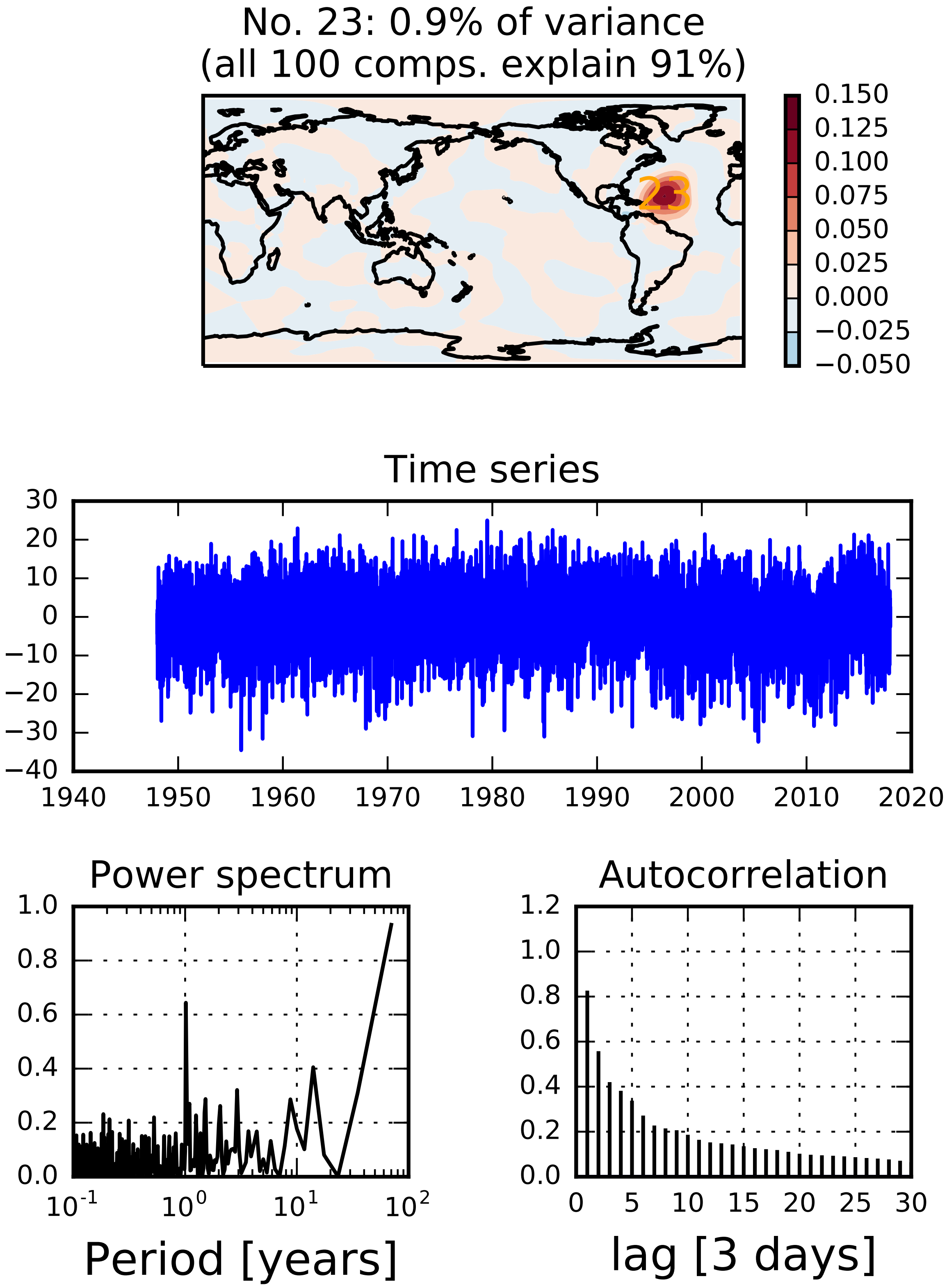
No. 23: 0.9% of variance
(all 100 comps. explain 91%)
23
0.150
0.125
0.100
0.075
0.050
0.025
0.000
−0.025
−0.050
Time series
30
20
10
0
−10
−20
−30
−40
1940
1950
1960
1970
1980
1990
2000
2010
2020
Power spectrum
1.0
0.8
0.6
0.4
0.2
0.0
10^{-1}
10^{0}
10^{1}
10^{2}
Period [years]
Autocorrelation
1.2
1.0
0.8
0.6
0.4
0.2
0.0
0
5
10
15
20
25
30
lag [3 days]

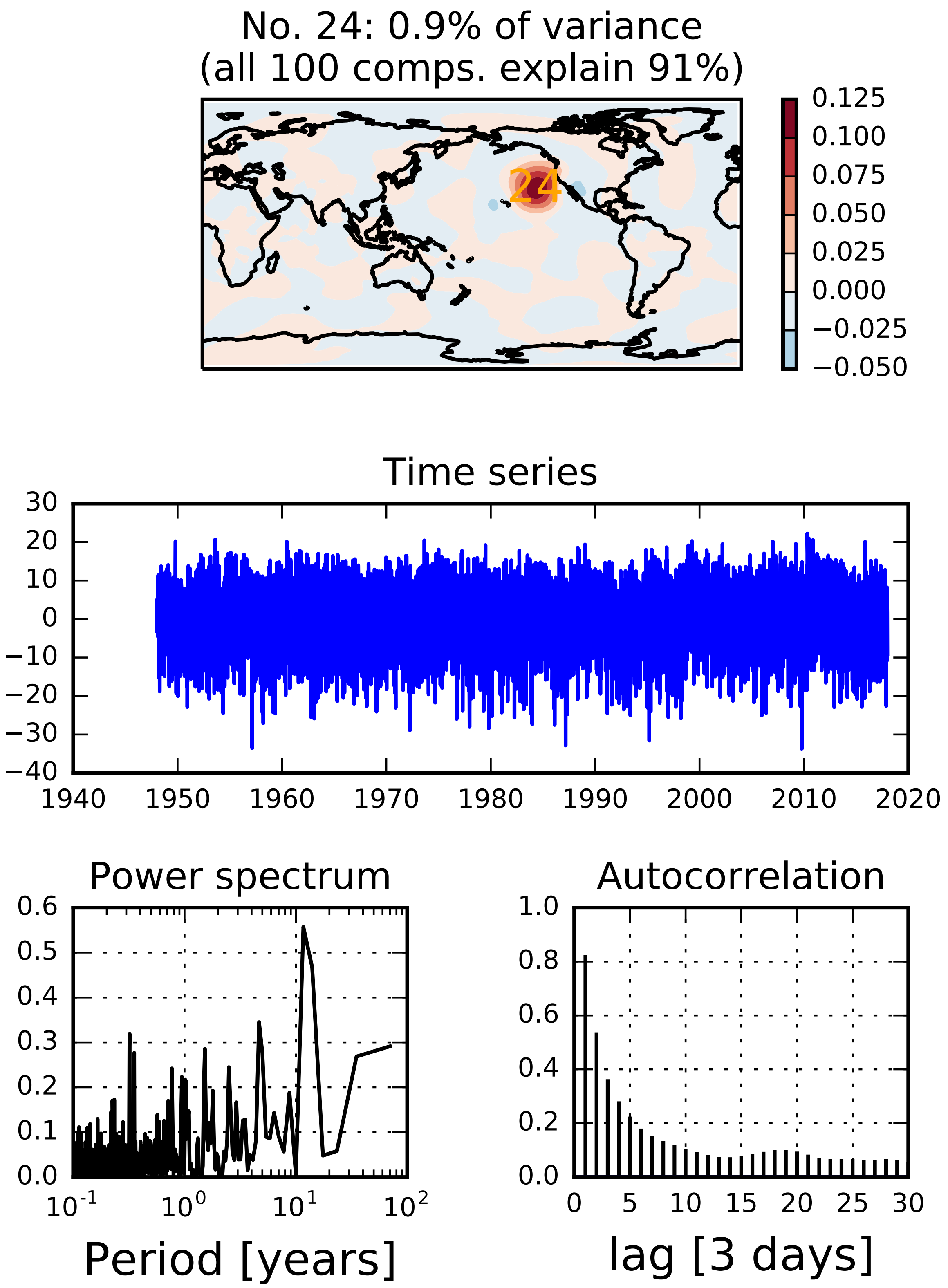
No. 24: 0.9% of variance
(all 100 comps. explain 91%)
24
0.125
0.100
0.075
0.050
0.025
0.000
−0.025
−0.050
Time series
30
20
10
0
−10
−20
−30
−40
1940
1950
1960
1970
1980
1990
2000
2010
2020
Power spectrum
0.6
0.5
0.4
0.3
0.2
0.1
0.0
10^{-1}
10^{0}
10^{1}
10^{2}
Period [years]
Autocorrelation
1.0
0.8
0.6
0.4
0.2
0.0
0
5
10
15
20
25
30
lag [3 days]

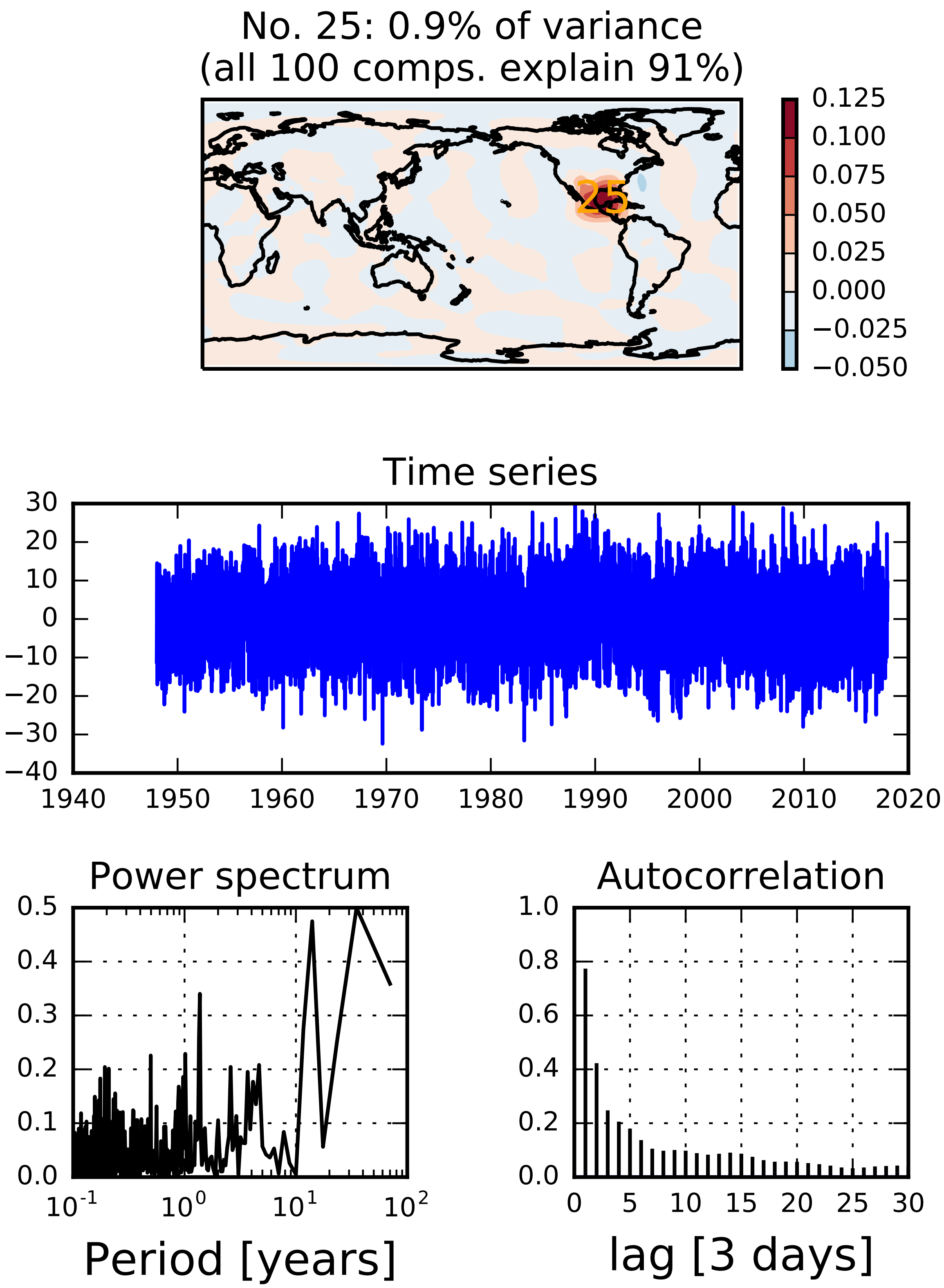
No. 25: 0.9% of variance
(all 100 comps. explain 91%)
25
0.125
0.100
0.075
0.050
0.025
0.000
−0.025
−0.050
Time series
30
20
10
0
−10
−20
−30
−40
1940
1950
1960
1970
1980
1990
2000
2010
2020
Power spectrum
0.5
0.4
0.3
0.2
0.1
0.0
10^{-1}
10^{0}
10^{1}
10^{2}
Period [years]
Autocorrelation
1.0
0.8
0.6
0.4
0.2
0.0
0
5
10
15
20
25
30
lag [3 days]

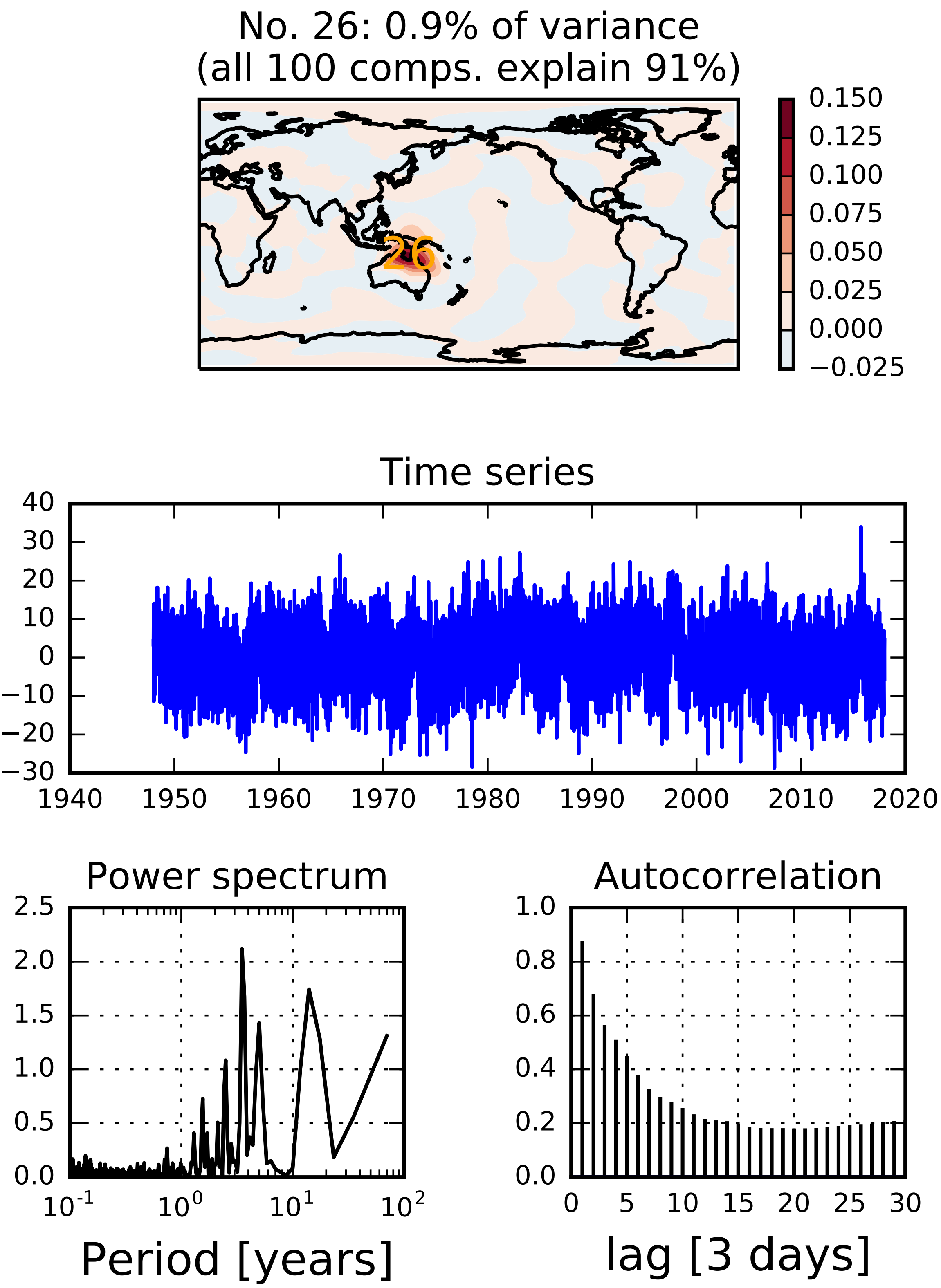
No. 26: 0.9% of variance
(all 100 comps. explain 91%)
26
0.150
0.125
0.100
0.075
0.050
0.025
0.000
−0.025
Time series
40
30
20
10
0
−10
−20
−30
1940
1950
1960
1970
1980
1990
2000
2010
2020
Power spectrum
2.5
2.0
1.5
1.0
0.5
0.0
10^{-1}
10^{0}
10^{1}
10^{2}
Period [years]
Autocorrelation
1.0
0.8
0.6
0.4
0.2
0.0
0
5
10
15
20
25
30
lag [3 days]

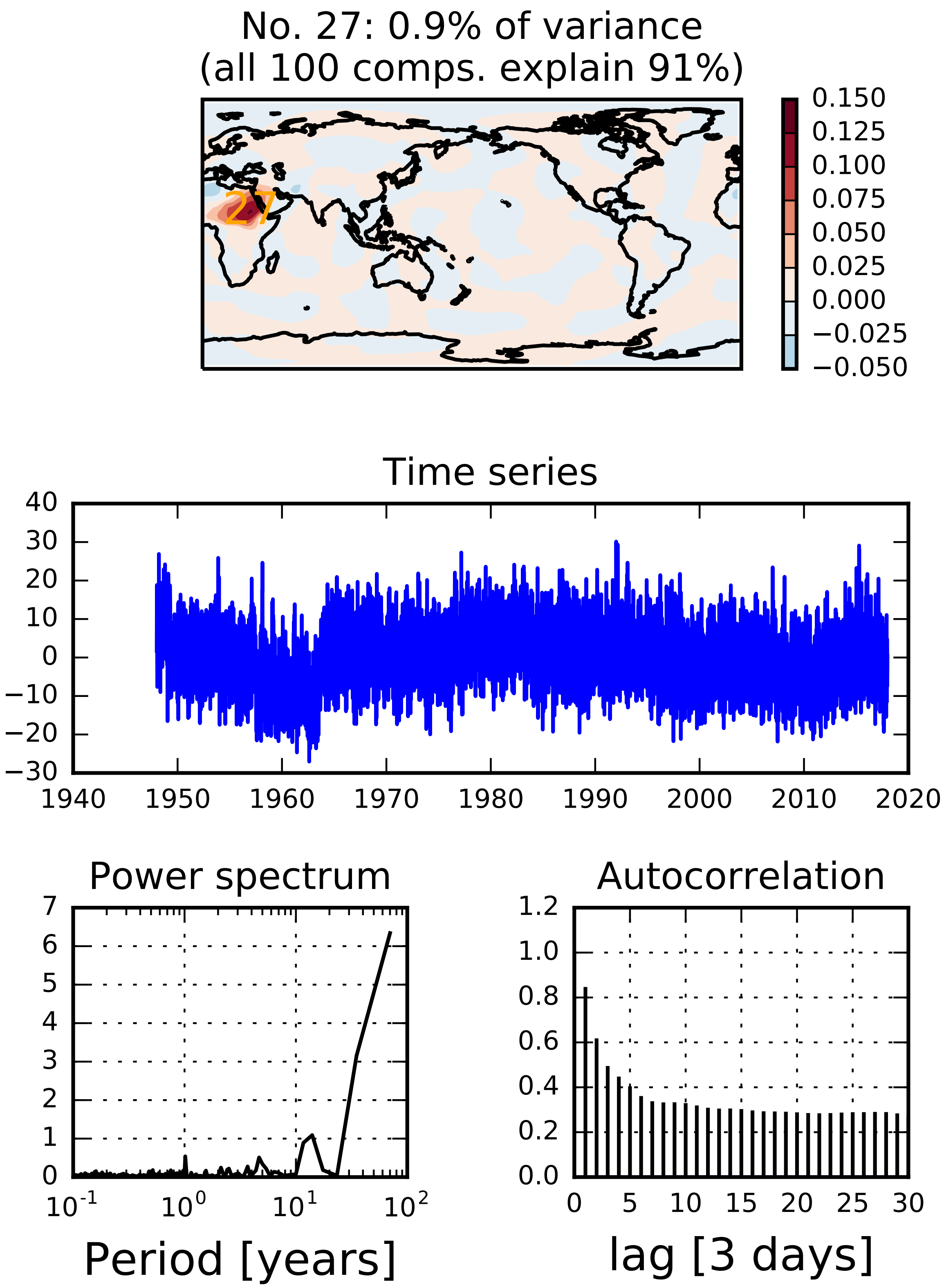
No. 27: 0.9% of variance
(all 100 comps. explain 91%)
27
0.150
0.125
0.100
0.075
0.050
0.025
0.000
−0.025
−0.050
Time series
40
30
20
10
0
−10
−20
−30
1940
1950
1960
1970
1980
1990
2000
2010
2020
Power spectrum
7
6
5
4
3
2
1
0
10^{-1}
10^{0}
10^{1}
10^{2}
Period [years]
Autocorrelation
1.2
1.0
0.8
0.6
0.4
0.2
0.0
0
5
10
15
20
25
30
lag [3 days]

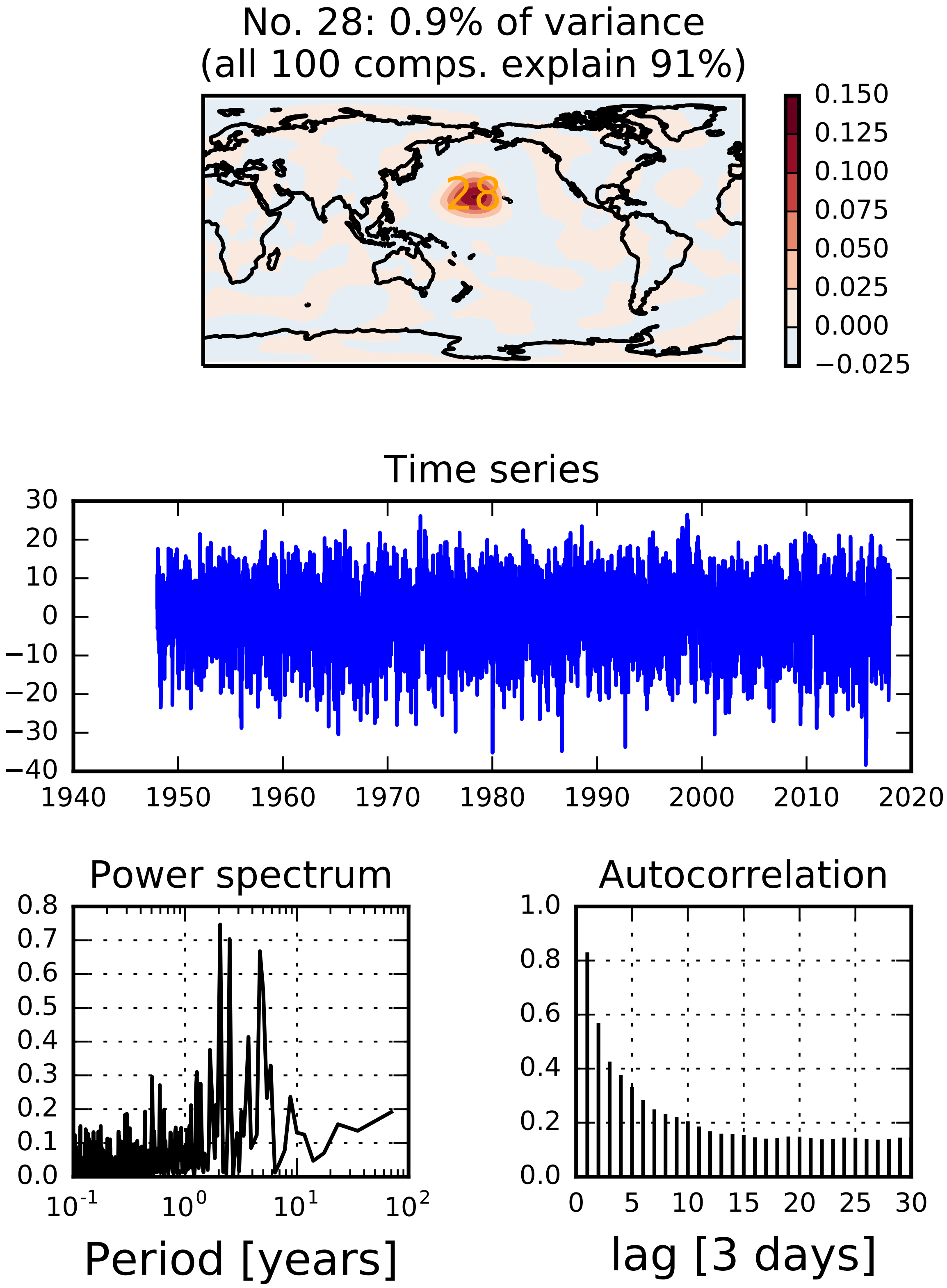
No. 28: 0.9% of variance
(all 100 comps. explain 91%)
0.150
0.125
0.100
0.075
0.050
0.025
0.000
−0.025
28
Time series
30
20
10
0
−10
−20
−30
−40
1940
1950
1960
1970
1980
1990
2000
2010
2020
Power spectrum
0.8
0.7
0.6
0.5
0.4
0.3
0.2
0.1
0.0
10^-1
10^0
10^1
10^2
Period [years]
Autocorrelation
1.0
0.8
0.6
0.4
0.2
0.0
0
5
10
15
20
25
30
lag [3 days]

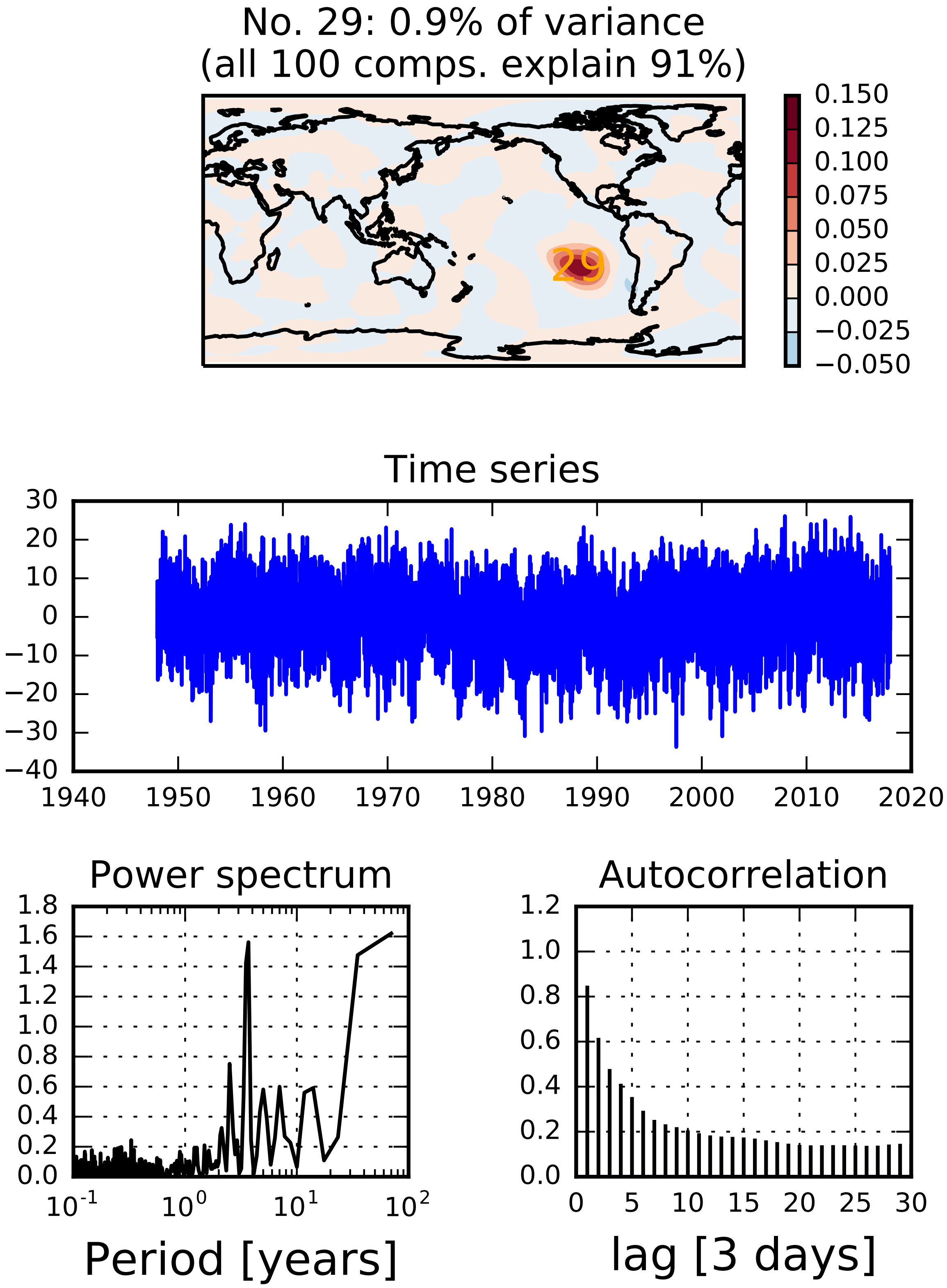
No. 29: 0.9% of variance
(all 100 comps. explain 91%)
29
0.150
0.125
0.100
0.075
0.050
0.025
0.000
−0.025
−0.050
Time series
30
20
10
0
−10
−20
−30
−40
1940
1950
1960
1970
1980
1990
2000
2010
2020
Power spectrum
1.8
1.6
1.4
1.2
1.0
0.8
0.6
0.4
0.2
0.0
10^{-1}
10^{0}
10^{1}
10^{2}
Period [years]
Autocorrelation
1.2
1.0
0.8
0.6
0.4
0.2
0.0
0
5
10
15
20
25
30
lag [3 days]

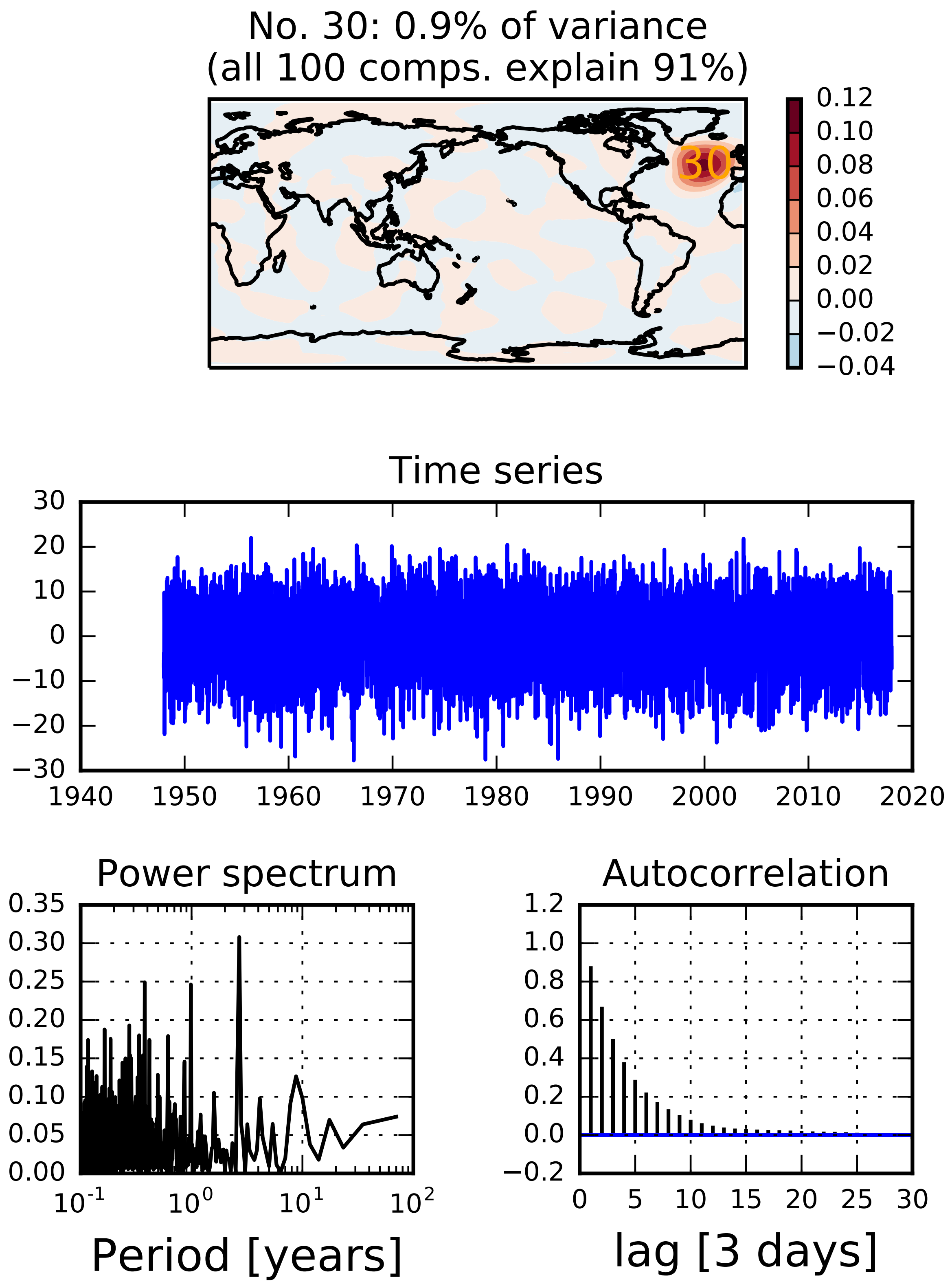
No. 30: 0.9% of variance
(all 100 comps. explain 91%)
30
0.12
0.10
0.08
0.06
0.04
0.02
0.00
−0.02
−0.04
Time series
30
20
10
0
−10
−20
−30
1940
1950
1960
1970
1980
1990
2000
2010
2020
Power spectrum
0.35
0.30
0.25
0.20
0.15
0.10
0.05
0.00
10^{-1}
10^{0}
10^{1}
10^{2}
Period [years]
Autocorrelation
1.2
1.0
0.8
0.6
0.4
0.2
0.0
−0.2
0
5
10
15
20
25
30
lag [3 days]

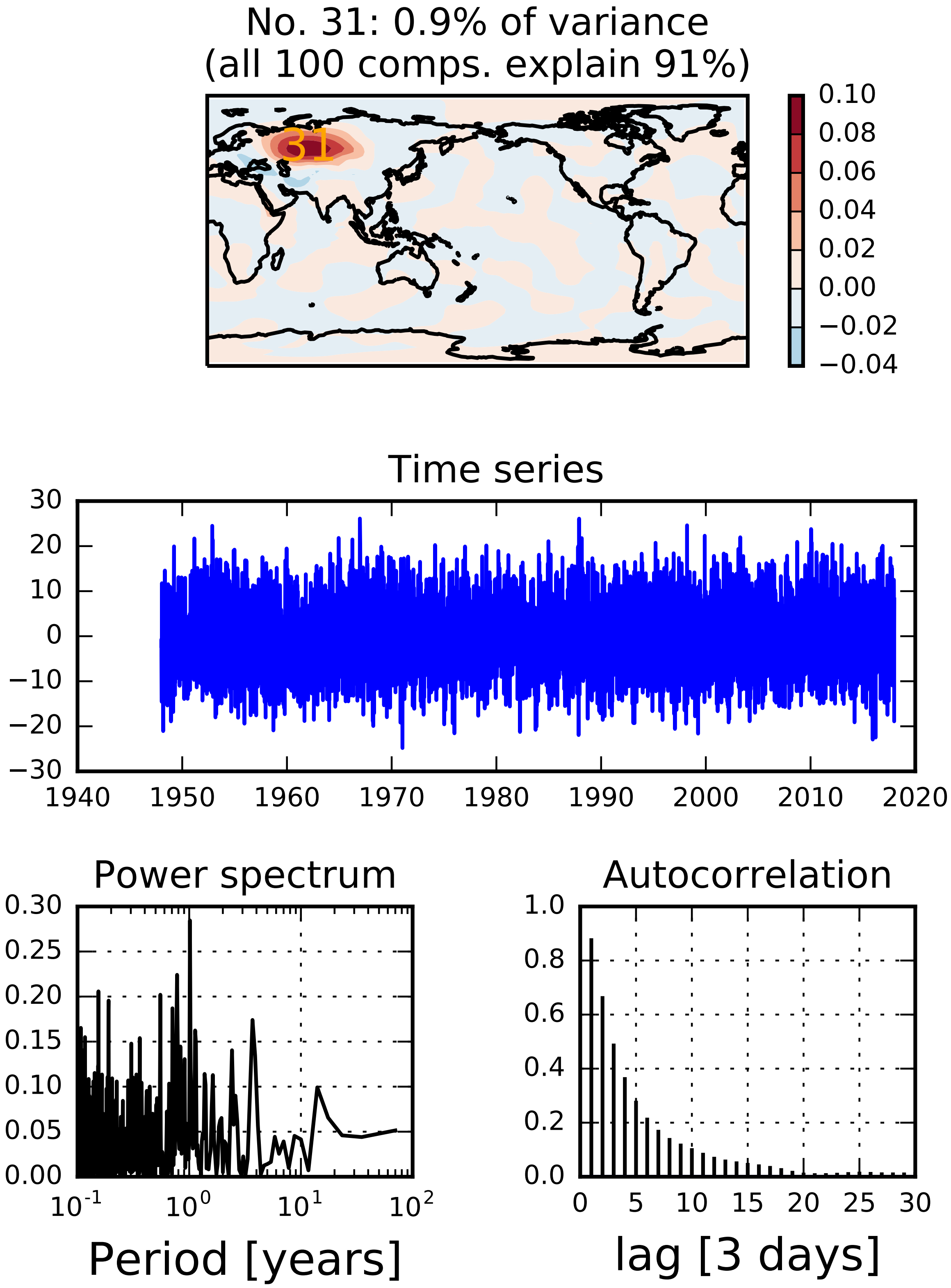
No. 31: 0.9% of variance
(all 100 comps. explain 91%)
31
0.10
0.08
0.06
0.04
0.02
0.00
−0.02
−0.04
Time series
30
20
10
0
−10
−20
−30
1940
1950
1960
1970
1980
1990
2000
2010
2020
Power spectrum
0.30
0.25
0.20
0.15
0.10
0.05
0.00
10^{-1}
10^{0}
10^{1}
10^{2}
Period [years]
Autocorrelation
1.0
0.8
0.6
0.4
0.2
0.0
0
5
10
15
20
25
30
lag [3 days]

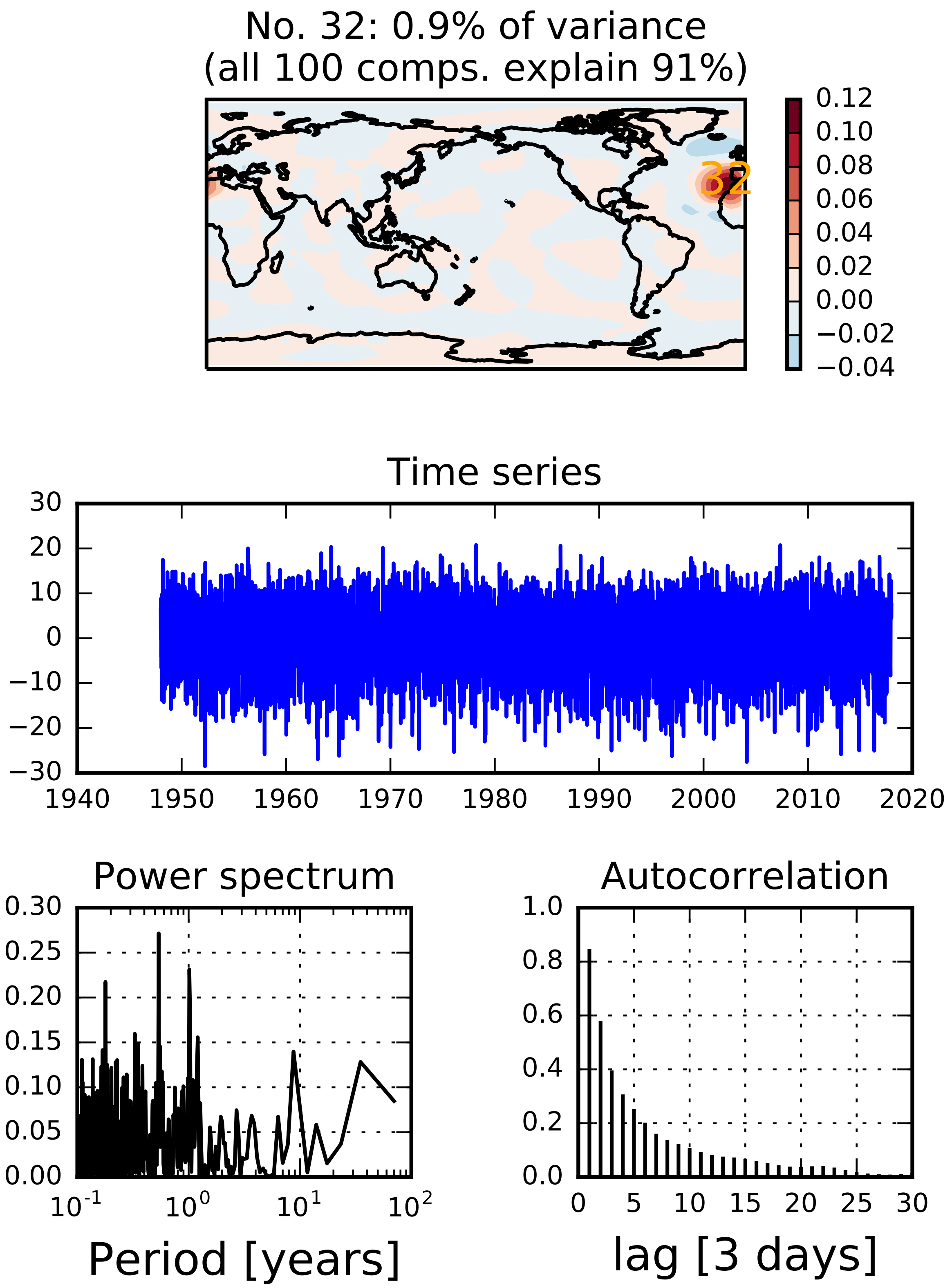
No. 32: 0.9% of variance
(all 100 comps. explain 91%)
32
0.12
0.10
0.08
0.06
0.04
0.02
0.00
−0.02
−0.04
Time series
30
20
10
0
−10
−20
−30
1940
1950
1960
1970
1980
1990
2000
2010
2020
Power spectrum
0.30
0.25
0.20
0.15
0.10
0.05
0.00
10^{-1}
10^{0}
10^{1}
10^{2}
Period [years]
Autocorrelation
1.0
0.8
0.6
0.4
0.2
0.0
0
5
10
15
20
25
30
lag [3 days]

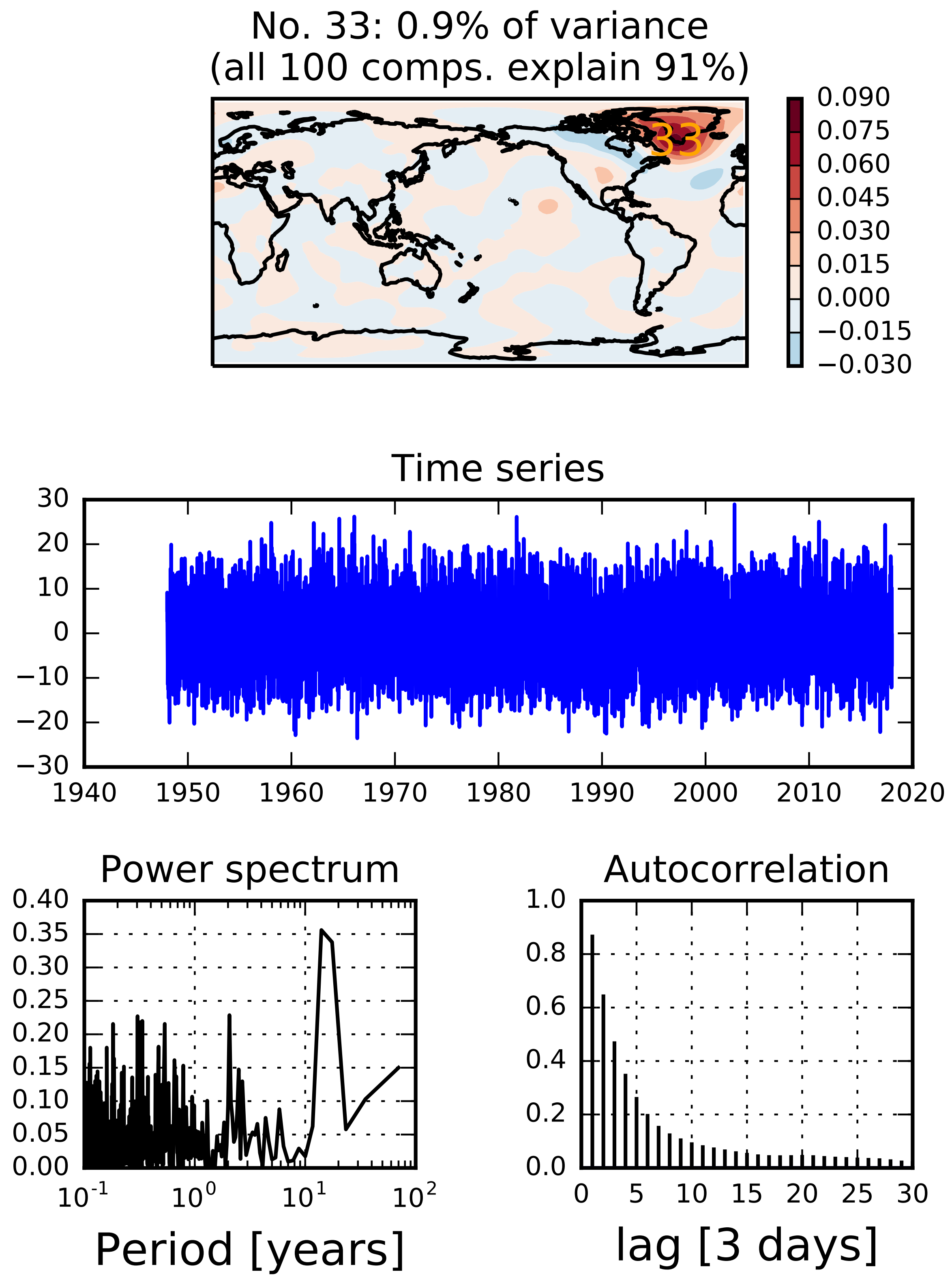
No. 33: 0.9% of variance
(all 100 comps. explain 91%)
33
0.090
0.075
0.060
0.045
0.030
0.015
0.000
−0.015
−0.030
Time series
30
20
10
0
−10
−20
−30
1940
1950
1960
1970
1980
1990
2000
2010
2020
Power spectrum
0.40
0.35
0.30
0.25
0.20
0.15
0.10
0.05
0.00
10^{-1}
10^{0}
10^{1}
10^{2}
Period [years]
Autocorrelation
1.0
0.8
0.6
0.4
0.2
0.0
0
5
10
15
20
25
30
lag [3 days]

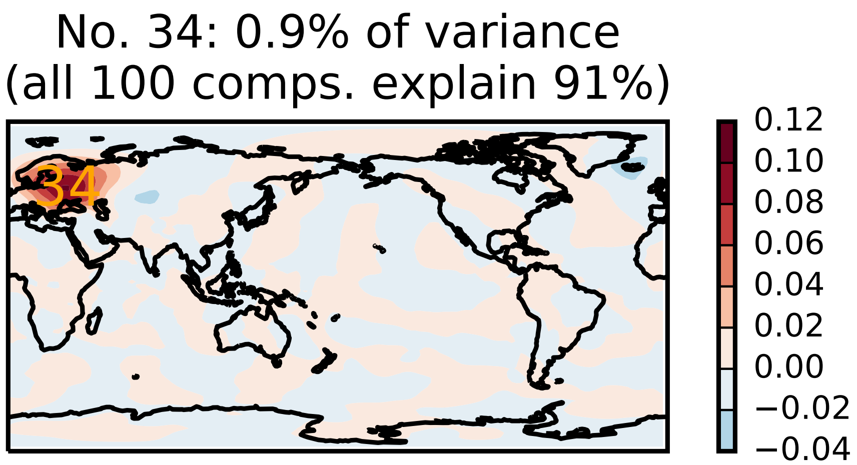
No. 34: 0.9% of variance
(all 100 comps. explain 91%)
34
0.12
0.10
0.08
0.06
0.04
0.02
0.00
−0.02
−0.04

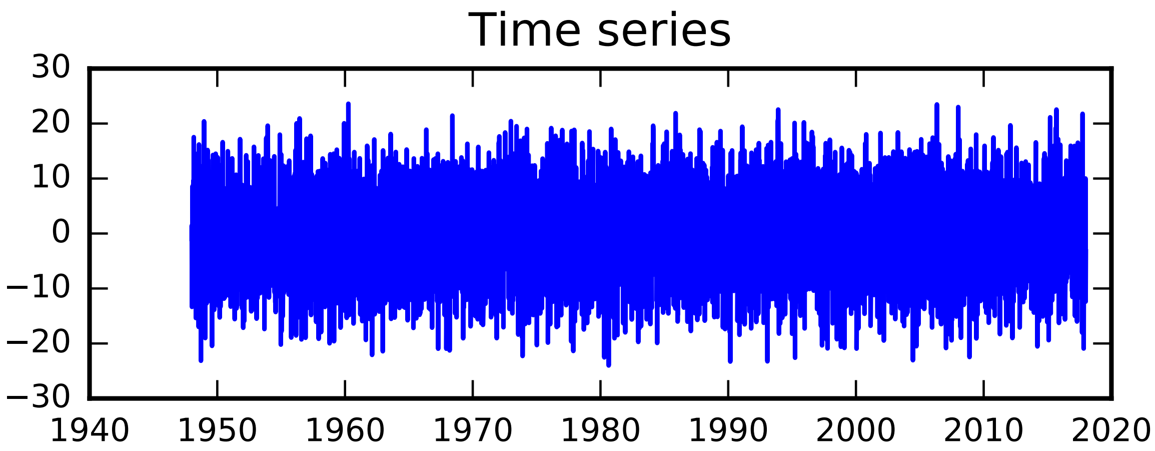
Time series
30
20
10
0
−10
−20
−30
1940
1950
1960
1970
1980
1990
2000
2010
2020

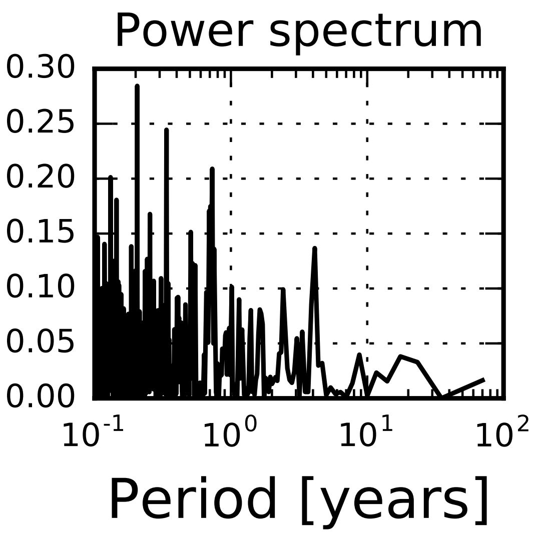
Power spectrum
0.30
0.25
0.20
0.15
0.10
0.05
0.00
10^{-1}
10^{0}
10^{1}
10^{2}
Period [years]

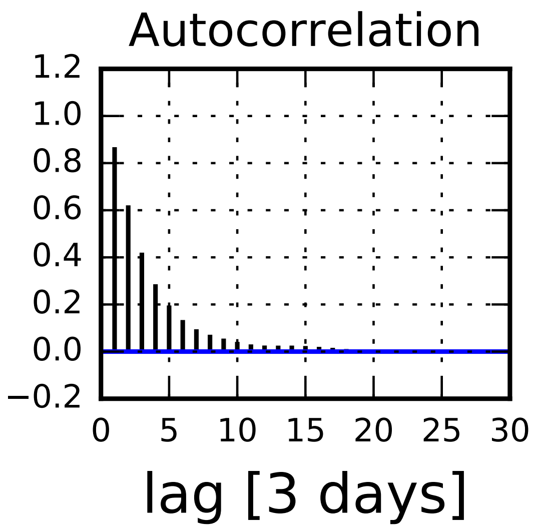
Autocorrelation
1.2
1.0
0.8
0.6
0.4
0.2
0.0
−0.2
0
5
10
15
20
25
30
lag [3 days]

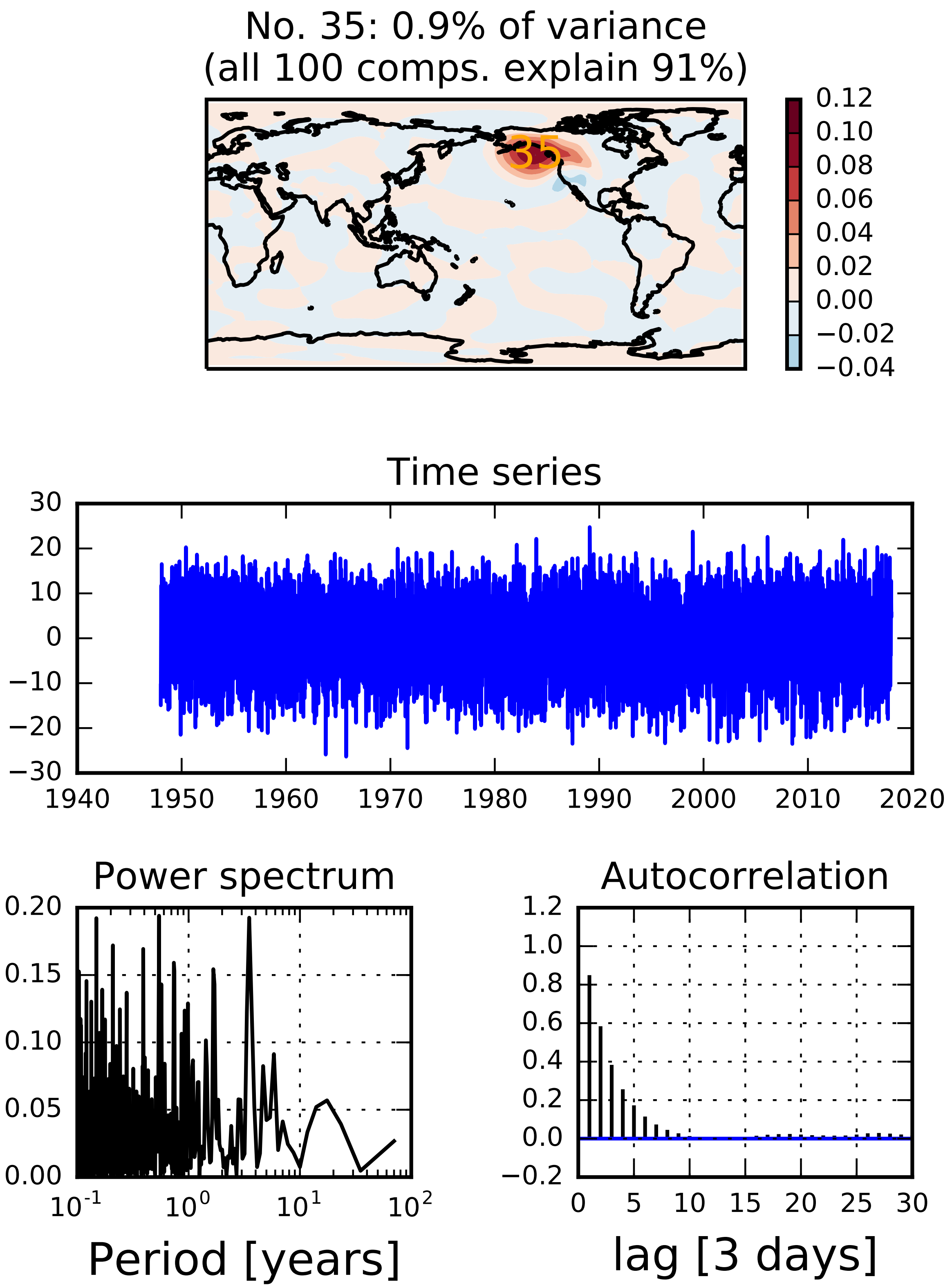
No. 35: 0.9% of variance
(all 100 comps. explain 91%)
35
0.12
0.10
0.08
0.06
0.04
0.02
0.00
−0.02
−0.04
Time series
30
20
10
0
−10
−20
−30
1940
1950
1960
1970
1980
1990
2000
2010
2020
Power spectrum
0.20
0.15
0.10
0.05
0.00
10^{-1}
10^{0}
10^{1}
10^{2}
Period [years]
Autocorrelation
1.2
1.0
0.8
0.6
0.4
0.2
0.0
−0.2
0
5
10
15
20
25
30
lag [3 days]

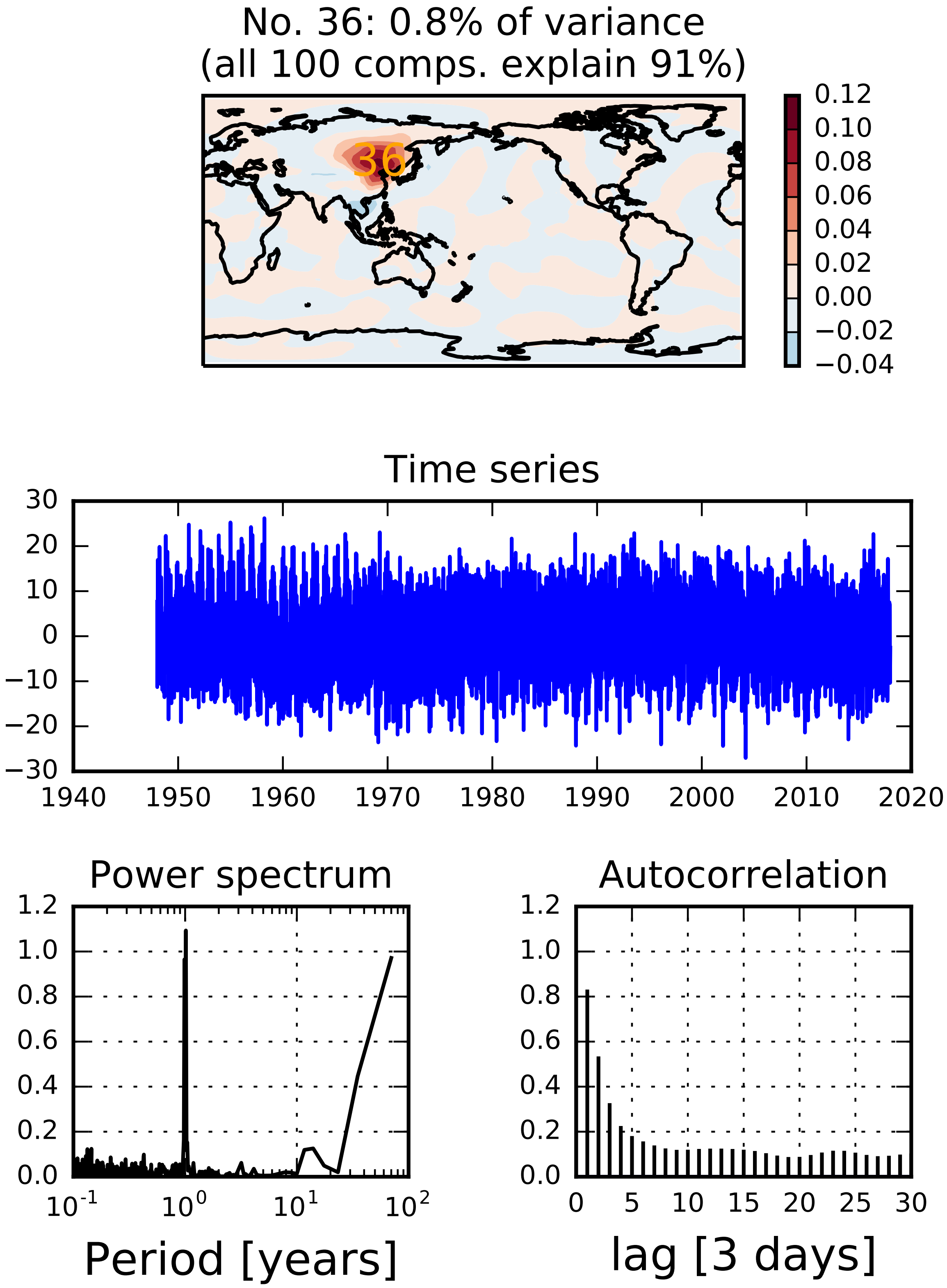
No. 36: 0.8% of variance
(all 100 comps. explain 91%)
36
0.12
0.10
0.08
0.06
0.04
0.02
0.00
−0.02
−0.04
Time series
30
20
10
0
−10
−20
−30
1940
1950
1960
1970
1980
1990
2000
2010
2020
Power spectrum
1.2
1.0
0.8
0.6
0.4
0.2
0.0
10^{-1}
10^{0}
10^{1}
10^{2}
Period [years]
Autocorrelation
1.2
1.0
0.8
0.6
0.4
0.2
0.0
0
5
10
15
20
25
30
lag [3 days]

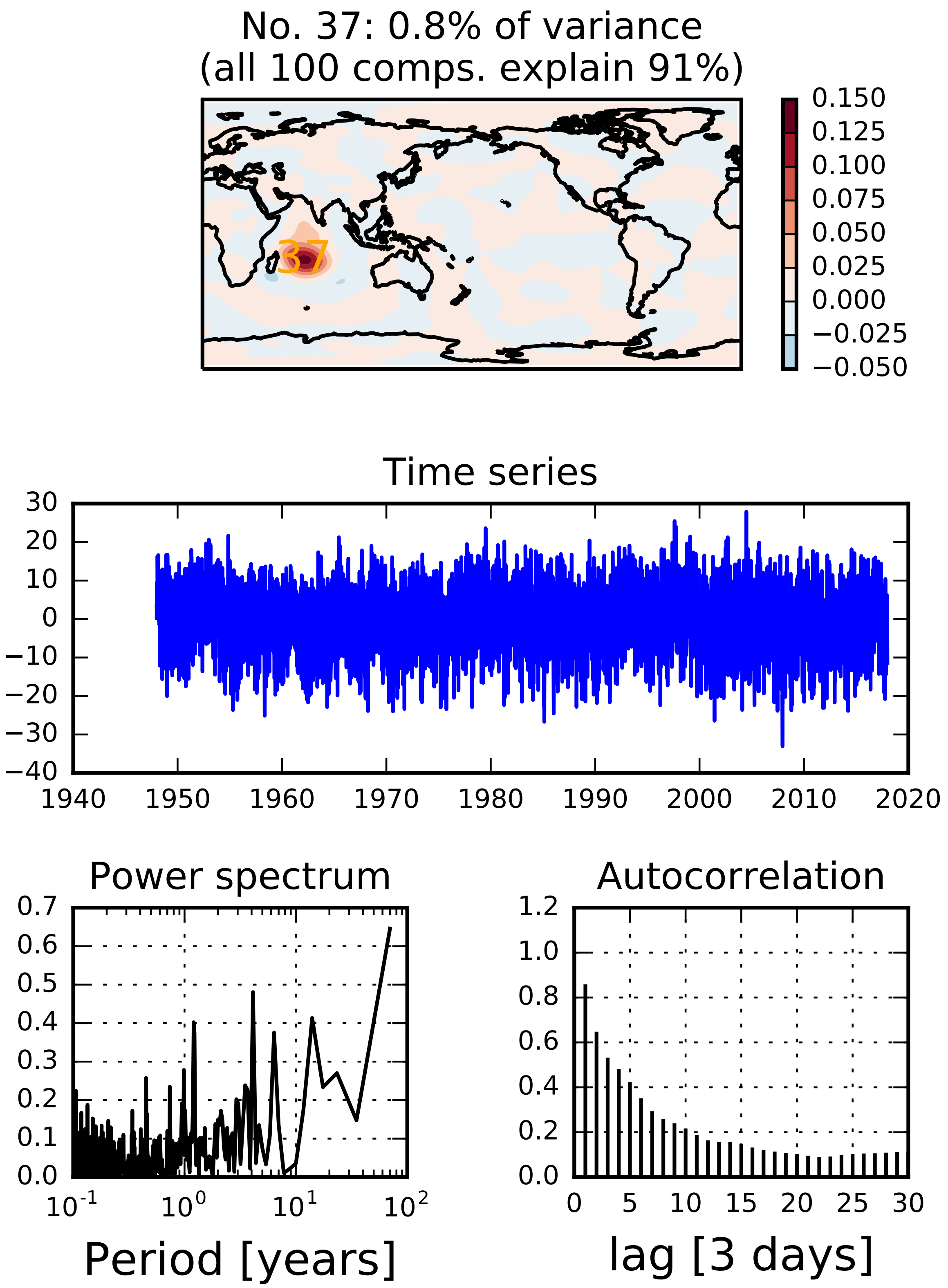
No. 37: 0.8% of variance
(all 100 comps. explain 91%)
37
0.150
0.125
0.100
0.075
0.050
0.025
0.000
−0.025
−0.050
Time series
30
20
10
0
−10
−20
−30
−40
1940
1950
1960
1970
1980
1990
2000
2010
2020
Power spectrum
0.7
0.6
0.5
0.4
0.3
0.2
0.1
0.0
10^{-1}
10^{0}
10^{1}
10^{2}
Period [years]
Autocorrelation
1.2
1.0
0.8
0.6
0.4
0.2
0.0
0
5
10
15
20
25
30
lag [3 days]

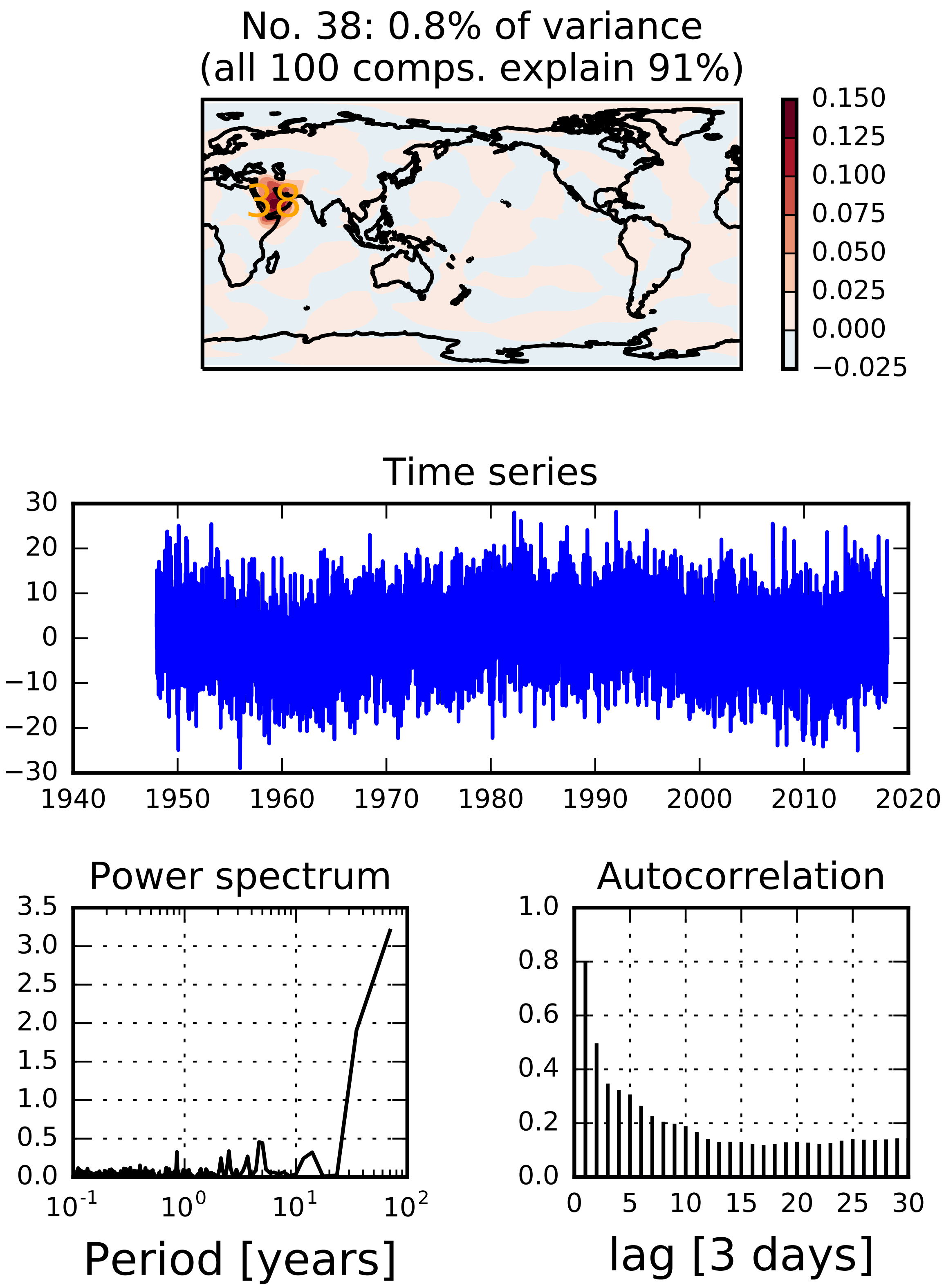
No. 38: 0.8% of variance
(all 100 comps. explain 91%)
38
0.150
0.125
0.100
0.075
0.050
0.025
0.000
−0.025
Time series
30
20
10
0
−10
−20
−30
1940
1950
1960
1970
1980
1990
2000
2010
2020
Power spectrum
3.5
3.0
2.5
2.0
1.5
1.0
0.5
0.0
10^{-1}
10^{0}
10^{1}
10^{2}
Period [years]
Autocorrelation
1.0
0.8
0.6
0.4
0.2
0.0
0
5
10
15
20
25
30
lag [3 days]

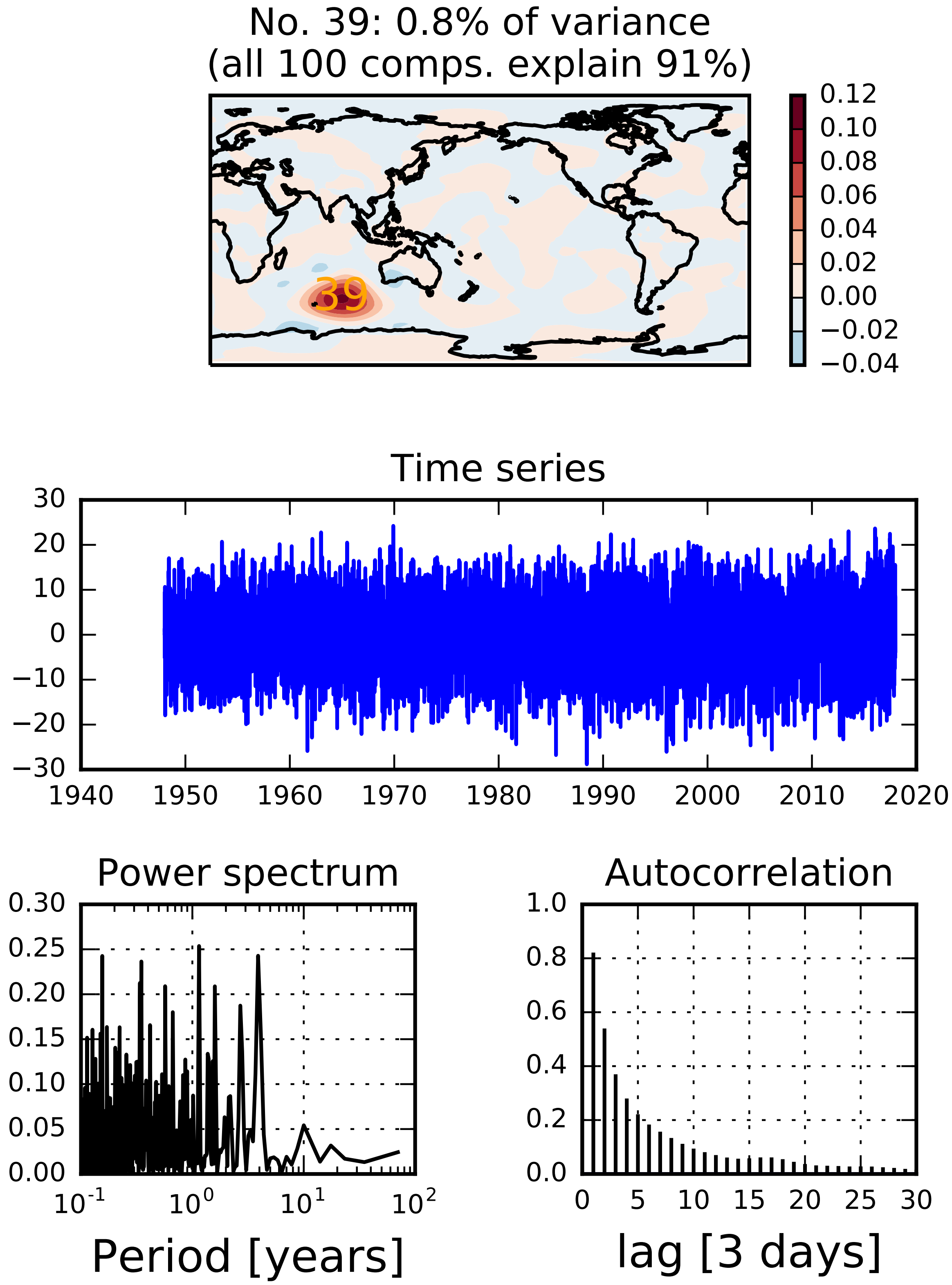
No. 39: 0.8% of variance
(all 100 comps. explain 91%)
39
0.12
0.10
0.08
0.06
0.04
0.02
0.00
−0.02
−0.04
Time series
30
20
10
0
−10
−20
−30
1940
1950
1960
1970
1980
1990
2000
2010
2020
Power spectrum
0.30
0.25
0.20
0.15
0.10
0.05
0.00
10^{-1}
10^{0}
10^{1}
10^{2}
Period [years]
Autocorrelation
1.0
0.8
0.6
0.4
0.2
0.0
0
5
10
15
20
25
30
lag [3 days]

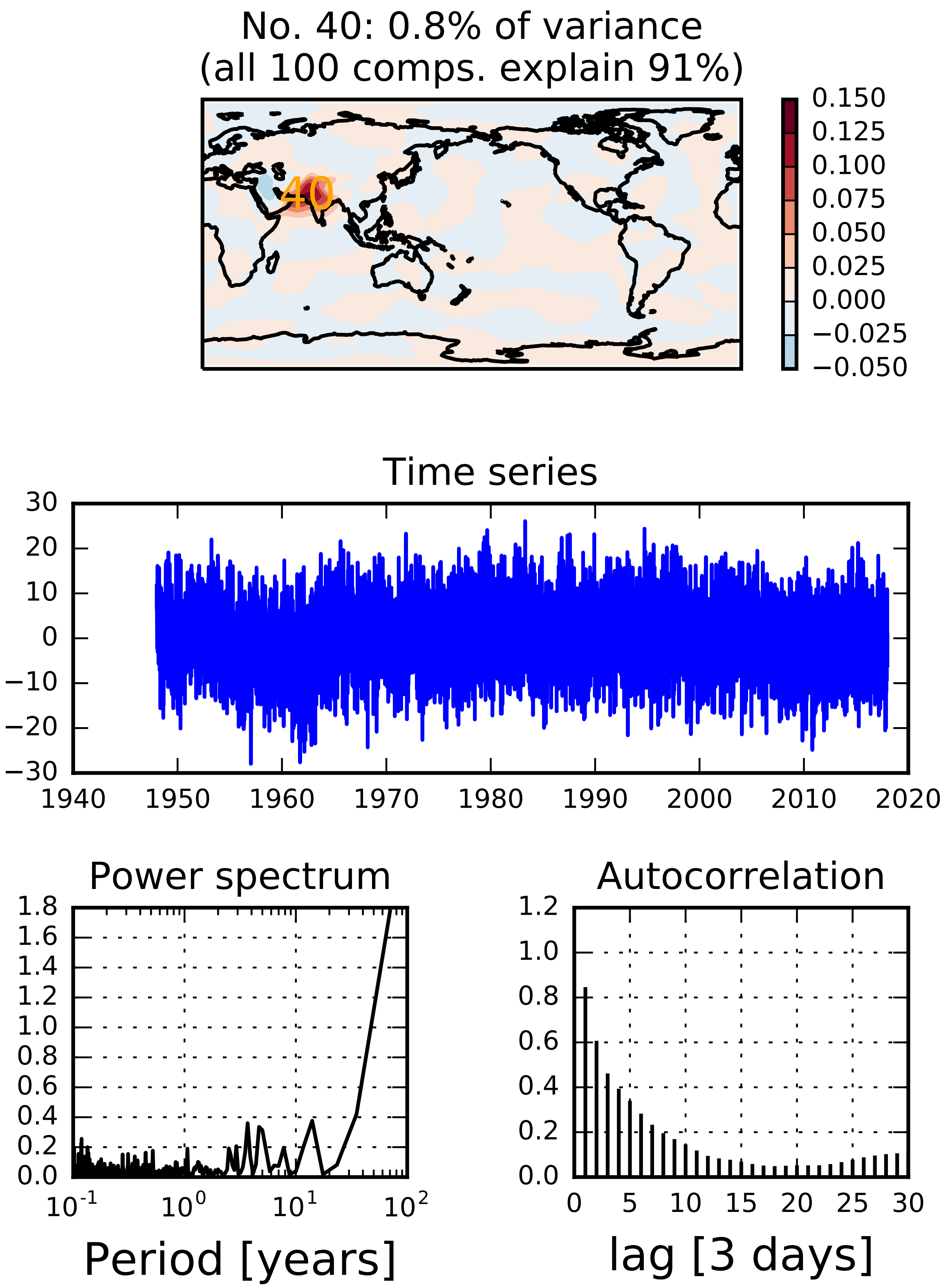
No. 40: 0.8% of variance
(all 100 comps. explain 91%)
40
0.150
0.125
0.100
0.075
0.050
0.025
0.000
−0.025
−0.050
Time series
30
20
10
0
−10
−20
−30
1940
1950
1960
1970
1980
1990
2000
2010
2020
Power spectrum
1.8
1.6
1.4
1.2
1.0
0.8
0.6
0.4
0.2
0.0
10^{-1}
10^{0}
10^{1}
10^{2}
Period [years]
Autocorrelation
1.2
1.0
0.8
0.6
0.4
0.2
0.0
0
5
10
15
20
25
30
lag [3 days]

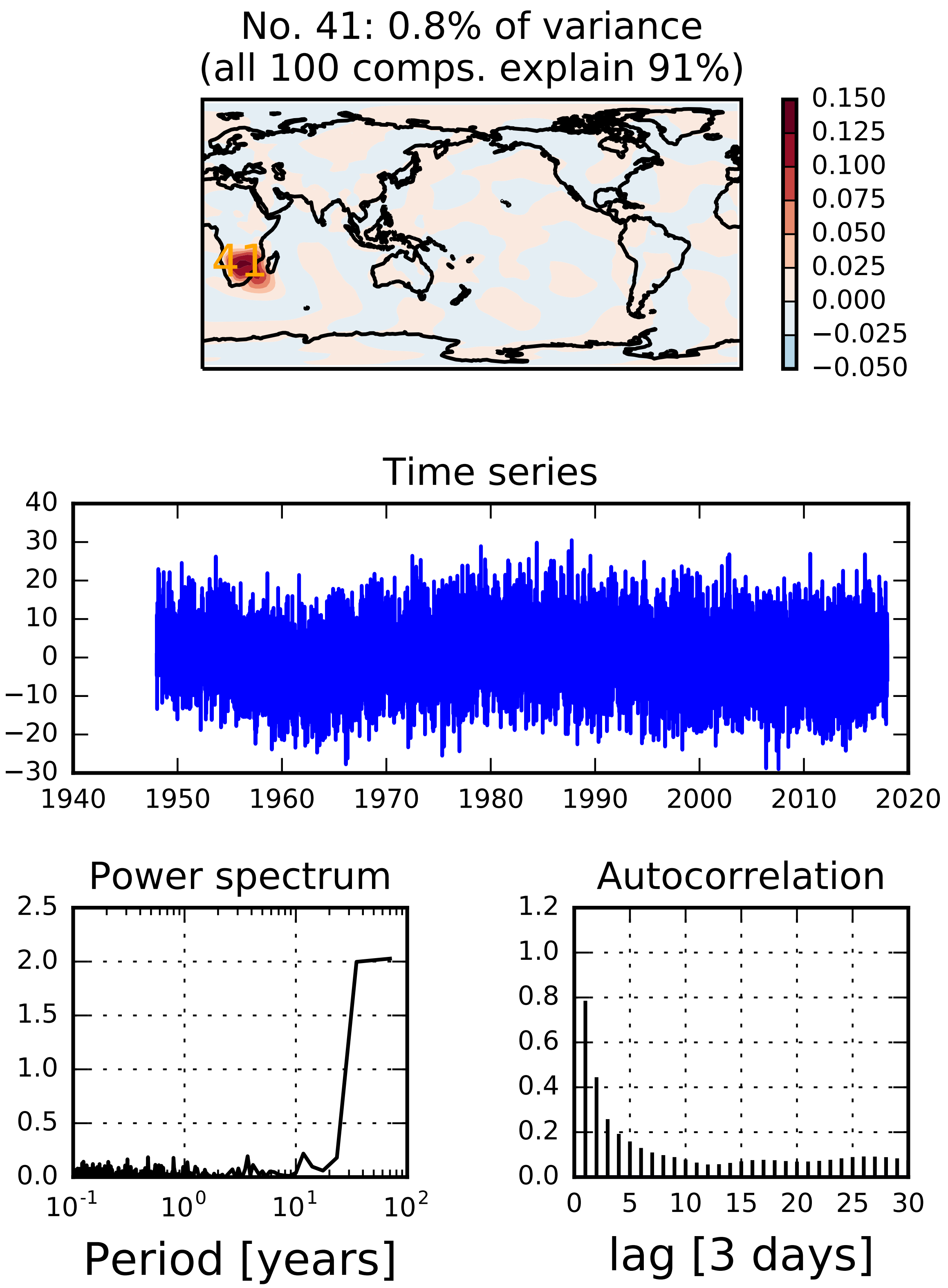
No. 41: 0.8% of variance
(all 100 comps. explain 91%)
41
0.150
0.125
0.100
0.075
0.050
0.025
0.000
−0.025
−0.050
Time series
40
30
20
10
0
−10
−20
−30
1940
1950
1960
1970
1980
1990
2000
2010
2020
Power spectrum
2.5
2.0
1.5
1.0
0.5
0.0
10^{-1}
10^{0}
10^{1}
10^{2}
Period [years]
Autocorrelation
1.2
1.0
0.8
0.6
0.4
0.2
0.0
0
5
10
15
20
25
30
lag [3 days]

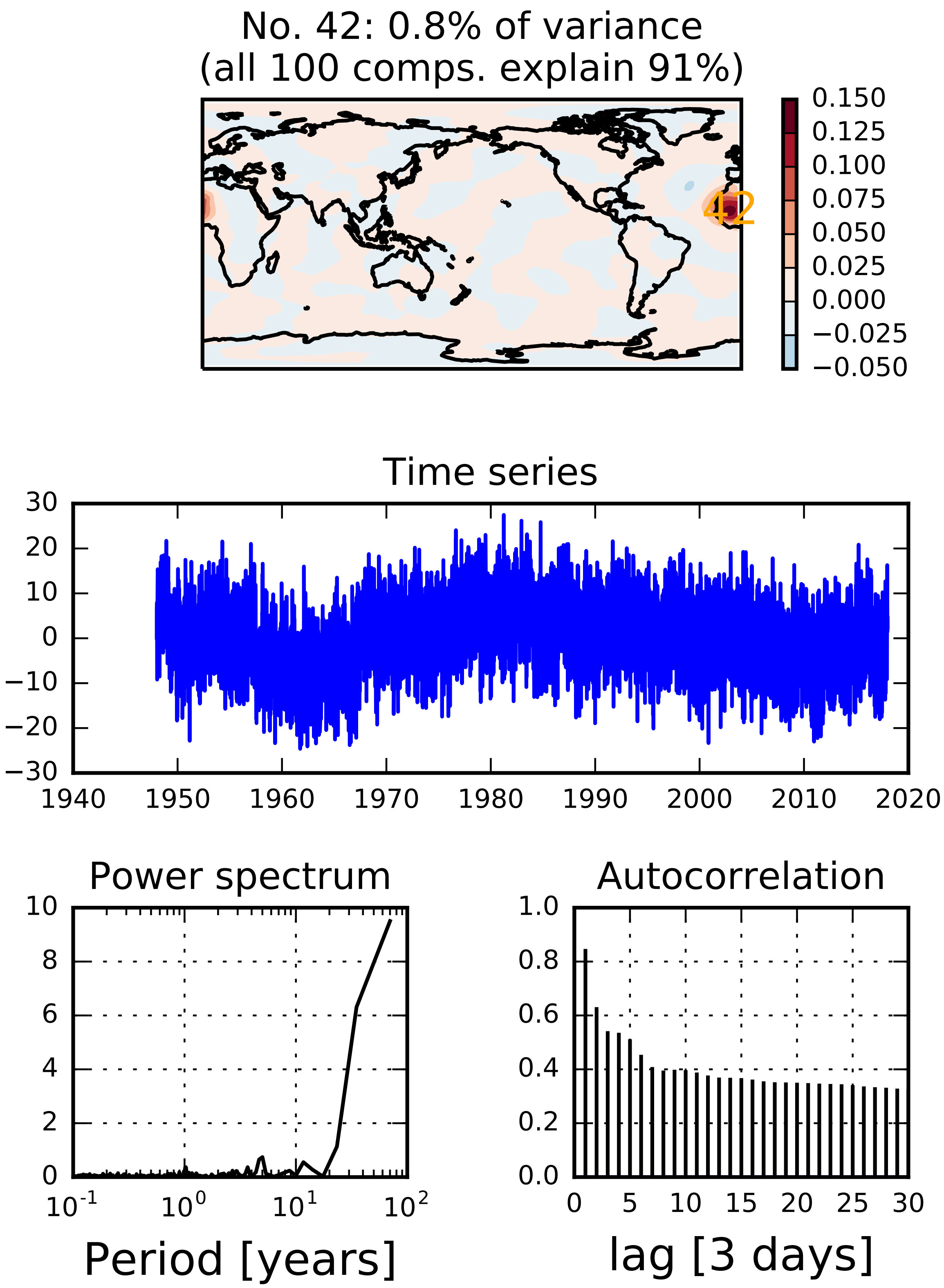
No. 42: 0.8% of variance
(all 100 comps. explain 91%)
42
0.150
0.125
0.100
0.075
0.050
0.025
0.000
−0.025
−0.050
Time series
30
20
10
0
−10
−20
−30
1940
1950
1960
1970
1980
1990
2000
2010
2020
Power spectrum
10
8
6
4
2
0
10^{-1}
10^{0}
10^{1}
10^{2}
Period [years]
Autocorrelation
1.0
0.8
0.6
0.4
0.2
0.0
0
5
10
15
20
25
30
lag [3 days]

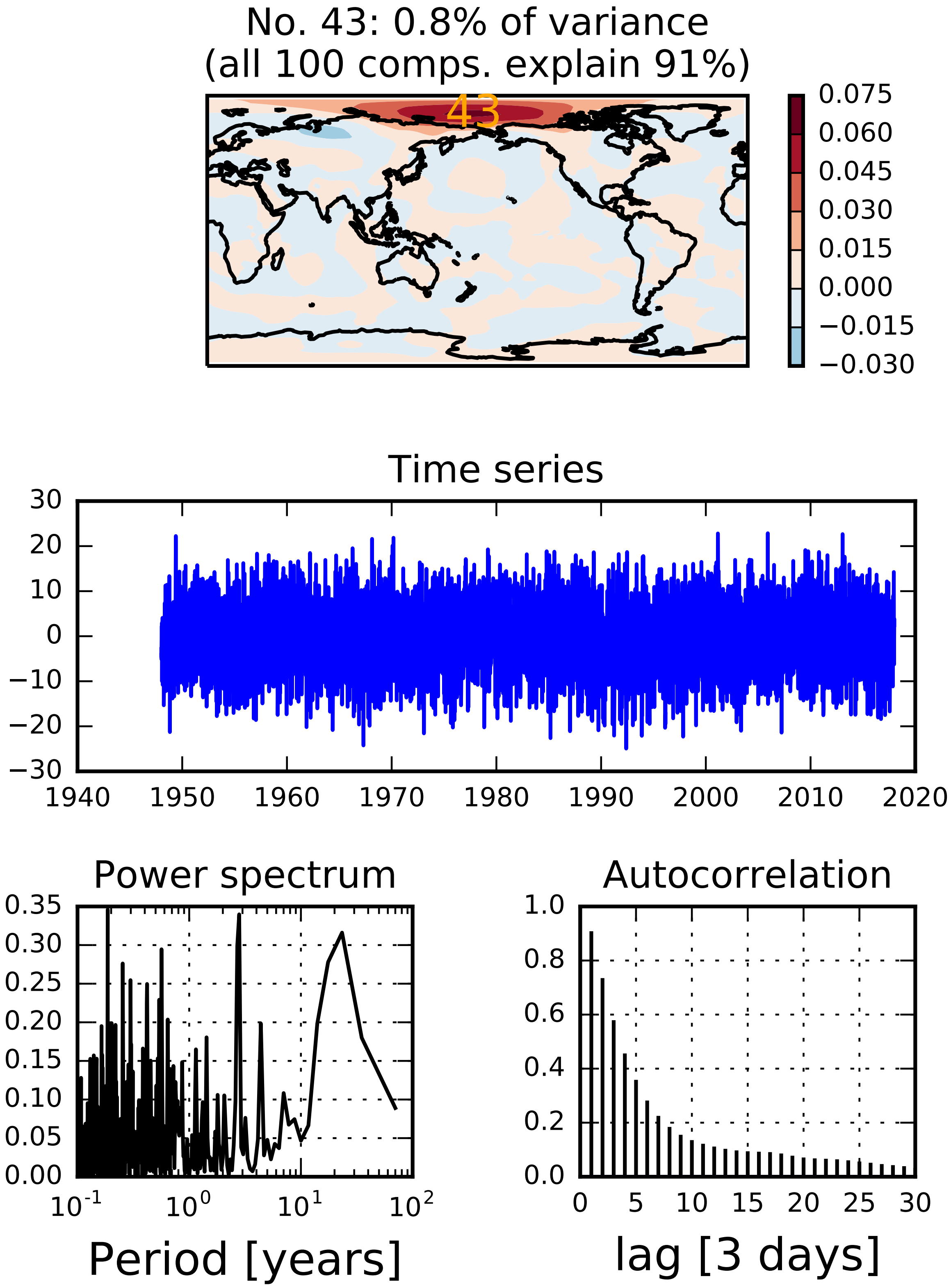
No. 43: 0.8% of variance
(all 100 comps. explain 91%)
43
0.075
0.060
0.045
0.030
0.015
0.000
−0.015
−0.030
Time series
30
20
10
0
−10
−20
−30
1940
1950
1960
1970
1980
1990
2000
2010
2020
Power spectrum
0.35
0.30
0.25
0.20
0.15
0.10
0.05
0.00
10^{-1}
10^{0}
10^{1}
10^{2}
Period [years]
Autocorrelation
1.0
0.8
0.6
0.4
0.2
0.0
0
5
10
15
20
25
30
lag [3 days]

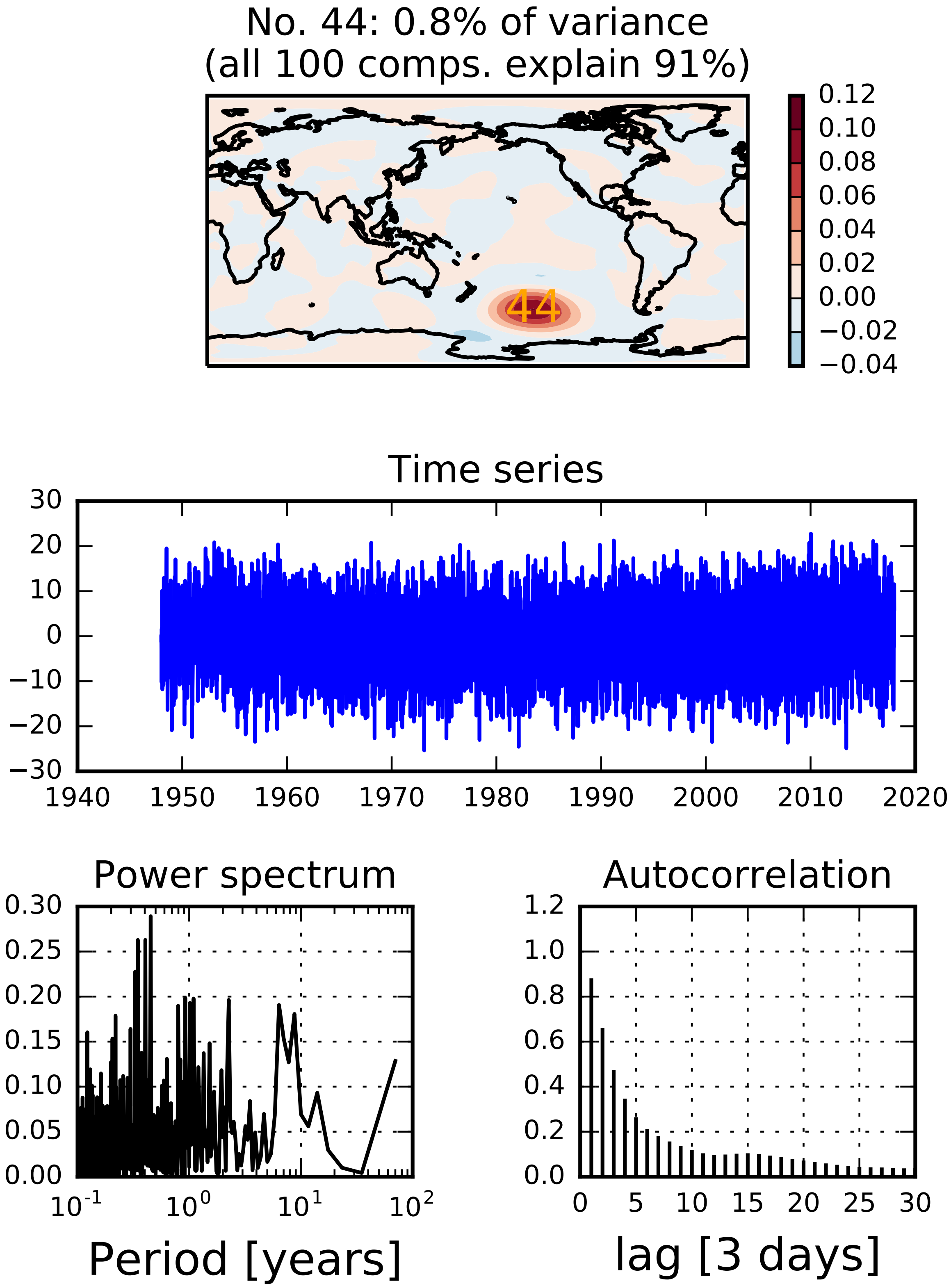
No. 44: 0.8% of variance
(all 100 comps. explain 91%)
44
0.12
0.10
0.08
0.06
0.04
0.02
0.00
−0.02
−0.04
Time series
30
20
10
0
−10
−20
−30
1940
1950
1960
1970
1980
1990
2000
2010
2020
Power spectrum
0.30
0.25
0.20
0.15
0.10
0.05
0.00
10^-1
10^0
10^1
10^2
Period [years]
Autocorrelation
1.2
1.0
0.8
0.6
0.4
0.2
0.0
0
5
10
15
20
25
30
lag [3 days]

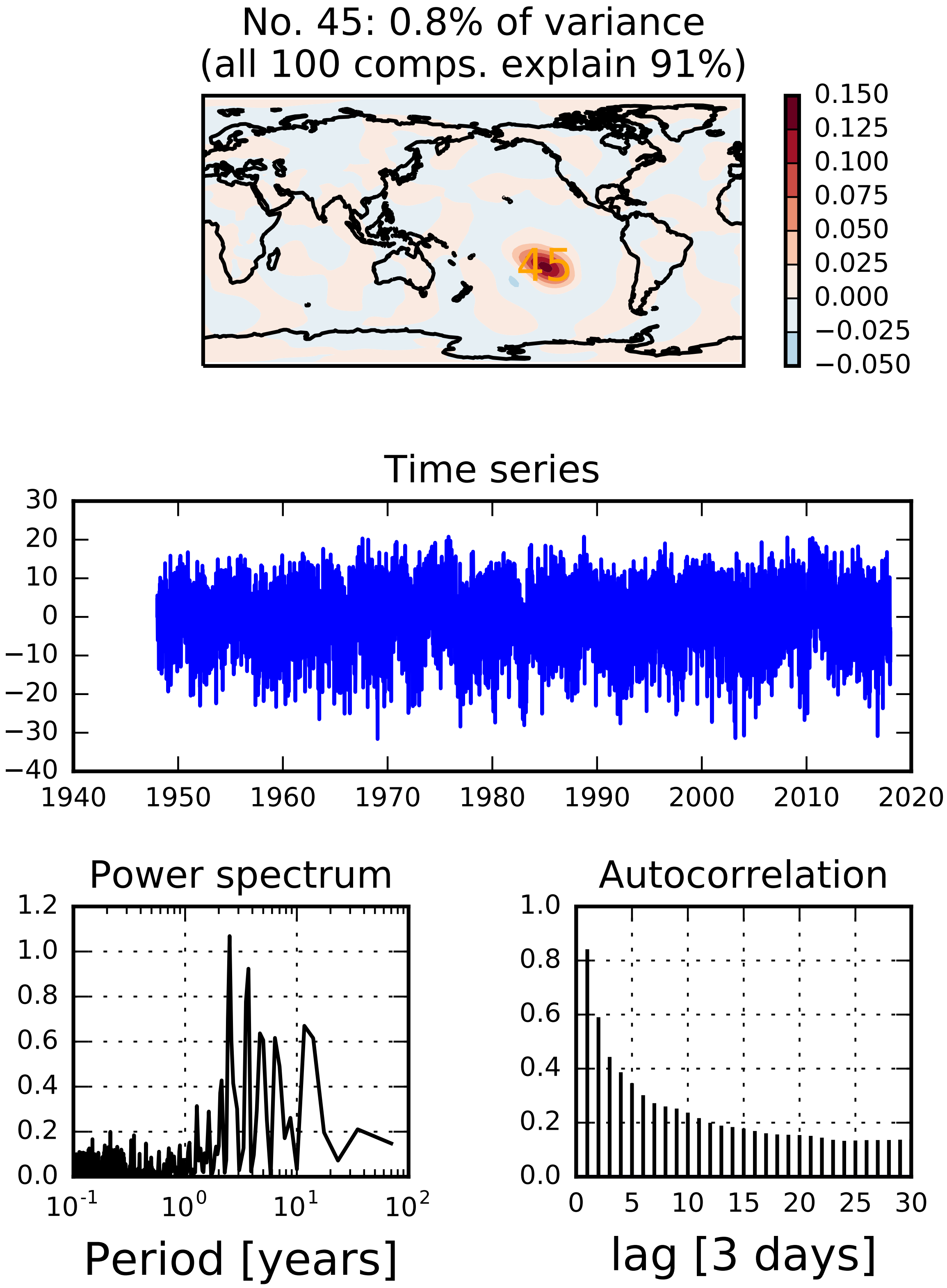
No. 45: 0.8% of variance
(all 100 comps. explain 91%)
45
0.150
0.125
0.100
0.075
0.050
0.025
0.000
−0.025
−0.050
Time series
30
20
10
0
−10
−20
−30
−40
1940
1950
1960
1970
1980
1990
2000
2010
2020
Power spectrum
1.2
1.0
0.8
0.6
0.4
0.2
0.0
10^{-1}
10^{0}
10^{1}
10^{2}
Period [years]
Autocorrelation
1.0
0.8
0.6
0.4
0.2
0.0
0
5
10
15
20
25
30
lag [3 days]

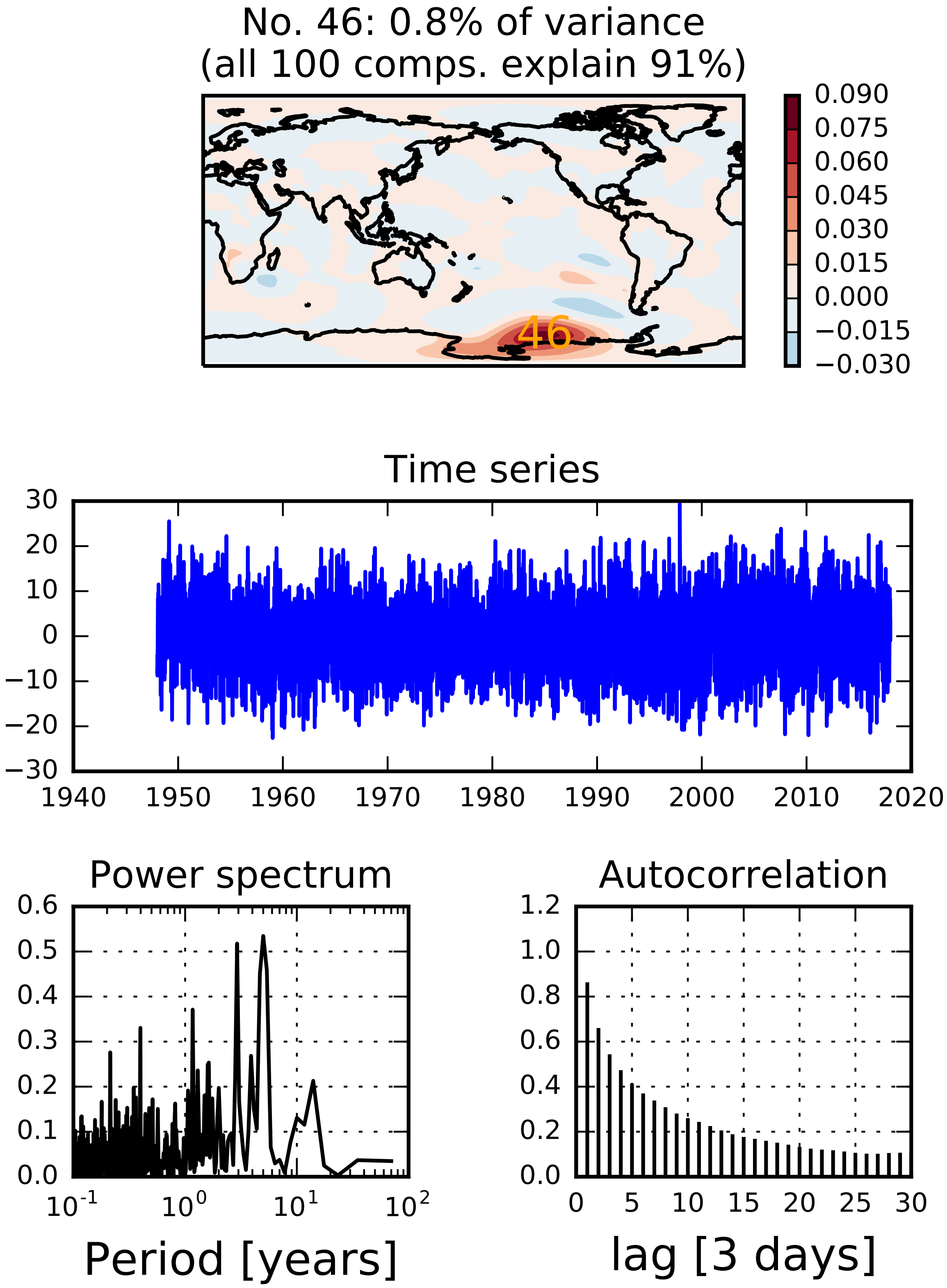
No. 46: 0.8% of variance
(all 100 comps. explain 91%)
46
0.090
0.075
0.060
0.045
0.030
0.015
0.000
−0.015
−0.030
Time series
30
20
10
0
−10
−20
−30
1940
1950
1960
1970
1980
1990
2000
2010
2020
Power spectrum
0.6
0.5
0.4
0.3
0.2
0.1
0.0
10^-1
10^0
10^1
10^2
Period [years]
Autocorrelation
1.2
1.0
0.8
0.6
0.4
0.2
0.0
0
5
10
15
20
25
30
lag [3 days]

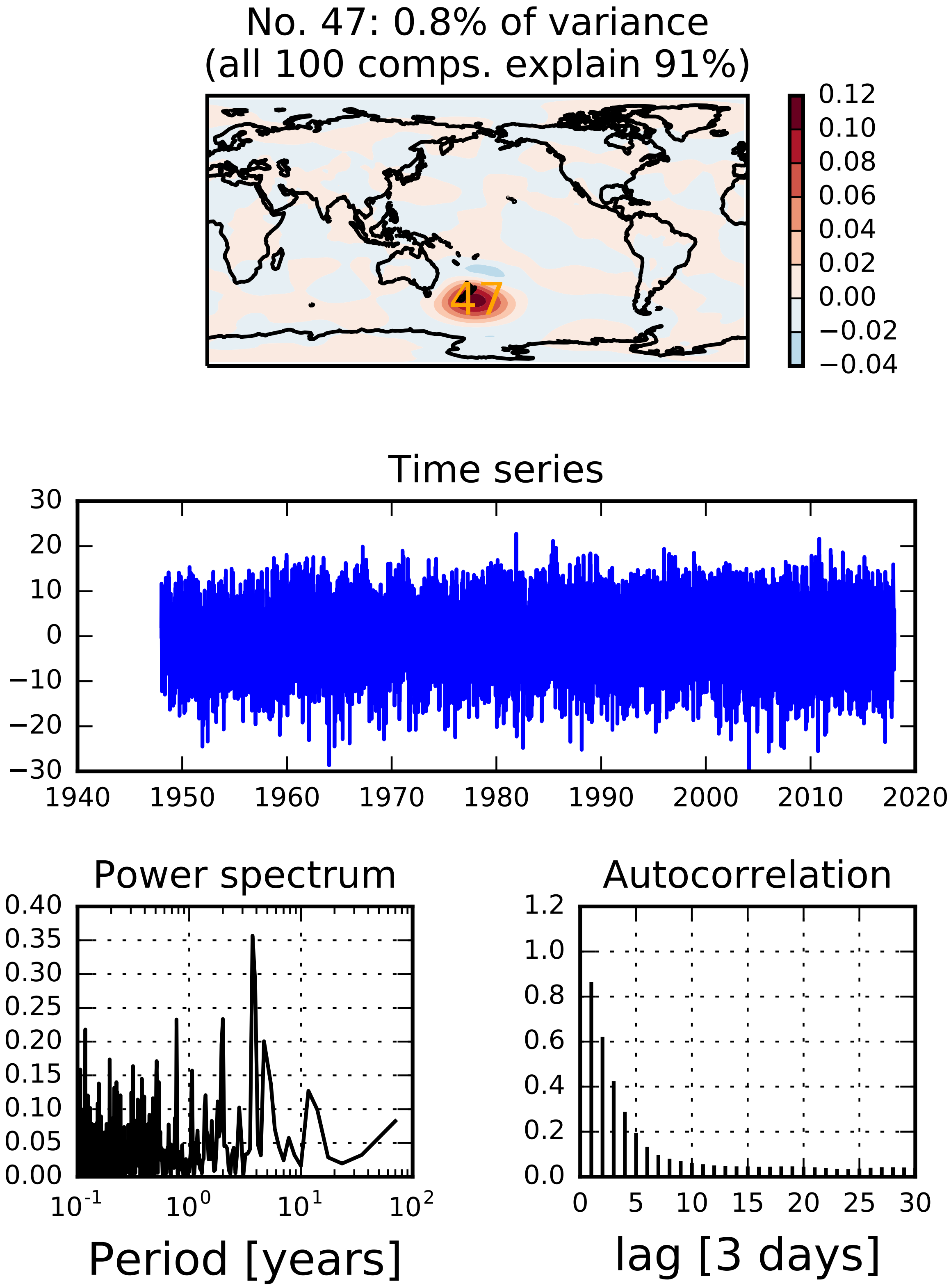
No. 47: 0.8% of variance
(all 100 comps. explain 91%)
47
0.12
0.10
0.08
0.06
0.04
0.02
0.00
−0.02
−0.04
Time series
30
20
10
0
−10
−20
−30
1940
1950
1960
1970
1980
1990
2000
2010
2020
Power spectrum
0.40
0.35
0.30
0.25
0.20
0.15
0.10
0.05
0.00
10^{-1}
10^{0}
10^{1}
10^{2}
Period [years]
Autocorrelation
1.2
1.0
0.8
0.6
0.4
0.2
0.0
0
5
10
15
20
25
30
lag [3 days]

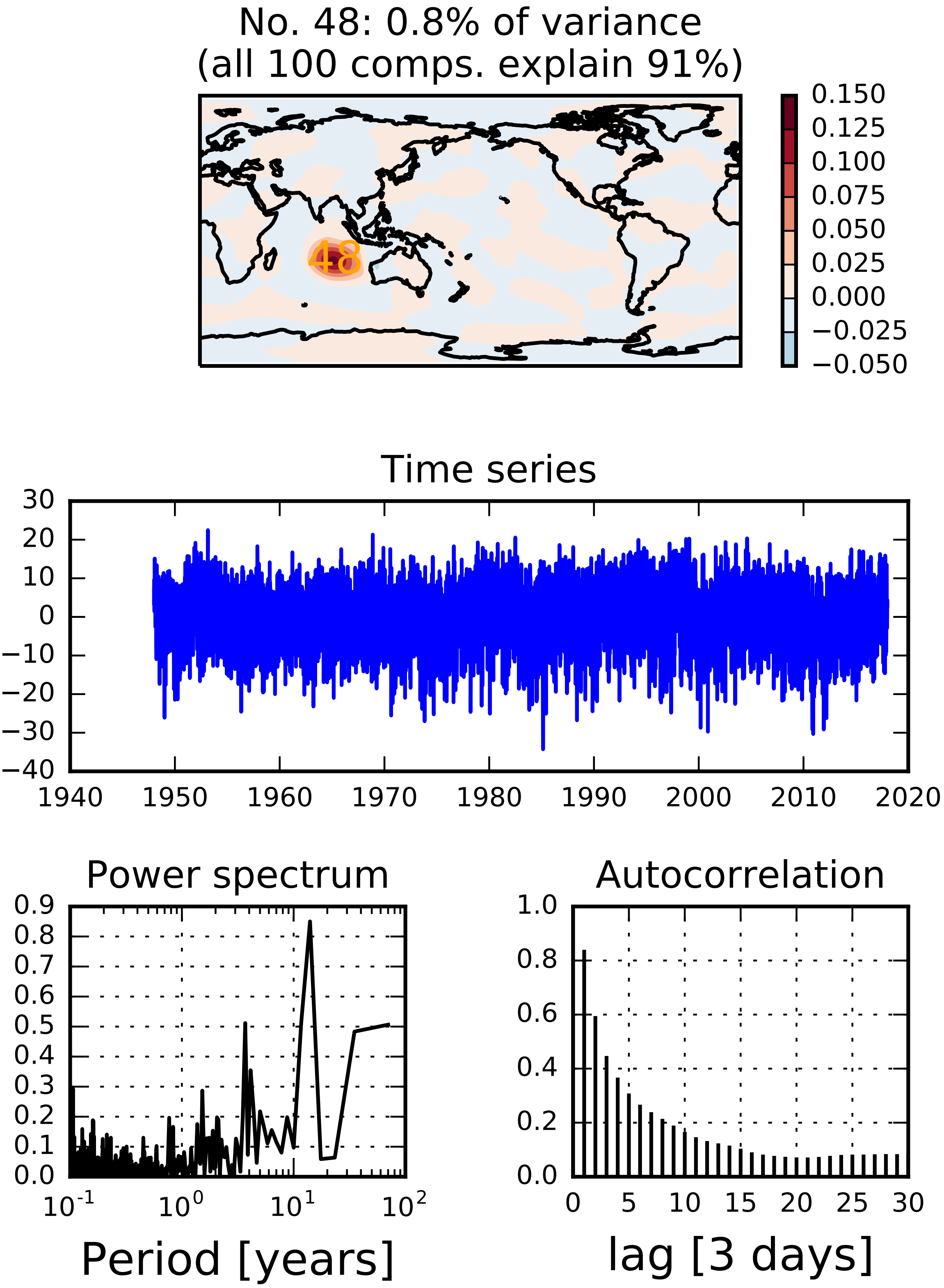
No. 48: 0.8% of variance
(all 100 comps. explain 91%)
48
0.150
0.125
0.100
0.075
0.050
0.025
0.000
−0.025
−0.050
Time series
30
20
10
0
−10
−20
−30
−40
1940
1950
1960
1970
1980
1990
2000
2010
2020
Power spectrum
0.9
0.8
0.7
0.6
0.5
0.4
0.3
0.2
0.1
0.0
10^{-1}
10^{0}
10^{1}
10^{2}
Period [years]
Autocorrelation
1.0
0.8
0.6
0.4
0.2
0.0
0
5
10
15
20
25
30
lag [3 days]

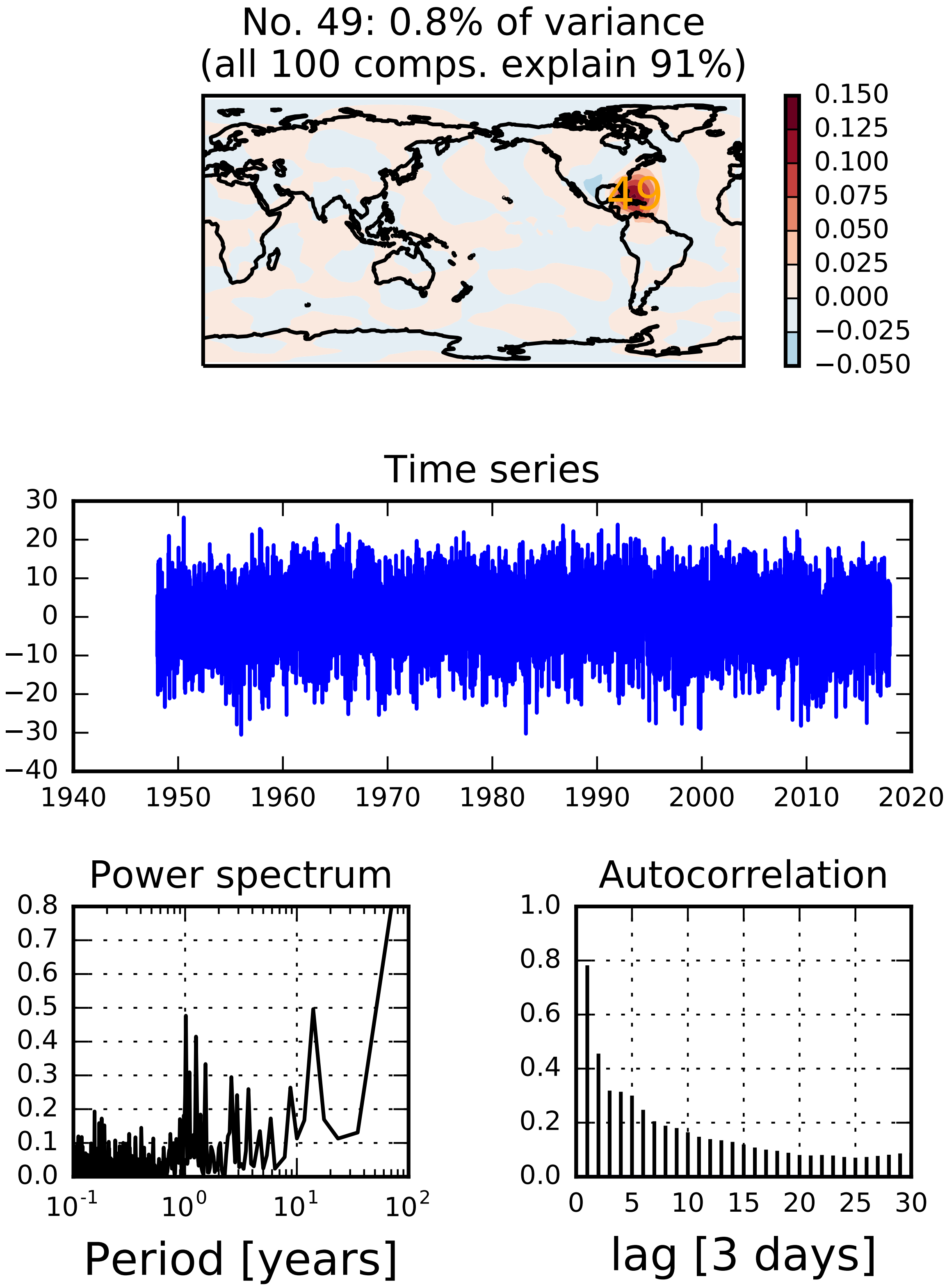
No. 49: 0.8% of variance
(all 100 comps. explain 91%)
49
0.150
0.125
0.100
0.075
0.050
0.025
0.000
−0.025
−0.050
Time series
30
20
10
0
−10
−20
−30
−40
1940
1950
1960
1970
1980
1990
2000
2010
2020
Power spectrum
0.8
0.7
0.6
0.5
0.4
0.3
0.2
0.1
0.0
10^{-1}
10^{0}
10^{1}
10^{2}
Period [years]
Autocorrelation
1.0
0.8
0.6
0.4
0.2
0.0
0
5
10
15
20
25
30
lag [3 days]

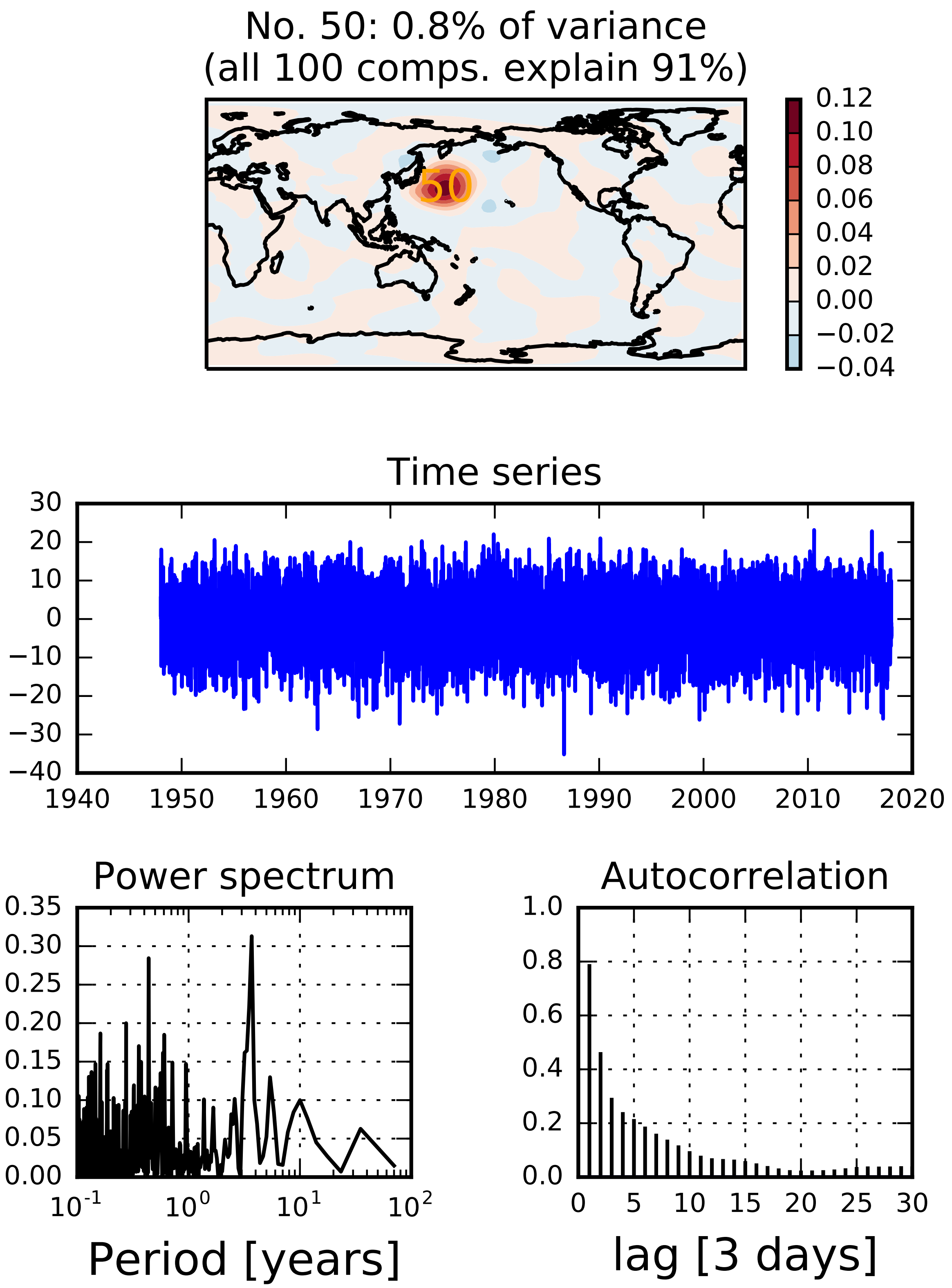
No. 50: 0.8% of variance
(all 100 comps. explain 91%)
50
0.12
0.10
0.08
0.06
0.04
0.02
0.00
−0.02
−0.04
Time series
30
20
10
0
−10
−20
−30
−40
1940
1950
1960
1970
1980
1990
2000
2010
2020
Power spectrum
0.35
0.30
0.25
0.20
0.15
0.10
0.05
0.00
10^{-1}
10^{0}
10^{1}
10^{2}
Period [years]
Autocorrelation
1.0
0.8
0.6
0.4
0.2
0.0
0
5
10
15
20
25
30
lag [3 days]

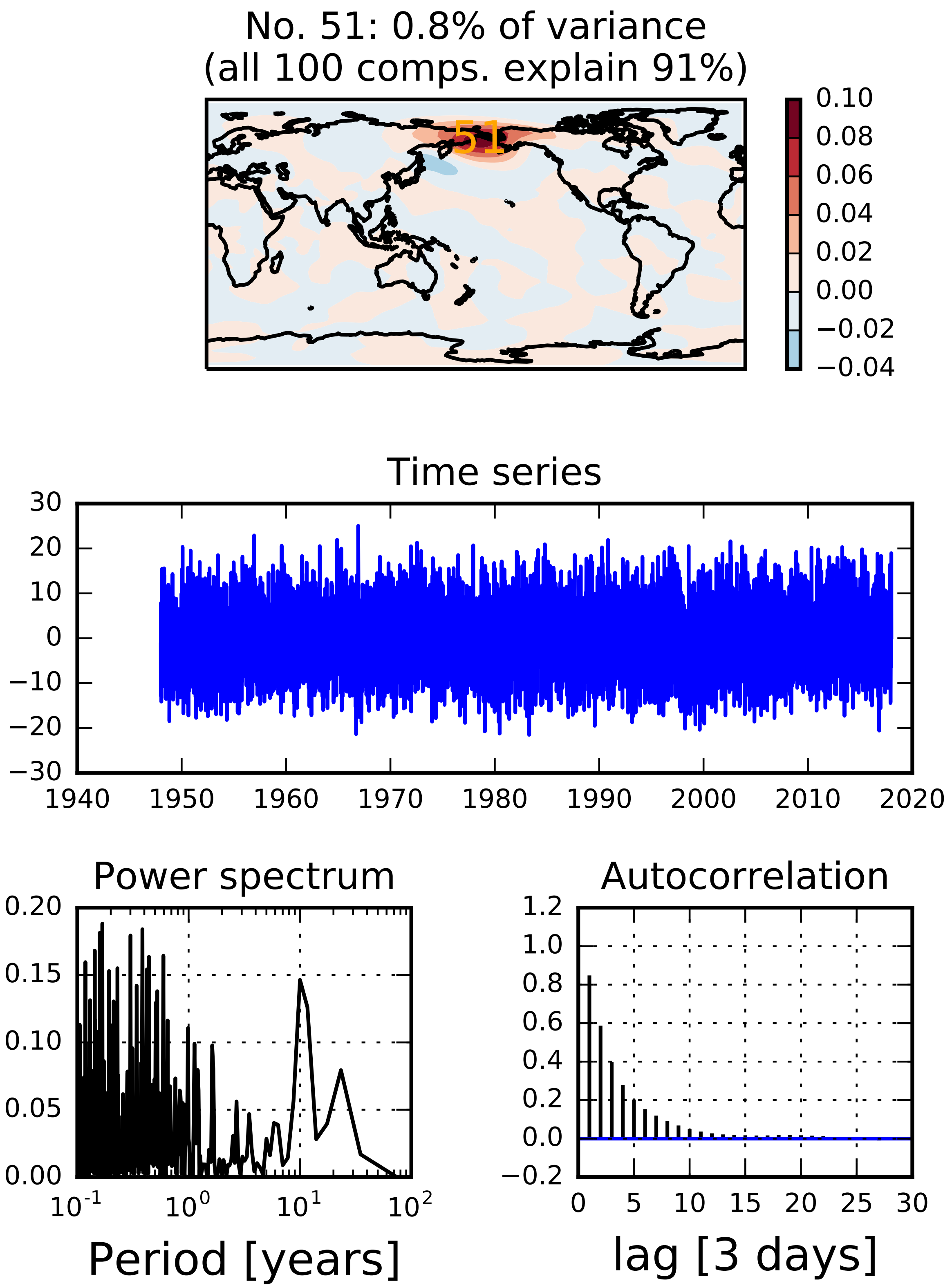
No. 51: 0.8% of variance
(all 100 comps. explain 91%)
51
0.10
0.08
0.06
0.04
0.02
0.00
−0.02
−0.04
Time series
30
20
10
0
−10
−20
−30
1940
1950
1960
1970
1980
1990
2000
2010
2020
Power spectrum
0.20
0.15
0.10
0.05
0.00
10^{-1}
10^{0}
10^{1}
10^{2}
Period [years]
Autocorrelation
1.2
1.0
0.8
0.6
0.4
0.2
0.0
−0.2
0
5
10
15
20
25
30
lag [3 days]

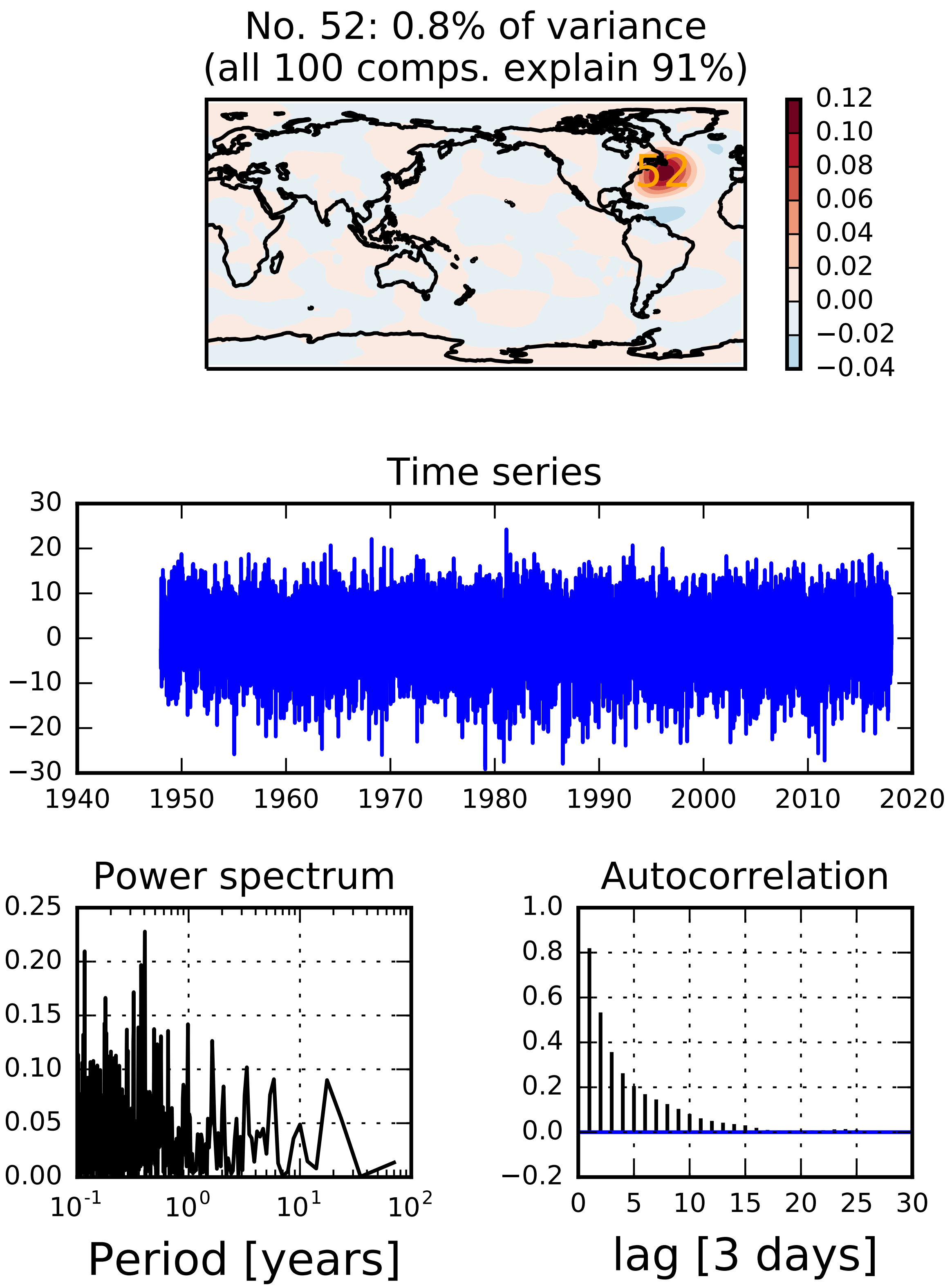
No. 52: 0.8% of variance
(all 100 comps. explain 91%)
52
0.12
0.10
0.08
0.06
0.04
0.02
0.00
−0.02
−0.04
Time series
30
20
10
0
−10
−20
−30
1940
1950
1960
1970
1980
1990
2000
2010
2020
Power spectrum
0.25
0.20
0.15
0.10
0.05
0.00
10^{-1}
10^{0}
10^{1}
10^{2}
Period [years]
Autocorrelation
1.0
0.8
0.6
0.4
0.2
0.0
−0.2
0
5
10
15
20
25
30
lag [3 days]

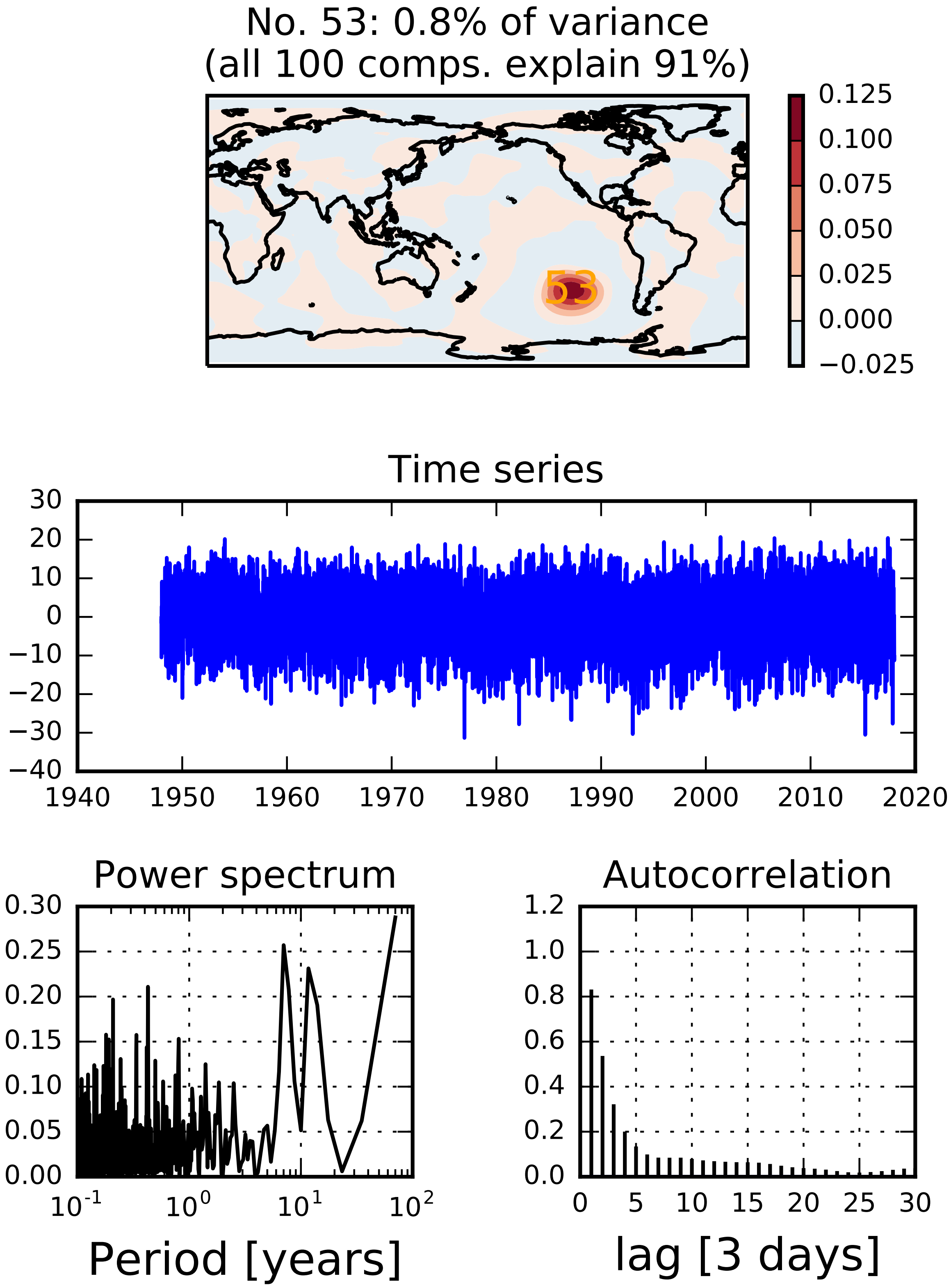
No. 53: 0.8% of variance
(all 100 comps. explain 91%)
53
0.125
0.100
0.075
0.050
0.025
0.000
−0.025
Time series
30
20
10
0
−10
−20
−30
−40
1940
1950
1960
1970
1980
1990
2000
2010
2020
Power spectrum
0.30
0.25
0.20
0.15
0.10
0.05
0.00
10^-1
10^0
10^1
10^2
Period [years]
Autocorrelation
1.2
1.0
0.8
0.6
0.4
0.2
0.0
0
5
10
15
20
25
30
lag [3 days]

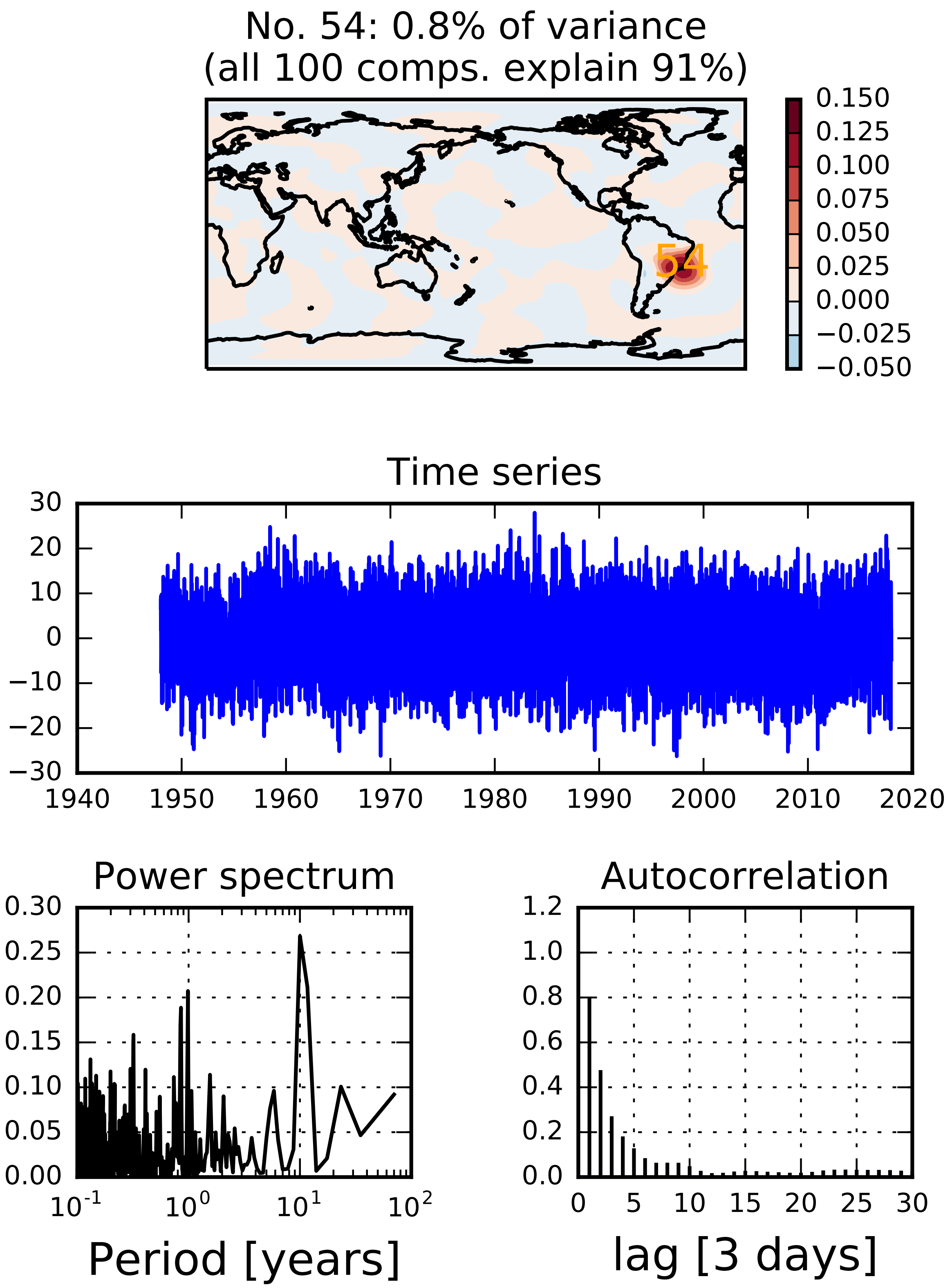
No. 54: 0.8% of variance
(all 100 comps. explain 91%)
54
0.150
0.125
0.100
0.075
0.050
0.025
0.000
−0.025
−0.050
Time series
30
20
10
0
−10
−20
−30
1940
1950
1960
1970
1980
1990
2000
2010
2020
Power spectrum
0.30
0.25
0.20
0.15
0.10
0.05
0.00
10^{-1}
10^{0}
10^{1}
10^{2}
Period [years]
Autocorrelation
1.2
1.0
0.8
0.6
0.4
0.2
0.0
0
5
10
15
20
25
30
lag [3 days]

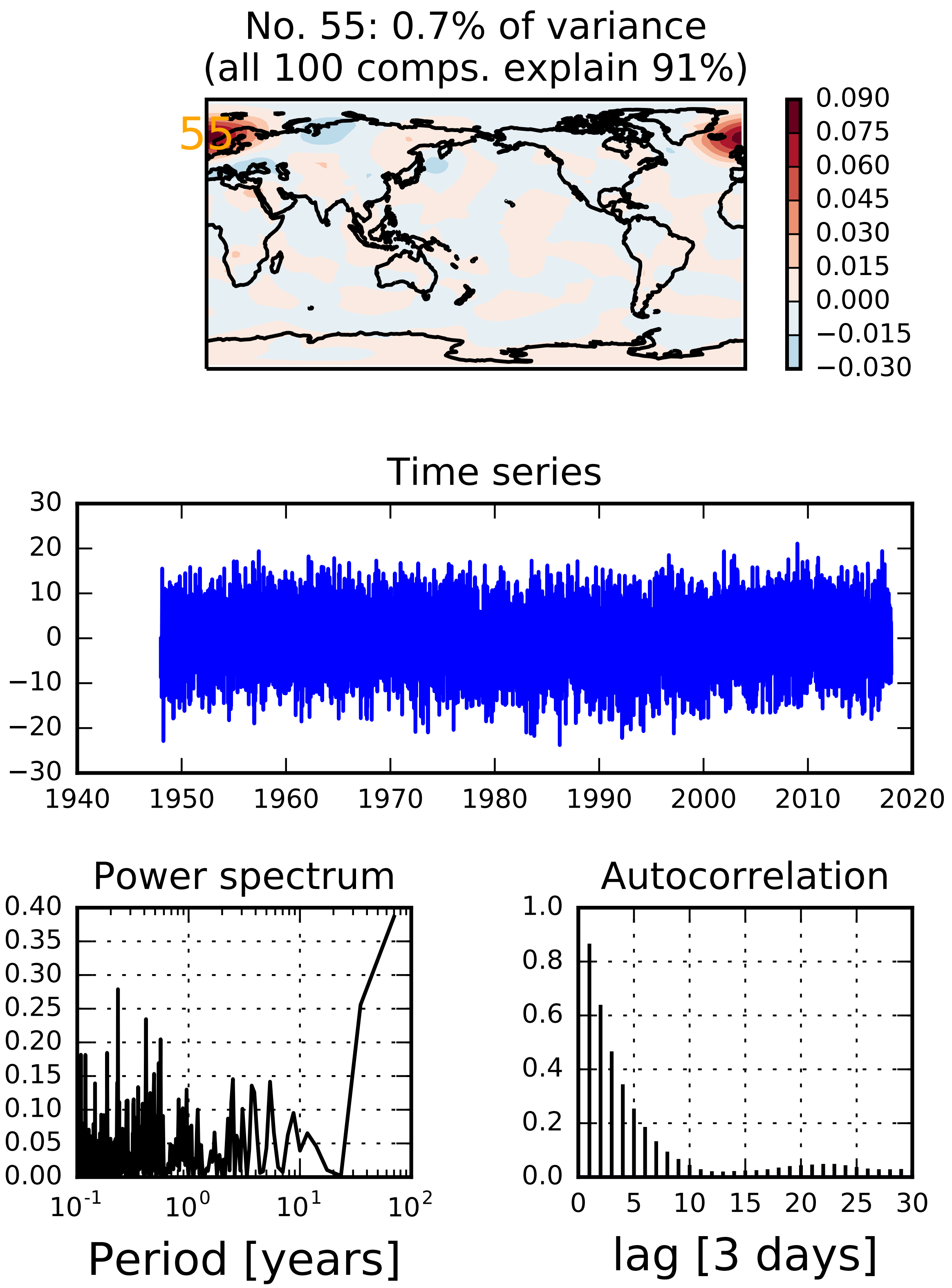
No. 55: 0.7% of variance
(all 100 comps. explain 91%)
55
0.090
0.075
0.060
0.045
0.030
0.015
0.000
−0.015
−0.030
Time series
30
20
10
0
−10
−20
−30
1940
1950
1960
1970
1980
1990
2000
2010
2020
Power spectrum
0.40
0.35
0.30
0.25
0.20
0.15
0.10
0.05
0.00
10^{-1}
10^{0}
10^{1}
10^{2}
Period [years]
Autocorrelation
1.0
0.8
0.6
0.4
0.2
0.0
0
5
10
15
20
25
30
lag [3 days]

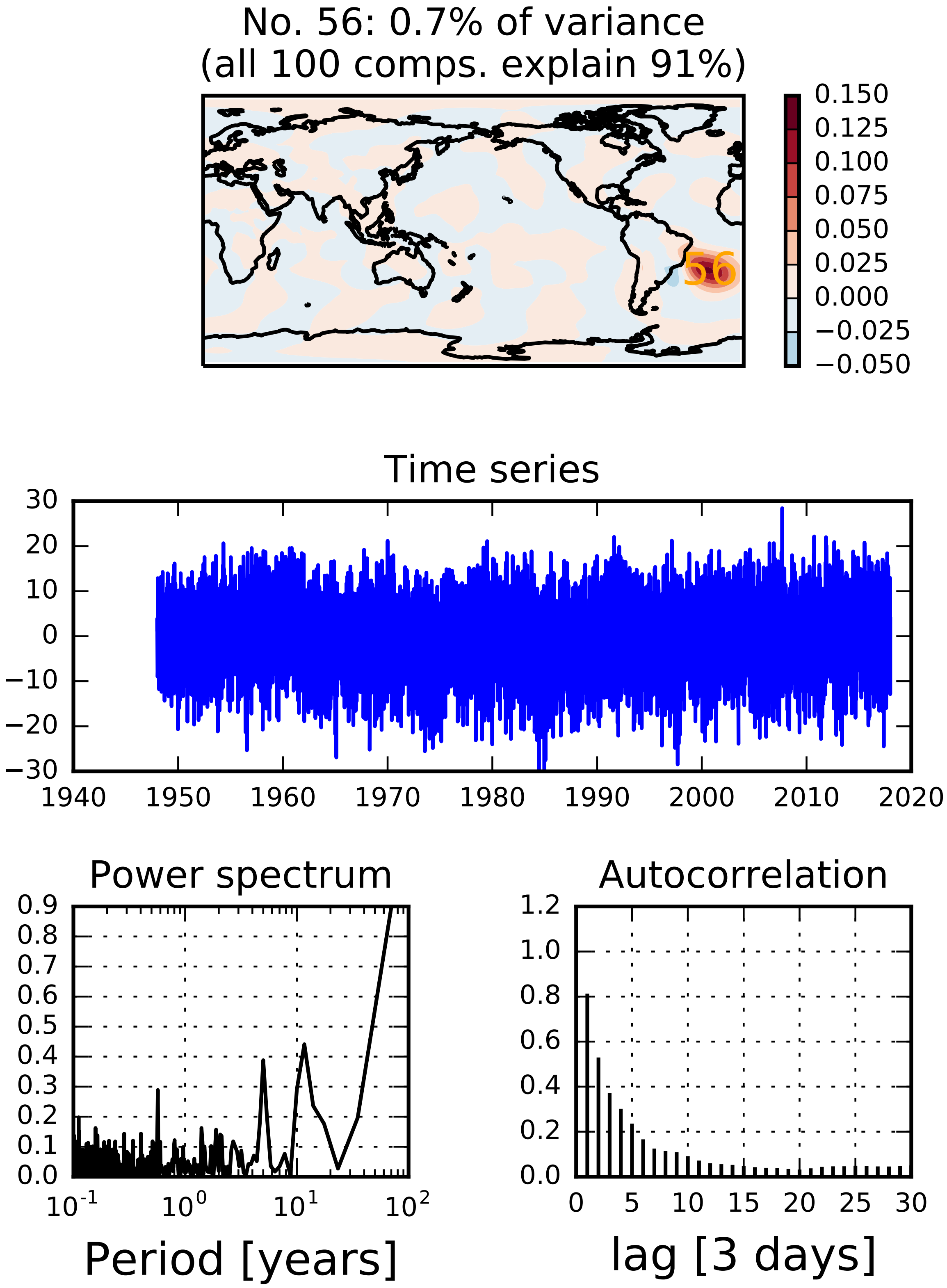
No. 56: 0.7% of variance
(all 100 comps. explain 91%)
56
0.150
0.125
0.100
0.075
0.050
0.025
0.000
−0.025
−0.050
Time series
30
20
10
0
−10
−20
−30
1940
1950
1960
1970
1980
1990
2000
2010
2020
Power spectrum
0.9
0.8
0.7
0.6
0.5
0.4
0.3
0.2
0.1
0.0
10^{-1}
10^{0}
10^{1}
10^{2}
Period [years]
Autocorrelation
1.2
1.0
0.8
0.6
0.4
0.2
0.0
0
5
10
15
20
25
30
lag [3 days]

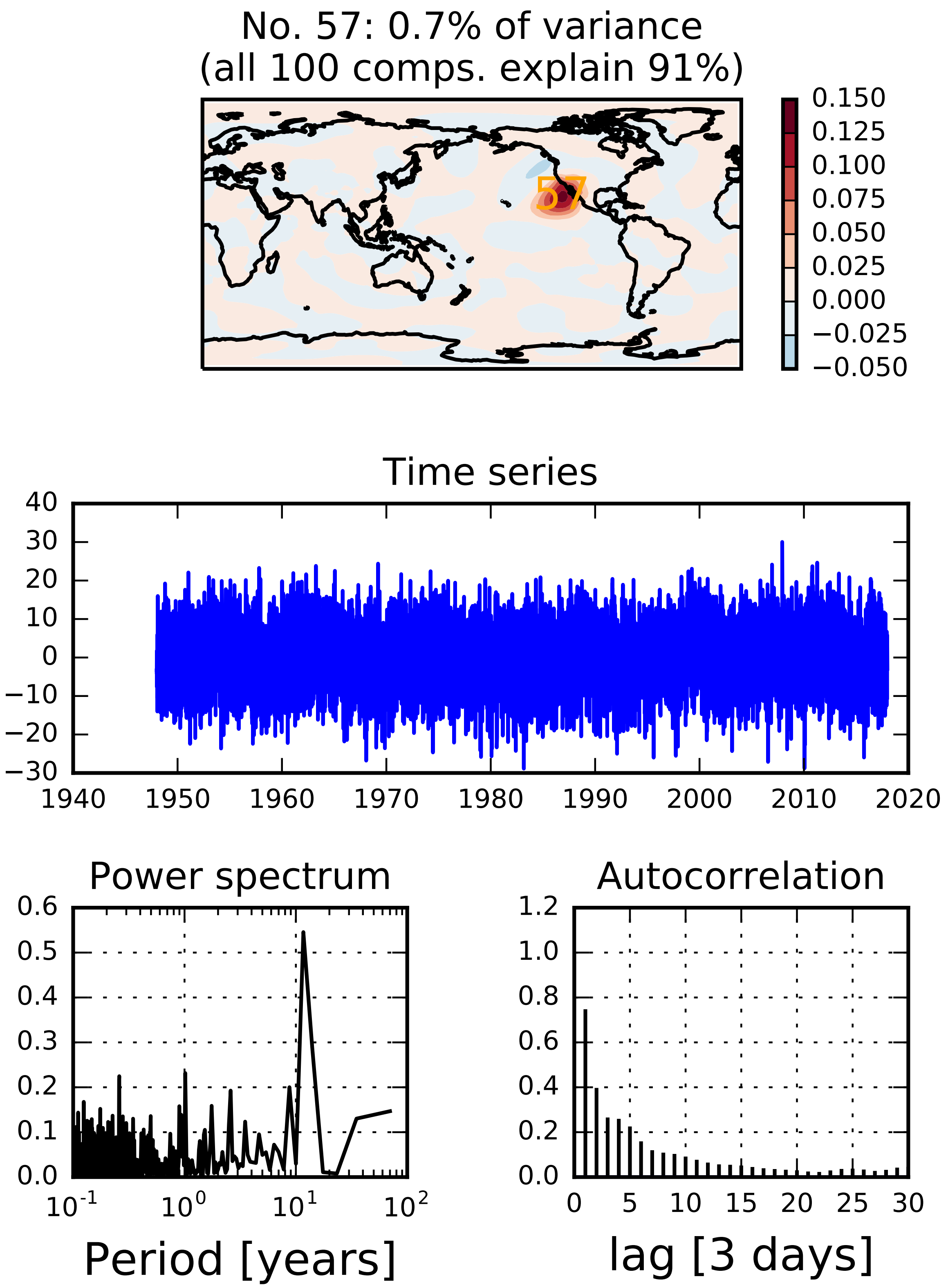
No. 57: 0.7% of variance
(all 100 comps. explain 91%)
57
0.150
0.125
0.100
0.075
0.050
0.025
0.000
−0.025
−0.050
Time series
40
30
20
10
0
−10
−20
−30
1940
1950
1960
1970
1980
1990
2000
2010
2020
Power spectrum
0.6
0.5
0.4
0.3
0.2
0.1
0.0
10^{-1}
10^{0}
10^{1}
10^{2}
Period [years]
Autocorrelation
1.2
1.0
0.8
0.6
0.4
0.2
0.0
0
5
10
15
20
25
30
lag [3 days]

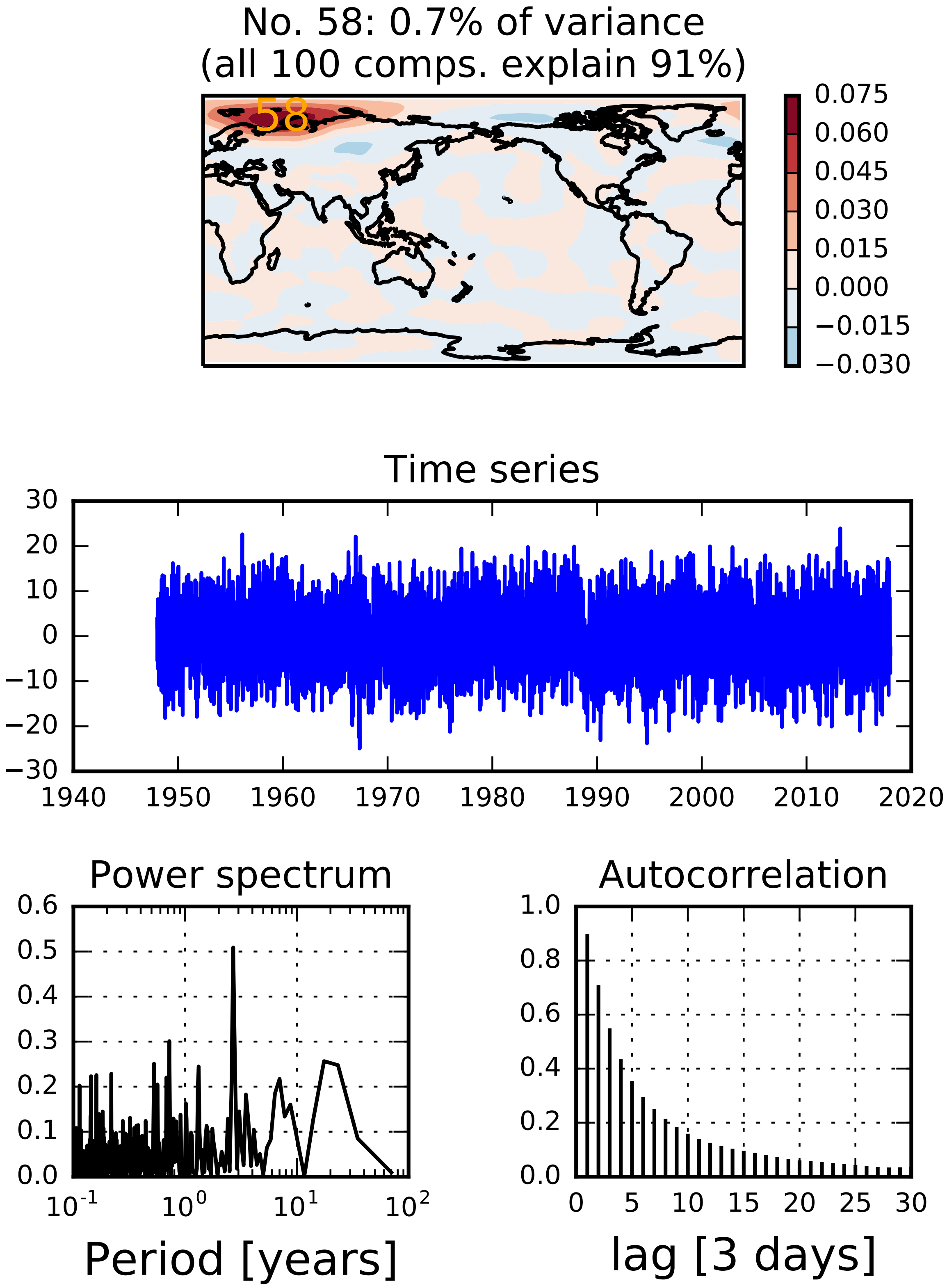
No. 58: 0.7% of variance
(all 100 comps. explain 91%)
58
0.075
0.060
0.045
0.030
0.015
0.000
−0.015
−0.030
Time series
30
20
10
0
−10
−20
−30
1940
1950
1960
1970
1980
1990
2000
2010
2020
Power spectrum
0.6
0.5
0.4
0.3
0.2
0.1
0.0
10^{-1}
10^{0}
10^{1}
10^{2}
Period [years]
Autocorrelation
1.0
0.8
0.6
0.4
0.2
0.0
0 5 10 15 20 25 30
lag [3 days]

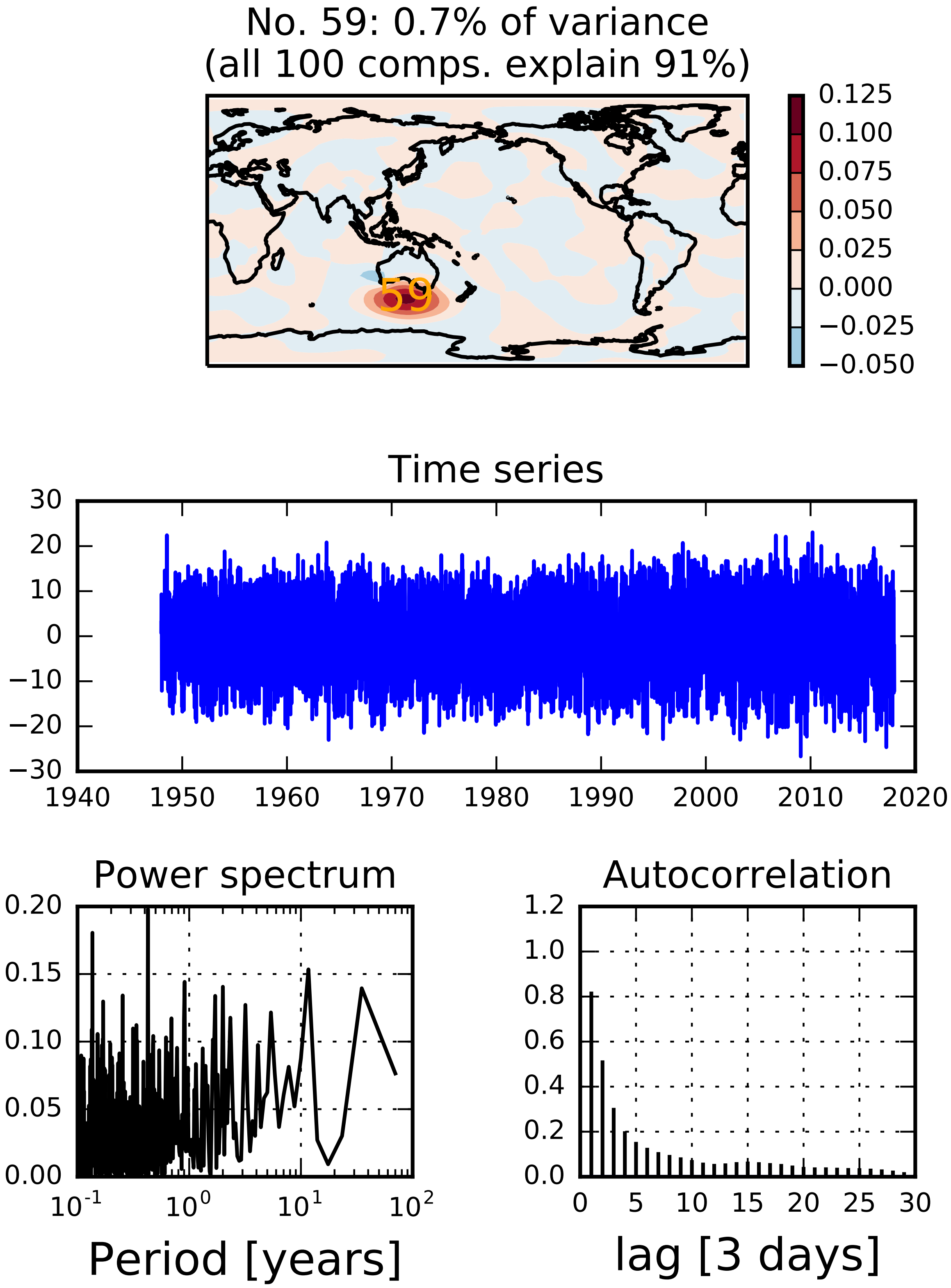
No. 59: 0.7% of variance
(all 100 comps. explain 91%)
59
0.125
0.100
0.075
0.050
0.025
0.000
−0.025
−0.050
Time series
30
20
10
0
−10
−20
−30
1940
1950
1960
1970
1980
1990
2000
2010
2020
Power spectrum
0.20
0.15
0.10
0.05
0.00
10^{-1}
10^{0}
10^{1}
10^{2}
Period [years]
Autocorrelation
1.2
1.0
0.8
0.6
0.4
0.2
0.0
0
5
10
15
20
25
30
lag [3 days]

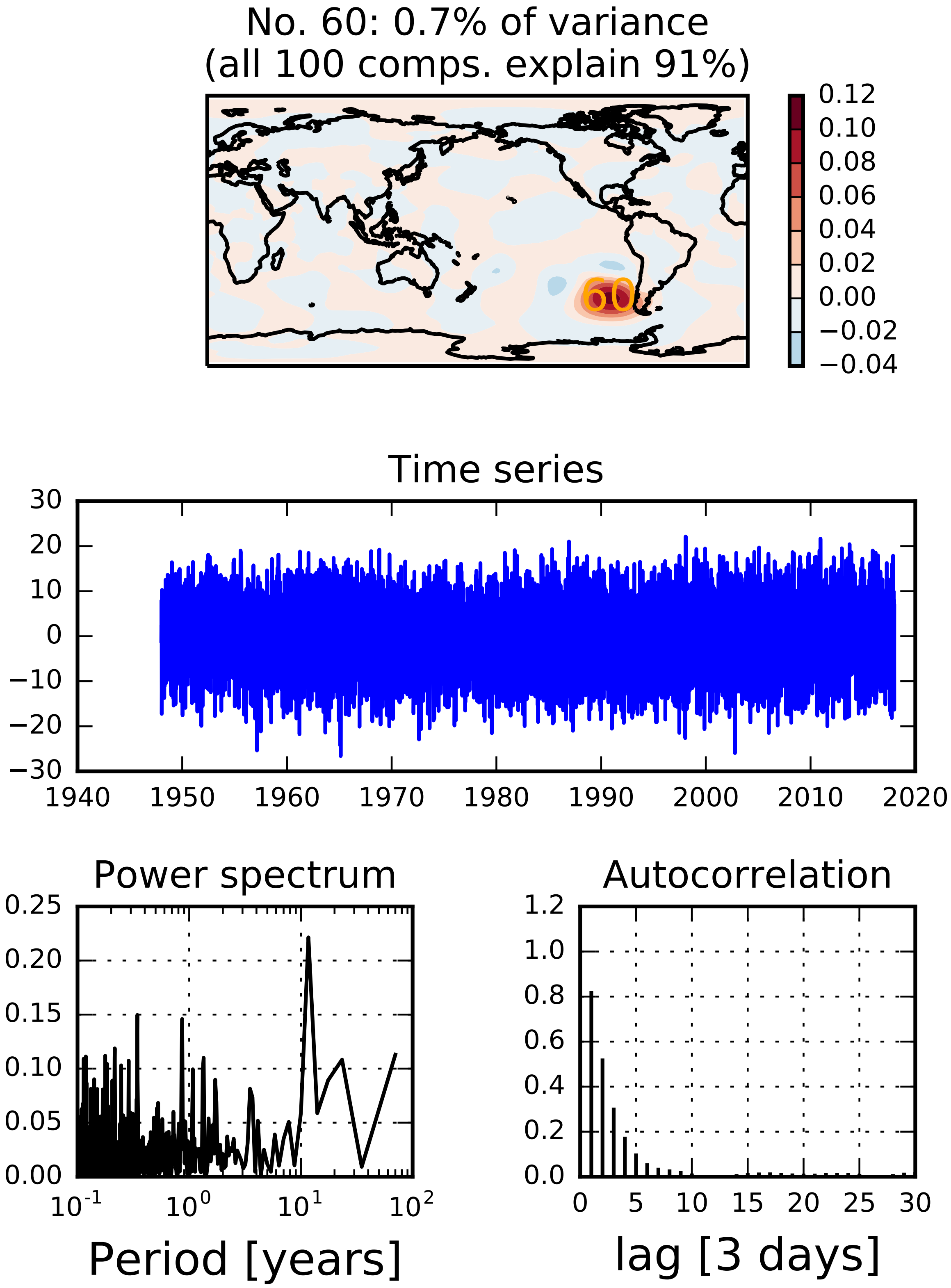
No. 60: 0.7% of variance
(all 100 comps. explain 91%)
0.12
0.10
0.08
0.06
0.04
0.02
0.00
−0.02
−0.04
60
Time series
30
20
10
0
−10
−20
−30
1940
1950
1960
1970
1980
1990
2000
2010
2020
Power spectrum
0.25
0.20
0.15
0.10
0.05
0.00
10^{-1}
10^{0}
10^{1}
10^{2}
Period [years]
Autocorrelation
1.2
1.0
0.8
0.6
0.4
0.2
0.0
0
5
10
15
20
25
30
lag [3 days]

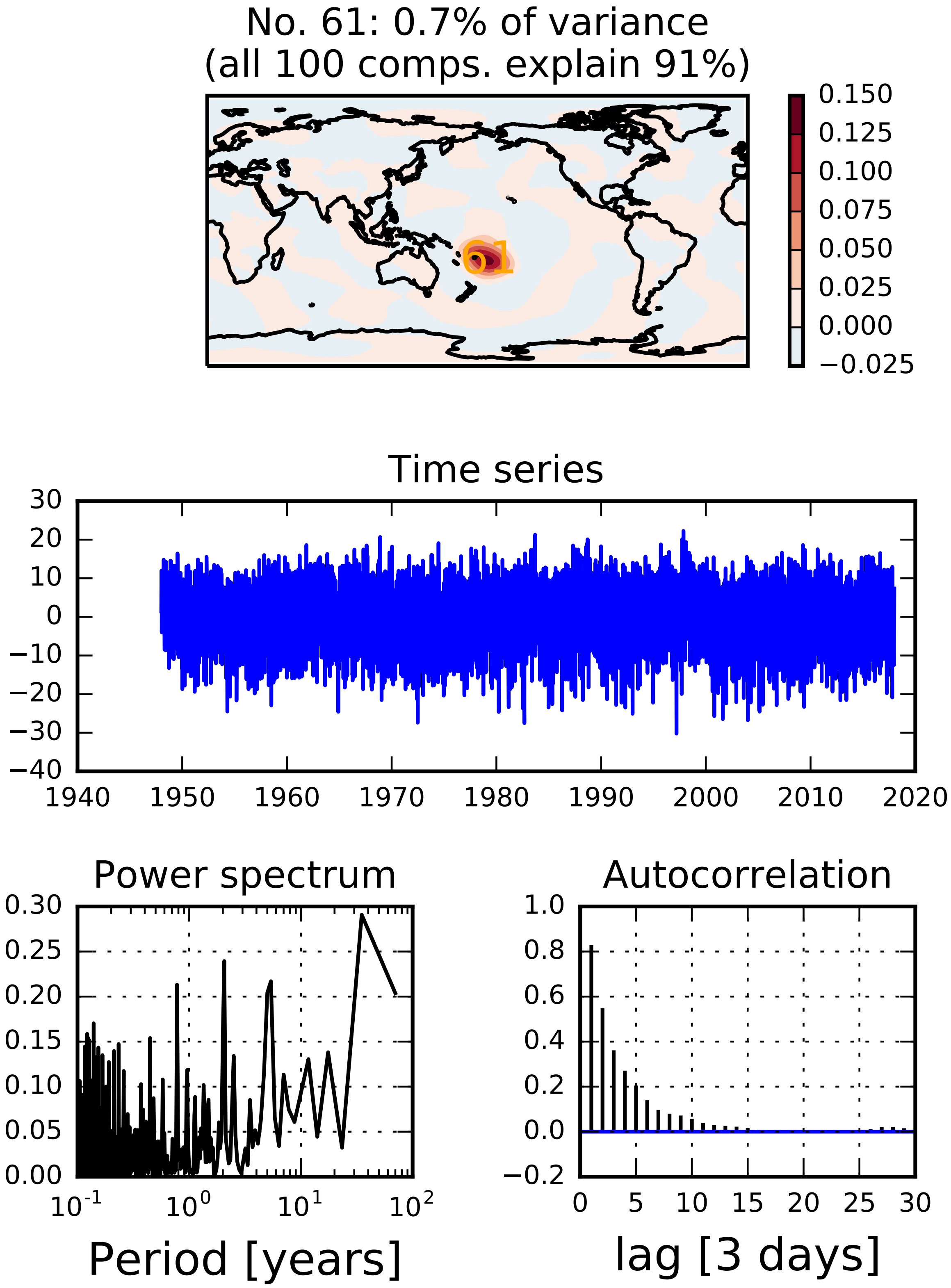
No. 61: 0.7% of variance
(all 100 comps. explain 91%)
0.150
0.125
0.100
0.075
0.050
0.025
0.000
−0.025
61
Time series
30
20
10
0
−10
−20
−30
−40
1940
1950
1960
1970
1980
1990
2000
2010
2020
Power spectrum
0.30
0.25
0.20
0.15
0.10
0.05
0.00
10^{-1}
10^{0}
10^{1}
10^{2}
Period [years]
Autocorrelation
1.0
0.8
0.6
0.4
0.2
0.0
−0.2
0
5
10
15
20
25
30
lag [3 days]

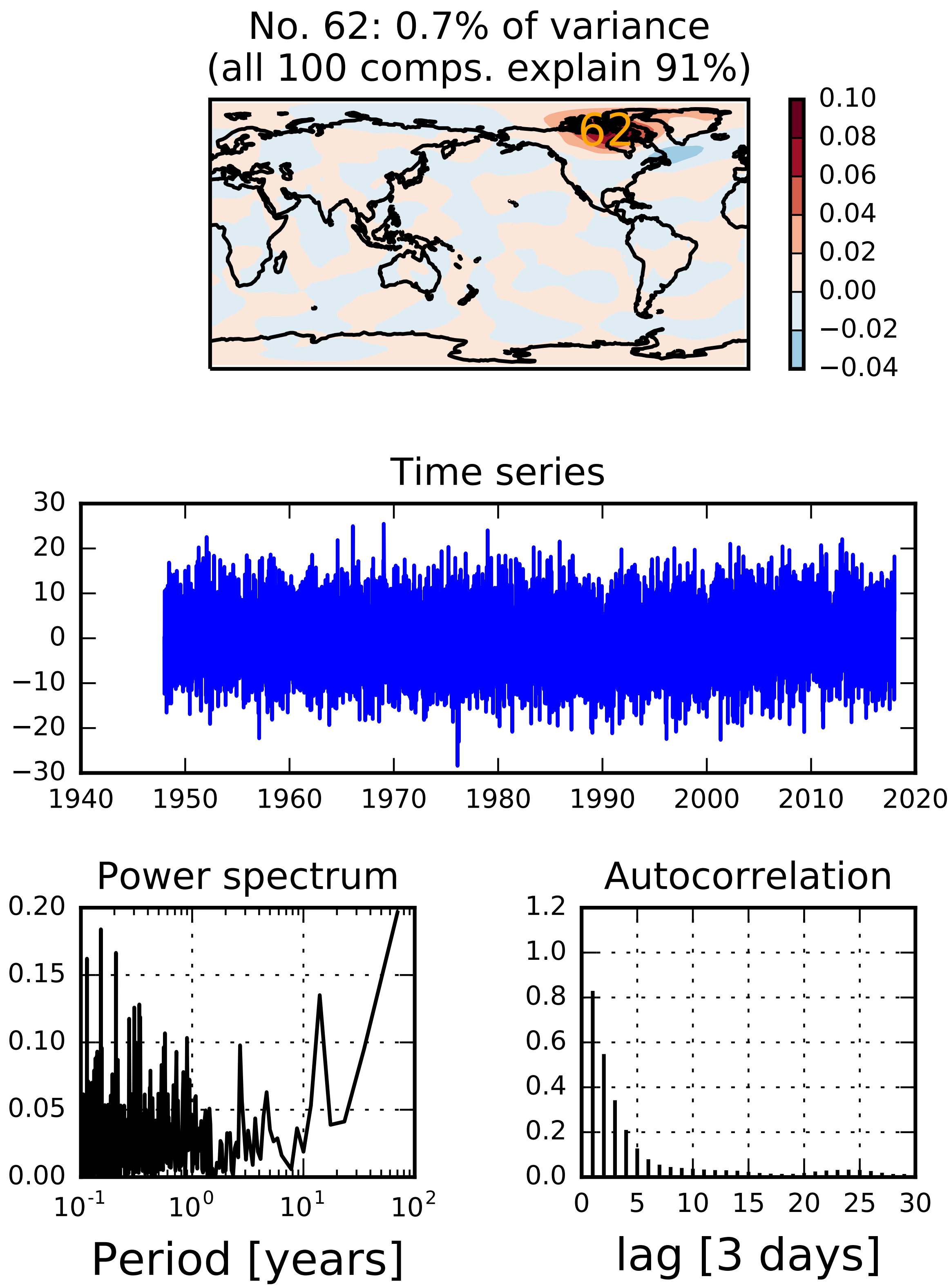
No. 62: 0.7% of variance
(all 100 comps. explain 91%)
62
0.10
0.08
0.06
0.04
0.02
0.00
−0.02
−0.04
Time series
30
20
10
0
−10
−20
−30
1940
1950
1960
1970
1980
1990
2000
2010
2020
Power spectrum
0.20
0.15
0.10
0.05
0.00
10^{-1}
10^{0}
10^{1}
10^{2}
Period [years]
Autocorrelation
1.2
1.0
0.8
0.6
0.4
0.2
0.0
0
5
10
15
20
25
30
lag [3 days]

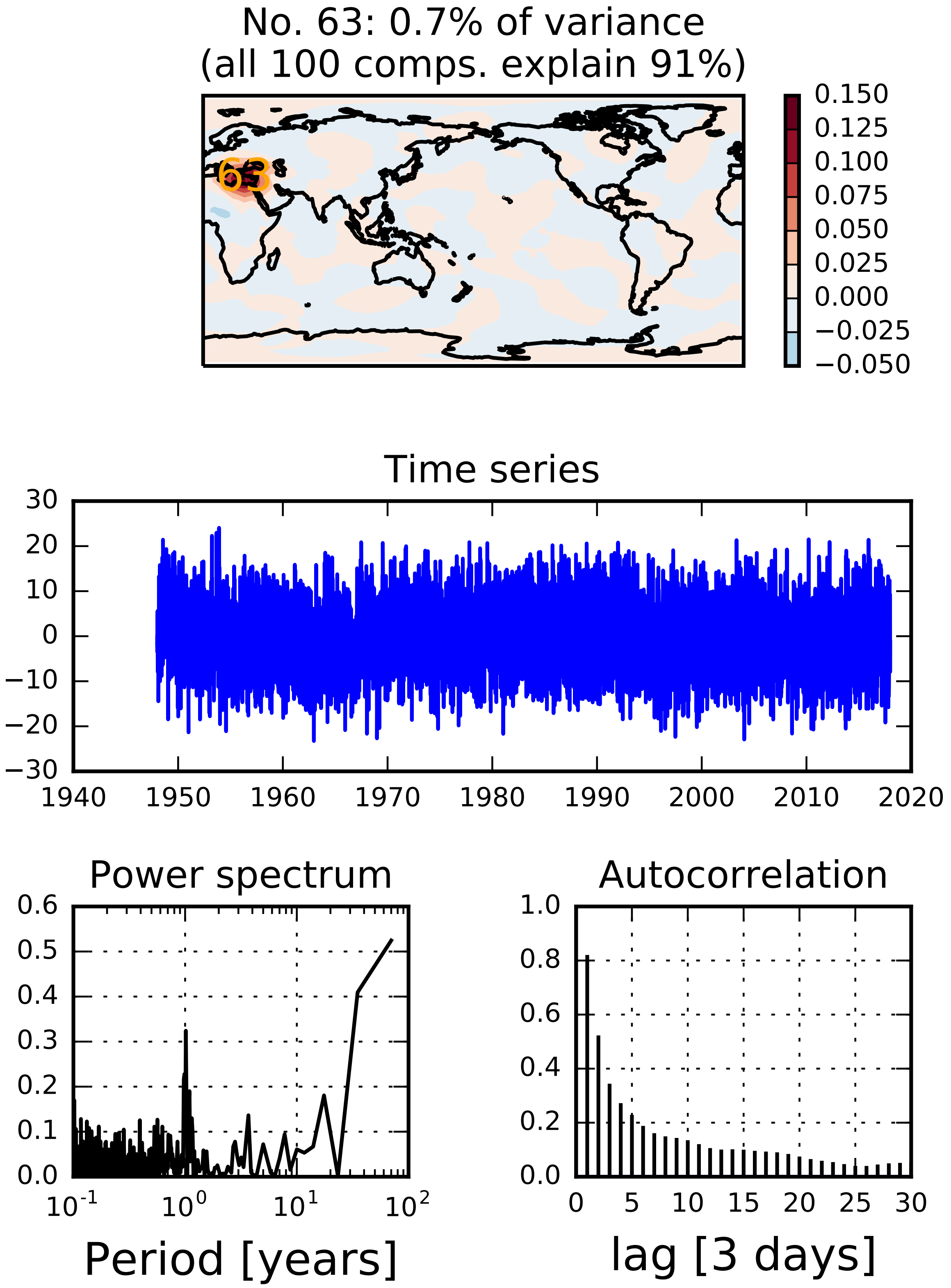
No. 63: 0.7% of variance
(all 100 comps. explain 91%)
63
0.150
0.125
0.100
0.075
0.050
0.025
0.000
−0.025
−0.050
Time series
30
20
10
0
−10
−20
−30
1940
1950
1960
1970
1980
1990
2000
2010
2020
Power spectrum
0.6
0.5
0.4
0.3
0.2
0.1
0.0
10^{-1}
10^{0}
10^{1}
10^{2}
Period [years]
Autocorrelation
1.0
0.8
0.6
0.4
0.2
0.0
0
5
10
15
20
25
30
lag [3 days]

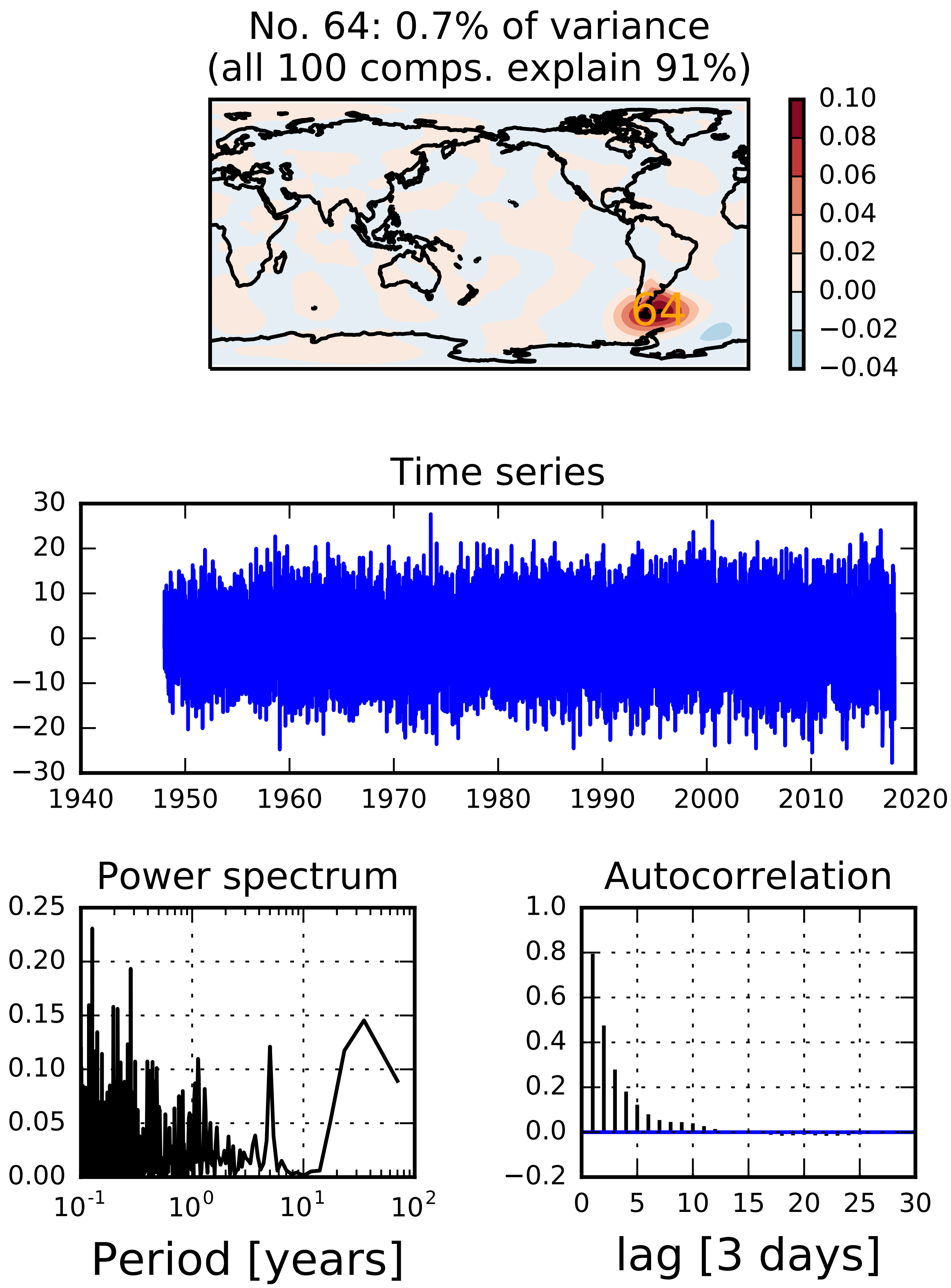
No. 64: 0.7% of variance
(all 100 comps. explain 91%)
64
0.10
0.08
0.06
0.04
0.02
0.00
−0.02
−0.04
Time series
30
20
10
0
−10
−20
−30
1940
1950
1960
1970
1980
1990
2000
2010
2020
Power spectrum
0.25
0.20
0.15
0.10
0.05
0.00
10^{-1}
10^{0}
10^{1}
10^{2}
Period [years]
Autocorrelation
1.0
0.8
0.6
0.4
0.2
0.0
−0.2
0
5
10
15
20
25
30
lag [3 days]

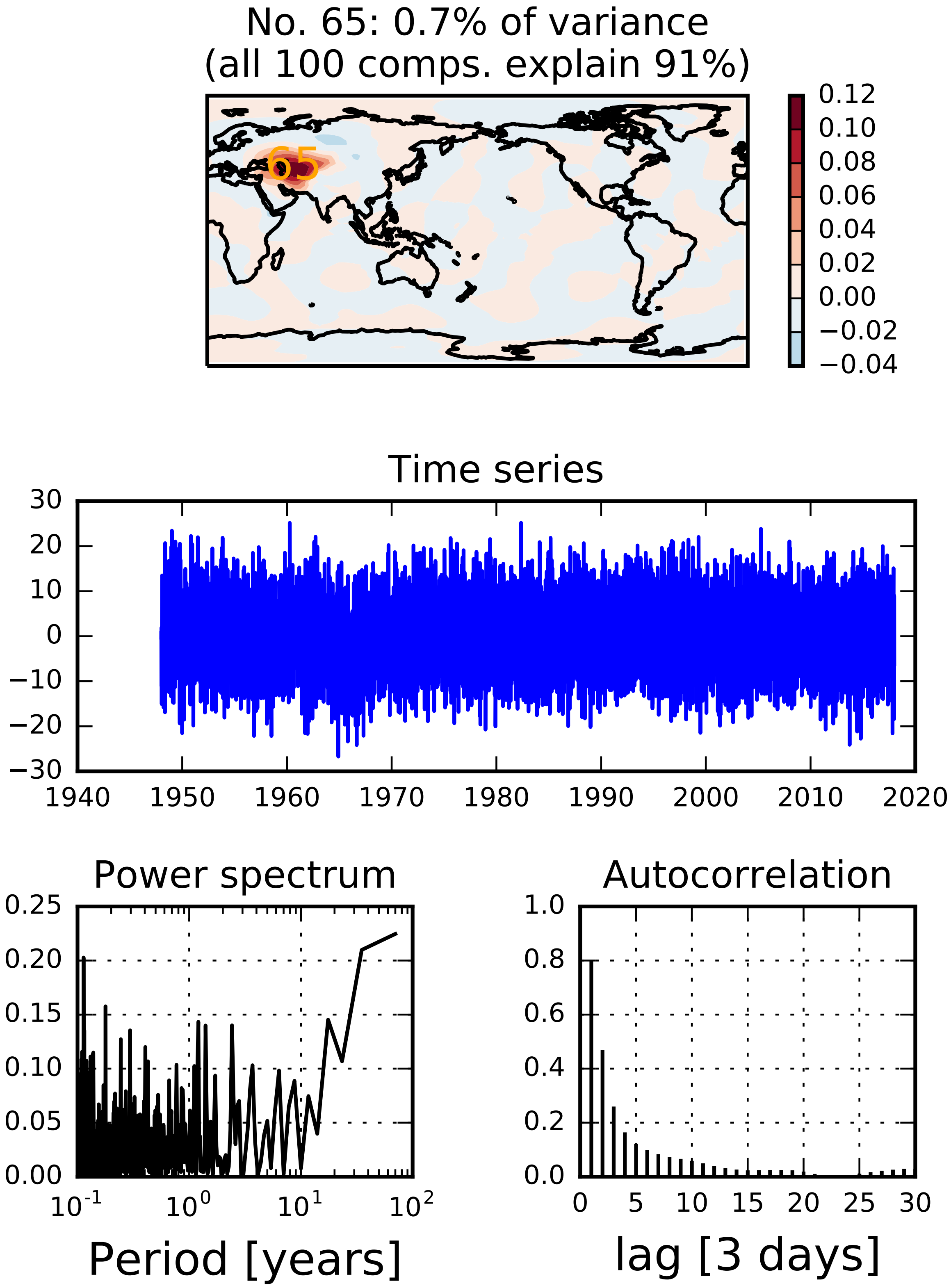
No. 65: 0.7% of variance
(all 100 comps. explain 91%)
65
0.12
0.10
0.08
0.06
0.04
0.02
0.00
−0.02
−0.04
Time series
30
20
10
0
−10
−20
−30
1940
1950
1960
1970
1980
1990
2000
2010
2020
Power spectrum
0.25
0.20
0.15
0.10
0.05
0.00
10^{-1}
10^{0}
10^{1}
10^{2}
Period [years]
Autocorrelation
1.0
0.8
0.6
0.4
0.2
0.0
0
5
10
15
20
25
30
lag [3 days]

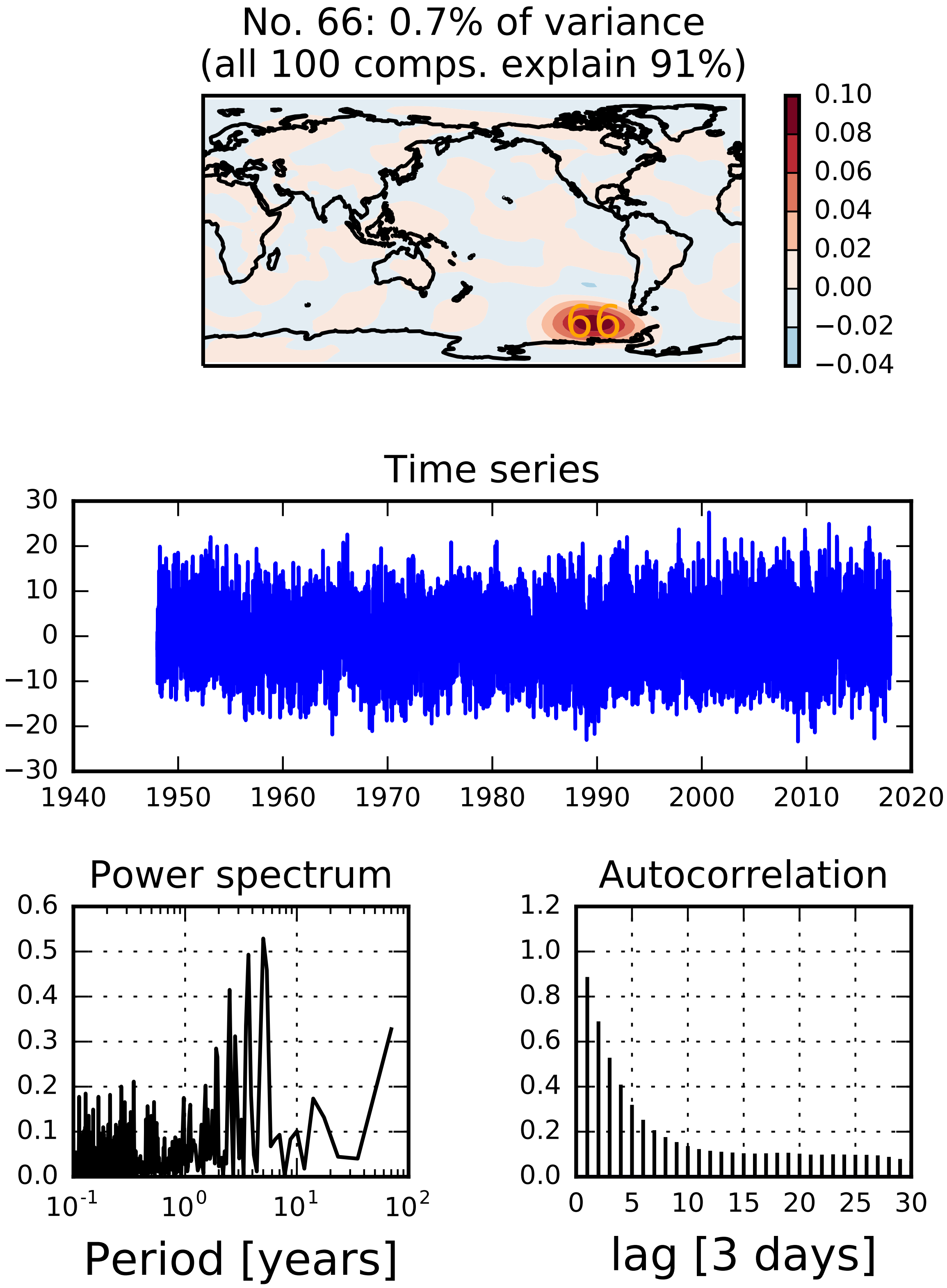
No. 66: 0.7% of variance
(all 100 comps. explain 91%)
66
0.10
0.08
0.06
0.04
0.02
0.00
−0.02
−0.04
Time series
30
20
10
0
−10
−20
−30
1940
1950
1960
1970
1980
1990
2000
2010
2020
Power spectrum
0.6
0.5
0.4
0.3
0.2
0.1
0.0
10^-1
10^0
10^1
10^2
Period [years]
Autocorrelation
1.2
1.0
0.8
0.6
0.4
0.2
0.0
0
5
10
15
20
25
30
lag [3 days]

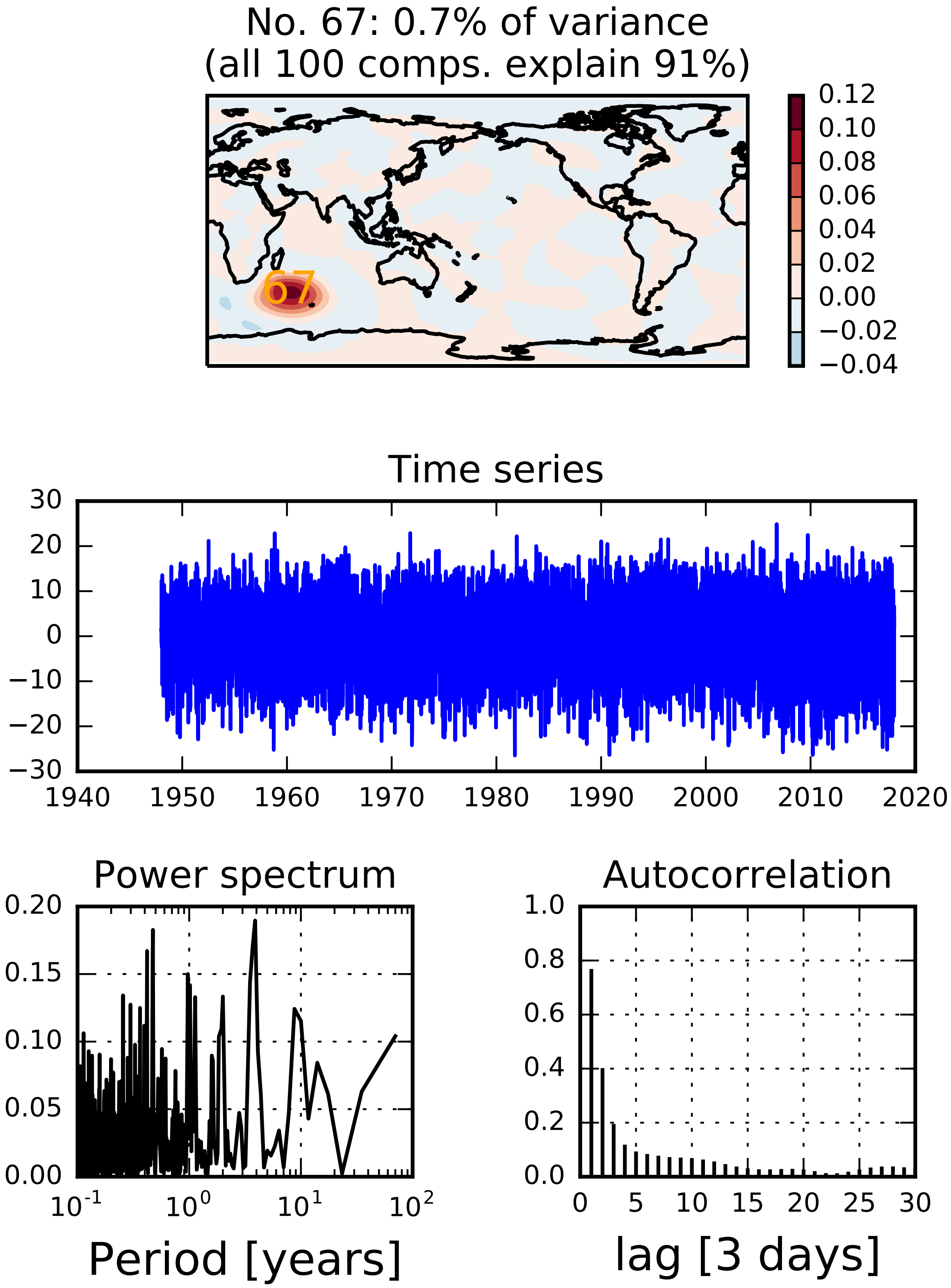
No. 67: 0.7% of variance
(all 100 comps. explain 91%)
67
0.12
0.10
0.08
0.06
0.04
0.02
0.00
−0.02
−0.04
Time series
30
20
10
0
−10
−20
−30
1940
1950
1960
1970
1980
1990
2000
2010
2020
Power spectrum
0.20
0.15
0.10
0.05
0.00
10^{-1}
10^{0}
10^{1}
10^{2}
Period [years]
Autocorrelation
1.0
0.8
0.6
0.4
0.2
0.0
0
5
10
15
20
25
30
lag [3 days]

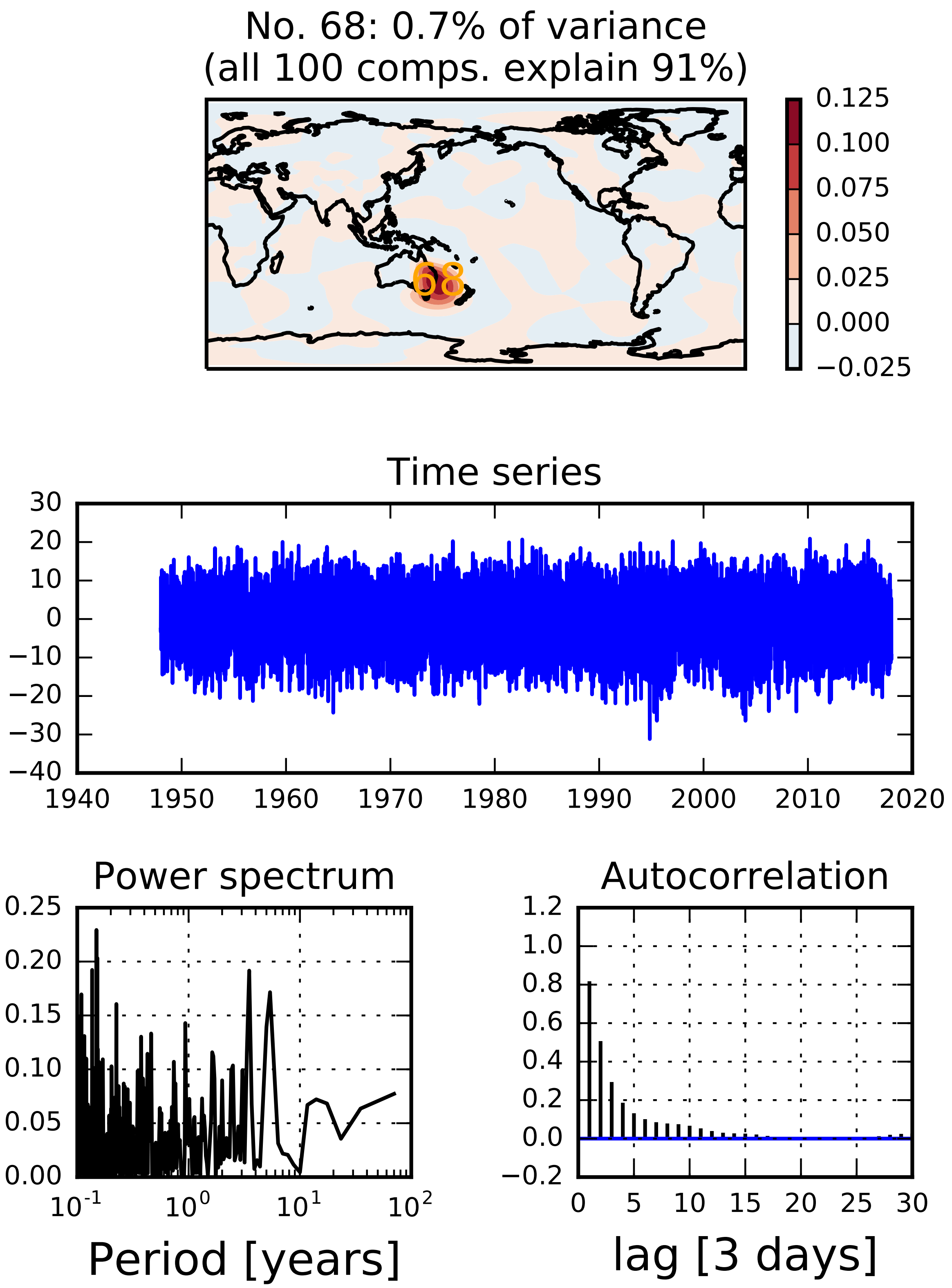
No. 68: 0.7% of variance
(all 100 comps. explain 91%)
68
0.125
0.100
0.075
0.050
0.025
0.000
−0.025
Time series
30
20
10
0
−10
−20
−30
−40
1940
1950
1960
1970
1980
1990
2000
2010
2020
Power spectrum
0.25
0.20
0.15
0.10
0.05
0.00
10^-1
10^0
10^1
10^2
Period [years]
Autocorrelation
1.2
1.0
0.8
0.6
0.4
0.2
0.0
−0.2
0
5
10
15
20
25
30
lag [3 days]

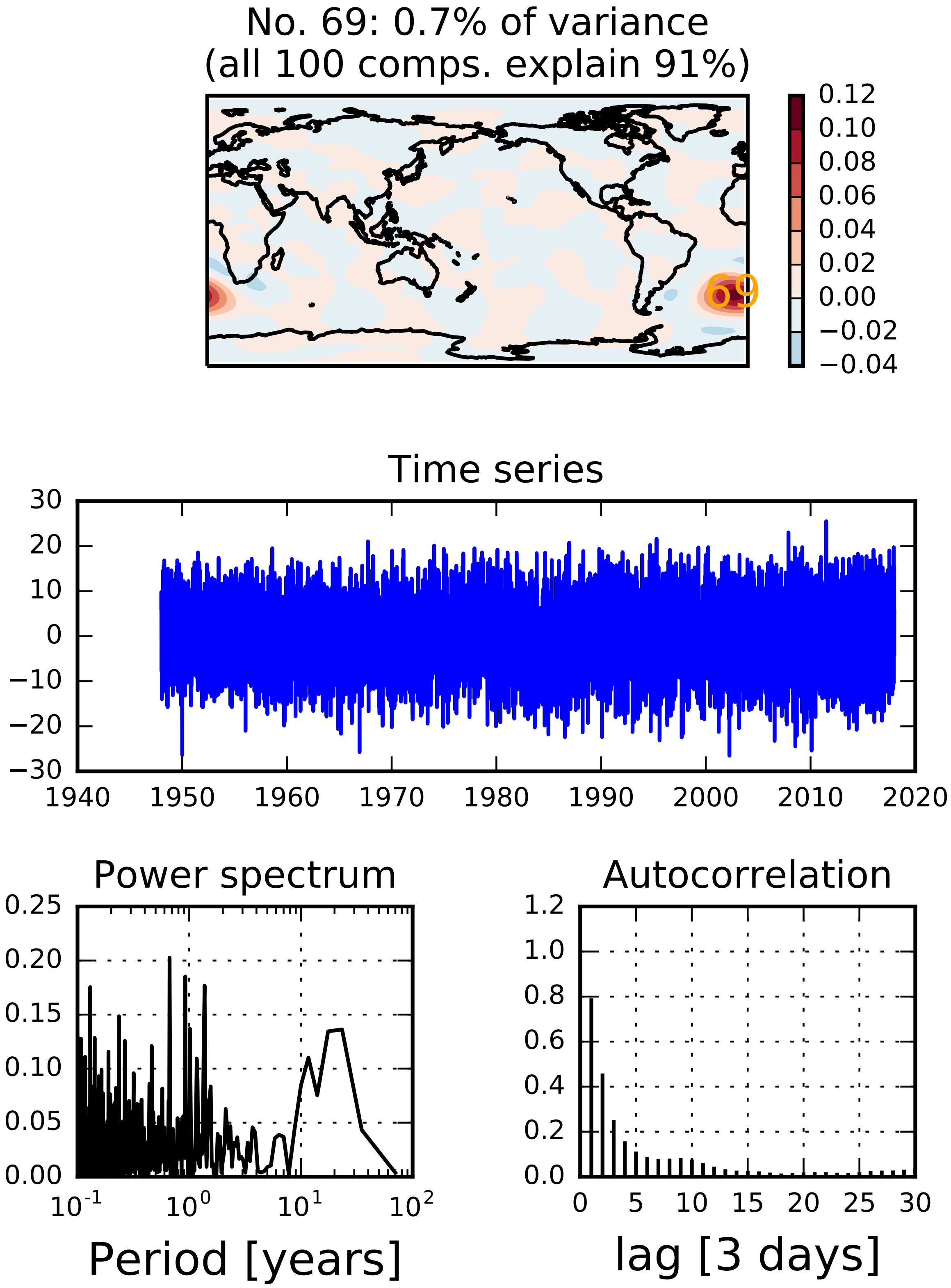
No. 69: 0.7% of variance
(all 100 comps. explain 91%)
69
0.12
0.10
0.08
0.06
0.04
0.02
0.00
−0.02
−0.04
Time series
30
20
10
0
−10
−20
−30
1940
1950
1960
1970
1980
1990
2000
2010
2020
Power spectrum
0.25
0.20
0.15
0.10
0.05
0.00
10^{-1}
10^{0}
10^{1}
10^{2}
Period [years]
Autocorrelation
1.2
1.0
0.8
0.6
0.4
0.2
0.0
0
5
10
15
20
25
30
lag [3 days]

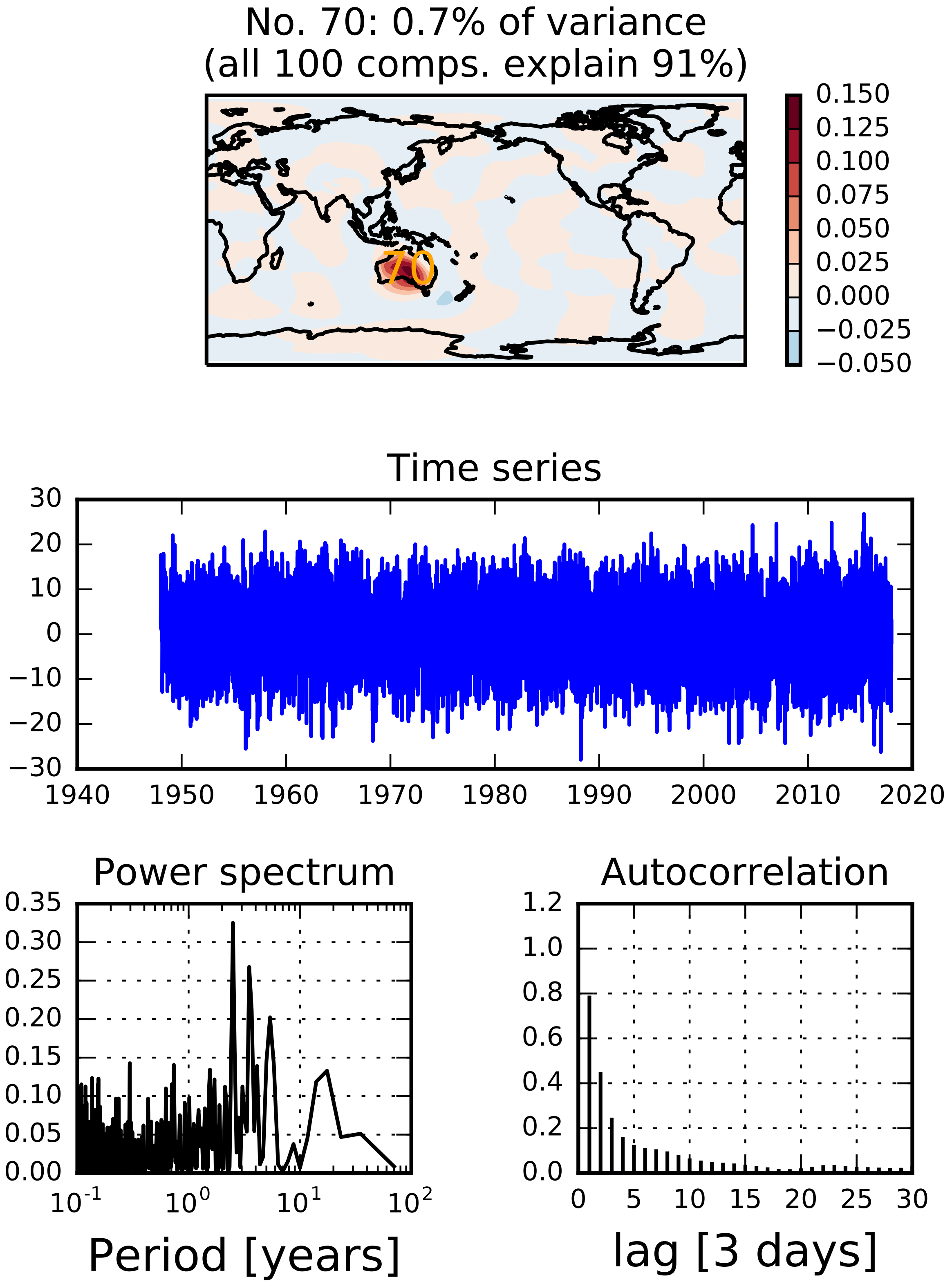
No. 70: 0.7% of variance
(all 100 comps. explain 91%)
70
0.150
0.125
0.100
0.075
0.050
0.025
0.000
−0.025
−0.050
Time series
30
20
10
0
−10
−20
−30
1940
1950
1960
1970
1980
1990
2000
2010
2020
Power spectrum
0.35
0.30
0.25
0.20
0.15
0.10
0.05
0.00
10^{-1}
10^{0}
10^{1}
10^{2}
Period [years]
Autocorrelation
1.2
1.0
0.8
0.6
0.4
0.2
0.0
0
5
10
15
20
25
30
lag [3 days]

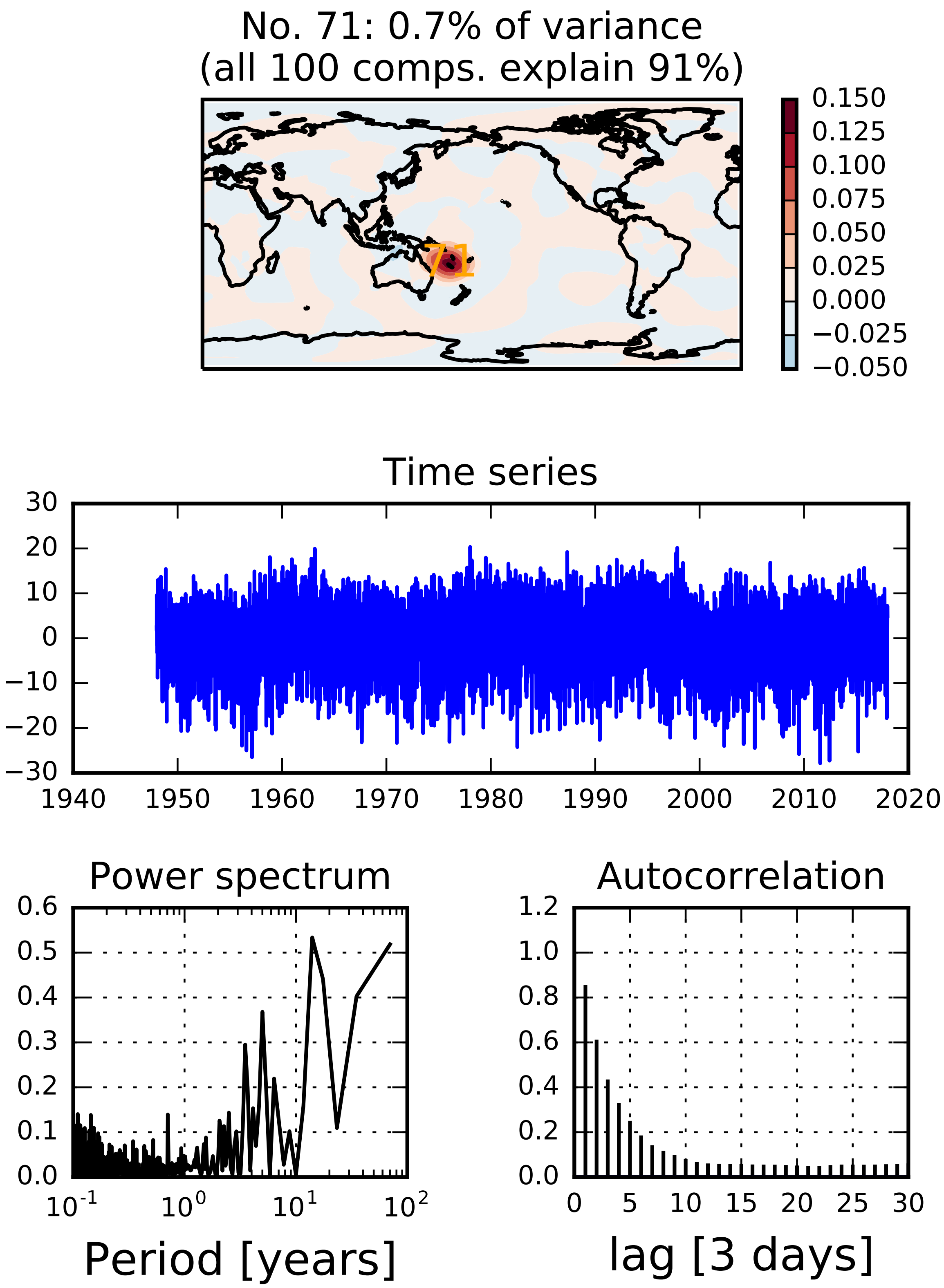
No. 71: 0.7% of variance
(all 100 comps. explain 91%)
71
0.150
0.125
0.100
0.075
0.050
0.025
0.000
−0.025
−0.050
Time series
30
20
10
0
−10
−20
−30
1940
1950
1960
1970
1980
1990
2000
2010
2020
Power spectrum
0.6
0.5
0.4
0.3
0.2
0.1
0.0
10^{-1}
10^{0}
10^{1}
10^{2}
Period [years]
Autocorrelation
1.2
1.0
0.8
0.6
0.4
0.2
0.0
0
5
10
15
20
25
30
lag [3 days]

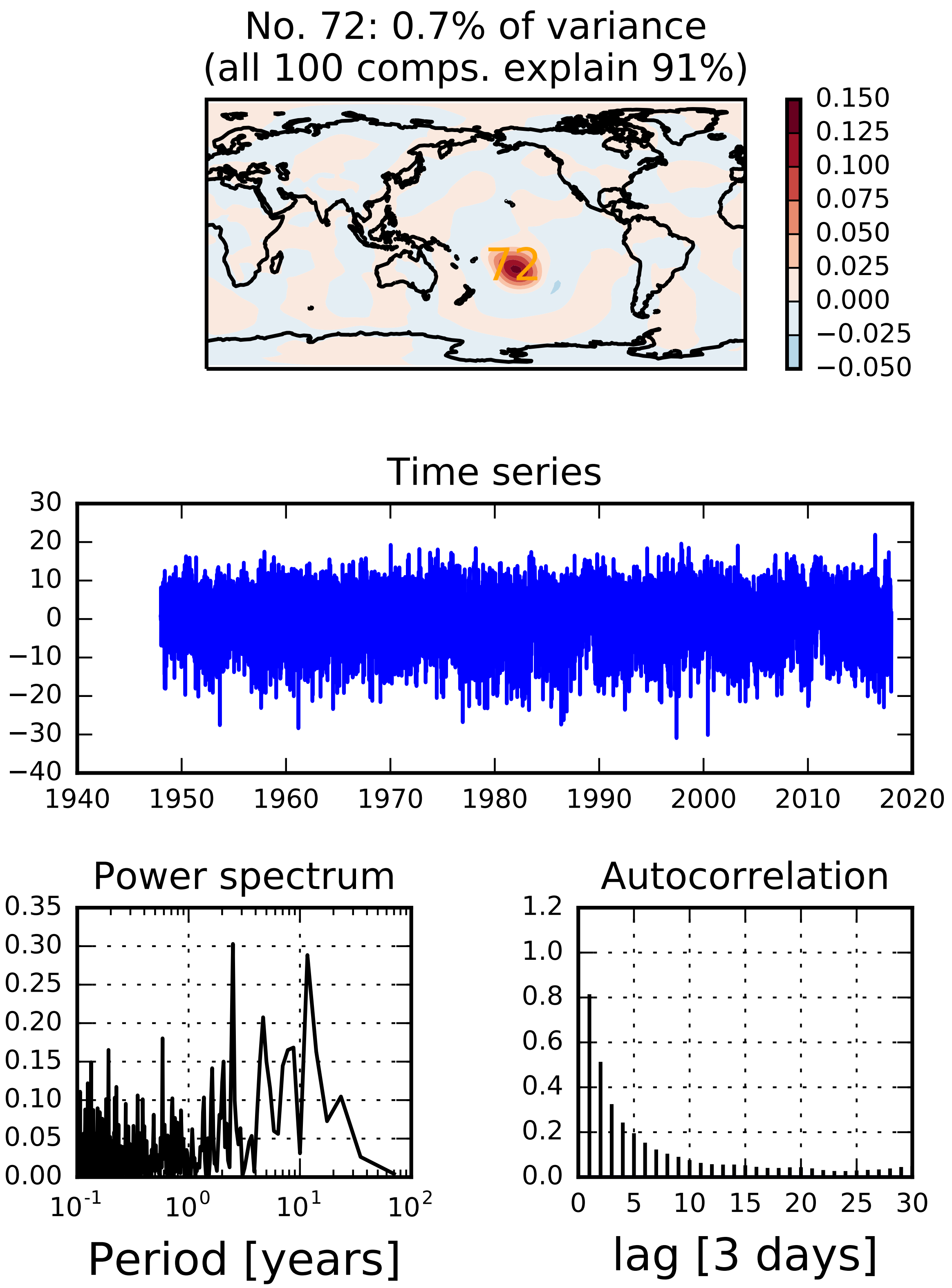
No. 72: 0.7% of variance
(all 100 comps. explain 91%)
72
0.150
0.125
0.100
0.075
0.050
0.025
0.000
−0.025
−0.050
Time series
30
20
10
0
−10
−20
−30
−40
1940
1950
1960
1970
1980
1990
2000
2010
2020
Power spectrum
0.35
0.30
0.25
0.20
0.15
0.10
0.05
0.00
10^{-1}
10^{0}
10^{1}
10^{2}
Period [years]
Autocorrelation
1.2
1.0
0.8
0.6
0.4
0.2
0.0
0
5
10
15
20
25
30
lag [3 days]

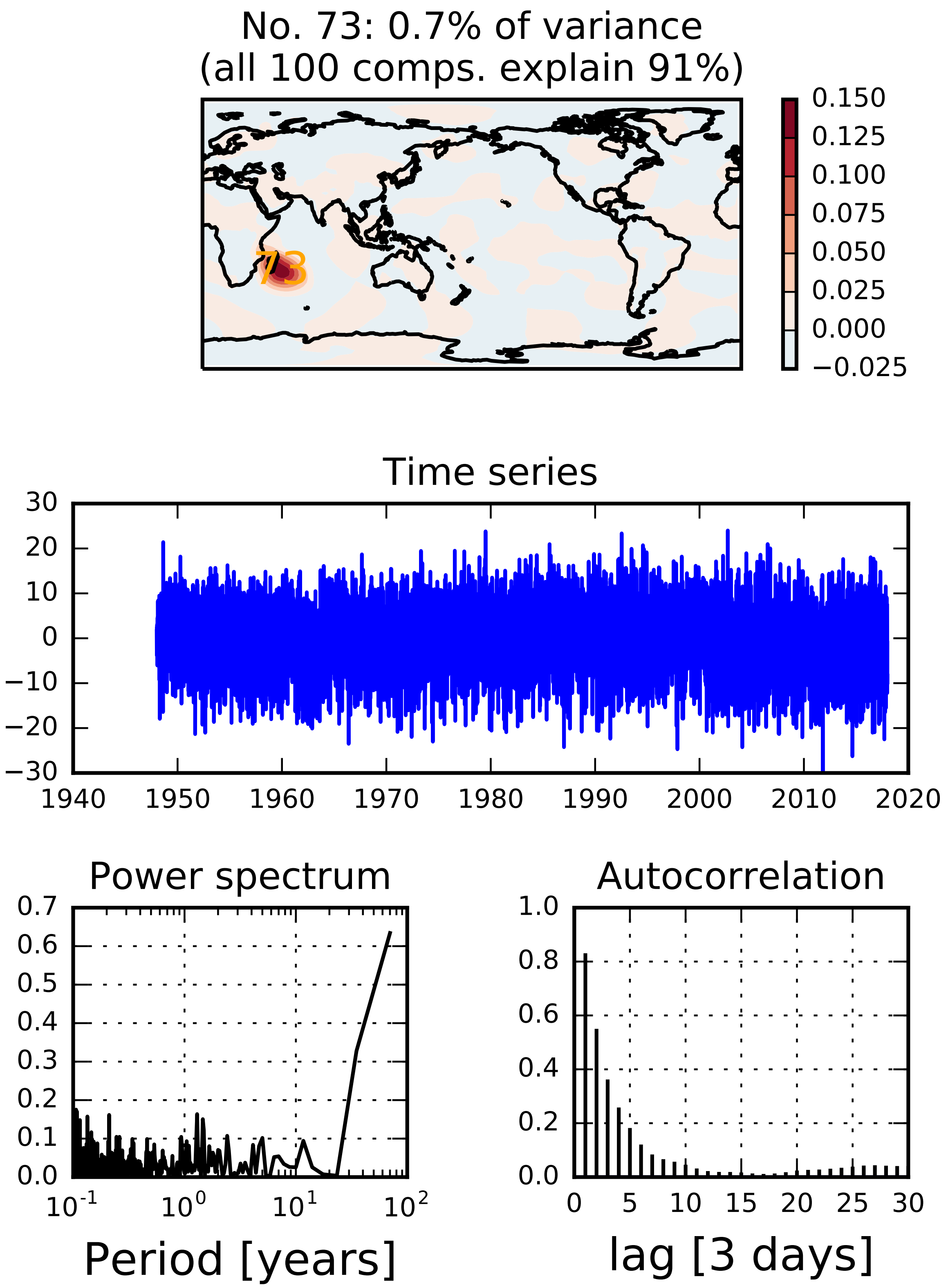
No. 73: 0.7% of variance
(all 100 comps. explain 91%)
73
0.150
0.125
0.100
0.075
0.050
0.025
0.000
−0.025
Time series
30
20
10
0
−10
−20
−30
1940
1950
1960
1970
1980
1990
2000
2010
2020
Power spectrum
0.7
0.6
0.5
0.4
0.3
0.2
0.1
0.0
10^{-1}
10^{0}
10^{1}
10^{2}
Period [years]
Autocorrelation
1.0
0.8
0.6
0.4
0.2
0.0
0
5
10
15
20
25
30
lag [3 days]

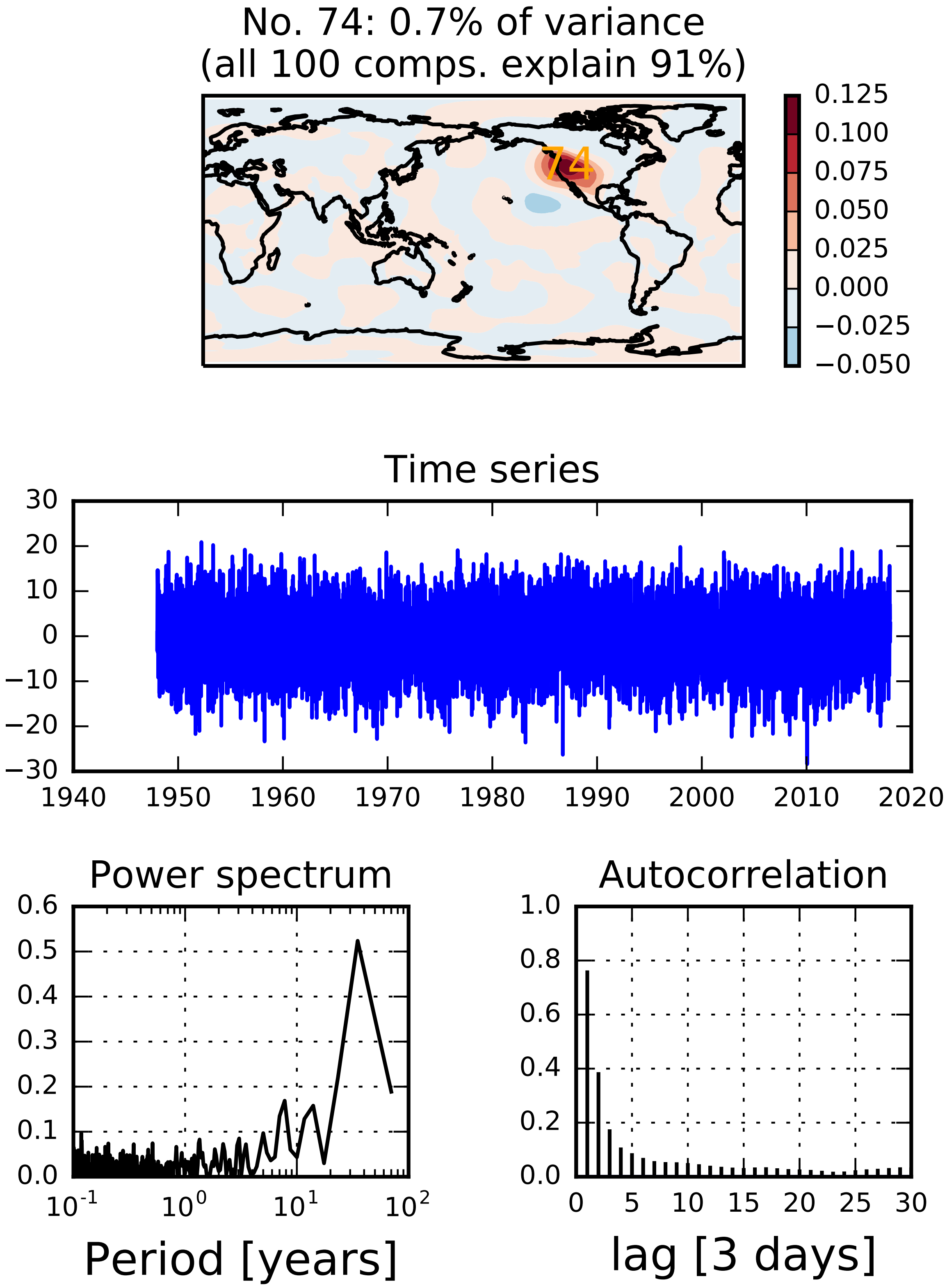
No. 74: 0.7% of variance
(all 100 comps. explain 91%)
74
0.125
0.100
0.075
0.050
0.025
0.000
−0.025
−0.050
Time series
30
20
10
0
−10
−20
−30
1940
1950
1960
1970
1980
1990
2000
2010
2020
Power spectrum
0.6
0.5
0.4
0.3
0.2
0.1
0.0
10^-1
10^0
10^1
10^2
Period [years]
Autocorrelation
1.0
0.8
0.6
0.4
0.2
0.0
0
5
10
15
20
25
30
lag [3 days]

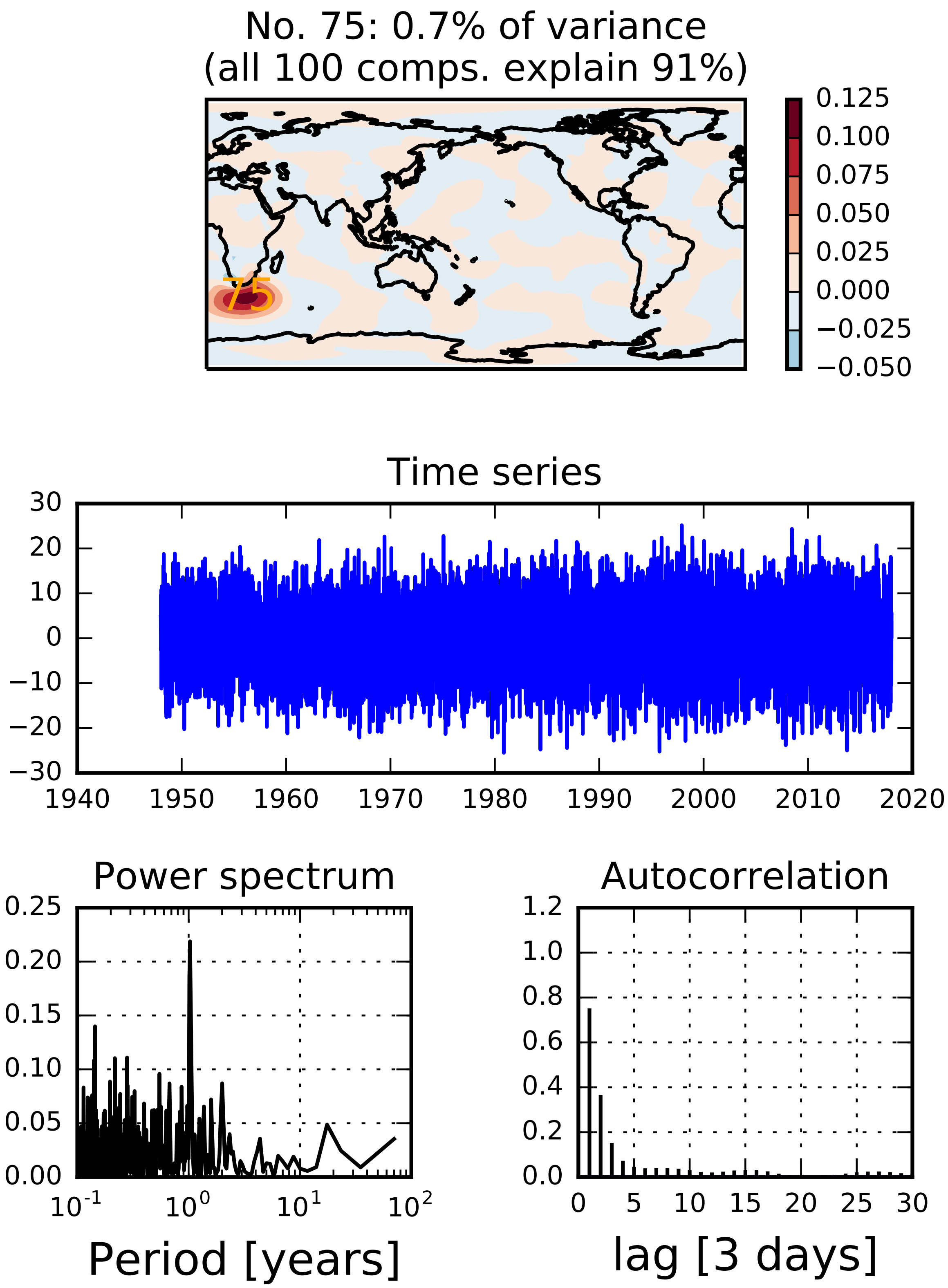
No. 75: 0.7% of variance
(all 100 comps. explain 91%)
75
0.125
0.100
0.075
0.050
0.025
0.000
−0.025
−0.050
Time series
30
20
10
0
−10
−20
−30
1940
1950
1960
1970
1980
1990
2000
2010
2020
Power spectrum
0.25
0.20
0.15
0.10
0.05
0.00
10^{-1}
10^{0}
10^{1}
10^{2}
Period [years]
Autocorrelation
1.2
1.0
0.8
0.6
0.4
0.2
0.0
0
5
10
15
20
25
30
lag [3 days]

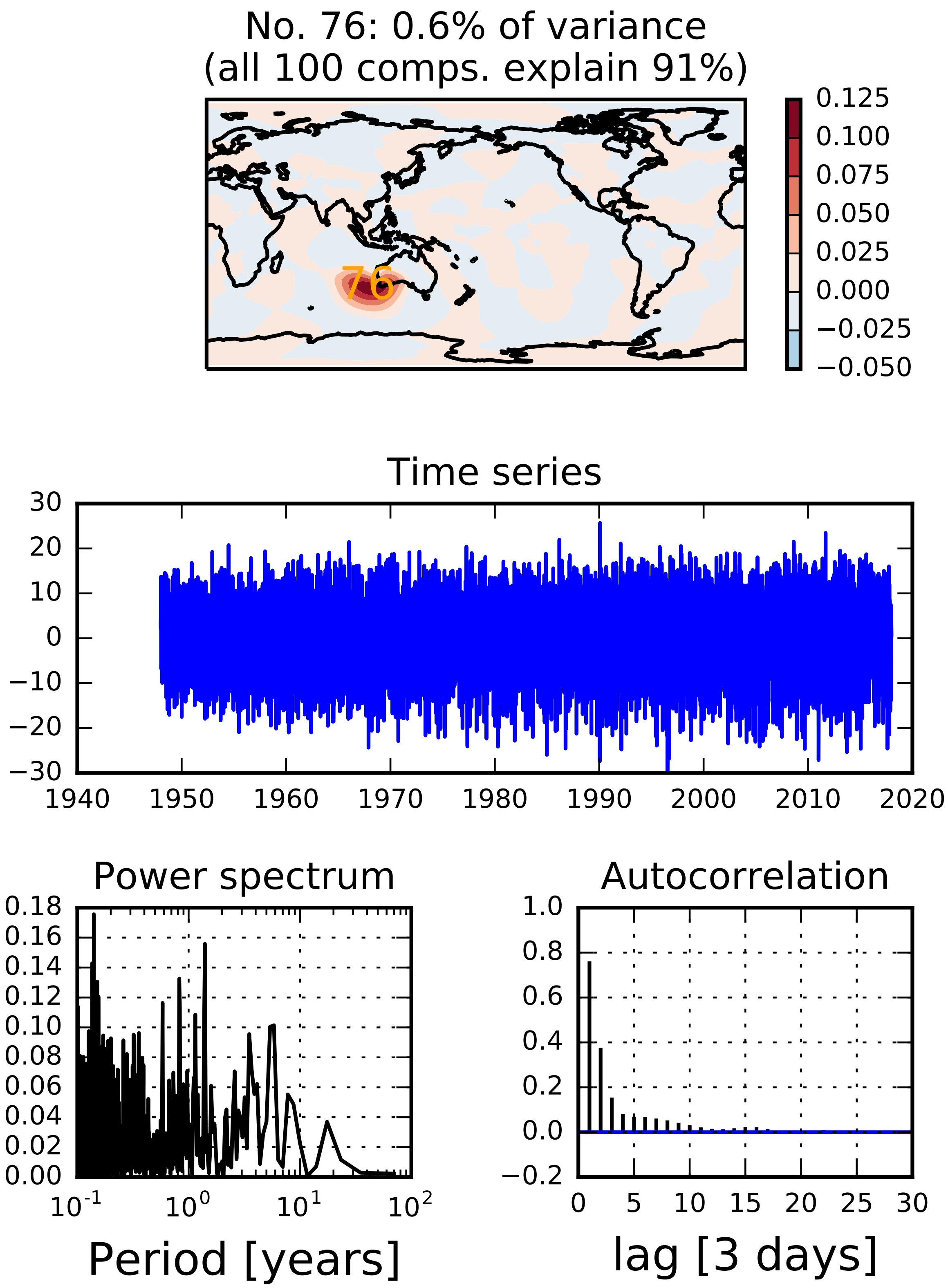
No. 76: 0.6% of variance
(all 100 comps. explain 91%)
76
0.125
0.100
0.075
0.050
0.025
0.000
−0.025
−0.050
Time series
30
20
10
0
−10
−20
−30
1940 1950 1960 1970 1980 1990 2000 2010 2020
Power spectrum
0.18 0.16 0.14 0.12 0.10 0.08 0.06 0.04 0.02 0.00
10^{-1} 10^{0} 10^{1} 10^{2}
Period [years]
Autocorrelation
1.0 0.8 0.6 0.4 0.2 0.0 −0.2
0 5 10 15 20 25 30
lag [3 days]

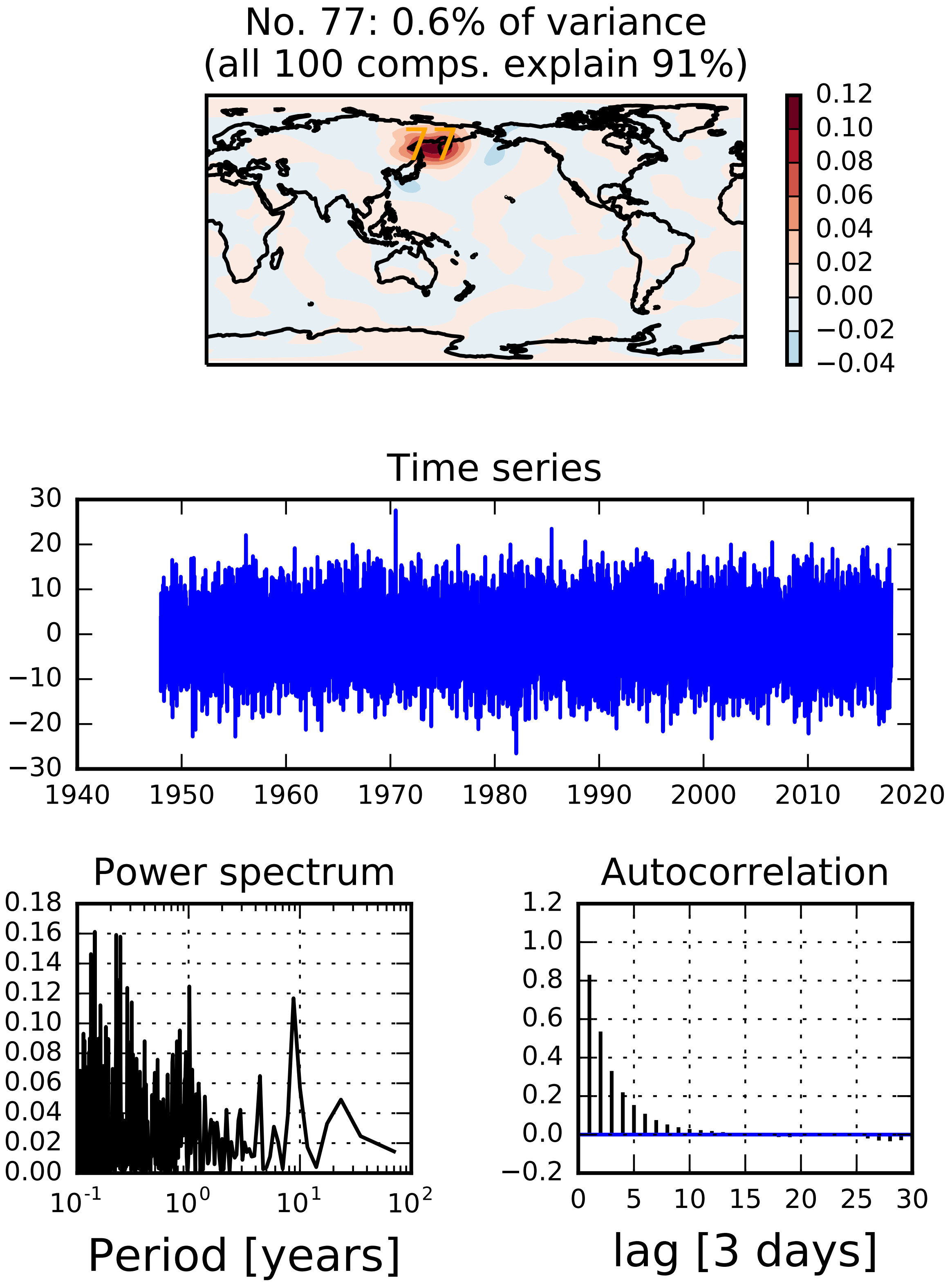
No. 77: 0.6% of variance
(all 100 comps. explain 91%)
77
0.12
0.10
0.08
0.06
0.04
0.02
0.00
−0.02
−0.04
Time series
30
20
10
0
−10
−20
−30
1940
1950
1960
1970
1980
1990
2000
2010
2020
Power spectrum
0.18
0.16
0.14
0.12
0.10
0.08
0.06
0.04
0.02
0.00
10^-1
10^0
10^1
10^2
Period [years]
Autocorrelation
1.2
1.0
0.8
0.6
0.4
0.2
0.0
−0.2
0
5
10
15
20
25
30
lag [3 days]

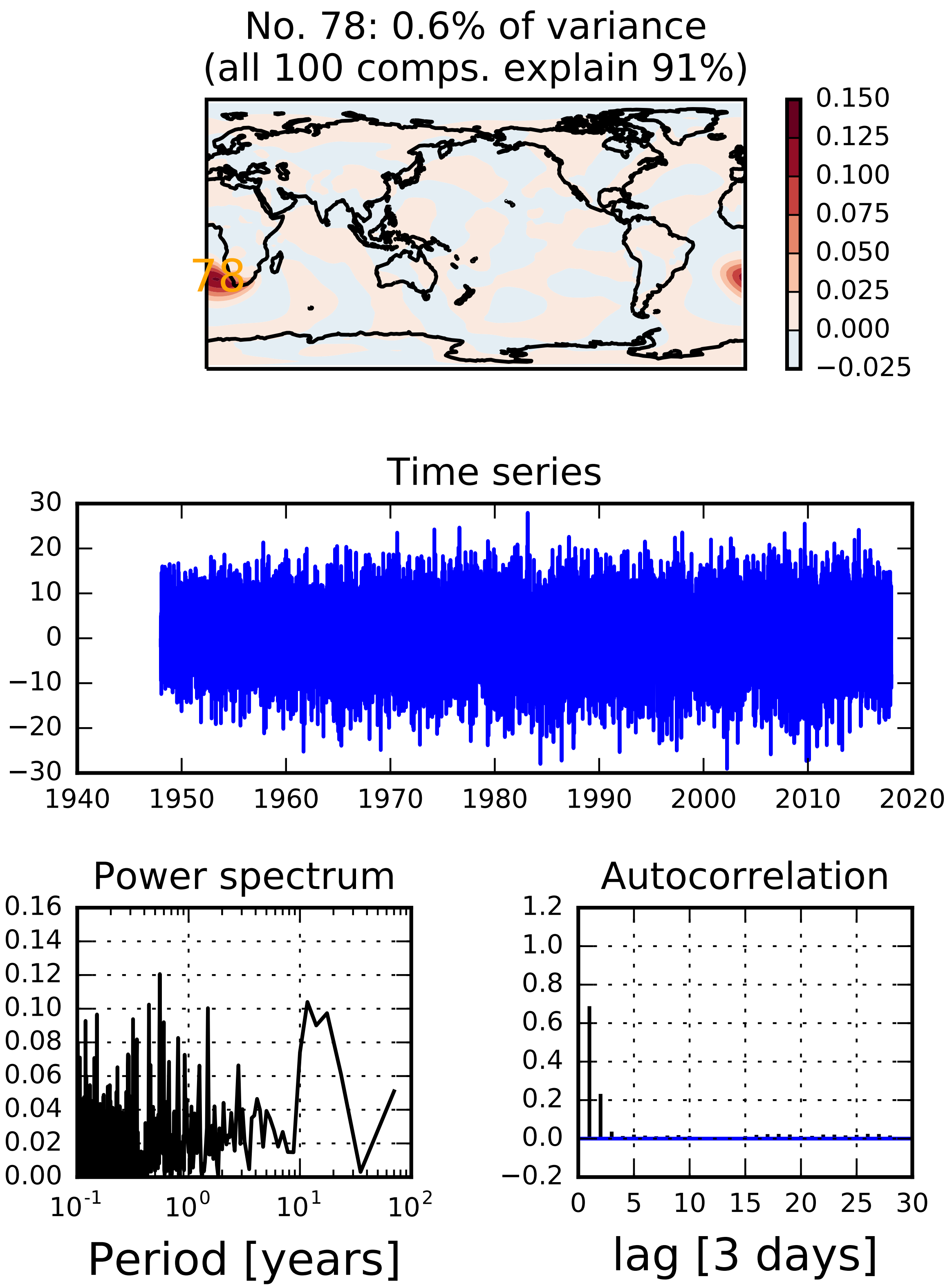
No. 78: 0.6% of variance
(all 100 comps. explain 91%)
78
0.150
0.125
0.100
0.075
0.050
0.025
0.000
−0.025
Time series
30
20
10
0
−10
−20
−30
1940
1950
1960
1970
1980
1990
2000
2010
2020
Power spectrum
0.16
0.14
0.12
0.10
0.08
0.06
0.04
0.02
0.00
10^-1
10^0
10^1
10^2
Period [years]
Autocorrelation
1.2
1.0
0.8
0.6
0.4
0.2
0.0
−0.2
0
5
10
15
20
25
30
lag [3 days]

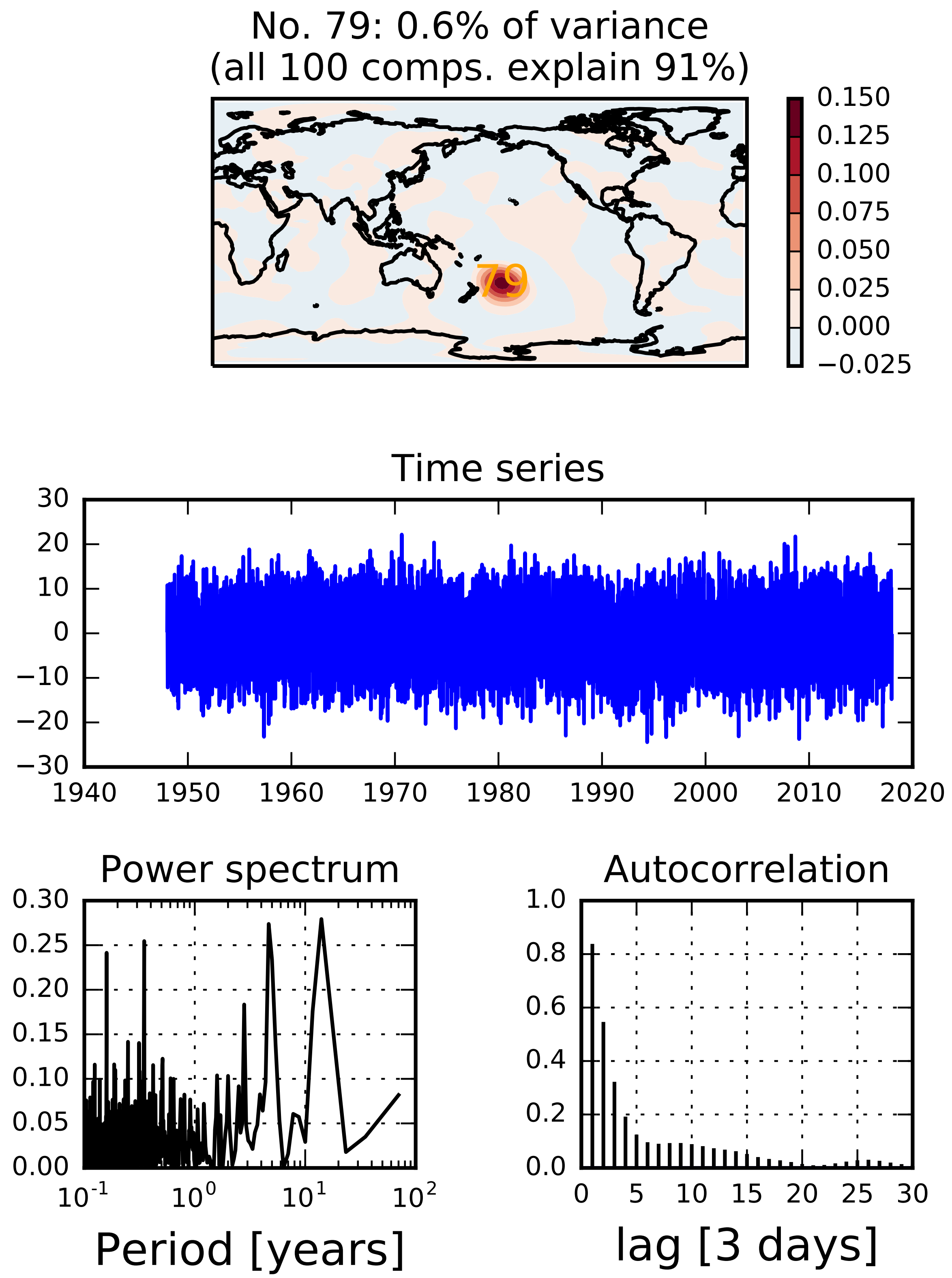
No. 79: 0.6% of variance
(all 100 comps. explain 91%)
79
0.150
0.125
0.100
0.075
0.050
0.025
0.000
−0.025
Time series
30
20
10
0
−10
−20
−30
1940
1950
1960
1970
1980
1990
2000
2010
2020
Power spectrum
0.30
0.25
0.20
0.15
0.10
0.05
0.00
10^{-1}
10^{0}
10^{1}
10^{2}
Period [years]
Autocorrelation
1.0
0.8
0.6
0.4
0.2
0.0
0
5
10
15
20
25
30
lag [3 days]

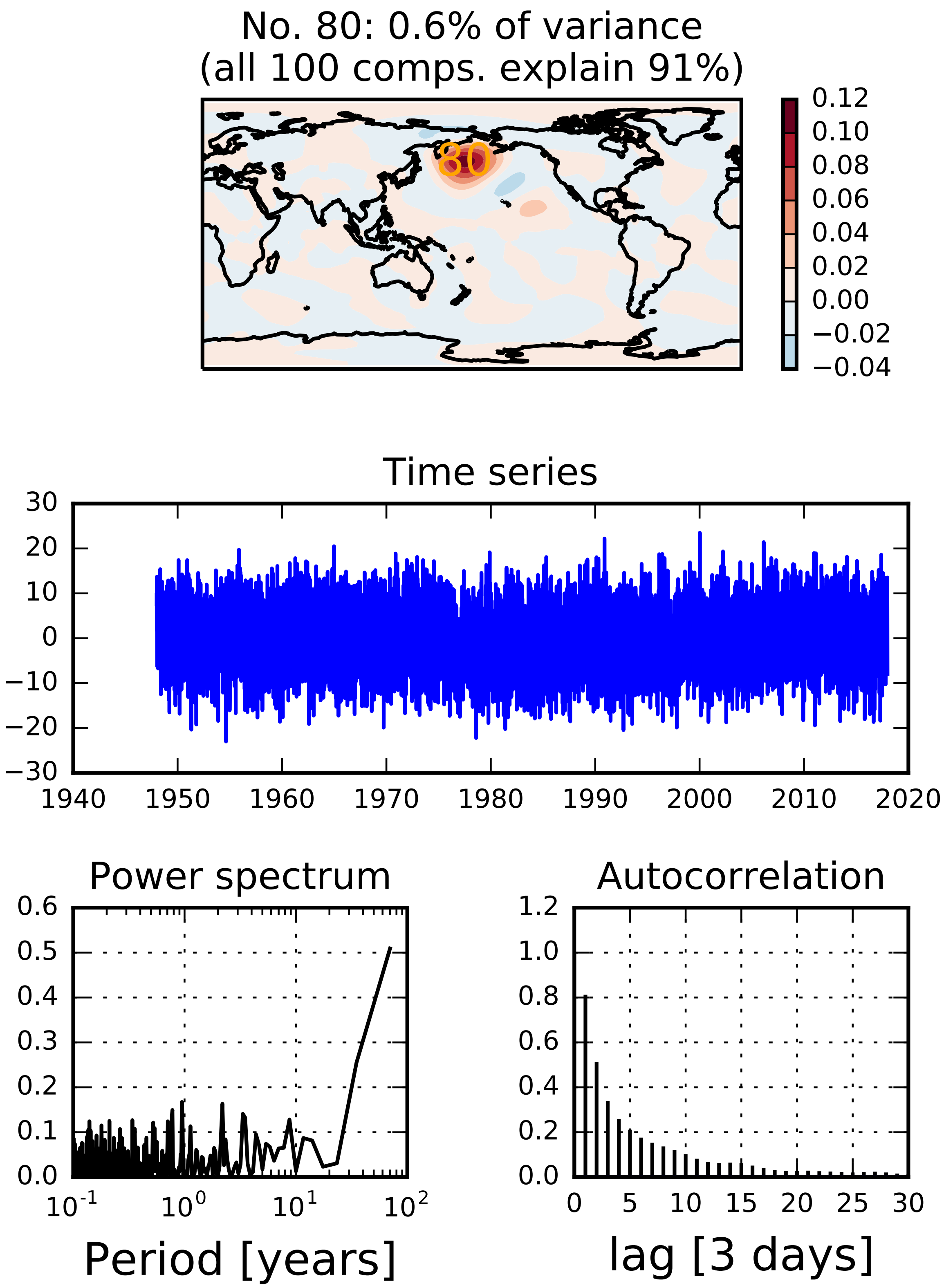
No. 80: 0.6% of variance
(all 100 comps. explain 91%)
80
0.12
0.10
0.08
0.06
0.04
0.02
0.00
−0.02
−0.04
Time series
30
20
10
0
−10
−20
−30
1940
1950
1960
1970
1980
1990
2000
2010
2020
Power spectrum
0.6
0.5
0.4
0.3
0.2
0.1
0.0
10^{-1}
10^{0}
10^{1}
10^{2}
Period [years]
Autocorrelation
1.2
1.0
0.8
0.6
0.4
0.2
0.0
0
5
10
15
20
25
30
lag [3 days]

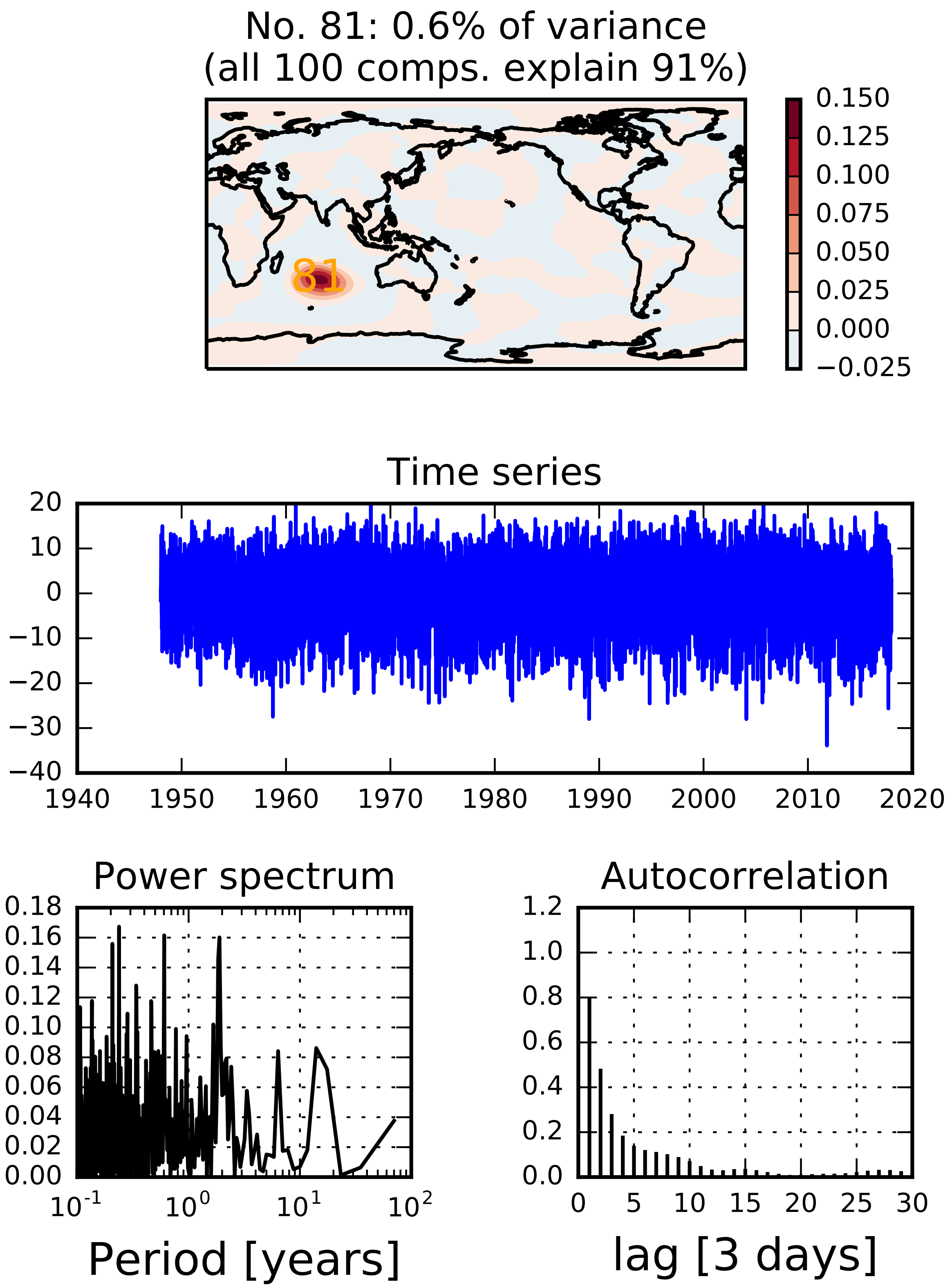
No. 81: 0.6% of variance
(all 100 comps. explain 91%)
81
0.150
0.125
0.100
0.075
0.050
0.025
0.000
−0.025
Time series
20
10
0
−10
−20
−30
−40
1940
1950
1960
1970
1980
1990
2000
2010
2020
Power spectrum
0.18
0.16
0.14
0.12
0.10
0.08
0.06
0.04
0.02
0.00
10^{-1}
10^{0}
10^{1}
10^{2}
Period [years]
Autocorrelation
1.2
1.0
0.8
0.6
0.4
0.2
0.0
0
5
10
15
20
25
30
lag [3 days]

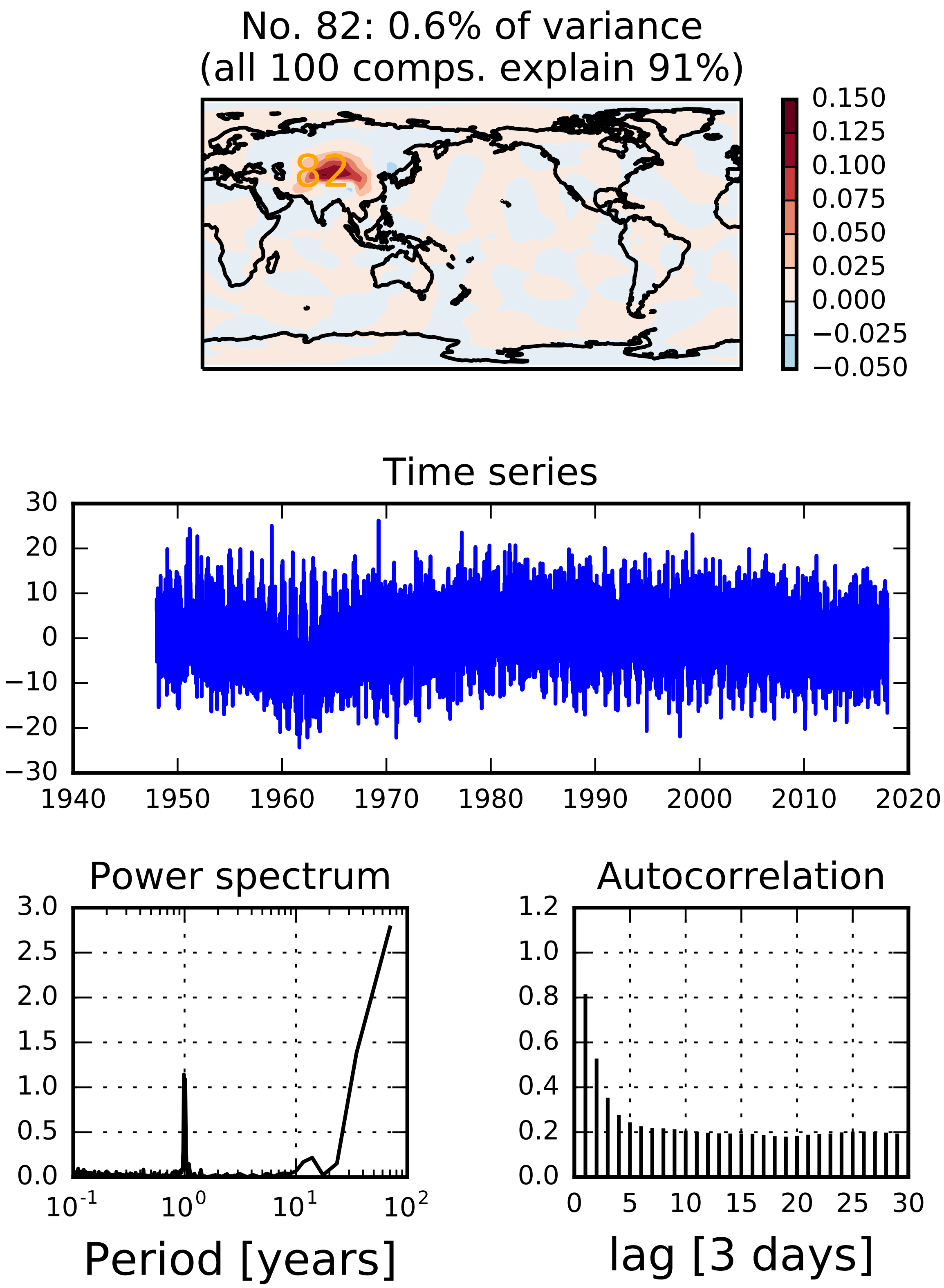
No. 82: 0.6% of variance
(all 100 comps. explain 91%)
82
0.150
0.125
0.100
0.075
0.050
0.025
0.000
−0.025
−0.050
Time series
30
20
10
0
−10
−20
−30
1940
1950
1960
1970
1980
1990
2000
2010
2020
Power spectrum
3.0
2.5
2.0
1.5
1.0
0.5
0.0
10^{-1}
10^{0}
10^{1}
10^{2}
Period [years]
Autocorrelation
1.2
1.0
0.8
0.6
0.4
0.2
0.0
0
5
10
15
20
25
30
lag [3 days]

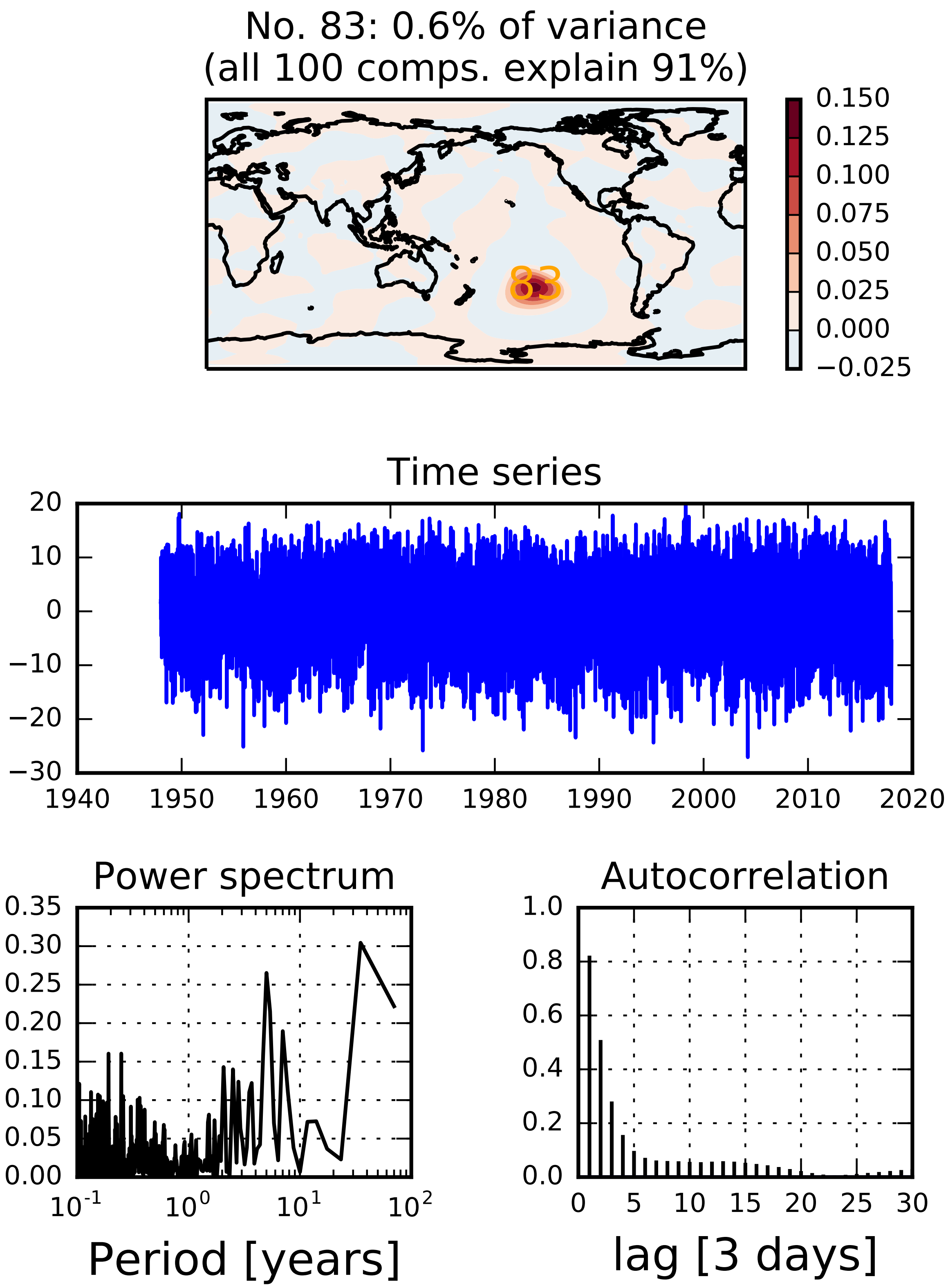
No. 83: 0.6% of variance
(all 100 comps. explain 91%)
0.150
0.125
0.100
0.075
0.050
0.025
0.000
−0.025
83
Time series
20
10
0
−10
−20
−30
1940
1950
1960
1970
1980
1990
2000
2010
2020
Power spectrum
0.35
0.30
0.25
0.20
0.15
0.10
0.05
0.00
Period [years]
Autocorrelation
1.0
0.8
0.6
0.4
0.2
0.0
0
5
10
15
20
25
30
lag [3 days]

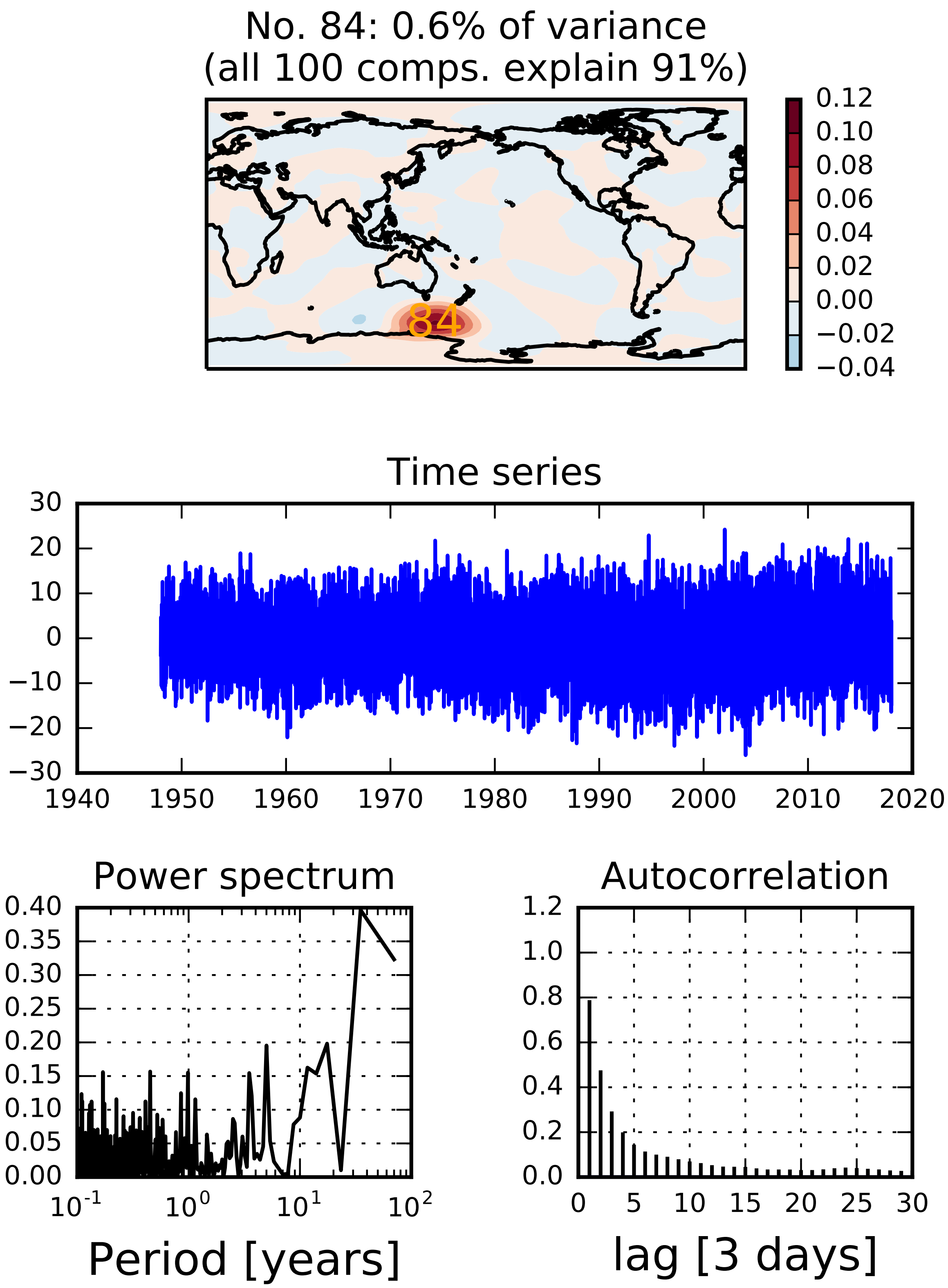
No. 84: 0.6% of variance
(all 100 comps. explain 91%)
84
0.12
0.10
0.08
0.06
0.04
0.02
0.00
−0.02
−0.04
Time series
30
20
10
0
−10
−20
−30
1940
1950
1960
1970
1980
1990
2000
2010
2020
Power spectrum
0.40
0.35
0.30
0.25
0.20
0.15
0.10
0.05
0.00
10^{-1}
10^{0}
10^{1}
10^{2}
Period [years]
Autocorrelation
1.2
1.0
0.8
0.6
0.4
0.2
0.0
0
5
10
15
20
25
30
lag [3 days]

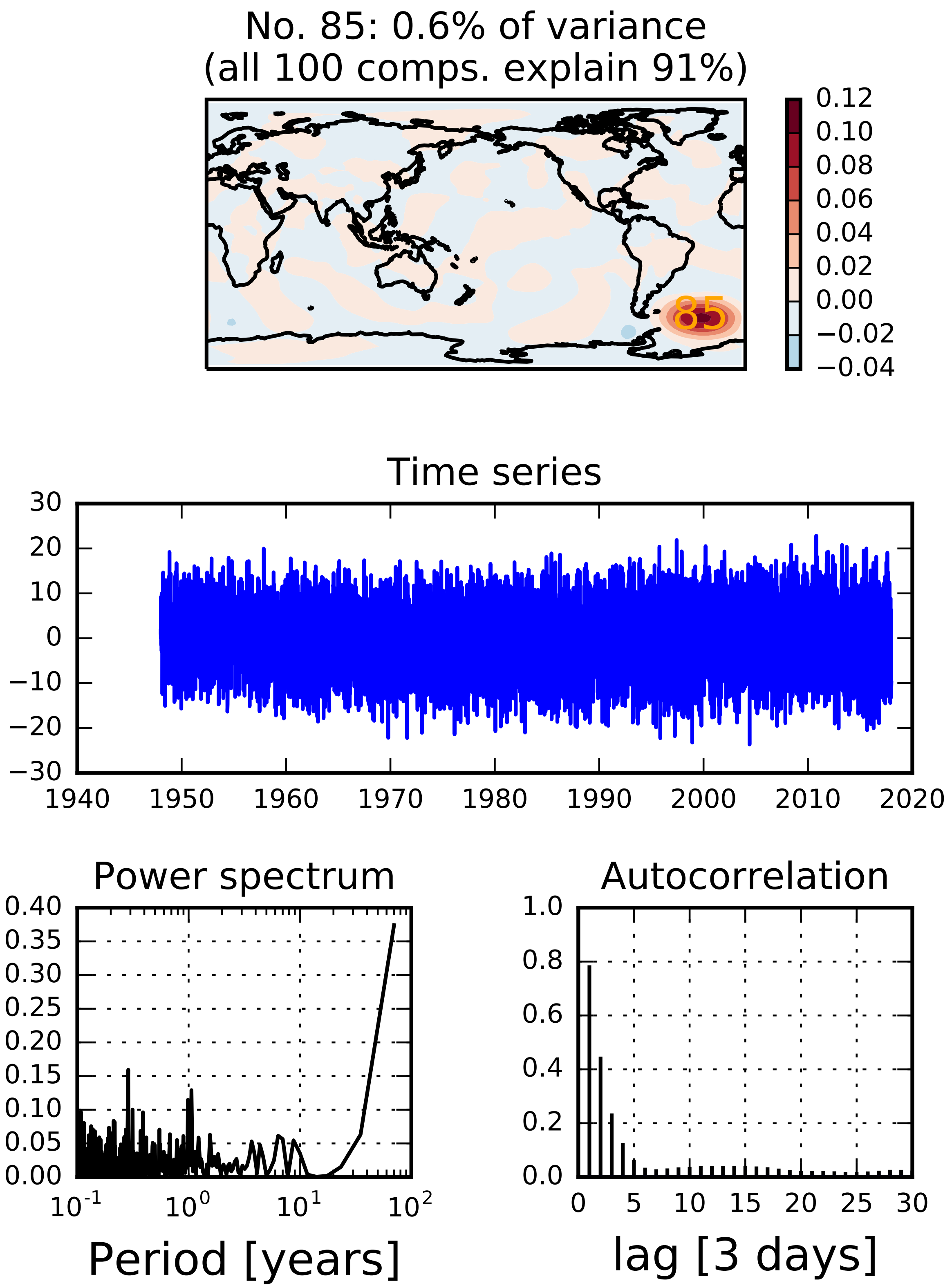
No. 85: 0.6% of variance
(all 100 comps. explain 91%)
85
0.12
0.10
0.08
0.06
0.04
0.02
0.00
−0.02
−0.04
Time series
30
20
10
0
−10
−20
−30
1940
1950
1960
1970
1980
1990
2000
2010
2020
Power spectrum
0.40
0.35
0.30
0.25
0.20
0.15
0.10
0.05
0.00
10^-1
10^0
10^1
10^2
Period [years]
Autocorrelation
1.0
0.8
0.6
0.4
0.2
0.0
0
5
10
15
20
25
30
lag [3 days]

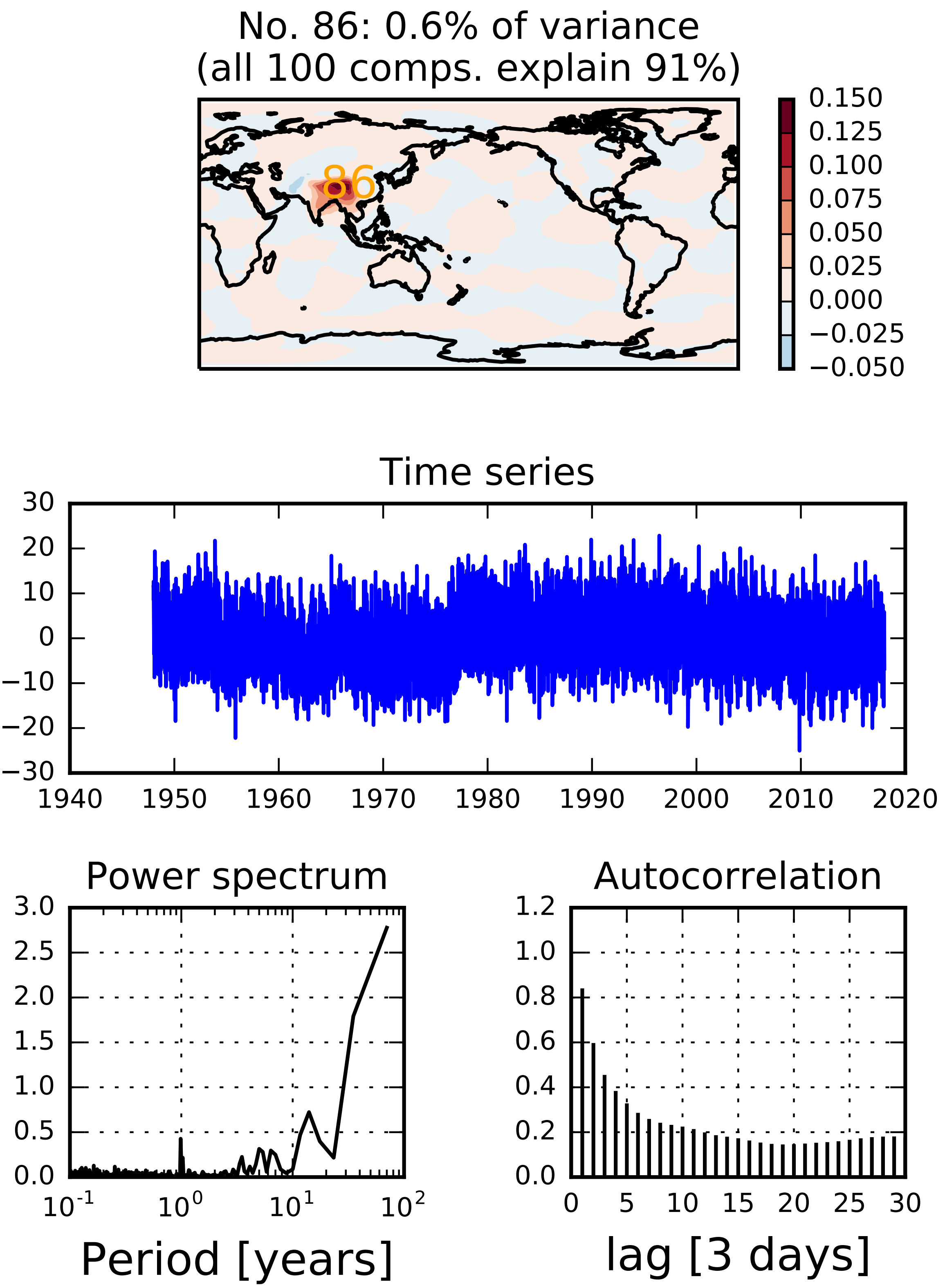
No. 86: 0.6% of variance
(all 100 comps. explain 91%)
86
0.150
0.125
0.100
0.075
0.050
0.025
0.000
−0.025
−0.050
Time series
30
20
10
0
−10
−20
−30
1940
1950
1960
1970
1980
1990
2000
2010
2020
Power spectrum
3.0
2.5
2.0
1.5
1.0
0.5
0.0
10^{-1}
10^{0}
10^{1}
10^{2}
Period [years]
Autocorrelation
1.2
1.0
0.8
0.6
0.4
0.2
0.0
0
5
10
15
20
25
30
lag [3 days]

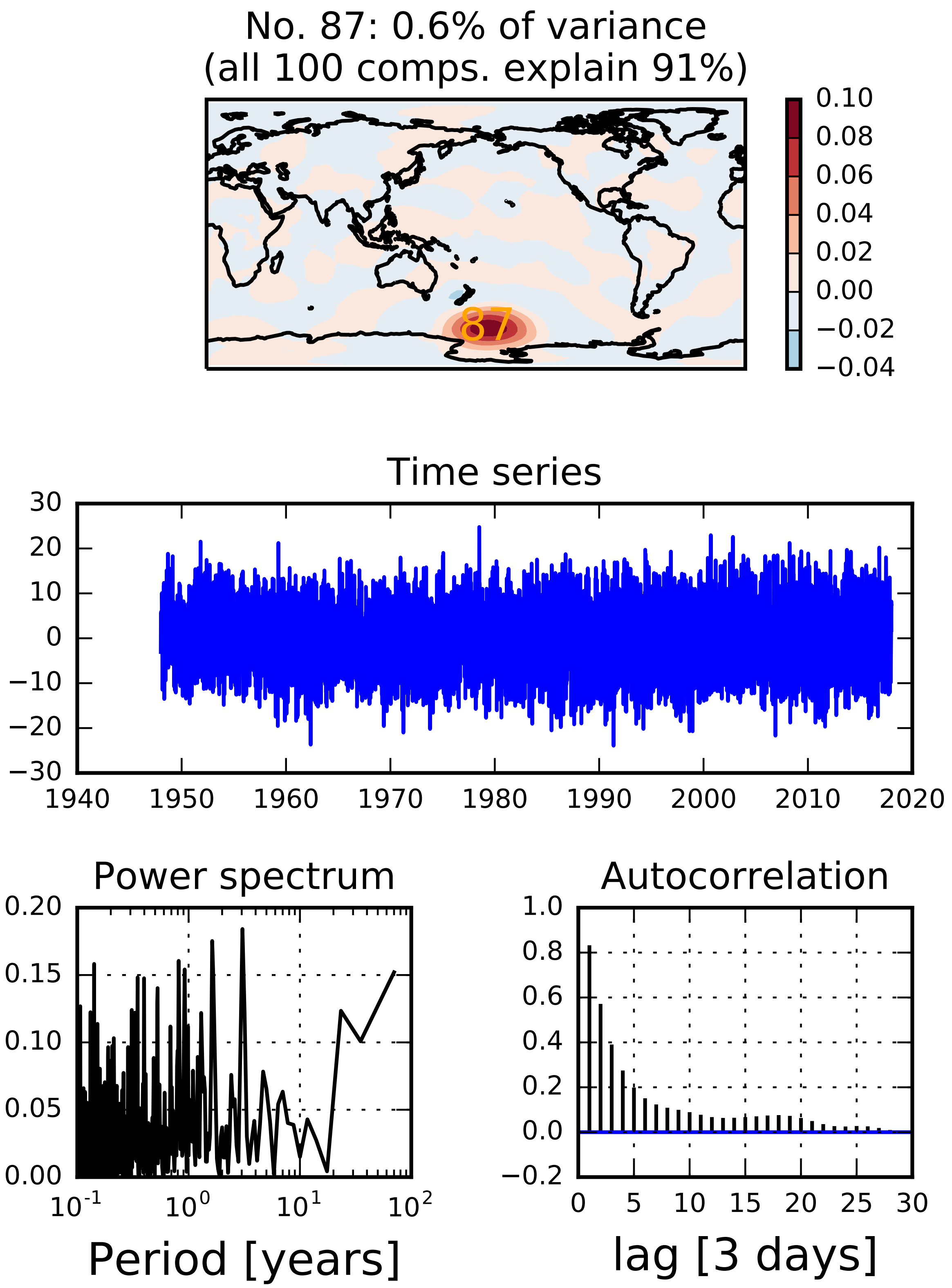
No. 87: 0.6% of variance
(all 100 comps. explain 91%)
87
0.10
0.08
0.06
0.04
0.02
0.00
−0.02
−0.04
Time series
30
20
10
0
−10
−20
−30
1940
1950
1960
1970
1980
1990
2000
2010
2020
Power spectrum
0.20
0.15
0.10
0.05
0.00
10^-1
10^0
10^1
10^2
Period [years]
Autocorrelation
1.0
0.8
0.6
0.4
0.2
0.0
−0.2
0
5
10
15
20
25
30
lag [3 days]

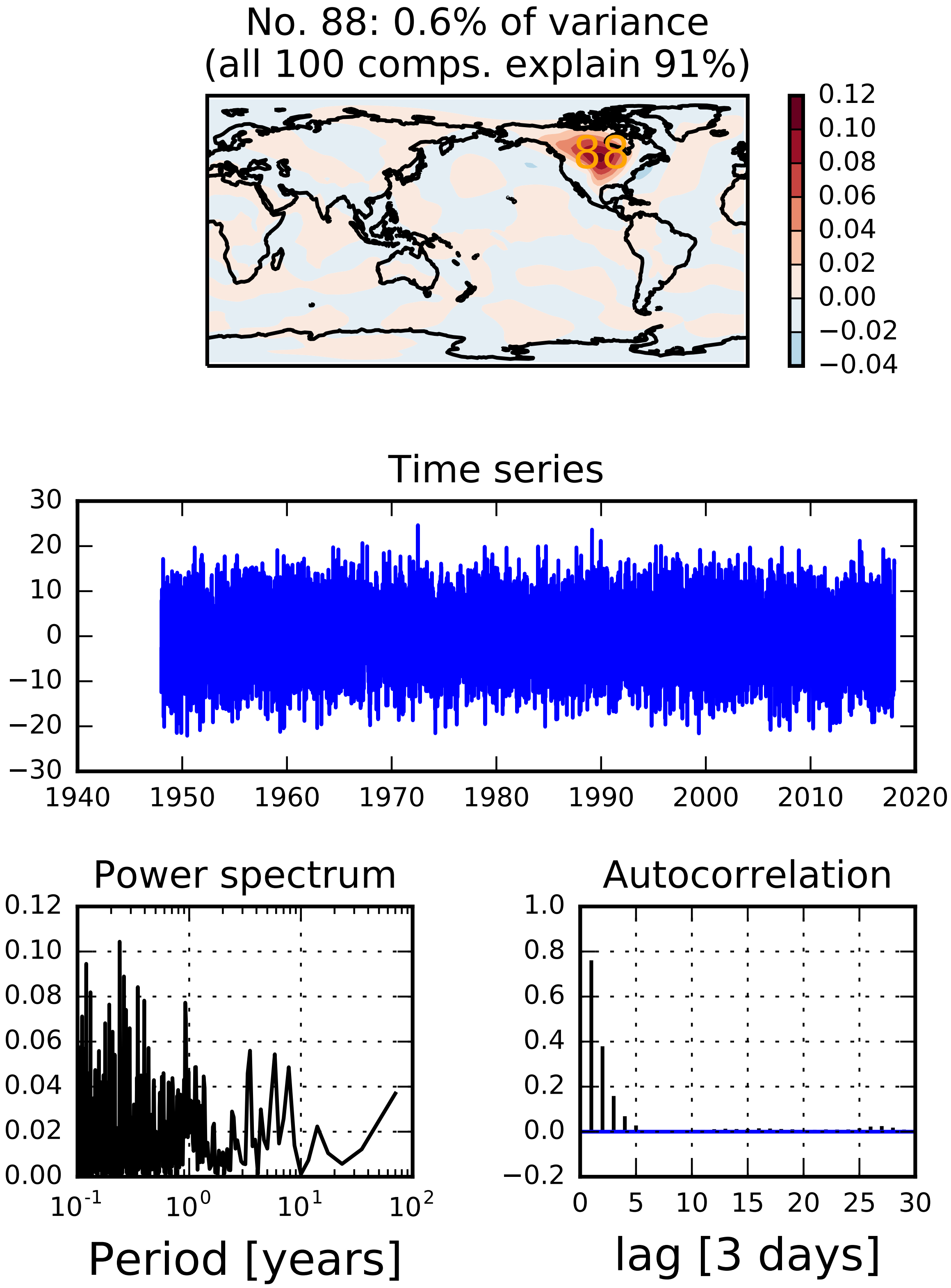
No. 88: 0.6% of variance
(all 100 comps. explain 91%)
88
0.12
0.10
0.08
0.06
0.04
0.02
0.00
−0.02
−0.04
Time series
30
20
10
0
−10
−20
−30
1940
1950
1960
1970
1980
1990
2000
2010
2020
Power spectrum
0.12
0.10
0.08
0.06
0.04
0.02
0.00
10^-1
10^0
10^1
10^2
Period [years]
Autocorrelation
1.0
0.8
0.6
0.4
0.2
0.0
−0.2
0
5
10
15
20
25
30
lag [3 days]

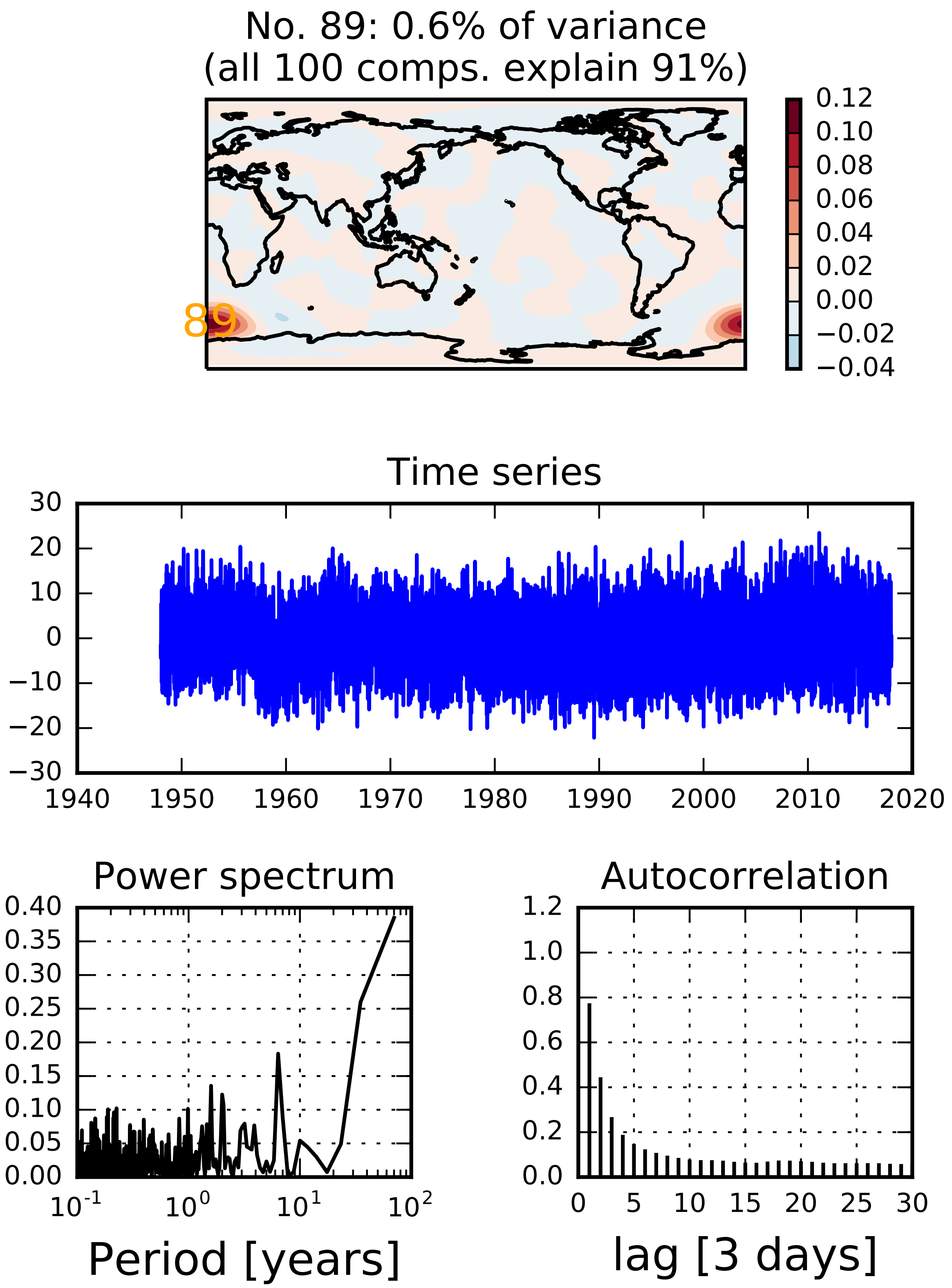
No. 89: 0.6% of variance
(all 100 comps. explain 91%)
89
0.12
0.10
0.08
0.06
0.04
0.02
0.00
−0.02
−0.04
Time series
30
20
10
0
−10
−20
−30
1940
1950
1960
1970
1980
1990
2000
2010
2020
Power spectrum
0.40
0.35
0.30
0.25
0.20
0.15
0.10
0.05
0.00
10^{-1}
10^{0}
10^{1}
10^{2}
Period [years]
Autocorrelation
1.2
1.0
0.8
0.6
0.4
0.2
0.0
0
5
10
15
20
25
30
lag [3 days]

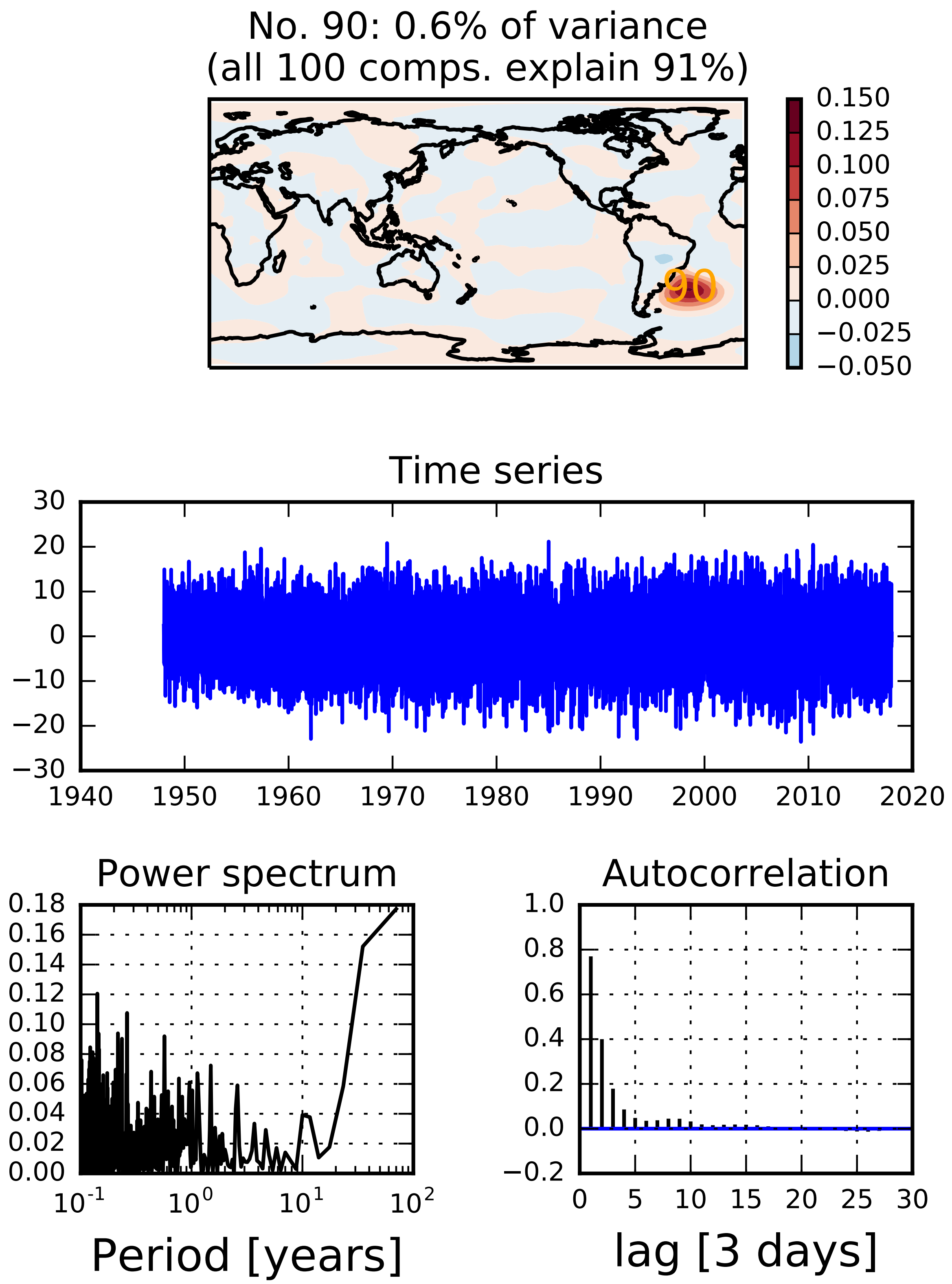
No. 90: 0.6% of variance
(all 100 comps. explain 91%)
90
0.150
0.125
0.100
0.075
0.050
0.025
0.000
−0.025
−0.050
Time series
30
20
10
0
−10
−20
−30
1940
1950
1960
1970
1980
1990
2000
2010
2020
Power spectrum
0.18
0.16
0.14
0.12
0.10
0.08
0.06
0.04
0.02
0.00
10^{-1}
10^{0}
10^{1}
10^{2}
Period [years]
Autocorrelation
1.0
0.8
0.6
0.4
0.2
0.0
−0.2
0
5
10
15
20
25
30
lag [3 days]

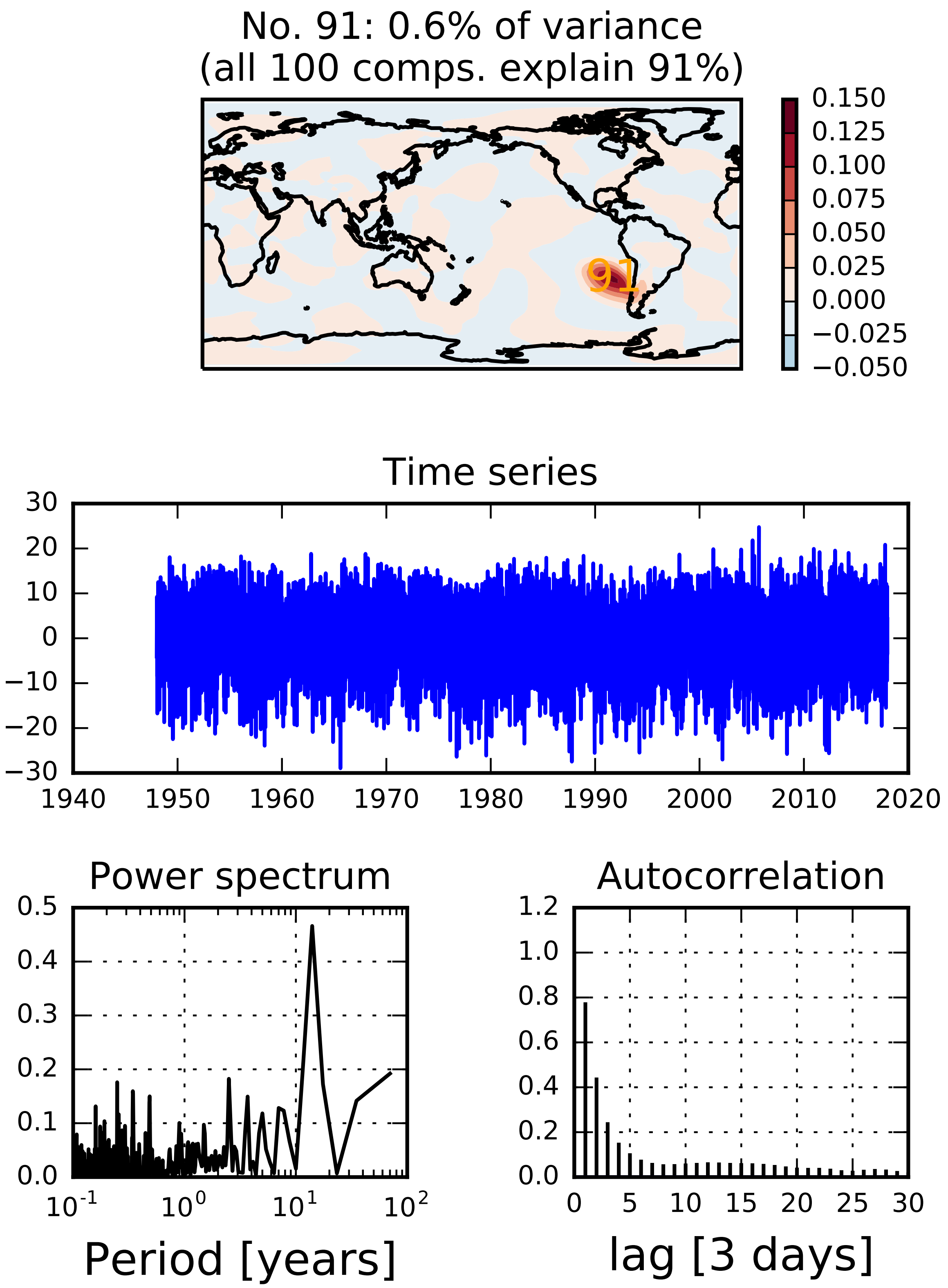
No. 91: 0.6% of variance
(all 100 comps. explain 91%)
0.150
0.125
0.100
0.075
0.050
0.025
0.000
−0.025
−0.050
91
Time series
30
20
10
0
−10
−20
−30
1940
1950
1960
1970
1980
1990
2000
2010
2020
Power spectrum
0.5
0.4
0.3
0.2
0.1
0.0
10^-1
10^0
10^1
10^2
Period [years]
Autocorrelation
1.2
1.0
0.8
0.6
0.4
0.2
0.0
0
5
10
15
20
25
30
lag [3 days]

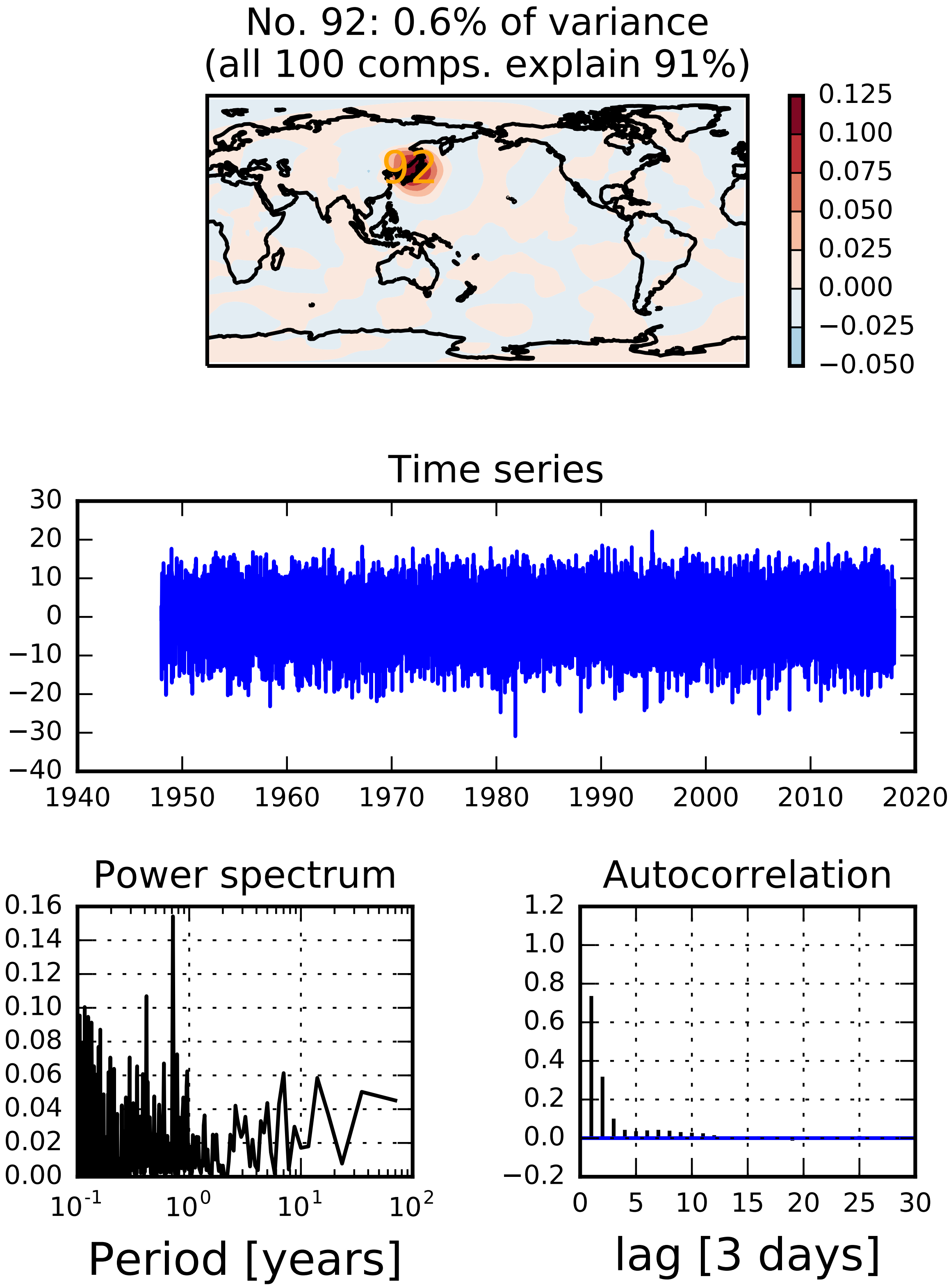
No. 92: 0.6% of variance
(all 100 comps. explain 91%)
92
0.125
0.100
0.075
0.050
0.025
0.000
−0.025
−0.050
Time series
30
20
10
0
−10
−20
−30
−40
1940
1950
1960
1970
1980
1990
2000
2010
2020
Power spectrum
0.16
0.14
0.12
0.10
0.08
0.06
0.04
0.02
0.00
10^{-1}
10^{0}
10^{1}
10^{2}
Period [years]
Autocorrelation
1.2
1.0
0.8
0.6
0.4
0.2
0.0
−0.2
0
5
10
15
20
25
30
lag [3 days]

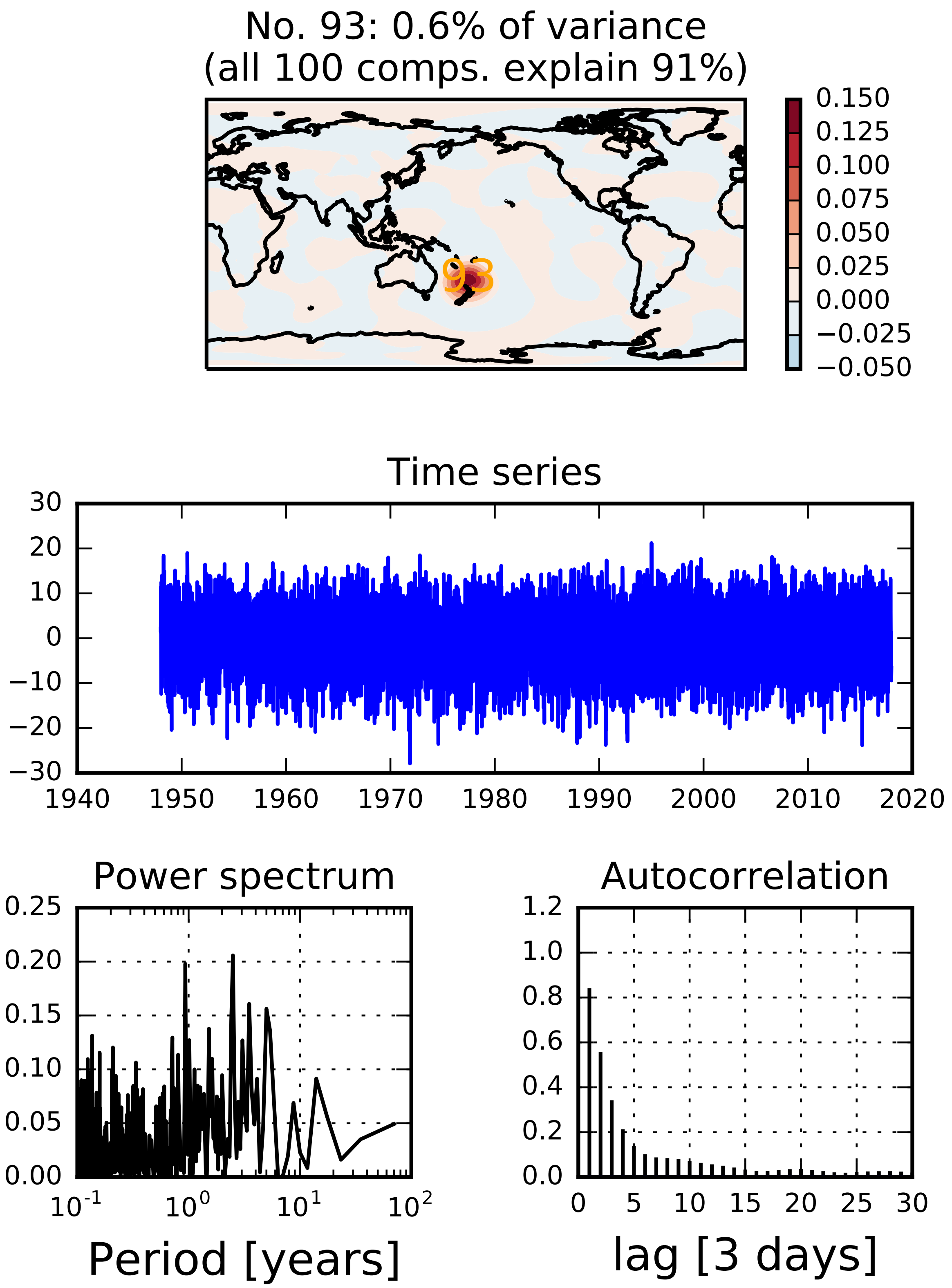
No. 93: 0.6% of variance
(all 100 comps. explain 91%)
93
0.150
0.125
0.100
0.075
0.050
0.025
0.000
−0.025
−0.050
Time series
30
20
10
0
−10
−20
−30
1940
1950
1960
1970
1980
1990
2000
2010
2020
Power spectrum
0.25
0.20
0.15
0.10
0.05
0.00
10^-1
10^0
10^1
10^2
Period [years]
Autocorrelation
1.2
1.0
0.8
0.6
0.4
0.2
0.0
0
5
10
15
20
25
30
lag [3 days]

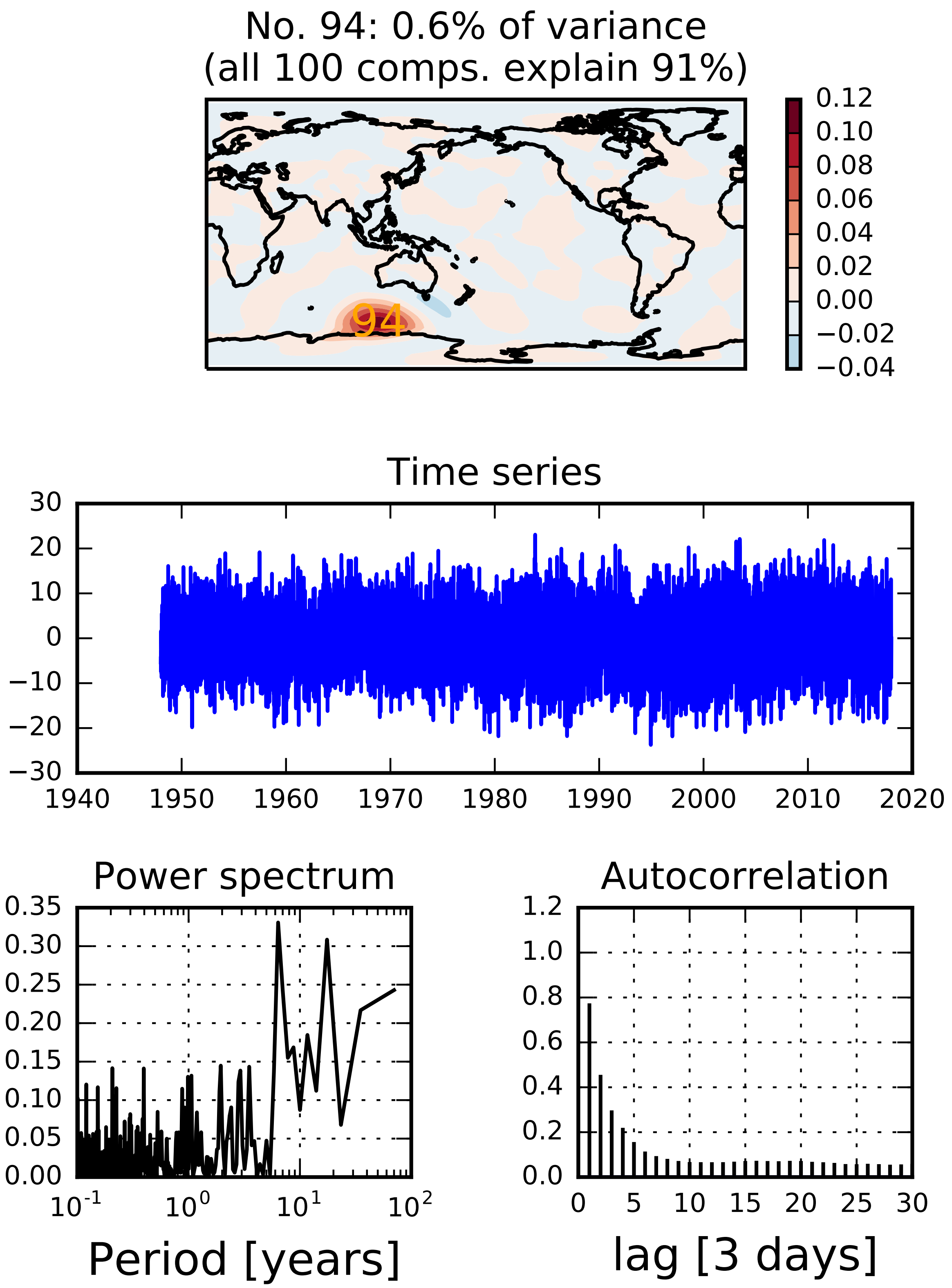
No. 94: 0.6% of variance
(all 100 comps. explain 91%)
94
0.12
0.10
0.08
0.06
0.04
0.02
0.00
−0.02
−0.04
Time series
30
20
10
0
−10
−20
−30
1940
1950
1960
1970
1980
1990
2000
2010
2020
Power spectrum
0.35
0.30
0.25
0.20
0.15
0.10
0.05
0.00
10^-1
10^0
10^1
10^2
Period [years]
Autocorrelation
1.2
1.0
0.8
0.6
0.4
0.2
0.0
0
5
10
15
20
25
30
lag [3 days]

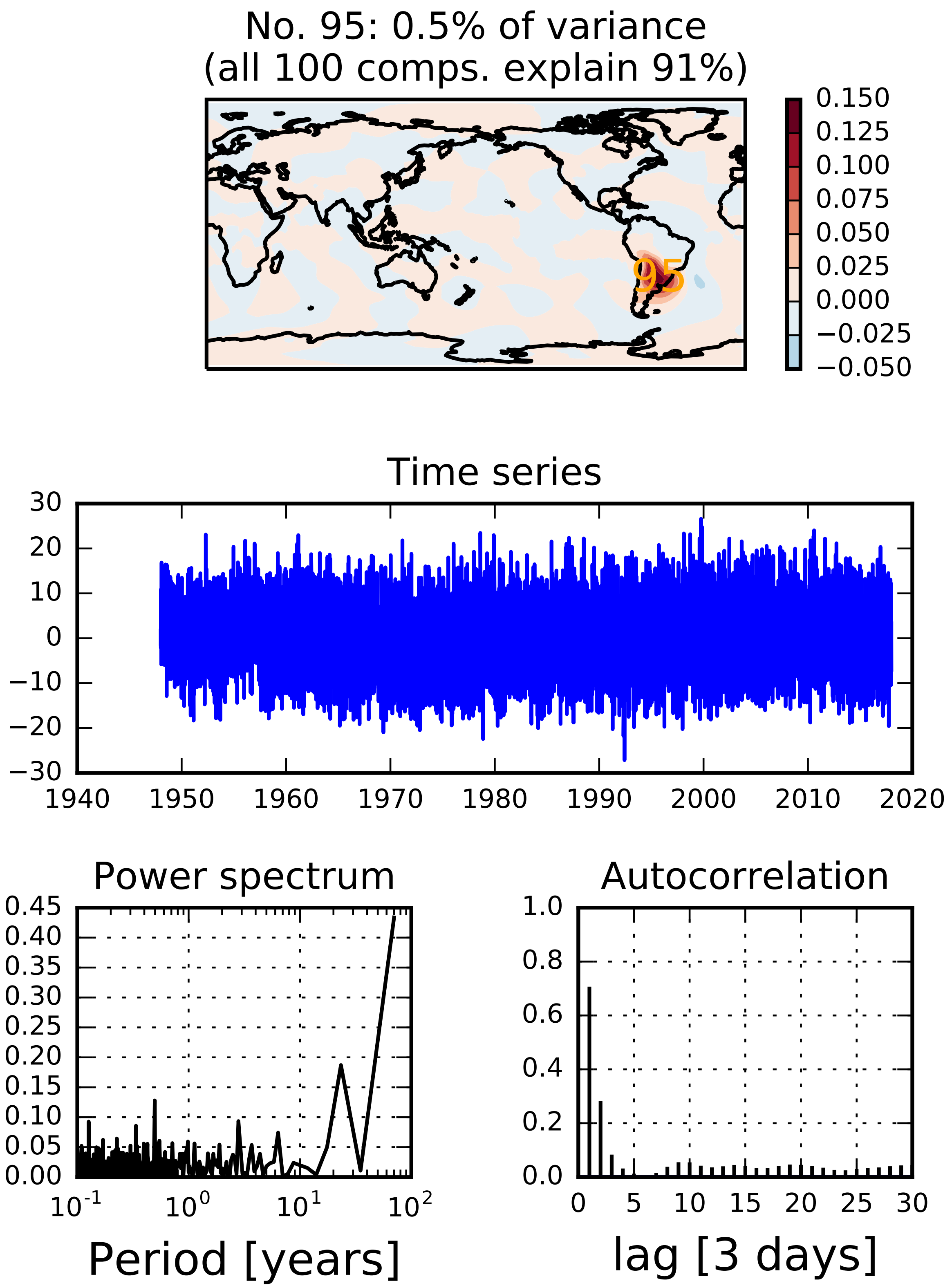
No. 95: 0.5% of variance
(all 100 comps. explain 91%)
0.150
0.125
0.100
0.075
0.050
0.025
0.000
−0.025
−0.050
95
Time series
30
20
10
0
−10
−20
−30
1940
1950
1960
1970
1980
1990
2000
2010
2020
Power spectrum
0.45
0.40
0.35
0.30
0.25
0.20
0.15
0.10
0.05
0.00
10^{-1}
10^{0}
10^{1}
10^{2}
Period [years]
Autocorrelation
1.0
0.8
0.6
0.4
0.2
0.0
0
5
10
15
20
25
30
lag [3 days]

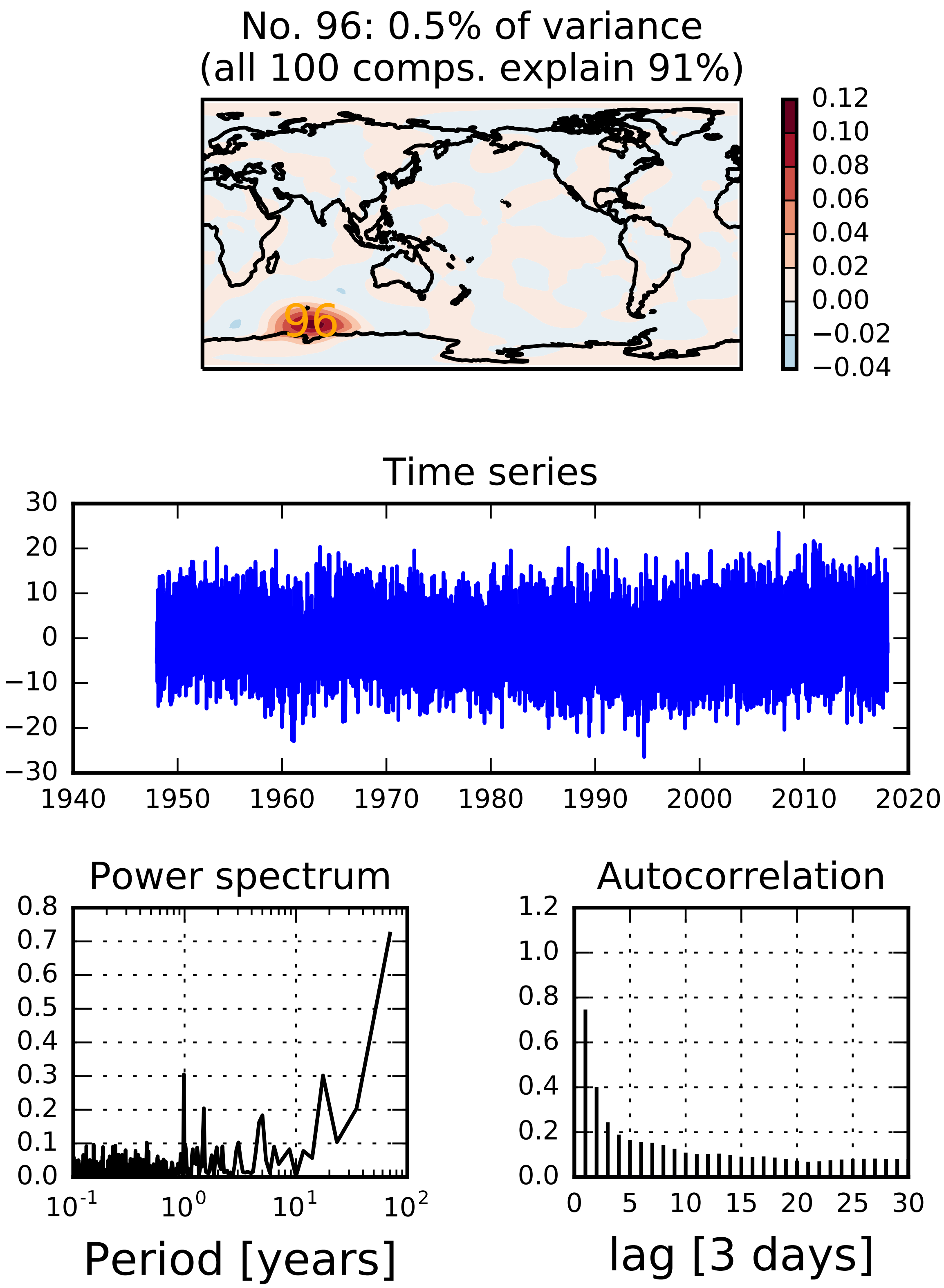
No. 96: 0.5% of variance
(all 100 comps. explain 91%)
96
0.12
0.10
0.08
0.06
0.04
0.02
0.00
−0.02
−0.04
Time series
30
20
10
0
−10
−20
−30
1940
1950
1960
1970
1980
1990
2000
2010
2020
Power spectrum
0.8
0.7
0.6
0.5
0.4
0.3
0.2
0.1
0.0
10^-1
10^0
10^1
10^2
Period [years]
Autocorrelation
1.2
1.0
0.8
0.6
0.4
0.2
0.0
0
5
10
15
20
25
30
lag [3 days]

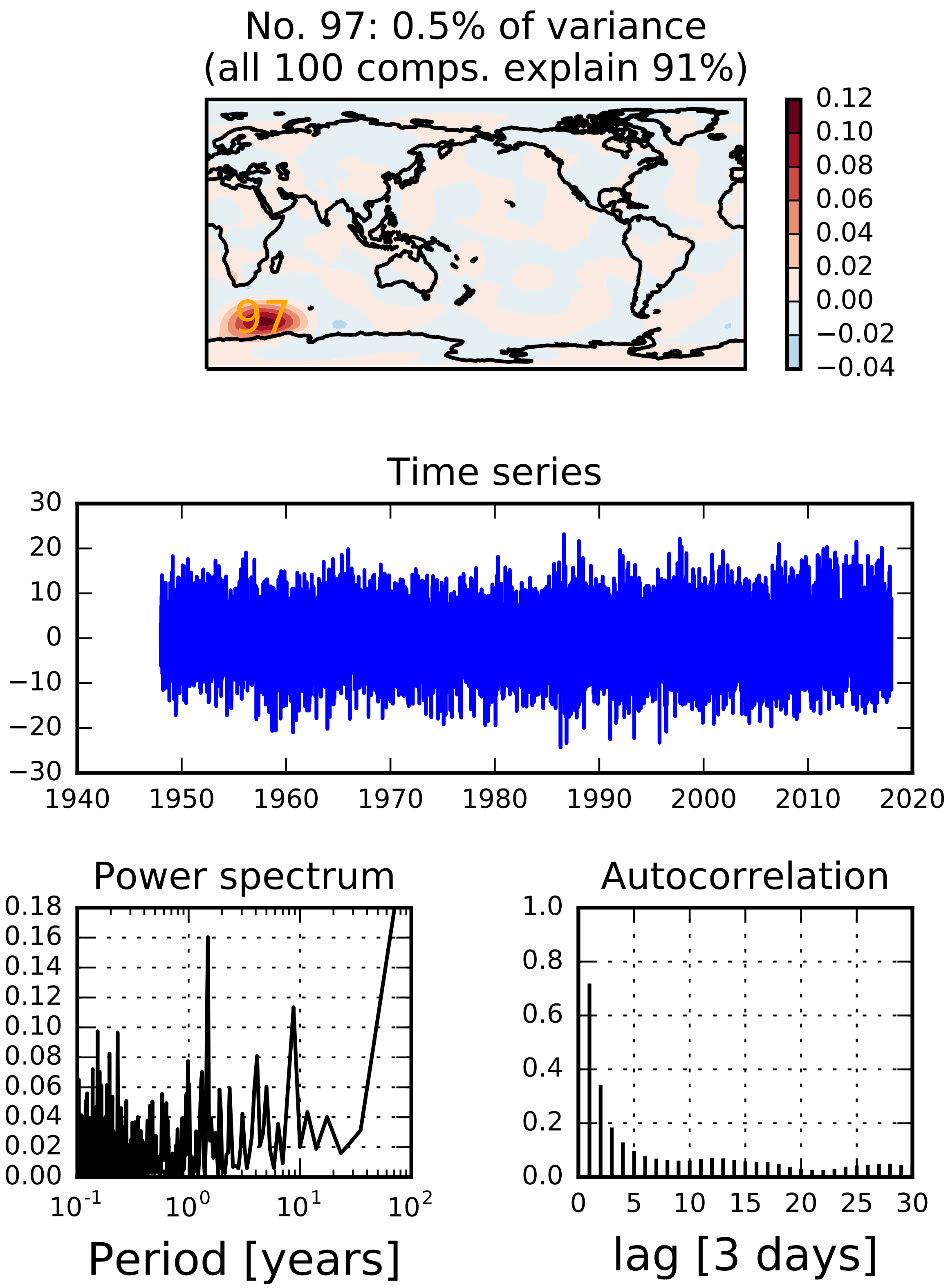
No. 97: 0.5% of variance
(all 100 comps. explain 91%)
97
0.12
0.10
0.08
0.06
0.04
0.02
0.00
−0.02
−0.04
Time series
30
20
10
0
−10
−20
−30
1940
1950
1960
1970
1980
1990
2000
2010
2020
Power spectrum
0.18
0.16
0.14
0.12
0.10
0.08
0.06
0.04
0.02
0.00
10^{-1}
10^{0}
10^{1}
10^{2}
Period [years]
Autocorrelation
1.0
0.8
0.6
0.4
0.2
0.0
0
5
10
15
20
25
30
lag [3 days]

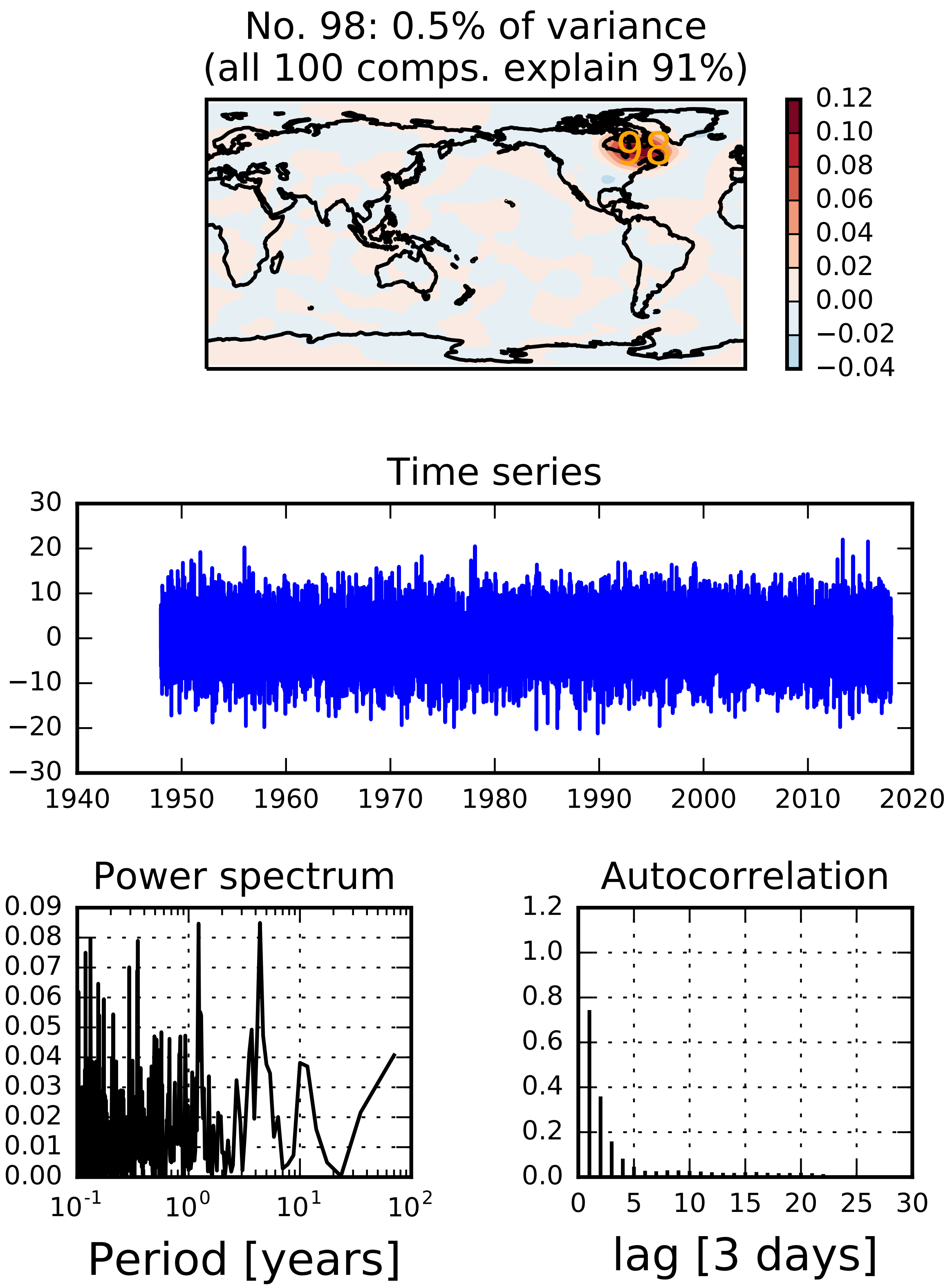
No. 98: 0.5% of variance
(all 100 comps. explain 91%)
98
0.12
0.10
0.08
0.06
0.04
0.02
0.00
−0.02
−0.04
Time series
30
20
10
0
−10
−20
−30
1940
1950
1960
1970
1980
1990
2000
2010
2020
Power spectrum
0.09
0.08
0.07
0.06
0.05
0.04
0.03
0.02
0.01
0.00
10^{-1}
10^{0}
10^{1}
10^{2}
Period [years]
Autocorrelation
1.2
1.0
0.8
0.6
0.4
0.2
0.0
0
5
10
15
20
25
30
lag [3 days]

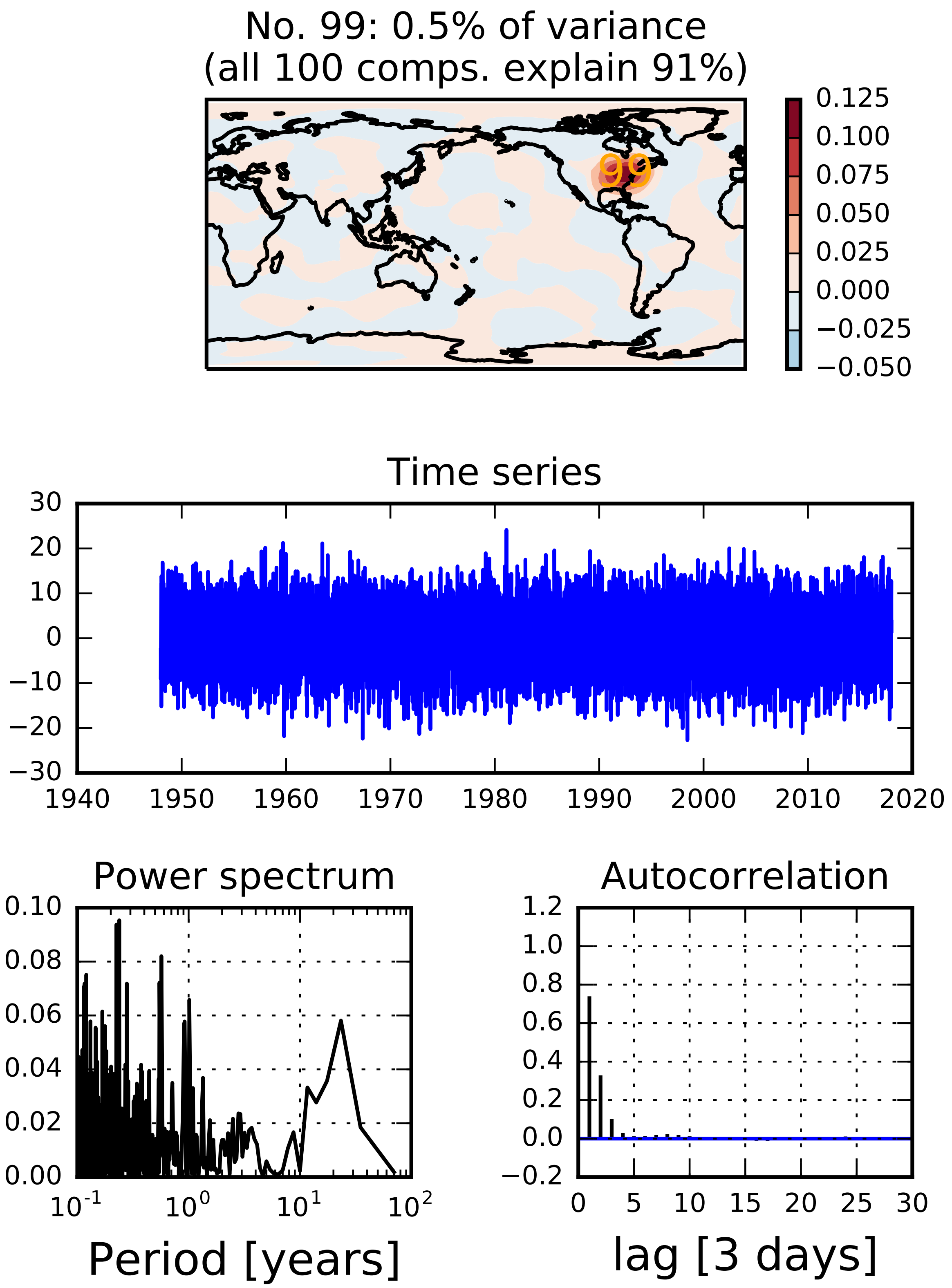

No. 99: 0.5% of variance
(all 100 comps. explain 91%)
99
0.125
0.100
0.075
0.050
0.025
0.000
−0.025
−0.050
Time series
30
20
10
0
−10
−20
−30
1940
1950
1960
1970
1980
1990
2000
2010
2020
Power spectrum
0.10
0.08
0.06
0.04
0.02
0.00
10^{-1}
10^{0}
10^{1}
10^{2}
Period [years]
Autocorrelation
1.2
1.0
0.8
0.6
0.4
0.2
0.0
−0.2
0
5
10
15
20
25
30
lag [3 days]

Supplementary Figures 122 to 221

MAM PCA Varimax components. For each component, we provide the component index and the component's spatial loadings (top subfigure), the component time series (centre) as well as the component's power spectrum (bottom left) and autocorrelation properties (bottom right). For each component we further provide the fraction of explained variance as defined prior to the Varimax rotation.

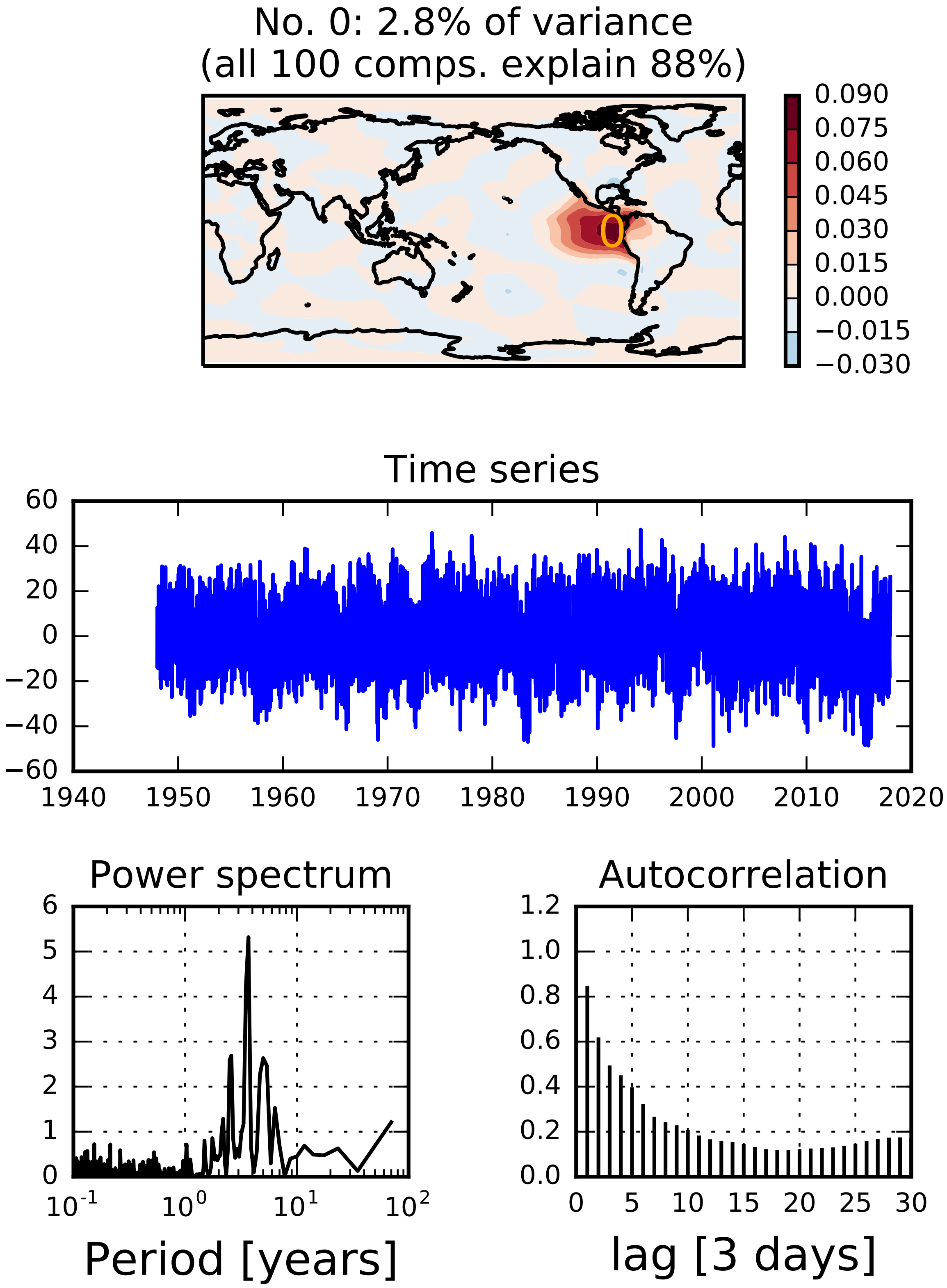

No. 0: 2.8% of variance
(all 100 comps. explain 88%)
0
0.090
0.075
0.060
0.045
0.030
0.015
0.000
−0.015
−0.030
Time series
60
40
20
0
−20
−40
−60
1940
1950
1960
1970
1980
1990
2000
2010
2020
Power spectrum
6
5
4
3
2
1
0
10^{-1}
10^{0}
10^{1}
10^{2}
Period [years]
Autocorrelation
1.2
1.0
0.8
0.6
0.4
0.2
0.0
0
5
10
15
20
25
30
lag [3 days]

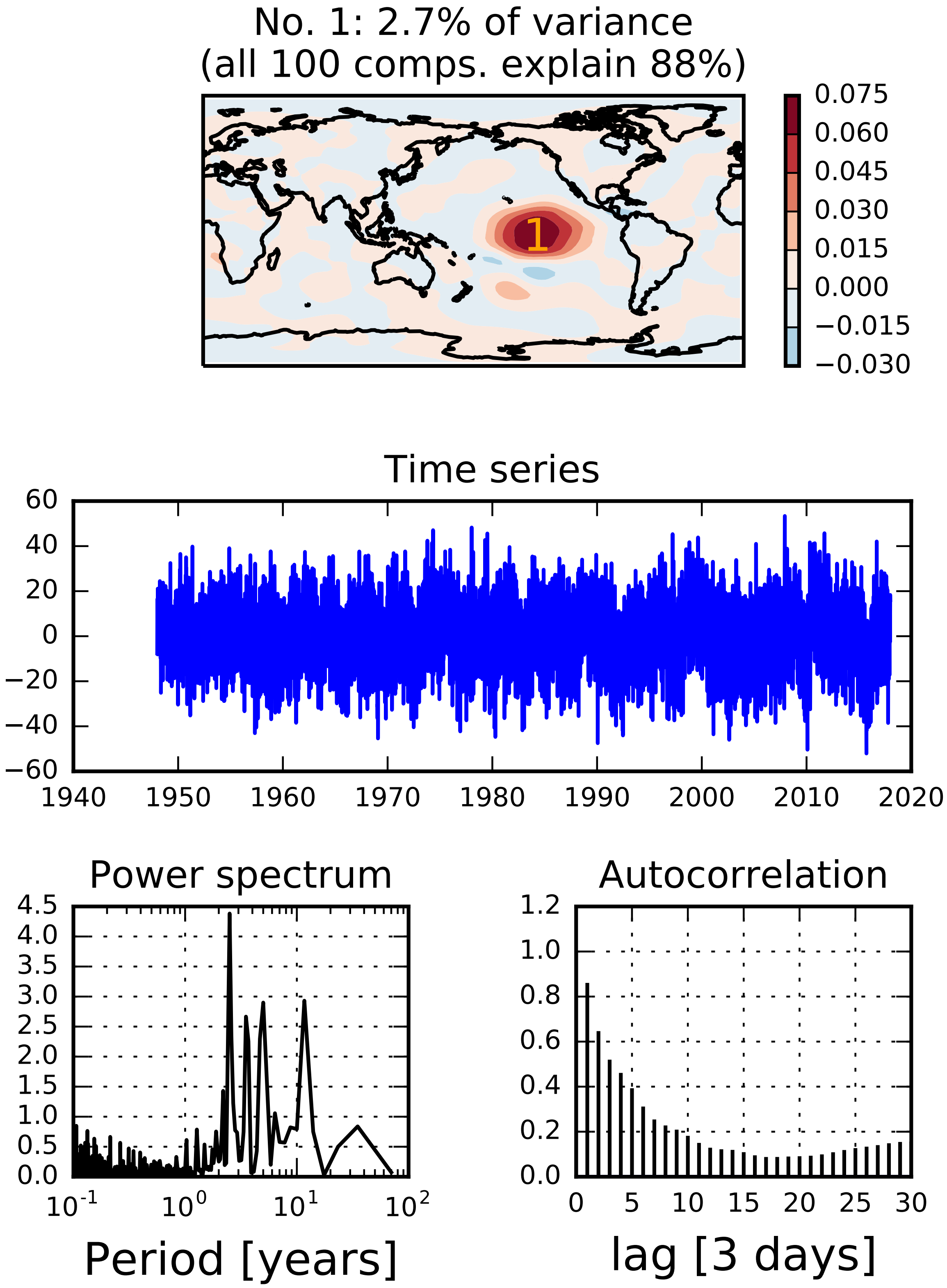
No. 1: 2.7% of variance
(all 100 comps. explain 88%)
1
0.075
0.060
0.045
0.030
0.015
0.000
−0.015
−0.030
Time series
60
40
20
0
−20
−40
−60
1940
1950
1960
1970
1980
1990
2000
2010
2020
Power spectrum
4.5
4.0
3.5
3.0
2.5
2.0
1.5
1.0
0.5
0.0
10^{-1}
10^{0}
10^{1}
10^{2}
Period [years]
Autocorrelation
1.2
1.0
0.8
0.6
0.4
0.2
0.0
0
5
10
15
20
25
30
lag [3 days]

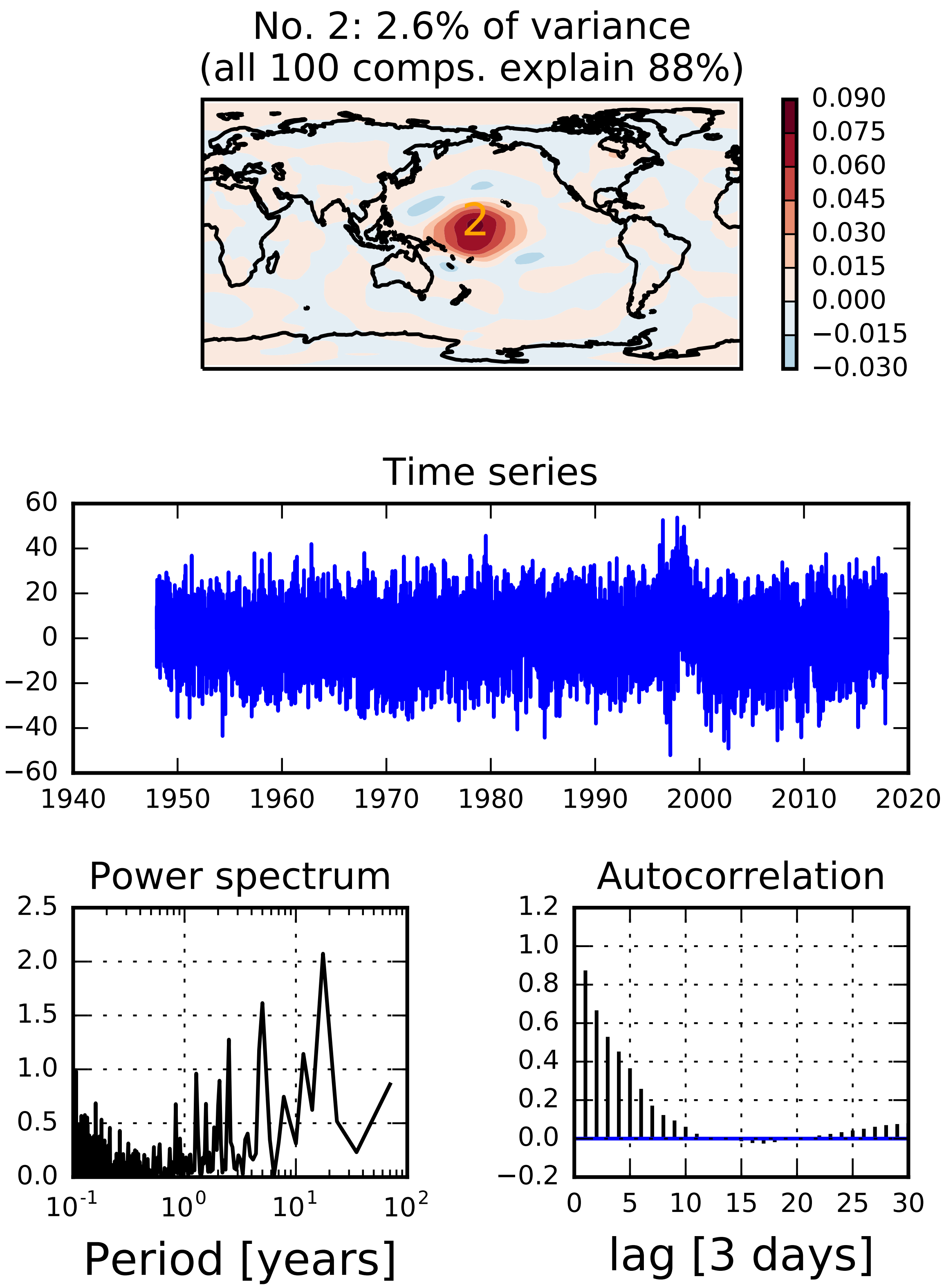
No. 2: 2.6% of variance
(all 100 comps. explain 88%)
2
0.090
0.075
0.060
0.045
0.030
0.015
0.000
−0.015
−0.030
Time series
60
40
20
0
−20
−40
−60
1940
1950
1960
1970
1980
1990
2000
2010
2020
Power spectrum
2.5
2.0
1.5
1.0
0.5
0.0
10^{-1}
10^{0}
10^{1}
10^{2}
Period [years]
Autocorrelation
1.2
1.0
0.8
0.6
0.4
0.2
0.0
−0.2
0
5
10
15
20
25
30
lag [3 days]

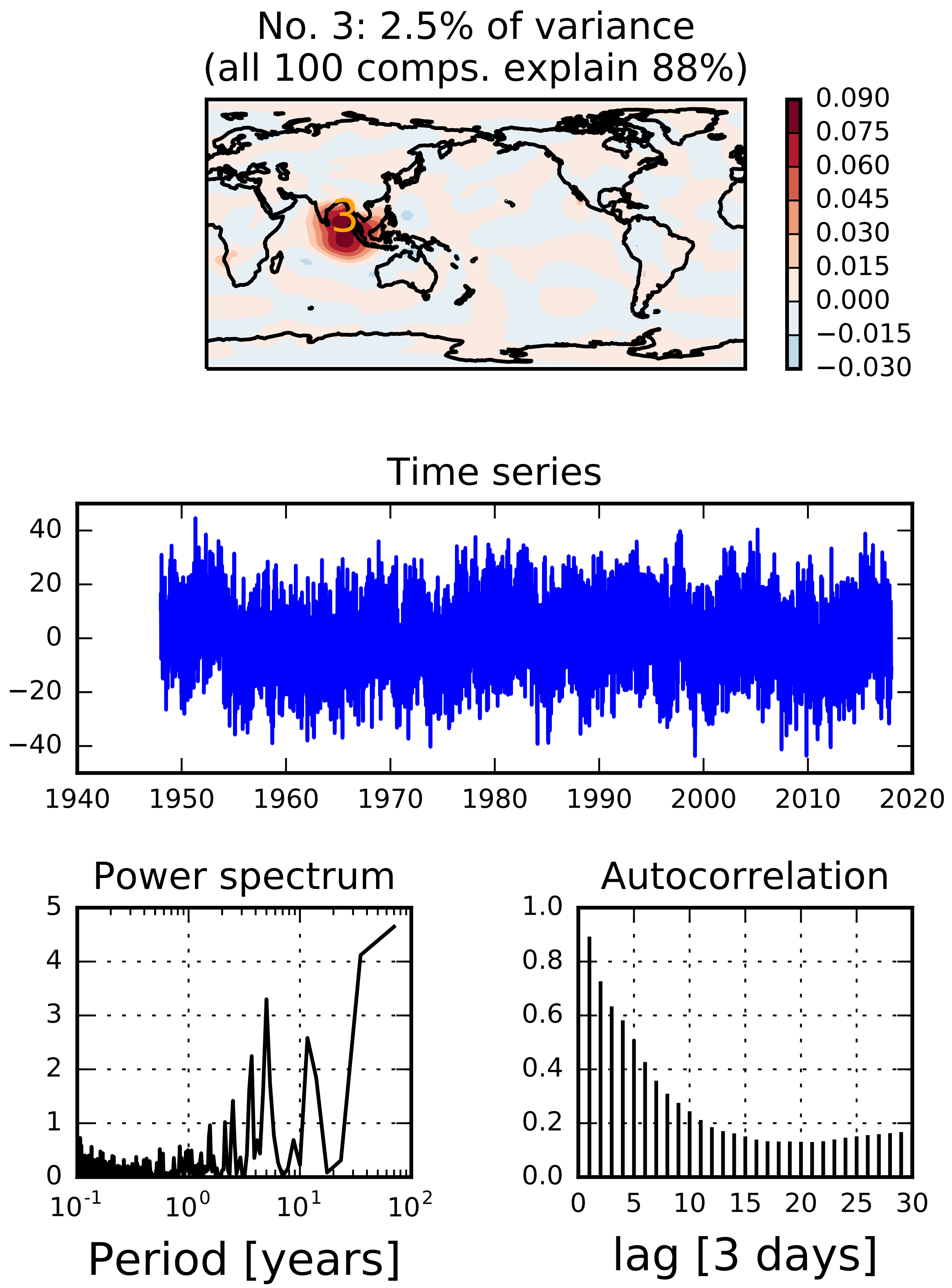
No. 3: 2.5% of variance
(all 100 comps. explain 88%)
3
0.090
0.075
0.060
0.045
0.030
0.015
0.000
−0.015
−0.030
Time series
40
20
0
−20
−40
1940
1950
1960
1970
1980
1990
2000
2010
2020
Power spectrum
5
4
3
2
1
0
10^{-1}
10^{0}
10^{1}
10^{2}
Period [years]
Autocorrelation
1.0
0.8
0.6
0.4
0.2
0.0
0
5
10
15
20
25
30
lag [3 days]

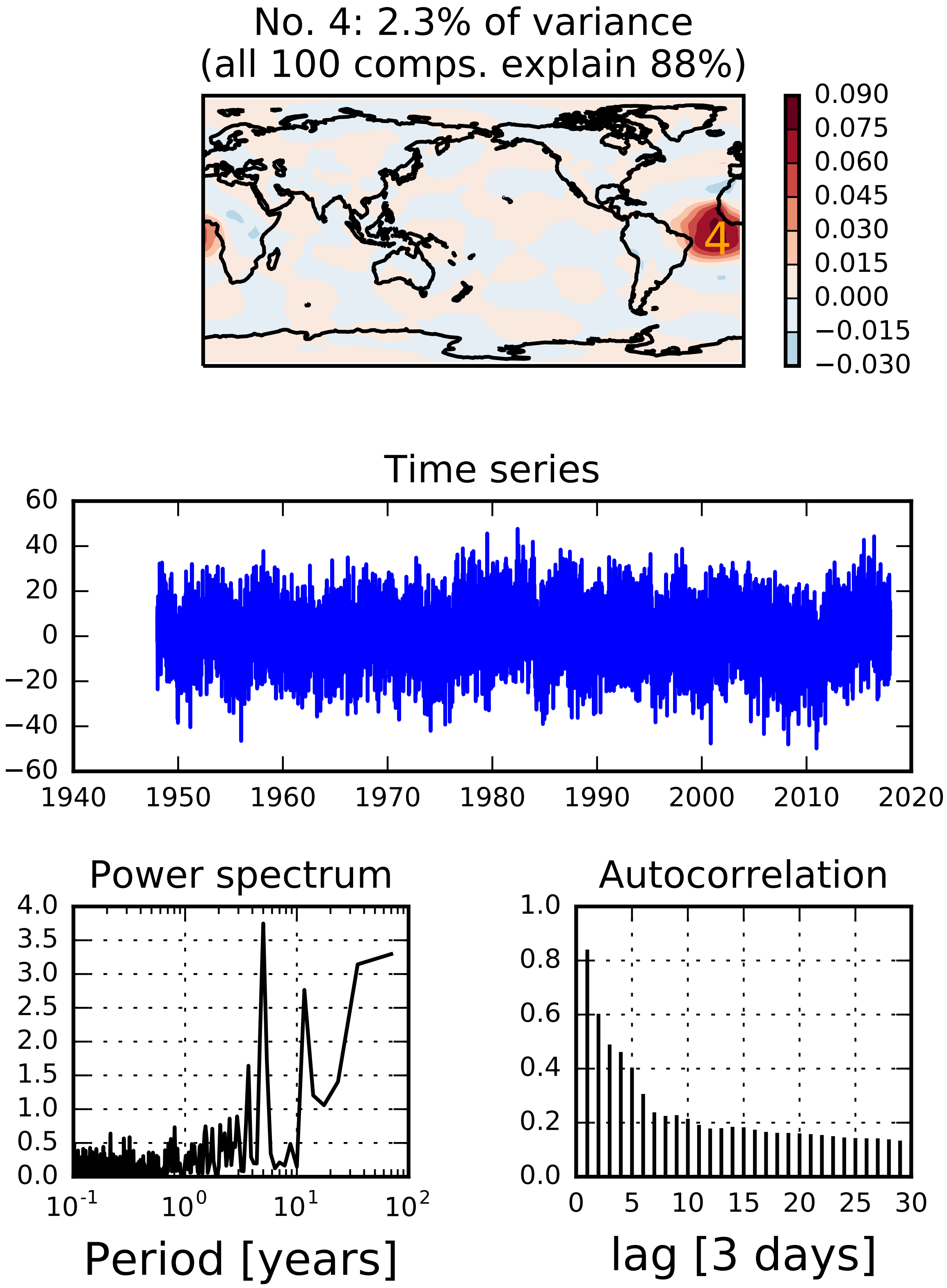
No. 4: 2.3% of variance
(all 100 comps. explain 88%)
4
0.090
0.075
0.060
0.045
0.030
0.015
0.000
−0.015
−0.030
Time series
60
40
20
0
−20
−40
−60
1940
1950
1960
1970
1980
1990
2000
2010
2020
Power spectrum
4.0
3.5
3.0
2.5
2.0
1.5
1.0
0.5
0.0
10^-1
10^0
10^1
10^2
Period [years]
Autocorrelation
1.0
0.8
0.6
0.4
0.2
0.0
0
5
10
15
20
25
30
lag [3 days]

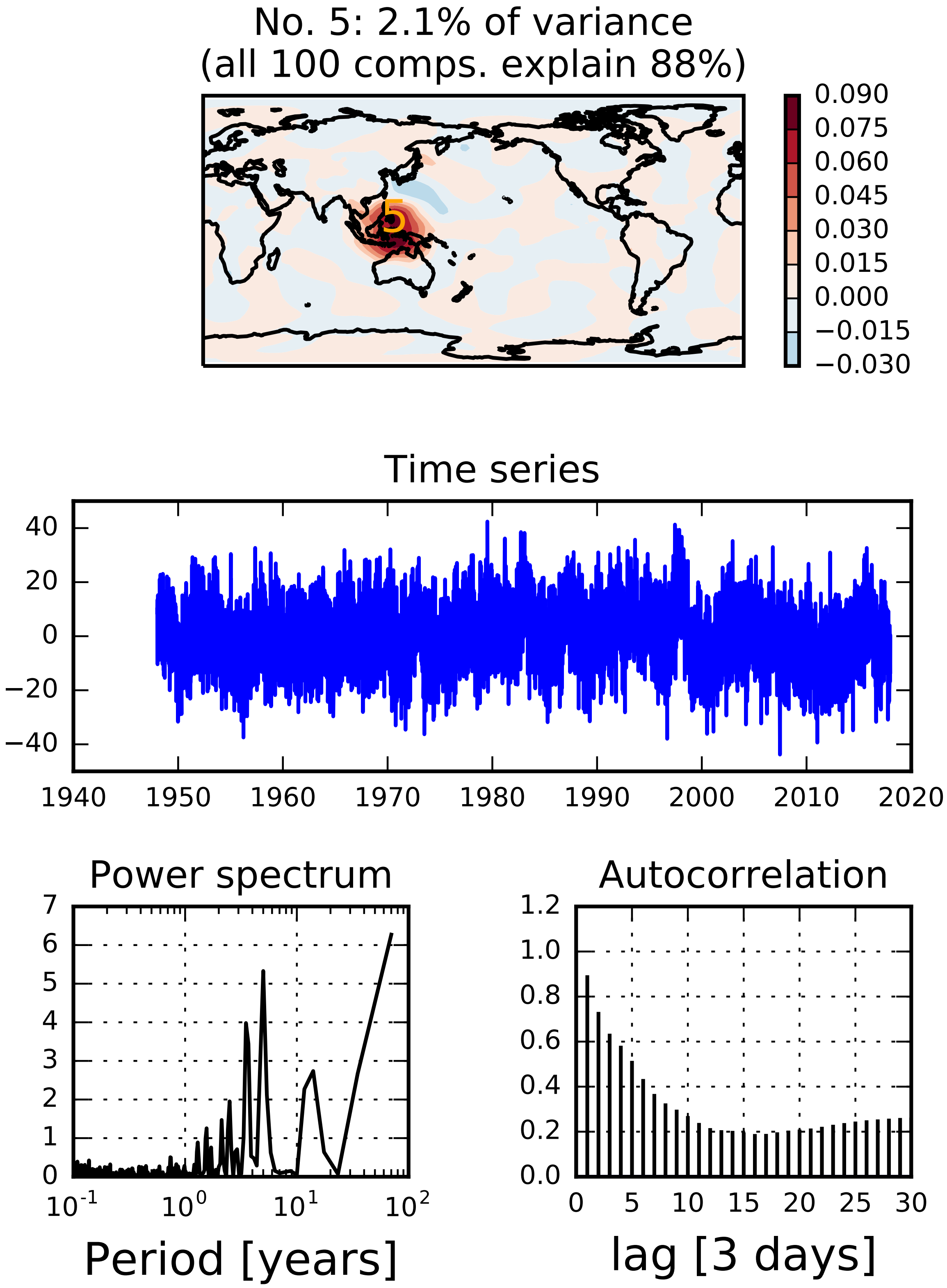
No. 5: 2.1% of variance
(all 100 comps. explain 88%)
5
0.090
0.075
0.060
0.045
0.030
0.015
0.000
−0.015
−0.030
Time series
40
20
0
−20
−40
1940
1950
1960
1970
1980
1990
2000
2010
2020
Power spectrum
7
6
5
4
3
2
1
0
10^{-1}
10^{0}
10^{1}
10^{2}
Period [years]
Autocorrelation
1.2
1.0
0.8
0.6
0.4
0.2
0.0
0
5
10
15
20
25
30
lag [3 days]

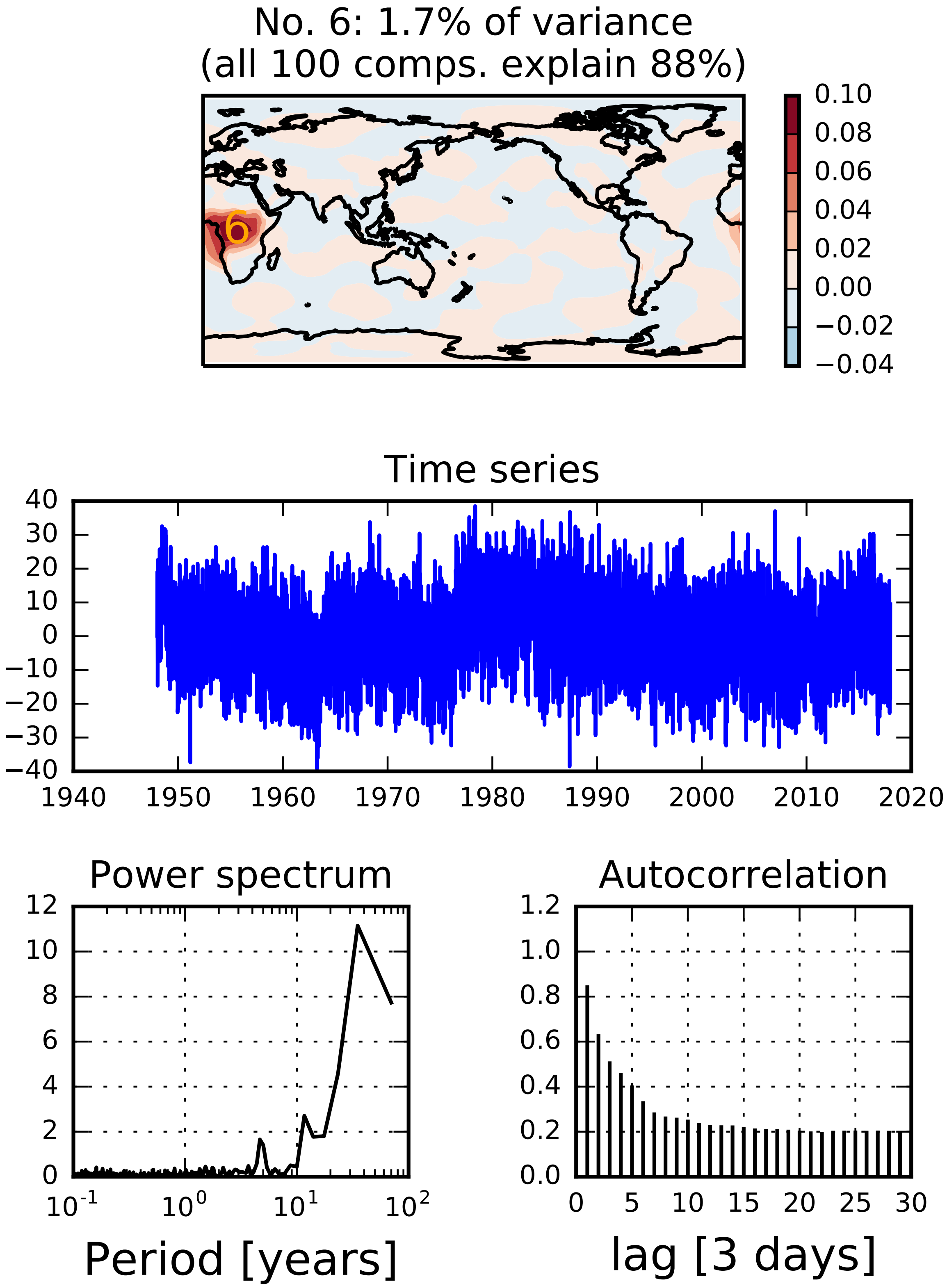
No. 6: 1.7% of variance
(all 100 comps. explain 88%)
6
0.10
0.08
0.06
0.04
0.02
0.00
−0.02
−0.04
Time series
40
30
20
10
0
−10
−20
−30
−40
1940
1950
1960
1970
1980
1990
2000
2010
2020
Power spectrum
12
10
8
6
4
2
0
10^{-1}
10^{0}
10^{1}
10^{2}
Period [years]
Autocorrelation
1.2
1.0
0.8
0.6
0.4
0.2
0.0
0
5
10
15
20
25
30
lag [3 days]

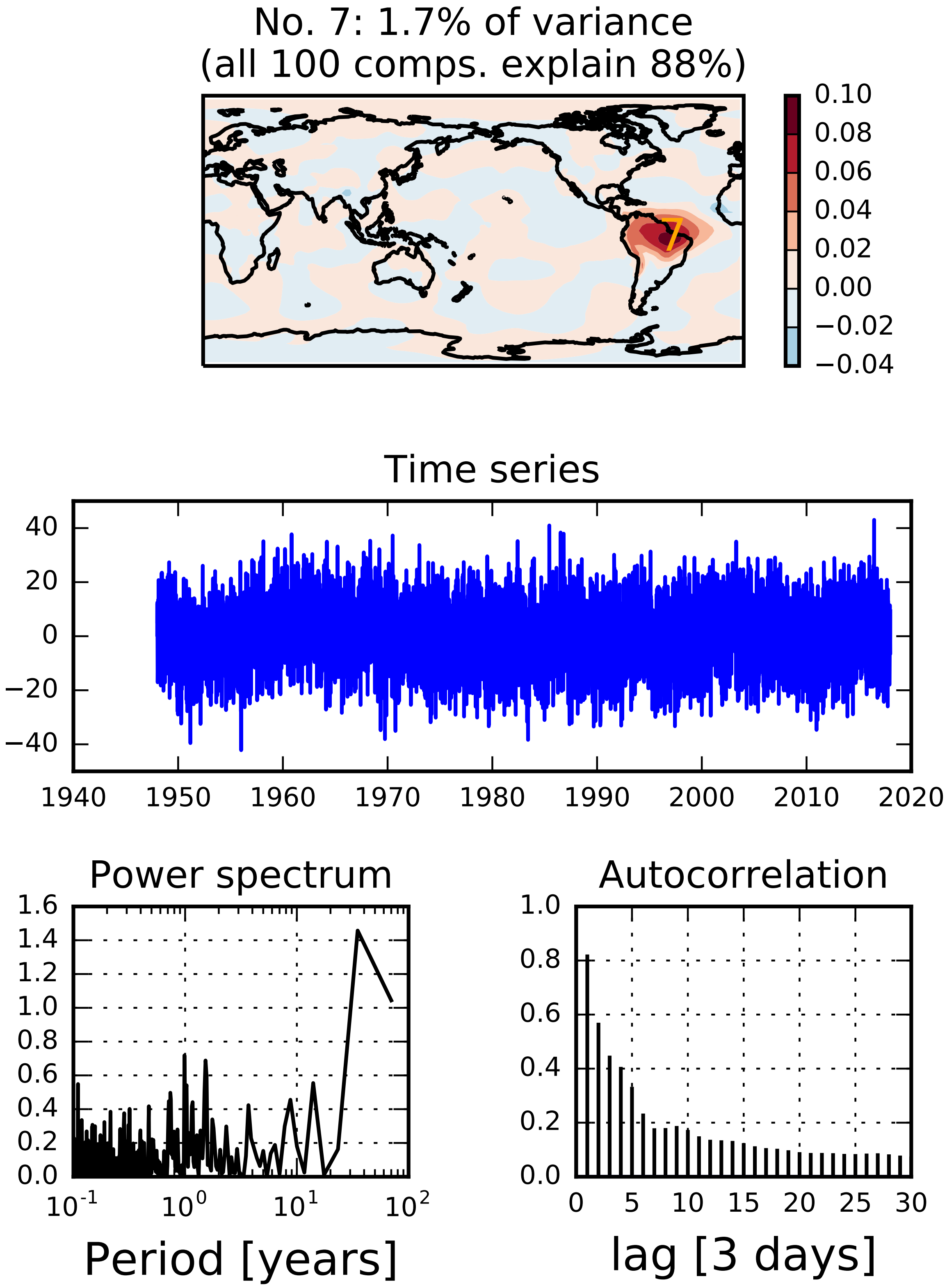
No. 7: 1.7% of variance
(all 100 comps. explain 88%)
0.10
0.08
0.06
0.04
0.02
0.00
−0.02
−0.04
Time series
40
20
0
−20
−40
1940
1950
1960
1970
1980
1990
2000
2010
2020
Power spectrum
1.6
1.4
1.2
1.0
0.8
0.6
0.4
0.2
0.0
10^-1
10^0
10^1
10^2
Period [years]
Autocorrelation
1.0
0.8
0.6
0.4
0.2
0.0
0
5
10
15
20
25
30
lag [3 days]

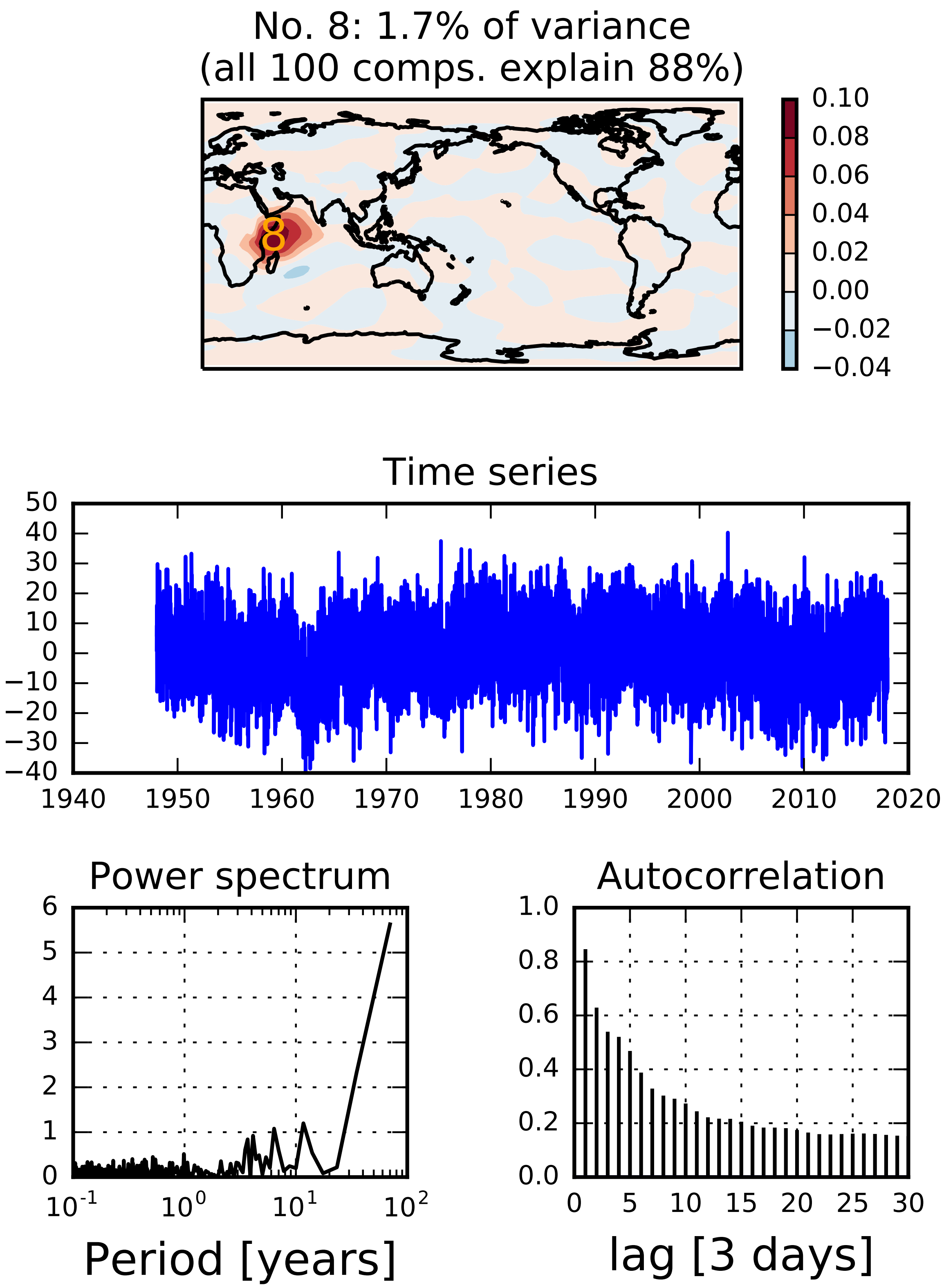
No. 8: 1.7% of variance
(all 100 comps. explain 88%)
8
0.10
0.08
0.06
0.04
0.02
0.00
−0.02
−0.04
Time series
50
40
30
20
10
0
−10
−20
−30
−40
1940
1950
1960
1970
1980
1990
2000
2010
2020
Power spectrum
6
5
4
3
2
1
0
10^{-1}
10^{0}
10^{1}
10^{2}
Period [years]
Autocorrelation
1.0
0.8
0.6
0.4
0.2
0.0
0
5
10
15
20
25
30
lag [3 days]

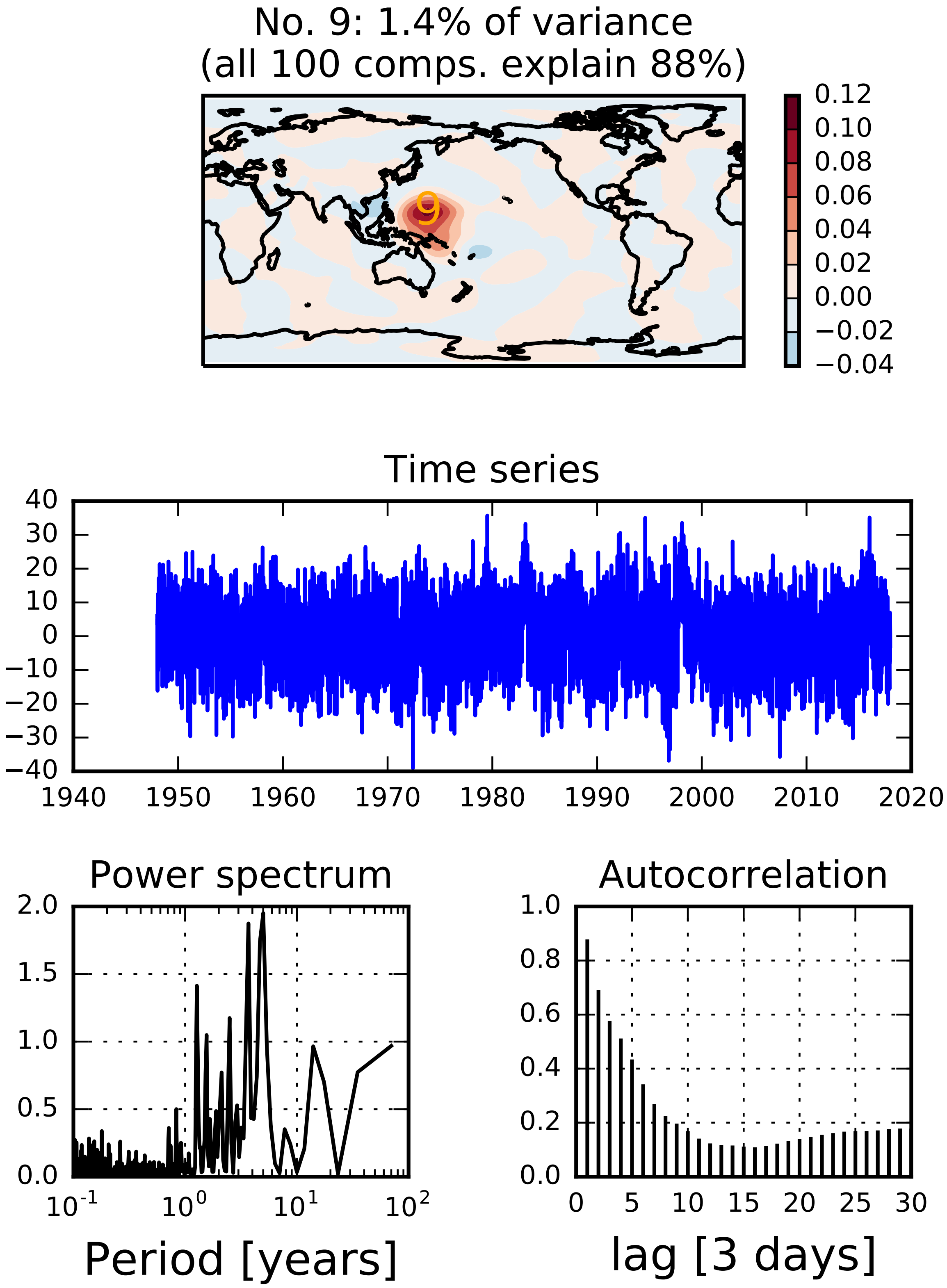
No. 9: 1.4% of variance
(all 100 comps. explain 88%)
9
0.12
0.10
0.08
0.06
0.04
0.02
0.00
−0.02
−0.04
Time series
40
30
20
10
0
−10
−20
−30
−40
1940
1950
1960
1970
1980
1990
2000
2010
2020
Power spectrum
2.0
1.5
1.0
0.5
0.0
10^{-1}
10^{0}
10^{1}
10^{2}
Period [years]
Autocorrelation
1.0
0.8
0.6
0.4
0.2
0.0
0
5
10
15
20
25
30
lag [3 days]

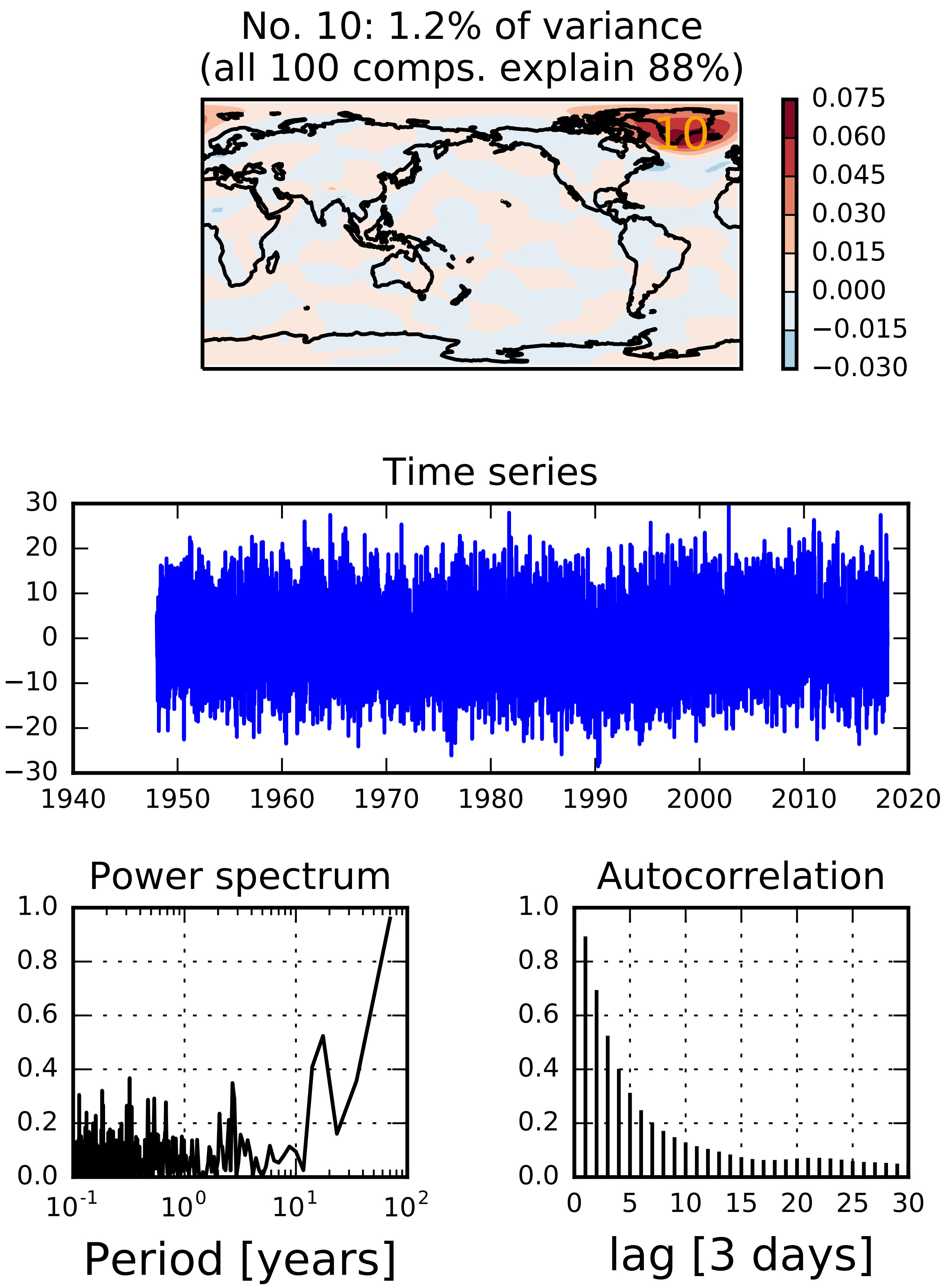
No. 10: 1.2% of variance
(all 100 comps. explain 88%)
10
0.075
0.060
0.045
0.030
0.015
0.000
−0.015
−0.030
Time series
30
20
10
0
−10
−20
−30
1940
1950
1960
1970
1980
1990
2000
2010
2020
Power spectrum
1.0
0.8
0.6
0.4
0.2
0.0
Period [years]
Autocorrelation
0
5
10
15
20
25
30
lag [3 days]

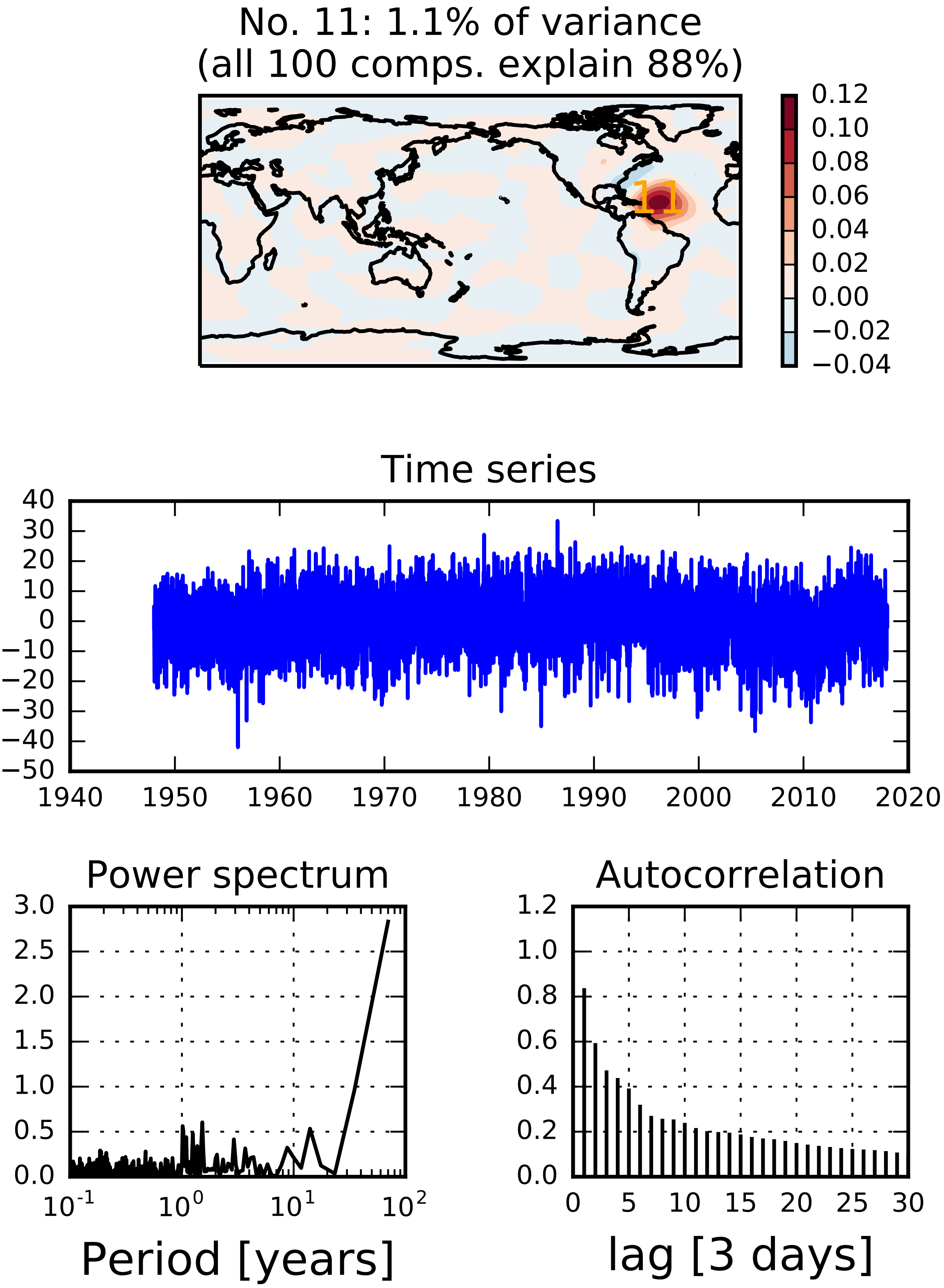
No. 11: 1.1% of variance
(all 100 comps. explain 88%)
11
0.12
0.10
0.08
0.06
0.04
0.02
0.00
−0.02
−0.04
Time series
40
30
20
10
0
−10
−20
−30
−40
−50
1940
1950
1960
1970
1980
1990
2000
2010
2020
Power spectrum
3.0
2.5
2.0
1.5
1.0
0.5
0.0
10^{-1}
10^{0}
10^{1}
10^{2}
Period [years]
Autocorrelation
1.2
1.0
0.8
0.6
0.4
0.2
0.0
0
5
10
15
20
25
30
lag [3 days]

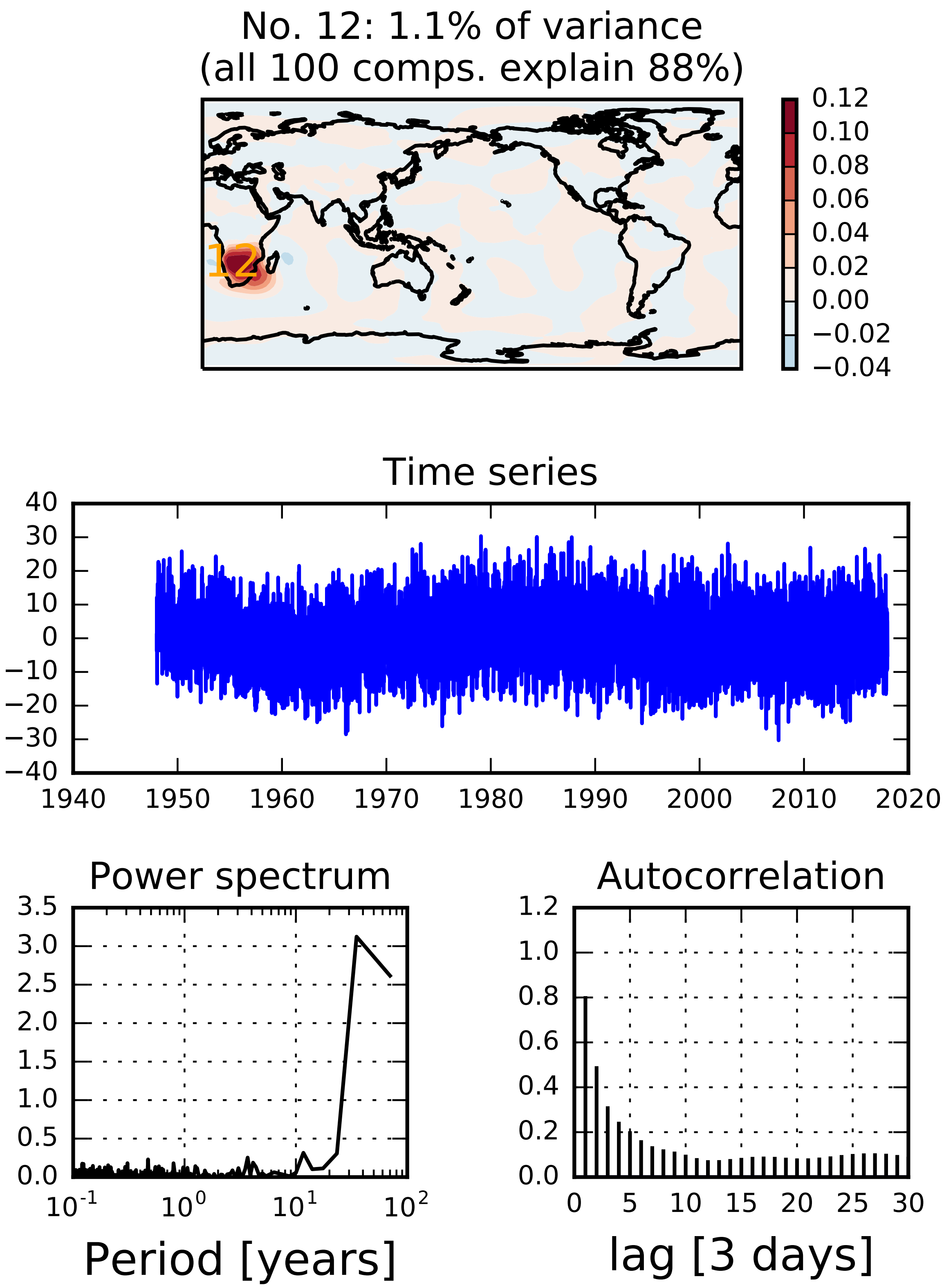
No. 12: 1.1% of variance
(all 100 comps. explain 88%)
12
0.12
0.10
0.08
0.06
0.04
0.02
0.00
−0.02
−0.04
Time series
40
30
20
10
0
−10
−20
−30
−40
1940
1950
1960
1970
1980
1990
2000
2010
2020
Power spectrum
3.5
3.0
2.5
2.0
1.5
1.0
0.5
0.0
10^{-1}
10^{0}
10^{1}
10^{2}
Period [years]
Autocorrelation
1.2
1.0
0.8
0.6
0.4
0.2
0.0
0
5
10
15
20
25
30
lag [3 days]

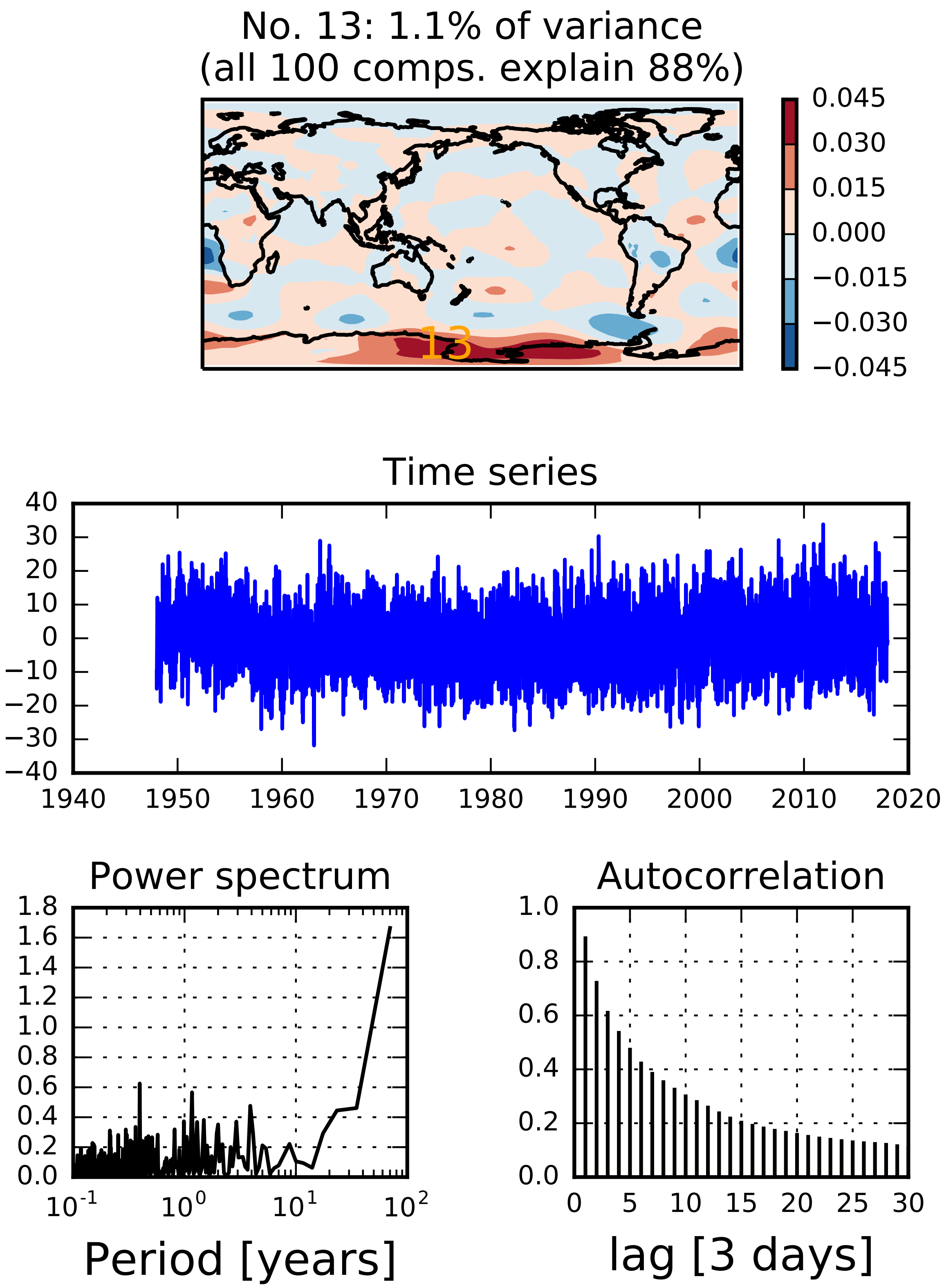
No. 13: 1.1% of variance
(all 100 comps. explain 88%)
0.045
0.030
0.015
0.000
−0.015
−0.030
−0.045
13
Time series
40
30
20
10
0
−10
−20
−30
−40
1940
1950
1960
1970
1980
1990
2000
2010
2020
Power spectrum
1.8
1.6
1.4
1.2
1.0
0.8
0.6
0.4
0.2
0.0
10^{-1}
10^{0}
10^{1}
10^{2}
Period [years]
Autocorrelation
1.0
0.8
0.6
0.4
0.2
0.0
0
5
10
15
20
25
30
lag [3 days]

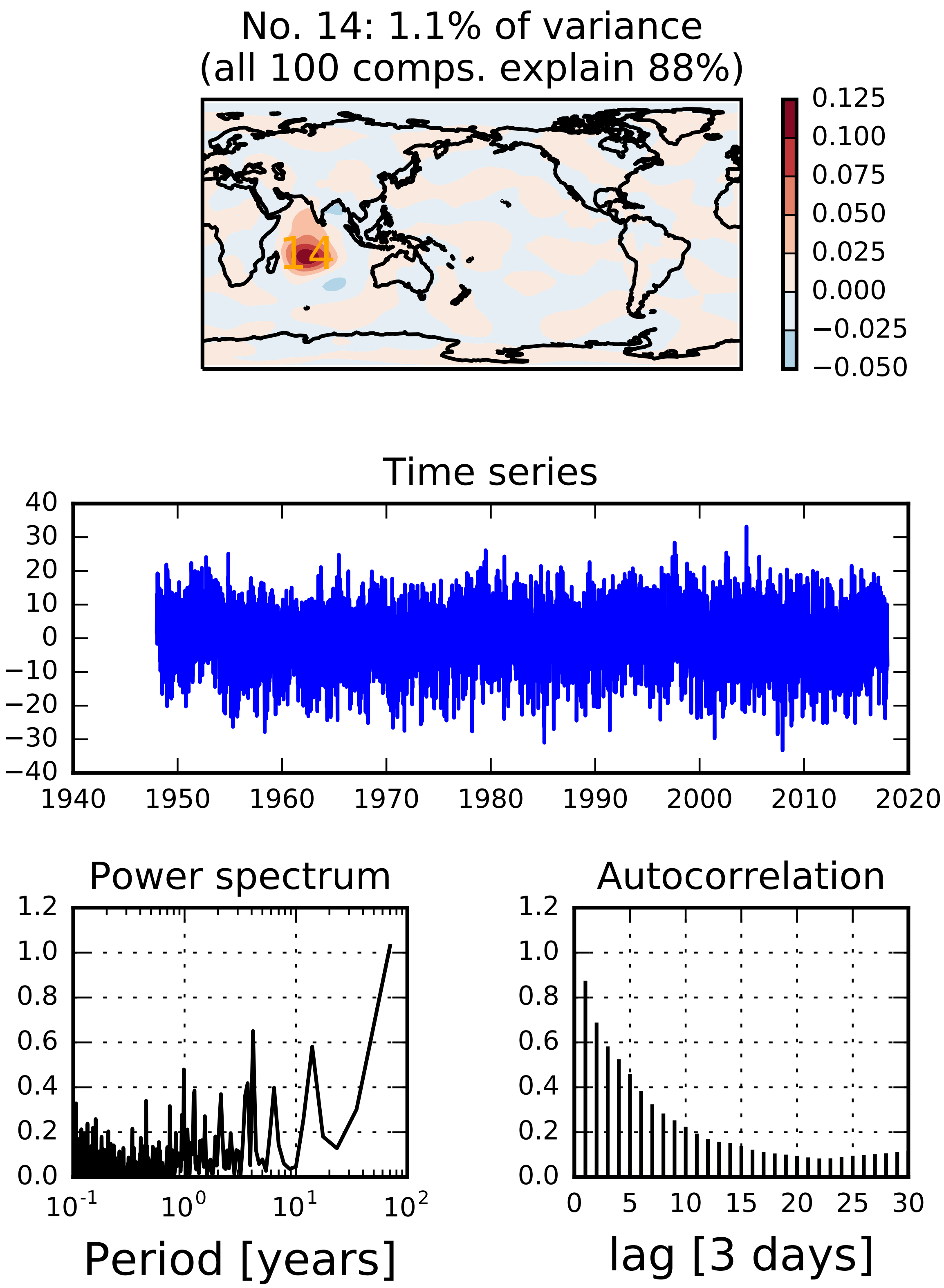
No. 14: 1.1% of variance
(all 100 comps. explain 88%)
14
0.125
0.100
0.075
0.050
0.025
0.000
−0.025
−0.050
Time series
40
30
20
10
0
−10
−20
−30
−40
1940
1950
1960
1970
1980
1990
2000
2010
2020
Power spectrum
1.2
1.0
0.8
0.6
0.4
0.2
0.0
10^{-1}
10^{0}
10^{1}
10^{2}
Period [years]
Autocorrelation
1.2
1.0
0.8
0.6
0.4
0.2
0.0
0
5
10
15
20
25
30
lag [3 days]

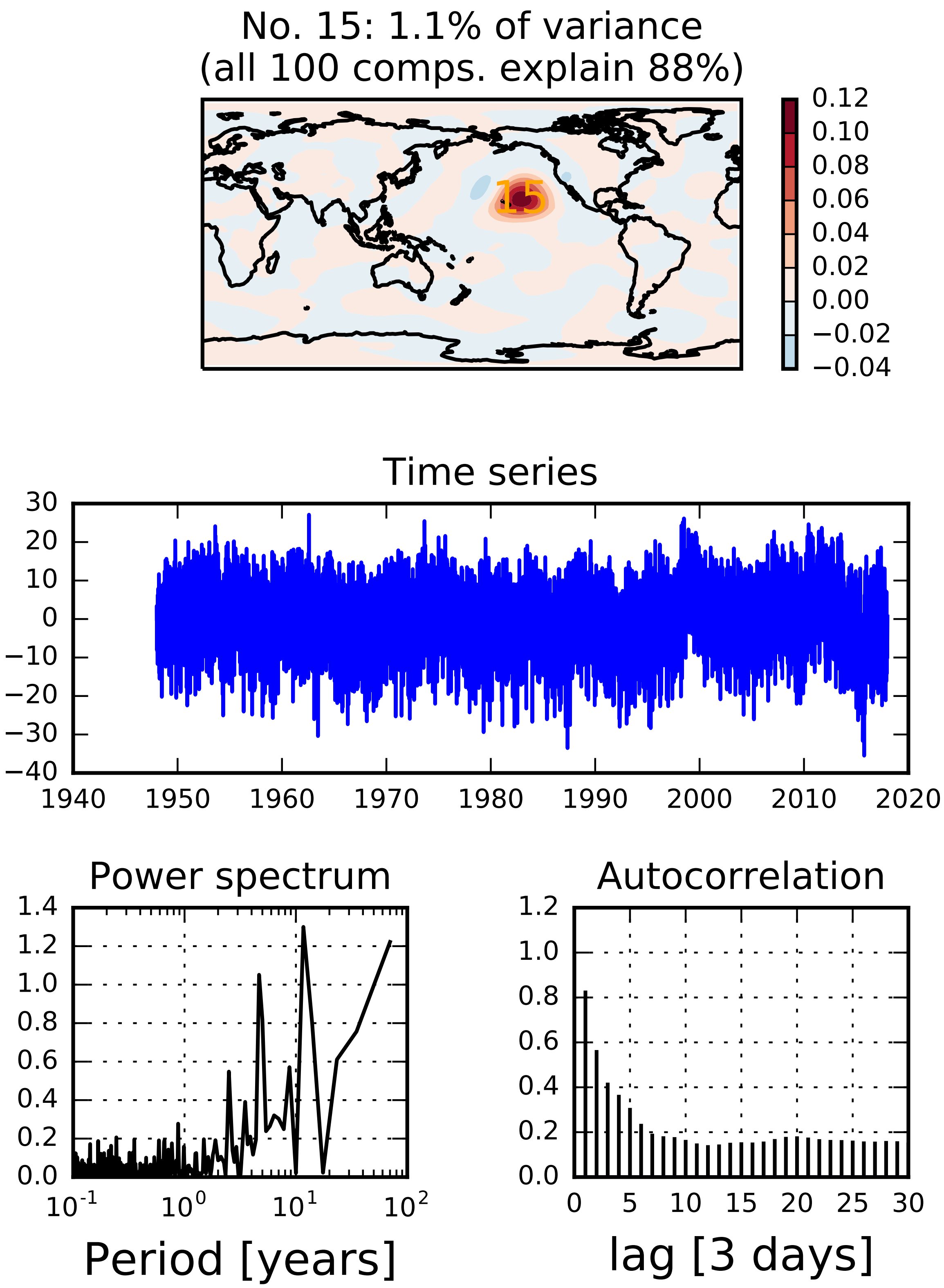
No. 15: 1.1% of variance
(all 100 comps. explain 88%)
15
0.12
0.10
0.08
0.06
0.04
0.02
0.00
−0.02
−0.04
Time series
30
20
10
0
−10
−20
−30
−40
1940
1950
1960
1970
1980
1990
2000
2010
2020
Power spectrum
1.4
1.2
1.0
0.8
0.6
0.4
0.2
0.0
10^{-1}
10^{0}
10^{1}
10^{2}
Period [years]
Autocorrelation
1.2
1.0
0.8
0.6
0.4
0.2
0.0
0
5
10
15
20
25
30
lag [3 days]

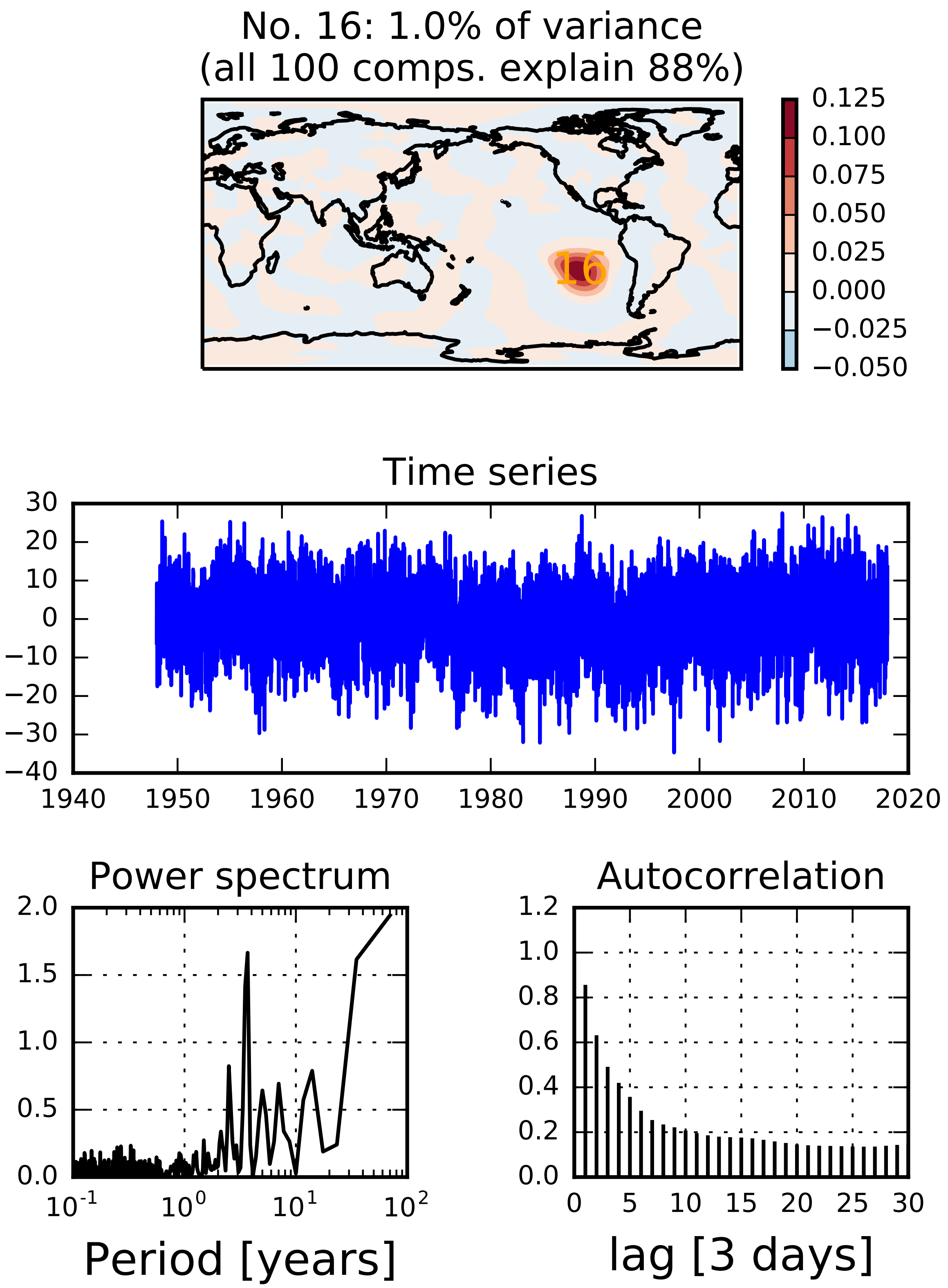
No. 16: 1.0% of variance
(all 100 comps. explain 88%)
16
0.125
0.100
0.075
0.050
0.025
0.000
−0.025
−0.050
Time series
30
20
10
0
−10
−20
−30
−40
1940
1950
1960
1970
1980
1990
2000
2010
2020
Power spectrum
2.0
1.5
1.0
0.5
0.0
10^{-1}
10^{0}
10^{1}
10^{2}
Period [years]
Autocorrelation
1.2
1.0
0.8
0.6
0.4
0.2
0.0
0
5
10
15
20
25
30
lag [3 days]

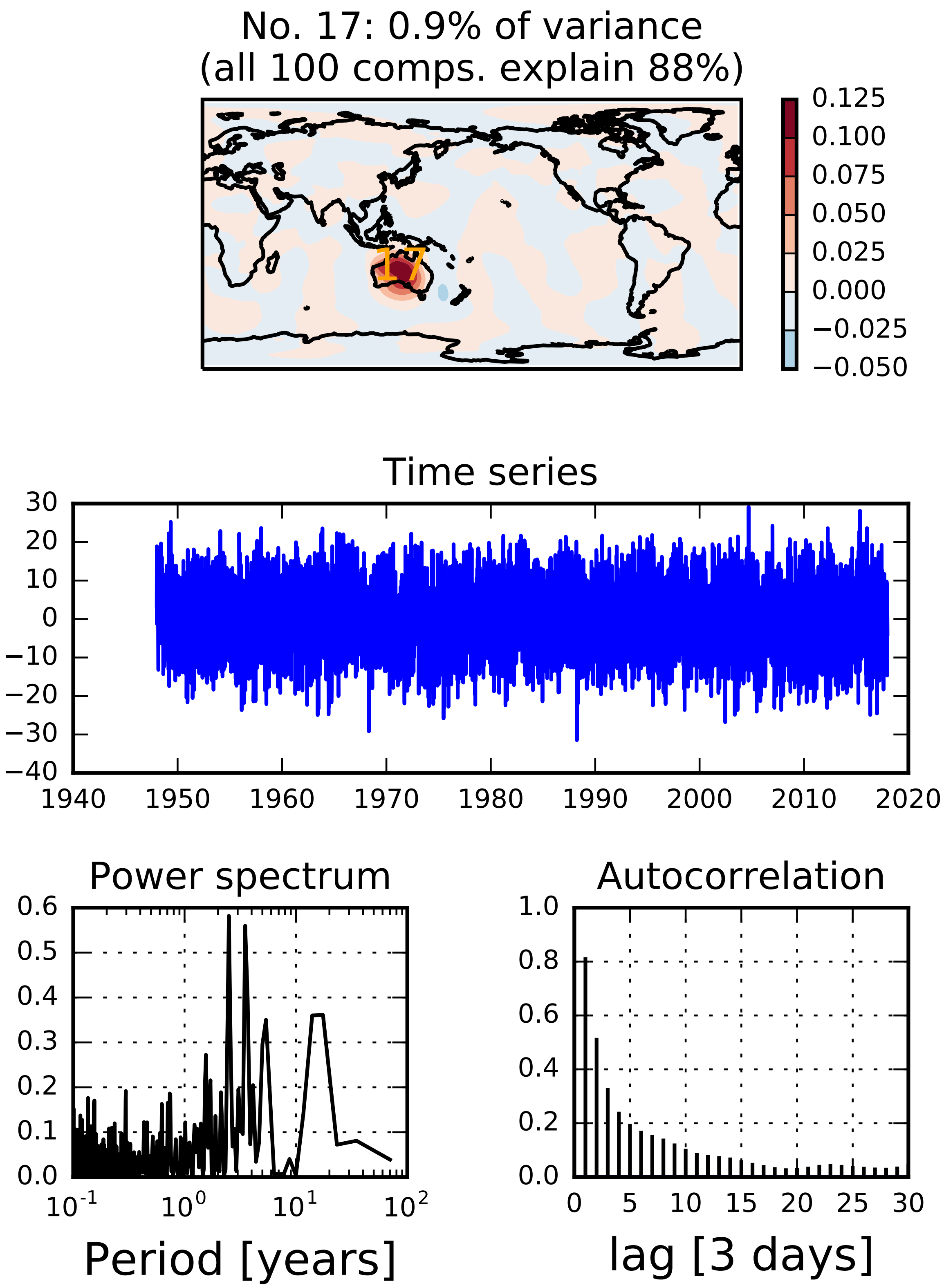
No. 17: 0.9% of variance
(all 100 comps. explain 88%)
0.125
0.100
0.075
0.050
0.025
0.000
−0.025
−0.050
17
Time series
30
20
10
0
−10
−20
−30
−40
1940
1950
1960
1970
1980
1990
2000
2010
2020
Power spectrum
0.6
0.5
0.4
0.3
0.2
0.1
0.0
10^{-1}
10^{0}
10^{1}
10^{2}
Period [years]
Autocorrelation
1.0
0.8
0.6
0.4
0.2
0.0
0
5
10
15
20
25
30
lag [3 days]

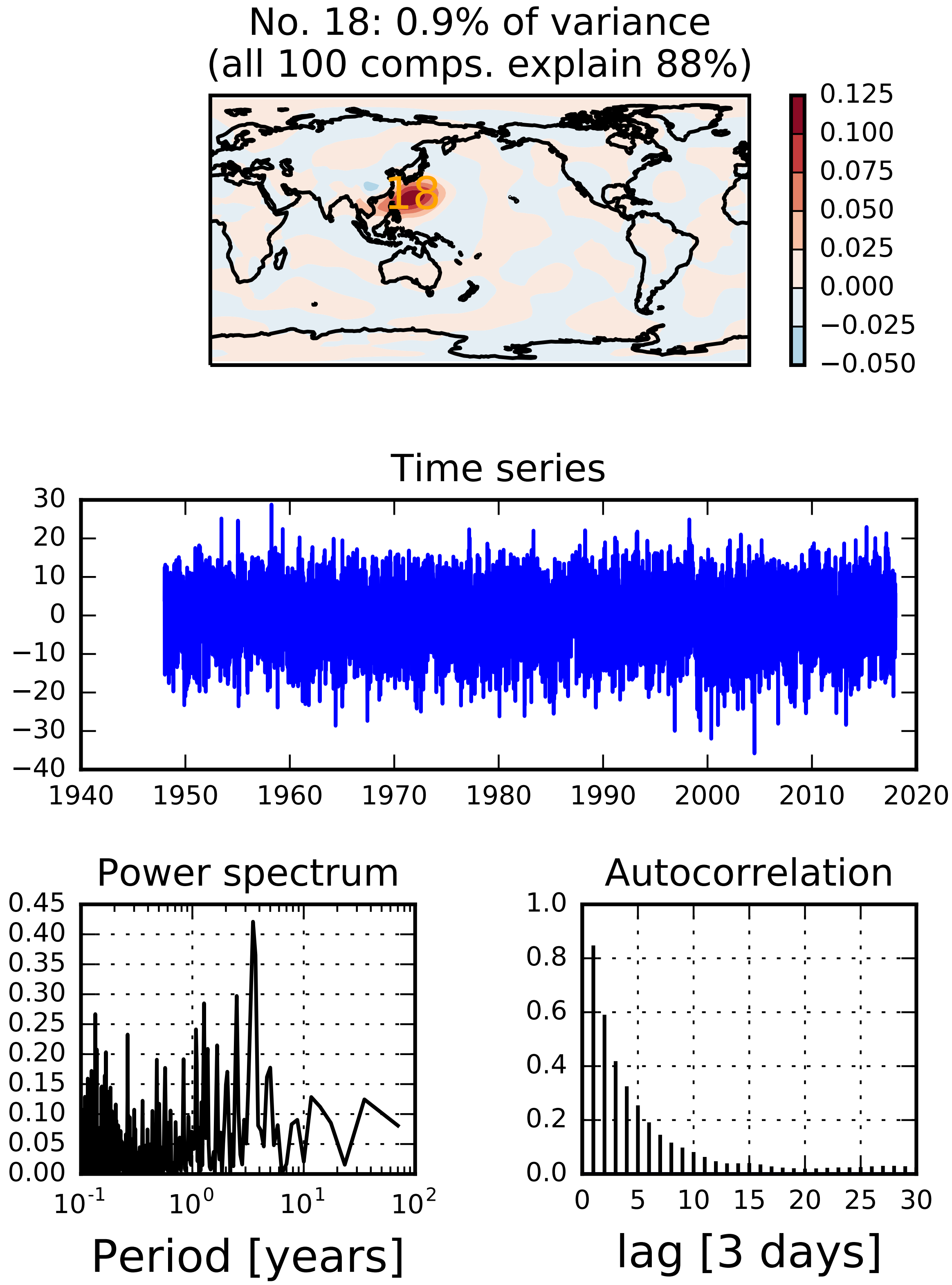
No. 18: 0.9% of variance
(all 100 comps. explain 88%)
18
0.125
0.100
0.075
0.050
0.025
0.000
−0.025
−0.050
Time series
30
20
10
0
−10
−20
−30
−40
1940
1950
1960
1970
1980
1990
2000
2010
2020
Power spectrum
0.45
0.40
0.35
0.30
0.25
0.20
0.15
0.10
0.05
0.00
10^{-1}
10^{0}
10^{1}
10^{2}
Period [years]
Autocorrelation
1.0
0.8
0.6
0.4
0.2
0.0
0
5
10
15
20
25
30
lag [3 days]

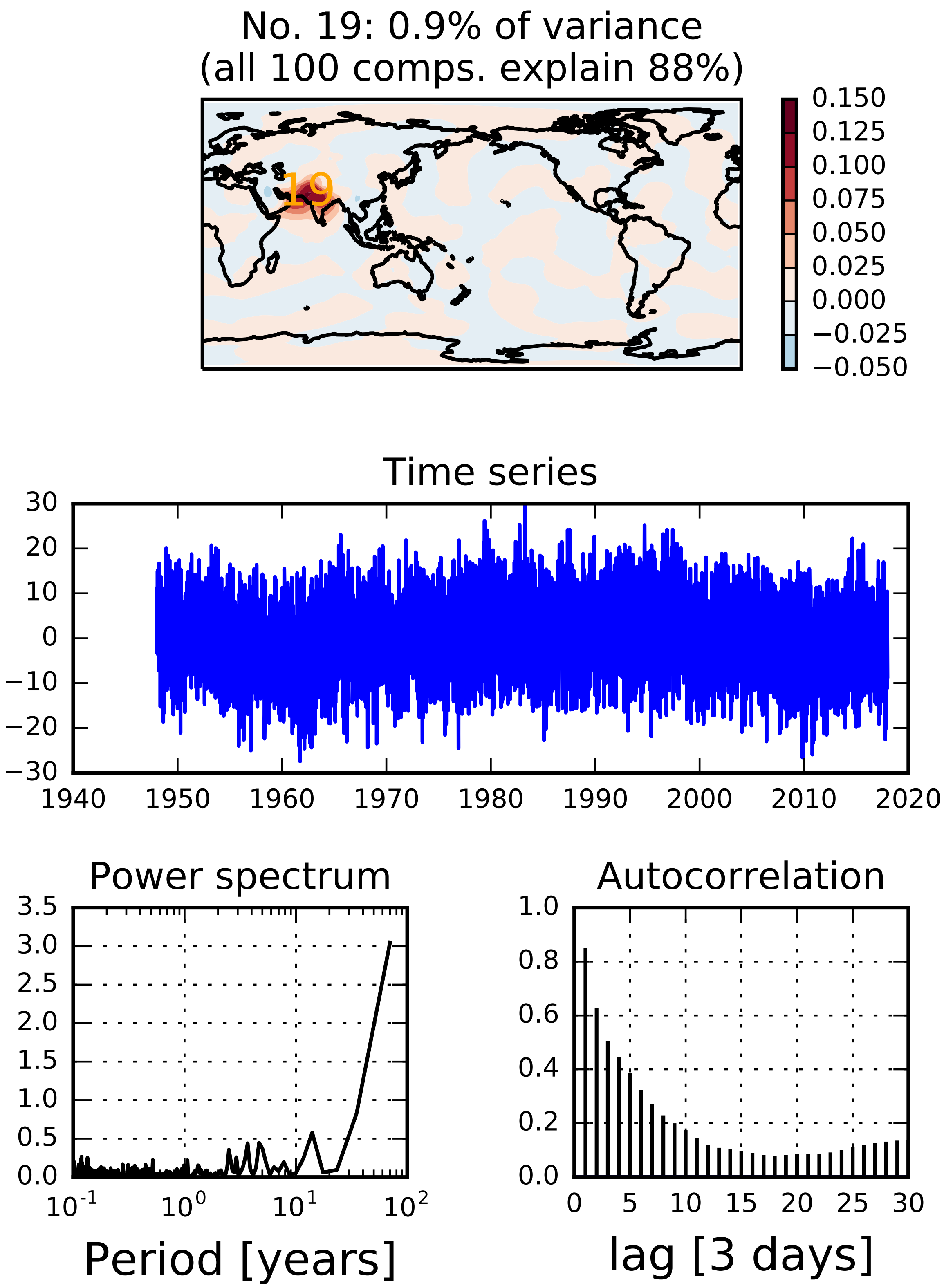
No. 19: 0.9% of variance
(all 100 comps. explain 88%)
19
0.150
0.125
0.100
0.075
0.050
0.025
0.000
−0.025
−0.050
Time series
30
20
10
0
−10
−20
−30
1940
1950
1960
1970
1980
1990
2000
2010
2020
Power spectrum
3.5
3.0
2.5
2.0
1.5
1.0
0.5
0.0
10^{-1}
10^{0}
10^{1}
10^{2}
Period [years]
Autocorrelation
1.0
0.8
0.6
0.4
0.2
0.0
0
5
10
15
20
25
30
lag [3 days]

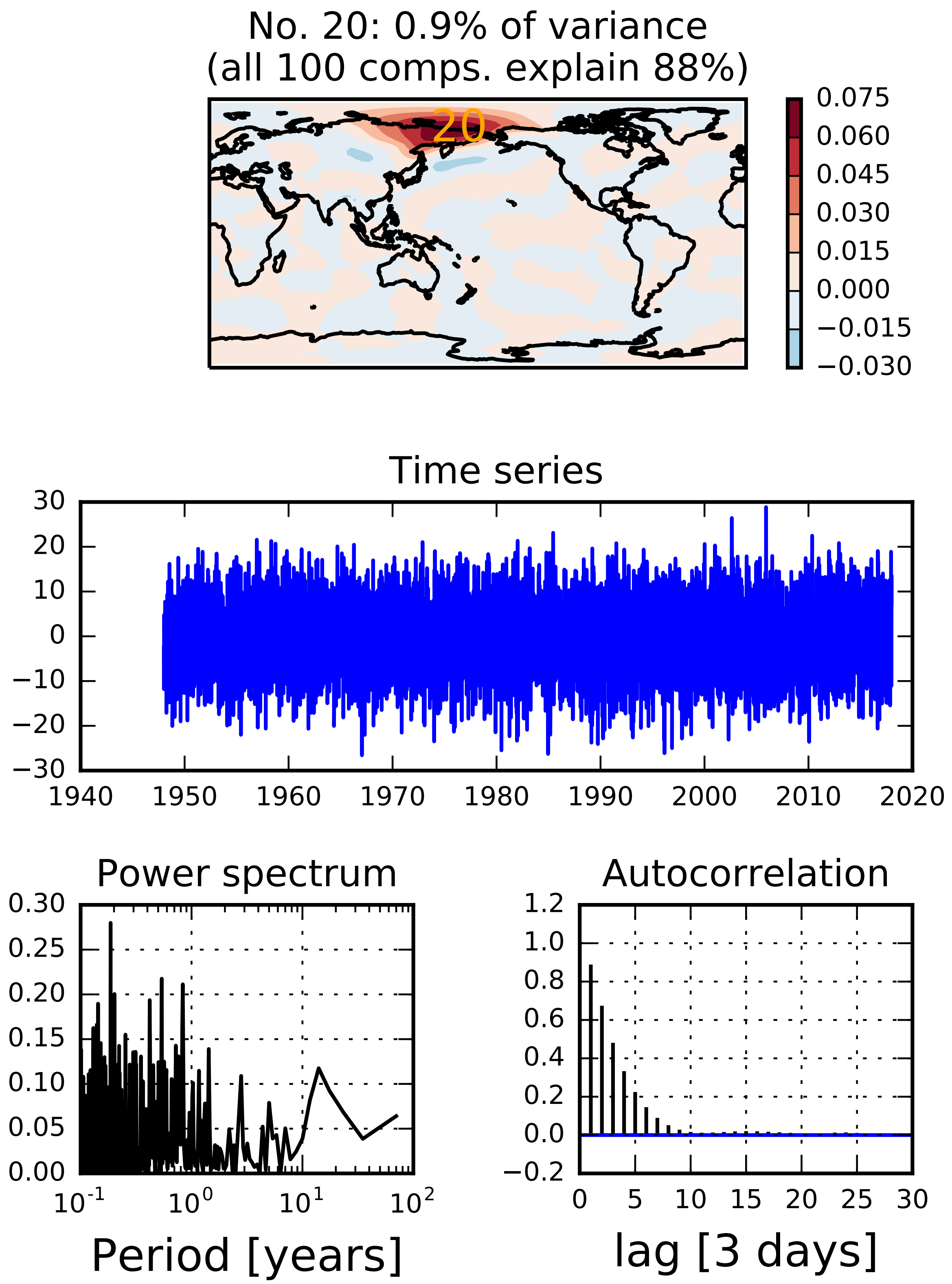
No. 20: 0.9% of variance
(all 100 comps. explain 88%)
20
0.075
0.060
0.045
0.030
0.015
0.000
−0.015
−0.030
Time series
30
20
10
0
−10
−20
−30
1940
1950
1960
1970
1980
1990
2000
2010
2020
Power spectrum
0.30
0.25
0.20
0.15
0.10
0.05
0.00
10^{-1}
10^{0}
10^{1}
10^{2}
Period [years]
Autocorrelation
1.2
1.0
0.8
0.6
0.4
0.2
0.0
−0.2
0
5
10
15
20
25
30
lag [3 days]

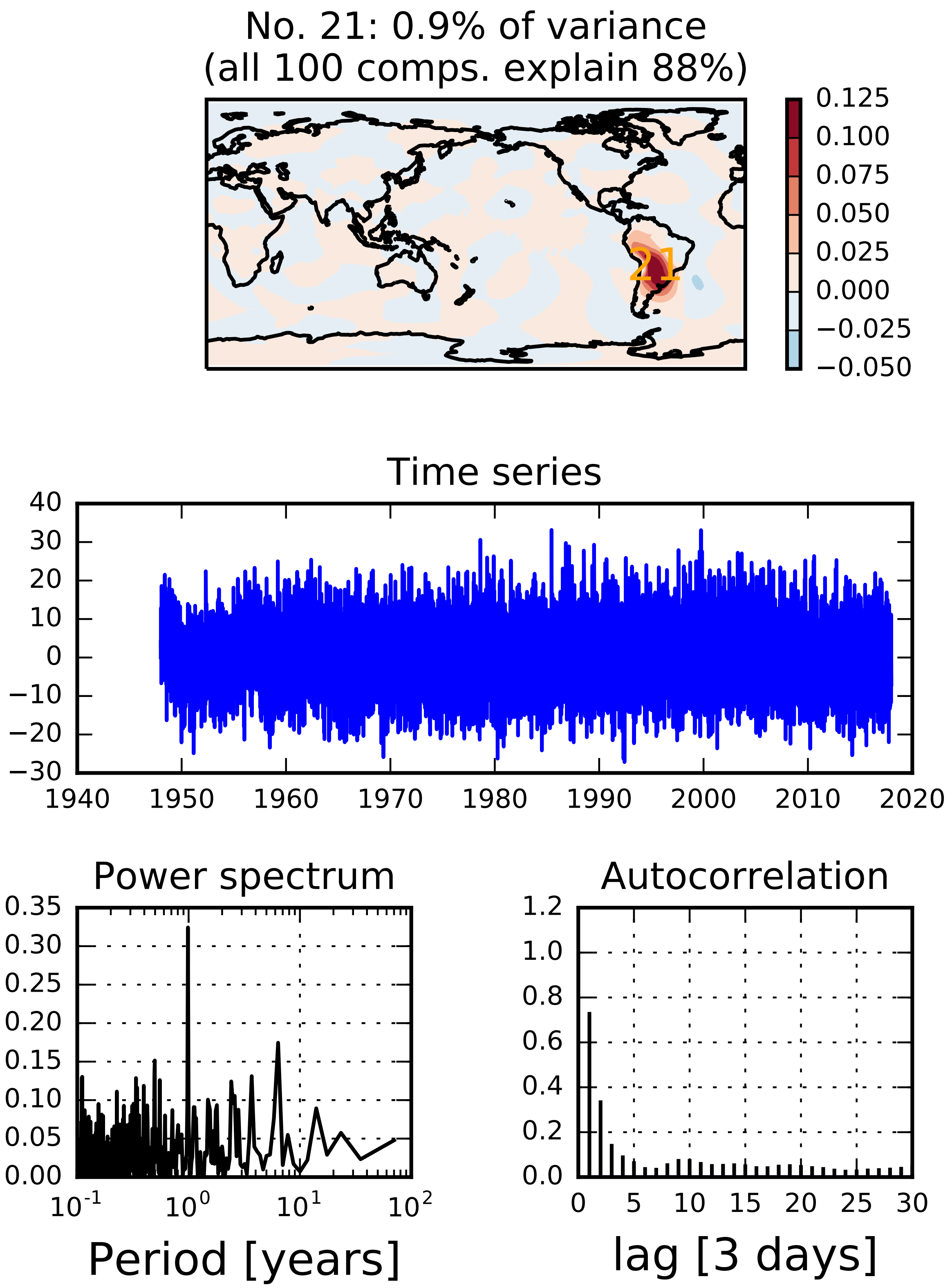
No. 21: 0.9% of variance
(all 100 comps. explain 88%)
21
0.125
0.100
0.075
0.050
0.025
0.000
−0.025
−0.050
Time series
40
30
20
10
0
−10
−20
−30
1940
1950
1960
1970
1980
1990
2000
2010
2020
Power spectrum
0.35
0.30
0.25
0.20
0.15
0.10
0.05
0.00
10^-1
10^0
10^1
10^2
Period [years]
Autocorrelation
1.2
1.0
0.8
0.6
0.4
0.2
0.0
0
5
10
15
20
25
30
lag [3 days]

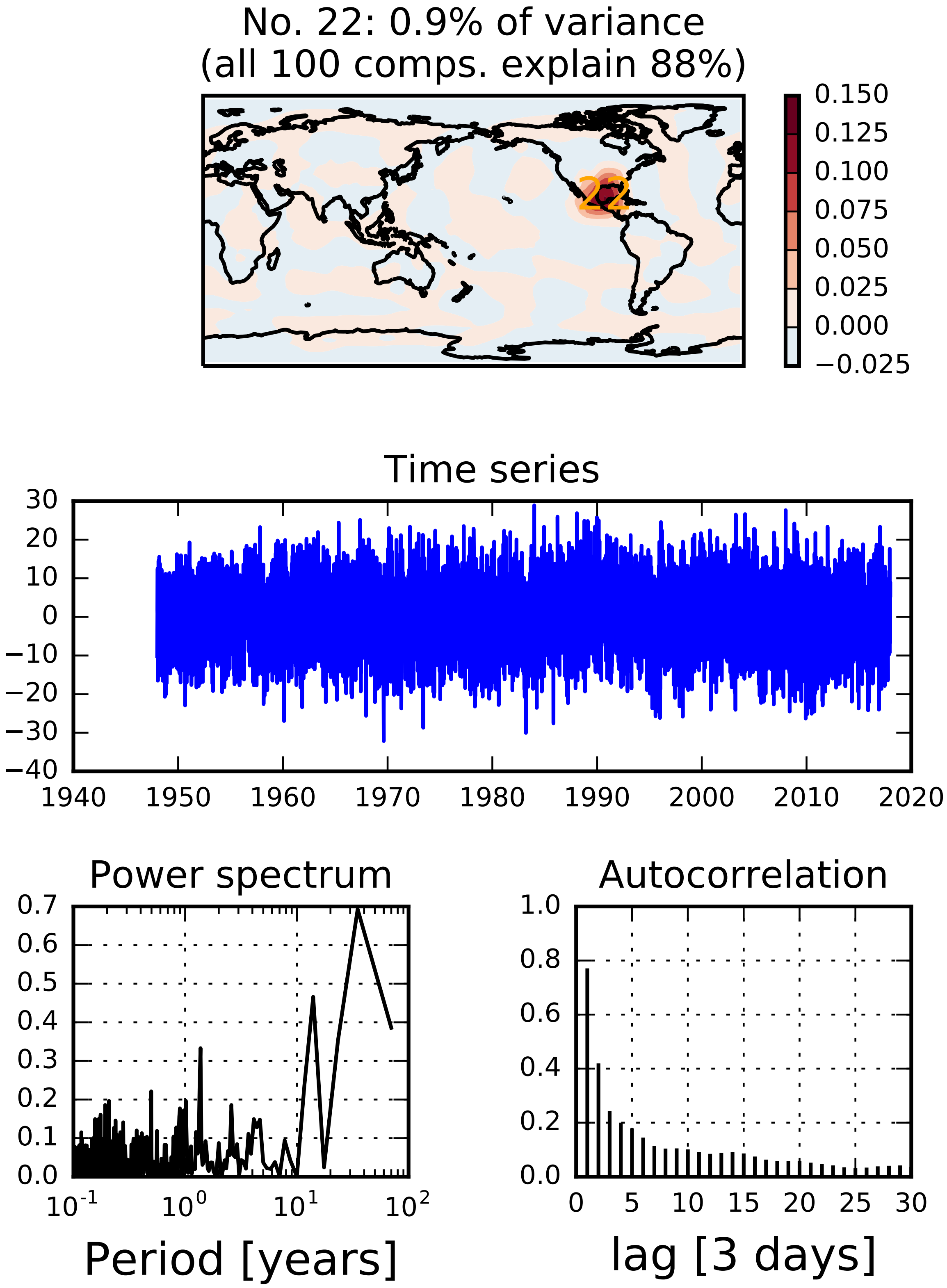
No. 22: 0.9% of variance
(all 100 comps. explain 88%)
22
0.150
0.125
0.100
0.075
0.050
0.025
0.000
−0.025
Time series
30
20
10
0
−10
−20
−30
−40
1940
1950
1960
1970
1980
1990
2000
2010
2020
Power spectrum
0.7
0.6
0.5
0.4
0.3
0.2
0.1
0.0
10^{-1}
10^{0}
10^{1}
10^{2}
Period [years]
Autocorrelation
1.0
0.8
0.6
0.4
0.2
0.0
0
5
10
15
20
25
30
lag [3 days]

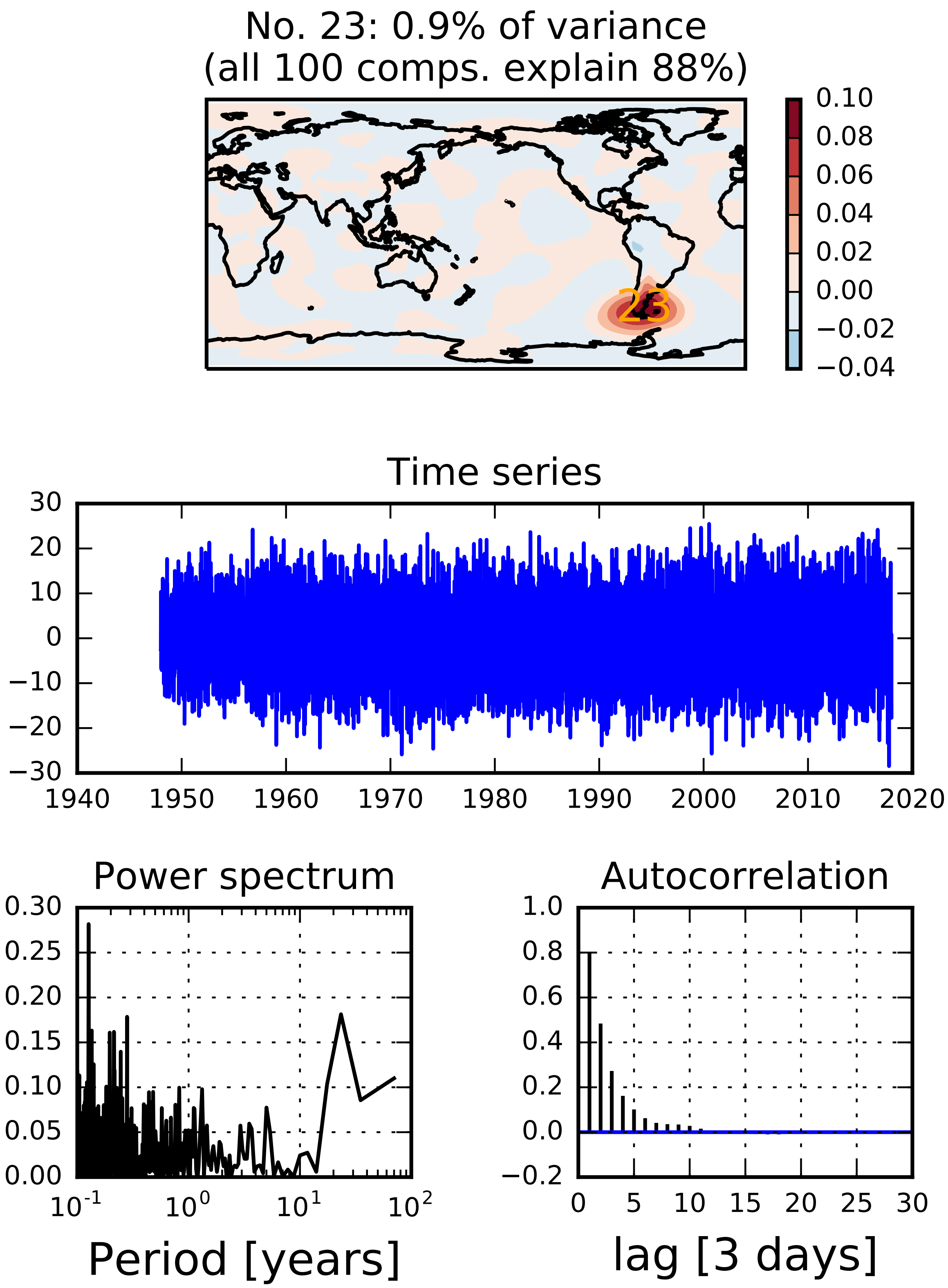
No. 23: 0.9% of variance
(all 100 comps. explain 88%)
0.10
0.08
0.06
0.04
0.02
0.00
−0.02
−0.04
23
Time series
30
20
10
0
−10
−20
−30
1940
1950
1960
1970
1980
1990
2000
2010
2020
Power spectrum
0.30
0.25
0.20
0.15
0.10
0.05
0.00
10^-1
10^0
10^1
10^2
Period [years]
Autocorrelation
1.0
0.8
0.6
0.4
0.2
0.0
−0.2
0
5
10
15
20
25
30
lag [3 days]

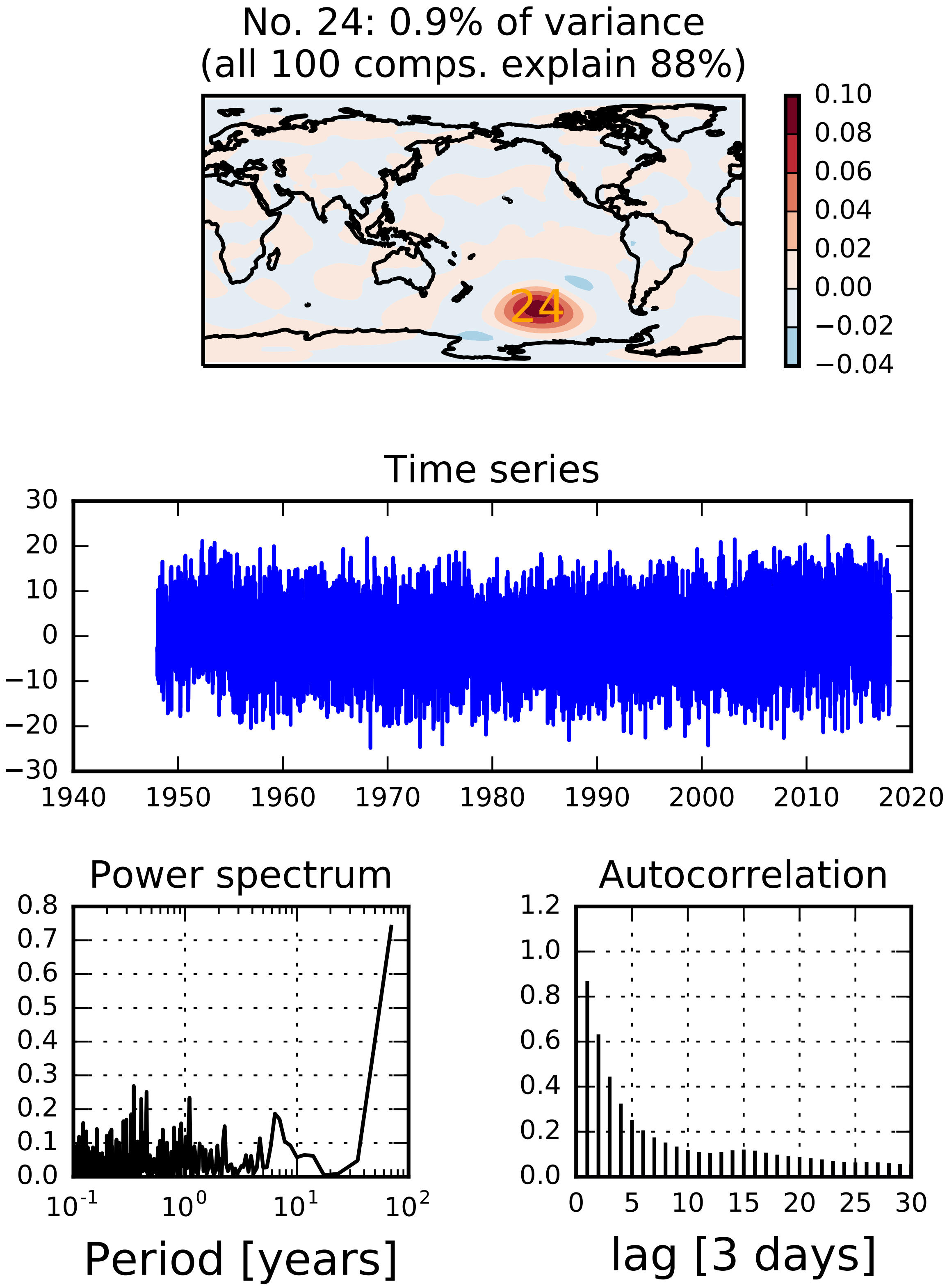
No. 24: 0.9% of variance
(all 100 comps. explain 88%)
24
0.10
0.08
0.06
0.04
0.02
0.00
−0.02
−0.04
Time series
30
20
10
0
−10
−20
−30
1940
1950
1960
1970
1980
1990
2000
2010
2020
Power spectrum
0.8
0.7
0.6
0.5
0.4
0.3
0.2
0.1
0.0
10-1
100
101
102
Period [years]
Autocorrelation
1.2
1.0
0.8
0.6
0.4
0.2
0.0
0
5
10
15
20
25
30
lag [3 days]

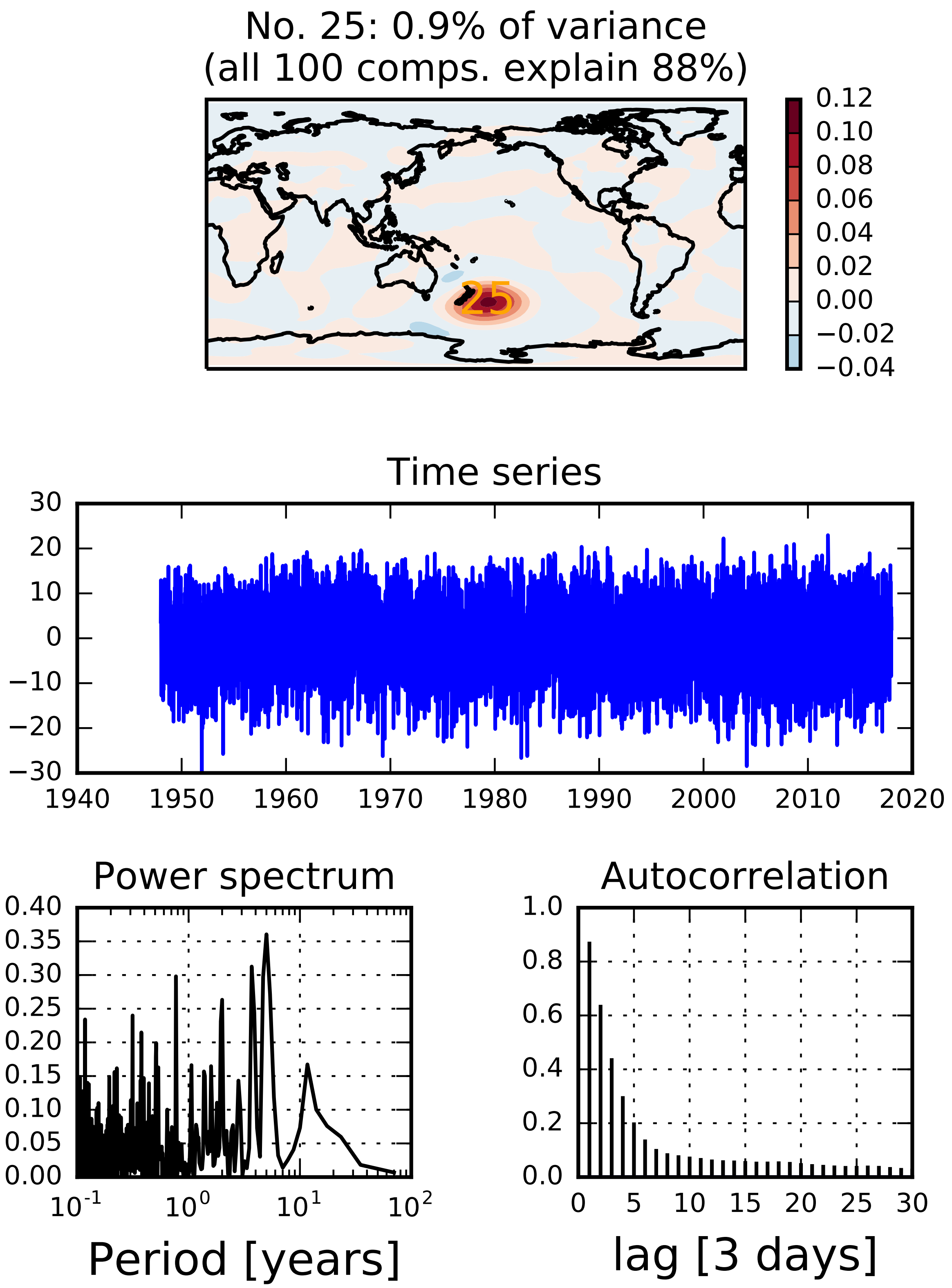
No. 25: 0.9% of variance
(all 100 comps. explain 88%)
25
0.12
0.10
0.08
0.06
0.04
0.02
0.00
−0.02
−0.04
Time series
30
20
10
0
−10
−20
−30
1940
1950
1960
1970
1980
1990
2000
2010
2020
Power spectrum
0.40
0.35
0.30
0.25
0.20
0.15
0.10
0.05
0.00
10^{-1}
10^{0}
10^{1}
10^{2}
Period [years]
Autocorrelation
1.0
0.8
0.6
0.4
0.2
0.0
0
5
10
15
20
25
30
lag [3 days]

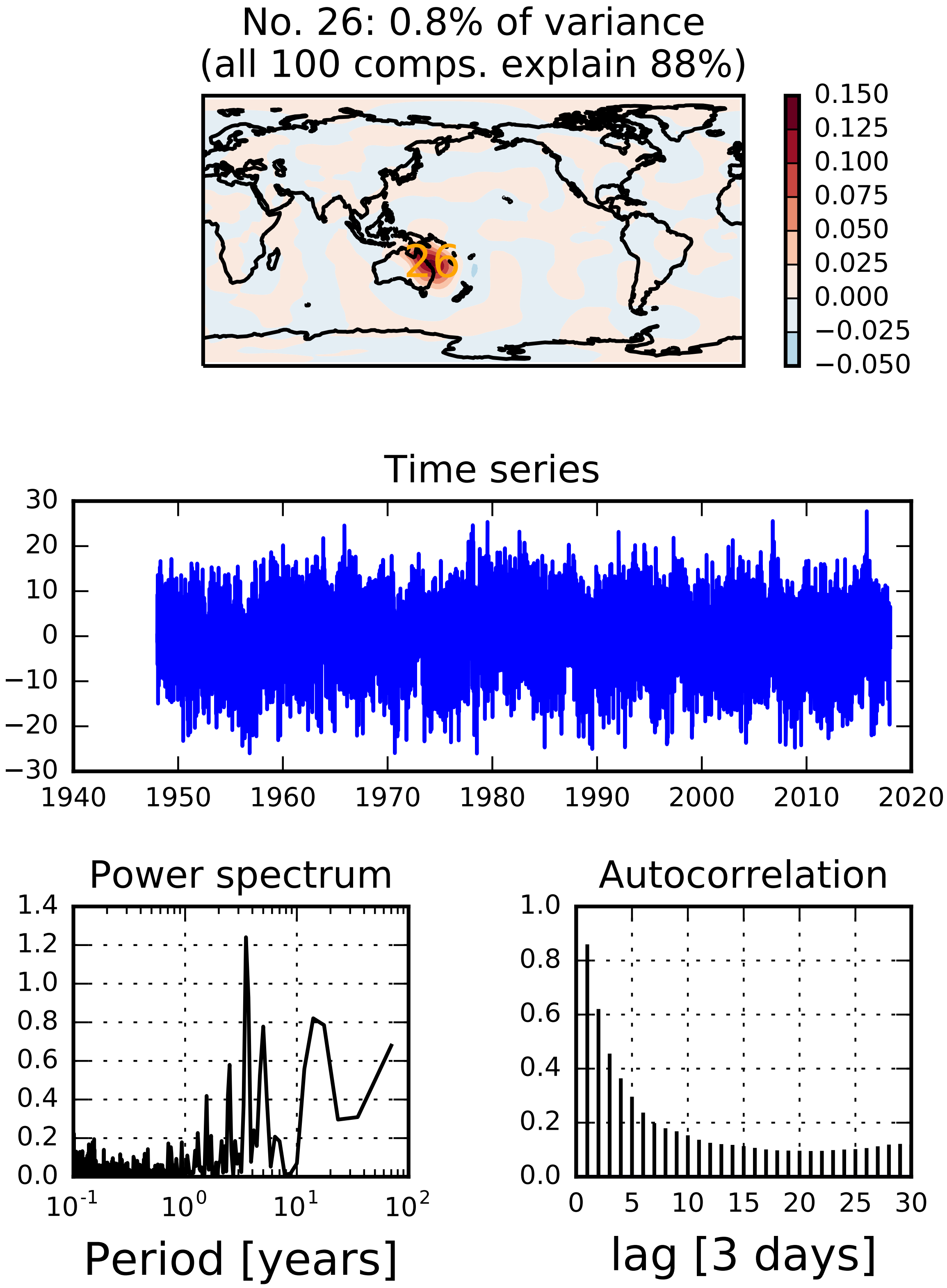
No. 26: 0.8% of variance
(all 100 comps. explain 88%)
26
0.150
0.125
0.100
0.075
0.050
0.025
0.000
−0.025
−0.050
Time series
30
20
10
0
−10
−20
−30
1940
1950
1960
1970
1980
1990
2000
2010
2020
Power spectrum
1.4
1.2
1.0
0.8
0.6
0.4
0.2
0.0
10^{-1}
10^{0}
10^{1}
10^{2}
Period [years]
Autocorrelation
1.0
0.8
0.6
0.4
0.2
0.0
0
5
10
15
20
25
30
lag [3 days]

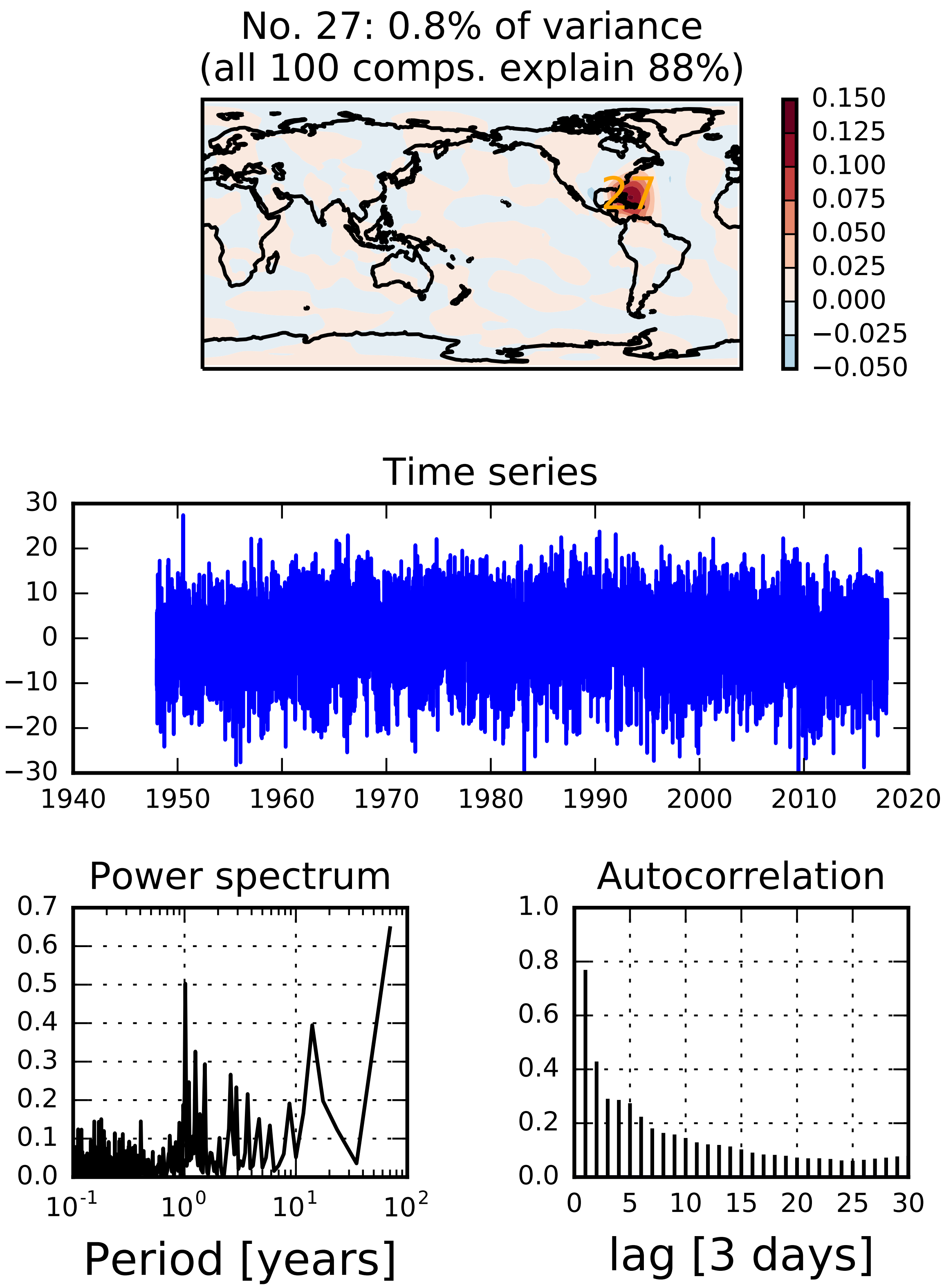
No. 27: 0.8% of variance
(all 100 comps. explain 88%)
27
0.150
0.125
0.100
0.075
0.050
0.025
0.000
−0.025
−0.050
Time series
30
20
10
0
−10
−20
−30
1940
1950
1960
1970
1980
1990
2000
2010
2020
Power spectrum
0.7
0.6
0.5
0.4
0.3
0.2
0.1
0.0
10^{-1}
10^{0}
10^{1}
10^{2}
Period [years]
Autocorrelation
1.0
0.8
0.6
0.4
0.2
0.0
0
5
10
15
20
25
30
lag [3 days]

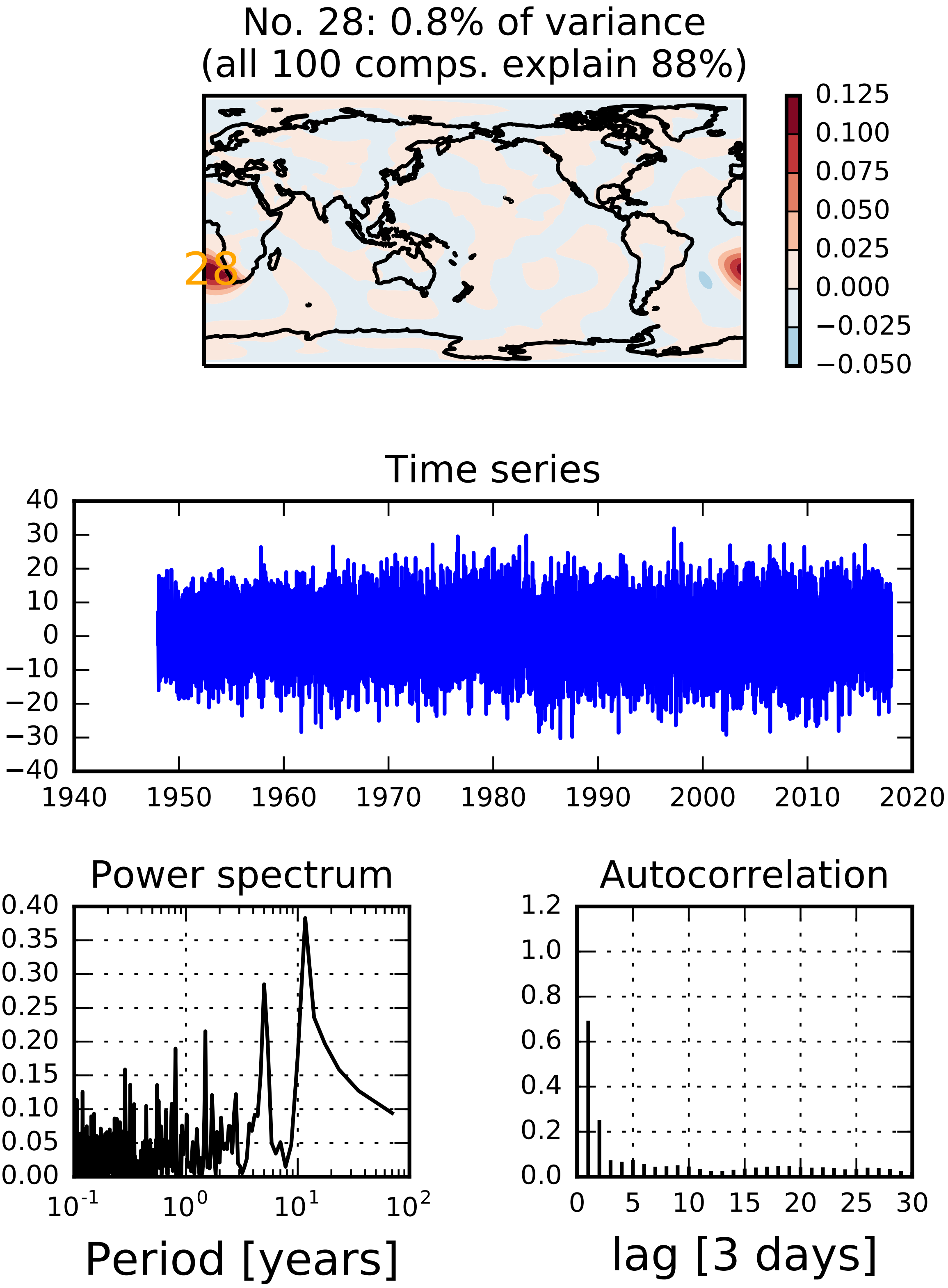
No. 28: 0.8% of variance
(all 100 comps. explain 88%)
28
0.125
0.100
0.075
0.050
0.025
0.000
−0.025
−0.050
Time series
40
30
20
10
0
−10
−20
−30
−40
1940
1950
1960
1970
1980
1990
2000
2010
2020
Power spectrum
0.40
0.35
0.30
0.25
0.20
0.15
0.10
0.05
0.00
10^{-1}
10^{0}
10^{1}
10^{2}
Period [years]
Autocorrelation
1.2
1.0
0.8
0.6
0.4
0.2
0.0
0
5
10
15
20
25
30
lag [3 days]

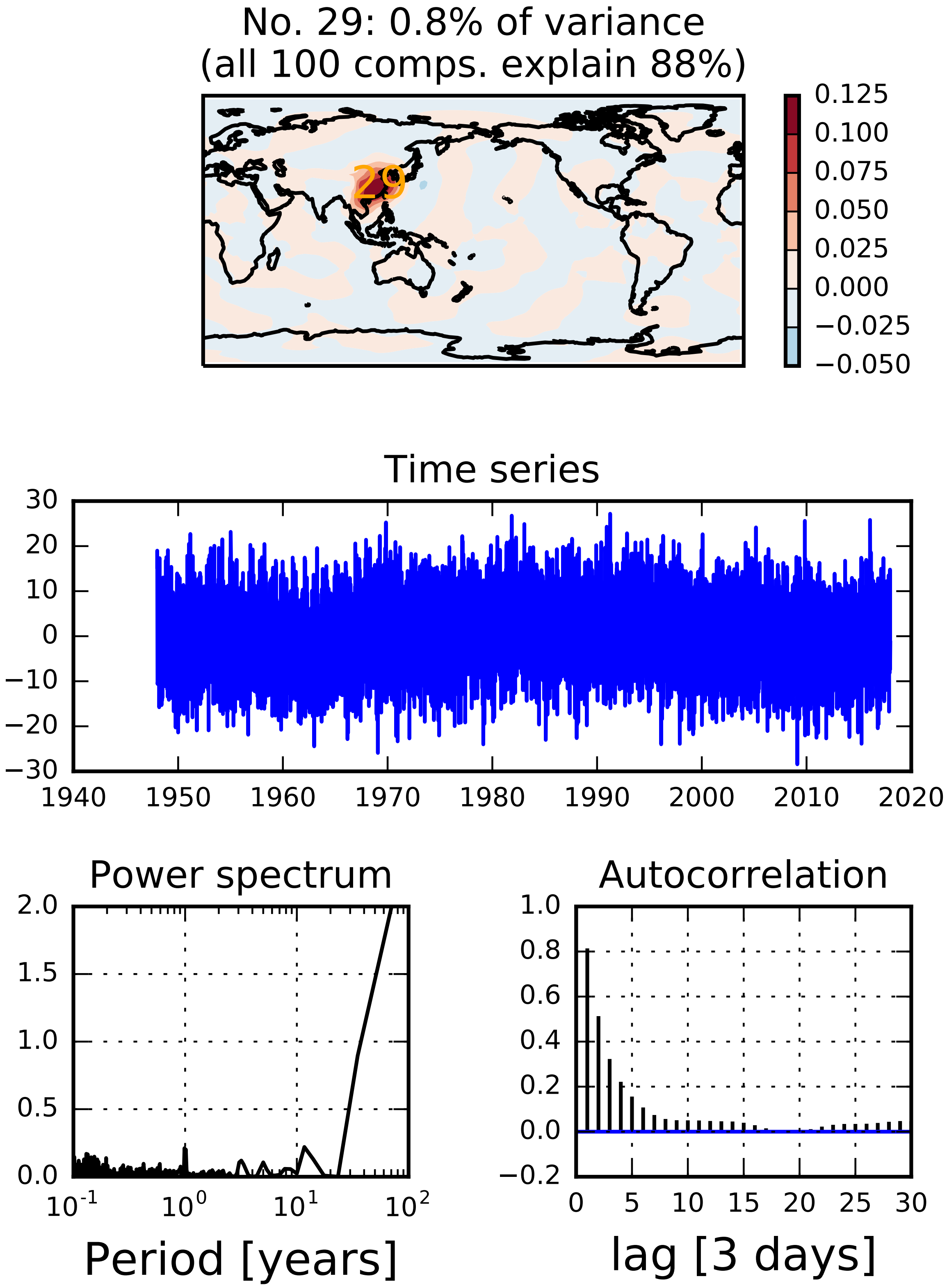
No. 29: 0.8% of variance
(all 100 comps. explain 88%)
29
0.125
0.100
0.075
0.050
0.025
0.000
−0.025
−0.050
Time series
30
20
10
0
−10
−20
−30
1940
1950
1960
1970
1980
1990
2000
2010
2020
Power spectrum
2.0
1.5
1.0
0.5
0.0
10^-1
10^0
10^1
10^2
Period [years]
Autocorrelation
1.0
0.8
0.6
0.4
0.2
0.0
−0.2
0
5
10
15
20
25
30
lag [3 days]

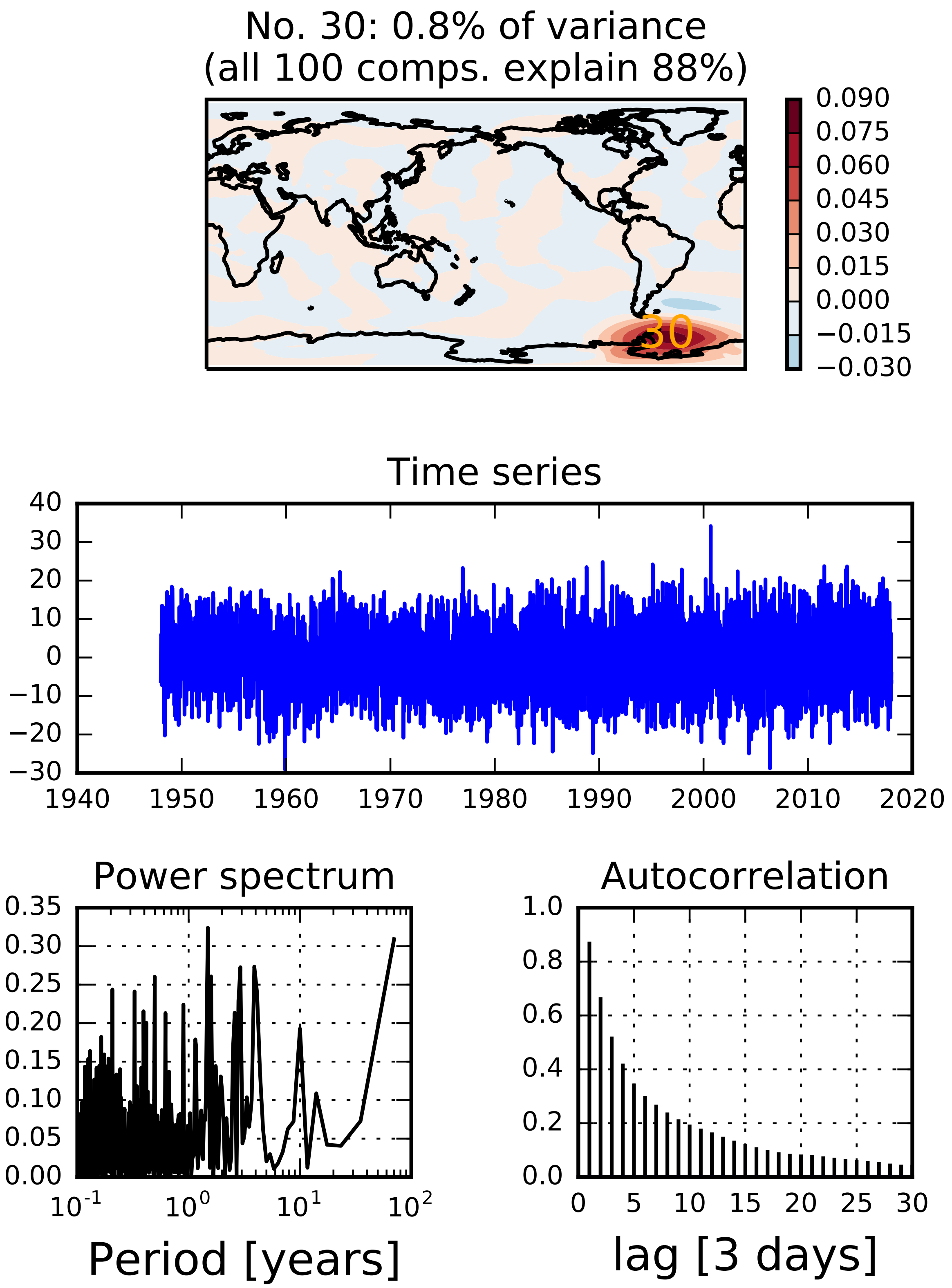
No. 30: 0.8% of variance
(all 100 comps. explain 88%)
0.090
0.075
0.060
0.045
0.030
0.015
0.000
−0.015
−0.030
30
Time series
40
30
20
10
0
−10
−20
−30
1940
1950
1960
1970
1980
1990
2000
2010
2020
Power spectrum
0.35
0.30
0.25
0.20
0.15
0.10
0.05
0.00
10^{-1}
10^{0}
10^{1}
10^{2}
Period [years]
Autocorrelation
1.0
0.8
0.6
0.4
0.2
0.0
0
5
10
15
20
25
30
lag [3 days]

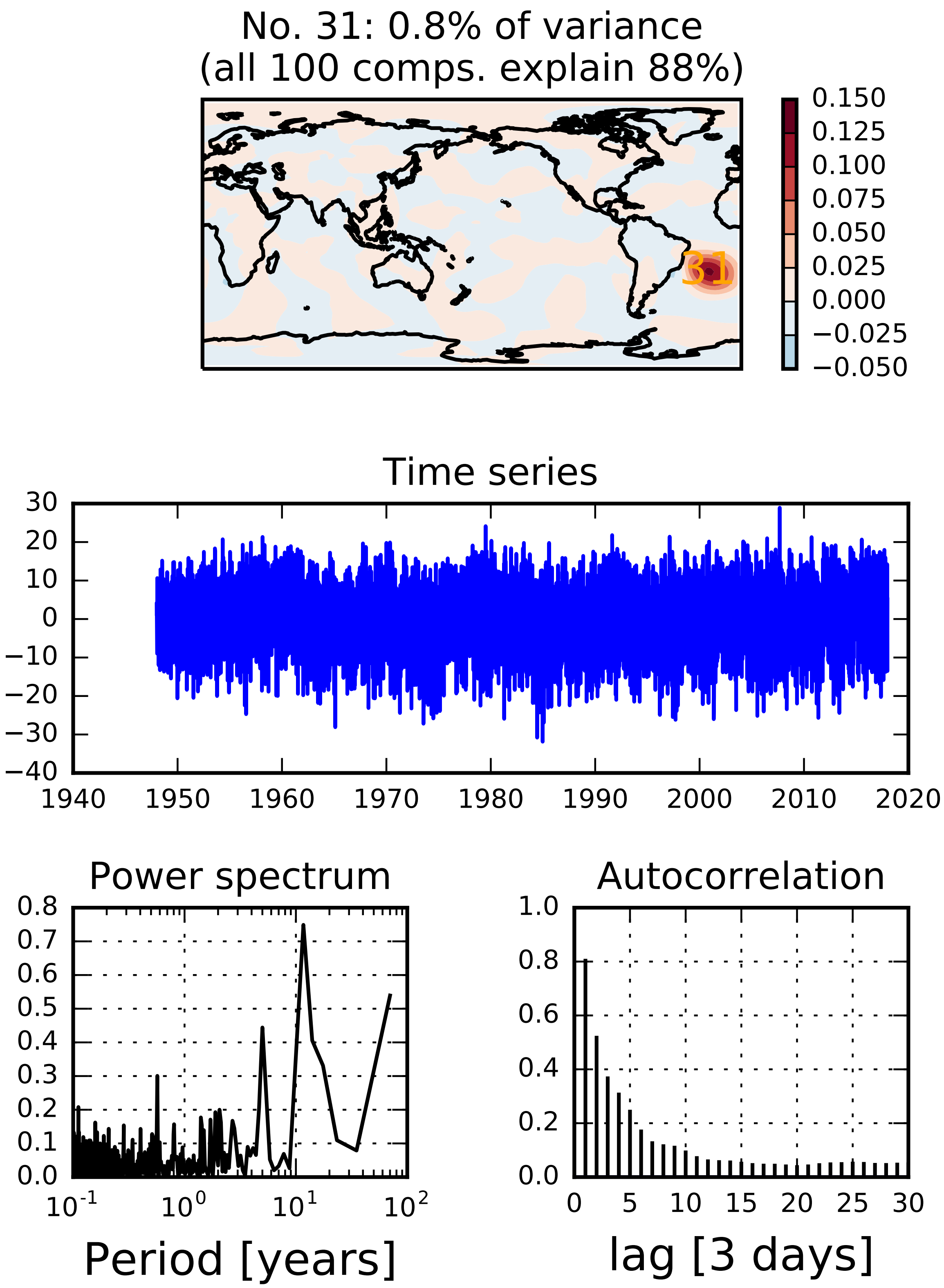
No. 31: 0.8% of variance
(all 100 comps. explain 88%)
31
0.150
0.125
0.100
0.075
0.050
0.025
0.000
−0.025
−0.050
Time series
30
20
10
0
−10
−20
−30
−40
1940
1950
1960
1970
1980
1990
2000
2010
2020
Power spectrum
0.8
0.7
0.6
0.5
0.4
0.3
0.2
0.1
0.0
10^{-1}
10^{0}
10^{1}
10^{2}
Period [years]
Autocorrelation
1.0
0.8
0.6
0.4
0.2
0.0
0
5
10
15
20
25
30
lag [3 days]

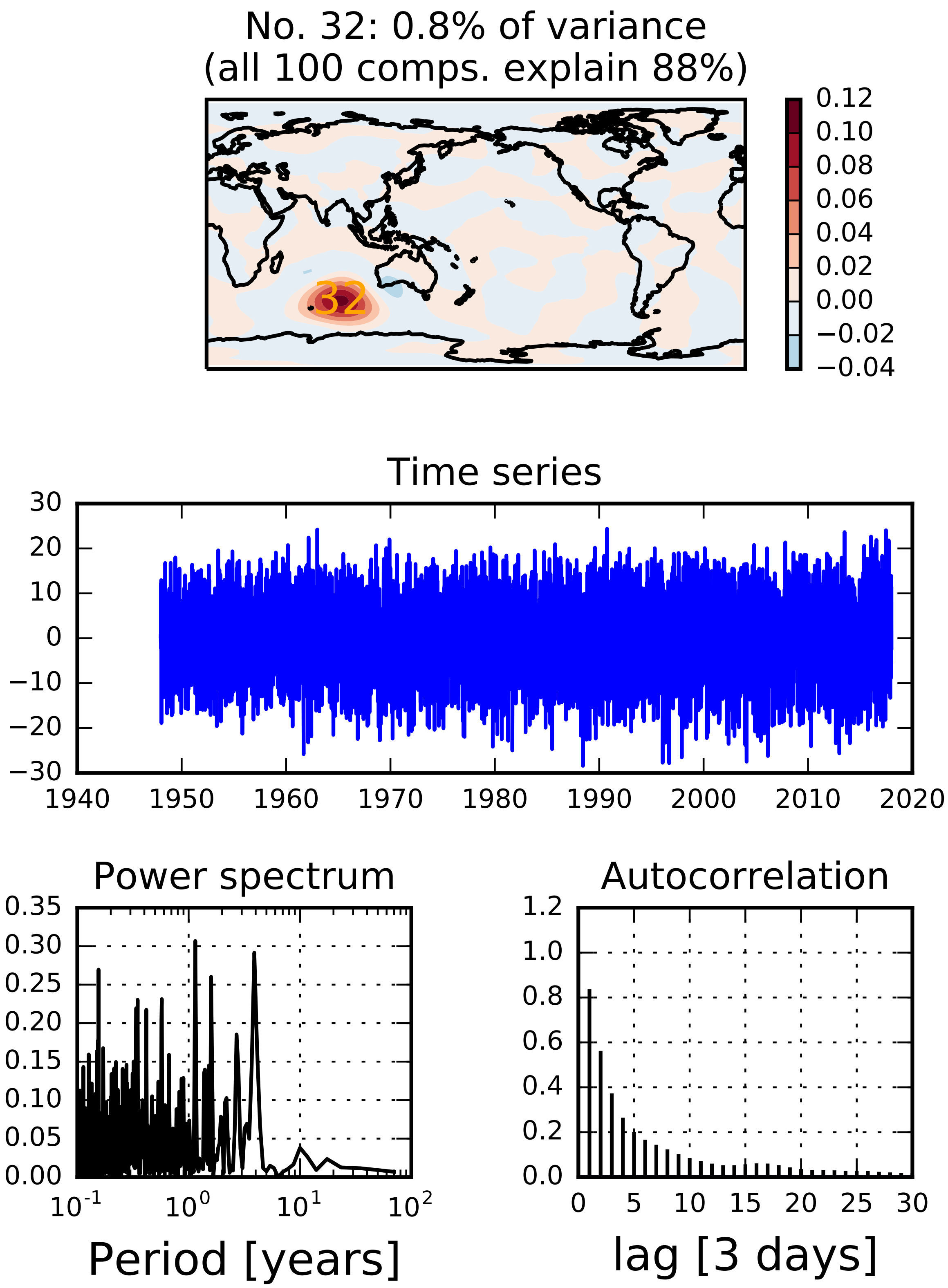
No. 32: 0.8% of variance
(all 100 comps. explain 88%)
32
0.12
0.10
0.08
0.06
0.04
0.02
0.00
−0.02
−0.04
Time series
30
20
10
0
−10
−20
−30
1940
1950
1960
1970
1980
1990
2000
2010
2020
Power spectrum
0.35
0.30
0.25
0.20
0.15
0.10
0.05
0.00
10^{-1}
10^{0}
10^{1}
10^{2}
Period [years]
Autocorrelation
1.2
1.0
0.8
0.6
0.4
0.2
0.0
0
5
10
15
20
25
30
lag [3 days]

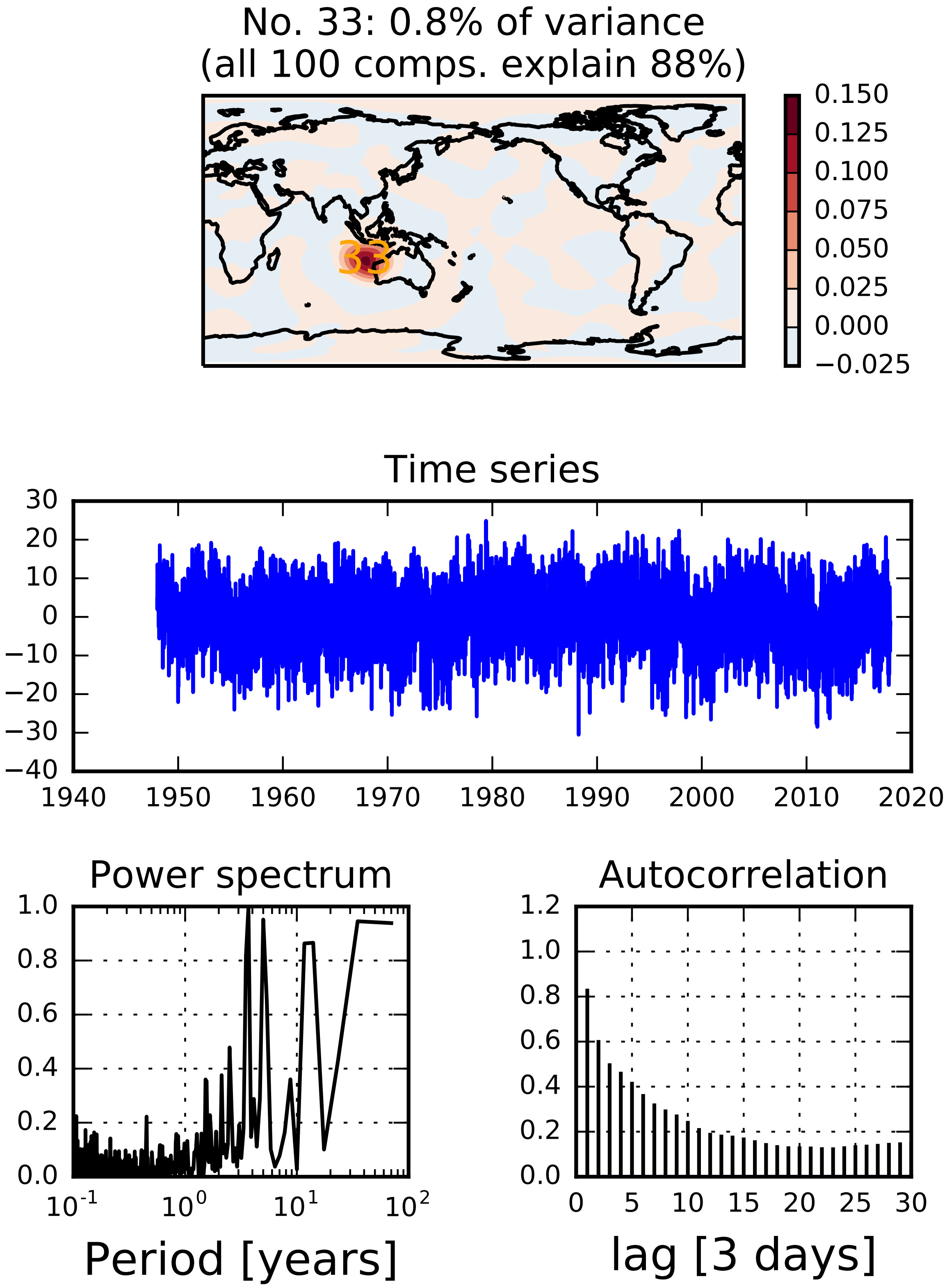
No. 33: 0.8% of variance
(all 100 comps. explain 88%)
33
0.150
0.125
0.100
0.075
0.050
0.025
0.000
−0.025
Time series
30
20
10
0
−10
−20
−30
−40
1940
1950
1960
1970
1980
1990
2000
2010
2020
Power spectrum
1.0
0.8
0.6
0.4
0.2
0.0
10^-1
10^0
10^1
10^2
Period [years]
Autocorrelation
1.2
1.0
0.8
0.6
0.4
0.2
0.0
0
5
10
15
20
25
30
lag [3 days]

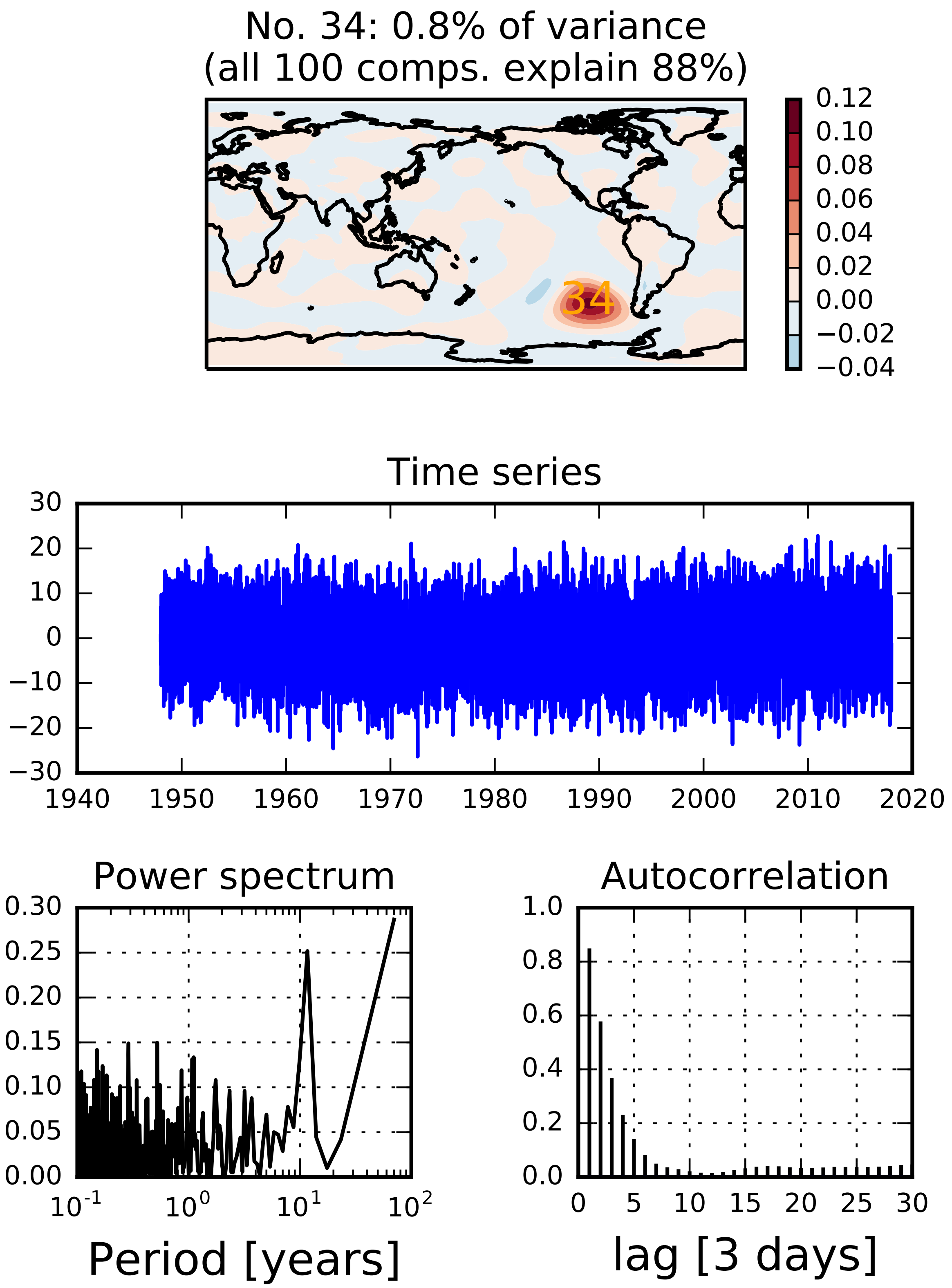
No. 34: 0.8% of variance
(all 100 comps. explain 88%)
34
0.12
0.10
0.08
0.06
0.04
0.02
0.00
−0.02
−0.04
Time series
30
20
10
0
−10
−20
−30
1940
1950
1960
1970
1980
1990
2000
2010
2020
Power spectrum
0.30
0.25
0.20
0.15
0.10
0.05
0.00
10^-1
10^0
10^1
10^2
Period [years]
Autocorrelation
1.0
0.8
0.6
0.4
0.2
0.0
0
5
10
15
20
25
30
lag [3 days]

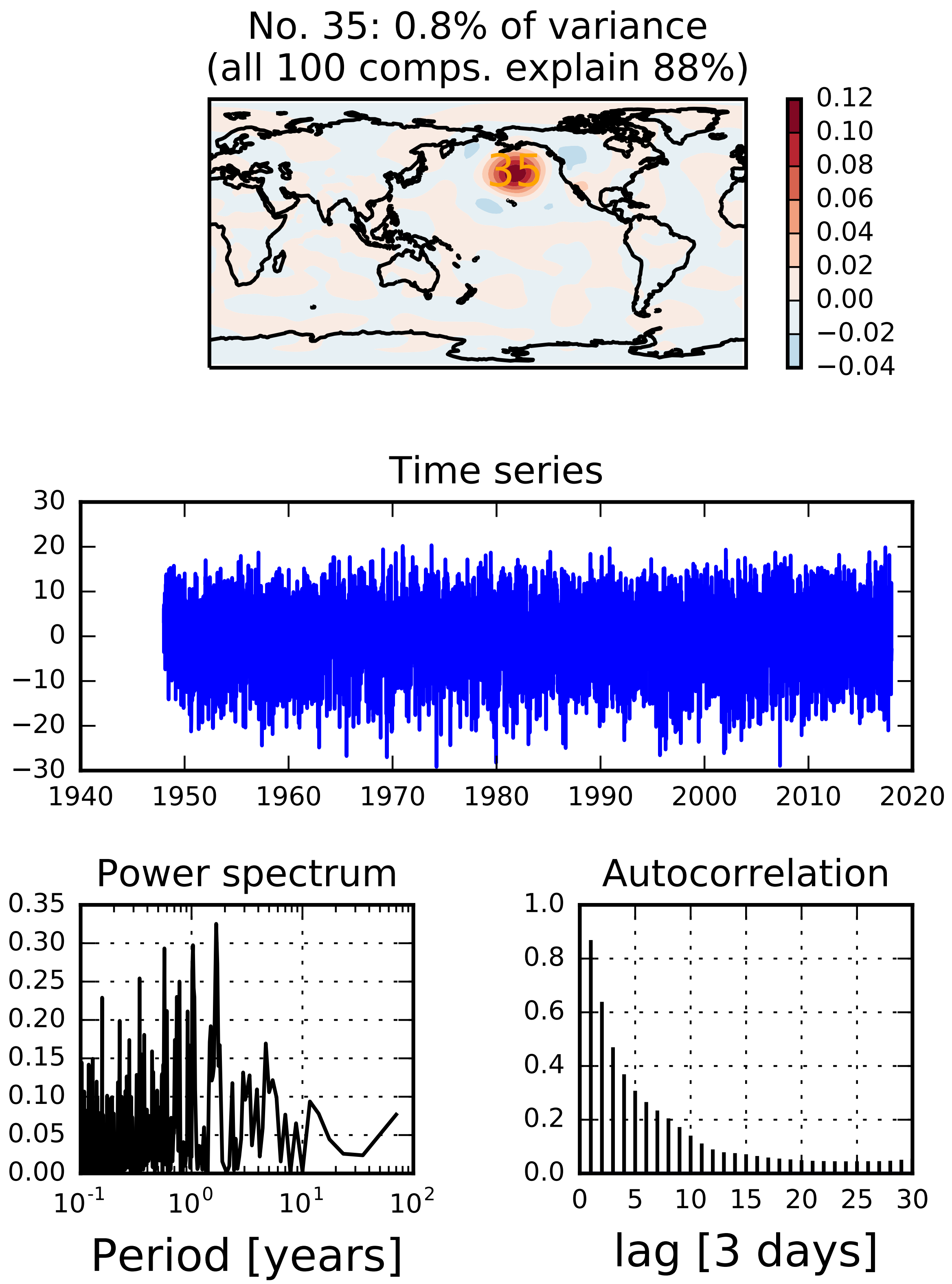
No. 35: 0.8% of variance
(all 100 comps. explain 88%)
35
0.12
0.10
0.08
0.06
0.04
0.02
0.00
−0.02
−0.04
Time series
30
20
10
0
−10
−20
−30
1940
1950
1960
1970
1980
1990
2000
2010
2020
Power spectrum
0.35
0.30
0.25
0.20
0.15
0.10
0.05
0.00
10^{-1}
10^{0}
10^{1}
10^{2}
Period [years]
Autocorrelation
1.0
0.8
0.6
0.4
0.2
0.0
0
5
10
15
20
25
30
lag [3 days]

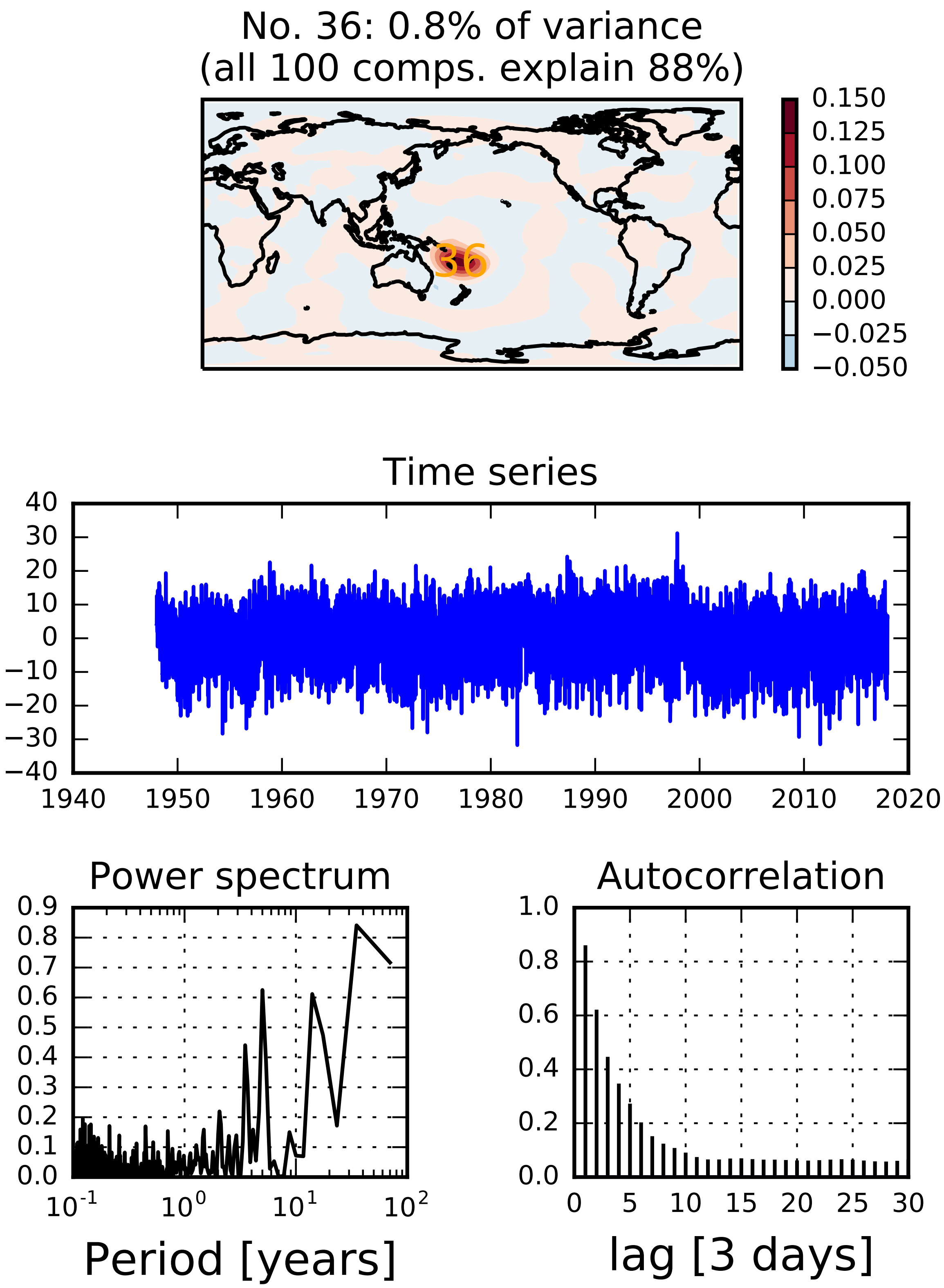
No. 36: 0.8% of variance
(all 100 comps. explain 88%)
36
0.150
0.125
0.100
0.075
0.050
0.025
0.000
−0.025
−0.050
Time series
40
30
20
10
0
−10
−20
−30
−40
1940
1950
1960
1970
1980
1990
2000
2010
2020
Power spectrum
0.9
0.8
0.7
0.6
0.5
0.4
0.3
0.2
0.1
0.0
10^-1
10^0
10^1
10^2
Period [years]
Autocorrelation
1.0
0.8
0.6
0.4
0.2
0.0
0
5
10
15
20
25
30
lag [3 days]

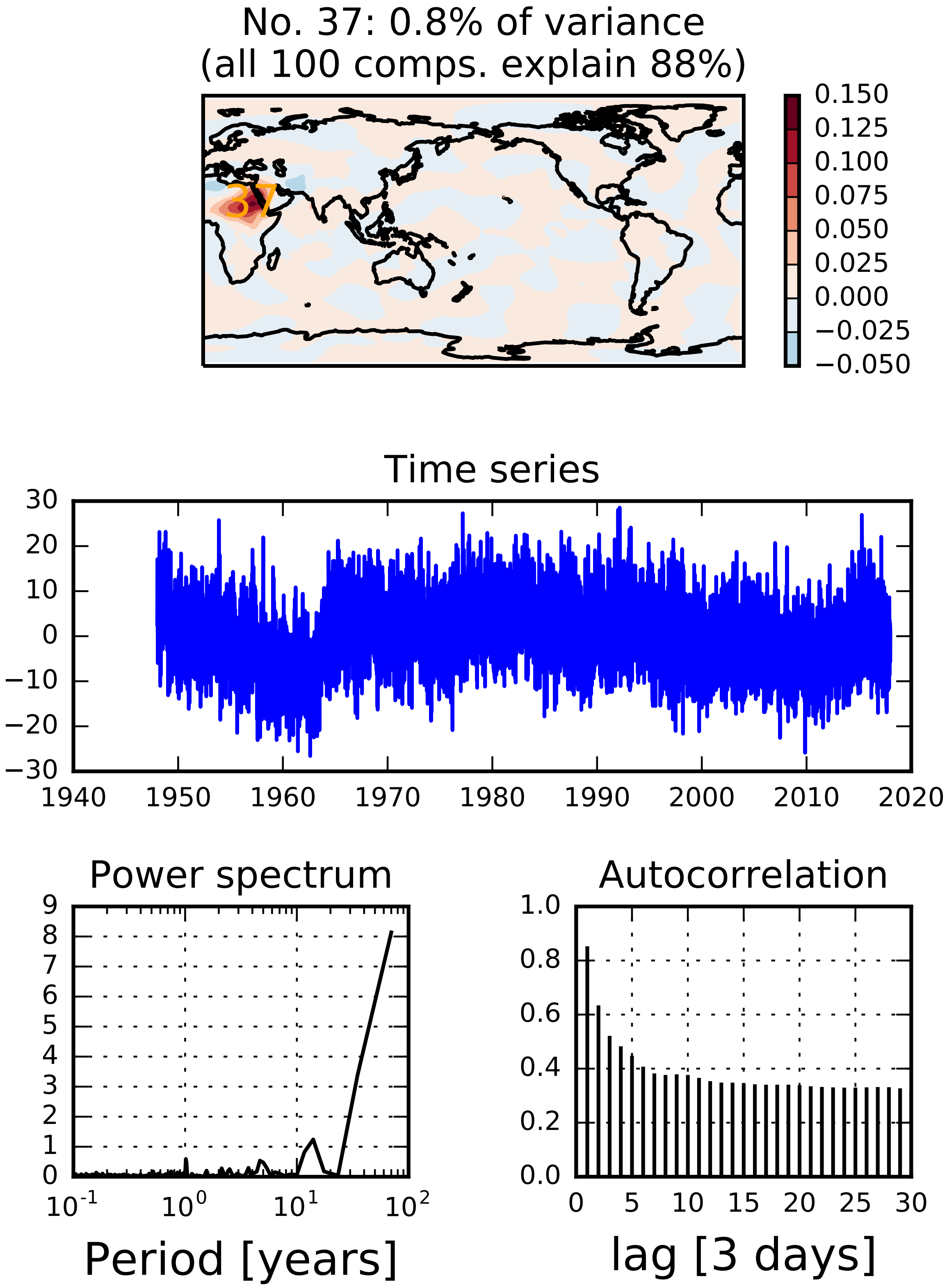
No. 37: 0.8% of variance
(all 100 comps. explain 88%)
37
0.150
0.125
0.100
0.075
0.050
0.025
0.000
−0.025
−0.050
Time series
30
20
10
0
−10
−20
−30
1940
1950
1960
1970
1980
1990
2000
2010
2020
Power spectrum
9
8
7
6
5
4
3
2
1
0
10^{-1}
10^{0}
10^{1}
10^{2}
Period [years]
Autocorrelation
1.0
0.8
0.6
0.4
0.2
0.0
0
5
10
15
20
25
30
lag [3 days]

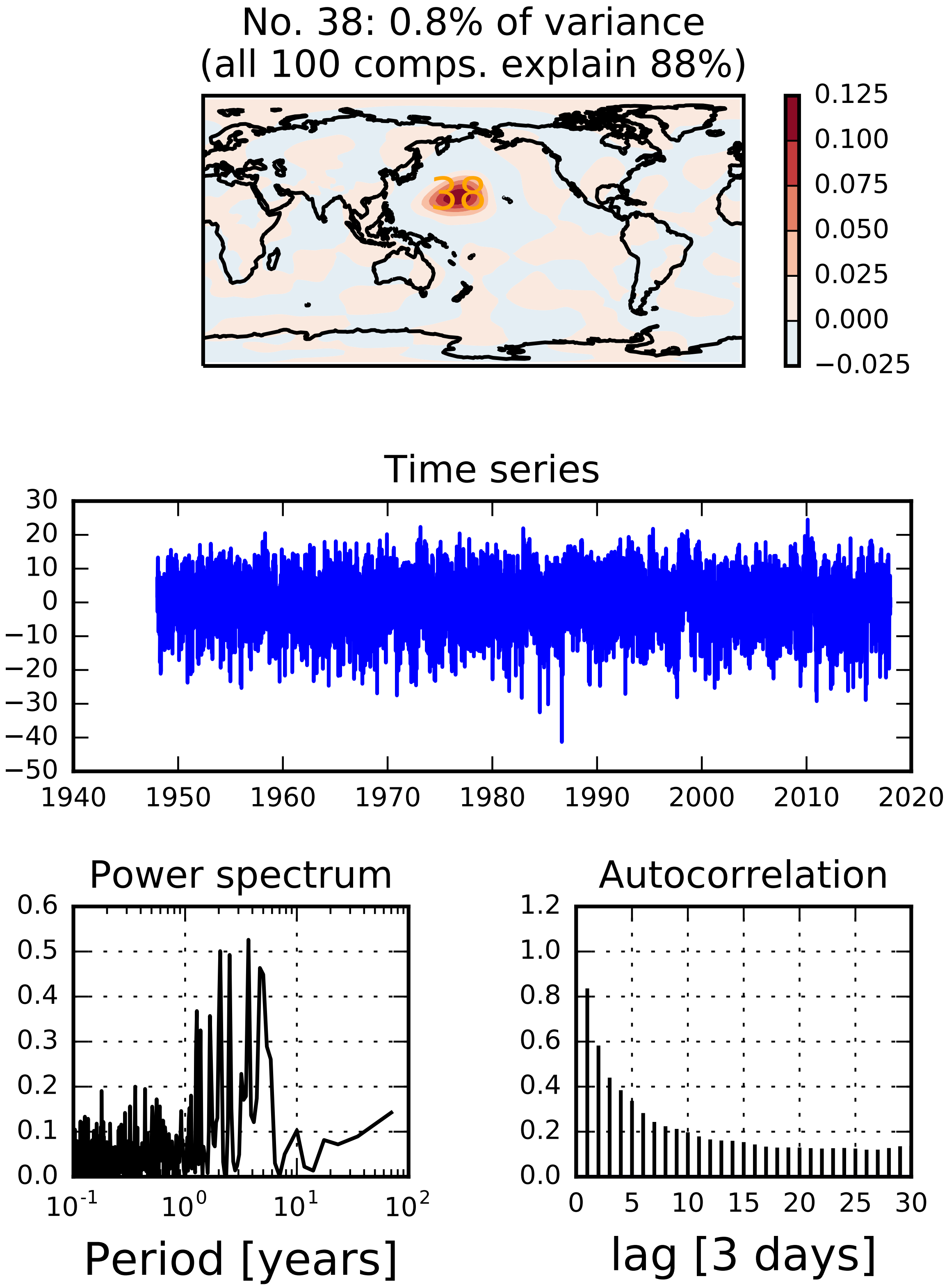
No. 38: 0.8% of variance
(all 100 comps. explain 88%)
38
0.125
0.100
0.075
0.050
0.025
0.000
−0.025
Time series
30
20
10
0
−10
−20
−30
−40
−50
1940
1950
1960
1970
1980
1990
2000
2010
2020
Power spectrum
0.6
0.5
0.4
0.3
0.2
0.1
0.0
10^{-1}
10^{0}
10^{1}
10^{2}
Period [years]
Autocorrelation
1.2
1.0
0.8
0.6
0.4
0.2
0.0
0
5
10
15
20
25
30
lag [3 days]

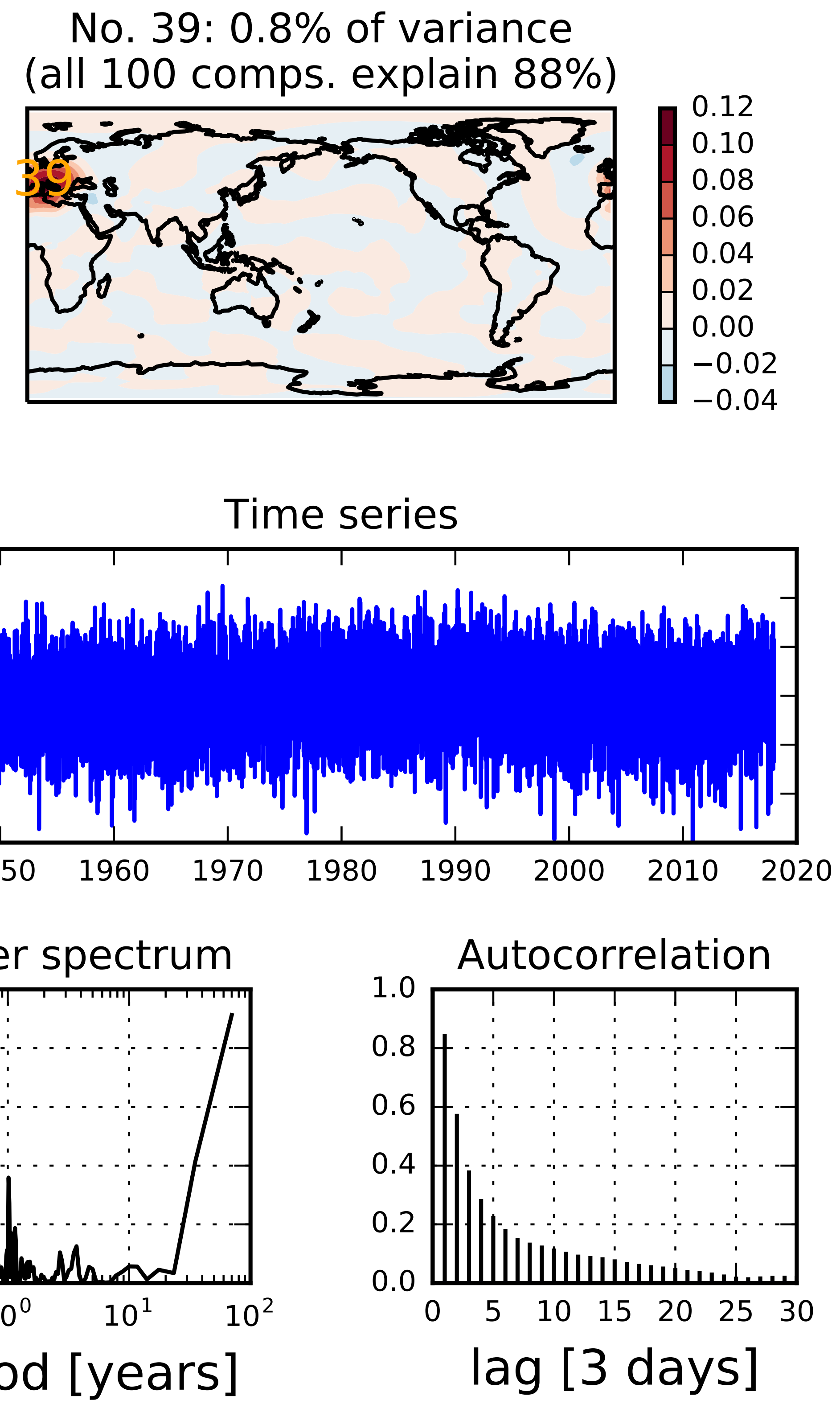
No. 39: 0.8% of variance
(all 100 comps. explain 88%)
39
0.12
0.10
0.08
0.06
0.04
0.02
0.00
−0.02
−0.04
Time series
30
20
10
0
−10
−20
−30
1940
1950
1960
1970
1980
1990
2000
2010
2020
Power spectrum
1.0
0.8
0.6
0.4
0.2
0.0
10^-1
10^0
10^1
10^2
Period [years]
Autocorrelation
1.0
0.8
0.6
0.4
0.2
0.0
0
5
10
15
20
25
30
lag [3 days]

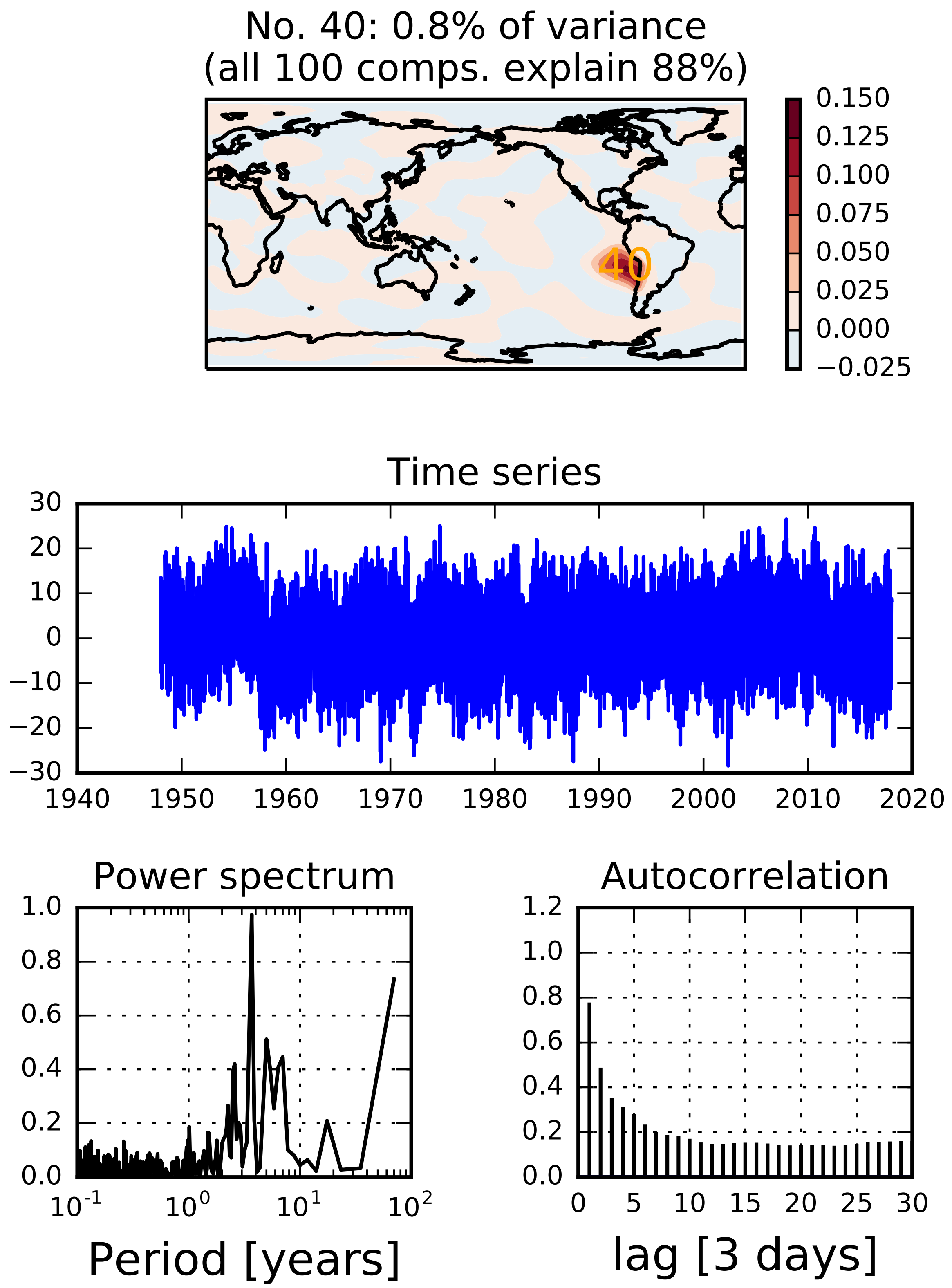
No. 40: 0.8% of variance
(all 100 comps. explain 88%)
40
0.150
0.125
0.100
0.075
0.050
0.025
0.000
−0.025
Time series
30
20
10
0
−10
−20
−30
1940
1950
1960
1970
1980
1990
2000
2010
2020
Power spectrum
1.0
0.8
0.6
0.4
0.2
0.0
10^{-1}
10^{0}
10^{1}
10^{2}
Period [years]
Autocorrelation
1.2
1.0
0.8
0.6
0.4
0.2
0.0
0
5
10
15
20
25
30
lag [3 days]

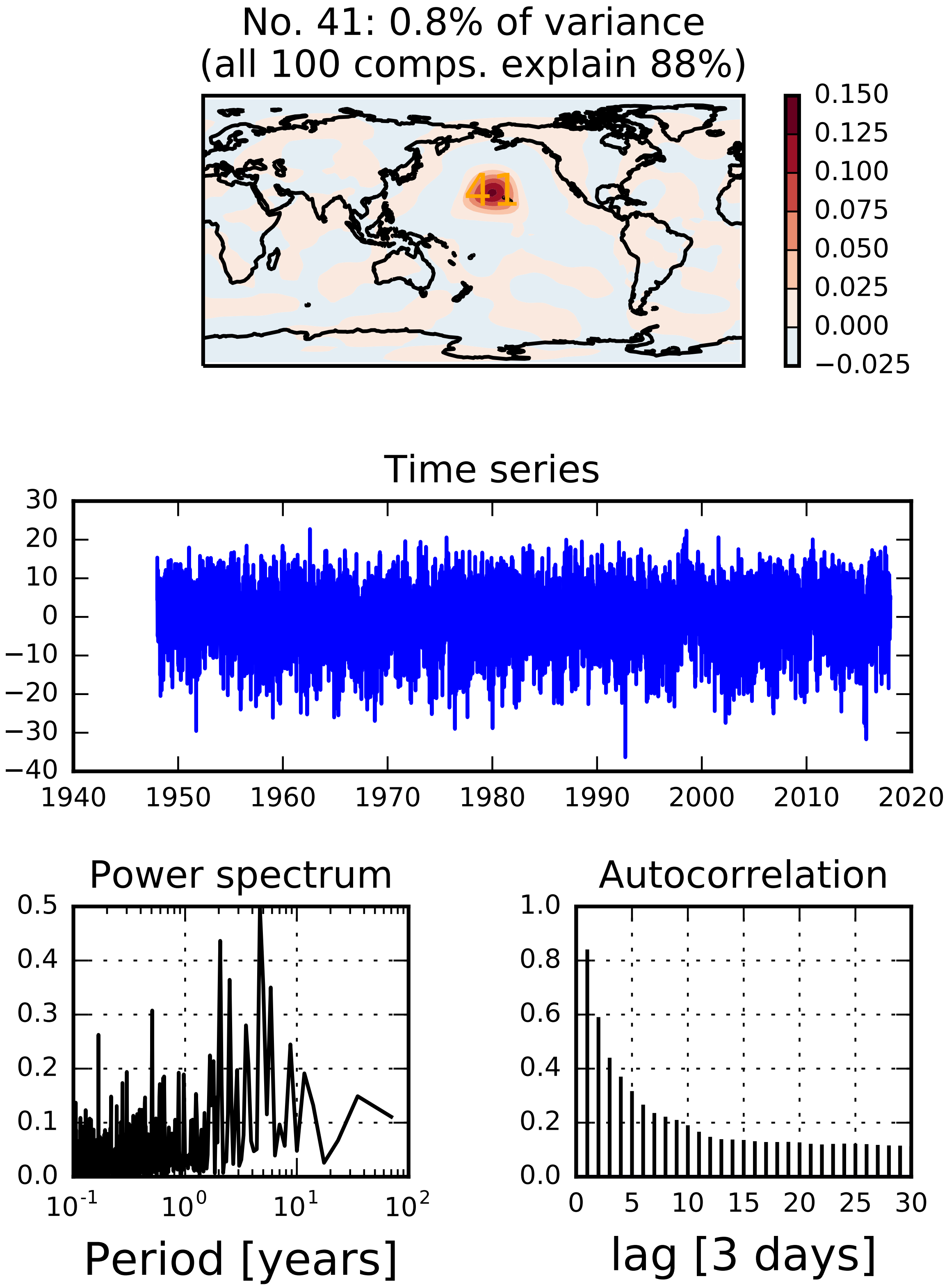
No. 41: 0.8% of variance
(all 100 comps. explain 88%)
41
0.150
0.125
0.100
0.075
0.050
0.025
0.000
−0.025
Time series
30
20
10
0
−10
−20
−30
−40
1940
1950
1960
1970
1980
1990
2000
2010
2020
Power spectrum
0.5
0.4
0.3
0.2
0.1
0.0
10^-1
10^0
10^1
10^2
Period [years]
Autocorrelation
1.0
0.8
0.6
0.4
0.2
0.0
0
5
10
15
20
25
30
lag [3 days]

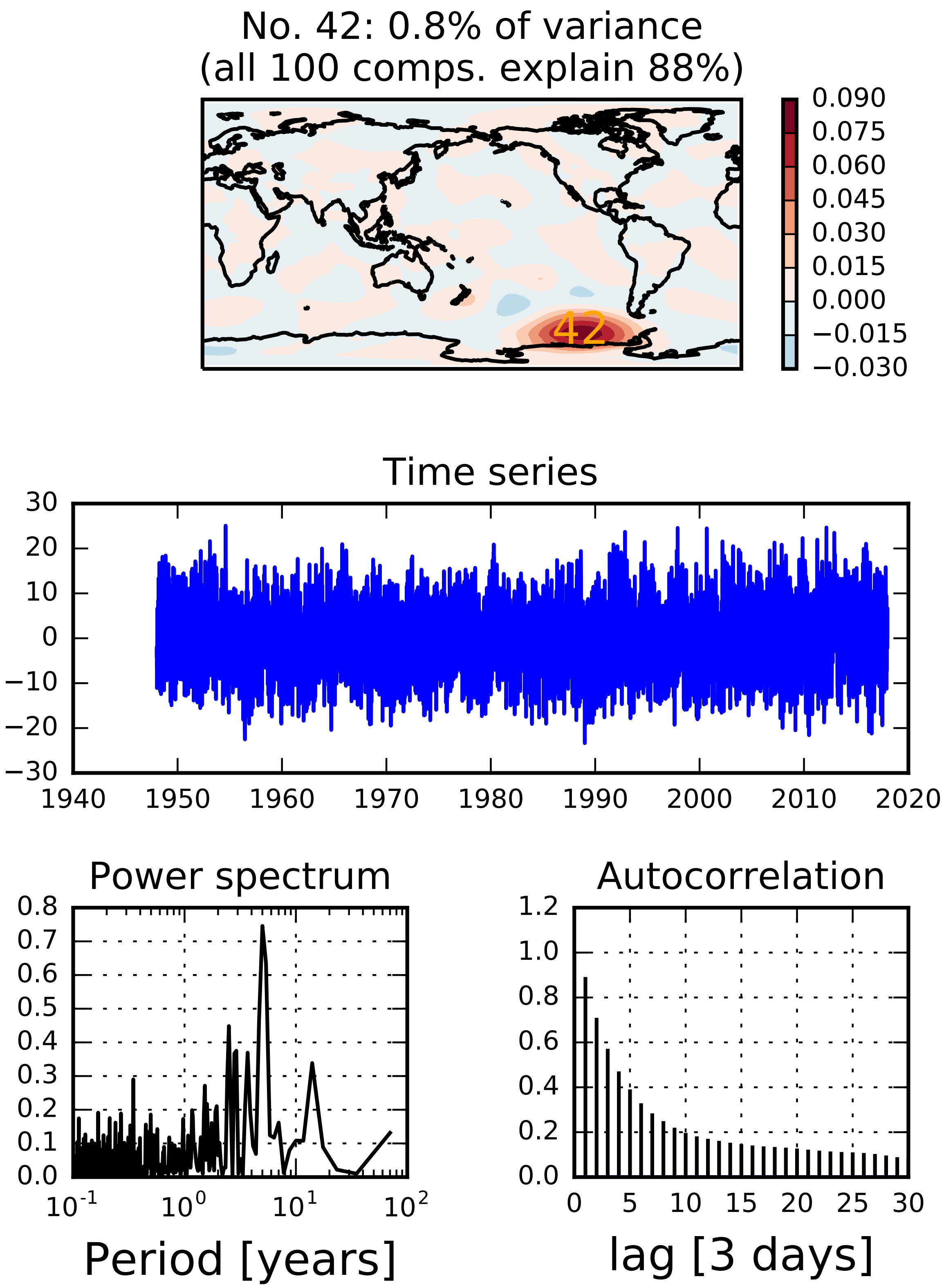
No. 42: 0.8% of variance
(all 100 comps. explain 88%)
42
0.090
0.075
0.060
0.045
0.030
0.015
0.000
−0.015
−0.030
Time series
30
20
10
0
−10
−20
−30
1940
1950
1960
1970
1980
1990
2000
2010
2020
Power spectrum
Period [years]
Autocorrelation
lag [3 days]

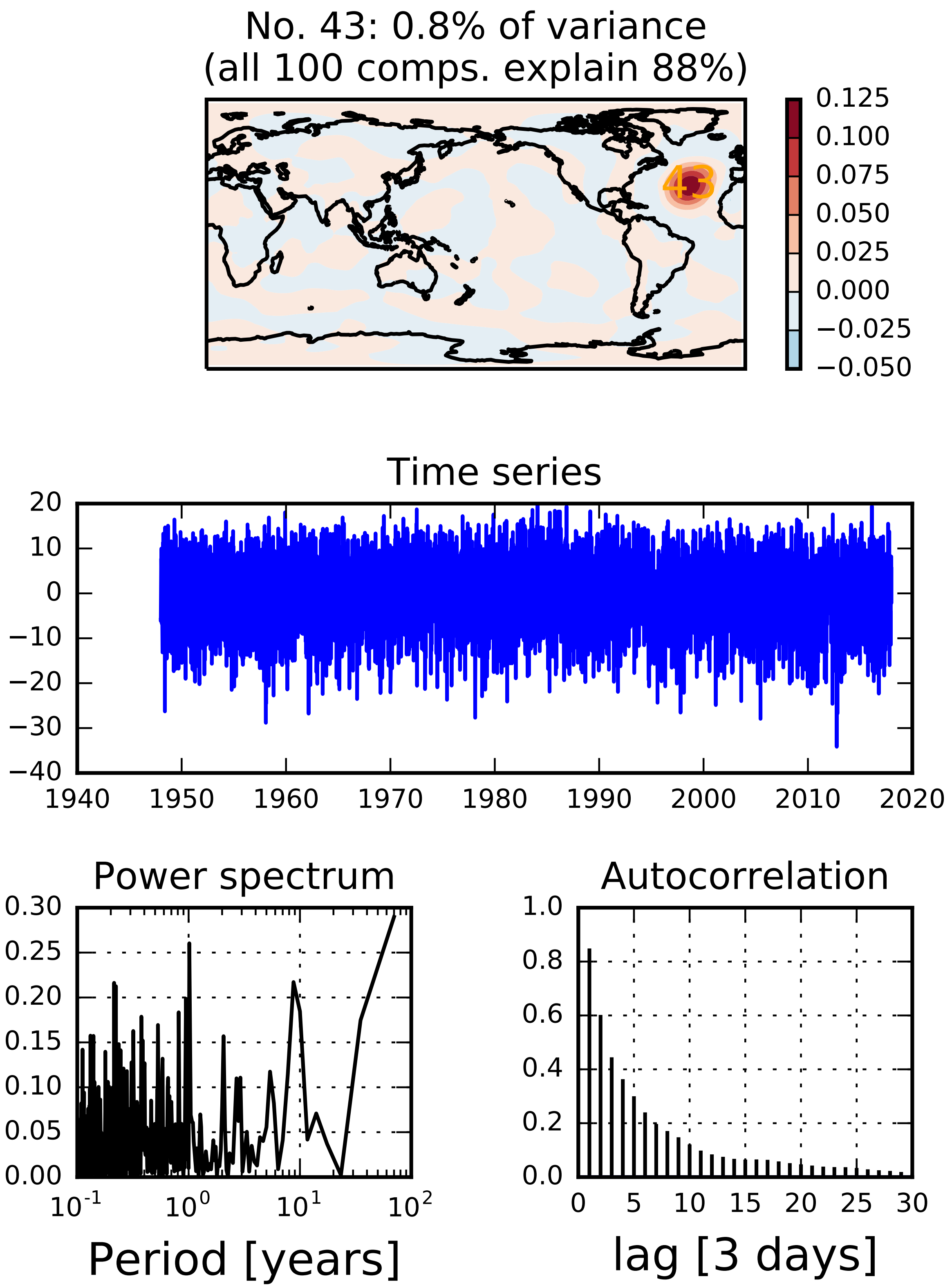
No. 43: 0.8% of variance
(all 100 comps. explain 88%)
43
0.125
0.100
0.075
0.050
0.025
0.000
−0.025
−0.050
Time series
20
10
0
−10
−20
−30
−40
1940
1950
1960
1970
1980
1990
2000
2010
2020
Power spectrum
0.30
0.25
0.20
0.15
0.10
0.05
0.00
10^{-1}
10^{0}
10^{1}
10^{2}
Period [years]
Autocorrelation
1.0
0.8
0.6
0.4
0.2
0.0
0
5
10
15
20
25
30
lag [3 days]

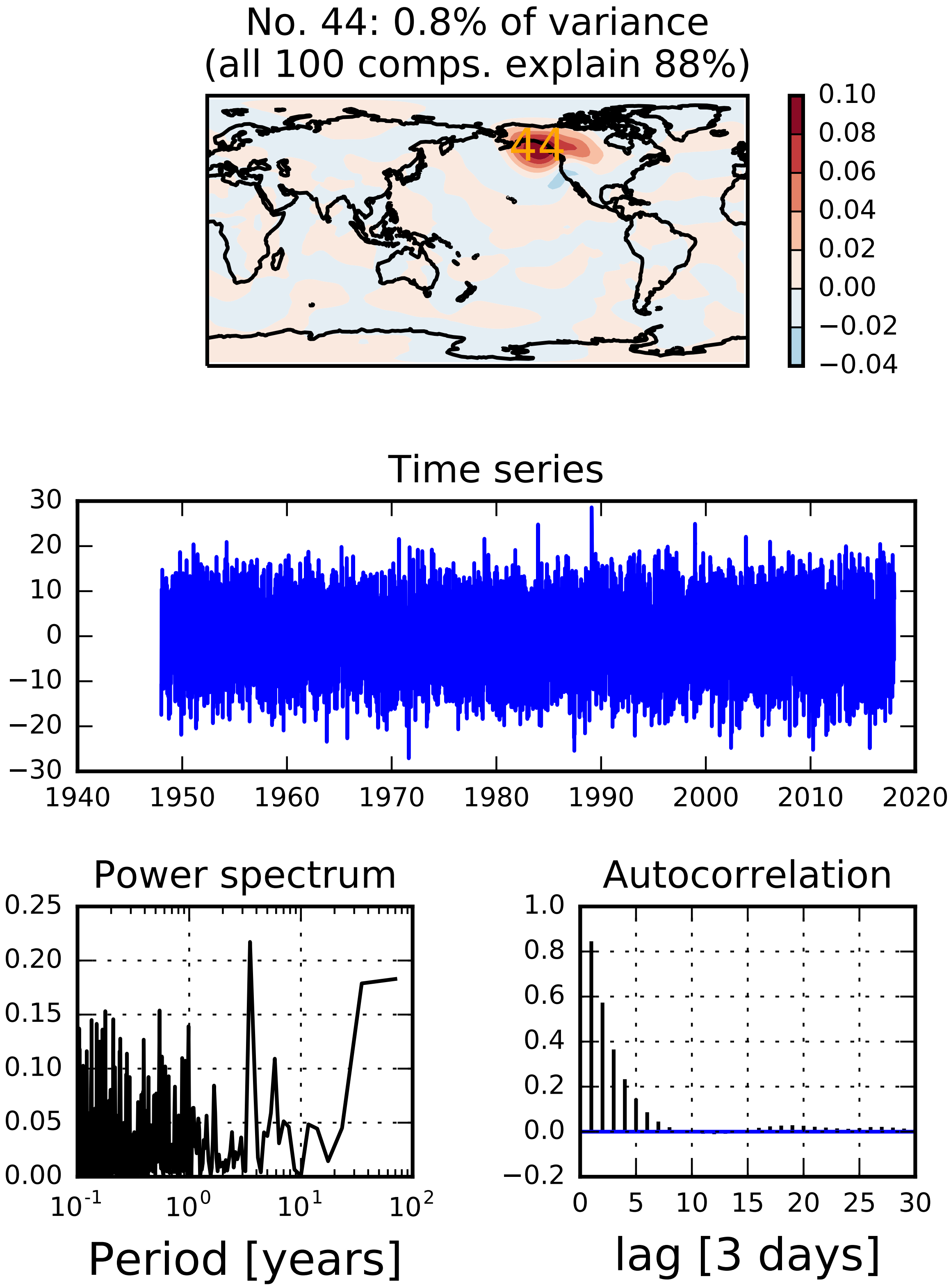
No. 44: 0.8% of variance
(all 100 comps. explain 88%)
44
0.10
0.08
0.06
0.04
0.02
0.00
−0.02
−0.04
Time series
30
20
10
0
−10
−20
−30
1940
1950
1960
1970
1980
1990
2000
2010
2020
Power spectrum
0.25
0.20
0.15
0.10
0.05
0.00
10^{-1}
10^{0}
10^{1}
10^{2}
Period [years]
Autocorrelation
1.0
0.8
0.6
0.4
0.2
0.0
−0.2
0
5
10
15
20
25
30
lag [3 days]

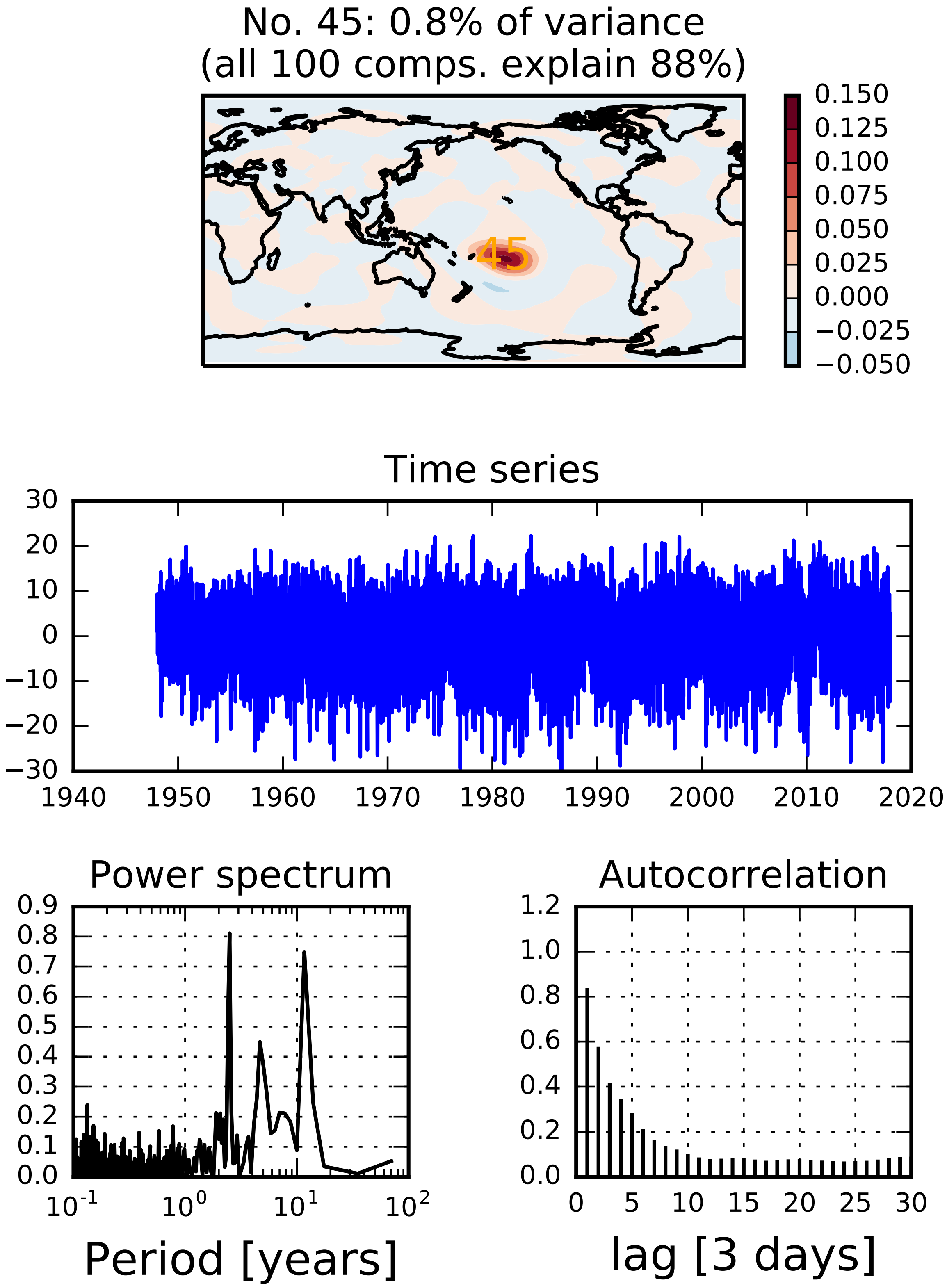

No. 45: 0.8% of variance
(all 100 comps. explain 88%)
45
0.150
0.125
0.100
0.075
0.050
0.025
0.000
−0.025
−0.050
Time series
30
20
10
0
−10
−20
−30
1940
1950
1960
1970
1980
1990
2000
2010
2020
Power spectrum
0.9
0.8
0.7
0.6
0.5
0.4
0.3
0.2
0.1
0.0
10^{-1}
10^{0}
10^{1}
10^{2}
Period [years]
Autocorrelation
1.2
1.0
0.8
0.6
0.4
0.2
0.0
0
5
10
15
20
25
30
lag [3 days]

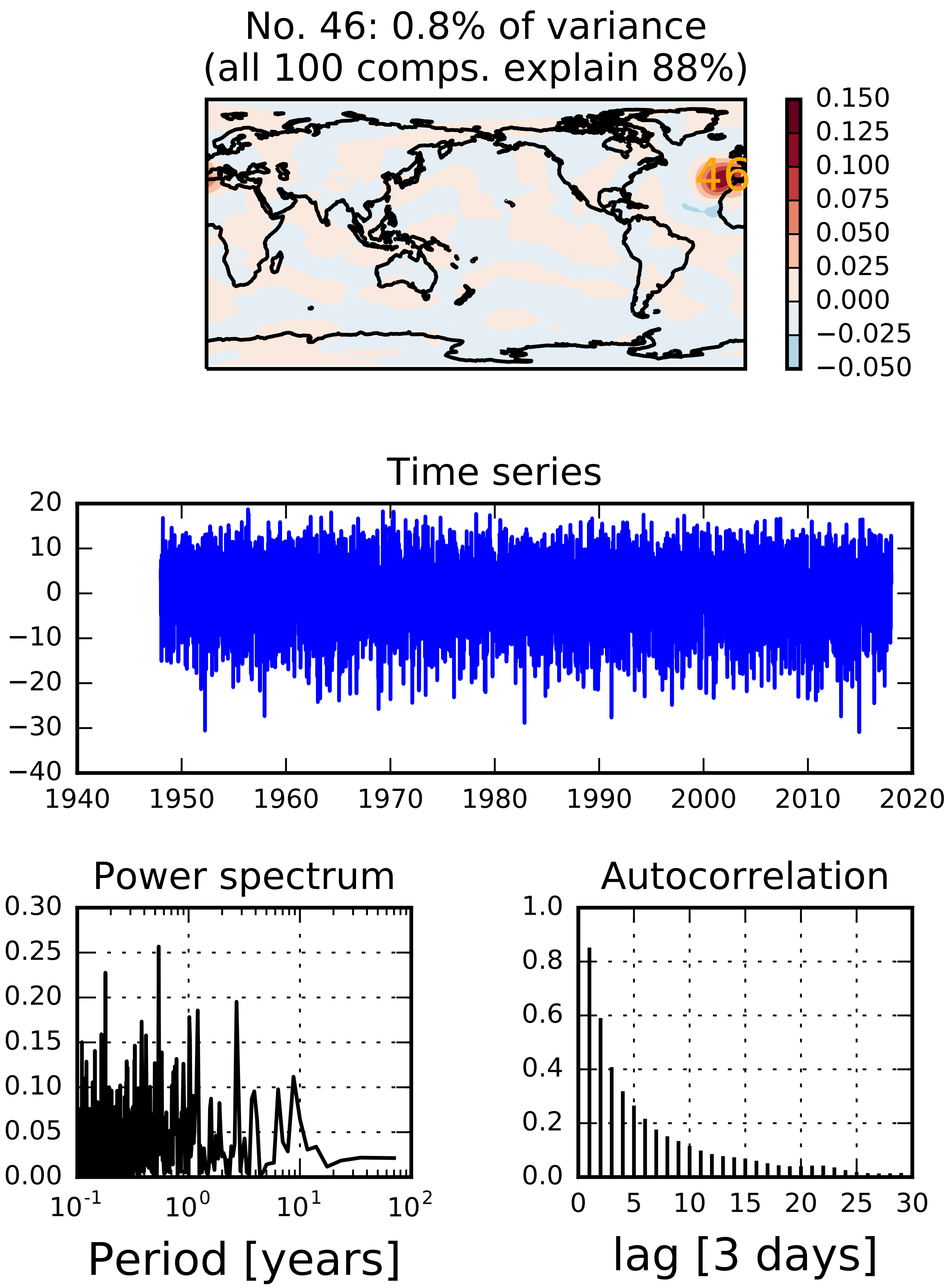
No. 46: 0.8% of variance
(all 100 comps. explain 88%)
46
0.150
0.125
0.100
0.075
0.050
0.025
0.000
−0.025
−0.050
Time series
20
10
0
−10
−20
−30
−40
1940
1950
1960
1970
1980
1990
2000
2010
2020
Power spectrum
0.30
0.25
0.20
0.15
0.10
0.05
0.00
10^{-1}
10^{0}
10^{1}
10^{2}
Period [years]
Autocorrelation
1.0
0.8
0.6
0.4
0.2
0.0
0
5
10
15
20
25
30
lag [3 days]

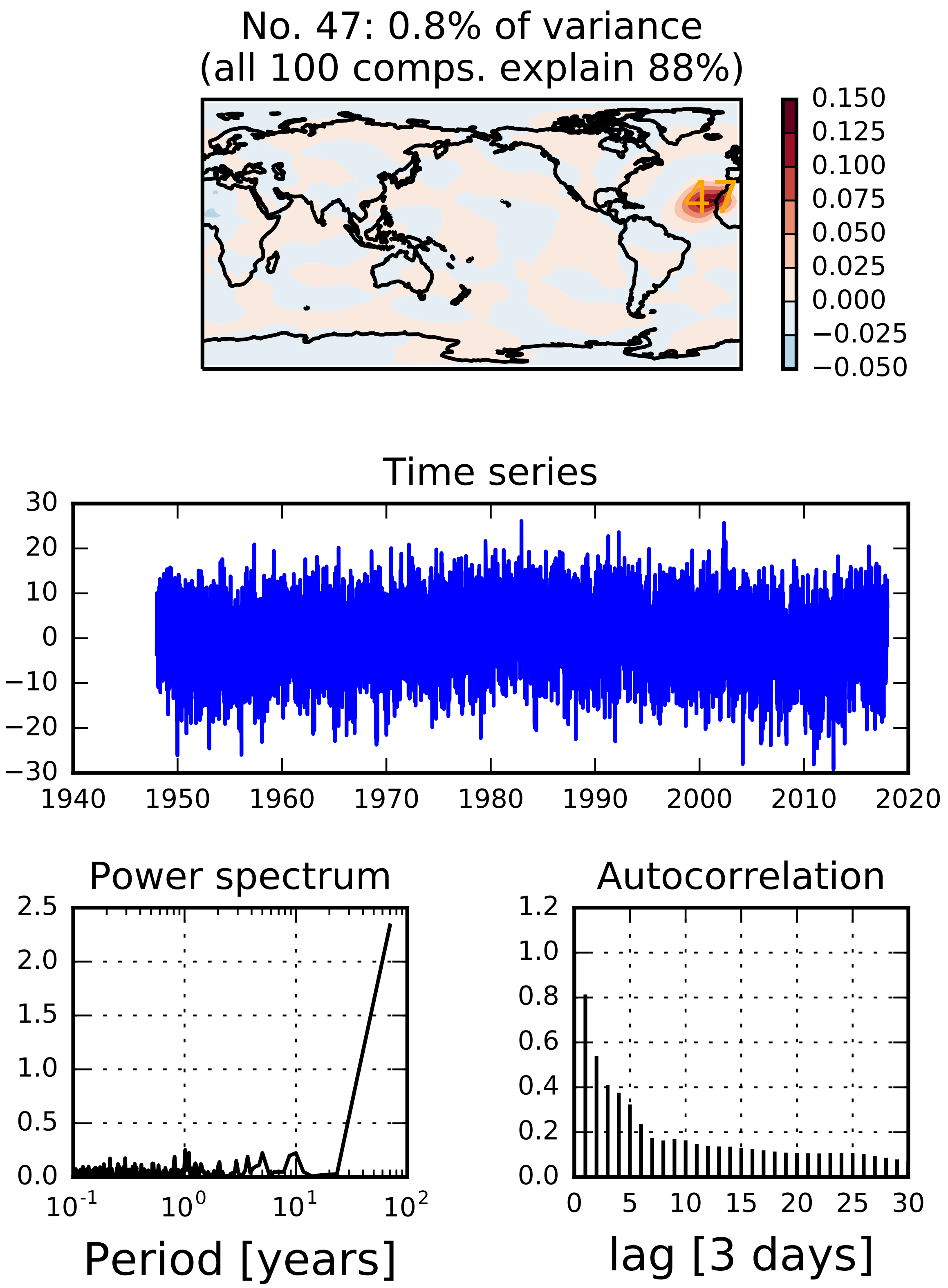
No. 47: 0.8% of variance
(all 100 comps. explain 88%)
47
0.150
0.125
0.100
0.075
0.050
0.025
0.000
−0.025
−0.050
Time series
30
20
10
0
−10
−20
−30
1940
1950
1960
1970
1980
1990
2000
2010
2020
Power spectrum
2.5
2.0
1.5
1.0
0.5
0.0
10^{-1}
10^{0}
10^{1}
10^{2}
Period [years]
Autocorrelation
1.2
1.0
0.8
0.6
0.4
0.2
0.0
0
5
10
15
20
25
30
lag [3 days]

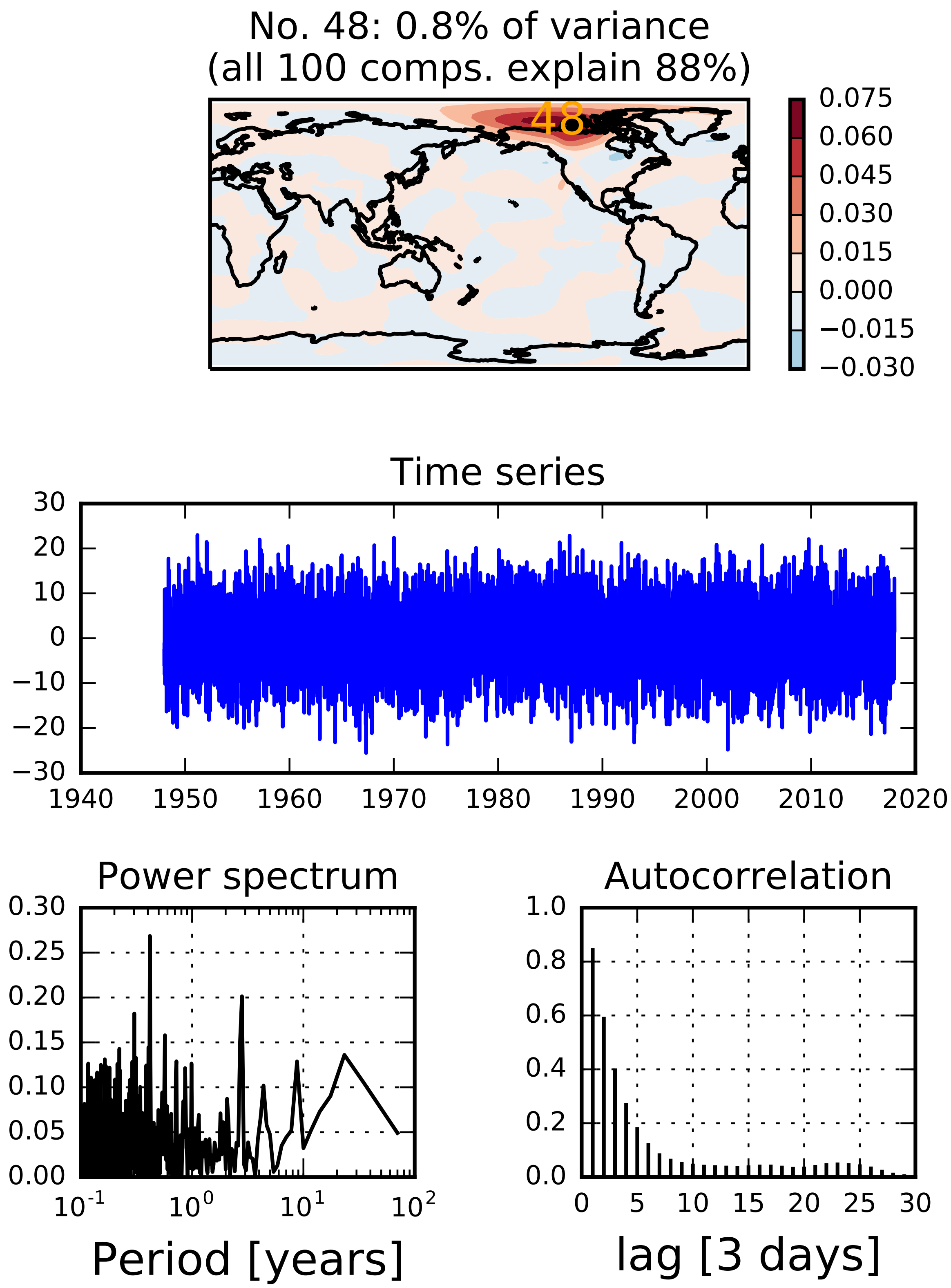
No. 48: 0.8% of variance
(all 100 comps. explain 88%)
48
0.075
0.060
0.045
0.030
0.015
0.000
−0.015
−0.030
Time series
30
20
10
0
−10
−20
−30
1940
1950
1960
1970
1980
1990
2000
2010
2020
Power spectrum
0.30
0.25
0.20
0.15
0.10
0.05
0.00
10^{-1}
10^{0}
10^{1}
10^{2}
Period [years]
Autocorrelation
1.0
0.8
0.6
0.4
0.2
0.0
0
5
10
15
20
25
30
lag [3 days]

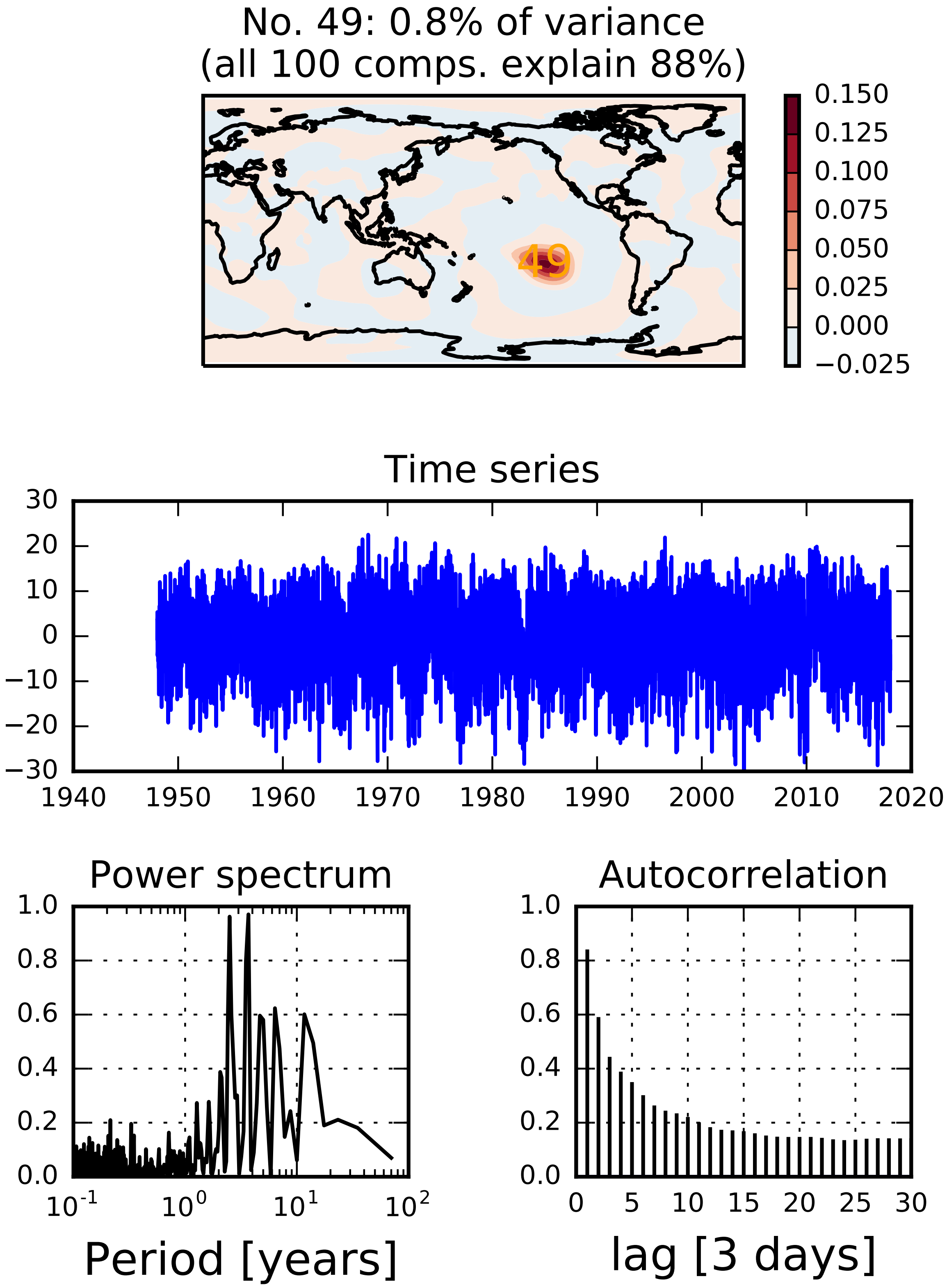
No. 49: 0.8% of variance
(all 100 comps. explain 88%)
49
0.150
0.125
0.100
0.075
0.050
0.025
0.000
−0.025
Time series
30
20
10
0
−10
−20
−30
1940
1950
1960
1970
1980
1990
2000
2010
2020
Power spectrum
1.0
0.8
0.6
0.4
0.2
0.0
10^{-1}
10^{0}
10^{1}
10^{2}
Period [years]
Autocorrelation
1.0
0.8
0.6
0.4
0.2
0.0
0
5
10
15
20
25
30
lag [3 days]

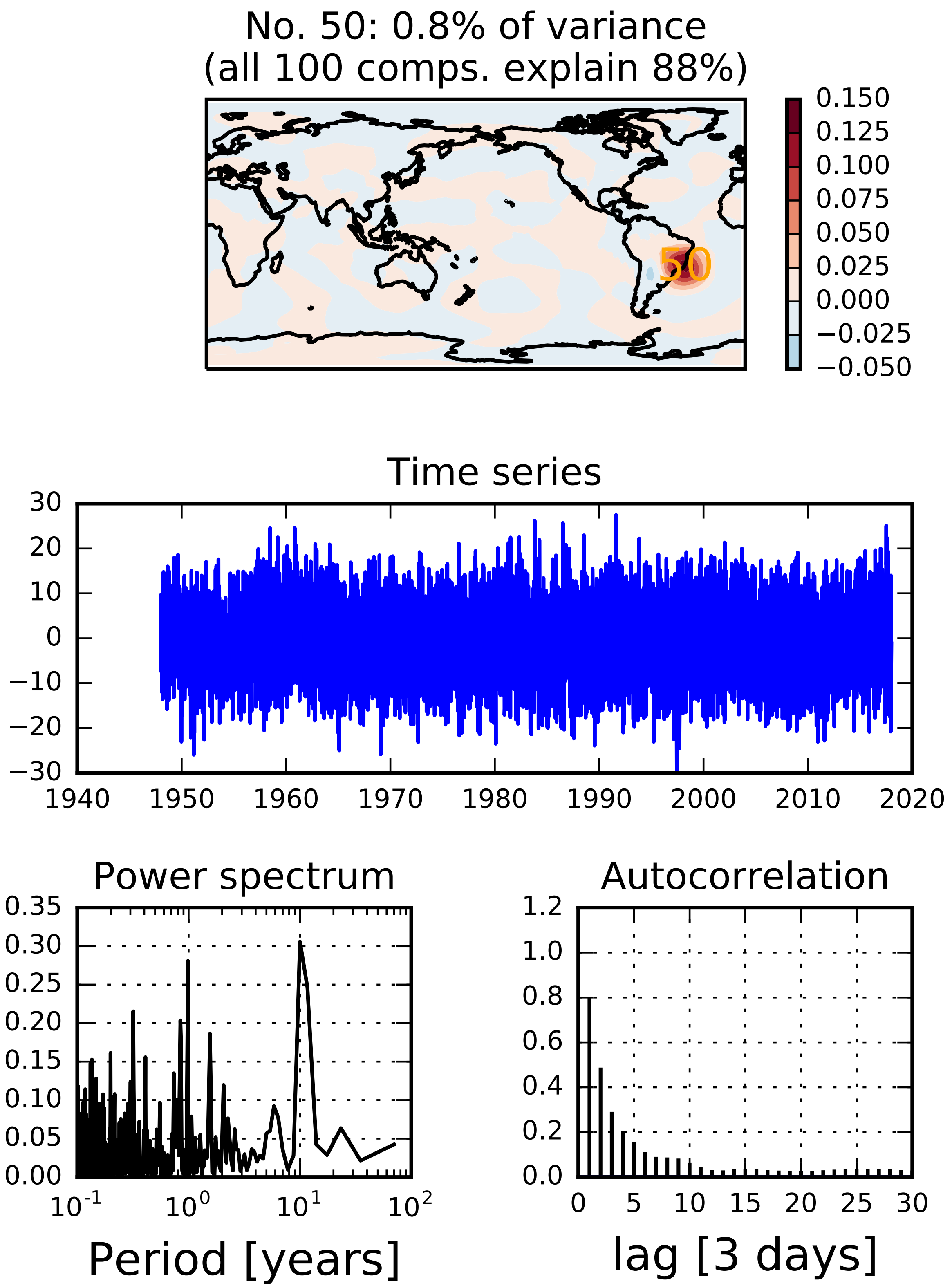
No. 50: 0.8% of variance
(all 100 comps. explain 88%)
50
0.150
0.125
0.100
0.075
0.050
0.025
0.000
−0.025
−0.050
Time series
30
20
10
0
−10
−20
−30
1940
1950
1960
1970
1980
1990
2000
2010
2020
Power spectrum
0.35
0.30
0.25
0.20
0.15
0.10
0.05
0.00
10^-1
10^0
10^1
10^2
Period [years]
Autocorrelation
1.2
1.0
0.8
0.6
0.4
0.2
0.0
0
5
10
15
20
25
30
lag [3 days]

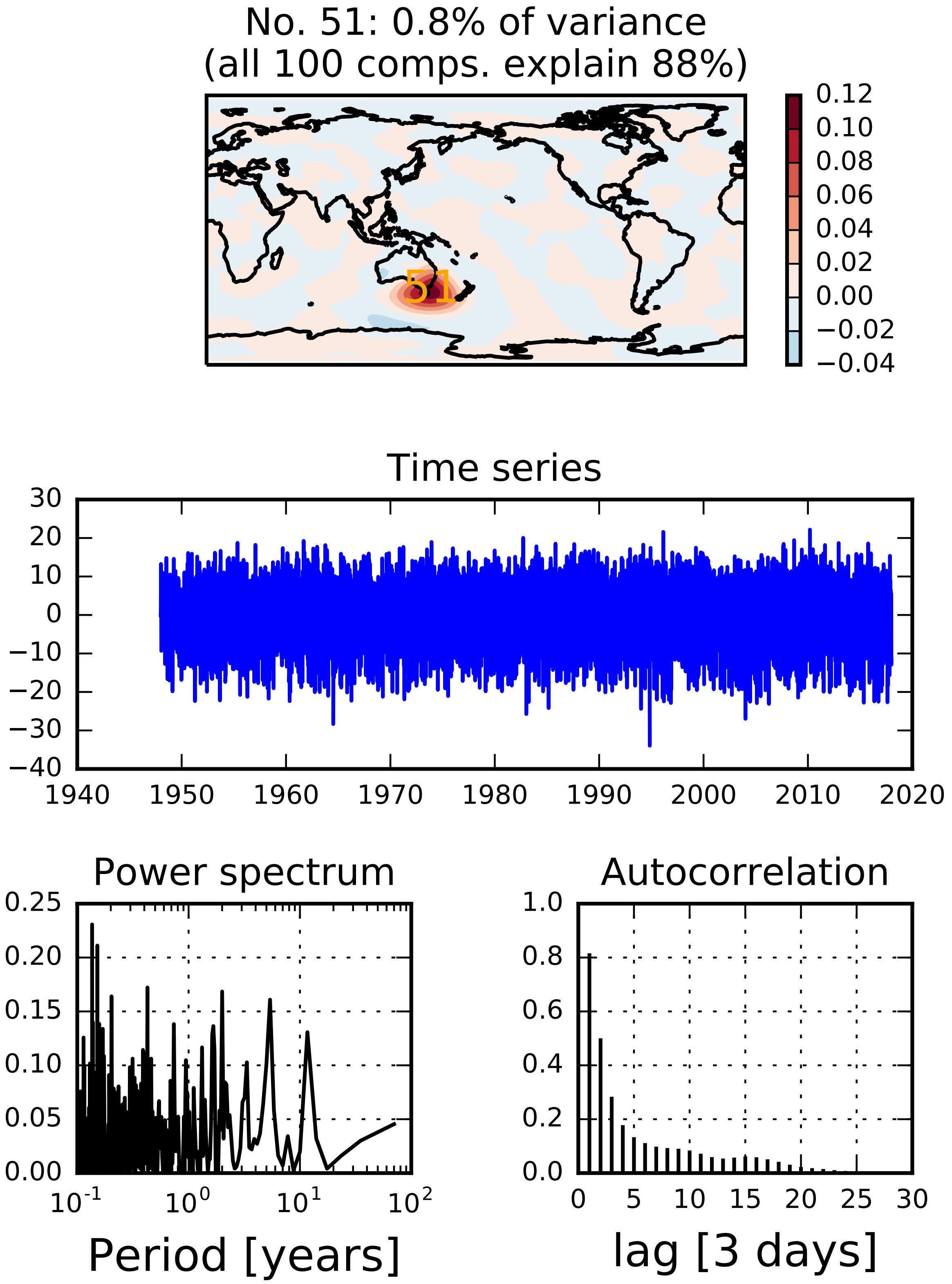

No. 51: 0.8% of variance
(all 100 comps. explain 88%)
51
0.12
0.10
0.08
0.06
0.04
0.02
0.00
−0.02
−0.04
Time series
30
20
10
0
−10
−20
−30
−40
1940
1950
1960
1970
1980
1990
2000
2010
2020
Power spectrum
0.25
0.20
0.15
0.10
0.05
0.00
10^{-1}
10^{0}
10^{1}
10^{2}
Period [years]
Autocorrelation
1.0
0.8
0.6
0.4
0.2
0.0
0
5
10
15
20
25
30
lag [3 days]

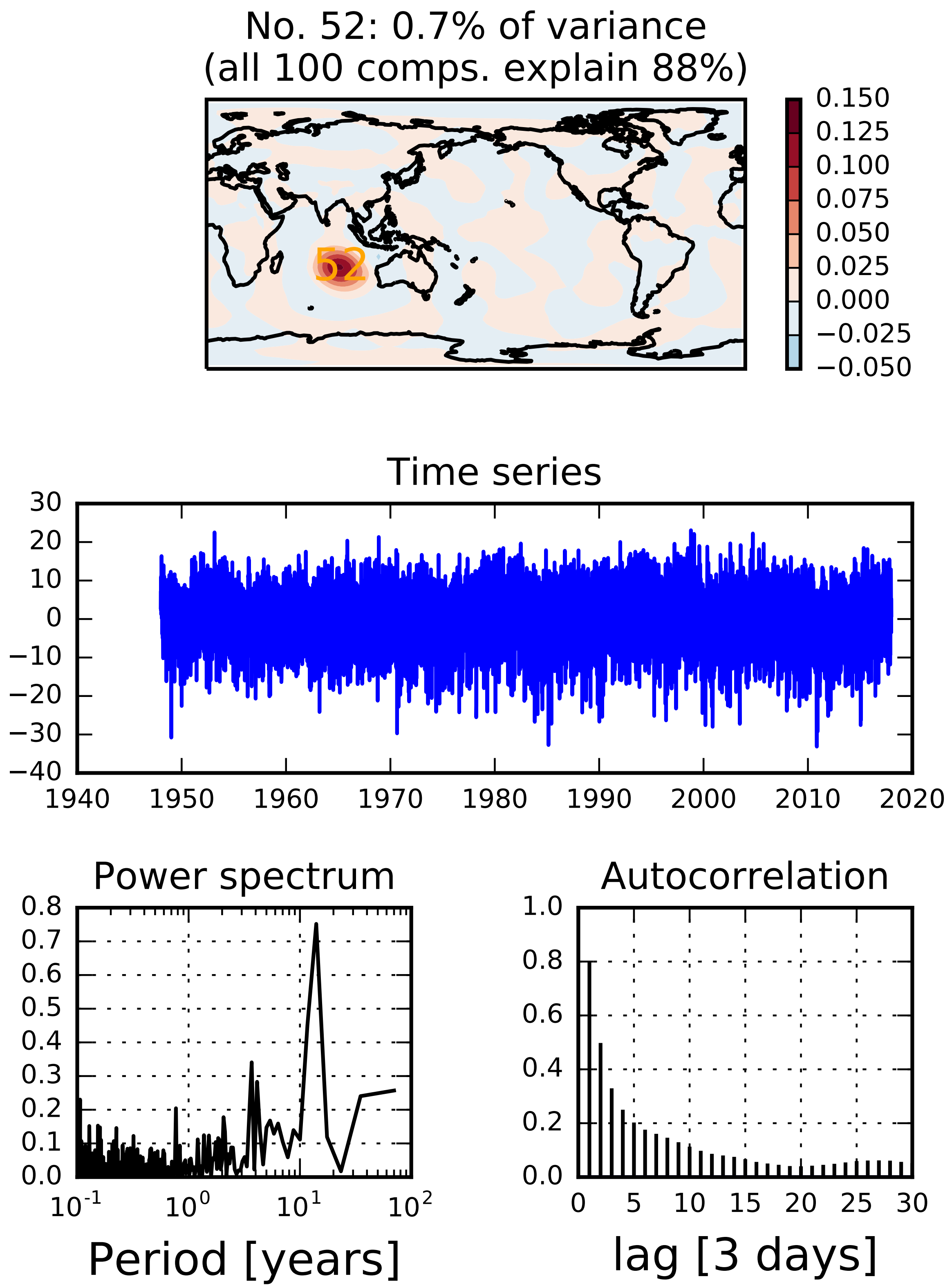
No. 52: 0.7% of variance
(all 100 comps. explain 88%)
52
0.150
0.125
0.100
0.075
0.050
0.025
0.000
−0.025
−0.050
Time series
30
20
10
0
−10
−20
−30
−40
1940
1950
1960
1970
1980
1990
2000
2010
2020
Power spectrum
0.8
0.7
0.6
0.5
0.4
0.3
0.2
0.1
0.0
10^{-1}
10^{0}
10^{1}
10^{2}
Period [years]
Autocorrelation
1.0
0.8
0.6
0.4
0.2
0.0
0
5
10
15
20
25
30
lag [3 days]

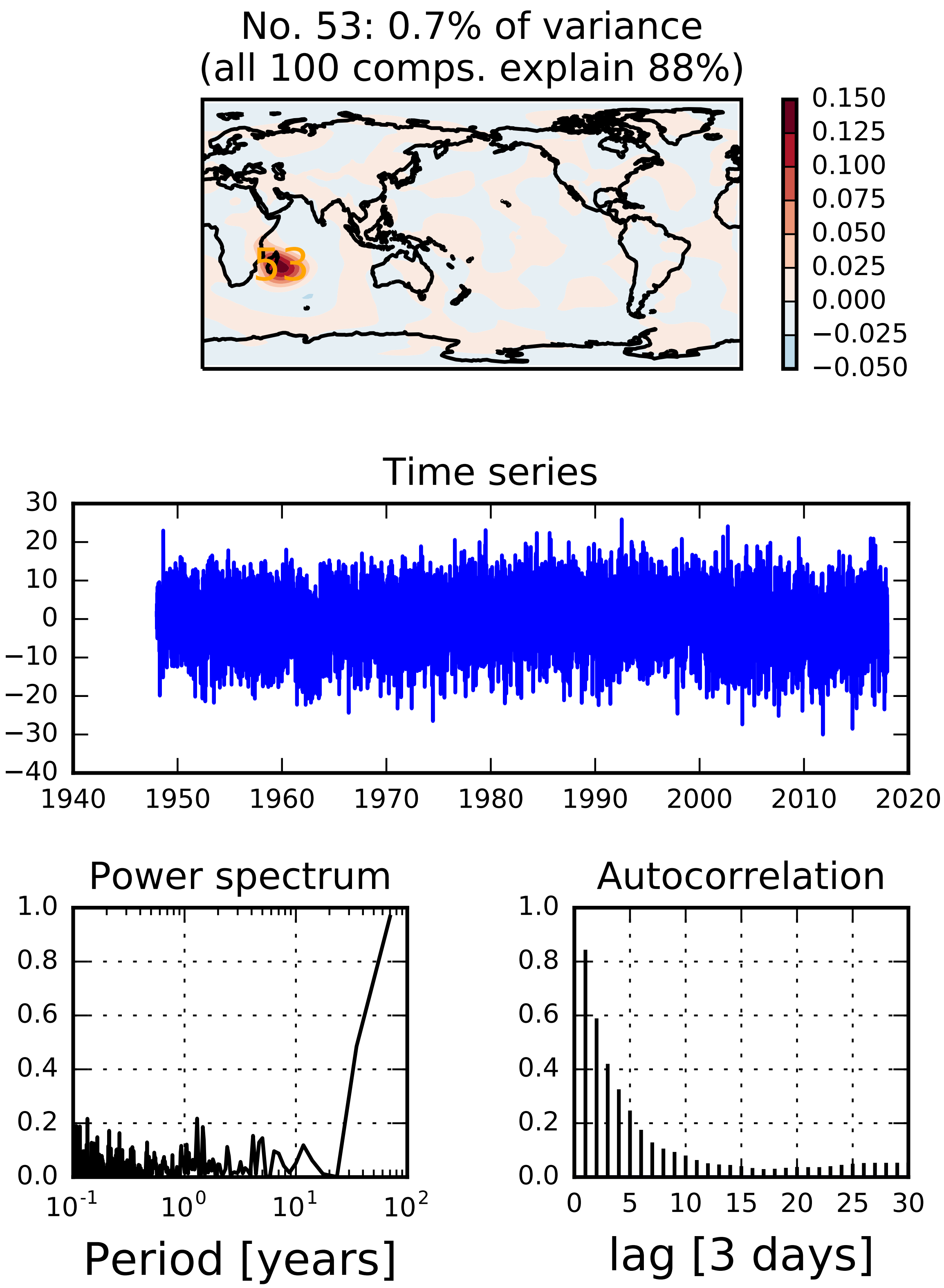
No. 53: 0.7% of variance
(all 100 comps. explain 88%)
0.150
0.125
0.100
0.075
0.050
0.025
0.000
−0.025
−0.050
53
Time series
30
20
10
0
−10
−20
−30
−40
1940
1950
1960
1970
1980
1990
2000
2010
2020
Power spectrum
1.0
0.8
0.6
0.4
0.2
0.0
10^{-1}
10^{0}
10^{1}
10^{2}
Period [years]
Autocorrelation
0
5
10
15
20
25
30
lag [3 days]

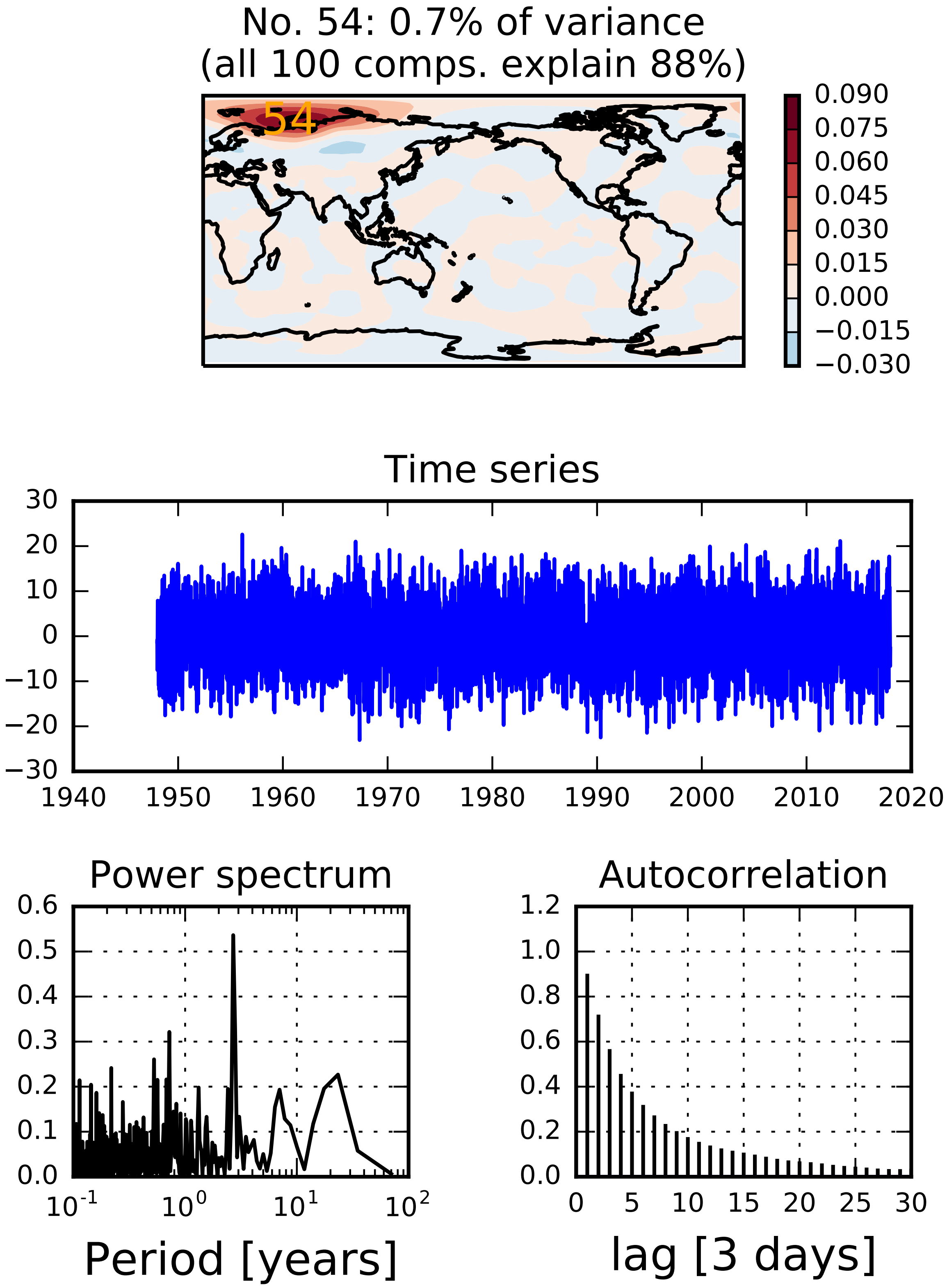
No. 54: 0.7% of variance
(all 100 comps. explain 88%)
54
0.090
0.075
0.060
0.045
0.030
0.015
0.000
−0.015
−0.030
Time series
30
20
10
0
−10
−20
−30
1940
1950
1960
1970
1980
1990
2000
2010
2020
Power spectrum
0.6
0.5
0.4
0.3
0.2
0.1
0.0
10^{-1}
10^{0}
10^{1}
10^{2}
Period [years]
Autocorrelation
1.2
1.0
0.8
0.6
0.4
0.2
0.0
0
5
10
15
20
25
30
lag [3 days]

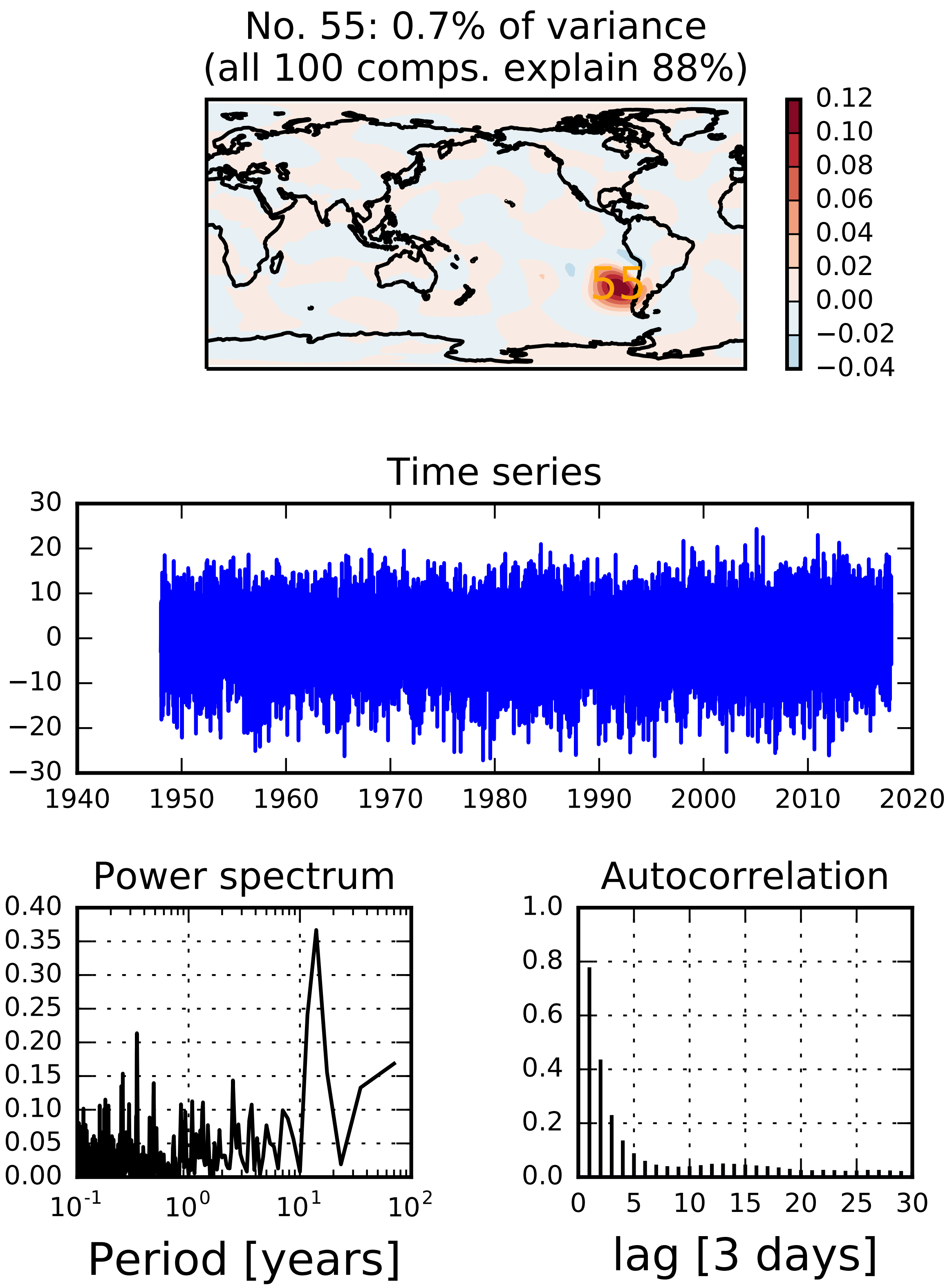
No. 55: 0.7% of variance
(all 100 comps. explain 88%)
0.12
0.10
0.08
0.06
0.04
0.02
0.00
−0.02
−0.04
55
Time series
30
20
10
0
−10
−20
−30
1940
1950
1960
1970
1980
1990
2000
2010
2020
Power spectrum
0.40
0.35
0.30
0.25
0.20
0.15
0.10
0.05
0.00
10^-1
10^0
10^1
10^2
Period [years]
Autocorrelation
1.0
0.8
0.6
0.4
0.2
0.0
0
5
10
15
20
25
30
lag [3 days]

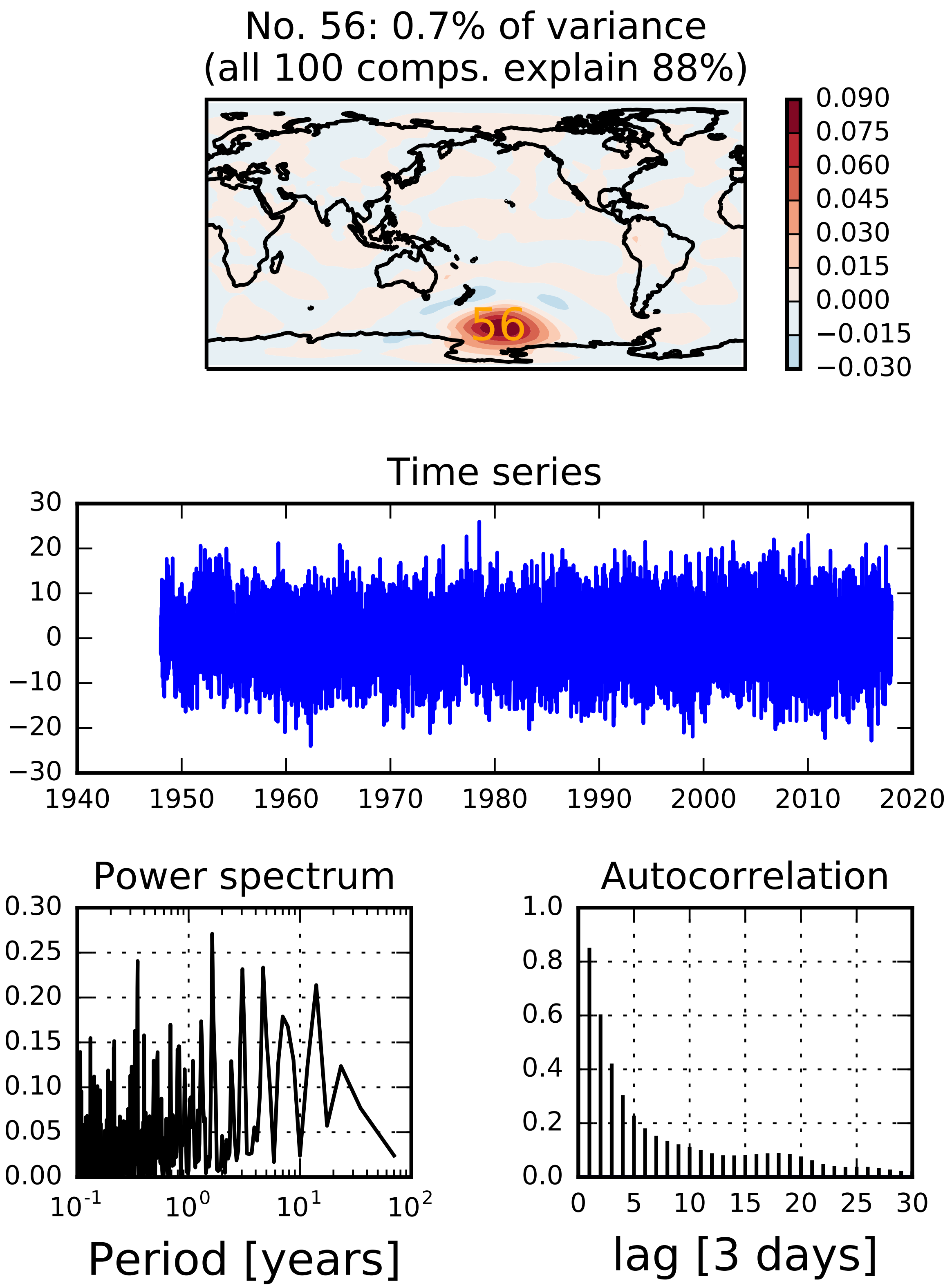
No. 56: 0.7% of variance
(all 100 comps. explain 88%)
56
0.090
0.075
0.060
0.045
0.030
0.015
0.000
−0.015
−0.030
Time series
30
20
10
0
−10
−20
−30
1940
1950
1960
1970
1980
1990
2000
2010
2020
Power spectrum
0.30
0.25
0.20
0.15
0.10
0.05
0.00
10^{-1}
10^{0}
10^{1}
10^{2}
Period [years]
Autocorrelation
1.0
0.8
0.6
0.4
0.2
0.0
0
5
10
15
20
25
30
lag [3 days]

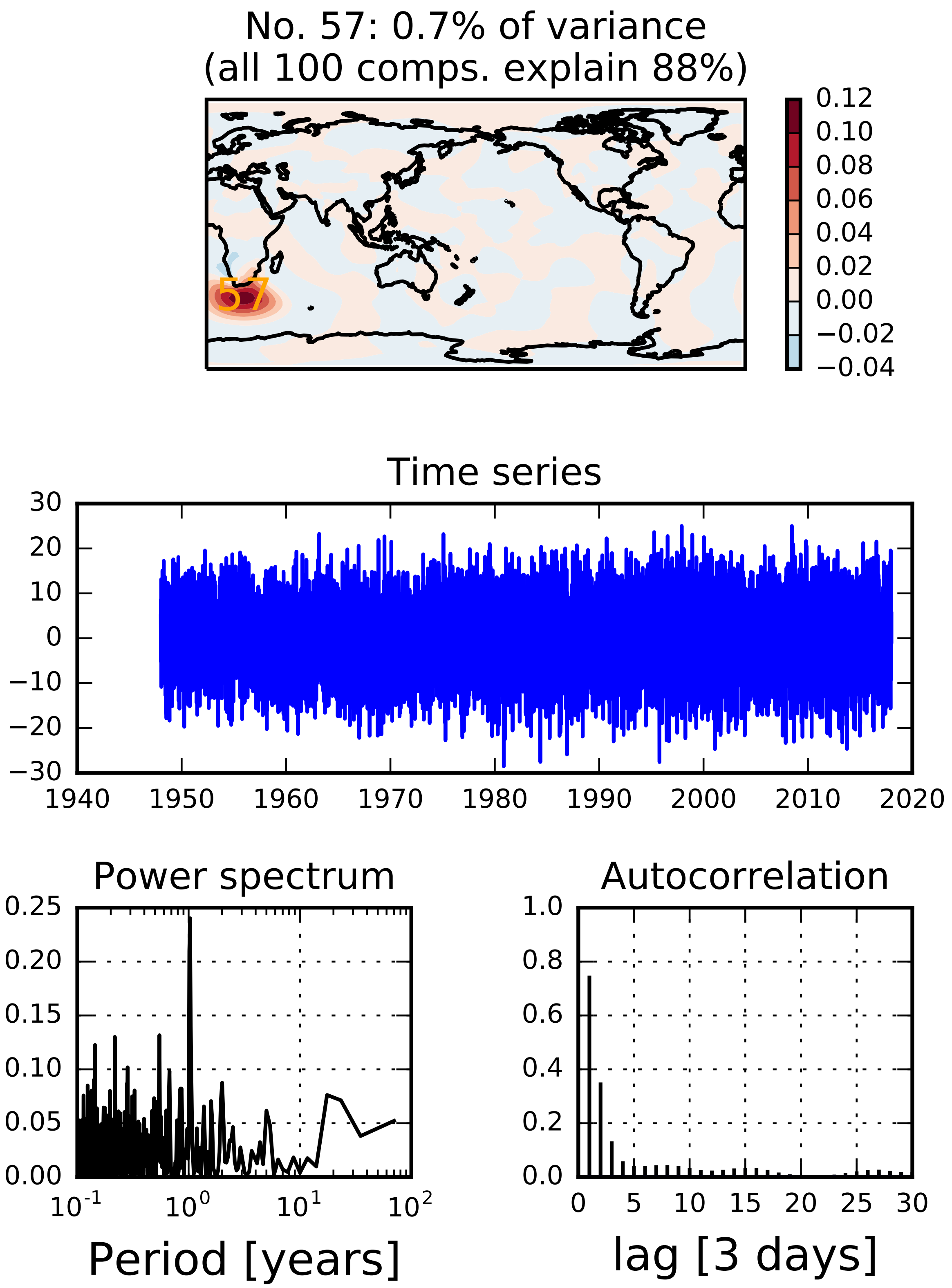

No. 57: 0.7% of variance
(all 100 comps. explain 88%)
57
0.12
0.10
0.08
0.06
0.04
0.02
0.00
−0.02
−0.04
Time series
30
20
10
0
−10
−20
−30
1940
1950
1960
1970
1980
1990
2000
2010
2020
Power spectrum
0.25
0.20
0.15
0.10
0.05
0.00
10^-1
10^0
10^1
10^2
Period [years]
Autocorrelation
1.0
0.8
0.6
0.4
0.2
0.0
0
5
10
15
20
25
30
lag [3 days]

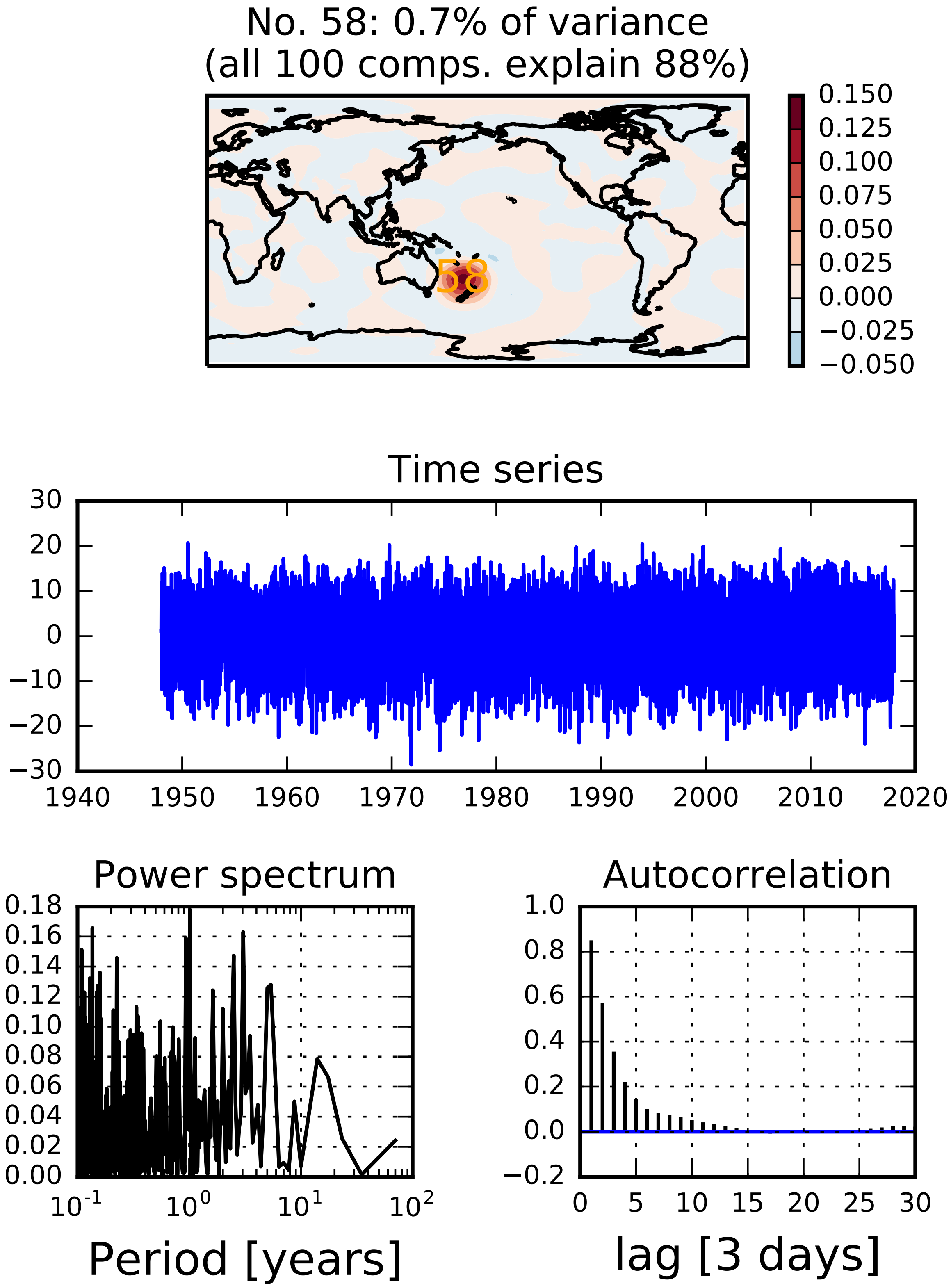
No. 58: 0.7% of variance
(all 100 comps. explain 88%)
58
0.150
0.125
0.100
0.075
0.050
0.025
0.000
−0.025
−0.050
Time series
30
20
10
0
−10
−20
−30
1940
1950
1960
1970
1980
1990
2000
2010
2020
Power spectrum
0.18
0.16
0.14
0.12
0.10
0.08
0.06
0.04
0.02
0.00
10^{-1}
10^{0}
10^{1}
10^{2}
Period [years]
Autocorrelation
1.0
0.8
0.6
0.4
0.2
0.0
−0.2
0
5
10
15
20
25
30
lag [3 days]

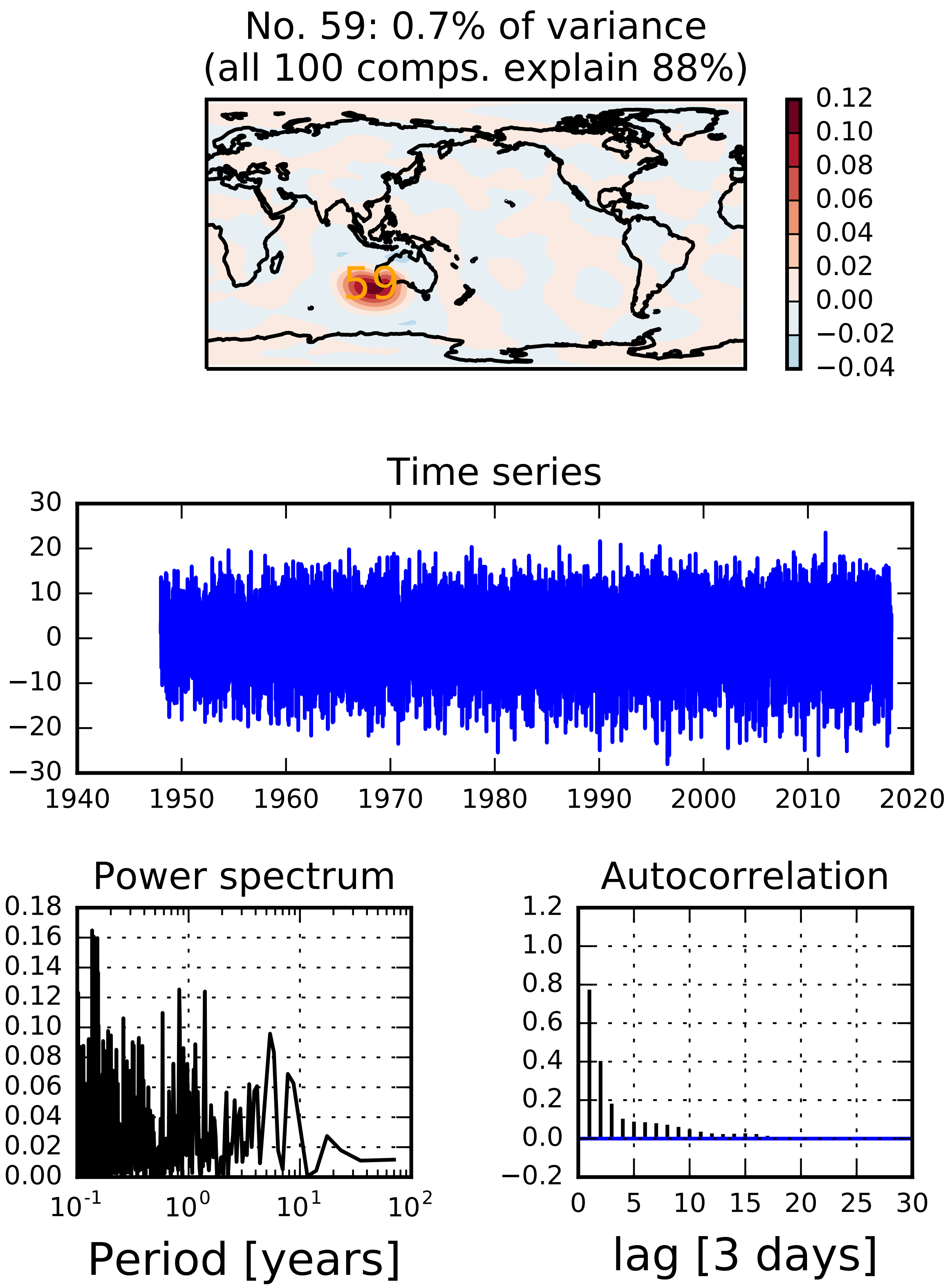

No. 59: 0.7% of variance
(all 100 comps. explain 88%)
59
0.12
0.10
0.08
0.06
0.04
0.02
0.00
−0.02
−0.04
Time series
30
20
10
0
−10
−20
−30
1940
1950
1960
1970
1980
1990
2000
2010
2020
Power spectrum
0.18
0.16
0.14
0.12
0.10
0.08
0.06
0.04
0.02
0.00
10^{-1}
10^{0}
10^{1}
10^{2}
Period [years]
Autocorrelation
1.2
1.0
0.8
0.6
0.4
0.2
0.0
−0.2
0
5
10
15
20
25
30
lag [3 days]

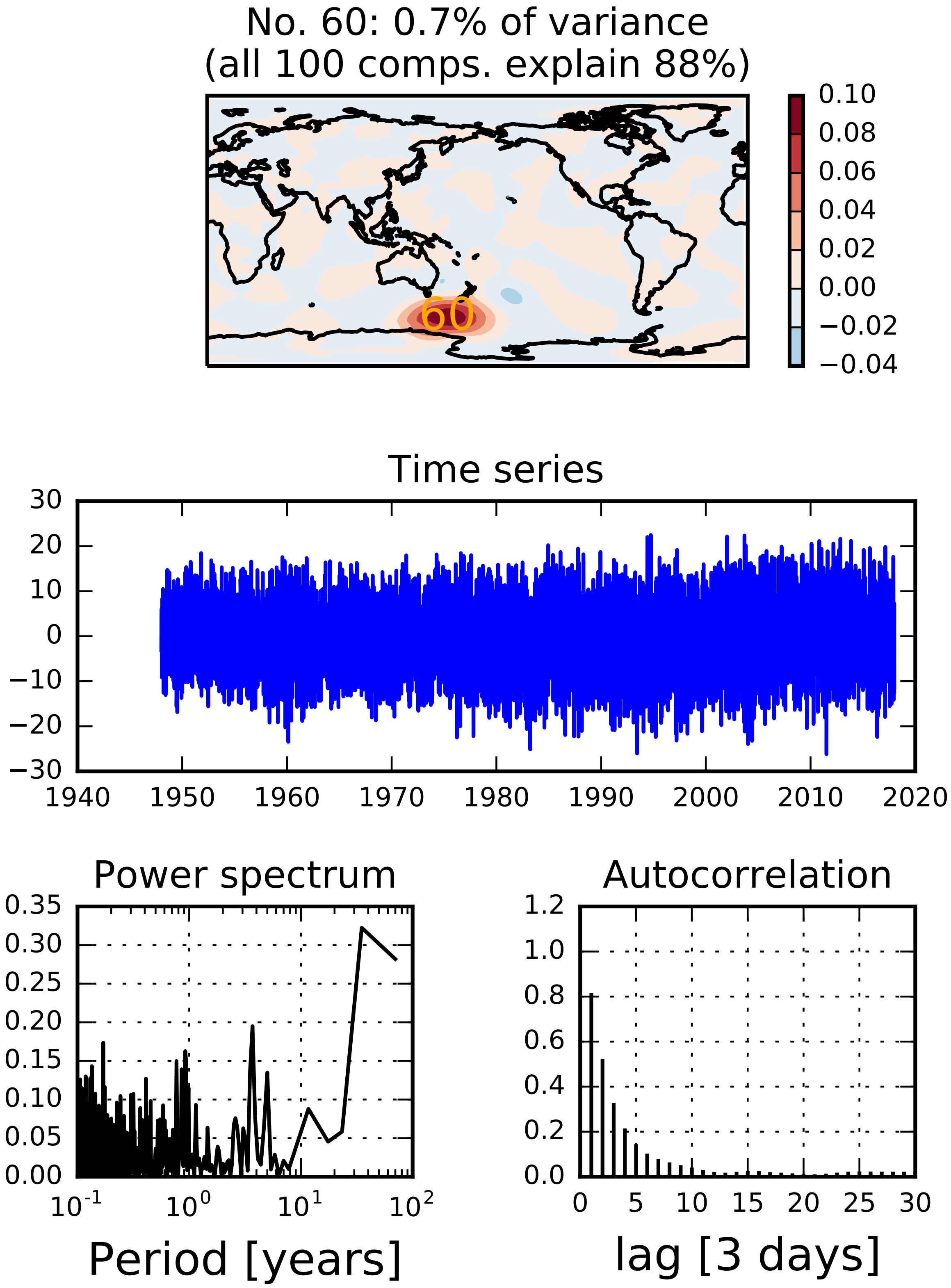
No. 60: 0.7% of variance
(all 100 comps. explain 88%)
60
0.10
0.08
0.06
0.04
0.02
0.00
−0.02
−0.04
Time series
30
20
10
0
−10
−20
−30
1940
1950
1960
1970
1980
1990
2000
2010
2020
Power spectrum
0.35
0.30
0.25
0.20
0.15
0.10
0.05
0.00
10^-1
10^0
10^1
10^2
Period [years]
Autocorrelation
1.2
1.0
0.8
0.6
0.4
0.2
0.0
0
5
10
15
20
25
30
lag [3 days]

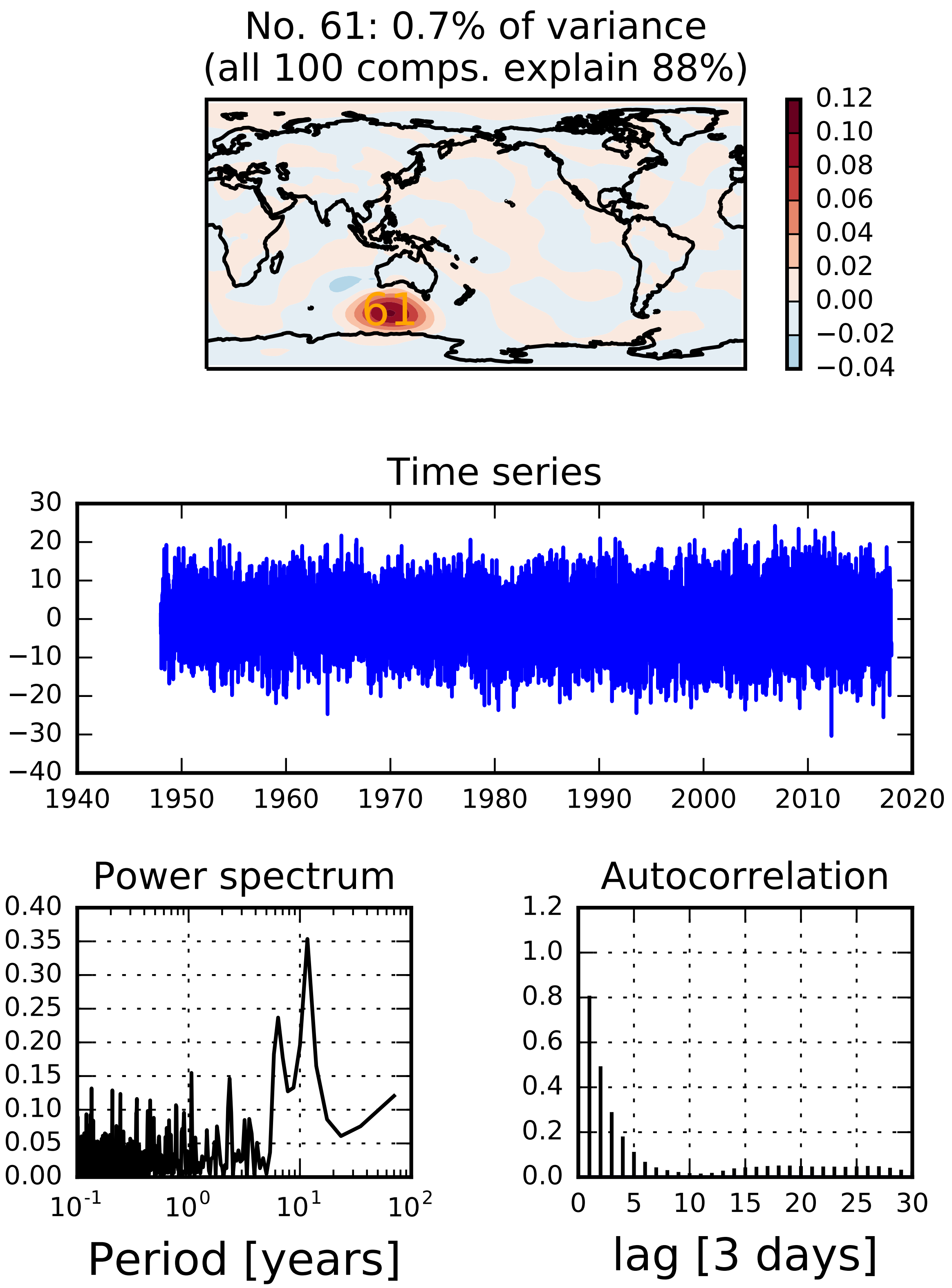
No. 61: 0.7% of variance
(all 100 comps. explain 88%)
61
0.12
0.10
0.08
0.06
0.04
0.02
0.00
−0.02
−0.04
Time series
30
20
10
0
−10
−20
−30
−40
1940
1950
1960
1970
1980
1990
2000
2010
2020
Power spectrum
0.40
0.35
0.30
0.25
0.20
0.15
0.10
0.05
0.00
10^{-1}
10^{0}
10^{1}
10^{2}
Period [years]
Autocorrelation
1.2
1.0
0.8
0.6
0.4
0.2
0.0
0
5
10
15
20
25
30
lag [3 days]

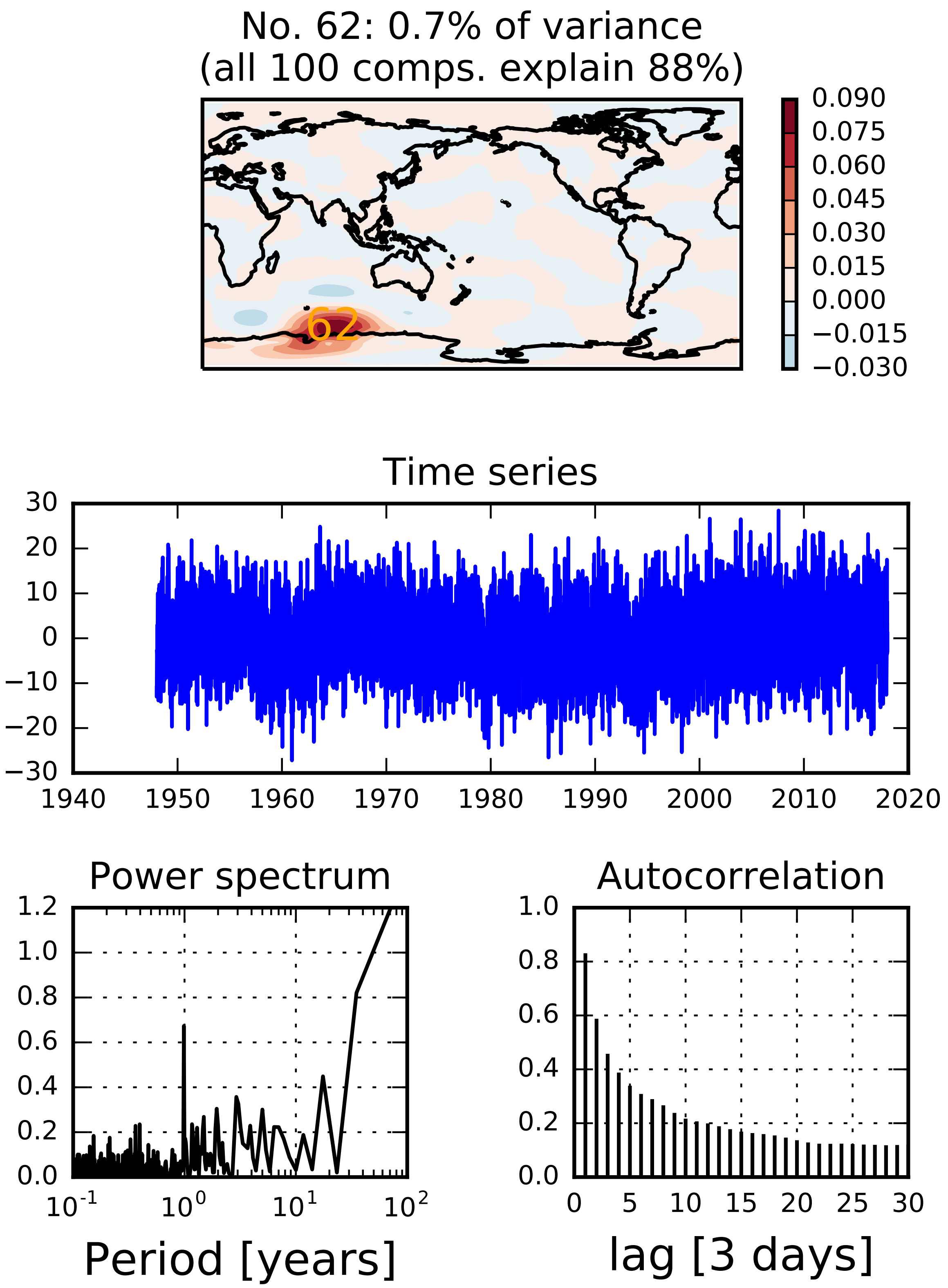
No. 62: 0.7% of variance
(all 100 comps. explain 88%)
62
0.090
0.075
0.060
0.045
0.030
0.015
0.000
−0.015
−0.030
Time series
30
20
10
0
−10
−20
−30
1940
1950
1960
1970
1980
1990
2000
2010
2020
Power spectrum
1.2
1.0
0.8
0.6
0.4
0.2
0.0
10^{-1}
10^{0}
10^{1}
10^{2}
Period [years]
Autocorrelation
1.0
0.8
0.6
0.4
0.2
0.0
0
5
10
15
20
25
30
lag [3 days]

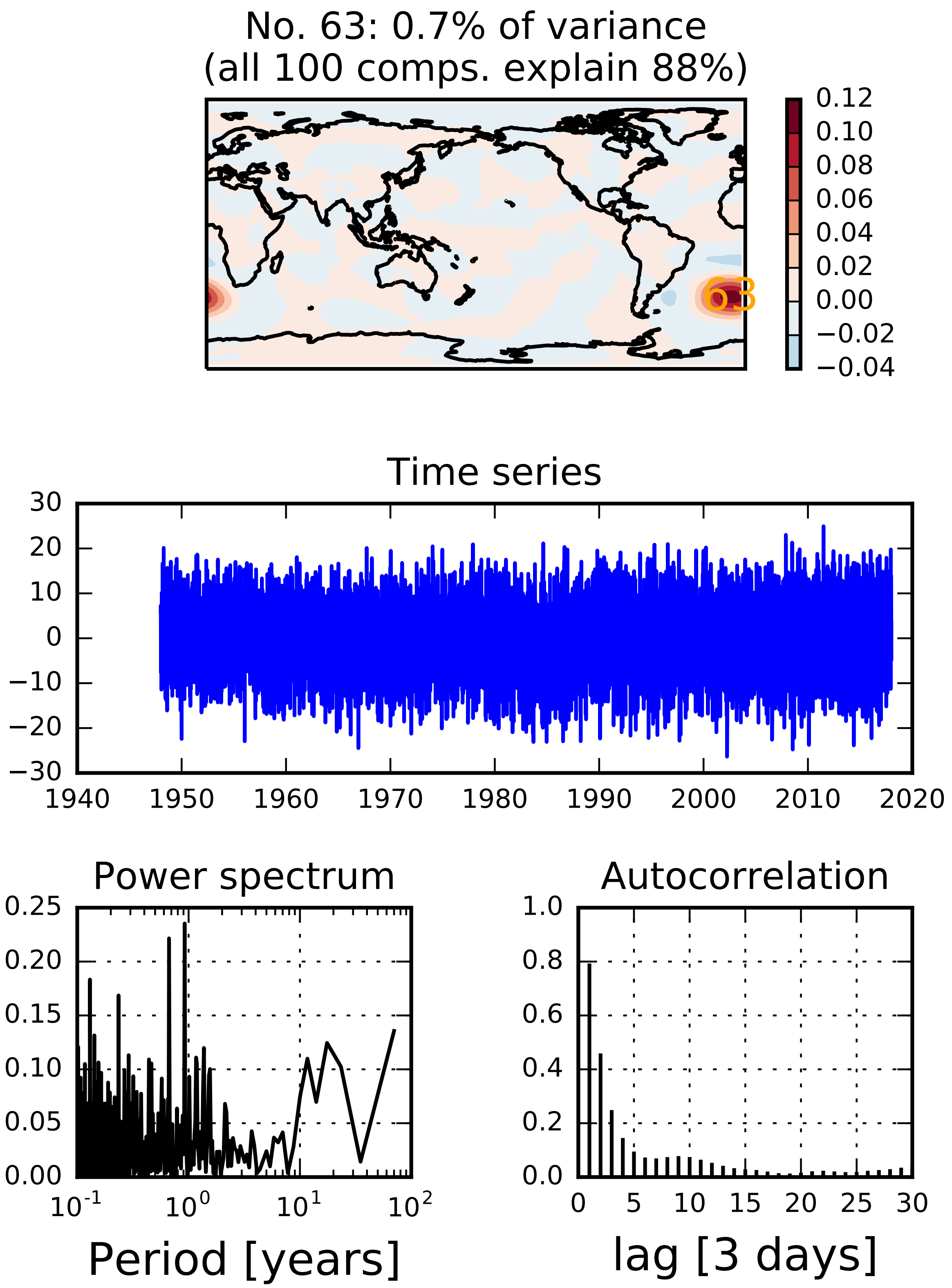
No. 63: 0.7% of variance
(all 100 comps. explain 88%)
63
0.12
0.10
0.08
0.06
0.04
0.02
0.00
−0.02
−0.04
Time series
30
20
10
0
−10
−20
−30
1940
1950
1960
1970
1980
1990
2000
2010
2020
Power spectrum
0.25
0.20
0.15
0.10
0.05
0.00
10^{-1}
10^{0}
10^{1}
10^{2}
Period [years]
Autocorrelation
1.0
0.8
0.6
0.4
0.2
0.0
0
5
10
15
20
25
30
lag [3 days]

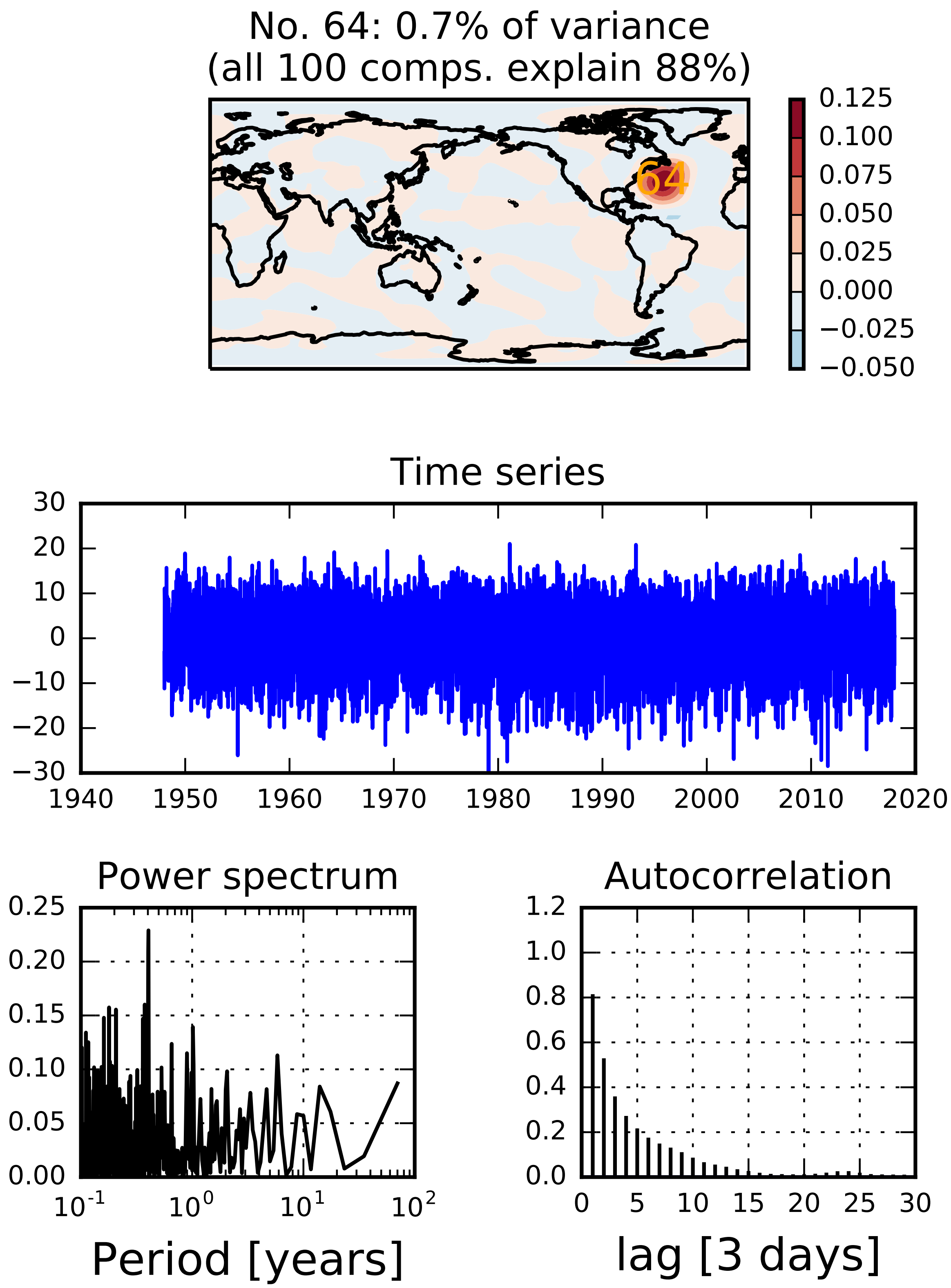
No. 64: 0.7% of variance
(all 100 comps. explain 88%)
64
0.125
0.100
0.075
0.050
0.025
0.000
−0.025
−0.050
Time series
30
20
10
0
−10
−20
−30
1940
1950
1960
1970
1980
1990
2000
2010
2020
Power spectrum
0.25
0.20
0.15
0.10
0.05
0.00
10^{-1}
10^{0}
10^{1}
10^{2}
Period [years]
Autocorrelation
1.2
1.0
0.8
0.6
0.4
0.2
0.0
0
5
10
15
20
25
30
lag [3 days]

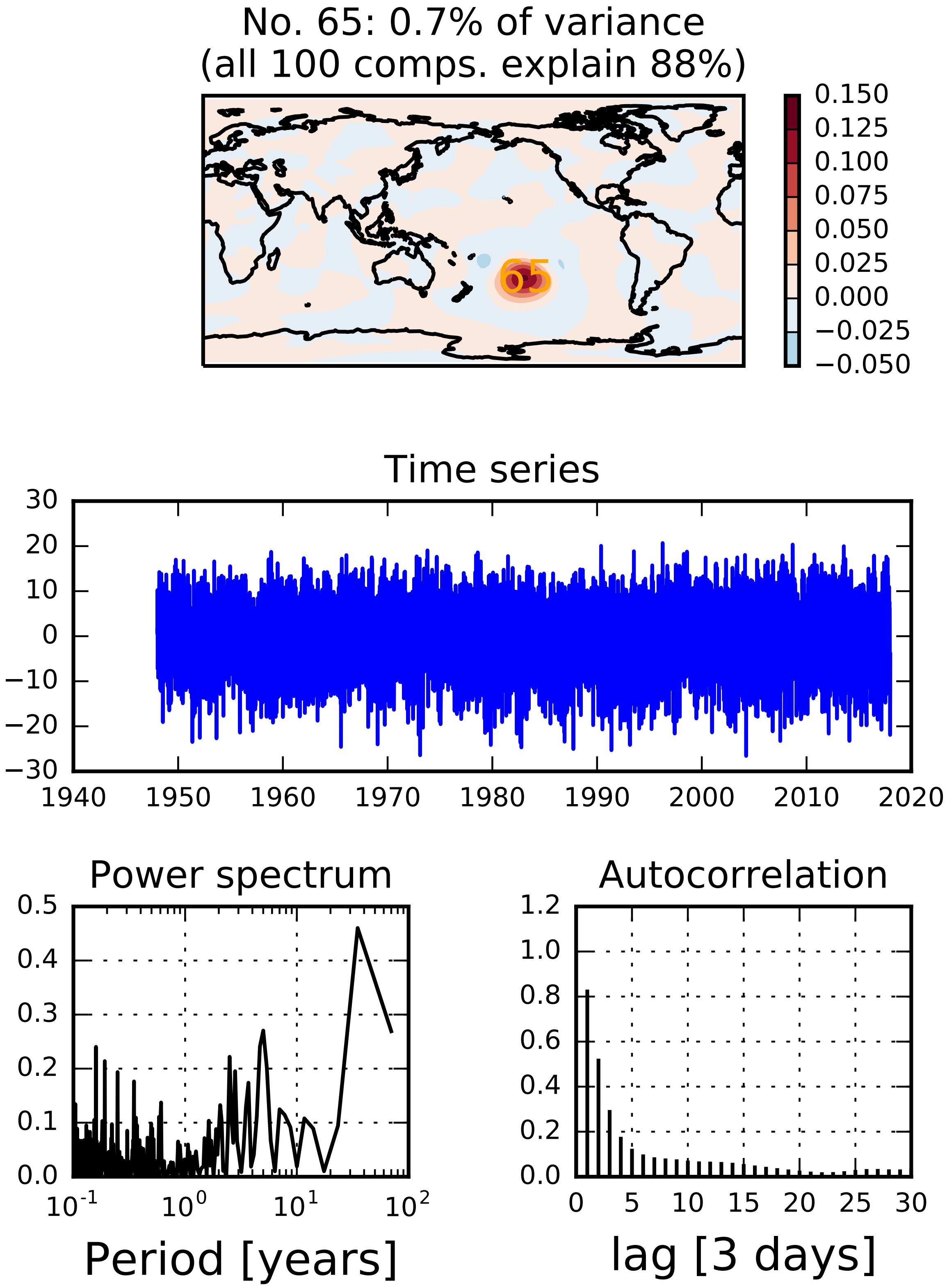
No. 65: 0.7% of variance
(all 100 comps. explain 88%)
0.150
0.125
0.100
0.075
0.050
0.025
0.000
−0.025
−0.050
65
Time series
30
20
10
0
−10
−20
−30
1940
1950
1960
1970
1980
1990
2000
2010
2020
Power spectrum
0.5
0.4
0.3
0.2
0.1
0.0
10^{-1}
10^{0}
10^{1}
10^{2}
Period [years]
Autocorrelation
1.2
1.0
0.8
0.6
0.4
0.2
0.0
0
5
10
15
20
25
30
lag [3 days]

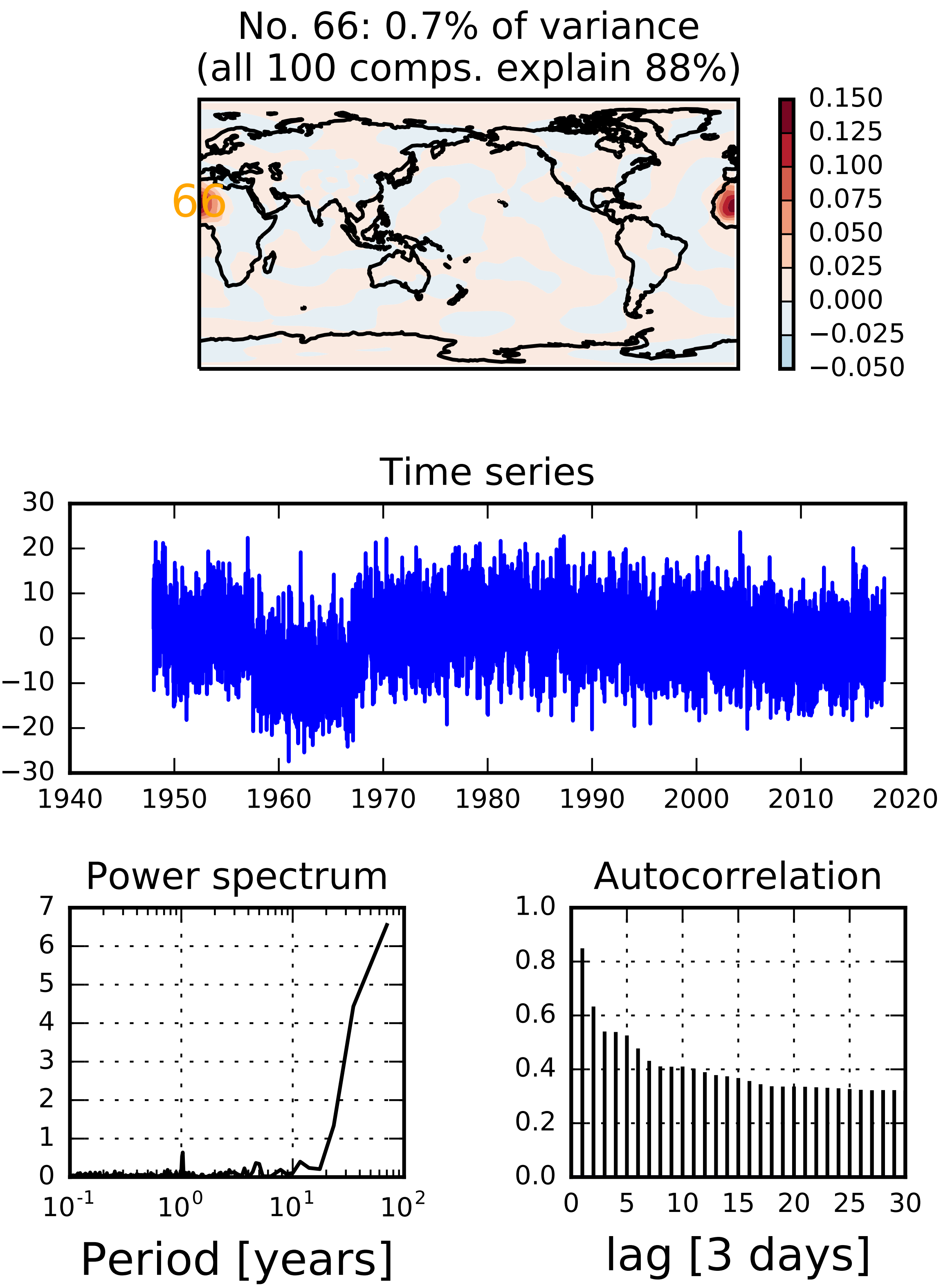

No. 66: 0.7% of variance
(all 100 comps. explain 88%)
66
0.150
0.125
0.100
0.075
0.050
0.025
0.000
−0.025
−0.050
Time series
30
20
10
0
−10
−20
−30
1940
1950
1960
1970
1980
1990
2000
2010
2020
Power spectrum
7
6
5
4
3
2
1
0
10^{-1}
10^{0}
10^{1}
10^{2}
Period [years]
Autocorrelation
1.0
0.8
0.6
0.4
0.2
0.0
0
5
10
15
20
25
30
lag [3 days]

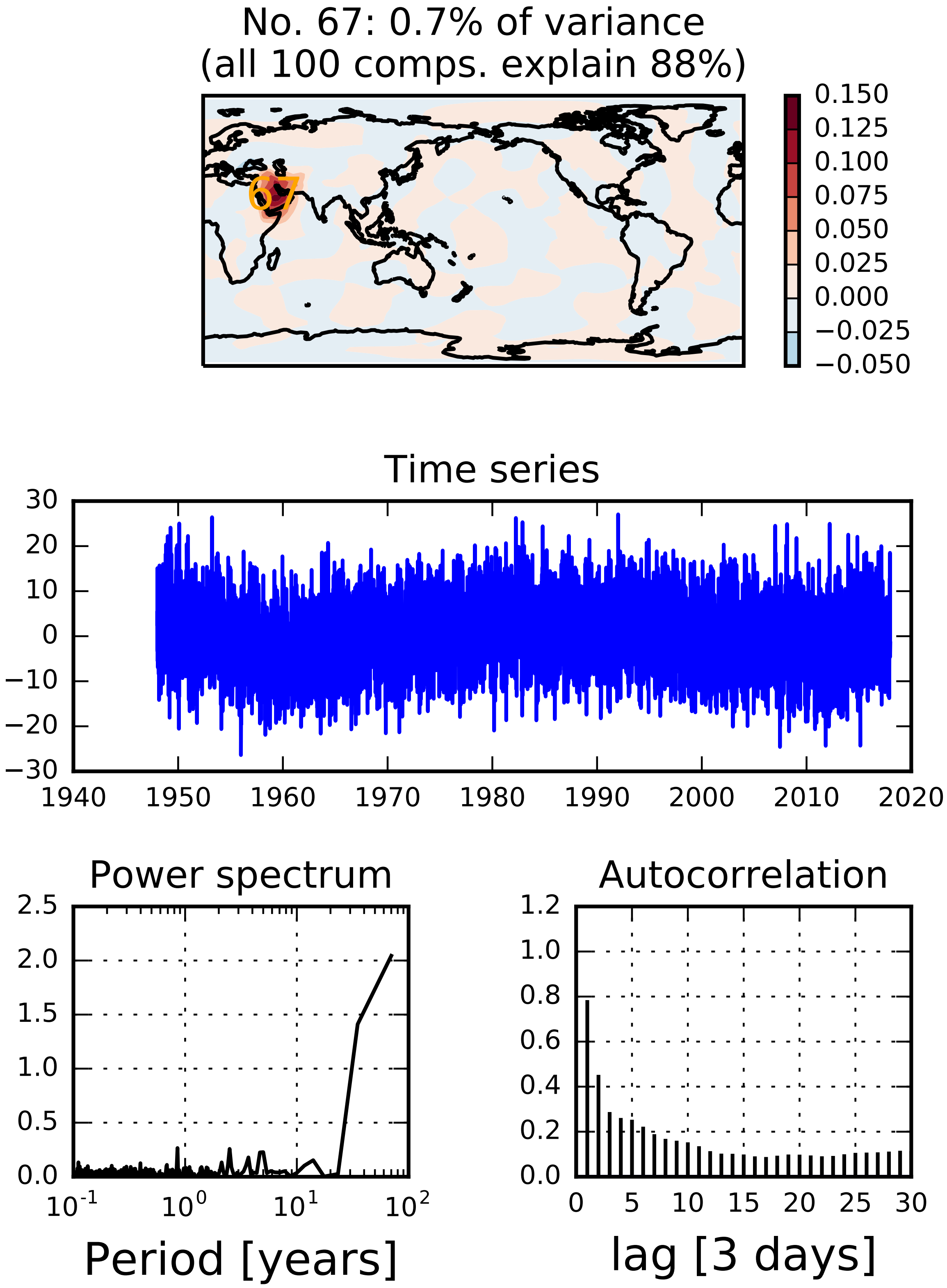
No. 67: 0.7% of variance
(all 100 comps. explain 88%)
67
0.150
0.125
0.100
0.075
0.050
0.025
0.000
−0.025
−0.050
Time series
30
20
10
0
−10
−20
−30
1940
1950
1960
1970
1980
1990
2000
2010
2020
Power spectrum
2.5
2.0
1.5
1.0
0.5
0.0
10^{-1}
10^{0}
10^{1}
10^{2}
Period [years]
Autocorrelation
1.2
1.0
0.8
0.6
0.4
0.2
0.0
0
5
10
15
20
25
30
lag [3 days]

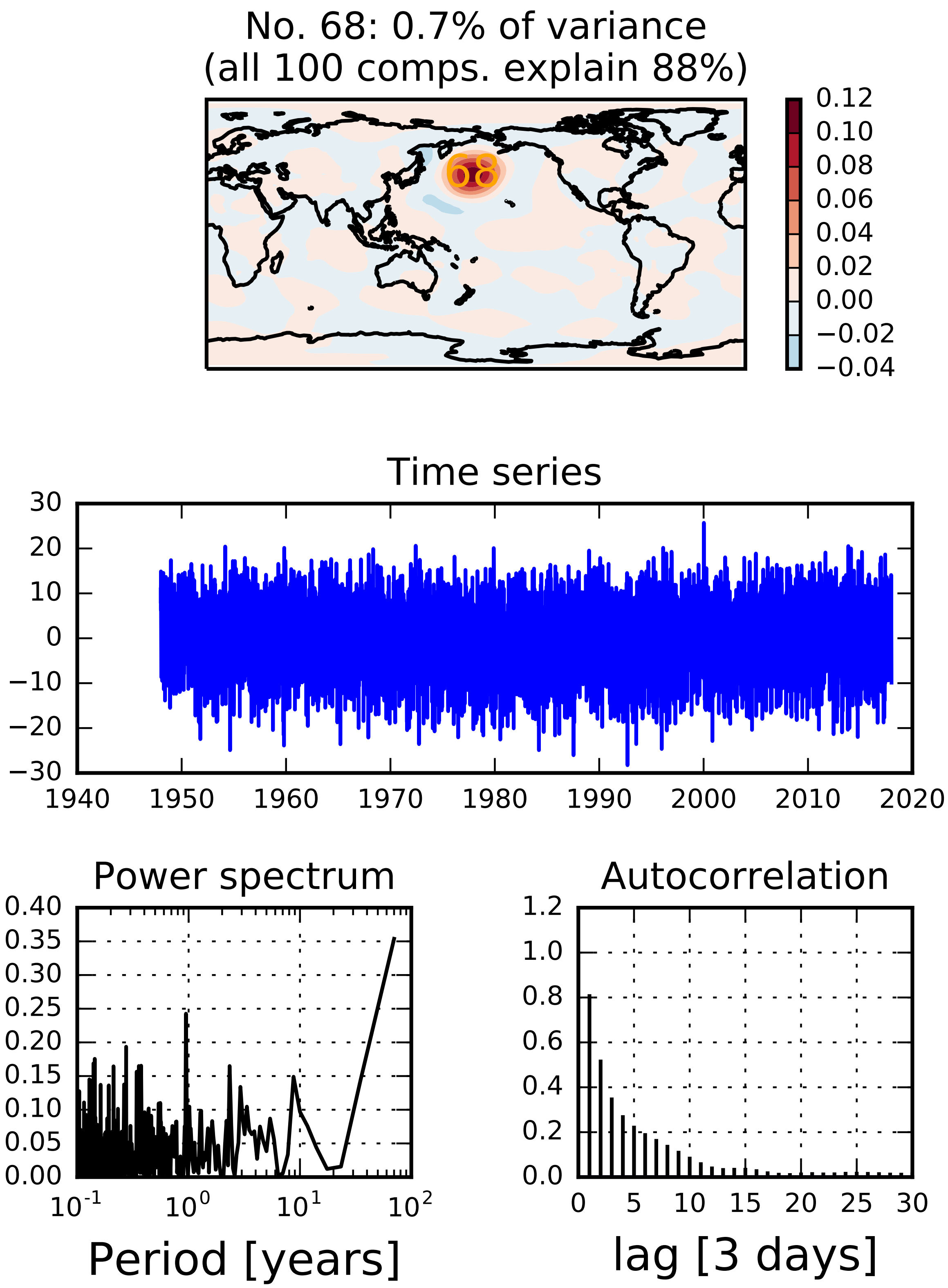
No. 68: 0.7% of variance
(all 100 comps. explain 88%)
68
0.12
0.10
0.08
0.06
0.04
0.02
0.00
−0.02
−0.04
Time series
30
20
10
0
−10
−20
−30
1940
1950
1960
1970
1980
1990
2000
2010
2020
Power spectrum
0.40
0.35
0.30
0.25
0.20
0.15
0.10
0.05
0.00
10^{-1}
10^{0}
10^{1}
10^{2}
Period [years]
Autocorrelation
1.2
1.0
0.8
0.6
0.4
0.2
0.0
0
5
10
15
20
25
30
lag [3 days]

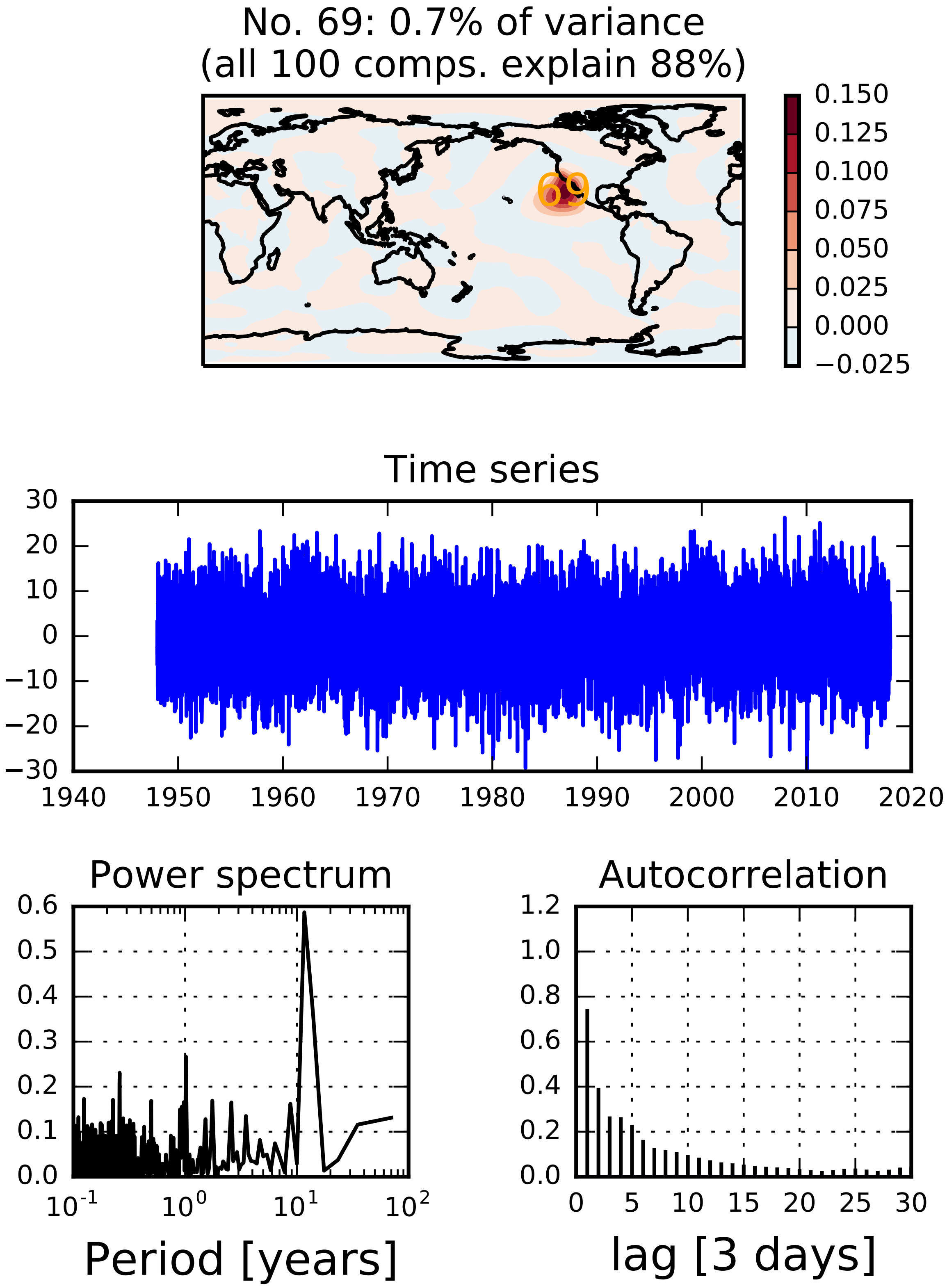
No. 69: 0.7% of variance
(all 100 comps. explain 88%)
69
0.150
0.125
0.100
0.075
0.050
0.025
0.000
−0.025
Time series
30
20
10
0
−10
−20
−30
1940
1950
1960
1970
1980
1990
2000
2010
2020
Power spectrum
0.6
0.5
0.4
0.3
0.2
0.1
0.0
10^{-1}
10^{0}
10^{1}
10^{2}
Period [years]
Autocorrelation
1.2
1.0
0.8
0.6
0.4
0.2
0.0
0
5
10
15
20
25
30
lag [3 days]

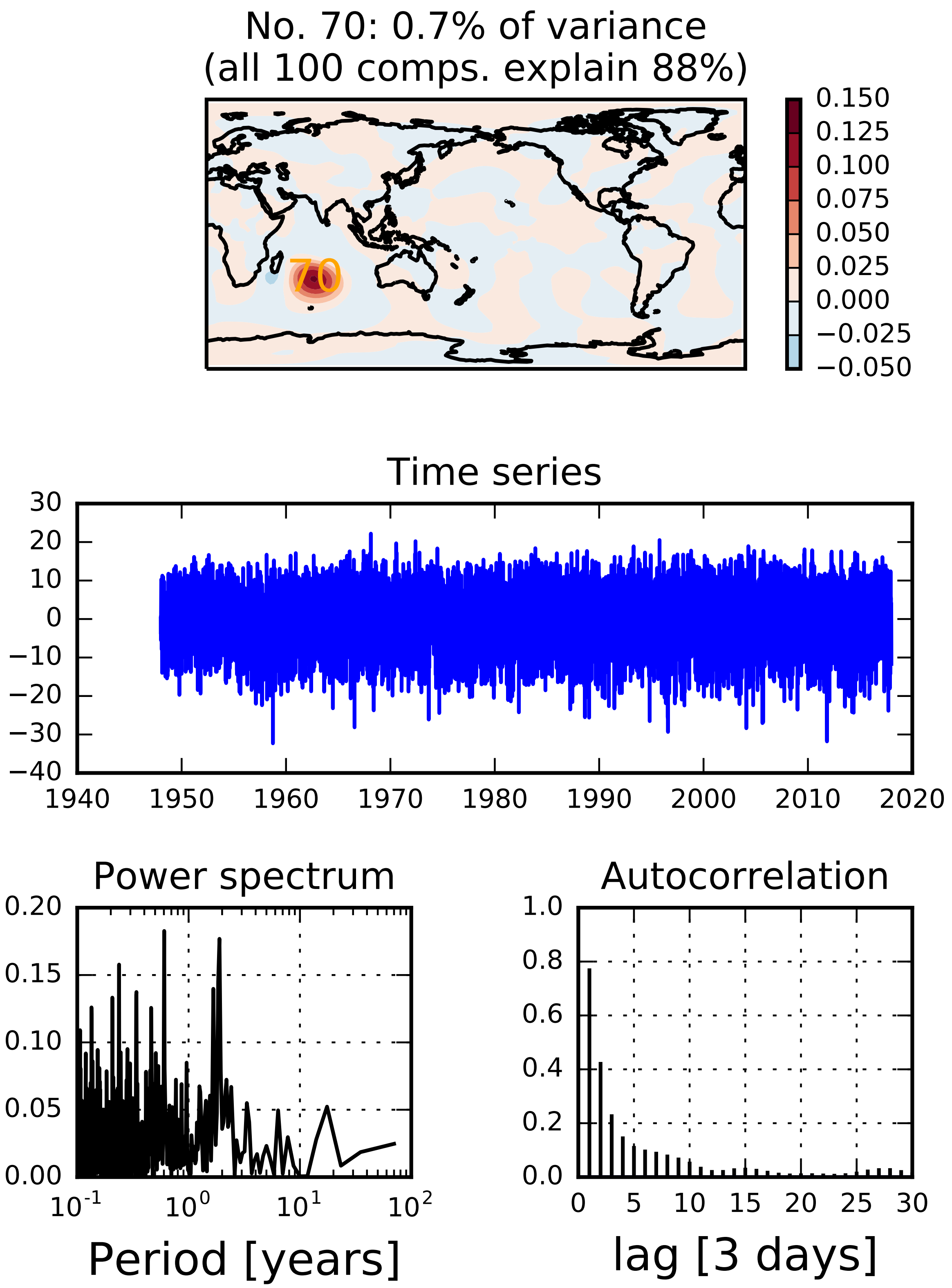
No. 70: 0.7% of variance
(all 100 comps. explain 88%)
70
0.150
0.125
0.100
0.075
0.050
0.025
0.000
−0.025
−0.050
Time series
30
20
10
0
−10
−20
−30
−40
1940
1950
1960
1970
1980
1990
2000
2010
2020
Power spectrum
0.20
0.15
0.10
0.05
0.00
10^{-1}
10^{0}
10^{1}
10^{2}
Period [years]
Autocorrelation
1.0
0.8
0.6
0.4
0.2
0.0
0
5
10
15
20
25
30
lag [3 days]

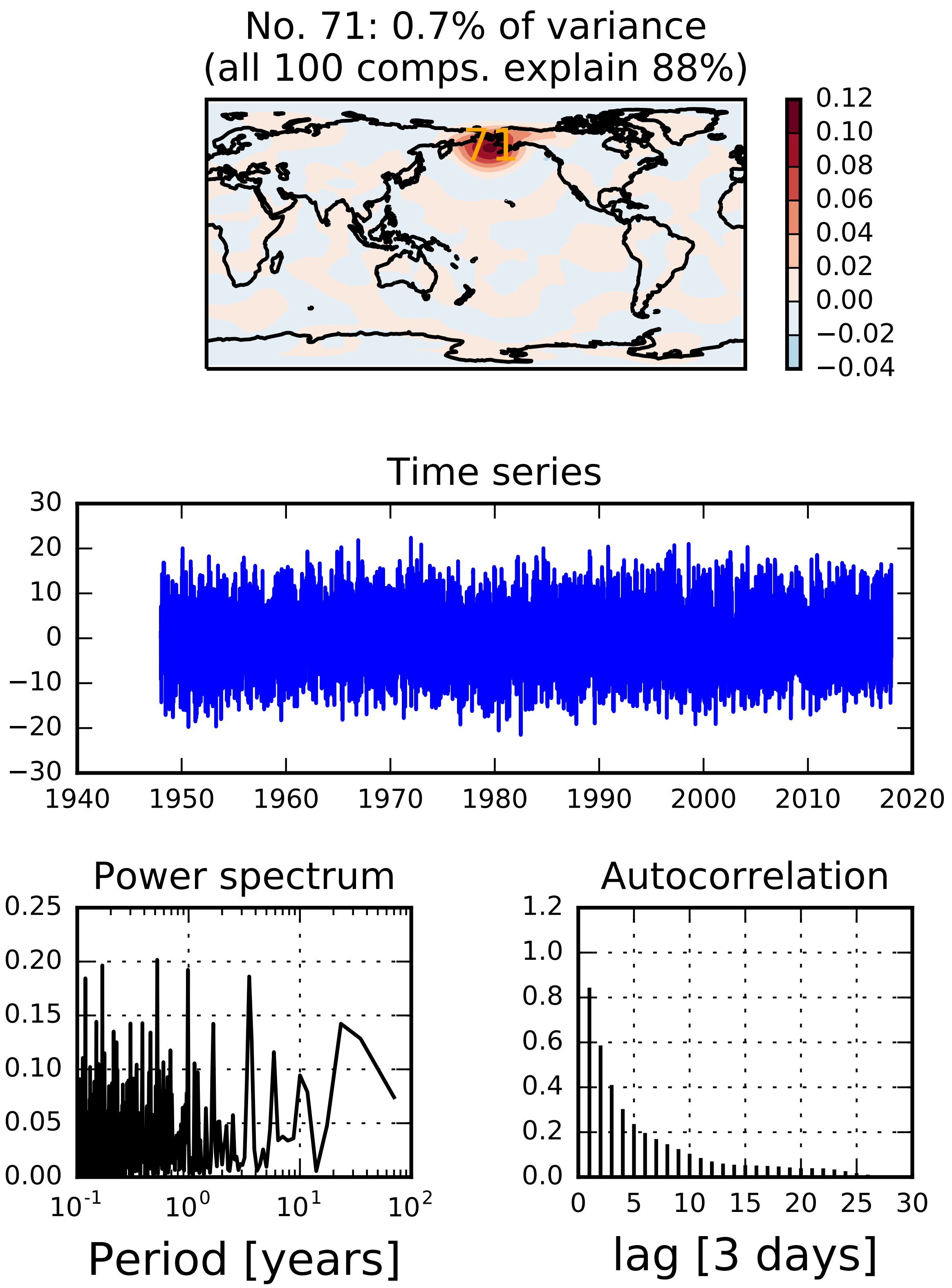
No. 71: 0.7% of variance
(all 100 comps. explain 88%)
71
0.12
0.10
0.08
0.06
0.04
0.02
0.00
−0.02
−0.04
Time series
30
20
10
0
−10
−20
−30
1940
1950
1960
1970
1980
1990
2000
2010
2020
Power spectrum
0.25
0.20
0.15
0.10
0.05
0.00
10^{-1}
10^{0}
10^{1}
10^{2}
Period [years]
Autocorrelation
1.2
1.0
0.8
0.6
0.4
0.2
0.0
0
5
10
15
20
25
30
lag [3 days]

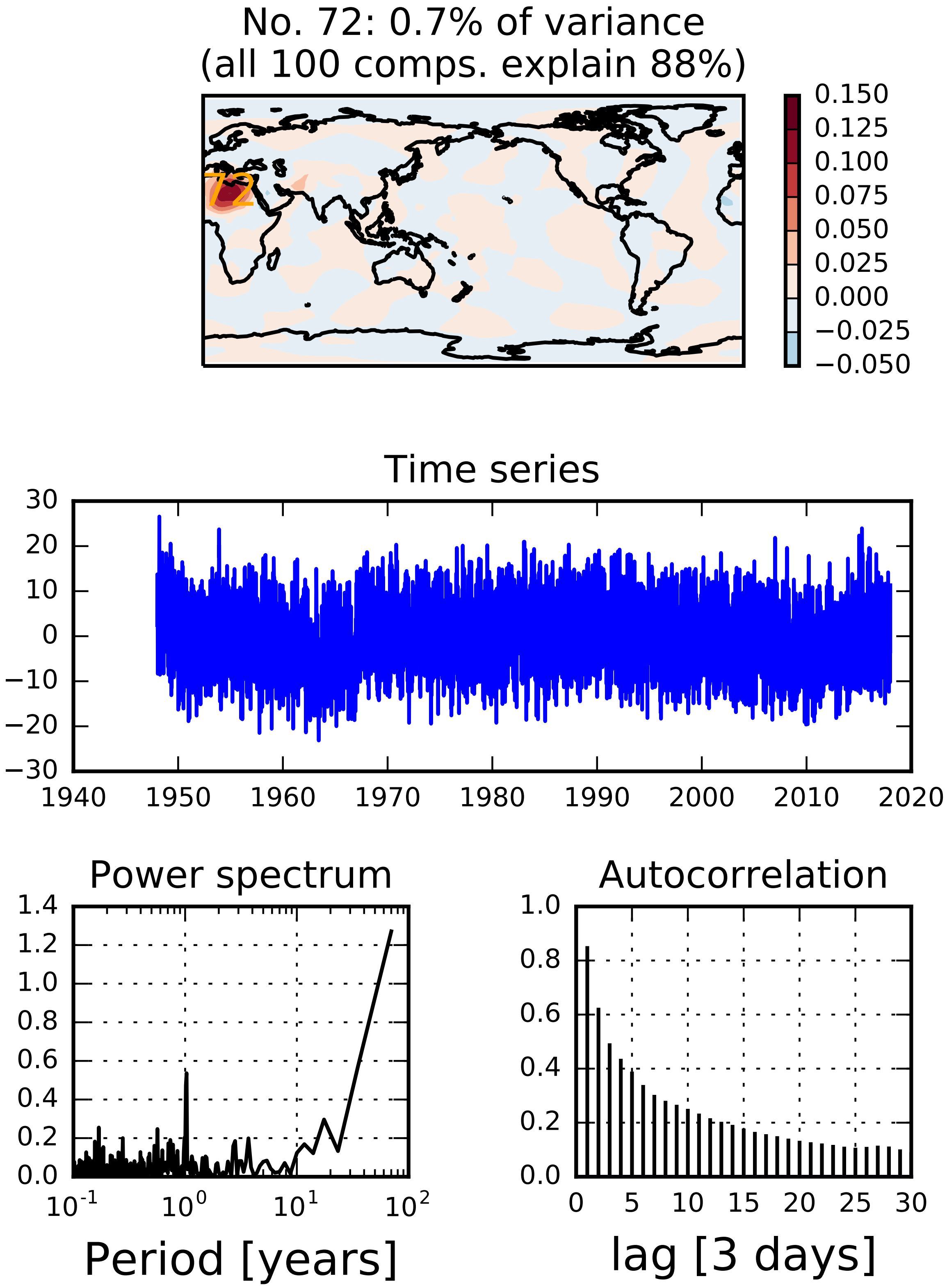

No. 72: 0.7% of variance
(all 100 comps. explain 88%)
72
0.150
0.125
0.100
0.075
0.050
0.025
0.000
−0.025
−0.050
Time series
30
20
10
0
−10
−20
−30
1940
1950
1960
1970
1980
1990
2000
2010
2020
Power spectrum
1.4
1.2
1.0
0.8
0.6
0.4
0.2
0.0
10^{-1}
10^{0}
10^{1}
10^{2}
Period [years]
Autocorrelation
1.0
0.8
0.6
0.4
0.2
0.0
0
5
10
15
20
25
30
lag [3 days]

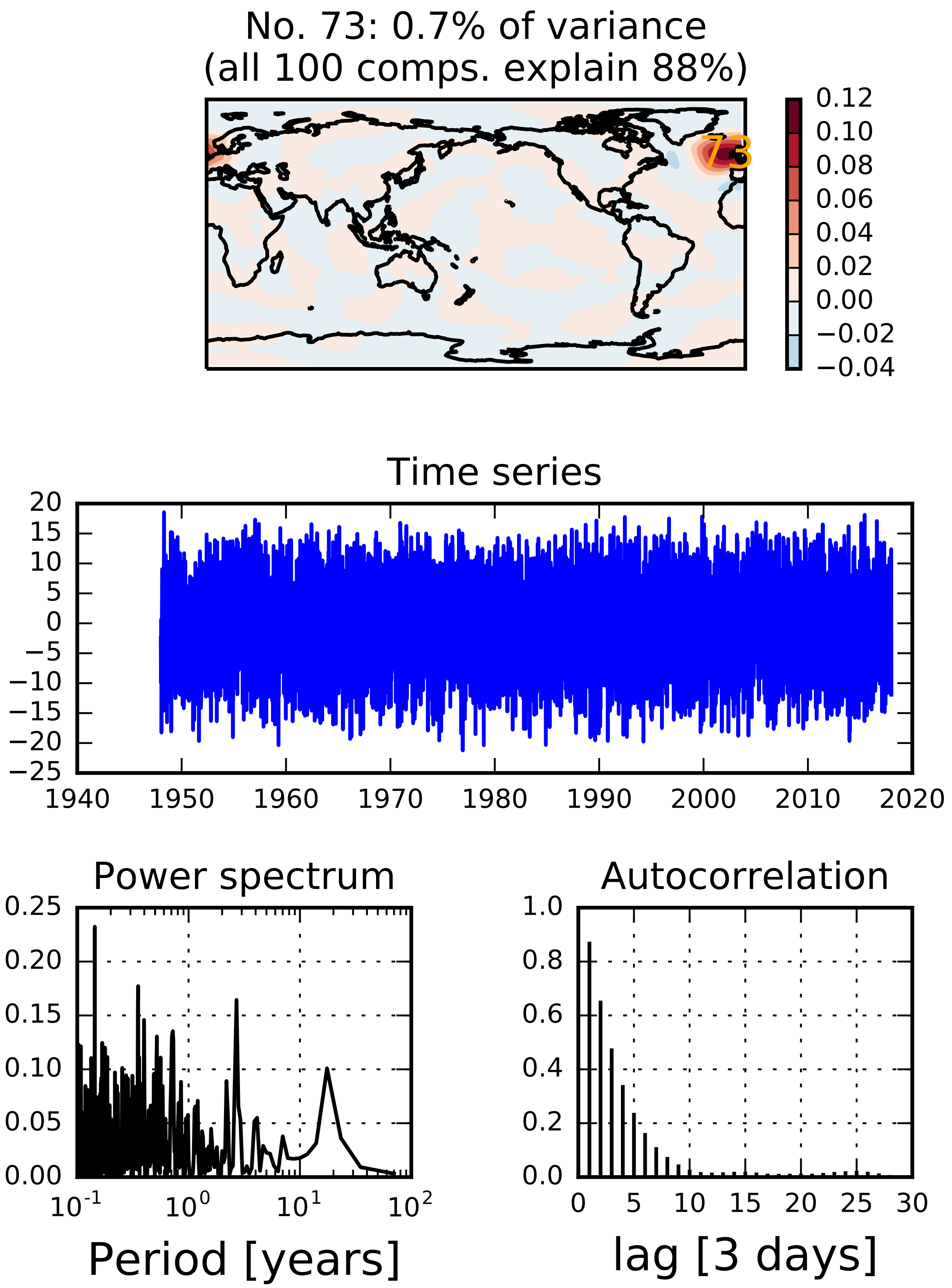
No. 73: 0.7% of variance
(all 100 comps. explain 88%)
73
0.12
0.10
0.08
0.06
0.04
0.02
0.00
−0.02
−0.04
Time series
20
15
10
5
0
−5
−10
−15
−20
−25
1940
1950
1960
1970
1980
1990
2000
2010
2020
Power spectrum
0.25
0.20
0.15
0.10
0.05
0.00
10^{-1}
10^{0}
10^{1}
10^{2}
Period [years]
Autocorrelation
1.0
0.8
0.6
0.4
0.2
0.0
0
5
10
15
20
25
30
lag [3 days]

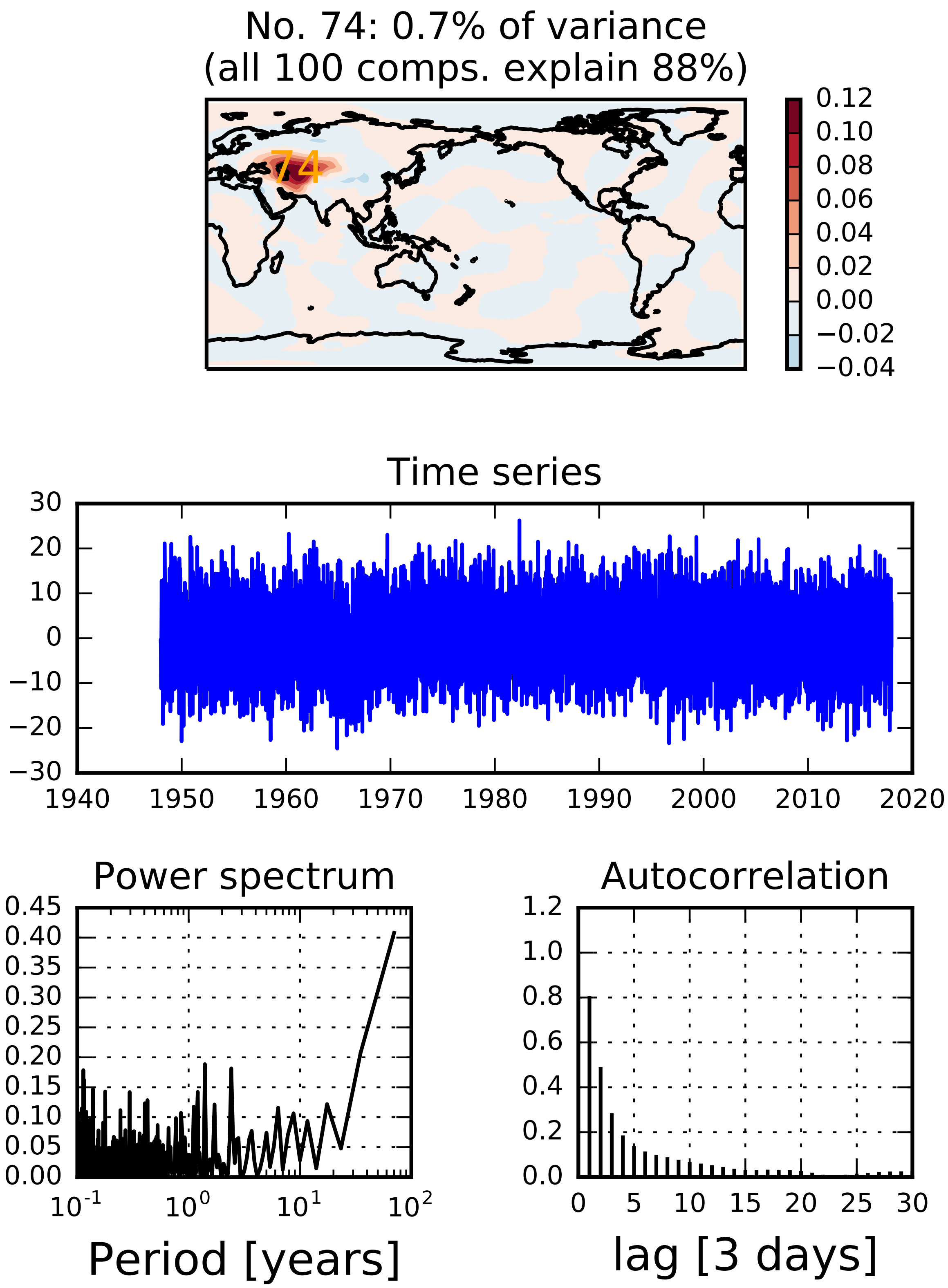
No. 74: 0.7% of variance
(all 100 comps. explain 88%)
74
0.12
0.10
0.08
0.06
0.04
0.02
0.00
−0.02
−0.04
Time series
30
20
10
0
−10
−20
−30
1940
1950
1960
1970
1980
1990
2000
2010
2020
Power spectrum
0.45
0.40
0.35
0.30
0.25
0.20
0.15
0.10
0.05
0.00
10^{-1}
10^{0}
10^{1}
10^{2}
Period [years]
Autocorrelation
1.2
1.0
0.8
0.6
0.4
0.2
0.0
0
5
10
15
20
25
30
lag [3 days]

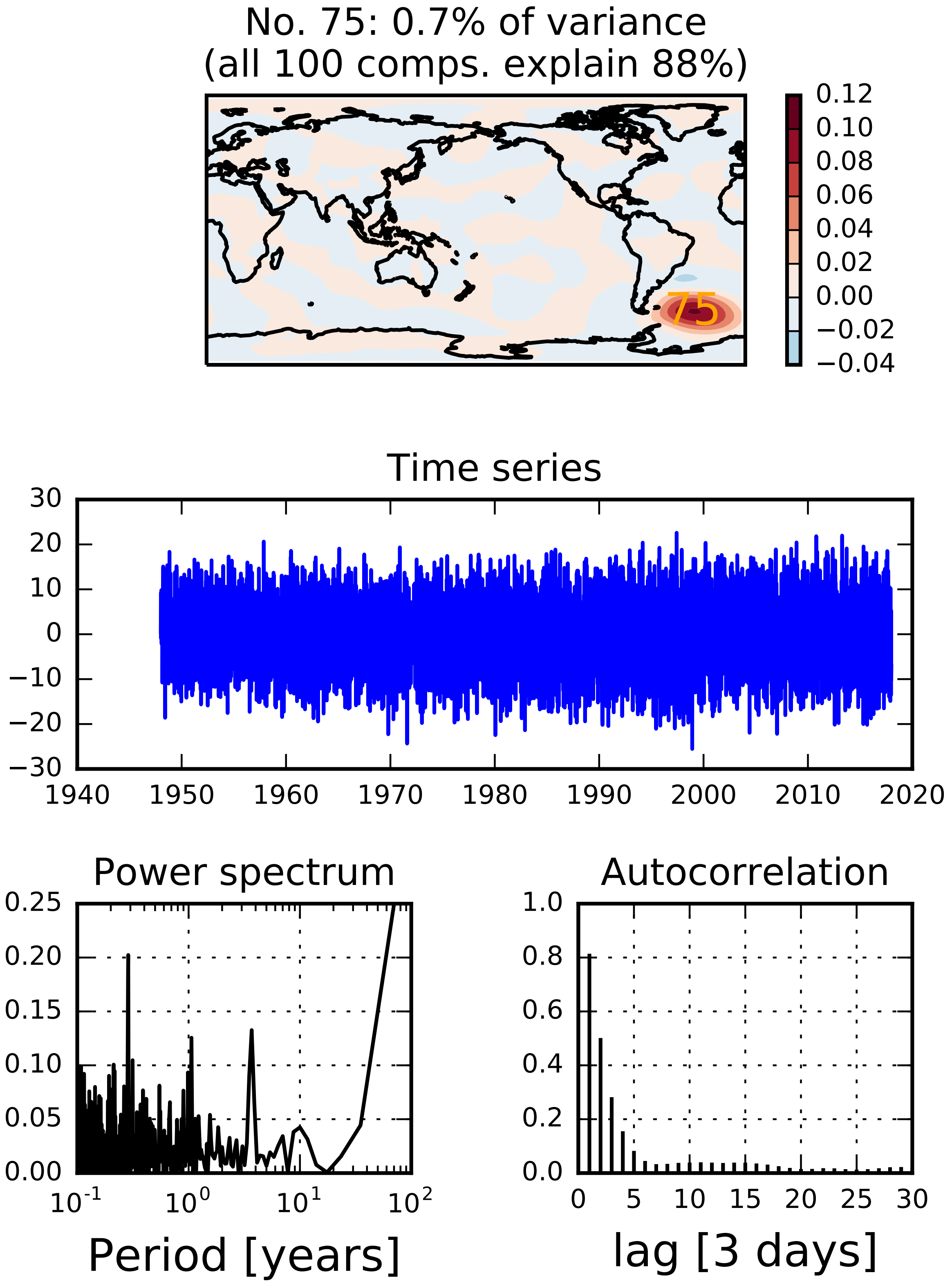
No. 75: 0.7% of variance
(all 100 comps. explain 88%)
75
0.12
0.10
0.08
0.06
0.04
0.02
0.00
−0.02
−0.04
Time series
30
20
10
0
−10
−20
−30
1940
1950
1960
1970
1980
1990
2000
2010
2020
Power spectrum
0.25
0.20
0.15
0.10
0.05
0.00
10^{-1}
10^{0}
10^{1}
10^{2}
Period [years]
Autocorrelation
1.0
0.8
0.6
0.4
0.2
0.0
0
5
10
15
20
25
30
lag [3 days]

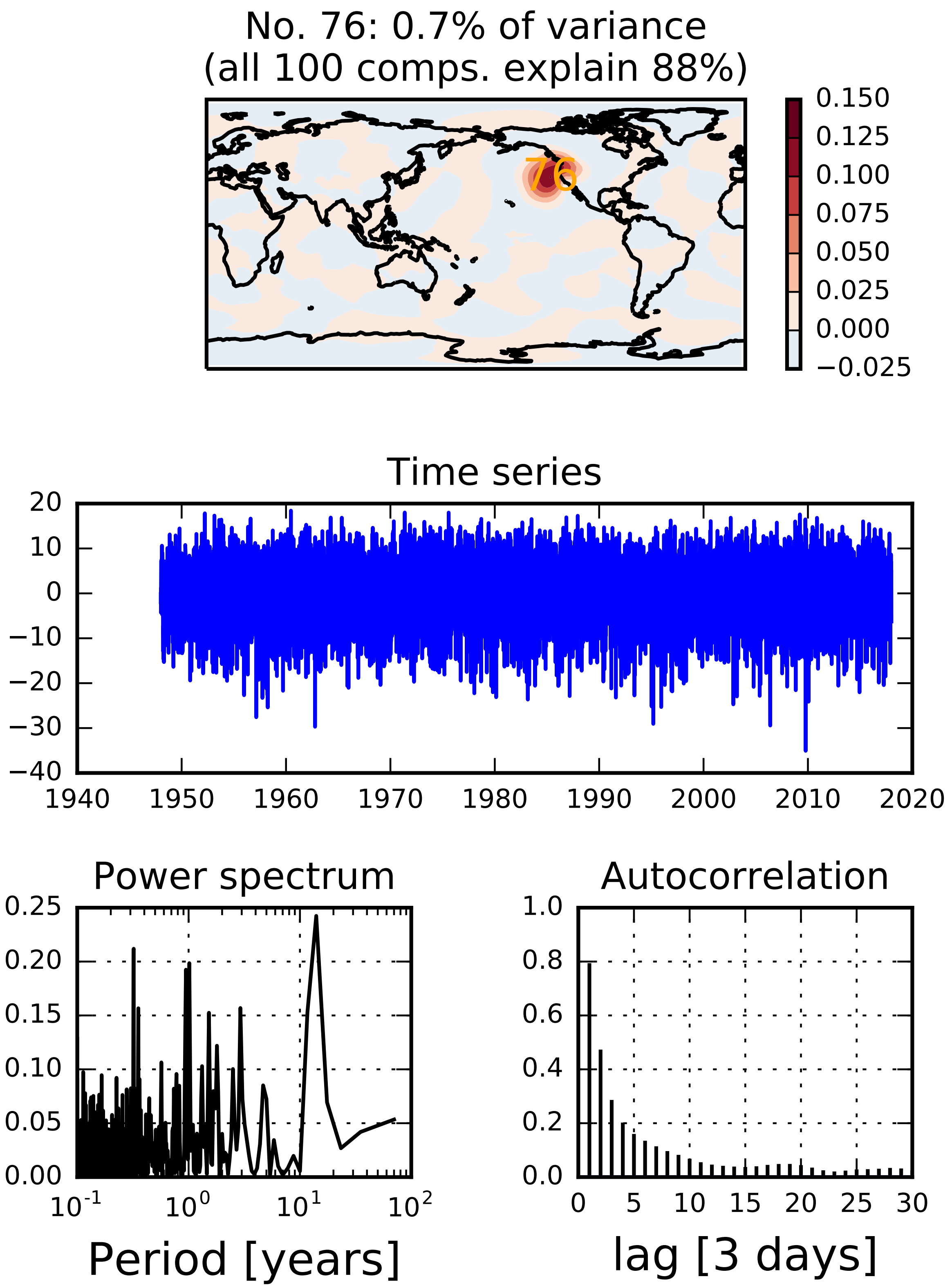
No. 76: 0.7% of variance
(all 100 comps. explain 88%)
76
0.150
0.125
0.100
0.075
0.050
0.025
0.000
−0.025
Time series
20
10
0
−10
−20
−30
−40
1940
1950
1960
1970
1980
1990
2000
2010
2020
Power spectrum
0.25
0.20
0.15
0.10
0.05
0.00
10^-1
10^0
10^1
10^2
Period [years]
Autocorrelation
1.0
0.8
0.6
0.4
0.2
0.0
0
5
10
15
20
25
30
lag [3 days]

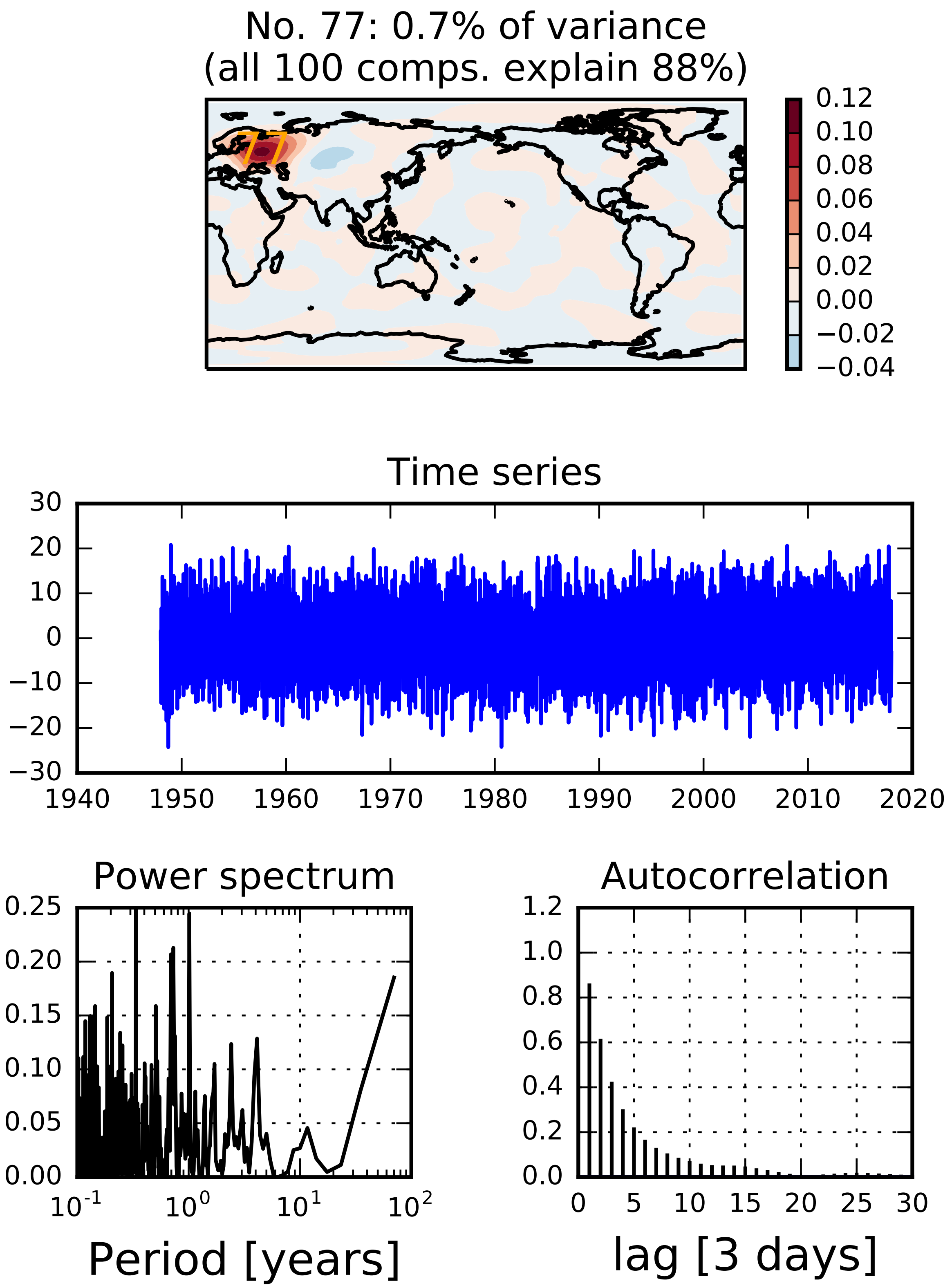
No. 77: 0.7% of variance
(all 100 comps. explain 88%)
0.12
0.10
0.08
0.06
0.04
0.02
0.00
−0.02
−0.04
Time series
30
20
10
0
−10
−20
−30
1940
1950
1960
1970
1980
1990
2000
2010
2020
Power spectrum
0.25
0.20
0.15
0.10
0.05
0.00
Period [years]
Autocorrelation
1.2
1.0
0.8
0.6
0.4
0.2
0.0
0
5
10
15
20
25
30
lag [3 days]

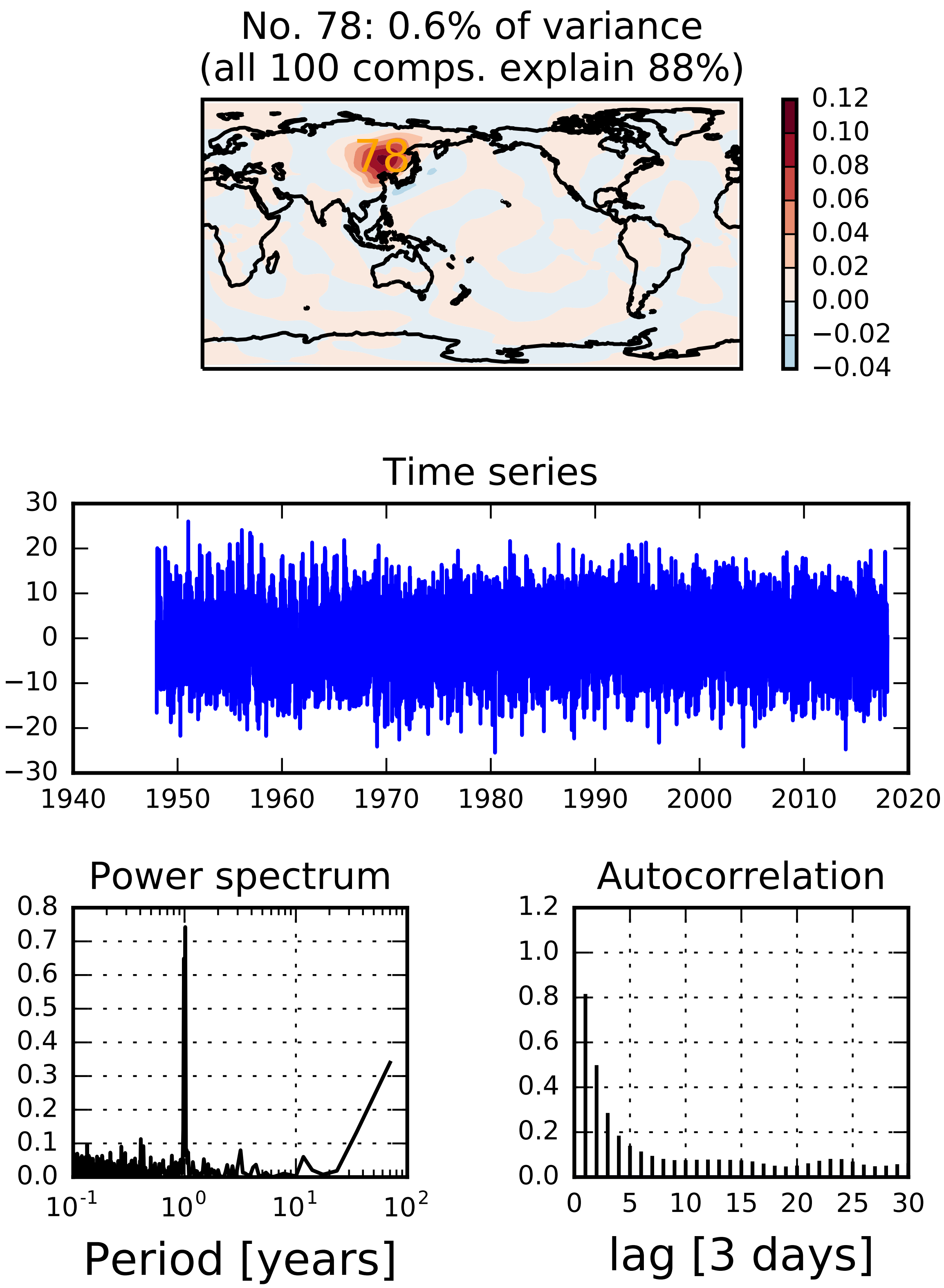
No. 78: 0.6% of variance
(all 100 comps. explain 88%)
78
0.12
0.10
0.08
0.06
0.04
0.02
0.00
−0.02
−0.04
Time series
30
20
10
0
−10
−20
−30
1940
1950
1960
1970
1980
1990
2000
2010
2020
Power spectrum
0.8
0.7
0.6
0.5
0.4
0.3
0.2
0.1
0.0
10^-1
10^0
10^1
10^2
Period [years]
Autocorrelation
1.2
1.0
0.8
0.6
0.4
0.2
0.0
0
5
10
15
20
25
30
lag [3 days]

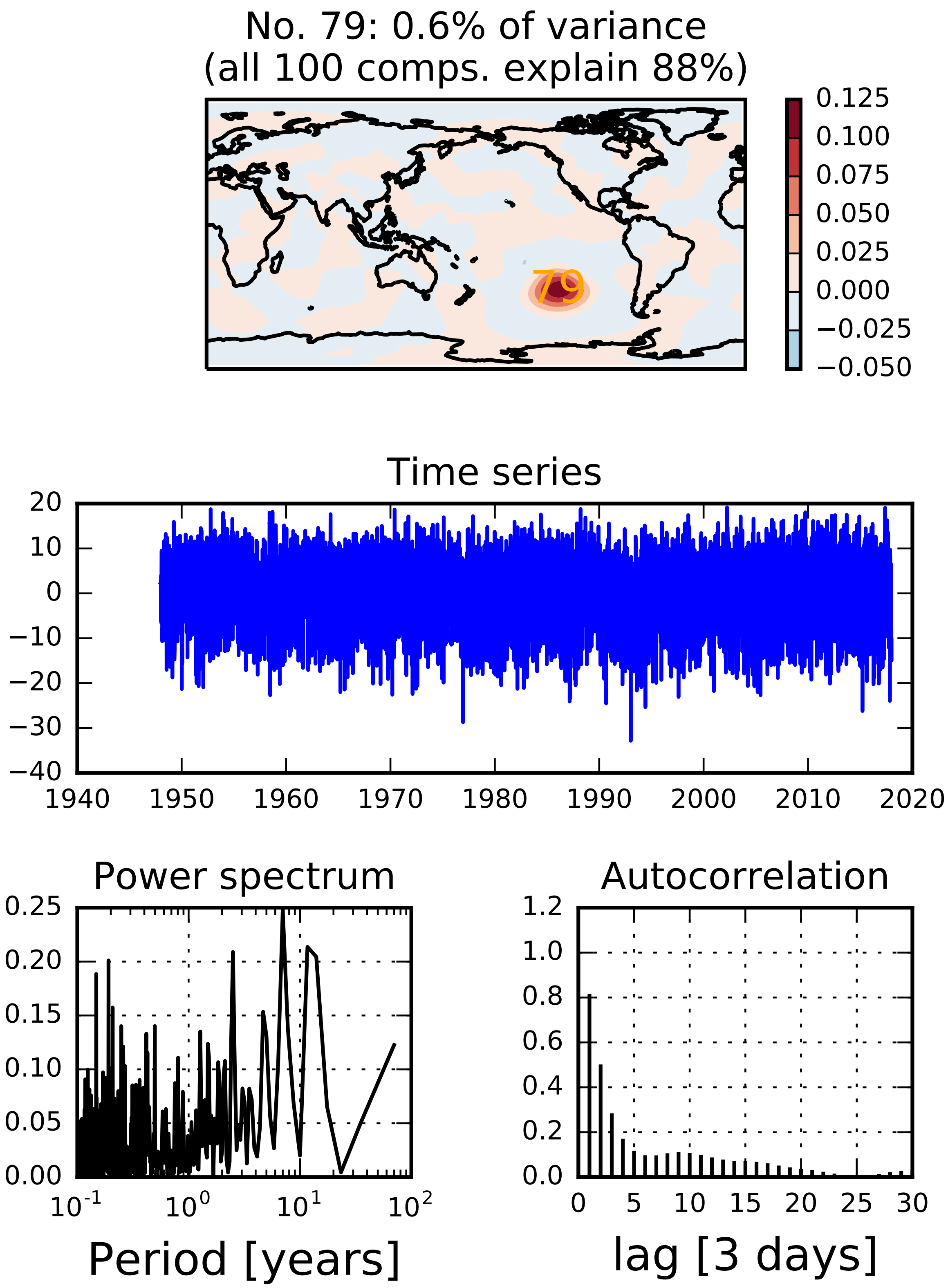
No. 79: 0.6% of variance
(all 100 comps. explain 88%)
0.125
0.100
0.075
0.050
0.025
0.000
−0.025
−0.050
79
Time series
20
10
0
−10
−20
−30
−40
1940
1950
1960
1970
1980
1990
2000
2010
2020
Power spectrum
0.25
0.20
0.15
0.10
0.05
0.00
10^-1
10^0
10^1
10^2
Period [years]
Autocorrelation
1.2
1.0
0.8
0.6
0.4
0.2
0.0
0
5
10
15
20
25
30
lag [3 days]

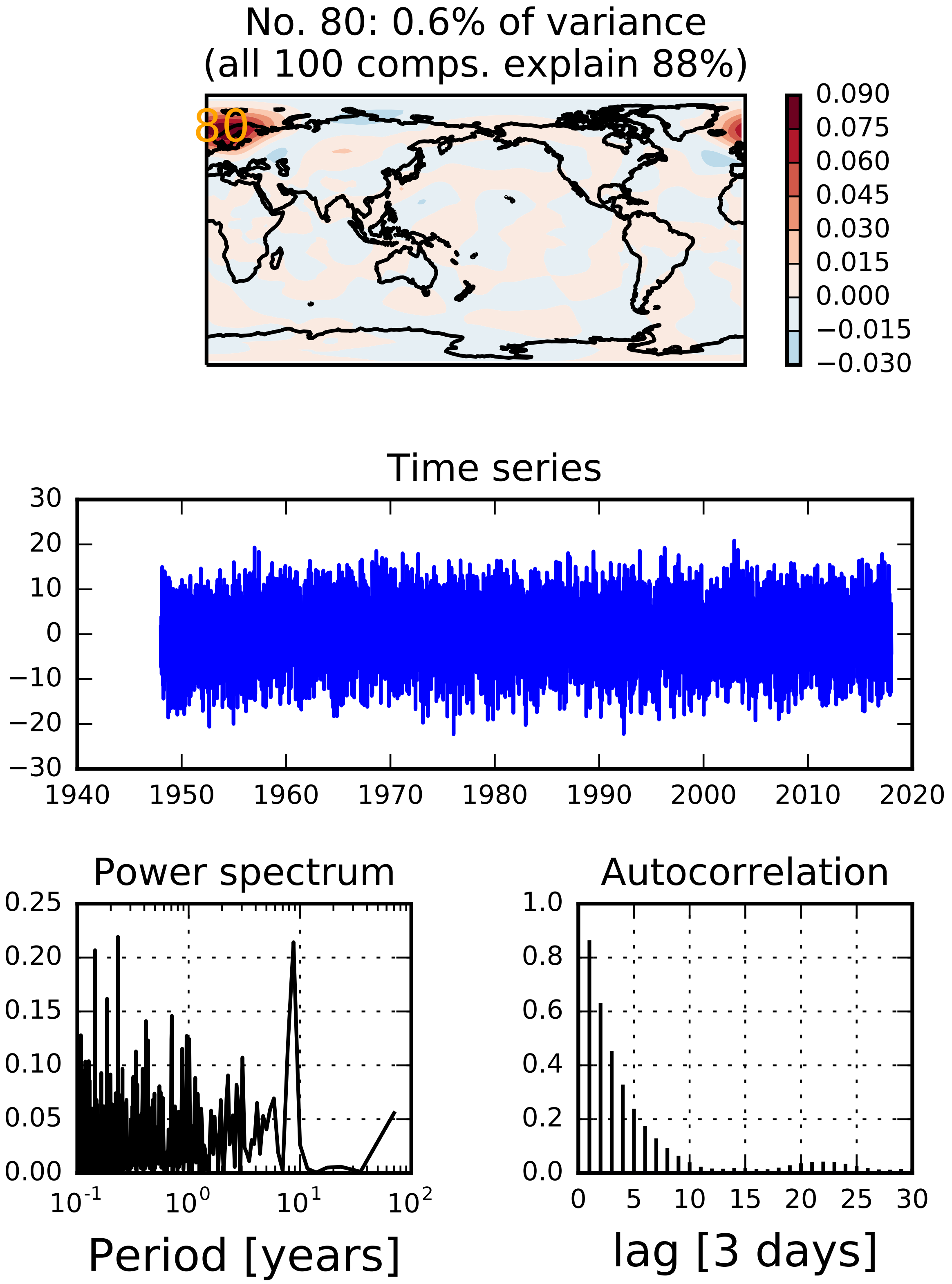

No. 80: 0.6% of variance
(all 100 comps. explain 88%)
80
0.090
0.075
0.060
0.045
0.030
0.015
0.000
−0.015
−0.030
Time series
30
20
10
0
−10
−20
−30
1940
1950
1960
1970
1980
1990
2000
2010
2020
Power spectrum
0.25
0.20
0.15
0.10
0.05
0.00
10^{-1}
10^{0}
10^{1}
10^{2}
Period [years]
Autocorrelation
1.0
0.8
0.6
0.4
0.2
0.0
0
5
10
15
20
25
30
lag [3 days]

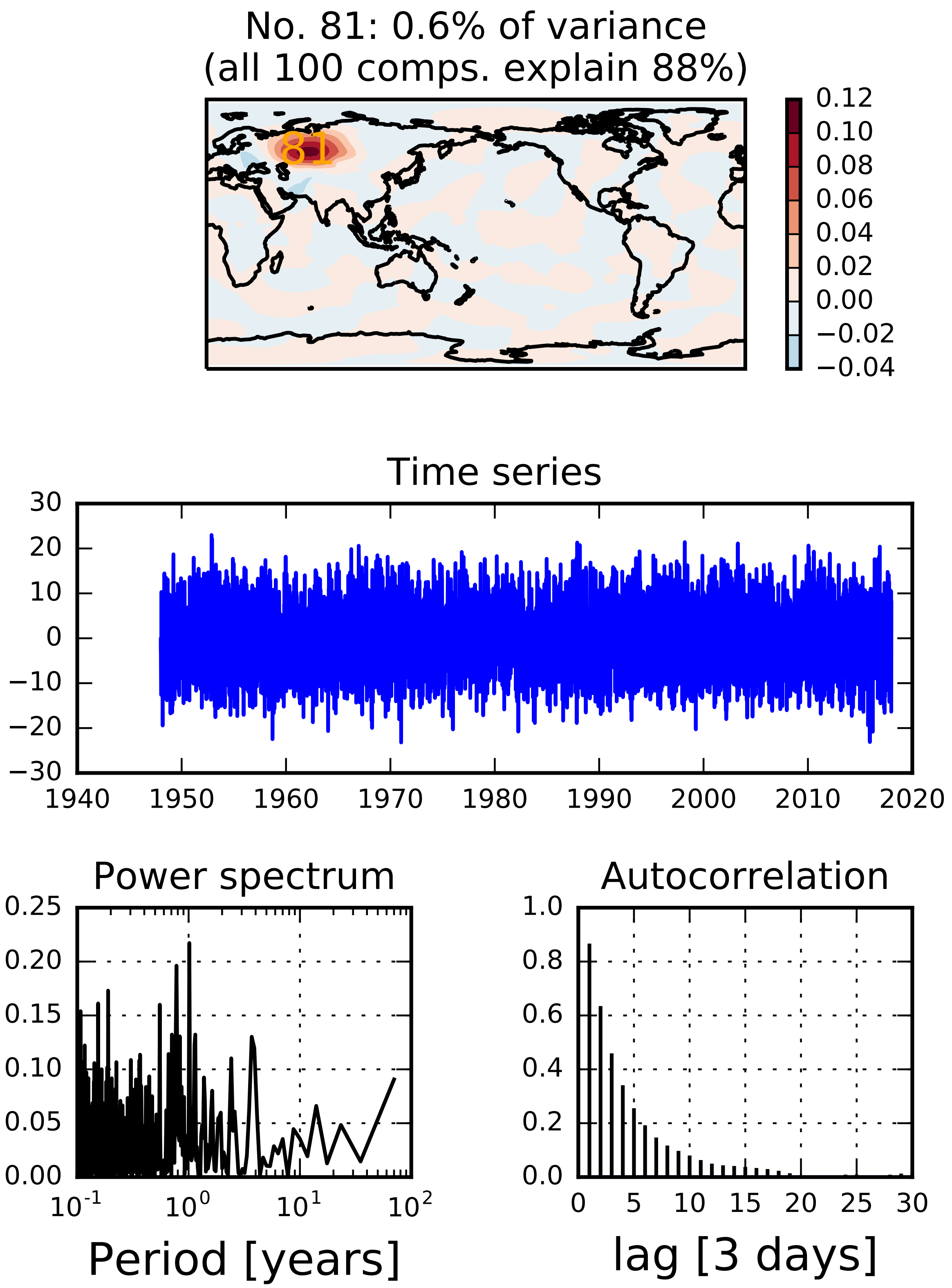
No. 81: 0.6% of variance
(all 100 comps. explain 88%)
81
0.12
0.10
0.08
0.06
0.04
0.02
0.00
−0.02
−0.04
Time series
30
20
10
0
−10
−20
−30
1940
1950
1960
1970
1980
1990
2000
2010
2020
Power spectrum
0.25
0.20
0.15
0.10
0.05
0.00
10^-1
10^0
10^1
10^2
Period [years]
Autocorrelation
1.0
0.8
0.6
0.4
0.2
0.0
0
5
10
15
20
25
30
lag [3 days]

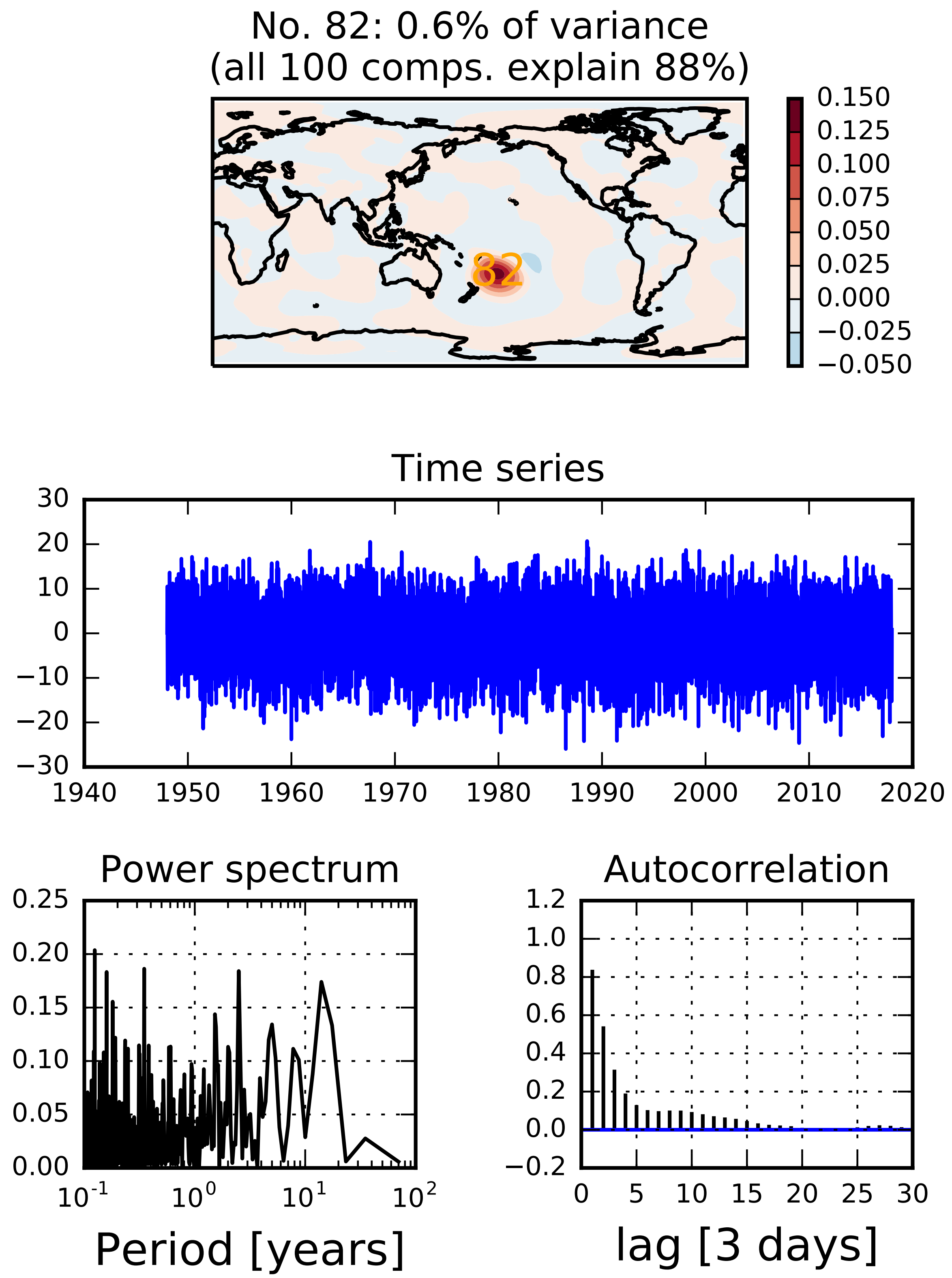
No. 82: 0.6% of variance
(all 100 comps. explain 88%)
82
0.150
0.125
0.100
0.075
0.050
0.025
0.000
−0.025
−0.050
Time series
30
20
10
0
−10
−20
−30
1940
1950
1960
1970
1980
1990
2000
2010
2020
Power spectrum
0.25
0.20
0.15
0.10
0.05
0.00
10^{-1}
10^{0}
10^{1}
10^{2}
Period [years]
Autocorrelation
1.2
1.0
0.8
0.6
0.4
0.2
0.0
−0.2
0
5
10
15
20
25
30
lag [3 days]

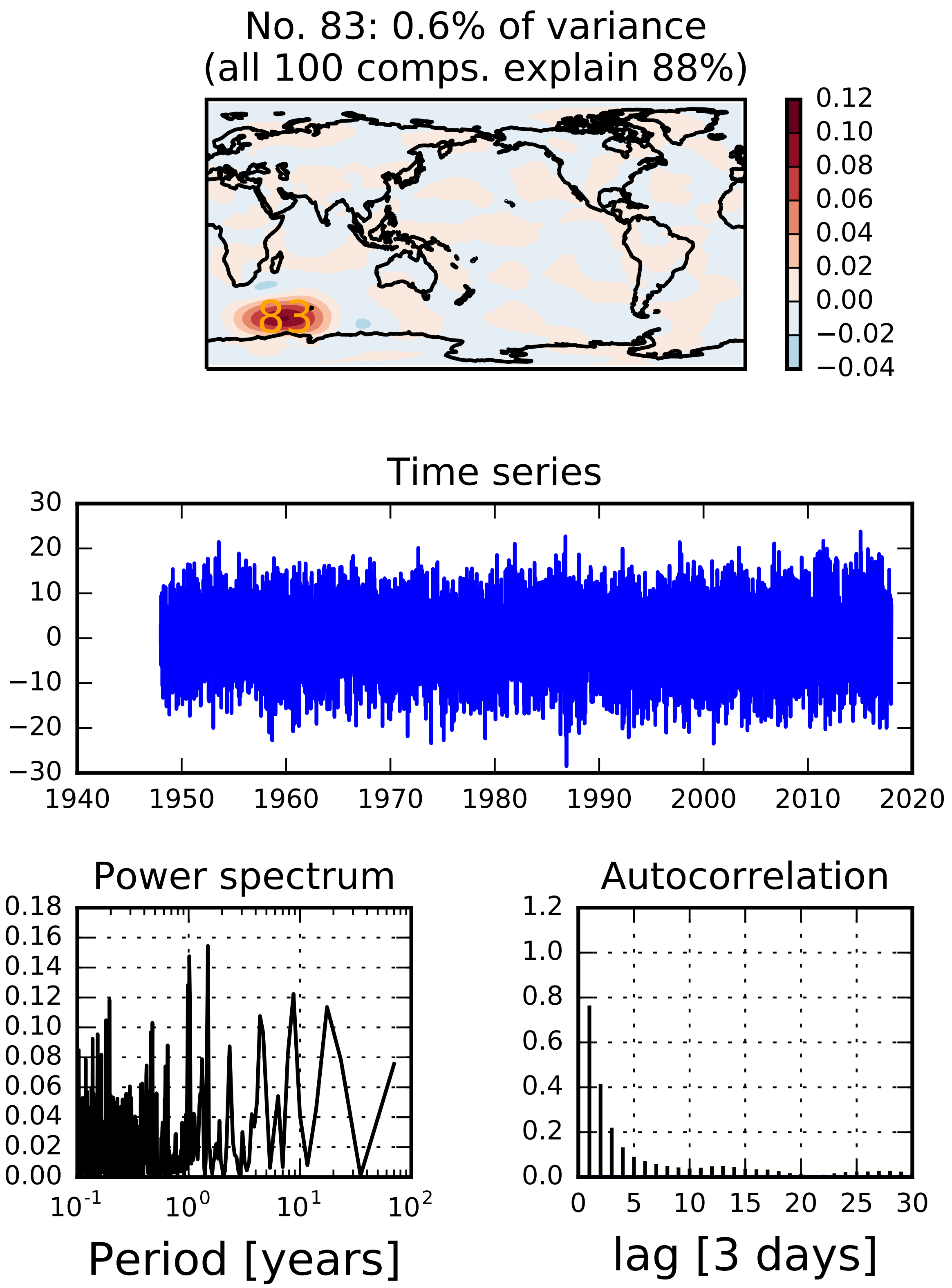
No. 83: 0.6% of variance
(all 100 comps. explain 88%)
83
0.12
0.10
0.08
0.06
0.04
0.02
0.00
−0.02
−0.04
Time series
30
20
10
0
−10
−20
−30
1940
1950
1960
1970
1980
1990
2000
2010
2020
Power spectrum
0.18
0.16
0.14
0.12
0.10
0.08
0.06
0.04
0.02
0.00
10^{-1}
10^{0}
10^{1}
10^{2}
Period [years]
Autocorrelation
1.2
1.0
0.8
0.6
0.4
0.2
0.0
0
5
10
15
20
25
30
lag [3 days]

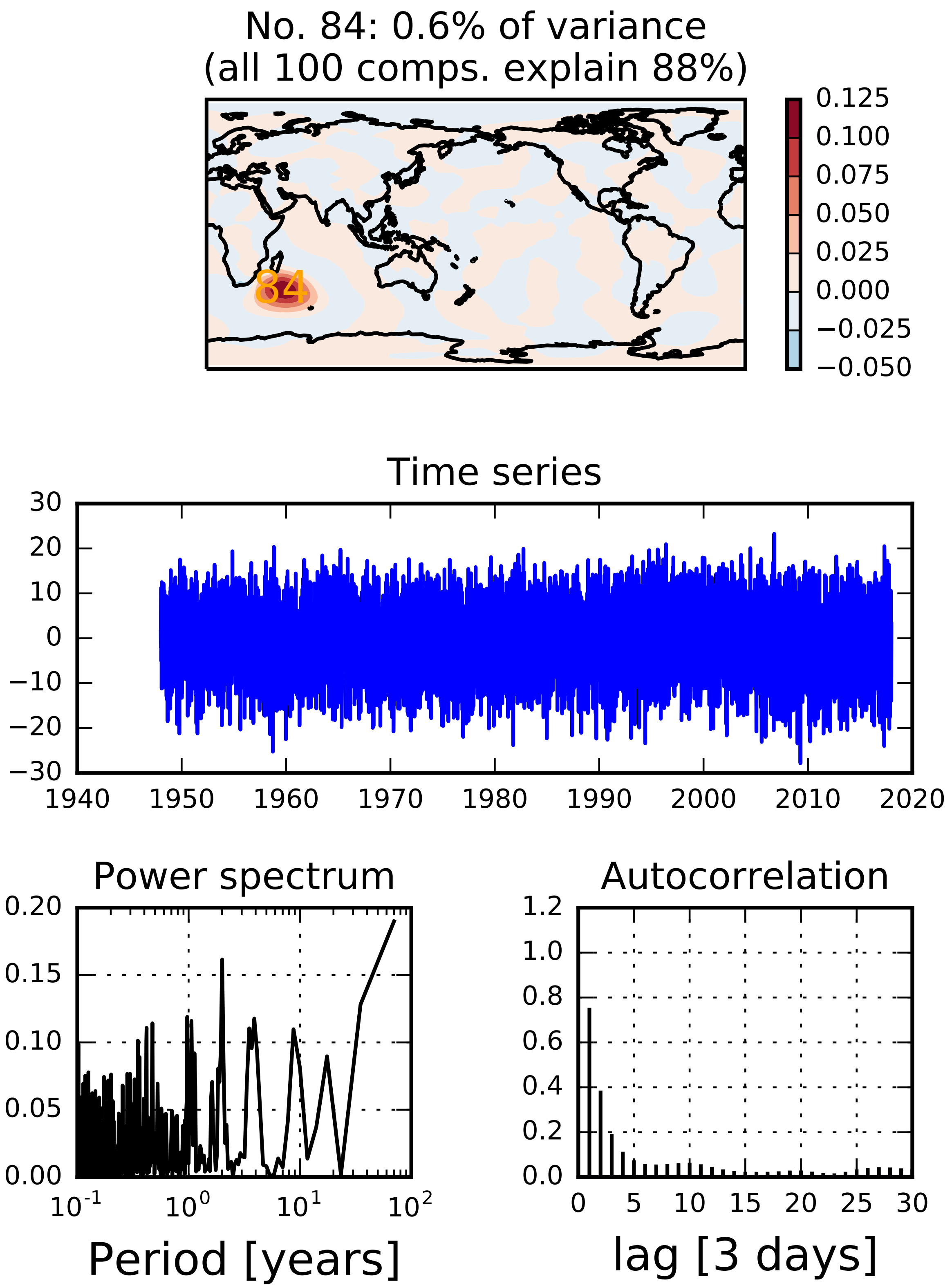
No. 84: 0.6% of variance
(all 100 comps. explain 88%)
84
0.125
0.100
0.075
0.050
0.025
0.000
−0.025
−0.050
Time series
30
20
10
0
−10
−20
−30
1940
1950
1960
1970
1980
1990
2000
2010
2020
Power spectrum
0.20
0.15
0.10
0.05
0.00
10^-1
10^0
10^1
10^2
Period [years]
Autocorrelation
1.2
1.0
0.8
0.6
0.4
0.2
0.0
0
5
10
15
20
25
30
lag [3 days]

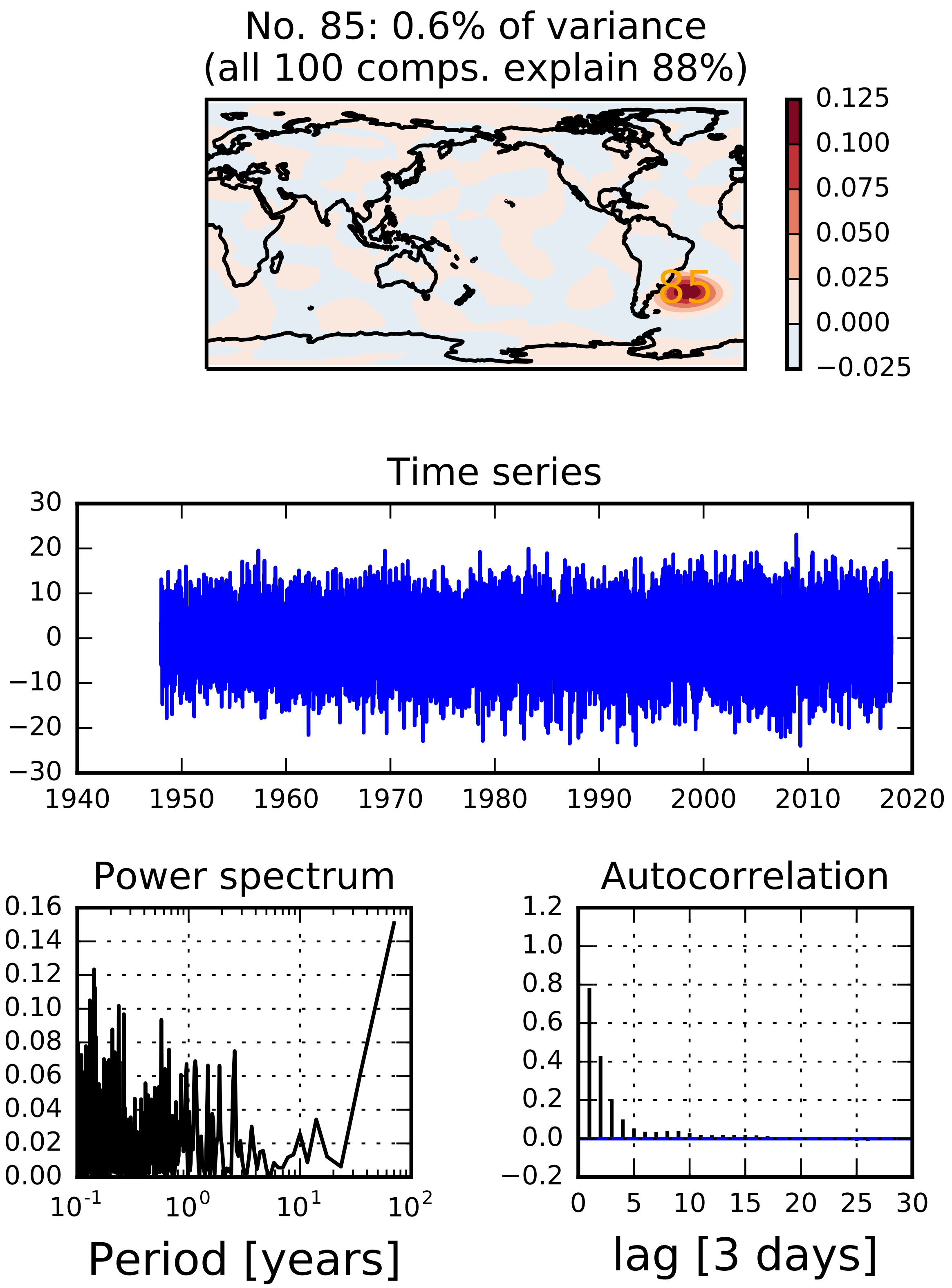
No. 85: 0.6% of variance
(all 100 comps. explain 88%)
85
0.125
0.100
0.075
0.050
0.025
0.000
−0.025
Time series
30
20
10
0
−10
−20
−30
1940
1950
1960
1970
1980
1990
2000
2010
2020
Power spectrum
0.16
0.14
0.12
0.10
0.08
0.06
0.04
0.02
0.00
10^-1
10^0
10^1
10^2
Period [years]
Autocorrelation
1.2
1.0
0.8
0.6
0.4
0.2
0.0
−0.2
0
5
10
15
20
25
30
lag [3 days]

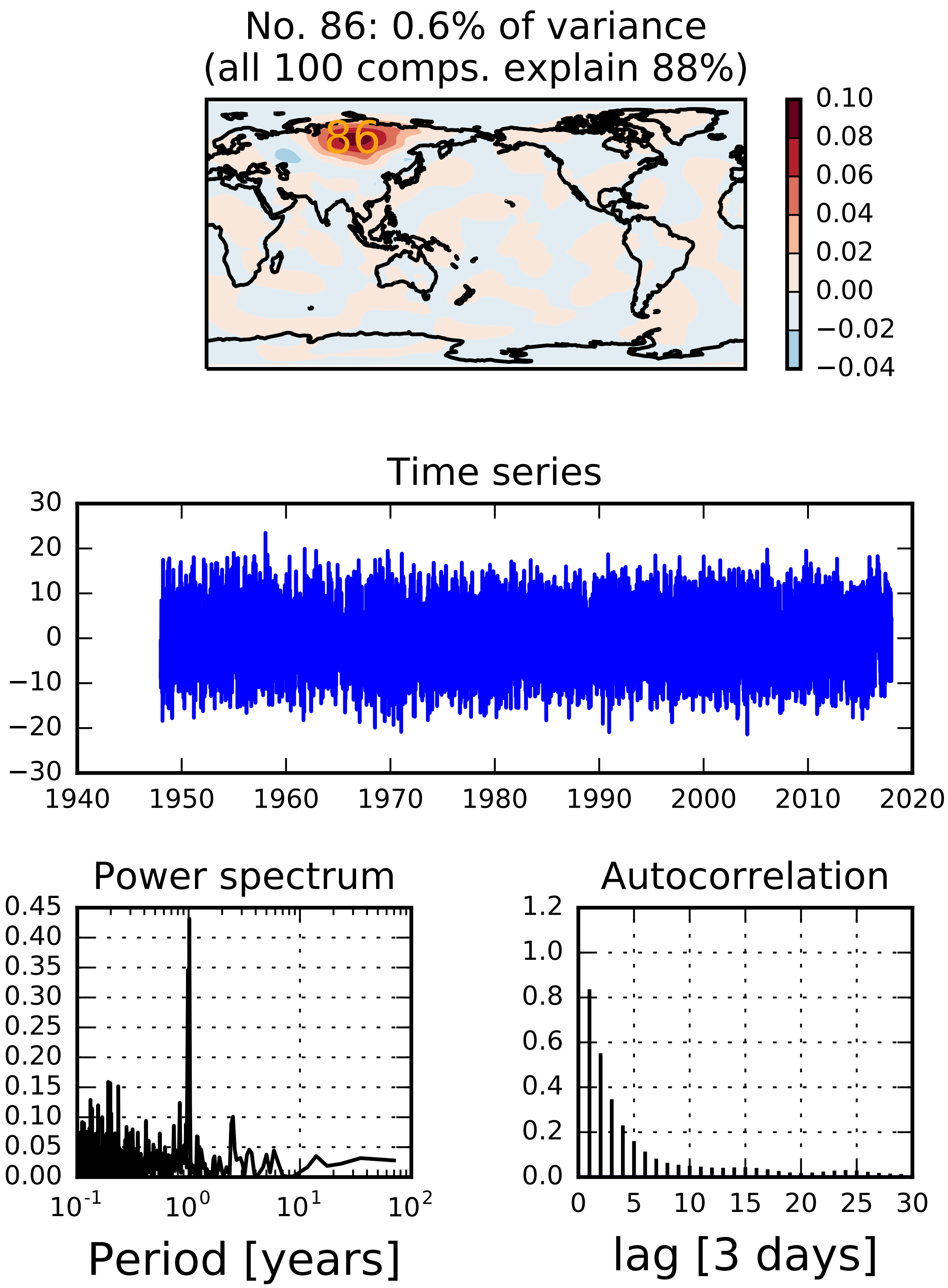
No. 86: 0.6% of variance
(all 100 comps. explain 88%)
86
0.10
0.08
0.06
0.04
0.02
0.00
−0.02
−0.04
Time series
30
20
10
0
−10
−20
−30
1940
1950
1960
1970
1980
1990
2000
2010
2020
Power spectrum
0.45
0.40
0.35
0.30
0.25
0.20
0.15
0.10
0.05
0.00
10^-1
10^0
10^1
10^2
Period [years]
Autocorrelation
1.2
1.0
0.8
0.6
0.4
0.2
0.0
0
5
10
15
20
25
30
lag [3 days]

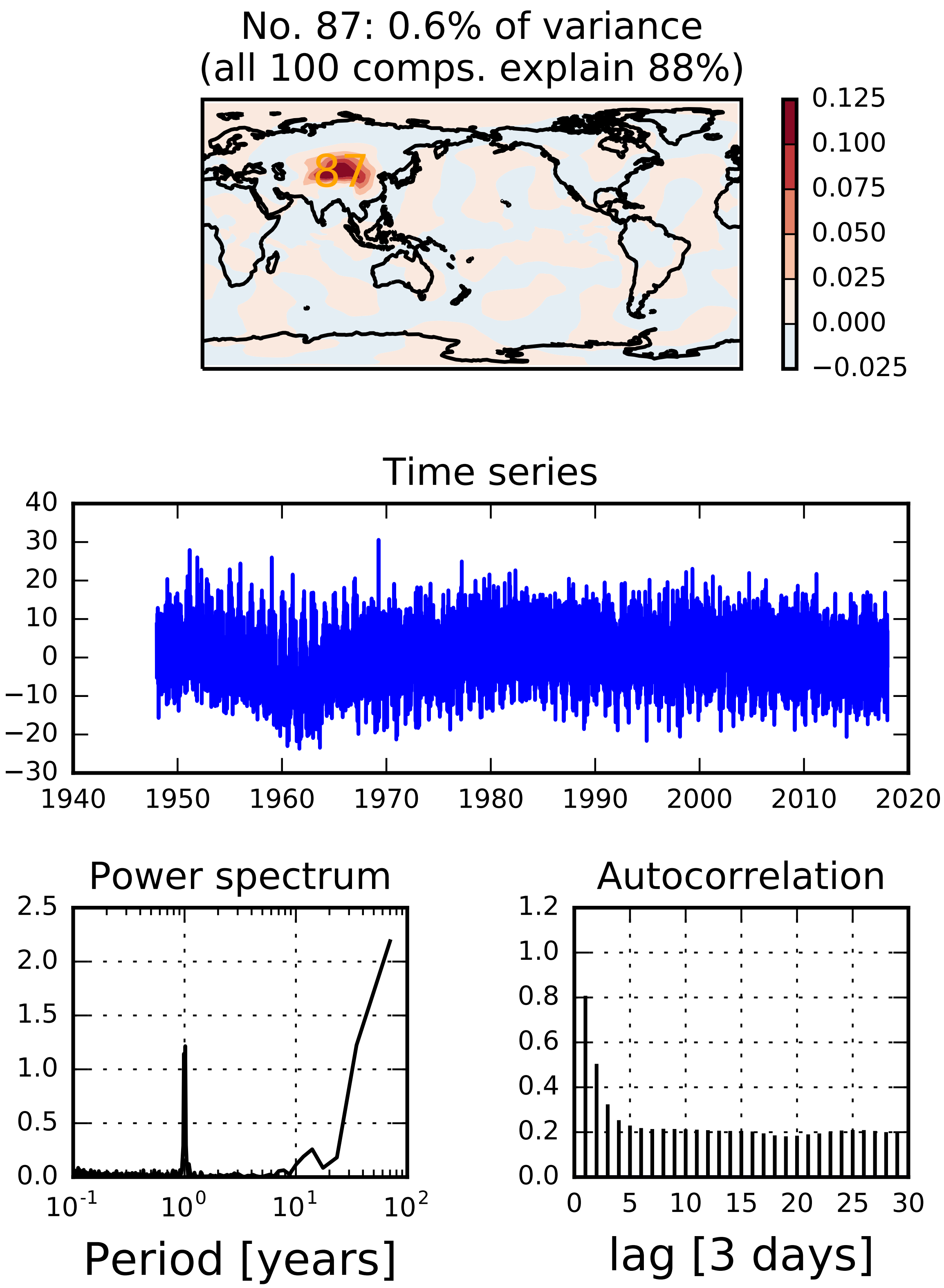
No. 87: 0.6% of variance
(all 100 comps. explain 88%)
87
0.125
0.100
0.075
0.050
0.025
0.000
−0.025
Time series
40
30
20
10
0
−10
−20
−30
1940
1950
1960
1970
1980
1990
2000
2010
2020
Power spectrum
2.5
2.0
1.5
1.0
0.5
0.0
10^-1
10^0
10^1
10^2
Period [years]
Autocorrelation
1.2
1.0
0.8
0.6
0.4
0.2
0.0
0
5
10
15
20
25
30
lag [3 days]

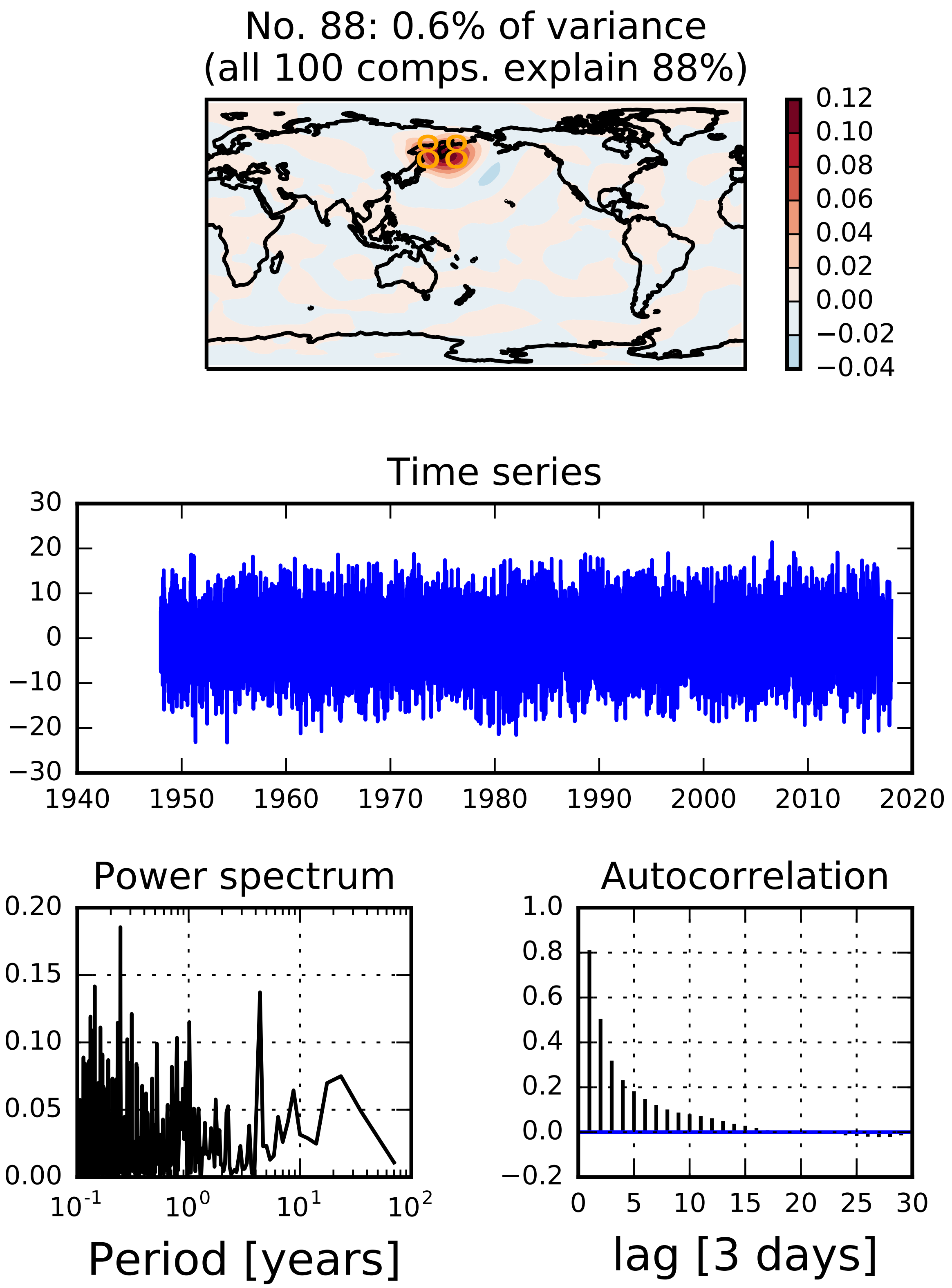
No. 88: 0.6% of variance
(all 100 comps. explain 88%)
88
0.12
0.10
0.08
0.06
0.04
0.02
0.00
−0.02
−0.04
Time series
30
20
10
0
−10
−20
−30
1940
1950
1960
1970
1980
1990
2000
2010
2020
Power spectrum
0.20
0.15
0.10
0.05
0.00
10^-1
10^0
10^1
10^2
Period [years]
Autocorrelation
1.0
0.8
0.6
0.4
0.2
0.0
−0.2
0
5
10
15
20
25
30
lag [3 days]

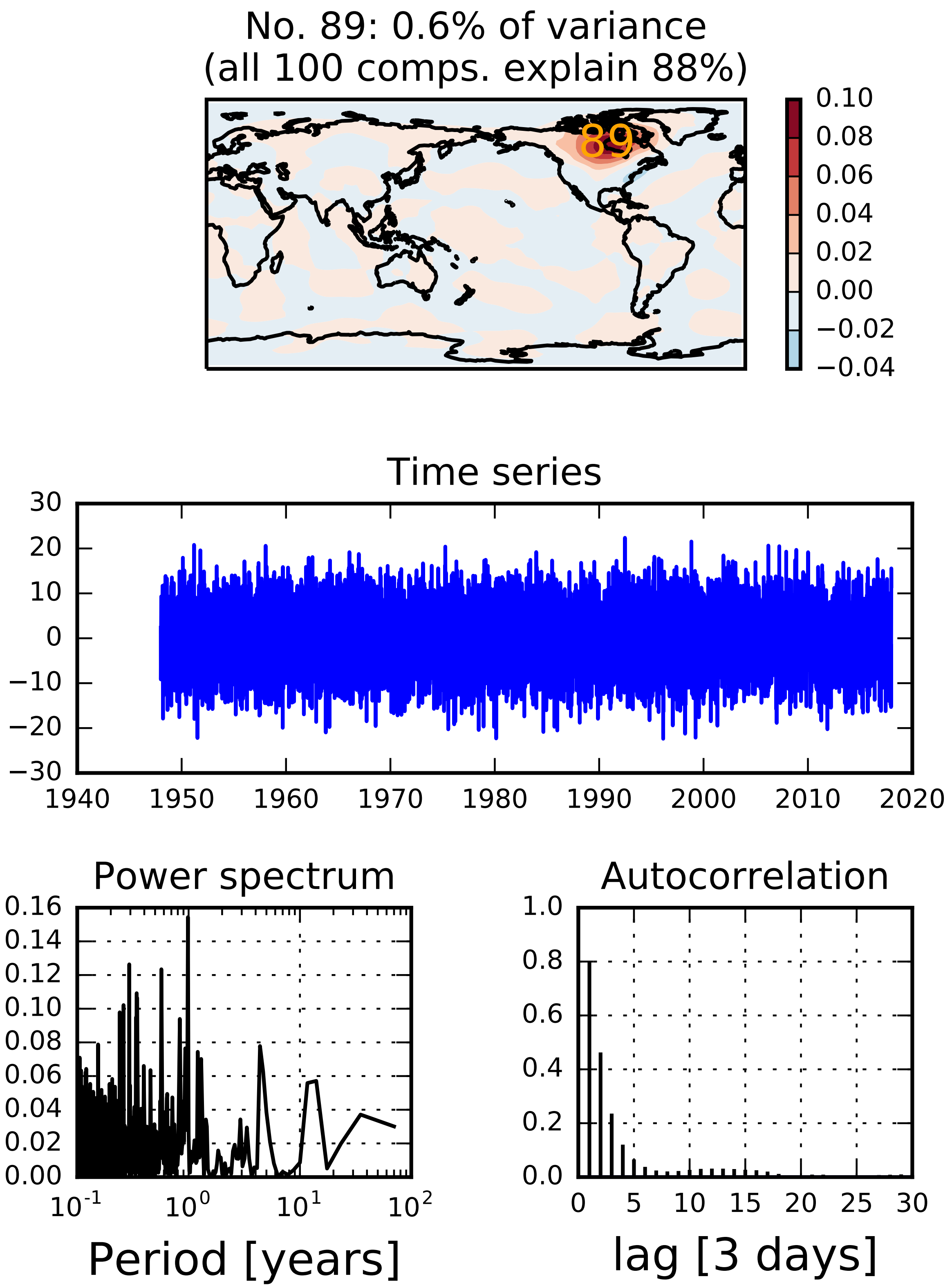
No. 89: 0.6% of variance
(all 100 comps. explain 88%)
89
0.10
0.08
0.06
0.04
0.02
0.00
−0.02
−0.04
Time series
30
20
10
0
−10
−20
−30
1940
1950
1960
1970
1980
1990
2000
2010
2020
Power spectrum
0.16
0.14
0.12
0.10
0.08
0.06
0.04
0.02
0.00
10^-1
10^0
10^1
10^2
Period [years]
Autocorrelation
1.0
0.8
0.6
0.4
0.2
0.0
0
5
10
15
20
25
30
lag [3 days]

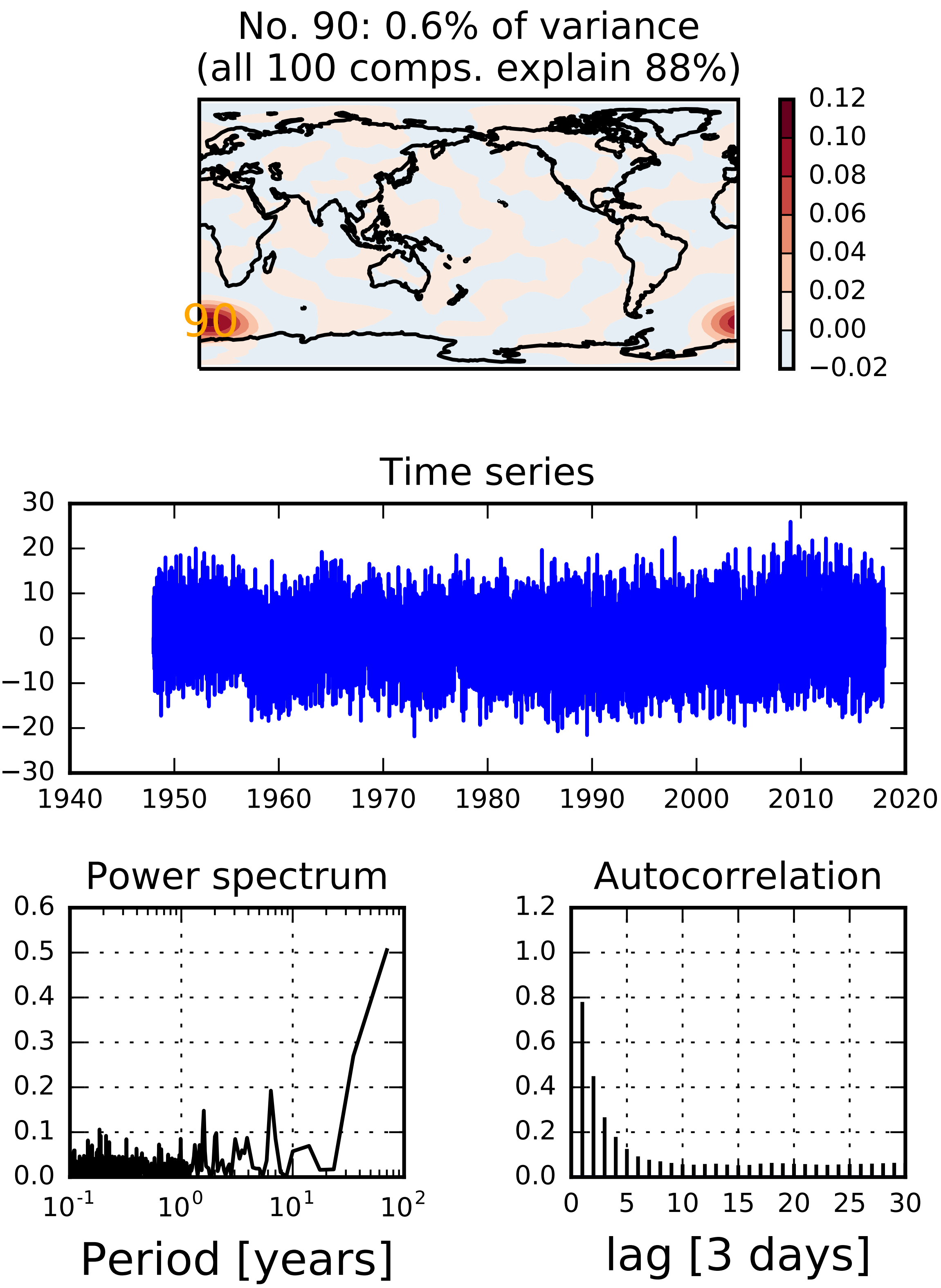
No. 90: 0.6% of variance
(all 100 comps. explain 88%)
90
0.12
0.10
0.08
0.06
0.04
0.02
0.00
−0.02
Time series
30
20
10
0
−10
−20
−30
1940
1950
1960
1970
1980
1990
2000
2010
2020
Power spectrum
0.6
0.5
0.4
0.3
0.2
0.1
0.0
10^-1
10^0
10^1
10^2
Period [years]
Autocorrelation
1.2
1.0
0.8
0.6
0.4
0.2
0.0
0
5
10
15
20
25
30
lag [3 days]

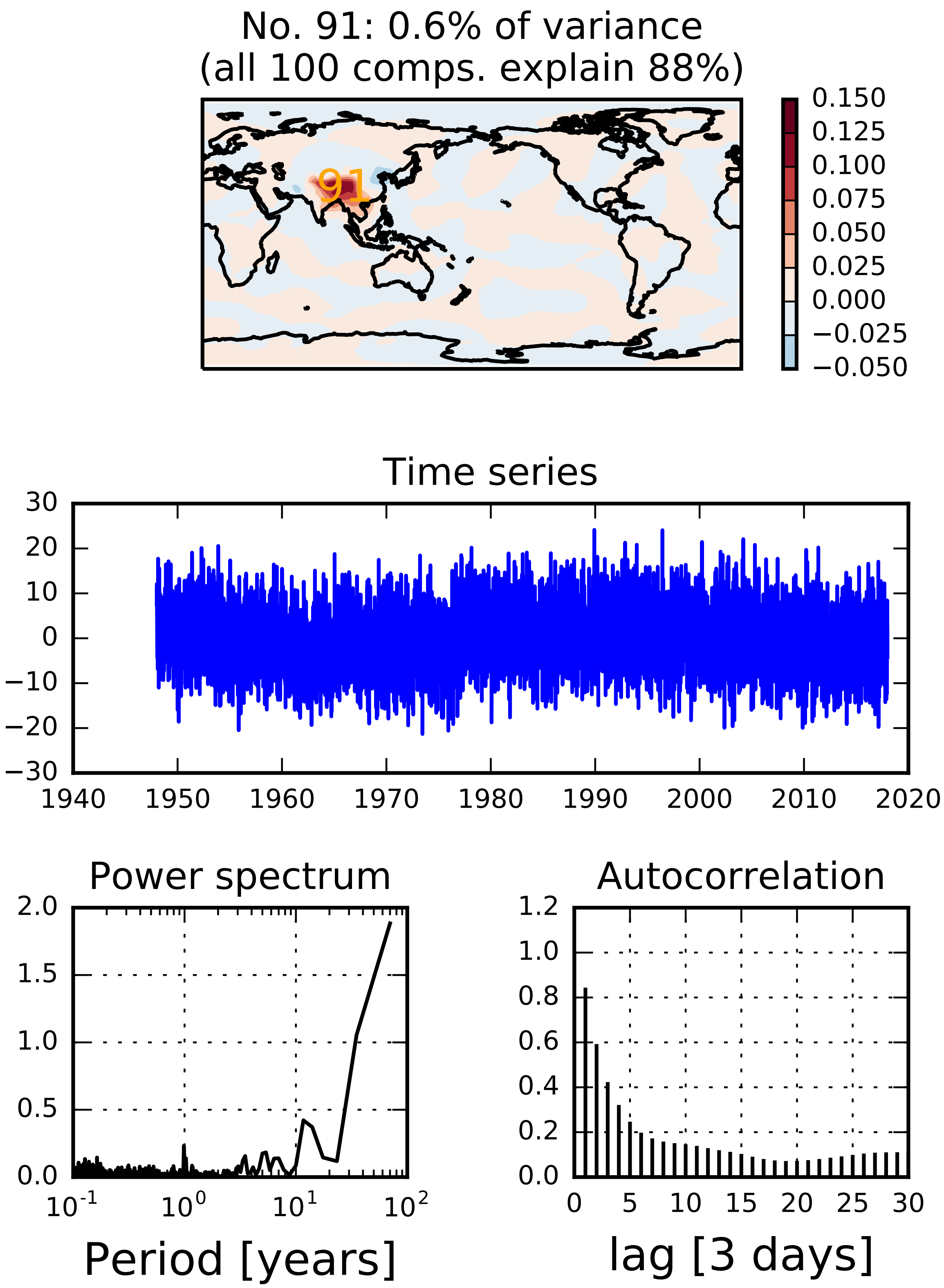

No. 91: 0.6% of variance
(all 100 comps. explain 88%)
91
0.150
0.125
0.100
0.075
0.050
0.025
0.000
−0.025
−0.050
Time series
30
20
10
0
−10
−20
−30
1940
1950
1960
1970
1980
1990
2000
2010
2020
Power spectrum
2.0
1.5
1.0
0.5
0.0
Period [years]
Autocorrelation
1.2
1.0
0.8
0.6
0.4
0.2
0.0
0
5
10
15
20
25
30
lag [3 days]

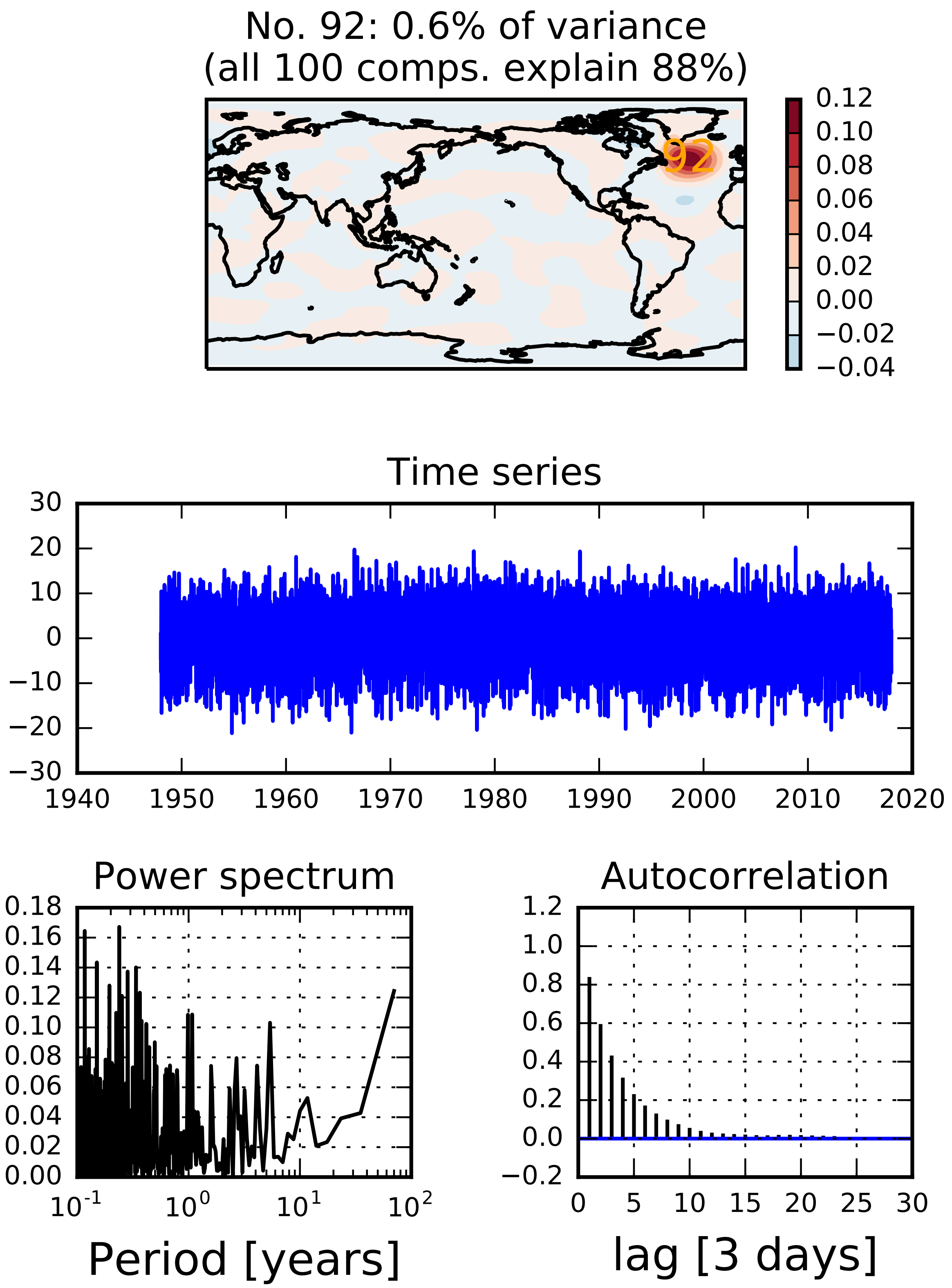
No. 92: 0.6% of variance
(all 100 comps. explain 88%)
92
0.12
0.10
0.08
0.06
0.04
0.02
0.00
−0.02
−0.04
Time series
30
20
10
0
−10
−20
−30
1940
1950
1960
1970
1980
1990
2000
2010
2020
Power spectrum
0.18
0.16
0.14
0.12
0.10
0.08
0.06
0.04
0.02
0.00
10^-1
10^0
10^1
10^2
Period [years]
Autocorrelation
1.2
1.0
0.8
0.6
0.4
0.2
0.0
−0.2
0
5
10
15
20
25
30
lag [3 days]

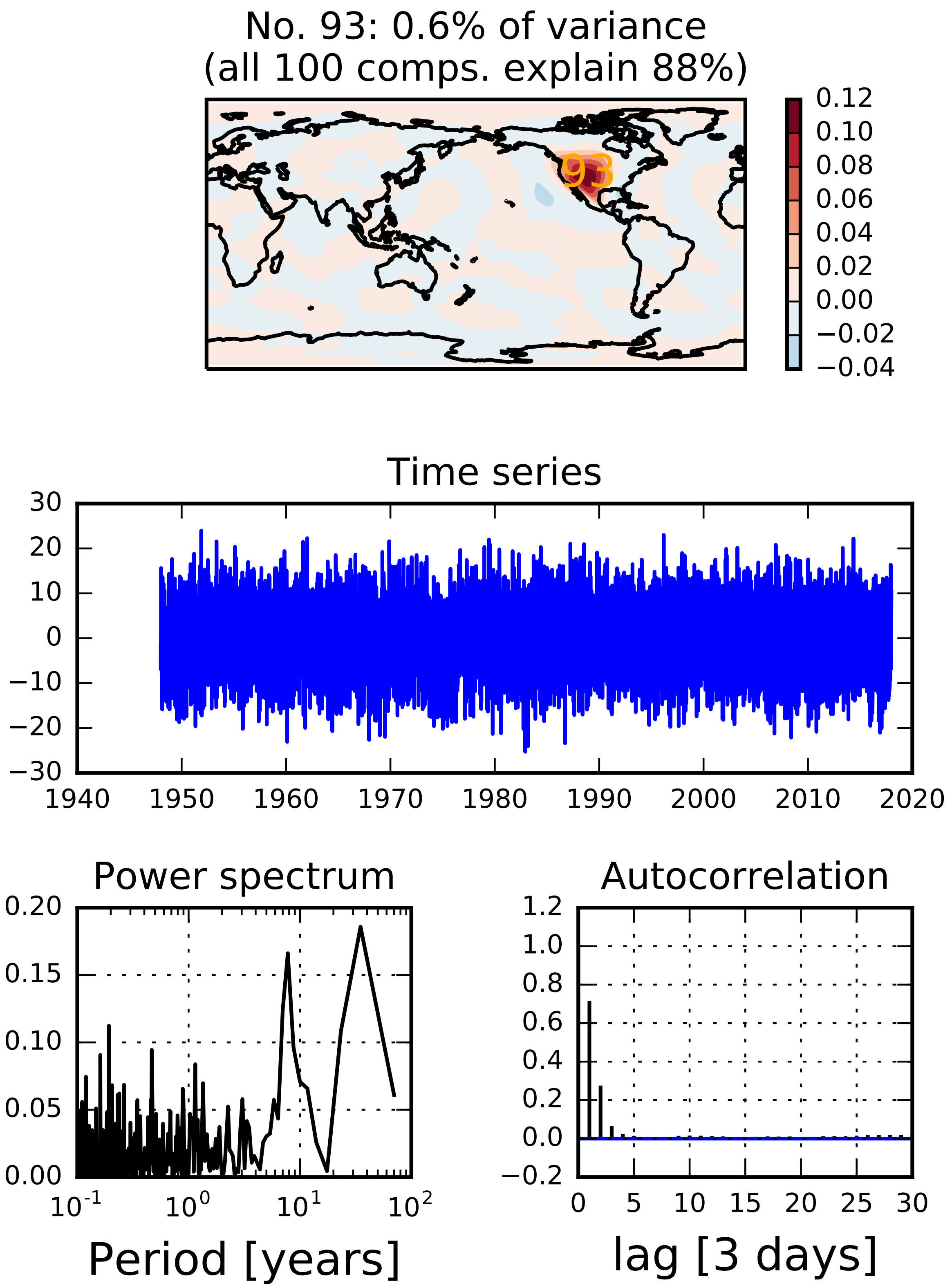
No. 93: 0.6% of variance
(all 100 comps. explain 88%)
93
0.12
0.10
0.08
0.06
0.04
0.02
0.00
−0.02
−0.04
Time series
30
20
10
0
−10
−20
−30
1940
1950
1960
1970
1980
1990
2000
2010
2020
Power spectrum
0.20
0.15
0.10
0.05
0.00
10^-1
10^0
10^1
10^2
Period [years]
Autocorrelation
1.2
1.0
0.8
0.6
0.4
0.2
0.0
−0.2
0
5
10
15
20
25
30
lag [3 days]

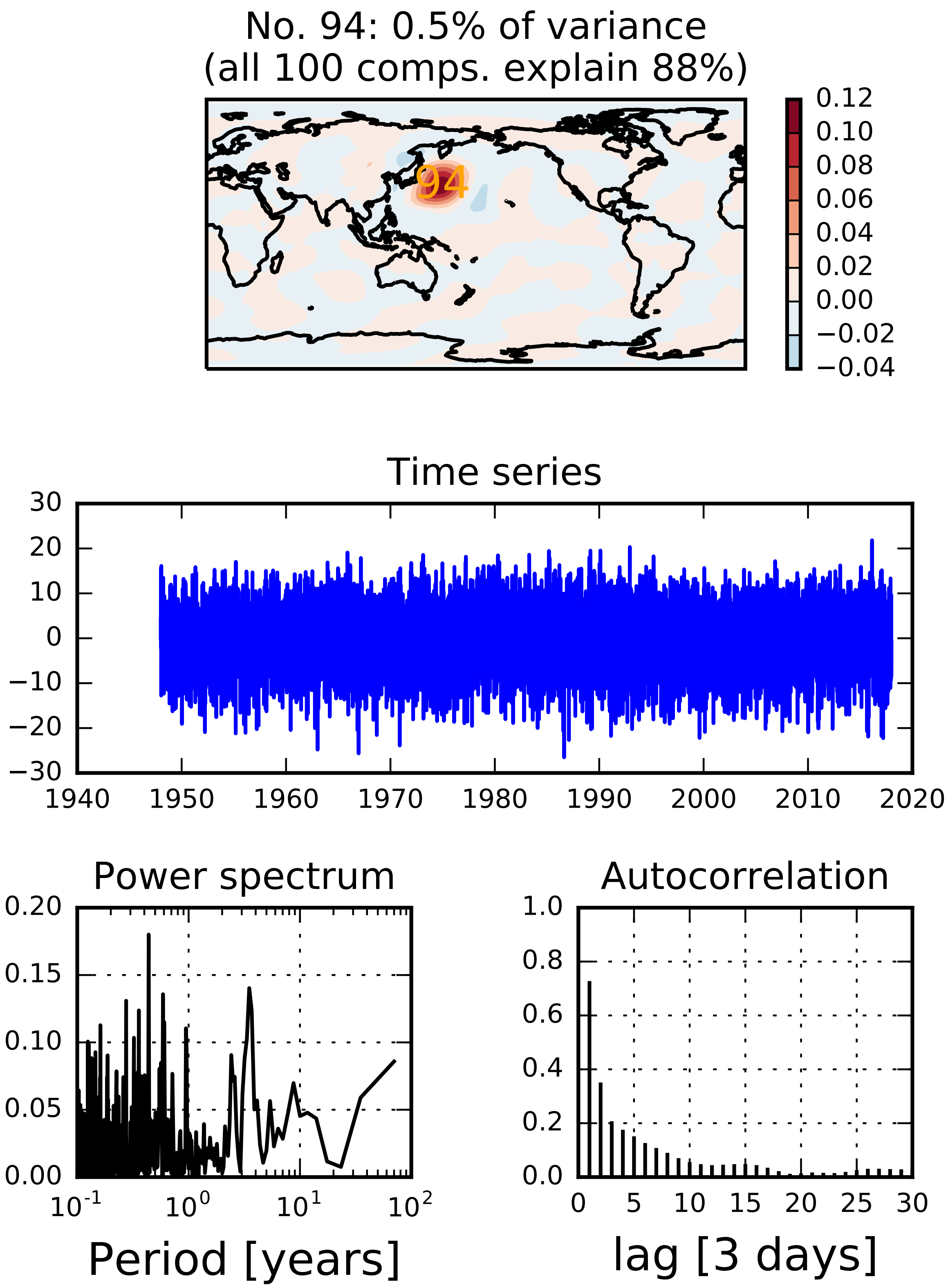

No. 94: 0.5% of variance
(all 100 comps. explain 88%)
94
0.12
0.10
0.08
0.06
0.04
0.02
0.00
−0.02
−0.04
Time series
30
20
10
0
−10
−20
−30
1940
1950
1960
1970
1980
1990
2000
2010
2020
Power spectrum
0.20
0.15
0.10
0.05
0.00
10^{-1}
10^{0}
10^{1}
10^{2}
Period [years]
Autocorrelation
1.0
0.8
0.6
0.4
0.2
0.0
0
5
10
15
20
25
30
lag [3 days]

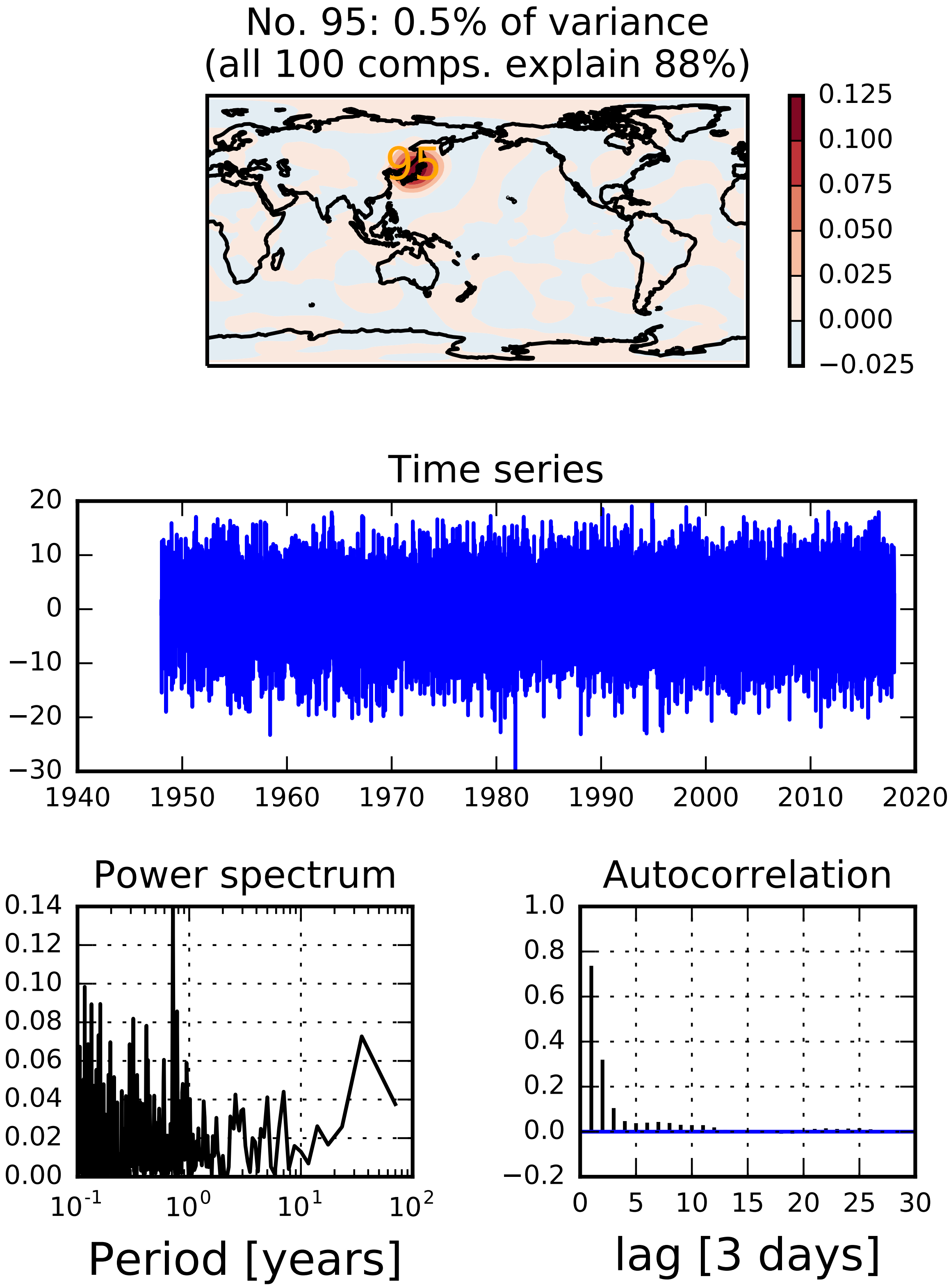
No. 95: 0.5% of variance
(all 100 comps. explain 88%)
95
0.125
0.100
0.075
0.050
0.025
0.000
−0.025
Time series
20
10
0
−10
−20
−30
1940
1950
1960
1970
1980
1990
2000
2010
2020
Power spectrum
0.14
0.12
0.10
0.08
0.06
0.04
0.02
0.00
10^{-1}
10^{0}
10^{1}
10^{2}
Period [years]
Autocorrelation
1.0
0.8
0.6
0.4
0.2
0.0
−0.2
0
5
10
15
20
25
30
lag [3 days]

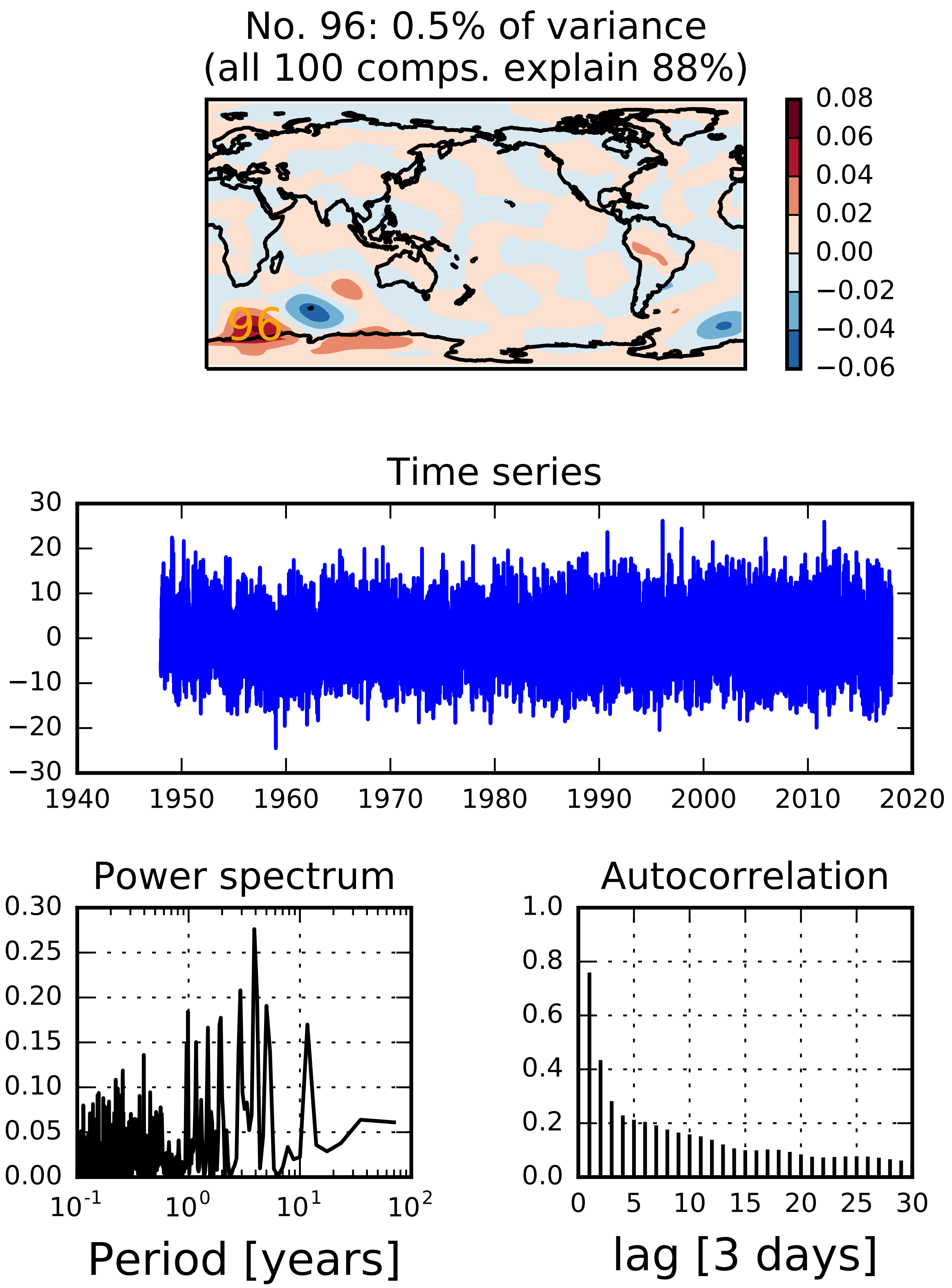
No. 96: 0.5% of variance
(all 100 comps. explain 88%)
96
0.08
0.06
0.04
0.02
0.00
−0.02
−0.04
−0.06
Time series
30
20
10
0
−10
−20
−30
1940
1950
1960
1970
1980
1990
2000
2010
2020
Power spectrum
0.30
0.25
0.20
0.15
0.10
0.05
0.00
10^-1
10^0
10^1
10^2
Period [years]
Autocorrelation
1.0
0.8
0.6
0.4
0.2
0.0
0
5
10
15
20
25
30
lag [3 days]

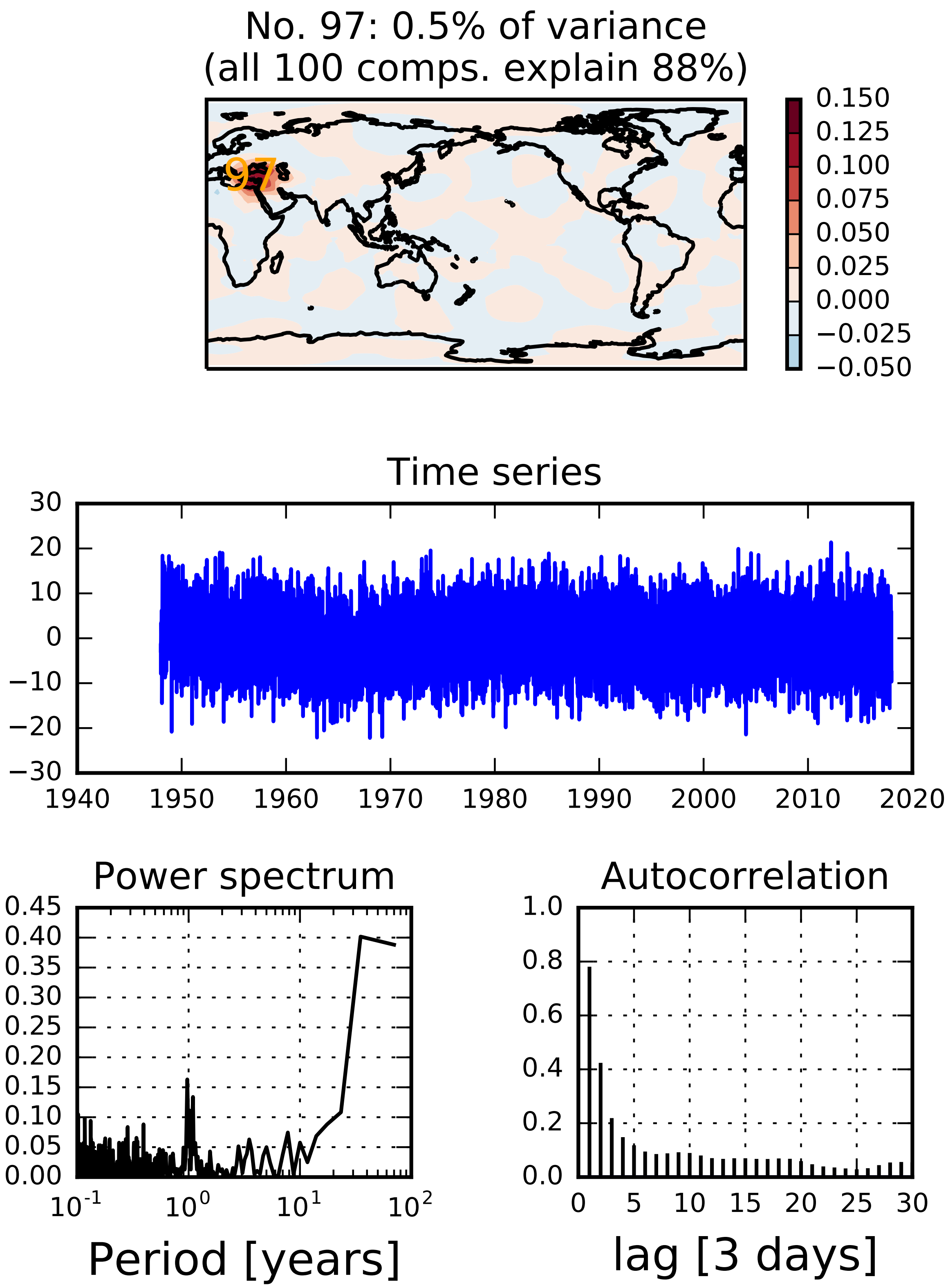
No. 97: 0.5% of variance
(all 100 comps. explain 88%)
97
0.150
0.125
0.100
0.075
0.050
0.025
0.000
−0.025
−0.050
Time series
30
20
10
0
−10
−20
−30
1940
1950
1960
1970
1980
1990
2000
2010
2020
Power spectrum
0.45
0.40
0.35
0.30
0.25
0.20
0.15
0.10
0.05
0.00
10^{-1}
10^{0}
10^{1}
10^{2}
Period [years]
Autocorrelation
1.0
0.8
0.6
0.4
0.2
0.0
0
5
10
15
20
25
30
lag [3 days]

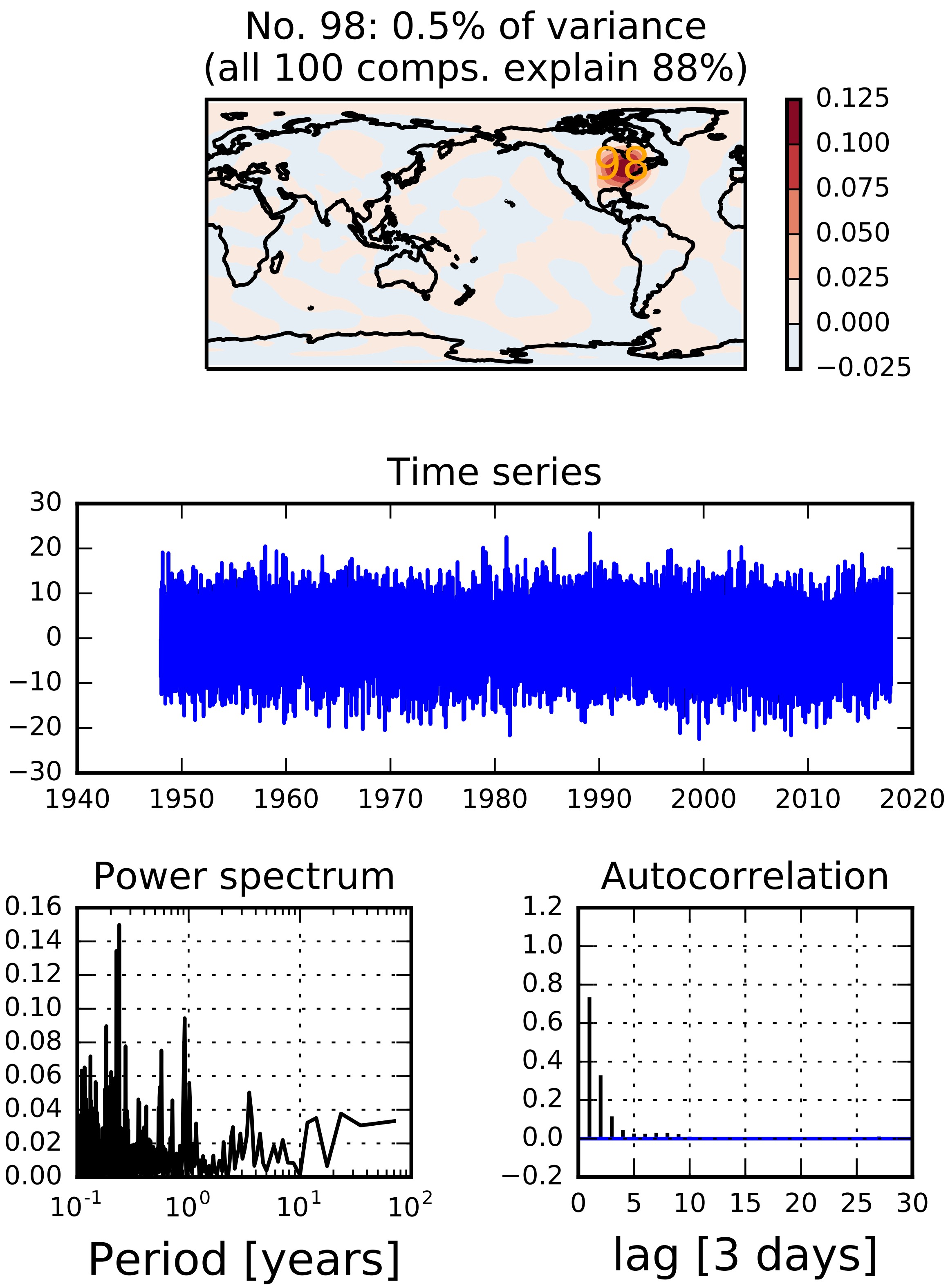
No. 98: 0.5% of variance
(all 100 comps. explain 88%)
98
0.125
0.100
0.075
0.050
0.025
0.000
−0.025
Time series
30
20
10
0
−10
−20
−30
1940
1950
1960
1970
1980
1990
2000
2010
2020
Power spectrum
0.16
0.14
0.12
0.10
0.08
0.06
0.04
0.02
0.00
10^-1
10^0
10^1
10^2
Period [years]
Autocorrelation
1.2
1.0
0.8
0.6
0.4
0.2
0.0
−0.2
0
5
10
15
20
25
30
lag [3 days]

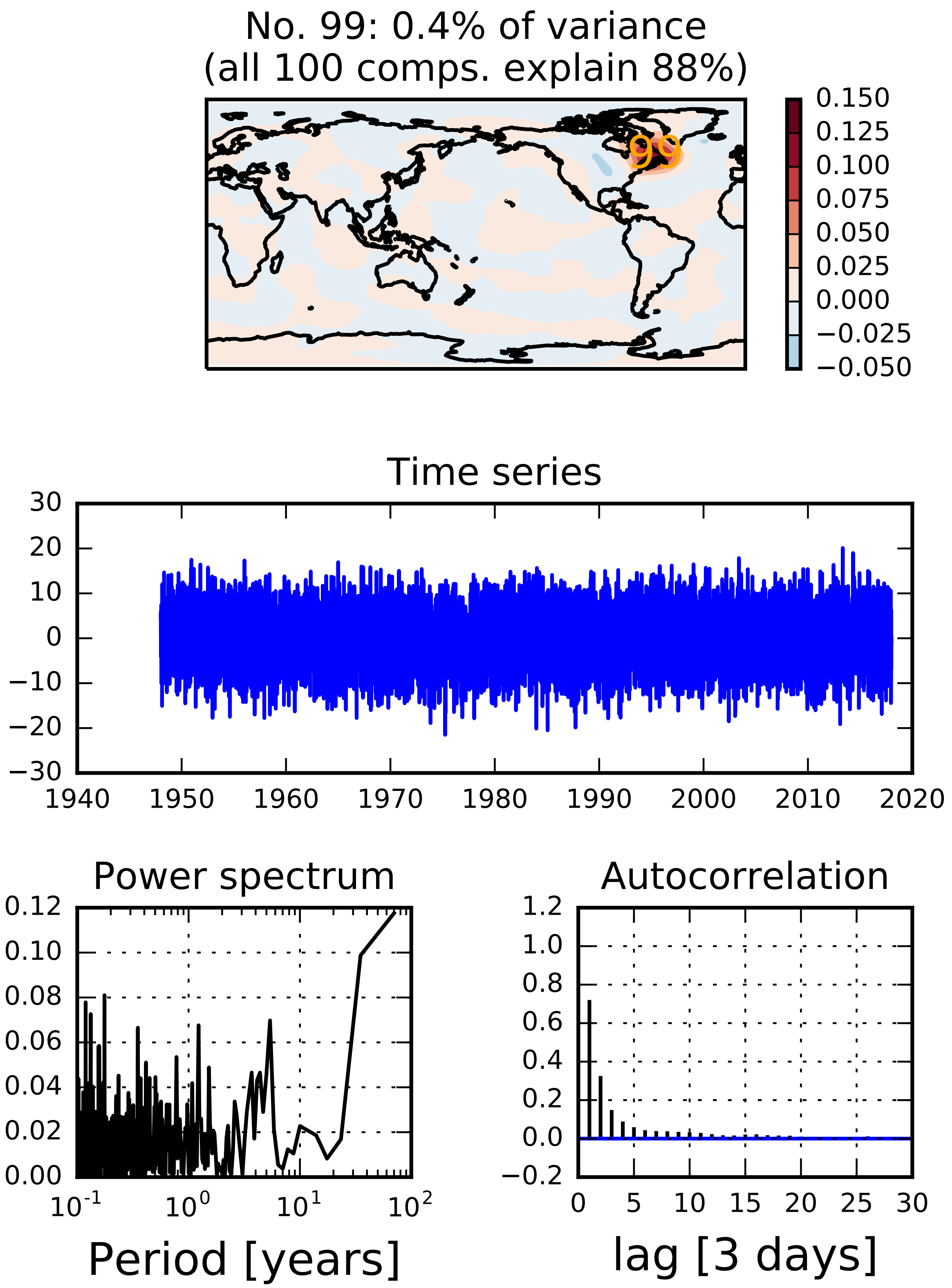
No. 99: 0.4% of variance
(all 100 comps. explain 88%)
99
0.150
0.125
0.100
0.075
0.050
0.025
0.000
−0.025
−0.050
Time series
30
20
10
0
−10
−20
−30
1940
1950
1960
1970
1980
1990
2000
2010
2020
Power spectrum
0.12
0.10
0.08
0.06
0.04
0.02
0.00
10^{-1}
10^{0}
10^{1}
10^{2}
Period [years]
Autocorrelation
1.2
1.0
0.8
0.6
0.4
0.2
0.0
−0.2
0
5
10
15
20
25
30
lag [3 days]

Supplementary Figures 222 to 321

JJA PCA Varimax components. For each component, we provide the component index and the component's spatial loadings (top subfigure), the component time series (centre) as well as the component's power spectrum (bottom left) and autocorrelation properties (bottom right). For each component we further provide the fraction of explained variance as defined prior to the Varimax rotation.

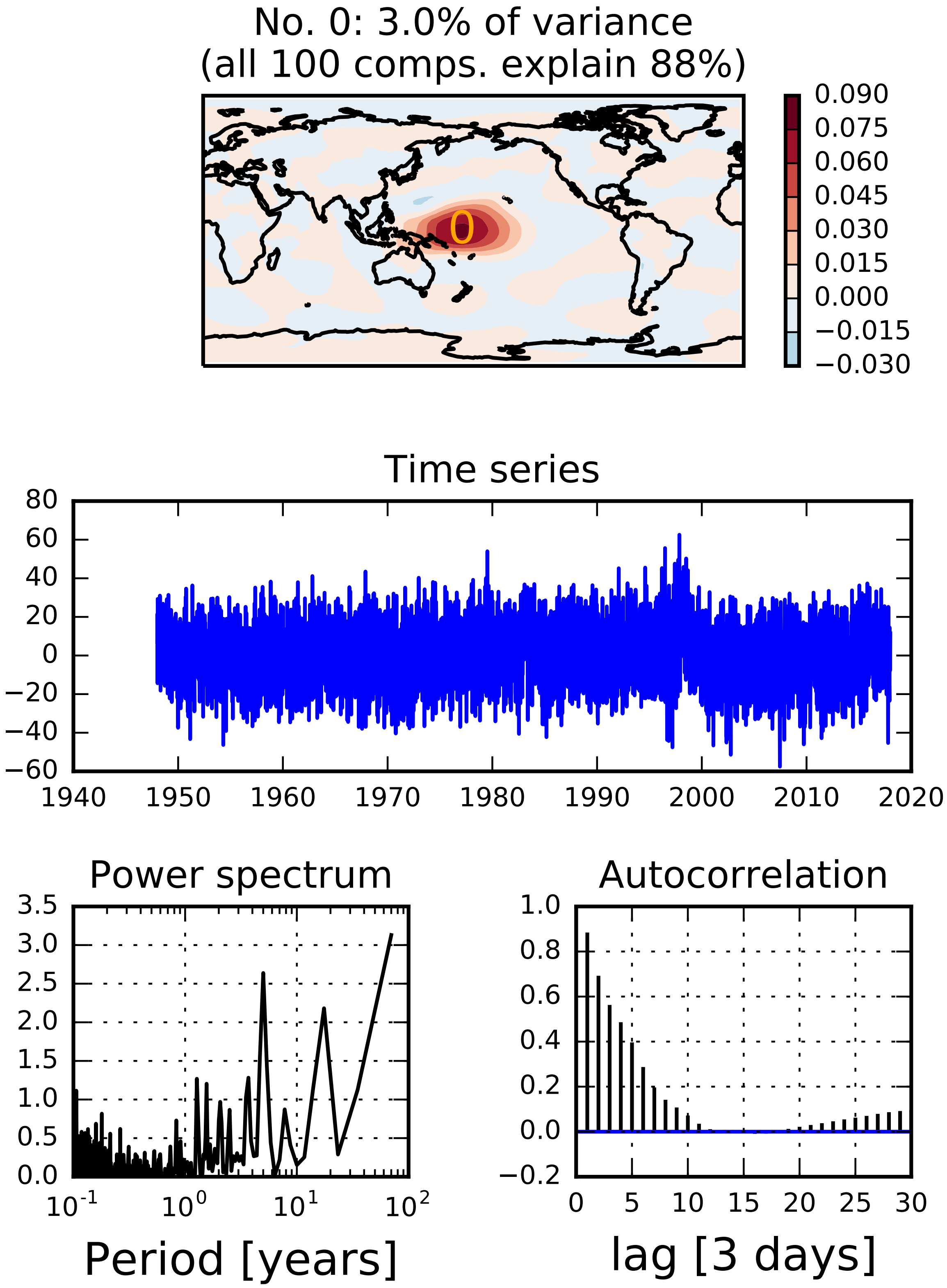

No. 0: 3.0% of variance
(all 100 comps. explain 88%)
0
0.090
0.075
0.060
0.045
0.030
0.015
0.000
−0.015
−0.030
Time series
80
60
40
20
0
−20
−40
−60
1940
1950
1960
1970
1980
1990
2000
2010
2020
Power spectrum
3.5
3.0
2.5
2.0
1.5
1.0
0.5
0.0
10^{-1}
10^{0}
10^{1}
10^{2}
Period [years]
Autocorrelation
1.0
0.8
0.6
0.4
0.2
0.0
−0.2
0
5
10
15
20
25
30
lag [3 days]

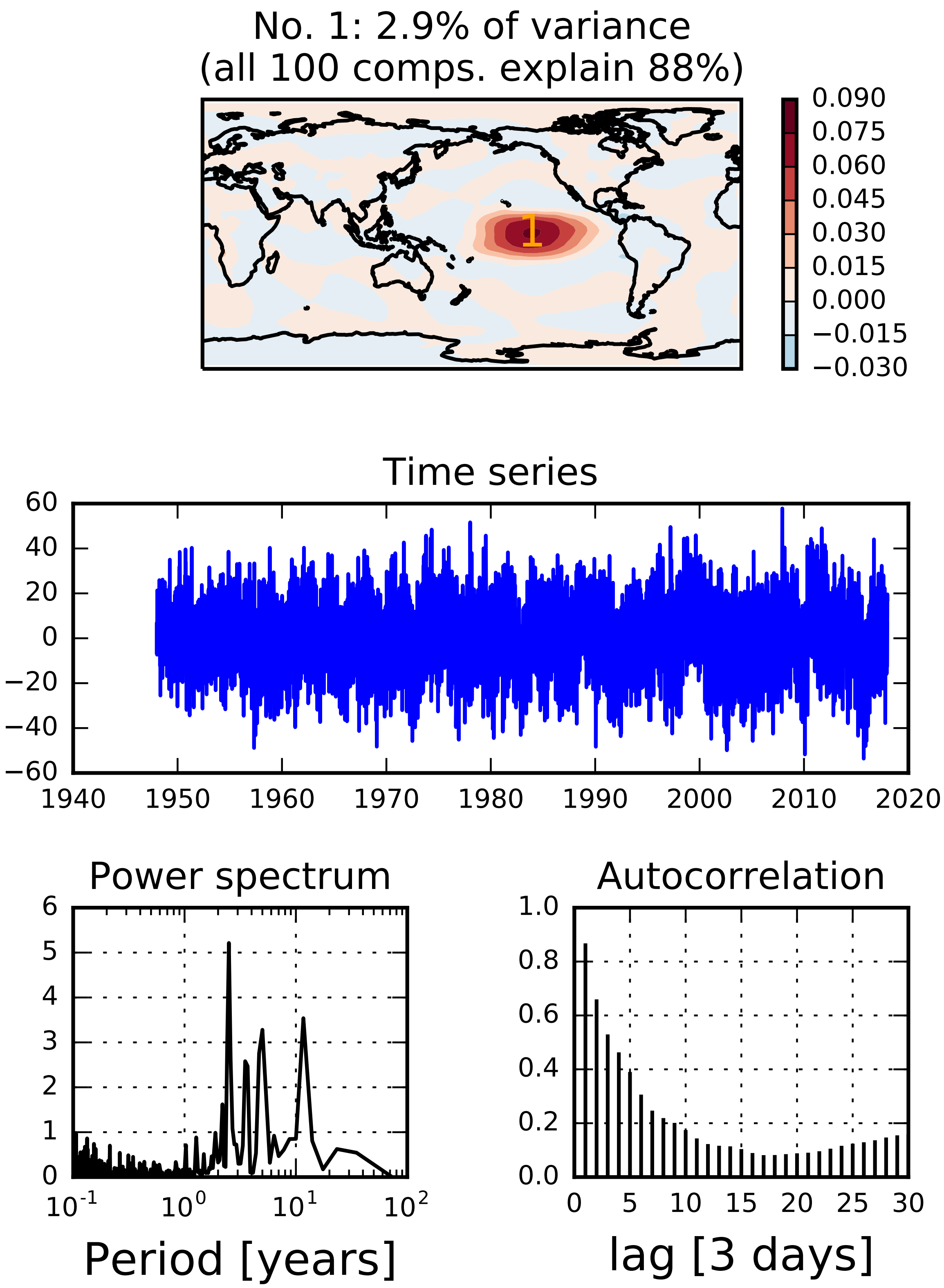
No. 1: 2.9% of variance
(all 100 comps. explain 88%)
1
0.090
0.075
0.060
0.045
0.030
0.015
0.000
−0.015
−0.030
Time series
60
40
20
0
−20
−40
−60
1940
1950
1960
1970
1980
1990
2000
2010
2020
Power spectrum
6
5
4
3
2
1
0
10^{-1}
10^{0}
10^{1}
10^{2}
Period [years]
Autocorrelation
1.0
0.8
0.6
0.4
0.2
0.0
0
5
10
15
20
25
30
lag [3 days]

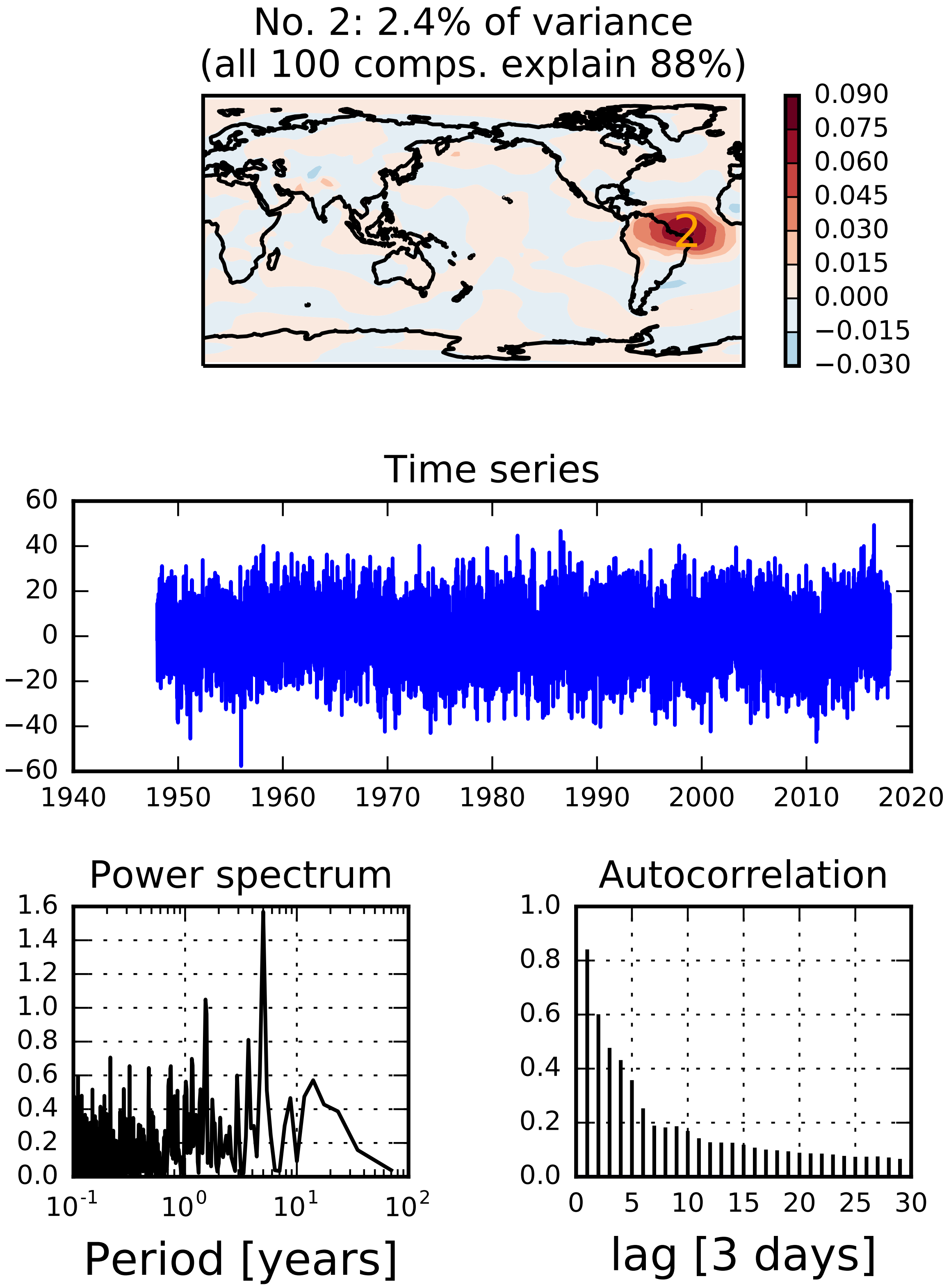
No. 2: 2.4% of variance
(all 100 comps. explain 88%)
2
0.090
0.075
0.060
0.045
0.030
0.015
0.000
−0.015
−0.030
Time series
60
40
20
0
−20
−40
−60
1940
1950
1960
1970
1980
1990
2000
2010
2020
Power spectrum
1.6
1.4
1.2
1.0
0.8
0.6
0.4
0.2
0.0
10^{-1}
10^{0}
10^{1}
10^{2}
Period [years]
Autocorrelation
1.0
0.8
0.6
0.4
0.2
0.0
0
5
10
15
20
25
30
lag [3 days]

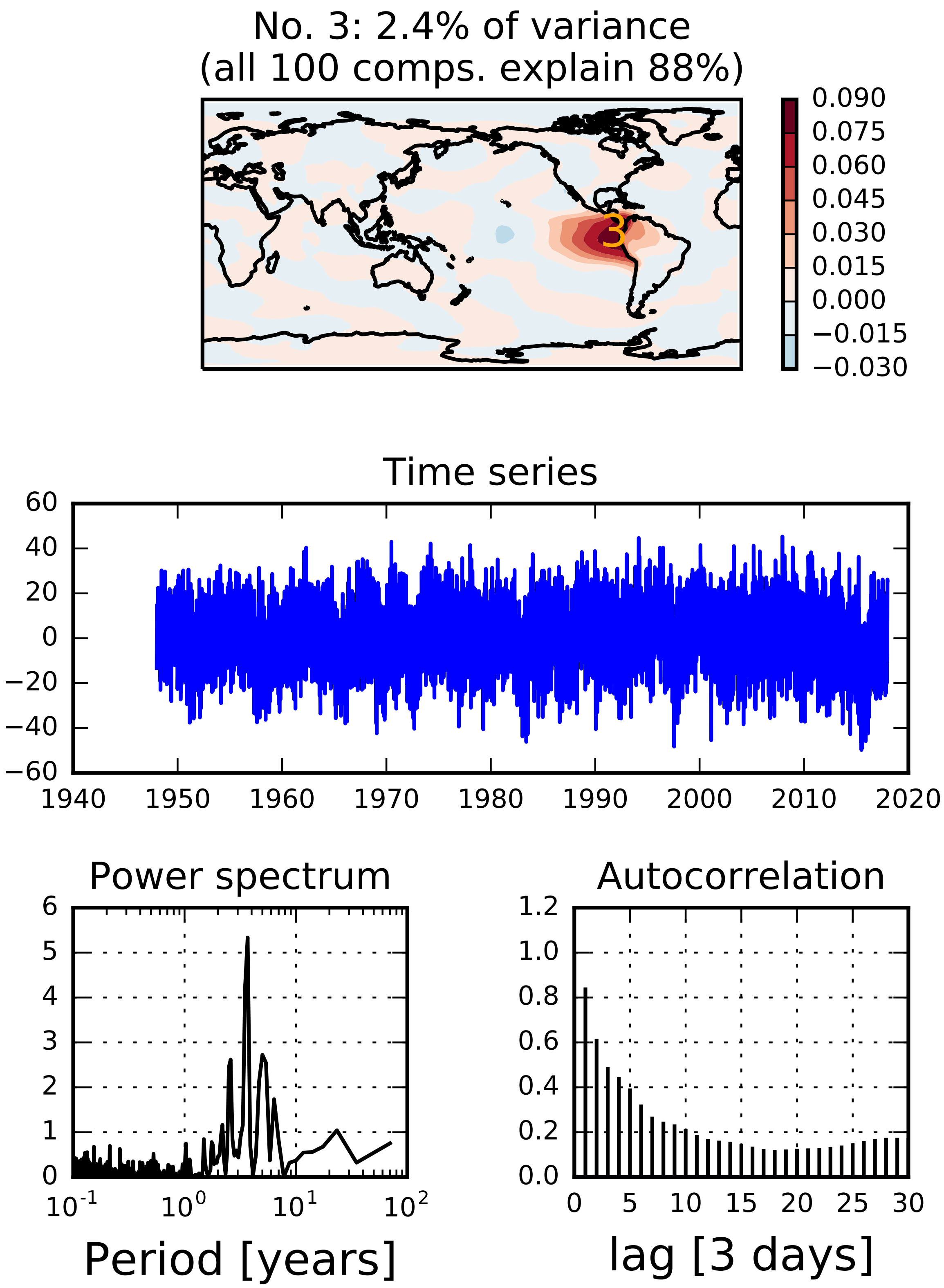

No. 3: 2.4% of variance
(all 100 comps. explain 88%)
3
0.090
0.075
0.060
0.045
0.030
0.015
0.000
−0.015
−0.030
Time series
60
40
20
0
−20
−40
−60
1940
1950
1960
1970
1980
1990
2000
2010
2020
Power spectrum
6
5
4
3
2
1
0
10^{-1}
10^{0}
10^{1}
10^{2}
Period [years]
Autocorrelation
1.2
1.0
0.8
0.6
0.4
0.2
0.0
0
5
10
15
20
25
30
lag [3 days]

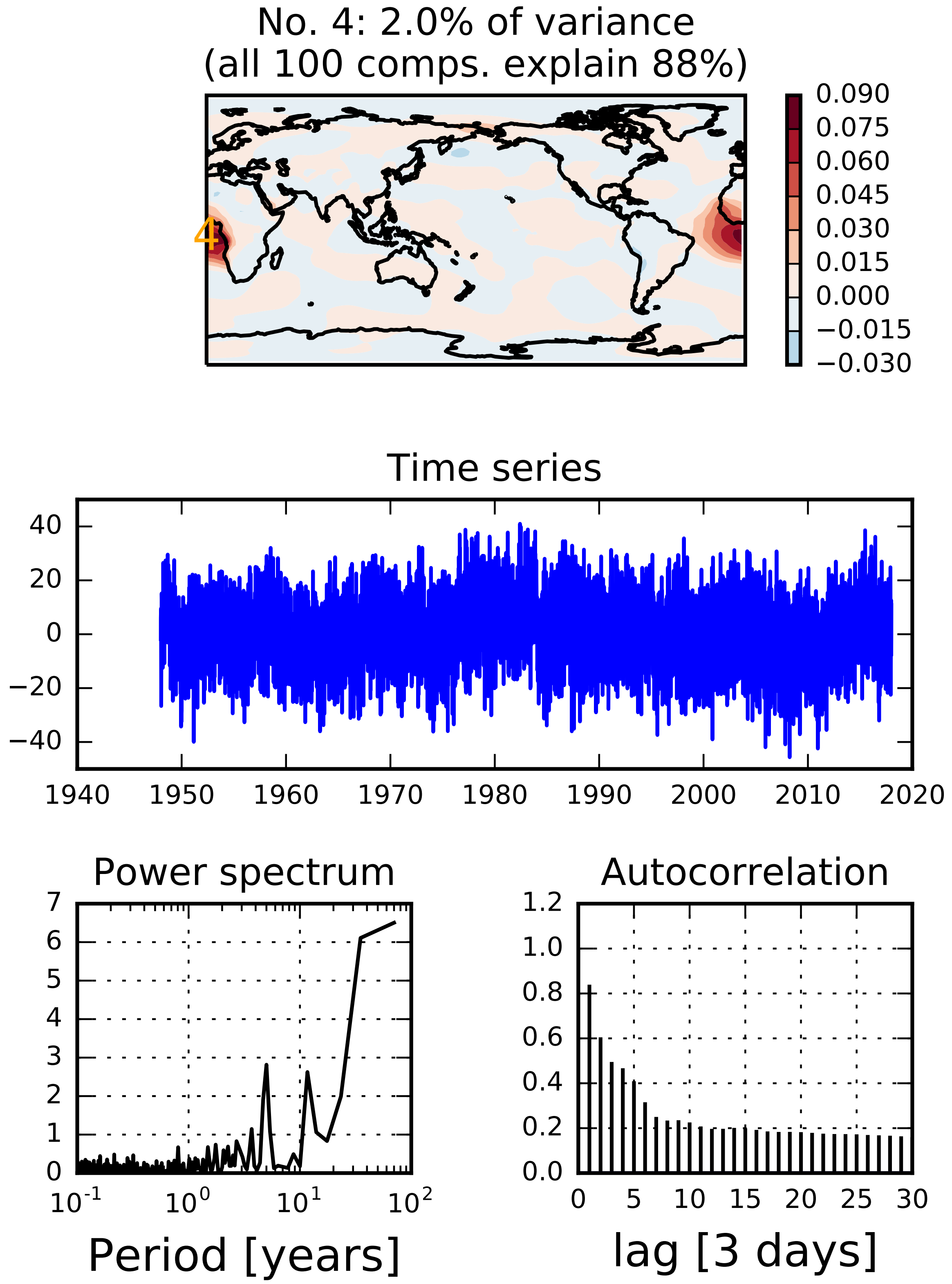
No. 4: 2.0% of variance
(all 100 comps. explain 88%)
4
0.090
0.075
0.060
0.045
0.030
0.015
0.000
−0.015
−0.030
Time series
40
20
0
−20
−40
1940
1950
1960
1970
1980
1990
2000
2010
2020
Power spectrum
7
6
5
4
3
2
1
0
10^{-1}
10^{0}
10^{1}
10^{2}
Period [years]
Autocorrelation
1.2
1.0
0.8
0.6
0.4
0.2
0.0
0
5
10
15
20
25
30
lag [3 days]

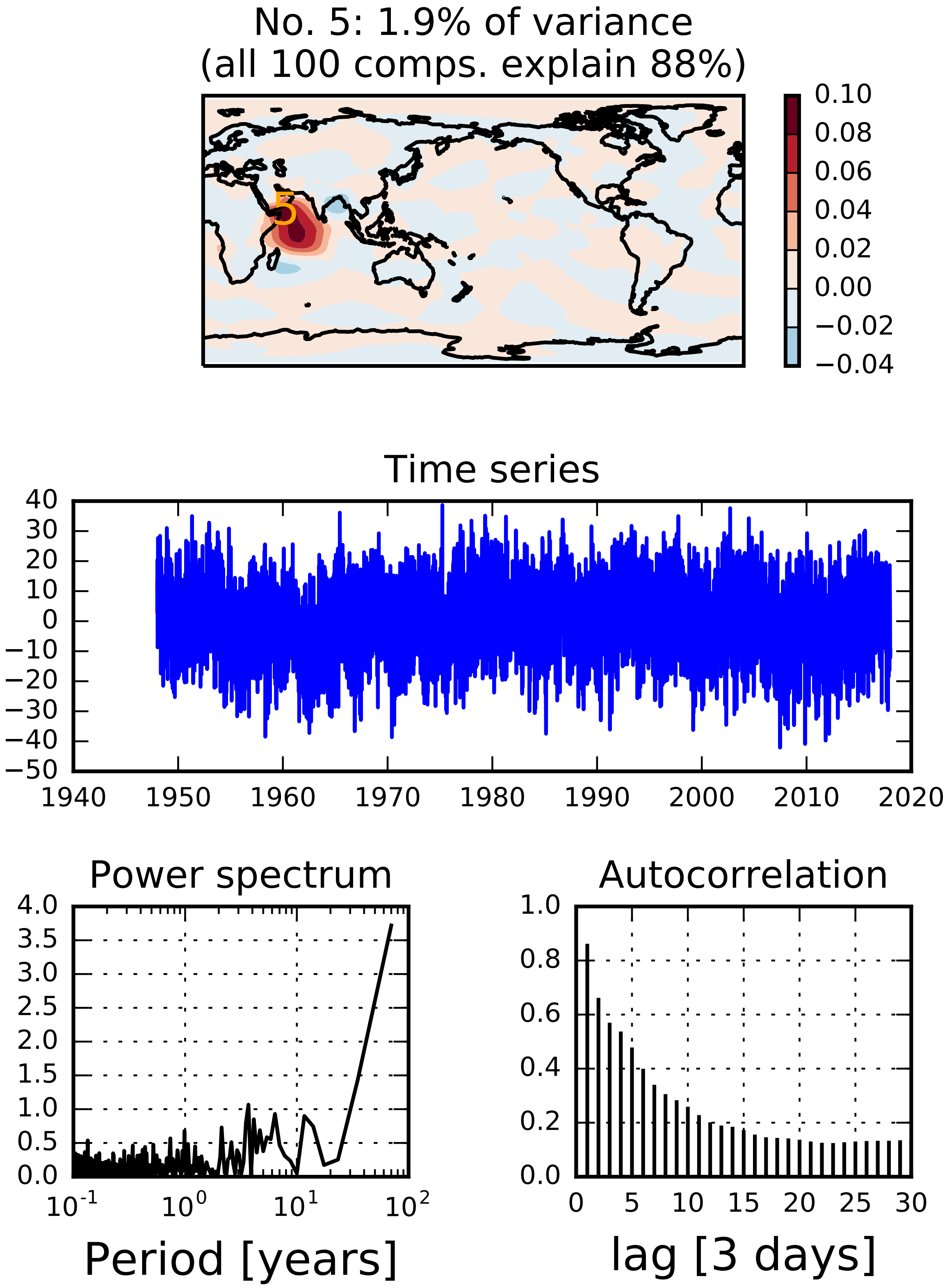
No. 5: 1.9% of variance
(all 100 comps. explain 88%)
5
0.10
0.08
0.06
0.04
0.02
0.00
−0.02
−0.04
Time series
40
30
20
10
0
−10
−20
−30
−40
−50
1940
1950
1960
1970
1980
1990
2000
2010
2020
Power spectrum
4.0
3.5
3.0
2.5
2.0
1.5
1.0
0.5
0.0
10^{-1}
10^{0}
10^{1}
10^{2}
Period [years]
Autocorrelation
1.0
0.8
0.6
0.4
0.2
0.0
0
5
10
15
20
25
30
lag [3 days]

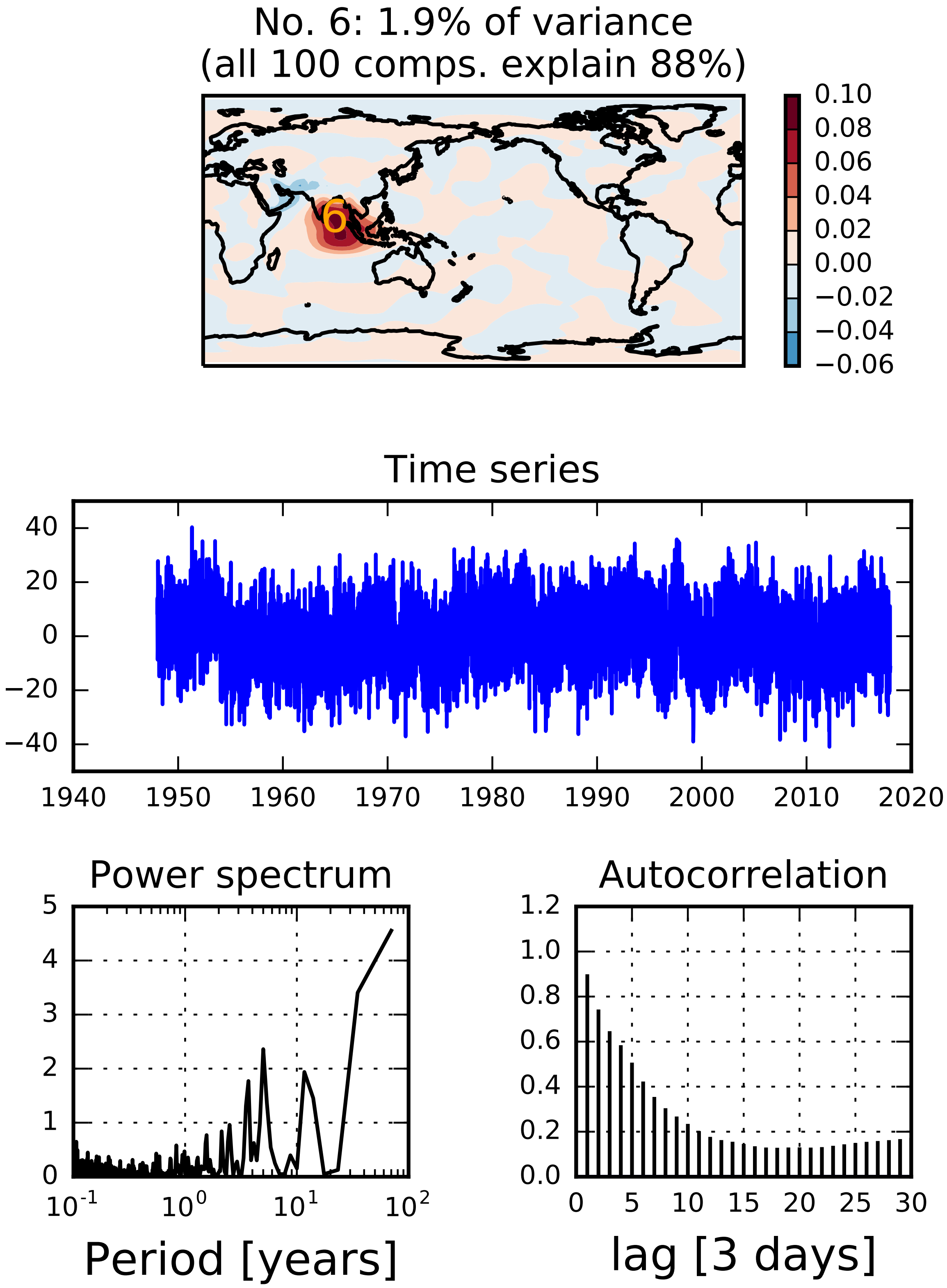
No. 6: 1.9% of variance
(all 100 comps. explain 88%)
6
0.10
0.08
0.06
0.04
0.02
0.00
−0.02
−0.04
−0.06
Time series
40
20
0
−20
−40
1940
1950
1960
1970
1980
1990
2000
2010
2020
Power spectrum
5
4
3
2
1
0
10^{-1}
10^{0}
10^{1}
10^{2}
Period [years]
Autocorrelation
1.2
1.0
0.8
0.6
0.4
0.2
0.0
0
5
10
15
20
25
30
lag [3 days]

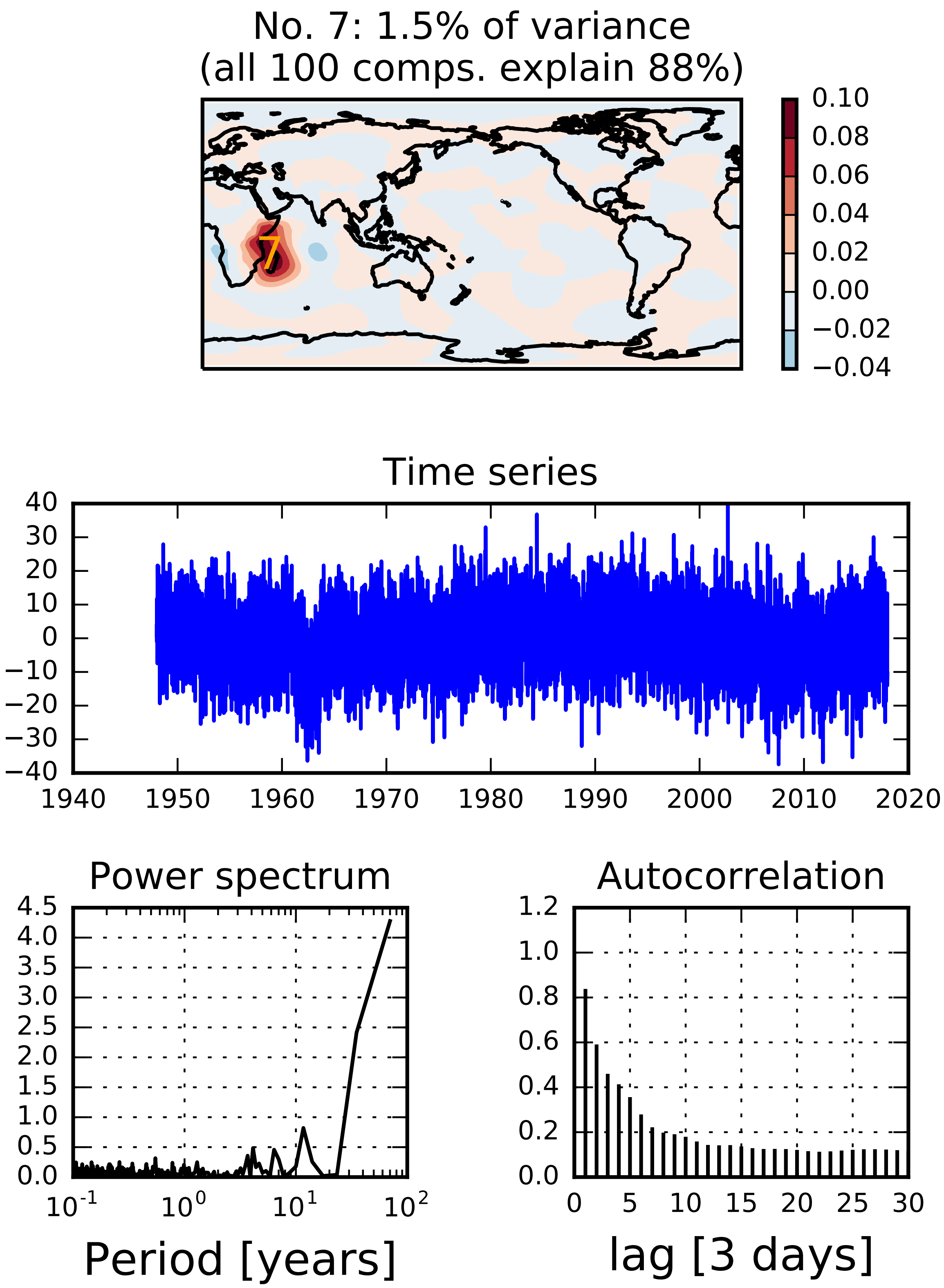
No. 7: 1.5% of variance
(all 100 comps. explain 88%)
7
0.10
0.08
0.06
0.04
0.02
0.00
−0.02
−0.04
Time series
40
30
20
10
0
−10
−20
−30
−40
1940
1950
1960
1970
1980
1990
2000
2010
2020
Power spectrum
4.5
4.0
3.5
3.0
2.5
2.0
1.5
1.0
0.5
0.0
10^{-1}
10^{0}
10^{1}
10^{2}
Period [years]
Autocorrelation
1.2
1.0
0.8
0.6
0.4
0.2
0.0
0
5
10
15
20
25
30
lag [3 days]

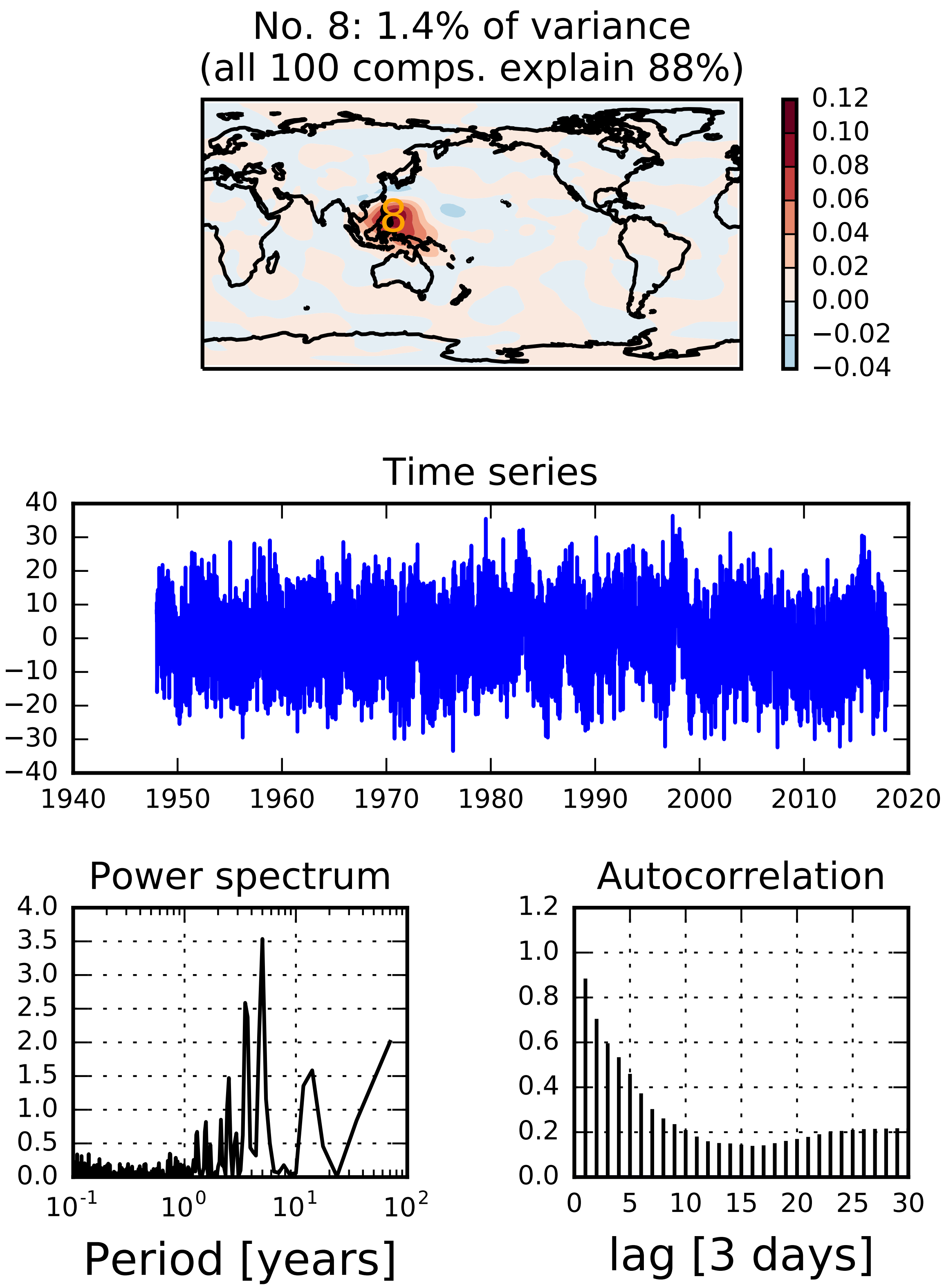
No. 8: 1.4% of variance
(all 100 comps. explain 88%)
8
0.12
0.10
0.08
0.06
0.04
0.02
0.00
−0.02
−0.04
Time series
40
30
20
10
0
−10
−20
−30
−40
1940
1950
1960
1970
1980
1990
2000
2010
2020
Power spectrum
4.0
3.5
3.0
2.5
2.0
1.5
1.0
0.5
0.0
10^-1
10^0
10^1
10^2
Period [years]
Autocorrelation
1.2
1.0
0.8
0.6
0.4
0.2
0.0
0
5
10
15
20
25
30
lag [3 days]

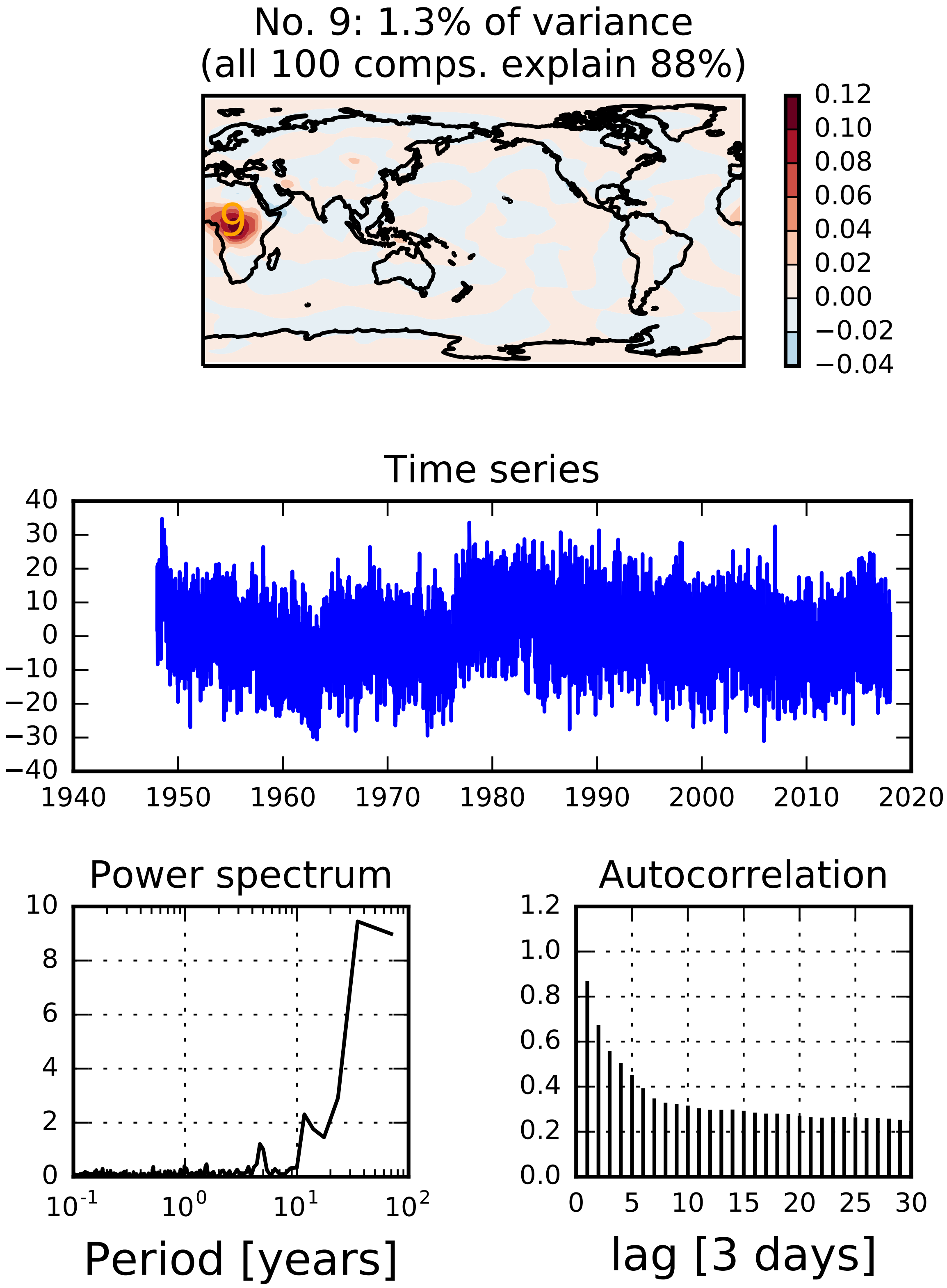
No. 9: 1.3% of variance
(all 100 comps. explain 88%)
9
0.12
0.10
0.08
0.06
0.04
0.02
0.00
−0.02
−0.04
Time series
40
30
20
10
0
−10
−20
−30
−40
1940
1950
1960
1970
1980
1990
2000
2010
2020
Power spectrum
10
8
6
4
2
0
10^{-1}
10^{0}
10^{1}
10^{2}
Period [years]
Autocorrelation
1.2
1.0
0.8
0.6
0.4
0.2
0.0
0
5
10
15
20
25
30
lag [3 days]

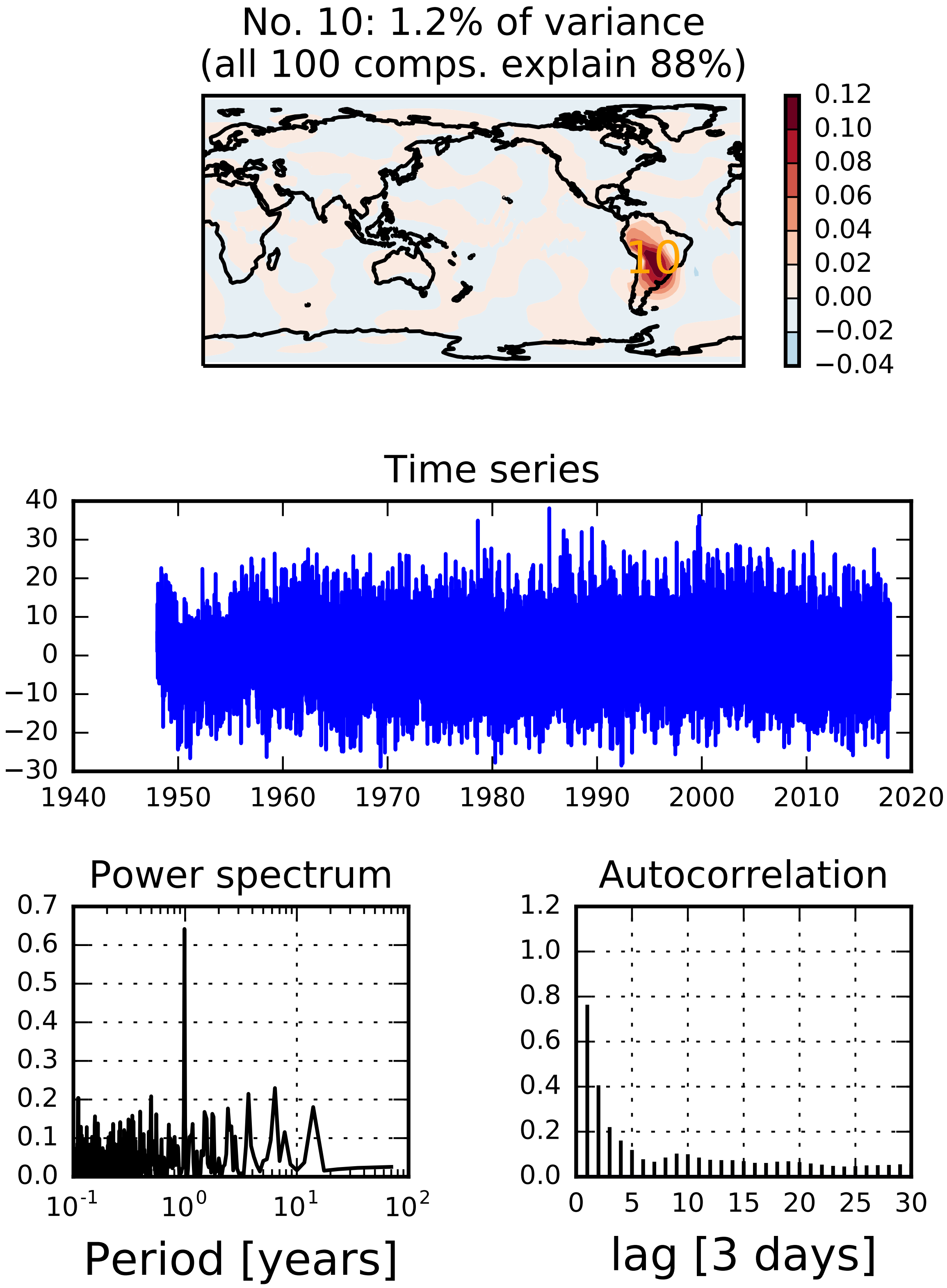
No. 10: 1.2% of variance
(all 100 comps. explain 88%)
10
0.12
0.10
0.08
0.06
0.04
0.02
0.00
−0.02
−0.04
Time series
40
30
20
10
0
−10
−20
−30
1940
1950
1960
1970
1980
1990
2000
2010
2020
Power spectrum
0.7
0.6
0.5
0.4
0.3
0.2
0.1
0.0
10^-1
10^0
10^1
10^2
Period [years]
Autocorrelation
1.2
1.0
0.8
0.6
0.4
0.2
0.0
0
5
10
15
20
25
30
lag [3 days]

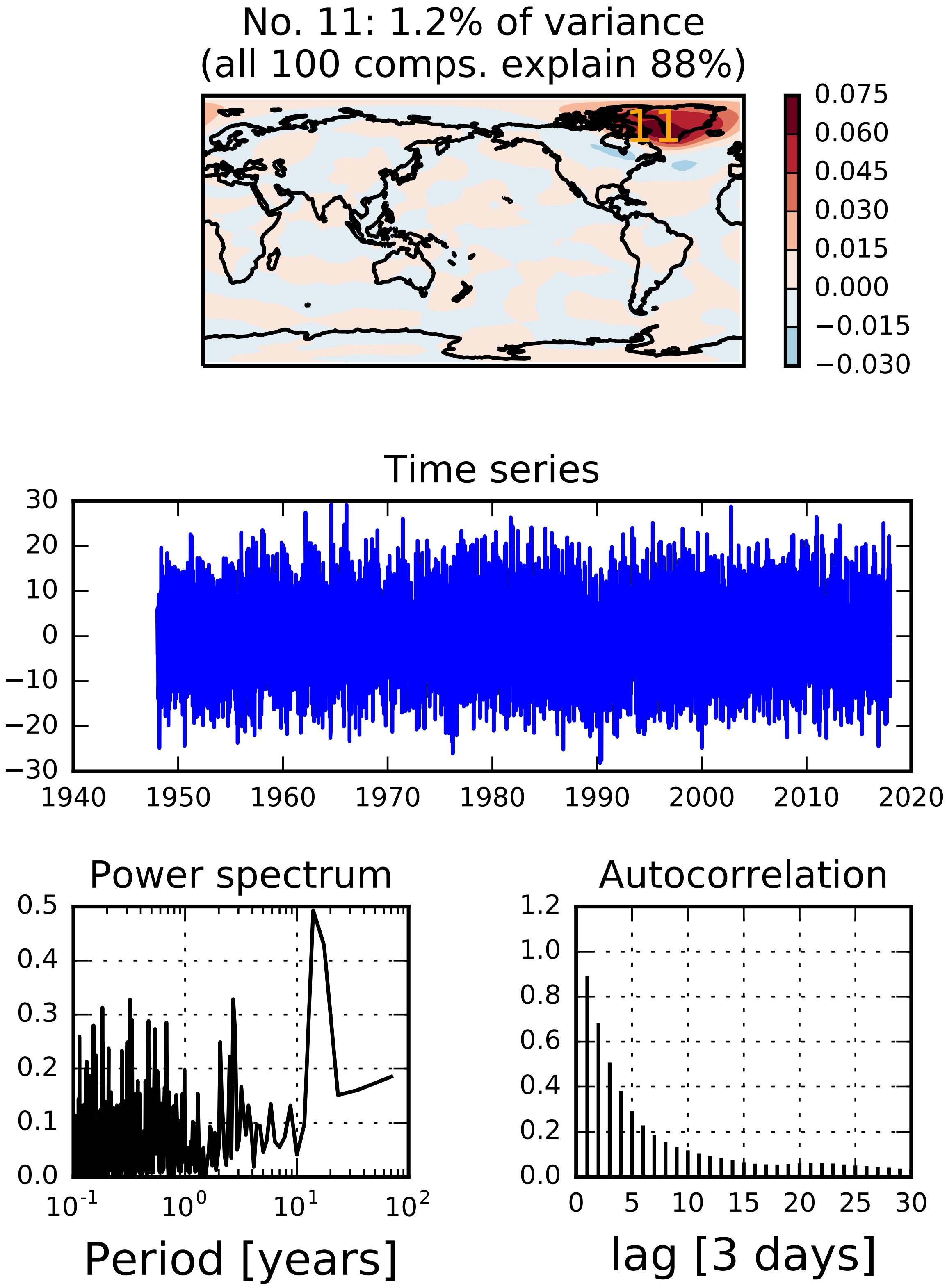
No. 11: 1.2% of variance
(all 100 comps. explain 88%)
11
0.075
0.060
0.045
0.030
0.015
0.000
−0.015
−0.030
Time series
30
20
10
0
−10
−20
−30
1940
1950
1960
1970
1980
1990
2000
2010
2020
Power spectrum
0.5
0.4
0.3
0.2
0.1
0.0
10^{-1}
10^{0}
10^{1}
10^{2}
Period [years]
Autocorrelation
1.2
1.0
0.8
0.6
0.4
0.2
0.0
0
5
10
15
20
25
30
lag [3 days]

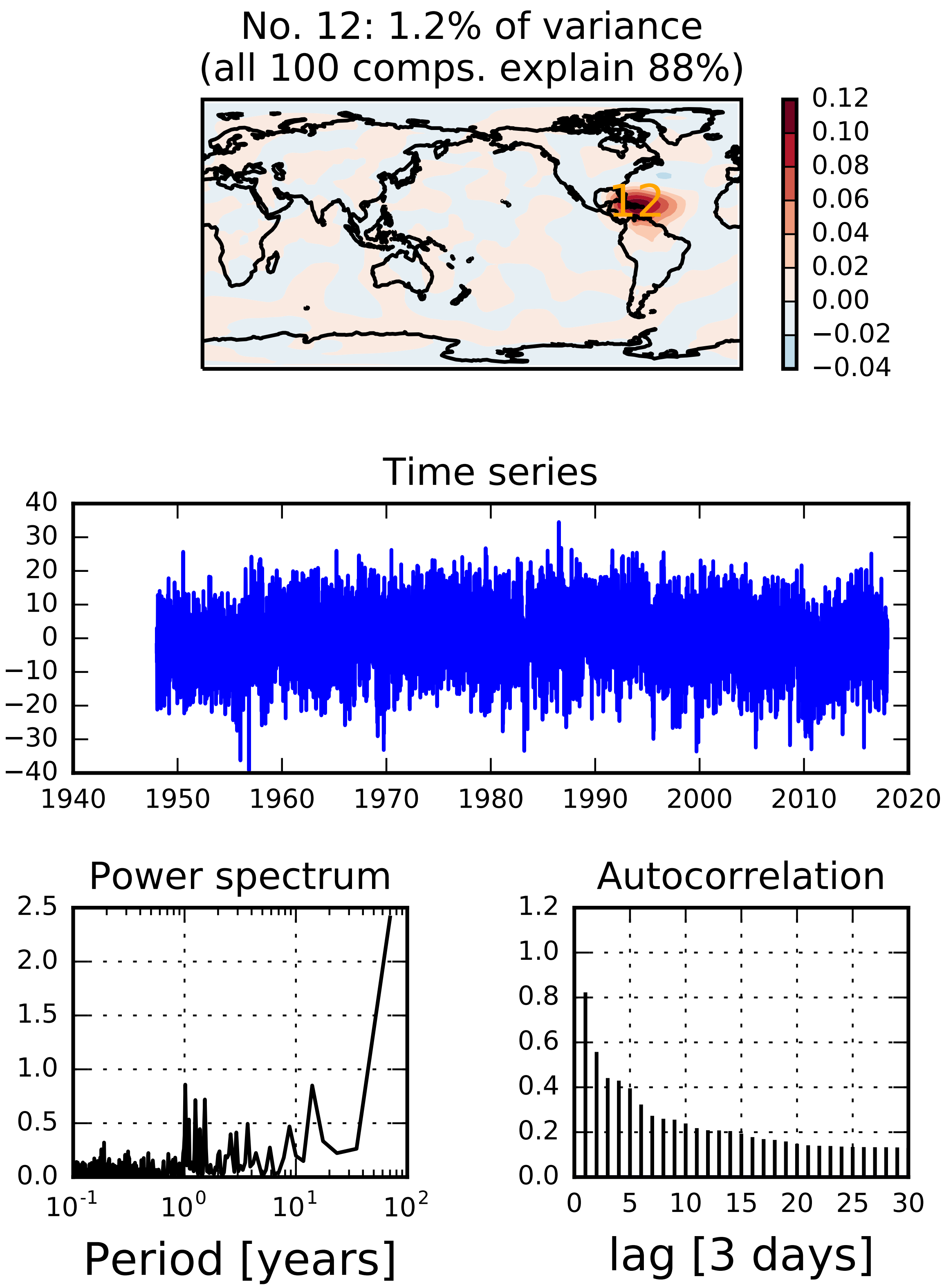
No. 12: 1.2% of variance
(all 100 comps. explain 88%)
12
0.12
0.10
0.08
0.06
0.04
0.02
0.00
−0.02
−0.04
Time series
40
30
20
10
0
−10
−20
−30
−40
1940
1950
1960
1970
1980
1990
2000
2010
2020
Power spectrum
2.5
2.0
1.5
1.0
0.5
0.0
10^{-1}
10^{0}
10^{1}
10^{2}
Period [years]
Autocorrelation
1.2
1.0
0.8
0.6
0.4
0.2
0.0
0
5
10
15
20
25
30
lag [3 days]

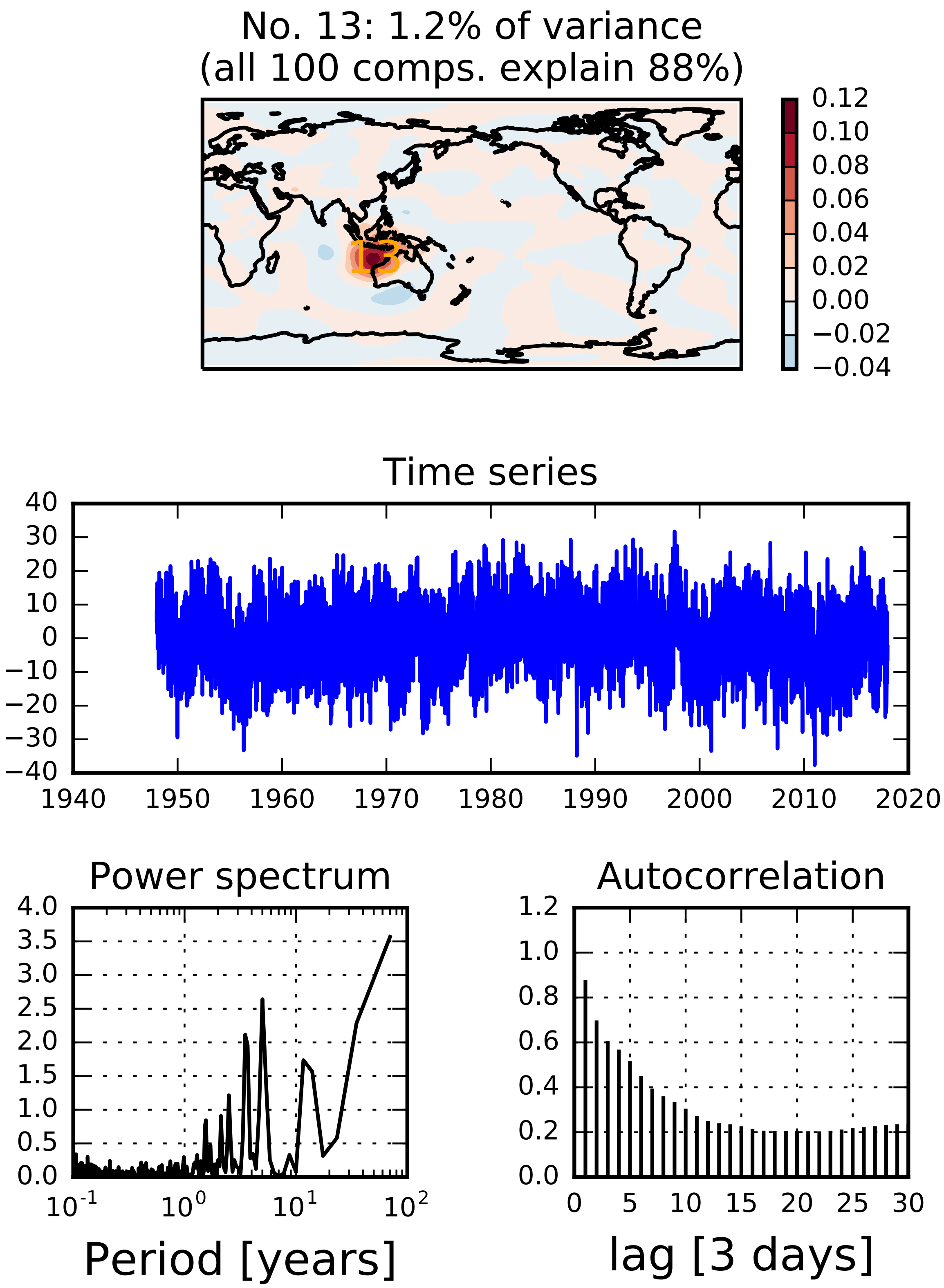

No. 13: 1.2% of variance
(all 100 comps. explain 88%)
0.12
0.10
0.08
0.06
0.04
0.02
0.00
−0.02
−0.04
Time series
40
30
20
10
0
−10
−20
−30
−40
1940
1950
1960
1970
1980
1990
2000
2010
2020
Power spectrum
4.0
3.5
3.0
2.5
2.0
1.5
1.0
0.5
0.0
10^{-1}
10^{0}
10^{1}
10^{2}
Period [years]
Autocorrelation
1.2
1.0
0.8
0.6
0.4
0.2
0.0
0
5
10
15
20
25
30
lag [3 days]

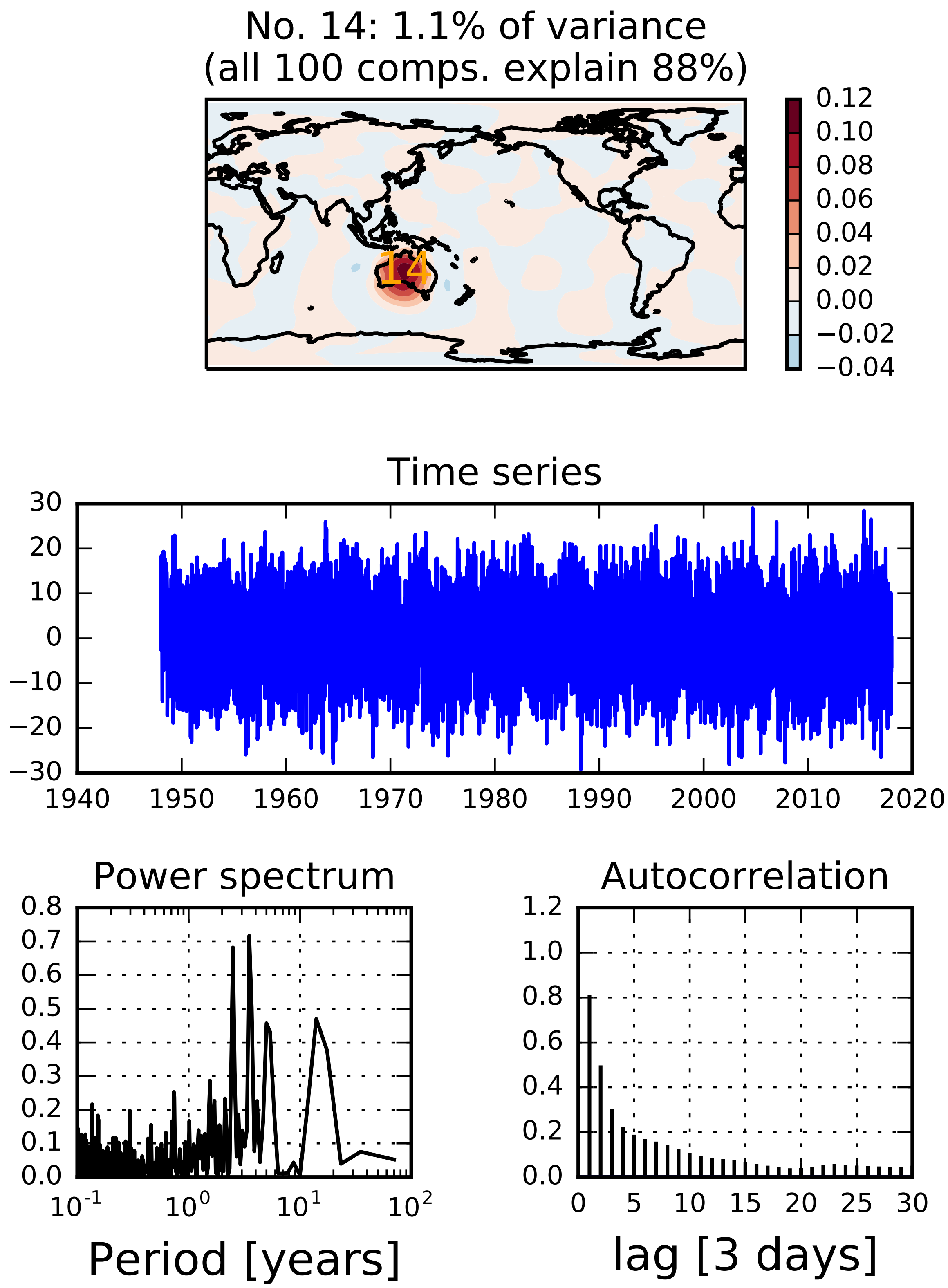
No. 14: 1.1% of variance
(all 100 comps. explain 88%)
14
0.12
0.10
0.08
0.06
0.04
0.02
0.00
−0.02
−0.04
Time series
30
20
10
0
−10
−20
−30
1940
1950
1960
1970
1980
1990
2000
2010
2020
Power spectrum
0.8
0.7
0.6
0.5
0.4
0.3
0.2
0.1
0.0
10^-1
10^0
10^1
10^2
Period [years]
Autocorrelation
1.2
1.0
0.8
0.6
0.4
0.2
0.0
0
5
10
15
20
25
30
lag [3 days]

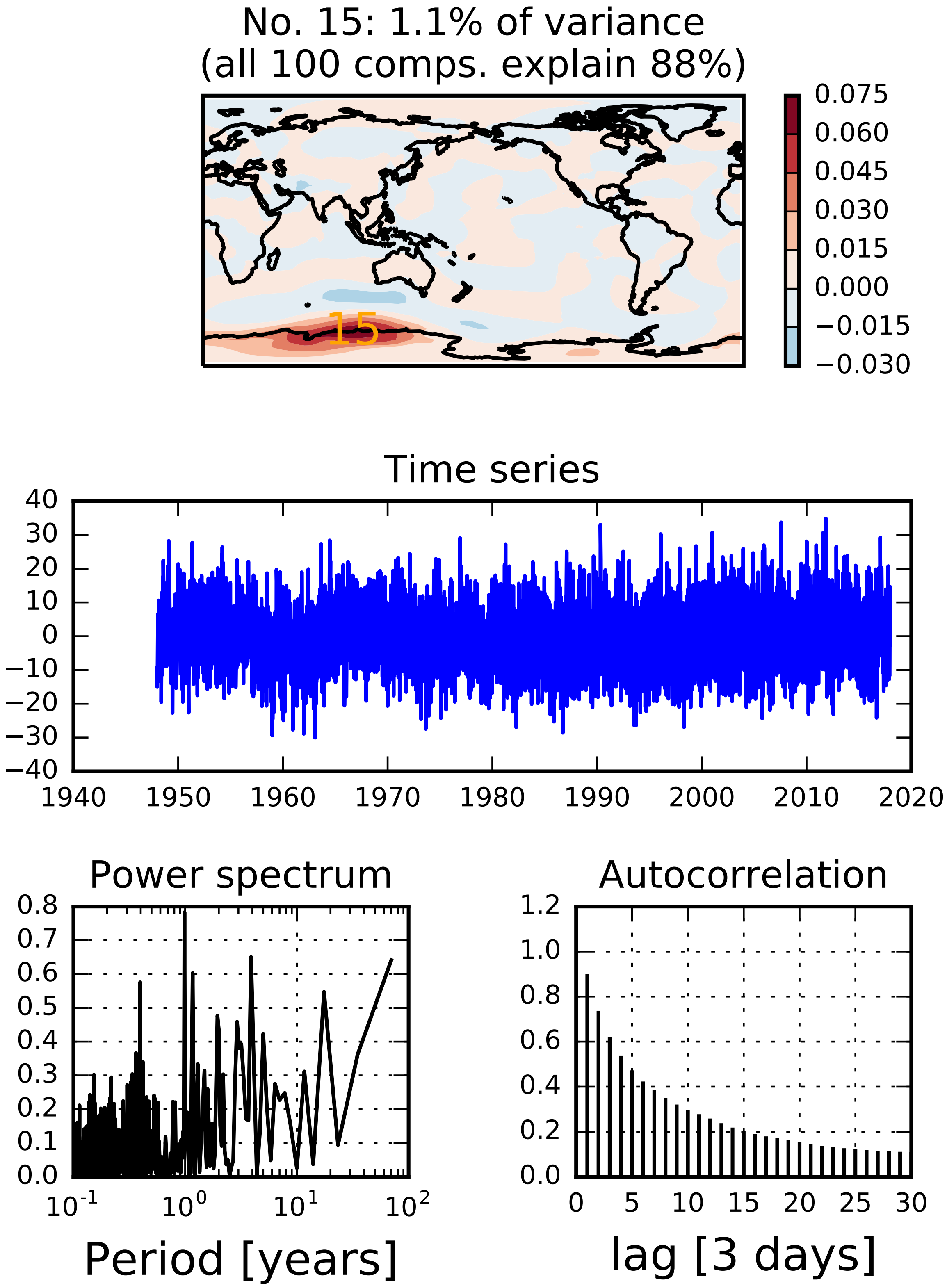
No. 15: 1.1% of variance
(all 100 comps. explain 88%)
15
0.075
0.060
0.045
0.030
0.015
0.000
−0.015
−0.030
Time series
40
30
20
10
0
−10
−20
−30
−40
1940
1950
1960
1970
1980
1990
2000
2010
2020
Power spectrum
0.8
0.7
0.6
0.5
0.4
0.3
0.2
0.1
0.0
10^-1
10^0
10^1
10^2
Period [years]
Autocorrelation
1.2
1.0
0.8
0.6
0.4
0.2
0.0
0
5
10
15
20
25
30
lag [3 days]

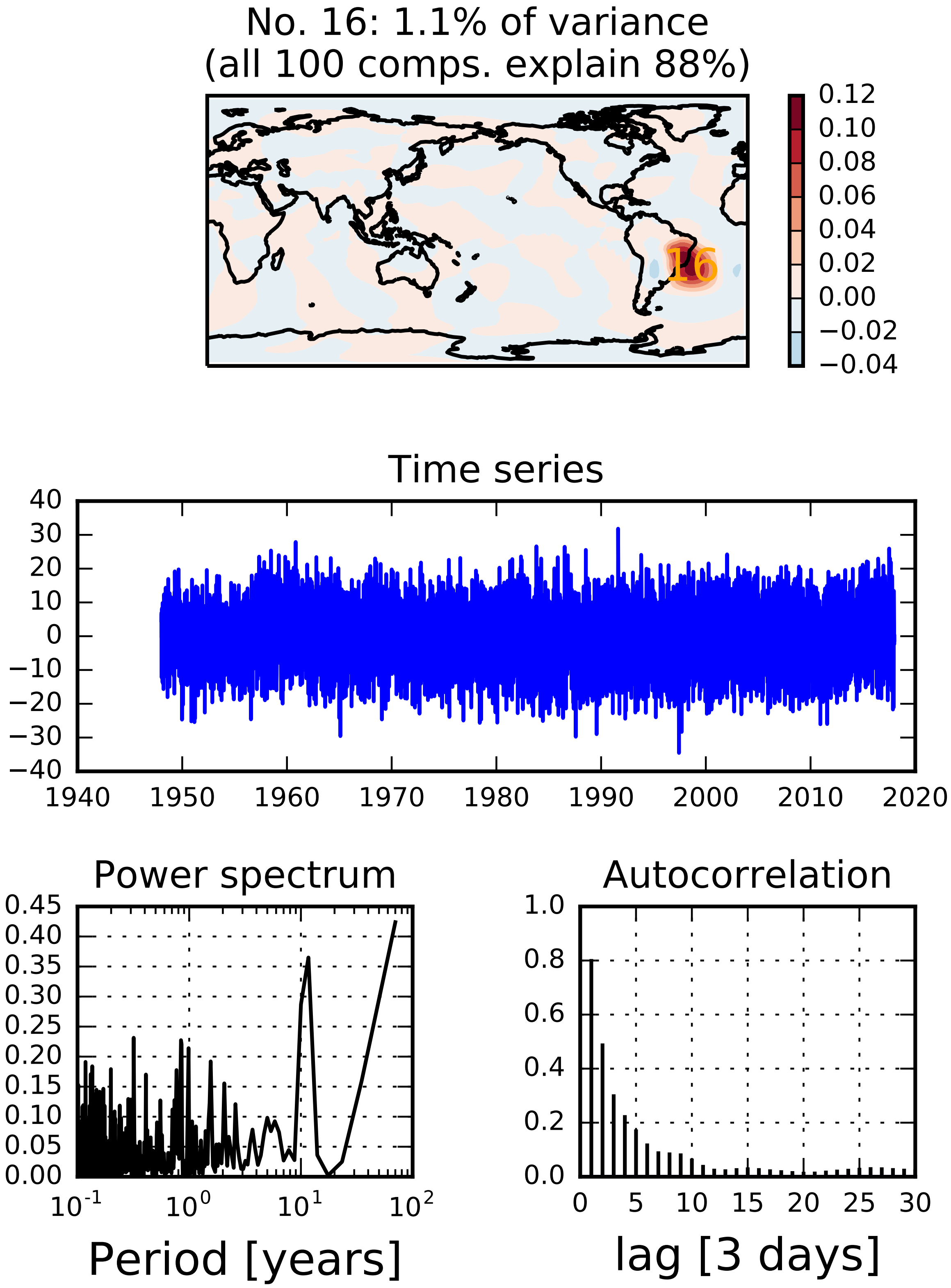
No. 16: 1.1% of variance
(all 100 comps. explain 88%)
16
0.12
0.10
0.08
0.06
0.04
0.02
0.00
−0.02
−0.04
Time series
40
30
20
10
0
−10
−20
−30
−40
1940
1950
1960
1970
1980
1990
2000
2010
2020
Power spectrum
0.45
0.40
0.35
0.30
0.25
0.20
0.15
0.10
0.05
0.00
10^-1
10^0
10^1
10^2
Period [years]
Autocorrelation
1.0
0.8
0.6
0.4
0.2
0.0
0
5
10
15
20
25
30
lag [3 days]

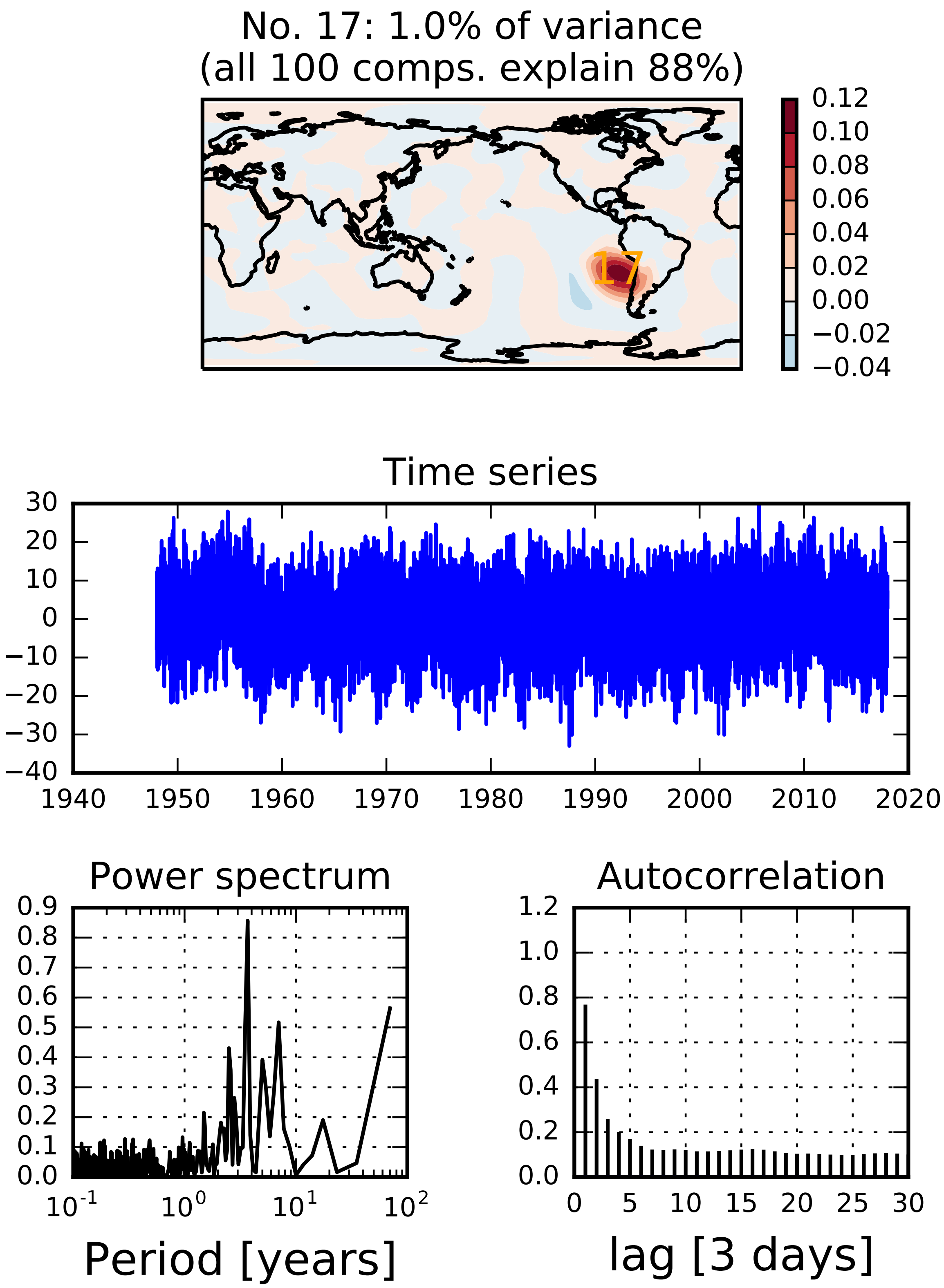
No. 17: 1.0% of variance
(all 100 comps. explain 88%)
17
0.12
0.10
0.08
0.06
0.04
0.02
0.00
−0.02
−0.04
Time series
30
20
10
0
−10
−20
−30
−40
1940
1950
1960
1970
1980
1990
2000
2010
2020
Power spectrum
0.9
0.8
0.7
0.6
0.5
0.4
0.3
0.2
0.1
0.0
10^{-1}
10^{0}
10^{1}
10^{2}
Period [years]
Autocorrelation
1.2
1.0
0.8
0.6
0.4
0.2
0.0
0
5
10
15
20
25
30
lag [3 days]

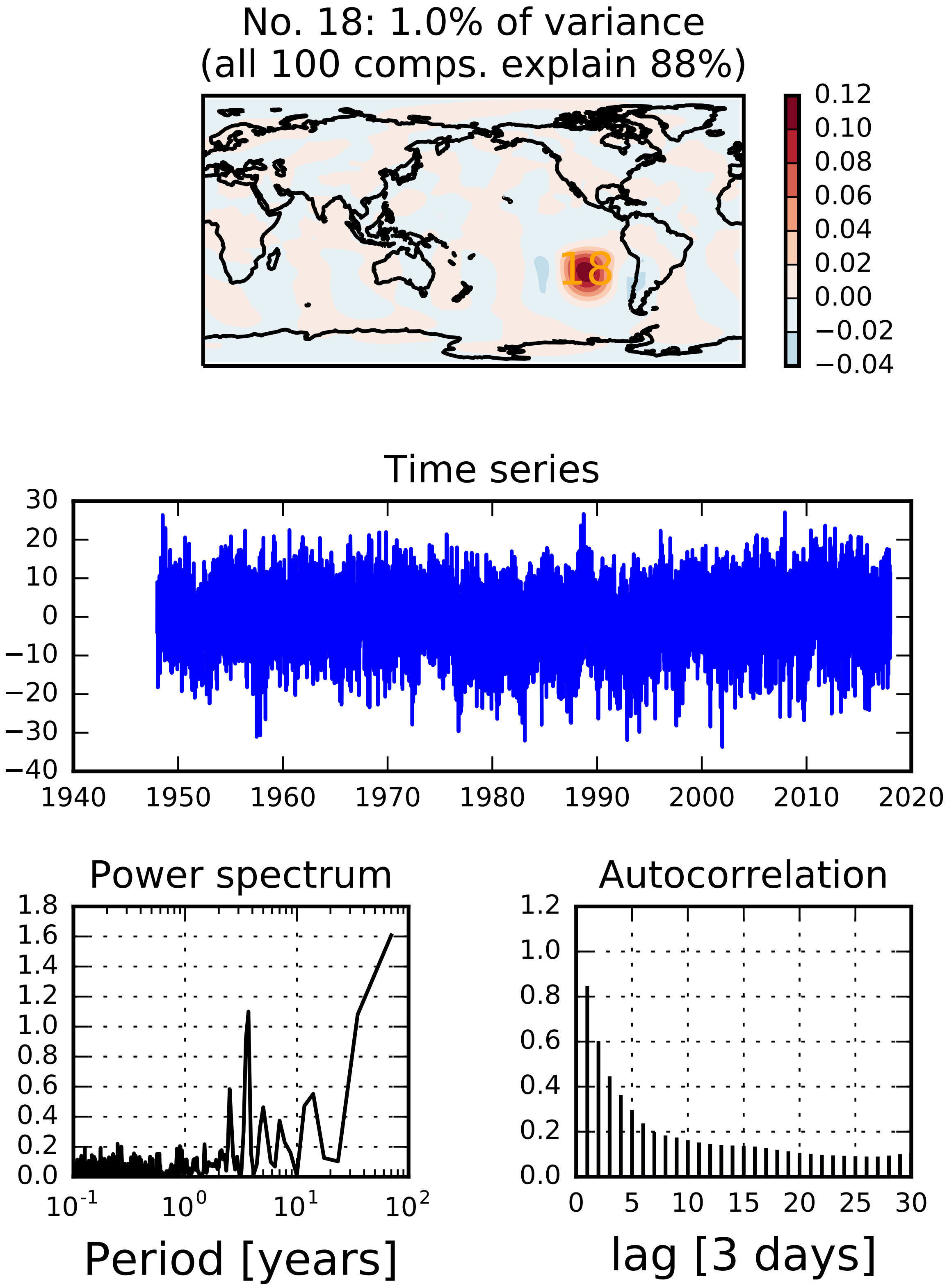
No. 18: 1.0% of variance
(all 100 comps. explain 88%)
18
0.12
0.10
0.08
0.06
0.04
0.02
0.00
−0.02
−0.04
Time series
30
20
10
0
−10
−20
−30
−40
1940
1950
1960
1970
1980
1990
2000
2010
2020
Power spectrum
1.8
1.6
1.4
1.2
1.0
0.8
0.6
0.4
0.2
0.0
10^{-1}
10^{0}
10^{1}
10^{2}
Period [years]
Autocorrelation
1.2
1.0
0.8
0.6
0.4
0.2
0.0
0
5
10
15
20
25
30
lag [3 days]

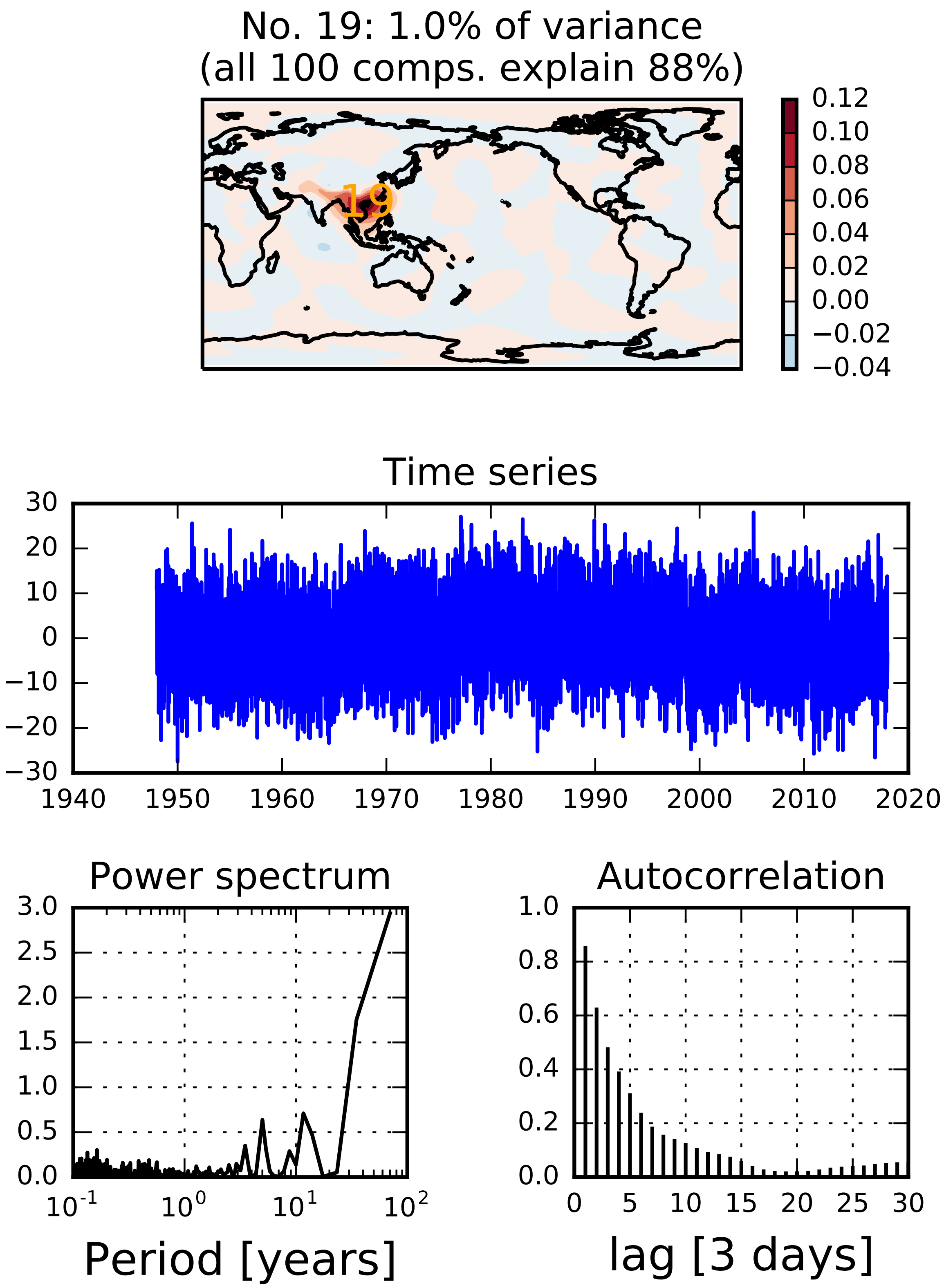

No. 19: 1.0% of variance
(all 100 comps. explain 88%)
19
0.12
0.10
0.08
0.06
0.04
0.02
0.00
−0.02
−0.04
Time series
30
20
10
0
−10
−20
−30
1940
1950
1960
1970
1980
1990
2000
2010
2020
Power spectrum
3.0
2.5
2.0
1.5
1.0
0.5
0.0
10^{-1}
10^{0}
10^{1}
10^{2}
Period [years]
Autocorrelation
1.0
0.8
0.6
0.4
0.2
0.0
0
5
10
15
20
25
30
lag [3 days]

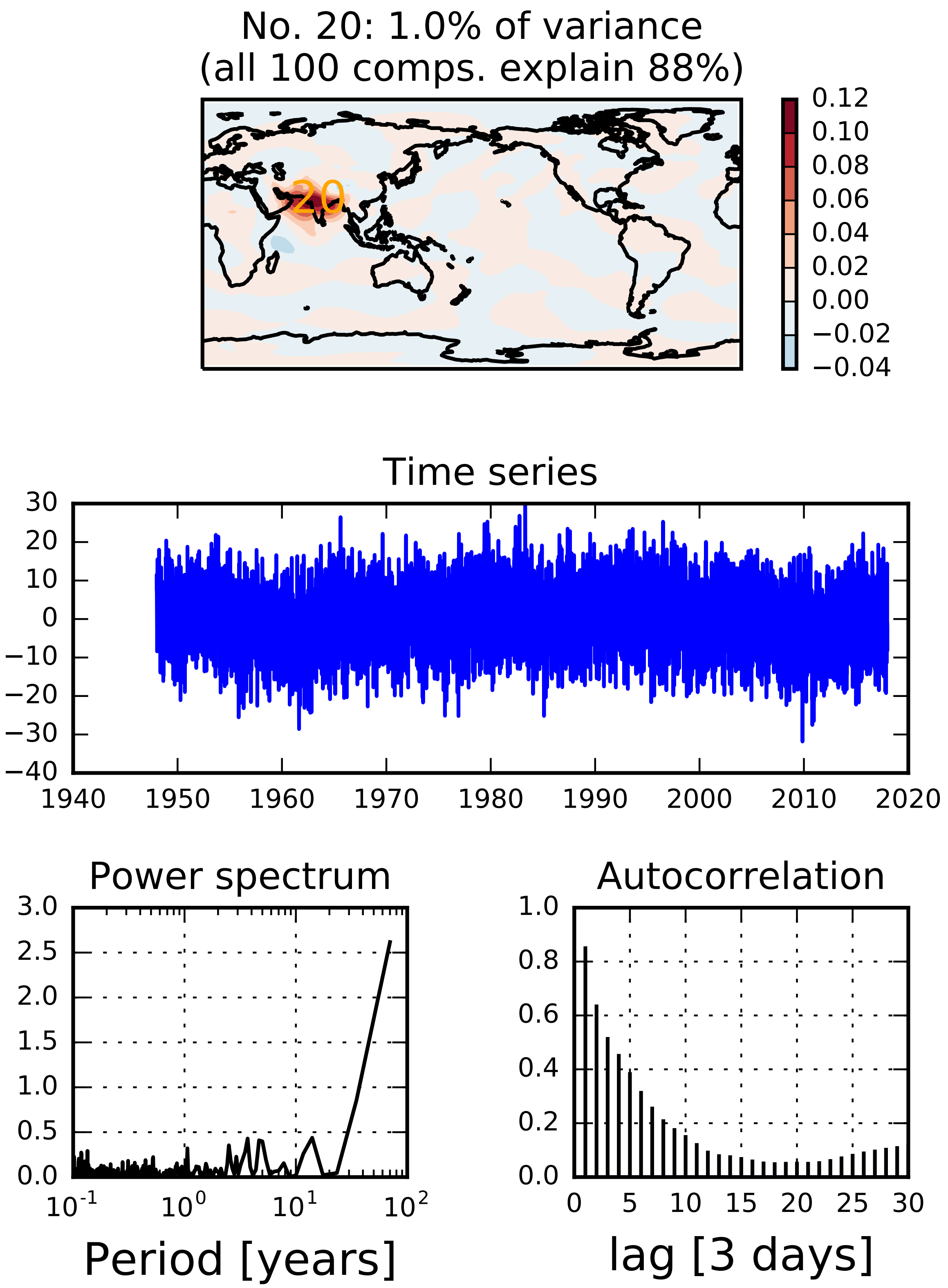
No. 20: 1.0% of variance
(all 100 comps. explain 88%)
20
0.12
0.10
0.08
0.06
0.04
0.02
0.00
−0.02
−0.04
Time series
30
20
10
0
−10
−20
−30
−40
1940
1950
1960
1970
1980
1990
2000
2010
2020
Power spectrum
3.0
2.5
2.0
1.5
1.0
0.5
0.0
10^{-1}
10^{0}
10^{1}
10^{2}
Period [years]
Autocorrelation
1.0
0.8
0.6
0.4
0.2
0.0
0
5
10
15
20
25
30
lag [3 days]

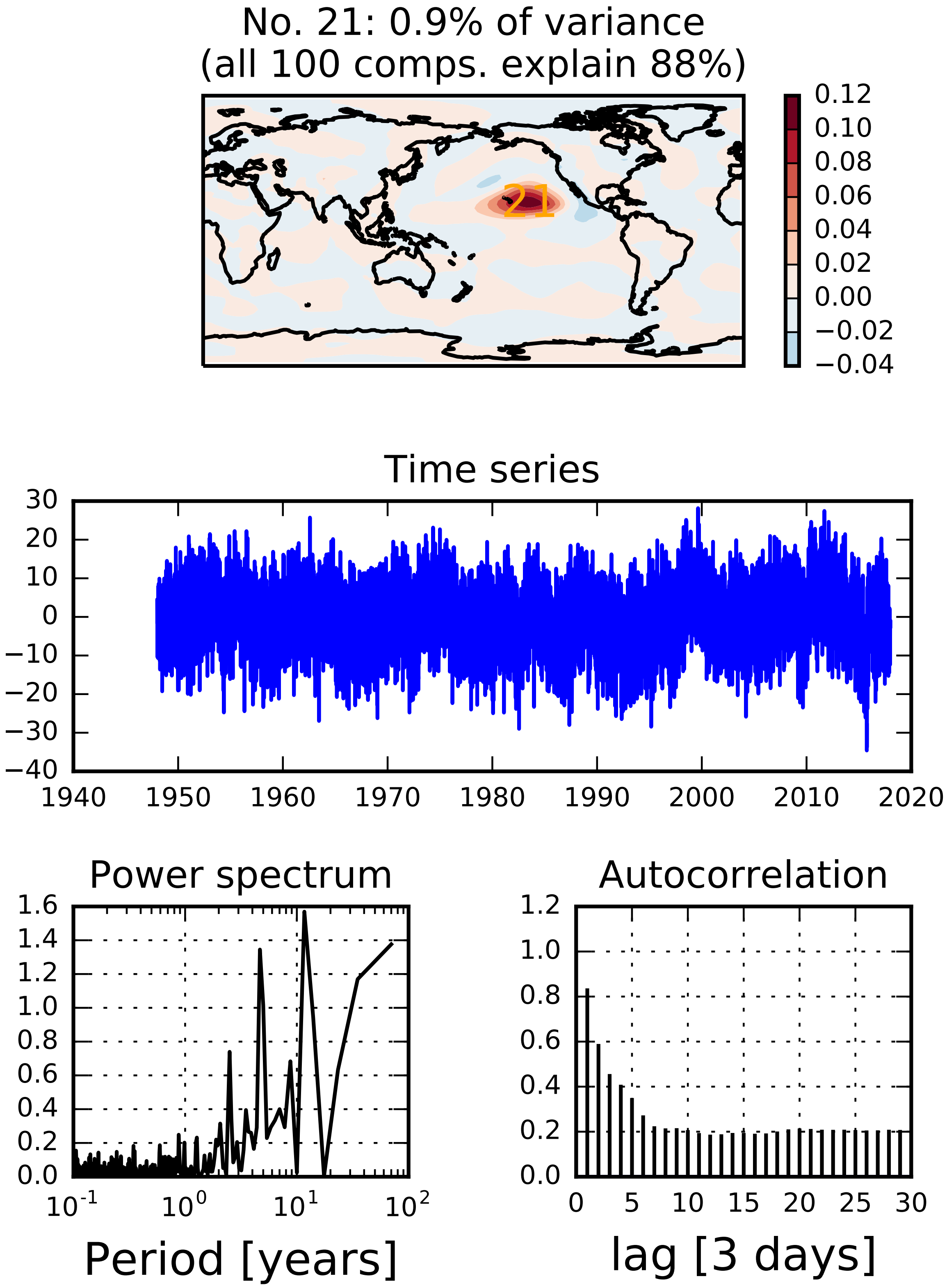
No. 21: 0.9% of variance
(all 100 comps. explain 88%)
21
0.12
0.10
0.08
0.06
0.04
0.02
0.00
−0.02
−0.04
Time series
30
20
10
0
−10
−20
−30
−40
1940
1950
1960
1970
1980
1990
2000
2010
2020
Power spectrum
1.6
1.4
1.2
1.0
0.8
0.6
0.4
0.2
0.0
10^{-1}
10^{0}
10^{1}
10^{2}
Period [years]
Autocorrelation
1.2
1.0
0.8
0.6
0.4
0.2
0.0
0
5
10
15
20
25
30
lag [3 days]

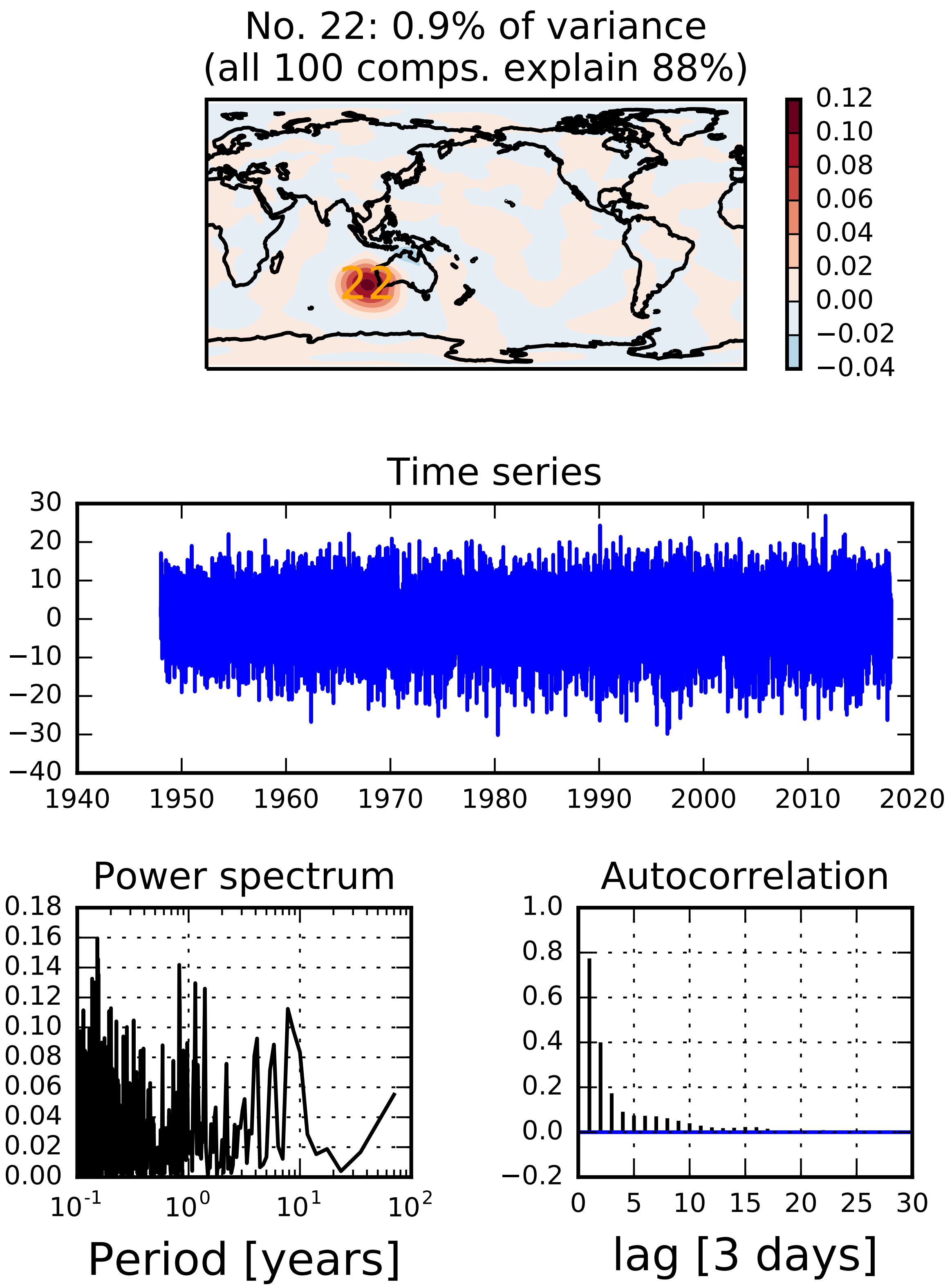
No. 22: 0.9% of variance
(all 100 comps. explain 88%)
22
0.12
0.10
0.08
0.06
0.04
0.02
0.00
−0.02
−0.04
Time series
30
20
10
0
−10
−20
−30
−40
1940
1950
1960
1970
1980
1990
2000
2010
2020
Power spectrum
0.18
0.16
0.14
0.12
0.10
0.08
0.06
0.04
0.02
0.00
10^{-1}
10^{0}
10^{1}
10^{2}
Period [years]
Autocorrelation
1.0
0.8
0.6
0.4
0.2
0.0
−0.2
0
5
10
15
20
25
30
lag [3 days]

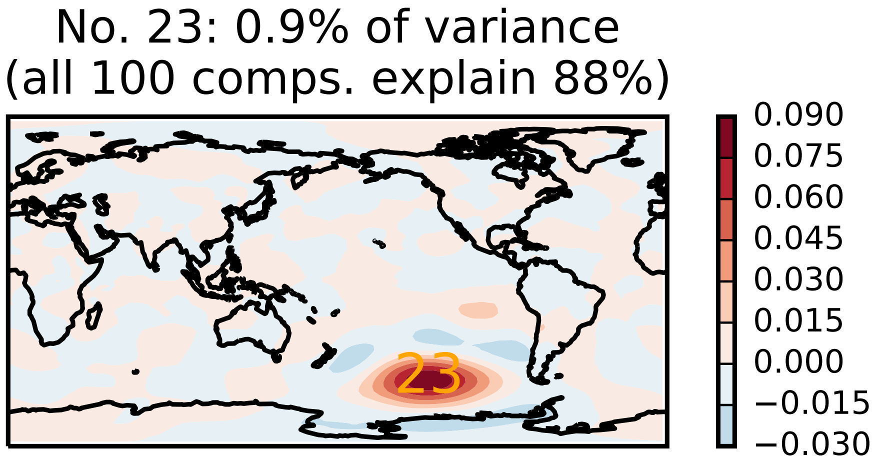
No. 23: 0.9% of variance
(all 100 comps. explain 88%)
23
0.090
0.075
0.060
0.045
0.030
0.015
0.000
−0.015
−0.030

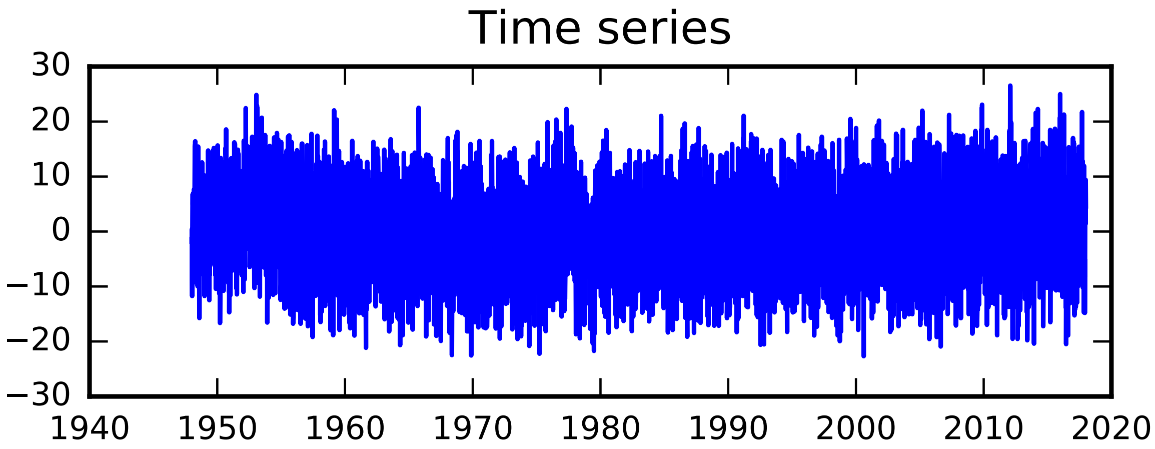
Time series
30
20
10
0
−10
−20
−30
1940
1950
1960
1970
1980
1990
2000
2010
2020

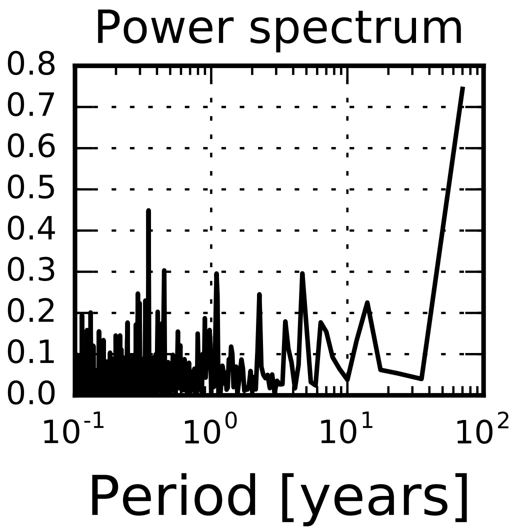
Power spectrum
0.8
0.7
0.6
0.5
0.4
0.3
0.2
0.1
0.0
10^{-1}
10^{0}
10^{1}
10^{2}
Period [years]

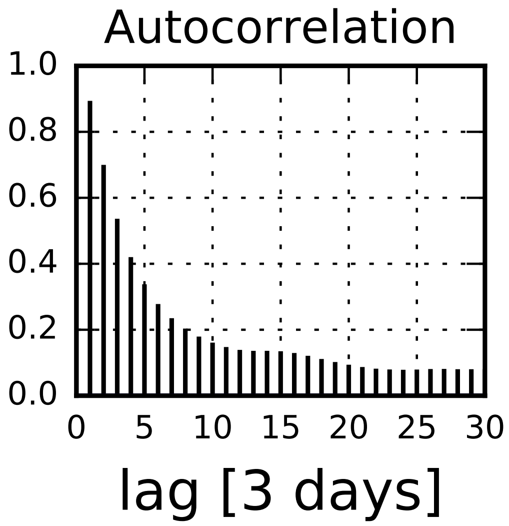
Autocorrelation
1.0
0.8
0.6
0.4
0.2
0.0
0
5
10
15
20
25
30
lag [3 days]

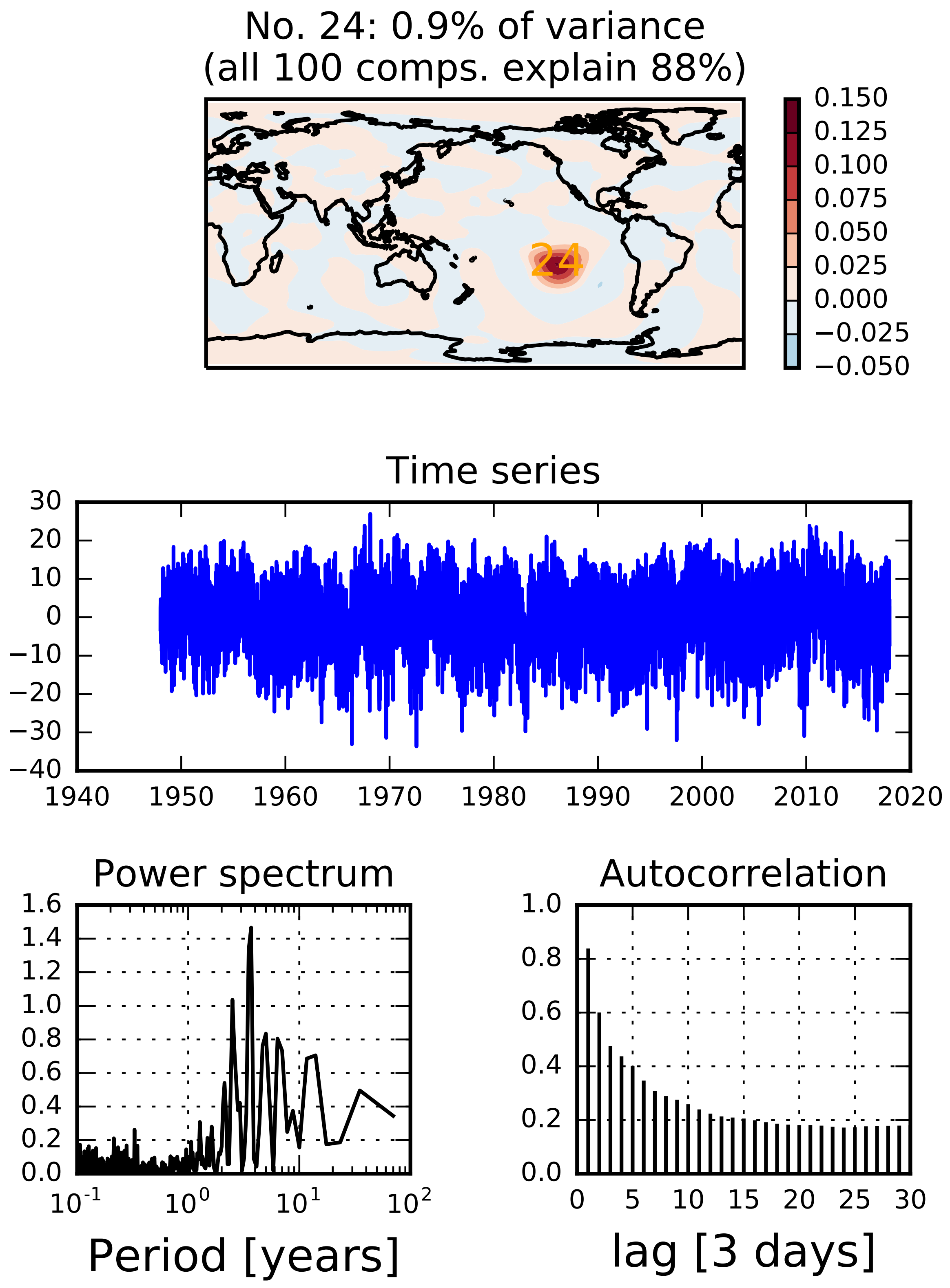
No. 24: 0.9% of variance
(all 100 comps. explain 88%)
24
0.150
0.125
0.100
0.075
0.050
0.025
0.000
−0.025
−0.050
Time series
30
20
10
0
−10
−20
−30
−40
1940
1950
1960
1970
1980
1990
2000
2010
2020
Power spectrum
1.6
1.4
1.2
1.0
0.8
0.6
0.4
0.2
0.0
10^{-1}
10^{0}
10^{1}
10^{2}
Period [years]
Autocorrelation
1.0
0.8
0.6
0.4
0.2
0.0
0
5
10
15
20
25
30
lag [3 days]

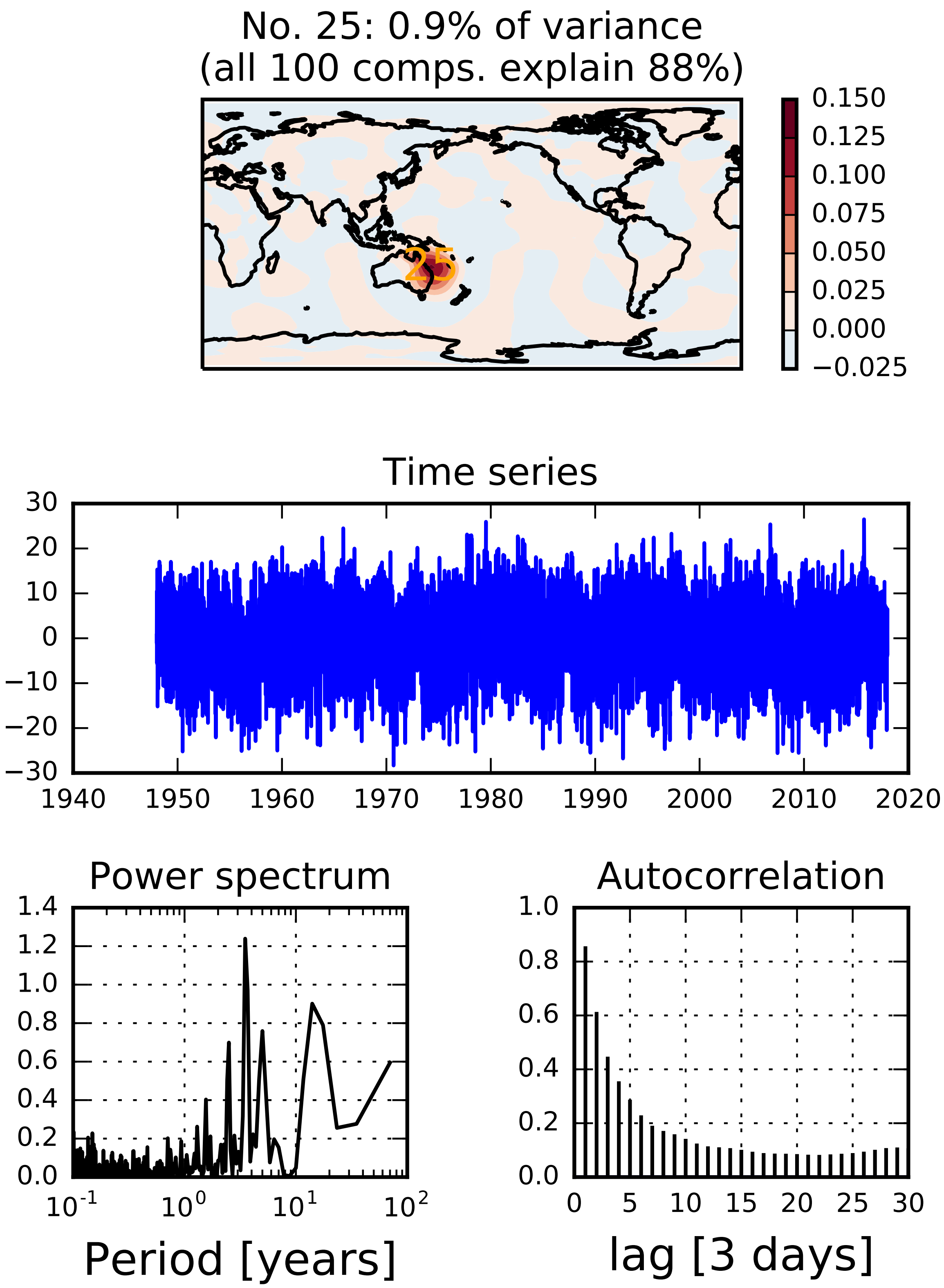
No. 25: 0.9% of variance
(all 100 comps. explain 88%)
0.150
0.125
0.100
0.075
0.050
0.025
0.000
−0.025
25
Time series
30
20
10
0
−10
−20
−30
1940
1950
1960
1970
1980
1990
2000
2010
2020
Power spectrum
1.4
1.2
1.0
0.8
0.6
0.4
0.2
0.0
10^{-1}
10^{0}
10^{1}
10^{2}
Period [years]
Autocorrelation
1.0
0.8
0.6
0.4
0.2
0.0
0
5
10
15
20
25
30
lag [3 days]

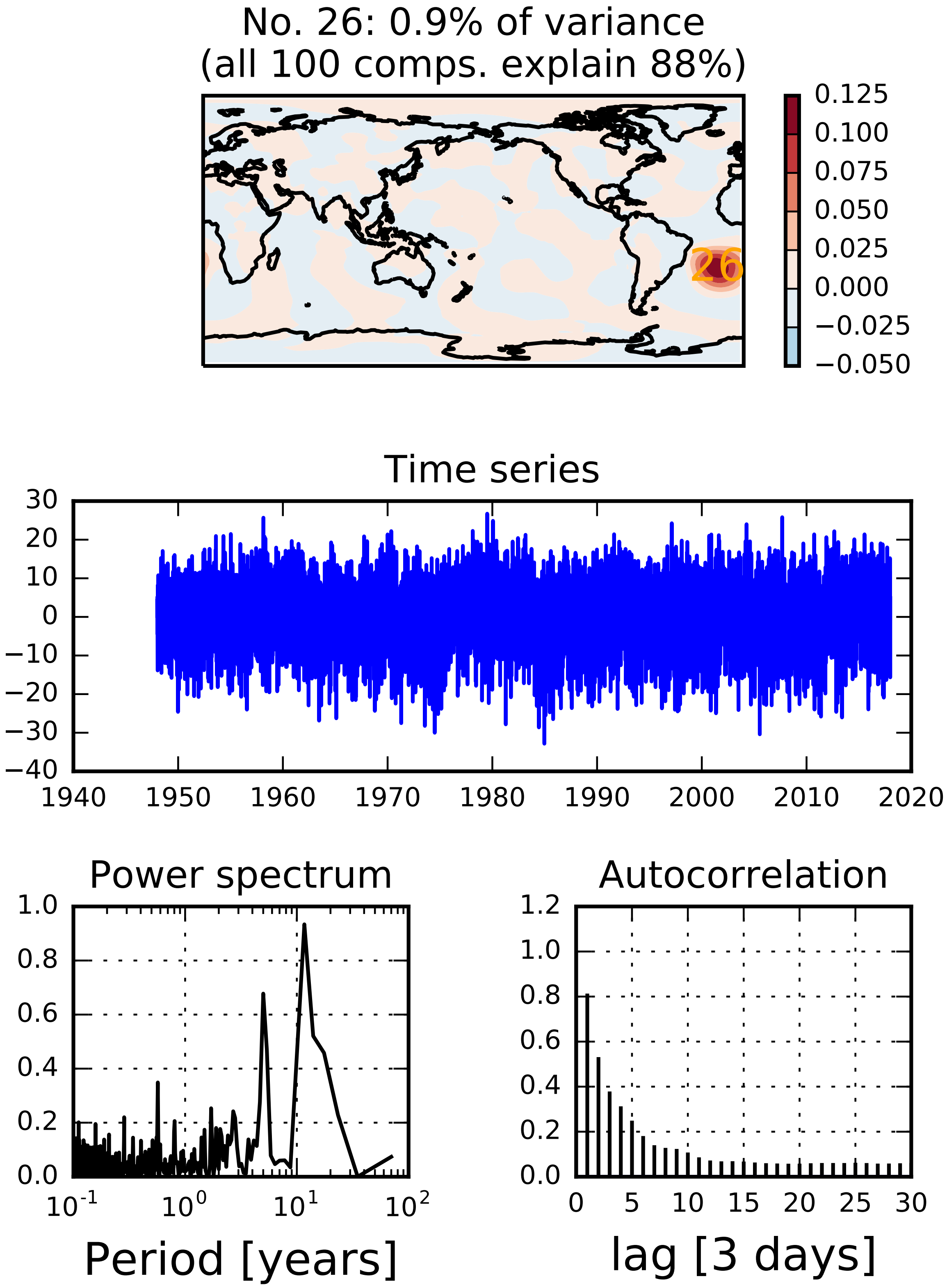
No. 26: 0.9% of variance
(all 100 comps. explain 88%)
26
0.125
0.100
0.075
0.050
0.025
0.000
−0.025
−0.050
Time series
30
20
10
0
−10
−20
−30
−40
1940
1950
1960
1970
1980
1990
2000
2010
2020
Power spectrum
1.0
0.8
0.6
0.4
0.2
0.0
10^{-1}
10^{0}
10^{1}
10^{2}
Period [years]
Autocorrelation
1.2
1.0
0.8
0.6
0.4
0.2
0.0
0
5
10
15
20
25
30
lag [3 days]

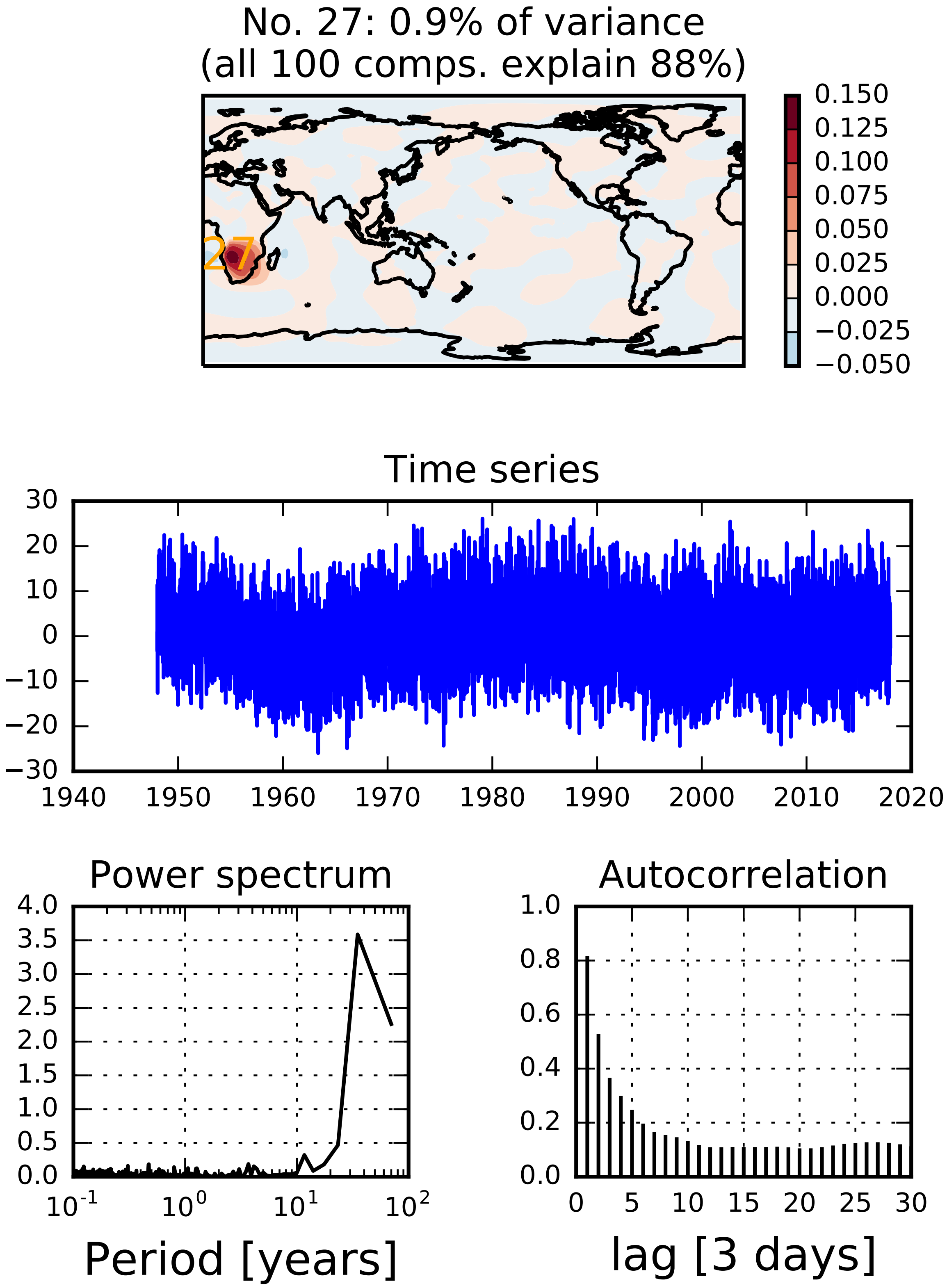
No. 27: 0.9% of variance
(all 100 comps. explain 88%)
27
0.150
0.125
0.100
0.075
0.050
0.025
0.000
−0.025
−0.050
Time series
30
20
10
0
−10
−20
−30
1940
1950
1960
1970
1980
1990
2000
2010
2020
Power spectrum
4.0
3.5
3.0
2.5
2.0
1.5
1.0
0.5
0.0
10^{-1}
10^{0}
10^{1}
10^{2}
Period [years]
Autocorrelation
1.0
0.8
0.6
0.4
0.2
0.0
0
5
10
15
20
25
30
lag [3 days]

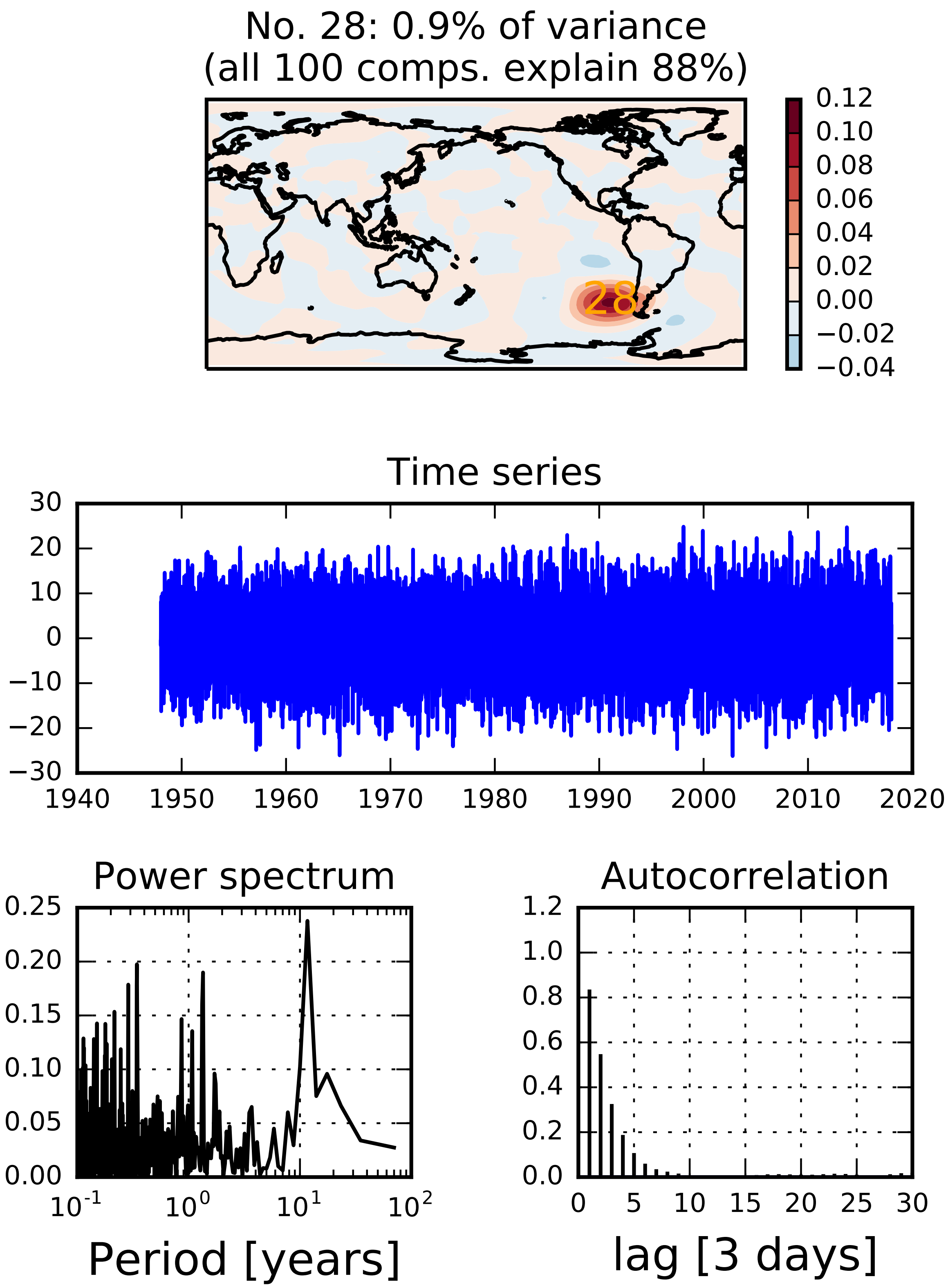
No. 28: 0.9% of variance
(all 100 comps. explain 88%)
28
0.12
0.10
0.08
0.06
0.04
0.02
0.00
−0.02
−0.04
Time series
30
20
10
0
−10
−20
−30
1940
1950
1960
1970
1980
1990
2000
2010
2020
Power spectrum
0.25
0.20
0.15
0.10
0.05
0.00
10^-1
10^0
10^1
10^2
Period [years]
Autocorrelation
1.2
1.0
0.8
0.6
0.4
0.2
0.0
0
5
10
15
20
25
30
lag [3 days]

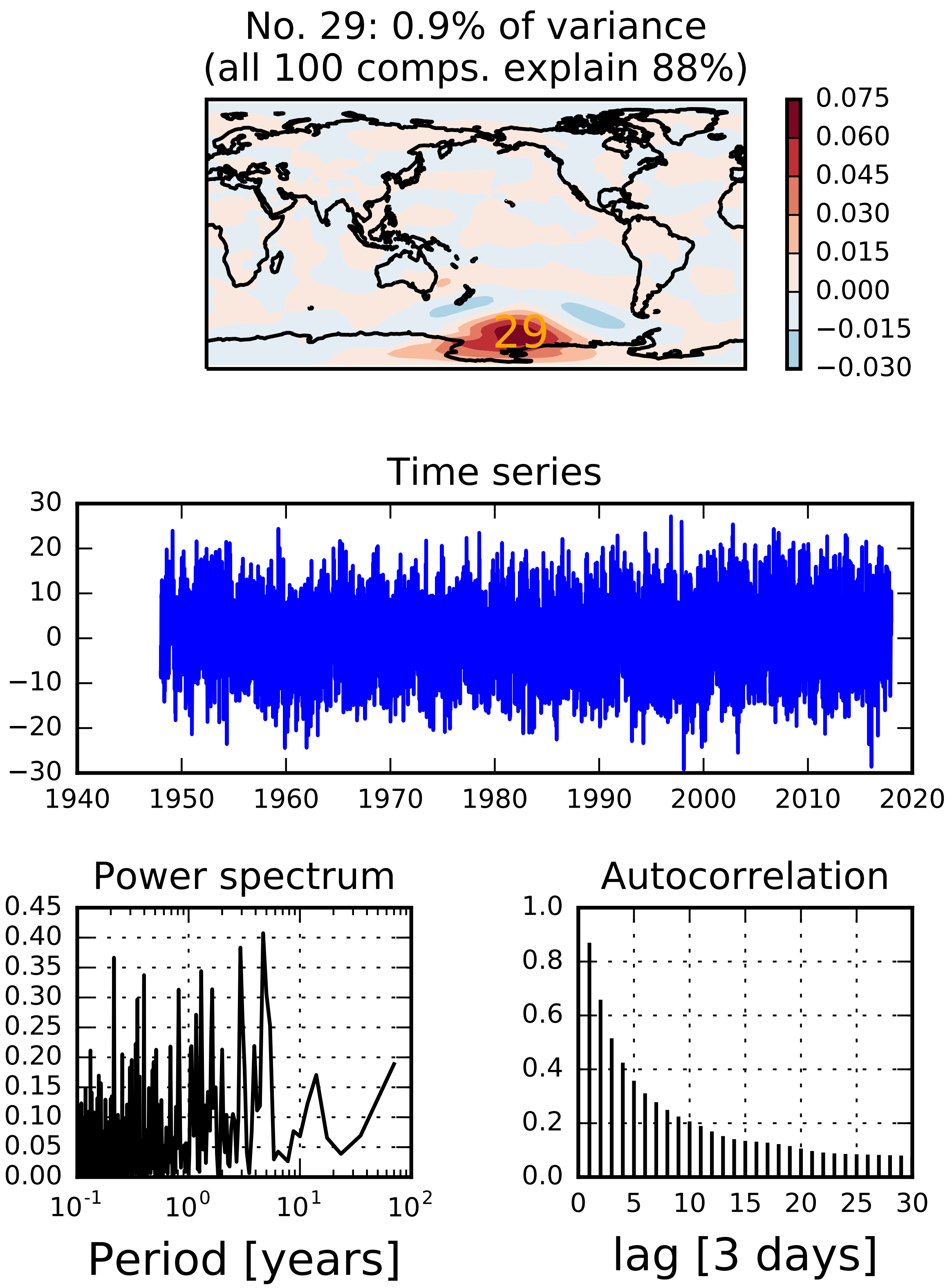
No. 29: 0.9% of variance
(all 100 comps. explain 88%)
29
0.075
0.060
0.045
0.030
0.015
0.000
−0.015
−0.030
Time series
30
20
10
0
−10
−20
−30
1940
1950
1960
1970
1980
1990
2000
2010
2020
Power spectrum
0.45
0.40
0.35
0.30
0.25
0.20
0.15
0.10
0.05
0.00
10^{-1}
10^{0}
10^{1}
10^{2}
Period [years]
Autocorrelation
1.0
0.8
0.6
0.4
0.2
0.0
0
5
10
15
20
25
30
lag [3 days]

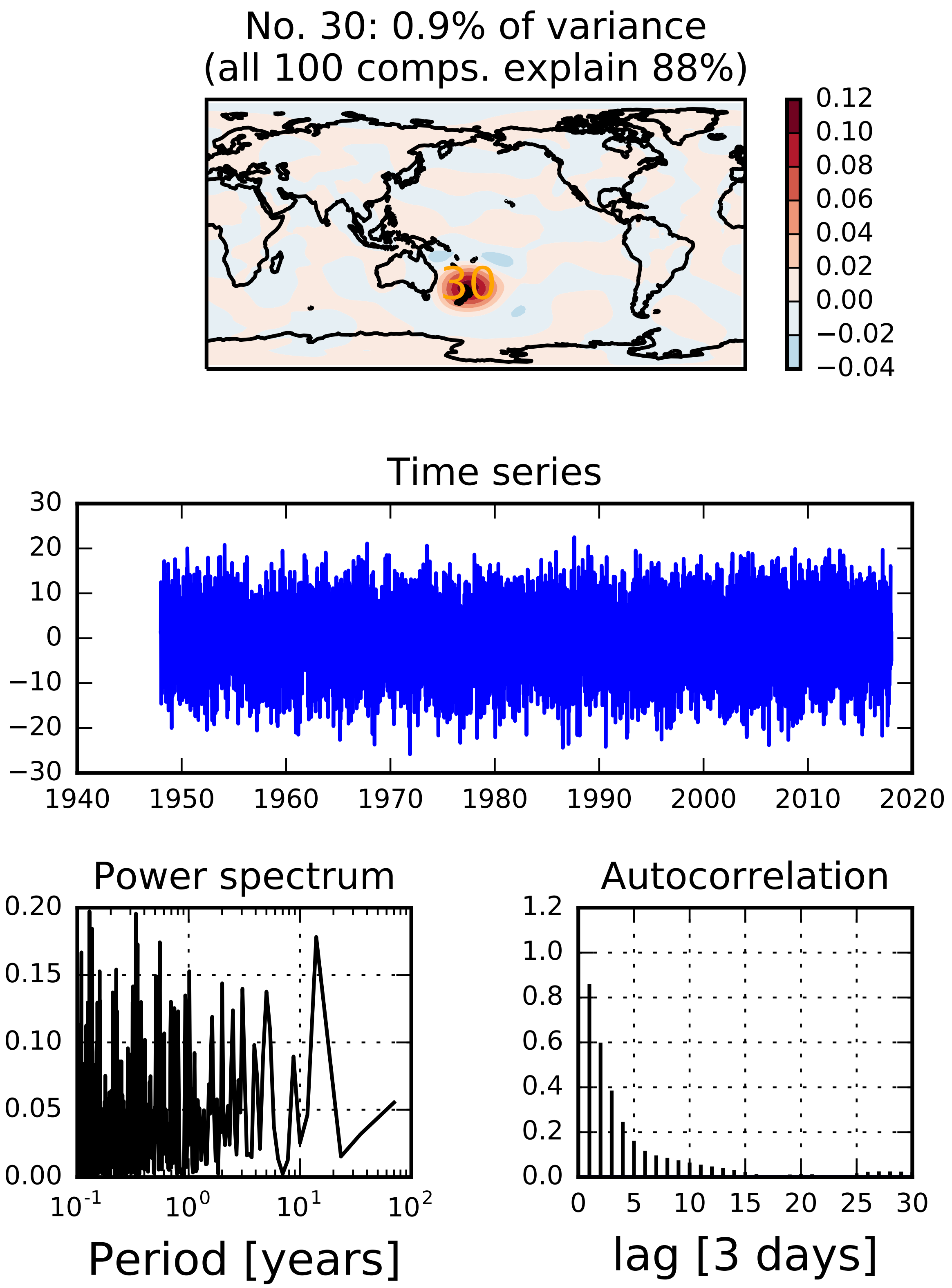
No. 30: 0.9% of variance
(all 100 comps. explain 88%)
30
0.12
0.10
0.08
0.06
0.04
0.02
0.00
−0.02
−0.04
Time series
30
20
10
0
−10
−20
−30
1940
1950
1960
1970
1980
1990
2000
2010
2020
Power spectrum
0.20
0.15
0.10
0.05
0.00
10^{-1}
10^{0}
10^{1}
10^{2}
Period [years]
Autocorrelation
1.2
1.0
0.8
0.6
0.4
0.2
0.0
0
5
10
15
20
25
30
lag [3 days]

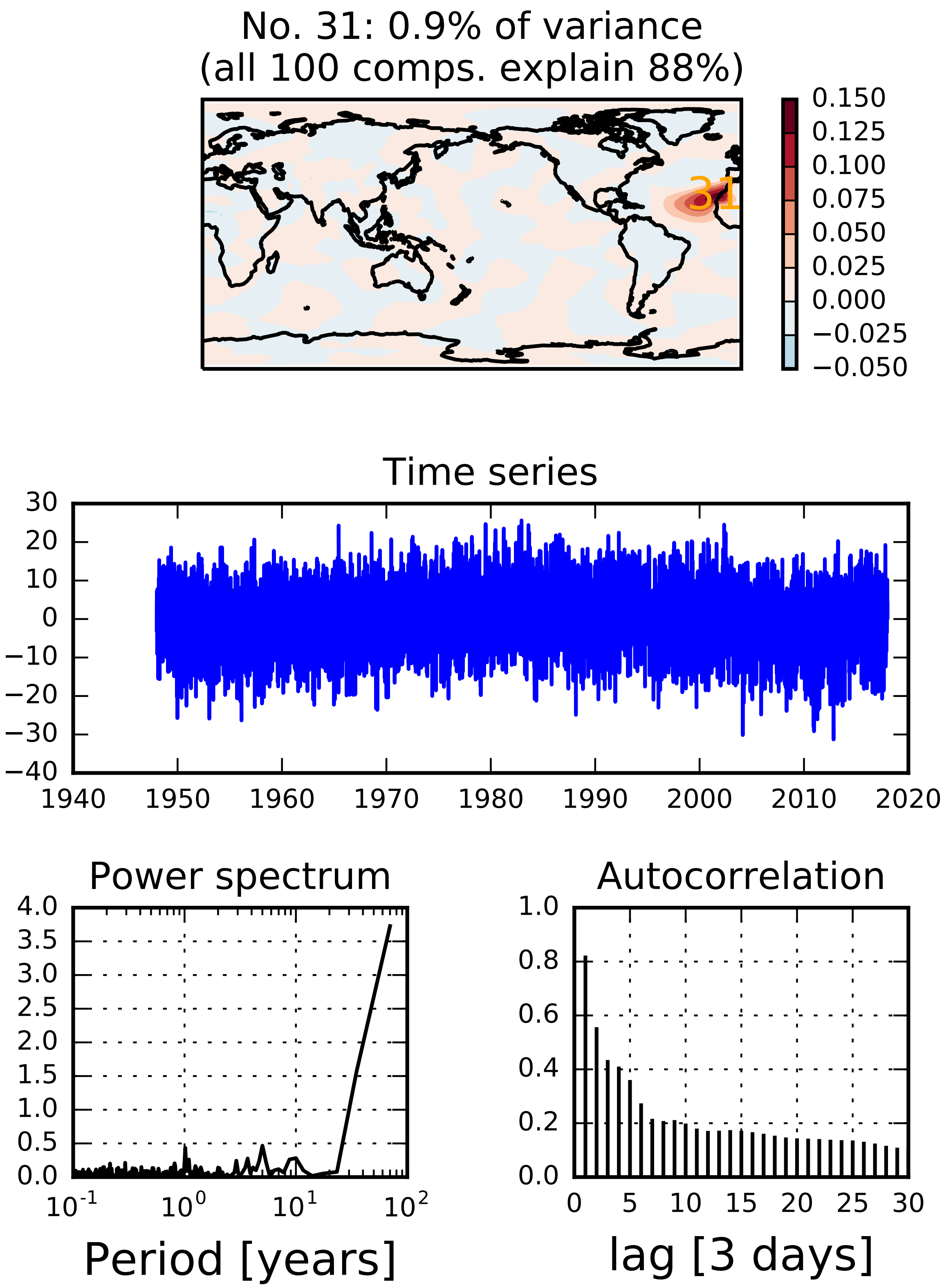
No. 31: 0.9% of variance
(all 100 comps. explain 88%)
31
0.150
0.125
0.100
0.075
0.050
0.025
0.000
−0.025
−0.050
Time series
30
20
10
0
−10
−20
−30
−40
1940
1950
1960
1970
1980
1990
2000
2010
2020
Power spectrum
4.0
3.5
3.0
2.5
2.0
1.5
1.0
0.5
0.0
10^{-1}
10^{0}
10^{1}
10^{2}
Period [years]
Autocorrelation
1.0
0.8
0.6
0.4
0.2
0.0
0
5
10
15
20
25
30
lag [3 days]

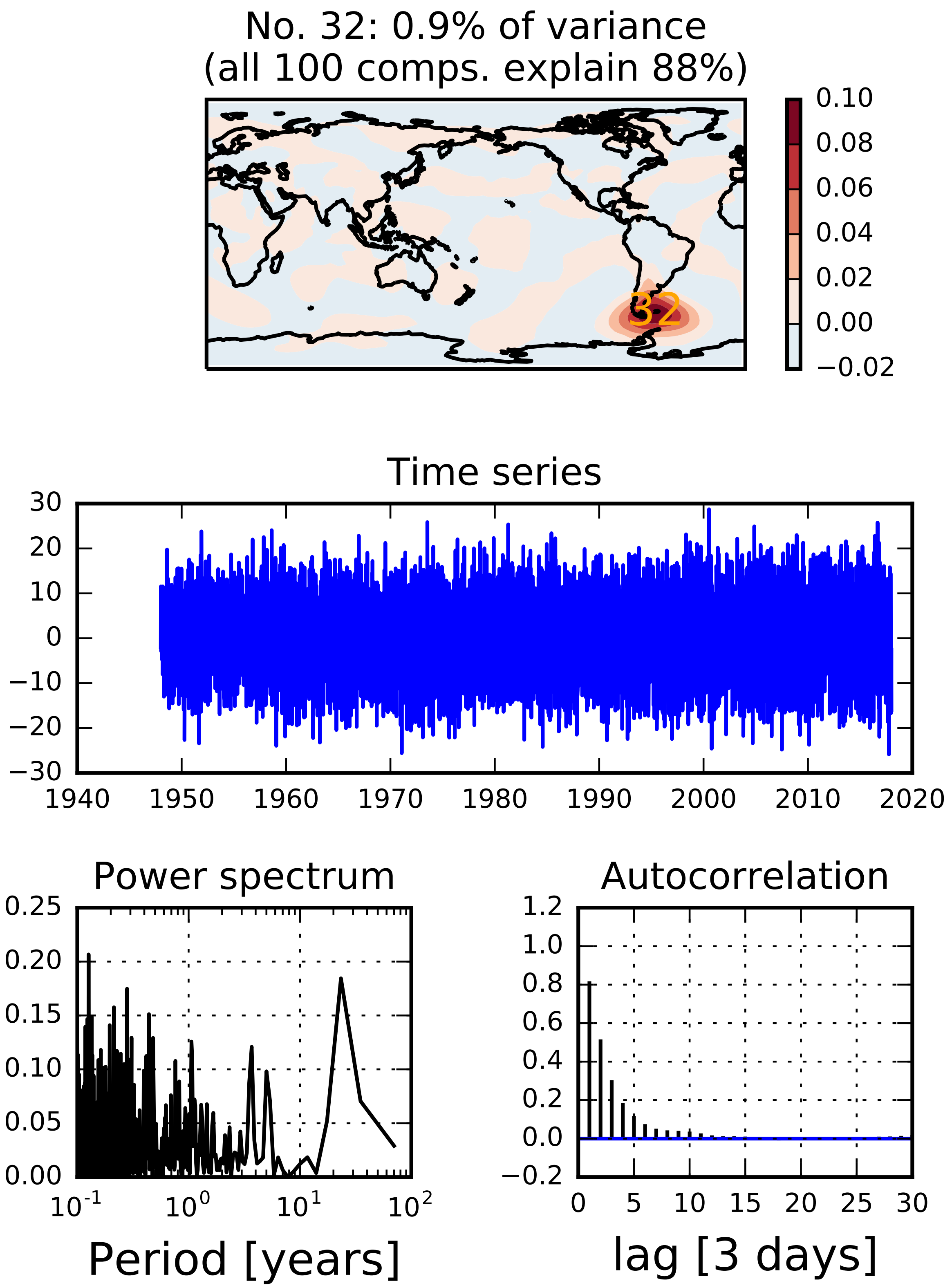
No. 32: 0.9% of variance
(all 100 comps. explain 88%)
32
0.10
0.08
0.06
0.04
0.02
0.00
−0.02
Time series
30
20
10
0
−10
−20
−30
1940
1950
1960
1970
1980
1990
2000
2010
2020
Power spectrum
0.25
0.20
0.15
0.10
0.05
0.00
10^{-1}
10^{0}
10^{1}
10^{2}
Period [years]
Autocorrelation
1.2
1.0
0.8
0.6
0.4
0.2
0.0
−0.2
0
5
10
15
20
25
30
lag [3 days]

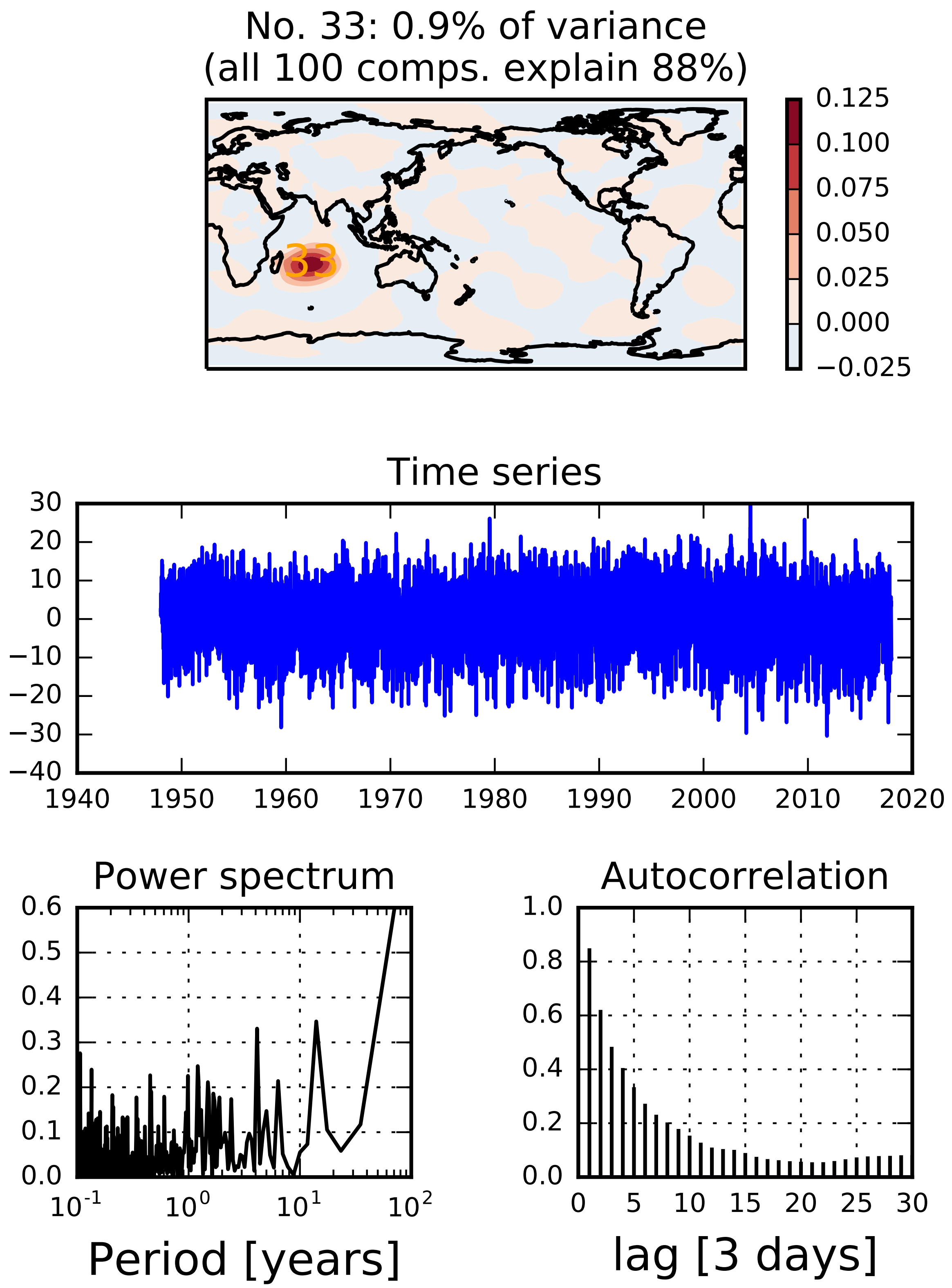
No. 33: 0.9% of variance
(all 100 comps. explain 88%)
33
0.125
0.100
0.075
0.050
0.025
0.000
−0.025
Time series
30
20
10
0
−10
−20
−30
−40
1940
1950
1960
1970
1980
1990
2000
2010
2020
Power spectrum
0.6
0.5
0.4
0.3
0.2
0.1
0.0
10^{-1}
10^{0}
10^{1}
10^{2}
Period [years]
Autocorrelation
1.0
0.8
0.6
0.4
0.2
0.0
0
5
10
15
20
25
30
lag [3 days]

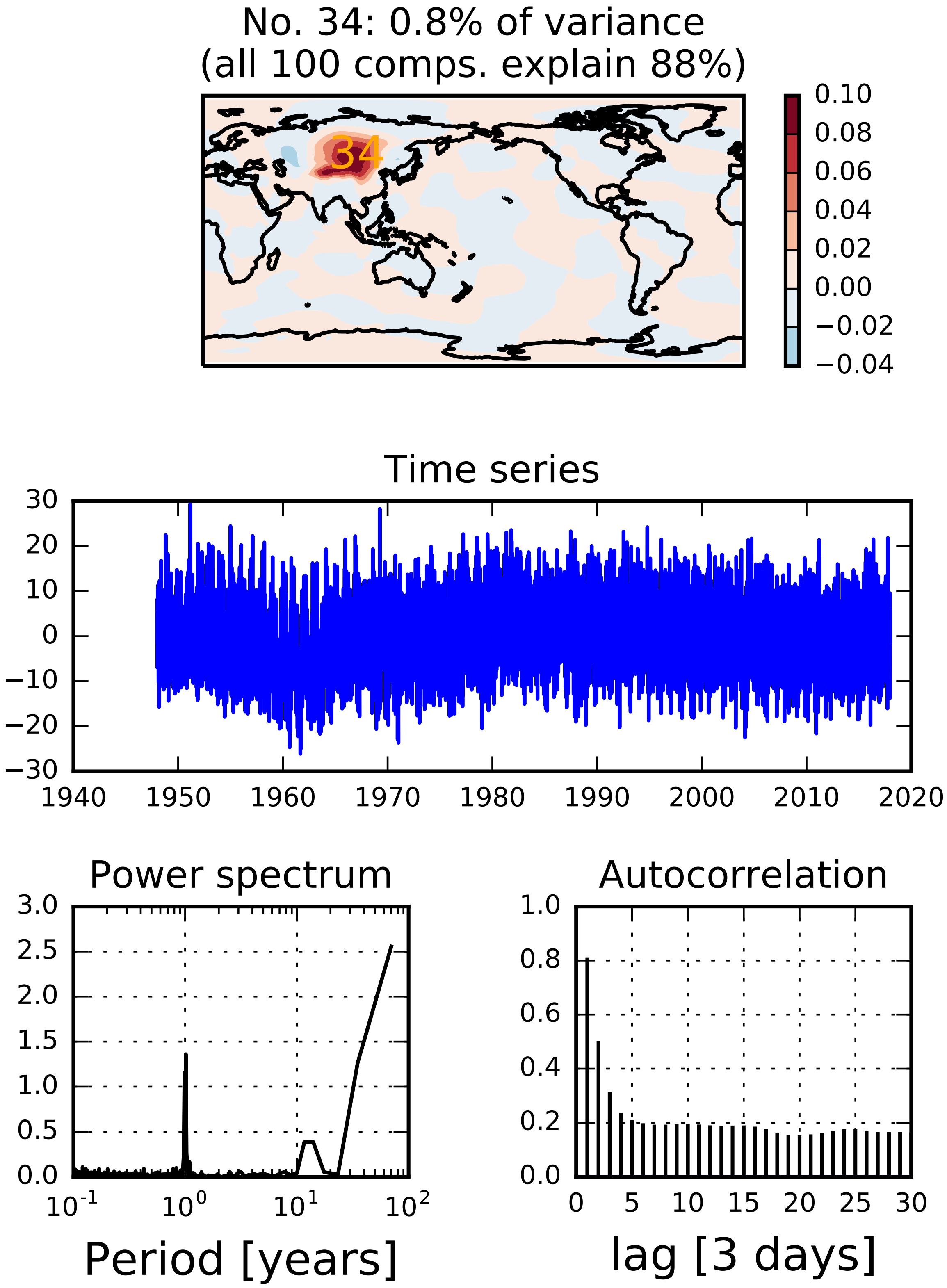
No. 34: 0.8% of variance
(all 100 comps. explain 88%)
34
0.10
0.08
0.06
0.04
0.02
0.00
−0.02
−0.04
Time series
30
20
10
0
−10
−20
−30
1940
1950
1960
1970
1980
1990
2000
2010
2020
Power spectrum
3.0
2.5
2.0
1.5
1.0
0.5
0.0
10^{-1}
10^{0}
10^{1}
10^{2}
Period [years]
Autocorrelation
1.0
0.8
0.6
0.4
0.2
0.0
0
5
10
15
20
25
30
lag [3 days]

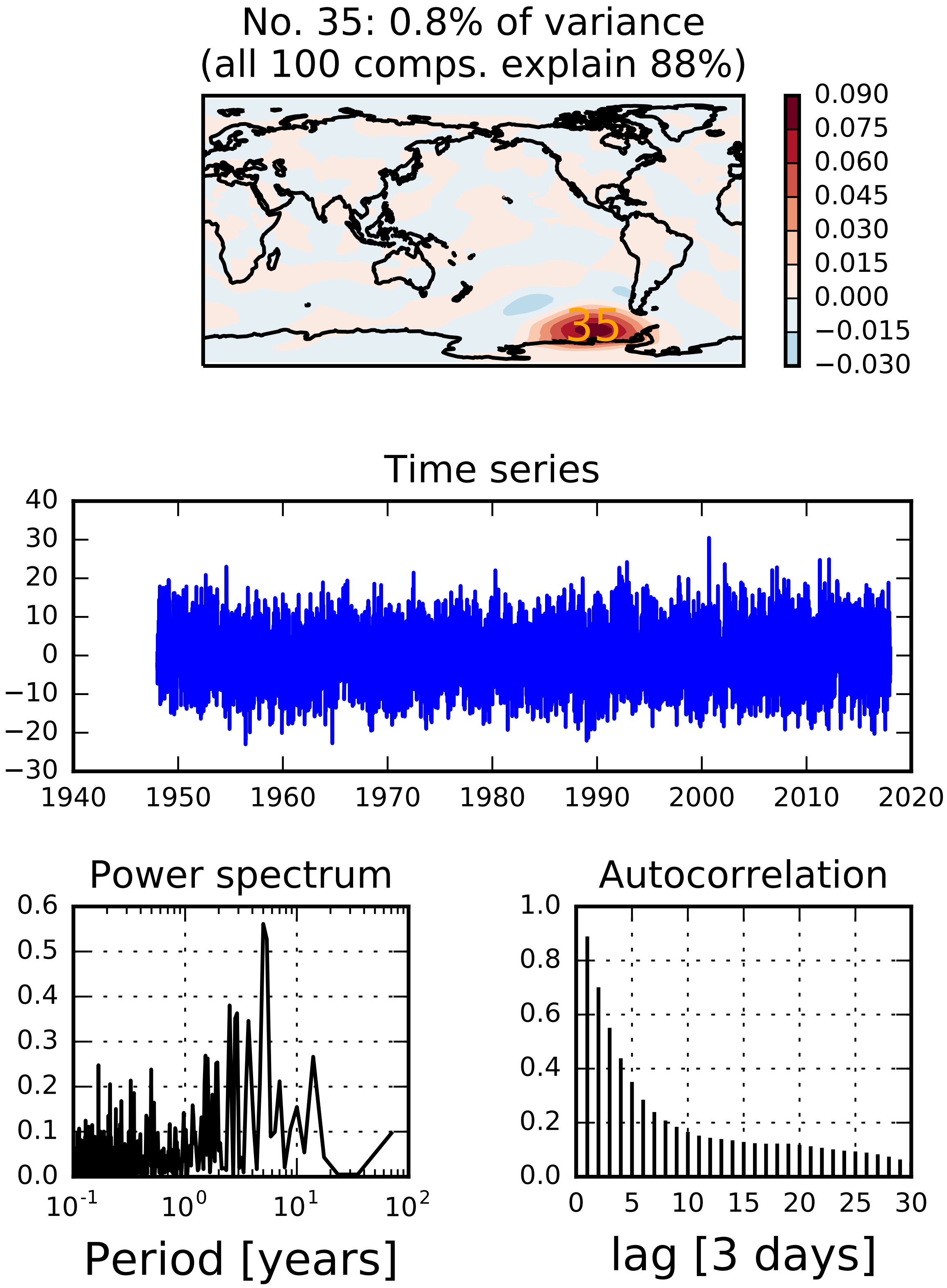

No. 35: 0.8% of variance
(all 100 comps. explain 88%)
35
0.090
0.075
0.060
0.045
0.030
0.015
0.000
−0.015
−0.030
Time series
40
30
20
10
0
−10
−20
−30
1940
1950
1960
1970
1980
1990
2000
2010
2020
Power spectrum
0.6
0.5
0.4
0.3
0.2
0.1
0.0
10^{-1}
10^{0}
10^{1}
10^{2}
Period [years]
Autocorrelation
1.0
0.8
0.6
0.4
0.2
0.0
0
5
10
15
20
25
30
lag [3 days]

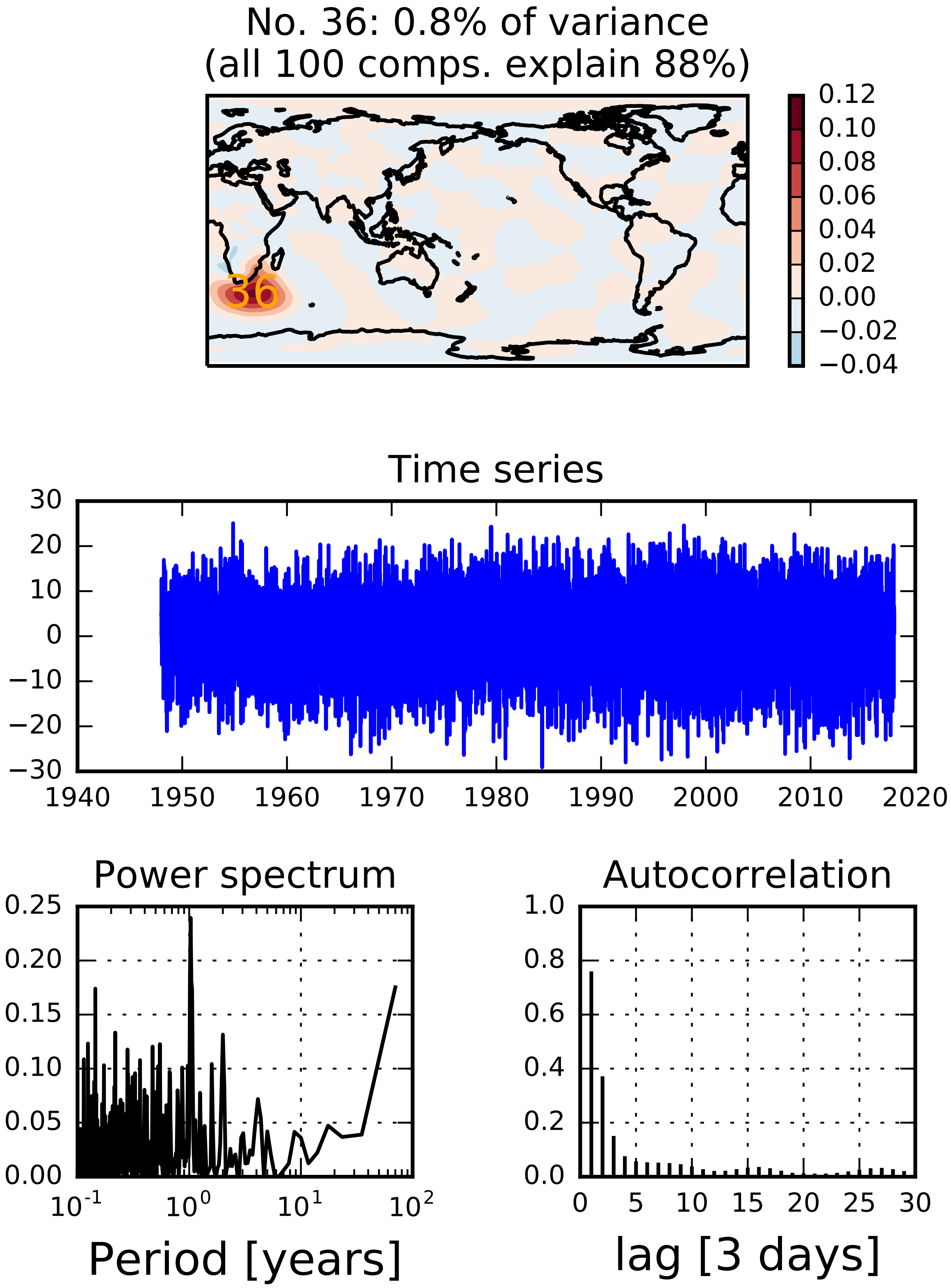
No. 36: 0.8% of variance
(all 100 comps. explain 88%)
36
0.12
0.10
0.08
0.06
0.04
0.02
0.00
−0.02
−0.04
Time series
30
20
10
0
−10
−20
−30
1940
1950
1960
1970
1980
1990
2000
2010
2020
Power spectrum
0.25
0.20
0.15
0.10
0.05
0.00
10^{-1}
10^{0}
10^{1}
10^{2}
Period [years]
Autocorrelation
1.0
0.8
0.6
0.4
0.2
0.0
0
5
10
15
20
25
30
lag [3 days]

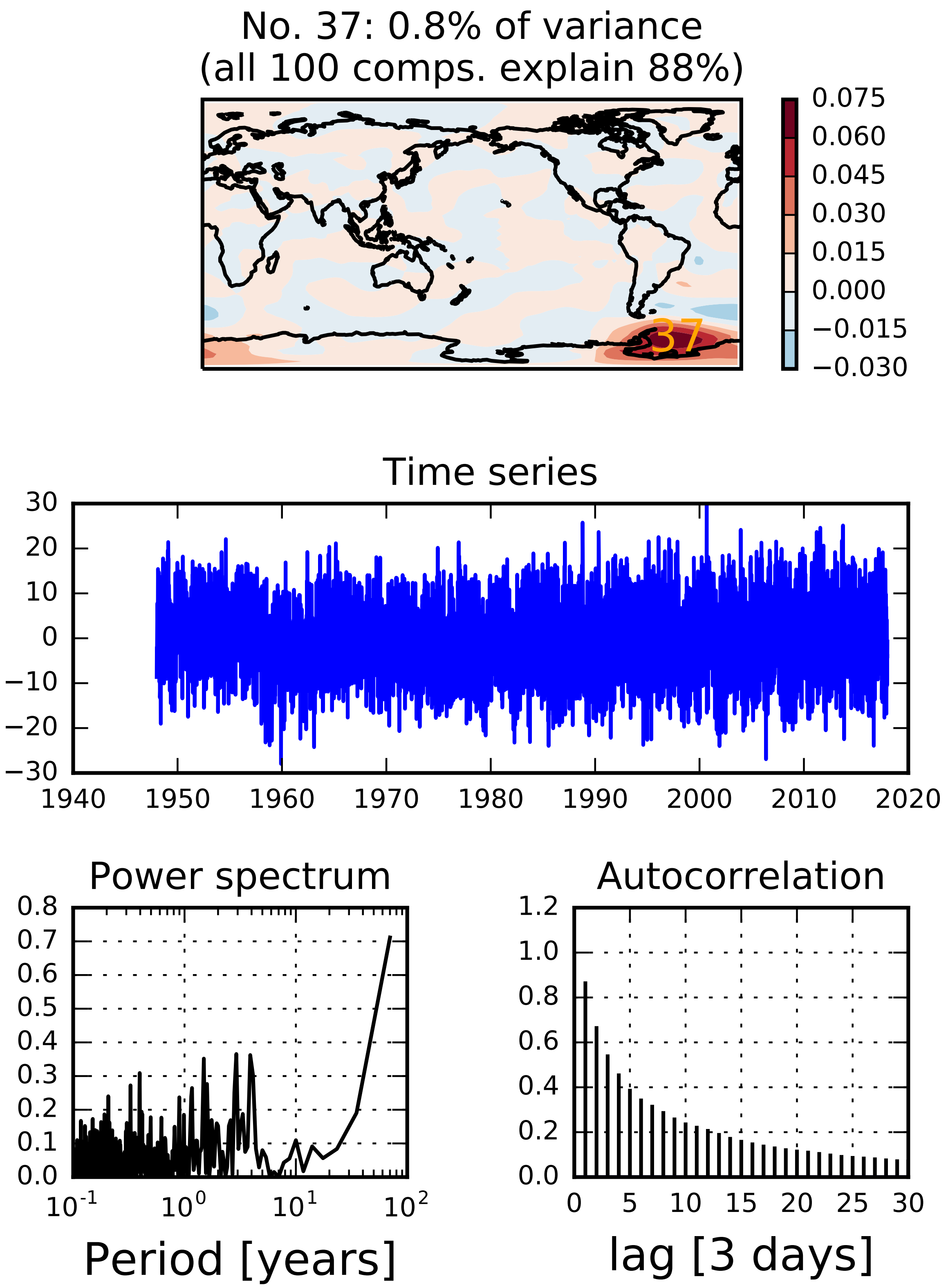

No. 37: 0.8% of variance
(all 100 comps. explain 88%)
0.075
0.060
0.045
0.030
0.015
0.000
−0.015
−0.030
37
Time series
30
20
10
0
−10
−20
−30
1940
1950
1960
1970
1980
1990
2000
2010
2020
Power spectrum
0.8
0.7
0.6
0.5
0.4
0.3
0.2
0.1
0.0
10^-1
10^0
10^1
10^2
Period [years]
Autocorrelation
1.2
1.0
0.8
0.6
0.4
0.2
0.0
0
5
10
15
20
25
30
lag [3 days]

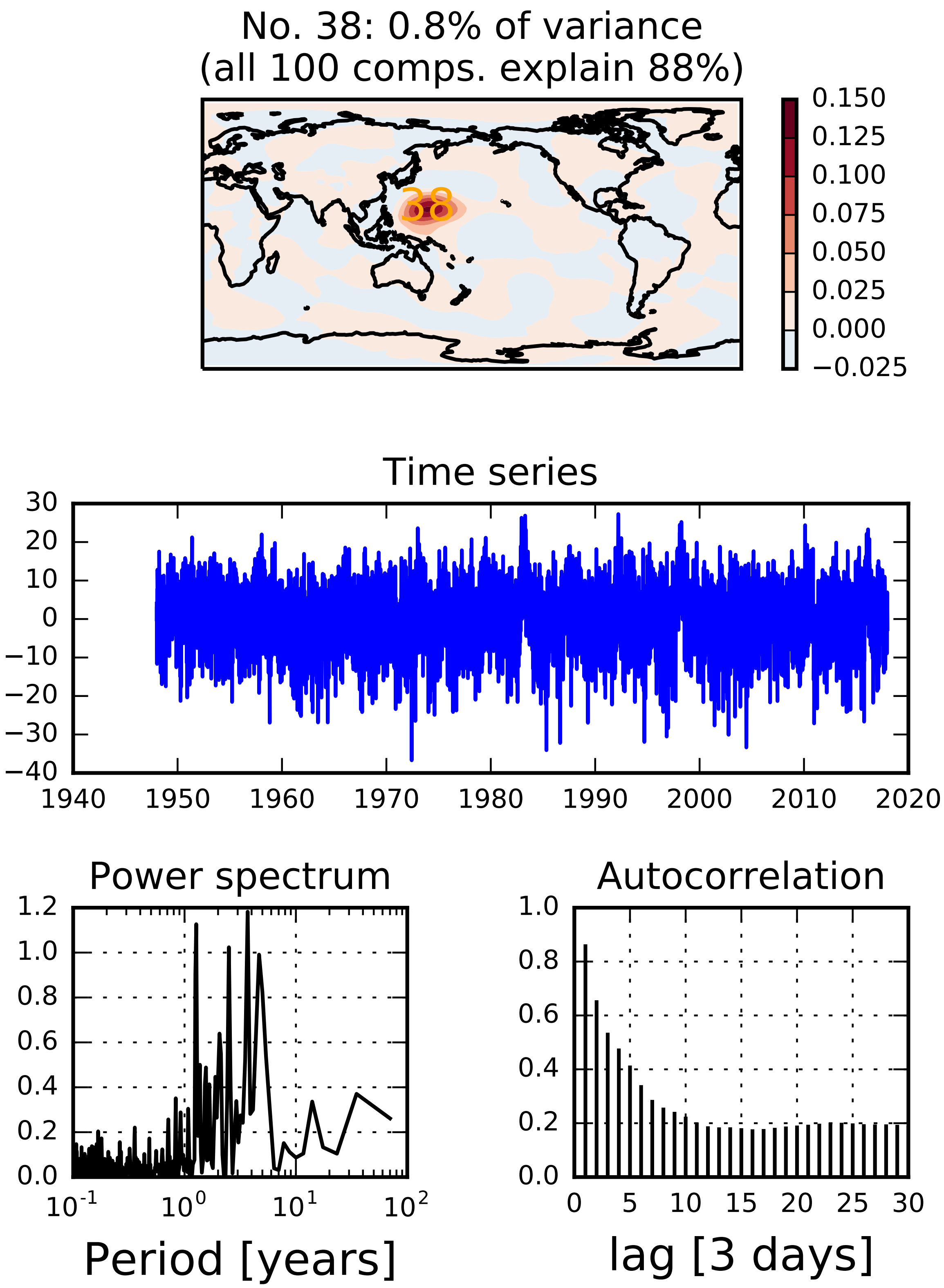
No. 38: 0.8% of variance
(all 100 comps. explain 88%)
38
0.150
0.125
0.100
0.075
0.050
0.025
0.000
−0.025
Time series
30
20
10
0
−10
−20
−30
−40
1940
1950
1960
1970
1980
1990
2000
2010
2020
Power spectrum
1.2
1.0
0.8
0.6
0.4
0.2
0.0
10^{-1}
10^{0}
10^{1}
10^{2}
Period [years]
Autocorrelation
1.0
0.8
0.6
0.4
0.2
0.0
0
5
10
15
20
25
30
lag [3 days]

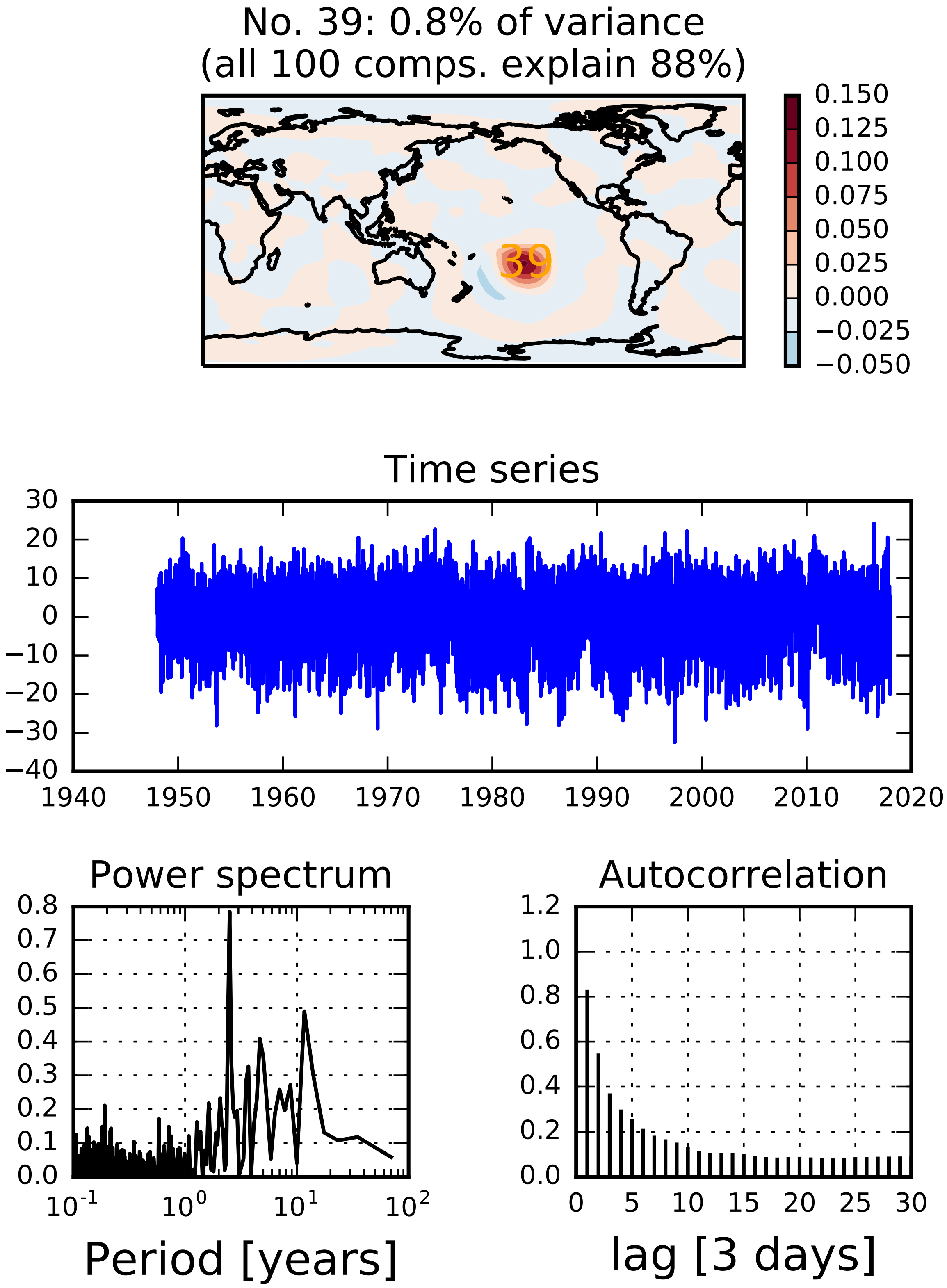
No. 39: 0.8% of variance
(all 100 comps. explain 88%)
39
0.150
0.125
0.100
0.075
0.050
0.025
0.000
−0.025
−0.050
Time series
30
20
10
0
−10
−20
−30
−40
1940
1950
1960
1970
1980
1990
2000
2010
2020
Power spectrum
0.8
0.7
0.6
0.5
0.4
0.3
0.2
0.1
0.0
10^-1
10^0
10^1
10^2
Period [years]
Autocorrelation
1.2
1.0
0.8
0.6
0.4
0.2
0.0
0
5
10
15
20
25
30
lag [3 days]

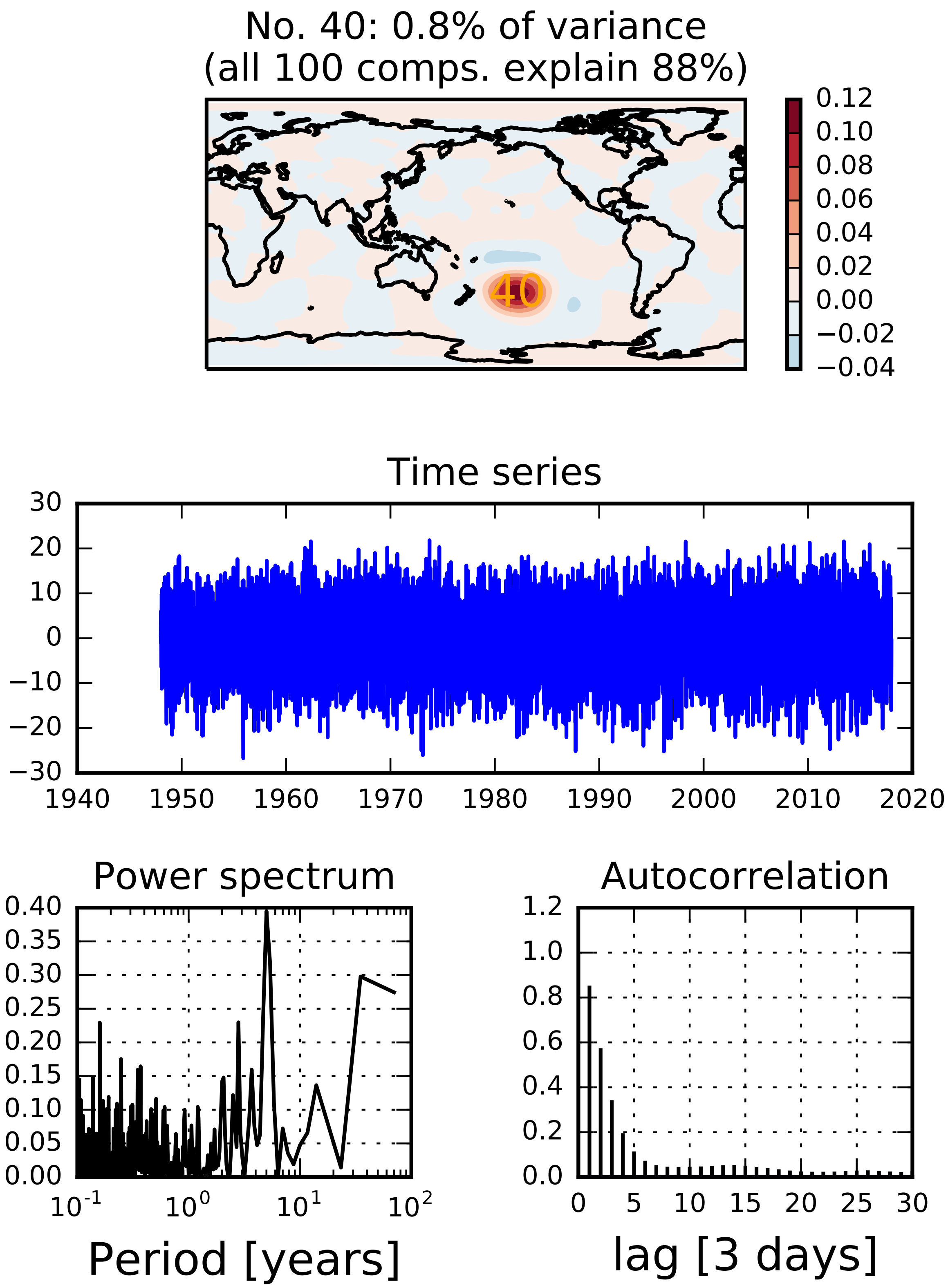
No. 40: 0.8% of variance
(all 100 comps. explain 88%)
0.12
0.10
0.08
0.06
0.04
0.02
0.00
−0.02
−0.04
40
Time series
30
20
10
0
−10
−20
−30
1940
1950
1960
1970
1980
1990
2000
2010
2020
Power spectrum
0.40
0.35
0.30
0.25
0.20
0.15
0.10
0.05
0.00
10^{-1}
10^{0}
10^{1}
10^{2}
Period [years]
Autocorrelation
1.2
1.0
0.8
0.6
0.4
0.2
0.0
0
5
10
15
20
25
30
lag [3 days]

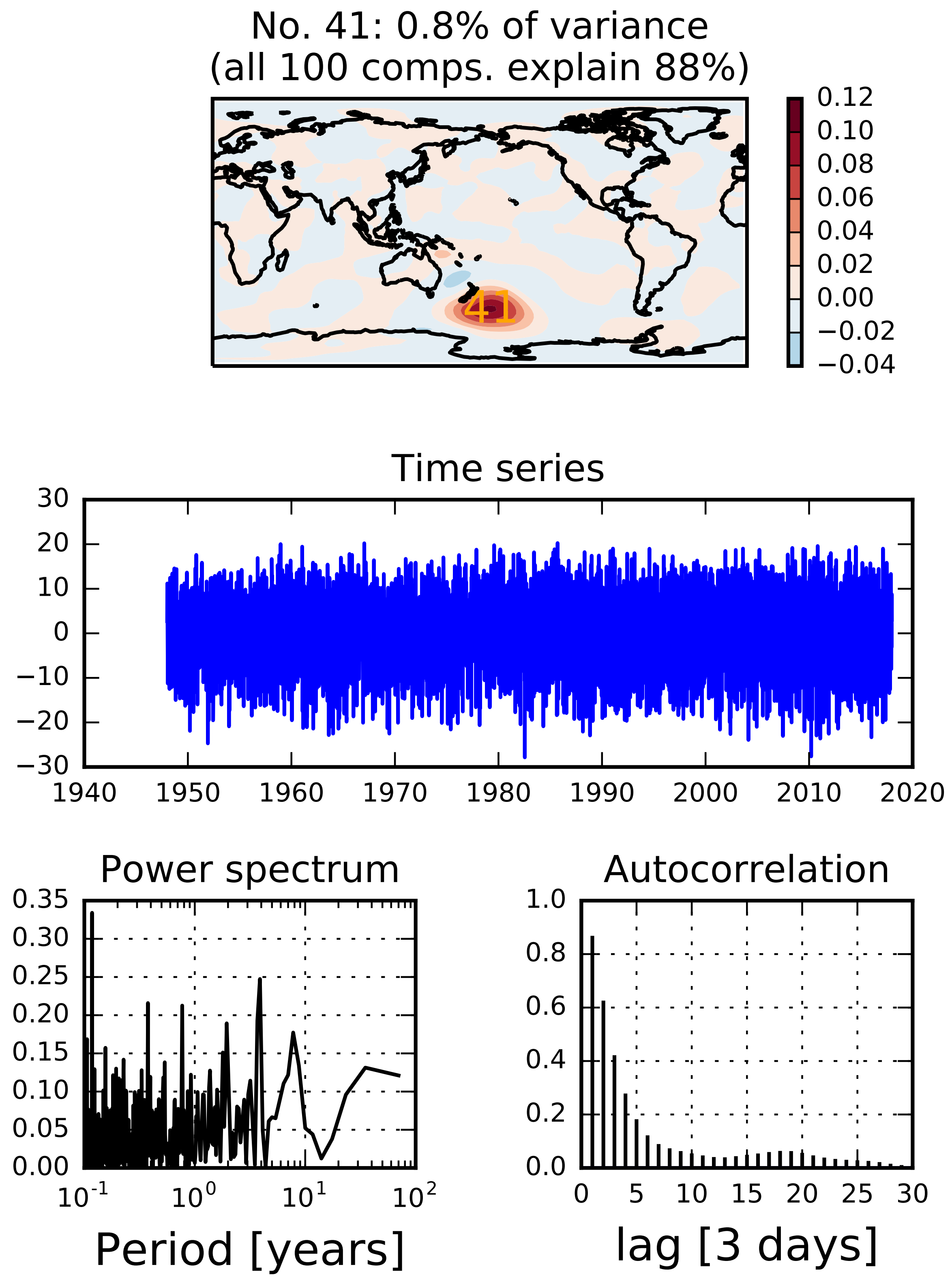
No. 41: 0.8% of variance
(all 100 comps. explain 88%)
41
0.12
0.10
0.08
0.06
0.04
0.02
0.00
−0.02
−0.04
Time series
30
20
10
0
−10
−20
−30
1940
1950
1960
1970
1980
1990
2000
2010
2020
Power spectrum
0.35
0.30
0.25
0.20
0.15
0.10
0.05
0.00
10^{-1}
10^{0}
10^{1}
10^{2}
Period [years]
Autocorrelation
1.0
0.8
0.6
0.4
0.2
0.0
0
5
10
15
20
25
30
lag [3 days]

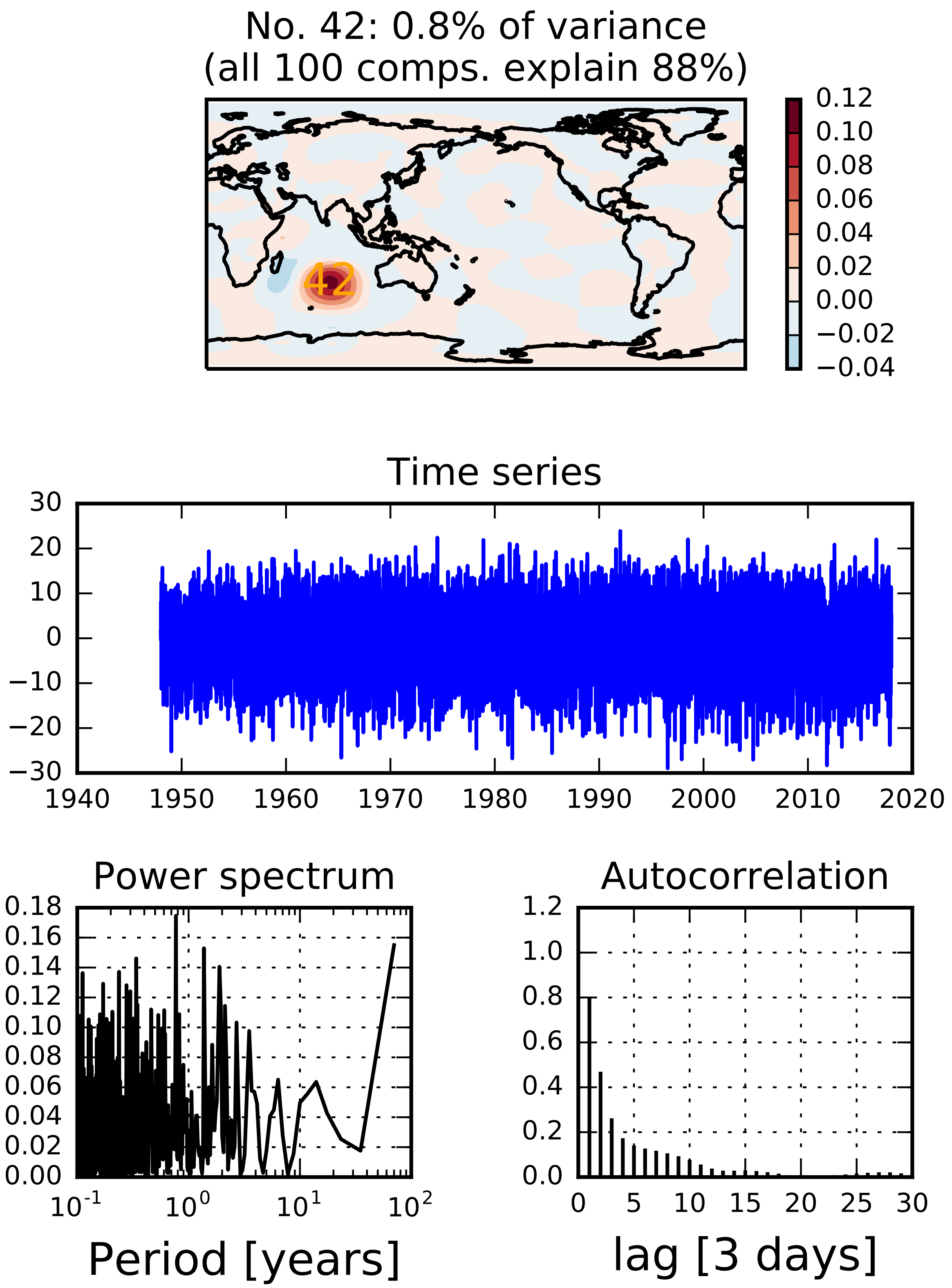

No. 42: 0.8% of variance
(all 100 comps. explain 88%)
42
0.12
0.10
0.08
0.06
0.04
0.02
0.00
−0.02
−0.04
Time series
30
20
10
0
−10
−20
−30
1940
1950
1960
1970
1980
1990
2000
2010
2020
Power spectrum
0.18
0.16
0.14
0.12
0.10
0.08
0.06
0.04
0.02
0.00
10^{-1}
10^{0}
10^{1}
10^{2}
Period [years]
Autocorrelation
1.2
1.0
0.8
0.6
0.4
0.2
0.0
0
5
10
15
20
25
30
lag [3 days]

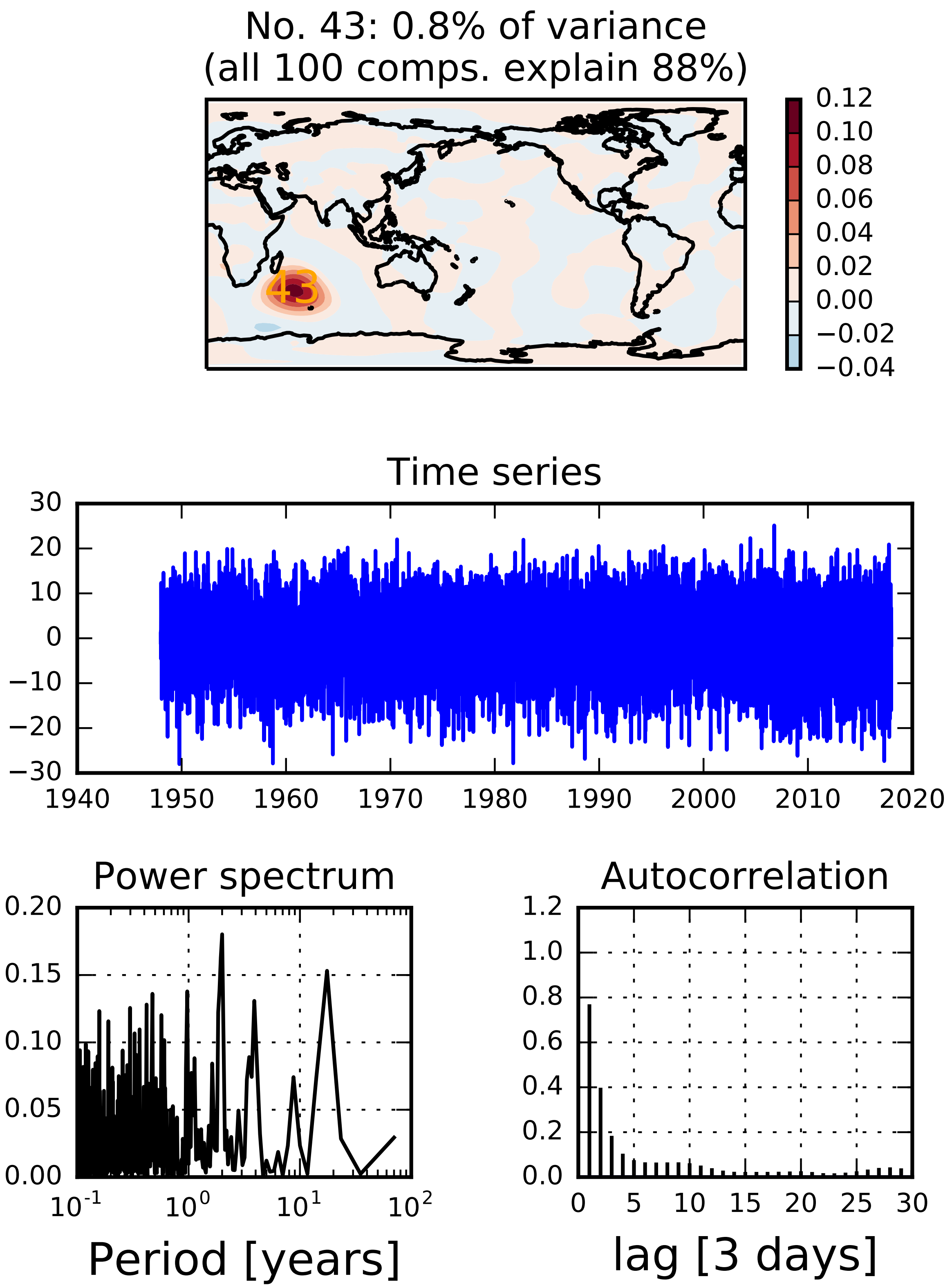
No. 43: 0.8% of variance
(all 100 comps. explain 88%)
43
0.12
0.10
0.08
0.06
0.04
0.02
0.00
−0.02
−0.04
Time series
30
20
10
0
−10
−20
−30
1940
1950
1960
1970
1980
1990
2000
2010
2020
Power spectrum
0.20
0.15
0.10
0.05
0.00
10^-1
10^0
10^1
10^2
Period [years]
Autocorrelation
1.2
1.0
0.8
0.6
0.4
0.2
0.0
0
5
10
15
20
25
30
lag [3 days]

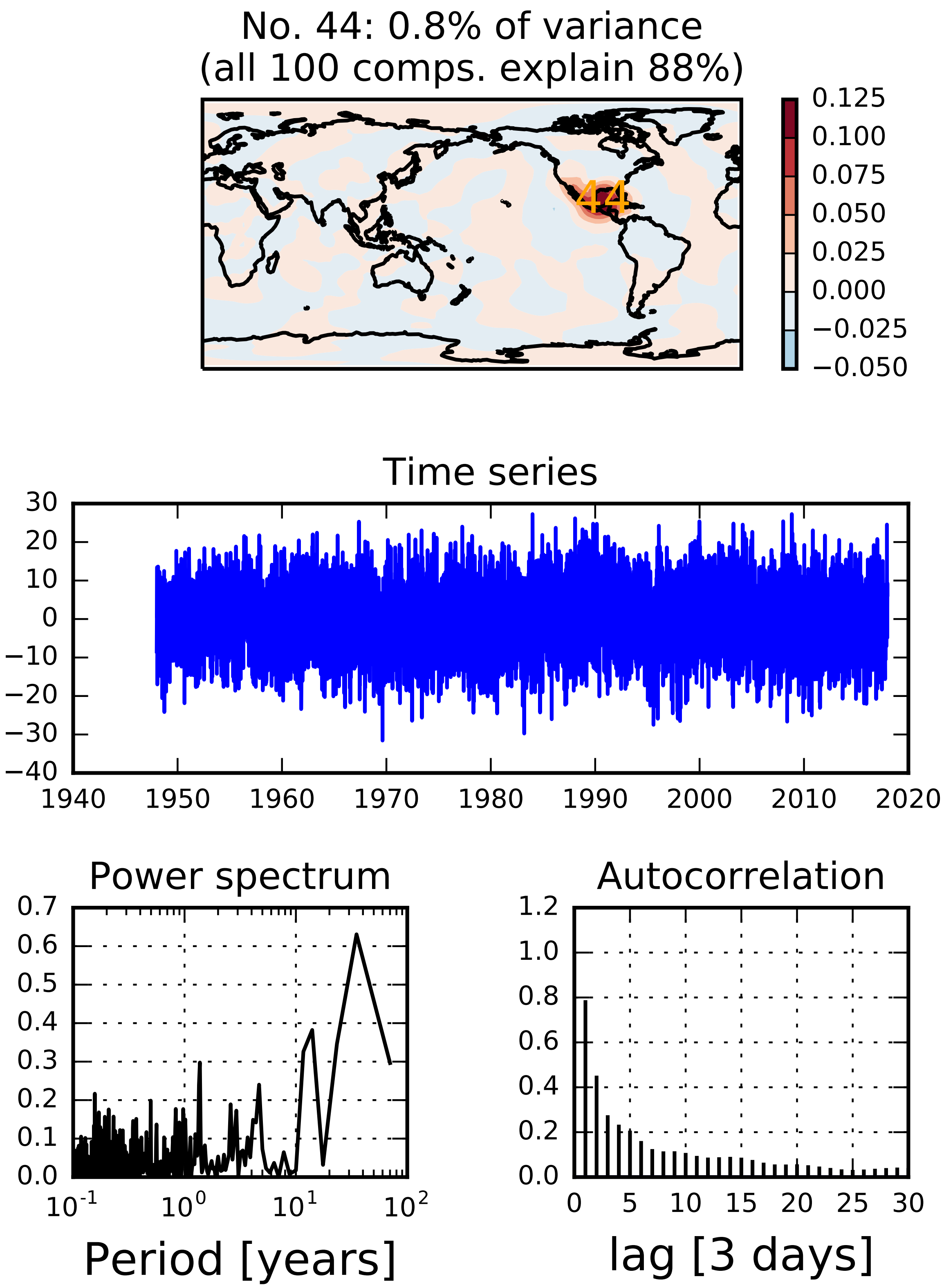
No. 44: 0.8% of variance
(all 100 comps. explain 88%)
44
0.125
0.100
0.075
0.050
0.025
0.000
−0.025
−0.050
Time series
30
20
10
0
−10
−20
−30
−40
1940
1950
1960
1970
1980
1990
2000
2010
2020
Power spectrum
0.7
0.6
0.5
0.4
0.3
0.2
0.1
0.0
10^{-1}
10^{0}
10^{1}
10^{2}
Period [years]
Autocorrelation
1.2
1.0
0.8
0.6
0.4
0.2
0.0
0
5
10
15
20
25
30
lag [3 days]

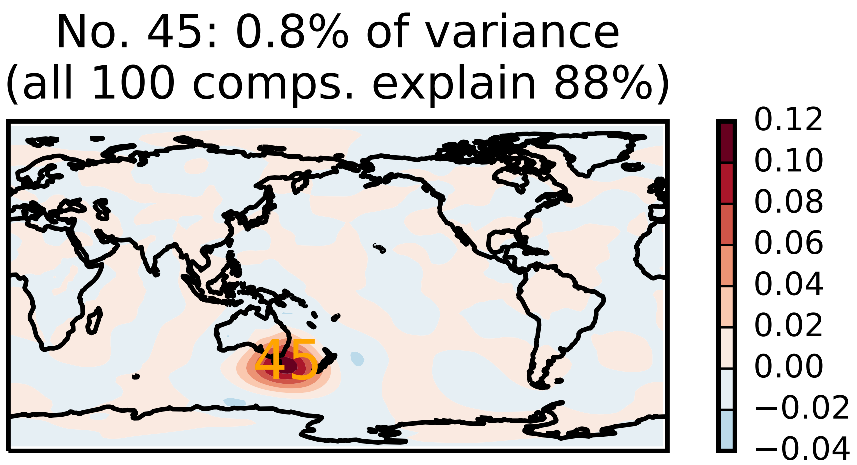
No. 45: 0.8% of variance
(all 100 comps. explain 88%)
45
0.12
0.10
0.08
0.06
0.04
0.02
0.00
−0.02
−0.04

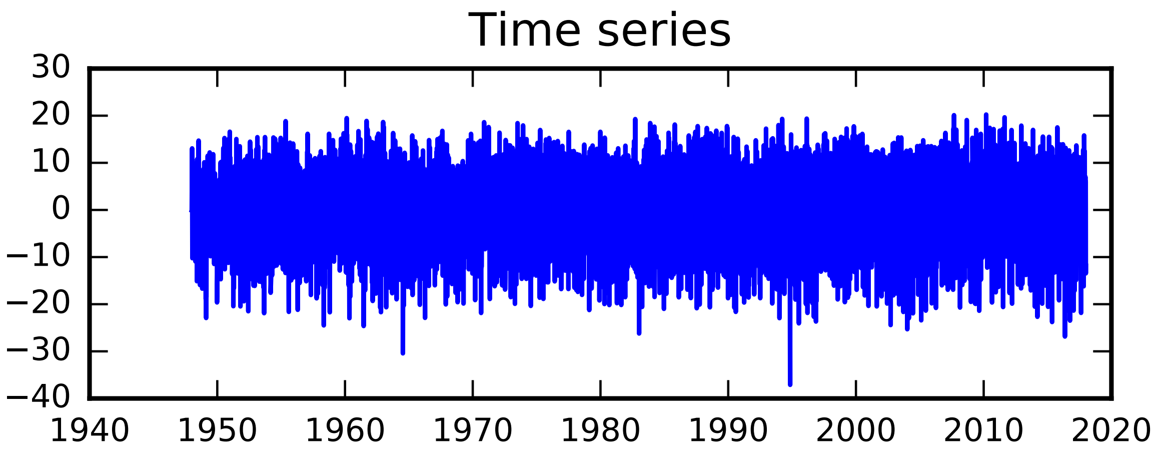
Time series
30
20
10
0
−10
−20
−30
−40
1940
1950
1960
1970
1980
1990
2000
2010
2020

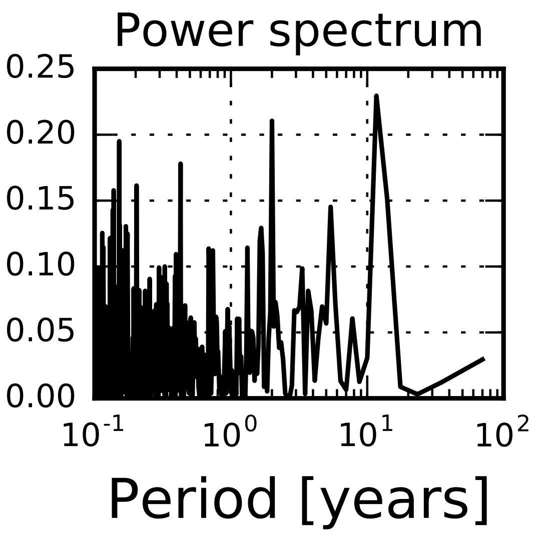
Power spectrum
0.25
0.20
0.15
0.10
0.05
0.00
10^-1
10^0
10^1
10^2
Period [years]

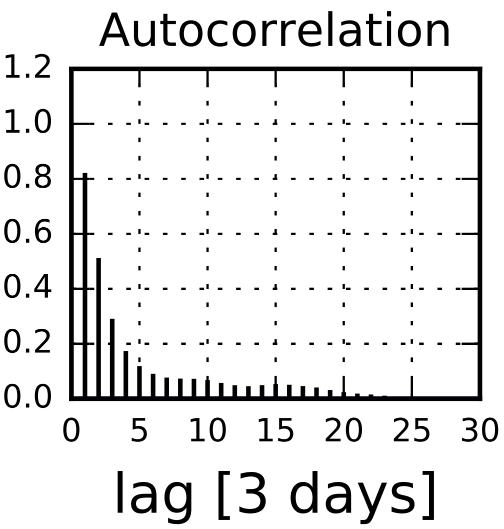
Autocorrelation
1.2
1.0
0.8
0.6
0.4
0.2
0.0
0
5
10
15
20
25
30
lag [3 days]

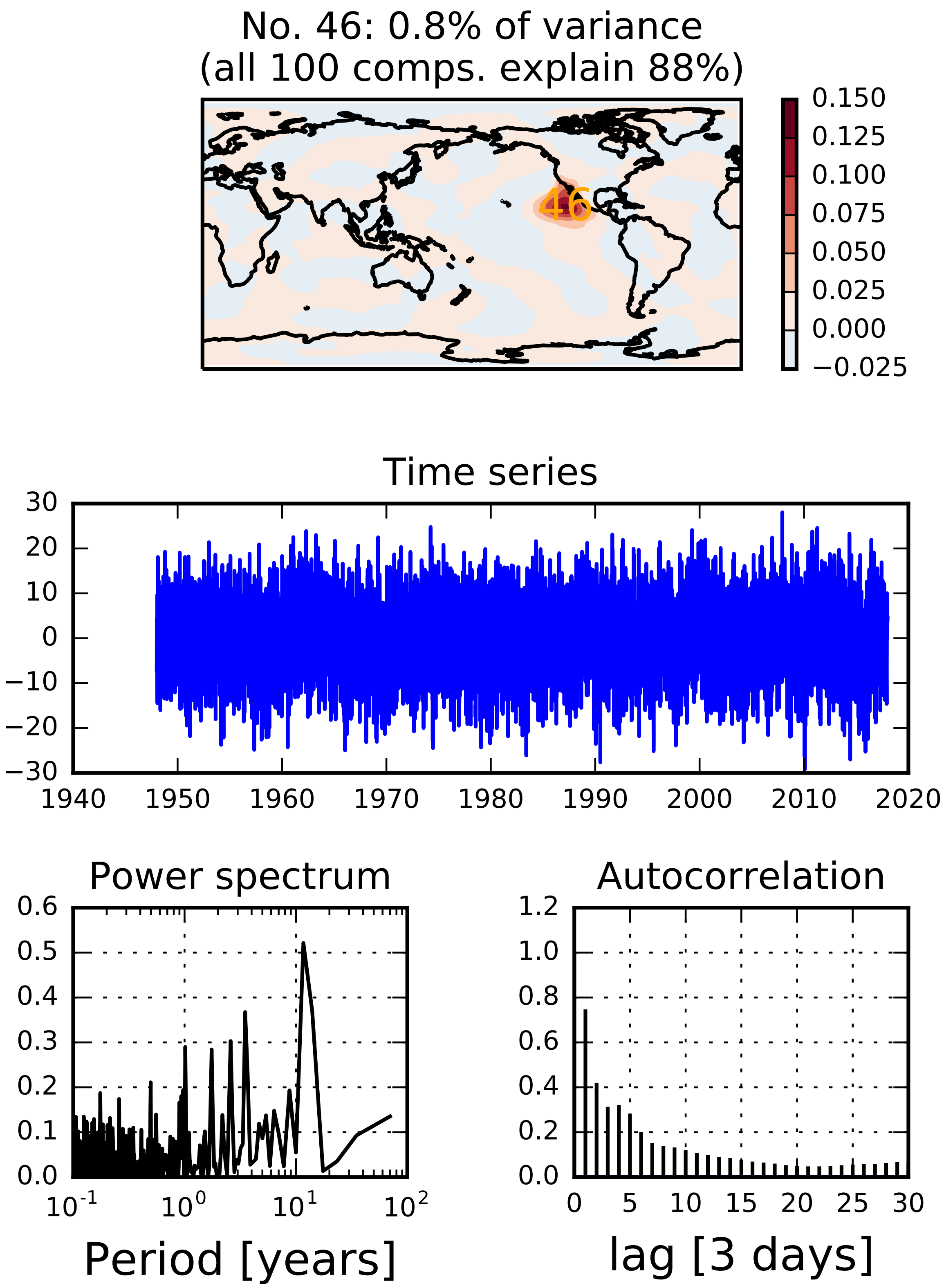
No. 46: 0.8% of variance
(all 100 comps. explain 88%)
46
0.150
0.125
0.100
0.075
0.050
0.025
0.000
−0.025
Time series
30
20
10
0
−10
−20
−30
1940
1950
1960
1970
1980
1990
2000
2010
2020
Power spectrum
0.6
0.5
0.4
0.3
0.2
0.1
0.0
10^{-1}
10^{0}
10^{1}
10^{2}
Period [years]
Autocorrelation
1.2
1.0
0.8
0.6
0.4
0.2
0.0
0
5
10
15
20
25
30
lag [3 days]

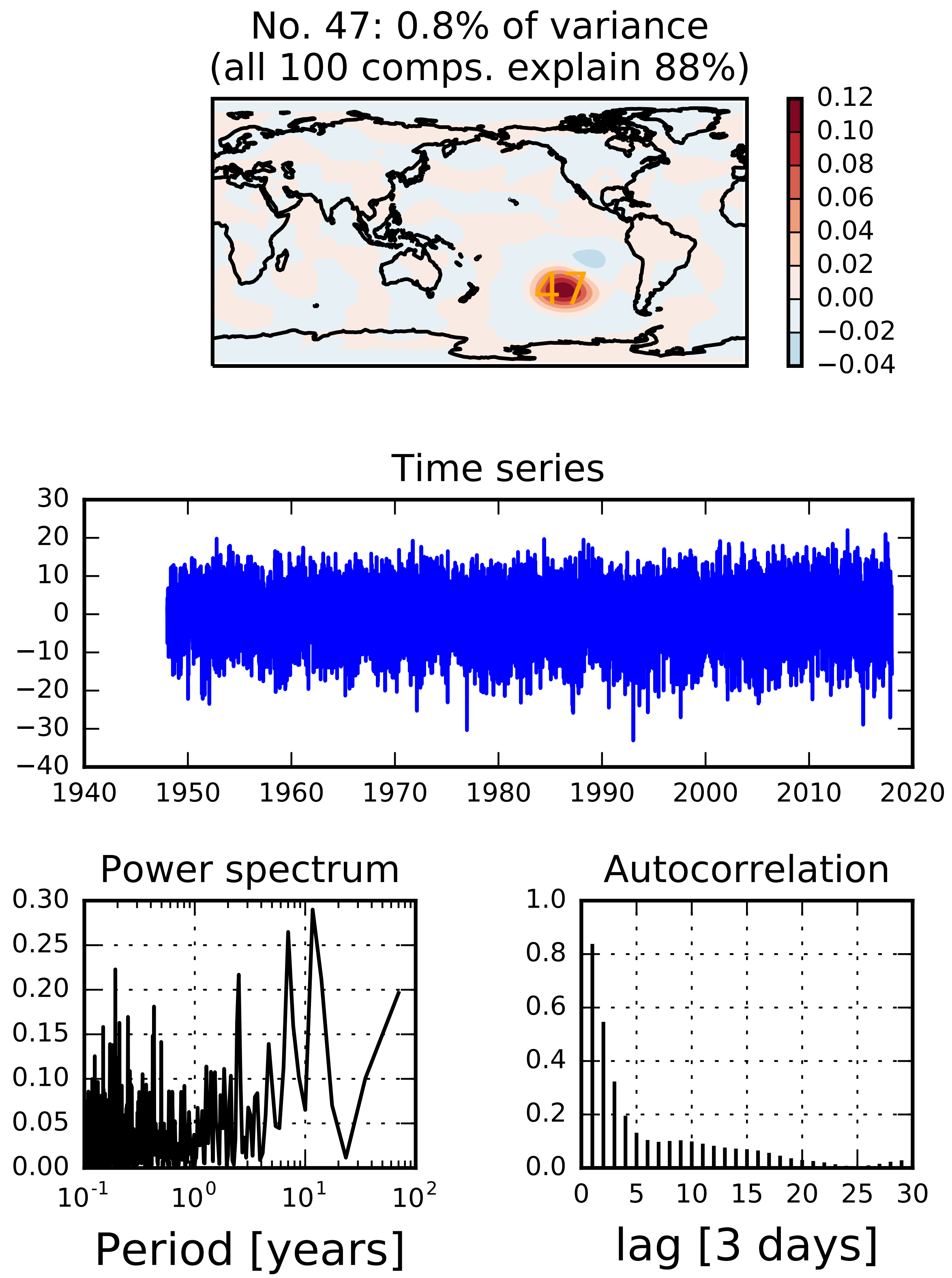
No. 47: 0.8% of variance
(all 100 comps. explain 88%)
47
0.12
0.10
0.08
0.06
0.04
0.02
0.00
−0.02
−0.04
Time series
30
20
10
0
−10
−20
−30
−40
1940
1950
1960
1970
1980
1990
2000
2010
2020
Power spectrum
0.30
0.25
0.20
0.15
0.10
0.05
0.00
10^{-1}
10^{0}
10^{1}
10^{2}
Period [years]
Autocorrelation
1.0
0.8
0.6
0.4
0.2
0.0
0
5
10
15
20
25
30
lag [3 days]

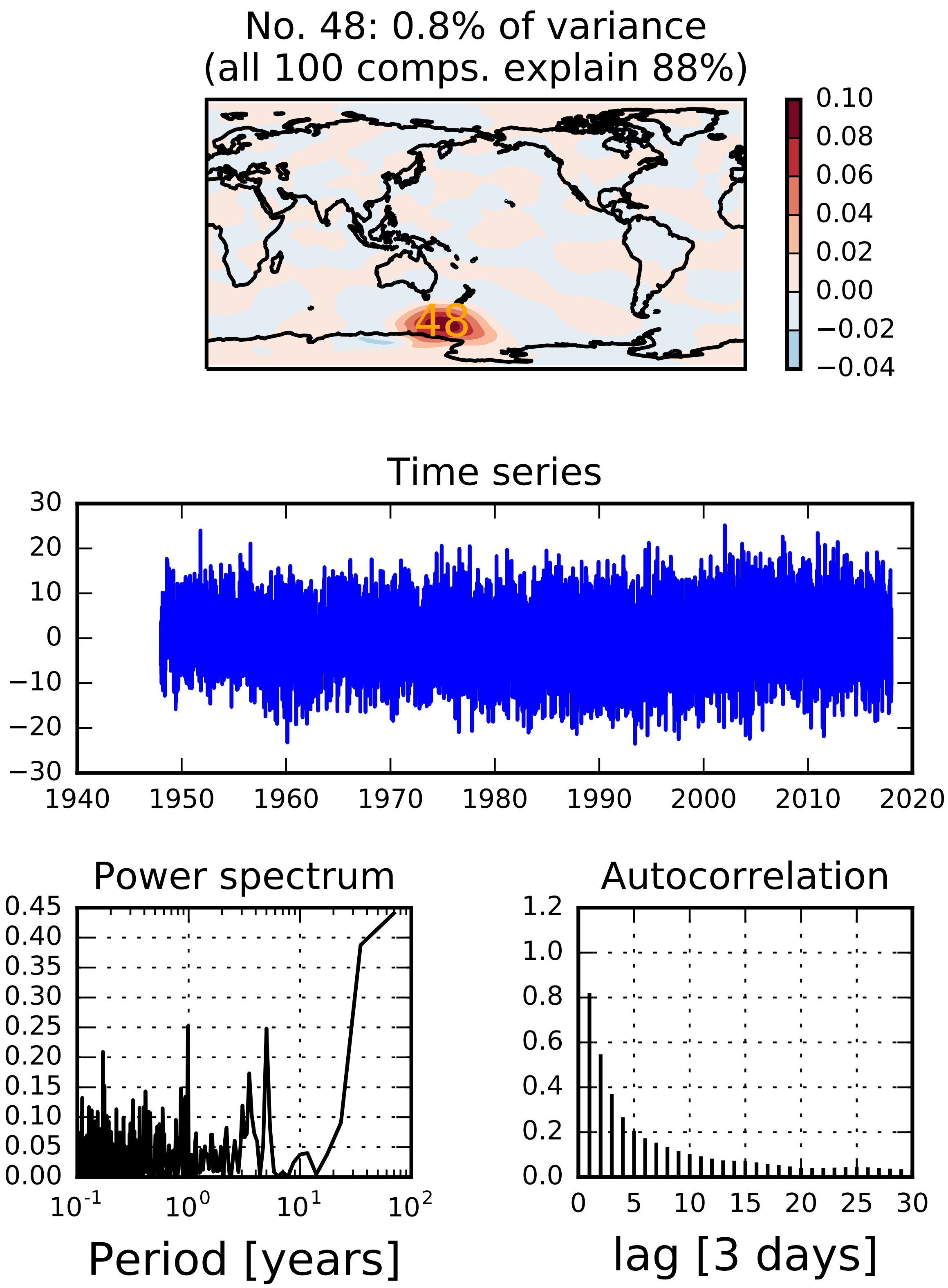
No. 48: 0.8% of variance
(all 100 comps. explain 88%)
48
0.10
0.08
0.06
0.04
0.02
0.00
−0.02
−0.04
Time series
30
20
10
0
−10
−20
−30
1940
1950
1960
1970
1980
1990
2000
2010
2020
Power spectrum
0.45
0.40
0.35
0.30
0.25
0.20
0.15
0.10
0.05
0.00
10^{-1}
10^{0}
10^{1}
10^{2}
Period [years]
Autocorrelation
1.2
1.0
0.8
0.6
0.4
0.2
0.0
0
5
10
15
20
25
30
lag [3 days]

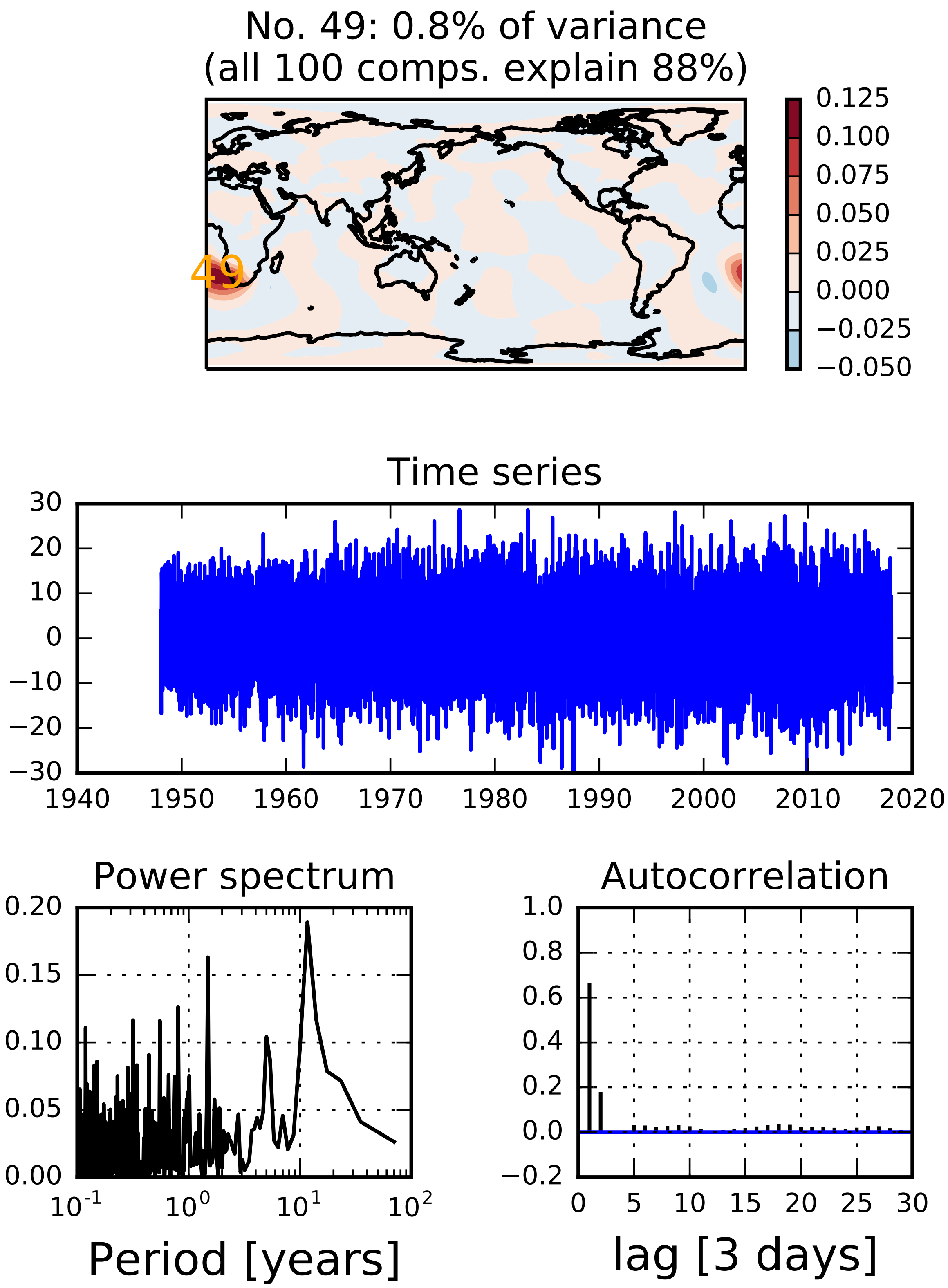
No. 49: 0.8% of variance
(all 100 comps. explain 88%)
49
0.125
0.100
0.075
0.050
0.025
0.000
−0.025
−0.050
Time series
30
20
10
0
−10
−20
−30
1940
1950
1960
1970
1980
1990
2000
2010
2020
Power spectrum
0.20
0.15
0.10
0.05
0.00
10^-1
10^0
10^1
10^2
Period [years]
Autocorrelation
1.0
0.8
0.6
0.4
0.2
0.0
−0.2
0
5
10
15
20
25
30
lag [3 days]

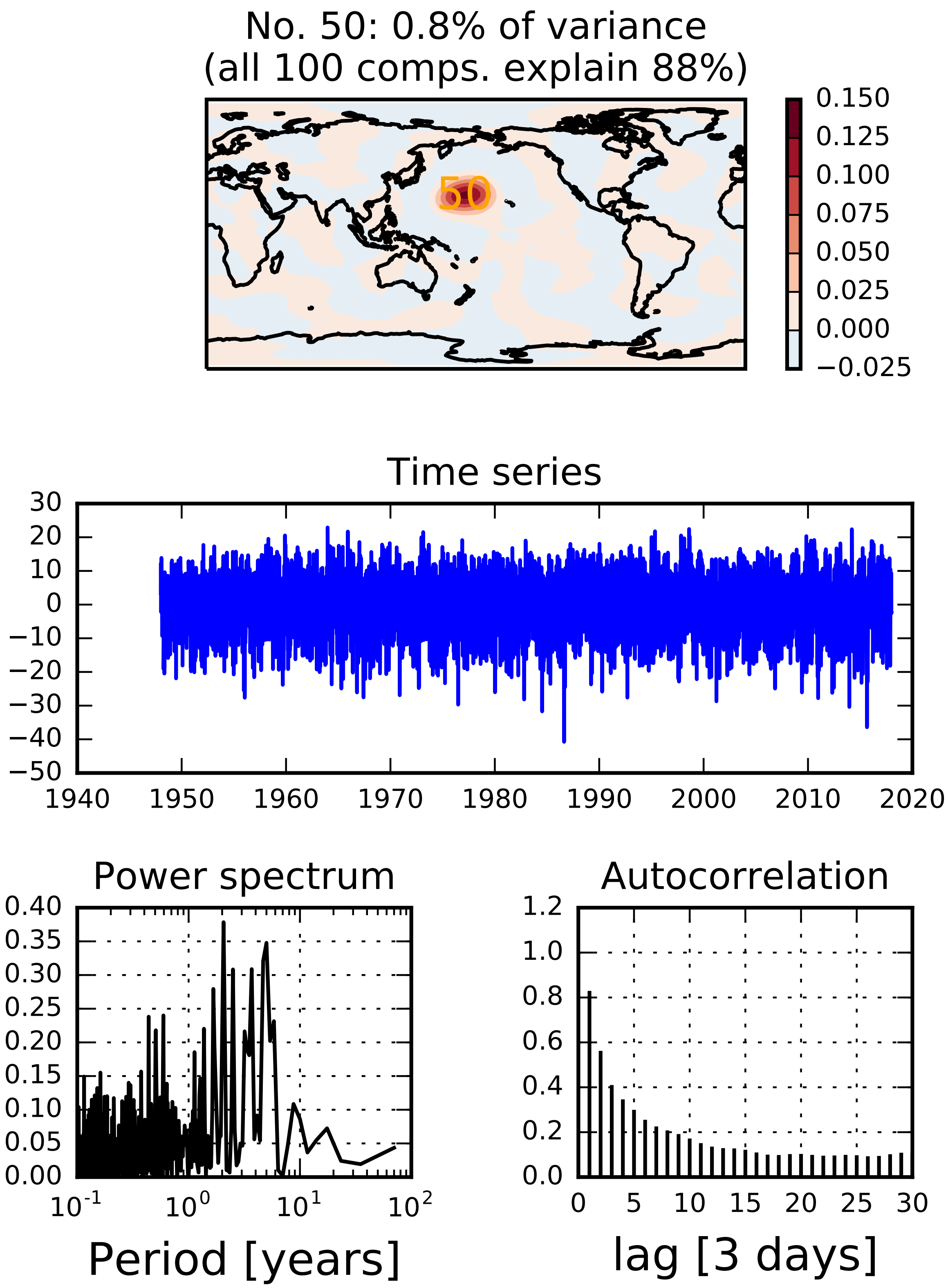
No. 50: 0.8% of variance
(all 100 comps. explain 88%)
50
0.150
0.125
0.100
0.075
0.050
0.025
0.000
−0.025
Time series
30
20
10
0
−10
−20
−30
−40
−50
1940
1950
1960
1970
1980
1990
2000
2010
2020
Power spectrum
0.40
0.35
0.30
0.25
0.20
0.15
0.10
0.05
0.00
10^{-1}
10^{0}
10^{1}
10^{2}
Period [years]
Autocorrelation
1.2
1.0
0.8
0.6
0.4
0.2
0.0
0
5
10
15
20
25
30
lag [3 days]

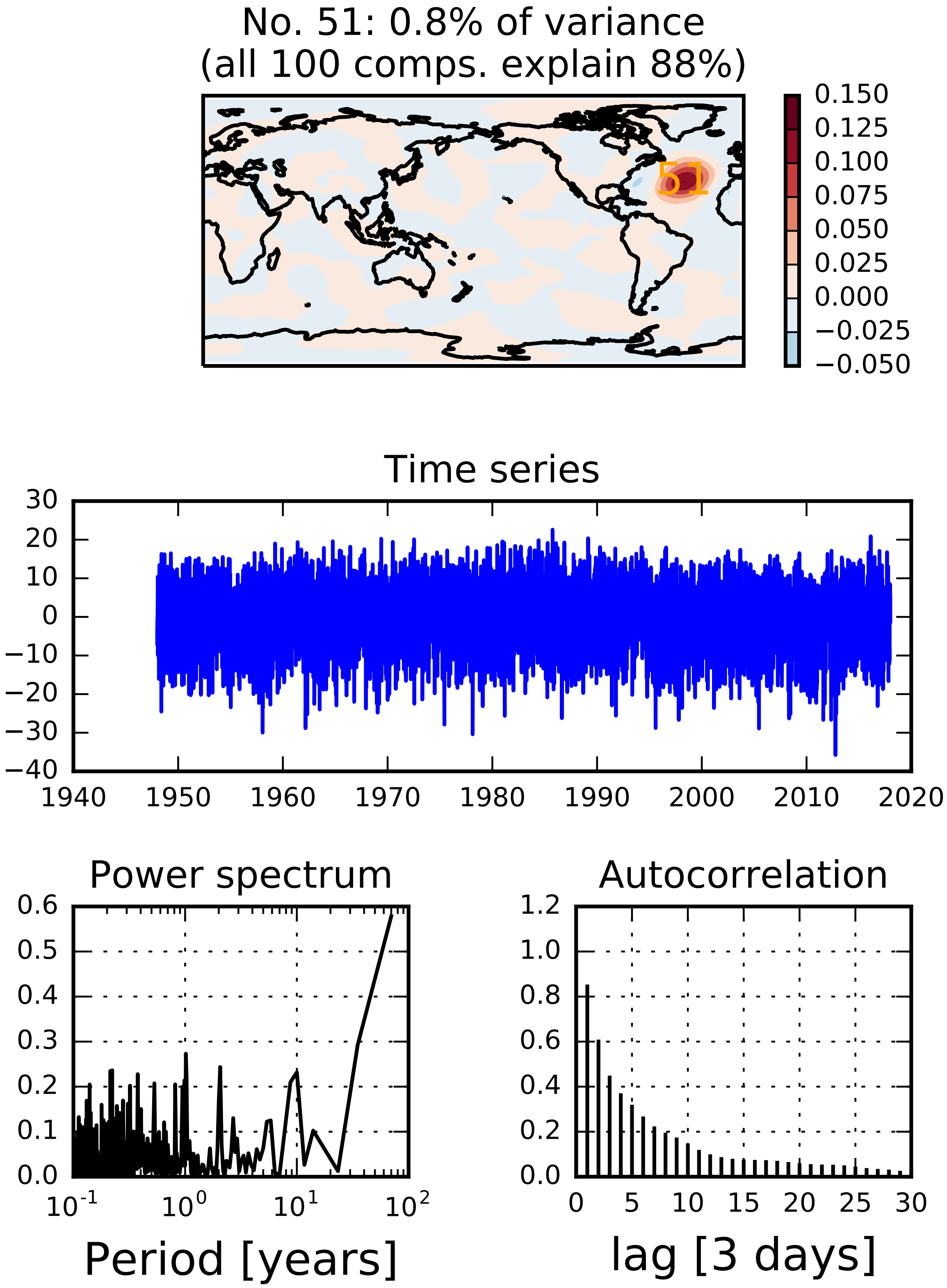
No. 51: 0.8% of variance
(all 100 comps. explain 88%)
51
0.150
0.125
0.100
0.075
0.050
0.025
0.000
−0.025
−0.050
Time series
30
20
10
0
−10
−20
−30
−40
1940
1950
1960
1970
1980
1990
2000
2010
2020
Power spectrum
0.6
0.5
0.4
0.3
0.2
0.1
0.0
10^-1
10^0
10^1
10^2
Period [years]
Autocorrelation
1.2
1.0
0.8
0.6
0.4
0.2
0.0
0
5
10
15
20
25
30
lag [3 days]

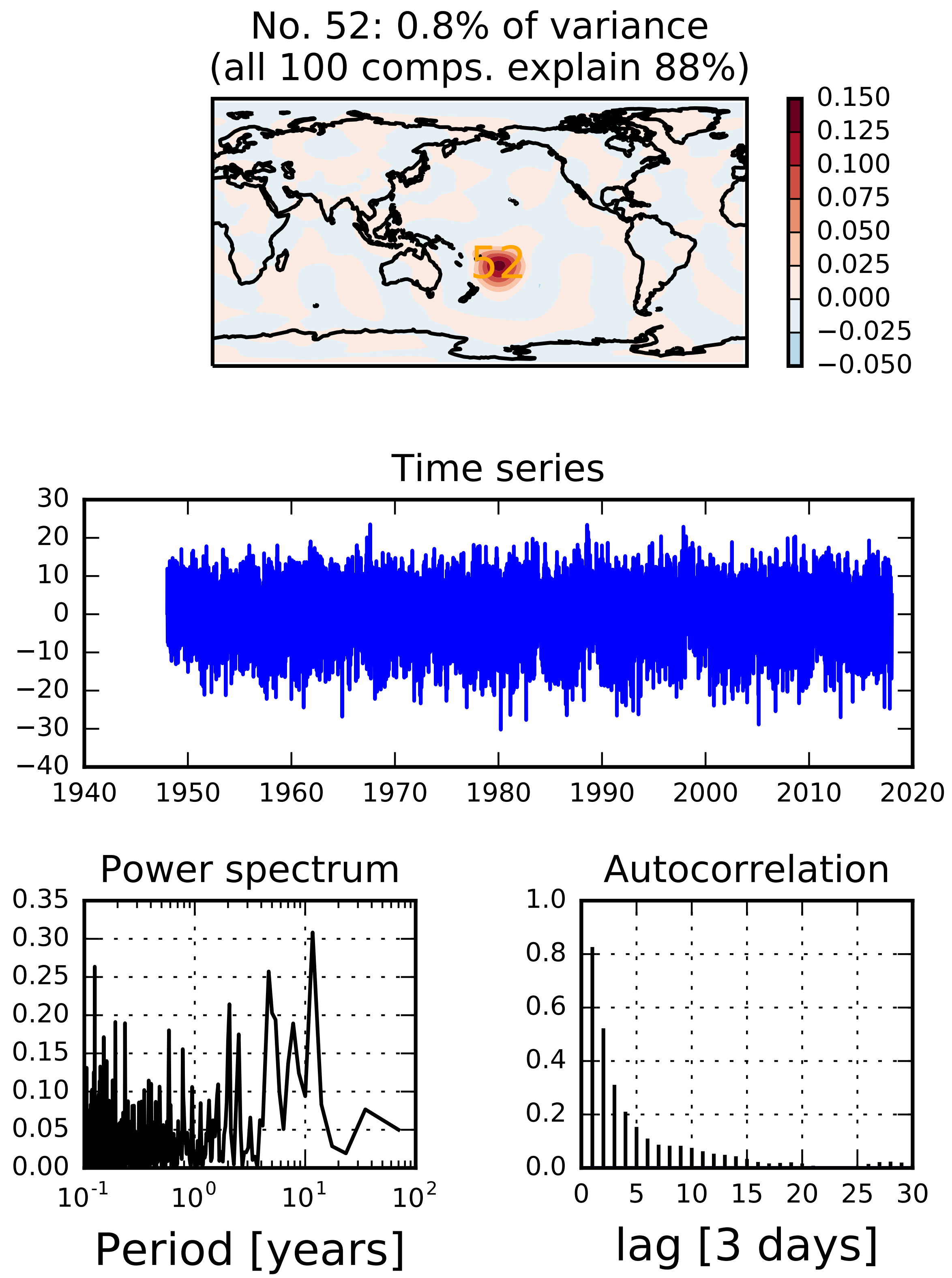
No. 52: 0.8% of variance
(all 100 comps. explain 88%)
52
0.150
0.125
0.100
0.075
0.050
0.025
0.000
−0.025
−0.050
Time series
30
20
10
0
−10
−20
−30
−40
1940
1950
1960
1970
1980
1990
2000
2010
2020
Power spectrum
0.35
0.30
0.25
0.20
0.15
0.10
0.05
0.00
10^-1
10^0
10^1
10^2
Period [years]
Autocorrelation
1.0
0.8
0.6
0.4
0.2
0.0
0
5
10
15
20
25
30
lag [3 days]

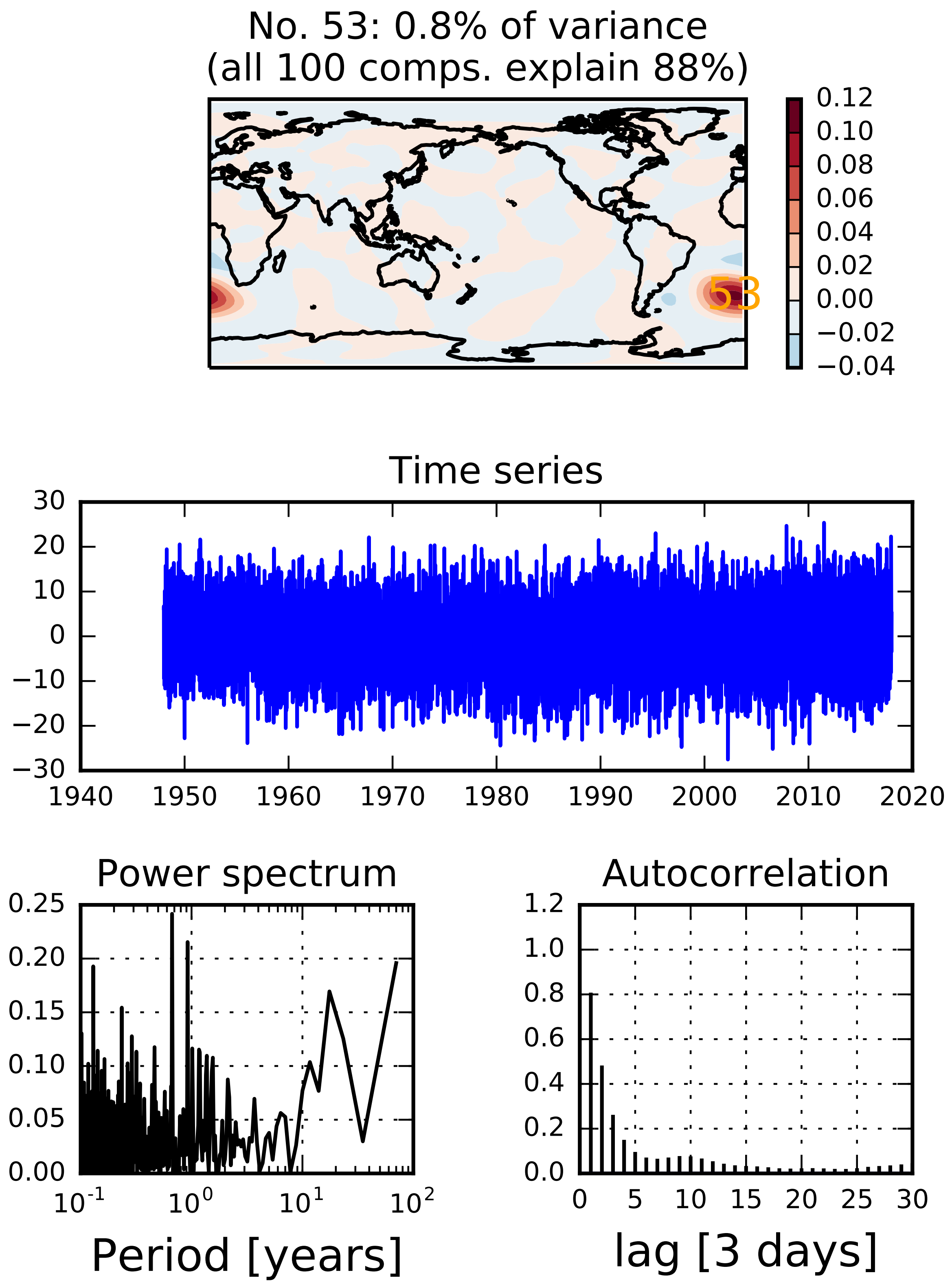
No. 53: 0.8% of variance
(all 100 comps. explain 88%)
53
0.12
0.10
0.08
0.06
0.04
0.02
0.00
−0.02
−0.04
Time series
30
20
10
0
−10
−20
−30
1940
1950
1960
1970
1980
1990
2000
2010
2020
Power spectrum
0.25
0.20
0.15
0.10
0.05
0.00
10^{-1}
10^{0}
10^{1}
10^{2}
Period [years]
Autocorrelation
1.2
1.0
0.8
0.6
0.4
0.2
0.0
0
5
10
15
20
25
30
lag [3 days]

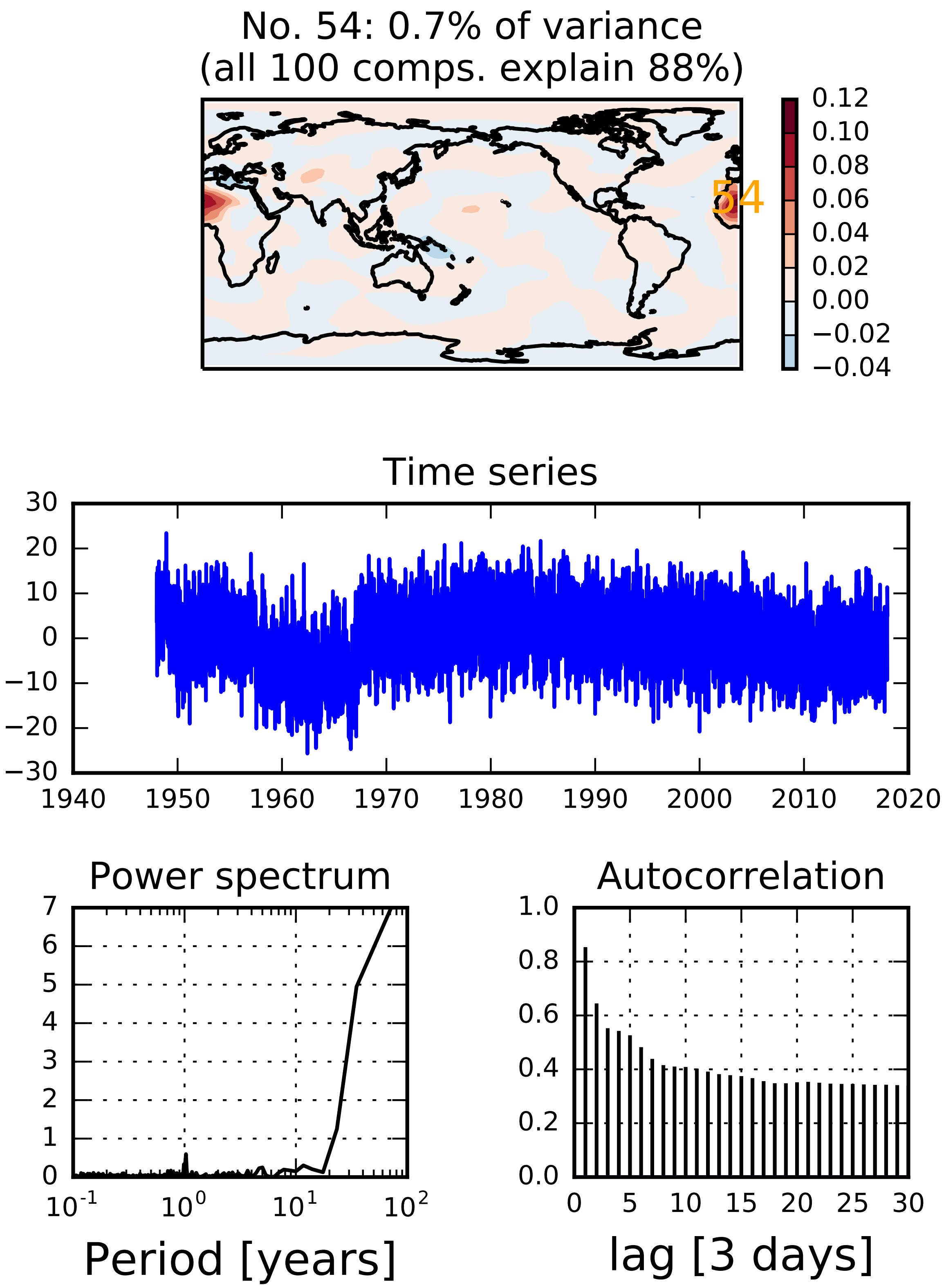
No. 54: 0.7% of variance
(all 100 comps. explain 88%)
54
0.12
0.10
0.08
0.06
0.04
0.02
0.00
−0.02
−0.04
Time series
30
20
10
0
−10
−20
−30
1940
1950
1960
1970
1980
1990
2000
2010
2020
Power spectrum
7
6
5
4
3
2
1
0
10^{-1}
10^{0}
10^{1}
10^{2}
Period [years]
Autocorrelation
1.0
0.8
0.6
0.4
0.2
0.0
0
5
10
15
20
25
30
lag [3 days]

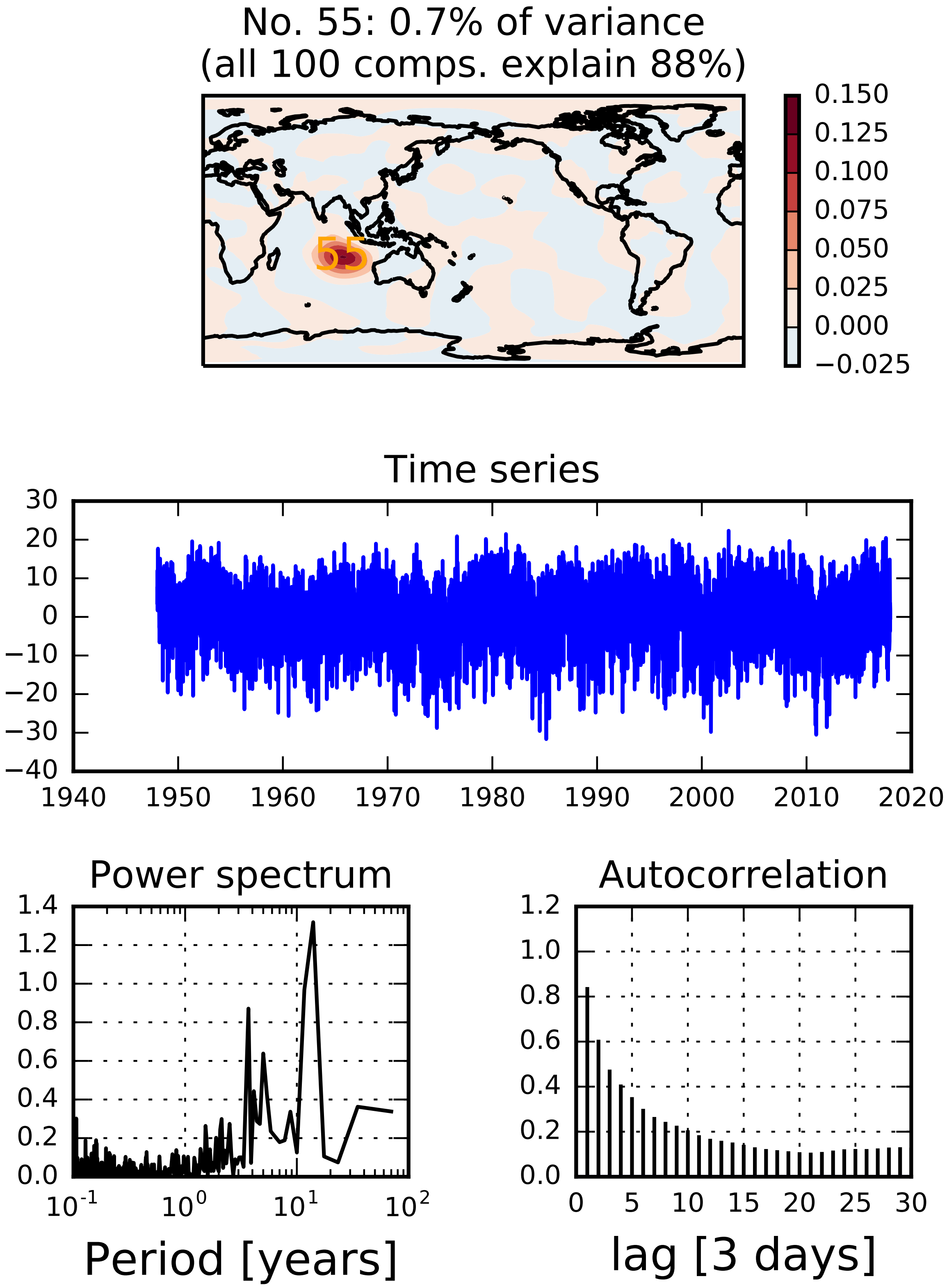
No. 55: 0.7% of variance
(all 100 comps. explain 88%)
55
0.150
0.125
0.100
0.075
0.050
0.025
0.000
−0.025
Time series
30
20
10
0
−10
−20
−30
−40
1940
1950
1960
1970
1980
1990
2000
2010
2020
Power spectrum
1.4
1.2
1.0
0.8
0.6
0.4
0.2
0.0
10^{-1}
10^{0}
10^{1}
10^{2}
Period [years]
Autocorrelation
1.2
1.0
0.8
0.6
0.4
0.2
0.0
0
5
10
15
20
25
30
lag [3 days]

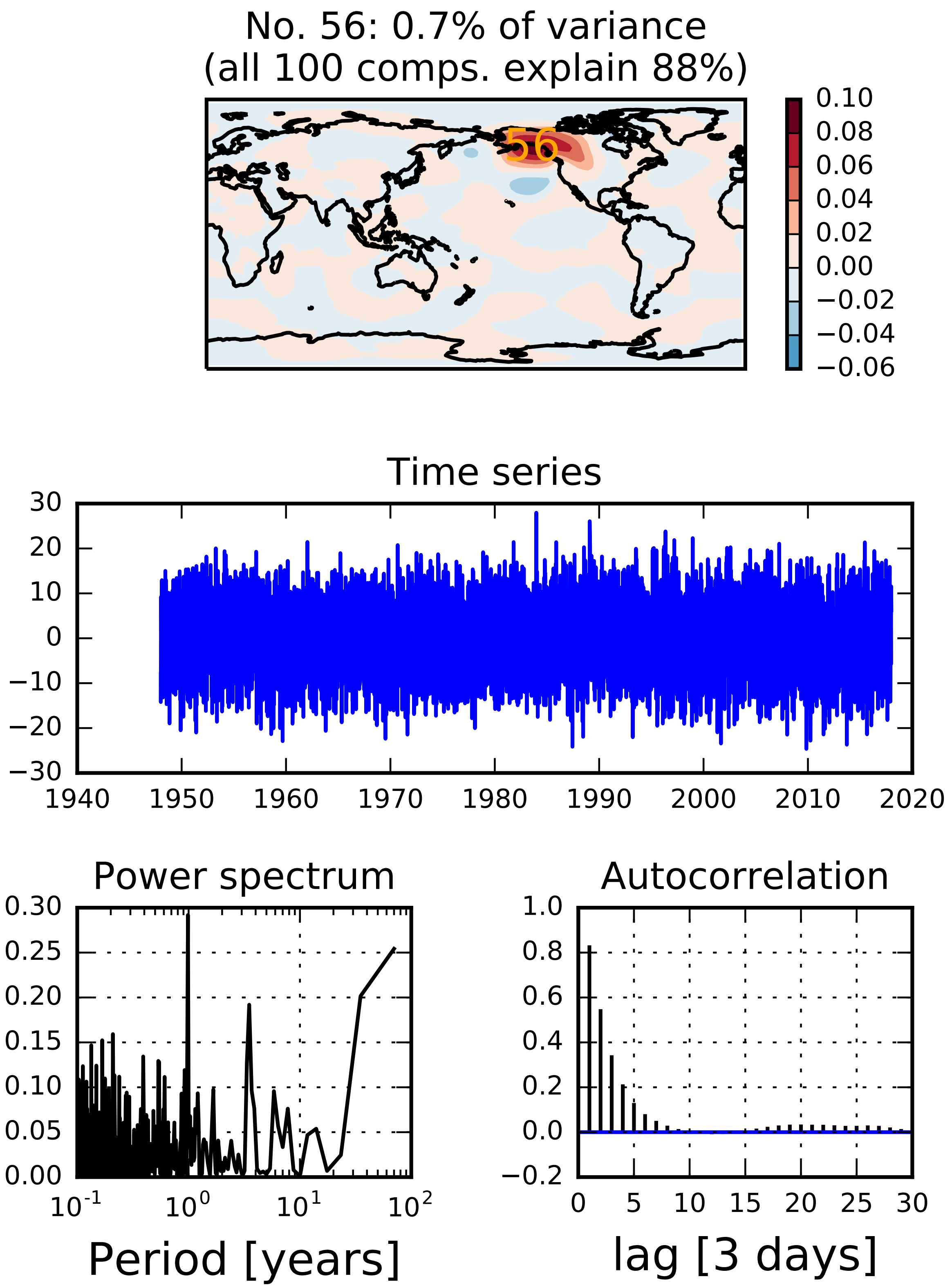
No. 56: 0.7% of variance
(all 100 comps. explain 88%)
56
0.10
0.08
0.06
0.04
0.02
0.00
−0.02
−0.04
−0.06
Time series
30
20
10
0
−10
−20
−30
1940
1950
1960
1970
1980
1990
2000
2010
2020
Power spectrum
0.30
0.25
0.20
0.15
0.10
0.05
0.00
10^{-1}
10^{0}
10^{1}
10^{2}
Period [years]
Autocorrelation
1.0
0.8
0.6
0.4
0.2
0.0
−0.2
0
5
10
15
20
25
30
lag [3 days]

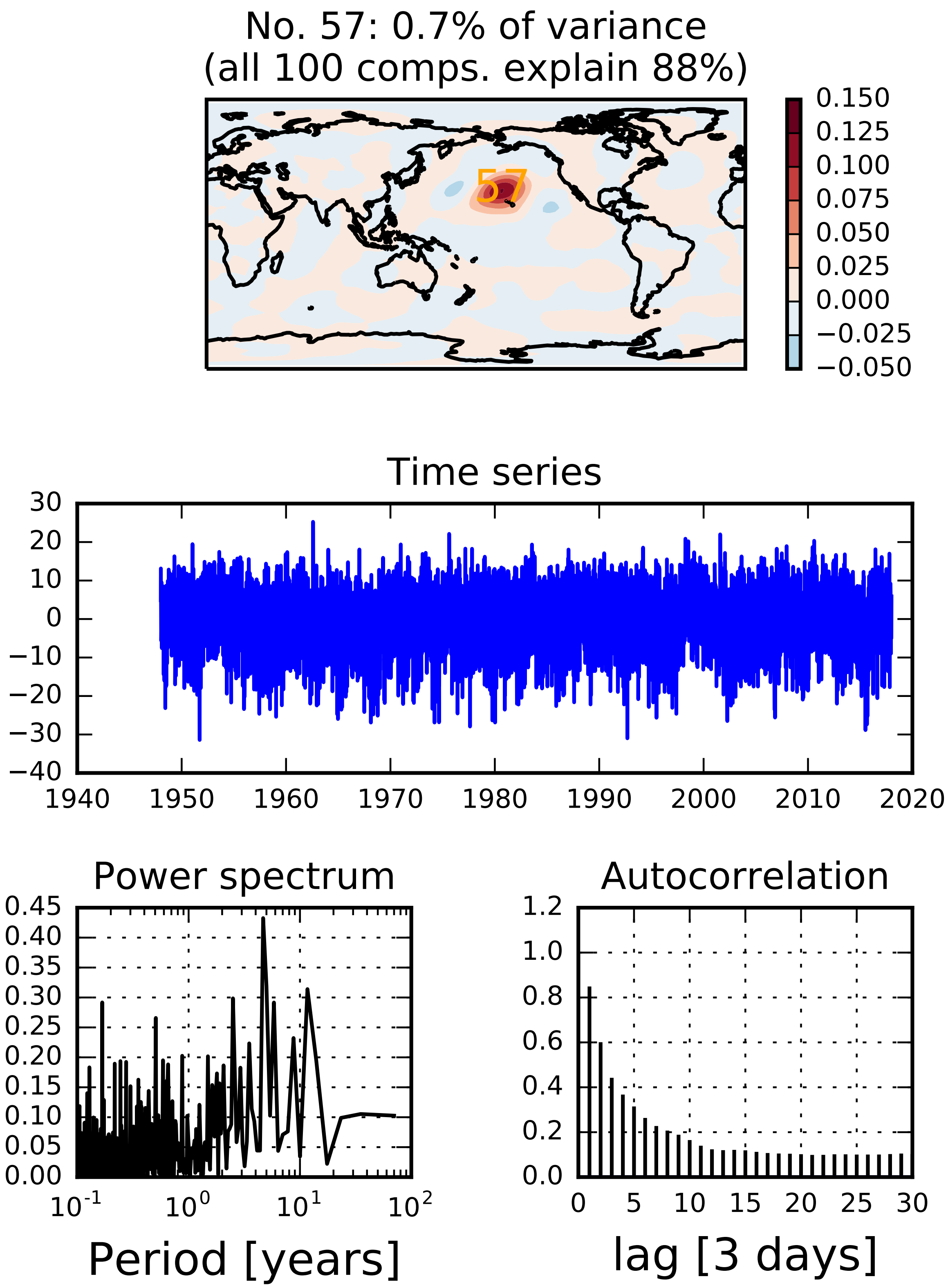
No. 57: 0.7% of variance
(all 100 comps. explain 88%)
57
0.150
0.125
0.100
0.075
0.050
0.025
0.000
−0.025
−0.050
Time series
30
20
10
0
−10
−20
−30
−40
1940
1950
1960
1970
1980
1990
2000
2010
2020
Power spectrum
0.45
0.40
0.35
0.30
0.25
0.20
0.15
0.10
0.05
0.00
10^{-1}
10^{0}
10^{1}
10^{2}
Period [years]
Autocorrelation
1.2
1.0
0.8
0.6
0.4
0.2
0.0
0
5
10
15
20
25
30
lag [3 days]

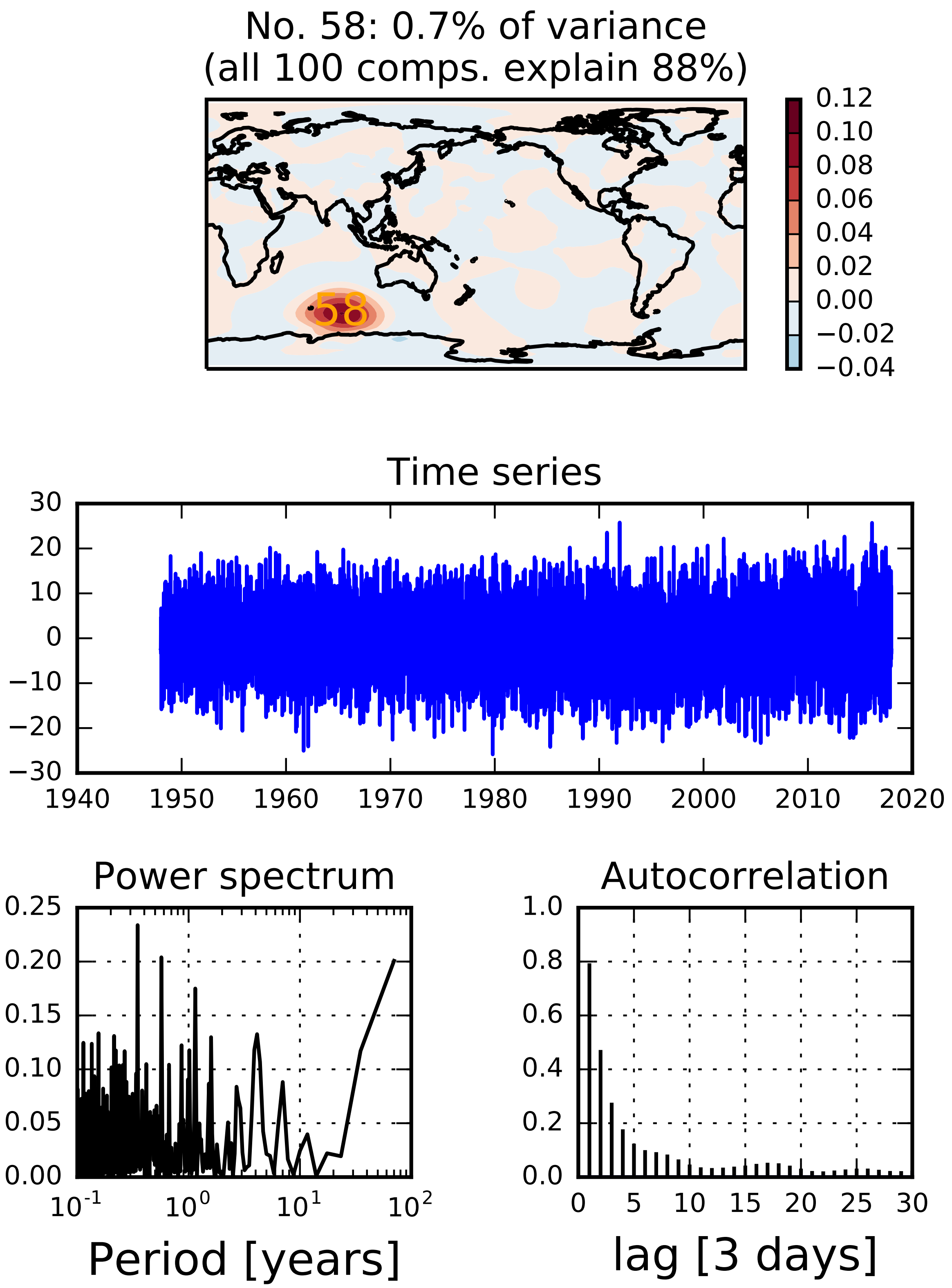
No. 58: 0.7% of variance
(all 100 comps. explain 88%)
0.12
0.10
0.08
0.06
0.04
0.02
0.00
−0.02
−0.04
58
Time series
30
20
10
0
−10
−20
−30
1940
1950
1960
1970
1980
1990
2000
2010
2020
Power spectrum
0.25
0.20
0.15
0.10
0.05
0.00
10^{-1}
10^{0}
10^{1}
10^{2}
Period [years]
Autocorrelation
1.0
0.8
0.6
0.4
0.2
0.0
0
5
10
15
20
25
30
lag [3 days]

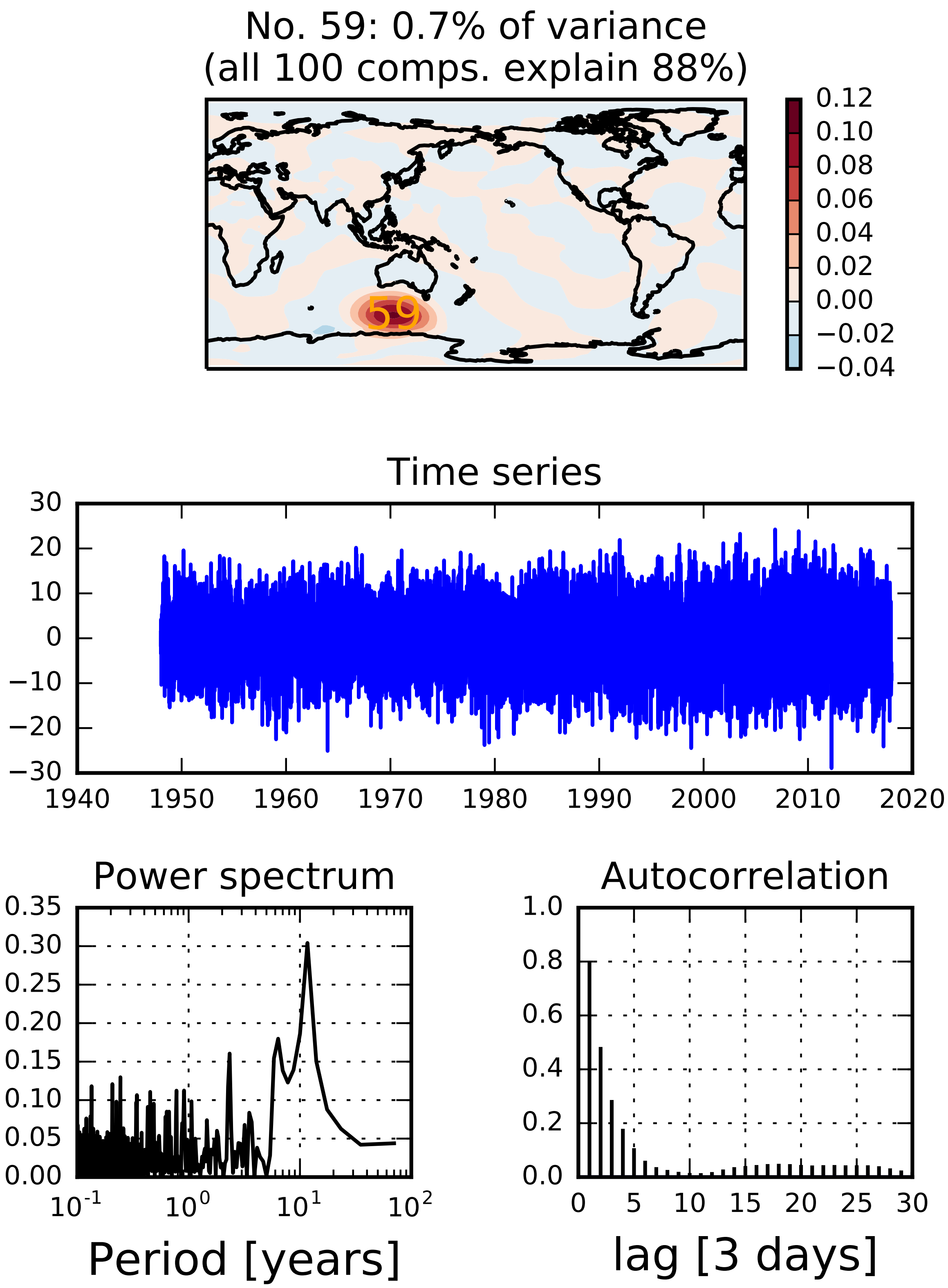
No. 59: 0.7% of variance
(all 100 comps. explain 88%)
59
0.12
0.10
0.08
0.06
0.04
0.02
0.00
−0.02
−0.04
Time series
30
20
10
0
−10
−20
−30
1940
1950
1960
1970
1980
1990
2000
2010
2020
Power spectrum
0.35
0.30
0.25
0.20
0.15
0.10
0.05
0.00
10^{-1}
10^{0}
10^{1}
10^{2}
Period [years]
Autocorrelation
1.0
0.8
0.6
0.4
0.2
0.0
0
5
10
15
20
25
30
lag [3 days]

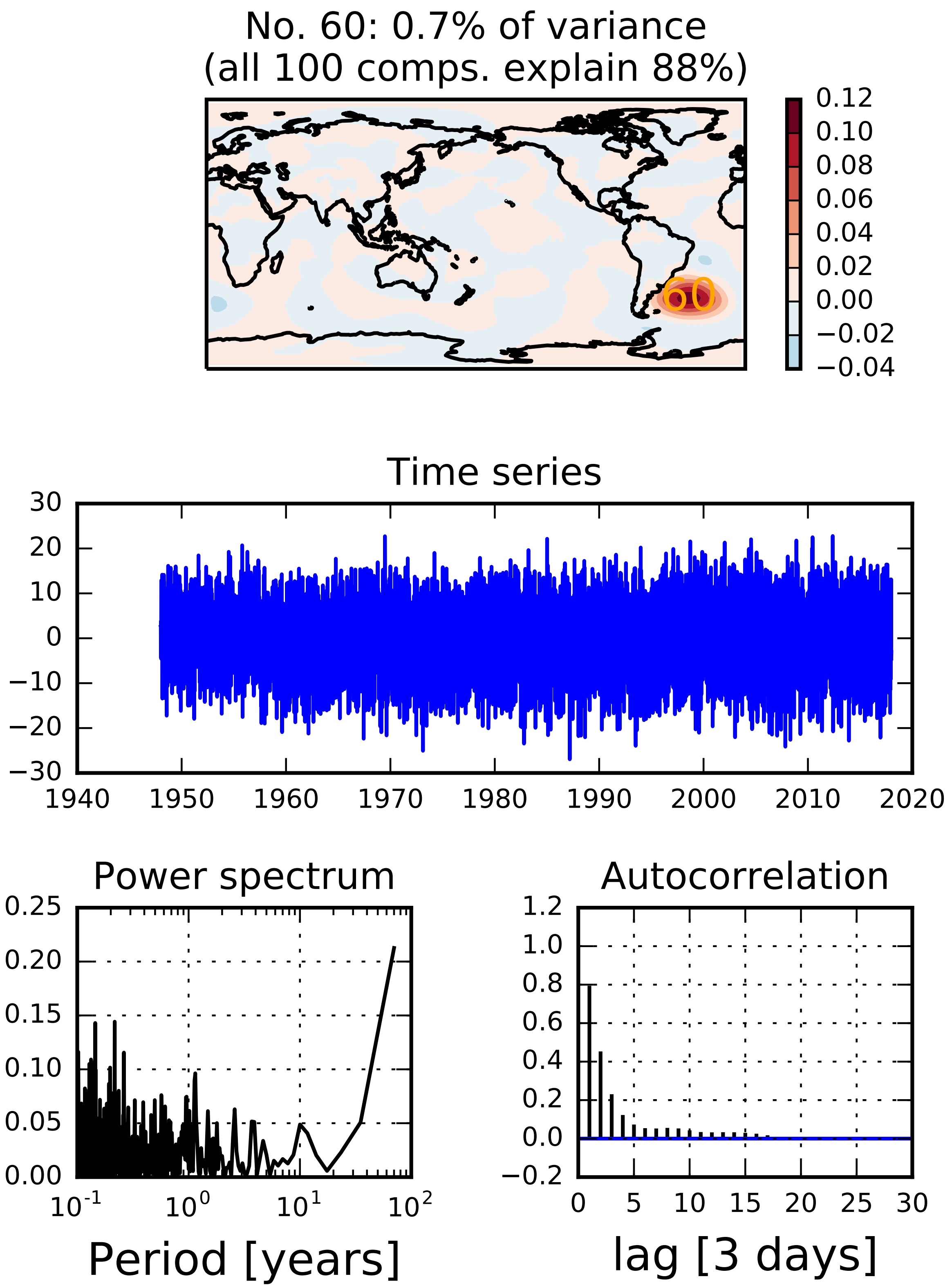
No. 60: 0.7% of variance
(all 100 comps. explain 88%)
60
0.12
0.10
0.08
0.06
0.04
0.02
0.00
−0.02
−0.04
Time series
30
20
10
0
−10
−20
−30
1940
1950
1960
1970
1980
1990
2000
2010
2020
Power spectrum
0.25
0.20
0.15
0.10
0.05
0.00
10^{-1}
10^{0}
10^{1}
10^{2}
Period [years]
Autocorrelation
1.2
1.0
0.8
0.6
0.4
0.2
0.0
−0.2
0
5
10
15
20
25
30
lag [3 days]

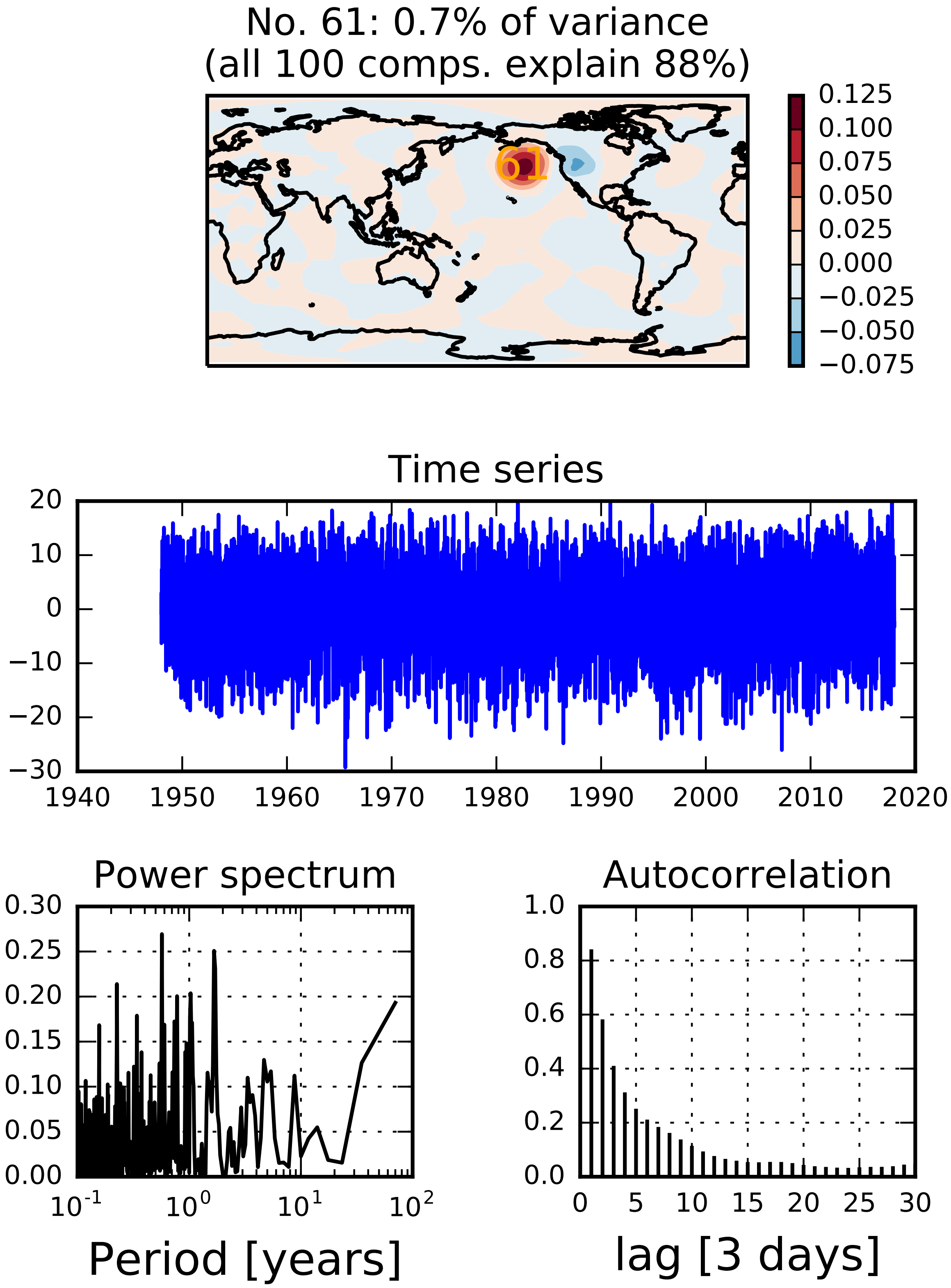
No. 61: 0.7% of variance
(all 100 comps. explain 88%)
61
0.125
0.100
0.075
0.050
0.025
0.000
−0.025
−0.050
−0.075
Time series
20
10
0
−10
−20
−30
1940
1950
1960
1970
1980
1990
2000
2010
2020
Power spectrum
0.30
0.25
0.20
0.15
0.10
0.05
0.00
10^{-1}
10^{0}
10^{1}
10^{2}
Period [years]
Autocorrelation
1.0
0.8
0.6
0.4
0.2
0.0
0
5
10
15
20
25
30
lag [3 days]

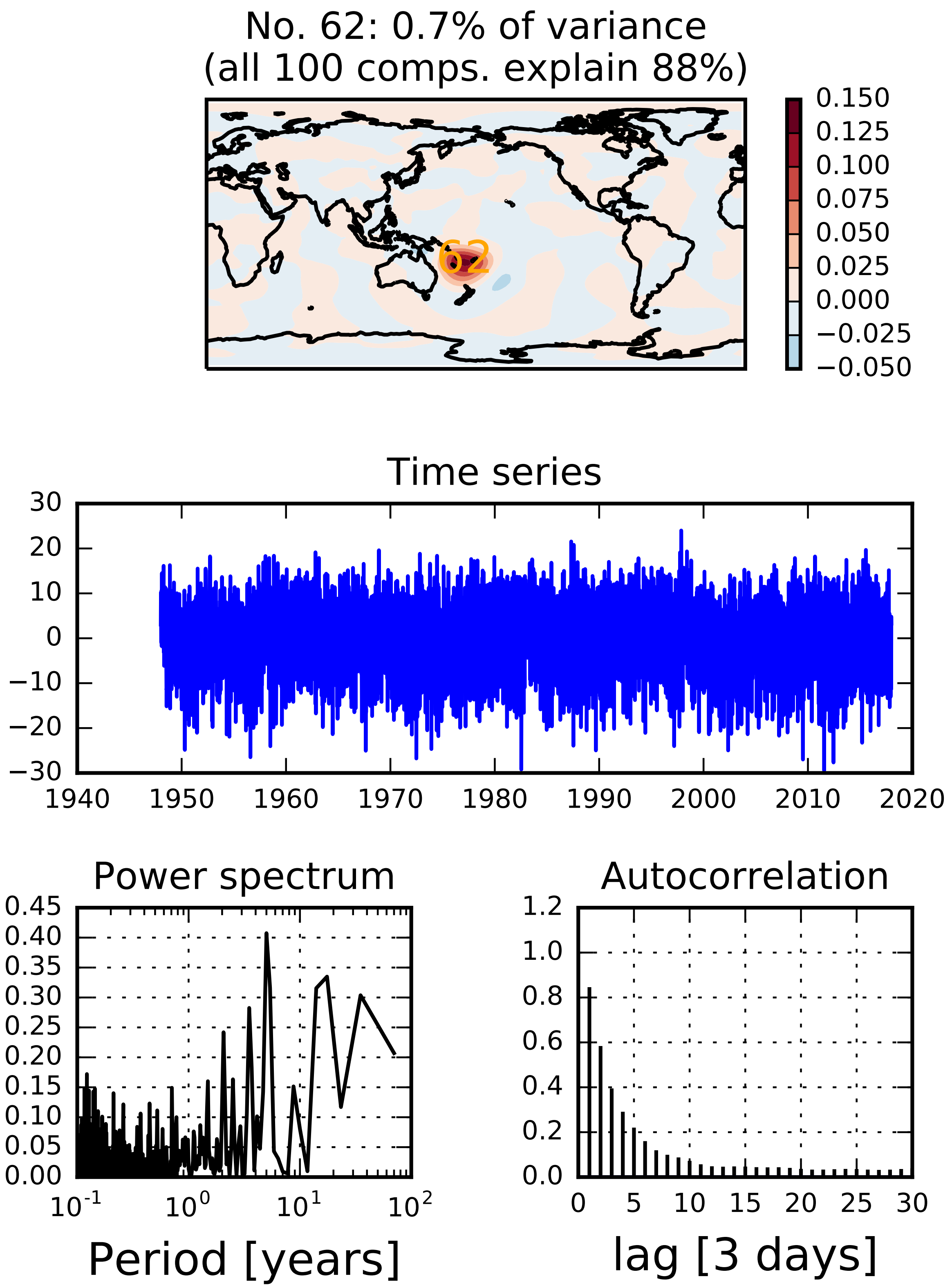
No. 62: 0.7% of variance
(all 100 comps. explain 88%)
62
0.150
0.125
0.100
0.075
0.050
0.025
0.000
−0.025
−0.050
Time series
30
20
10
0
−10
−20
−30
1940
1950
1960
1970
1980
1990
2000
2010
2020
Power spectrum
0.45
0.40
0.35
0.30
0.25
0.20
0.15
0.10
0.05
0.00
10^-1
10^0
10^1
10^2
Period [years]
Autocorrelation
1.2
1.0
0.8
0.6
0.4
0.2
0.0
0
5
10
15
20
25
30
lag [3 days]

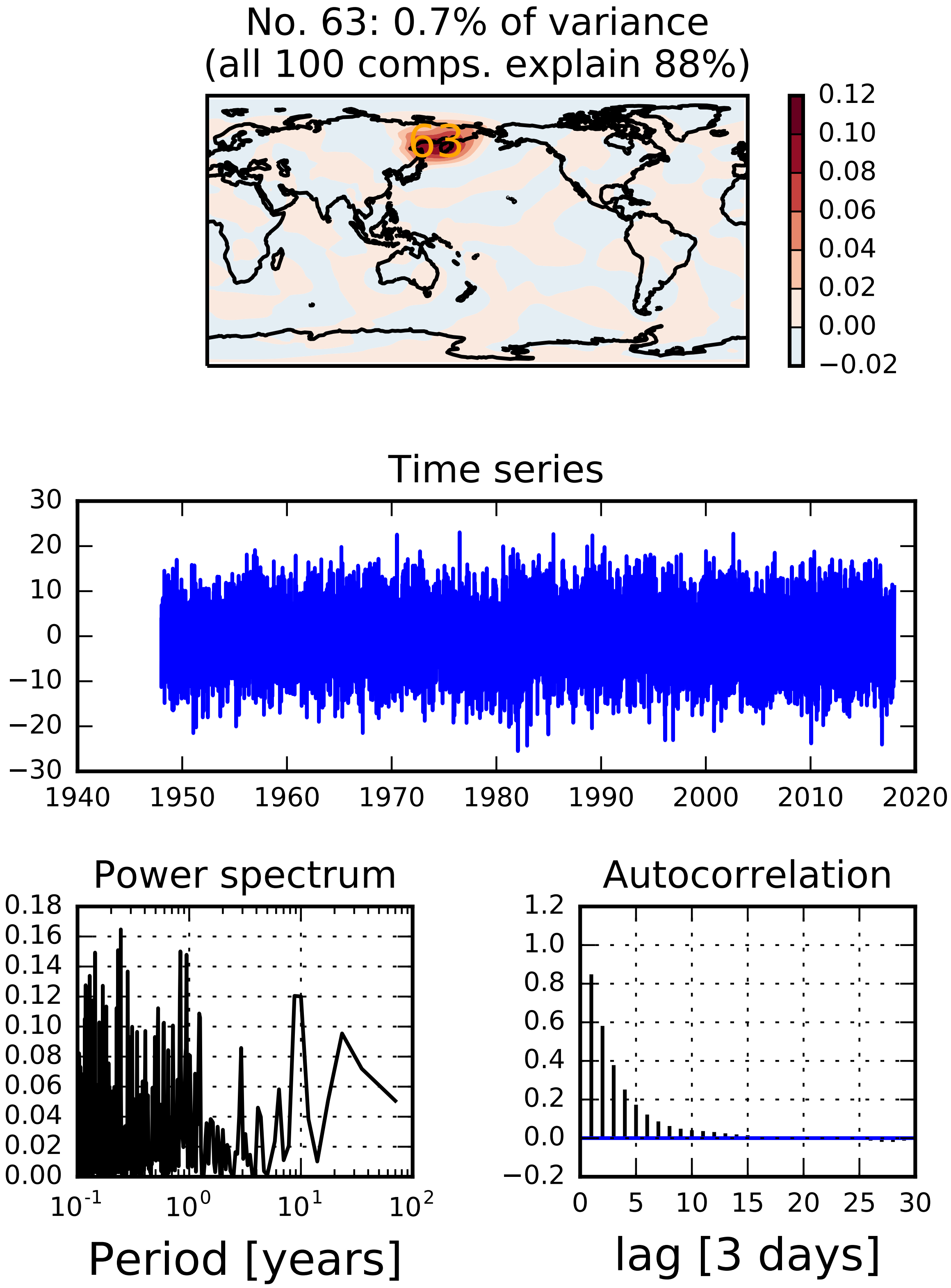
No. 63: 0.7% of variance
(all 100 comps. explain 88%)
63
0.12
0.10
0.08
0.06
0.04
0.02
0.00
−0.02
Time series
30
20
10
0
−10
−20
−30
1940
1950
1960
1970
1980
1990
2000
2010
2020
Power spectrum
0.18
0.16
0.14
0.12
0.10
0.08
0.06
0.04
0.02
0.00
10^-1
10^0
10^1
10^2
Period [years]
Autocorrelation
1.2
1.0
0.8
0.6
0.4
0.2
0.0
−0.2
0
5
10
15
20
25
30
lag [3 days]

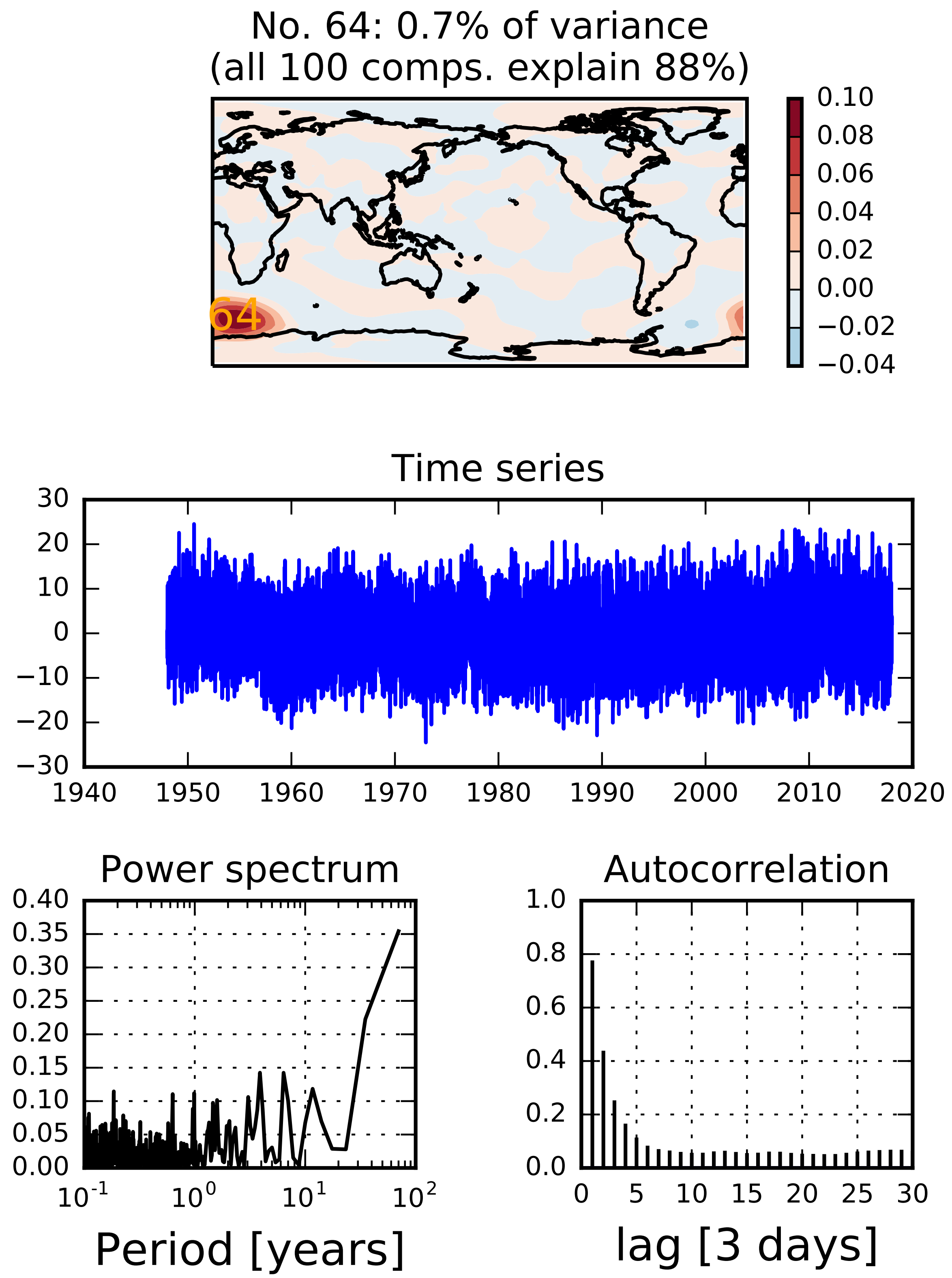
No. 64: 0.7% of variance
(all 100 comps. explain 88%)
64
0.10
0.08
0.06
0.04
0.02
0.00
−0.02
−0.04
Time series
30
20
10
0
−10
−20
−30
1940
1950
1960
1970
1980
1990
2000
2010
2020
Power spectrum
0.40
0.35
0.30
0.25
0.20
0.15
0.10
0.05
0.00
10-1
100
101
102
Period [years]
Autocorrelation
1.0
0.8
0.6
0.4
0.2
0.0
0
5
10
15
20
25
30
lag [3 days]

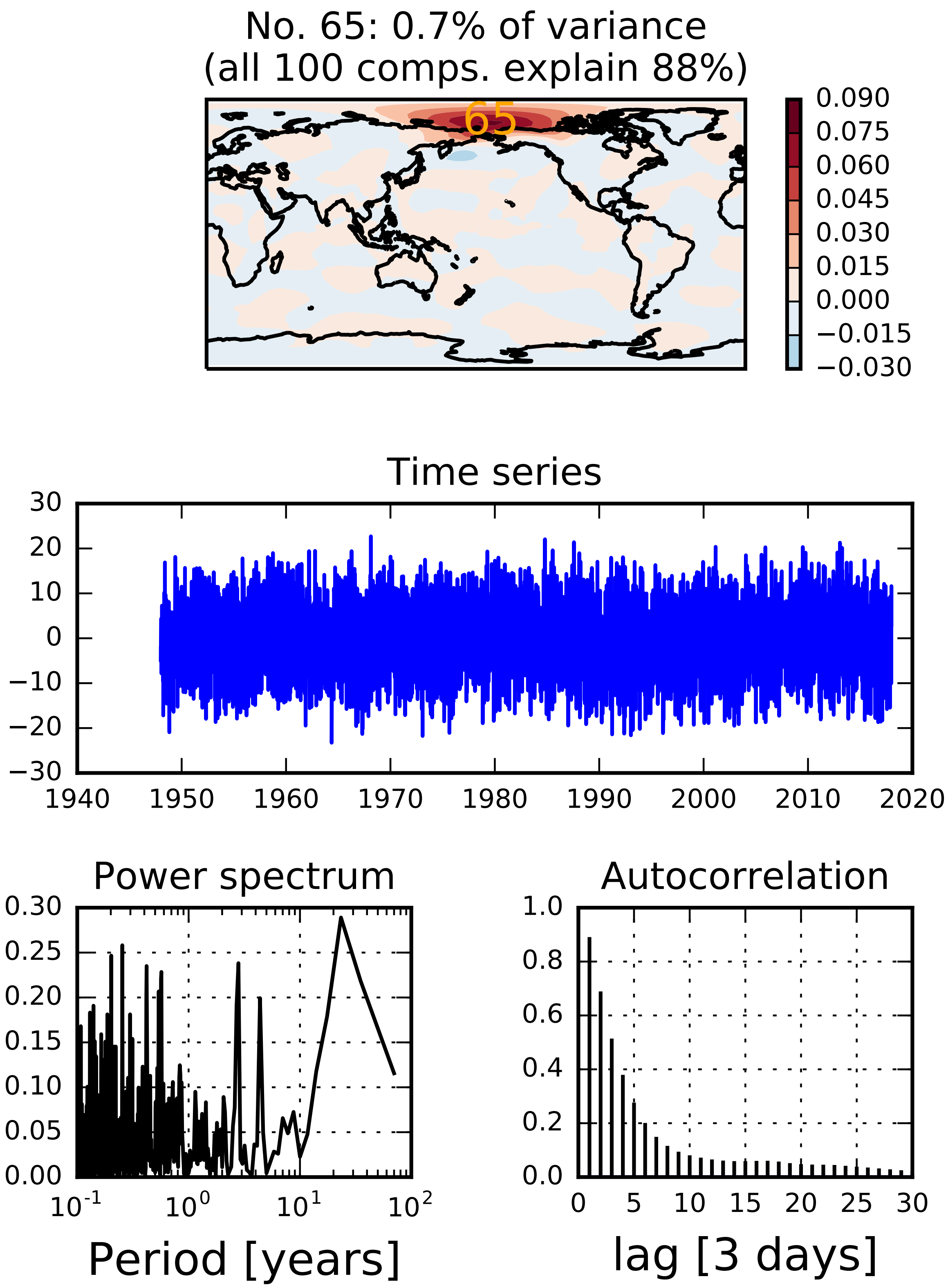
No. 65: 0.7% of variance
(all 100 comps. explain 88%)
65
0.090
0.075
0.060
0.045
0.030
0.015
0.000
−0.015
−0.030
Time series
30
20
10
0
−10
−20
−30
1940
1950
1960
1970
1980
1990
2000
2010
2020
Power spectrum
0.30
0.25
0.20
0.15
0.10
0.05
0.00
10^{-1}
10^{0}
10^{1}
10^{2}
Period [years]
Autocorrelation
1.0
0.8
0.6
0.4
0.2
0.0
0
5
10
15
20
25
30
lag [3 days]

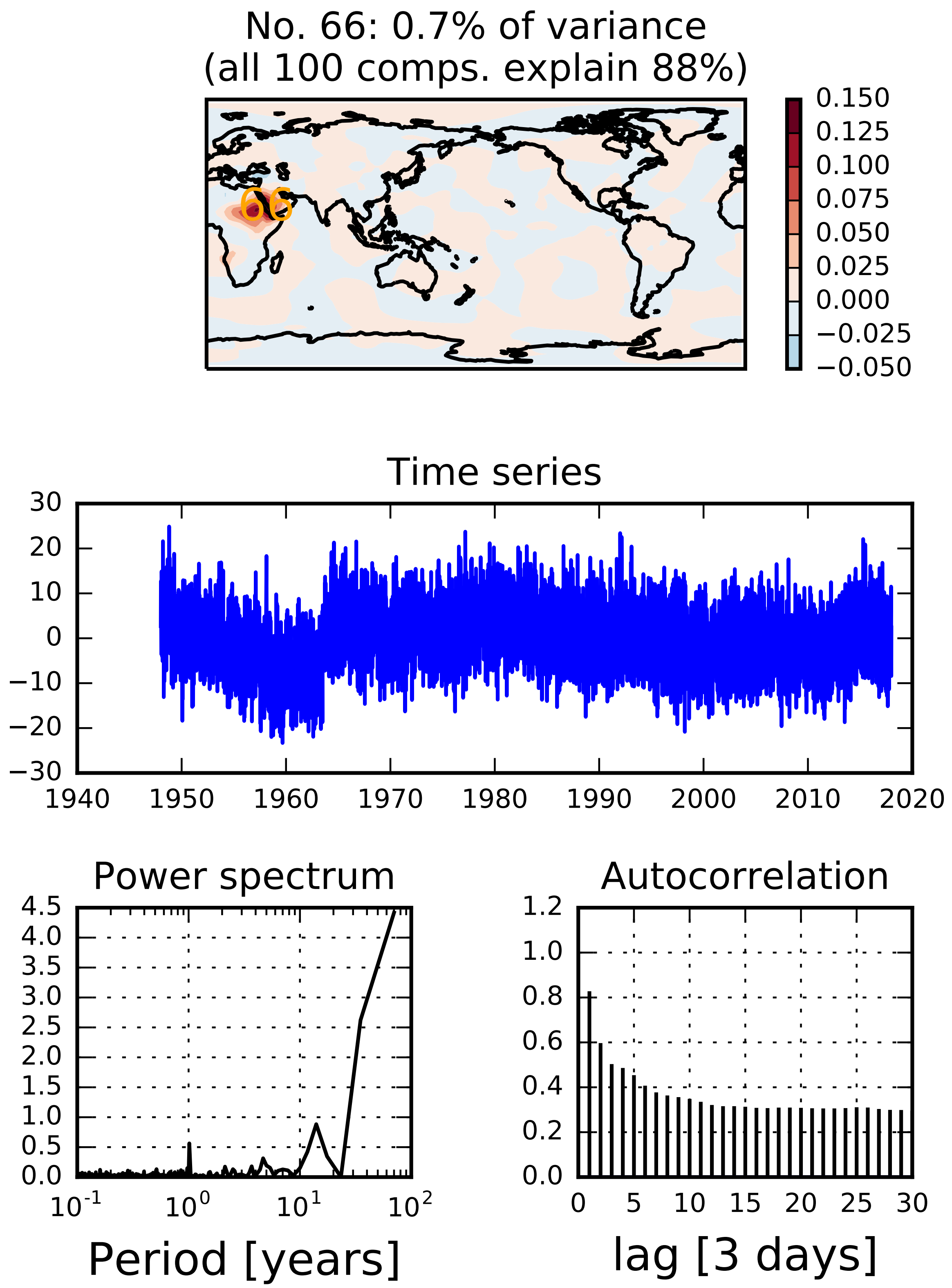
No. 66: 0.7% of variance
(all 100 comps. explain 88%)
66
0.150
0.125
0.100
0.075
0.050
0.025
0.000
−0.025
−0.050
Time series
30
20
10
0
−10
−20
−30
1940
1950
1960
1970
1980
1990
2000
2010
2020
Power spectrum
4.5
4.0
3.5
3.0
2.5
2.0
1.5
1.0
0.5
0.0
10^{-1}
10^{0}
10^{1}
10^{2}
Period [years]
Autocorrelation
1.2
1.0
0.8
0.6
0.4
0.2
0.0
0
5
10
15
20
25
30
lag [3 days]

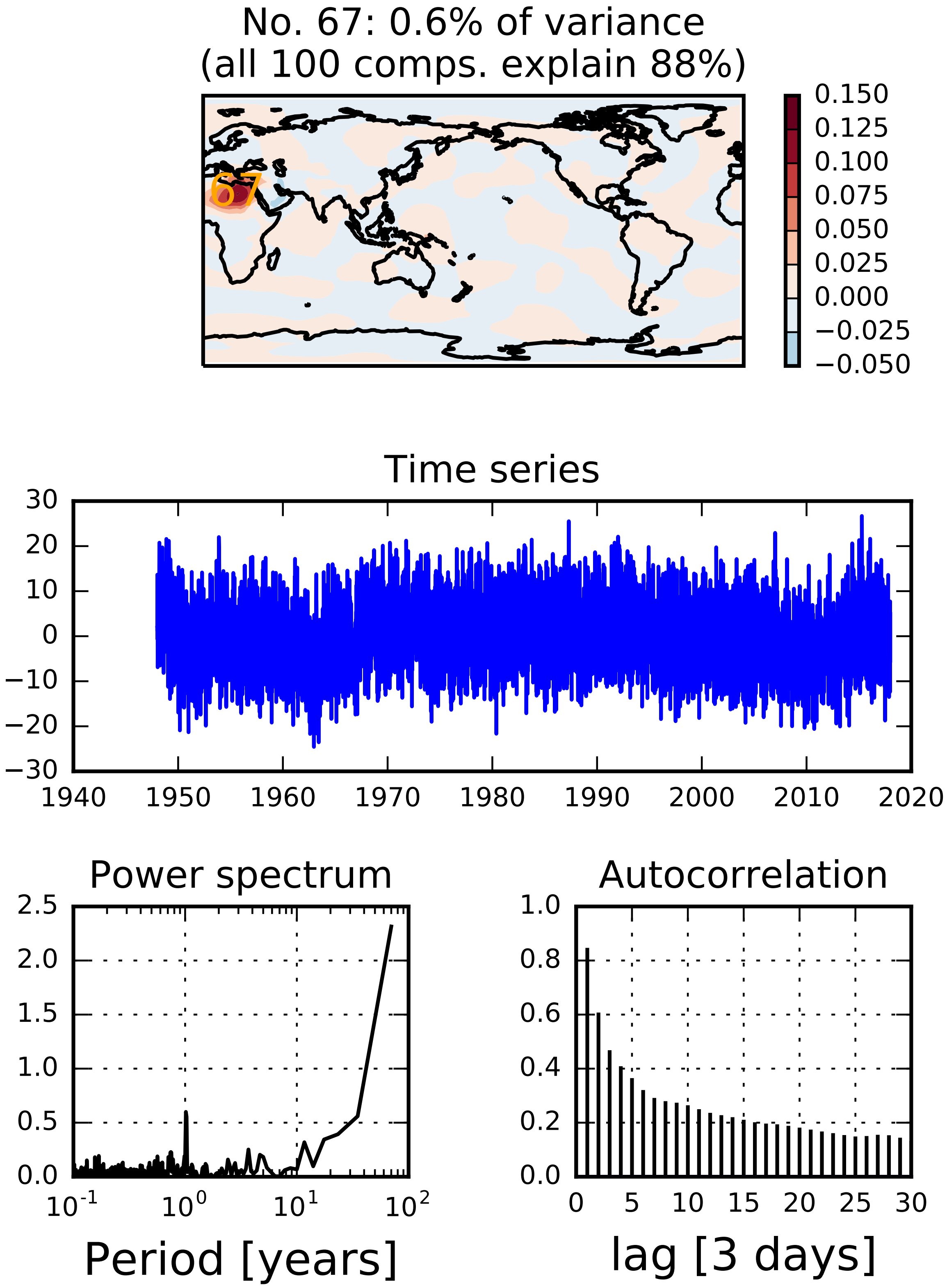
No. 67: 0.6% of variance
(all 100 comps. explain 88%)
67
0.150
0.125
0.100
0.075
0.050
0.025
0.000
−0.025
−0.050
Time series
30
20
10
0
−10
−20
−30
1940
1950
1960
1970
1980
1990
2000
2010
2020
Power spectrum
2.5
2.0
1.5
1.0
0.5
0.0
10^{-1}
10^{0}
10^{1}
10^{2}
Period [years]
Autocorrelation
1.0
0.8
0.6
0.4
0.2
0.0
0
5
10
15
20
25
30
lag [3 days]

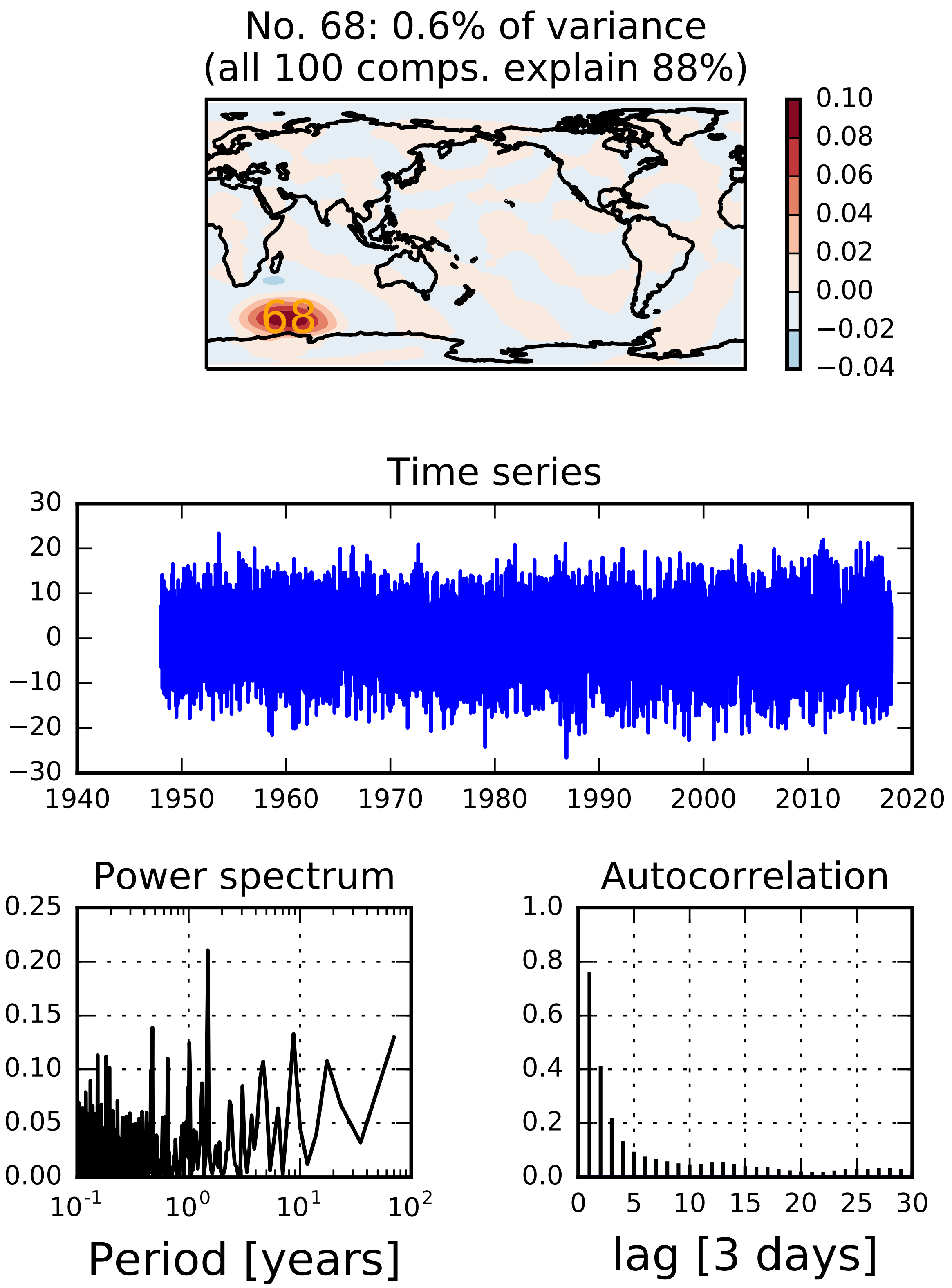
No. 68: 0.6% of variance
(all 100 comps. explain 88%)
68
0.10
0.08
0.06
0.04
0.02
0.00
−0.02
−0.04
Time series
30
20
10
0
−10
−20
−30
1940
1950
1960
1970
1980
1990
2000
2010
2020
Power spectrum
0.25
0.20
0.15
0.10
0.05
0.00
Period [years]
Autocorrelation
1.0
0.8
0.6
0.4
0.2
0.0
0
5
10
15
20
25
30
lag [3 days]

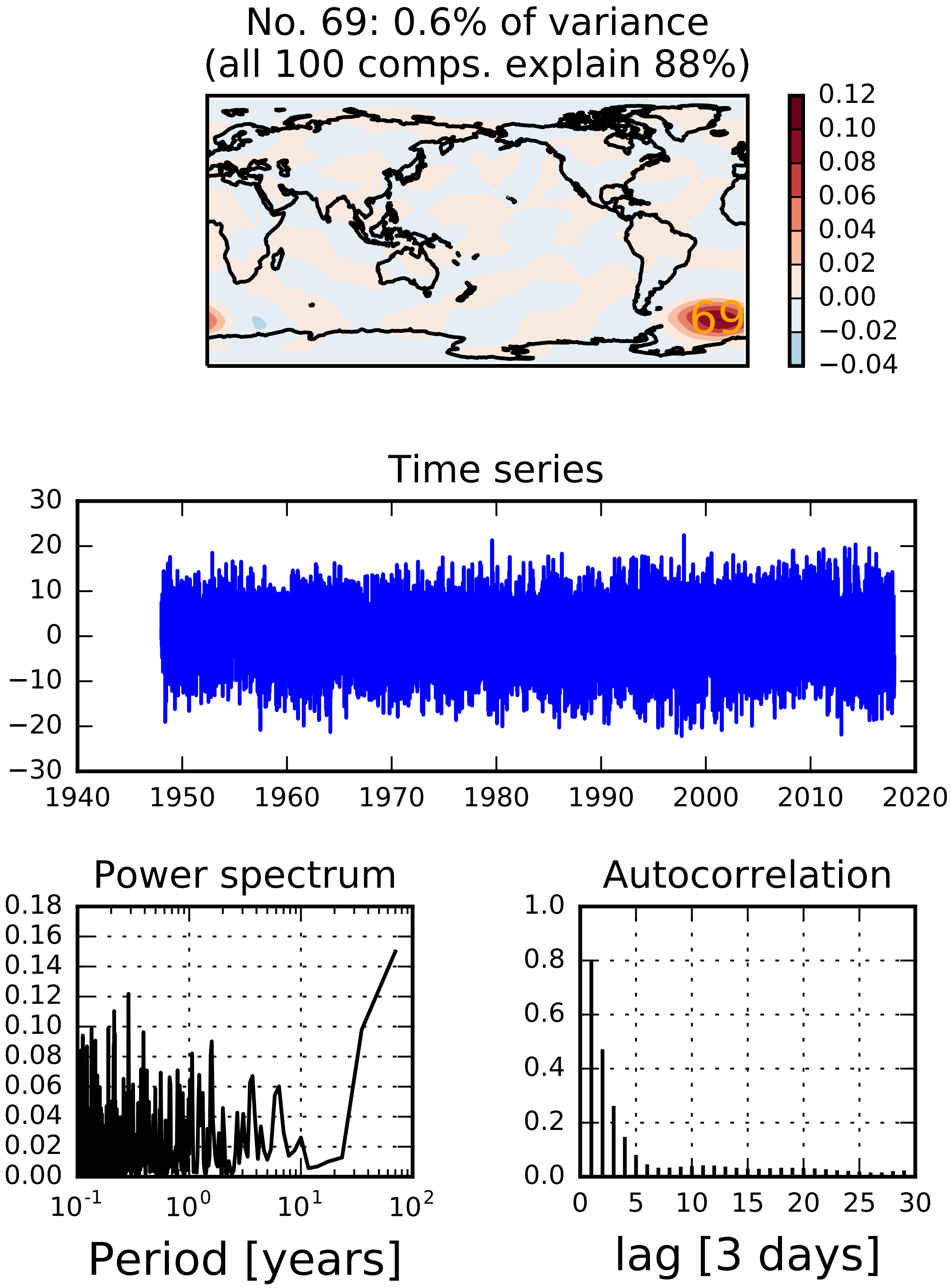
No. 69: 0.6% of variance
(all 100 comps. explain 88%)
69
0.12
0.10
0.08
0.06
0.04
0.02
0.00
−0.02
−0.04
Time series
30
20
10
0
−10
−20
−30
1940
1950
1960
1970
1980
1990
2000
2010
2020
Power spectrum
0.18
0.16
0.14
0.12
0.10
0.08
0.06
0.04
0.02
0.00
10^-1
10^0
10^1
10^2
Period [years]
Autocorrelation
1.0
0.8
0.6
0.4
0.2
0.0
0
5
10
15
20
25
30
lag [3 days]

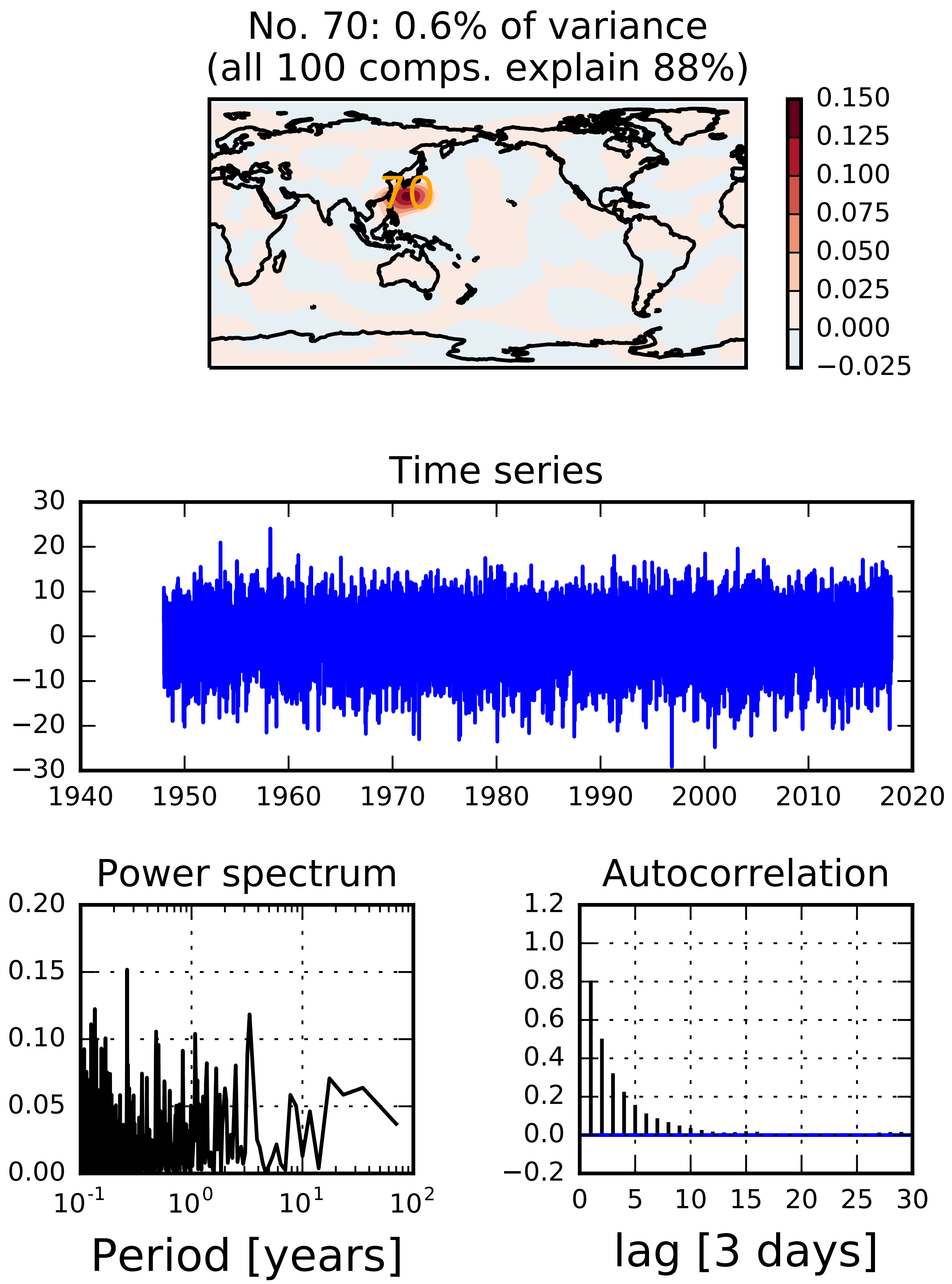

No. 70: 0.6% of variance
(all 100 comps. explain 88%)
70
0.150
0.125
0.100
0.075
0.050
0.025
0.000
−0.025
Time series
30
20
10
0
−10
−20
−30
1940
1950
1960
1970
1980
1990
2000
2010
2020
Power spectrum
0.20
0.15
0.10
0.05
0.00
10^{-1}
10^{0}
10^{1}
10^{2}
Period [years]
Autocorrelation
1.2
1.0
0.8
0.6
0.4
0.2
0.0
−0.2
0
5
10
15
20
25
30
lag [3 days]

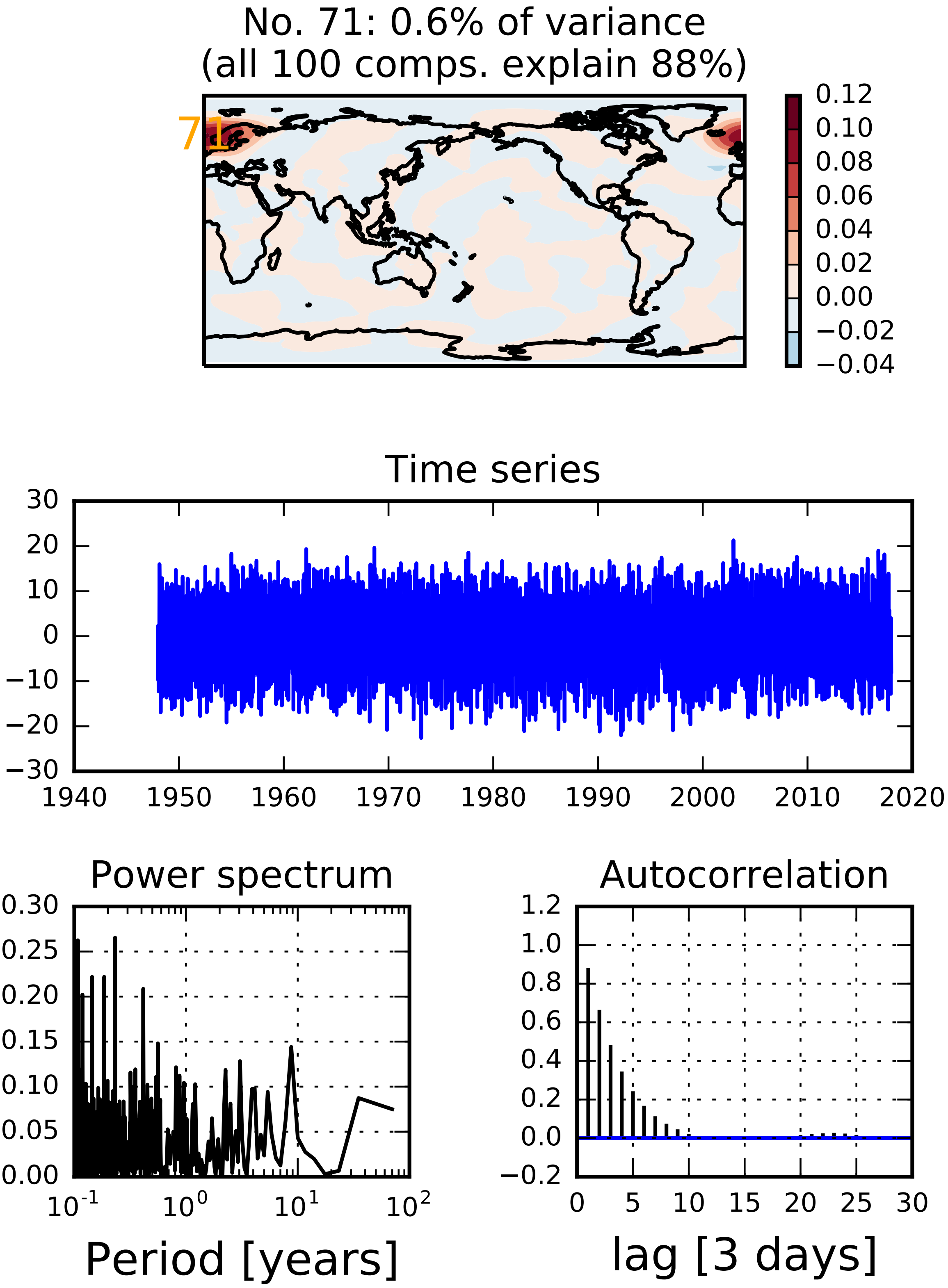
No. 71: 0.6% of variance
(all 100 comps. explain 88%)
71
0.12
0.10
0.08
0.06
0.04
0.02
0.00
−0.02
−0.04
Time series
30
20
10
0
−10
−20
−30
1940
1950
1960
1970
1980
1990
2000
2010
2020
Power spectrum
0.30
0.25
0.20
0.15
0.10
0.05
0.00
10^-1
10^0
10^1
10^2
Period [years]
Autocorrelation
1.2
1.0
0.8
0.6
0.4
0.2
0.0
−0.2
0
5
10
15
20
25
30
lag [3 days]

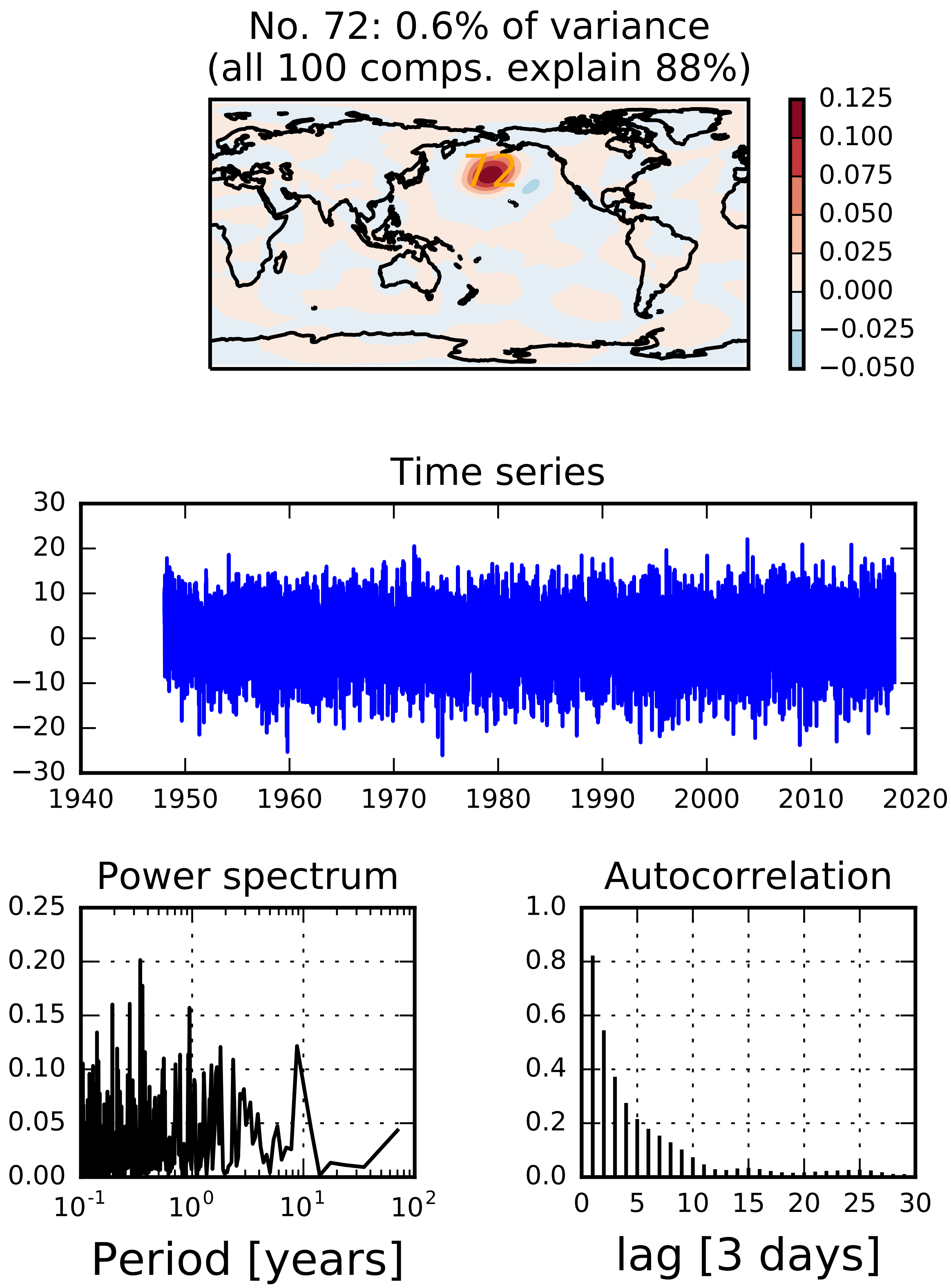
No. 72: 0.6% of variance
(all 100 comps. explain 88%)
72
0.125
0.100
0.075
0.050
0.025
0.000
−0.025
−0.050
Time series
30
20
10
0
−10
−20
−30
1940
1950
1960
1970
1980
1990
2000
2010
2020
Power spectrum
0.25
0.20
0.15
0.10
0.05
0.00
10^{-1}
10^{0}
10^{1}
10^{2}
Period [years]
Autocorrelation
1.0
0.8
0.6
0.4
0.2
0.0
0
5
10
15
20
25
30
lag [3 days]

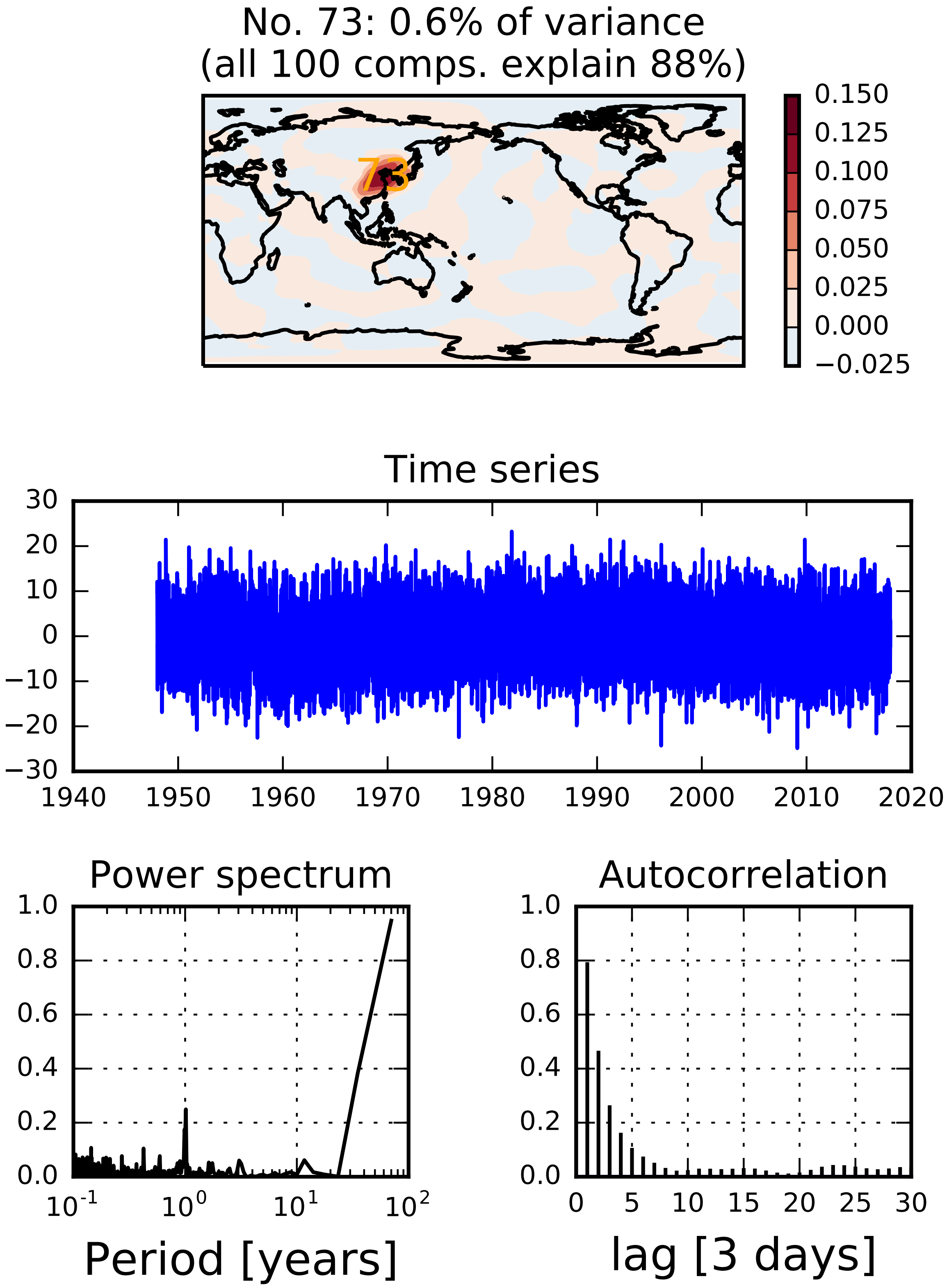
No. 73: 0.6% of variance
(all 100 comps. explain 88%)
73
0.150
0.125
0.100
0.075
0.050
0.025
0.000
−0.025
Time series
30
20
10
0
−10
−20
−30
1940
1950
1960
1970
1980
1990
2000
2010
2020
Power spectrum
1.0
0.8
0.6
0.4
0.2
0.0
10^{-1}
10^{0}
10^{1}
10^{2}
Period [years]
Autocorrelation
1.0
0.8
0.6
0.4
0.2
0.0
0
5
10
15
20
25
30
lag [3 days]

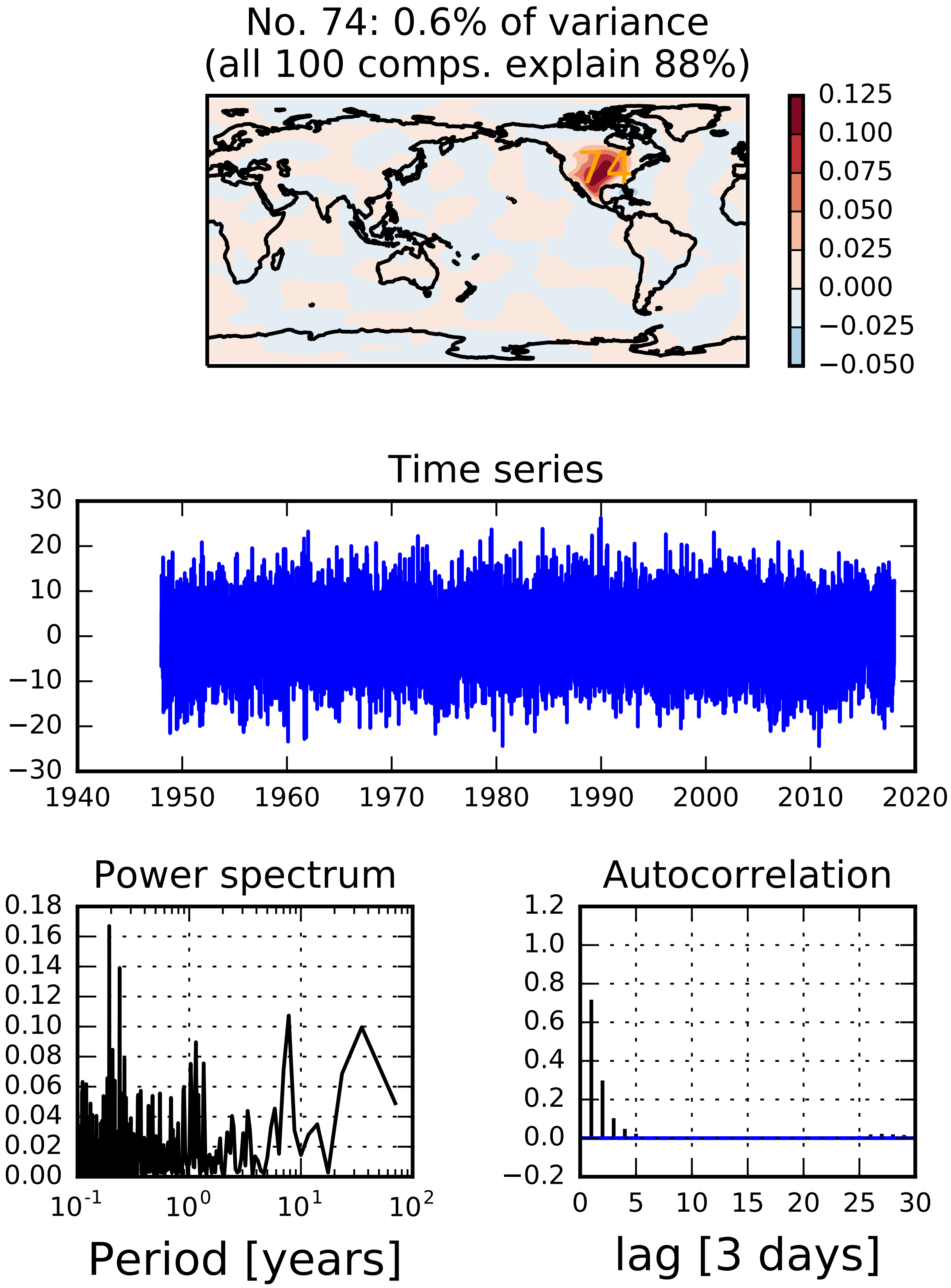
No. 74: 0.6% of variance
(all 100 comps. explain 88%)
74
0.125
0.100
0.075
0.050
0.025
0.000
−0.025
−0.050
Time series
30
20
10
0
−10
−20
−30
1940
1950
1960
1970
1980
1990
2000
2010
2020
Power spectrum
0.18
0.16
0.14
0.12
0.10
0.08
0.06
0.04
0.02
0.00
10^-1
10^0
10^1
10^2
Period [years]
Autocorrelation
1.2
1.0
0.8
0.6
0.4
0.2
0.0
−0.2
0
5
10
15
20
25
30
lag [3 days]

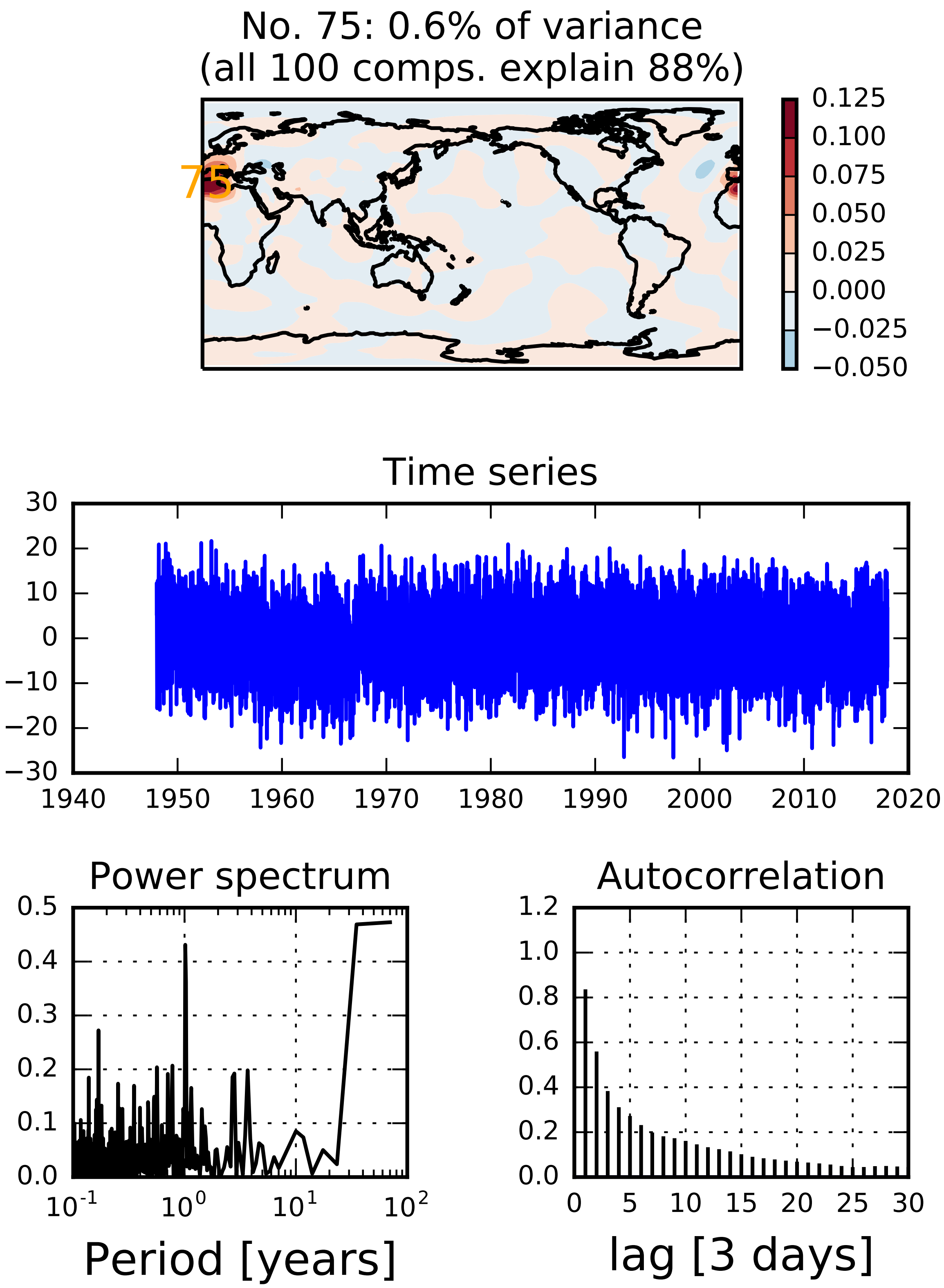
No. 75: 0.6% of variance
(all 100 comps. explain 88%)
75
0.125
0.100
0.075
0.050
0.025
0.000
−0.025
−0.050
Time series
30
20
10
0
−10
−20
−30
1940
1950
1960
1970
1980
1990
2000
2010
2020
Power spectrum
0.5
0.4
0.3
0.2
0.1
0.0
10^{-1}
10^{0}
10^{1}
10^{2}
Period [years]
Autocorrelation
1.2
1.0
0.8
0.6
0.4
0.2
0.0
0
5
10
15
20
25
30
lag [3 days]

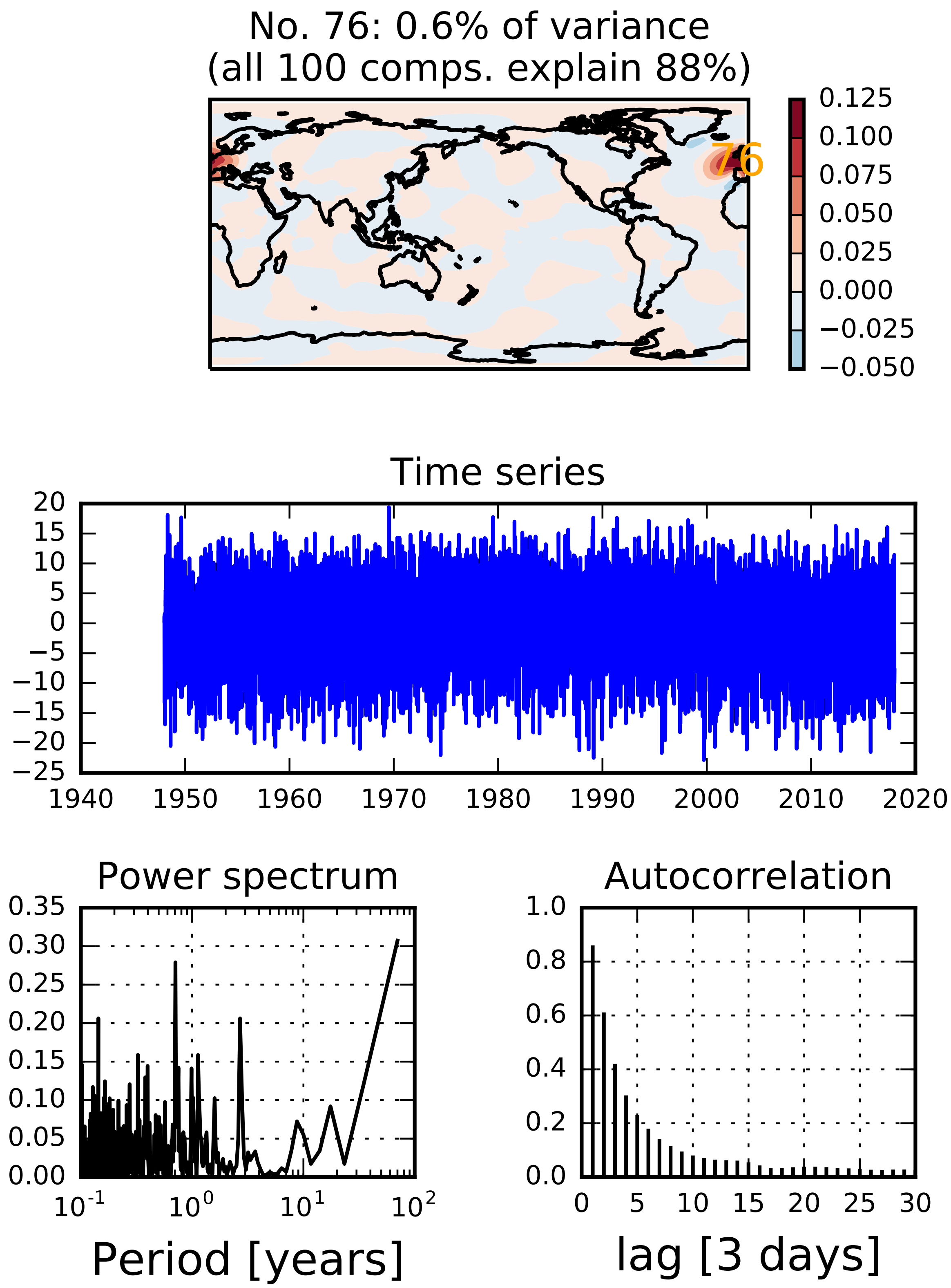
No. 76: 0.6% of variance
(all 100 comps. explain 88%)
76
0.125
0.100
0.075
0.050
0.025
0.000
−0.025
−0.050
Time series
20
15
10
5
0
−5
−10
−15
−20
−25
1940
1950
1960
1970
1980
1990
2000
2010
2020
Power spectrum
0.35
0.30
0.25
0.20
0.15
0.10
0.05
0.00
10^-1
10^0
10^1
10^2
Period [years]
Autocorrelation
1.0
0.8
0.6
0.4
0.2
0.0
0
5
10
15
20
25
30
lag [3 days]

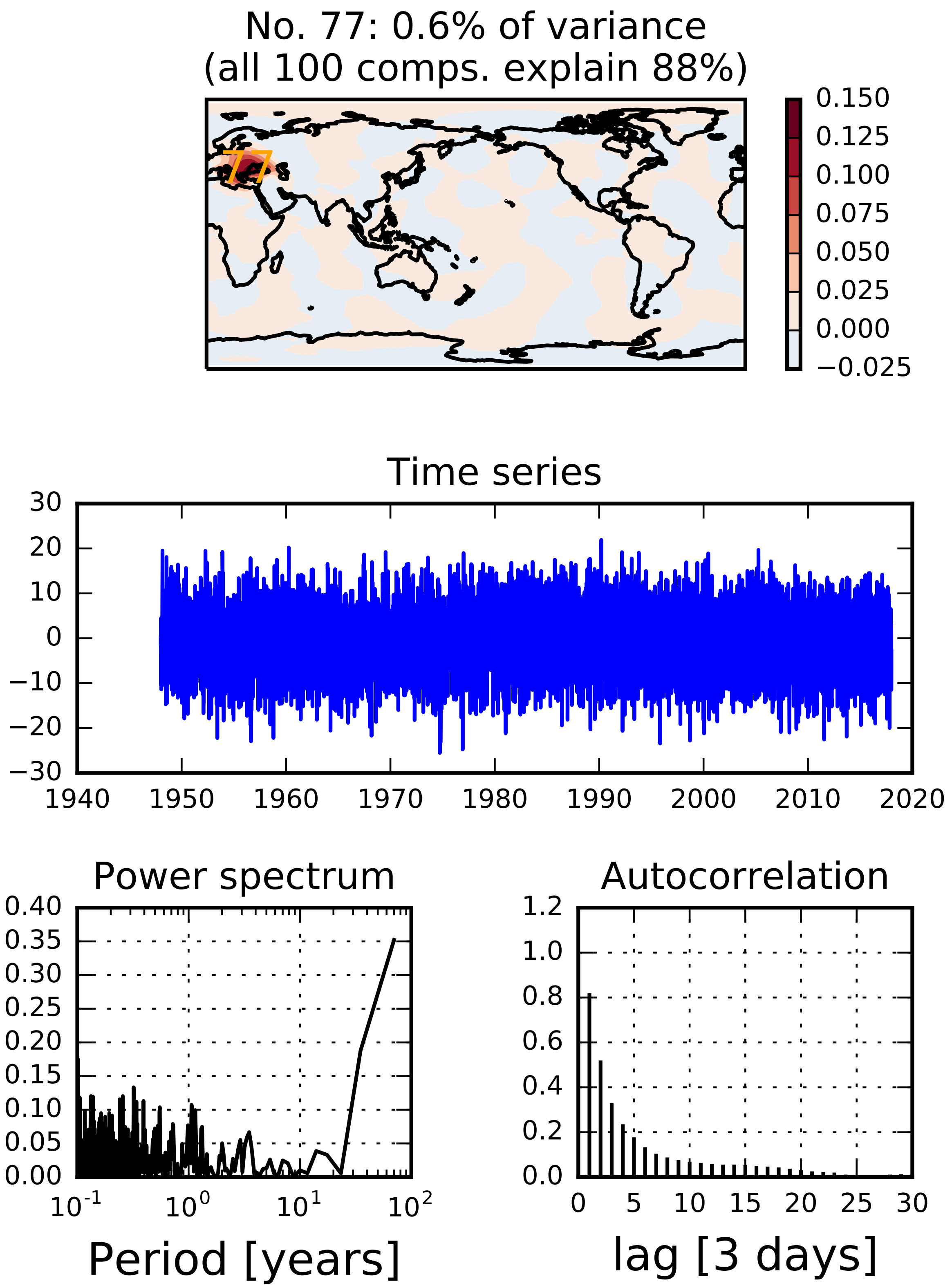
No. 77: 0.6% of variance
(all 100 comps. explain 88%)
77
0.150
0.125
0.100
0.075
0.050
0.025
0.000
−0.025
Time series
30
20
10
0
−10
−20
−30
1940
1950
1960
1970
1980
1990
2000
2010
2020
Power spectrum
0.40
0.35
0.30
0.25
0.20
0.15
0.10
0.05
0.00
10^{-1}
10^{0}
10^{1}
10^{2}
Period [years]
Autocorrelation
1.2
1.0
0.8
0.6
0.4
0.2
0.0
0
5
10
15
20
25
30
lag [3 days]

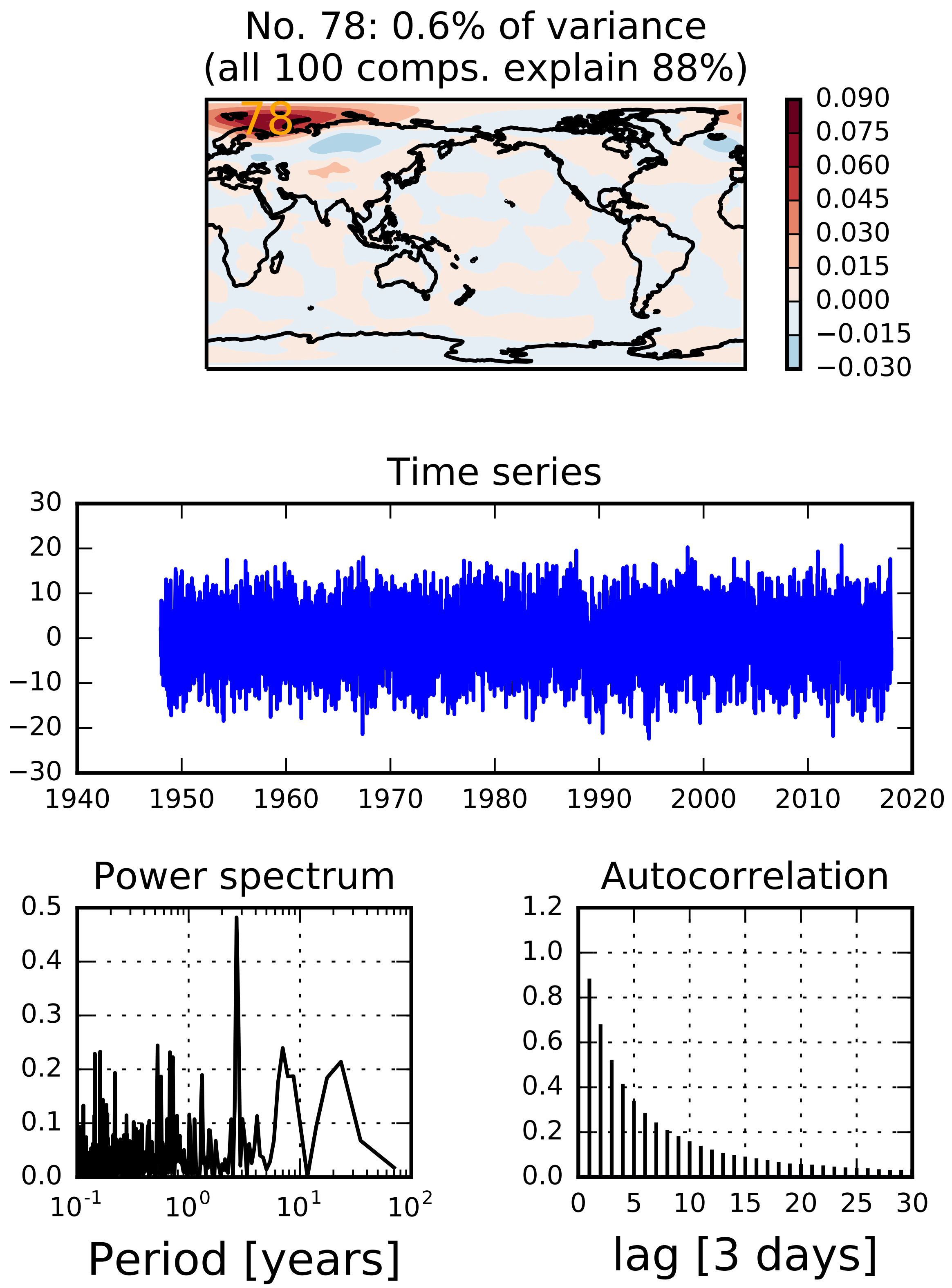

No. 78: 0.6% of variance
(all 100 comps. explain 88%)
78
0.090
0.075
0.060
0.045
0.030
0.015
0.000
−0.015
−0.030
Time series
30
20
10
0
−10
−20
−30
1940
1950
1960
1970
1980
1990
2000
2010
2020
Power spectrum
0.5
0.4
0.3
0.2
0.1
0.0
10^{-1}
10^{0}
10^{1}
10^{2}
Period [years]
Autocorrelation
1.2
1.0
0.8
0.6
0.4
0.2
0.0
0 5 10 15 20 25 30
lag [3 days]

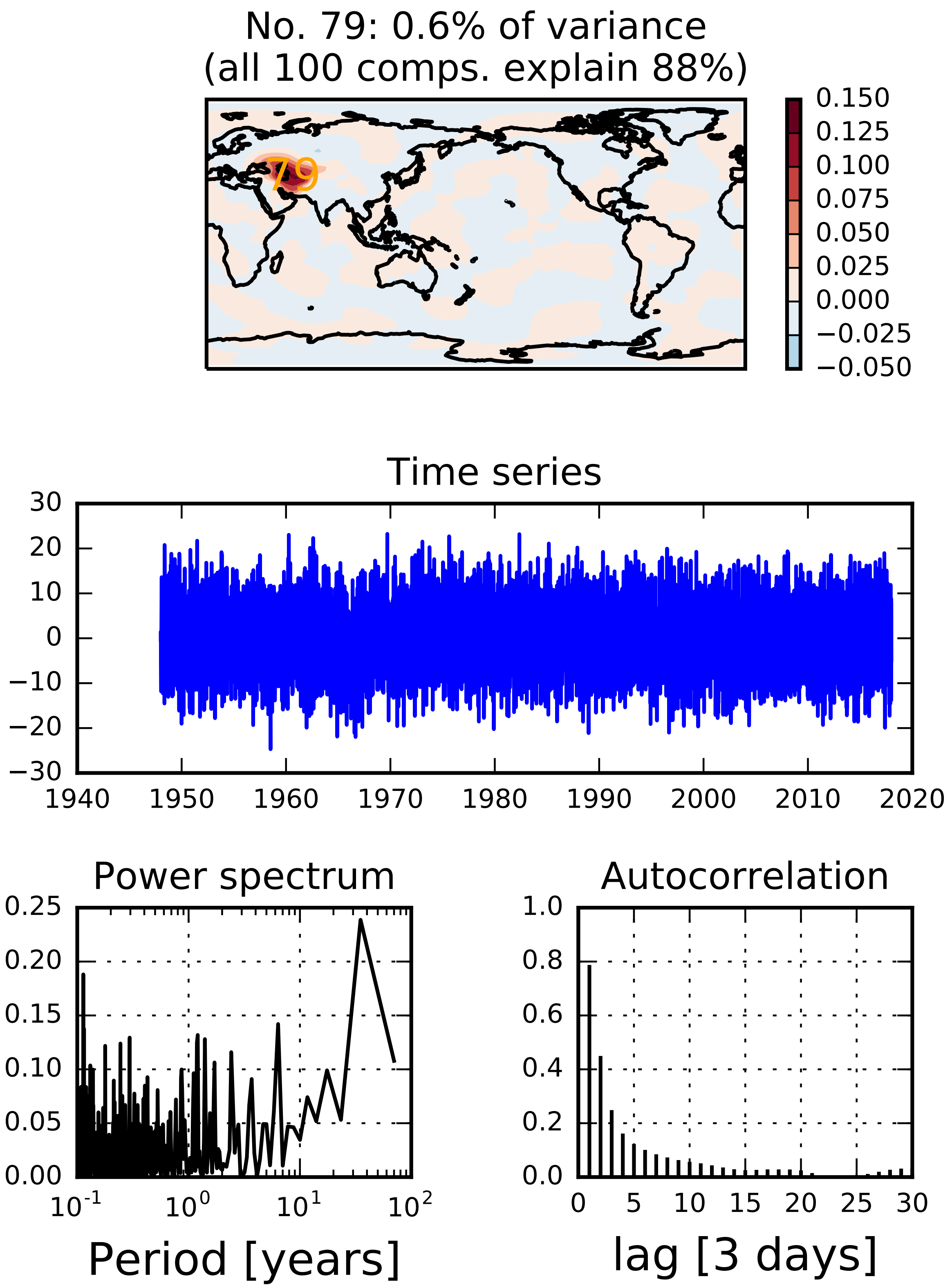
No. 79: 0.6% of variance
(all 100 comps. explain 88%)
79
0.150
0.125
0.100
0.075
0.050
0.025
0.000
−0.025
−0.050
Time series
30
20
10
0
−10
−20
−30
1940
1950
1960
1970
1980
1990
2000
2010
2020
Power spectrum
0.25
0.20
0.15
0.10
0.05
0.00
10^{-1}
10^{0}
10^{1}
10^{2}
Period [years]
Autocorrelation
1.0
0.8
0.6
0.4
0.2
0.0
0
5
10
15
20
25
30
lag [3 days]

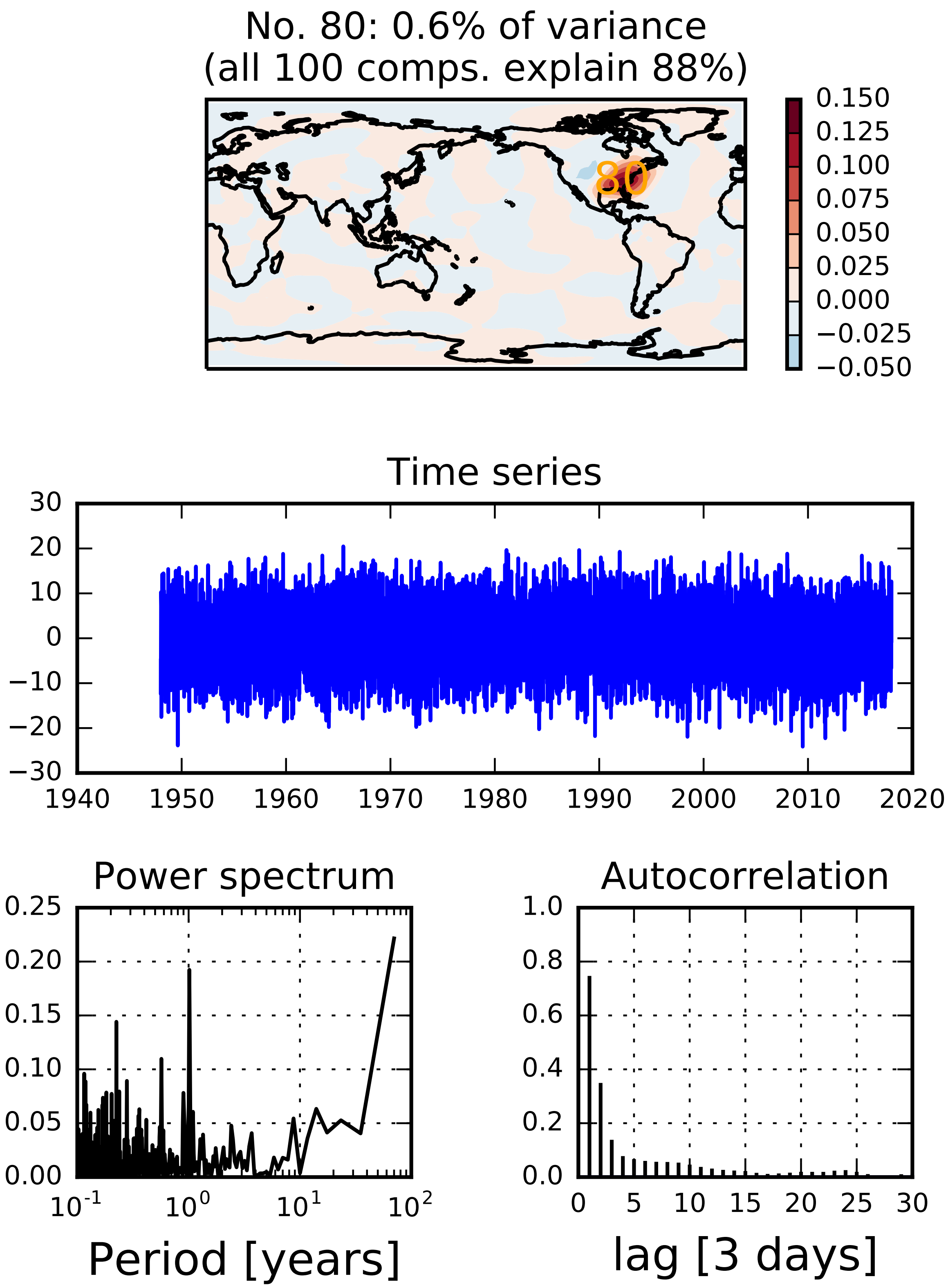
No. 80: 0.6% of variance
(all 100 comps. explain 88%)
80
0.150
0.125
0.100
0.075
0.050
0.025
0.000
−0.025
−0.050
Time series
30
20
10
0
−10
−20
−30
1940
1950
1960
1970
1980
1990
2000
2010
2020
Power spectrum
0.25
0.20
0.15
0.10
0.05
0.00
10^{-1}
10^{0}
10^{1}
10^{2}
Period [years]
Autocorrelation
1.0
0.8
0.6
0.4
0.2
0.0
0
5
10
15
20
25
30
lag [3 days]

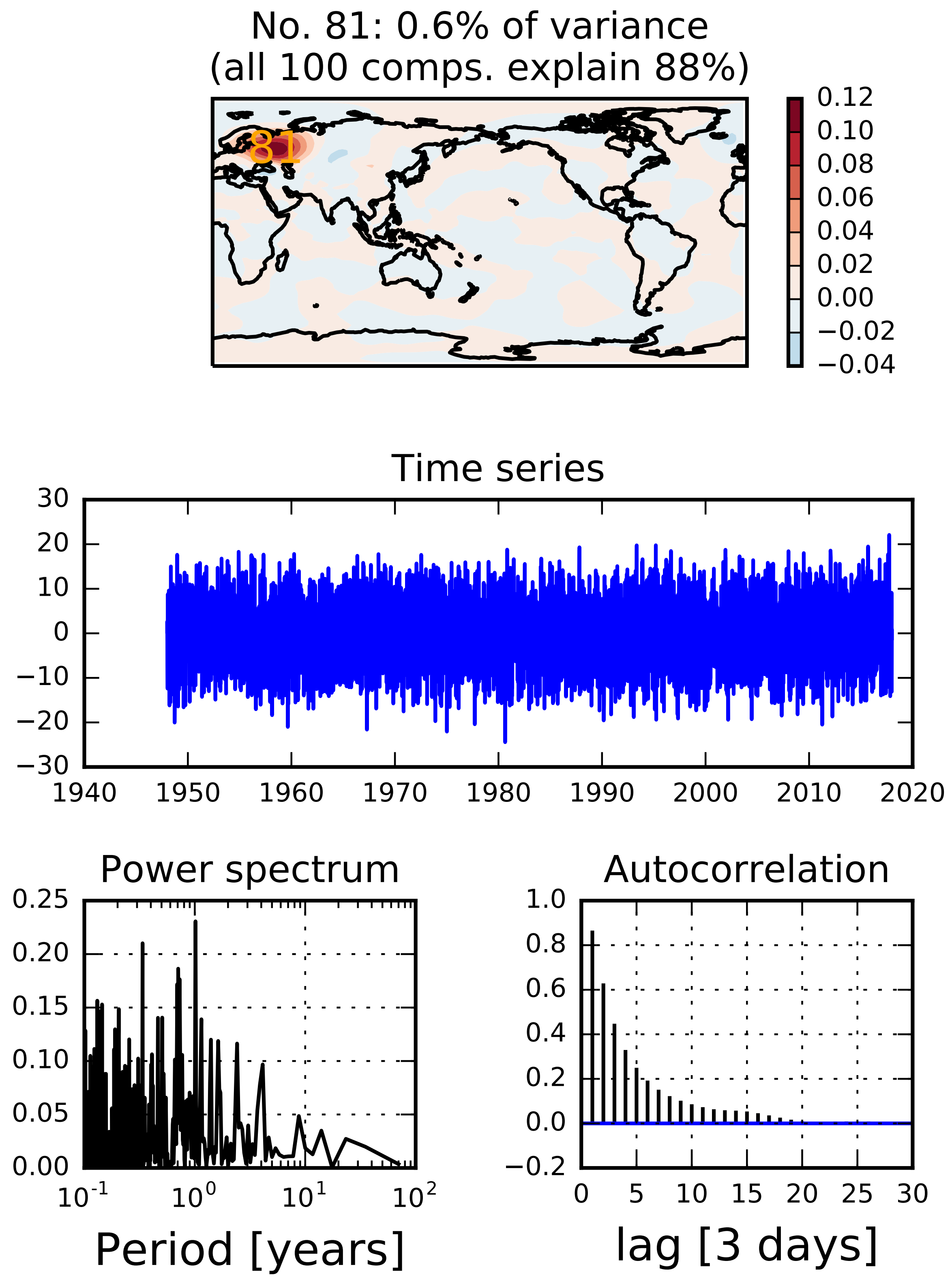
No. 81: 0.6% of variance
(all 100 comps. explain 88%)
81
0.12
0.10
0.08
0.06
0.04
0.02
0.00
−0.02
−0.04
Time series
30
20
10
0
−10
−20
−30
1940
1950
1960
1970
1980
1990
2000
2010
2020
Power spectrum
0.25
0.20
0.15
0.10
0.05
0.00
10^{-1}
10^{0}
10^{1}
10^{2}
Period [years]
Autocorrelation
1.0
0.8
0.6
0.4
0.2
0.0
−0.2
0
5
10
15
20
25
30
lag [3 days]

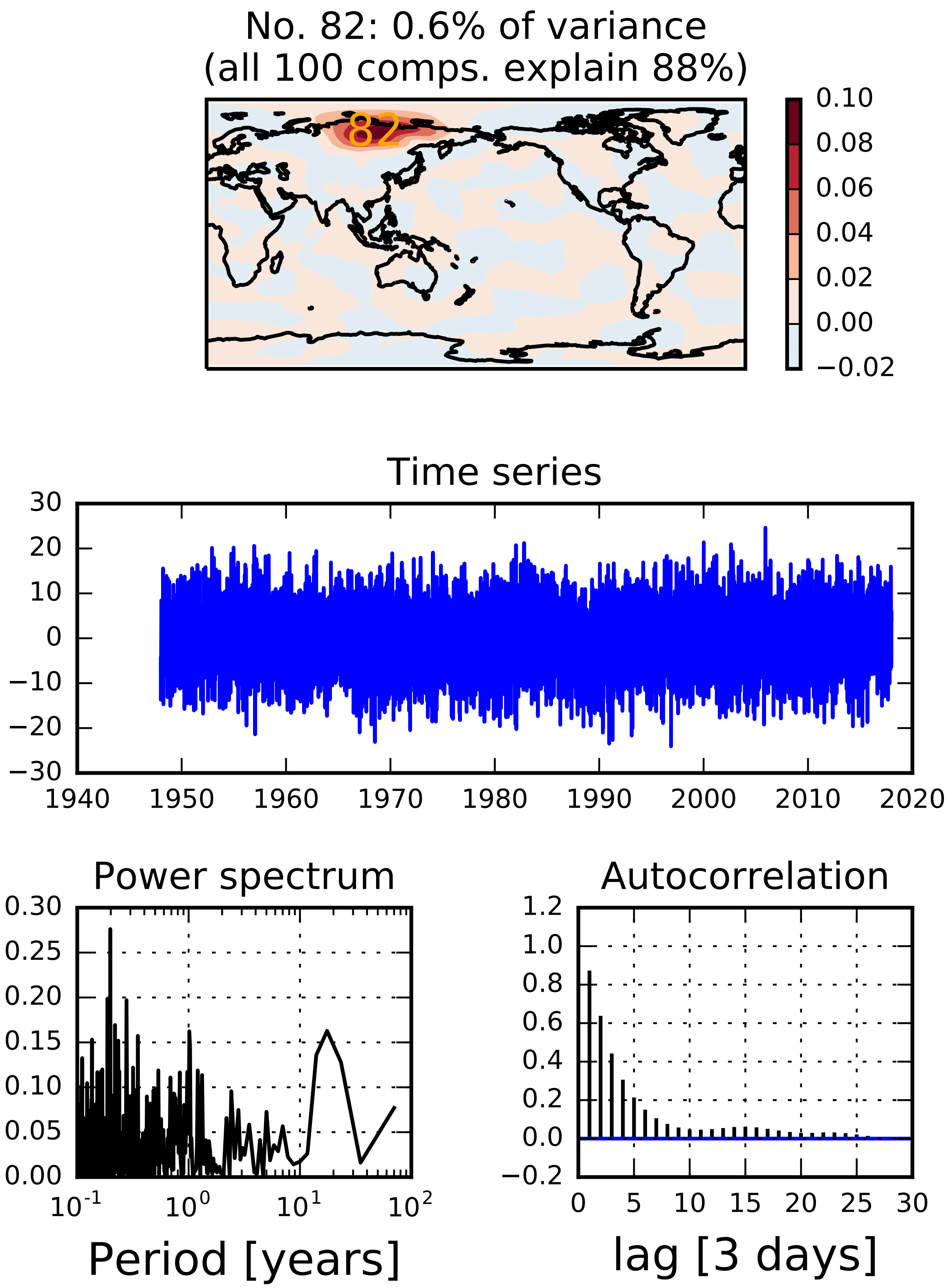
No. 82: 0.6% of variance
(all 100 comps. explain 88%)
82
0.10
0.08
0.06
0.04
0.02
0.00
−0.02
Time series
30
20
10
0
−10
−20
−30
1940
1950
1960
1970
1980
1990
2000
2010
2020
Power spectrum
0.30
0.25
0.20
0.15
0.10
0.05
0.00
10^{-1}
10^{0}
10^{1}
10^{2}
Period [years]
Autocorrelation
1.2
1.0
0.8
0.6
0.4
0.2
0.0
−0.2
0
5
10
15
20
25
30
lag [3 days]

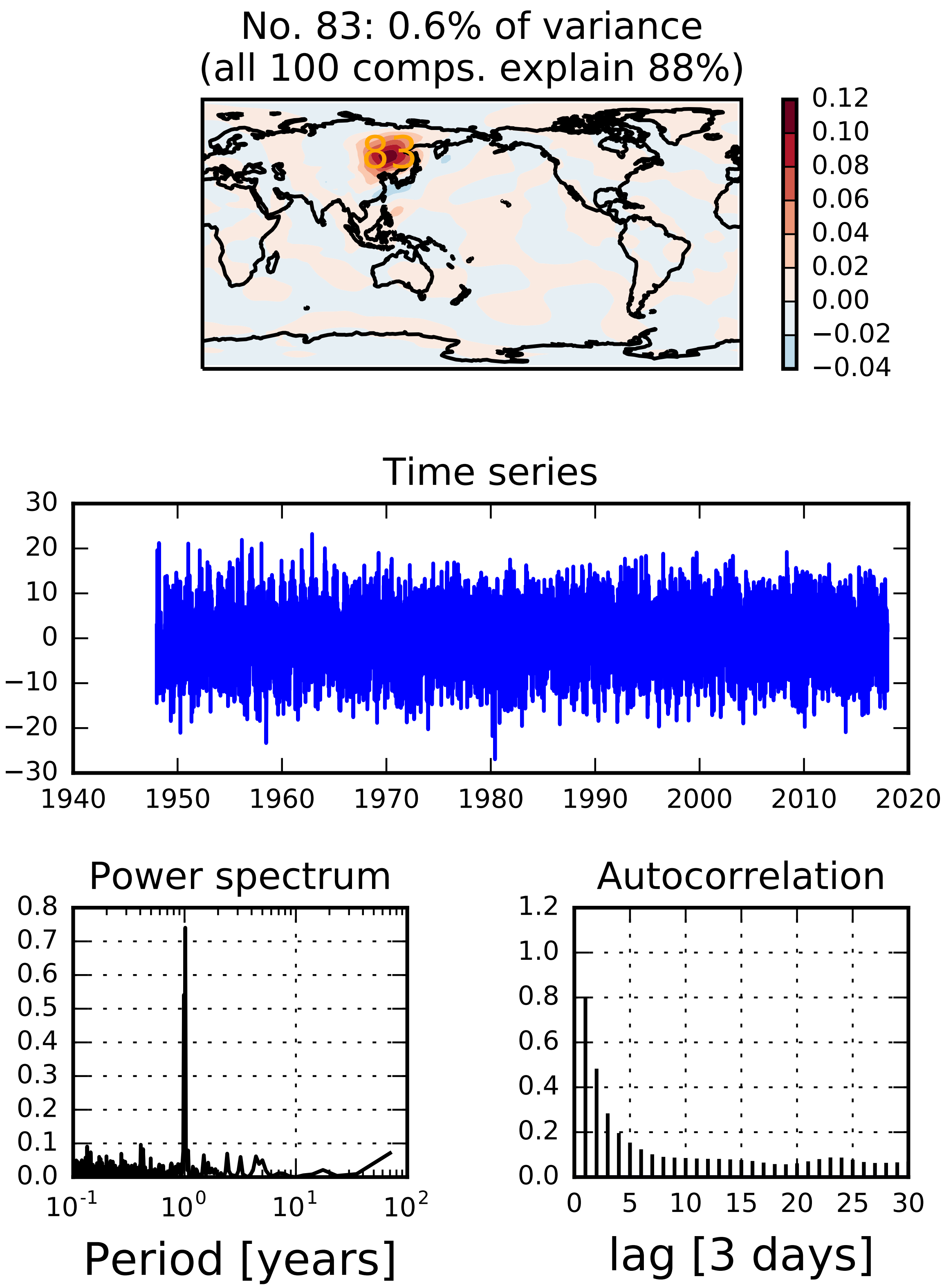
No. 83: 0.6% of variance
(all 100 comps. explain 88%)
83
0.12
0.10
0.08
0.06
0.04
0.02
0.00
−0.02
−0.04
Time series
30
20
10
0
−10
−20
−30
1940
1950
1960
1970
1980
1990
2000
2010
2020
Power spectrum
0.8
0.7
0.6
0.5
0.4
0.3
0.2
0.1
0.0
10^-1
10^0
10^1
10^2
Period [years]
Autocorrelation
1.2
1.0
0.8
0.6
0.4
0.2
0.0
0
5
10
15
20
25
30
lag [3 days]

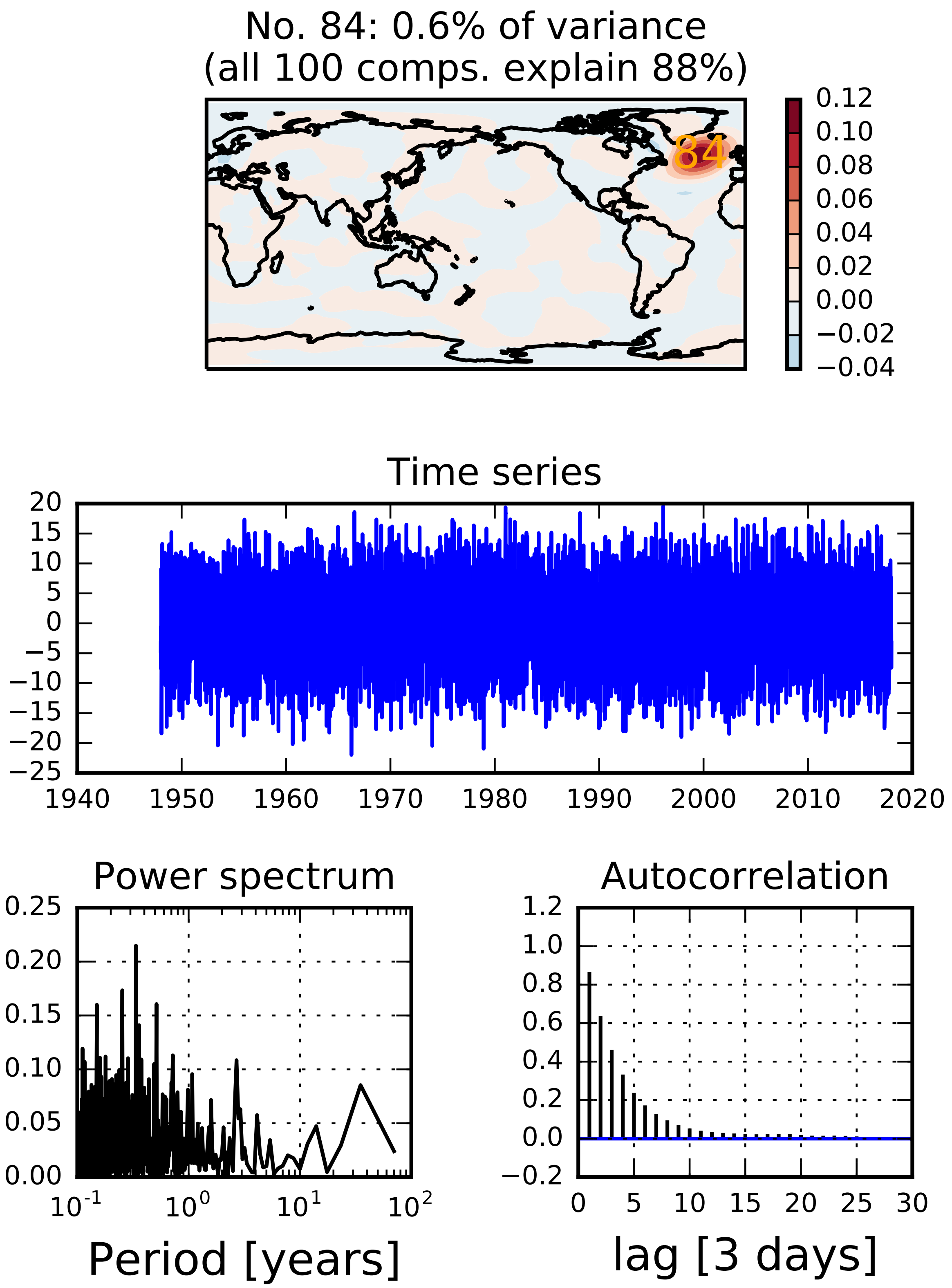
No. 84: 0.6% of variance
(all 100 comps. explain 88%)
84
0.12
0.10
0.08
0.06
0.04
0.02
0.00
−0.02
−0.04
Time series
20
15
10
5
0
−5
−10
−15
−20
−25
1940
1950
1960
1970
1980
1990
2000
2010
2020
Power spectrum
0.25
0.20
0.15
0.10
0.05
0.00
10^-1
10^0
10^1
10^2
Period [years]
Autocorrelation
1.2
1.0
0.8
0.6
0.4
0.2
0.0
−0.2
0
5
10
15
20
25
30
lag [3 days]

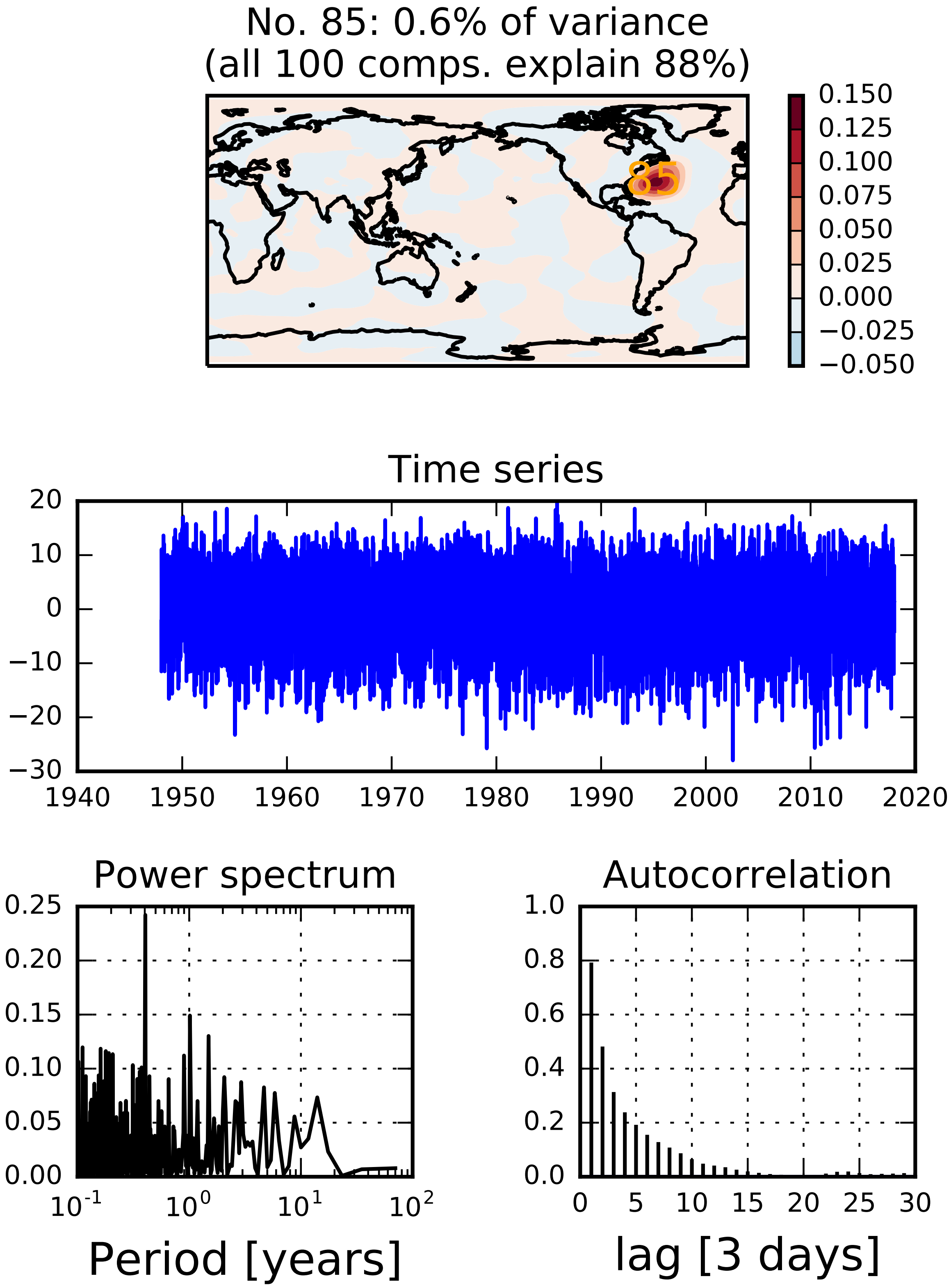
No. 85: 0.6% of variance
(all 100 comps. explain 88%)
85
0.150
0.125
0.100
0.075
0.050
0.025
0.000
−0.025
−0.050
Time series
20
10
0
−10
−20
−30
1940
1950
1960
1970
1980
1990
2000
2010
2020
Power spectrum
0.25
0.20
0.15
0.10
0.05
0.00
10^{-1}
10^{0}
10^{1}
10^{2}
Period [years]
Autocorrelation
1.0
0.8
0.6
0.4
0.2
0.0
0
5
10
15
20
25
30
lag [3 days]

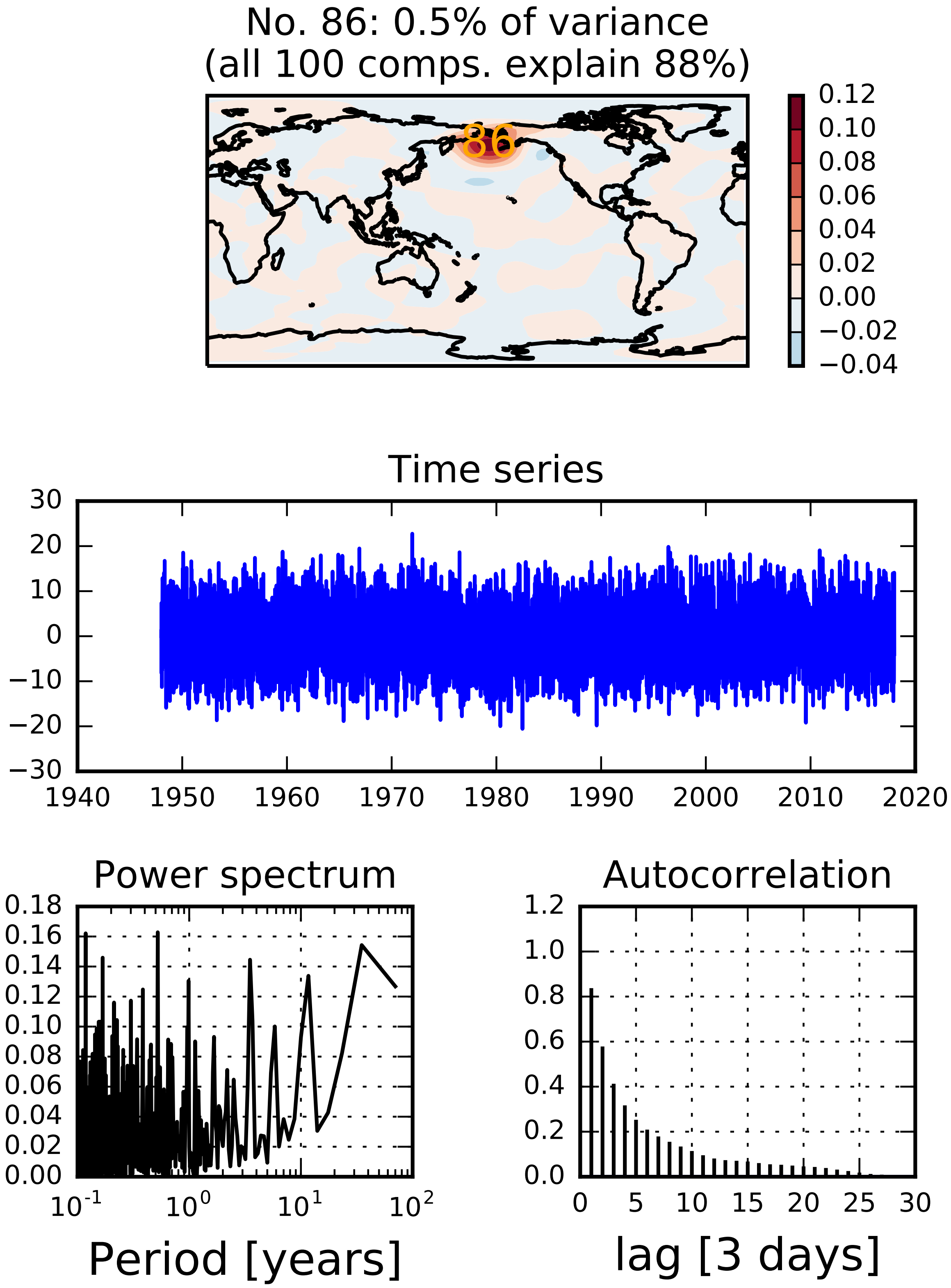
No. 86: 0.5% of variance
(all 100 comps. explain 88%)
86
0.12
0.10
0.08
0.06
0.04
0.02
0.00
−0.02
−0.04
Time series
30
20
10
0
−10
−20
−30
1940
1950
1960
1970
1980
1990
2000
2010
2020
Power spectrum
0.18
0.16
0.14
0.12
0.10
0.08
0.06
0.04
0.02
0.00
10^-1
10^0
10^1
10^2
Period [years]
Autocorrelation
1.2
1.0
0.8
0.6
0.4
0.2
0.0
0 5 10 15 20 25 30
lag [3 days]

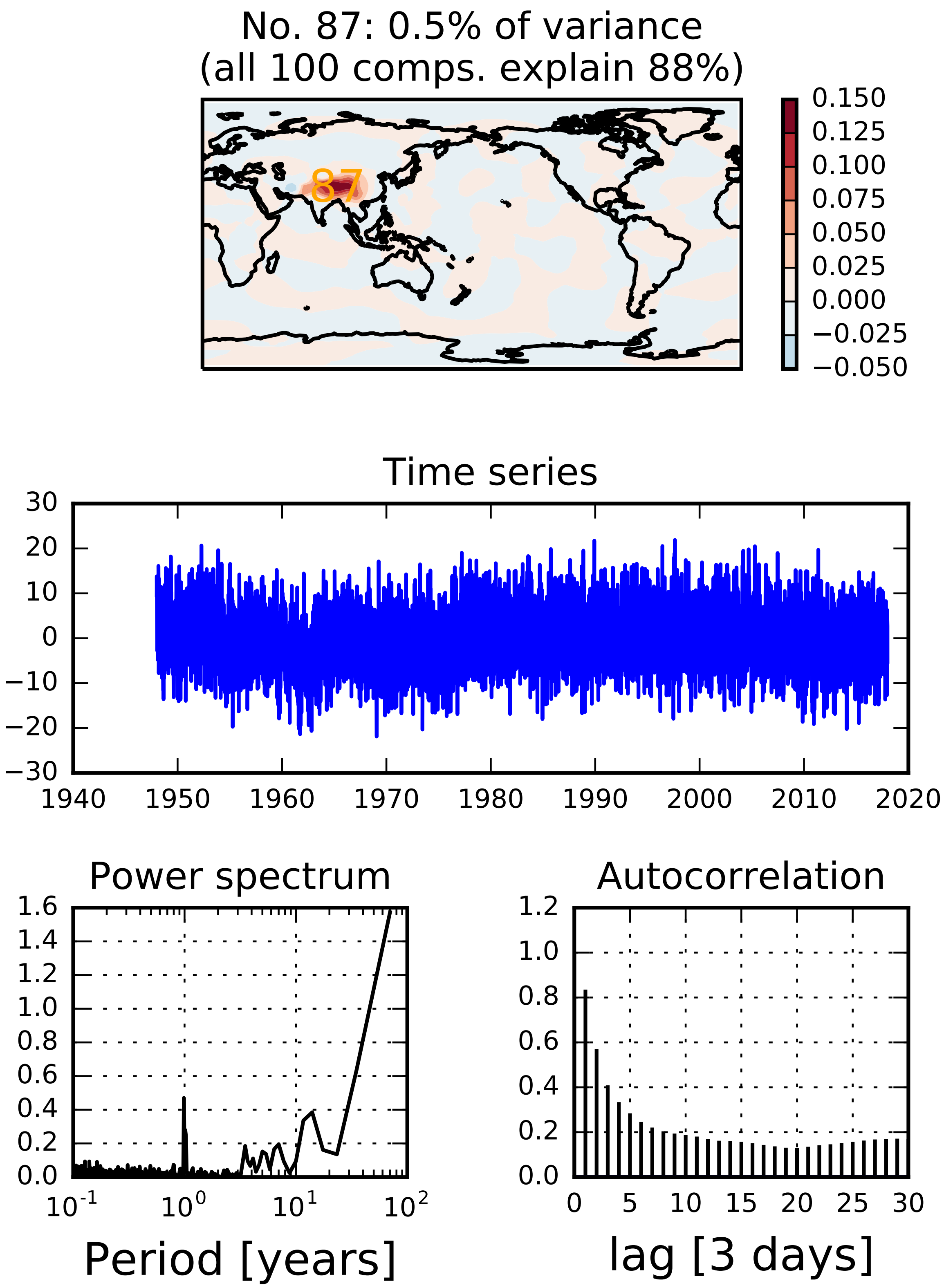
No. 87: 0.5% of variance
(all 100 comps. explain 88%)
87
0.150
0.125
0.100
0.075
0.050
0.025
0.000
−0.025
−0.050
Time series
30
20
10
0
−10
−20
−30
1940
1950
1960
1970
1980
1990
2000
2010
2020
Power spectrum
1.6
1.4
1.2
1.0
0.8
0.6
0.4
0.2
0.0
10^{-1}
10^{0}
10^{1}
10^{2}
Period [years]
Autocorrelation
1.2
1.0
0.8
0.6
0.4
0.2
0.0
0
5
10
15
20
25
30
lag [3 days]

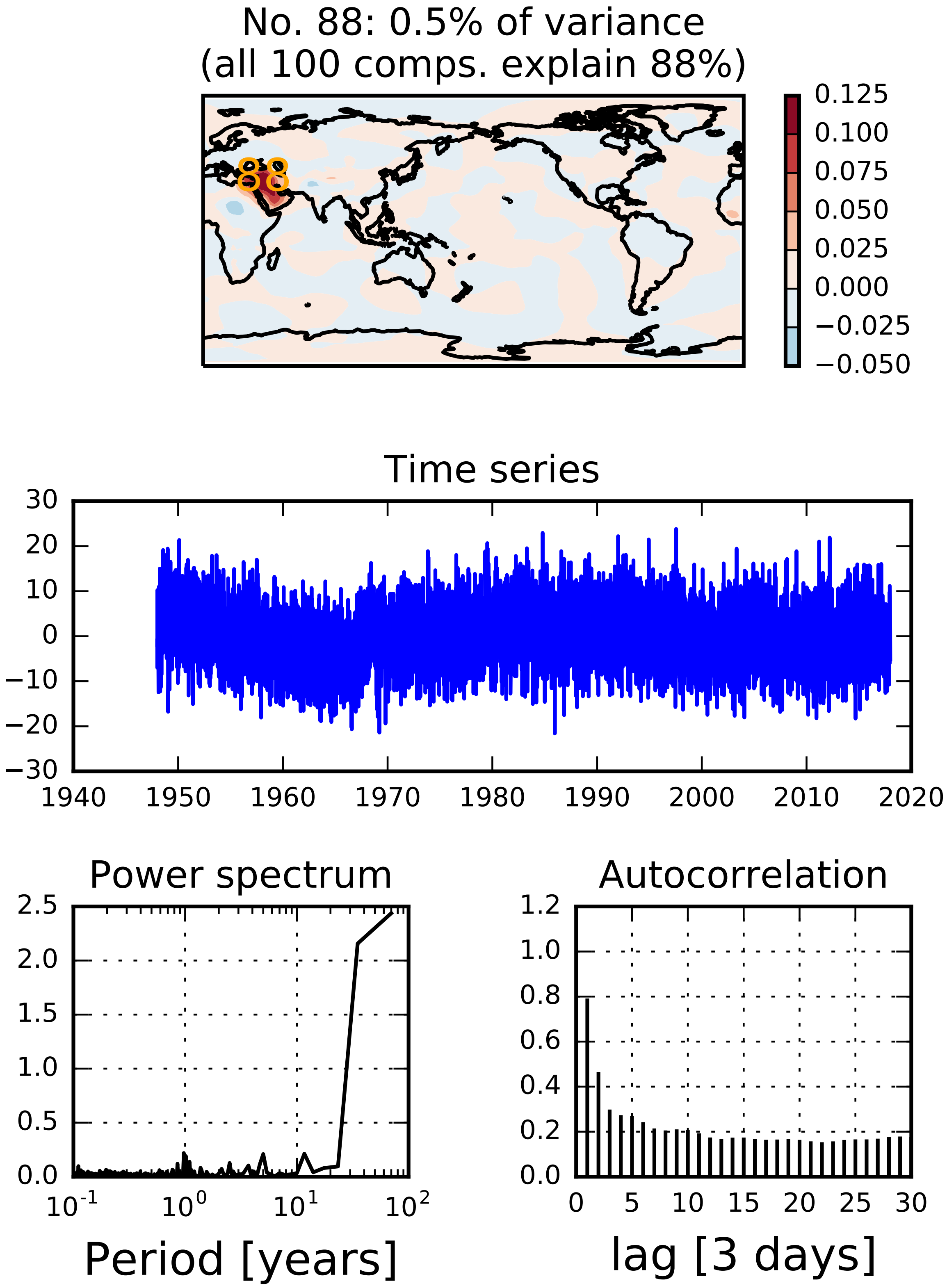
No. 88: 0.5% of variance
(all 100 comps. explain 88%)
0.125
0.100
0.075
0.050
0.025
0.000
−0.025
−0.050
88
Time series
30
20
10
0
−10
−20
−30
1940
1950
1960
1970
1980
1990
2000
2010
2020
Power spectrum
2.5
2.0
1.5
1.0
0.5
0.0
10^-1
10^0
10^1
10^2
Period [years]
Autocorrelation
1.2
1.0
0.8
0.6
0.4
0.2
0.0
0
5
10
15
20
25
30
lag [3 days]

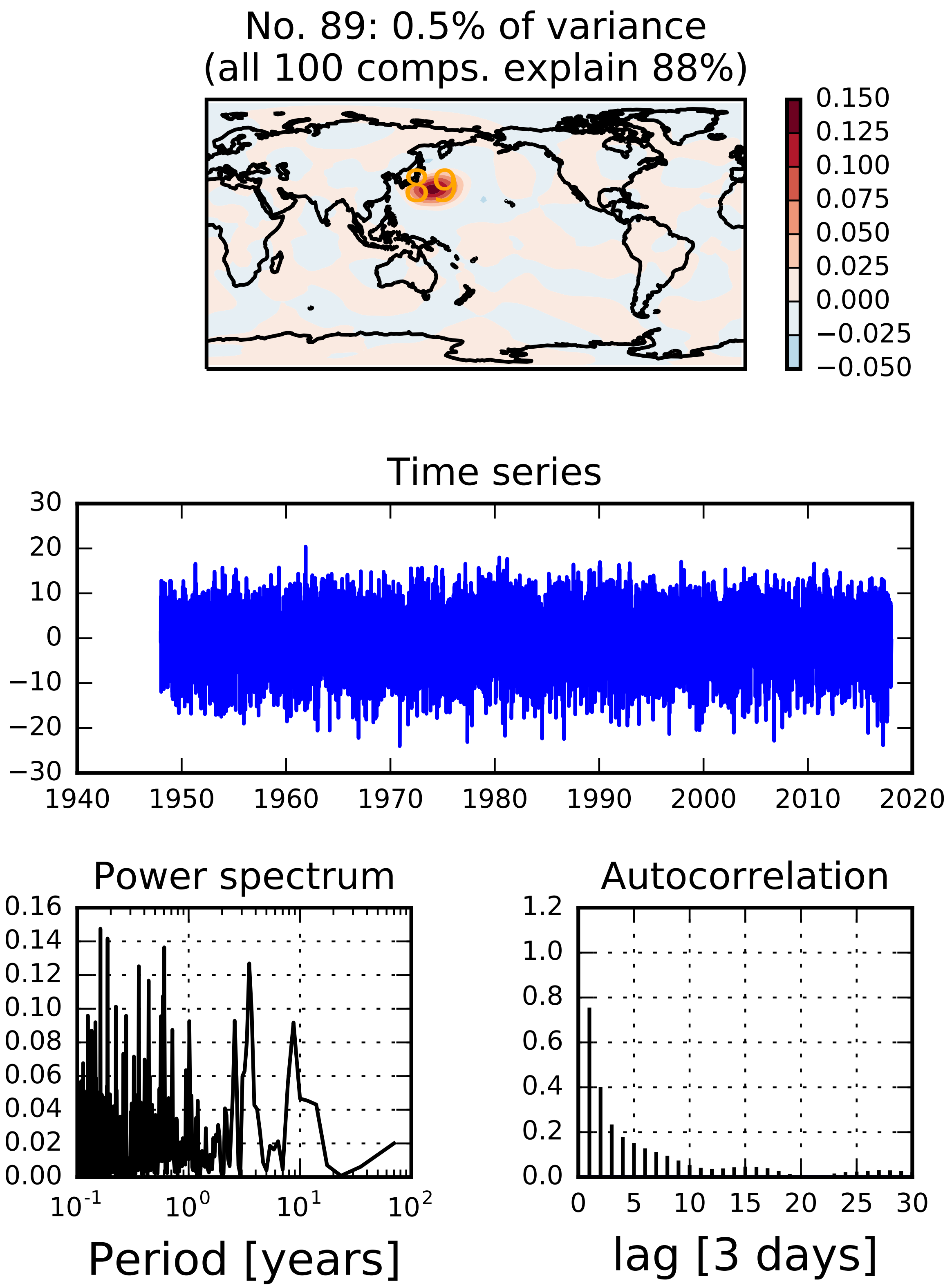
No. 89: 0.5% of variance
(all 100 comps. explain 88%)
89
0.150
0.125
0.100
0.075
0.050
0.025
0.000
−0.025
−0.050
Time series
30
20
10
0
−10
−20
−30
1940
1950
1960
1970
1980
1990
2000
2010
2020
Power spectrum
0.16
0.14
0.12
0.10
0.08
0.06
0.04
0.02
0.00
10^{-1}
10^{0}
10^{1}
10^{2}
Period [years]
Autocorrelation
1.2
1.0
0.8
0.6
0.4
0.2
0.0
0
5
10
15
20
25
30
lag [3 days]

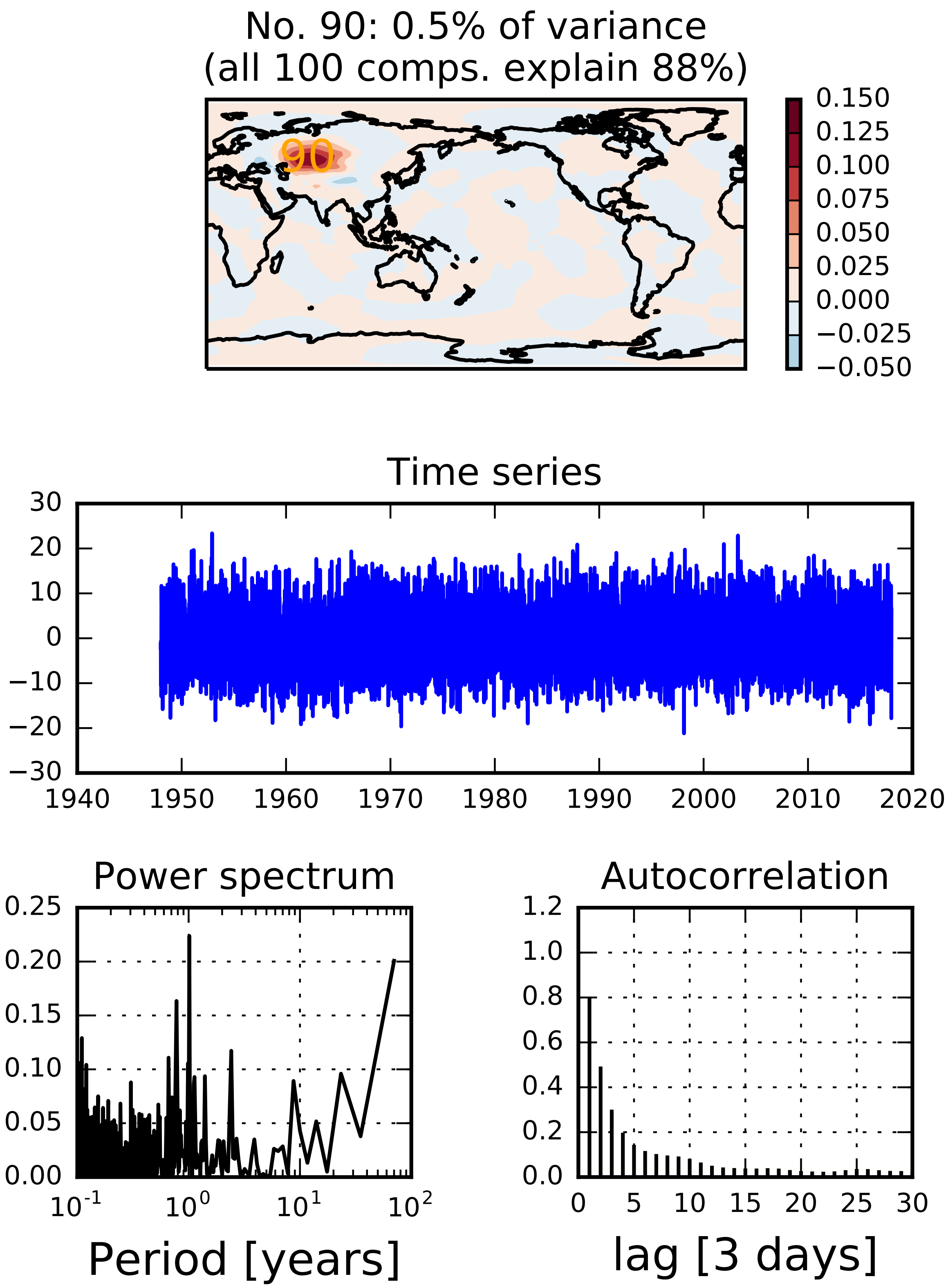
No. 90: 0.5% of variance
(all 100 comps. explain 88%)
90
0.150
0.125
0.100
0.075
0.050
0.025
0.000
−0.025
−0.050
Time series
30
20
10
0
−10
−20
−30
1940
1950
1960
1970
1980
1990
2000
2010
2020
Power spectrum
0.25
0.20
0.15
0.10
0.05
0.00
10^{-1}
10^{0}
10^{1}
10^{2}
Period [years]
Autocorrelation
1.2
1.0
0.8
0.6
0.4
0.2
0.0
0
5
10
15
20
25
30
lag [3 days]

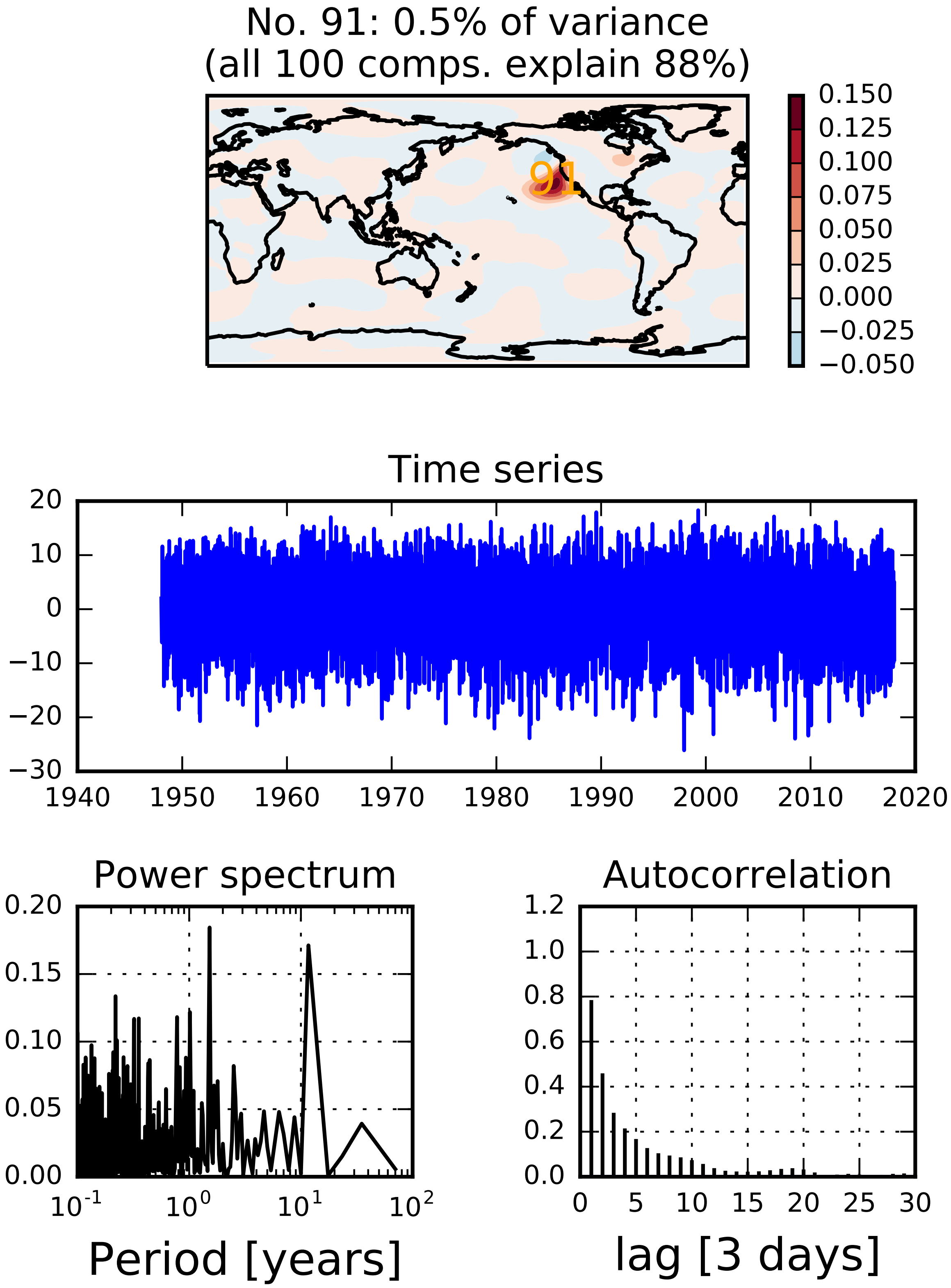
No. 91: 0.5% of variance
(all 100 comps. explain 88%)
91
0.150
0.125
0.100
0.075
0.050
0.025
0.000
−0.025
−0.050
Time series
20
10
0
−10
−20
−30
1940
1950
1960
1970
1980
1990
2000
2010
2020
Power spectrum
0.20
0.15
0.10
0.05
0.00
10^{-1}
10^{0}
10^{1}
10^{2}
Period [years]
Autocorrelation
1.2
1.0
0.8
0.6
0.4
0.2
0.0
0
5
10
15
20
25
30
lag [3 days]

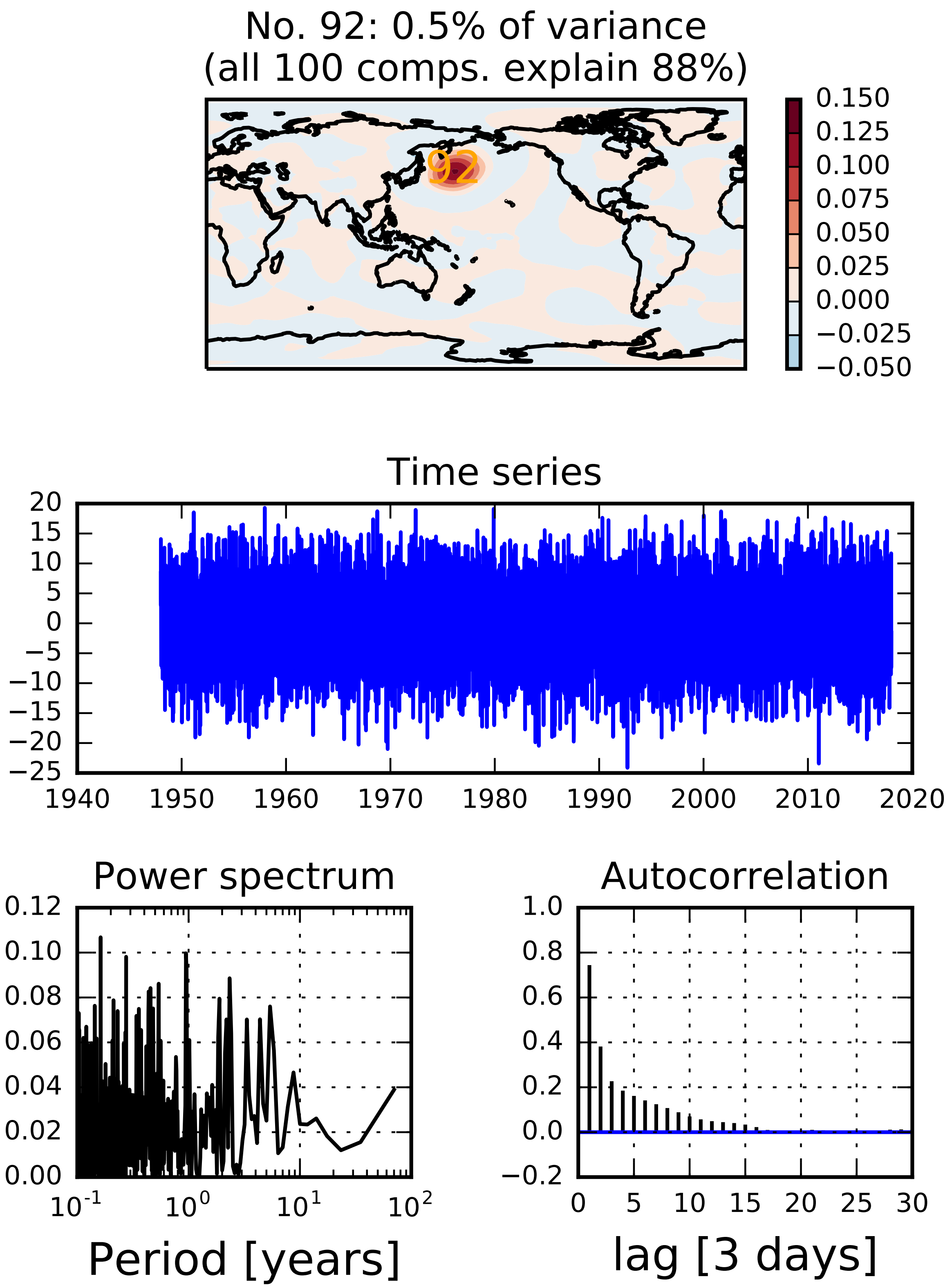
No. 92: 0.5% of variance
(all 100 comps. explain 88%)
92
0.150
0.125
0.100
0.075
0.050
0.025
0.000
−0.025
−0.050
Time series
20
15
10
5
0
−5
−10
−15
−20
−25
1940
1950
1960
1970
1980
1990
2000
2010
2020
Power spectrum
0.12
0.10
0.08
0.06
0.04
0.02
0.00
10^{-1}
10^{0}
10^{1}
10^{2}
Period [years]
Autocorrelation
1.0
0.8
0.6
0.4
0.2
0.0
−0.2
0
5
10
15
20
25
30
lag [3 days]

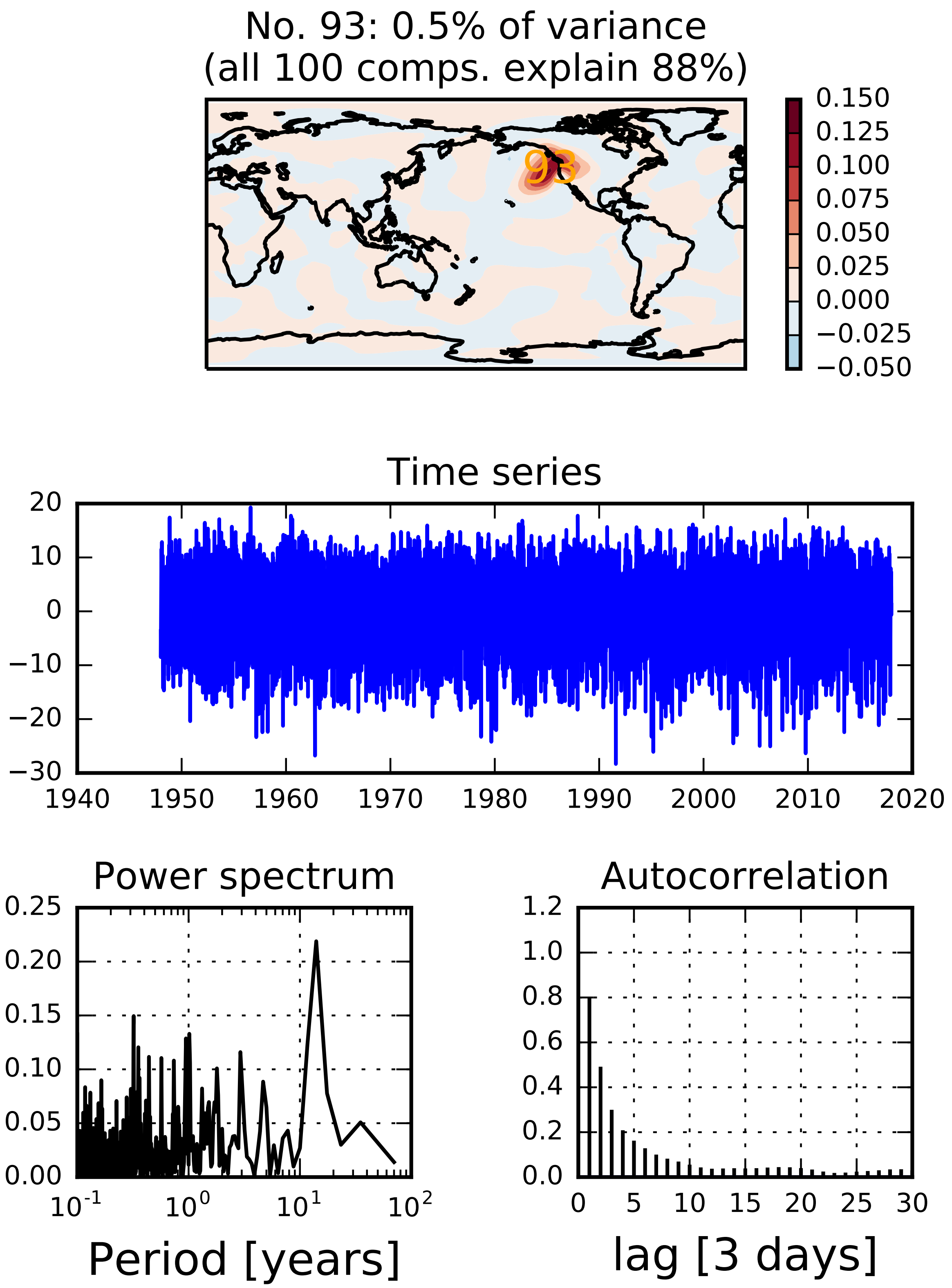
No. 93: 0.5% of variance
(all 100 comps. explain 88%)
93
0.150
0.125
0.100
0.075
0.050
0.025
0.000
−0.025
−0.050
Time series
20
10
0
−10
−20
−30
1940
1950
1960
1970
1980
1990
2000
2010
2020
Power spectrum
0.25
0.20
0.15
0.10
0.05
0.00
10^{-1}
10^{0}
10^{1}
10^{2}
Period [years]
Autocorrelation
1.2
1.0
0.8
0.6
0.4
0.2
0.0
0
5
10
15
20
25
30
lag [3 days]

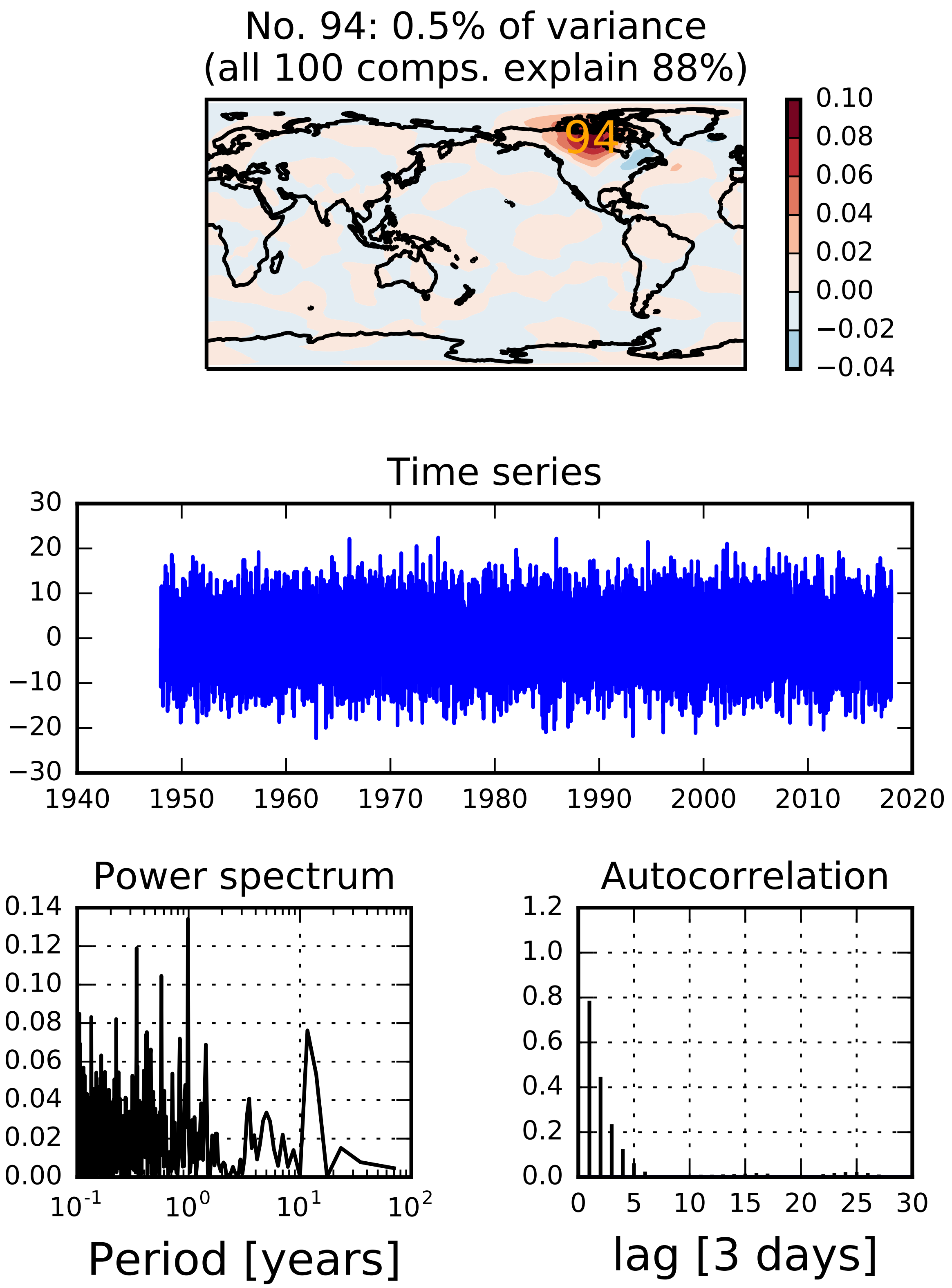
No. 94: 0.5% of variance
(all 100 comps. explain 88%)
94
0.10
0.08
0.06
0.04
0.02
0.00
−0.02
−0.04
Time series
30
20
10
0
−10
−20
−30
1940
1950
1960
1970
1980
1990
2000
2010
2020
Power spectrum
0.14
0.12
0.10
0.08
0.06
0.04
0.02
0.00
10^{-1}
10^{0}
10^{1}
10^{2}
Period [years]
Autocorrelation
1.2
1.0
0.8
0.6
0.4
0.2
0.0
0
5
10
15
20
25
30
lag [3 days]

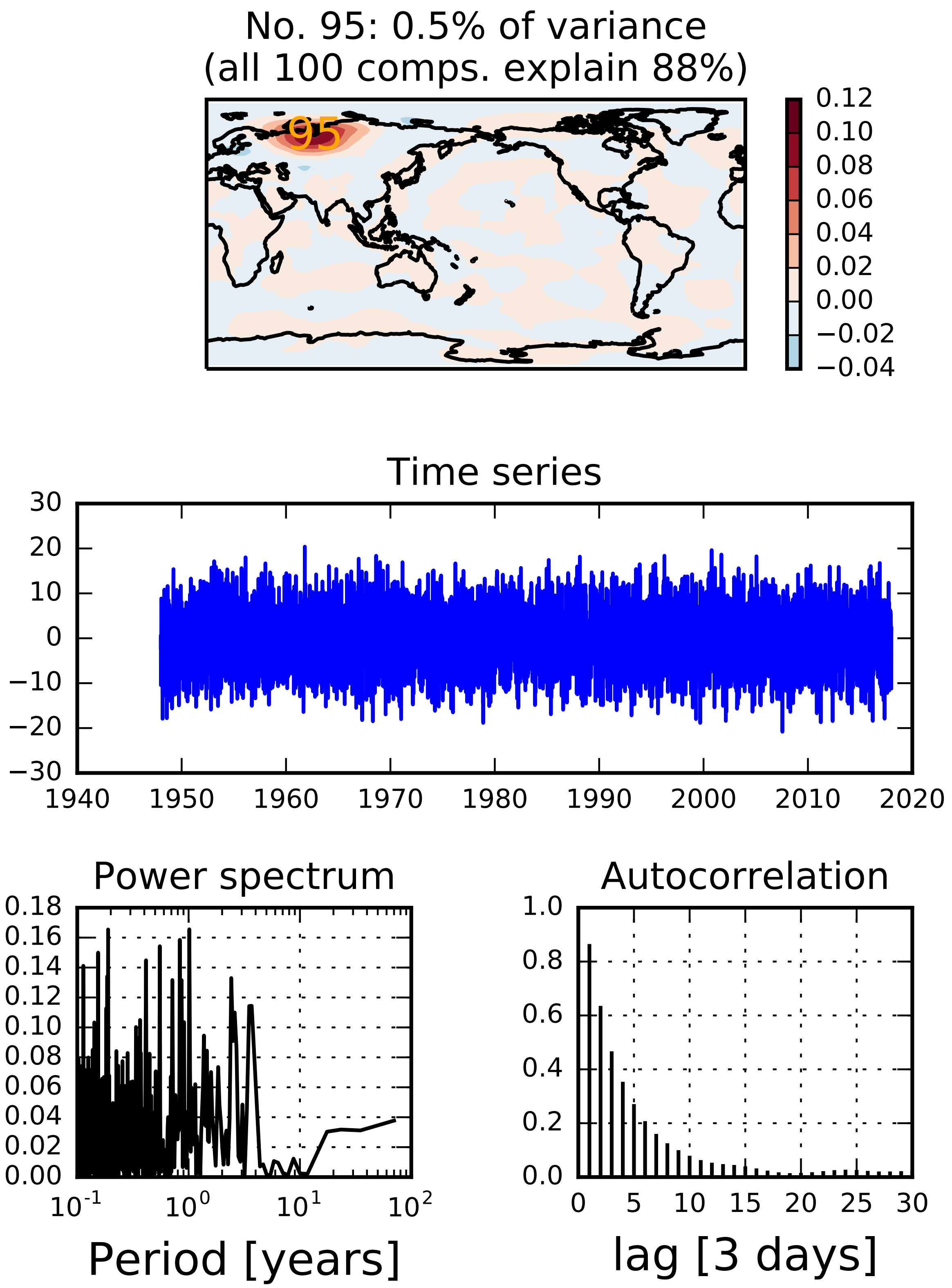
No. 95: 0.5% of variance
(all 100 comps. explain 88%)
95
0.12
0.10
0.08
0.06
0.04
0.02
0.00
−0.02
−0.04
Time series
30
20
10
0
−10
−20
−30
1940
1950
1960
1970
1980
1990
2000
2010
2020
Power spectrum
0.18
0.16
0.14
0.12
0.10
0.08
0.06
0.04
0.02
0.00
10^{-1}
10^{0}
10^{1}
10^{2}
Period [years]
Autocorrelation
1.0
0.8
0.6
0.4
0.2
0.0
0
5
10
15
20
25
30
lag [3 days]

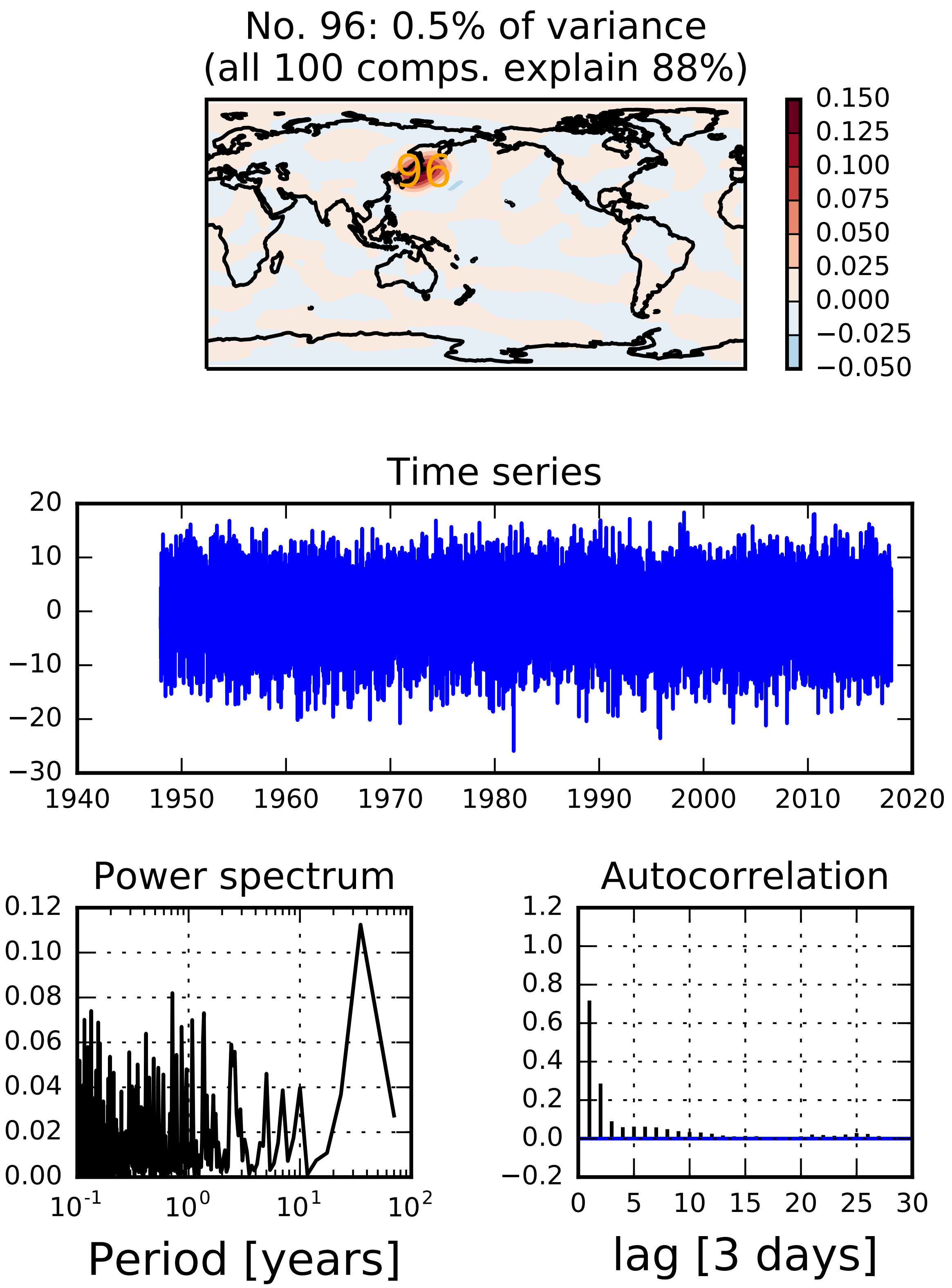
No. 96: 0.5% of variance
(all 100 comps. explain 88%)
96
0.150
0.125
0.100
0.075
0.050
0.025
0.000
−0.025
−0.050
Time series
20
10
0
−10
−20
−30
1940
1950
1960
1970
1980
1990
2000
2010
2020
Power spectrum
0.12
0.10
0.08
0.06
0.04
0.02
0.00
10^-1
10^0
10^1
10^2
Period [years]
Autocorrelation
1.2
1.0
0.8
0.6
0.4
0.2
0.0
−0.2
0
5
10
15
20
25
30
lag [3 days]

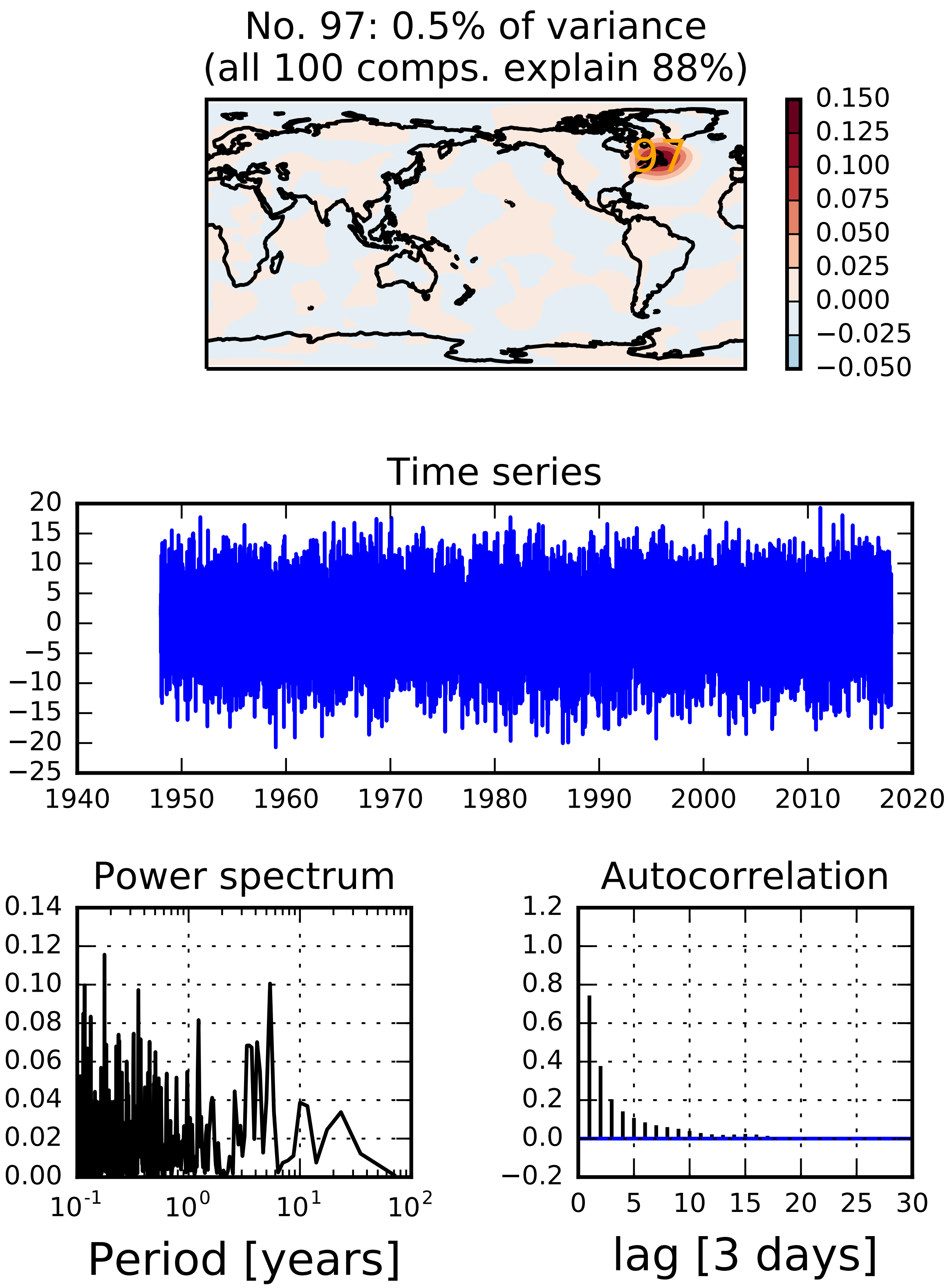
No. 97: 0.5% of variance
(all 100 comps. explain 88%)
97
0.150
0.125
0.100
0.075
0.050
0.025
0.000
−0.025
−0.050
Time series
20
15
10
5
0
−5
−10
−15
−20
−25
1940
1950
1960
1970
1980
1990
2000
2010
2020
Power spectrum
0.14
0.12
0.10
0.08
0.06
0.04
0.02
0.00
10^-1
10^0
10^1
10^2
Period [years]
Autocorrelation
1.2
1.0
0.8
0.6
0.4
0.2
0.0
−0.2
0
5
10
15
20
25
30
lag [3 days]

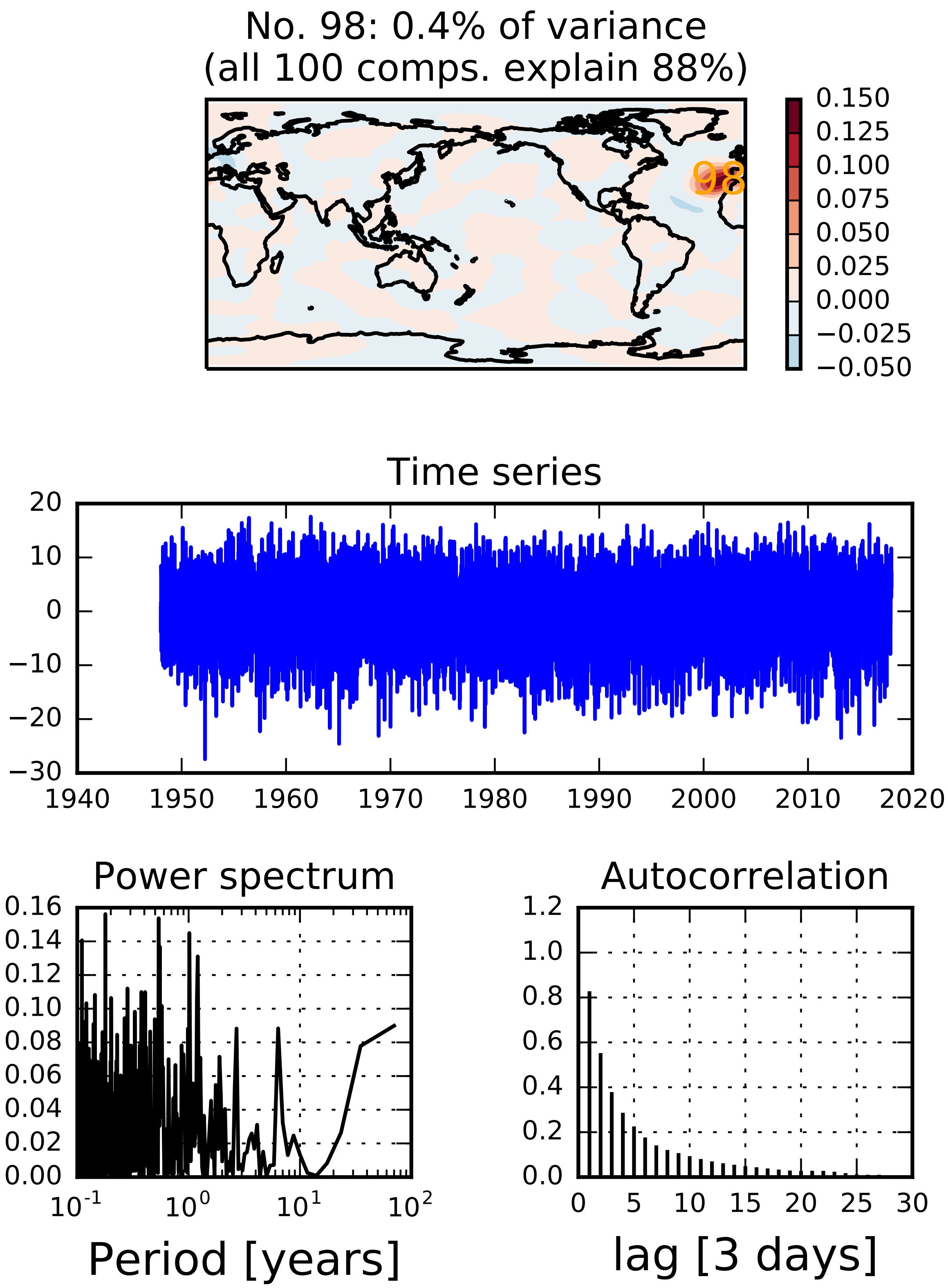
No. 98: 0.4% of variance
(all 100 comps. explain 88%)
98
0.150
0.125
0.100
0.075
0.050
0.025
0.000
−0.025
−0.050
Time series
20
10
0
−10
−20
−30
1940
1950
1960
1970
1980
1990
2000
2010
2020
Power spectrum
0.16
0.14
0.12
0.10
0.08
0.06
0.04
0.02
0.00
10^{-1}
10^{0}
10^{1}
10^{2}
Period [years]
Autocorrelation
1.2
1.0
0.8
0.6
0.4
0.2
0.0
0
5
10
15
20
25
30
lag [3 days]

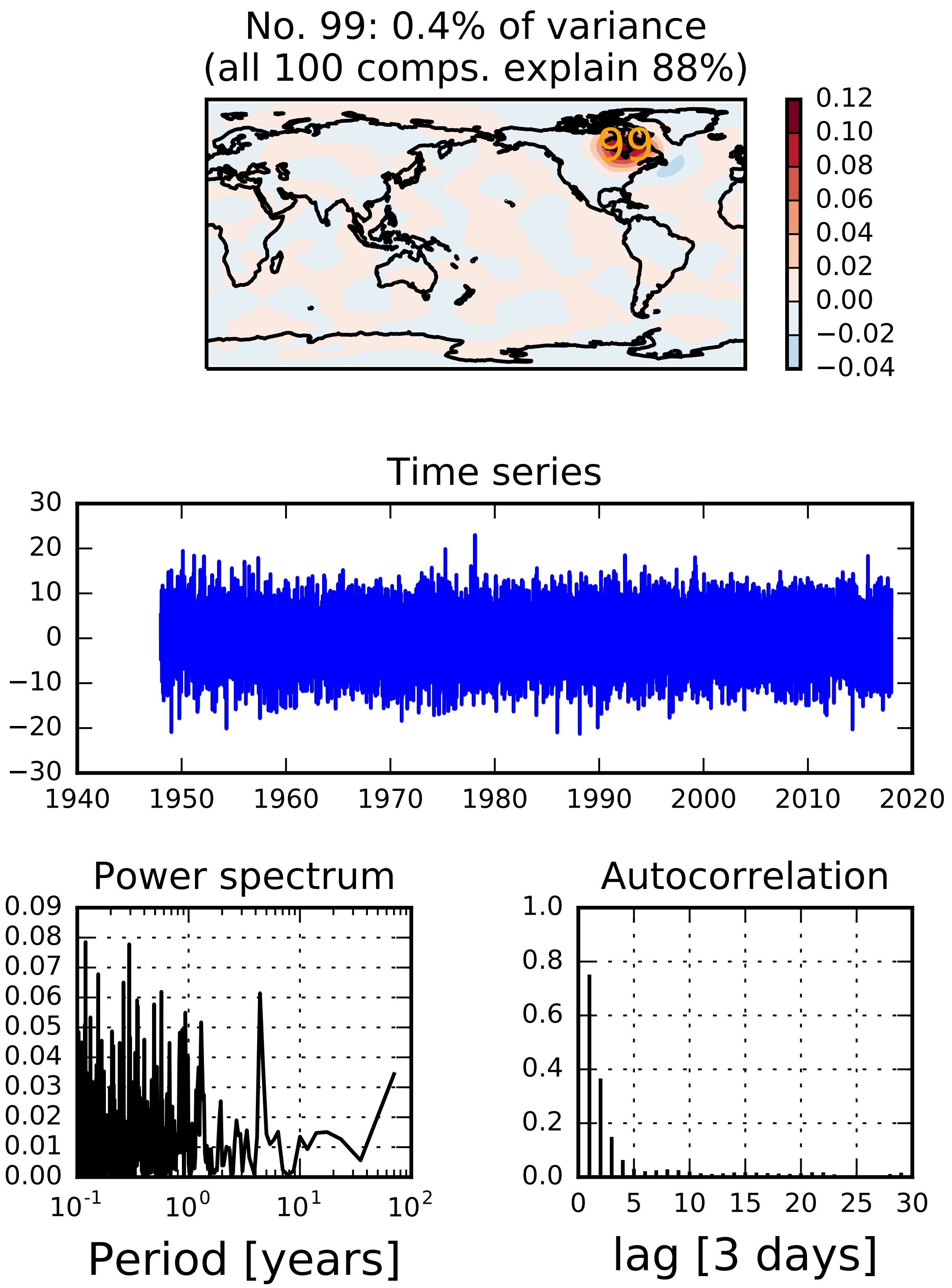
No. 99: 0.4% of variance
(all 100 comps. explain 88%)
99
0.12
0.10
0.08
0.06
0.04
0.02
0.00
−0.02
−0.04
Time series
30
20
10
0
−10
−20
−30
1940
1950
1960
1970
1980
1990
2000
2010
2020
Power spectrum
0.09
0.08
0.07
0.06
0.05
0.04
0.03
0.02
0.01
0.00
10^-1
10^0
10^1
10^2
Period [years]
Autocorrelation
1.0
0.8
0.6
0.4
0.2
0.0
0
5
10
15
20
25
30
lag [3 days]

Supplementary Figures 322 to 421

SON PCA Varimax components. For each component, we provide the component index and the component's spatial loadings (top subfigure), the component time series (centre) as well as the component's power spectrum (bottom left) and autocorrelation properties (bottom right). For each component we further provide the fraction of explained variance as defined prior to the Varimax rotation.

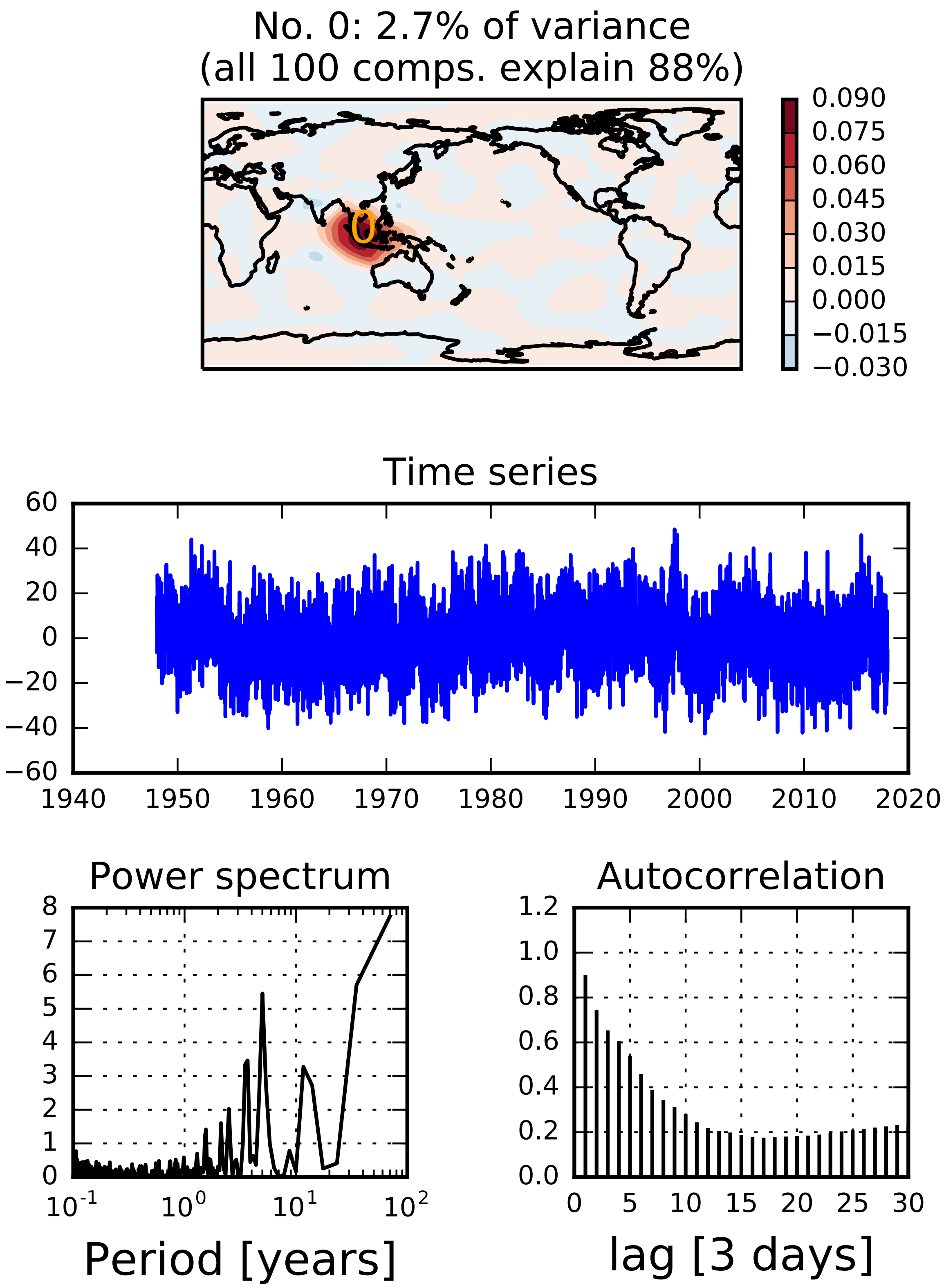
No. 0: 2.7% of variance
(all 100 comps. explain 88%)
0
0.090
0.075
0.060
0.045
0.030
0.015
0.000
−0.015
−0.030
Time series
60
40
20
0
−20
−40
−60
1940
1950
1960
1970
1980
1990
2000
2010
2020
Power spectrum
8
7
6
5
4
3
2
1
0
10^{-1}
10^{0}
10^{1}
10^{2}
Period [years]
Autocorrelation
1.2
1.0
0.8
0.6
0.4
0.2
0.0
0
5
10
15
20
25
30
lag [3 days]

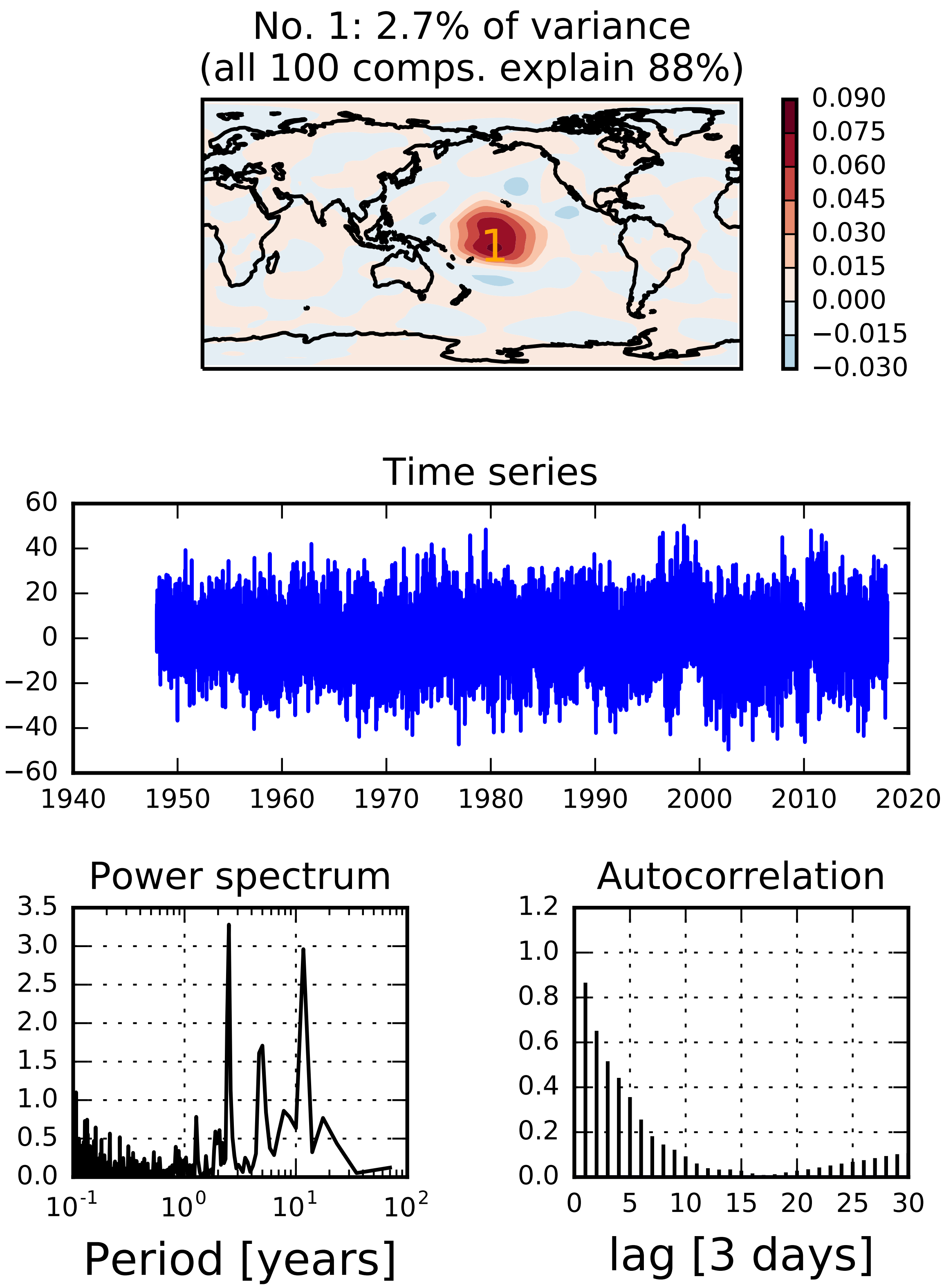
No. 1: 2.7% of variance
(all 100 comps. explain 88%)
1
0.090
0.075
0.060
0.045
0.030
0.015
0.000
−0.015
−0.030
Time series
60
40
20
0
−20
−40
−60
1940
1950
1960
1970
1980
1990
2000
2010
2020
Power spectrum
3.5
3.0
2.5
2.0
1.5
1.0
0.5
0.0
10^{-1}
10^{0}
10^{1}
10^{2}
Period [years]
Autocorrelation
1.2
1.0
0.8
0.6
0.4
0.2
0.0
0
5
10
15
20
25
30
lag [3 days]

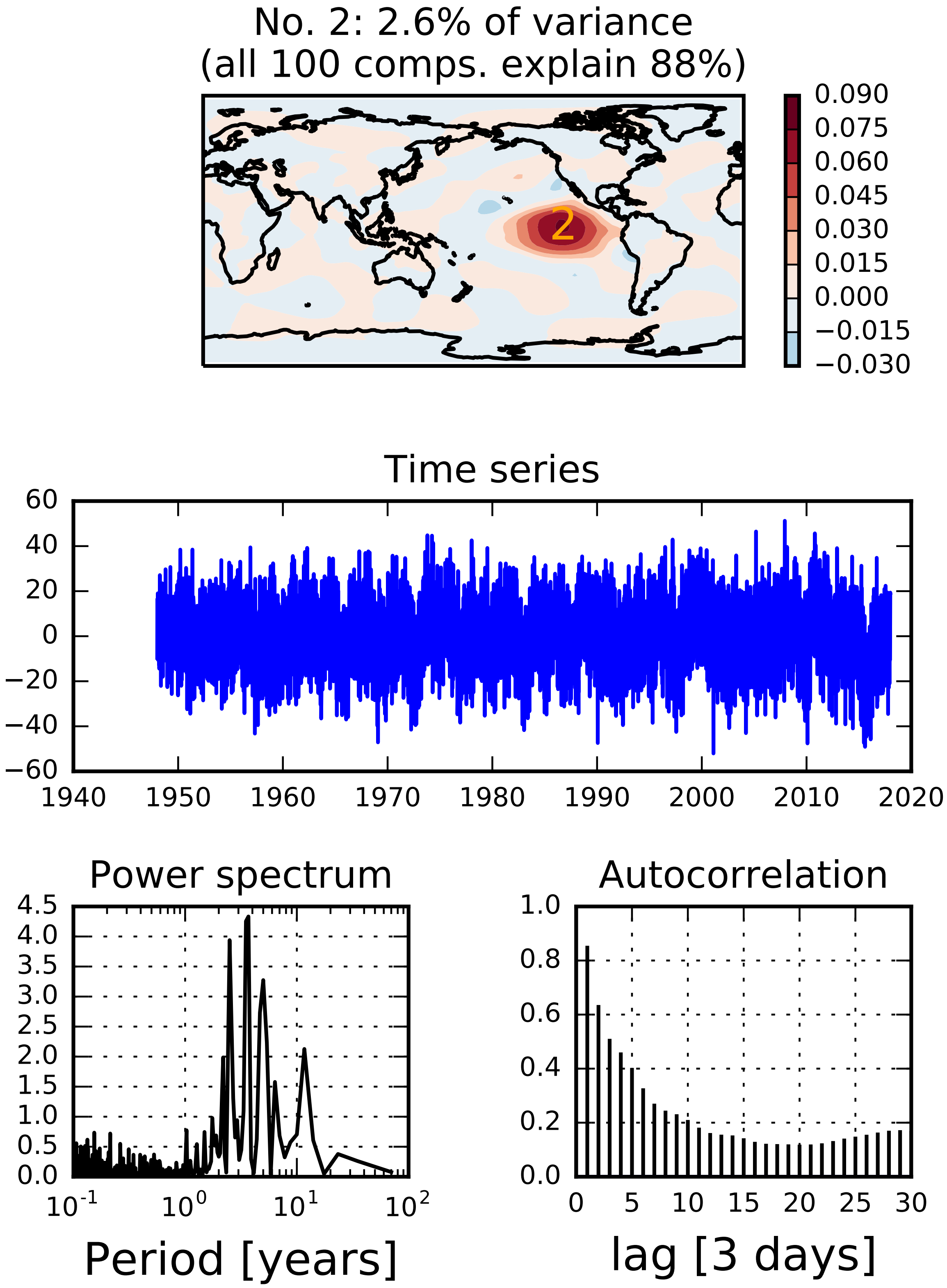
No. 2: 2.6% of variance
(all 100 comps. explain 88%)
2
0.090
0.075
0.060
0.045
0.030
0.015
0.000
−0.015
−0.030
Time series
60
40
20
0
−20
−40
−60
1940
1950
1960
1970
1980
1990
2000
2010
2020
Power spectrum
4.5
4.0
3.5
3.0
2.5
2.0
1.5
1.0
0.5
0.0
10-1
100
101
102
Period [years]
Autocorrelation
1.0
0.8
0.6
0.4
0.2
0.0
0
5
10
15
20
25
30
lag [3 days]

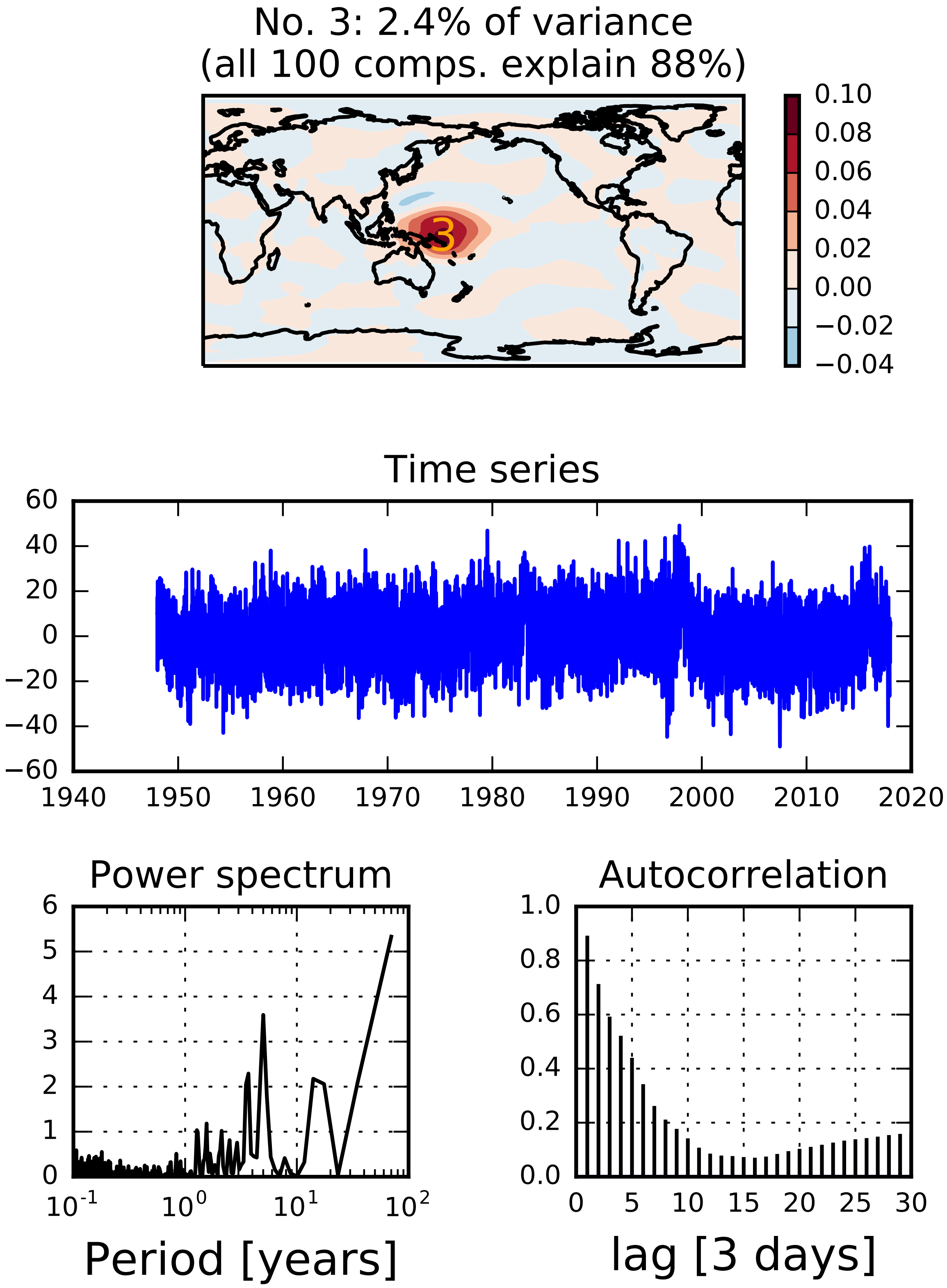

No. 3: 2.4% of variance
(all 100 comps. explain 88%)
3
0.10
0.08
0.06
0.04
0.02
0.00
−0.02
−0.04
Time series
60
40
20
0
−20
−40
−60
1940
1950
1960
1970
1980
1990
2000
2010
2020
Power spectrum
6
5
4
3
2
1
0
10^{-1}
10^{0}
10^{1}
10^{2}
Period [years]
Autocorrelation
1.0
0.8
0.6
0.4
0.2
0.0
0
5
10
15
20
25
30
lag [3 days]

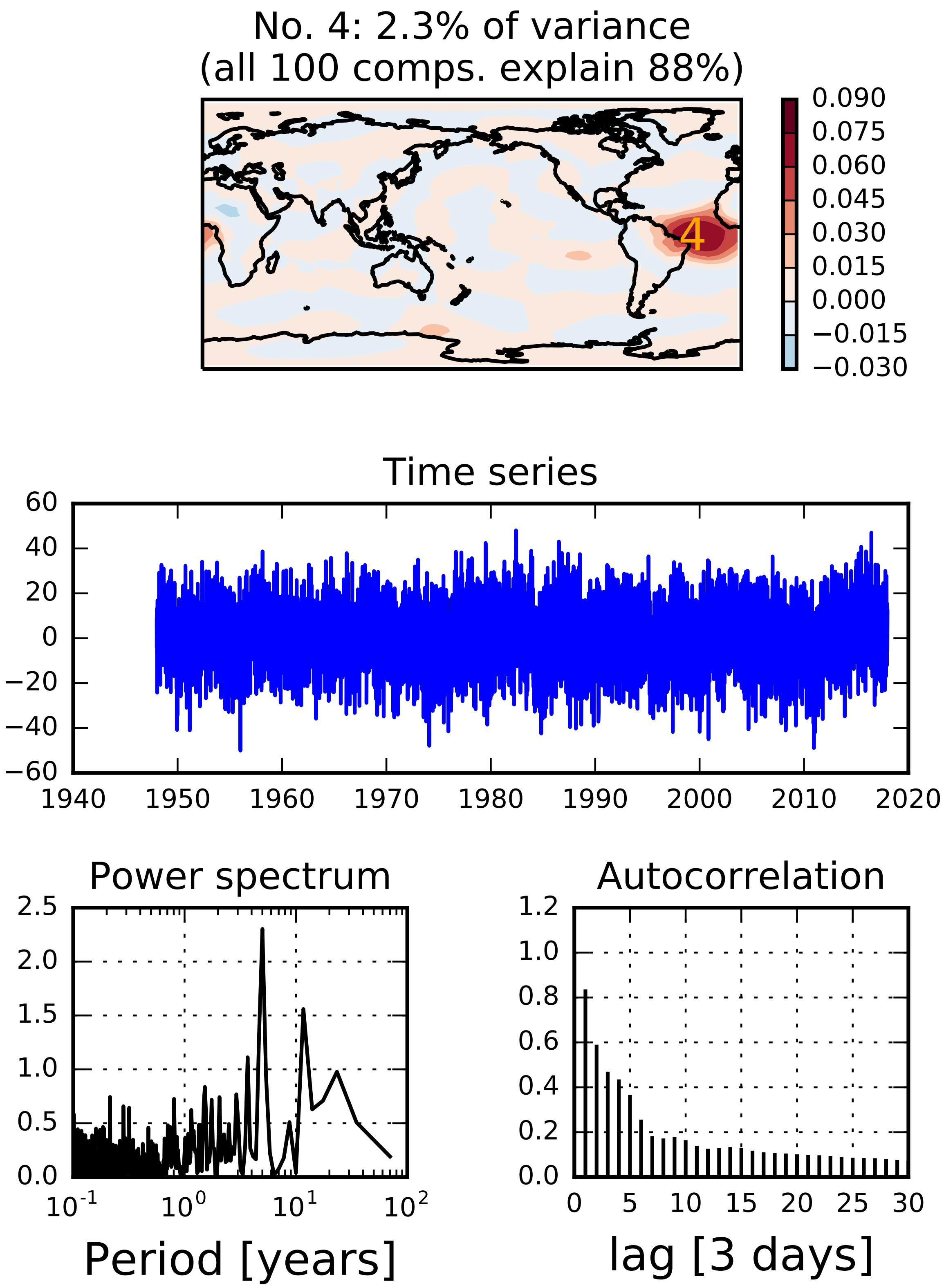
No. 4: 2.3% of variance
(all 100 comps. explain 88%)
4
0.090
0.075
0.060
0.045
0.030
0.015
0.000
−0.015
−0.030
Time series
60
40
20
0
−20
−40
−60
1940
1950
1960
1970
1980
1990
2000
2010
2020
Power spectrum
2.5
2.0
1.5
1.0
0.5
0.0
10-1
100
101
102
Period [years]
Autocorrelation
1.2
1.0
0.8
0.6
0.4
0.2
0.0
0
5
10
15
20
25
30
lag [3 days]

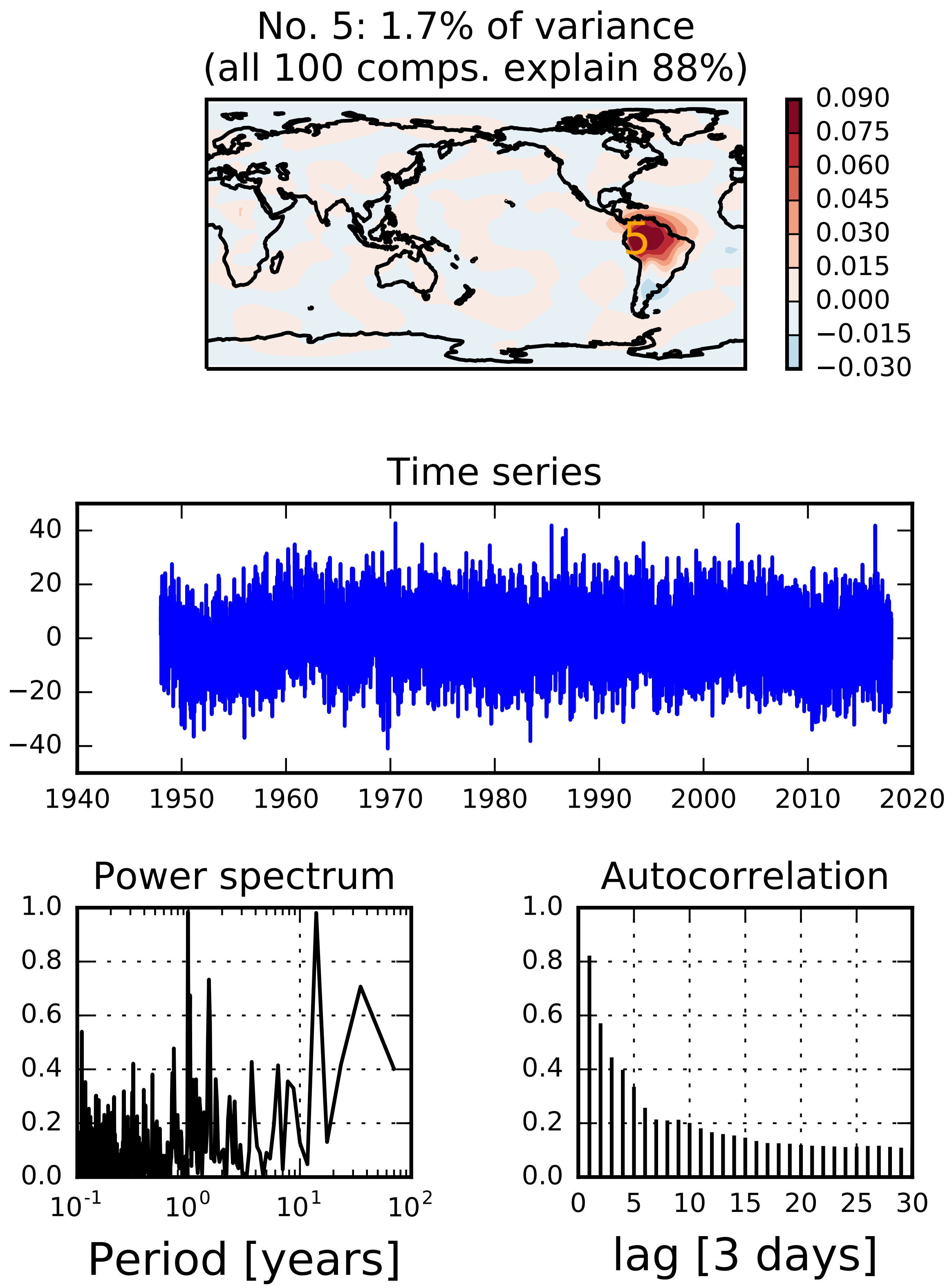
No. 5: 1.7% of variance
(all 100 comps. explain 88%)
5
0.090
0.075
0.060
0.045
0.030
0.015
0.000
−0.015
−0.030
Time series
40
20
0
−20
−40
1940
1950
1960
1970
1980
1990
2000
2010
2020
Power spectrum
1.0
0.8
0.6
0.4
0.2
0.0
10^-1
10^0
10^1
10^2
Period [years]
Autocorrelation
0
5
10
15
20
25
30
lag [3 days]

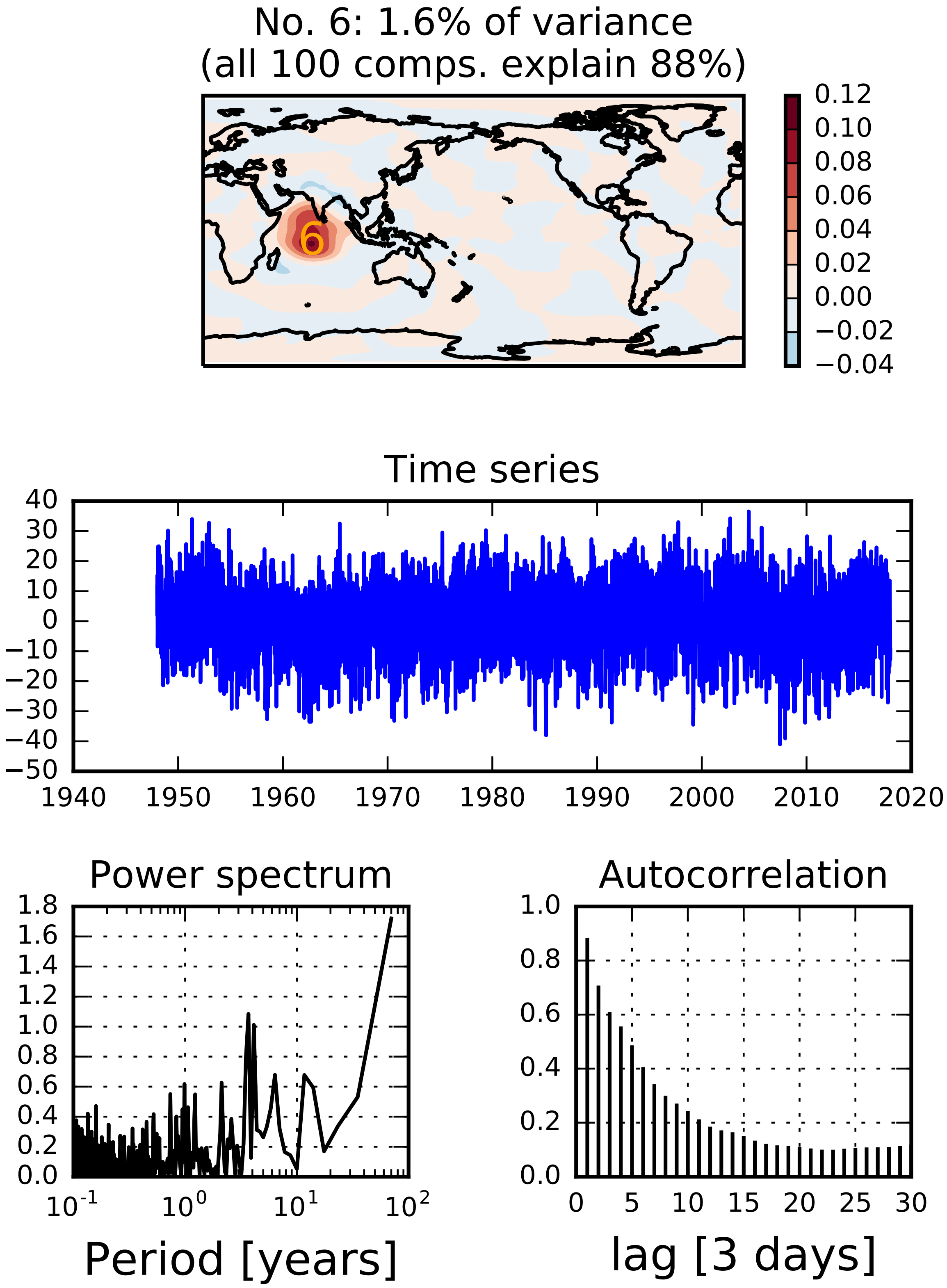
No. 6: 1.6% of variance
(all 100 comps. explain 88%)
6
0.12
0.10
0.08
0.06
0.04
0.02
0.00
−0.02
−0.04
Time series
40
30
20
10
0
−10
−20
−30
−40
−50
1940
1950
1960
1970
1980
1990
2000
2010
2020
Power spectrum
1.8
1.6
1.4
1.2
1.0
0.8
0.6
0.4
0.2
0.0
10^{-1}
10^{0}
10^{1}
10^{2}
Period [years]
Autocorrelation
1.0
0.8
0.6
0.4
0.2
0.0
0
5
10
15
20
25
30
lag [3 days]

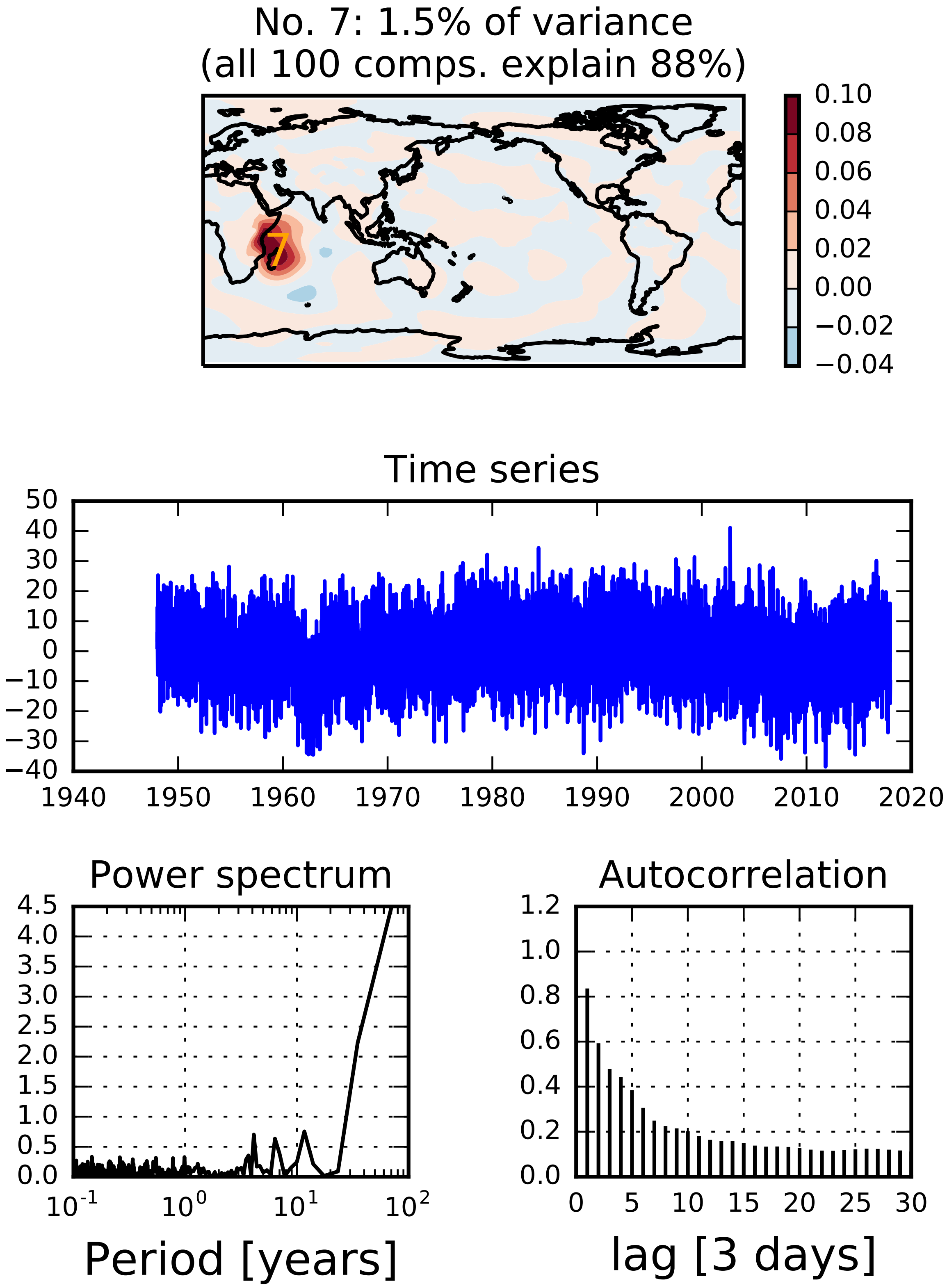
No. 7: 1.5% of variance
(all 100 comps. explain 88%)
7
0.10
0.08
0.06
0.04
0.02
0.00
−0.02
−0.04
Time series
50
40
30
20
10
0
−10
−20
−30
−40
1940 1950 1960 1970 1980 1990 2000 2010 2020
Power spectrum
4.5
4.0
3.5
3.0
2.5
2.0
1.5
1.0
0.5
0.0
10^-1 10^0 10^1 10^2
Period [years]
Autocorrelation
1.2
1.0
0.8
0.6
0.4
0.2
0.0
0 5 10 15 20 25 30
lag [3 days]

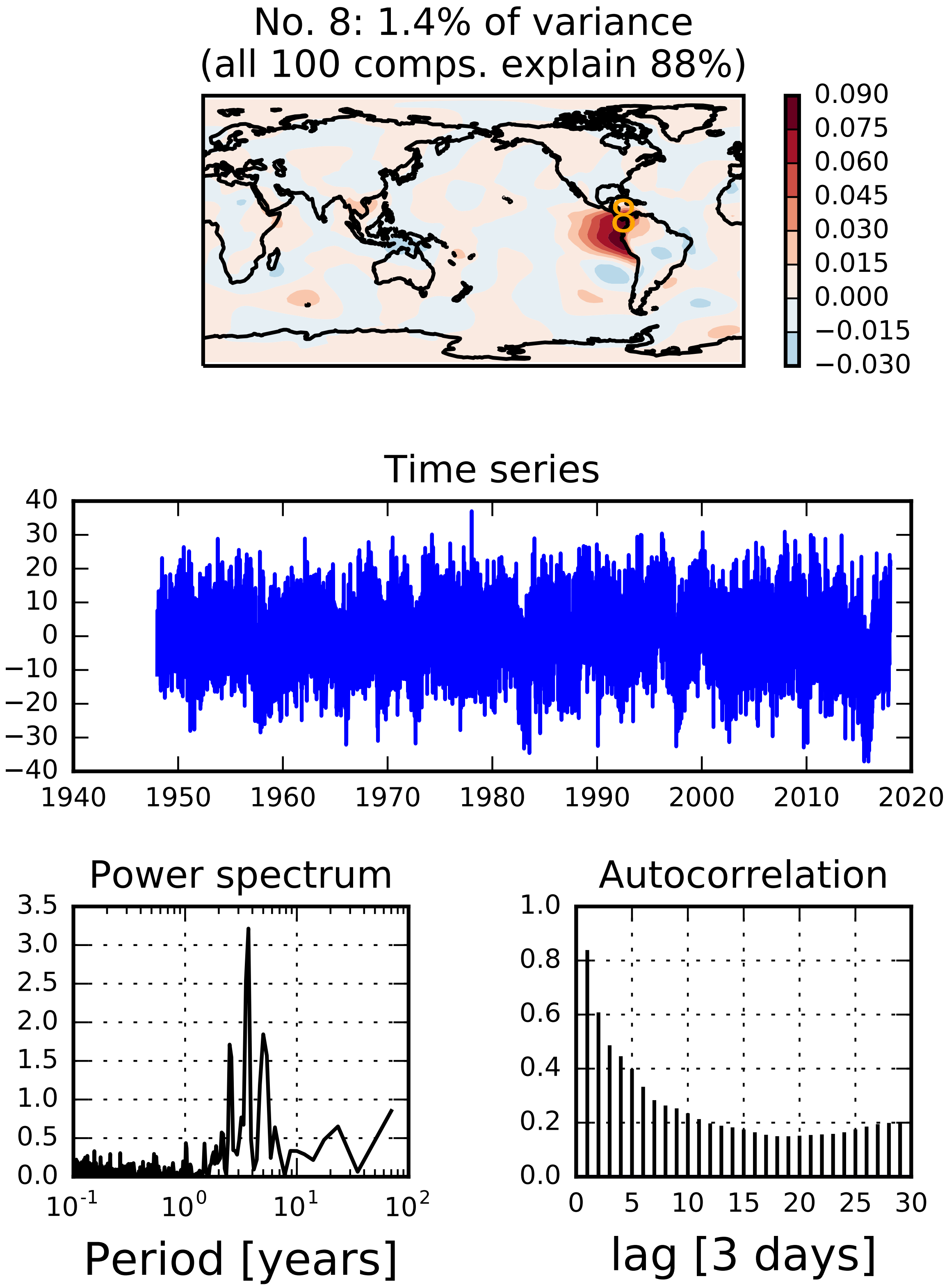
No. 8: 1.4% of variance
(all 100 comps. explain 88%)
0.090
0.075
0.060
0.045
0.030
0.015
0.000
−0.015
−0.030
8
Time series
40
30
20
10
0
−10
−20
−30
−40
1940
1950
1960
1970
1980
1990
2000
2010
2020
Power spectrum
3.5
3.0
2.5
2.0
1.5
1.0
0.5
0.0
10^{-1}
10^{0}
10^{1}
10^{2}
Period [years]
Autocorrelation
1.0
0.8
0.6
0.4
0.2
0.0
0
5
10
15
20
25
30
lag [3 days]

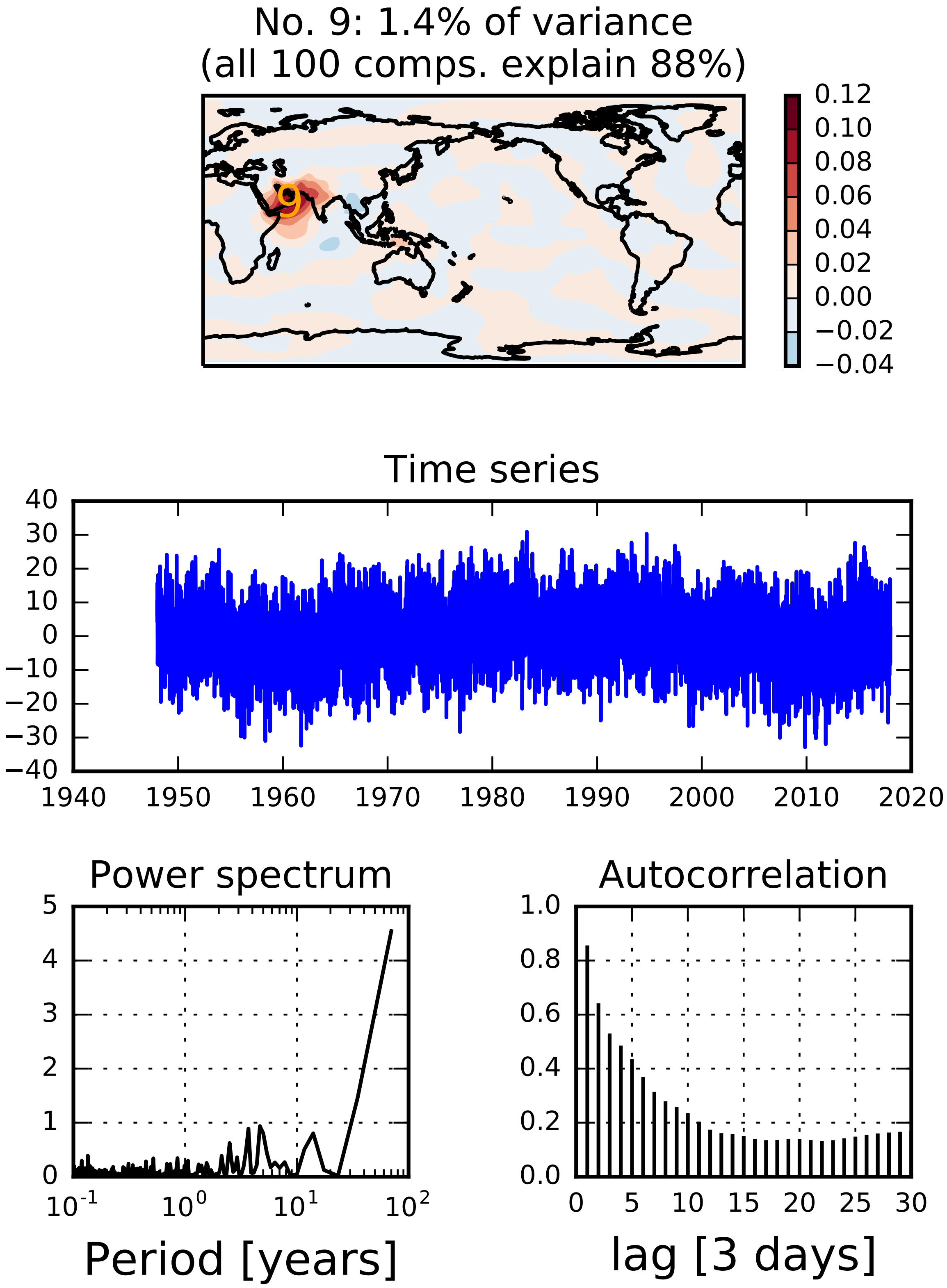
No. 9: 1.4% of variance
(all 100 comps. explain 88%)
9
0.12
0.10
0.08
0.06
0.04
0.02
0.00
−0.02
−0.04
Time series
40
30
20
10
0
−10
−20
−30
−40
1940
1950
1960
1970
1980
1990
2000
2010
2020
Power spectrum
5
4
3
2
1
0
10^{-1}
10^{0}
10^{1}
10^{2}
Period [years]
Autocorrelation
1.0
0.8
0.6
0.4
0.2
0.0
0
5
10
15
20
25
30
lag [3 days]

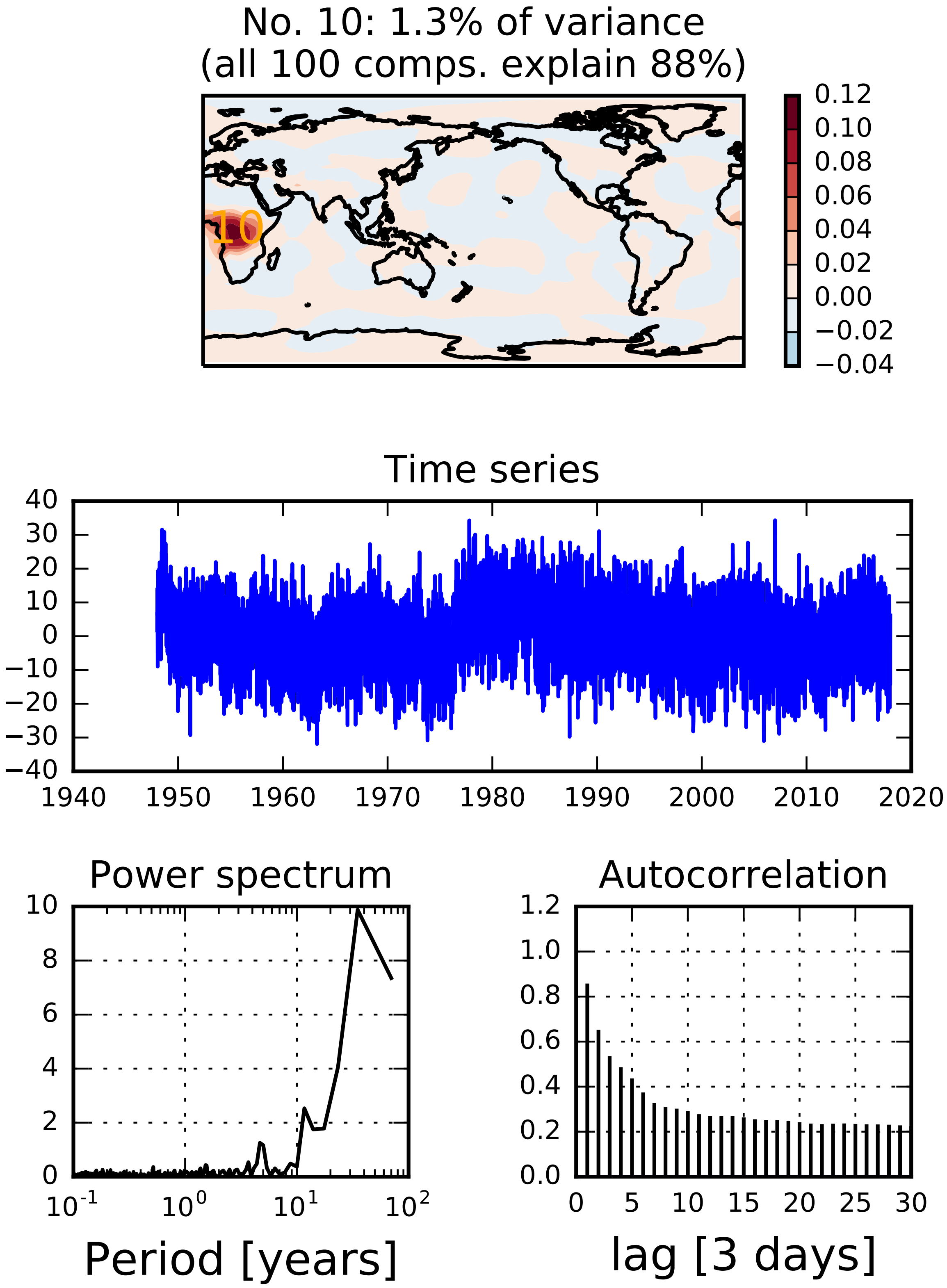
No. 10: 1.3% of variance
(all 100 comps. explain 88%)
10
0.12
0.10
0.08
0.06
0.04
0.02
0.00
−0.02
−0.04
Time series
40
30
20
10
0
−10
−20
−30
−40
1940
1950
1960
1970
1980
1990
2000
2010
2020
Power spectrum
10
8
6
4
2
0
10^{-1}
10^{0}
10^{1}
10^{2}
Period [years]
Autocorrelation
1.2
1.0
0.8
0.6
0.4
0.2
0.0
0
5
10
15
20
25
30
lag [3 days]

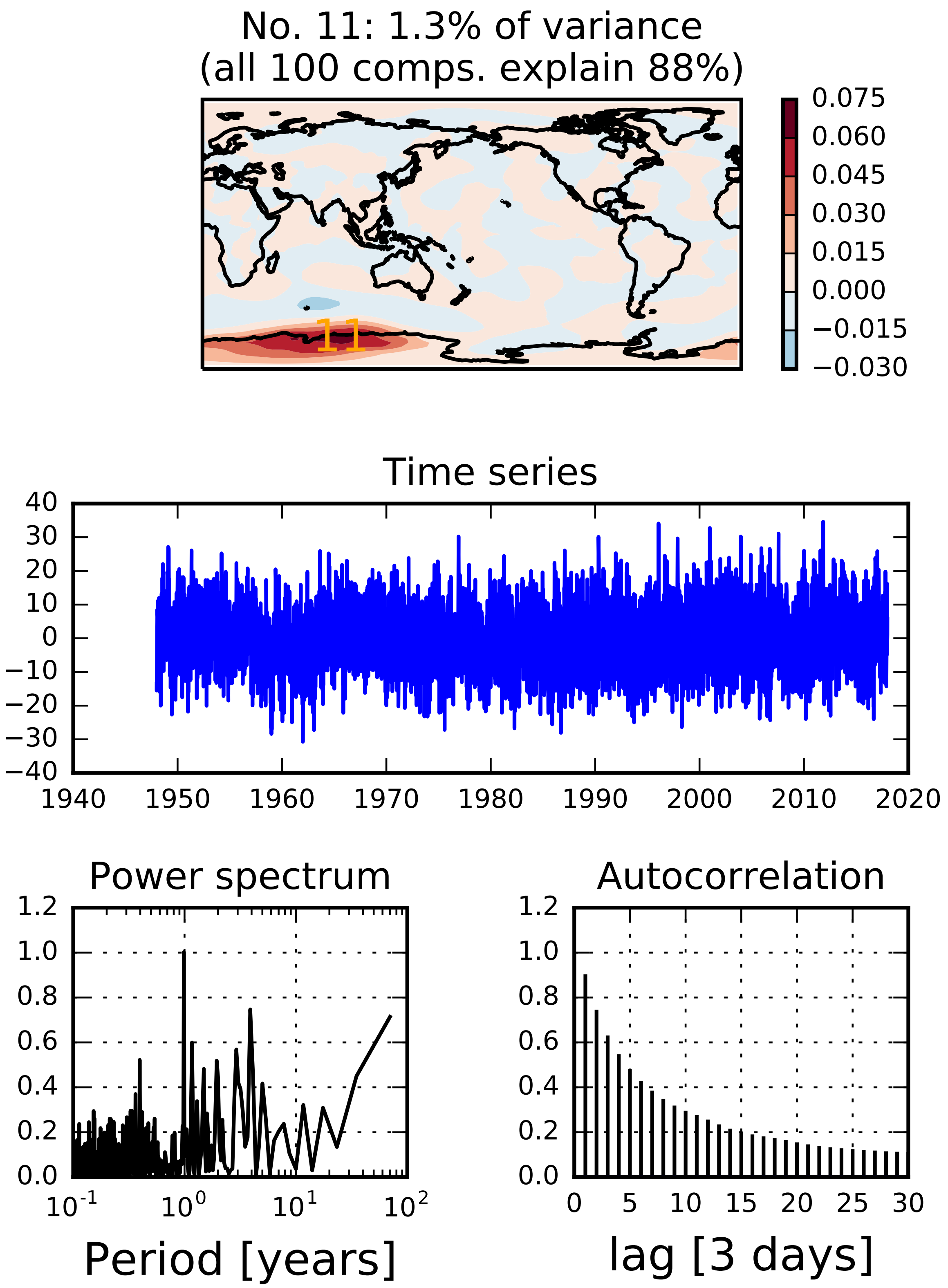
No. 11: 1.3% of variance
(all 100 comps. explain 88%)
11
0.075
0.060
0.045
0.030
0.015
0.000
−0.015
−0.030
Time series
40
30
20
10
0
−10
−20
−30
−40
1940 1950 1960 1970 1980 1990 2000 2010 2020
Power spectrum
1.2 1.0 0.8 0.6 0.4 0.2 0.0
10^-1 10^0 10^1 10^2
Period [years]
Autocorrelation
1.2 1.0 0.8 0.6 0.4 0.2 0.0
0 5 10 15 20 25 30
lag [3 days]

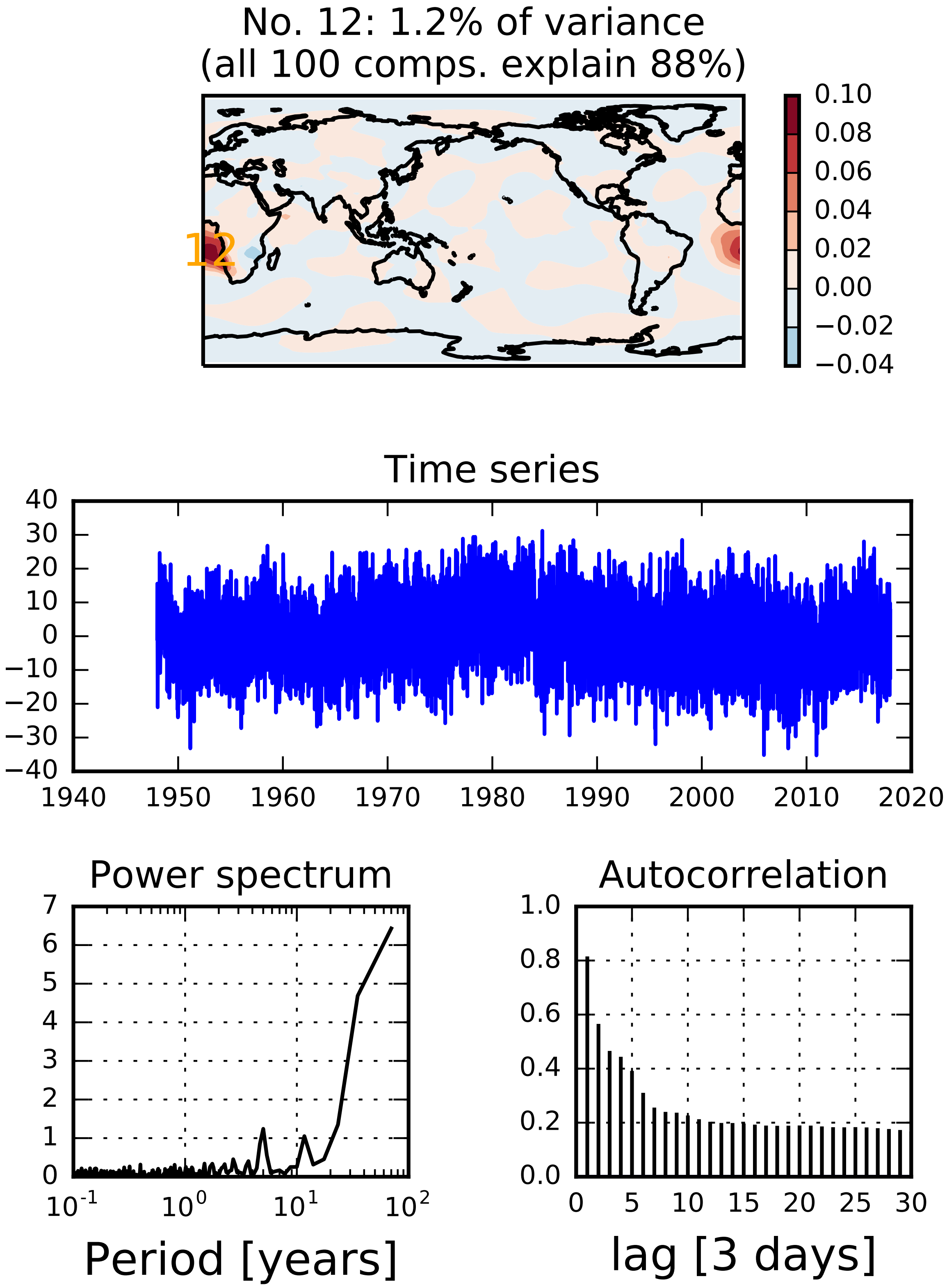
No. 12: 1.2% of variance
(all 100 comps. explain 88%)
12
0.10
0.08
0.06
0.04
0.02
0.00
−0.02
−0.04
Time series
40
30
20
10
0
−10
−20
−30
−40
1940
1950
1960
1970
1980
1990
2000
2010
2020
Power spectrum
7
6
5
4
3
2
1
0
10^-1
10^0
10^1
10^2
Period [years]
Autocorrelation
1.0
0.8
0.6
0.4
0.2
0.0
0
5
10
15
20
25
30
lag [3 days]

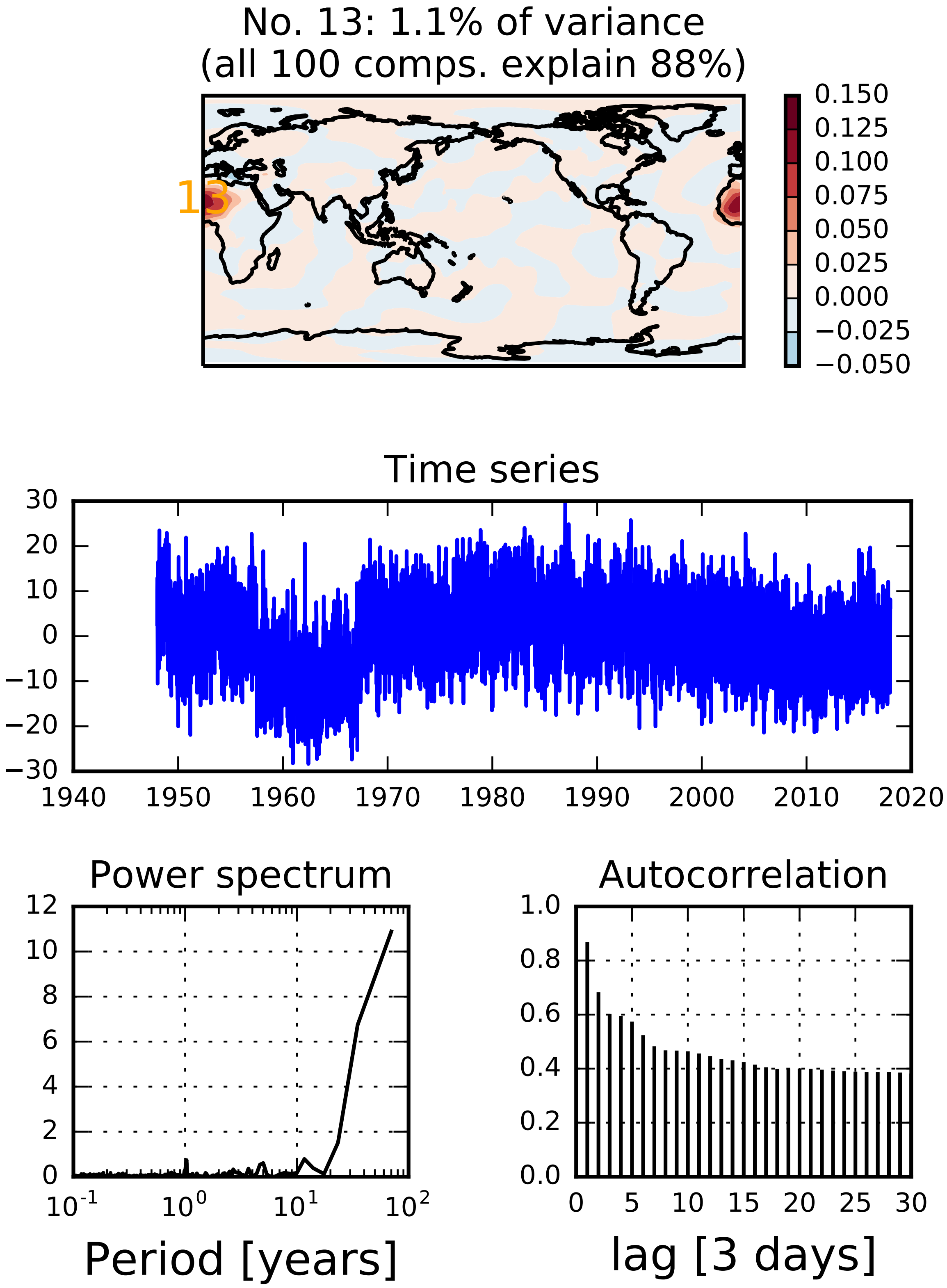
No. 13: 1.1% of variance
(all 100 comps. explain 88%)
13
0.150
0.125
0.100
0.075
0.050
0.025
0.000
−0.025
−0.050
Time series
30
20
10
0
−10
−20
−30
1940
1950
1960
1970
1980
1990
2000
2010
2020
Power spectrum
12
10
8
6
4
2
0
10^{-1}
10^{0}
10^{1}
10^{2}
Period [years]
Autocorrelation
1.0
0.8
0.6
0.4
0.2
0.0
0
5
10
15
20
25
30
lag [3 days]

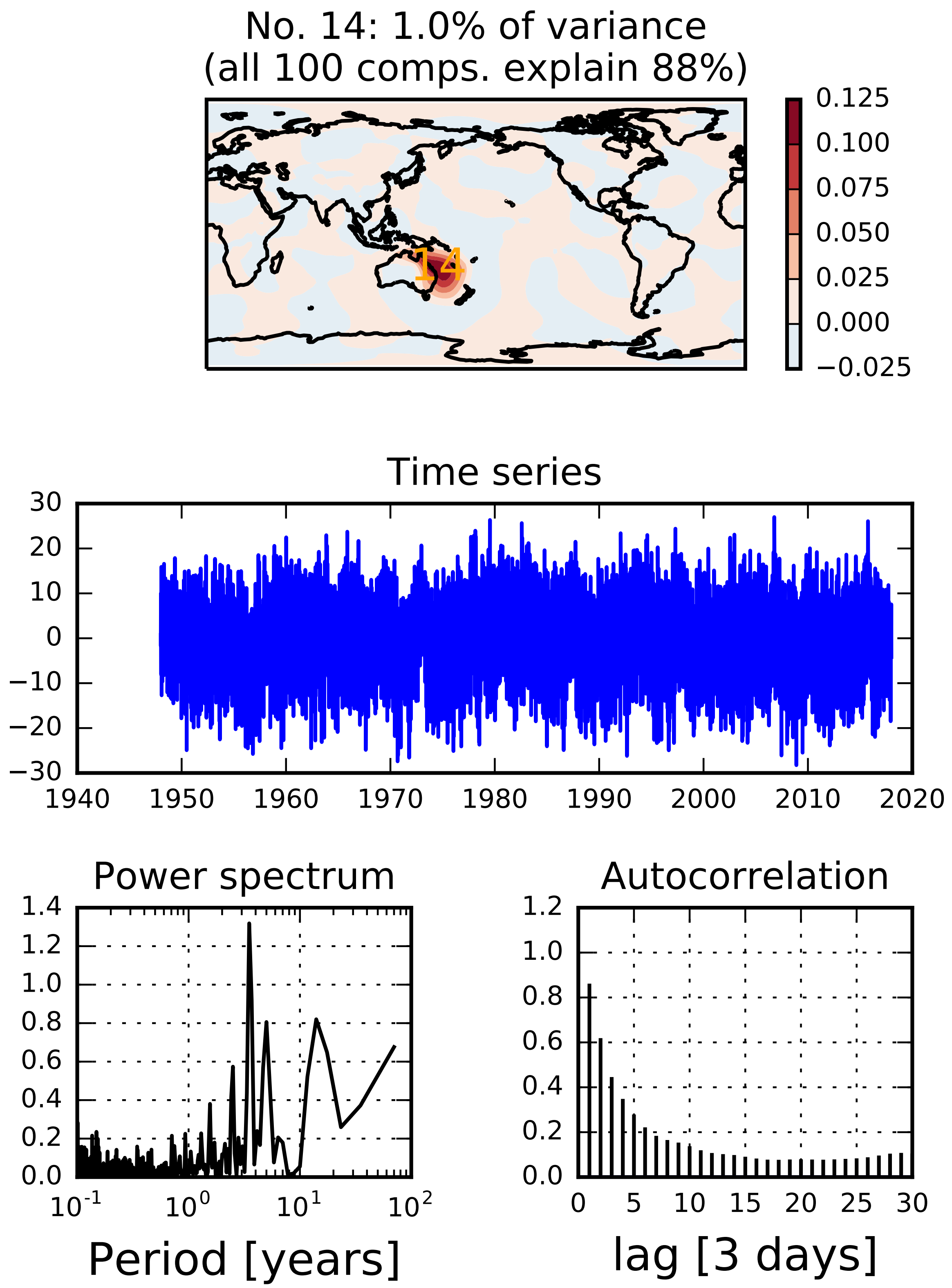
No. 14: 1.0% of variance
(all 100 comps. explain 88%)
0.125
0.100
0.075
0.050
0.025
0.000
−0.025
14
Time series
30
20
10
0
−10
−20
−30
1940
1950
1960
1970
1980
1990
2000
2010
2020
Power spectrum
1.4
1.2
1.0
0.8
0.6
0.4
0.2
0.0
10^{-1}
10^{0}
10^{1}
10^{2}
Period [years]
Autocorrelation
1.2
1.0
0.8
0.6
0.4
0.2
0.0
0
5
10
15
20
25
30
lag [3 days]

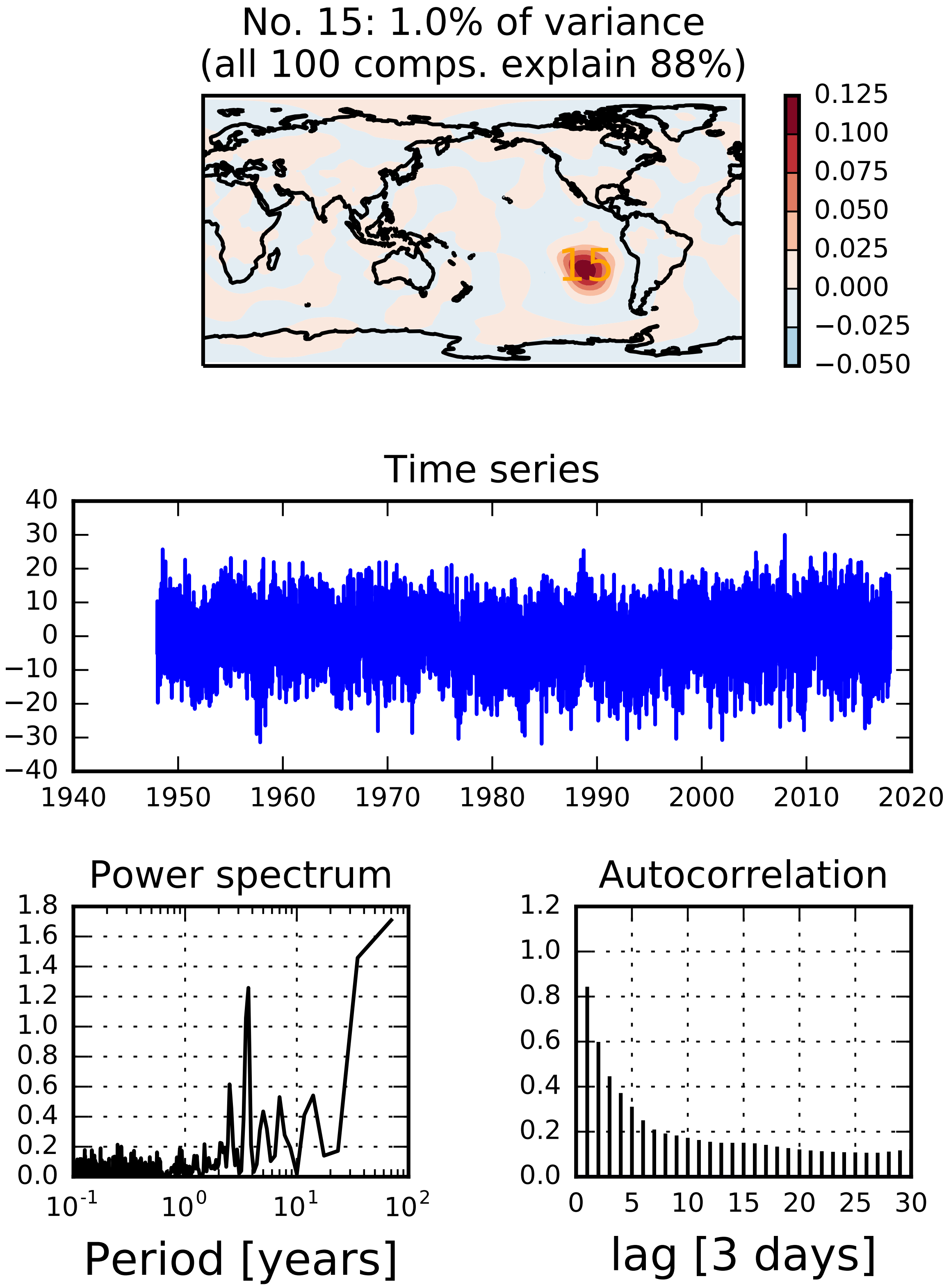
No. 15: 1.0% of variance
(all 100 comps. explain 88%)
15
0.125
0.100
0.075
0.050
0.025
0.000
−0.025
−0.050
Time series
40
30
20
10
0
−10
−20
−30
−40
1940
1950
1960
1970
1980
1990
2000
2010
2020
Power spectrum
1.8
1.6
1.4
1.2
1.0
0.8
0.6
0.4
0.2
0.0
10^-1
10^0
10^1
10^2
Period [years]
Autocorrelation
1.2
1.0
0.8
0.6
0.4
0.2
0.0
0
5
10
15
20
25
30
lag [3 days]

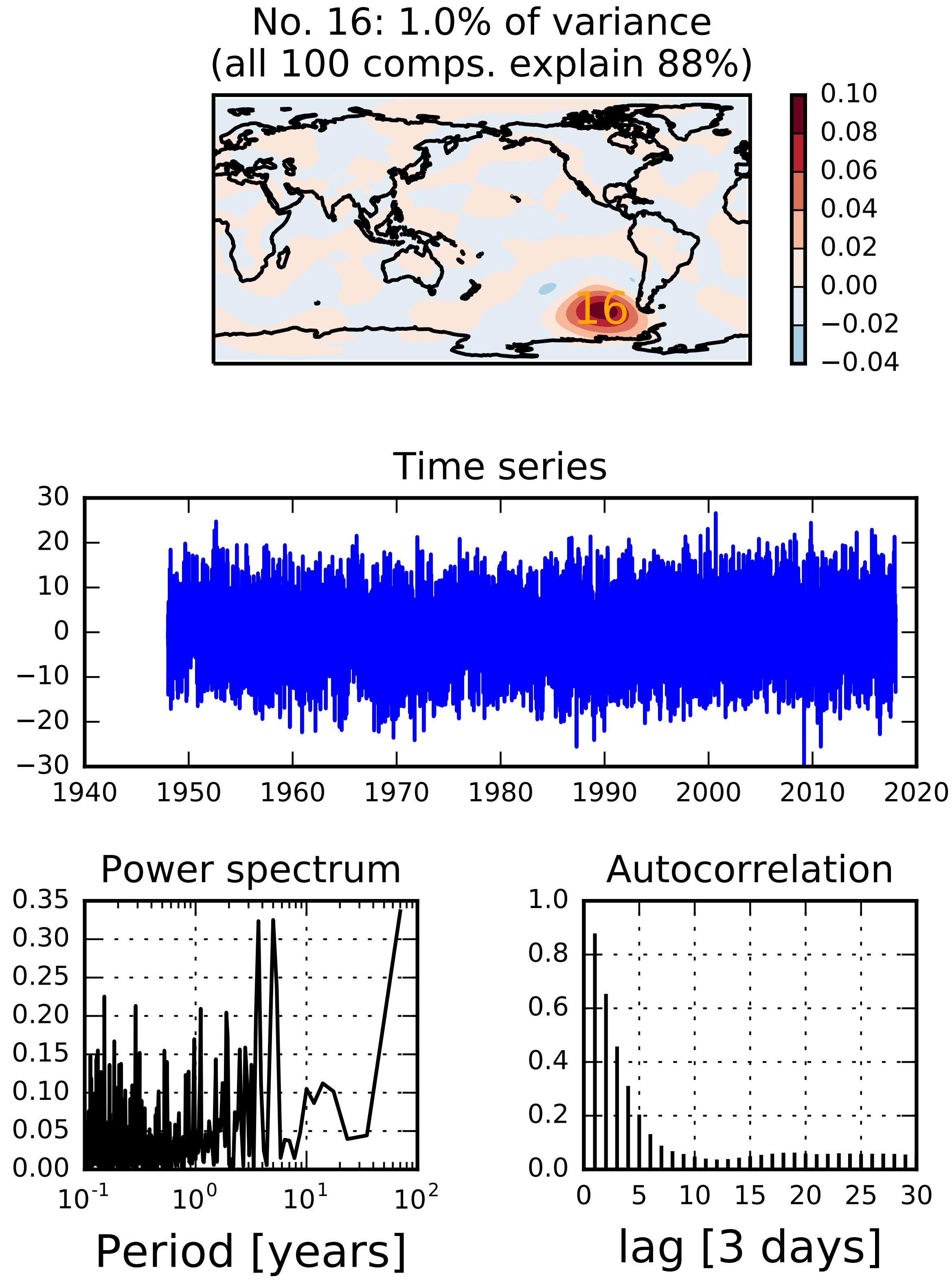
No. 16: 1.0% of variance
(all 100 comps. explain 88%)
16
0.10
0.08
0.06
0.04
0.02
0.00
−0.02
−0.04
Time series
30
20
10
0
−10
−20
−30
1940
1950
1960
1970
1980
1990
2000
2010
2020
Power spectrum
0.35
0.30
0.25
0.20
0.15
0.10
0.05
0.00
10^{-1}
10^{0}
10^{1}
10^{2}
Period [years]
Autocorrelation
1.0
0.8
0.6
0.4
0.2
0.0
0
5
10
15
20
25
30
lag [3 days]

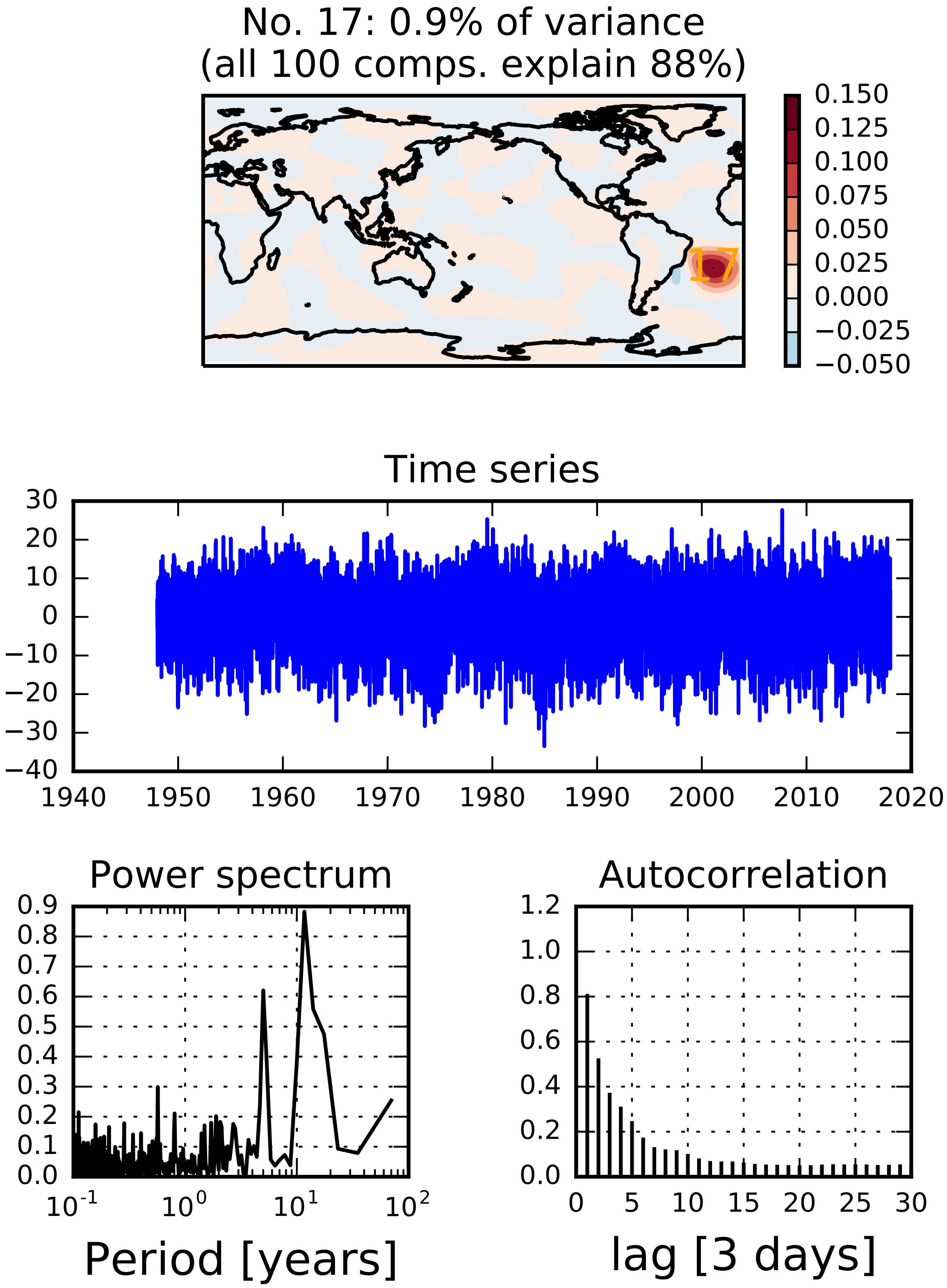
No. 17: 0.9% of variance
(all 100 comps. explain 88%)
0.150
0.125
0.100
0.075
0.050
0.025
0.000
−0.025
−0.050
17
Time series
30
20
10
0
−10
−20
−30
−40
1940
1950
1960
1970
1980
1990
2000
2010
2020
Power spectrum
0.9
0.8
0.7
0.6
0.5
0.4
0.3
0.2
0.1
0.0
10^{-1}
10^{0}
10^{1}
10^{2}
Period [years]
Autocorrelation
1.2
1.0
0.8
0.6
0.4
0.2
0.0
0
5
10
15
20
25
30
lag [3 days]

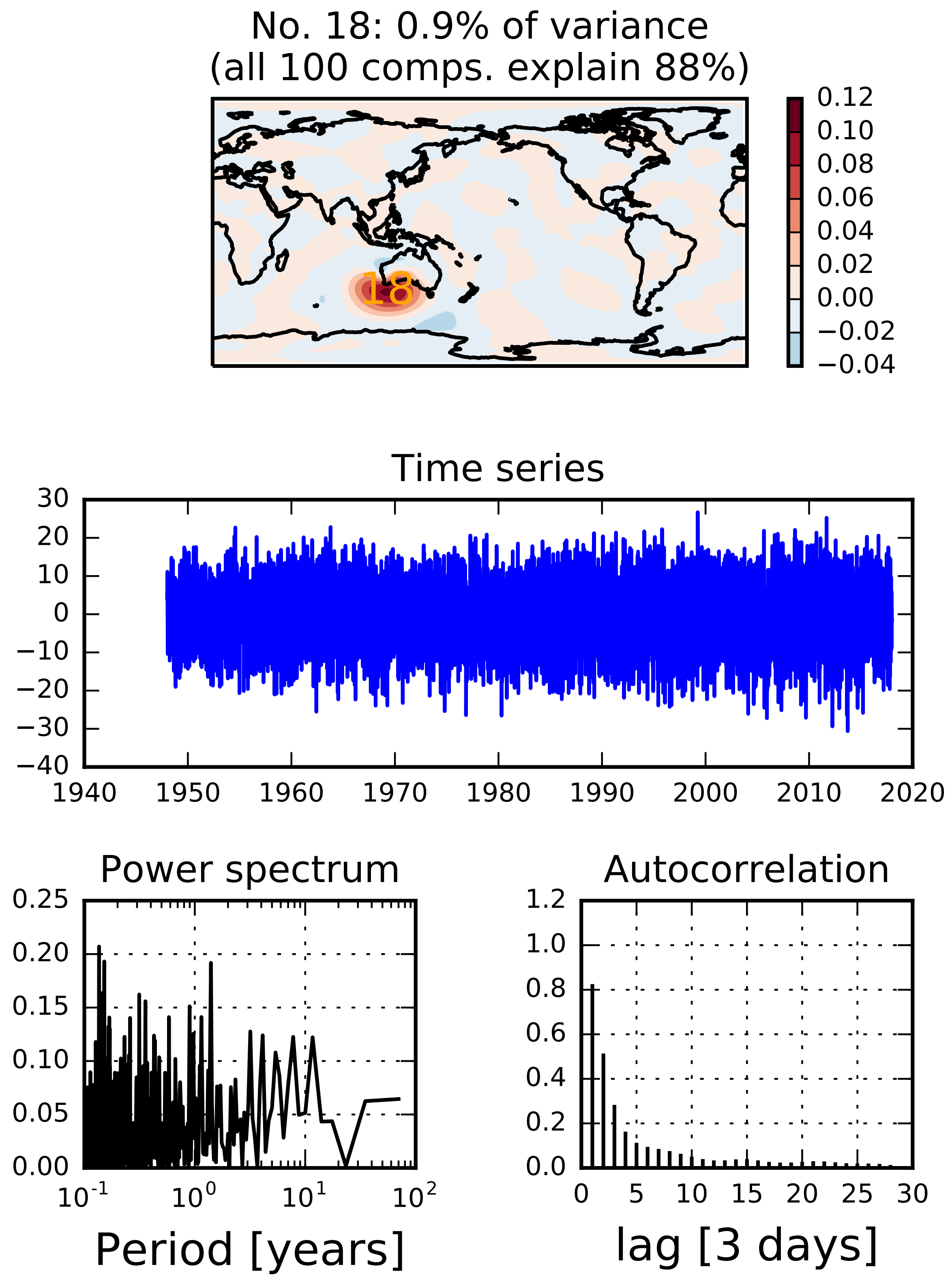
No. 18: 0.9% of variance
(all 100 comps. explain 88%)
18
0.12
0.10
0.08
0.06
0.04
0.02
0.00
−0.02
−0.04
Time series
30
20
10
0
−10
−20
−30
−40
1940
1950
1960
1970
1980
1990
2000
2010
2020
Power spectrum
0.25
0.20
0.15
0.10
0.05
0.00
10^-1
10^0
10^1
10^2
Period [years]
Autocorrelation
1.2
1.0
0.8
0.6
0.4
0.2
0.0
0
5
10
15
20
25
30
lag [3 days]

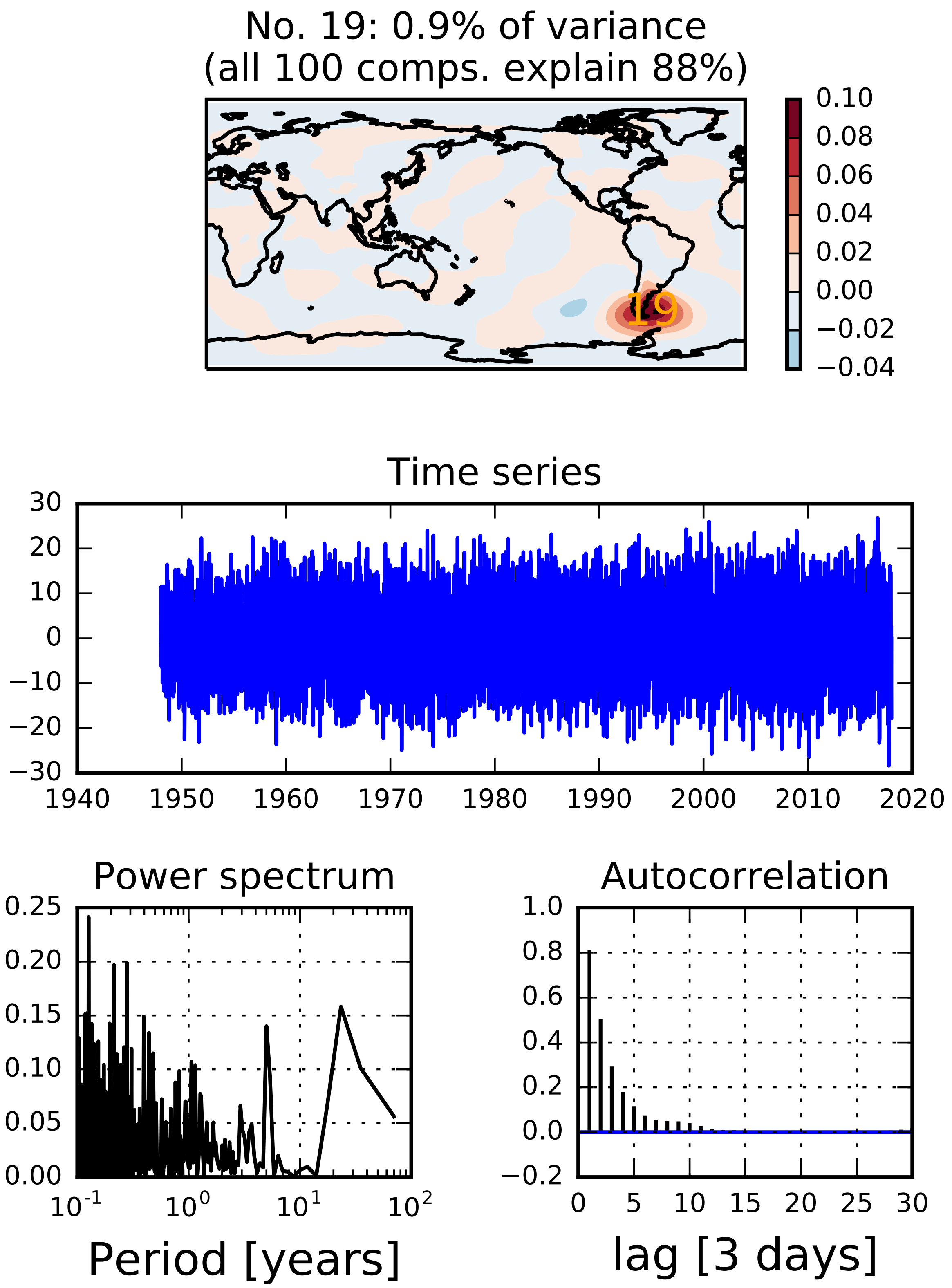
No. 19: 0.9% of variance
(all 100 comps. explain 88%)
19
0.10
0.08
0.06
0.04
0.02
0.00
−0.02
−0.04
Time series
30
20
10
0
−10
−20
−30
1940 1950 1960 1970 1980 1990 2000 2010 2020
Power spectrum
0.25
0.20
0.15
0.10
0.05
0.00
10^{-1} 10^{0} 10^{1} 10^{2}
Period [years]
Autocorrelation
1.0
0.8
0.6
0.4
0.2
0.0
−0.2
0 5 10 15 20 25 30
lag [3 days]

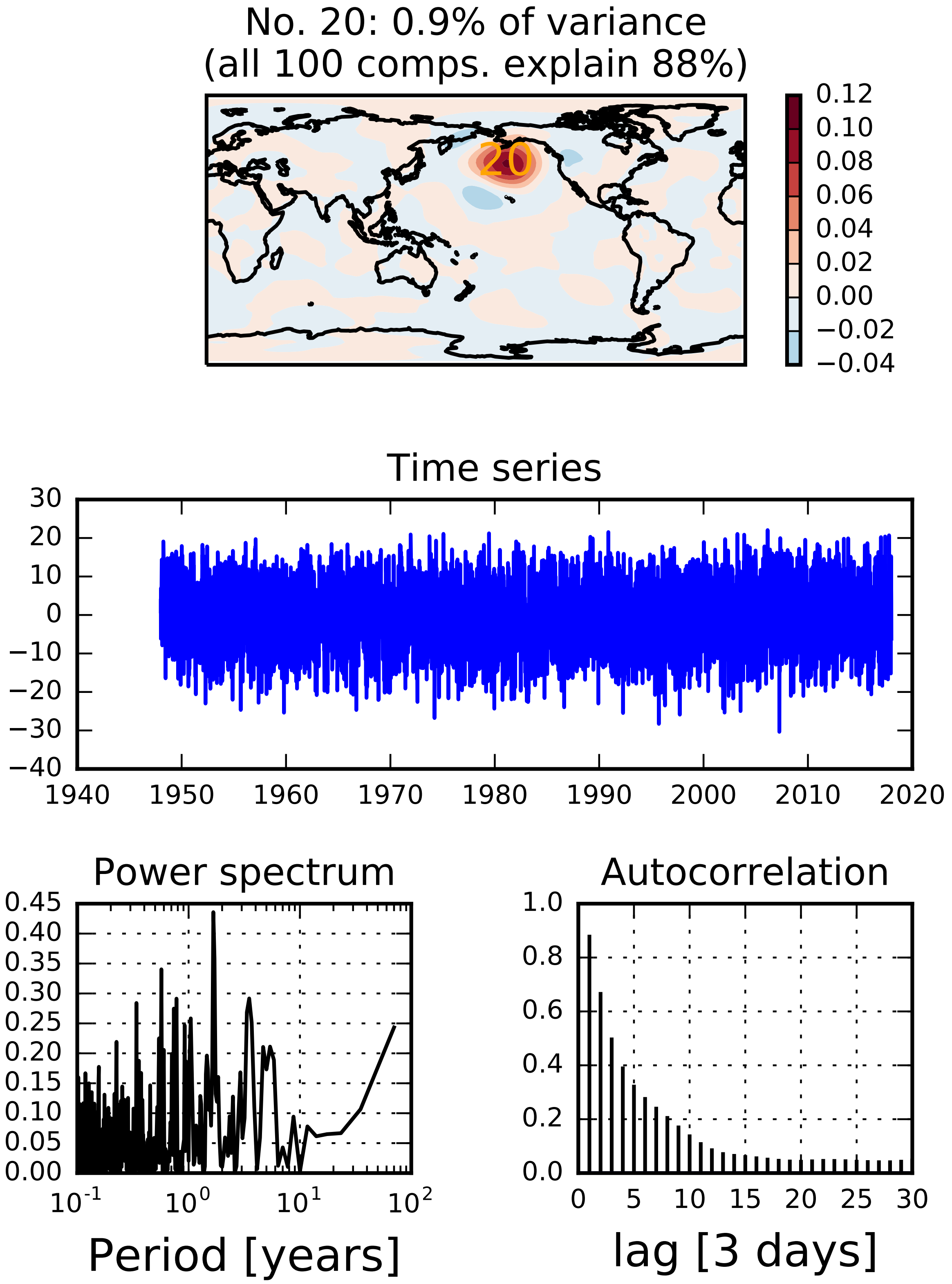
No. 20: 0.9% of variance
(all 100 comps. explain 88%)
20
0.12
0.10
0.08
0.06
0.04
0.02
0.00
−0.02
−0.04
Time series
30
20
10
0
−10
−20
−30
−40
1940
1950
1960
1970
1980
1990
2000
2010
2020
Power spectrum
0.45
0.40
0.35
0.30
0.25
0.20
0.15
0.10
0.05
0.00
10^{-1}
10^{0}
10^{1}
10^{2}
Period [years]
Autocorrelation
1.0
0.8
0.6
0.4
0.2
0.0
0
5
10
15
20
25
30
lag [3 days]

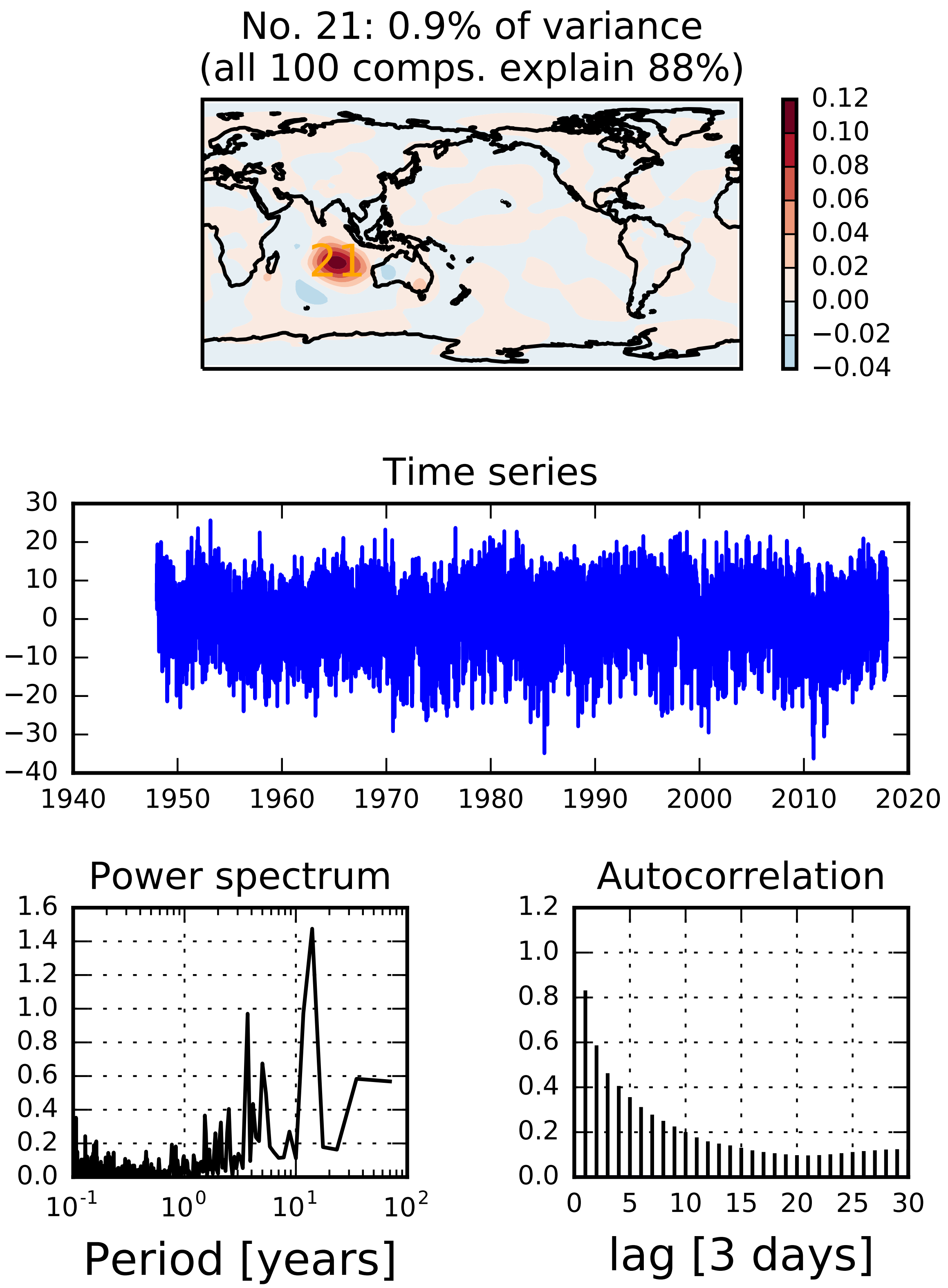
No. 21: 0.9% of variance
(all 100 comps. explain 88%)
21
0.12
0.10
0.08
0.06
0.04
0.02
0.00
−0.02
−0.04
Time series
30
20
10
0
−10
−20
−30
−40
1940
1950
1960
1970
1980
1990
2000
2010
2020
Power spectrum
1.6
1.4
1.2
1.0
0.8
0.6
0.4
0.2
0.0
10^{-1}
10^{0}
10^{1}
10^{2}
Period [years]
Autocorrelation
1.2
1.0
0.8
0.6
0.4
0.2
0.0
0
5
10
15
20
25
30
lag [3 days]

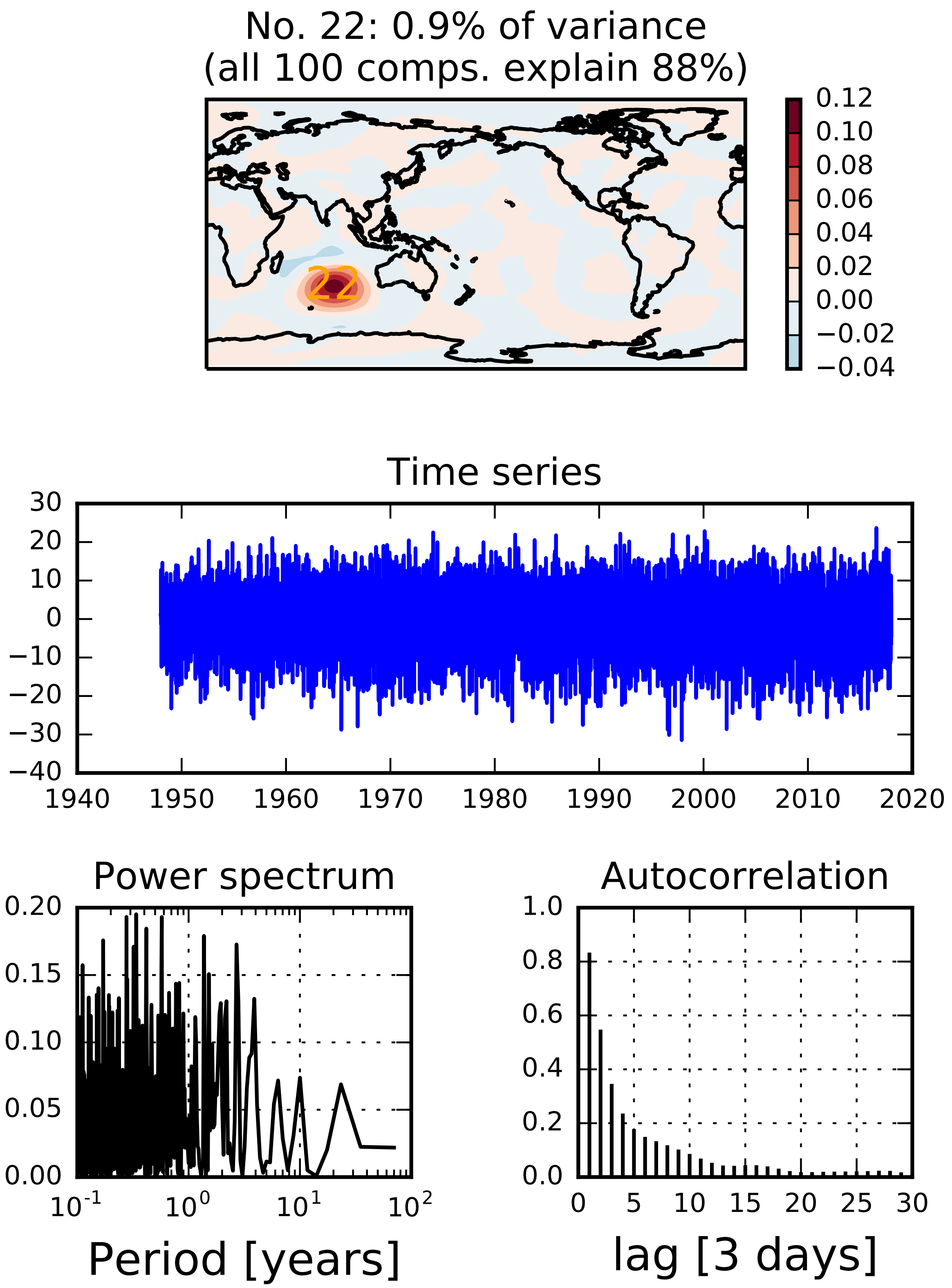
No. 22: 0.9% of variance
(all 100 comps. explain 88%)
22
0.12
0.10
0.08
0.06
0.04
0.02
0.00
−0.02
−0.04
Time series
30
20
10
0
−10
−20
−30
−40
1940
1950
1960
1970
1980
1990
2000
2010
2020
Power spectrum
0.20
0.15
0.10
0.05
0.00
10^-1
10^0
10^1
10^2
Period [years]
Autocorrelation
1.0
0.8
0.6
0.4
0.2
0.0
0
5
10
15
20
25
30
lag [3 days]

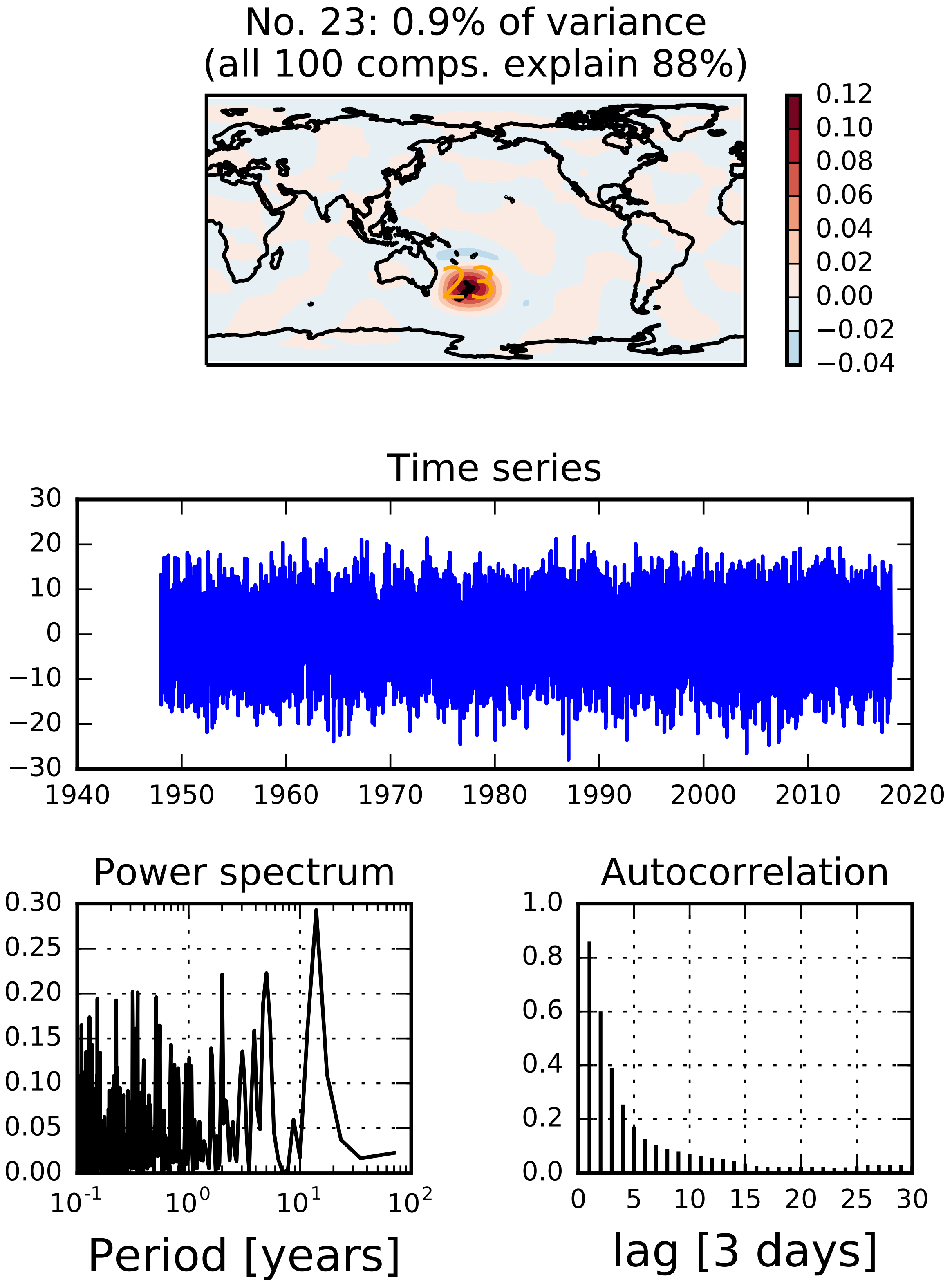
No. 23: 0.9% of variance
(all 100 comps. explain 88%)
0.12
0.10
0.08
0.06
0.04
0.02
0.00
−0.02
−0.04
23
Time series
30
20
10
0
−10
−20
−30
1940
1950
1960
1970
1980
1990
2000
2010
2020
Power spectrum
0.30
0.25
0.20
0.15
0.10
0.05
0.00
10^{-1}
10^{0}
10^{1}
10^{2}
Period [years]
Autocorrelation
1.0
0.8
0.6
0.4
0.2
0.0
0
5
10
15
20
25
30
lag [3 days]

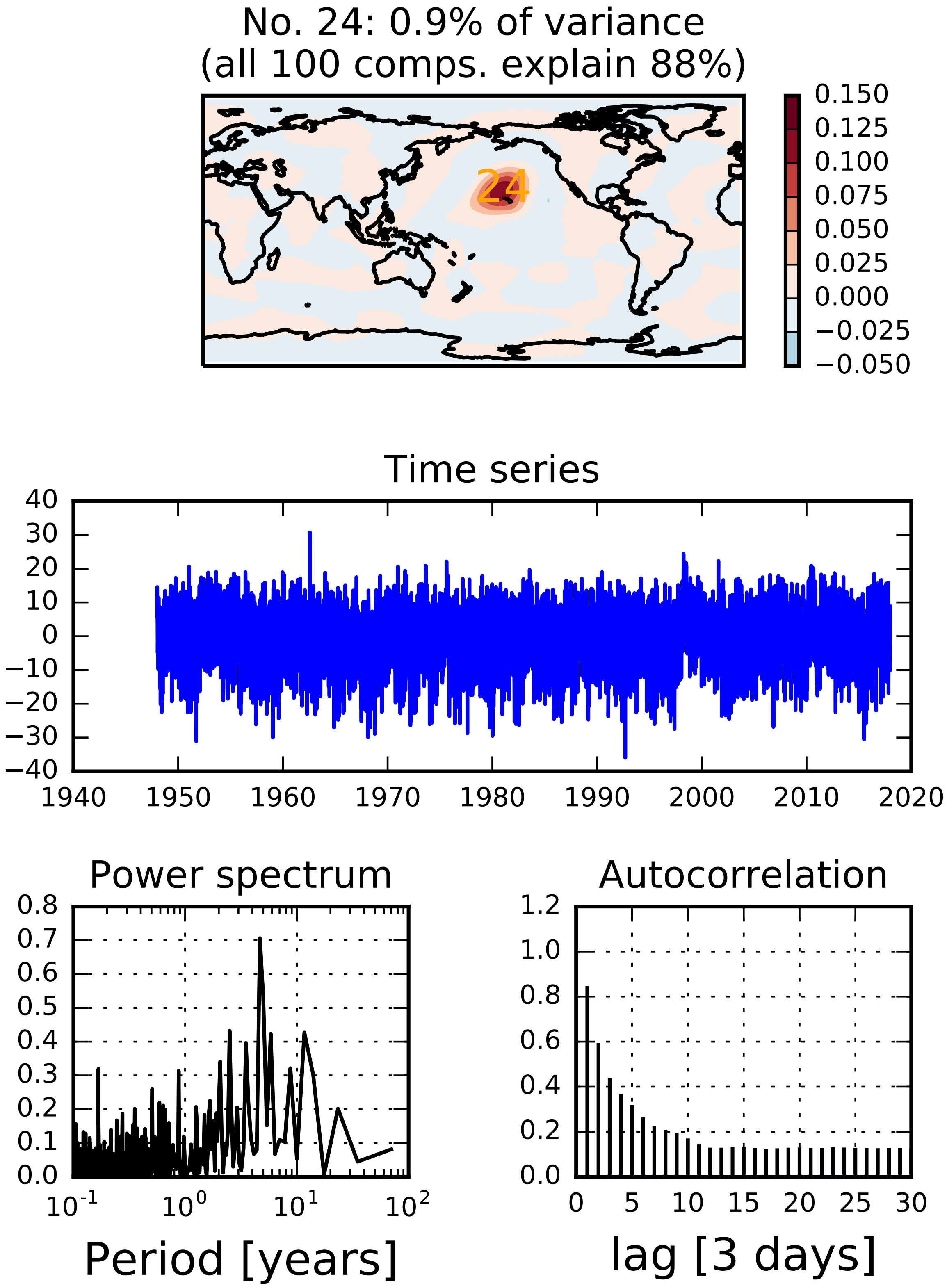
No. 24: 0.9% of variance
(all 100 comps. explain 88%)
24
0.150
0.125
0.100
0.075
0.050
0.025
0.000
−0.025
−0.050
Time series
40
30
20
10
0
−10
−20
−30
−40
1940
1950
1960
1970
1980
1990
2000
2010
2020
Power spectrum
0.8
0.7
0.6
0.5
0.4
0.3
0.2
0.1
0.0
10^-1
10^0
10^1
10^2
Period [years]
Autocorrelation
1.2
1.0
0.8
0.6
0.4
0.2
0.0
0
5
10
15
20
25
30
lag [3 days]

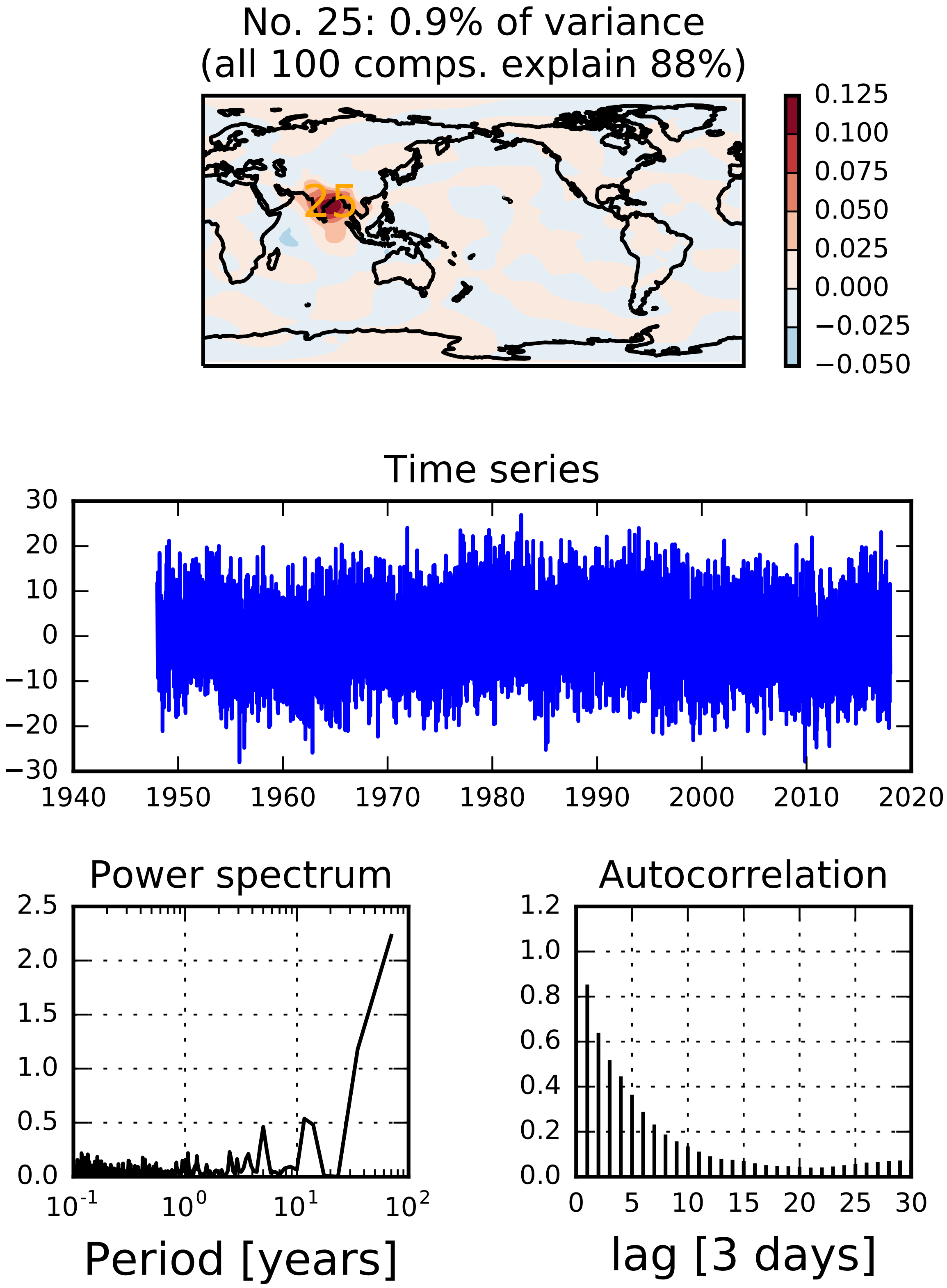
No. 25: 0.9% of variance
(all 100 comps. explain 88%)
25
0.125
0.100
0.075
0.050
0.025
0.000
−0.025
−0.050
Time series
30
20
10
0
−10
−20
−30
1940
1950
1960
1970
1980
1990
2000
2010
2020
Power spectrum
2.5
2.0
1.5
1.0
0.5
0.0
10^{-1}
10^{0}
10^{1}
10^{2}
Period [years]
Autocorrelation
1.2
1.0
0.8
0.6
0.4
0.2
0.0
0
5
10
15
20
25
30
lag [3 days]

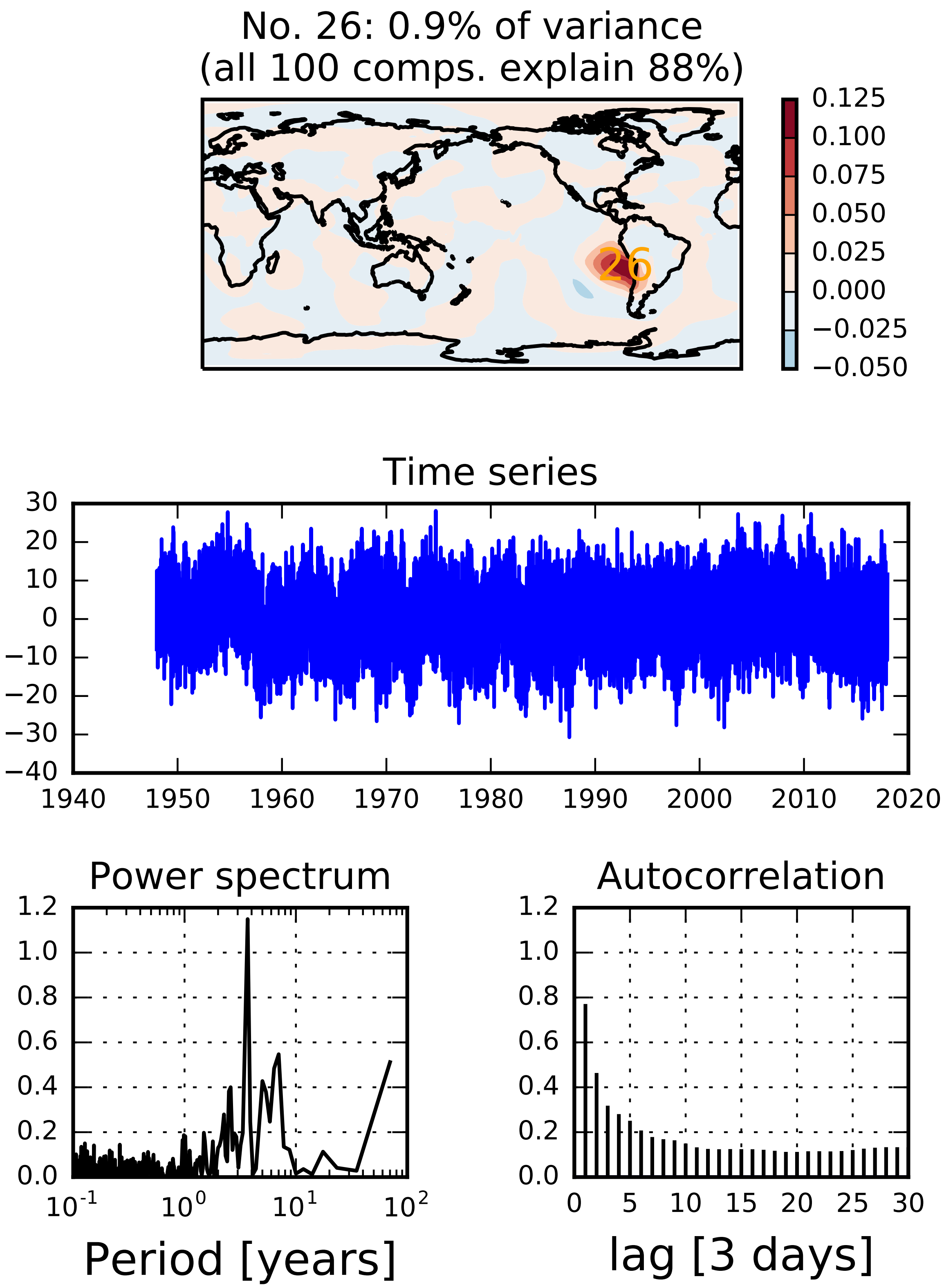
No. 26: 0.9% of variance
(all 100 comps. explain 88%)
0.125
0.100
0.075
0.050
0.025
0.000
−0.025
−0.050
26
Time series
30
20
10
0
−10
−20
−30
−40
1940
1950
1960
1970
1980
1990
2000
2010
2020
Power spectrum
1.2
1.0
0.8
0.6
0.4
0.2
0.0
10^{-1}
10^{0}
10^{1}
10^{2}
Period [years]
Autocorrelation
0
5
10
15
20
25
30
lag [3 days]

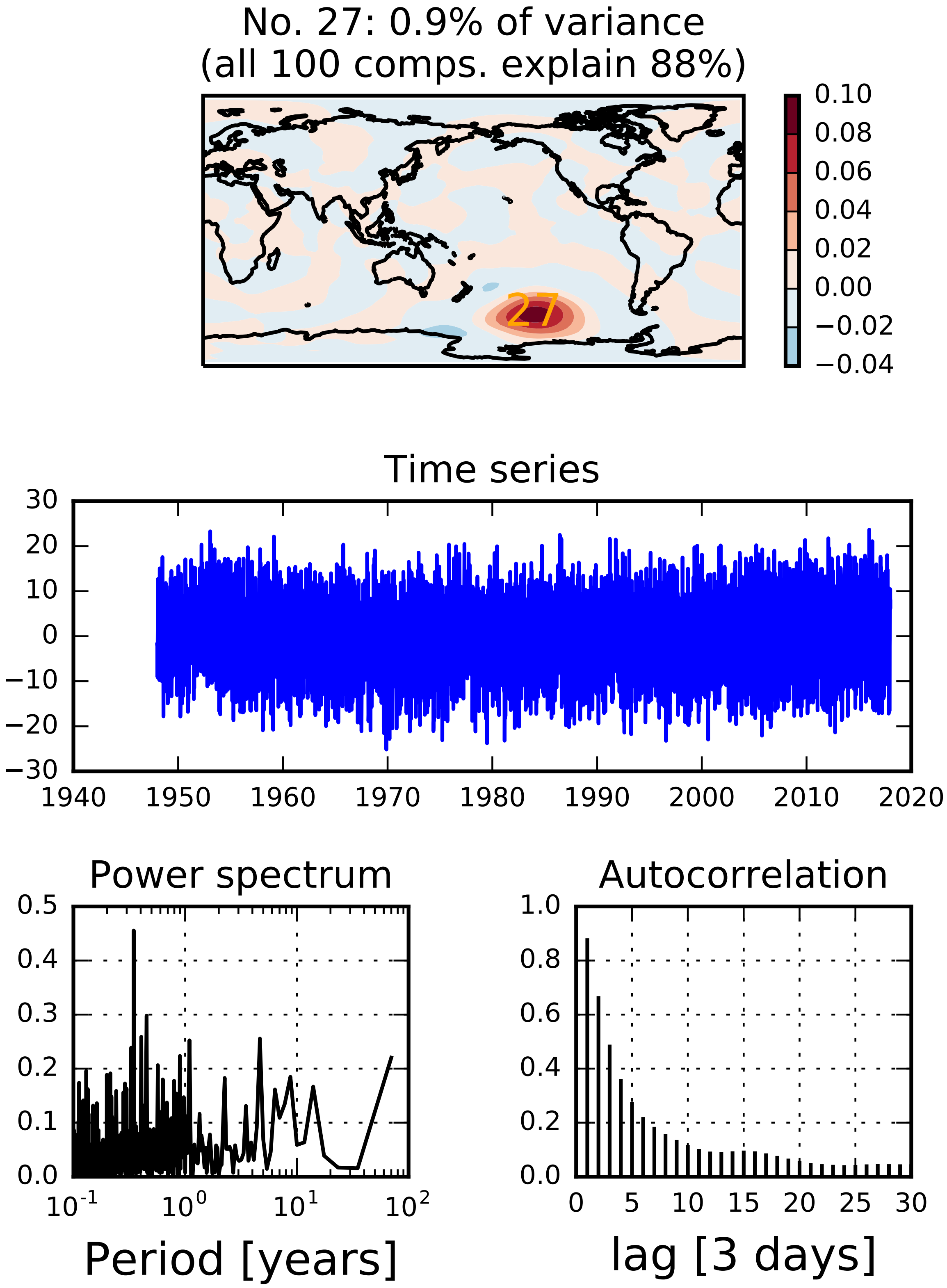
No. 27: 0.9% of variance
(all 100 comps. explain 88%)
27
0.10
0.08
0.06
0.04
0.02
0.00
−0.02
−0.04
Time series
30
20
10
0
−10
−20
−30
1940
1950
1960
1970
1980
1990
2000
2010
2020
Power spectrum
0.5
0.4
0.3
0.2
0.1
0.0
10-1
10^0
10^1
10^2
Period [years]
Autocorrelation
1.0
0.8
0.6
0.4
0.2
0.0
0
5
10
15
20
25
30
lag [3 days]

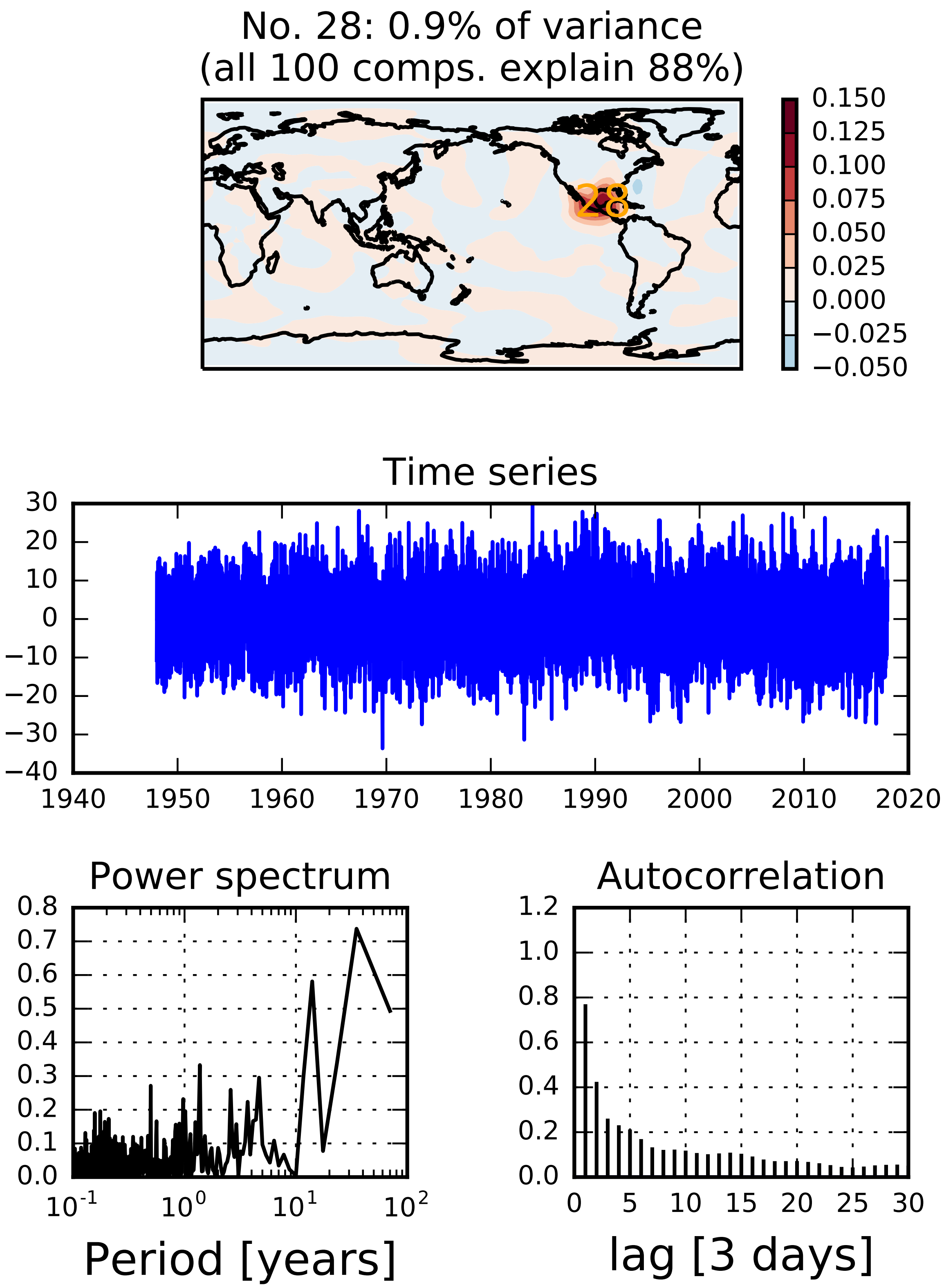
No. 28: 0.9% of variance
(all 100 comps. explain 88%)
28
0.150
0.125
0.100
0.075
0.050
0.025
0.000
−0.025
−0.050
Time series
30
20
10
0
−10
−20
−30
−40
1940
1950
1960
1970
1980
1990
2000
2010
2020
Power spectrum
0.8
0.7
0.6
0.5
0.4
0.3
0.2
0.1
0.0
10^-1
10^0
10^1
10^2
Period [years]
Autocorrelation
1.2
1.0
0.8
0.6
0.4
0.2
0.0
0
5
10
15
20
25
30
lag [3 days]

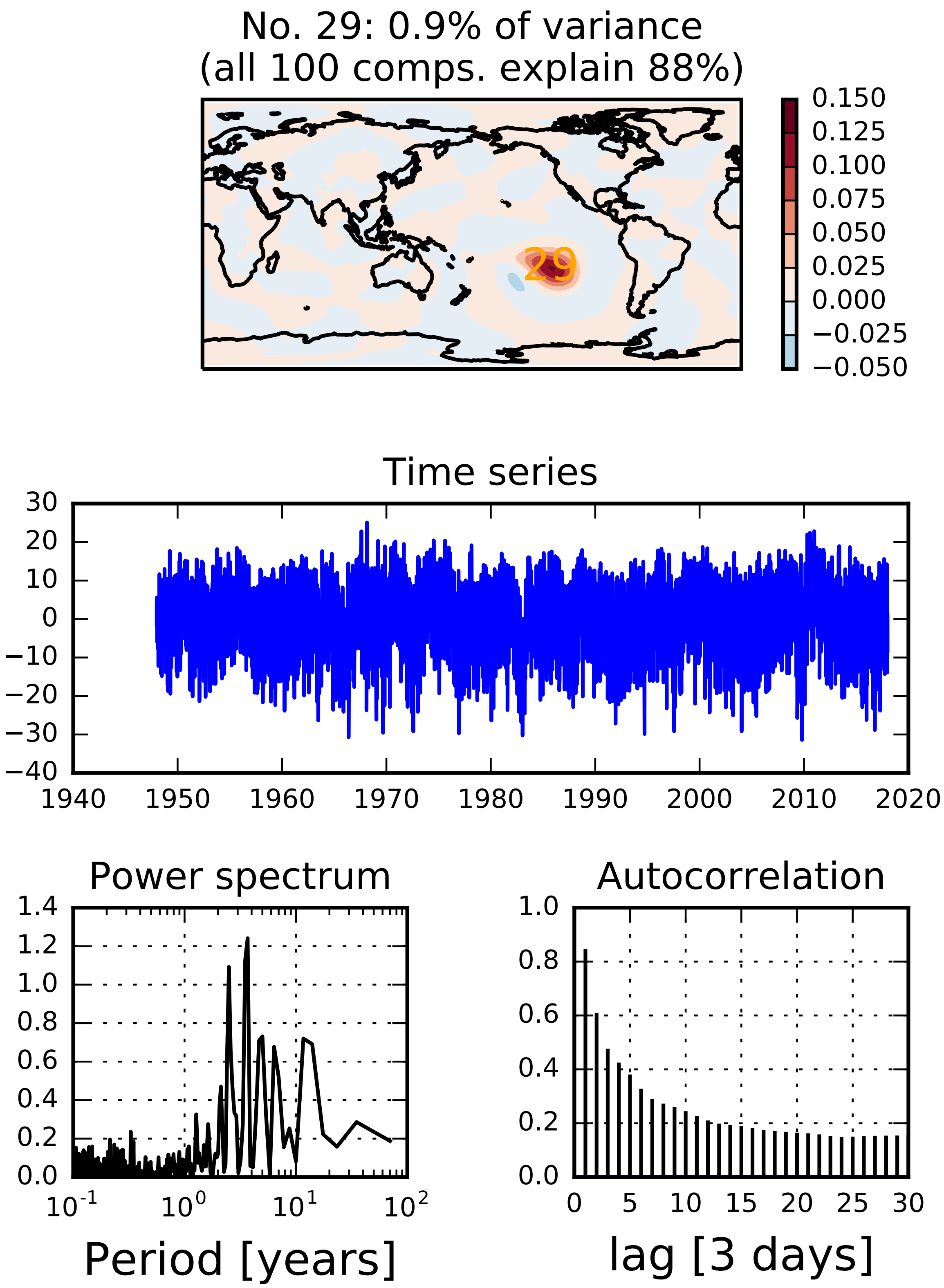
No. 29: 0.9% of variance
(all 100 comps. explain 88%)
29
0.150
0.125
0.100
0.075
0.050
0.025
0.000
−0.025
−0.050
Time series
30
20
10
0
−10
−20
−30
−40
1940
1950
1960
1970
1980
1990
2000
2010
2020
Power spectrum
1.4
1.2
1.0
0.8
0.6
0.4
0.2
0.0
10^{-1}
10^{0}
10^{1}
10^{2}
Period [years]
Autocorrelation
1.0
0.8
0.6
0.4
0.2
0.0
0
5
10
15
20
25
30
lag [3 days]

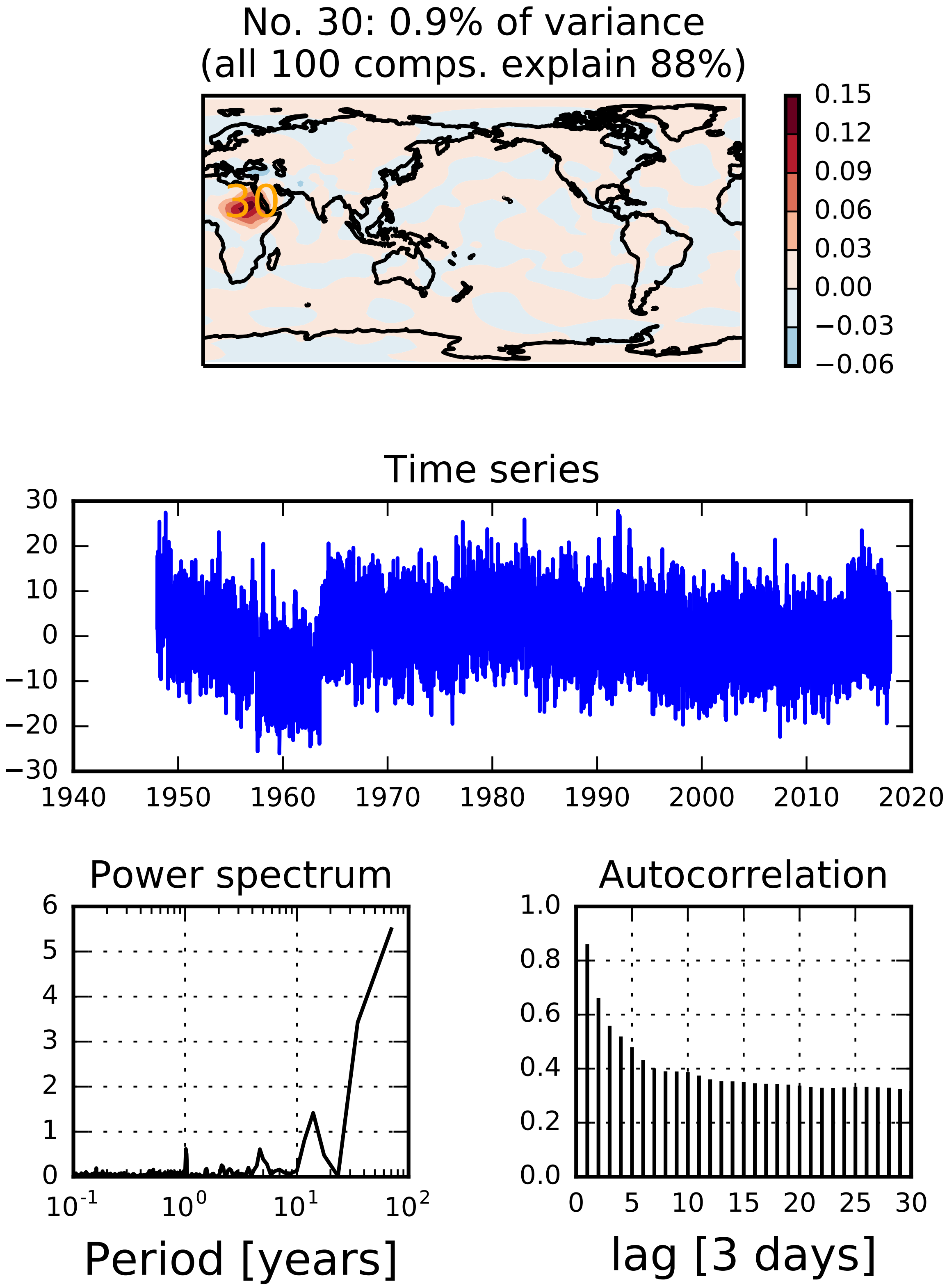
No. 30: 0.9% of variance
(all 100 comps. explain 88%)
30
0.15
0.12
0.09
0.06
0.03
0.00
−0.03
−0.06
Time series
30
20
10
0
−10
−20
−30
1940
1950
1960
1970
1980
1990
2000
2010
2020
Power spectrum
6
5
4
3
2
1
0
10^{-1}
10^{0}
10^{1}
10^{2}
Period [years]
Autocorrelation
1.0
0.8
0.6
0.4
0.2
0.0
0
5
10
15
20
25
30
lag [3 days]

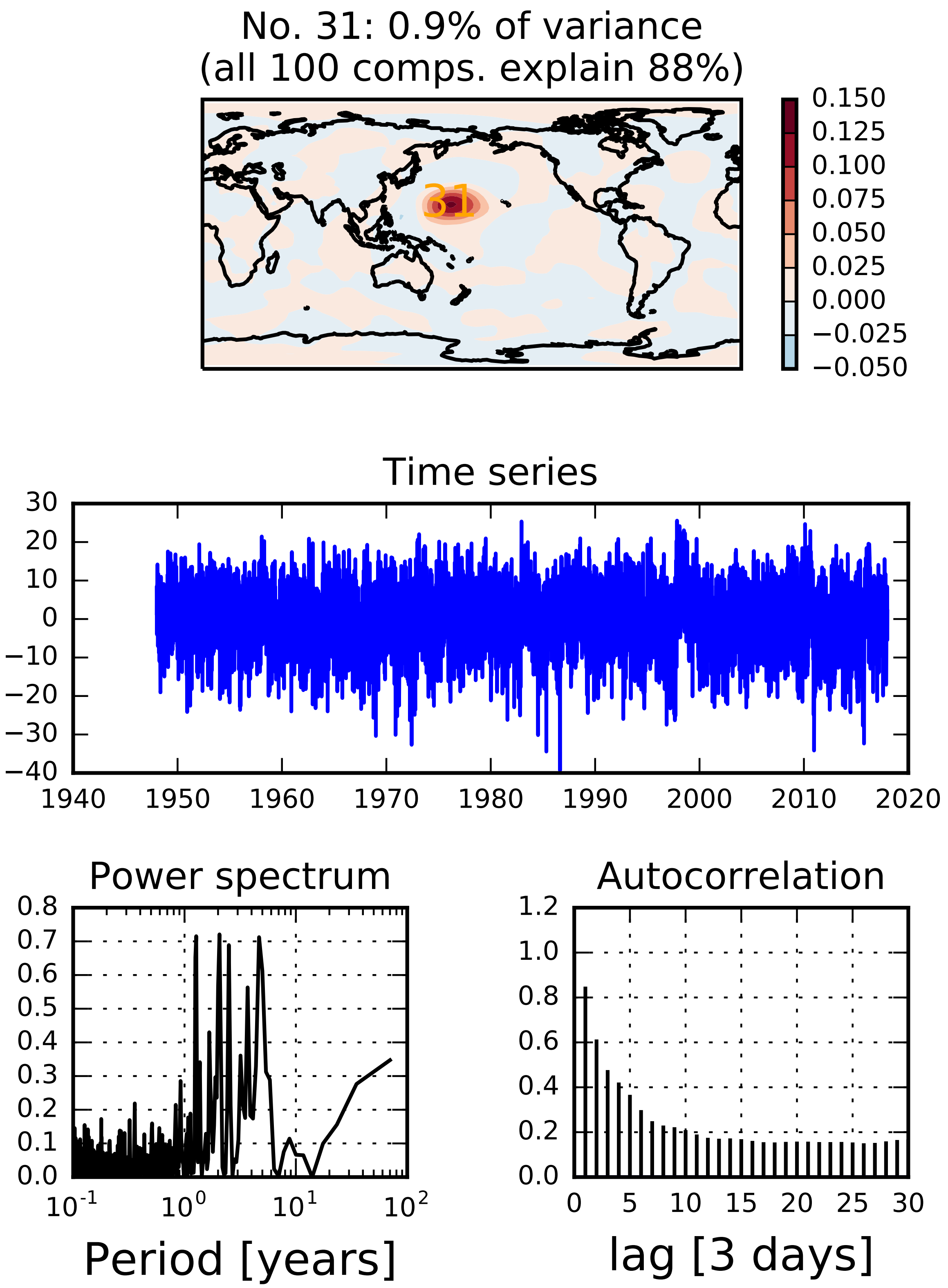
No. 31: 0.9% of variance
(all 100 comps. explain 88%)
31
0.150
0.125
0.100
0.075
0.050
0.025
0.000
−0.025
−0.050
Time series
30
20
10
0
−10
−20
−30
−40
1940
1950
1960
1970
1980
1990
2000
2010
2020
Power spectrum
0.8
0.7
0.6
0.5
0.4
0.3
0.2
0.1
0.0
10^{-1}
10^{0}
10^{1}
10^{2}
Period [years]
Autocorrelation
1.2
1.0
0.8
0.6
0.4
0.2
0.0
0
5
10
15
20
25
30
lag [3 days]

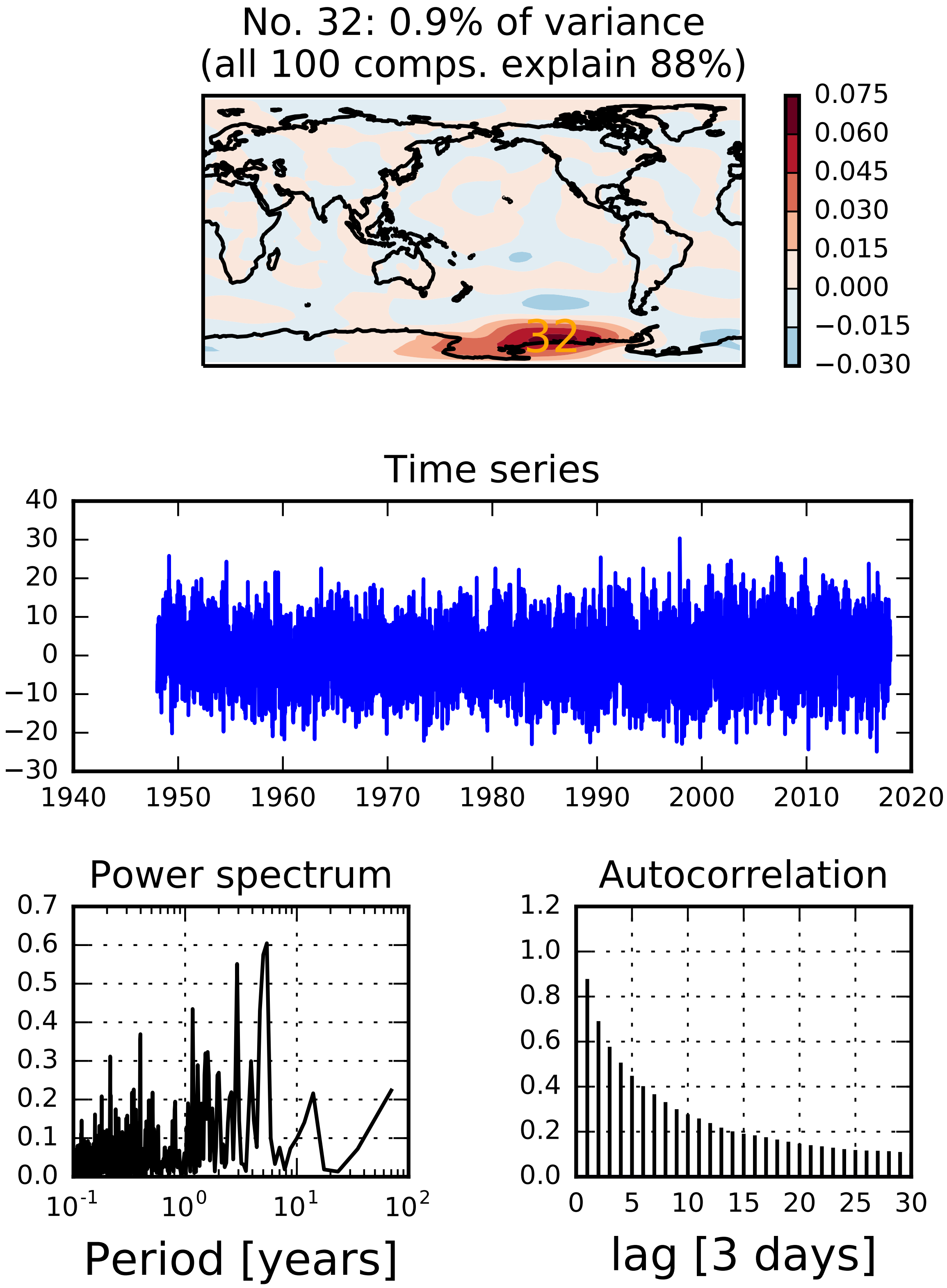
No. 32: 0.9% of variance
(all 100 comps. explain 88%)
32
0.075
0.060
0.045
0.030
0.015
0.000
−0.015
−0.030
Time series
40
30
20
10
0
−10
−20
−30
1940
1950
1960
1970
1980
1990
2000
2010
2020
Power spectrum
0.7
0.6
0.5
0.4
0.3
0.2
0.1
0.0
10^{-1}
10^{0}
10^{1}
10^{2}
Period [years]
Autocorrelation
1.2
1.0
0.8
0.6
0.4
0.2
0.0
0
5
10
15
20
25
30
lag [3 days]

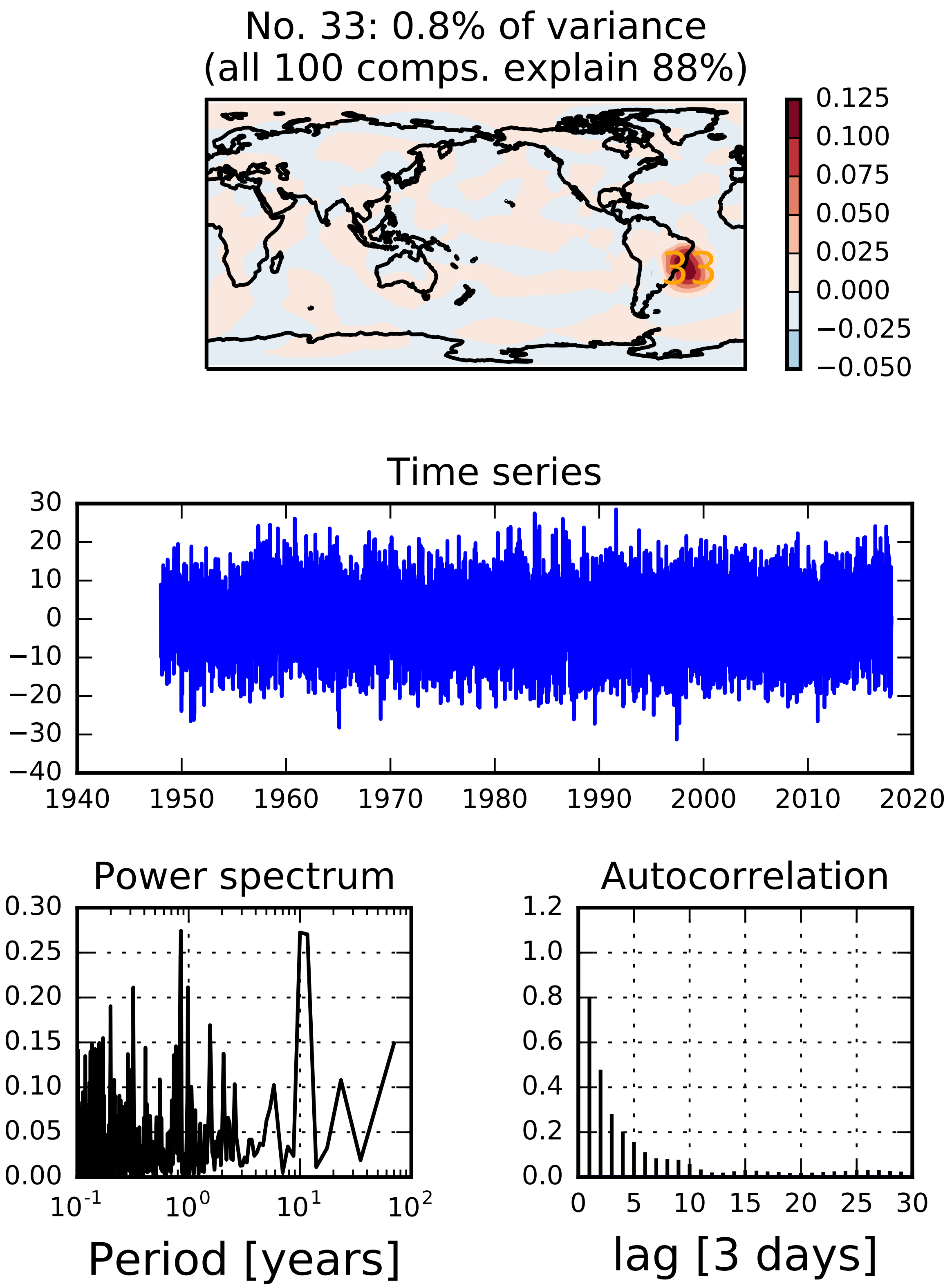
No. 33: 0.8% of variance
(all 100 comps. explain 88%)
0.125
0.100
0.075
0.050
0.025
0.000
−0.025
−0.050
33
Time series
30
20
10
0
−10
−20
−30
−40
1940 1950 1960 1970 1980 1990 2000 2010 2020
Power spectrum
0.30 0.25 0.20 0.15 0.10 0.05 0.00
10^{-1} 10^{0} 10^{1} 10^{2}
Period [years]
Autocorrelation
1.2 1.0 0.8 0.6 0.4 0.2 0.0
0 5 10 15 20 25 30
lag [3 days]

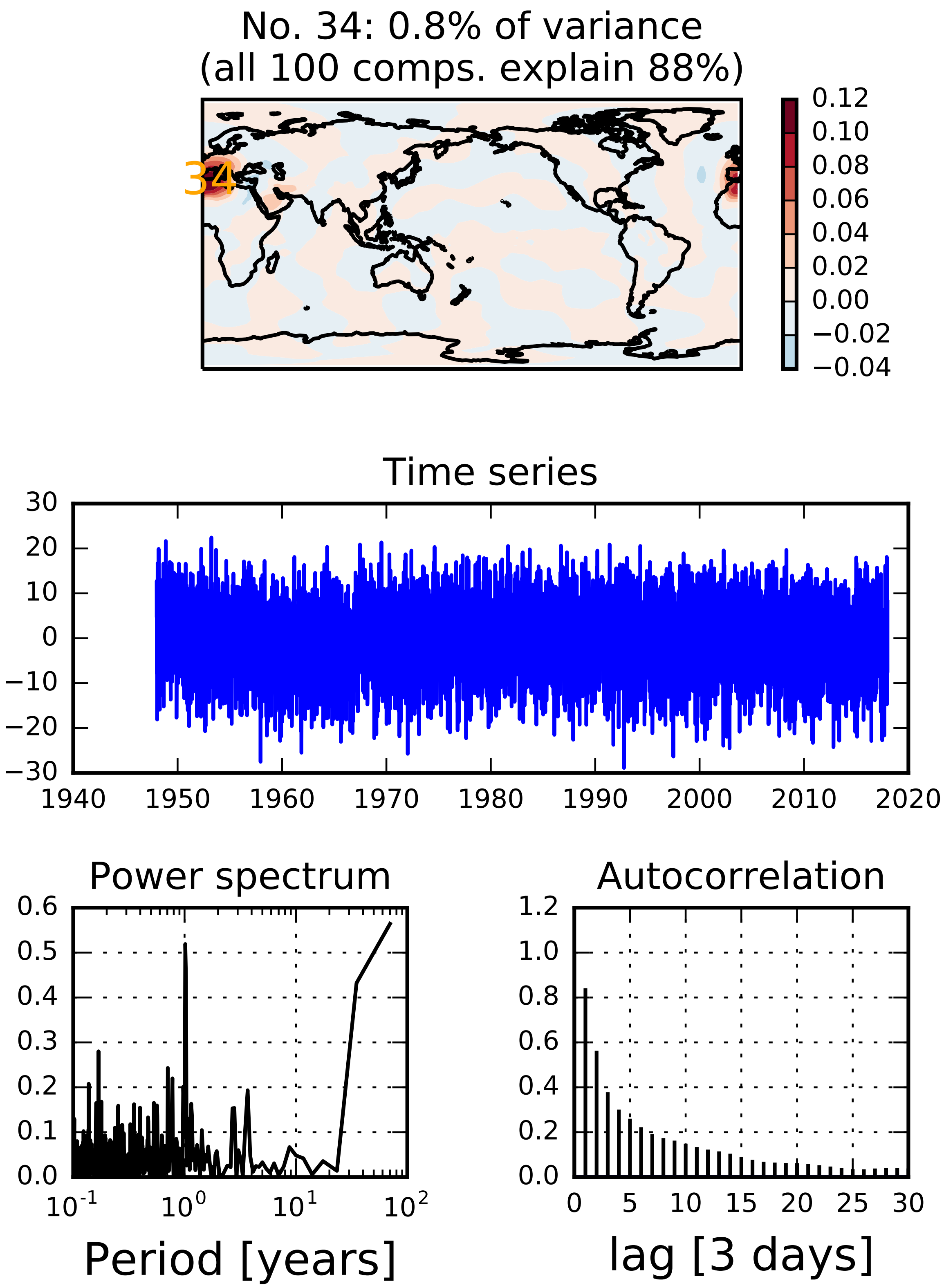
No. 34: 0.8% of variance
(all 100 comps. explain 88%)
34
0.12
0.10
0.08
0.06
0.04
0.02
0.00
−0.02
−0.04
Time series
30
20
10
0
−10
−20
−30
1940
1950
1960
1970
1980
1990
2000
2010
2020
Power spectrum
0.6
0.5
0.4
0.3
0.2
0.1
0.0
10^{-1}
10^{0}
10^{1}
10^{2}
Period [years]
Autocorrelation
1.2
1.0
0.8
0.6
0.4
0.2
0.0
0
5
10
15
20
25
30
lag [3 days]

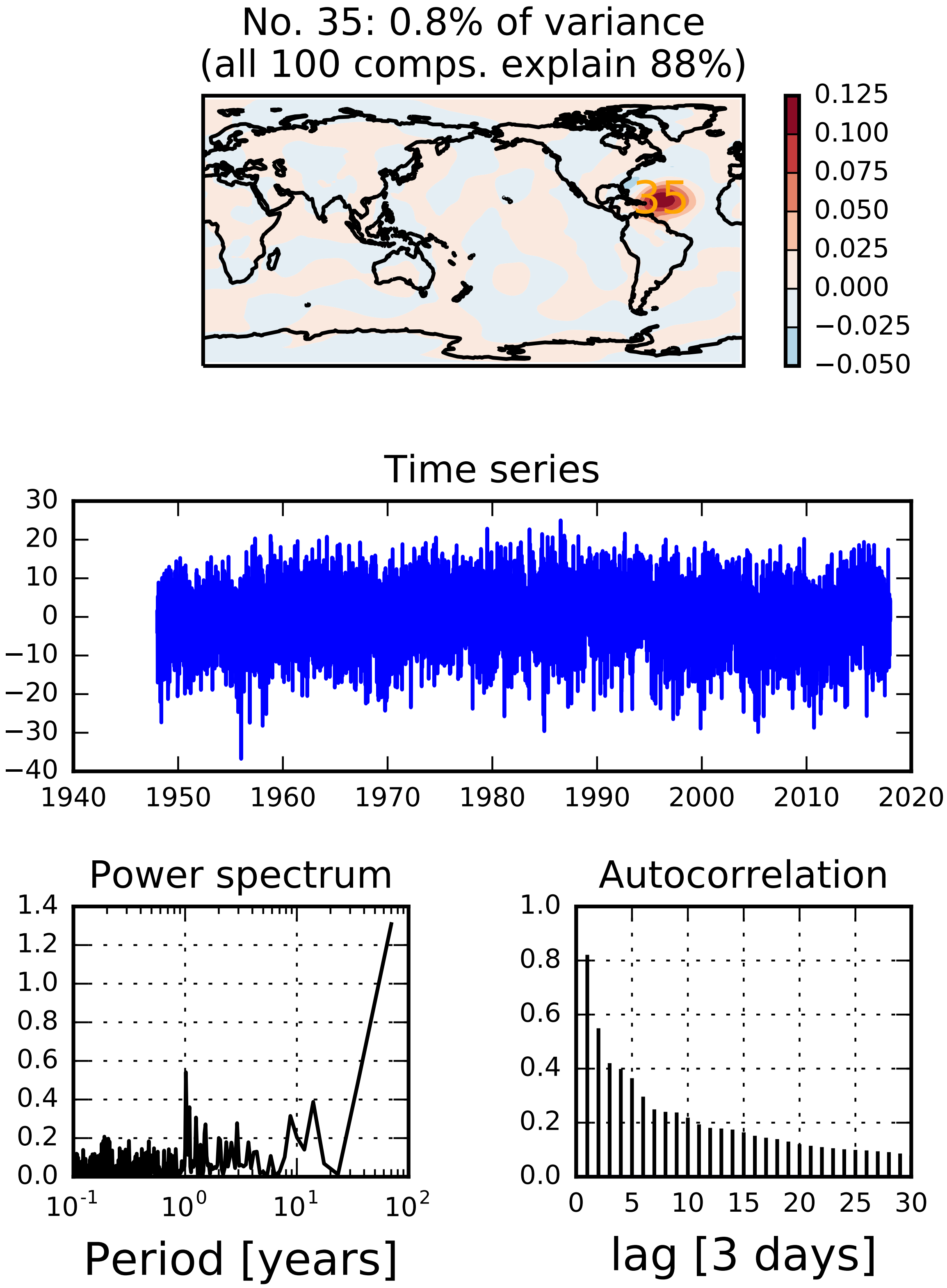
No. 35: 0.8% of variance
(all 100 comps. explain 88%)
35
0.125
0.100
0.075
0.050
0.025
0.000
−0.025
−0.050
Time series
30
20
10
0
−10
−20
−30
−40
1940
1950
1960
1970
1980
1990
2000
2010
2020
Power spectrum
1.4
1.2
1.0
0.8
0.6
0.4
0.2
0.0
10^-1
10^0
10^1
10^2
Period [years]
Autocorrelation
1.0
0.8
0.6
0.4
0.2
0.0
0
5
10
15
20
25
30
lag [3 days]

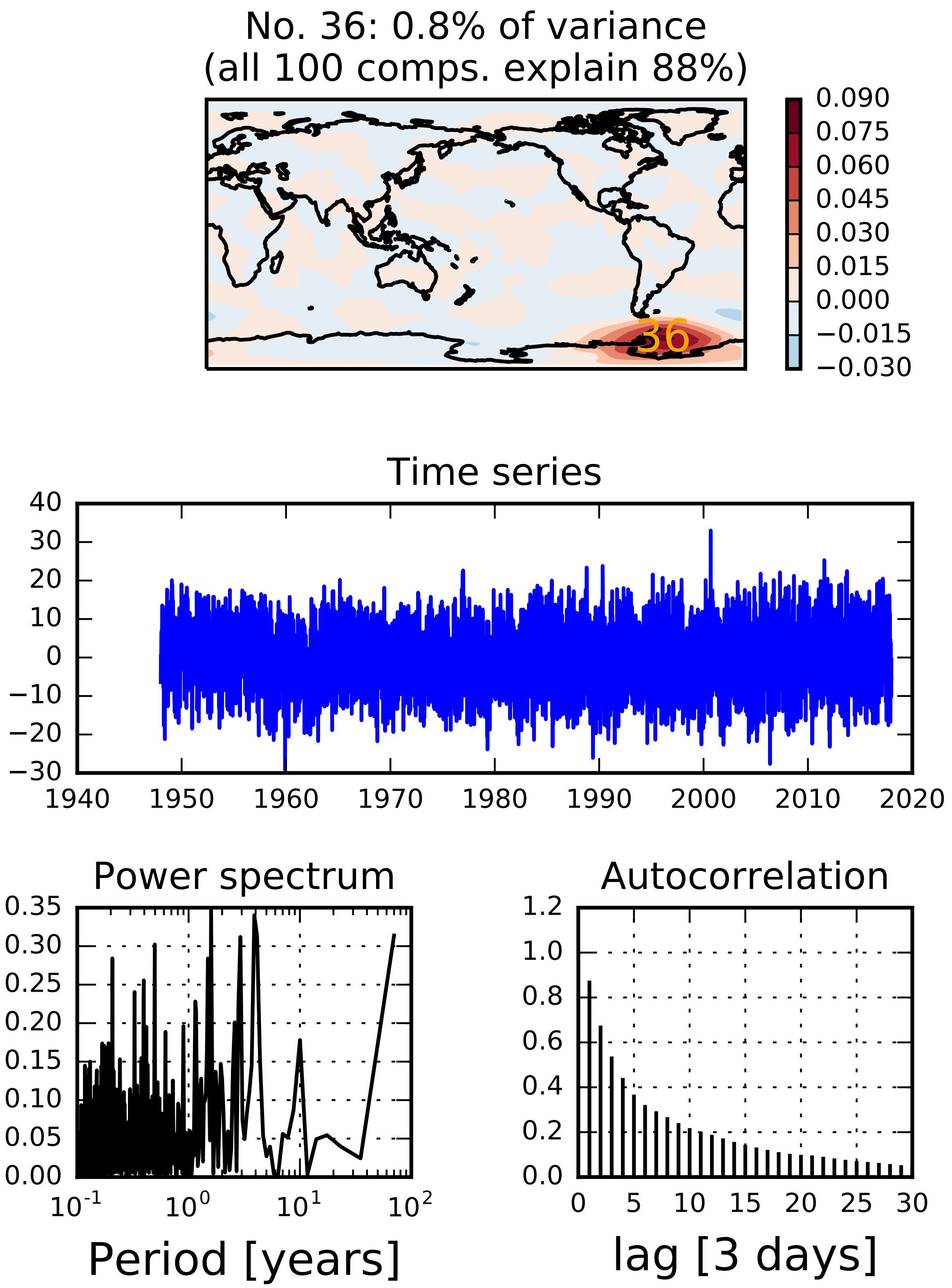
No. 36: 0.8% of variance
(all 100 comps. explain 88%)
36
0.090
0.075
0.060
0.045
0.030
0.015
0.000
−0.015
−0.030
Time series
40
30
20
10
0
−10
−20
−30
1940
1950
1960
1970
1980
1990
2000
2010
2020
Power spectrum
0.35
0.30
0.25
0.20
0.15
0.10
0.05
0.00
10^-1
10^0
10^1
10^2
Period [years]
Autocorrelation
1.2
1.0
0.8
0.6
0.4
0.2
0.0
0
5
10
15
20
25
30
lag [3 days]

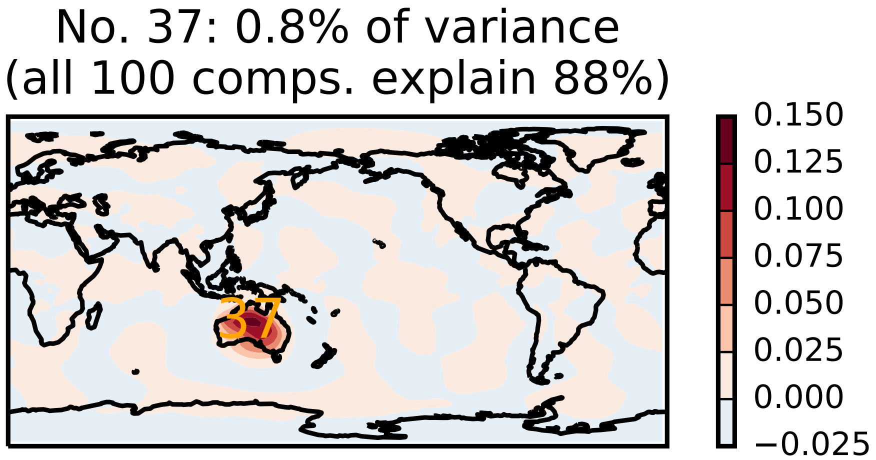
No. 37: 0.8% of variance
(all 100 comps. explain 88%)
37
0.150
0.125
0.100
0.075
0.050
0.025
0.000
−0.025

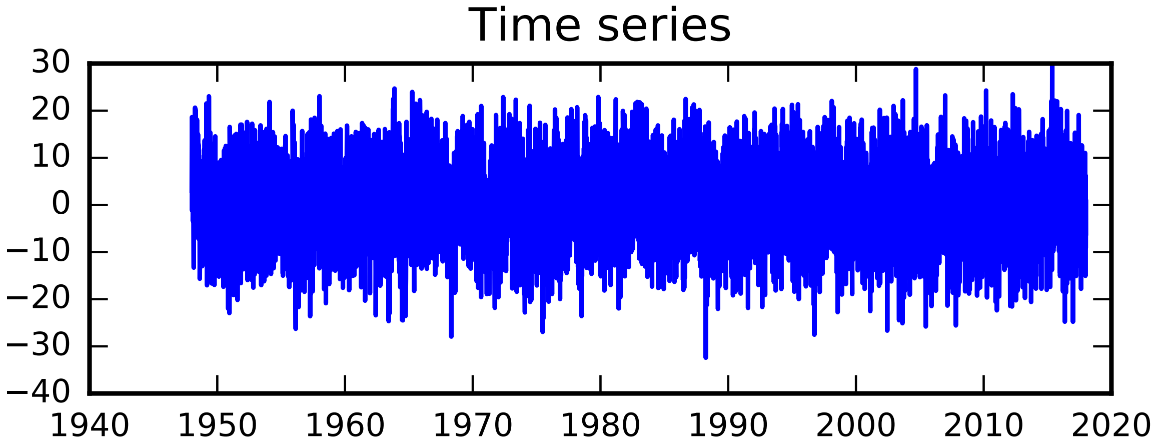
Time series
30
20
10
0
−10
−20
−30
−40
1940
1950
1960
1970
1980
1990
2000
2010
2020

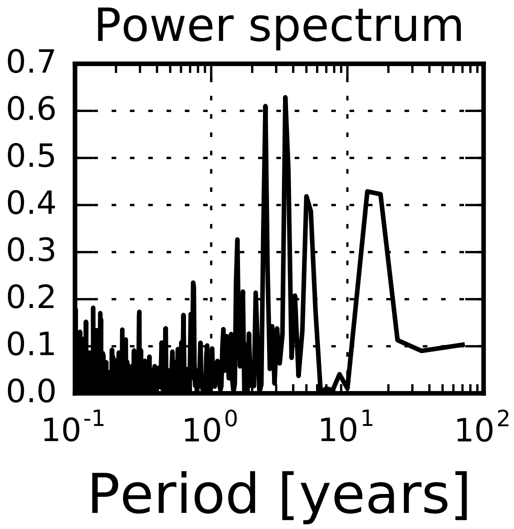
Power spectrum
0.7
0.6
0.5
0.4
0.3
0.2
0.1
0.0
10^-1
10^0
10^1
10^2
Period [years]

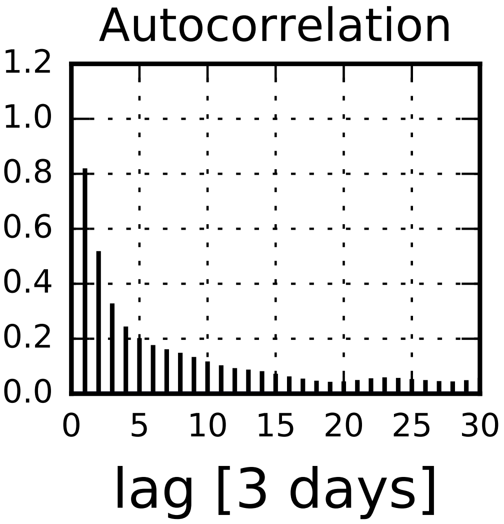
Autocorrelation
1.2
1.0
0.8
0.6
0.4
0.2
0.0
0
5
10
15
20
25
30
lag [3 days]

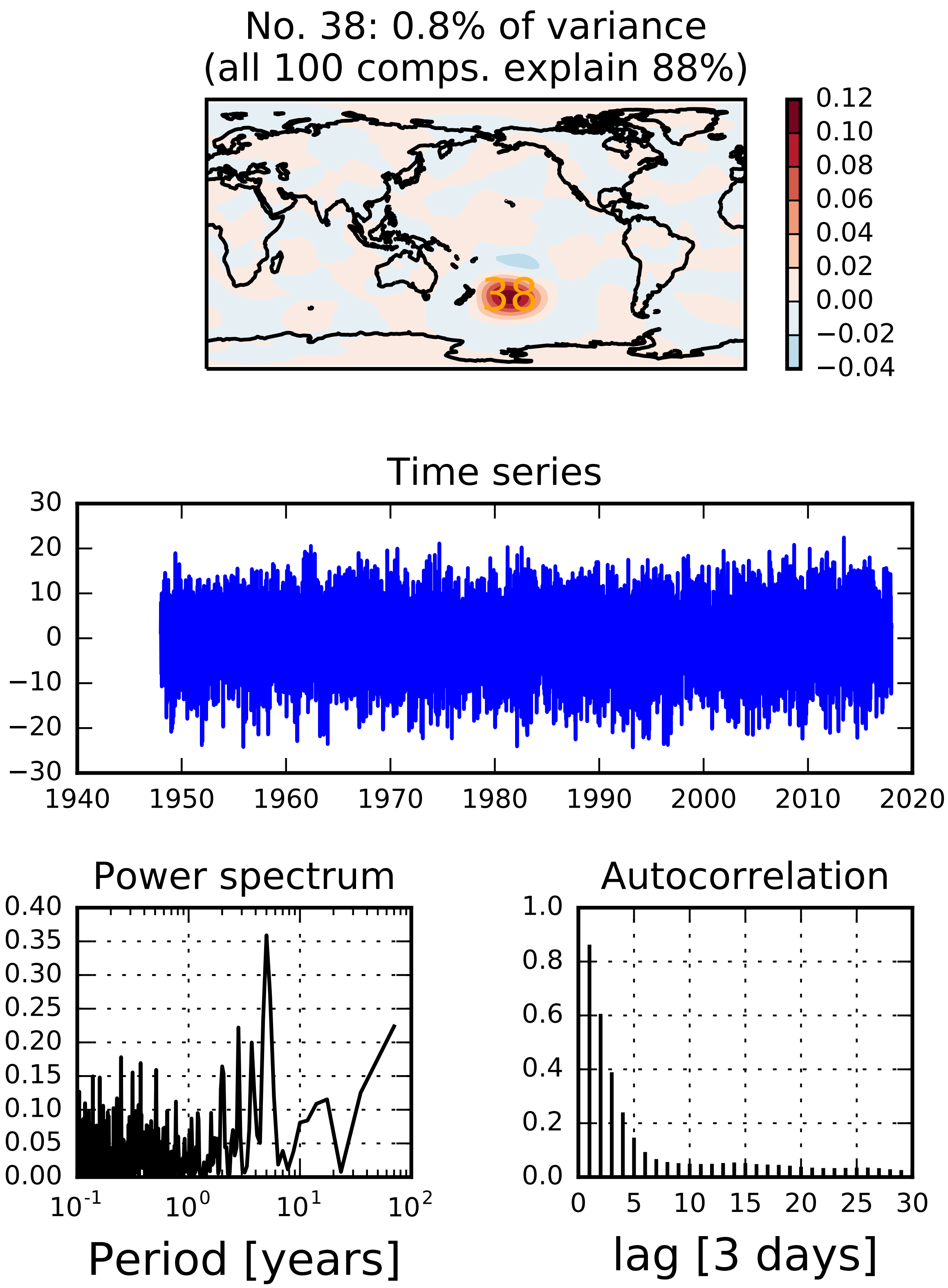
No. 38: 0.8% of variance
(all 100 comps. explain 88%)
38
0.12
0.10
0.08
0.06
0.04
0.02
0.00
−0.02
−0.04
Time series
30
20
10
0
−10
−20
−30
1940
1950
1960
1970
1980
1990
2000
2010
2020
Power spectrum
0.40
0.35
0.30
0.25
0.20
0.15
0.10
0.05
0.00
10^{-1}
10^{0}
10^{1}
10^{2}
Period [years]
Autocorrelation
1.0
0.8
0.6
0.4
0.2
0.0
0
5
10
15
20
25
30
lag [3 days]

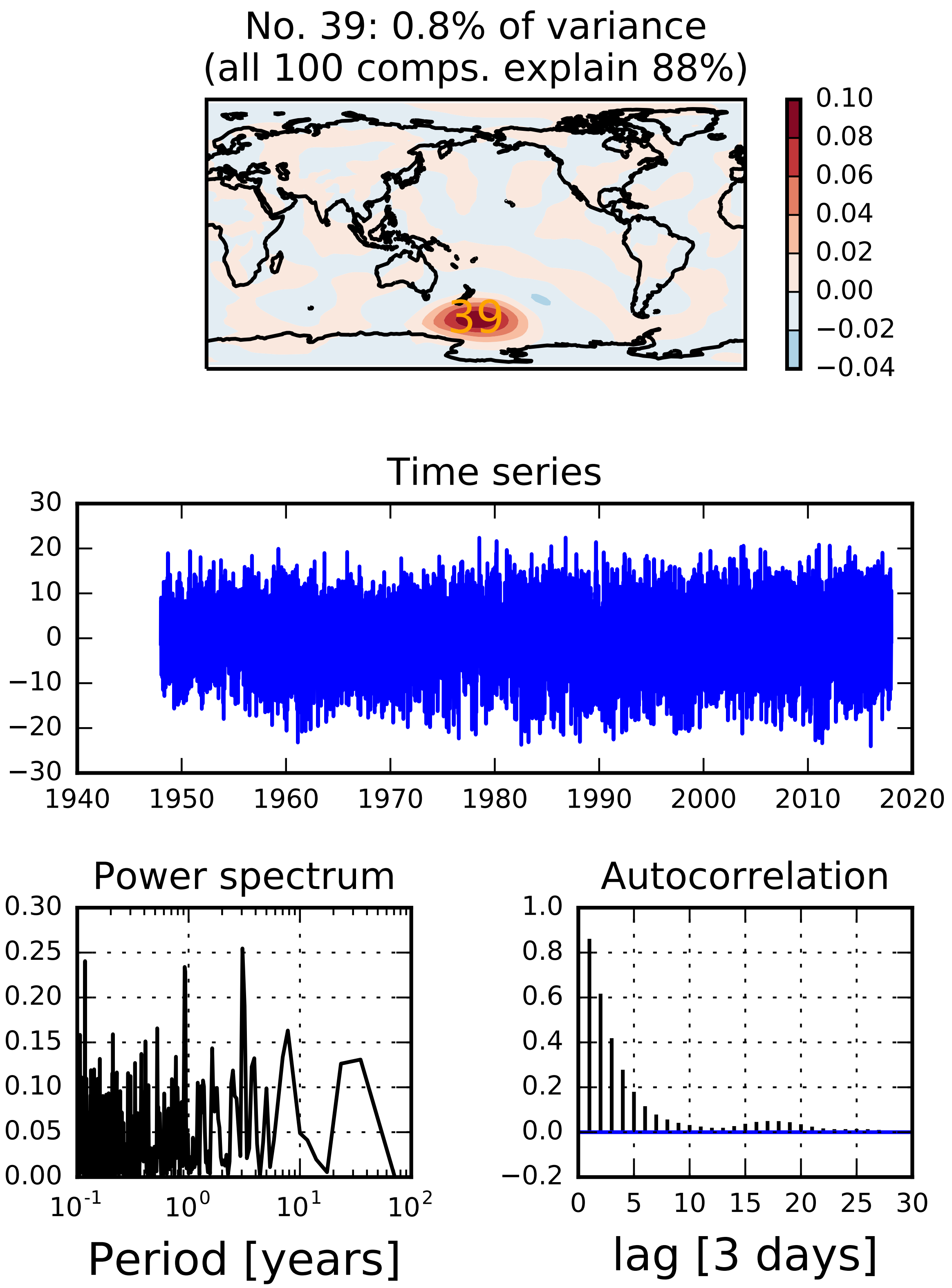
No. 39: 0.8% of variance
(all 100 comps. explain 88%)
39
0.10
0.08
0.06
0.04
0.02
0.00
−0.02
−0.04
Time series
30
20
10
0
−10
−20
−30
1940
1950
1960
1970
1980
1990
2000
2010
2020
Power spectrum
0.30
0.25
0.20
0.15
0.10
0.05
0.00
10^{-1}
10^{0}
10^{1}
10^{2}
Period [years]
Autocorrelation
1.0
0.8
0.6
0.4
0.2
0.0
−0.2
0
5
10
15
20
25
30
lag [3 days]

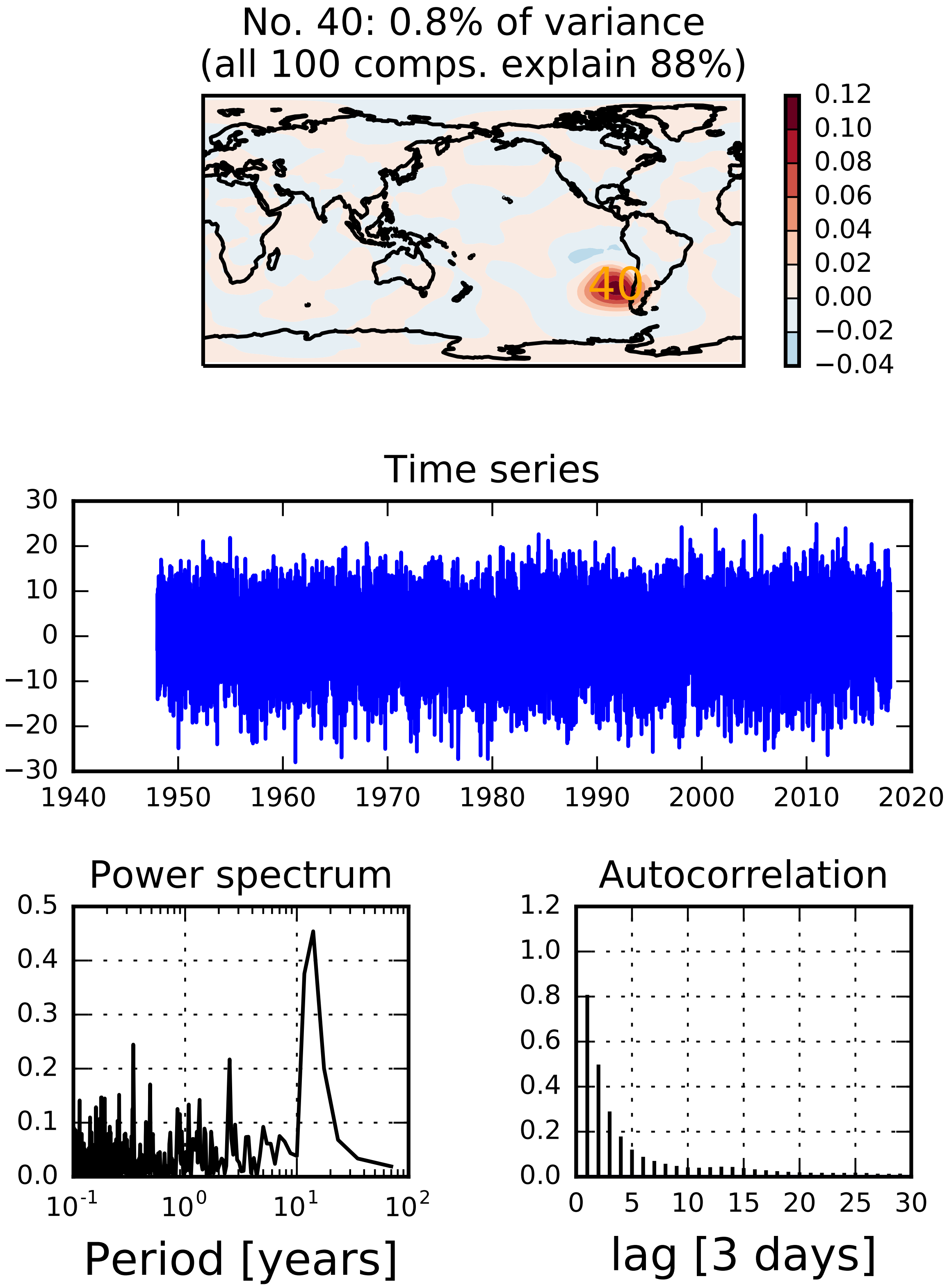
No. 40: 0.8% of variance
(all 100 comps. explain 88%)
40
0.12
0.10
0.08
0.06
0.04
0.02
0.00
−0.02
−0.04
Time series
30
20
10
0
−10
−20
−30
1940
1950
1960
1970
1980
1990
2000
2010
2020
Power spectrum
0.5
0.4
0.3
0.2
0.1
0.0
10^{-1}
10^{0}
10^{1}
10^{2}
Period [years]
Autocorrelation
1.2
1.0
0.8
0.6
0.4
0.2
0.0
0
5
10
15
20
25
30
lag [3 days]

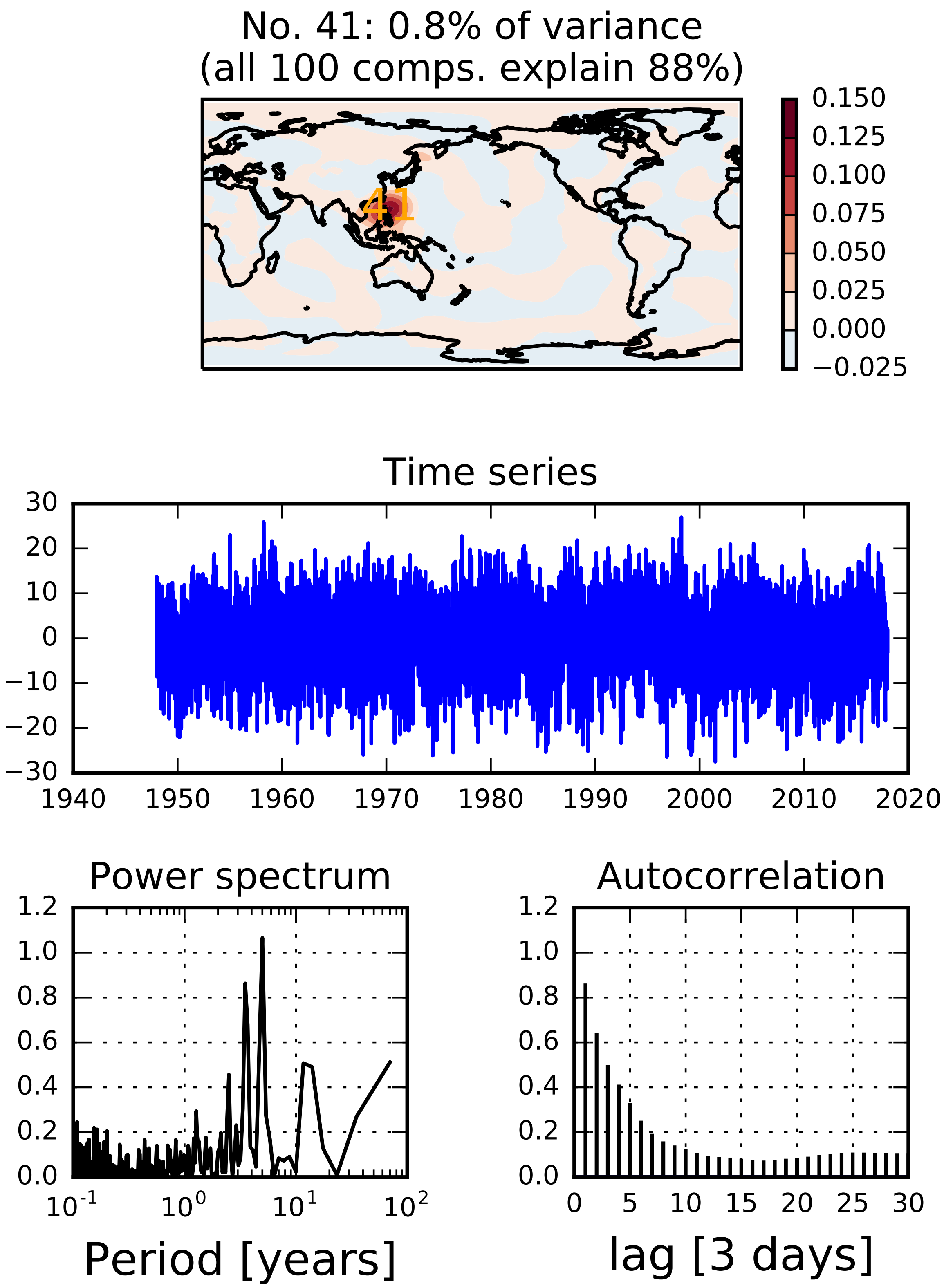
No. 41: 0.8% of variance
(all 100 comps. explain 88%)
41
0.150
0.125
0.100
0.075
0.050
0.025
0.000
−0.025
Time series
30
20
10
0
−10
−20
−30
1940
1950
1960
1970
1980
1990
2000
2010
2020
Power spectrum
1.2
1.0
0.8
0.6
0.4
0.2
0.0
10^{-1}
10^{0}
10^{1}
10^{2}
Period [years]
Autocorrelation
0
5
10
15
20
25
30
lag [3 days]

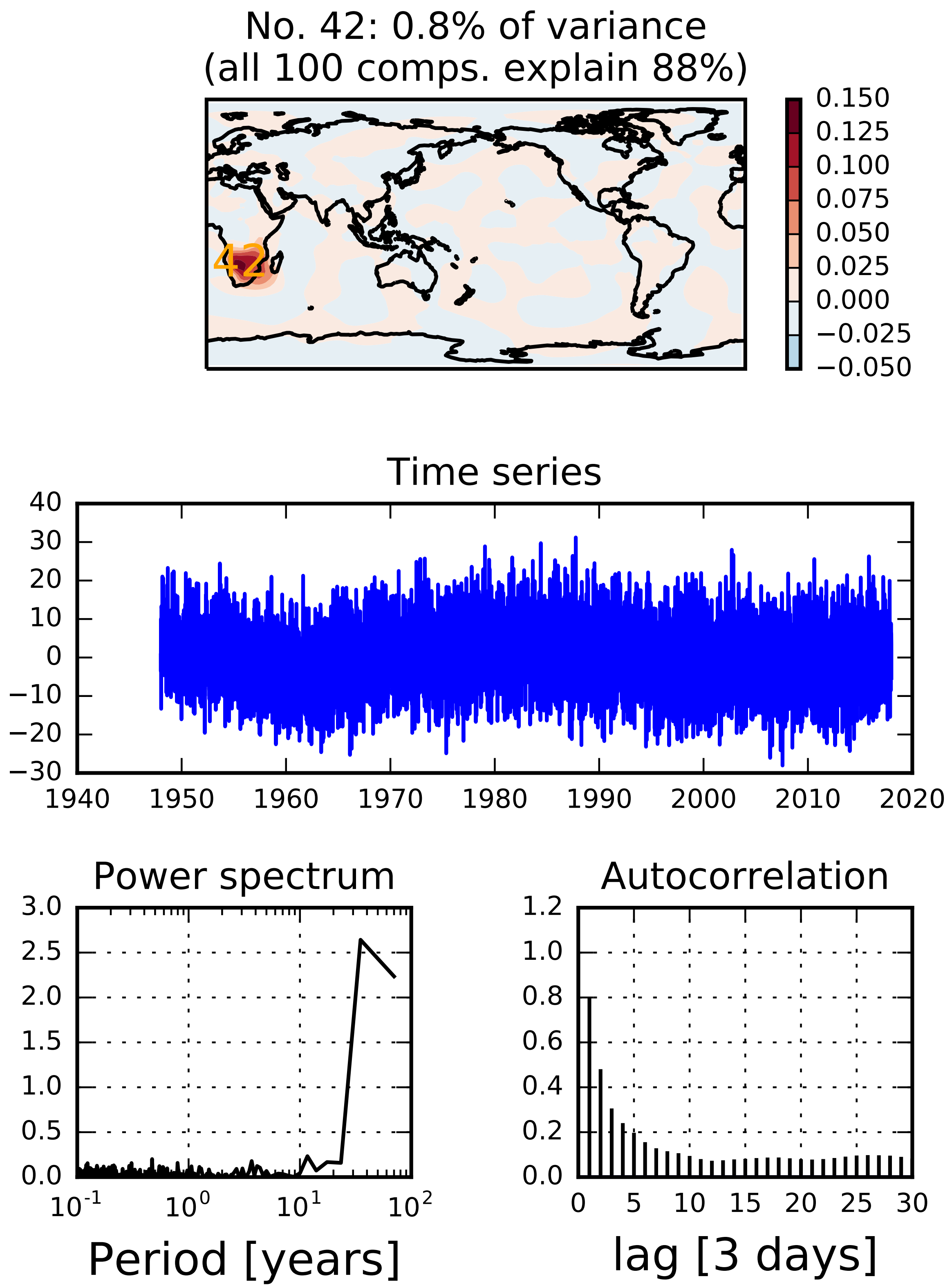
No. 42: 0.8% of variance
(all 100 comps. explain 88%)
42
0.150
0.125
0.100
0.075
0.050
0.025
0.000
−0.025
−0.050
Time series
40
30
20
10
0
−10
−20
−30
1940
1950
1960
1970
1980
1990
2000
2010
2020
Power spectrum
3.0
2.5
2.0
1.5
1.0
0.5
0.0
10^-1
10^0
10^1
10^2
Period [years]
Autocorrelation
1.2
1.0
0.8
0.6
0.4
0.2
0.0
0
5
10
15
20
25
30
lag [3 days]

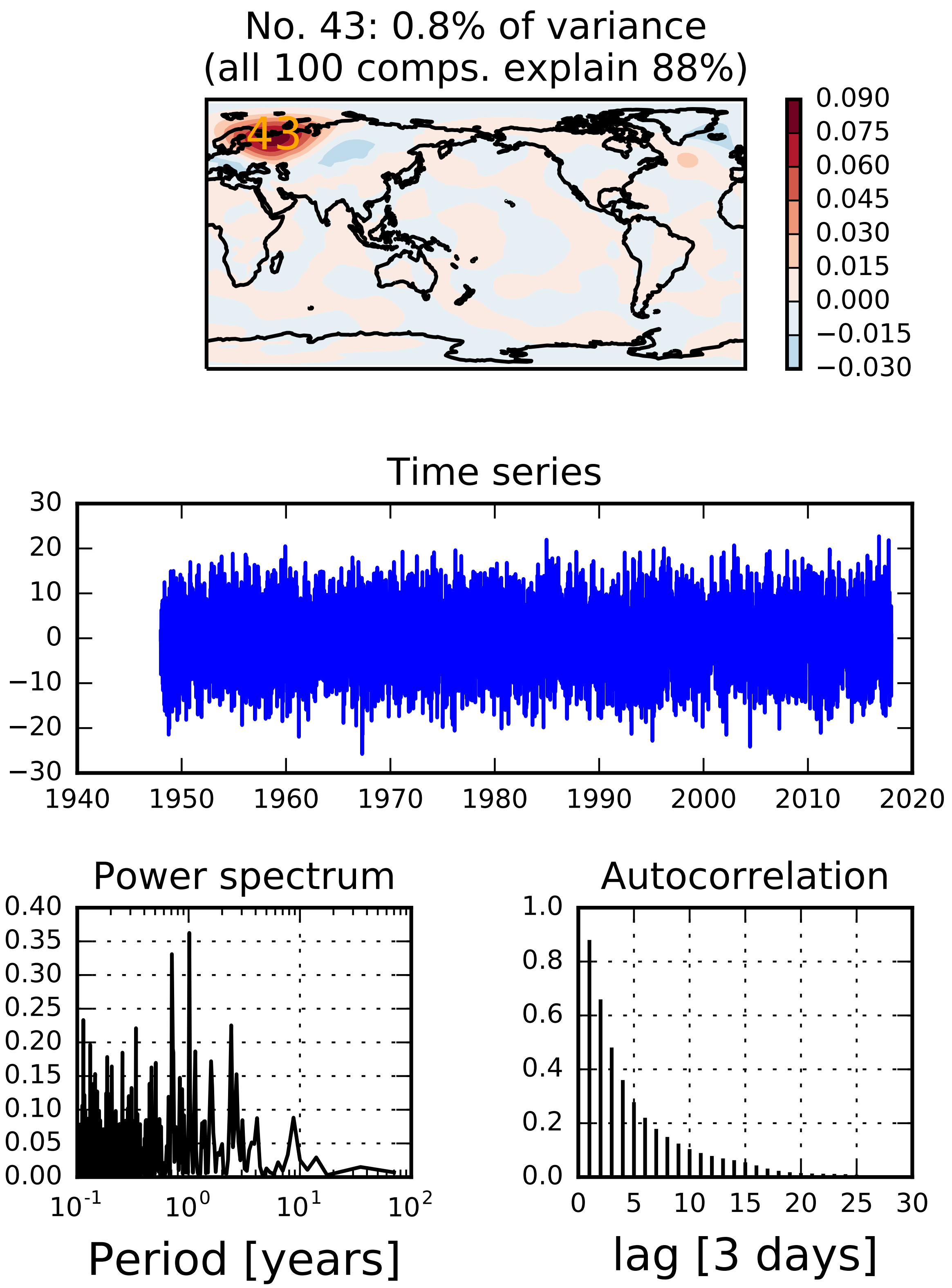
No. 43: 0.8% of variance
(all 100 comps. explain 88%)
43
0.090
0.075
0.060
0.045
0.030
0.015
0.000
−0.015
−0.030
Time series
30
20
10
0
−10
−20
−30
1940
1950
1960
1970
1980
1990
2000
2010
2020
Power spectrum
0.40
0.35
0.30
0.25
0.20
0.15
0.10
0.05
0.00
10^{-1}
10^{0}
10^{1}
10^{2}
Period [years]
Autocorrelation
1.0
0.8
0.6
0.4
0.2
0.0
0
5
10
15
20
25
30
lag [3 days]

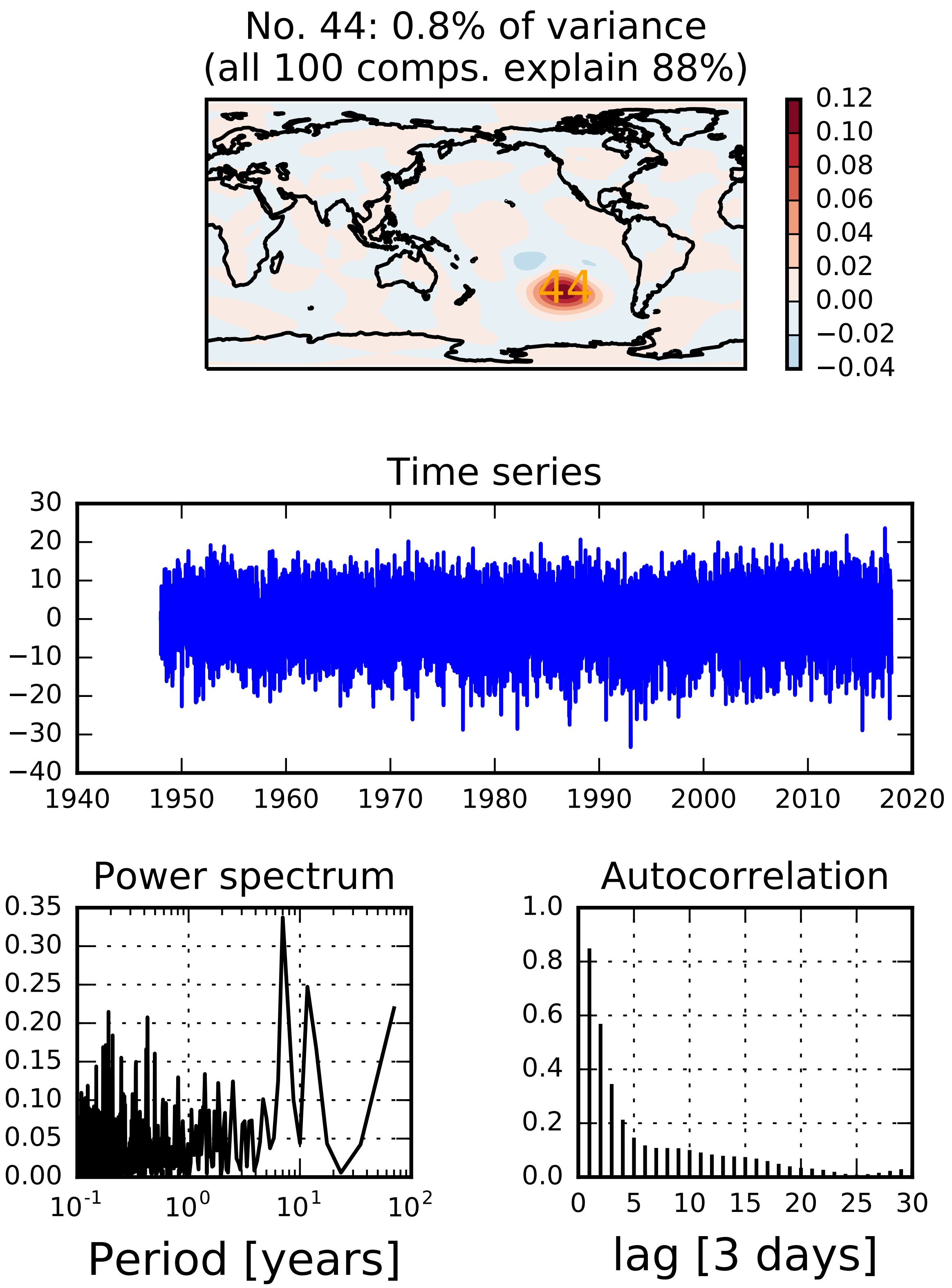
No. 44: 0.8% of variance
(all 100 comps. explain 88%)
44
0.12
0.10
0.08
0.06
0.04
0.02
0.00
−0.02
−0.04
Time series
30
20
10
0
−10
−20
−30
−40
1940
1950
1960
1970
1980
1990
2000
2010
2020
Power spectrum
0.35
0.30
0.25
0.20
0.15
0.10
0.05
0.00
10^{-1}
10^{0}
10^{1}
10^{2}
Period [years]
Autocorrelation
1.0
0.8
0.6
0.4
0.2
0.0
0
5
10
15
20
25
30
lag [3 days]

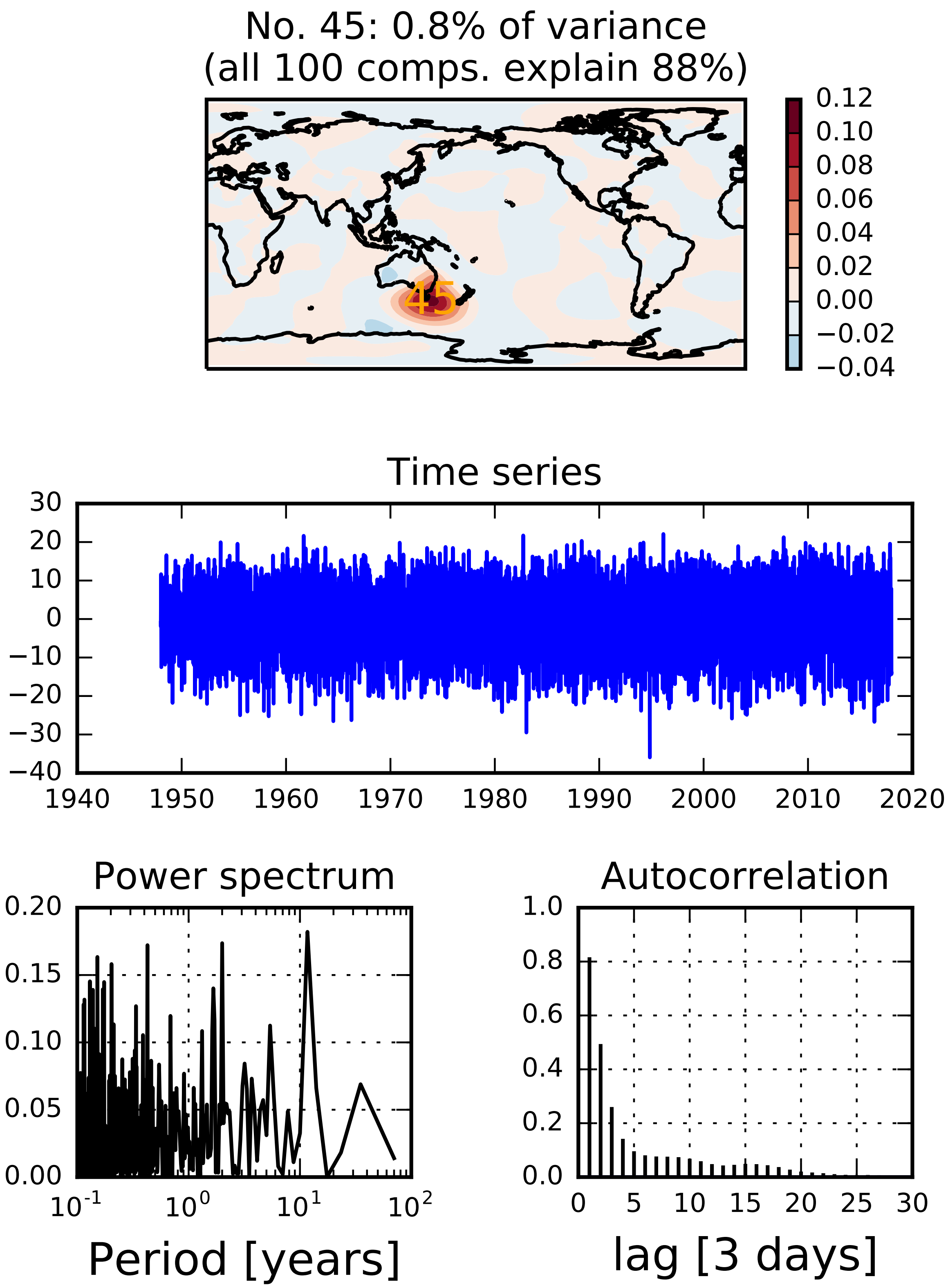
No. 45: 0.8% of variance
(all 100 comps. explain 88%)
45
0.12
0.10
0.08
0.06
0.04
0.02
0.00
−0.02
−0.04
Time series
30
20
10
0
−10
−20
−30
−40
1940
1950
1960
1970
1980
1990
2000
2010
2020
Power spectrum
0.20
0.15
0.10
0.05
0.00
10^-1
10^0
10^1
10^2
Period [years]
Autocorrelation
1.0
0.8
0.6
0.4
0.2
0.0
0
5
10
15
20
25
30
lag [3 days]

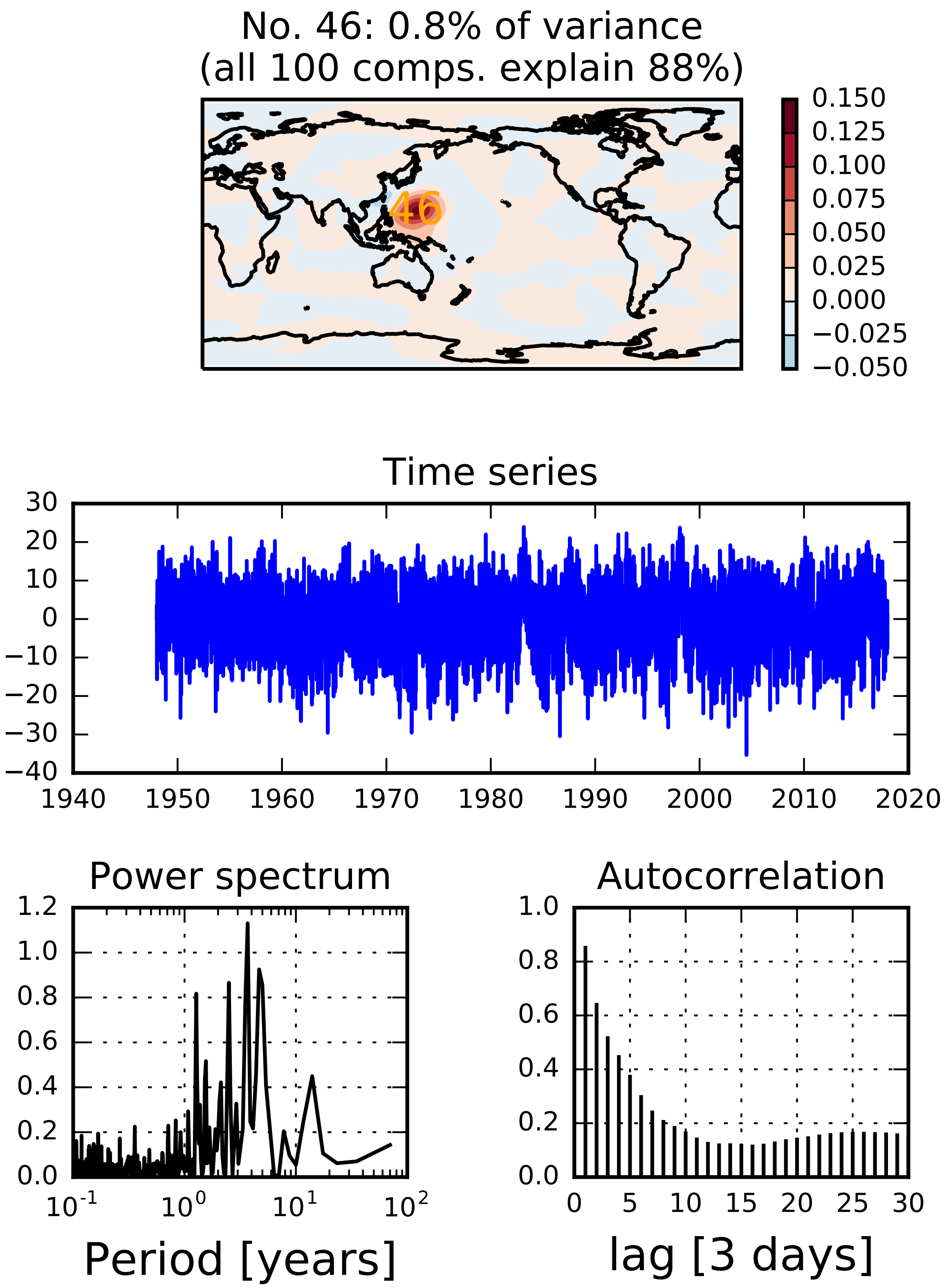
No. 46: 0.8% of variance
(all 100 comps. explain 88%)
46
0.150
0.125
0.100
0.075
0.050
0.025
0.000
−0.025
−0.050
Time series
30
20
10
0
−10
−20
−30
−40
1940
1950
1960
1970
1980
1990
2000
2010
2020
Power spectrum
1.2
1.0
0.8
0.6
0.4
0.2
0.0
10^{-1}
10^{0}
10^{1}
10^{2}
Period [years]
Autocorrelation
1.0
0.8
0.6
0.4
0.2
0.0
0
5
10
15
20
25
30
lag [3 days]

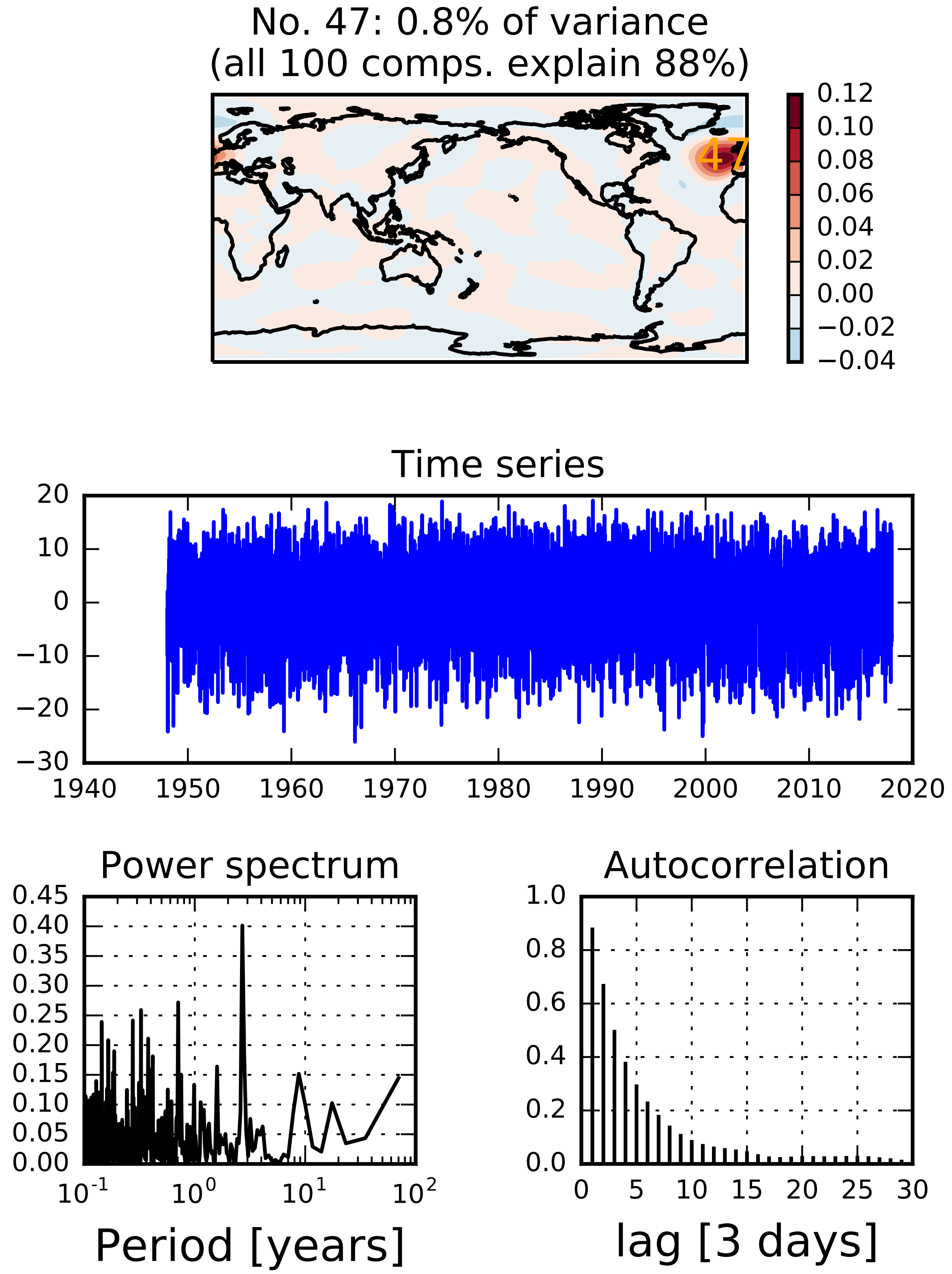
No. 47: 0.8% of variance
(all 100 comps. explain 88%)
47
0.12
0.10
0.08
0.06
0.04
0.02
0.00
−0.02
−0.04
Time series
20
10
0
−10
−20
−30
1940
1950
1960
1970
1980
1990
2000
2010
2020
Power spectrum
0.45
0.40
0.35
0.30
0.25
0.20
0.15
0.10
0.05
0.00
10^{-1}
10^{0}
10^{1}
10^{2}
Period [years]
Autocorrelation
1.0
0.8
0.6
0.4
0.2
0.0
0
5
10
15
20
25
30
lag [3 days]

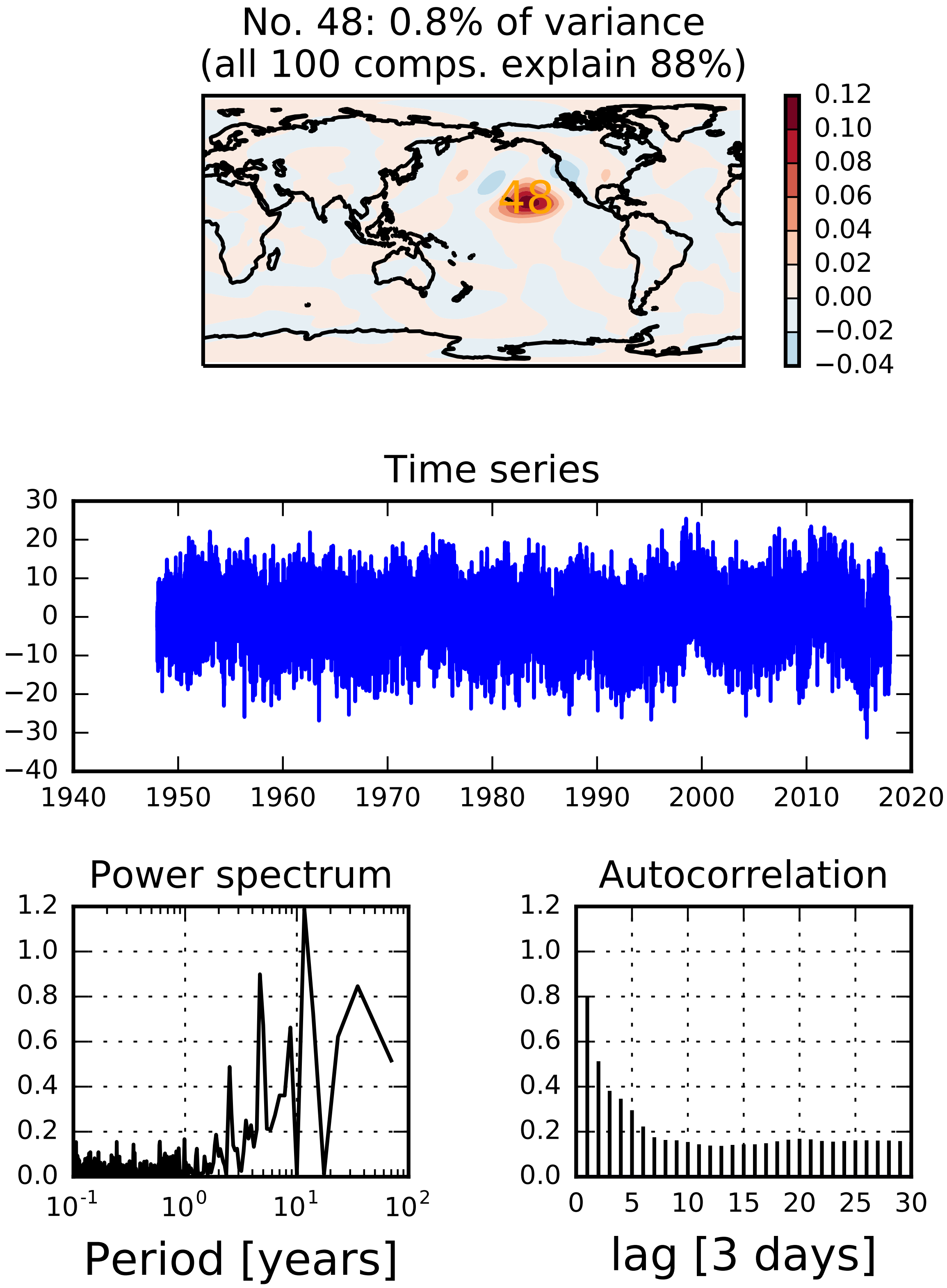
No. 48: 0.8% of variance
(all 100 comps. explain 88%)
48
0.12
0.10
0.08
0.06
0.04
0.02
0.00
−0.02
−0.04
Time series
30
20
10
0
−10
−20
−30
−40
1940
1950
1960
1970
1980
1990
2000
2010
2020
Power spectrum
1.2
1.0
0.8
0.6
0.4
0.2
0.0
10^{-1}
10^{0}
10^{1}
10^{2}
Period [years]
Autocorrelation
1.2
1.0
0.8
0.6
0.4
0.2
0.0
0
5
10
15
20
25
30
lag [3 days]

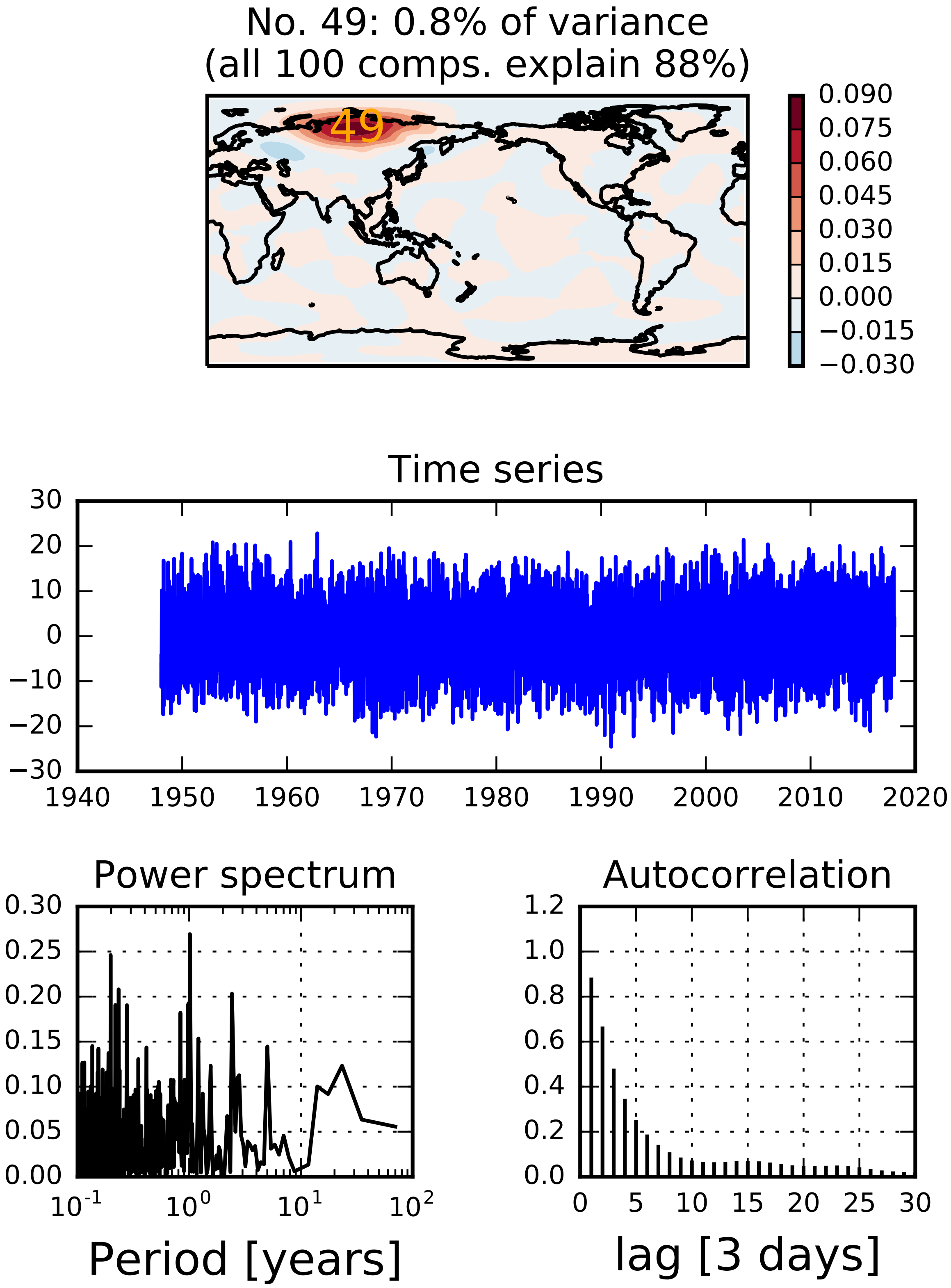
No. 49: 0.8% of variance
(all 100 comps. explain 88%)
49
0.090
0.075
0.060
0.045
0.030
0.015
0.000
−0.015
−0.030
Time series
30
20
10
0
−10
−20
−30
1940
1950
1960
1970
1980
1990
2000
2010
2020
Power spectrum
0.30
0.25
0.20
0.15
0.10
0.05
0.00
10^{-1}
10^{0}
10^{1}
10^{2}
Period [years]
Autocorrelation
1.2
1.0
0.8
0.6
0.4
0.2
0.0
0
5
10
15
20
25
30
lag [3 days]

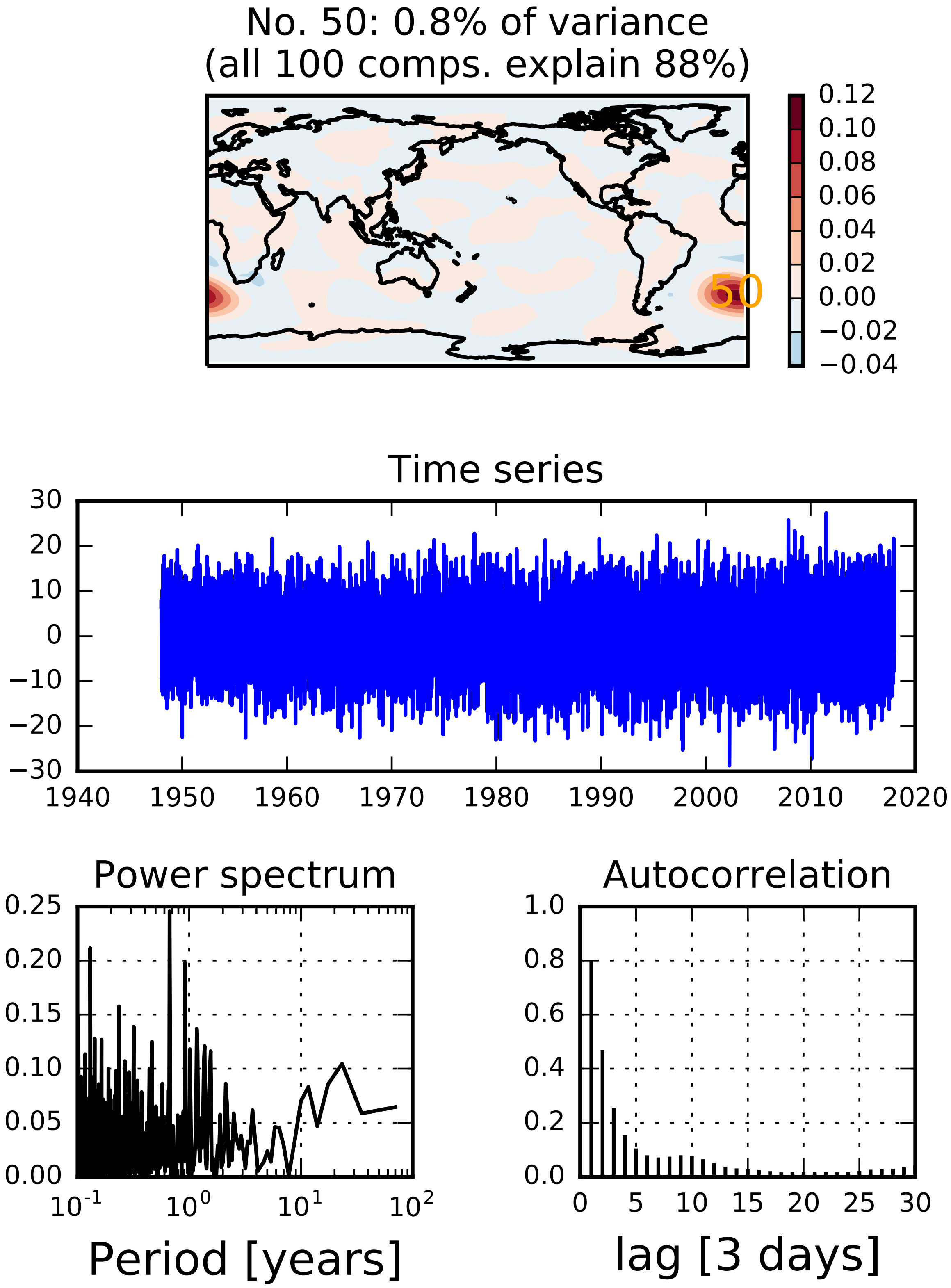
No. 50: 0.8% of variance
(all 100 comps. explain 88%)
50
0.12
0.10
0.08
0.06
0.04
0.02
0.00
−0.02
−0.04
Time series
30
20
10
0
−10
−20
−30
1940
1950
1960
1970
1980
1990
2000
2010
2020
Power spectrum
0.25
0.20
0.15
0.10
0.05
0.00
10^{-1}
10^{0}
10^{1}
10^{2}
Period [years]
Autocorrelation
1.0
0.8
0.6
0.4
0.2
0.0
0
5
10
15
20
25
30
lag [3 days]

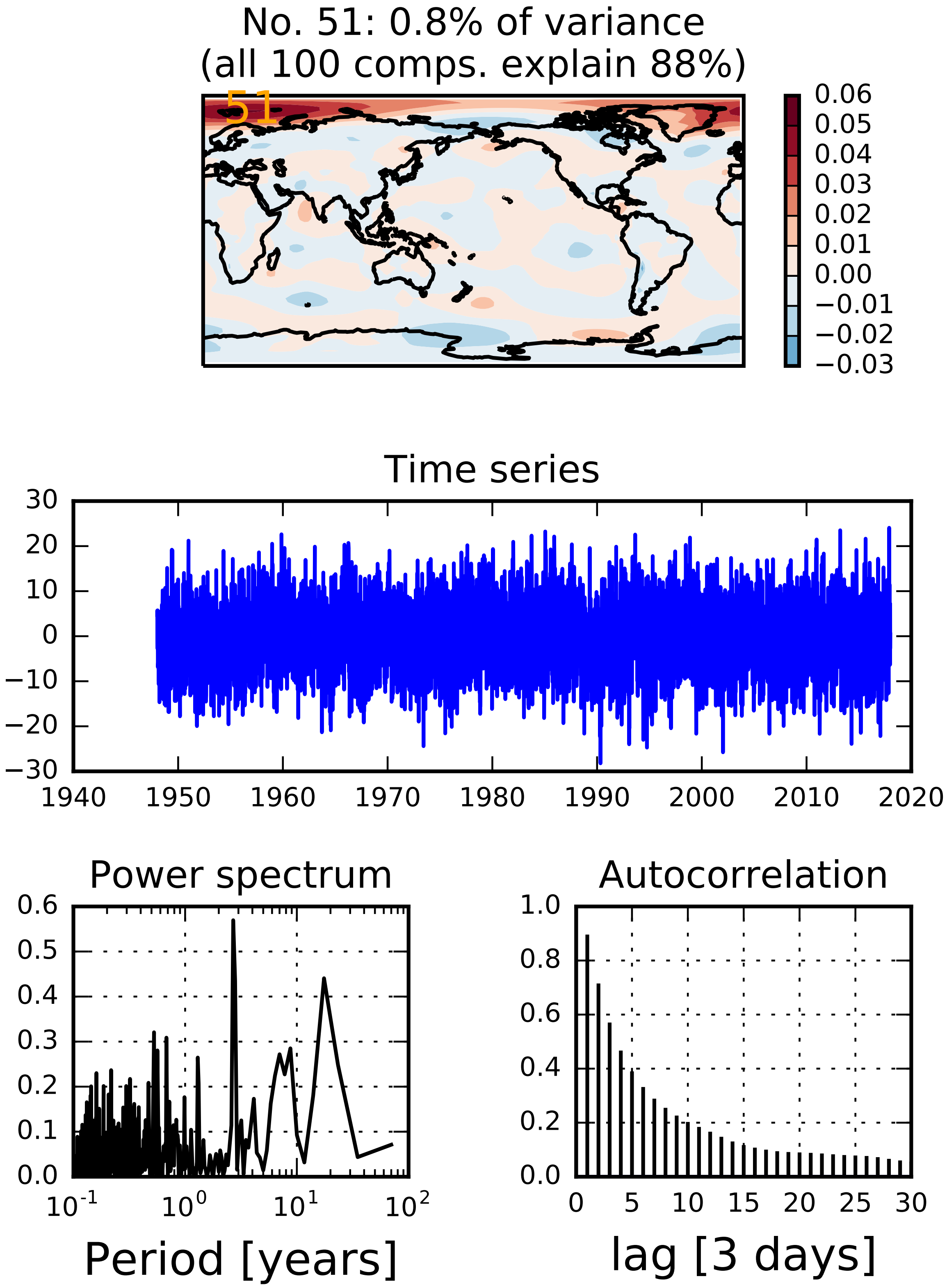
No. 51: 0.8% of variance
(all 100 comps. explain 88%)
51
0.06
0.05
0.04
0.03
0.02
0.01
0.00
−0.01
−0.02
−0.03
Time series
30
20
10
0
−10
−20
−30
1940
1950
1960
1970
1980
1990
2000
2010
2020
Power spectrum
0.6
0.5
0.4
0.3
0.2
0.1
0.0
10^{-1}
10^{0}
10^{1}
10^{2}
Period [years]
Autocorrelation
1.0
0.8
0.6
0.4
0.2
0.0
0
5
10
15
20
25
30
lag [3 days]

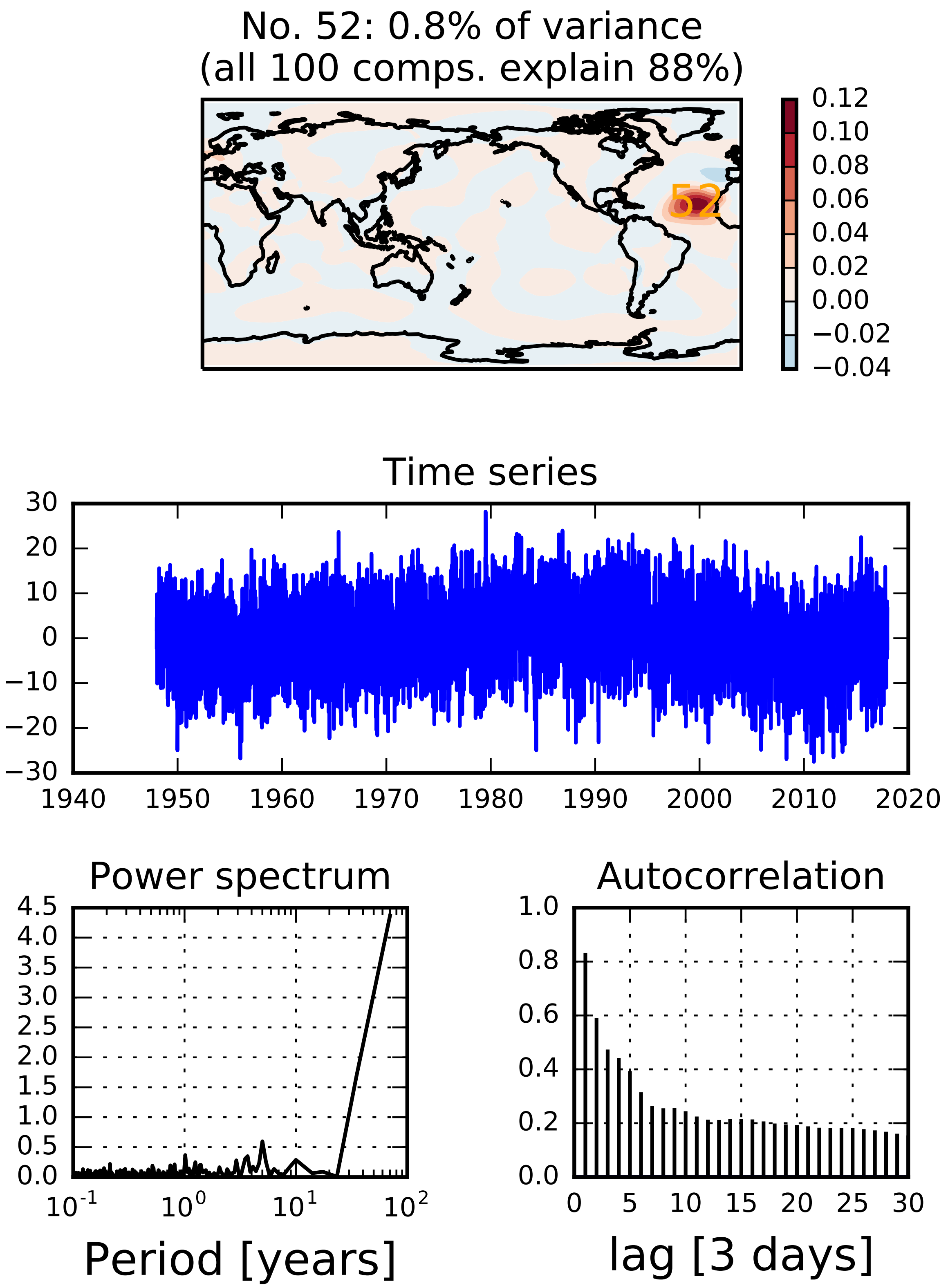
No. 52: 0.8% of variance
(all 100 comps. explain 88%)
52
0.12
0.10
0.08
0.06
0.04
0.02
0.00
−0.02
−0.04
Time series
30
20
10
0
−10
−20
−30
1940
1950
1960
1970
1980
1990
2000
2010
2020
Power spectrum
4.5
4.0
3.5
3.0
2.5
2.0
1.5
1.0
0.5
0.0
10^-1
10^0
10^1
10^2
Period [years]
Autocorrelation
1.0
0.8
0.6
0.4
0.2
0.0
0
5
10
15
20
25
30
lag [3 days]

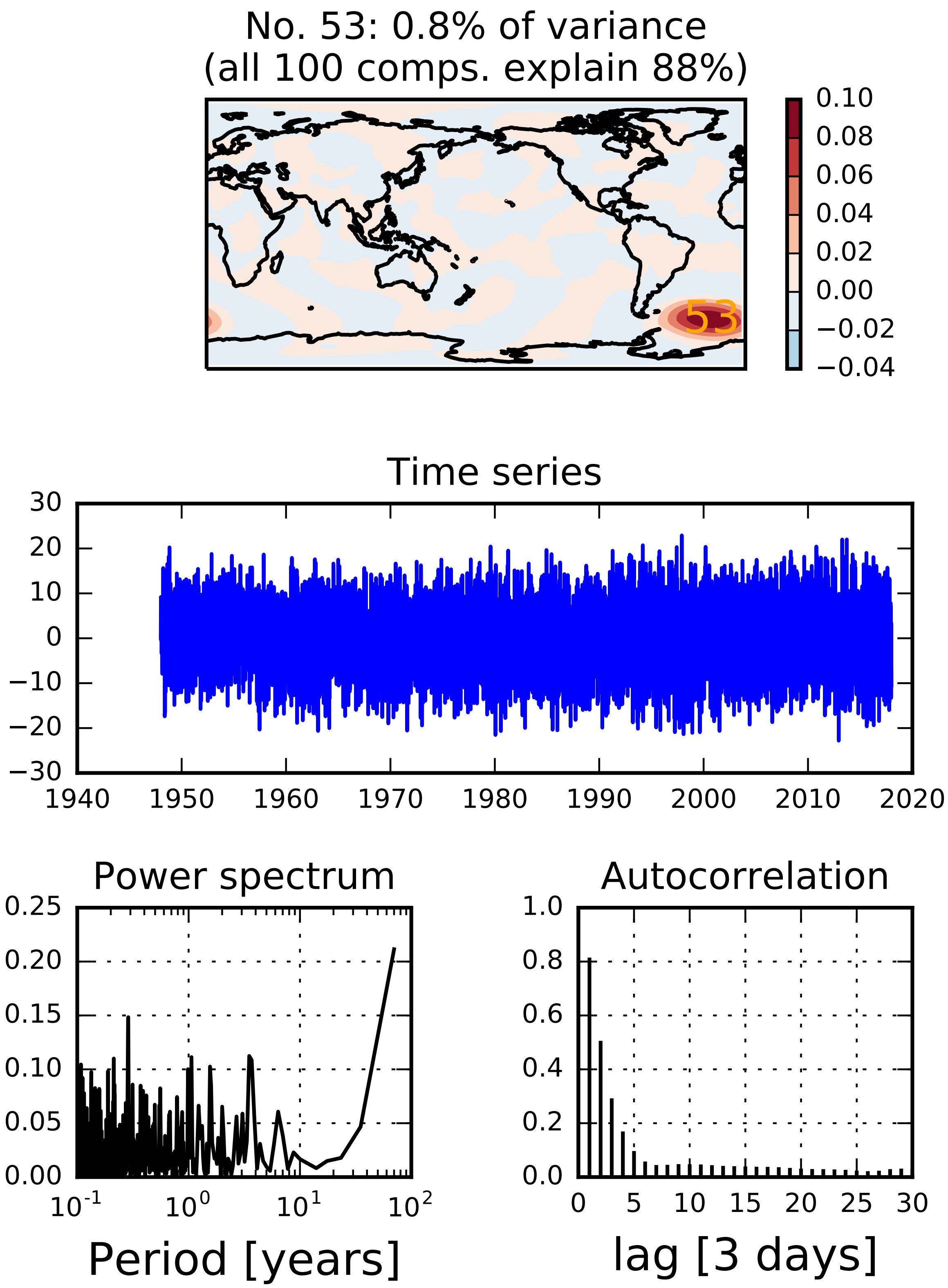
No. 53: 0.8% of variance
(all 100 comps. explain 88%)
0.10
0.08
0.06
0.04
0.02
0.00
−0.02
−0.04
53
Time series
30
20
10
0
−10
−20
−30
1940
1950
1960
1970
1980
1990
2000
2010
2020
Power spectrum
0.25
0.20
0.15
0.10
0.05
0.00
10^{-1}
10^{0}
10^{1}
10^{2}
Period [years]
Autocorrelation
1.0
0.8
0.6
0.4
0.2
0.0
0
5
10
15
20
25
30
lag [3 days]

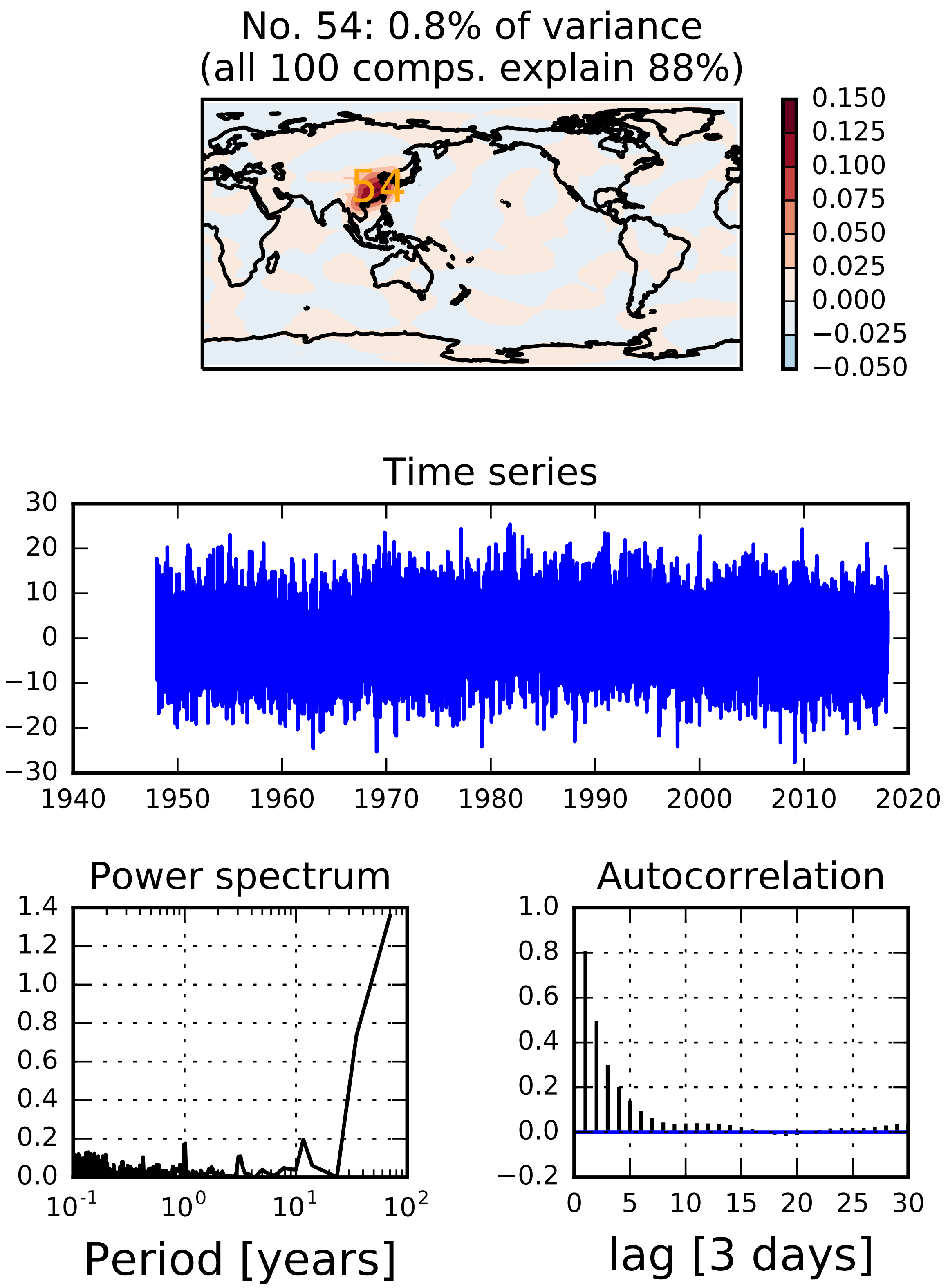
No. 54: 0.8% of variance
(all 100 comps. explain 88%)
54
0.150
0.125
0.100
0.075
0.050
0.025
0.000
−0.025
−0.050
Time series
30
20
10
0
−10
−20
−30
1940
1950
1960
1970
1980
1990
2000
2010
2020
Power spectrum
1.4
1.2
1.0
0.8
0.6
0.4
0.2
0.0
10^{-1}
10^{0}
10^{1}
10^{2}
Period [years]
Autocorrelation
1.0
0.8
0.6
0.4
0.2
0.0
−0.2
0
5
10
15
20
25
30
lag [3 days]

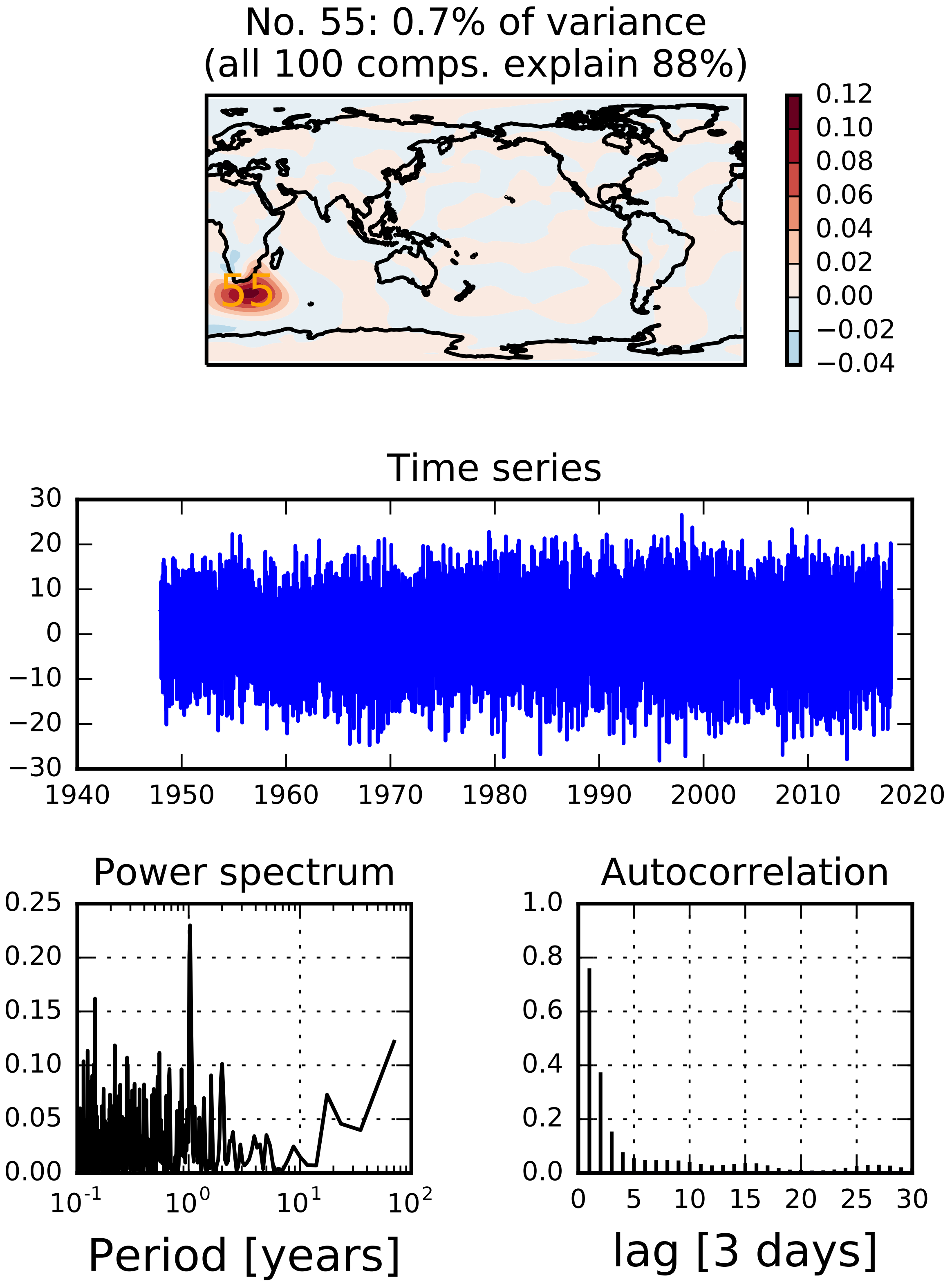
No. 55: 0.7% of variance
(all 100 comps. explain 88%)
55
0.12
0.10
0.08
0.06
0.04
0.02
0.00
−0.02
−0.04
Time series
30
20
10
0
−10
−20
−30
1940
1950
1960
1970
1980
1990
2000
2010
2020
Power spectrum
0.25
0.20
0.15
0.10
0.05
0.00
10^{-1}
10^{0}
10^{1}
10^{2}
Period [years]
Autocorrelation
1.0
0.8
0.6
0.4
0.2
0.0
0
5
10
15
20
25
30
lag [3 days]

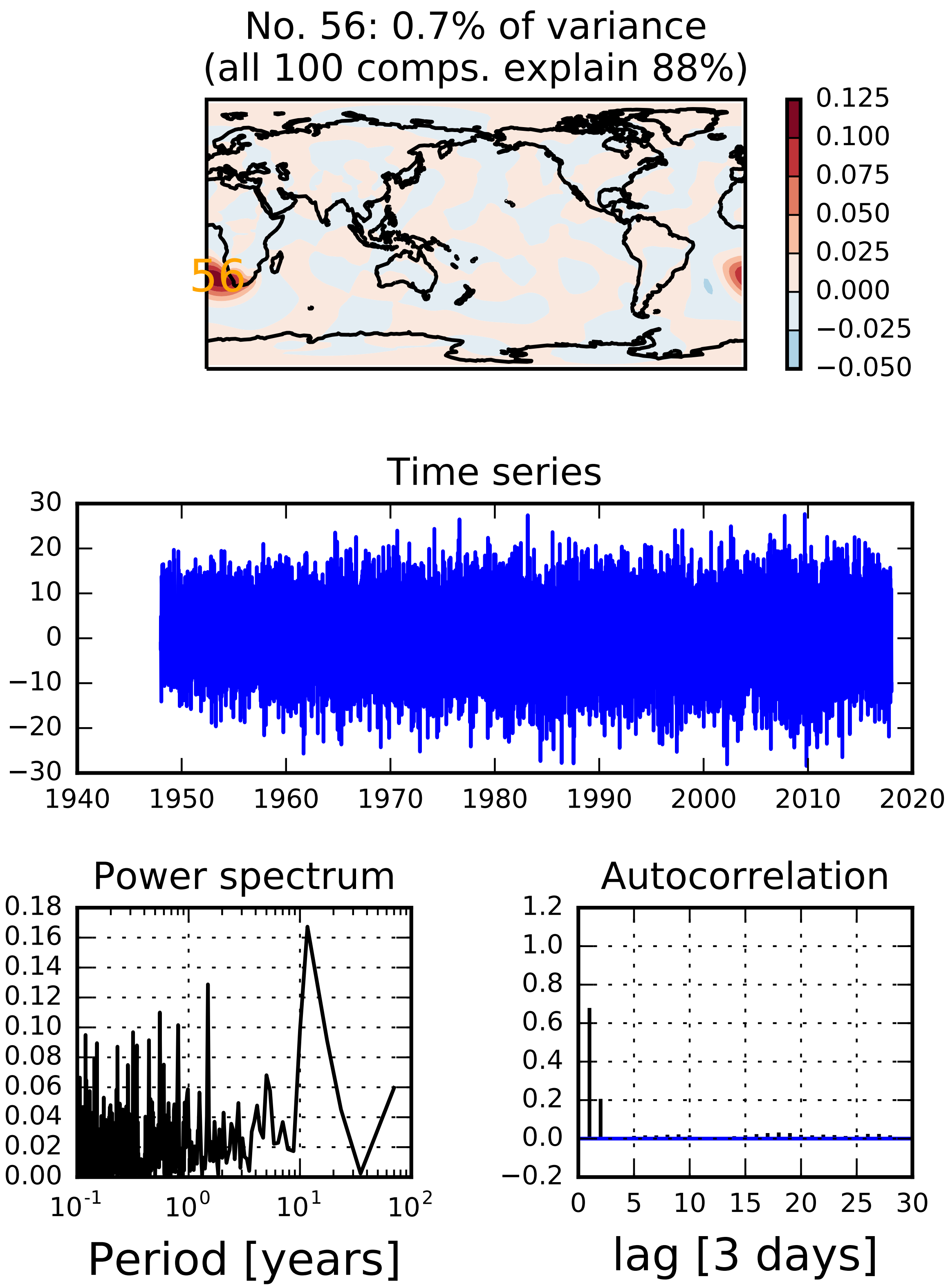
No. 56: 0.7% of variance
(all 100 comps. explain 88%)
56
0.125
0.100
0.075
0.050
0.025
0.000
−0.025
−0.050
Time series
30
20
10
0
−10
−20
−30
1940
1950
1960
1970
1980
1990
2000
2010
2020
Power spectrum
0.18
0.16
0.14
0.12
0.10
0.08
0.06
0.04
0.02
0.00
10^{-1}
10^{0}
10^{1}
10^{2}
Period [years]
Autocorrelation
1.2
1.0
0.8
0.6
0.4
0.2
0.0
−0.2
0
5
10
15
20
25
30
lag [3 days]

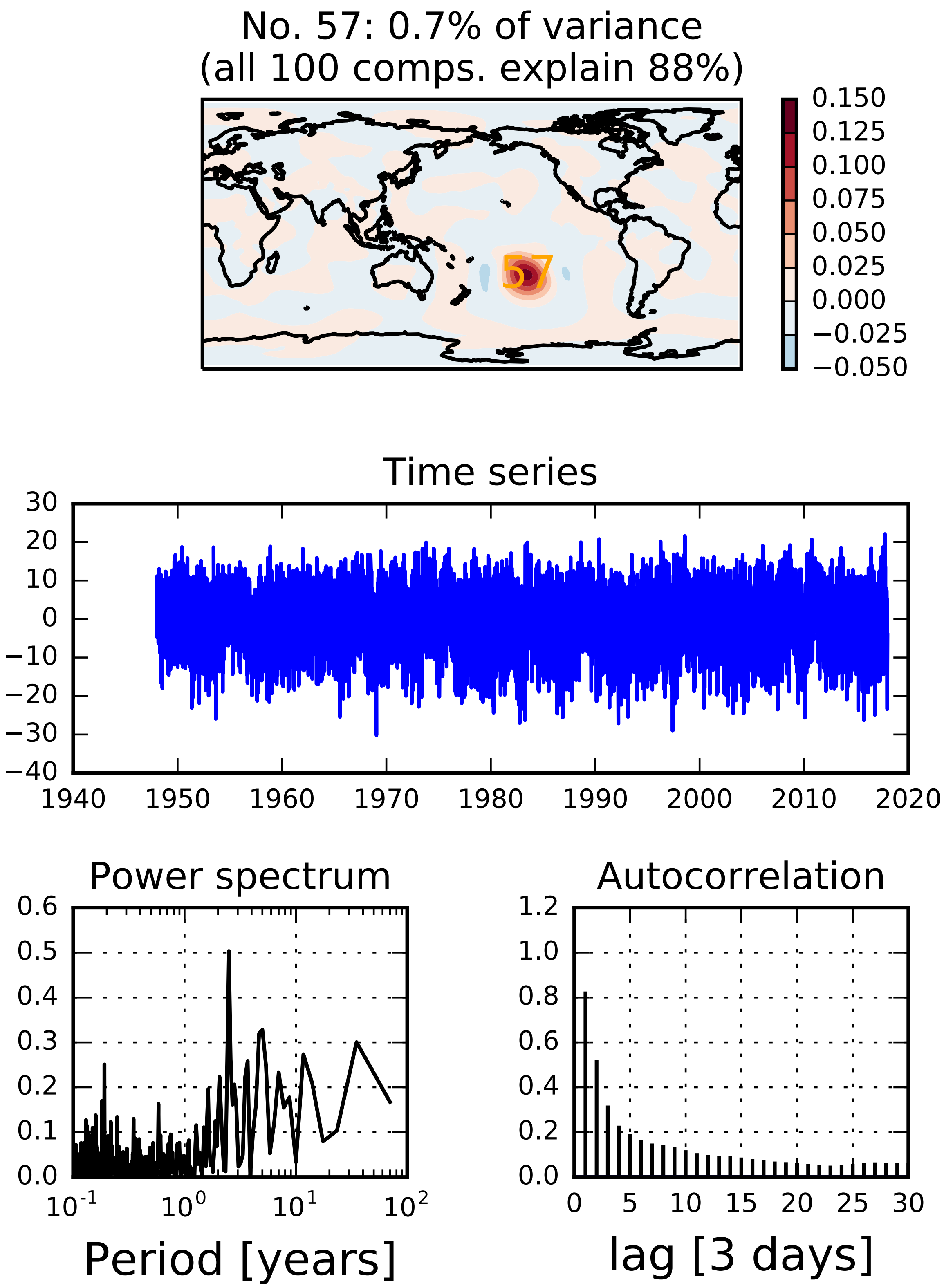
No. 57: 0.7% of variance
(all 100 comps. explain 88%)
57
0.150
0.125
0.100
0.075
0.050
0.025
0.000
−0.025
−0.050
Time series
30
20
10
0
−10
−20
−30
−40
1940
1950
1960
1970
1980
1990
2000
2010
2020
Power spectrum
0.6
0.5
0.4
0.3
0.2
0.1
0.0
10^-1
10^0
10^1
10^2
Period [years]
Autocorrelation
1.2
1.0
0.8
0.6
0.4
0.2
0.0
0
5
10
15
20
25
30
lag [3 days]

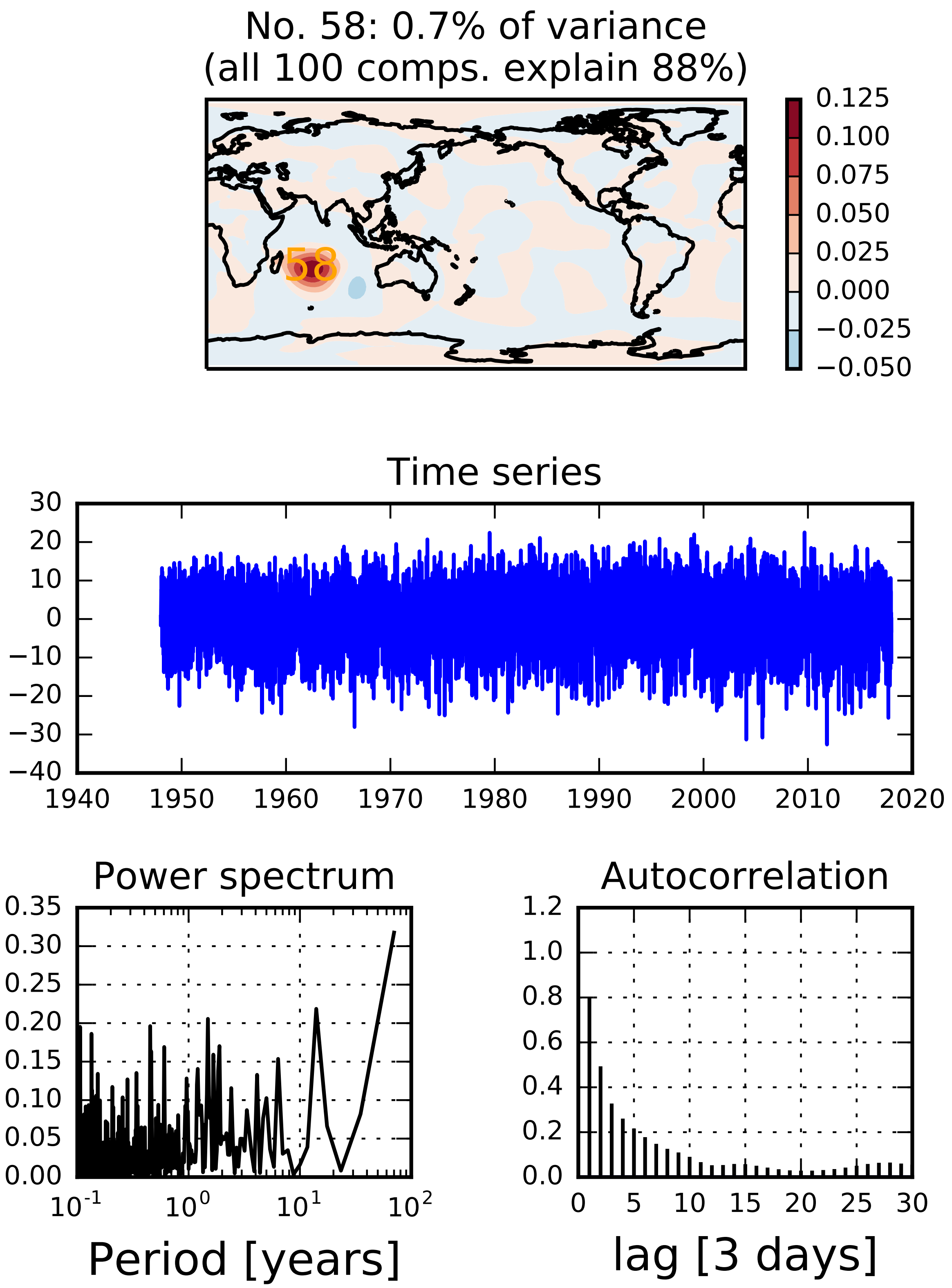
No. 58: 0.7% of variance
(all 100 comps. explain 88%)
58
0.125
0.100
0.075
0.050
0.025
0.000
−0.025
−0.050
Time series
30
20
10
0
−10
−20
−30
−40
1940
1950
1960
1970
1980
1990
2000
2010
2020
Power spectrum
0.35
0.30
0.25
0.20
0.15
0.10
0.05
0.00
10^-1
10^0
10^1
10^2
Period [years]
Autocorrelation
1.2
1.0
0.8
0.6
0.4
0.2
0.0
0
5
10
15
20
25
30
lag [3 days]

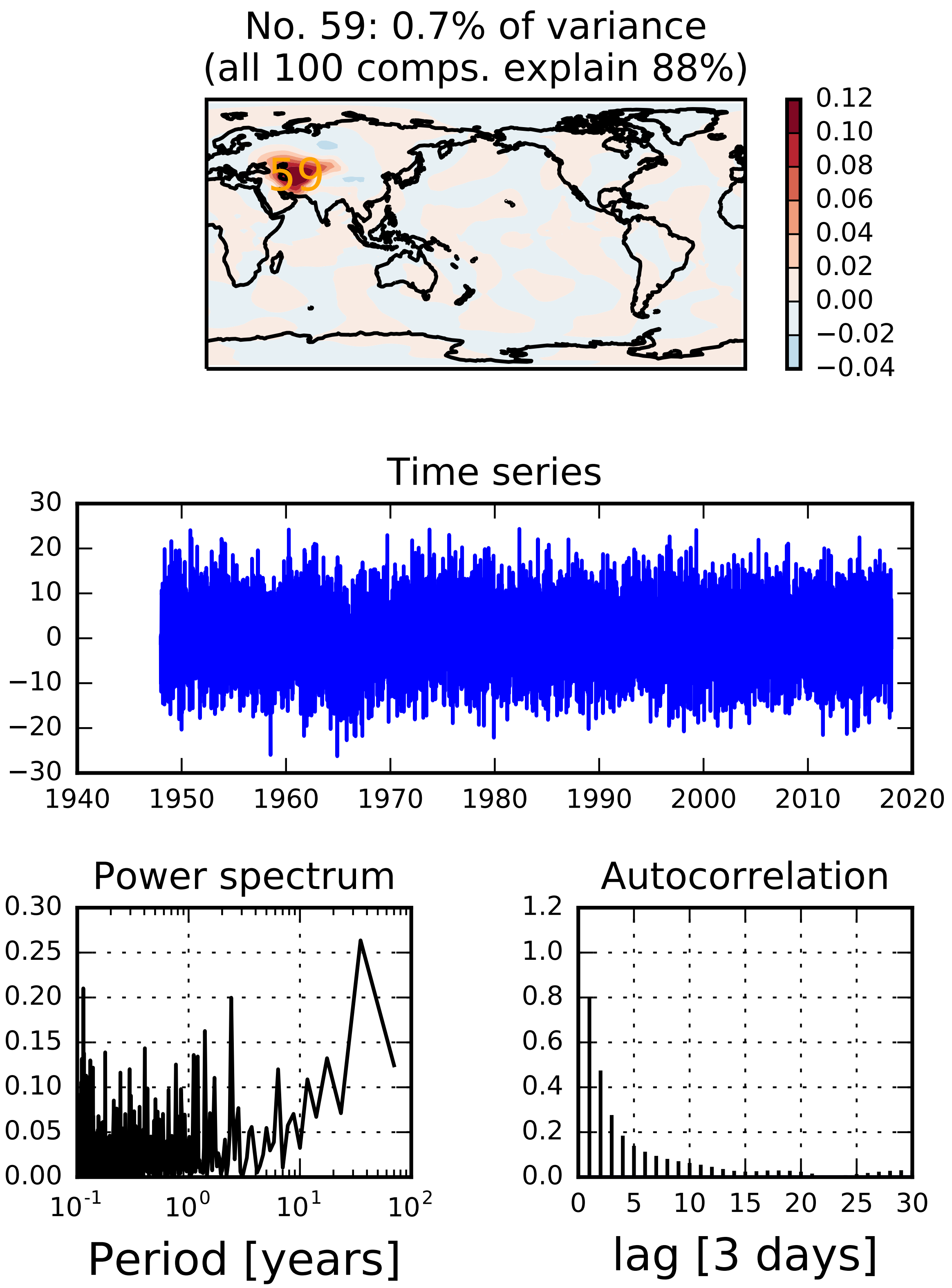
No. 59: 0.7% of variance
(all 100 comps. explain 88%)
59
0.12
0.10
0.08
0.06
0.04
0.02
0.00
−0.02
−0.04
Time series
30
20
10
0
−10
−20
−30
1940
1950
1960
1970
1980
1990
2000
2010
2020
Power spectrum
0.30
0.25
0.20
0.15
0.10
0.05
0.00
10^-1
10^0
10^1
10^2
Period [years]
Autocorrelation
1.2
1.0
0.8
0.6
0.4
0.2
0.0
0
5
10
15
20
25
30
lag [3 days]

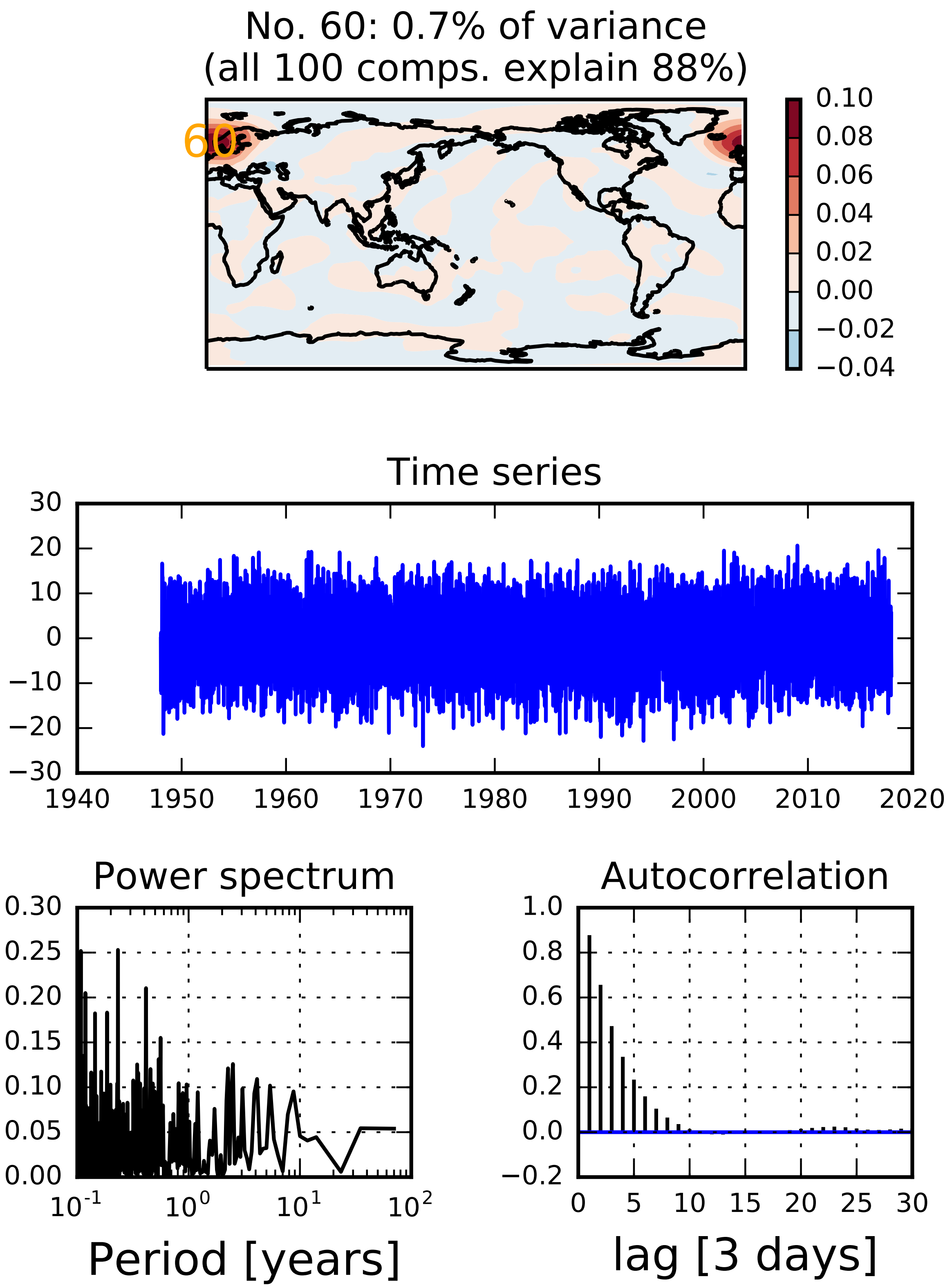
No. 60: 0.7% of variance
(all 100 comps. explain 88%)
60
0.10
0.08
0.06
0.04
0.02
0.00
−0.02
−0.04
Time series
30
20
10
0
−10
−20
−30
1940
1950
1960
1970
1980
1990
2000
2010
2020
Power spectrum
0.30
0.25
0.20
0.15
0.10
0.05
0.00
10^{-1}
10^{0}
10^{1}
10^{2}
Period [years]
Autocorrelation
1.0
0.8
0.6
0.4
0.2
0.0
−0.2
0
5
10
15
20
25
30
lag [3 days]

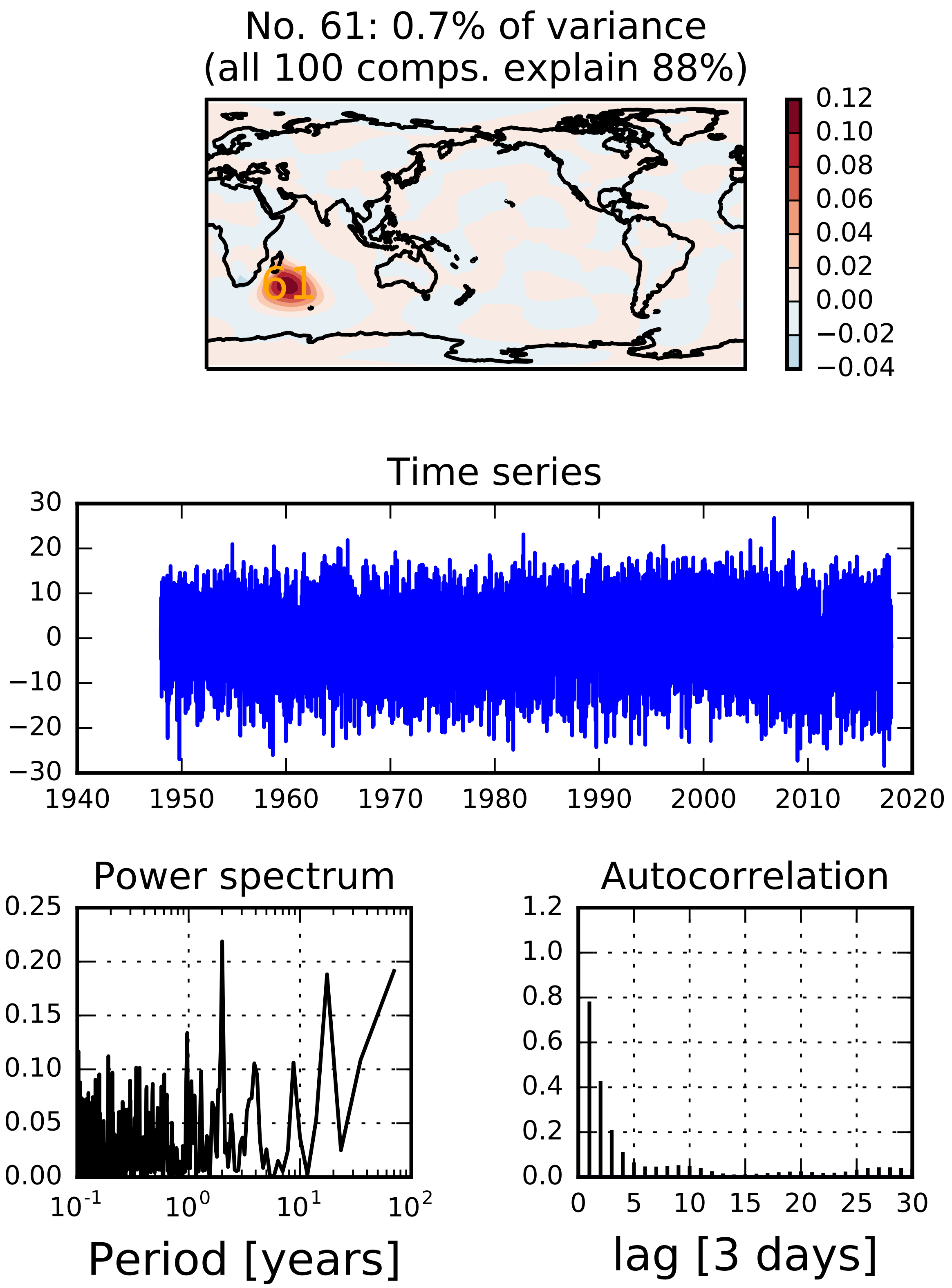
No. 61: 0.7% of variance
(all 100 comps. explain 88%)
61
0.12
0.10
0.08
0.06
0.04
0.02
0.00
−0.02
−0.04
Time series
30
20
10
0
−10
−20
−30
1940
1950
1960
1970
1980
1990
2000
2010
2020
Power spectrum
0.25
0.20
0.15
0.10
0.05
0.00
10^-1
10^0
10^1
10^2
Period [years]
Autocorrelation
1.2
1.0
0.8
0.6
0.4
0.2
0.0
0
5
10
15
20
25
30
lag [3 days]

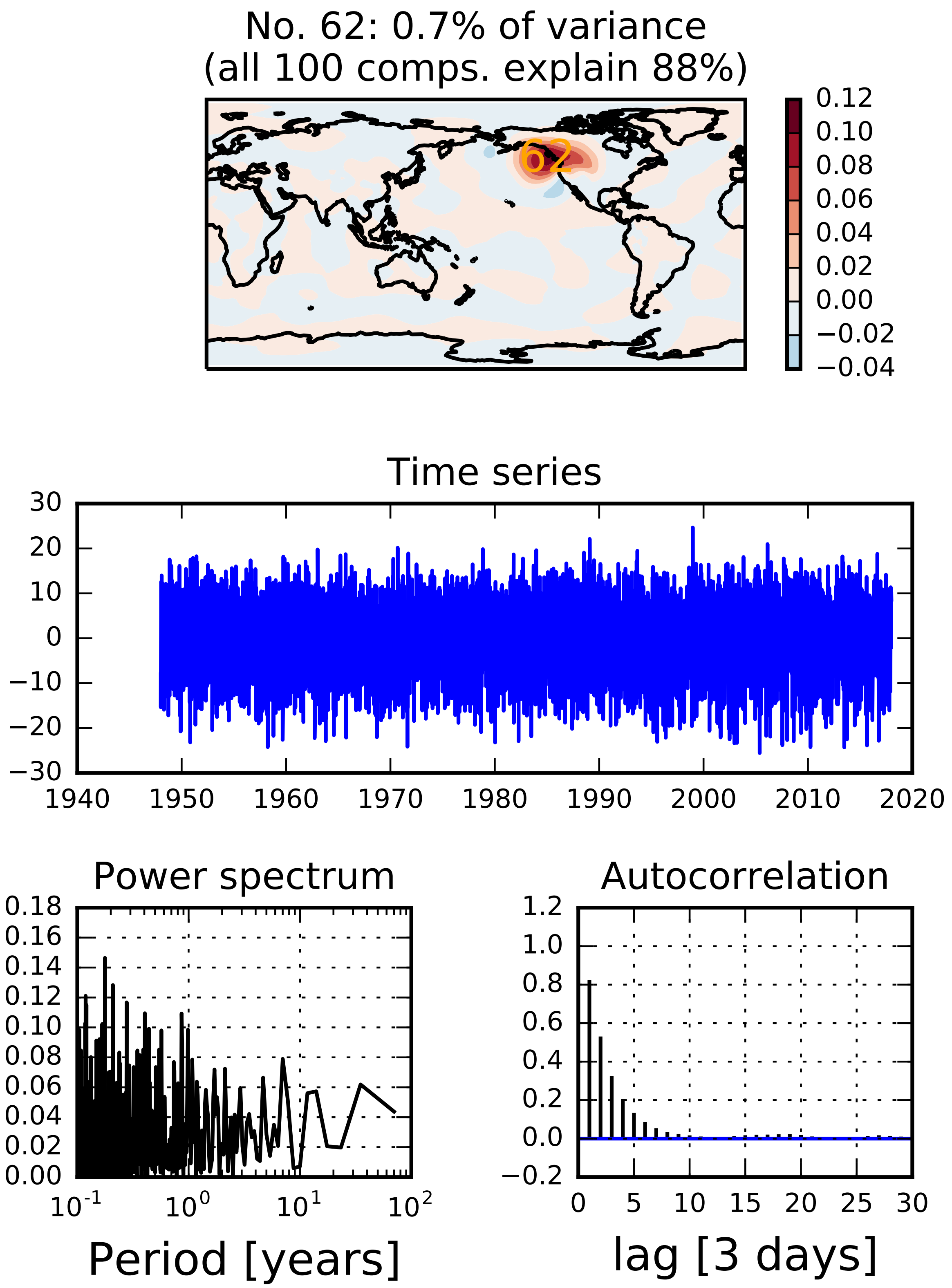
No. 62: 0.7% of variance
(all 100 comps. explain 88%)
62
0.12
0.10
0.08
0.06
0.04
0.02
0.00
−0.02
−0.04
Time series
30
20
10
0
−10
−20
−30
1940
1950
1960
1970
1980
1990
2000
2010
2020
Power spectrum
0.18
0.16
0.14
0.12
0.10
0.08
0.06
0.04
0.02
0.00
10^{-1}
10^{0}
10^{1}
10^{2}
Period [years]
Autocorrelation
1.2
1.0
0.8
0.6
0.4
0.2
0.0
−0.2
0
5
10
15
20
25
30
lag [3 days]

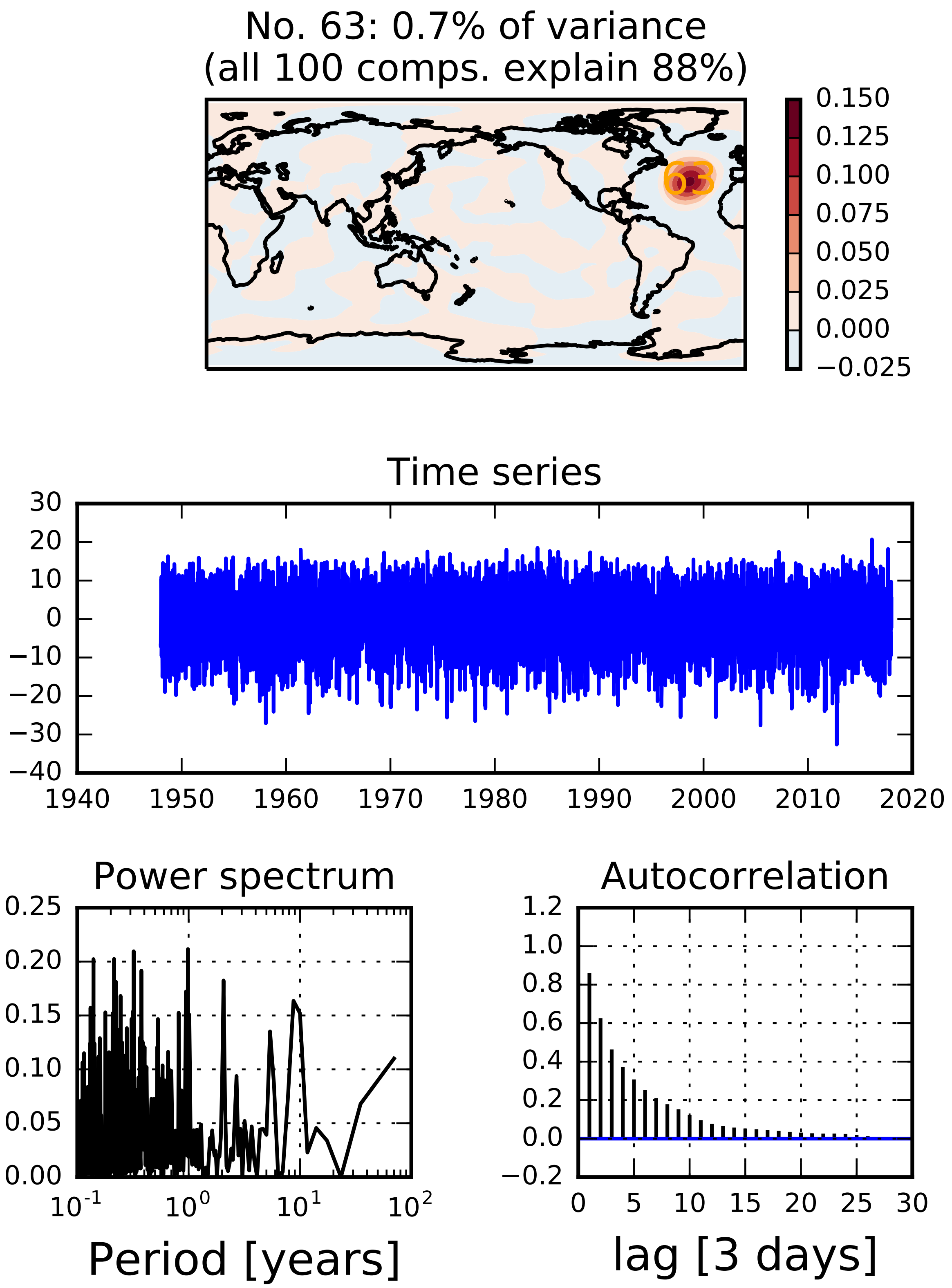
No. 63: 0.7% of variance
(all 100 comps. explain 88%)
0.150
0.125
0.100
0.075
0.050
0.025
0.000
−0.025
63
Time series
30
20
10
0
−10
−20
−30
−40
1940
1950
1960
1970
1980
1990
2000
2010
2020
Power spectrum
0.25
0.20
0.15
0.10
0.05
0.00
Period [years]
Autocorrelation
1.2
1.0
0.8
0.6
0.4
0.2
0.0
−0.2
0
5
10
15
20
25
30
lag [3 days]

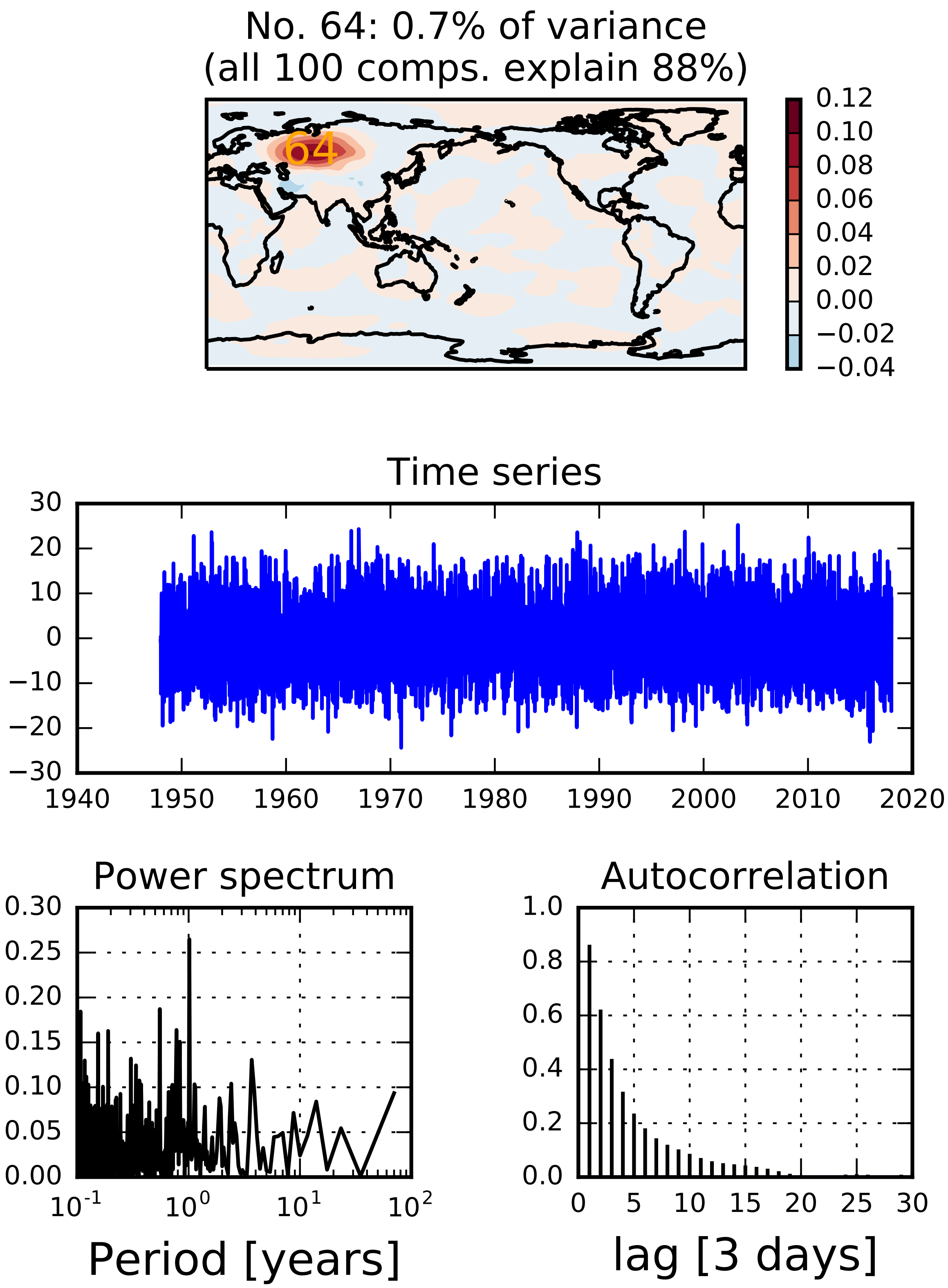
No. 64: 0.7% of variance
(all 100 comps. explain 88%)
64
0.12
0.10
0.08
0.06
0.04
0.02
0.00
−0.02
−0.04
Time series
30
20
10
0
−10
−20
−30
1940
1950
1960
1970
1980
1990
2000
2010
2020
Power spectrum
0.30
0.25
0.20
0.15
0.10
0.05
0.00
10^{-1}
10^{0}
10^{1}
10^{2}
Period [years]
Autocorrelation
1.0
0.8
0.6
0.4
0.2
0.0
0
5
10
15
20
25
30
lag [3 days]

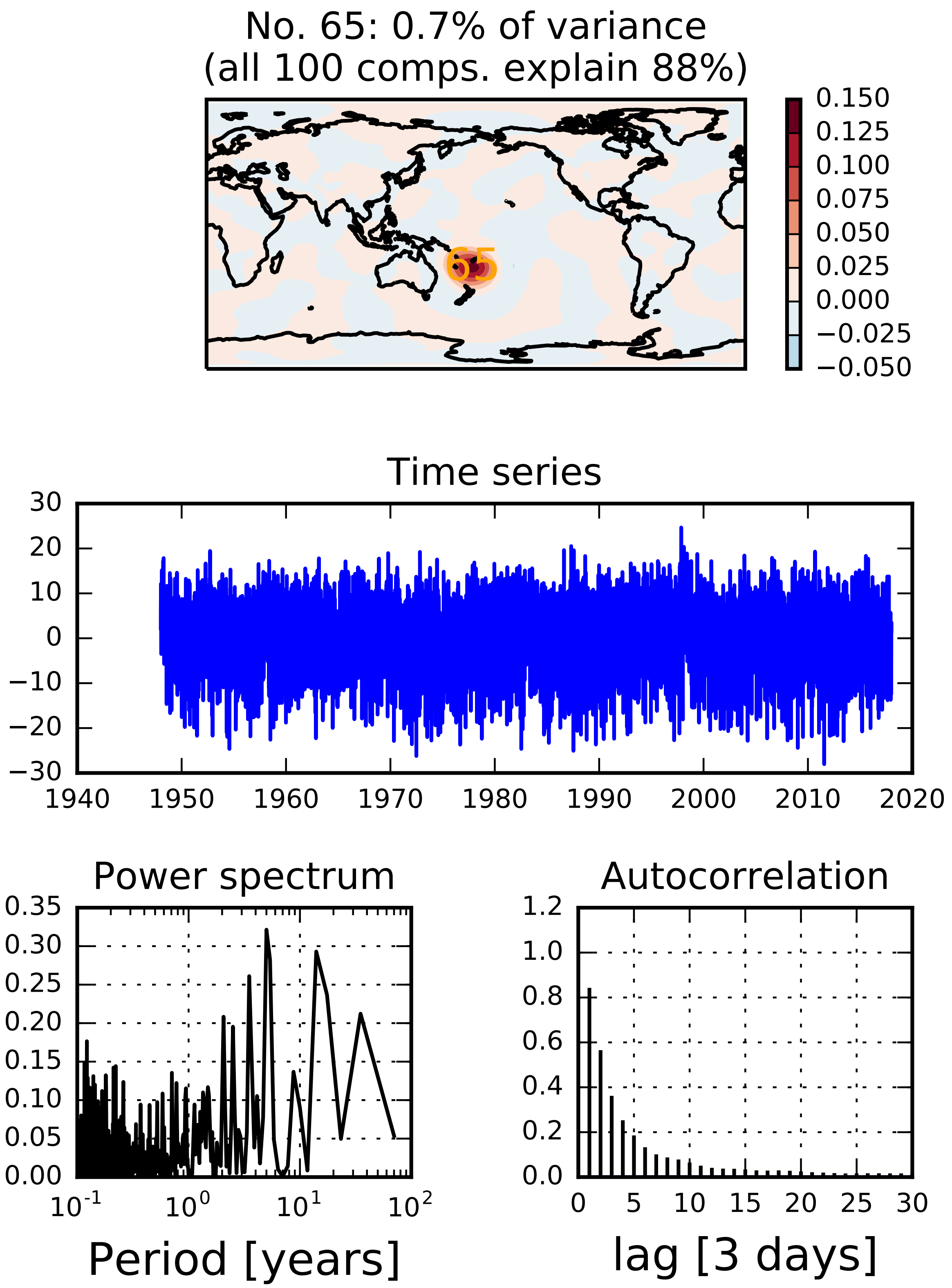
No. 65: 0.7% of variance
(all 100 comps. explain 88%)
65
0.150
0.125
0.100
0.075
0.050
0.025
0.000
−0.025
−0.050
Time series
30
20
10
0
−10
−20
−30
1940
1950
1960
1970
1980
1990
2000
2010
2020
Power spectrum
0.35
0.30
0.25
0.20
0.15
0.10
0.05
0.00
10^{-1}
10^{0}
10^{1}
10^{2}
Period [years]
Autocorrelation
1.2
1.0
0.8
0.6
0.4
0.2
0.0
0
5
10
15
20
25
30
lag [3 days]

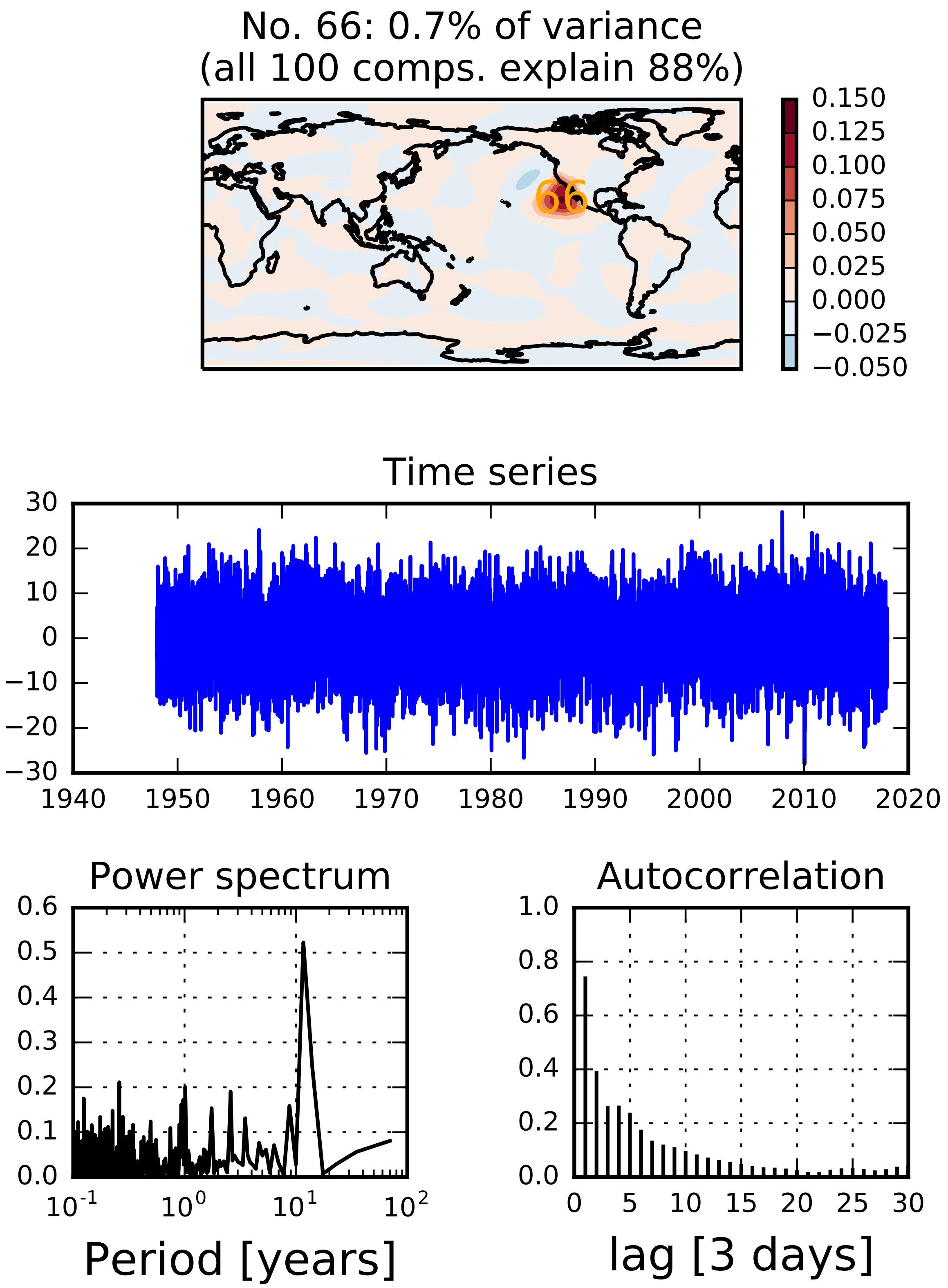
No. 66: 0.7% of variance
(all 100 comps. explain 88%)
66
0.150
0.125
0.100
0.075
0.050
0.025
0.000
−0.025
−0.050
Time series
30
20
10
0
−10
−20
−30
1940
1950
1960
1970
1980
1990
2000
2010
2020
Power spectrum
0.6
0.5
0.4
0.3
0.2
0.1
0.0
10^{-1}
10^{0}
10^{1}
10^{2}
Period [years]
Autocorrelation
1.0
0.8
0.6
0.4
0.2
0.0
0
5
10
15
20
25
30
lag [3 days]

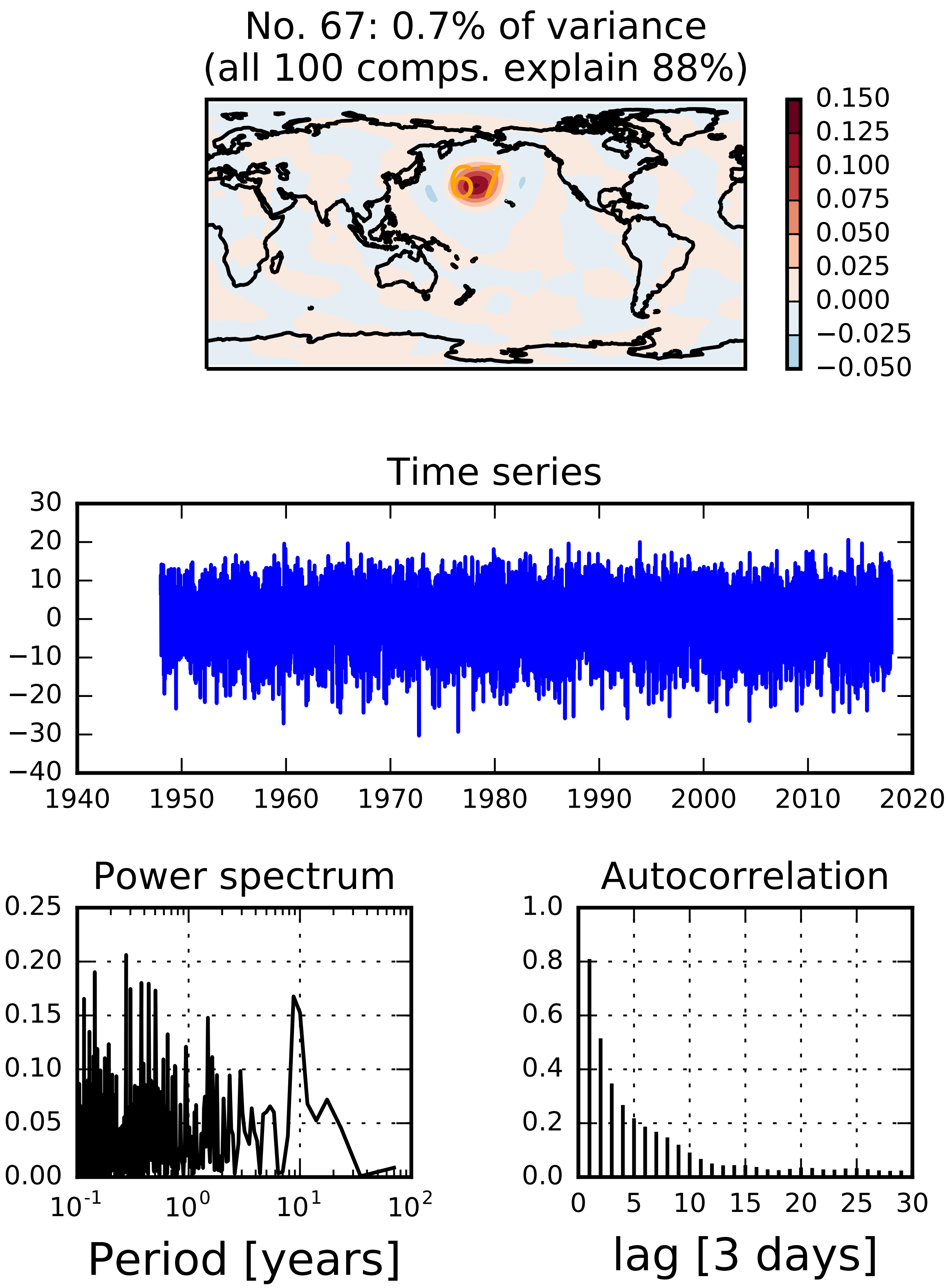
No. 67: 0.7% of variance
(all 100 comps. explain 88%)
67
0.150
0.125
0.100
0.075
0.050
0.025
0.000
−0.025
−0.050
Time series
30
20
10
0
−10
−20
−30
−40
1940
1950
1960
1970
1980
1990
2000
2010
2020
Power spectrum
0.25
0.20
0.15
0.10
0.05
0.00
10^{-1}
10^{0}
10^{1}
10^{2}
Period [years]
Autocorrelation
1.0
0.8
0.6
0.4
0.2
0.0
0
5
10
15
20
25
30
lag [3 days]

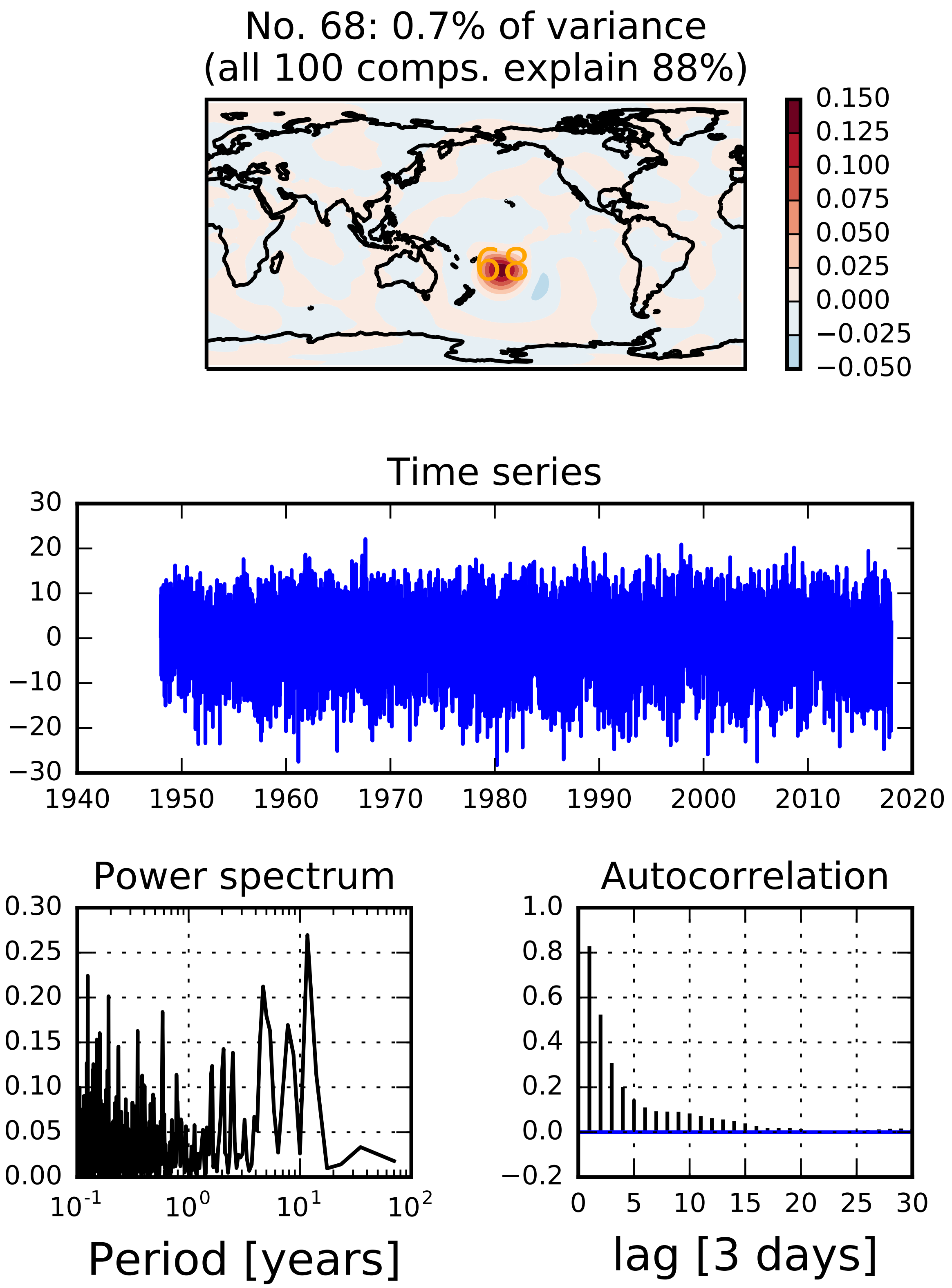
No. 68: 0.7% of variance
(all 100 comps. explain 88%)
68
0.150
0.125
0.100
0.075
0.050
0.025
0.000
−0.025
−0.050
Time series
30
20
10
0
−10
−20
−30
1940
1950
1960
1970
1980
1990
2000
2010
2020
Power spectrum
0.30
0.25
0.20
0.15
0.10
0.05
0.00
10^{-1}
10^{0}
10^{1}
10^{2}
Period [years]
Autocorrelation
1.0
0.8
0.6
0.4
0.2
0.0
−0.2
0
5
10
15
20
25
30
lag [3 days]

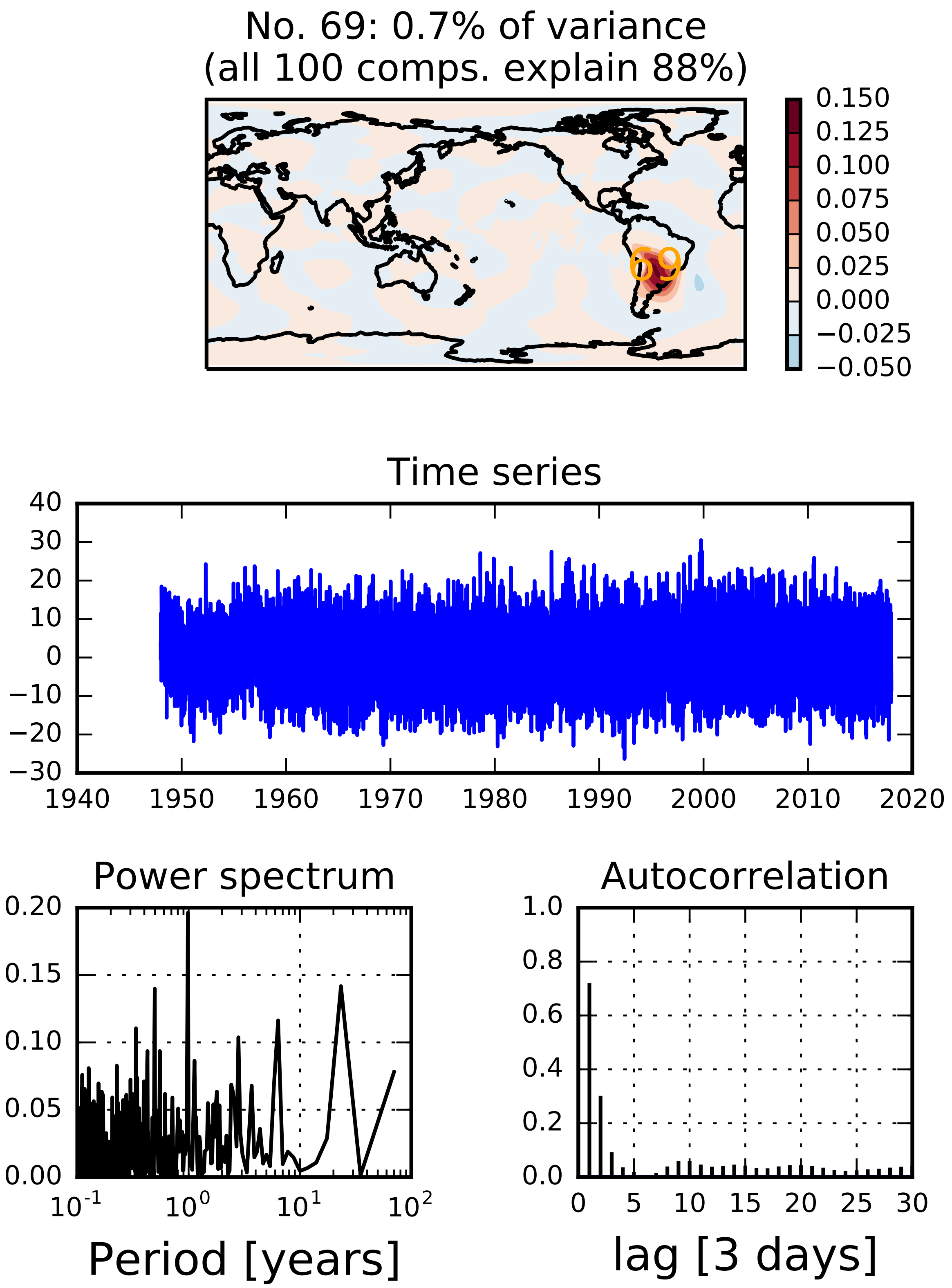
No. 69: 0.7% of variance
(all 100 comps. explain 88%)
69
0.150
0.125
0.100
0.075
0.050
0.025
0.000
−0.025
−0.050
Time series
40
30
20
10
0
−10
−20
−30
1940
1950
1960
1970
1980
1990
2000
2010
2020
Power spectrum
0.20
0.15
0.10
0.05
0.00
10^-1
10^0
10^1
10^2
Period [years]
Autocorrelation
1.0
0.8
0.6
0.4
0.2
0.0
0
5
10
15
20
25
30
lag [3 days]

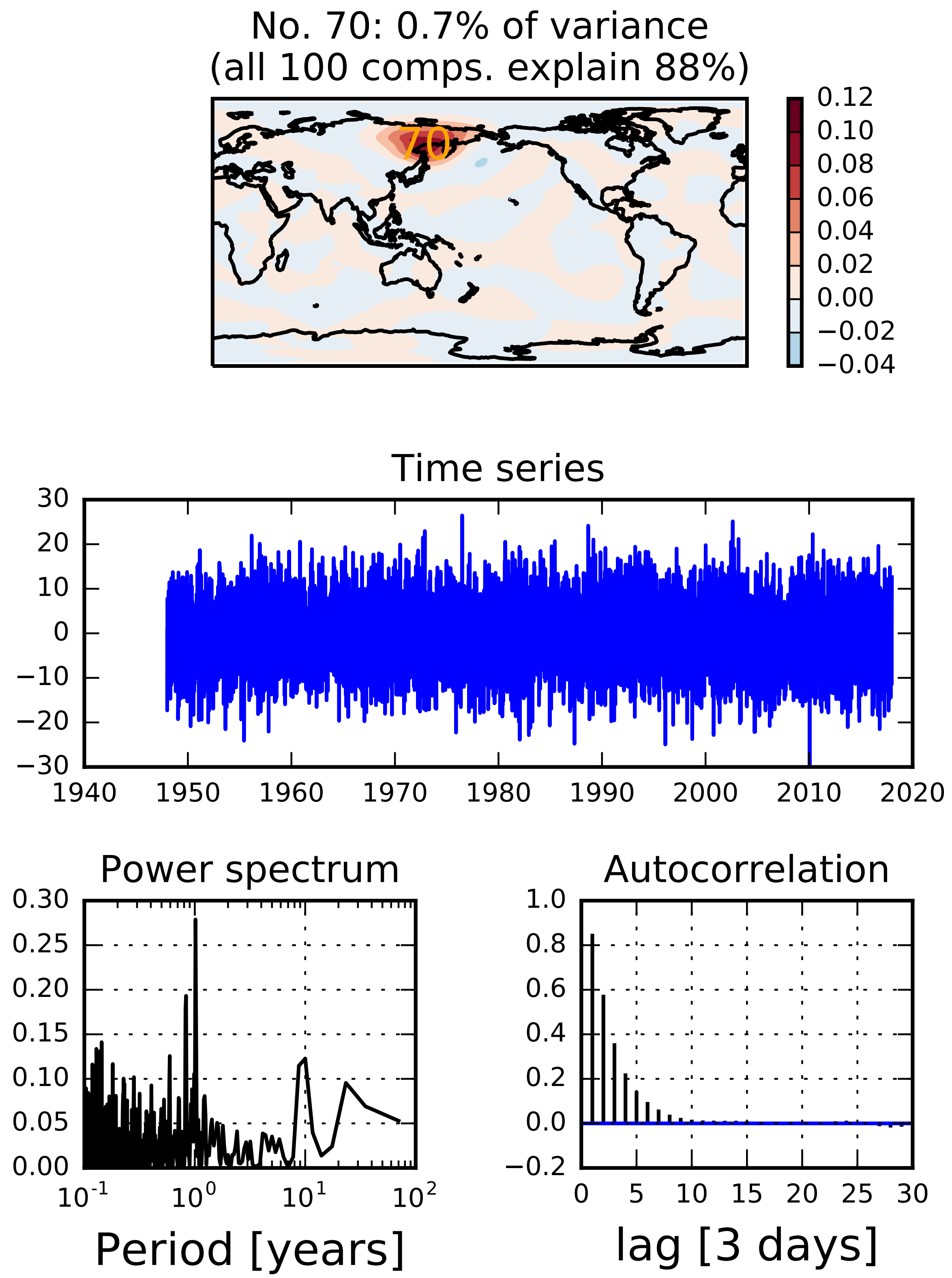
No. 70: 0.7% of variance
(all 100 comps. explain 88%)
70
0.12
0.10
0.08
0.06
0.04
0.02
0.00
−0.02
−0.04
Time series
30
20
10
0
−10
−20
−30
1940
1950
1960
1970
1980
1990
2000
2010
2020
Power spectrum
0.30
0.25
0.20
0.15
0.10
0.05
0.00
10^{-1}
10^{0}
10^{1}
10^{2}
Period [years]
Autocorrelation
1.0
0.8
0.6
0.4
0.2
0.0
−0.2
0
5
10
15
20
25
30
lag [3 days]

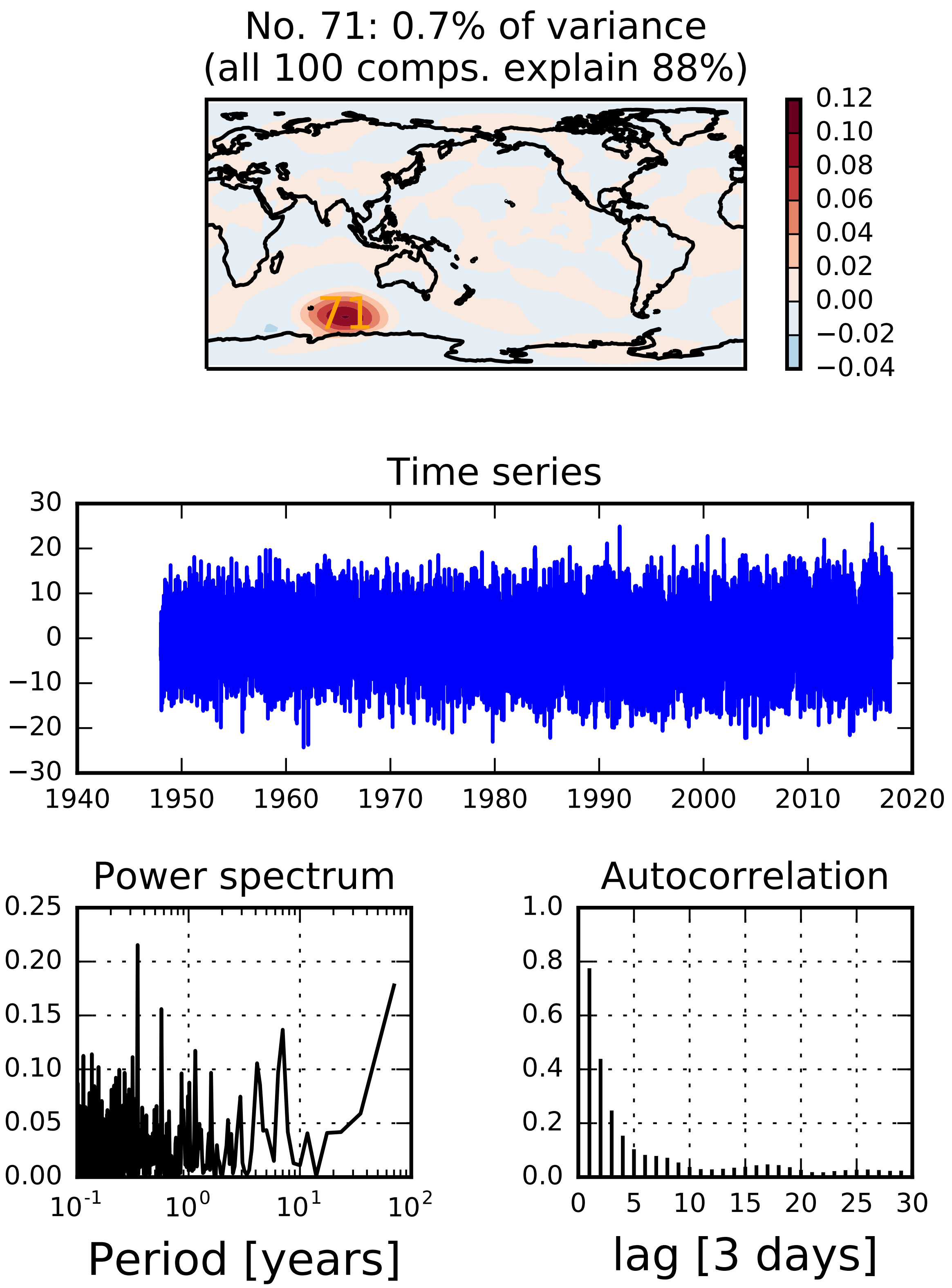
No. 71: 0.7% of variance
(all 100 comps. explain 88%)
71
0.12
0.10
0.08
0.06
0.04
0.02
0.00
−0.02
−0.04
Time series
30
20
10
0
−10
−20
−30
1940
1950
1960
1970
1980
1990
2000
2010
2020
Power spectrum
0.25
0.20
0.15
0.10
0.05
0.00
10^{-1}
10^{0}
10^{1}
10^{2}
Period [years]
Autocorrelation
1.0
0.8
0.6
0.4
0.2
0.0
0
5
10
15
20
25
30
lag [3 days]

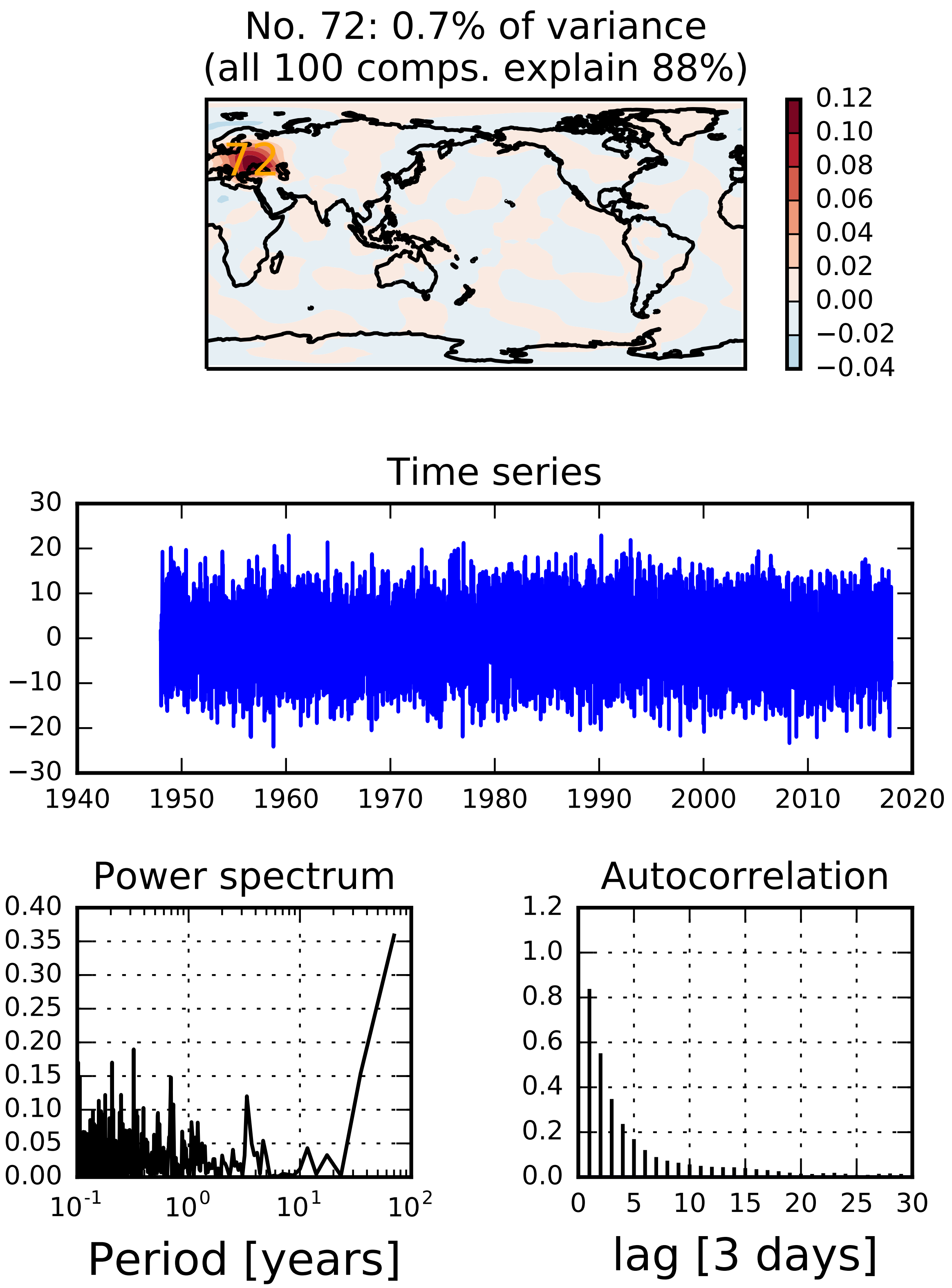
No. 72: 0.7% of variance
(all 100 comps. explain 88%)
72
0.12
0.10
0.08
0.06
0.04
0.02
0.00
−0.02
−0.04
Time series
30
20
10
0
−10
−20
−30
1940
1950
1960
1970
1980
1990
2000
2010
2020
Power spectrum
0.40
0.35
0.30
0.25
0.20
0.15
0.10
0.05
0.00
10^{-1}
10^{0}
10^{1}
10^{2}
Period [years]
Autocorrelation
1.2
1.0
0.8
0.6
0.4
0.2
0.0
0
5
10
15
20
25
30
lag [3 days]

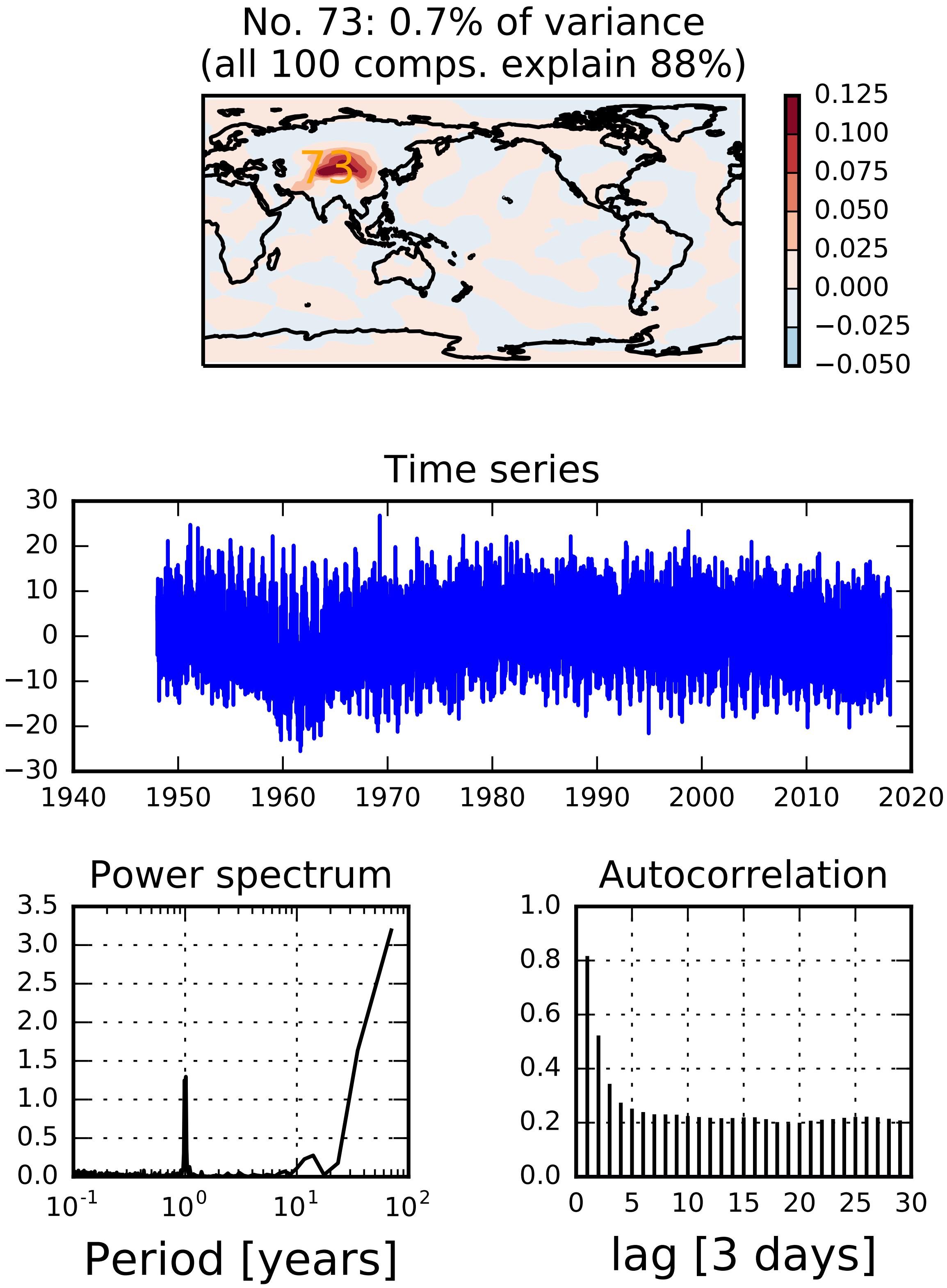
No. 73: 0.7% of variance
(all 100 comps. explain 88%)
73
0.125
0.100
0.075
0.050
0.025
0.000
−0.025
−0.050
Time series
30
20
10
0
−10
−20
−30
1940
1950
1960
1970
1980
1990
2000
2010
2020
Power spectrum
3.5
3.0
2.5
2.0
1.5
1.0
0.5
0.0
10^{-1}
10^{0}
10^{1}
10^{2}
Period [years]
Autocorrelation
1.0
0.8
0.6
0.4
0.2
0.0
0
5
10
15
20
25
30
lag [3 days]

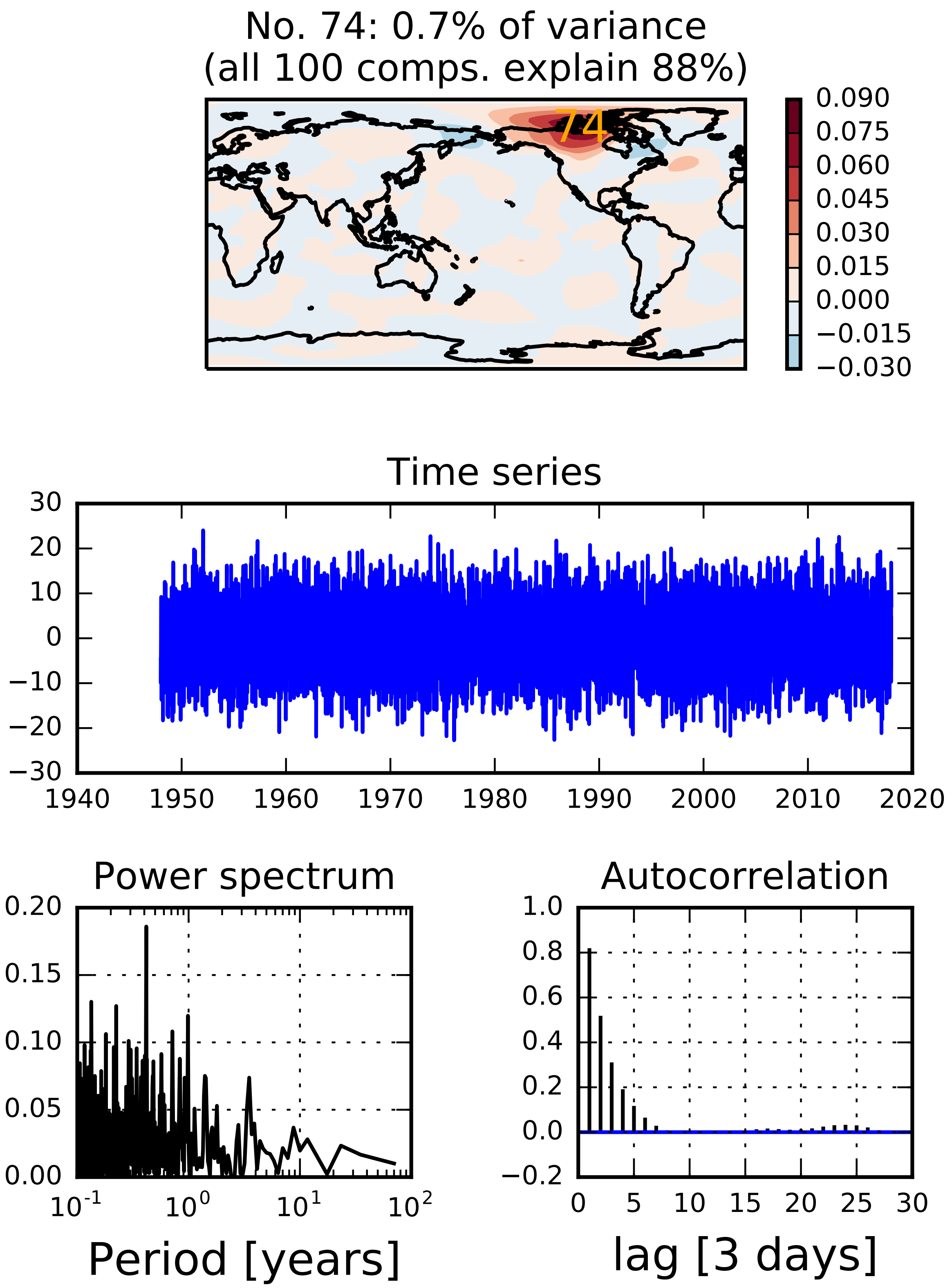
No. 74: 0.7% of variance
(all 100 comps. explain 88%)
74
0.090
0.075
0.060
0.045
0.030
0.015
0.000
−0.015
−0.030
Time series
30
20
10
0
−10
−20
−30
1940 1950 1960 1970 1980 1990 2000 2010 2020
Power spectrum
0.20
0.15
0.10
0.05
0.00
10^{-1} 10^{0} 10^{1} 10^{2}
Period [years]
Autocorrelation
1.0
0.8
0.6
0.4
0.2
0.0
−0.2
0 5 10 15 20 25 30
lag [3 days]

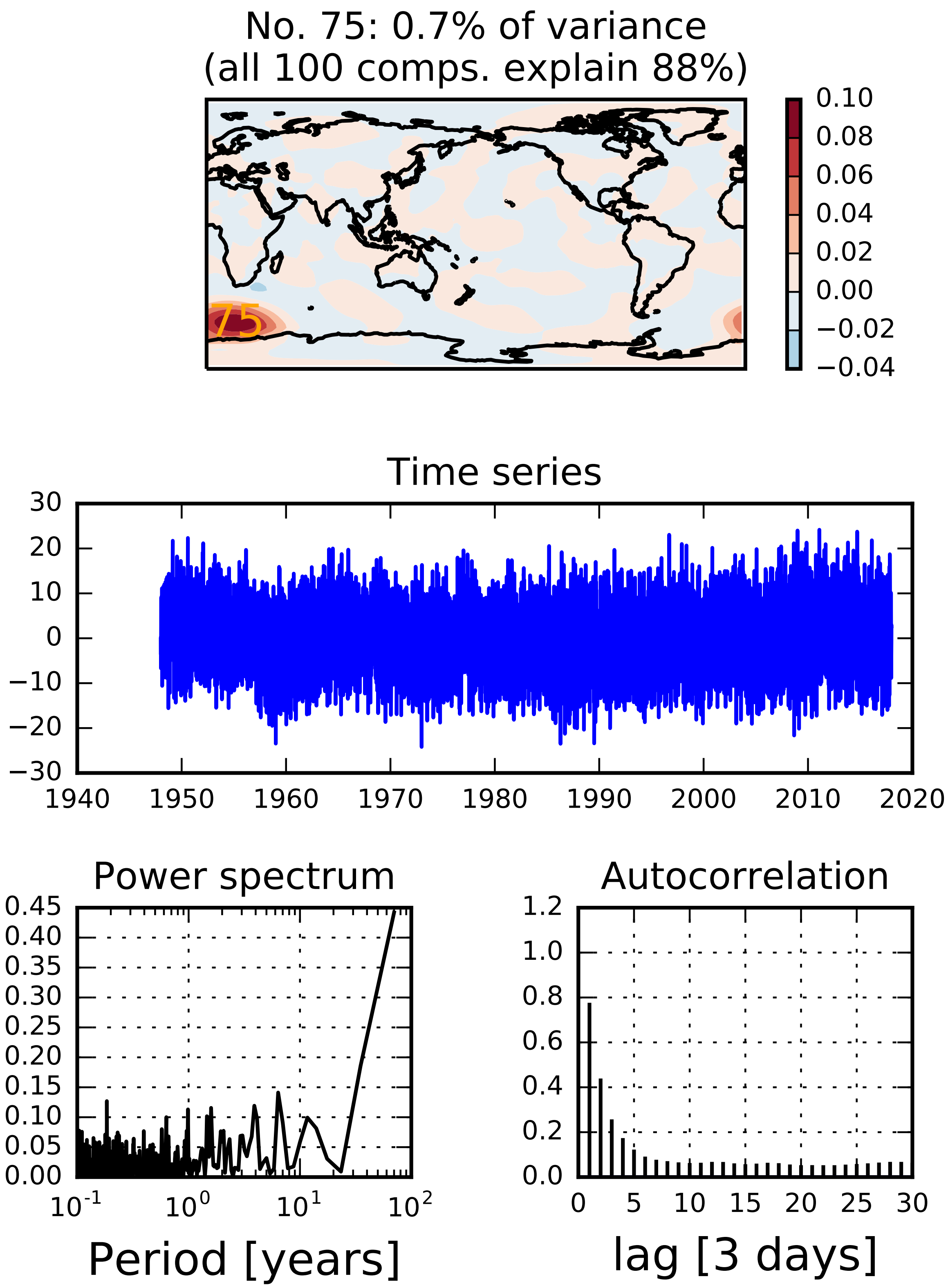
No. 75: 0.7% of variance
(all 100 comps. explain 88%)
75
0.10
0.08
0.06
0.04
0.02
0.00
−0.02
−0.04
Time series
30
20
10
0
−10
−20
−30
1940
1950
1960
1970
1980
1990
2000
2010
2020
Power spectrum
0.45
0.40
0.35
0.30
0.25
0.20
0.15
0.10
0.05
0.00
10^{-1}
10^{0}
10^{1}
10^{2}
Period [years]
Autocorrelation
1.2
1.0
0.8
0.6
0.4
0.2
0.0
0
5
10
15
20
25
30
lag [3 days]

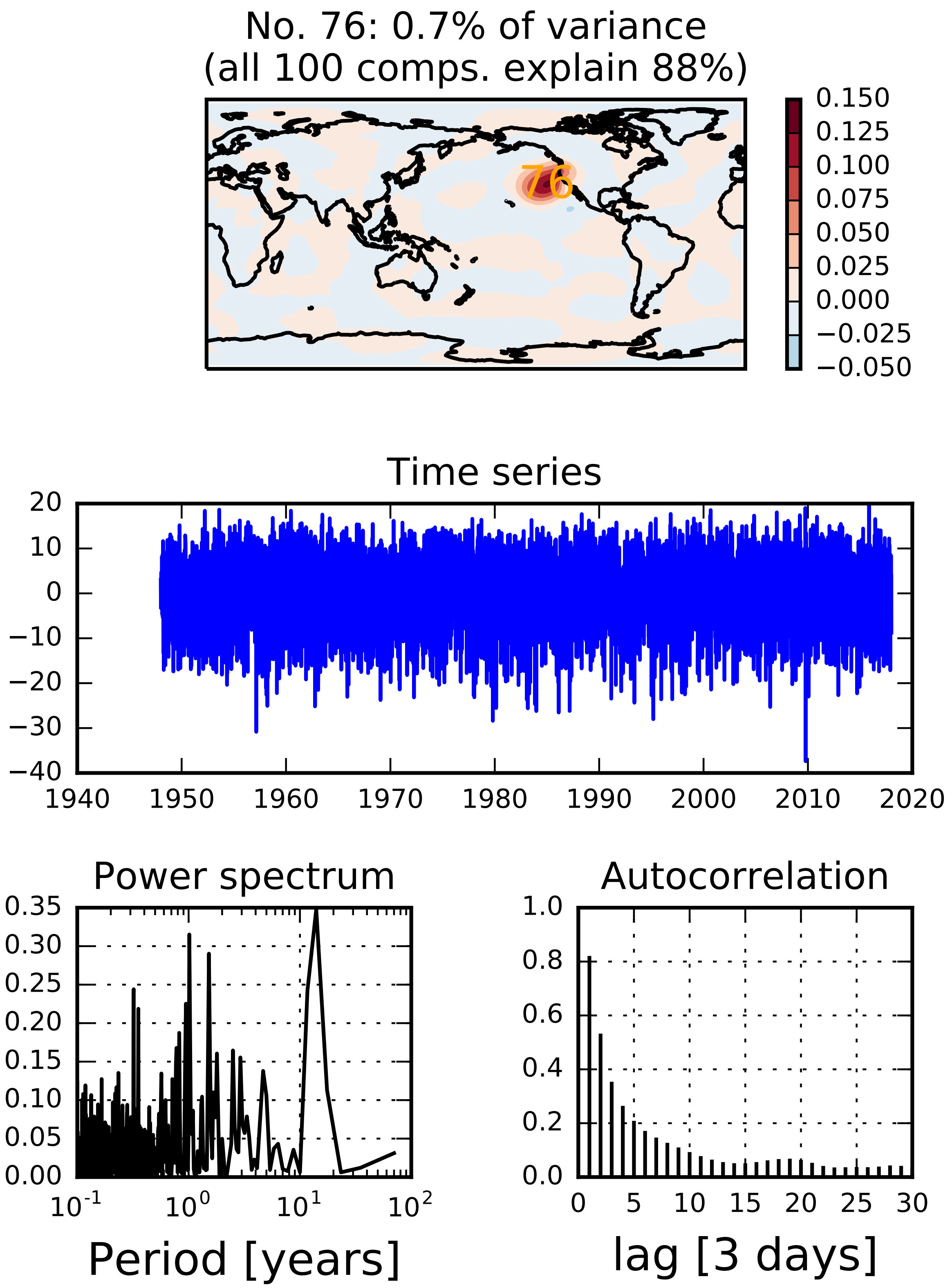
No. 76: 0.7% of variance
(all 100 comps. explain 88%)
76
0.150
0.125
0.100
0.075
0.050
0.025
0.000
−0.025
−0.050
Time series
20
10
0
−10
−20
−30
−40
1940
1950
1960
1970
1980
1990
2000
2010
2020
Power spectrum
0.35
0.30
0.25
0.20
0.15
0.10
0.05
0.00
10^-1
10^0
10^1
10^2
Period [years]
Autocorrelation
1.0
0.8
0.6
0.4
0.2
0.0
0
5
10
15
20
25
30
lag [3 days]

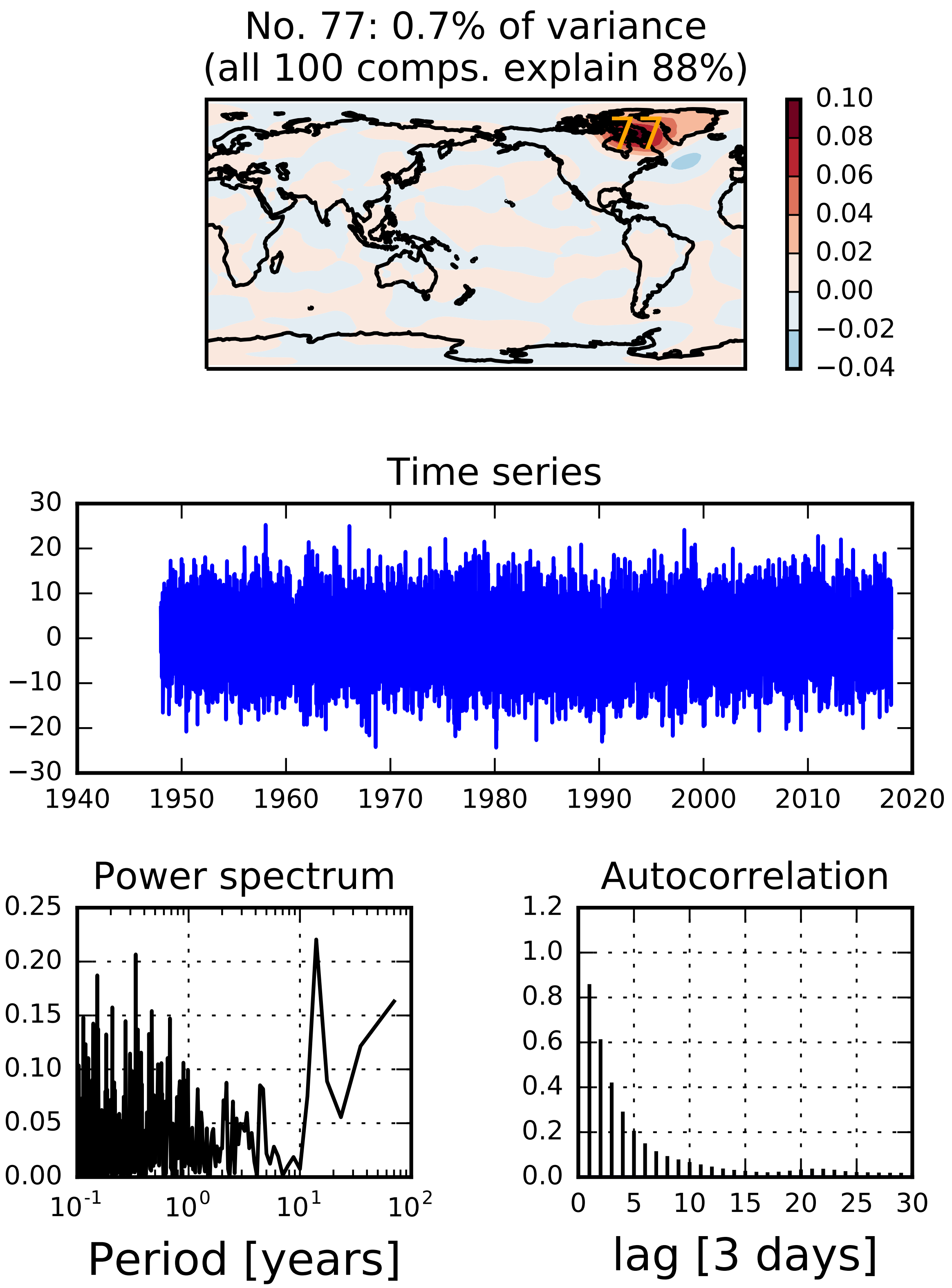
No. 77: 0.7% of variance
(all 100 comps. explain 88%)
77
0.10
0.08
0.06
0.04
0.02
0.00
−0.02
−0.04
Time series
30
20
10
0
−10
−20
−30
1940
1950
1960
1970
1980
1990
2000
2010
2020
Power spectrum
0.25
0.20
0.15
0.10
0.05
0.00
10^{-1}
10^{0}
10^{1}
10^{2}
Period [years]
Autocorrelation
1.2
1.0
0.8
0.6
0.4
0.2
0.0
0
5
10
15
20
25
30
lag [3 days]

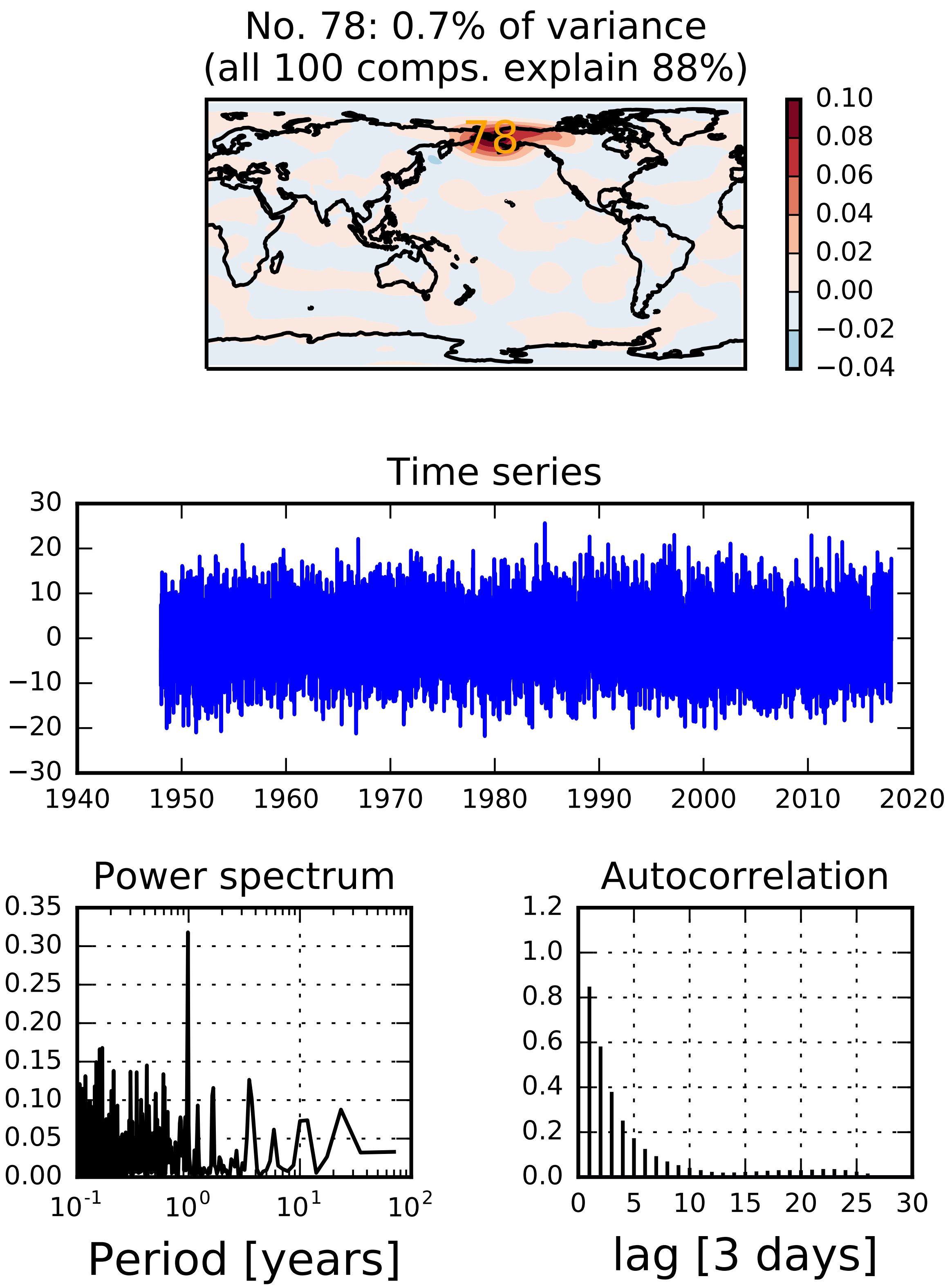
No. 78: 0.7% of variance
(all 100 comps. explain 88%)
78
0.10
0.08
0.06
0.04
0.02
0.00
−0.02
−0.04
Time series
Power spectrum
Period [years]
Autocorrelation
lag [3 days]

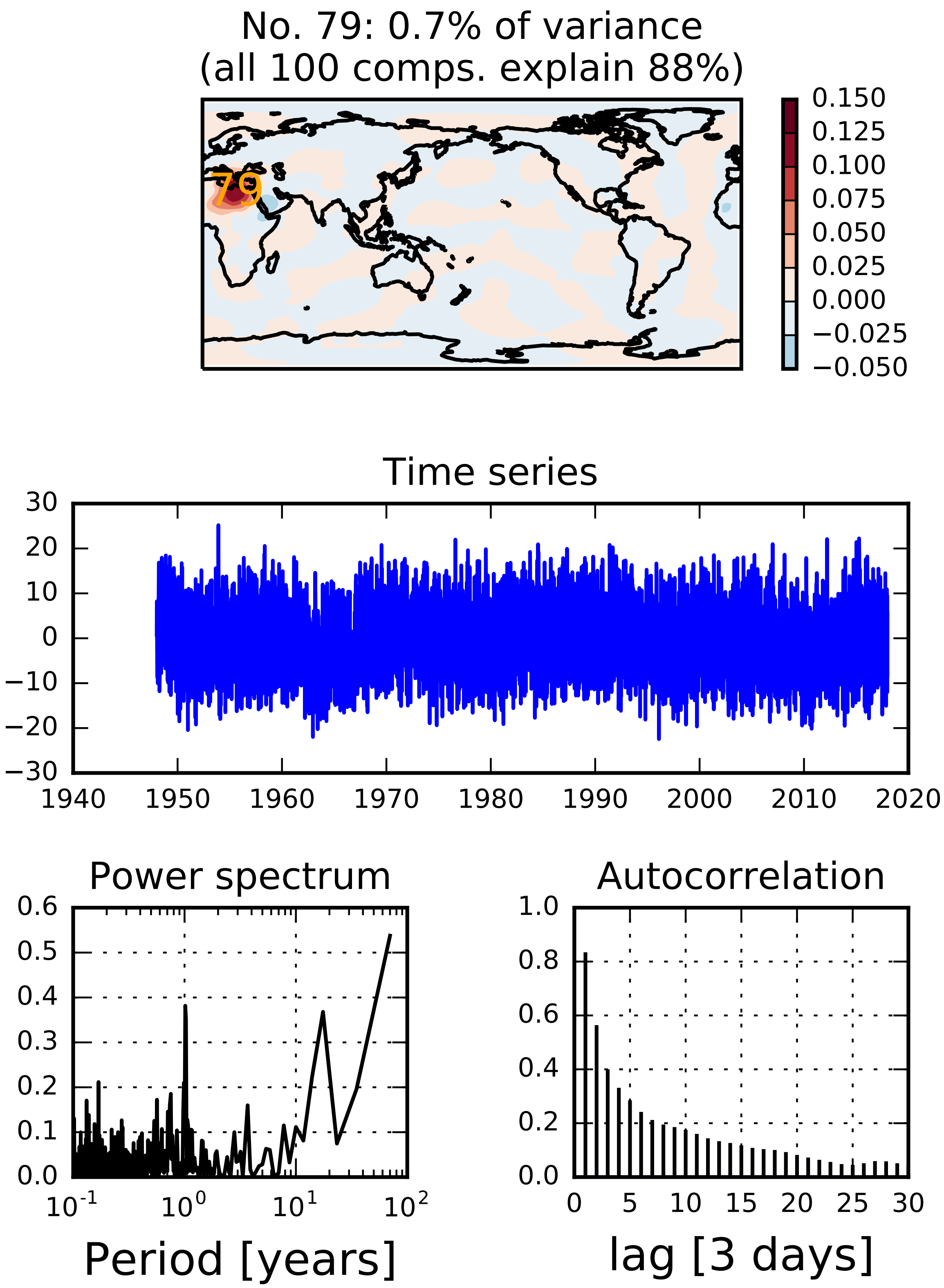
No. 79: 0.7% of variance
(all 100 comps. explain 88%)
79
0.150
0.125
0.100
0.075
0.050
0.025
0.000
−0.025
−0.050
Time series
30
20
10
0
−10
−20
−30
1940
1950
1960
1970
1980
1990
2000
2010
2020
Power spectrum
0.6
0.5
0.4
0.3
0.2
0.1
0.0
10^{-1}
10^{0}
10^{1}
10^{2}
Period [years]
Autocorrelation
1.0
0.8
0.6
0.4
0.2
0.0
0
5
10
15
20
25
30
lag [3 days]

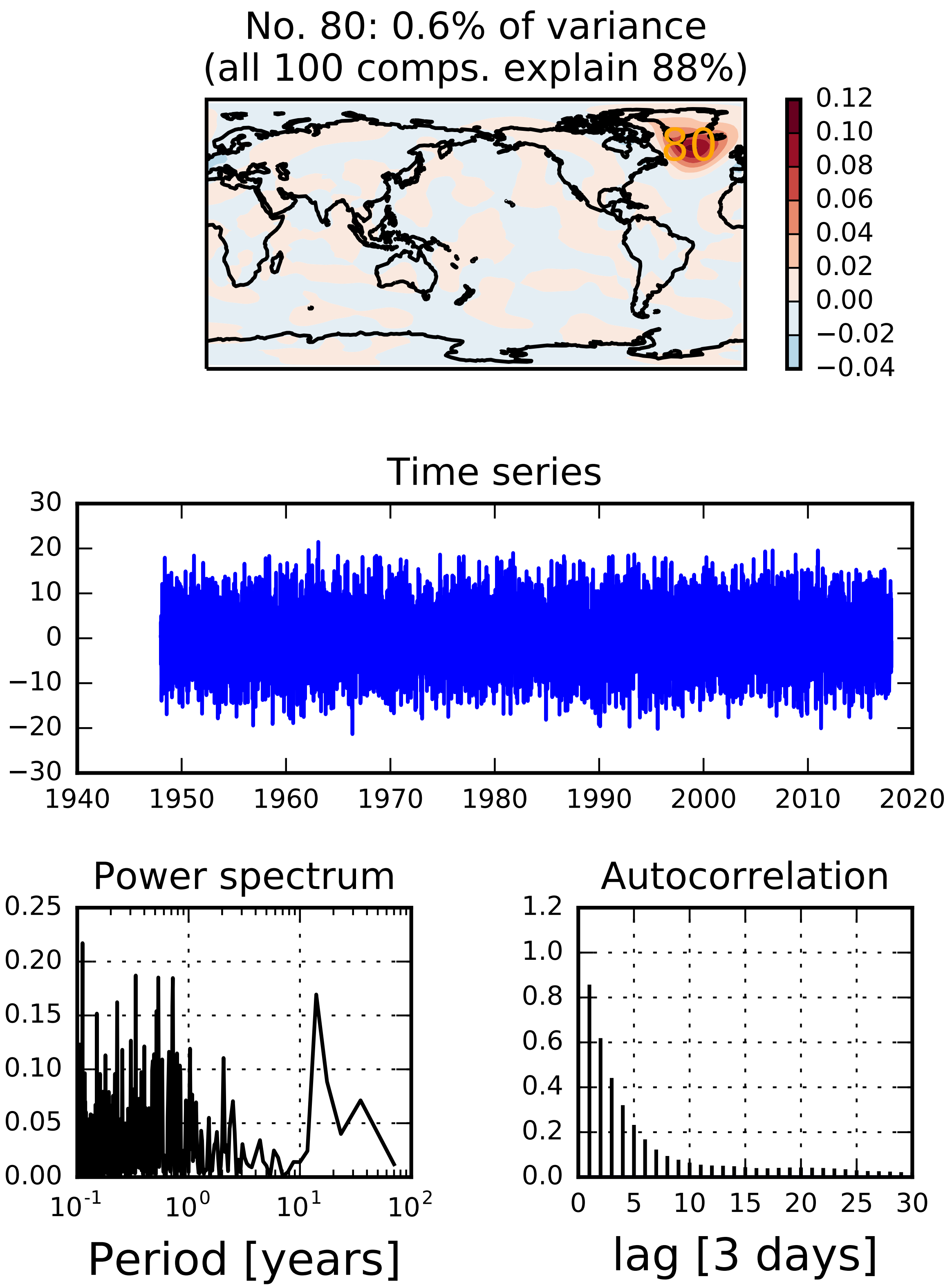
No. 80: 0.6% of variance
(all 100 comps. explain 88%)
80
0.12
0.10
0.08
0.06
0.04
0.02
0.00
−0.02
−0.04
Time series
30
20
10
0
−10
−20
−30
1940
1950
1960
1970
1980
1990
2000
2010
2020
Power spectrum
0.25
0.20
0.15
0.10
0.05
0.00
10^-1
10^0
10^1
10^2
Period [years]
Autocorrelation
1.2
1.0
0.8
0.6
0.4
0.2
0.0
0
5
10
15
20
25
30
lag [3 days]

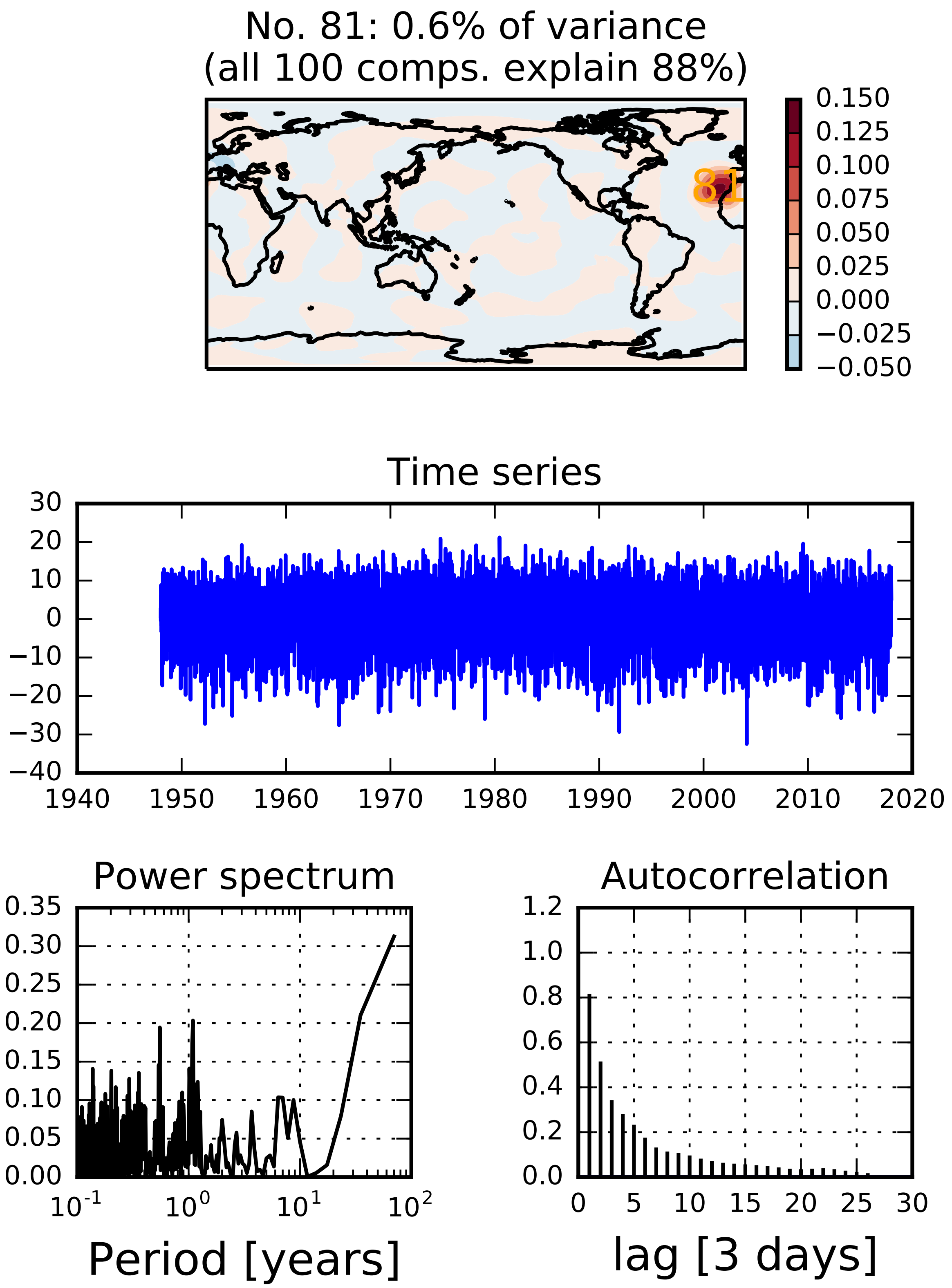
No. 81: 0.6% of variance
(all 100 comps. explain 88%)
81
0.150
0.125
0.100
0.075
0.050
0.025
0.000
−0.025
−0.050
Time series
30
20
10
0
−10
−20
−30
−40
1940
1950
1960
1970
1980
1990
2000
2010
2020
Power spectrum
0.35
0.30
0.25
0.20
0.15
0.10
0.05
0.00
10^{-1}
10^{0}
10^{1}
10^{2}
Period [years]
Autocorrelation
1.2
1.0
0.8
0.6
0.4
0.2
0.0
0
5
10
15
20
25
30
lag [3 days]

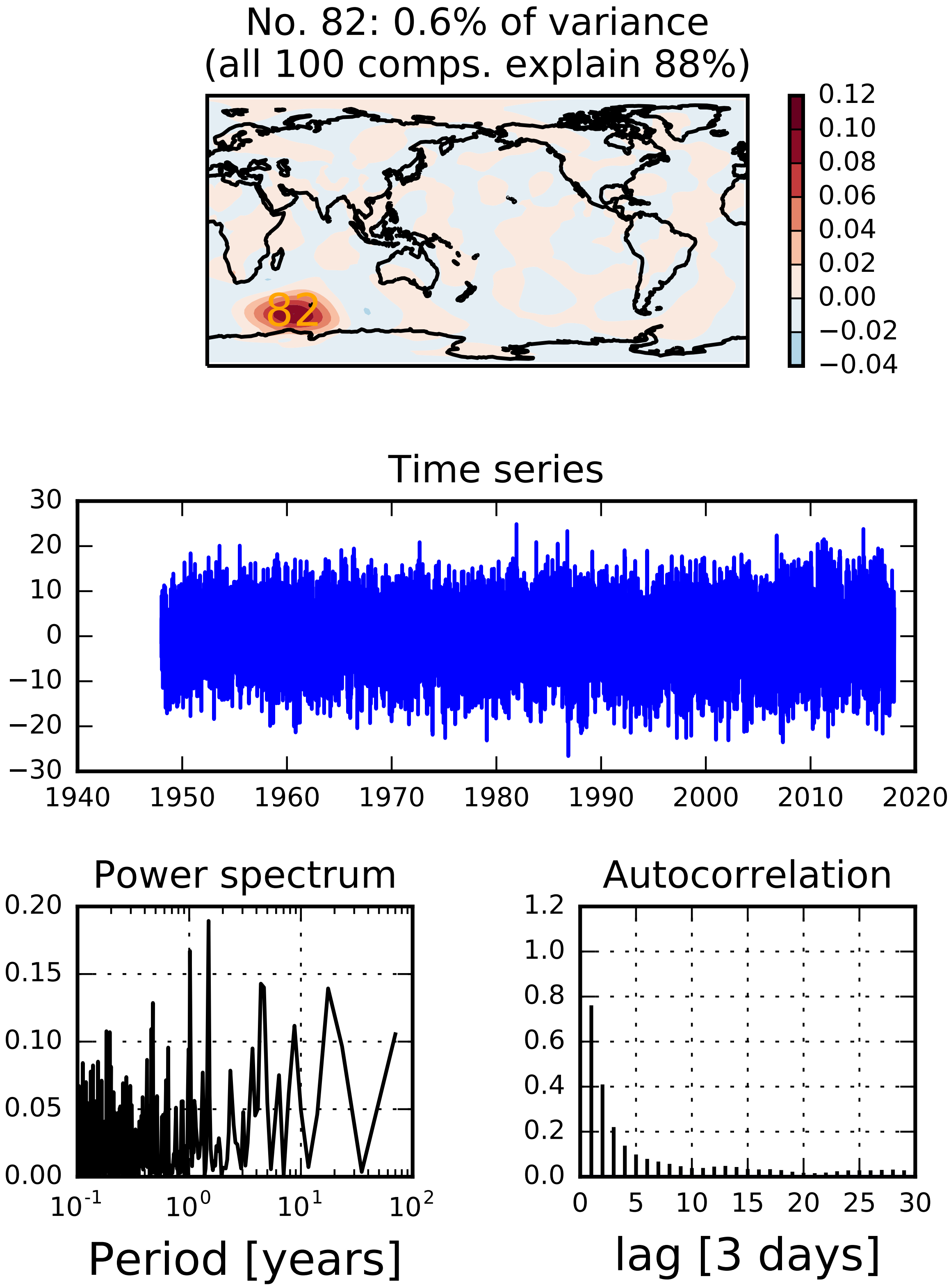
No. 82: 0.6% of variance
(all 100 comps. explain 88%)
82
0.12
0.10
0.08
0.06
0.04
0.02
0.00
−0.02
−0.04
Time series
30
20
10
0
−10
−20
−30
1940
1950
1960
1970
1980
1990
2000
2010
2020
Power spectrum
0.20
0.15
0.10
0.05
0.00
10^-1
10^0
10^1
10^2
Period [years]
Autocorrelation
1.2
1.0
0.8
0.6
0.4
0.2
0.0
0
5
10
15
20
25
30
lag [3 days]

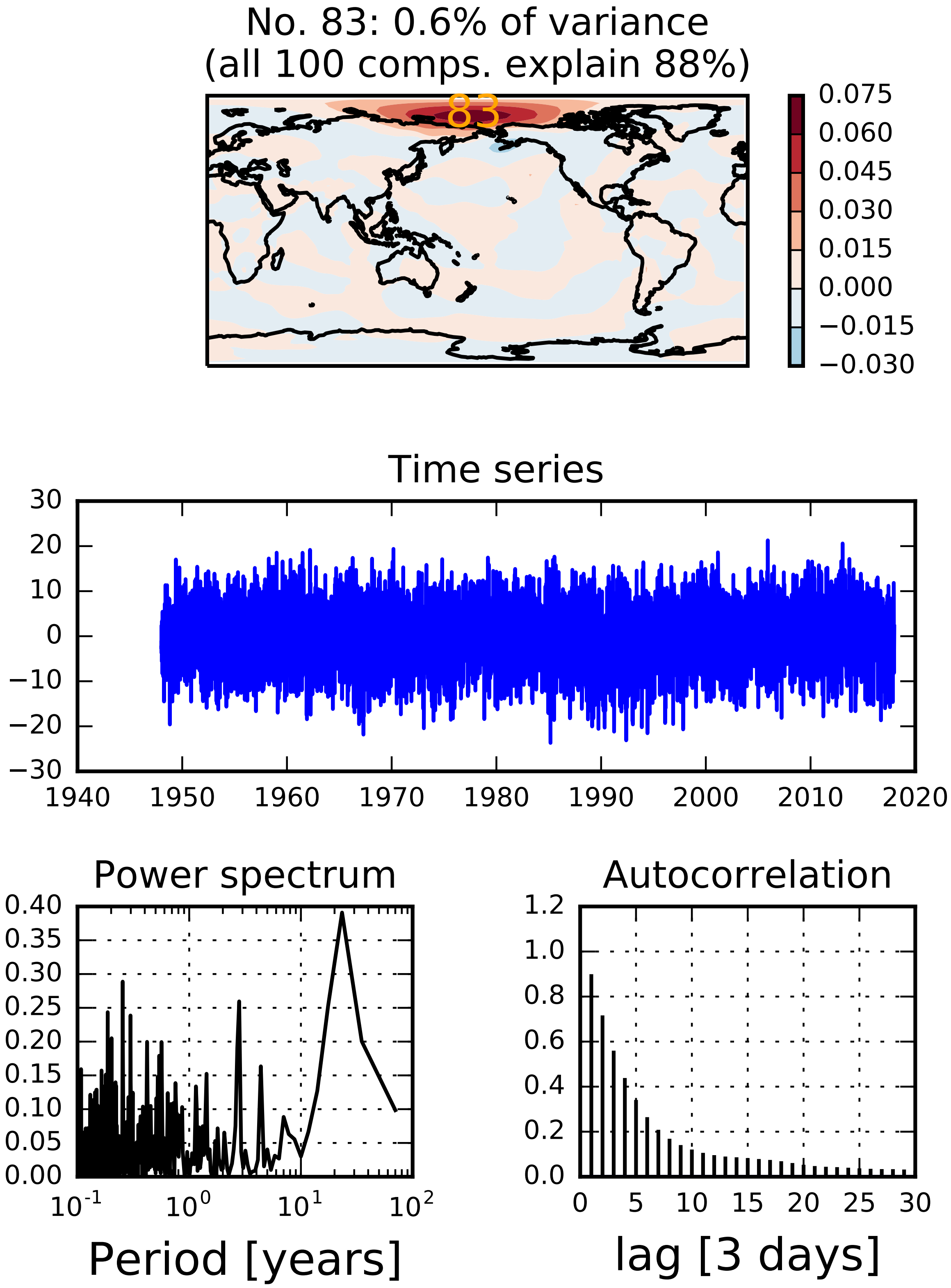
No. 83: 0.6% of variance
(all 100 comps. explain 88%)
83
0.075
0.060
0.045
0.030
0.015
0.000
−0.015
−0.030
Time series
30
20
10
0
−10
−20
−30
1940
1950
1960
1970
1980
1990
2000
2010
2020
Power spectrum
0.40
0.35
0.30
0.25
0.20
0.15
0.10
0.05
0.00
10^-1
10^0
10^1
10^2
Period [years]
Autocorrelation
1.2
1.0
0.8
0.6
0.4
0.2
0.0
0
5
10
15
20
25
30
lag [3 days]

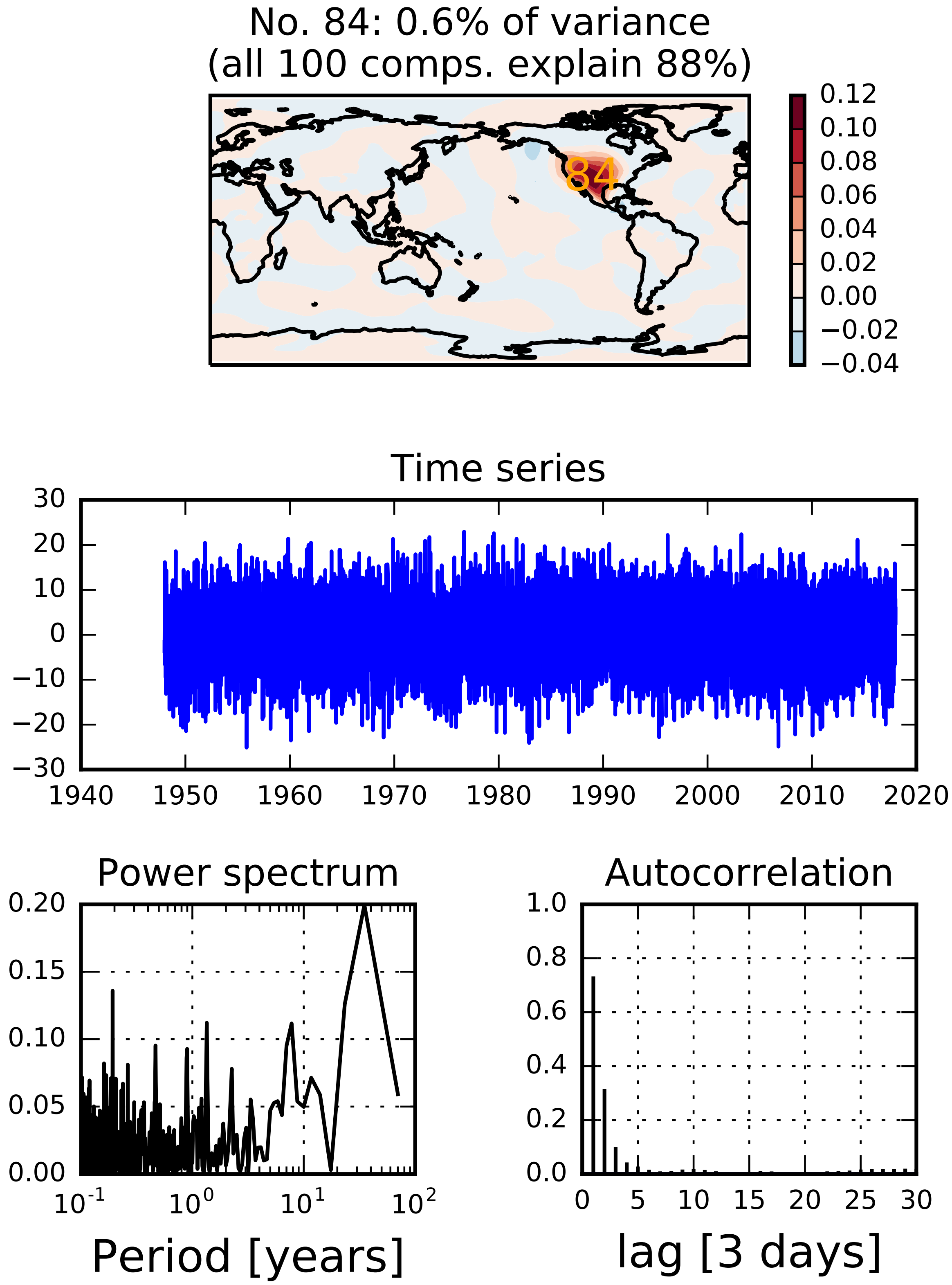
No. 84: 0.6% of variance
(all 100 comps. explain 88%)
84
0.12
0.10
0.08
0.06
0.04
0.02
0.00
−0.02
−0.04
Time series
30
20
10
0
−10
−20
−30
1940
1950
1960
1970
1980
1990
2000
2010
2020
Power spectrum
0.20
0.15
0.10
0.05
0.00
10^-1
10^0
10^1
10^2
Period [years]
Autocorrelation
1.0
0.8
0.6
0.4
0.2
0.0
0
5
10
15
20
25
30
lag [3 days]

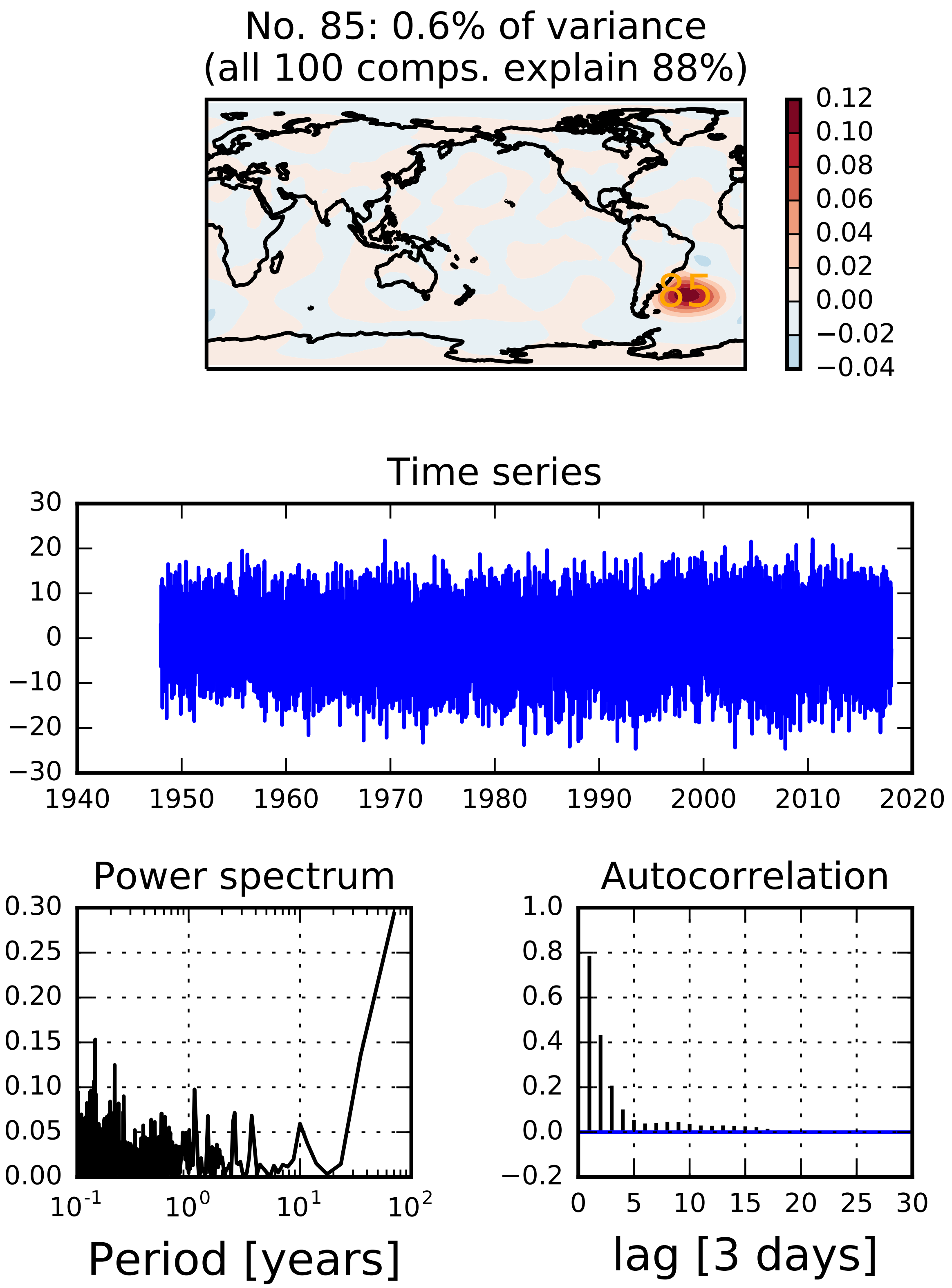
No. 85: 0.6% of variance
(all 100 comps. explain 88%)
85
0.12
0.10
0.08
0.06
0.04
0.02
0.00
−0.02
−0.04
Time series
30
20
10
0
−10
−20
−30
1940
1950
1960
1970
1980
1990
2000
2010
2020
Power spectrum
0.30
0.25
0.20
0.15
0.10
0.05
0.00
10^-1
10^0
10^1
10^2
Period [years]
Autocorrelation
1.0
0.8
0.6
0.4
0.2
0.0
−0.2
0
5
10
15
20
25
30
lag [3 days]

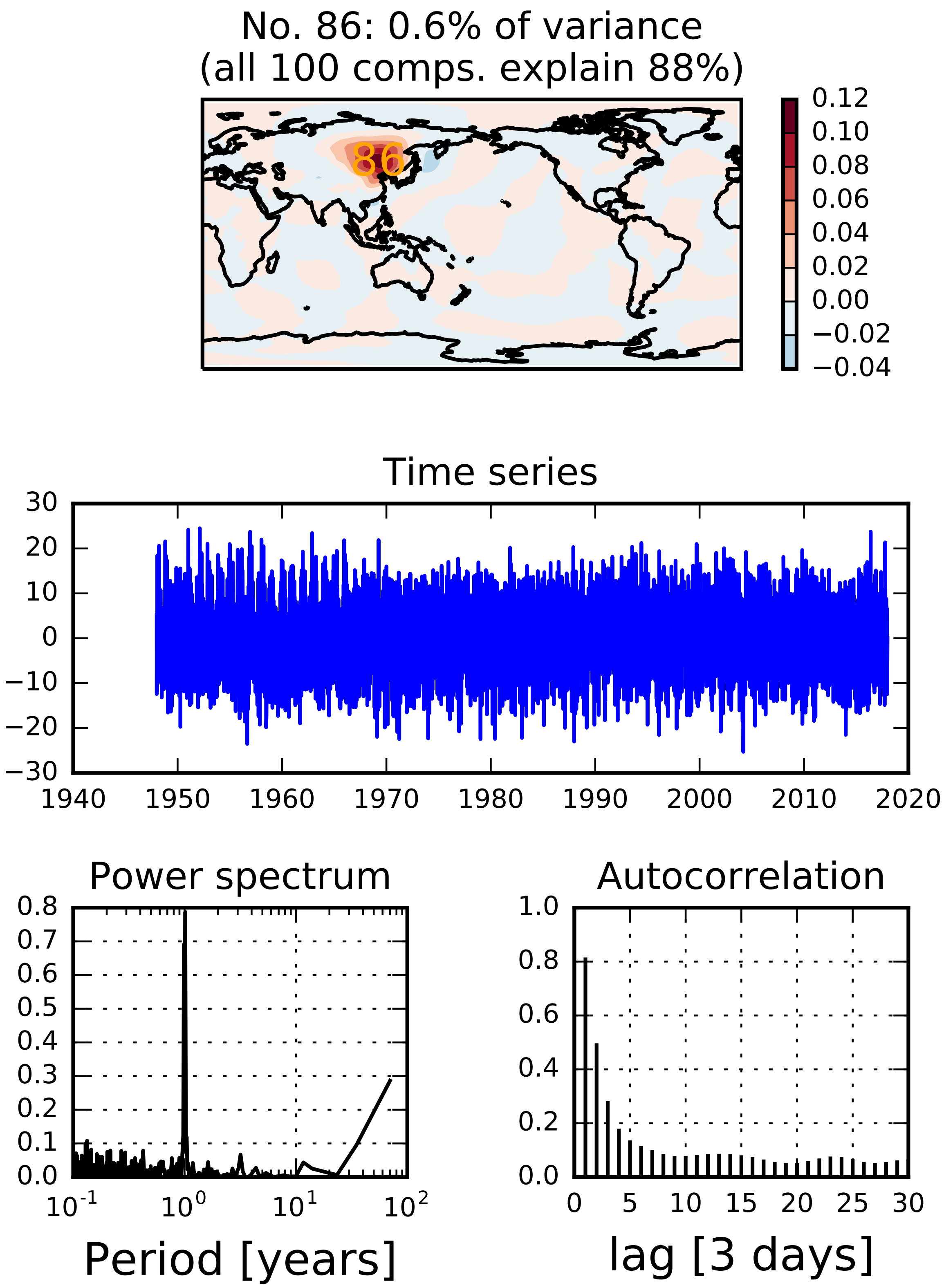
No. 86: 0.6% of variance
(all 100 comps. explain 88%)
86
0.12
0.10
0.08
0.06
0.04
0.02
0.00
−0.02
−0.04
Time series
30
20
10
0
−10
−20
−30
1940
1950
1960
1970
1980
1990
2000
2010
2020
Power spectrum
0.8
0.7
0.6
0.5
0.4
0.3
0.2
0.1
0.0
10^{-1}
10^{0}
10^{1}
10^{2}
Period [years]
Autocorrelation
1.0
0.8
0.6
0.4
0.2
0.0
0
5
10
15
20
25
30
lag [3 days]

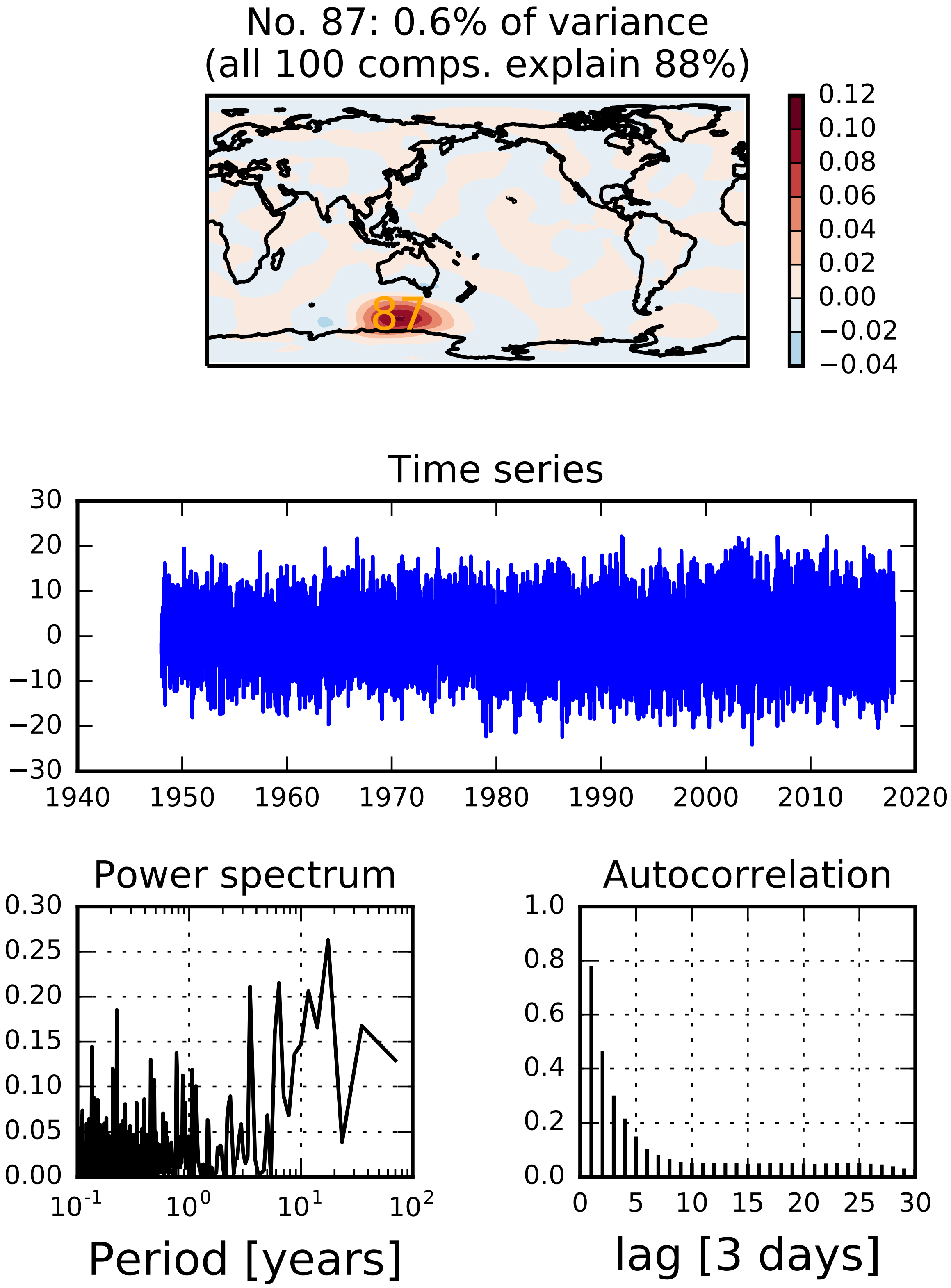
No. 87: 0.6% of variance
(all 100 comps. explain 88%)
87
0.12
0.10
0.08
0.06
0.04
0.02
0.00
−0.02
−0.04
Time series
30
20
10
0
−10
−20
−30
1940
1950
1960
1970
1980
1990
2000
2010
2020
Power spectrum
0.30
0.25
0.20
0.15
0.10
0.05
0.00
10^-1
10^0
10^1
10^2
Period [years]
Autocorrelation
1.0
0.8
0.6
0.4
0.2
0.0
0
5
10
15
20
25
30
lag [3 days]

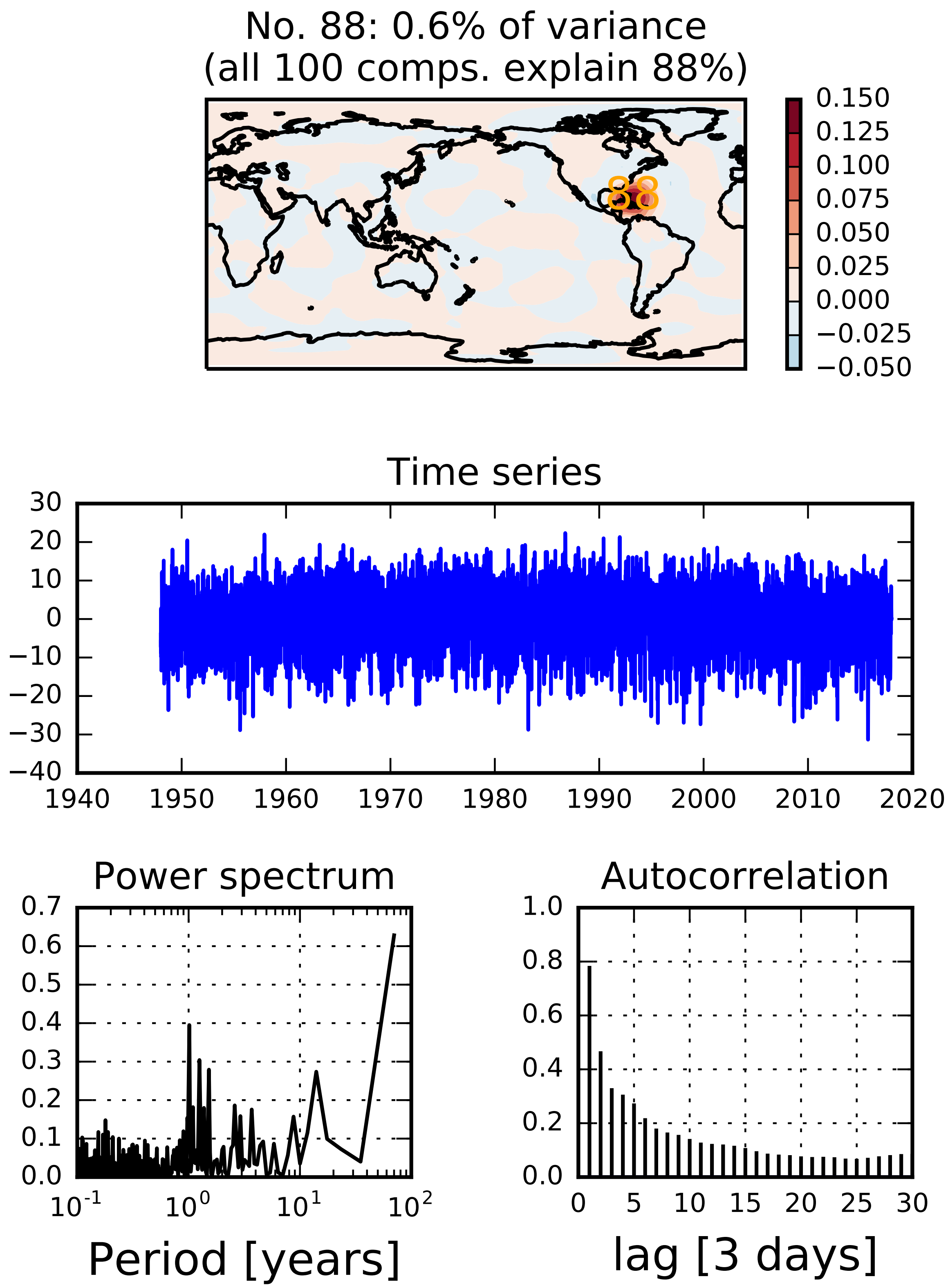
No. 88: 0.6% of variance
(all 100 comps. explain 88%)
0.150
0.125
0.100
0.075
0.050
0.025
0.000
−0.025
−0.050
88
Time series
30
20
10
0
−10
−20
−30
−40
1940
1950
1960
1970
1980
1990
2000
2010
2020
Power spectrum
0.7
0.6
0.5
0.4
0.3
0.2
0.1
0.0
10^{-1}
10^{0}
10^{1}
10^{2}
Period [years]
Autocorrelation
1.0
0.8
0.6
0.4
0.2
0.0
0
5
10
15
20
25
30
lag [3 days]

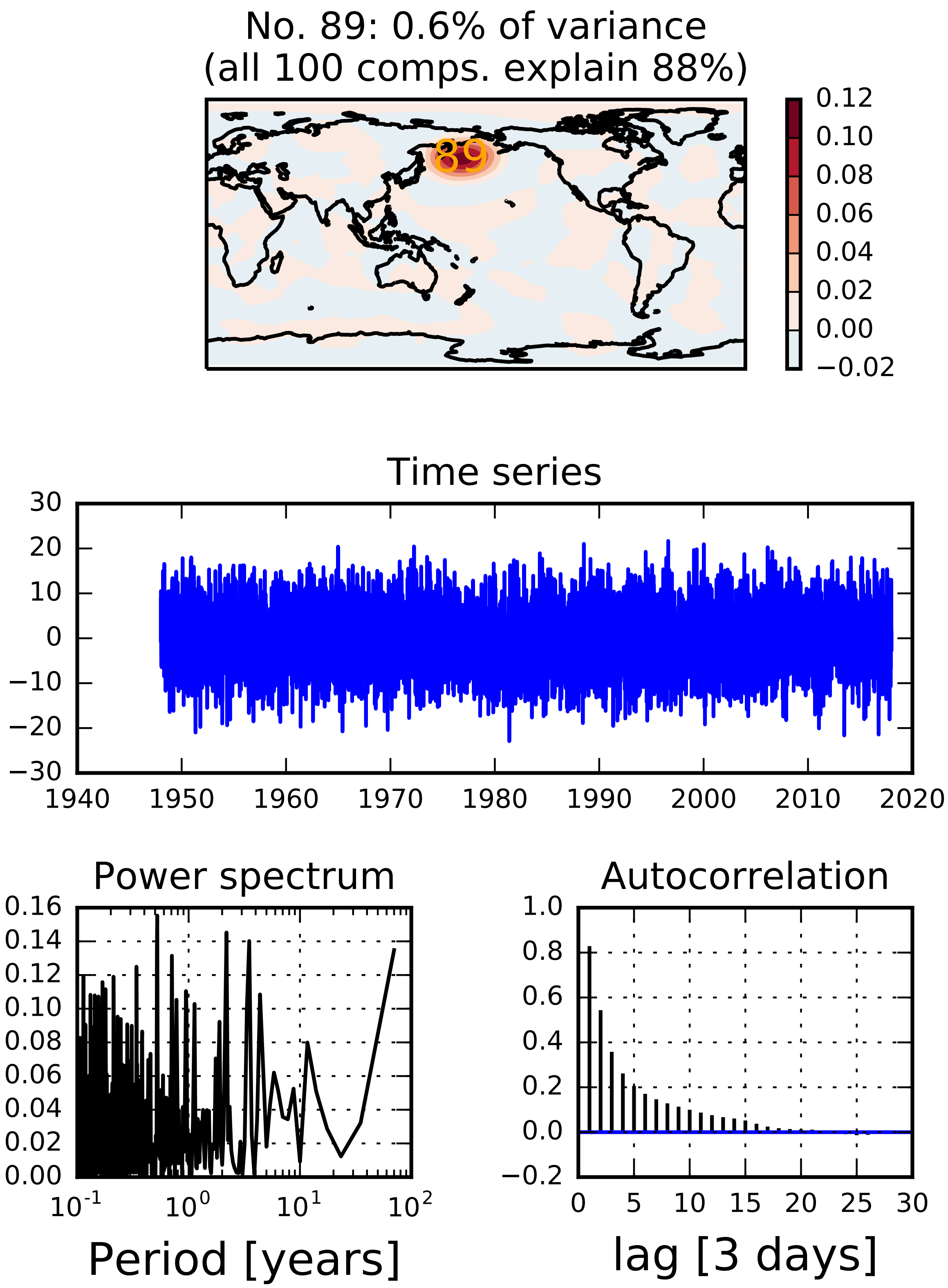
No. 89: 0.6% of variance
(all 100 comps. explain 88%)
89
0.12
0.10
0.08
0.06
0.04
0.02
0.00
−0.02
Time series
30
20
10
0
−10
−20
−30
1940
1950
1960
1970
1980
1990
2000
2010
2020
Power spectrum
0.16
0.14
0.12
0.10
0.08
0.06
0.04
0.02
0.00
10-1
100
101
102
Period [years]
Autocorrelation
1.0
0.8
0.6
0.4
0.2
0.0
−0.2
0
5
10
15
20
25
30
lag [3 days]

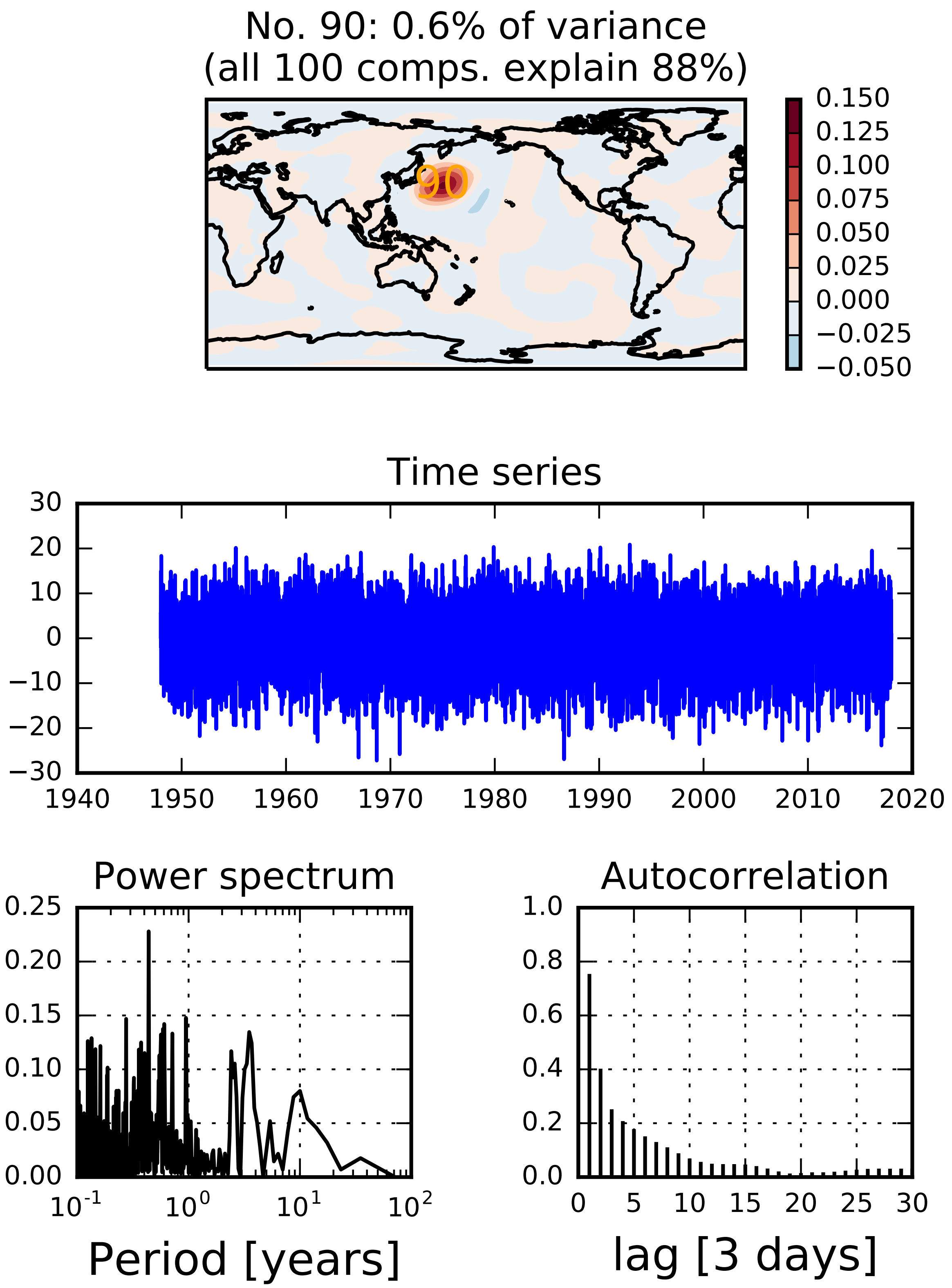
No. 90: 0.6% of variance
(all 100 comps. explain 88%)
90
0.150
0.125
0.100
0.075
0.050
0.025
0.000
−0.025
−0.050
Time series
30
20
10
0
−10
−20
−30
1940
1950
1960
1970
1980
1990
2000
2010
2020
Power spectrum
0.25
0.20
0.15
0.10
0.05
0.00
10^-1
10^0
10^1
10^2
Period [years]
Autocorrelation
1.0
0.8
0.6
0.4
0.2
0.0
0
5
10
15
20
25
30
lag [3 days]

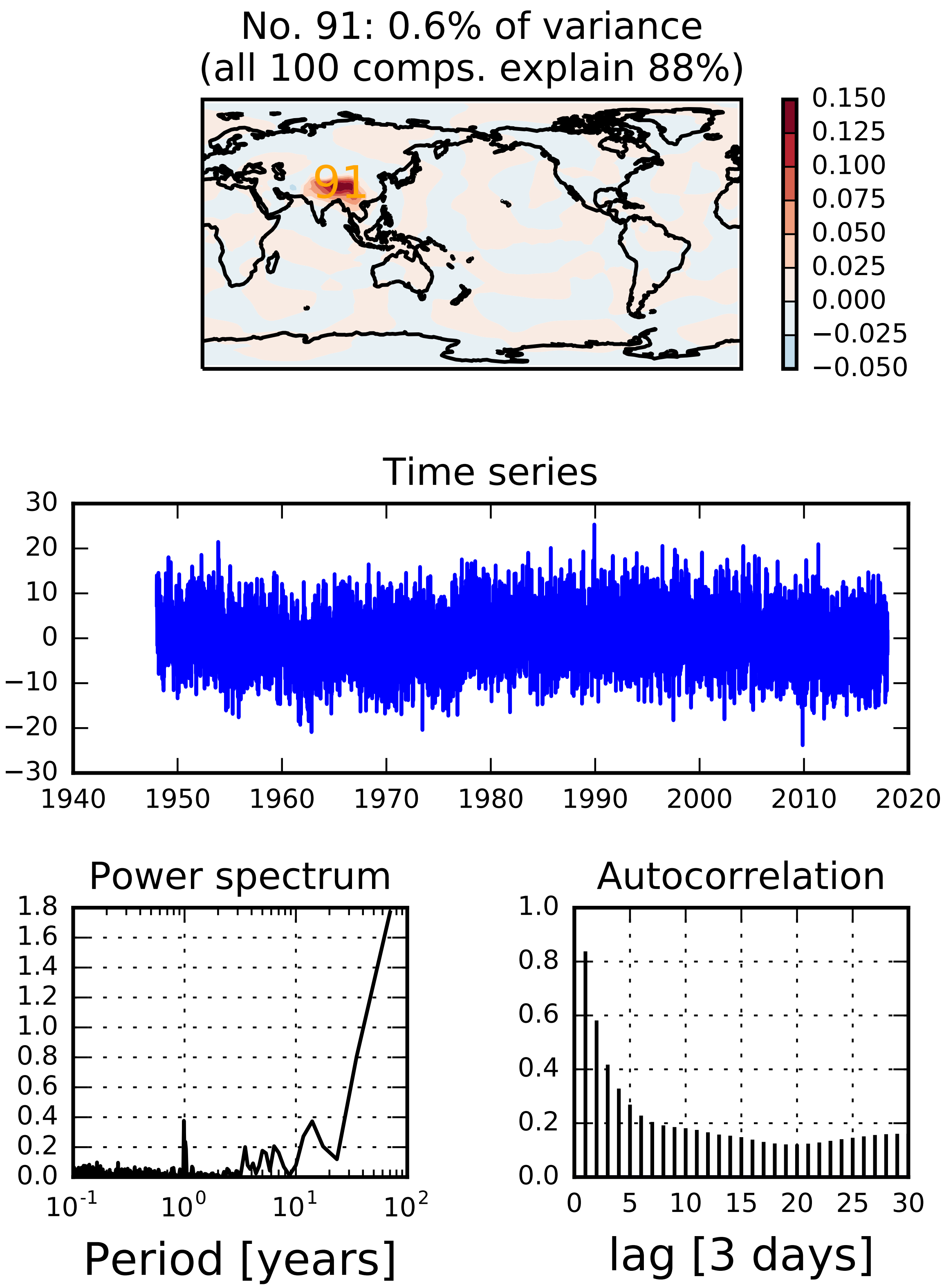
No. 91: 0.6% of variance
(all 100 comps. explain 88%)
91
0.150
0.125
0.100
0.075
0.050
0.025
0.000
−0.025
−0.050
Time series
30
20
10
0
−10
−20
−30
1940
1950
1960
1970
1980
1990
2000
2010
2020
Power spectrum
1.8
1.6
1.4
1.2
1.0
0.8
0.6
0.4
0.2
0.0
10^{-1}
10^{0}
10^{1}
10^{2}
Period [years]
Autocorrelation
1.0
0.8
0.6
0.4
0.2
0.0
0
5
10
15
20
25
30
lag [3 days]

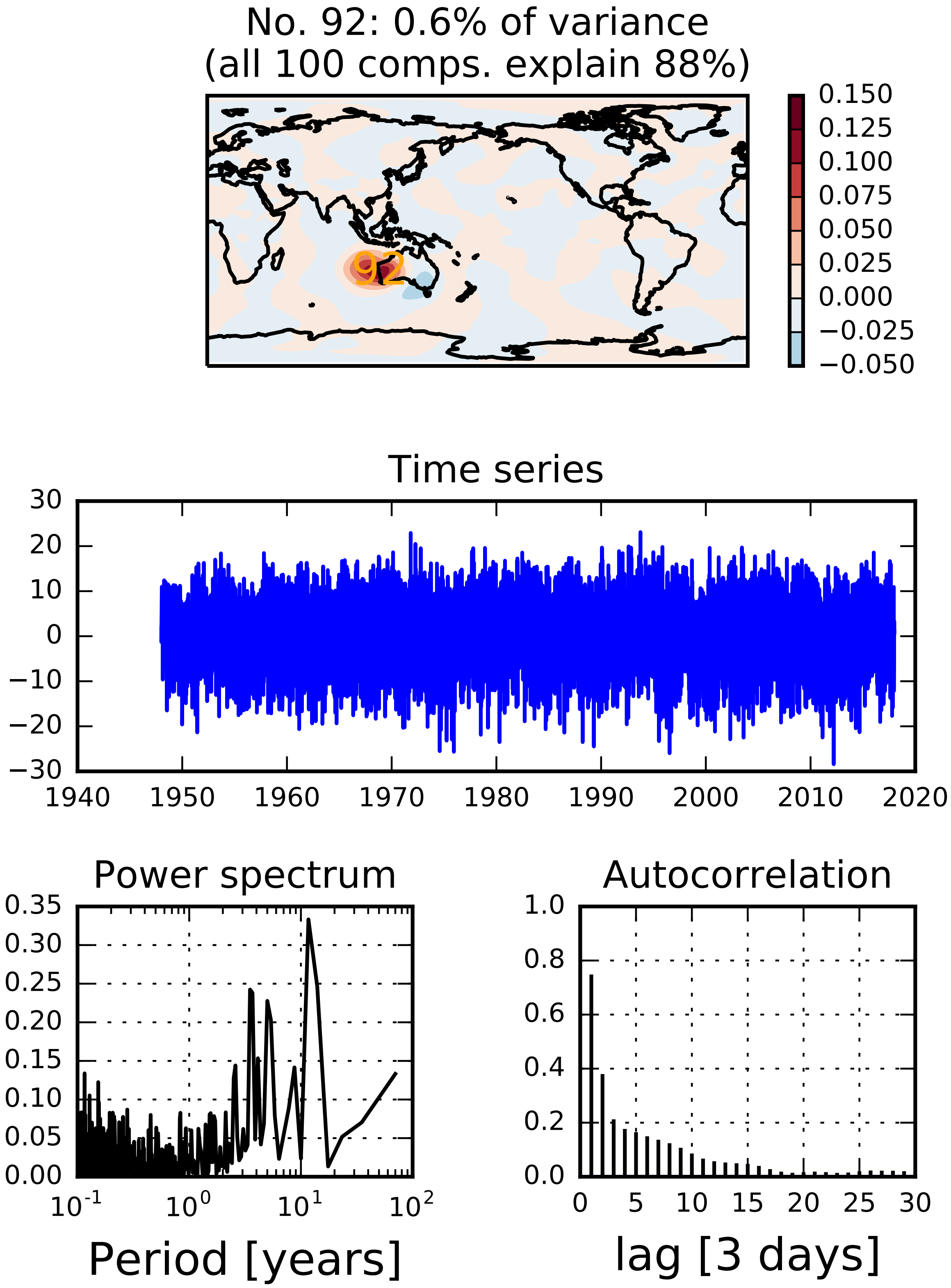
No. 92: 0.6% of variance
(all 100 comps. explain 88%)
92
0.150
0.125
0.100
0.075
0.050
0.025
0.000
−0.025
−0.050
Time series
30
20
10
0
−10
−20
−30
1940
1950
1960
1970
1980
1990
2000
2010
2020
Power spectrum
0.35
0.30
0.25
0.20
0.15
0.10
0.05
0.00
10^{-1}
10^{0}
10^{1}
10^{2}
Period [years]
Autocorrelation
1.0
0.8
0.6
0.4
0.2
0.0
0
5
10
15
20
25
30
lag [3 days]

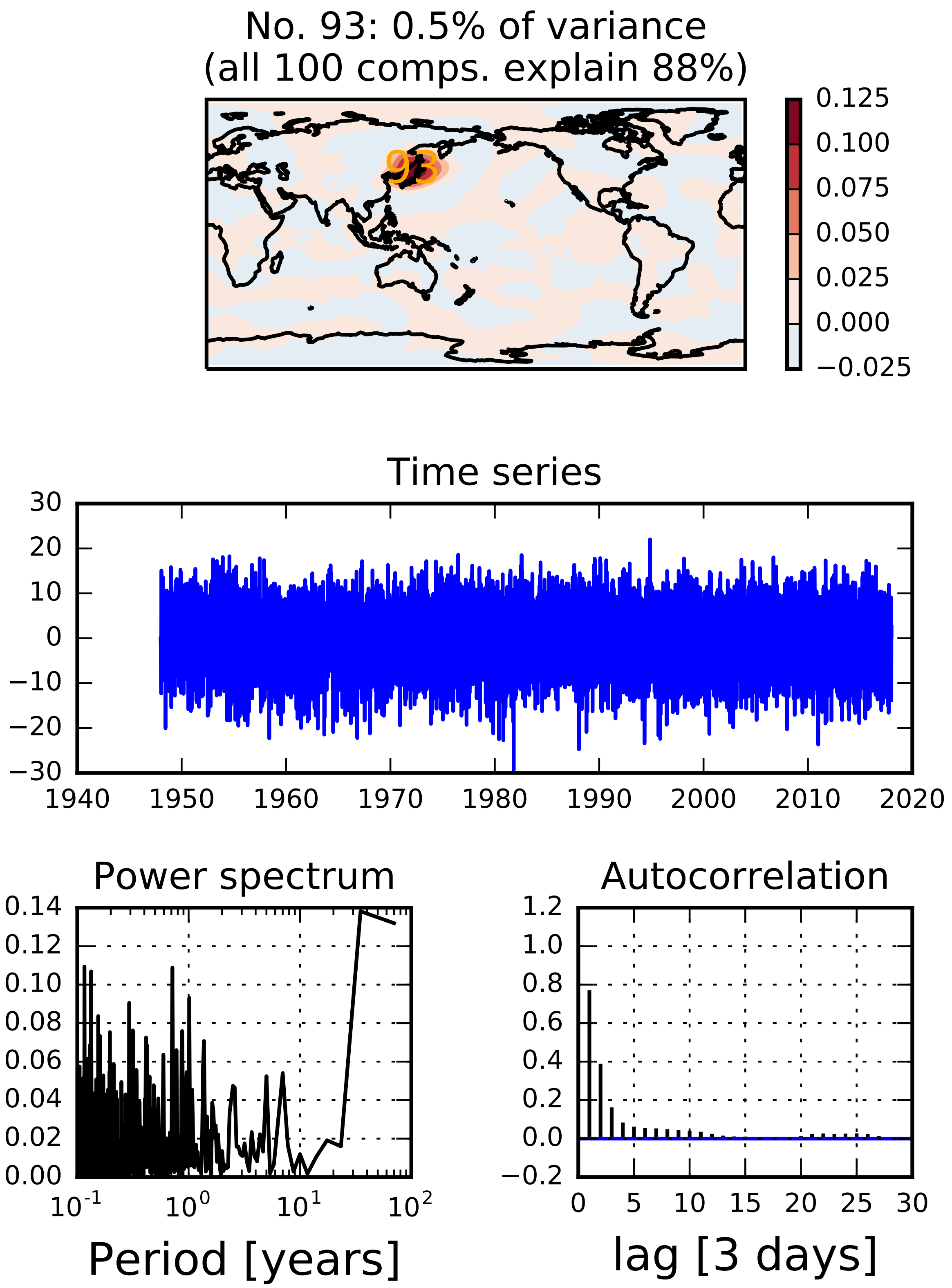

No. 93: 0.5% of variance
(all 100 comps. explain 88%)
93
0.125
0.100
0.075
0.050
0.025
0.000
−0.025
Time series
30
20
10
0
−10
−20
−30
1940
1950
1960
1970
1980
1990
2000
2010
2020
Power spectrum
0.14
0.12
0.10
0.08
0.06
0.04
0.02
0.00
10^{-1}
10^{0}
10^{1}
10^{2}
Period [years]
Autocorrelation
1.2
1.0
0.8
0.6
0.4
0.2
0.0
−0.2
0
5
10
15
20
25
30
lag [3 days]

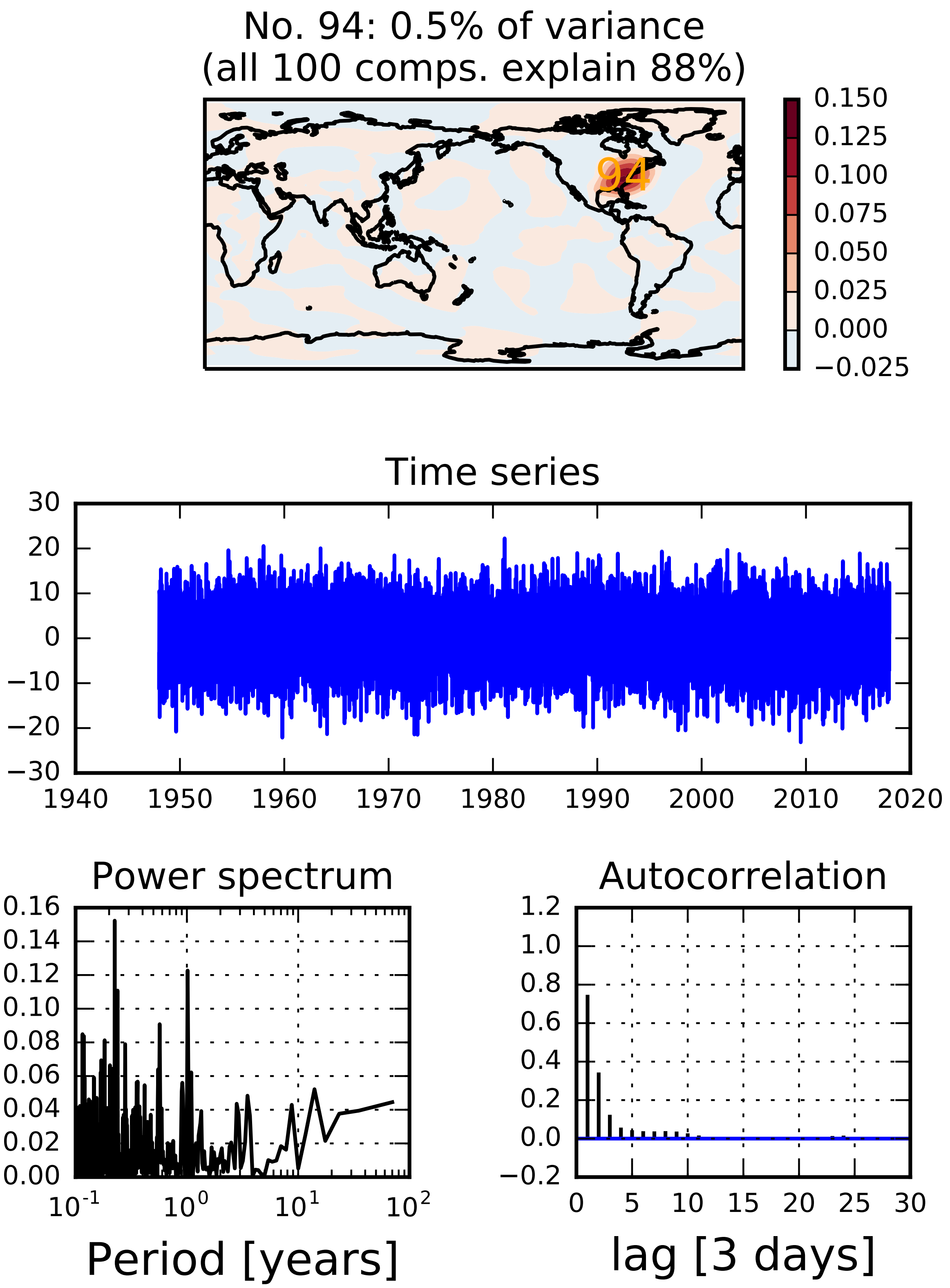

No. 94: 0.5% of variance
(all 100 comps. explain 88%)
94
0.150
0.125
0.100
0.075
0.050
0.025
0.000
−0.025
Time series
30
20
10
0
−10
−20
−30
1940
1950
1960
1970
1980
1990
2000
2010
2020
Power spectrum
0.16
0.14
0.12
0.10
0.08
0.06
0.04
0.02
0.00
10^{-1}
10^{0}
10^{1}
10^{2}
Period [years]
Autocorrelation
1.2
1.0
0.8
0.6
0.4
0.2
0.0
−0.2
0
5
10
15
20
25
30
lag [3 days]

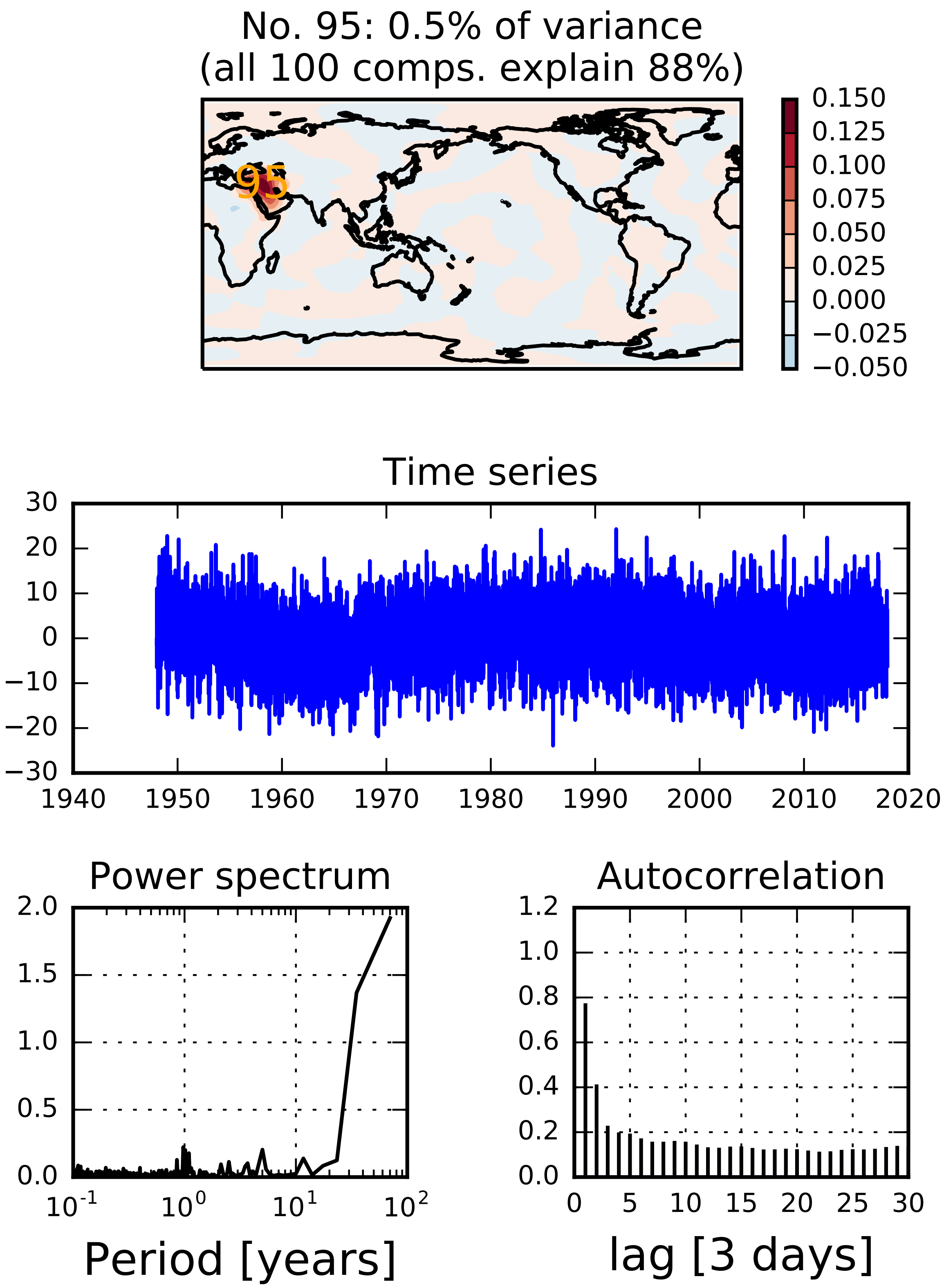
No. 95: 0.5% of variance
(all 100 comps. explain 88%)
95
0.150
0.125
0.100
0.075
0.050
0.025
0.000
−0.025
−0.050
Time series
30
20
10
0
−10
−20
−30
1940
1950
1960
1970
1980
1990
2000
2010
2020
Power spectrum
2.0
1.5
1.0
0.5
0.0
10^{-1}
10^{0}
10^{1}
10^{2}
Period [years]
Autocorrelation
1.2
1.0
0.8
0.6
0.4
0.2
0.0
0
5
10
15
20
25
30
lag [3 days]

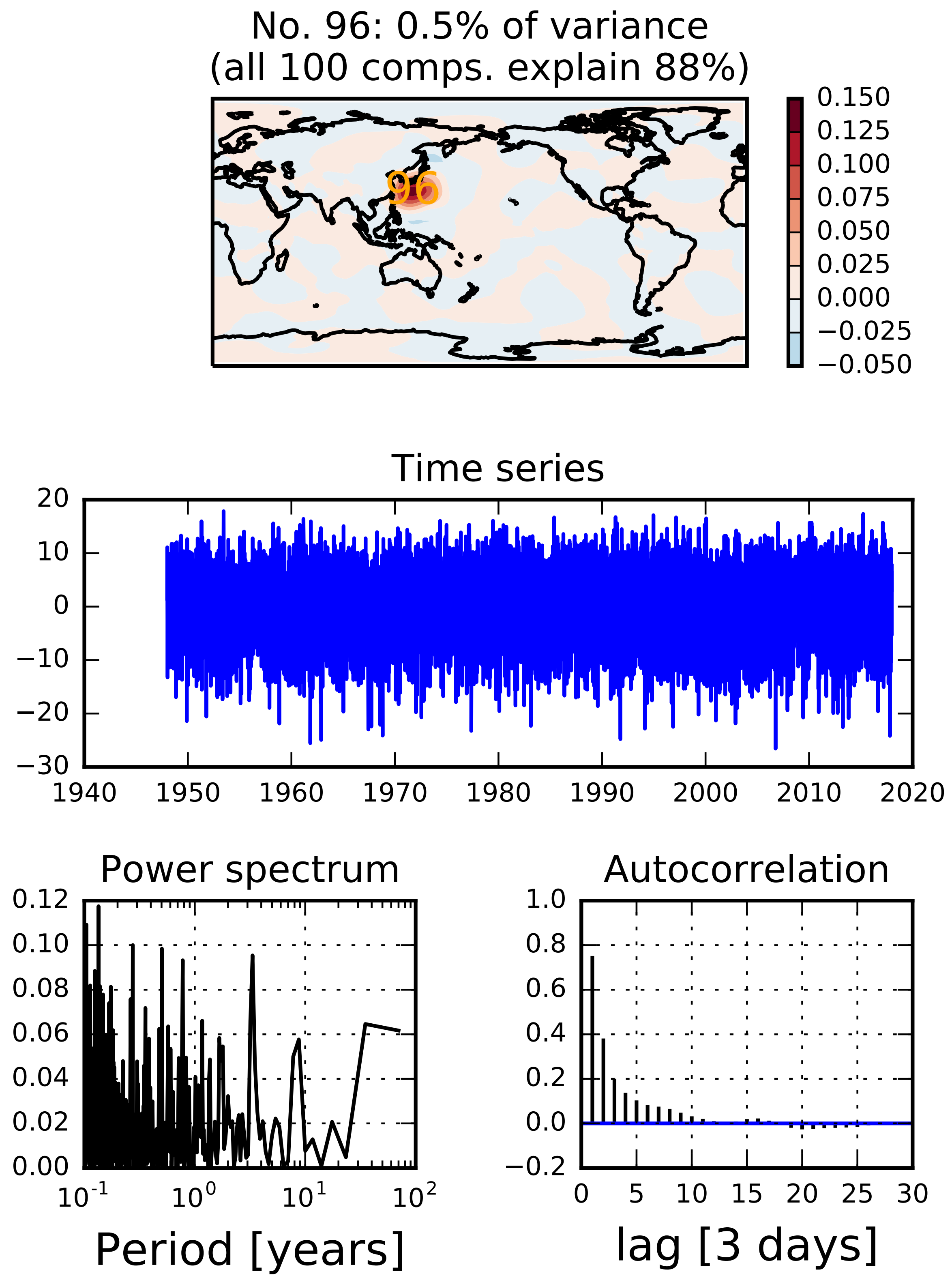
No. 96: 0.5% of variance
(all 100 comps. explain 88%)
96
0.150
0.125
0.100
0.075
0.050
0.025
0.000
−0.025
−0.050
Time series
20
10
0
−10
−20
−30
1940
1950
1960
1970
1980
1990
2000
2010
2020
Power spectrum
0.12
0.10
0.08
0.06
0.04
0.02
0.00
10^-1
10^0
10^1
10^2
Period [years]
Autocorrelation
1.0
0.8
0.6
0.4
0.2
0.0
−0.2
0
5
10
15
20
25
30
lag [3 days]

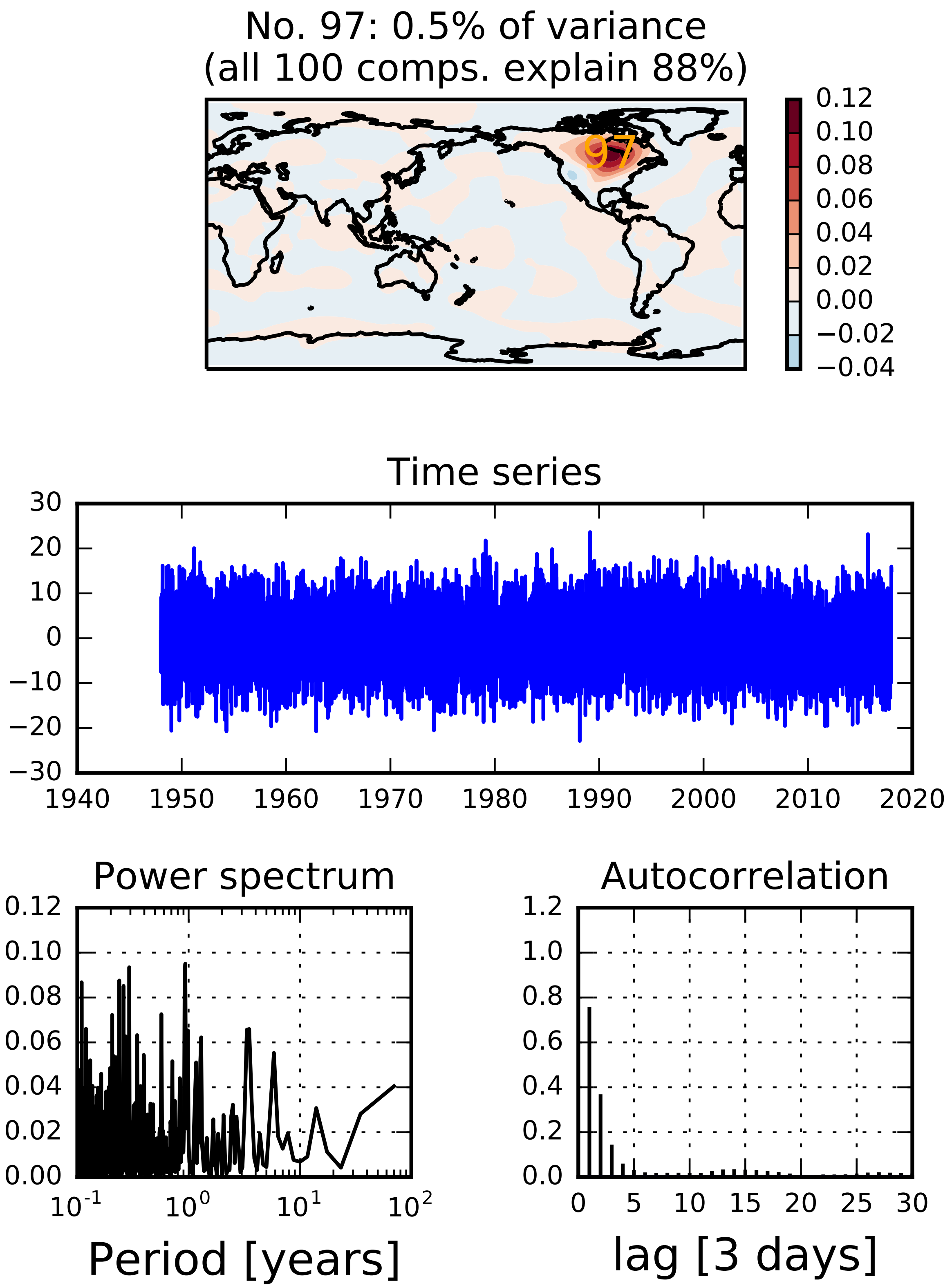
No. 97: 0.5% of variance
(all 100 comps. explain 88%)
97
0.12
0.10
0.08
0.06
0.04
0.02
0.00
−0.02
−0.04
Time series
30
20
10
0
−10
−20
−30
1940
1950
1960
1970
1980
1990
2000
2010
2020
Power spectrum
0.12
0.10
0.08
0.06
0.04
0.02
0.00
10^{-1}
10^{0}
10^{1}
10^{2}
Period [years]
Autocorrelation
1.2
1.0
0.8
0.6
0.4
0.2
0.0
0
5
10
15
20
25
30
lag [3 days]

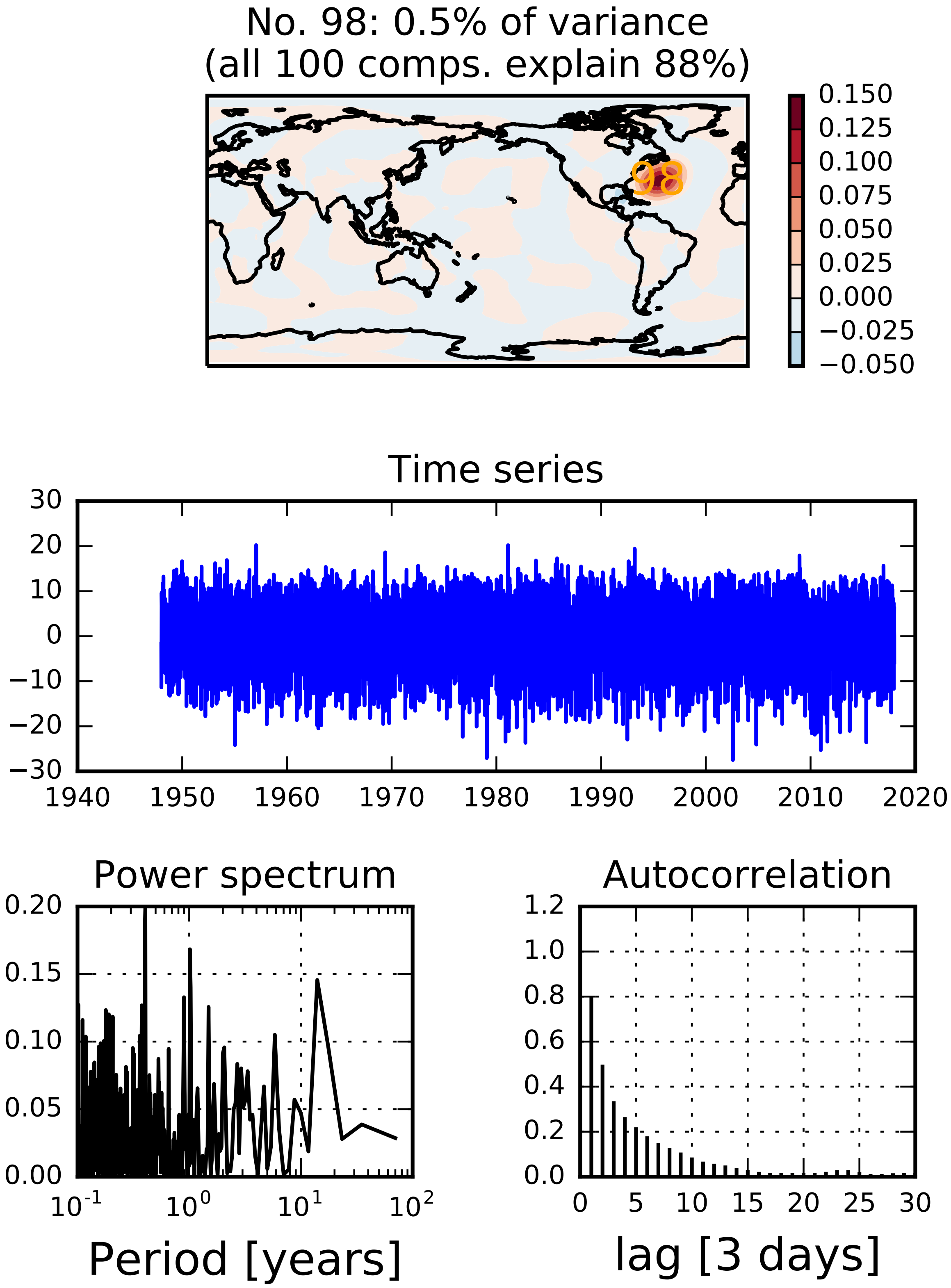
No. 98: 0.5% of variance
(all 100 comps. explain 88%)
98
0.150
0.125
0.100
0.075
0.050
0.025
0.000
−0.025
−0.050
Time series
30
20
10
0
−10
−20
−30
1940
1950
1960
1970
1980
1990
2000
2010
2020
Power spectrum
0.20
0.15
0.10
0.05
0.00
10^-1
10^0
10^1
10^2
Period [years]
Autocorrelation
1.2
1.0
0.8
0.6
0.4
0.2
0.0
0
5
10
15
20
25
30
lag [3 days]

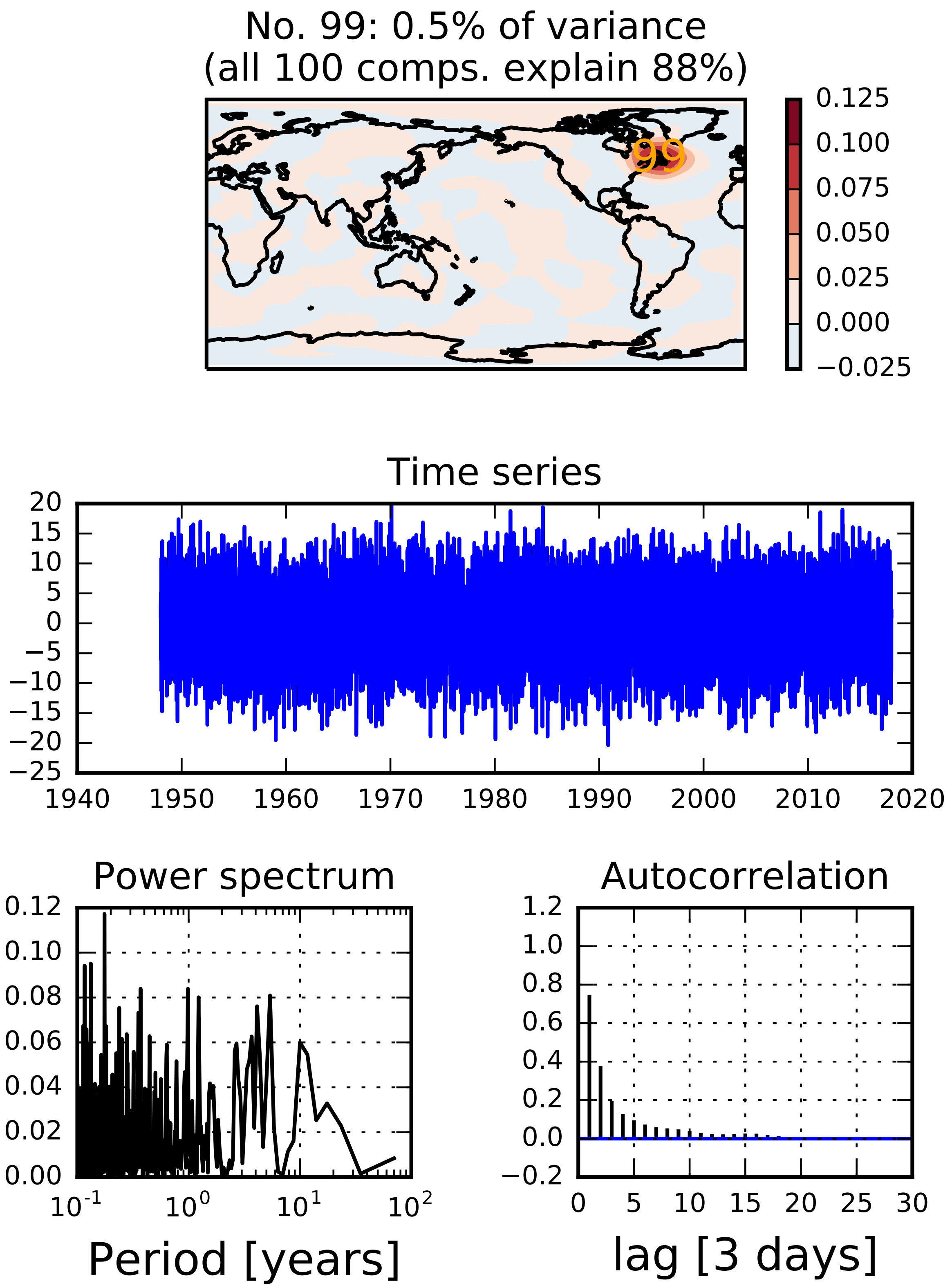
No. 99: 0.5% of variance
(all 100 comps. explain 88%)
99
0.125
0.100
0.075
0.050
0.025
0.000
−0.025
Time series
20
15
10
5
0
−5
−10
−15
−20
−25
1940
1950
1960
1970
1980
1990
2000
2010
2020
Power spectrum
0.12
0.10
0.08
0.06
0.04
0.02
0.00
10^-1
10^0
10^1
10^2
Period [years]
Autocorrelation
1.2
1.0
0.8
0.6
0.4
0.2
0.0
−0.2
0
5
10
15
20
25
30
lag [3 days]
